# Supplementary material for: Targeted sampling of natural product space to identify bioactive natural product-like polyketide macrolides
Source: Nat Commun. 2024 Mar 21;15:2534. doi: 10.1038/s41467-024-46721-x (PMC10958047; doi:10.1038/s41467-024-46721-x)
Supplement: Supplementary file 1 — Supplementary Information [file 41467_2024_46721_MOESM1_ESM.pdf]

# Supplementary Information

**Targeted Sampling of Natural Product Space to Identify Bioactive Natural Product-Like Polyketide Macrolides**

Darryl M. Wilson,[1] Daniel J. Driedger,[1] Dennis Y. Liu,[1] Sandra Keerthisinghe,[2] Adrian Hermann,[3] Christoph Bieniossek,[3] Roger G. Linington,[1,2,*] Robert A. Britton[1,*]

[1] Department of Chemistry, Simon Fraser University, Burnaby, British Columbia, V5A 1S6, Canada.

[2] Center for High-Throughput Chemical Biology, Simon Fraser University, Burnaby, British Columbia, V5A 1S6, Canada

[3] Roche Pharma Research and Early Development, Roche Innovation Center Basel, F. Hoffmann-La Roche Ltd, Grenzacherstrasse 124, 4070, Basel, Switzerland

*Correspondence to: rbritton@sfu.ca or rliningt@sfu.ca

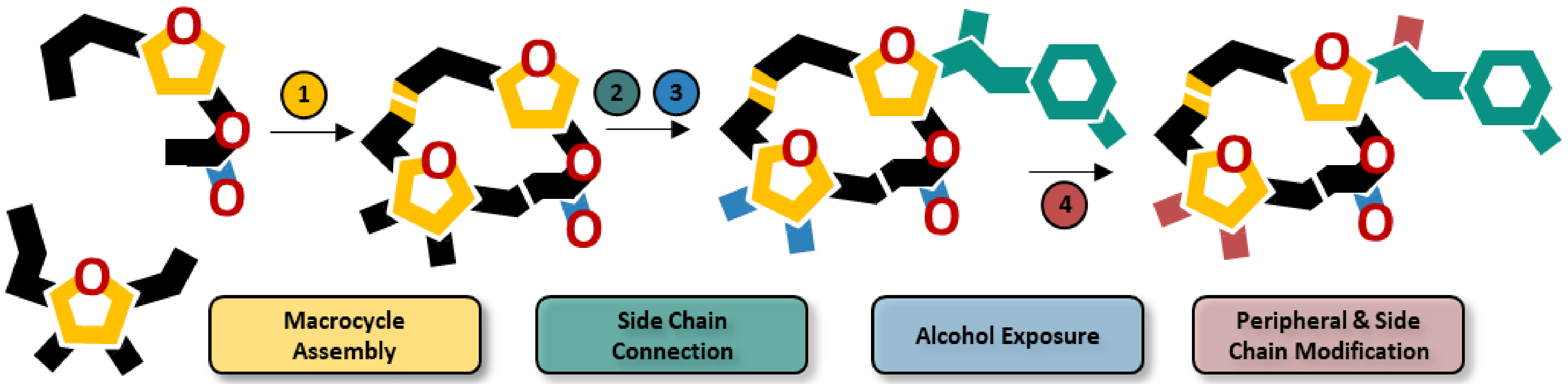

# Contents

**Supplementary Figures ........ 6**

Supplementary Fig. 1 | THF Macrolide Natural Products Used in Similarity Based Analysis. ........ 6

Supplementary Fig. 1 (cont) | THF Macrolide Natural Products Used in Similarity Based Analysis. ........ 7

Supplementary Fig. 2 | Library pML building blocks. ........ 8

Supplementary Fig. 2 (cont) | Library pML building blocks. ........ 9

Supplementary Fig. 3 | Visual examples of combination of building blocks in silico. ........ 9

Supplementary Fig. 4 | Polar plot depicting the $C_s$ (*r*) vs. library pML ID (*ϑ*) sorted by THF stereochemistry. ........ 10

Supplementary Fig. 5 | Polar plot depicting $C_s^{max}$ (*r*) vs. library pML ID (*ϑ*) sorted by THF stereochemistry with synthetic compounds highlighted. ........ 11

Supplementary Fig 6 | Polar plot of $C_s$ (*r*) vs. library pML ID (*ϑ*) sorted by Natural Product. ........ 12

Supplementary Fig. 7 | Cartoon Depiction of Evaluation of $C_s$ score. ........ 13

Supplementary Fig. 8 | Standard Deviations of $C_s$ scores (*Y*-axis) vs. Average $C_s$ scores (*X*-axis) for all 306 comparisons among the 18 simulated NPs for N=3 replicates of 2500 MD iterations. ........ 14

Supplementary Fig. 9 | Example Calculation of HA (heavy-atom) parameters for Fijianolide A. ........ 15

Supplementary Fig. 10 | Summary of Stereochemical Assignments for All Chiral Building Blocks. ........ 16

Supplementary Fig. 11 | Evidence for Stereochemistry of 1b, 1e, 1d, 2g 2c, 2e. ........ 17

Supplementary Fig. 12 | Evidence for Stereochemistry of 1a, 1c, 2f, 2h. ........ 18

Supplementary Fig. 13 | Evidence for Stereochemistry of 2a. ........ 19

Supplementary Fig. 14 | Evidence for Stereochemistry of 2b. ........ 20

Supplementary Fig. 15 | Evidence for Stereochemistry of 2d. ........ 21

Supplementary Fig. 16 | Evidence for Stereochemistry of 3b. ........ 21

Supplementary Fig. 17 | Evidence for E/Z configuration in pMLs. ........ 22

Supplementary Fig. 18A | Cell painting fingerprint for Fijianolide A ........ 23

Supplementary Fig. 18B | Cell painting activity threshold plot for Fijianolide A ..... 23
Supplementary Fig | 19A. Cell painting fingerprint for Fijianolide B ..... 24
Supplementary Fig. 19B | Cell painting activity threshold plot for Fijianolide B ..... 24
Supplementary Fig. 20A | Cell painting fingerprint for 7e ..... 25
Supplementary Fig. 20B | Cell painting activity threshold plot for 7e ..... 25
Supplementary Fig. 21A | Cell painting fingerprint for 7eAc ..... 26
Supplementary Fig. 21B | Cell painting activity threshold plot for 7eAc ..... 26
Supplementary Fig. 22A | Cell painting fingerprint for 11bAc ..... 27
Supplementary Fig. 22B | Cell painting activity threshold plot for 11bAc ..... 27
Supplementary Fig. 23A | Cell painting fingerprint for 12bAc ..... 28
Supplementary Fig. 23B | Cell painting activity threshold plot for 12bAc ..... 28
Supplementary Fig 24A | Cell painting fingerprint for 18b ..... 29
Supplementary Fig. 24B | Cell painting activity threshold plot for 18b ..... 29
Supplementary Fig. 25A | Cell painting fingerprint for 18bAc ..... 30
Supplementary Fig. 25B | Cell painting activity threshold plot for 18b ..... 30
Supplementary Fig. 26 | Custom glass apparatus for Nozaki-Hiyama-Kishi reactions. ..... 31
**Supplementary Tables ..... 32**
Supplementary Table 1 | Cheminformatics properties of in-silico pML library, synthesized pML library, and NP reference compounds. ..... 32
Supplementary Table 2 | Composition of in-silico pML library, synthesized pML library, and NP reference compounds. ..... 33
Supplementary Table 3 | % Bioactive conformer reproduction of 30 macrocyclic ligands bound to their biological targets for method used in this work (grey) and 3 representative methods reported in the literature.[1] ..... 34
Supplementary Table 4 | $C_s$ score variation between independent runs of the same number of MD iterations for all pairwise comparisons (306) among the natural products with self-overlaps removed. ..... 35
Supplementary Table 5 | Bacterial Target Panel Strains and Culture Conditions ..... 35

**Supplementary Methods** .......... **36**
I. Computational Methods and Results .......... 36
Similarity Comparisons and Prioritization of pMLs for Synthesis: .......... 36
3D Conformational and Molecular Similarity Scoring: .......... 36
Scoring of Library pMLs against THF natural products .......... 37
Prioritization of compounds for synthesis: .......... 38
Applicability of high temperature MD protocol to conformational sampling of macrocycles in general .......... 39
Consistency of the Conformation Generation and Similarity Scoring Protocols: .......... 39
II. Experimental Methods and Results .......... 40
Overview of Stereochemical Assignment for Building Blocks .......... 40
Procedures for the Synthesis and Characterization of Tetrahydrofuranol Building Blocks .......... 42
Supplementary Fig. 27 | Synthesis of Tetrahydrofuranols 1a, 1c and Phosphonates S8, S10. .......... 42
Supplementary Fig. 28 | Synthesis of Tetrahydrofuranol 1d and Phosphonate S18. .......... 68
Supplementary Fig. 29 | Synthesis of Tetrahydrofuranols 1b, 1e and Phosphonates S27, S29. .......... 87
Procedures for the Synthesis and Characterization of Acid/Aldehyde Building Blocks .......... 115
Supplementary Fig. 30 | Synthesis of Alcohol 2b and Aldehyde S39 from OTBDPS Protected Propanediol. .......... 115
Supplementary Fig. 31 | Synthesis of Alcohol 2a from methyl (*R*)-(-)-3-hydroxy-2-methylpropionate. .......... 136
Supplementary Fig. 32 | Synthesis of Tetrahydrofuranoate 2h. .......... 157
Supplementary Fig. 33 | Synthesis of Chlorinated Ester 2e. .......... 165
Supplementary Fig. 34 | Synthesis of Chlorinated Ester 2c. .......... 170
Supplementary Fig. 35 | Synthesis of PMB-protected alcohol 2d. .......... 183
Supplementary Fig. 36 | Synthesis of Tetrahydrofuranoate 2g. .......... 192
Supplementary Fig. 37 | Synthesis of Chlorinated Ester 2f. .......... 197

Procedures for the Synthesis and Characterization of Vinyl Iodide Building Blocks ........ 207
Supplementary Fig. 38 | Synthesis of Dihydropyran Vinyl Iodide 3b. ........ 207
Supplementary Fig. 39 | Synthesis of Methylamide Vinyl Iodide 3a. ........ 215
Supplementary Fig. 40 | Synthesis of Thiazole Vinyl Iodide 3c. ........ 222
Procedures for the Synthesis and Characterization of pMLs ........ 226
Supplementary Fig. 41 | Synthesis of pMLs 9a, 9b, 9d, 9aAc, 9bAc, and 9dAc. ........ 227
Supplementary Fig. 42 | Synthesis of pMLs 16a-d and 16aAc-dAc. ........ 240
Supplementary Fig. 43 | Synthesis of pMLs 7a-e and 7aAc-eAc. ........ 256
Supplementary Fig. 44 | Synthesis of pMLs 14a-d and 14aAc-dAc. ........ 287
Supplementary Fig. 45 | Synthesis of pMLs 8a-d and 8aAc-dAc. ........ 307
Supplementary Fig. 46 | Synthesis of pMLs 10a-d and 10aAc-dAc. ........ 323
Supplementary Fig. 46 (cont) | Synthesis of pMLs 10a-d and 10aAc-dAc. ........ 324
Supplementary Fig. 47 | Synthesis of pMLs 15a-d and 15aAc-dAc. ........ 339
Supplementary Fig. 48 | Synthesis of pMLs 18a-d and 18aAc-dAc. ........ 354
Supplementary Fig. 49 | Synthesis of pMLs 13a-d and 13aAc-dAc. ........ 368
Supplementary Fig. 50 | Synthesis of pMLs 17a-d and 17aAc-dAc. ........ 389
Supplementary Fig. 51 | Synthesis of pMLs 12a-d and 12aAc-dAc. ........ 416
Supplementary Fig. 52 | Synthesis of pMLs 11b-c and 11bAc-cAc. ........ 446
**Supplementary References ........ 461**

# Supplementary Figures

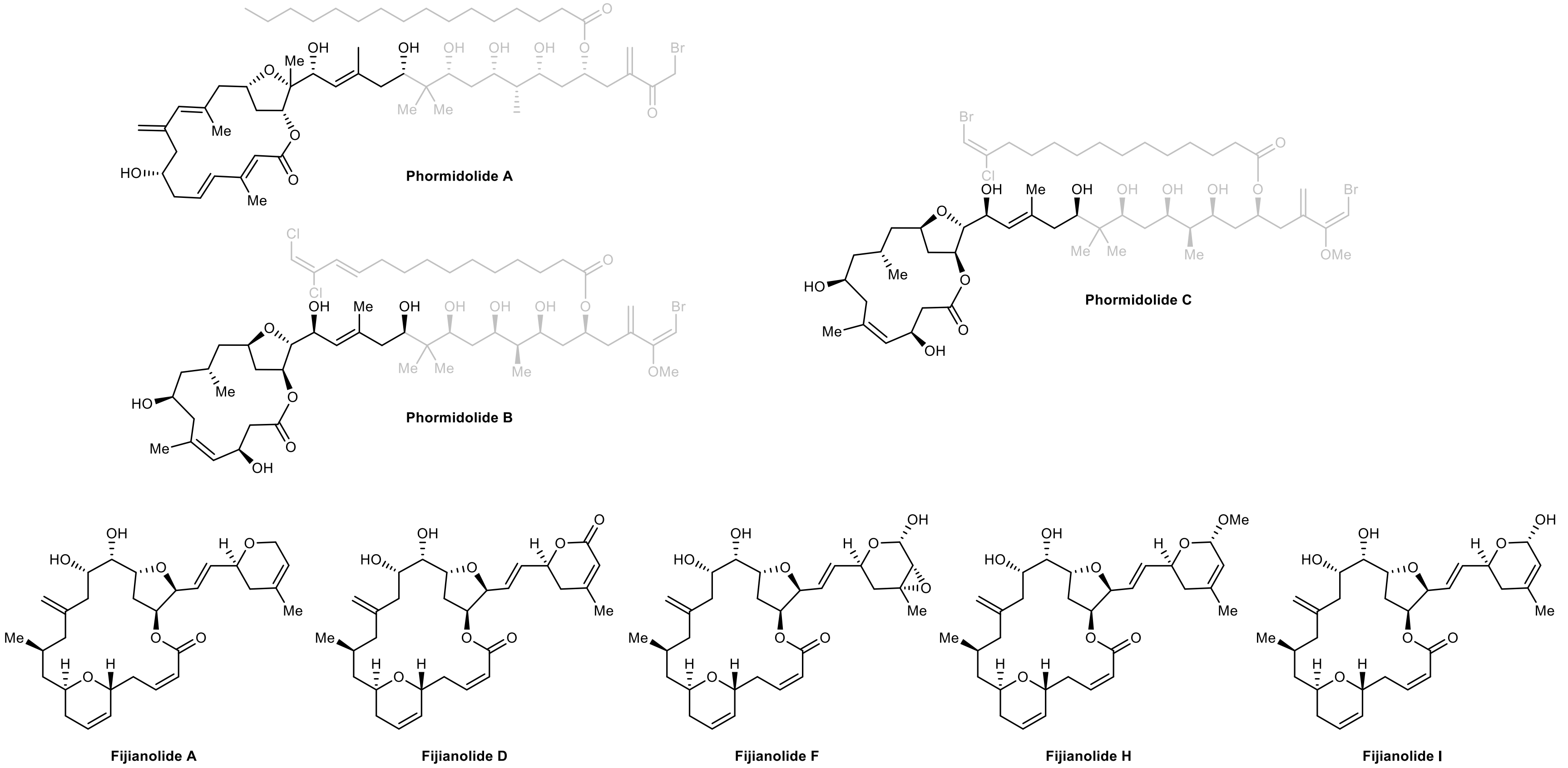


**Supplementary Fig. 1 | THF Macrolide Natural Products Used in Similarity Based Analysis.**
Structures of all natural THF macrolides that were used in similarity scoring analysis and cheminformatics analysis. The cheminformatics analysis used the full structure of all compounds (N=19 natural products). For the 3D similarity analysis some molecules with very flexible large side chains were truncated for simulation purposes, in these cases the portion of the molecule simulated is in black and the portion of the molecule that was removed is indicated in grey. Due to the truncation process, phormidolides B and C are identical for the purposes of the conformational search and 3D similarity comparisons (N=18 natural products).

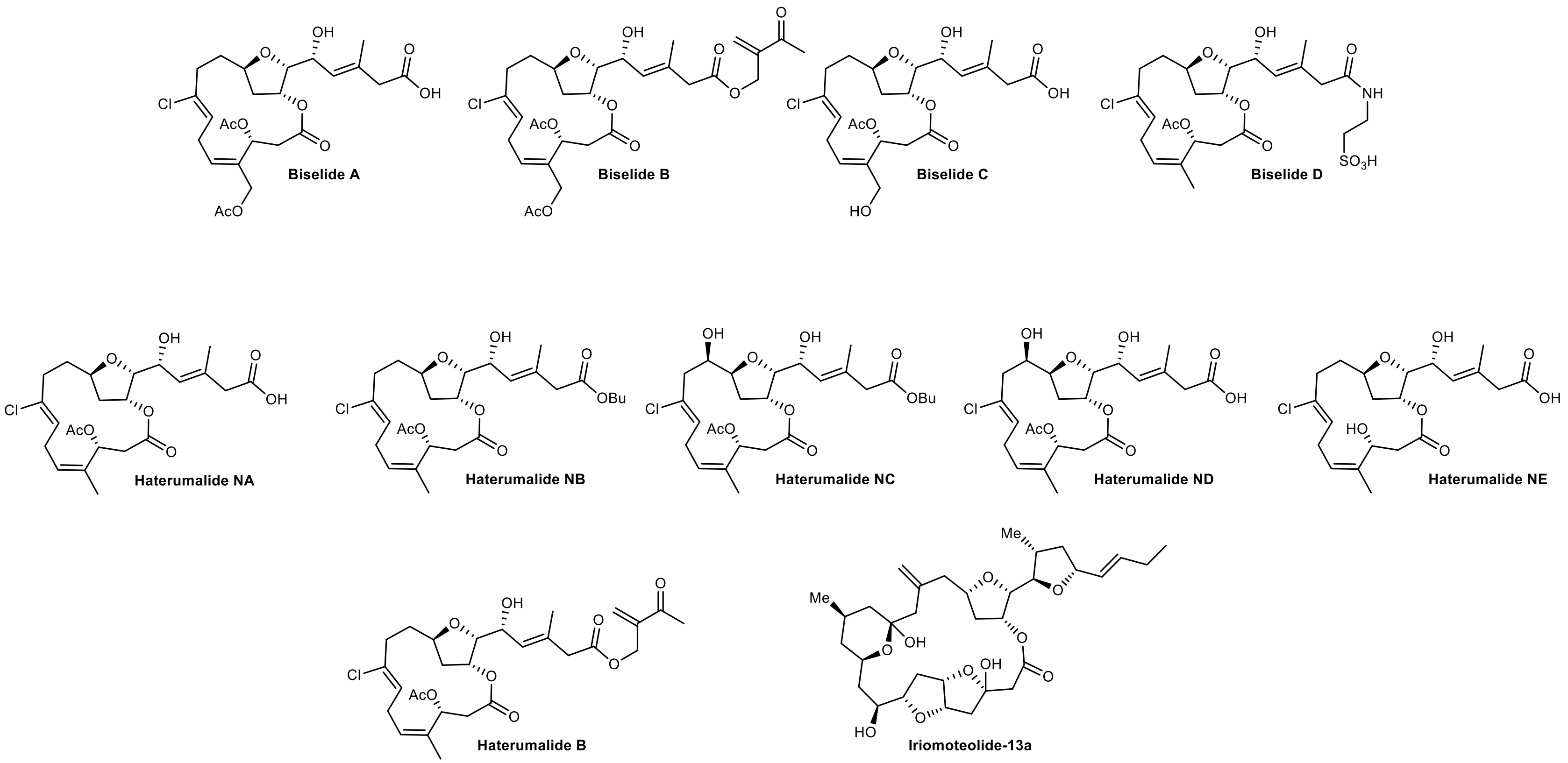


**Supplementary Fig. 1 (cont) | THF Macrolide Natural Products Used in Similarity Based Analysis.**
Structures of all natural THF macrolides that were used in similarity scoring analysis and cheminformatics analysis. The cheminformatics analysis used the full structure of all compounds (N=19 natural products). For the 3D similarity analysis some molecules with very flexible large side chains were truncated for simulation purposes, in these cases the portion of the molecule simulated is in black and the portion of the molecule that was removed is indicated in grey. Due to the truncation process, phormidolides B and C are identical for the purposes of the conformational search and 3D similarity comparisons (N=18 natural products).

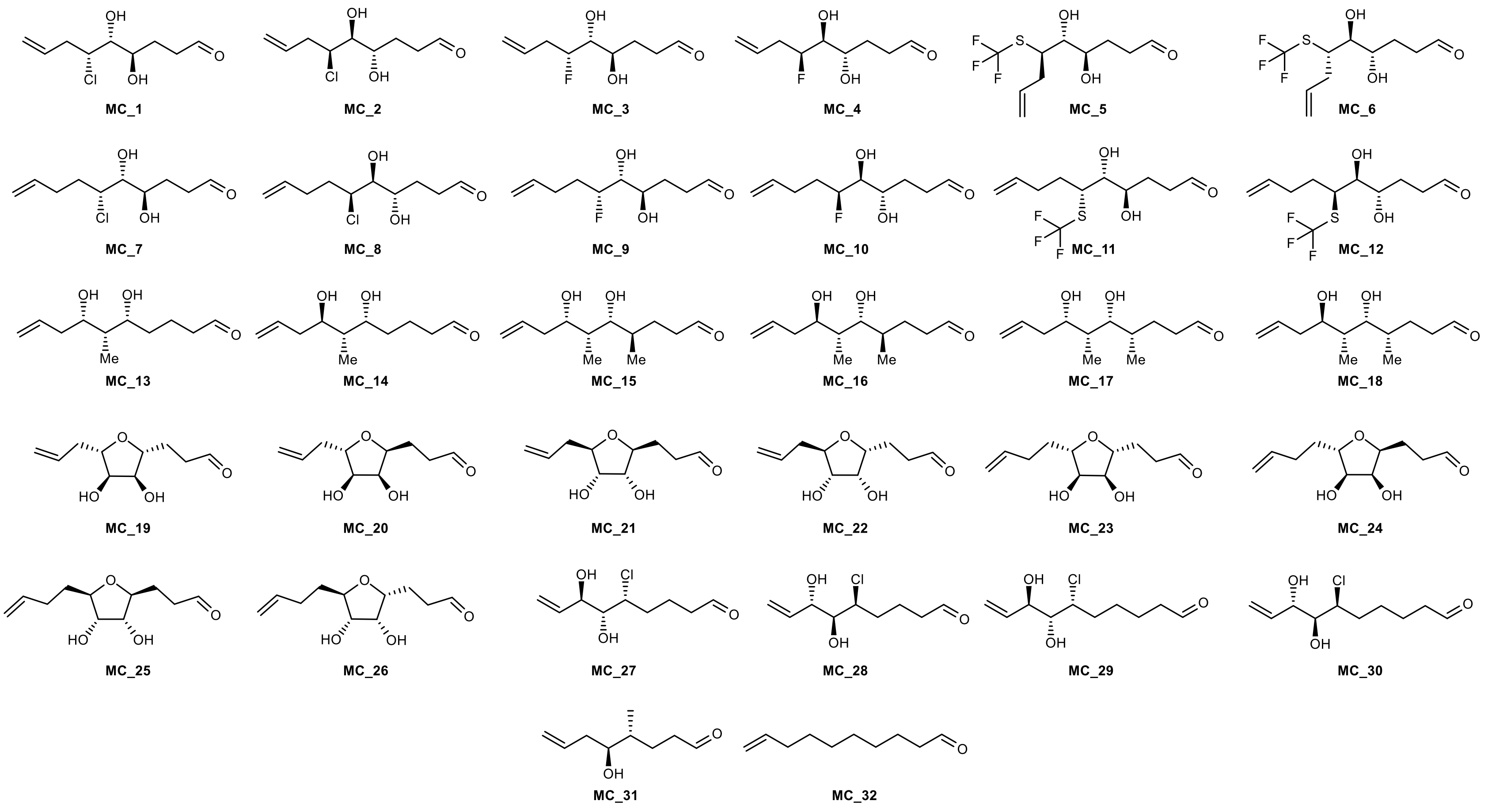


**Supplementary Fig. 2 | Library pML building blocks.**

Structures of the library pML building blocks that were systematically combined in-silico to generate the 3456 library pMLs which were subsequently scored against the NPs.

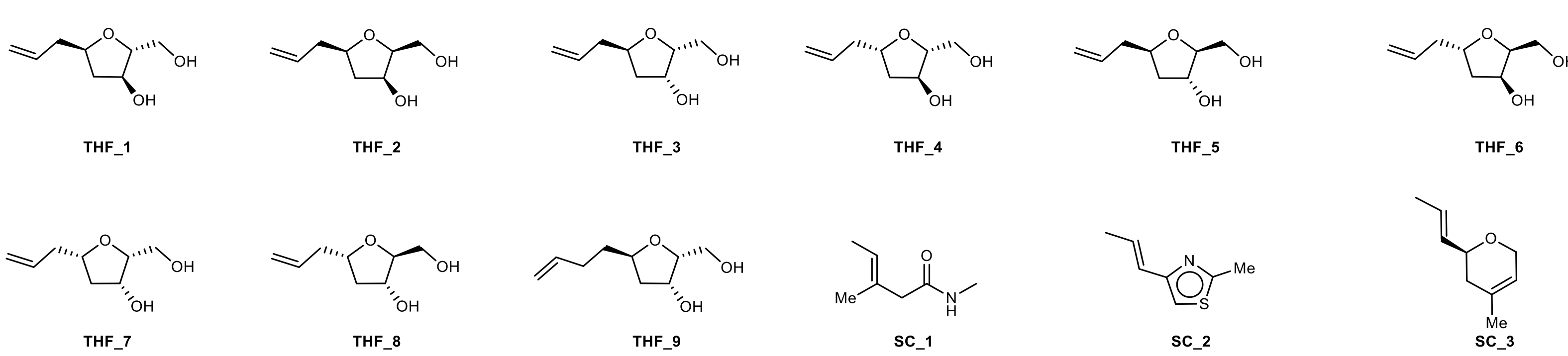


**Supplementary Fig. 2 (cont) | Library pML building blocks**.

Structures of the library pML building blocks that were systematically combined in-silico to generate the 3456 library pMLs which were subsequently scored against the NPs.

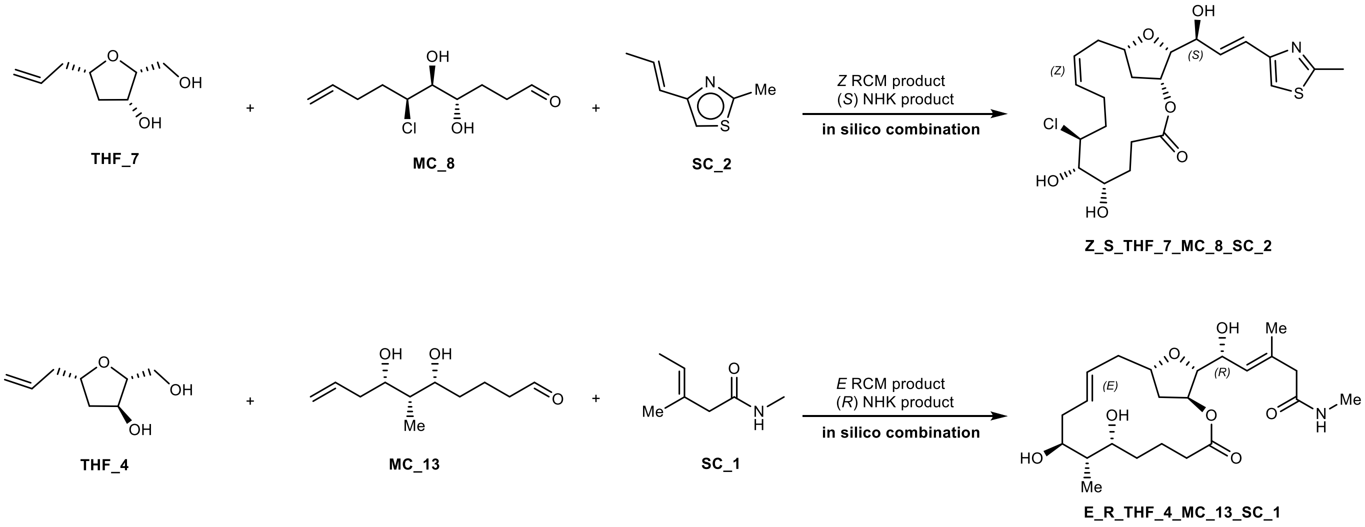


**Supplementary Fig. 3 | Visual examples of combination of building blocks in silico.**

Structures of two representative library pMLs and how they arise from joining of their respective building blocks in silico.

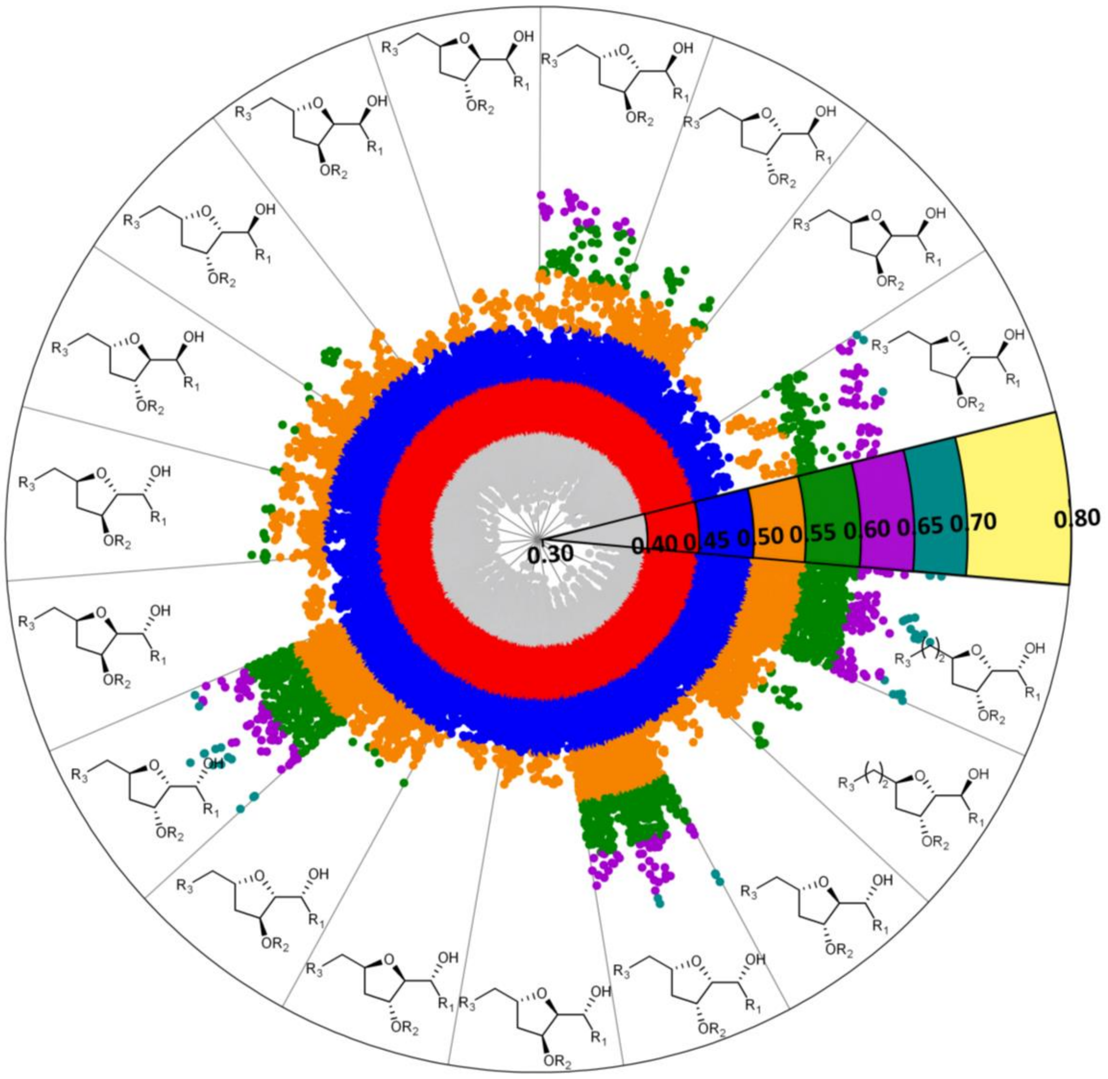


**Supplementary Fig. 4 | Polar plot depicting the $C_s$ (*r*) vs. library pML ID (*θ*) sorted by THF stereochemistry.** Each data point represents a unique comparison between a single library pML (N=3456) and a single natural THF macrolide (N=18) for a total of 62,208 comparisons. The data is sorted along the angular axis ($\theta$) by the stereochemistry present at the NHK centre and THF centres in the library pMLs (indicated by substructures shown in each wedge). Each pair of wedges contain near identical comparisons between library pMLs and natural THF macrolides, with the library pMLs differing only in the stereochemistry present in the pML at the 4 indicated stereocentres. The color scale ranges from 0.30 (grey) to 0.80 (yellow).

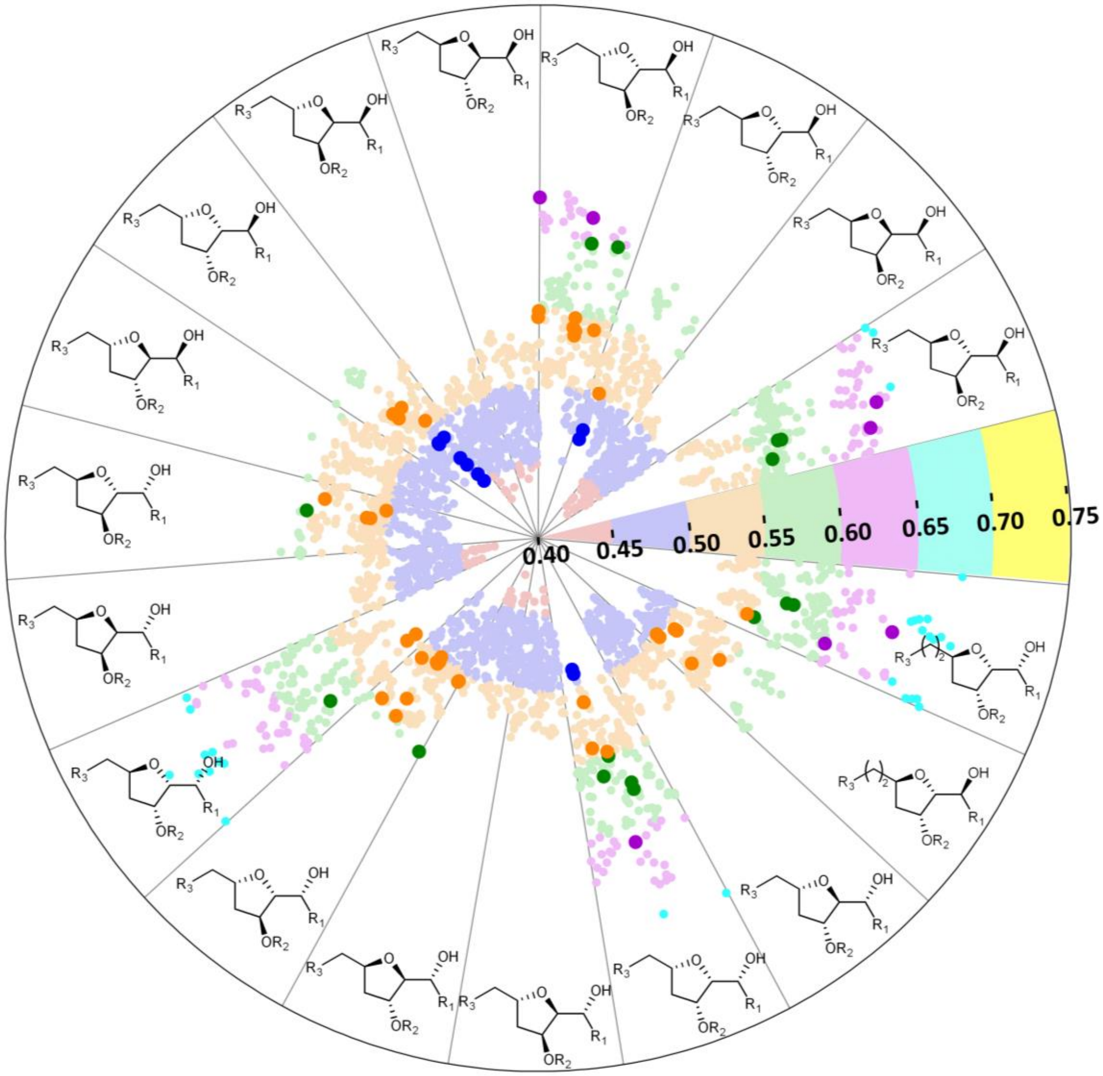


**Supplementary Fig. 5 | Polar plot depicting $C_s^{max}$ (*r*) vs. library pML ID (*θ*) sorted by THF stereochemistry with synthetic compounds highlighted.** Each data point represents a unique comparison between a single library pML (N=3456) and the single natural THF macrolide that it scores best against. The data is sorted along the angular axis (*θ*) by the stereochemistry present at the NHK centre and THF centres in the library pMLs (indicated by substructures shown in each wedge). The color scale ranges from 0.40 (light red) to 0.75 (yellow). Synthetic library pMLs are indicated in bold (excluding acylated pMLs or pMLs lacking a side chain).

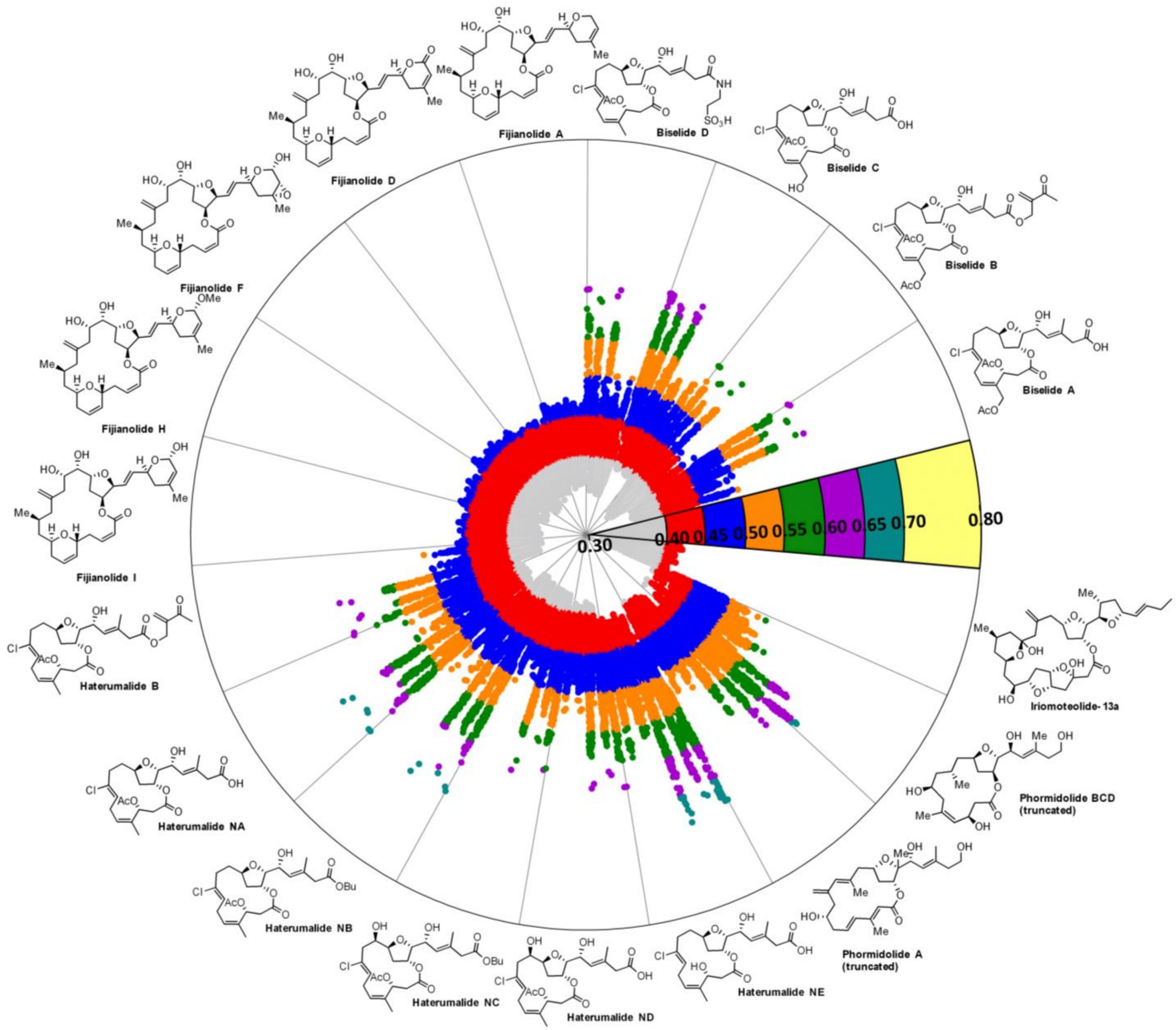


**Supplementary Fig 6 | Polar plot of $C_s$ (*r*) vs. library pML ID (*θ*) sorted by Natural Product.** Each data point represents a unique comparison between a single library pML (N=3456) and a single natural THF macrolide (N=18) for a total of 62,208 comparisons. Each wedge along the angular axis (*θ*) contains the $C_s$ scores for the entire in-silico pML library (N=3456 molecules) against a single natural product (N=1, indicated by its structure and name on the outside of the wedge). The color scale ranges from 0.30 (black/grey) to 0.80 (yellow).

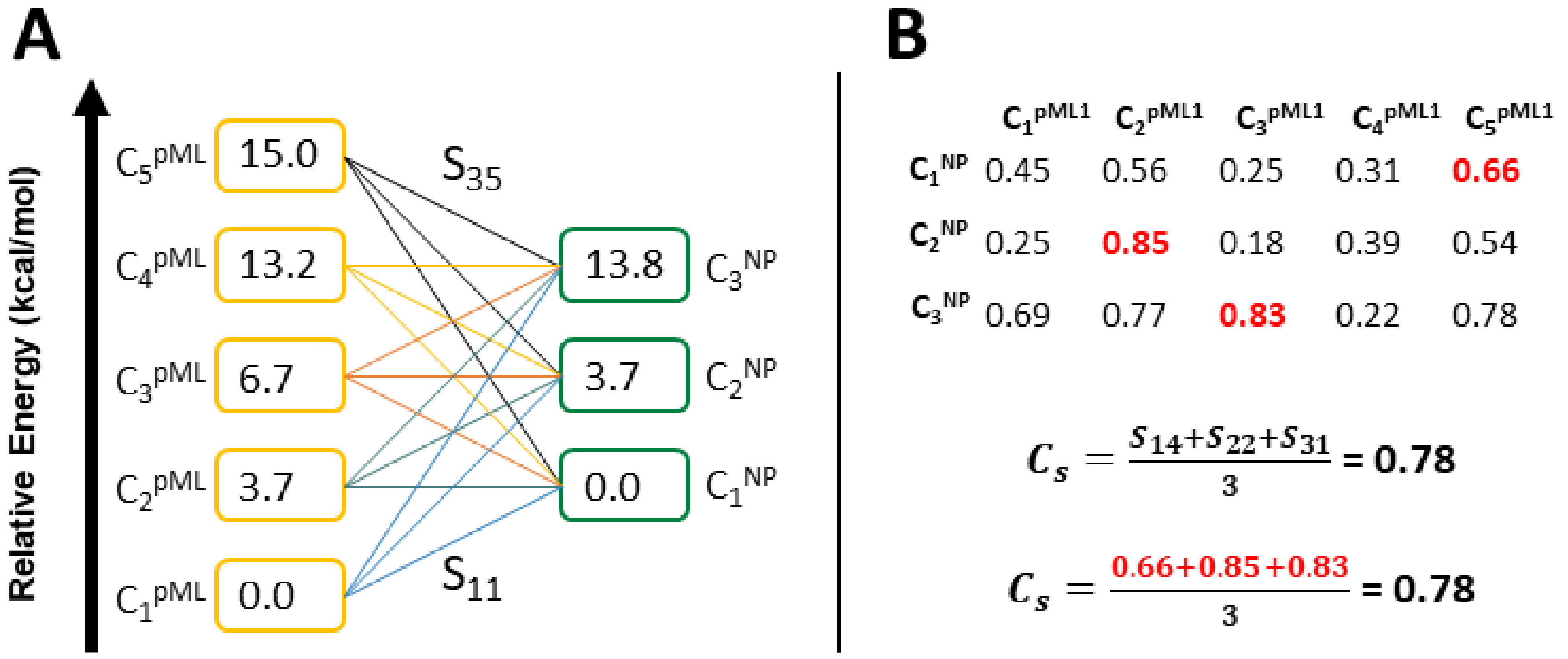


**Supplementary Fig. 7 | Cartoon Depiction of Evaluation of $C_s$ score.**
**A** cartoon depicting a hypothetical case showing all pairwise 3D similarity comparisons between a natural product (green boxes) that has 3 conformers ($C_1^{NP}$-$C_3^{NP}$) within 15 kcal/mol and a library pML molecule (yellow boxes) that has 5 conformers ($C_1^{pML}$-$C_5^{pML}$) within 15 kcal/mol. Numbers within colored boxes indicate the conformer energy relative to the lowest energy conformer identified. **B** example of what the scoring matrix might look like and how $C_S$ would be calculated for this hypothetical case. In each case the value of the maximum scoring pML conformer against each conformer of the NP (indicated in red) is averaged over the number of NP conformers (N=3 for this hypothetical case).

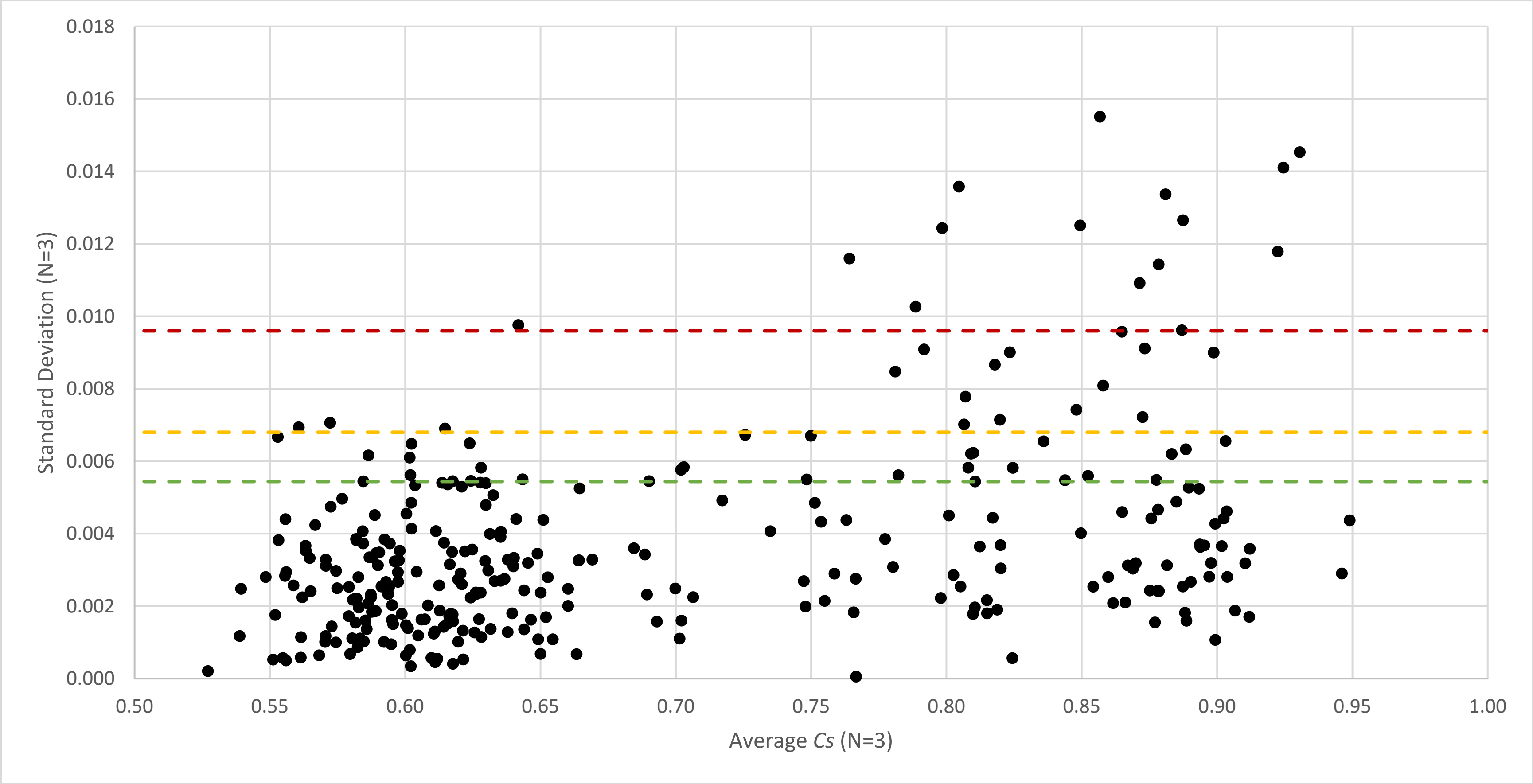


**Supplementary Fig. 8 | Standard Deviations of $C_s$ scores (*Y*-axis) vs. Average $C_s$ scores (*X*-axis) for all 306 comparisons among the 18 simulated NPs for N=3 replicates of 2500 MD iterations.**

95%, 90%, and 80% of the data lies below the red (*Y*=0.0096), orange (*Y*=0.0068), and green (*Y*=0.0054) lines respectively.

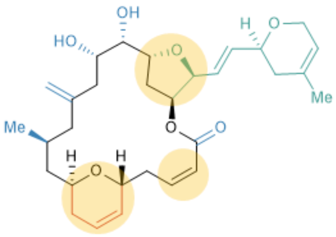


Fijianolide A

| | |
|---|---|
| # Ring HA | 18 |
| % total HA | 49 % |
| # Peripheral HA (Blue) | 5 |
| % total HA | 14 % |
| # Substituent HA | 14 |
| % total HA | 37 % |
| # Substituents (Teal and Red) | 2 |
| Ring Atoms per Peripheral Group | 3.6 |
| Degrees of Unsaturation in Ring (Yellow) | 3 |
| HA Composition of MCs (% Polar [N,O]/ % Nonpolar [C, S, Cl, Br, F]) | |
| Total | 19 % / 81 % |
| Peripheral | 60 % / 40 % |
| Substituent | 14 % / 86 % |

**Supplementary Fig. 9 | Example Calculation of HA (heavy-atom) parameters for Fijianolide A.**
HA regions of the example molecule (Fijianolide A) are highlighted as follows: macrocycle backbone (black), peripheral HA (blue), large and small substituents (teal and red respectively), yellow (rigidifying groups).

| building block | basis for stereochemical assignment |
|---|---|
| 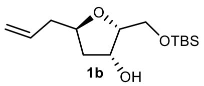 **1b** | agreement of spectroscopic and optical rotation data for known intermediate **S21** with literature; nOes on acetonide **S22** ***see Supplementary Figure 11*** |
| 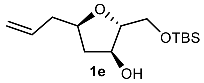 **1e** | diastereospecific $S_N2$ (Mitsunobu esterification) on **1b** followed by hydrolysis to furnish **1e** ***see Supplementary Figure 11*** |
| 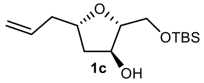 **1c** | agreement of spectroscopic and optical rotation data for known intermediate **S3** with literature; Agreement of spectroscopic data for **S1** and **S2** with literature ***see Supplementary Figure 12*** |
| 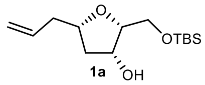 **1a** | diastereospecific $S_N2$ (Mitsunobu esterification) on **1c** followed by hydrolysis to furnish **1a** ***see Supplementary Figure 12*** |
| 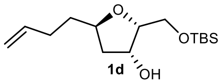 **1d** | nOes on **S14**; **1d** produced via homologation of intermediate **S25** (derived from known compound **S21**) had identical spectroscopic and optical rotation data to **1d** produced via **S14** ***see Supplementary Figure 11*** |
| 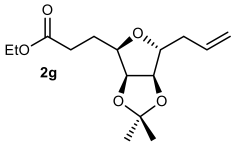 **2g** | nOes on **S22**; agreement of spectroscopic and optical rotation data for known intermediate **S21** with literature ***see Supplementary Figure 11*** |
| 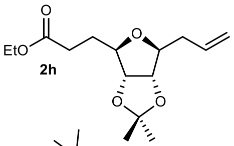 **2h** | agreement of spectroscopic and optical rotation data for known intermediate **S3** with literature ***see Supplementary Figure 12*** |
| 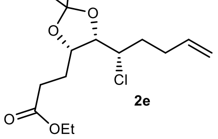 **2e** | reduction, diastereospecific cyclization ($S_N2$ displacement), and protection of intermediate **S11** to furnish **S14;** nOes on **S14**; **1d** produced via **S25** (derived from known compound **S21**) had identical spectroscopic and optical rotation data to **1d** produced via **S11** (common intermediate for synthesis of **2e**); nOes on **2e** ***see Supplementary Figure 11*** |

| building block | basis for stereochemical assignment |
|---|---|
| 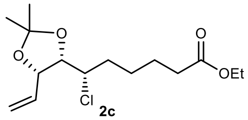 **2c** | reduction, diastereospecific cyclization ($S_N2$ displacement), and protection of intermediate **S11** to furnish **S14;** nOes on **S14**; **1d** produced via **S25** (derived from known compound **S21**) had identical spectroscopic and optical rotation data to **1d** produced via **S11** (common intermediate for synthesis of **2c**); nOes on **2c** ***see Supplementary Figure 11*** |
| 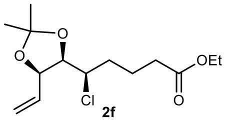 **2f** | agreement of spectroscopic and optical rotation data for known compound **S3** with literature which was derived from **S1** (common intermediate in synthesis of **2f**) via reduction/diastereospecific $S_N2$; nOes on **2f** ***see Supplementary Figure 12*** |
| 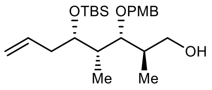 **2a** | enantiopure chiral pool starting material; use of enantiopure chiral auxiliary; agreement of spectroscopic and optical rotation data for known enantiomer of **S46** (**ent-S46**) which is an intermediate in the synthesis of **2a** ***see Supplementary Figure 13*** |
| 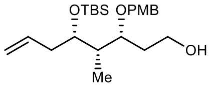 **2b** | use of enantiopure chiral auxiliary; agreement of spectroscopic and optical rotation data for **S31** which is an intermediate in the synthesis of **2b**; agreement of spectroscopic data for **S36** with literature which is an intermediate in the synthesis of **2b** ***see Supplementary Figure 14*** |
| 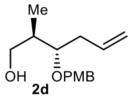 **2d** | use of enantiopure catalyst; agreement of spectroscopic and optical rotation data for **2d** with literature data for **ent-2d** ***see Supplementary Figure 15*** |
| 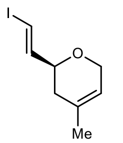 **3b** | use of enantiopure catalyst; agreement of spectroscopic and optical rotation data for **3b** with literature data ***see Supplementary Figure 16*** |

**Supplementary Fig. 10 | Summary of Stereochemical Assignments for All Chiral Building Blocks.**

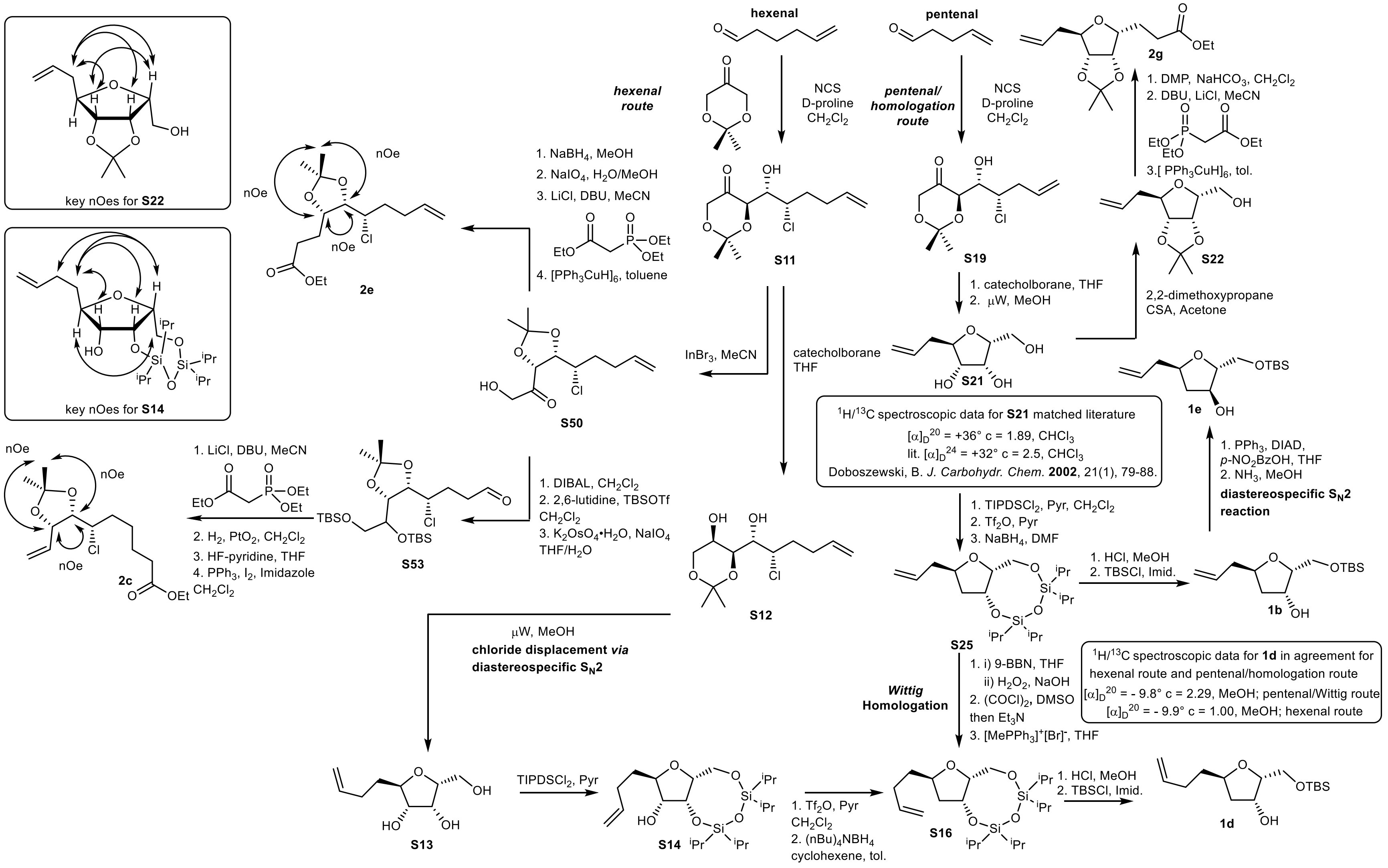


**Supplementary Fig. 11 | Evidence for Stereochemistry of 1b, 1e, 1d, 2g 2c, 2e.**

Abbreviations: TBS = tert-butyldimethylsilyl, THF = tetrahydrofuran, DBU = 1,8-Diazabicyclo [5.4.0]undec-7-ene, DIBAL = diisobutylaluminum hydride, NCS = N-chlorosuccinimide, $TIPDSCl_2$ = 1,3-Dichloro-1,1,3,3-tetraisopropyldisiloxane, DMP = Dess-

Martin periodinane, CSA = camphorsulfonic acid, DIAD = Diisopropyl azodicarboxylate, Pyr = Pyridine, $Tf_2O$ = Trifluoromethanesulfonic anhydride, TBSCl = tert-butyldimethylsilyl chloride, TBSOTf = tert-butyldimethylsilyl trifluoromethanesulfonate, 9-BBN = 9-Borabicyclo(3.3.1)nonane, tol. = toluene.

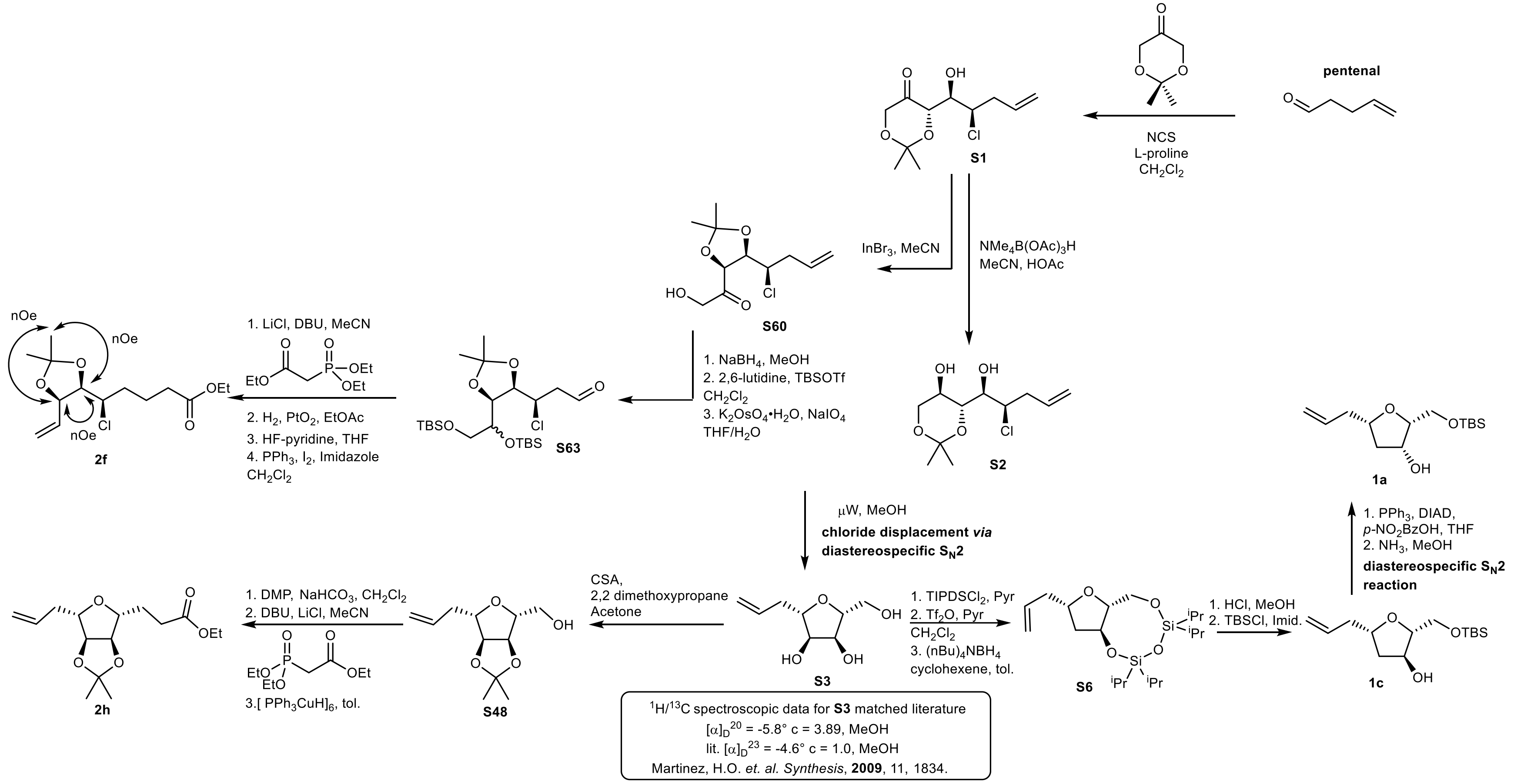


**Supplementary Fig. 12 | Evidence for Stereochemistry of 1a, 1c, 2f, 2h.**

Abbreviations: TBS = tert-butyldimethylsilyl, THF = tetrahydrofuran, DBU = 1,8-Diazabicyclo [5.4.0]undec-7-ene, DIBAL = diisobutylaluminum hydride, NCS = N-chlorosuccinimide, $TIPDSCl_2$ = 1,3-Dichloro-1,1,3,3-tetraisopropyldisiloxane, DMP = Dess-Martin periodinane, CSA = camphorsulfonic acid, DIAD = Diisopropyl azodicarboxylate, Pyr = Pyridine, $Tf_2O$ =

Trifluoromethanesulfonic anhydride, TBSCl = tert-butyldimethylsilyl chloride, TBSOTf = tert-butyldimethylsilyl trifluoromethanesulfonate.

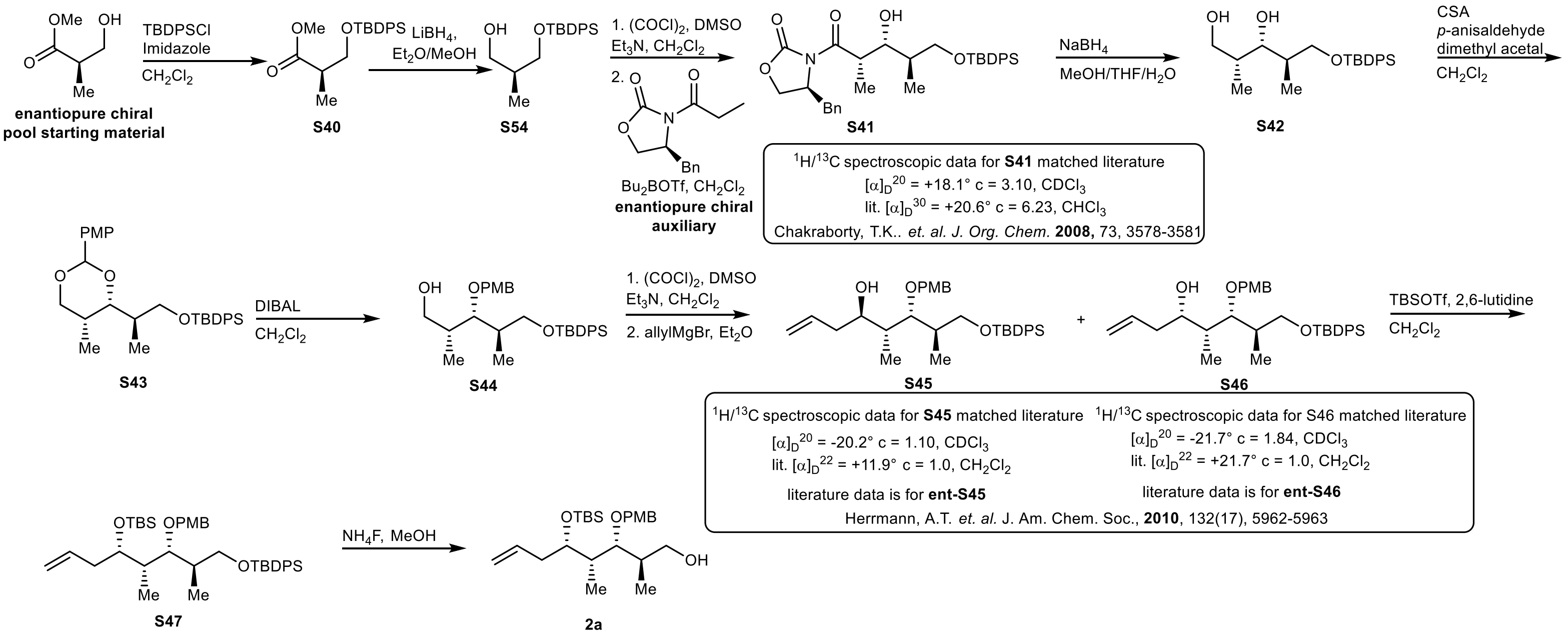


**Supplementary Fig. 13 | Evidence for Stereochemistry of 2a.**

Abbreviations: TBDPS = tert-butyldiphenylsilyl, TBDPSCl = tert-butyldiphenylsilyl chloride, THF = tetrahydrofuran, CSA = camphorsulfonic acid, PMB = *para*-methoxybenzyl, DIBAL = diisobutlyaluminum hydride, DMSO = dimethylsulfoxide, TBSOTf = tert-butyldimethylsilyl trifluoromethylsulfonate, TBS = tert-butyldimethylsilyl.

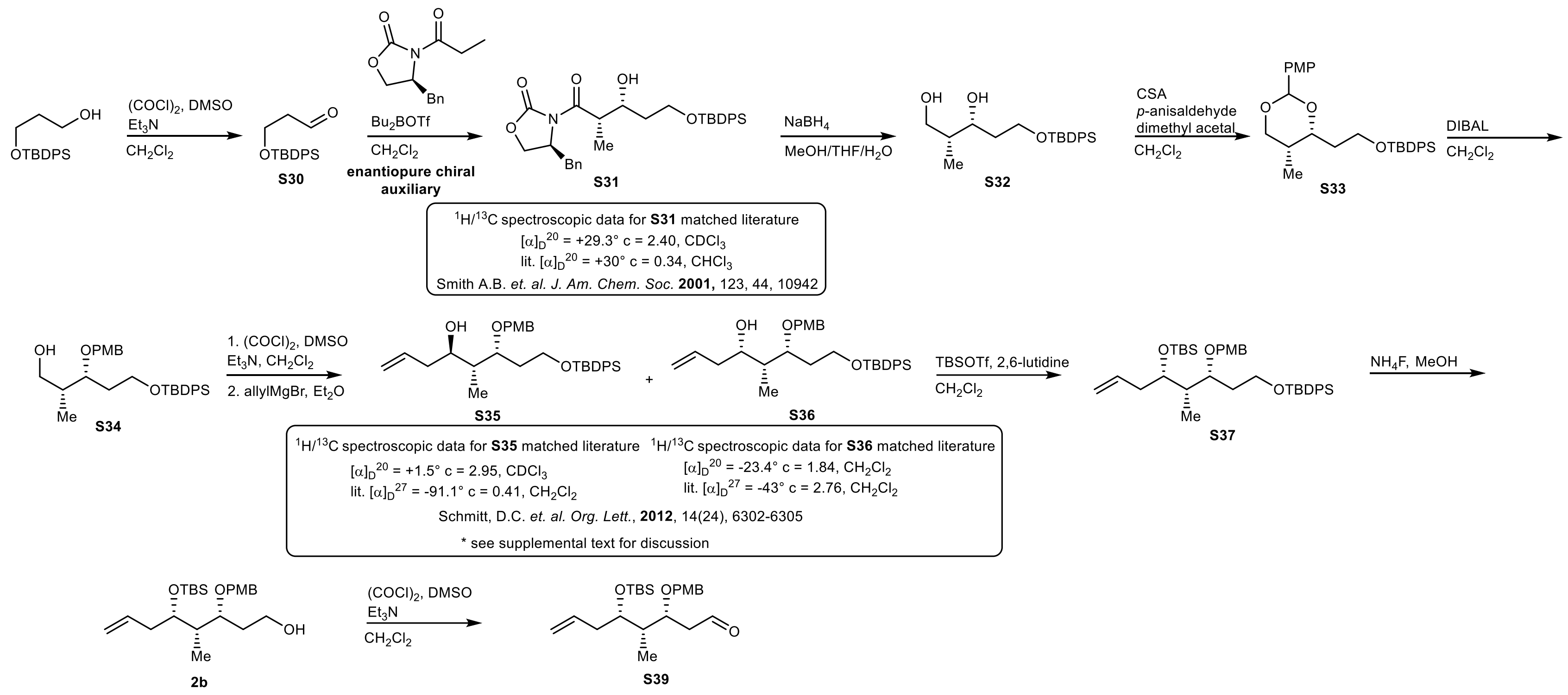


**Supplementary Fig. 14 | Evidence for Stereochemistry of 2b.**

Abbreviations: TBDPS = tert-butyldiphenylsilyl, Bn = benzyl, DMSO = dimethylsulfoxide, THF = tetrahydrofuran, CSA = camphorsulfonic acid, PMP = *para*-methoxyphenyl, DIBAL = diisobutylaluminum hydride, PMB = *para*-methoxybenzyl, TBSOTf = tert-butyldimethylsilyl trifluoromethanesulfonate.

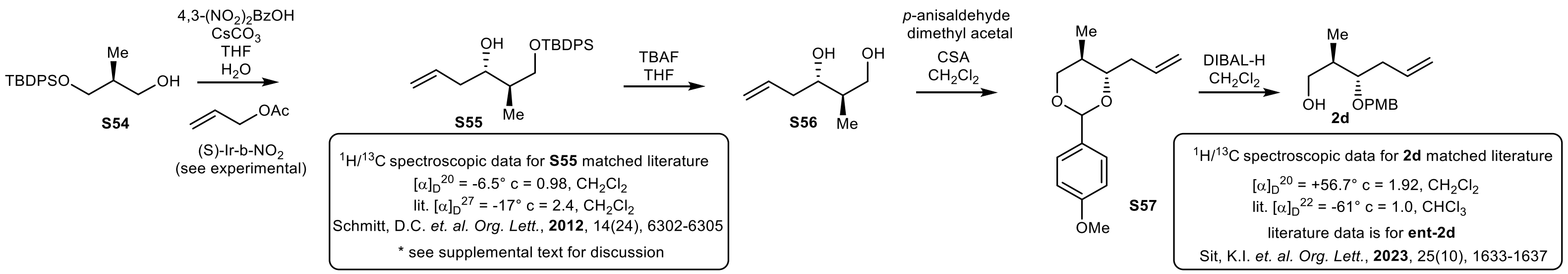


**Supplementary Fig. 15 | Evidence for Stereochemistry of 2d.**
Abbreviations: TBDPS = tert-butyldiphenylsilyl, THF = tetrahydrofuran, TBAF = tetra-n-butylammonium fluoride, CSA = camphorsulfonic acid, DIBAL-H = diisobutylaluminum hydride, PMB = *para*-methoxybenzyl

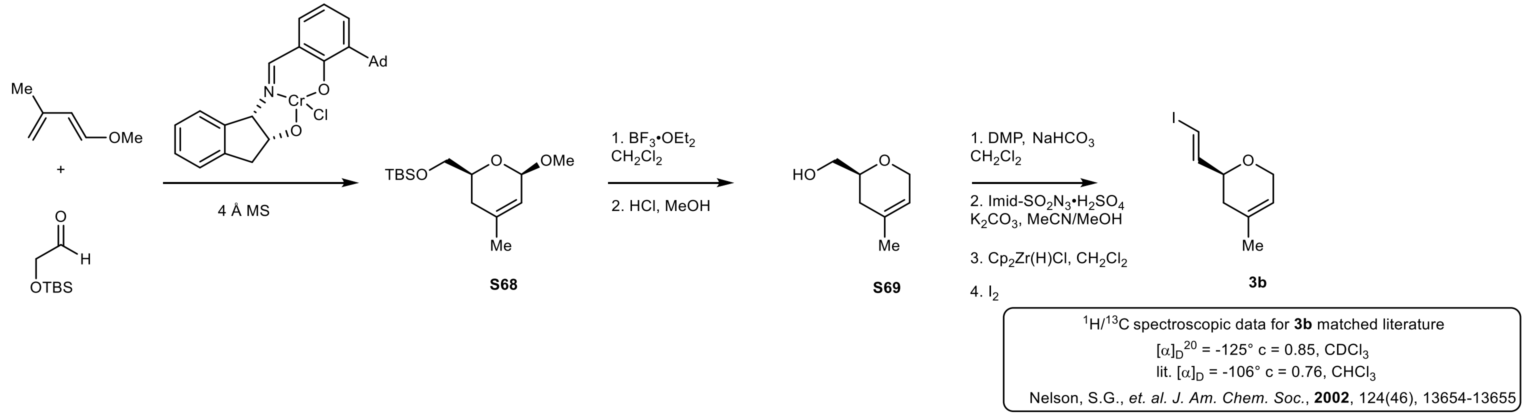


**Supplementary Fig. 16 | Evidence for Stereochemistry of 3b.**
Abbreviations: TBS = tert-butyldimethylsilyl, Ad = adamantyl, 4 Å MS = 4 Ångstrom molecular sieves, DMP = Dess-Martin periodinane, Imid-$SO_2N_3$•$H_2SO_4$ = imidazolesulfonylazide hydrogensulfate.

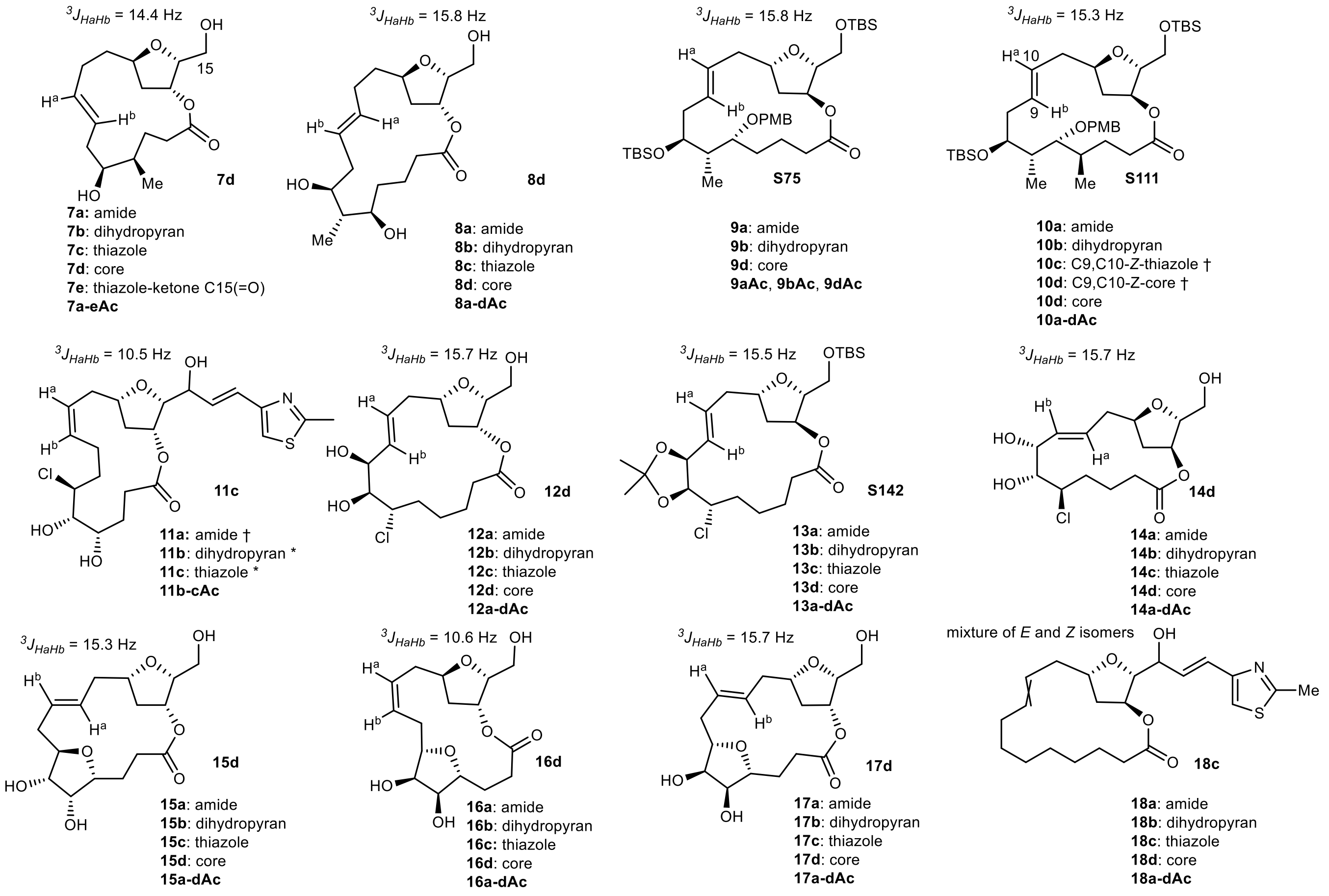


**Supplementary Fig. 17 | Evidence for E/Z configuration in pMLs.**

The configuration of the alkene formed via ring closing metathesis was confirmed by measurement of the $^3J$ $^1$H-$^1$H coupling. A single representative analogue is shown for each class of macrocycle with its corresponding $J$ value. The alkene was assigned as $E$ if the $^3J$ $^1$H-$^1$H for the alkenyl protons was >14 Hz or $Z$ if it was <11 Hz. The integrity of the macrolactone for all final pMLs was verified by detection of a $^1$H-$^{13}$C HMBC correlation from the oxymethine proton on the THF to the carbonyl of the lactone.

**Supplementary Fig. 18A | Cell painting fingerprint for Fijianolide A**

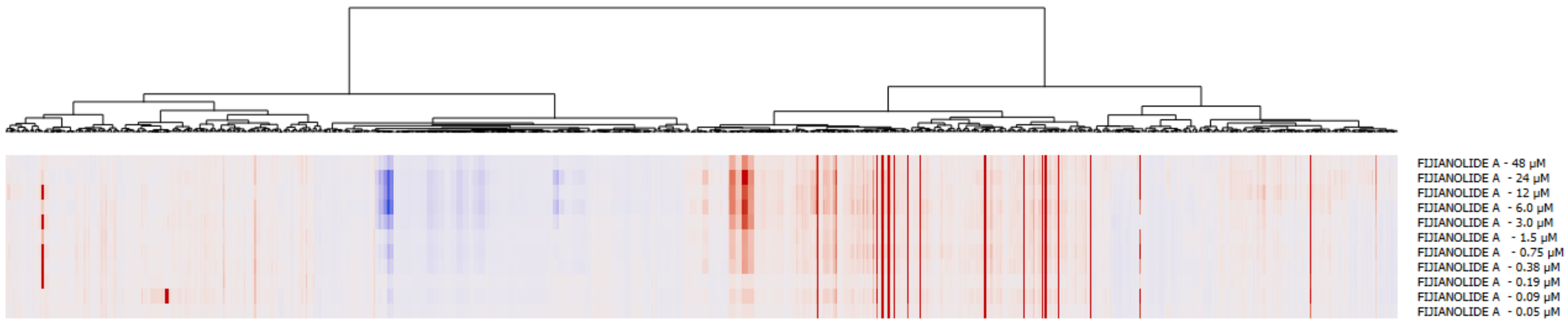


**Supplementary Fig. 18B | Cell painting activity threshold plot for Fijianolide A**

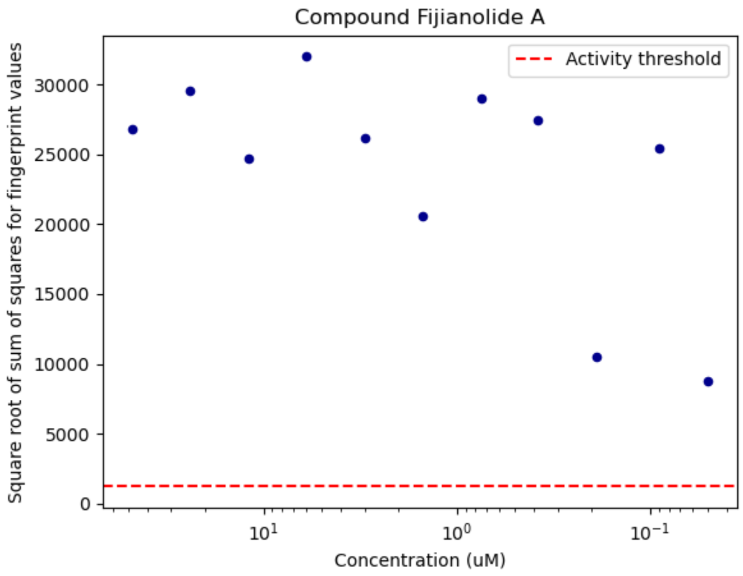

**Supplementary Fig | 19A. Cell painting fingerprint for Fijianolide B**

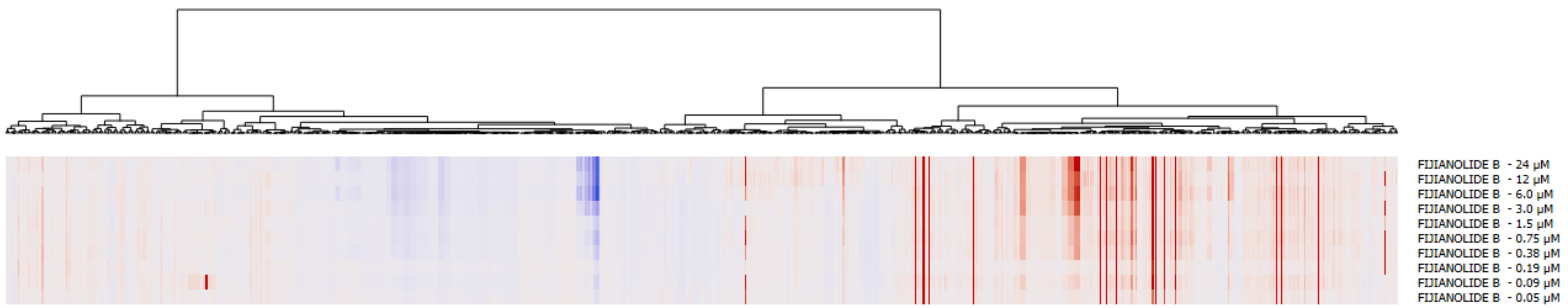


**Supplementary Fig. 19B | Cell painting activity threshold plot for Fijianolide B**

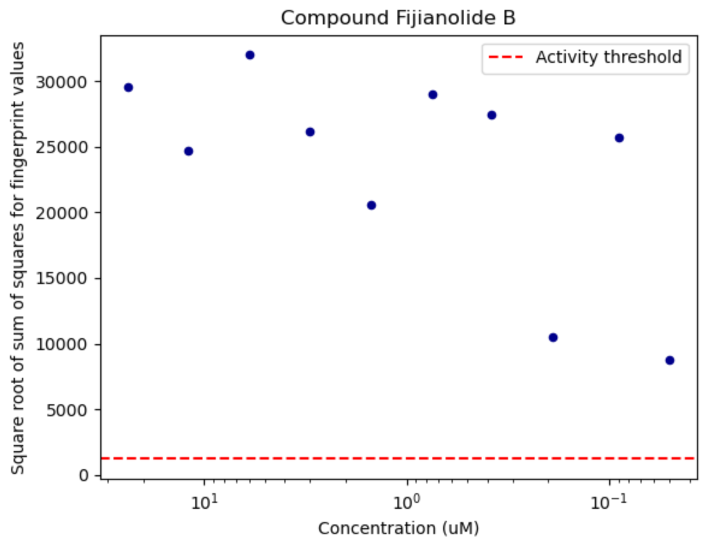

**Supplementary Fig. 20A | Cell painting fingerprint for 7e**

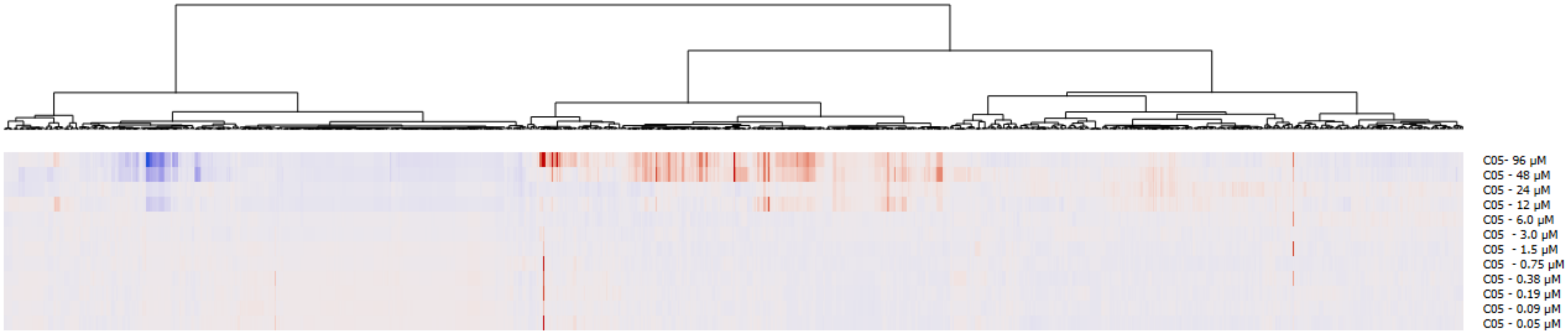


**Supplementary Fig. 20B | Cell painting activity threshold plot for 7e**

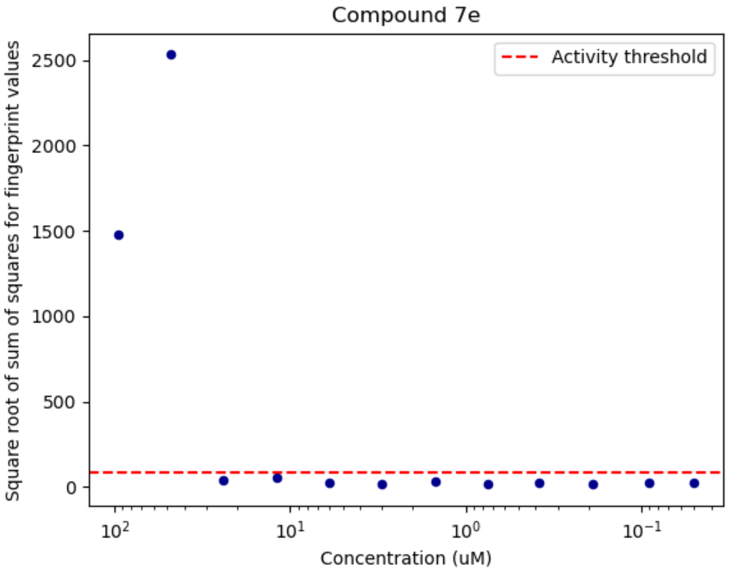

**Supplementary Fig. 21A | Cell painting fingerprint for 7eAc**

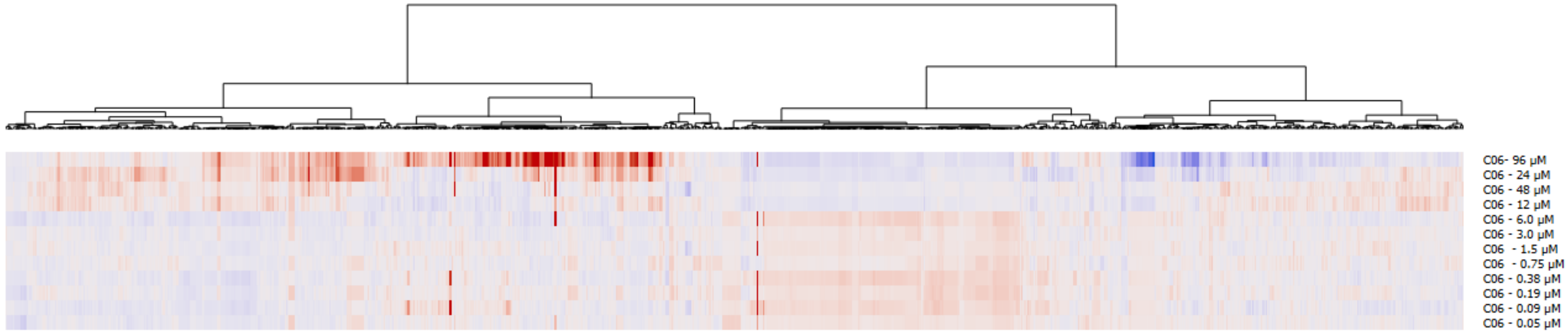


**Supplementary Fig. 21B | Cell painting activity threshold plot for 7eAc**

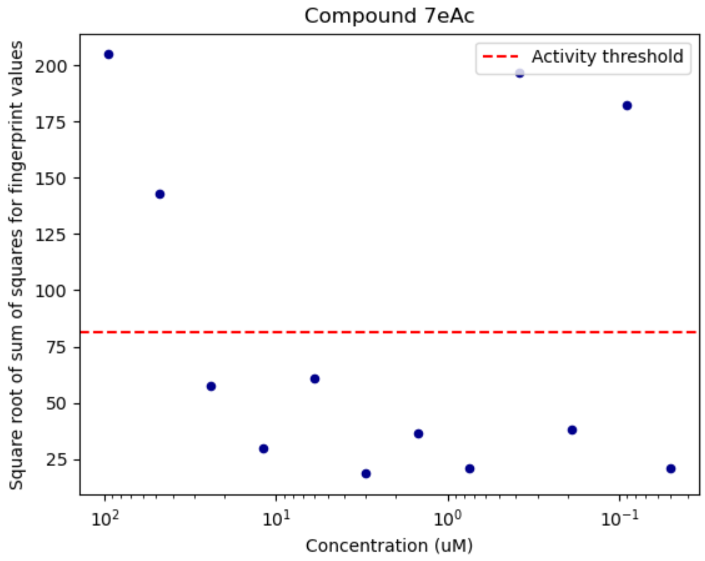

**Supplementary Fig. 22A | Cell painting fingerprint for 11bAc**

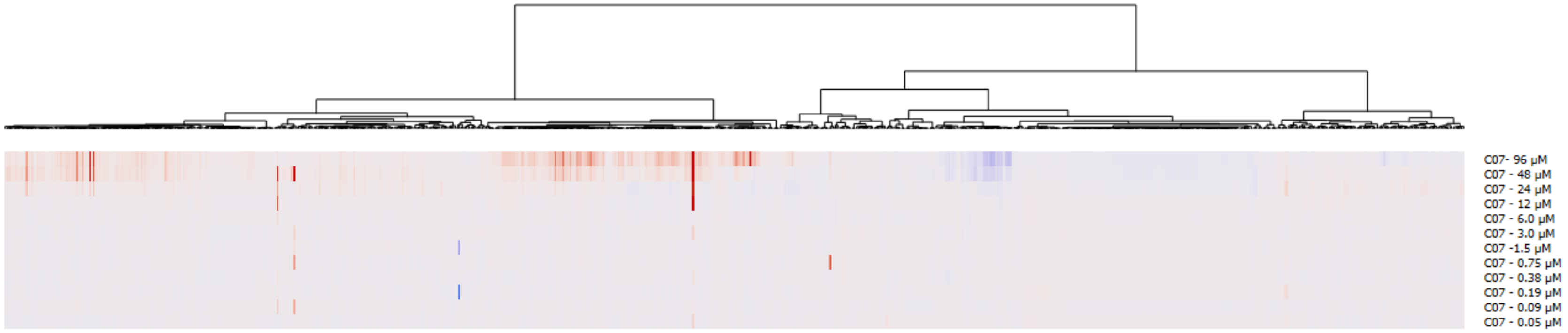


**Supplementary Fig. 22B | Cell painting activity threshold plot for 11bAc**

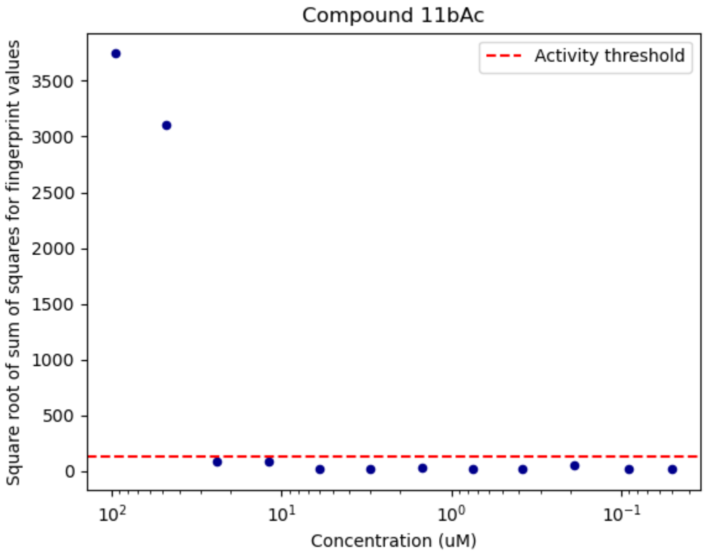

**Supplementary Fig. 23A | Cell painting fingerprint for 12bAc**

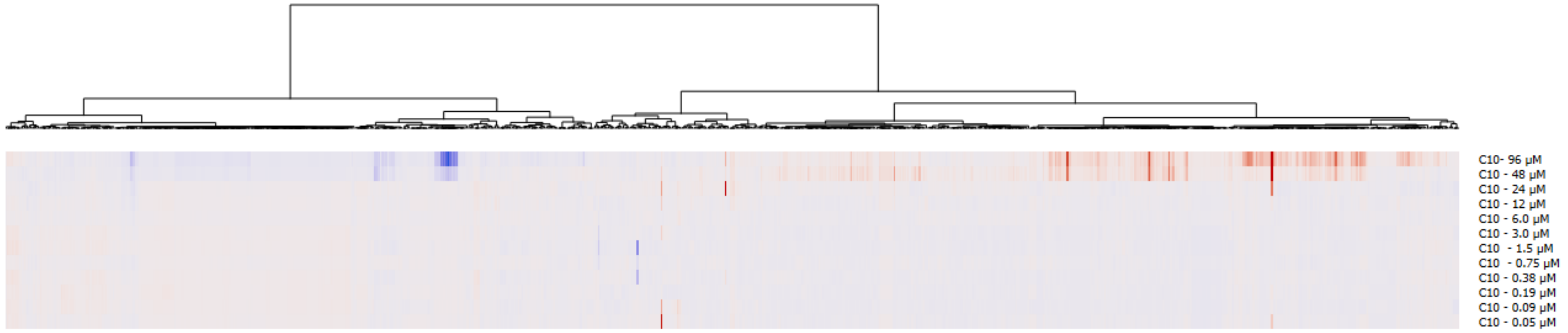


**Supplementary Fig. 23B | Cell painting activity threshold plot for 12bAc**

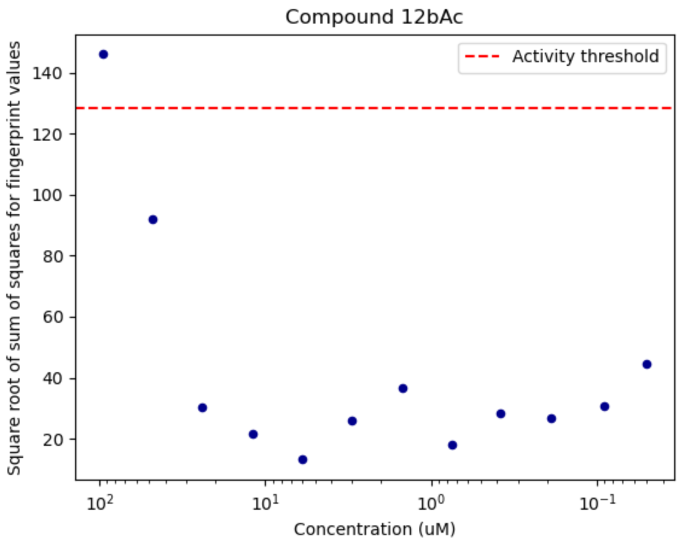

**Supplementary Fig 24A | Cell painting fingerprint for 18b**

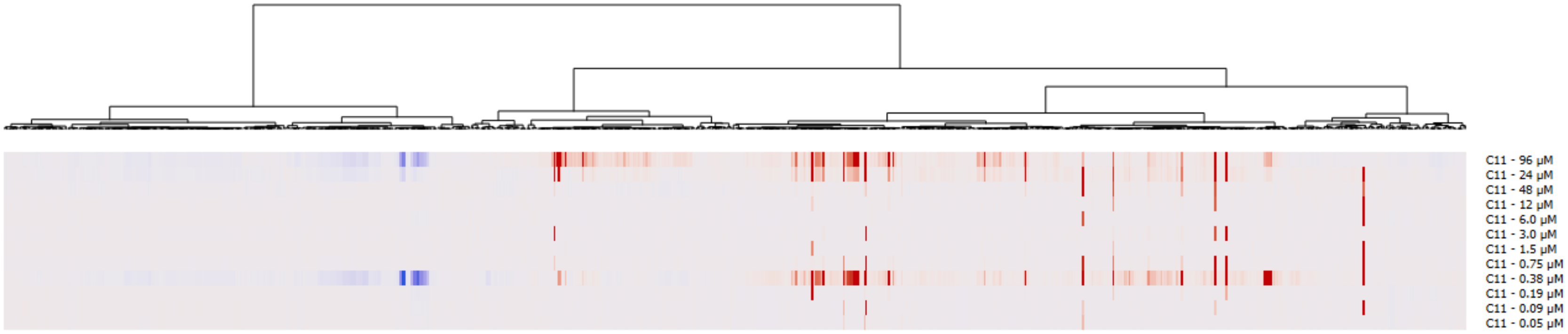


**Supplementary Fig. 24B | Cell painting activity threshold plot for 18b**

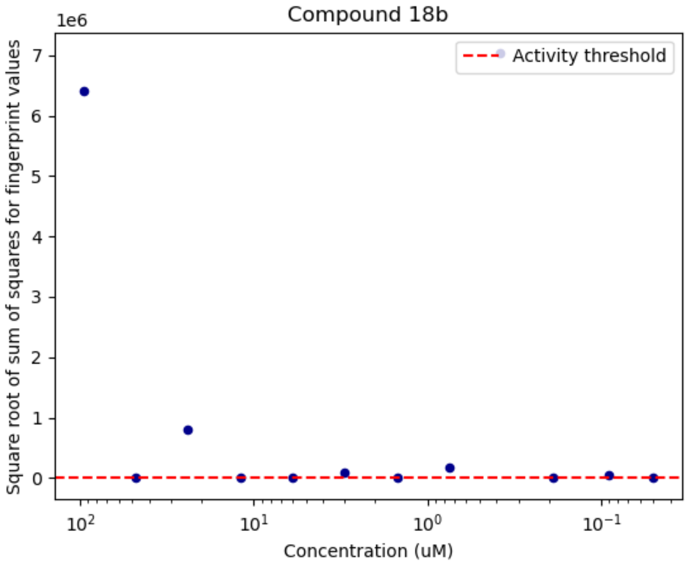

## Supplementary Fig. 25A | Cell painting fingerprint for 18bAc

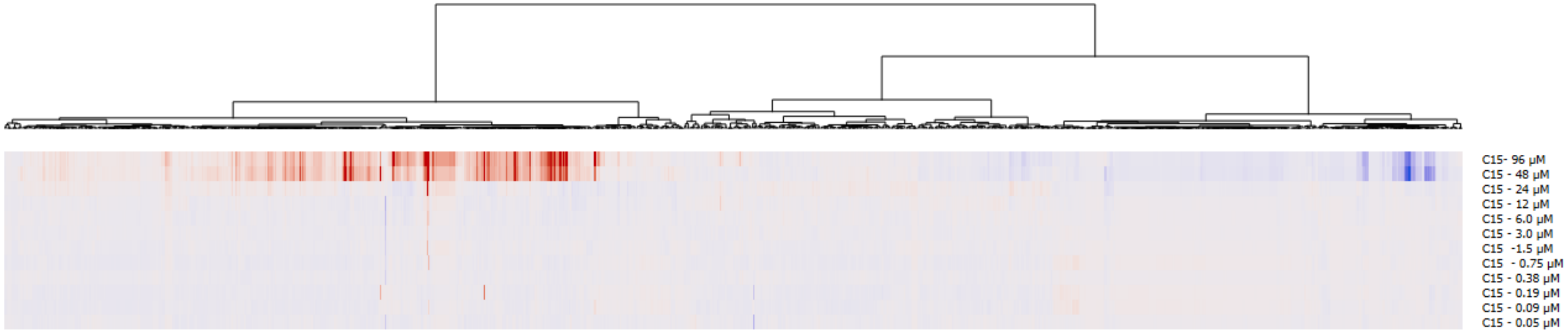


## Supplementary Fig. 25B | Cell painting activity threshold plot for 18b

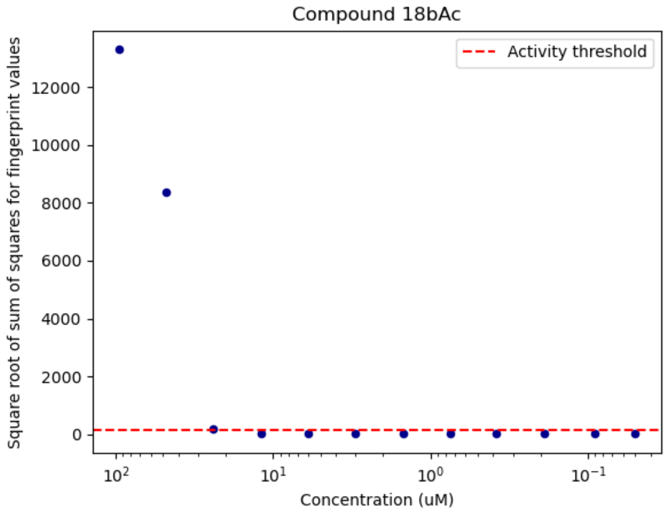

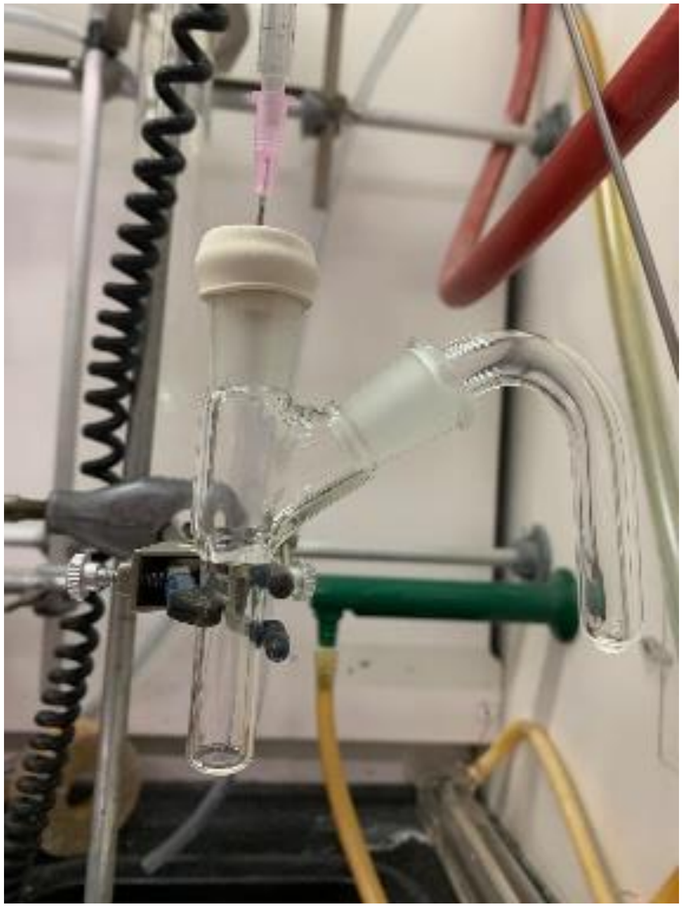

**Supplementary Fig. 26 | Custom glass apparatus for Nozaki-Hiyama-Kishi reactions.**

# Supplementary Tables

| | Average ± *SD* | Average ± *SD* | Average ± *SD* |
|---|---|---|---|
| **Property (Units)** | **in-silico pML Library* (N=3456)** | **Synthesized pML Library (N=170)†** | **NP reference compounds (N=19)††** |
| **Mol. Weight (g/mol)** | 461.9 ± 29.9 | 478.0 ± 82.5 | 618.7 ± 215.6 |
| **cLogP** | 2.1 ± 1.0 | 2.8 ± 1.3 | 4.5 ± 2.8 |
| **Polar Surface Area (Å$^2$)** | 113.7 ± 12.6 | 112.2 ± 23.3 | 138.9 ± 34.7 |
| **# Hydrogen Bond Donor** | 3.2 ± 0.6 | 1.6 ± 1.5 | 2.9 ± 1.8 |
| **# Hydrogen Bond Acceptor** | 7.6 ± 0.8 | 8.2 ± 2.0 | 9.1 ± 2.0 |
| **# Chiral Centres** | 7.6 ± 0.9 | 6.9 ± 1.5 | 8.1 ± 3.3 |
| **# Rotatable Bonds** | 3.5 ± 0.6 | 4.3 ± 1.7 | 9.0 ± 9.4 |
| **# Degrees of Unsaturation in Ring** | 2.3 ± 0.4 | 2.2 ± 0.4 | 3.0 ± 0.5 |

**Supplementary Table 1 | Cheminformatics properties of in-silico pML library, synthesized pML library, and NP reference compounds.**

* Includes all virtual THF-pMLs containing a side chain (e.g. amide, thiazole, or dihydropyran). Does not include any macrocyclic cores (macrocycles lacking a side chain), or acylated macrocycles. † Includes all synthesized pMLs (macrocycles lacking a side chain, macrocycles with a side chain, and acylated macrocycles). †† The full set of natural products depicted in Supplementary Fig. 1 without any truncation.

| | **Average ± *SD*** | **Average ± *SD*** | **Average ± *SD*** |
|---|---|---|---|
| **Property** | **in-silico Library (N=3456)*** | **Synthesized pML library (N=170)†** | **NP reference compounds (N=19)††** |
| **# Ring HA** | 14.7 ± 0.6 | 14.7 ± 0.6 | 15.4 ± 2.0 |
| **% total HA** | 46.5 ± 2.4 | 45.5 ± 8.8 | 39.1 ± 9.2 |
| **# Peripheral HA** | 3.1 ± 1.4 | 2.2 ± 1.3 | 3.9 ± 1.2 |
| **% total HA** | 9.9 ± 4.6 | 6.9 ± 4.5 | 9.7 ± 3.1 |
| **# Substituent HA** | 14.0 ± 2.2 | 16.6 ± 6.0 | 22.6 ± 12.8 |
| **% total HA** | 43.8 ± 5.5 | 48.2 ± 11.0 | 51.2 ± 11.5 |
| **Average # Substituents** | 1.4 ± 0.5 | 1.8 ± 0.8 | 2.0 ± 0.7 |
| **Ring Atoms per Peripheral Group** | 7.0 ± 5.1 | 9.2 ± 4.6 | 4.3 ± 1.3 |
| **HA Composition of MCs (%Polar [N, O]/% Nonpolar [C, S, Cl, Br])** | | | |
| **Total** | 24/76 ± 3 | 25/75 ± 3 | 23/77 ± 3 |
| **Peripheral** | 83/17 ± 15 | 77/23 ± 22 | 51/49 ± 12 |
| **Substituent** | 28/72 ± 6 | 33/67 ± 7 | 28/72 ± 10 |

**Supplementary Table 2 | Composition of in-silico pML library, synthesized pML library, and NP reference compounds.** HA = Heavy Atom (non-hydrogen atom). * Includes all virtual THF-pMLs containing a side chain (e.g. amide, thiazole, or dihydropyran). Does not include any macrocyclic cores (macrocycles lacking a side chain), or acylated macrocycles. † Includes all synthesized pMLs (macrocycles lacking a side chain, macrocycles with a side chain, and acylated macrocycles). †† The full set of natural products depicted in **Supplementary Fig. 1** without any truncation.

| | % Bioactive Conformer Reproduction | | | | | |
|---|---|---|---|---|---|---|
| | **RMSD (Å)** | **0.5** | **1.0** | **1.5** | **2.0** | **N confs** |
| High Temperature MD[a] | **Average** | 37 | 74 | 90 | 96 | 1733 |
| | **SD** | 8 | 2 | 1 | 1 | 36 |
| MOE LowModeMD[b] | **Average** | 34 | 72 | 84 | 89 | 1675 |
| | **SD** | 2 | 7 | 2 | 2 | 71 |
| MacroModel MT/LMOD[c] | **Average** | 39 | 79 | 96 | 97 | 2415 |
| | **SD** | 2 | 2 | 2 | 0 | 53 |
| MacroModel MT/LLMOD[d] | **Average** | 40 | 77 | 92 | 94 | 2123 |
| | **SD** | 3 | 6 | 2 | 4 | 62 |

**Supplementary Table 3 | % Bioactive conformer reproduction of 30 macrocyclic ligands bound to their biological targets for method used in this work (grey) and 3 representative methods reported in the literature.[1]**

All protocols used three runs of 10,000 total iterations with an energy cutoff of 15 kcal/mol and conformer RMSD duplicate elimination of 0.5 Å. N confs is average number of conformers found by each method within 15 kcal/mol. [a] The method used for conformational sampling as described in the methods of main text (conformational sampling of pMLs and NPs) but with 10,000 MD/minimize iterations. [b] Molecular Operating Environment LowModeMD method with generalized Born-Still implicit solvation and MMFF94x forcefield [c] MacroModel MT/LMOD protocol with generalized Born-Still implicit solvation and OPLS2005 forcefield with 400 rotational steps. [d] MacroModel MT/LLMOD protocol with generalized Born-Still implicit solvation and OPLS2005 forcefield with 400 rotational steps. Data for MOE, MT/LMOD, MT/LLMOD are from the original publication.[1]

| | # MD Iterations[a] | N[b] | Conformation Duplicate Elimination RMSD (Å)[c] | Trial to Trial RMSD Score Difference[d] |
|---|---|---|---|---|
| **Entry 1** | 1500 | 3 | 0.5 | 0.0100 |
| **Entry 2** | 2500 | 3 | 0.5 | 0.0065 |
| **Entry 3** | 3500 | 3 | 0.5 | 0.0075 |

**Supplementary Table 4 | $C_s$ score variation between independent runs of the same number of MD iterations for all pairwise comparisons (306) among the natural products with self-overlaps removed.**

[a] The number of MD iterations for the conformational search (see main text methods for other parameters). [b] Number of independent replicates [c] The RMSD atom position cutoff for duplicate conformations in Ångstroms [d] The root-mean-square-deviation among all matched score differences between replicates (e.g. replicate 1 - replicate 2, replicate 1 - replicate 3, replicate 2 - replicate 3).

| Strain Name | Strain Designation | BSL | Growth Medium | Growth Condition |
|---|---|---|---|---|
| **Gram-Positive** | | | | |
| *Bacillus subtilis* | ATCC 6051 | 1 | NB | 37°C |
| *Enterococcus faecalis* | ATCC 29212 | 2 | BHI | 37°C |
| *Enterococcus faecium* | ATCC 6569 | 2 | BHI | 37°C |
| *Listeria ivanovii* | BAA-139 | 1 | BHI-A; HTM | 37°C; 5% $CO_2$ |
| *Staphylococcus aureus* (Methicillin-Resistant) | BAA-44 | 2 | TSB | 37°C |
| *Staphylococcus aureus* (Methicillin-Sensitive) | ATCC 29213 | 2 | TSB | 37°C |
| *Staphylococcus epidermidis* | ATCC 14990 | 1 | TSB | 37°C |
| *Streptococcus pneumoniae* | ATCC 49619 | 2 | BHI | 37°C; 5% $CO_2$ |
| **Gram-Negative** | | | | |
| *Acinetobacter baumannii* | ATCC 19606 | 2 | TSB | 37°C |
| *Escherichia coli* | K-12 MG1655 | 1 | NB | 37°C |
| *Klebsiella aerogenes* | ATCC 35029 | 1 | NB | 37°C |
| *Klebsiella pneumoniae* | ATCC 700603 | 2 | NB | 37°C |
| *Ochrobactrum anthropi* | ATCC 49687 | 1 | TSB | 37°C |
| *Providencia alcalifaciens* | ATCC 9886 | 1 | TSB | 37°C |
| *Pseudomonas aeruginosa* | ATCC 27853 | 2 | TSB | 37°C |
| *Salmonella enterica* | ATCC 13311 | 2 | NB | 37°C |
| *Shigella sonnei* | ATCC 25931 | 2 | NB | 37°C |
| *Vibrio cholerae* | A1552 El Tor | 2 | TSB | 37°C |
| *Yersinia pseudotuberculosis* | ATCC 6904 | 2 | BHI | 37°C |

**Supplementary Table 5 | Bacterial Target Panel Strains and Culture Conditions**

# Supplementary Methods

# I. Computational Methods and Results

### Similarity Comparisons and Prioritization of pMLs for Synthesis:

### 3D Conformational and Molecular Similarity Scoring:

To evaluate the three-dimensional similarity of two compounds against one another, one must consider a matrix $\boldsymbol{M}$ with $N_1 \, x \, N_2$ 3D similarity scores ($S_{ij}$), where $N_1$ and $N_2$ are the number of conformations of the first and second molecules respectively.

General Matrix $\boldsymbol{M}$ of scores between pairs of conformations of two molecules

$$\begin{bmatrix} S_{11} & \dots & \dots & \dots & S_{1N_2} \\ \dots & \dots & \dots & \dots & \dots \\ \dots & \dots & \dots & \dots & \dots \\ \dots & \dots & \dots & \dots & \dots \\ S_{N_1 1} & \dots & \dots & \dots & S_{N_1 N_2} \end{bmatrix}$$

Each individual score ($S_{ij}$), formally referred to as the TanimotoCombo score, in the matrix was evaluated using the FastROCS[2,3] Toolkit as outlined in the main text. The procedure for calculating the TanimotoCombo similarity score between any two conformations can be summarized as follows:

1) Each molecule is pre-processed, describing the volumes occupied by atoms with soft gaussian functions and adding contracted gaussian functions on key functional groups such as hydrogen bond donors, hydrogen bond acceptors, ring systems, and other meaningful functional groups (e.g. alcohols, carbonyls, 5 & 6 membered rings, etc) as dictated by the Implicit Mills Dean color forcefield.
2) The two conformations are then overlaid using a rotational-translational optimization algorithm until maximal functional group and volume overlap between the two conformations is achieved.
3) The TanimotoCombo (3D functional and shape similarity) score of the two conformations is computed. The functional (color), and shape overlap components are each scored from 0 to 1, and the resultant TanimotoCombo score is the sum of these components, and thus ranges from 0 to 2. However, the values were normalized to between 0 and 1 by dividing each score by 2.

The above process is limited to calculating the TanimotoCombo similarity score between two individual conformations of either 1) two separate molecules, or 2) two conformations of the same molecule. Thus, the goal was to develop an ad hoc solution that would allow us to extend this approach to two multi-conformer molecules and obtain a meaningful measure of 3D similarity between our in-silico pML library compounds and the natural products. After careful consideration the following protocol was adopted:

> All pairwise conformations of a given natural product and a given library pML were scored, and only the highest score between pairs of conformations for each conformation of natural product were output and then averaged (i.e. the maximum score in each row of the matrix $\boldsymbol{M}$ was averaged) to give the conformational TanimotoCombo score ($C_s$) (or conformational score for short).

Critically, this meant that:

1) The score ($C_s$) directionally emphasizes the similarity of a candidate pML against a natural product. This is especially important since we hypothesized that at least some subset of each of the natural products' conformers must be privileged since, in general, the natural products are known to be bioactive and capable of interacting with biological systems.
2) When scored against itself, a given molecule produces a score of approximately 1.0 (within precision of calculation) representing perfect similarity. (Note that this is true for conformer TanimotoCombo scores, if a conformation is scored for similarity against itself it results in a score of 1.0)
3) The score ($C_s$) between two molecules (i.e. a reference natural product and a candidate pML) has the following interpretation: "For every conformation of a given reference natural product within 15 kcal/mol of its respective minimum there is a corresponding conformation in the candidate pML within 15 kcal/mol of its respective minimum that is on average $C_s * 100\%$ similar on the basis of volumetric and functional group overlap."

This approach is summarized in a cartoon depiction in **Supplementary Fig. 7** for a purely hypothetical case.

**Scoring of Library pMLs against THF natural products**

Using the previously generated conformers for the THF natural products and library pMLs coupled with the above $C_s$ scoring approach each of the 3456 library pMLs were scored against each of the 18 THF natural products for a total of 62,208 $C_s$ scores. The $C_s^{max}$ is defined as the largest $C_s$ score of a particular library pML against the set of 18 natural pMLs. After examining the data, the greatest structural predictors of similarity between a library pML and a THF natural product were 1) the structure of the side chains 2) the stereochemistry at the THF and 3) the stereochemistry of the Nozaki-Hiyama-Kishi centre. This is visually apparent by plotting the data in polar coordinates and grouping molecules with shared THF stereochemistry and NHK centre stereochemistry (**Supplementary Fig. 4**). When visualized in such a way, the impact of single stereocentre epimerizations on the $C_s$ can be seen. Moreover, it was clear

that pMLs containing certain stereochemical configurations at the THF and NHK centre showed much greater similarity to the natural products overall. This is a direct result of the stereocentres at the THF dictating the gross conformation of the macrocycle while the stereochemistry at the NHK centre dictates the conformational display of the side chains. Alternatively, the data was sorted by grouping comparisons between library pMLs and single natural products together to emphasize which natural products the library pMLs were most similar to (**Supplementary Fig. 6**). From this plot, it is apparent that the pML library most closely resembles the haterumalides, biselides, and phormidolides.

**Prioritization of compounds for synthesis:**

To aid in the prioritization of a subset of compounds for synthesis the maximum $C_s$ score for each library pML (3456) against the set of natural THF macrolides (18) was plotted in a polar plot **Supplementary Fig. 5**. After careful thought, a prioritization scheme consisting of the following 3 guidelines was chosen:

1) Library pMLs occupying the highest scoring wedges in **Supplementary Fig. 4-5** would be prioritized over all other compounds. Additionally, pMLs occupying some of the more moderate scoring wedges would also be targeted provided their requisite THF building block could be expediently synthesized from the corresponding THF building blocks that resulted in pMLs occupying the highest scoring wedges (i.e. wherever possible THF building blocks were converted to alternative stereochemistry within the THF via a Mitsunobu reaction to allow access to more diverse stereochemistry in the resultant pMLs).
2) Library pMLs with $C_s^{max}$ scores $\geq$ 0.55 against the set of natural products were considered good candidates for synthesis and every effort would be made to ensure at least one analogue in a series of synthesized library pMLs had a $C_s^{max} \geq 0.55$
3) Any molecule that arose from the same synthetic route as a compound prioritized by 2) would also be synthesized even if its $C_s^{max}$ score was < 0.55. These compounds would serve as an additional source of diversity and minimize synthetic effort, while simultaneously enabling prioritization of the macrocycles which contained the features most consistent with the natural products reference set.

In general, there are no exceptions to guidelines 1) or 3). There is 1 exception to guideline 2) which arose from misassigned natural products or natural products with uncertain stereochemistry that were removed early on from the analysis, however in this case there is still at least one pML analogue that had a $C_s^{max}$ close to 0.55 (0.54). After applying the above prioritization scheme a subset of 78 compounds were prioritized and 72 of these compounds (**Supplementary Fig. 5**, highlighted in bold color) were successfully synthesized. The subset of synthesized pMLs represents approximately 2 % of the total in-silico library pMLs. Importantly, the successfully synthesized set of 72 compounds does not include their macrocyclic cores (i.e. macrocycles lacking a side chain), acylated counterparts, or the serendipitous ketone pML.

**Applicability of high temperature MD protocol to conformational sampling of macrocycles in general**

To assess the suitability of our conformational sampling protocol (see conformational sampling methods in main text) for application to sampling conformations of macrocycles, we applied our conformational sampling protocol to a literature data set[1] of 30 protein bound macrocycles and compared the ability of high temperature MD to reproduce the x-ray structure of the protein bound macrocycles. These results are summarized in **Supplementary Table 3** along with a few of the methods outlined in the original publication. Shown in **Supplementary Table 3** is the percentage of bioactive macrocycle conformations that were reproduced to within 0.5, 1.0, 1.5 and 2.0 Å RMSD atom distances from three triplicate runs of 10,000 high temperature MD iterations. Duplicate conformations were removed for each molecule if they were within RMSD atom distances of 0.25 Å. Also shown for comparison is the results of some of the best conformational sampling protocols found in the original study.[1] High temperature MD (see main text methods section on conformational sampling – only change is RMSD elimination of duplicates at 0.25 Å and 10,000 MD iterations instead of 2500) performed reasonably well with respect to the other methods shown. Moreover, this benchmark set contains macrocycles with up to 30 atoms in the macrocycle, for which their bioactive conformers are highly challenging to reproduce. In contrast, the natural THF macrolides and in-silico library pMLs have a maximum of 19 atoms in the macrocycle (of the ones which were simulated), with the majority containing between 13-16 atoms in the macrocycle.

**Consistency of the Conformation Generation and Similarity Scoring Protocols:**

The computational demand associated with simulating the entire in-silico pML library multiple times to optimize the number of MD iterations or assess the variation in individual $C_s$ scores was prohibitive. Thus, we decided to simulate the reference set of 18 natural products in triplicate utilizing either 1500, 2500, or 3500 high temperature MD iterations. We then evaluated the variation of replicate matched $C_s$ scores within these data sets to estimate the optimal number of high temperature MD iterations to produce reliable single point estimates of the $C_s$ scores among similarly complex pMLs. It must be stressed that the natural products and in-silico library pMLs have considerable structural and physicochemical similarities (**Supplementary Tables 1-2**), but in general the natural products are more flexible (and therefore more prone to error). Thus, we felt that it was reasonable to assume that variation in $C_s$ scores from run to run among the natural products would give us a rough approximation of the magnitude of variation we could expect when scoring the synthetic pMLs against the natural product reference set.

Each of the natural product THF macrolides (see **Supplementary Fig. 1**) was subjected to 1500, 2500, or 3500 high temperature MD iterations. Each set of iterations was run in triplicate from independently generated initial geometries, for a total of 9 runs. Subsequently each natural product was scored against every other natural product (e.g. Fijianolide A vs. Biselide A, Fijianolide A vs. Biselide B, etc). In all replicates this amounts to 306 comparisons (18 x 18 – 18) after removal of self-comparisons (e.g. Fijianolide A vs. Fijianolide A). Importantly, the comparison between two compounds is order dependent and the resultant $C_s$ score between two compounds is different (though typically comparable) depending on which compound is acting as the reference. For example, Biselide A (reference) vs. Fijianolide A would have a different $C_s$ score than Fijianolide A (reference) vs Biselide A (see **Supplementary Fig. 7**).

Each of these 306 unique $C_s$ scores have their own average and standard deviation (N=3). To estimate how much the scores varied between replicates we calculated the differences between matched scores from replicate to replicate (e.g. replicate 1 – replicate 2, replicate 1 – replicate 3, replicate 2 – replicate 3). The RMSD of these $C_s$ score differences for identical comparisons between each replicate with the same number of MD iterations was calculated (**Supplementary Table 4**). The $C_s$ scores showed mild to moderate variation between independent MD runs of the same number of iterations. The RMSD of the $C_s$ score differences between replicates was expectedly worse when using 1500 iterations (**Supplementary Table 4,** entry 1), which is likely a consequence of missing important conformations of the individual natural products. In contrast the $C_s$ scores showed less variation when using 2500 or 3500 MD iterations (**Supplementary Table 4**, entries 2-3). The data suggests that we can expect most (~90%, st. dev. $\leq$ 0.0068) single point estimates of $C_s$ scores among the natural products to be within no more than ± 0.017 from of their anticipated mean values (**Supplementary Fig. 8**). Moreover, this may represent an overestimate of the error, due to the low number of replicates (N=3) and the associated critical value for a 95% confidence interval ($t_{\alpha=0.05,df=2}$ = 4.303). Ultimately, this experiment provided evidence that the use of 2500 MD iterations for sampling and scoring the synthetic pMLs against the natural products reference set using 2500 MD iterations was a reasonable choice, and that it is unlikely the scores would change dramatically between independent simulations.

### Calculation of Macrocycle Parameters on library pMLs and NPs

The library pMLs and the reference natural products were subjected to calculation of the macrocycle specific parameters as follows. The macrocycle backbone (and ring size) was defined as the smallest number of atoms that had to be traversed to result in closure of the macrocycle. Groups that were connected to this backbone (but not part of it) were then defined as 1) peripheral groups if they contained only a single heavy atom; 2) small substituents if they contained no more than 5 heavy atoms; and 3) large substituents if they contained more than 5 heavy atoms. The groups were further classified by their proportions of polar and non-polar heavy atoms by defining polar (N, O) and non-polar heavy atoms (C, F, Cl, S, Br, etc). Degrees of unsaturation within the macrocycle were defined as the sum of unique heterocycles and alkenes that were connected to the macrocycle backbone. Notably, this definition of degrees of unsaturation excludes exocyclic alkenes and carbonyls within the macrocycle. This process is summarized in **Supplementary Fig. 9** which showcases the breakdown of these properties for Fijianolide A.

# II. Experimental Methods and Results

### Overview of Stereochemical Assignment for Building Blocks

The stereochemical configuration for each building block was determined using a combination of methods such as: 1) comparison of spectral and/or optical rotation data for known building blocks (or intermediates/derivatives of those compounds) to data previously described in the literature; 2) 2D NOESY data (for relative configuration); 3) use of enantiopure chiral pool starting materials or

auxiliaries; 4) expected outcome of known diastereospecific reactions (e.g. Mitsunobu esterification, $S_N2$); and 5) synthesis of compounds via two independent routes, where one route passes through known intermediates. NOESY data and/or matching spectroscopic data (for known compounds) was considered good evidence for relative configuration. Optical rotation data for known compounds where the sign matched (or was opposite in the case of comparing enantiomers), and magnitude was comparable to data published in the literature was considered good evidence for establishing absolute stereochemical configuration. An executive summary of the basis for assigning stereochemical configurations for all chiral building blocks used in the construction of the pML library is given in **Supplementary Fig. 10**. Additionally, **Supplementary Fig. 10** directs readers to one of six stereochemical synopsis figures (**Supplementary Fig.s 11-16)** which visually show critical data used for the stereochemical assignment for each building block. Additional comparisons for known compounds can be found in the experimental section for the synthesis of individual intermediates.

Building blocks **2b** and **2d** passed through several intermediates where the spectroscopic data was consistent with the literature, but the magnitude of the optical rotation was not in good agreement with literature values.[4] However, data for **ent-2d** was previously reported elsewhere[5] and both the spectroscopic and magnitude of the optical rotation data are in good agreement. Synthesis of **2b** started from an enantiopure chiral pool building block and all data for intermediate **S31** were in agreement with data previously published in the literature.[6]

## Procedures for the Synthesis and Characterization of Tetrahydrofuranol Building Blocks

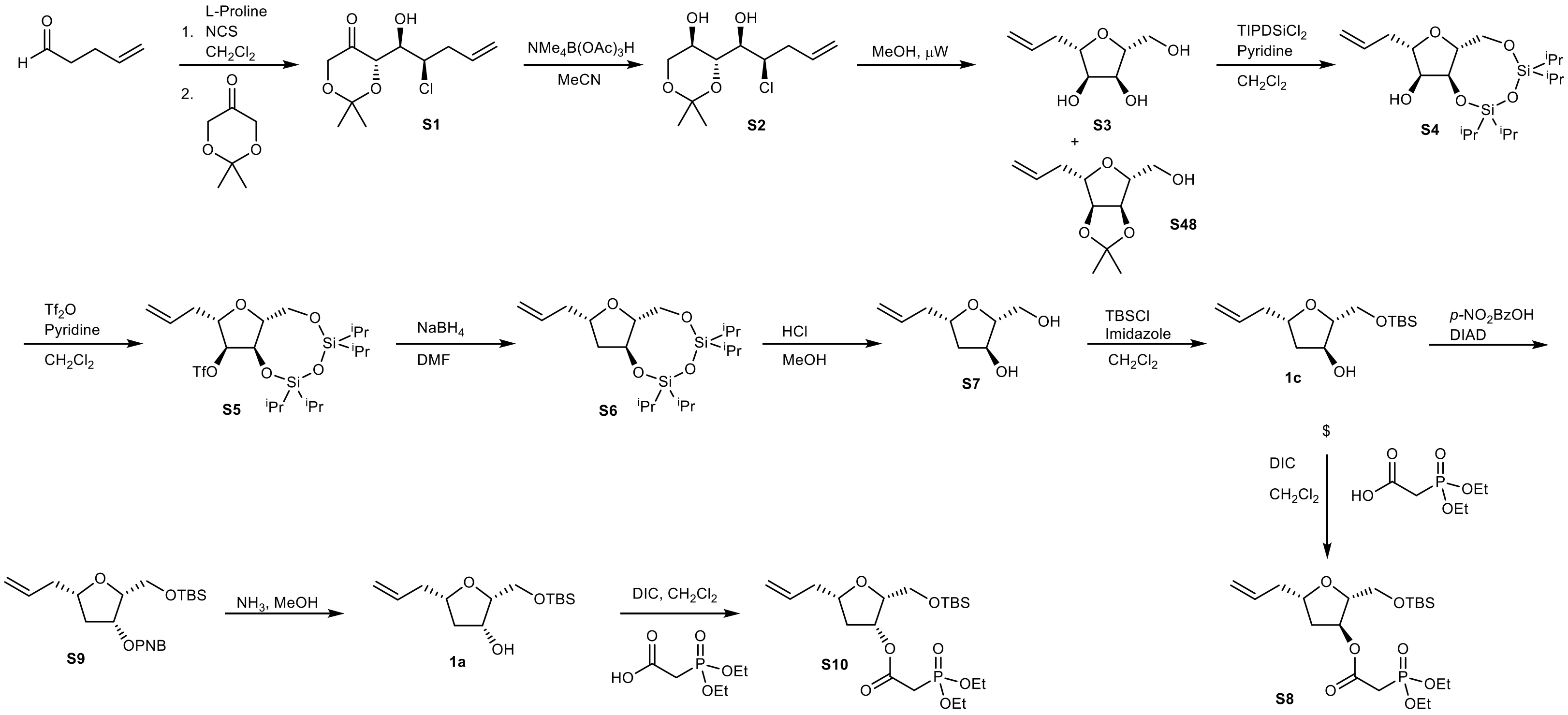


**Supplementary Fig. 27 | Synthesis of Tetrahydrofuranols 1a, 1c and Phosphonates S8, S10.**
Abbreviations: NCS = N-chlorosuccinimide, TIPDSiCl2 = 1,3-dichloro-1,1,3,3-tetraisopropyldisiloxane, Tf2O = trifluoromethanesulfonic anhydride, DMF = dimethylformamide, TBSCl = tert-butyldimethylsilyl chloride, TBS = tert-butyldimethylsilyl, DIAD = diisopropyl azodicarboxylate, DIC = N,N'-diisopropylcarbodiimide.

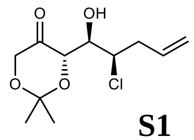

**S1**

Reaction performed open to atmosphere. To a cold (0 °C), stirred solution of pent-4-enal (2.83 g, 33.6 mmol, 1.0 eq) in $CH_2Cl_2$ (300 mL) was added in sequence (*S*)-proline (3.10 g, 26.9 mmol, 0.80 eq.) and N-chlorosuccinimide (4.49 g, 33.6 mmol, 1.0 eq.). The resultant pale-yellow reaction mixture was then allowed to slowly warm to rt over 60 min, after which time 2,2-dimethyl-1,3-dioxan-5-one (3.95 mL, 4.38 g, 33.6 mmol, 1.0 eq.) was added via syringe and the reaction mixture allowed to stir for 44 h. After this time, the reaction mixture was concentrated to *ca.* 150 mL in vacuo, poured into a

separatory funnel, and washed with brine (2x 50 mL). The aqueous layers were back extracted with $CH_2Cl_2$ (2x 20 mL). The combined organic layers were dried ($Na_2SO_4$), filtered, and the solvent removed in vacuo. The crude product was purified via flash column chromatography (9:1 Hexanes/EtOAc). Appropriate fractions were pooled and solvent removed in vacuo to yield **S1** (3.83 g, 15.4 mmol, 46%) as a pale yellow oil. Spectral data agreed with the data previously reported in the literature.[7]

**Analytical Data for S1:**

$R_f$ = 0.27 (8:2 Hexanes/EtOAc)

$[\alpha]_D^{20}$ = -78.0° (c = 1.00, MeOH)

$^1$H NMR (500 MHz, $CDCl_3$) δ 5.84 (ddt, *J* = 17.1, 10.1, 7.0 Hz, 1H), 5.19 (dt, *J* = 17.1, 1.5 Hz, 1H), 5.14 (ddd, *J* = 10.2, 2.0, 1.0 Hz, 1H), 4.40 (dt, *J* = 8.8, 1.2 Hz, 1H), 4.28 (dd, *J* = 17.6, 1.4 Hz, 1H), 4.22 (t, *J* = 7.5 Hz, 1H), 4.07 (d, *J* = 17.6 Hz, 1H), 3.97 (dt, *J* = 9.0, 2.1 Hz, 1H), 3.34 (d, *J* = 1.8 Hz, 1H), 2.76 – 2.59 (m, 2H), 1.51 (s, 3H), 1.42 (s, 3H).

$^{13}$C NMR (126 MHz, $CDCl_3$) δ 212.39, 134.15, 118.55, 101.78, 72.83, 70.93, 66.58, 61.02, 38.90, 23.94, 23.54.

HRMS (ESI): Anal. Calcd. for $C_{11}H_{18}ClO_4^+$ $[M+H]^+$ 249.0888, found 249.0905.

IR (neat): $\nu_{max}$ $(cm^{-1})$ = 3520 (br, OH), 3080 (w, C=*CH*), 2990 (m, CH), 2939 (m, CH), 2894 (m, CH), 1742 (s, C=O), 1642 (m, C=C), 1381 (s), 1256 (s), 1222 (s).

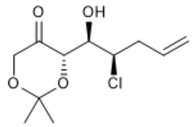

**S1**

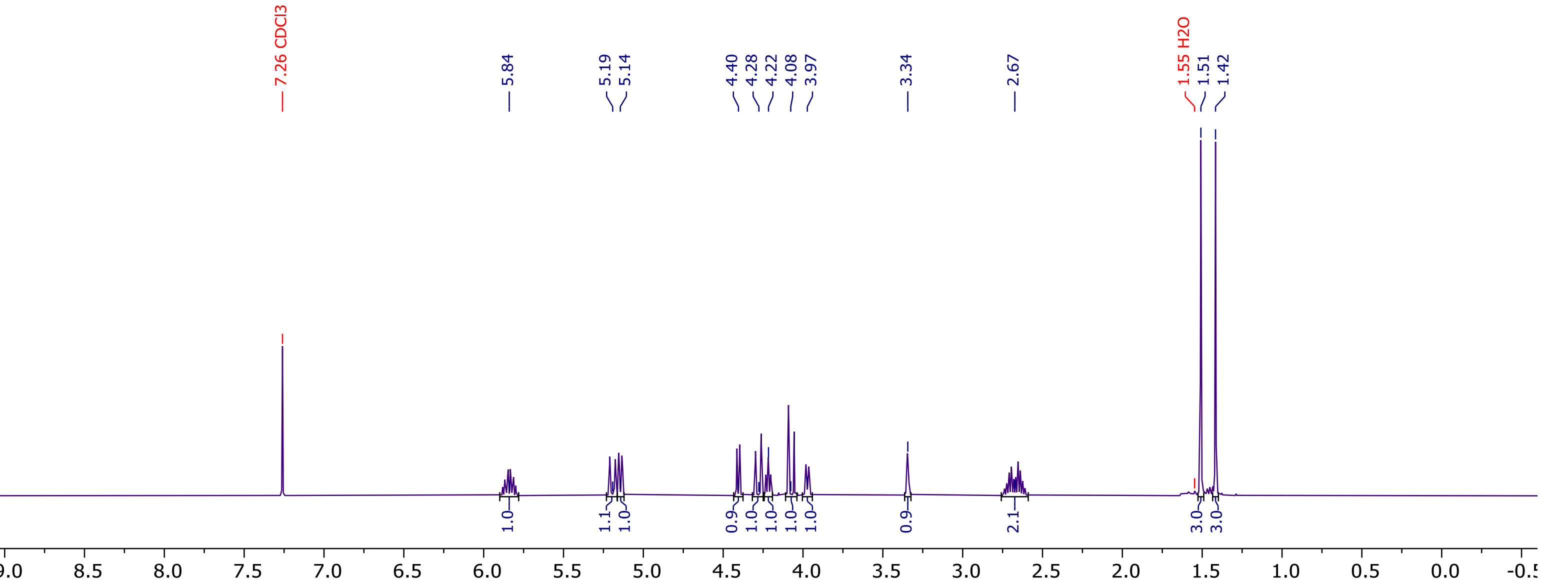
1H
CDCl3
500.13 MHz
7.26 CDCl3
5.84
5.19
5.14
4.40
4.28
4.22
4.08
3.97
3.34
2.67
1.55 H2O
1.51
1.42
1.0
1.1
1.0
0.9
1.0
1.0
1.0
1.0
0.9
2.1
3.0
3.0
10.0 9.5 9.0 8.5 8.0 7.5 7.0 6.5 6.0 5.5 5.0 4.5 4.0 3.5 3.0 2.5 2.0 1.5 1.0 0.5 0.0

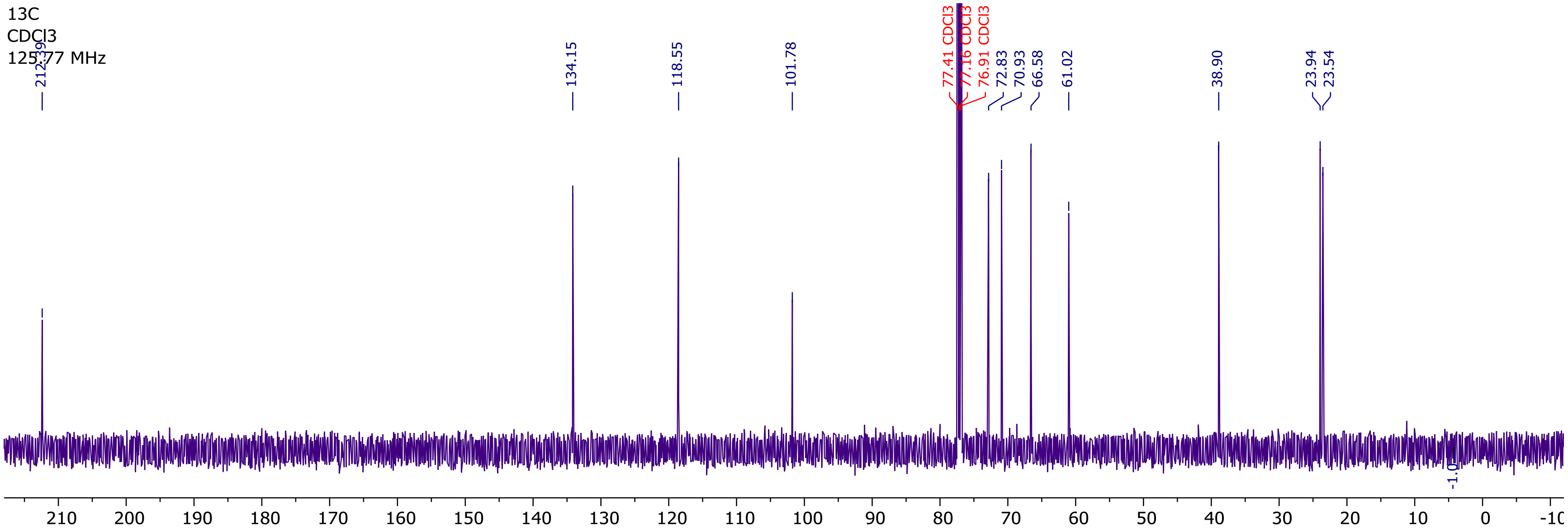
13C
CDCl3
125.77 MHz
212.39
134.15
118.55
101.78
77.41 CDCl3
77.16 CDCl3
76.91 CDCl3
72.83
70.93
66.58
61.02
38.90
23.94
23.54
-1.0
210 200 190 180 170 160 150 140 130 120 110 100 90 80 70 60 50 40 30 20 10 0

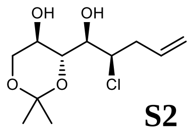


**S2**

To a cold (-20 °C), stirred solution of $Me_4NB(OAc)_3H$ (47.3 g, 180 mmol, 5.0 eq.) in anhydrous MeCN (60 mL) was added glacial acetic acid (20.6 mL, 21.6 g, 360 mmol, 10.0 eq.). A separate solution of **S1 (**8.98 g, 36.1 mmol, 1.0 eq.) in MeCN (25 mL) under $N_2$(g) was added dropwise via syringe to the reaction mixture. After complete addition of **S1**, the reaction mixture was transferred to a -18 °C freezer and maintained at this temperature for 47 hours (no stirring). After this time the reaction mixture was removed from the freezer and added to ice-cold saturated aqueous $NaHCO_3$ (200 mL) and stirred vigorously [**caution $CO_2$(g) evolved!**]. An additional 60 g of $NaHCO_3$ was then added to the mixture portion wise over 5 min. After evolution of $CO_2$(g) ceased, the biphasic mixture was poured into a separatory funnel and extracted with $CH_2Cl_2$ (3 x 200 mL). The combined organic extracts were dried ($Na_2SO_4$), filtered and the solvent removed in vacuo. The crude product was purified via flash column chromatography (2:3 EtOAc-hexanes). Appropriate fractions were pooled, and solvent removed in vacuo to yield **S2** (6.88 g, 27.4 mmol, 76%) as a colorless oil. Spectral data was in agreement with the data previously reported in the literature for **ent-S2**.[8]

**Analytical Data for S2:**

$R_f$ = 0.32 (7:3 Hexanes/EtOAc)

$[\alpha]_D^{20}$ = -14° (c = 0.60, MeOH)

$^1$H NMR (500 MHz, $CDCl_3$) δ 5.85 (ddt, *J* = 17.1, 10.1, 6.9 Hz, 1H), 5.23 – 5.13 (m, 2H), 4.37 (ddd, *J* = 8.1, 6.5, 1.5 Hz, 1H), 3.91 (dd, *J* = 11.4, 5.5 Hz, 1H), 3.84 – 3.75 (m, 2H), 3.69 (t, *J* = 8.7 Hz, 1H), 3.64 (dd, *J* = 11.4, 8.9 Hz, 1H), 3.23 (s, 1H), 2.69 – 2.55 (m, 2H), 2.51 (d, *J* = 8.9 Hz, 1H), 1.47 (s, 3H), 1.36 (s, 3H).

$^{13}$C NMR (126 MHz, $CDCl_3$) δ 133.84, 118.76, 99.18, 72.01, 67.99, 63.99, 62.38, 39.22, 28.46, 19.45.

HRMS (ESI): Anal. Calcd. for $C_{11}H_{20}ClO_4^+$ $[M+H]^+$, 251.1045, found 251.1049.

IR (neat): $\nu_{max}$ ($cm^{-1}$) = 3407 (br, OH), 2994 (m, CH), 1643 (m, C=C), 1202 (s).

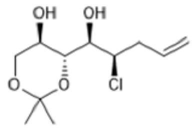
OH
OH
O
O
Cl

**S2**

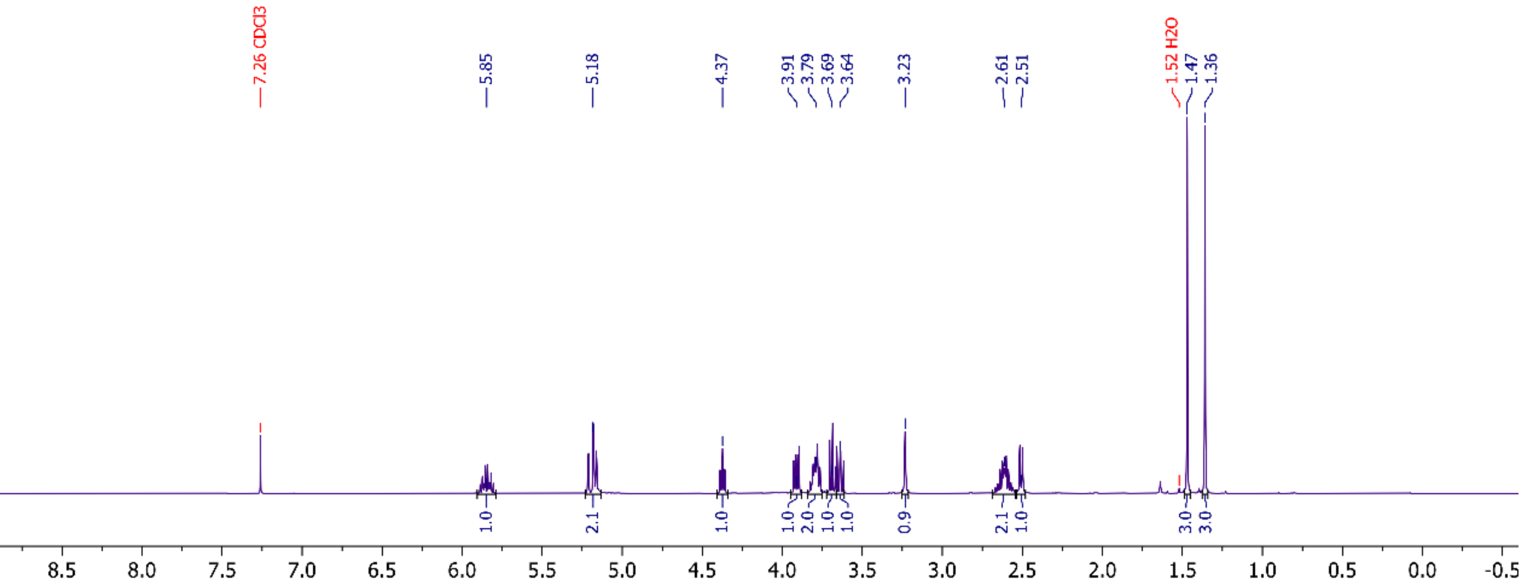
1H
CDCl3
500.13 MHz
7.26 CDCl3
5.85
5.18
4.37
3.91
3.79
3.69
3.64
3.23
2.61
2.51
1.52 H2O
1.47
1.36
1.0
2.1
1.0
1.0
2.0
1.0
1.0
0.9
2.1
1.0
3.0
3.0
10.0 9.5 9.0 8.5 8.0 7.5 7.0 6.5 6.0 5.5 5.0 4.5 4.0 3.5 3.0 2.5 2.0 1.5 1.0 0.5 0.0 -0.5

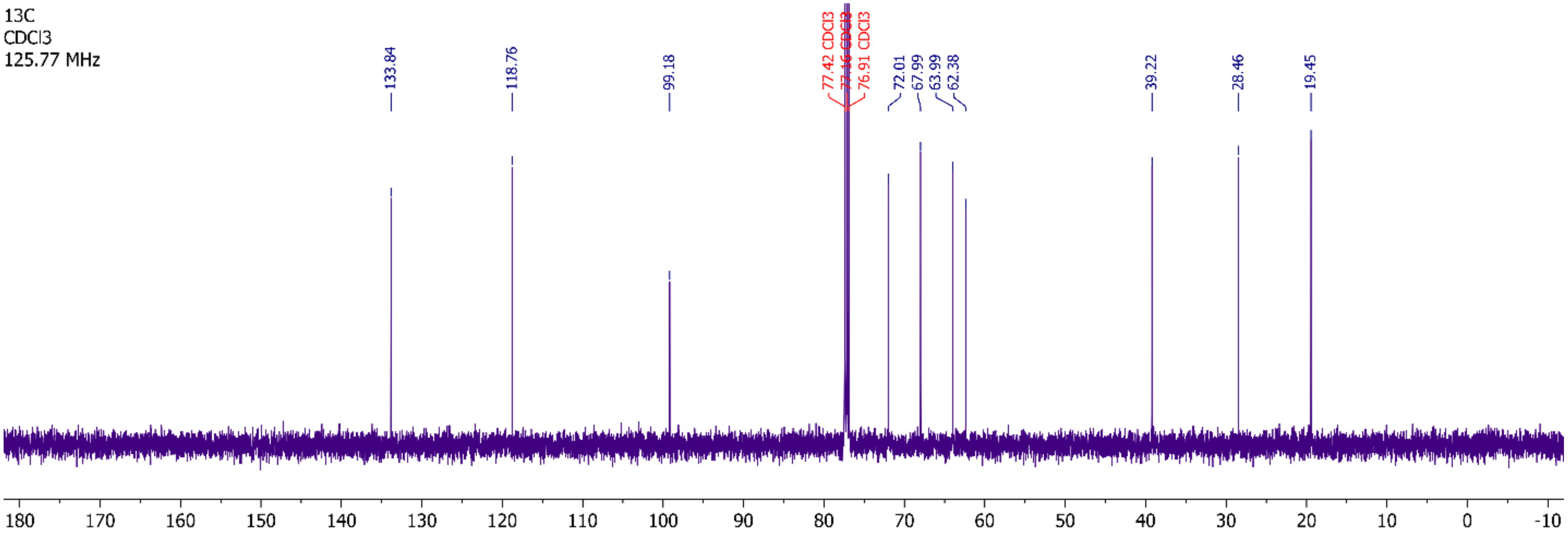
13C
CDCl3
125.77 MHz
133.84
118.76
99.18
77.42 CDCl3
77.16 CDCl3
76.91 CDCl3
72.01
67.99
63.99
62.38
39.22
28.46
19.45
180 170 160 150 140 130 120 110 100 90 80 70 60 50 40 30 20 10 0 -10

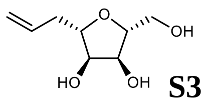
**S3**

Reaction was run in four equally portioned batches. **S2** (1.71 g, 6.82 mmol) was dissolved in 40 mL MeOH and heated to 120 °C in a microwave reactor for 90 min. The resultant dark brown solution was transferred into a 500 mL round bottomed flask. This process was repeated a further 3 times (for a total of 4 batches). The combined reaction mixtures were evaporated in vacuo and the crude product was purified via flash column chromatography (87.5:12.5 $CH_2Cl_2$/MeOH). Appropriate polar fractions were pooled, and solvent removed in vacuo to yield **S3** (3.10 g, 17.8 mmol, 67%) as a colorless oil. The non-polar fractions were re-purified via flash column chromatography (75:25 Hexanes/EtOAc) to yield **S48** (677 mg, 3.16 mmol, 13%) as an orange oil. Spectral and optical rotation data for **S3** agreed with the data previously reported in the literature.[9]

**Analytical Data for S3:**

$R_f$ = 0.45 (85:15 $CH_2Cl_2$/MeOH)

$[\alpha]_D^{20}$ = -5.8° (c = 3.9, MeOH); lit. $[\alpha]_D^{23}$ = -4.6° (c = 1.0, MeOH)[9]

$^1H$ NMR (600 MHz, $CD_3OD$) δ 5.90 (ddt, *J* = 17.2, 10.3, 6.9 Hz, 1H), 5.12 (dq, *J* = 17.2, 1.7 Hz, 1H), 5.05 (ddt, *J* = 10.3, 2.3, 1.2 Hz, 1H), 3.89 (t, *J* = 5.4 Hz, 1H), 3.82 – 3.74 (m, 2H), 3.72 (t, *J* = 5.8 Hz, 1H), 3.67 (dd, *J* = 11.9, 3.6 Hz, 1H), 3.56 (dd, *J* = 11.9, 5.0 Hz, 1H), 2.39 (dddt, *J* = 14.8, 6.7, 5.2, 1.4 Hz, 1H), 2.29 (dtt, *J* = 14.2, 7.0, 1.3 Hz, 1H).

$^{13}C$ NMR (151 MHz, $CD_3OD$) δ 135.90, 117.37, 85.54, 83.70, 75.48, 72.64, 63.52, 38.85.

$^1H$ NMR (400 MHz, DMSO) δ 5.83 (ddt, *J* = 17.1, 10.2, 6.8 Hz, 1H), 5.07 (ddd, *J* = 17.2, 2.6, 1.3 Hz, 1H), 5.01 (ddt, *J* = 10.2, 2.3, 1.2 Hz, 1H), 4.68 (dd, *J* = 5.6, 2.0 Hz, 2H), 4.59 (t, *J* = 5.7 Hz, 1H), 3.72 (td, *J* = 5.5, 4.6 Hz, 1H), 3.64 – 3.57 (m, 2H), 3.53 (q, *J* = 5.9 Hz, 1H), 3.42 (ddd, *J* = 11.6, 5.6, 4.2 Hz, 1H), 3.39 – 3.32 (m, 1H), 2.29 (dddt, *J* = 14.5, 6.5, 5.0, 1.4 Hz, 1H), 2.16 (dtt, *J* = 14.3, 7.0, 1.4 Hz, 1H).

$^{13}C$ NMR (101 MHz, DMSO) δ 135.30, 116.51, 84.33, 81.43, 73.79, 71.04, 62.02, 37.45.

HRMS (ESI): Anal. Calcd. for $C_8H_{15}O_4^+$ $[M+H]^+$ 175.0965, found 175.0964.

IR (neat) $\nu_{max}$ ($cm^{-1}$): 3362 (br, OH), 3079 (w, C=*C-H*), 2926 (m, C-C), 1641 (m, C=C).

1H
MeOD
600.13 MHz

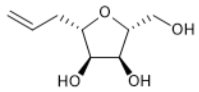

**S3**

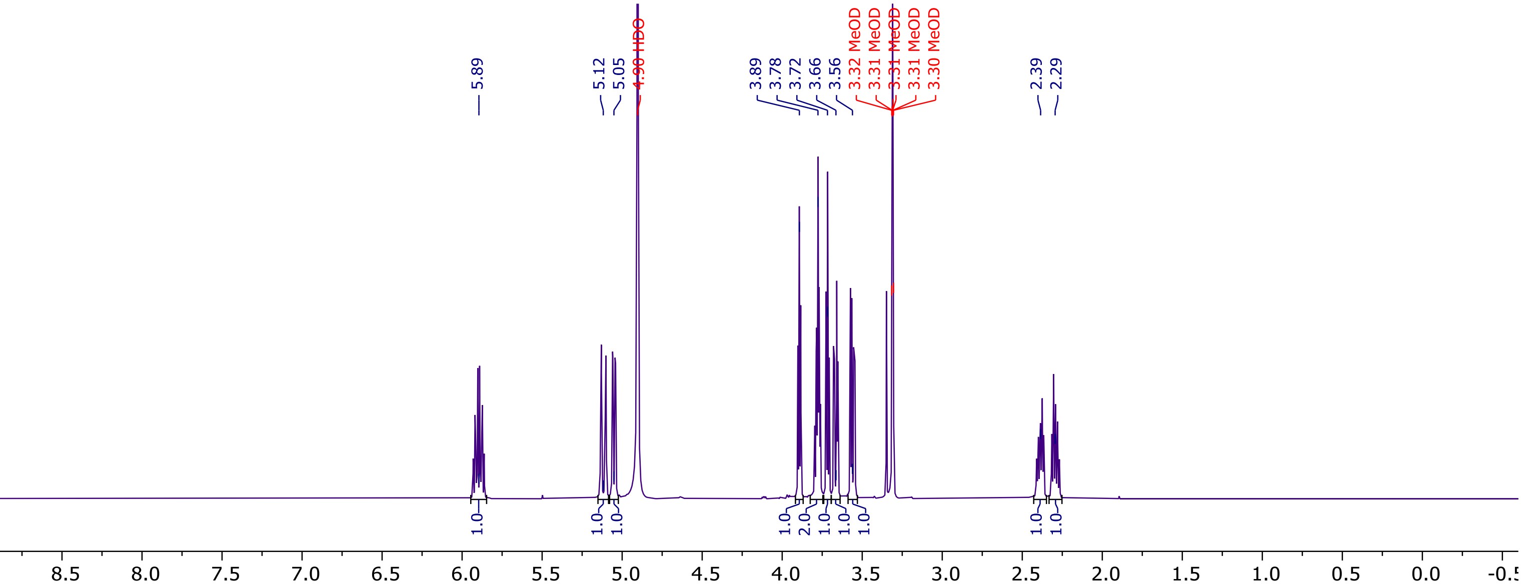


13C
MeOD
150.92 MHz

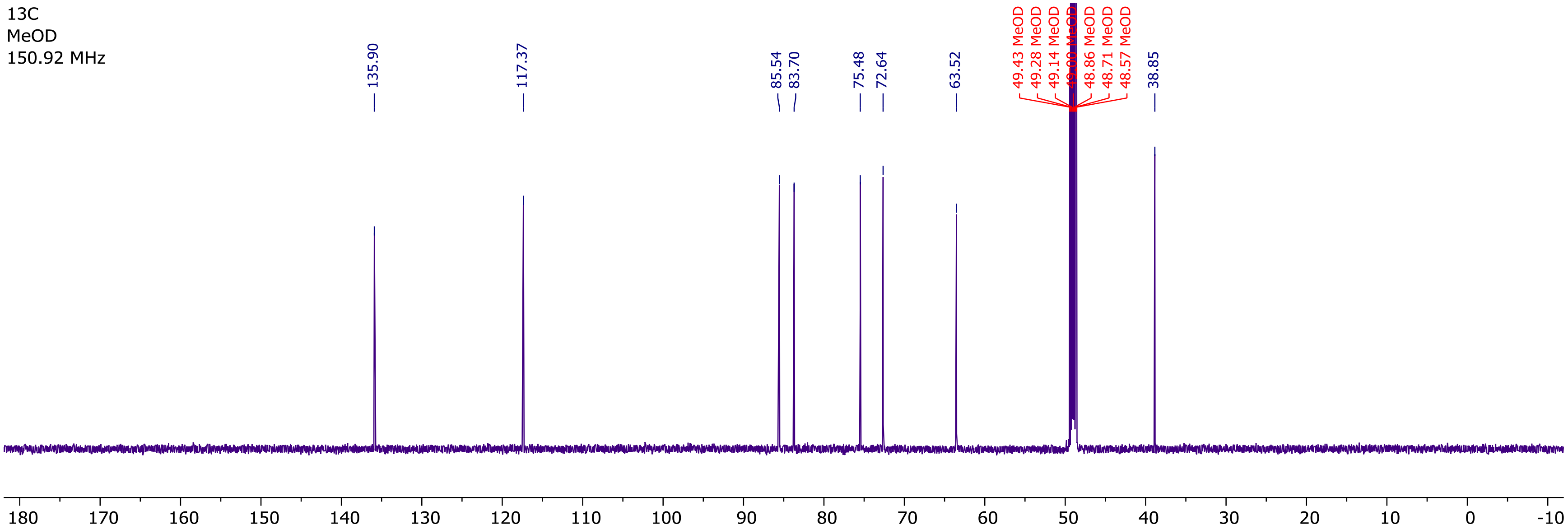

1H
DMSO
400.13 MHz

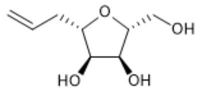


**S3**

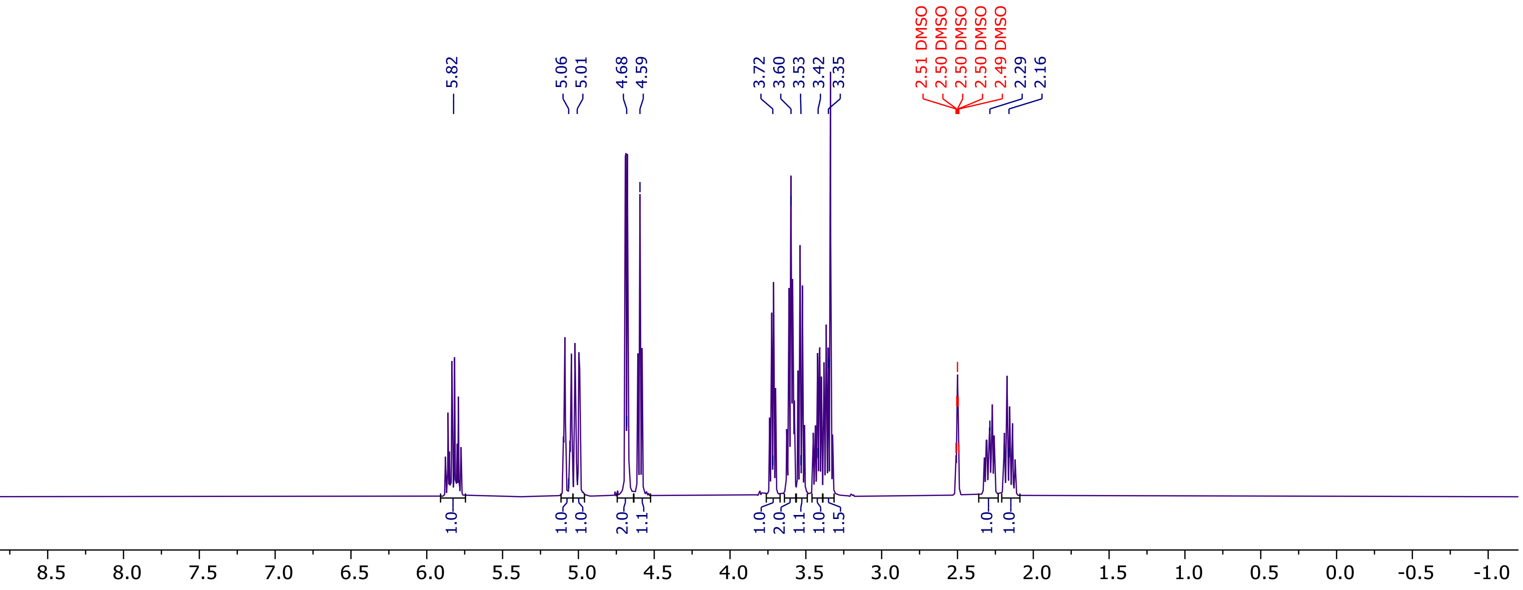


13C
DMSO
100.62 MHz

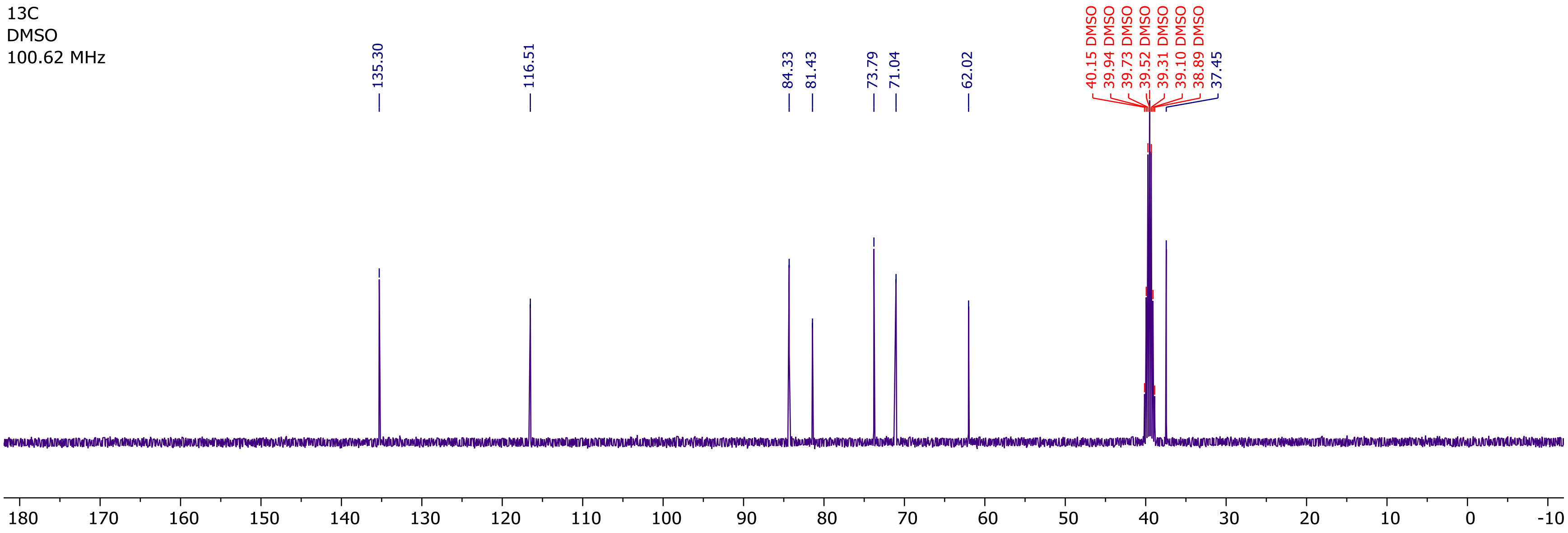

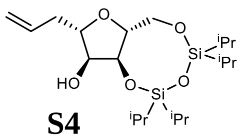

**S4**

To a cold (0 °C), stirred solution of **S3** (3.09 g, 17.7 mmol, 1.0 eq) in anhydrous $CH_2Cl_2$ (68 mL) was added in sequence anhydrous pyridine (16.6 g, 17.0 mL, 210 mmol, 11.9 eq.) and $TIPDSiCl_2$ (5.8 mL, 5.72 g, 18.1 mmol, 1.02 eq.) via syringe. The reaction mixture was then allowed to warm to rt and stirred for a further 18 h, after which time starting material was consumed as monitored by TLC analysis. The reaction mixture was diluted with $CH_2Cl_2$ (20 mL) and washed with $H_2O$ (3x 20 mL). The combined aqueous layers were back extracted with $CH_2Cl_2$ (3x 15 mL). The combined organic layers were washed with brine (50 mL), dried ($Na_2SO_4$), filtered, and the solvent removed in vacuo. The crude product was purified via flash column chromatography (9:1 Hexanes/EtOAc). Appropriate fractions were pooled, and the solvent removed in vacuo to yield **S4** (5.78 g, 13.9 mmol, 78%) as a colorless oil. Spectral and optical rotation data agreed with data previously reported in the literature.[9]

**Analytical Data for S4:**

$R_f$ = 0.33 (Hexanes/EtOAc)

$[\alpha]_D^{20}$ = -14.7° (c =5.91, $CH_2Cl_2$); lit. $[\alpha]_D^{24}$ -20.7° (c =1.00, $CH_2Cl_2$)[9]

$^1$H NMR (600 MHz, $CDCl_3$) δ 5.86 (ddt, *J* = 17.2, 10.3, 7.0 Hz, 1H), 5.14 (dd, *J* = 17.2, 1.8 Hz, 1H), 5.09 (dd, *J* = 10.3, 2.0 Hz, 1H), 4.19 (t, *J* = 6.6 Hz, 1H), 4.01 (dd, *J* = 12.2, 3.3 Hz, 1H), 3.91 – 3.84 (m, 2H), 3.83 – 3.76 (m, 2H), 2.83 (d, *J* = 3.6 Hz, 1H), 2.40 (dt, *J* = 14.2, 6.2 Hz, 1H), 2.32 (dt, *J* = 14.1, 6.7 Hz, 1H), 1.21 – 0.91 (m, 28H).

$^{13}$C NMR (151 MHz, $CDCl_3$) δ 134.02, 117.69, 83.51, 82.26, 73.92, 72.23, 62.73, 37.91, 17.59, 17.48, 17.45, 17.31, 17.19, 17.16, 17.09, 13.50, 13.33, 12.90, 12.75.

HRMS (ESI): Anal. Calcd. for $C_{20}H_{41}O_5Si_2^+$ $[M+H]^+$ 417.2487, found 417.2504.

IR (neat): $\nu_{max}$ $(cm^{-1})$ = 2946 (m, CH), 2869 (m, CH), 1643 (w, C=C).

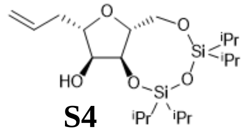

**S4**

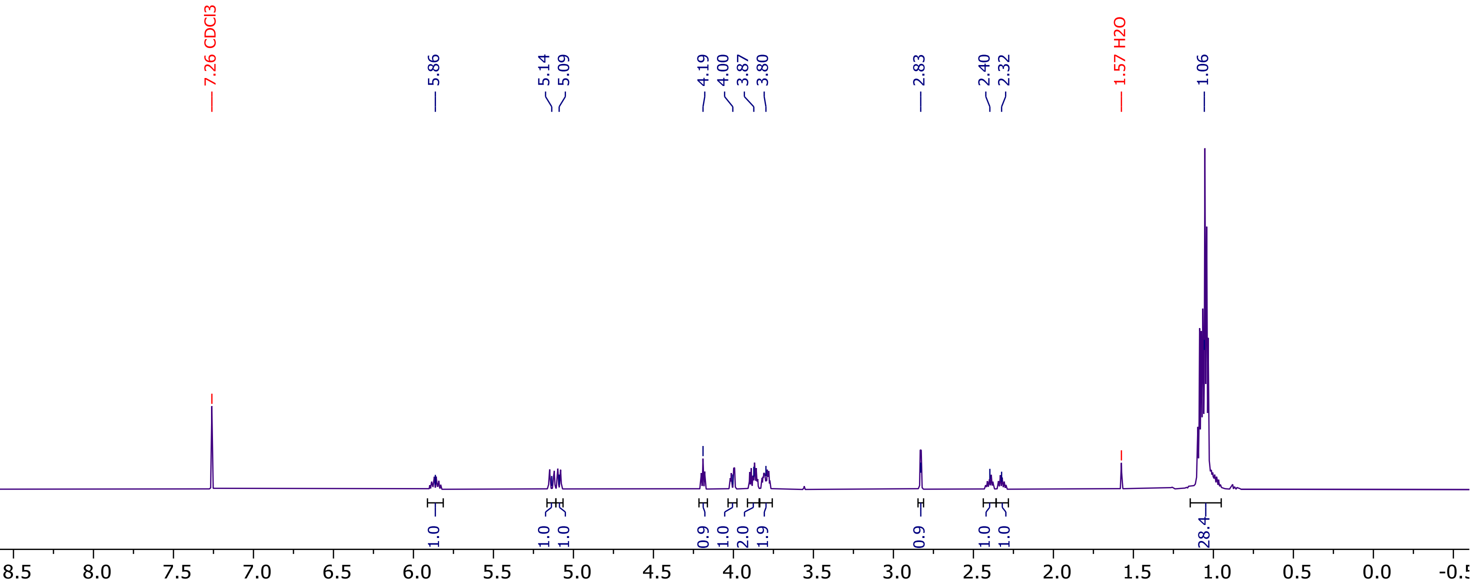
1H
CDCl3
600.13 MHz
7.26 CDCl3
5.86
5.14
5.09
4.19
4.00
3.87
3.80
2.83
2.40
2.32
1.57 H2O
1.06
1.0
1.0
1.0
0.9
1.0
2.0
1.9
0.9
1.0
1.0
28.4
10.0 9.5 9.0 8.5 8.0 7.5 7.0 6.5 6.0 5.5 5.0 4.5 4.0 3.5 3.0 2.5 2.0 1.5 1.0 0.5 0.0 -0.5

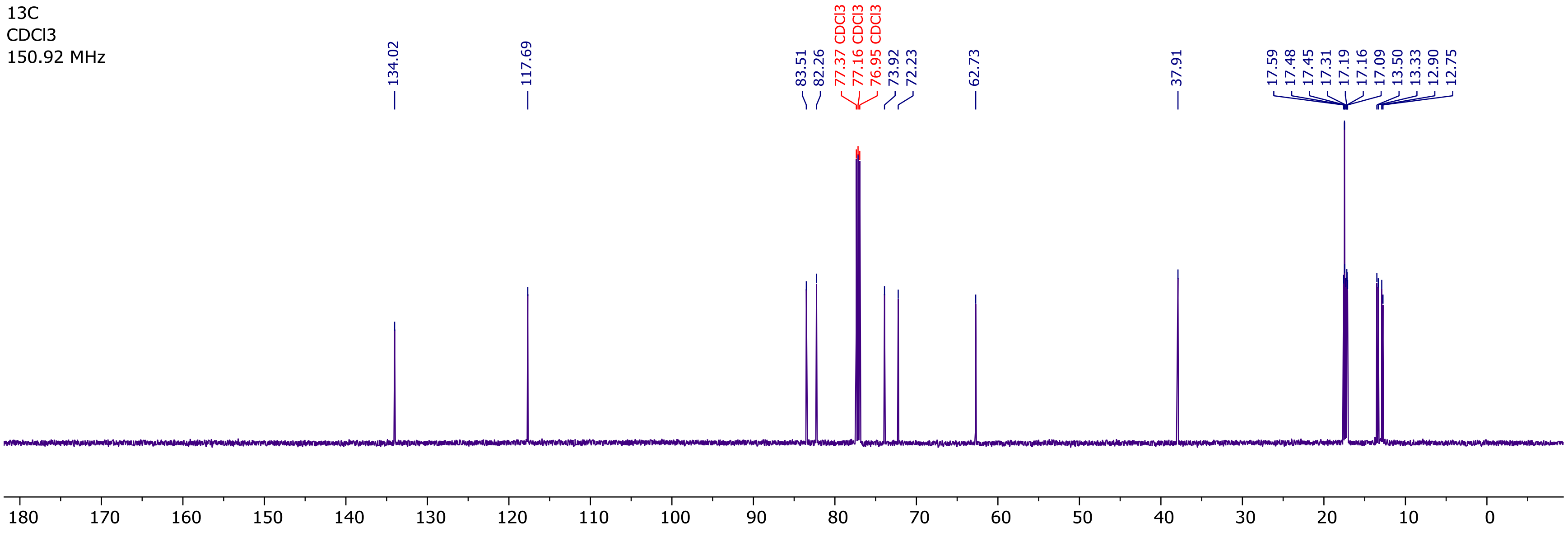
13C
CDCl3
150.92 MHz
134.02
117.69
83.51
82.26
77.37 CDCl3
77.16 CDCl3
76.95 CDCl3
73.92
72.23
62.73
37.91
17.59
17.48
17.45
17.31
17.19
17.16
17.09
13.50
13.33
12.90
12.75
180 170 160 150 140 130 120 110 100 90 80 70 60 50 40 30 20 10 0

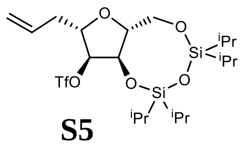

**S5**

To a cold (0°C), stirred solution of **S4** (5.78 g, 13.9 mmol, 1.0 eq.) in $CH_2Cl_2$ (45 mL) under $N_2$(g) was added anhydrous pyridine (5.6 mL) followed by $Tf_2O$ (5.8 mL, 9.8 g, 35 mmol, 2.5 eq.) dropwise via syringe. The reaction mixture was then allowed to warm to rt and stirred for 45 min, after which time starting material was consumed as monitored by TLC analysis. The reaction mixture was then cooled (0 °C) and washed with ice-cold saturated $NaHCO_3$ (2x 60 mL). The aqueous layers were extracted with $CH_2Cl_2$ (2 x 35 mL) and the combined organic extracts were dried ($Na_2SO_4$), filtered and solvent was removed in vacuo. The crude product was purified by flash column chromatography (19:1 Hexanes/EtOAc). Appropriate fractions were pooled, and solvent was removed in vacuo to yield **S5** (7.61 g, 13.9 mmol, 97%) as a colorless oil.

**Analytical Data for S5:**

$R_f$ = 0.52 (9:1 Hexanes/EtOAc)

$[\alpha]_D^{20}$ = -3.0° (c = 2.97, $CH_2Cl_2$)

$^1$H NMR (600 MHz, $CDCl_3$) δ 5.82 (ddt, *J* = 17.2, 10.3, 7.1 Hz, 1H), 5.20 – 5.12 (m, 2H), 4.97 (d, *J* = 4.8 Hz, 1H), 4.28 (dd, *J* = 9.4, 4.8 Hz, 1H), 4.26 (t, *J* = 5.8 Hz, 1H), 4.09 (d, *J* = 13.3 Hz, 1H), 3.94 (dd, *J* = 13.3, 2.6 Hz, 1H), 3.82 (d, *J* = 9.4 Hz, 1H), 2.44 – 2.33 (m, 2H), 1.23 – 0.90 (m, 28H).

$^{13}$C NMR (151 MHz, $CDCl_3$) δ 132.12, 121.85, 119.74, 119.41, 117.62, 115.51, 90.42, 81.18, 80.53, 69.18, 59.72, 37.91, 17.49, 17.41, 17.39, 17.36, 16.94, 16.91, 16.85, 13.39, 13.13, 13.00, 12.73.

HRMS (ESI): Anal. Calcd. for $C_{21}H_{43}F_3NO_7SSi_2^+$ 566.2245, found 566.2287.

IR (neat): $\nu_{max}$ $(cm^{-1})$ = 2948 (m, CH), 2871 (m, CH), 1643 (w, C=C), 1421 (s, S=O), 1210 (s).

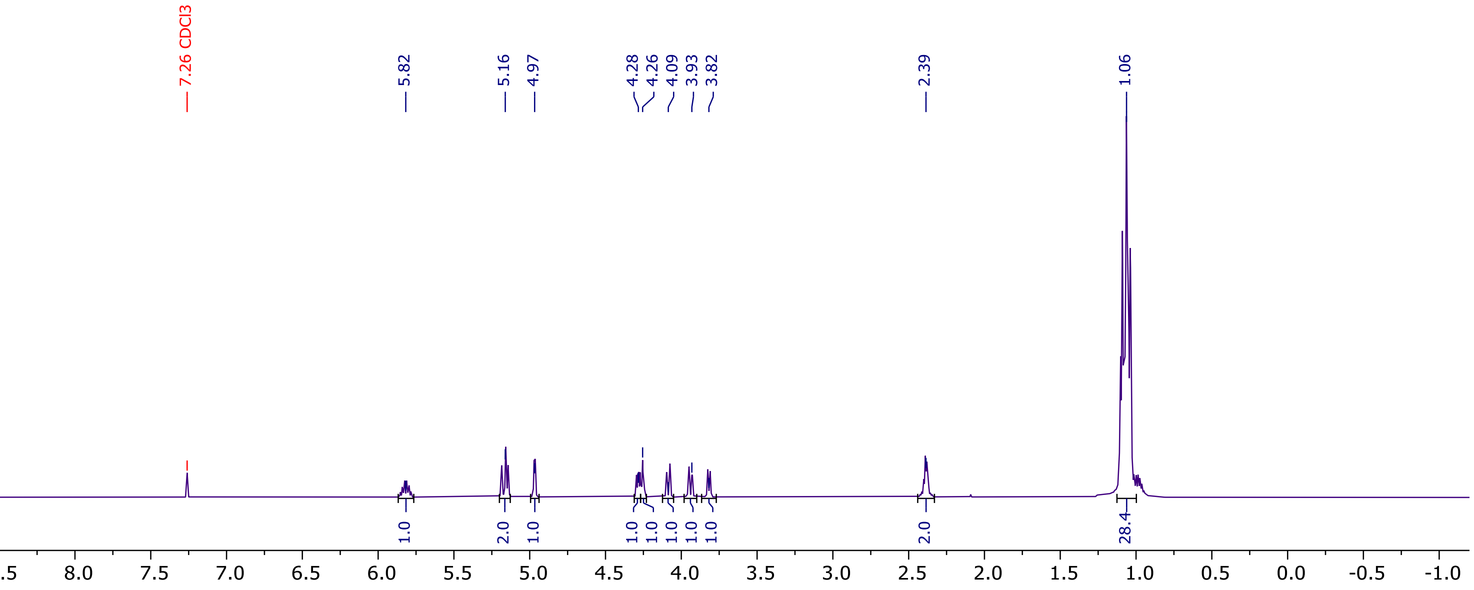

1H
CDCl3
600.13 MHz
7.26 CDCl3
5.82
5.16
4.97
4.28
4.26
4.09
3.93
3.82
2.39
1.06
1.0
2.0
1.0
1.0
1.0
1.0
1.0
1.0
2.0
28.4
10.0 9.5 9.0 8.5 8.0 7.5 7.0 6.5 6.0 5.5 5.0 4.5 4.0 3.5 3.0 2.5 2.0 1.5 1.0 0.5 0.0 -0.5 -1.0


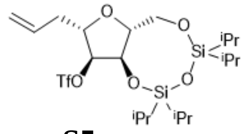

TfO
O
O
Si
iPr
iPr
O
O
Si
iPr
iPr
O


**S5**

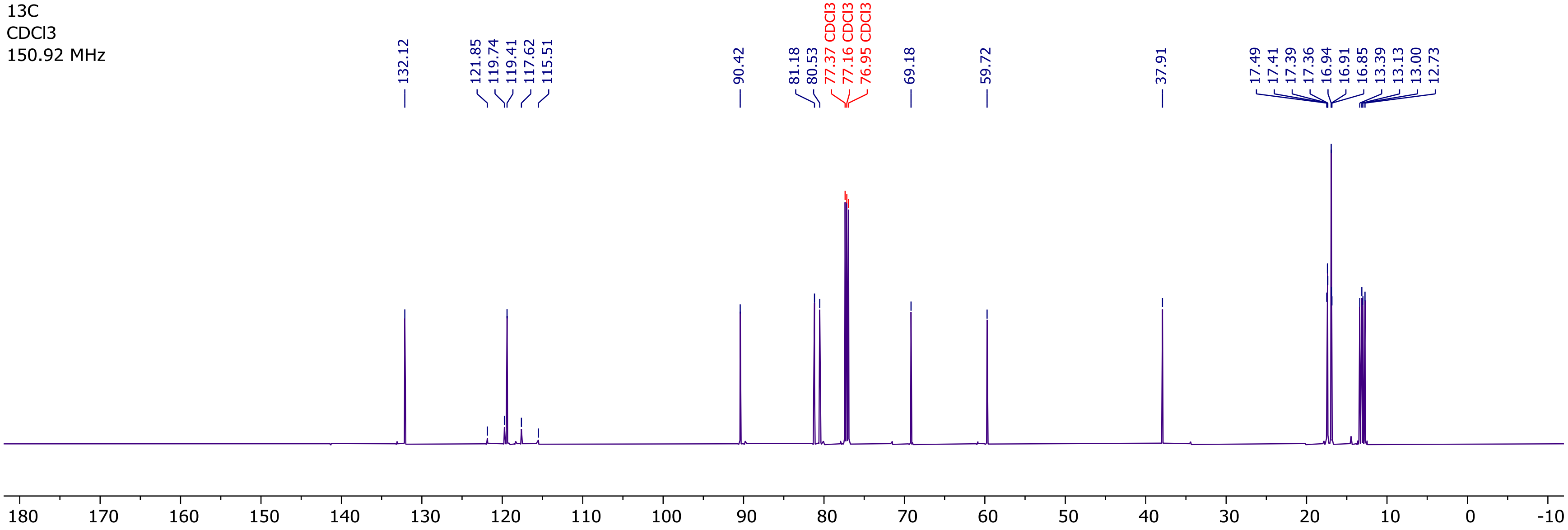

13C
CDCl3
150.92 MHz
132.12
121.85
119.74
119.41
117.62
115.51
90.42
81.18
80.53
77.37 CDCl3
77.16 CDCl3
76.95 CDCl3
69.18
59.72
37.91
17.49
17.41
17.39
17.36
16.94
16.91
16.85
13.39
13.13
13.00
12.73
180 170 160 150 140 130 120 110 100 90 80 70 60 50 40 30 20 10 0 -10

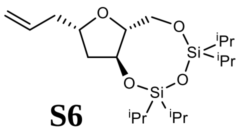

**S6**

To a stirred solution of **S5** (7.19 g, 13.1 mmol, 1.0 eq.) at rt in DMF (130 mL) was added $NaBH_4$ (991 mg, 26.2 mmol, 2.0 eq.). The reaction mixture was then submerged in a pre-heated oil bath (50 °C) and maintained at this temperature for 40 min, after which time starting material was consumed as monitored by TLC analysis. The oil bath was replaced with an ice bath (0 °C), and the reaction mixture was quenched by slow addition of saturated $NH_4Cl$ (150 mL) with vigorous stirring **[caution $H_2$ (g) evolved]**. After effervescence had subsided (*ca.* 20 min.), the biphasic reaction mixture was poured into a separatory funnel and extracted with $Et_2O$ (3x 170 mL). The combined organic extracts were washed with brine (150 mL), dried ($MgSO_4$), filtered, and solvent was removed in vacuo. The crude product was purified via flash column chromatography (24:1 Hexanes/$Et_2O$). Appropriate fractions were pooled, and solvent was removed in vacuo to yield **S6** (4.23 g, 10.6 mmol, 81%) as a colorless oil. Spectral and optical rotation data agreed with the data previously reported in the literature.[9]

**Analytical Data for S6:**

$R_f$ = 0.19 (97:3 Hexanes/$Et_2O$)

$[\alpha]_D^{20}$ = -12.0 ° (c = 1.13, $CH_2Cl_2$); lit. $[\alpha]_D^{23}$ = -13.2 ° (c = 1.0, $CH_2Cl_2$)[9]

$^1$H NMR (600 MHz, $CDCl_3$) δ 5.81 (ddt, $J$ = 17.2, 10.2, 6.9 Hz, 1H), 5.13 – 5.04 (m, 2H), 4.40 – 4.33 (m, 1H), 4.14 (p, $J$ = 6.6 Hz, 1H), 4.06 – 3.99 (m, 1H), 3.77 – 3.69 (m, 2H), 2.33 (dt, $J$ = 13.5, 6.5 Hz, 1H), 2.25 (dt, $J$ = 13.8, 6.6 Hz, 1H), 2.01 (ddd, $J$ = 11.7, 6.7, 4.5 Hz, 1H), 1.83 (dt, $J$ = 12.8, 7.8 Hz, 1H), 1.11 – 0.96 (m, 27H), 0.91 (p, $J$ = 7.4 Hz, 1H).

$^{13}$C NMR (151 MHz, $CDCl_3$) δ 134.47, 117.36, 86.03, 77.09, 73.52, 63.86, 39.99, 39.82, 17.72, 17.59, 17.56, 17.44, 17.30, 17.20, 17.12, 13.64, 13.54, 13.08, 12.67.

HRMS (ESI): Anal. Calcd. for $C_{20}H_{44}NO_4Si_2^+$ $[M+NH_4]^+$ 418.2803, found 418.2812

IR (neat): $\nu_{max}$ $(cm^{-1})$ = 3078 (w, C=*CH*), 2945 (m, CH), 2868 (m, CH), 1642 (w, C=C).

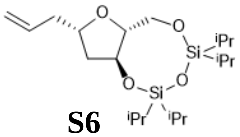
O
Si
O
O
Si
O
ⁱPr
ⁱPr
ⁱPr
ⁱPr

**S6**

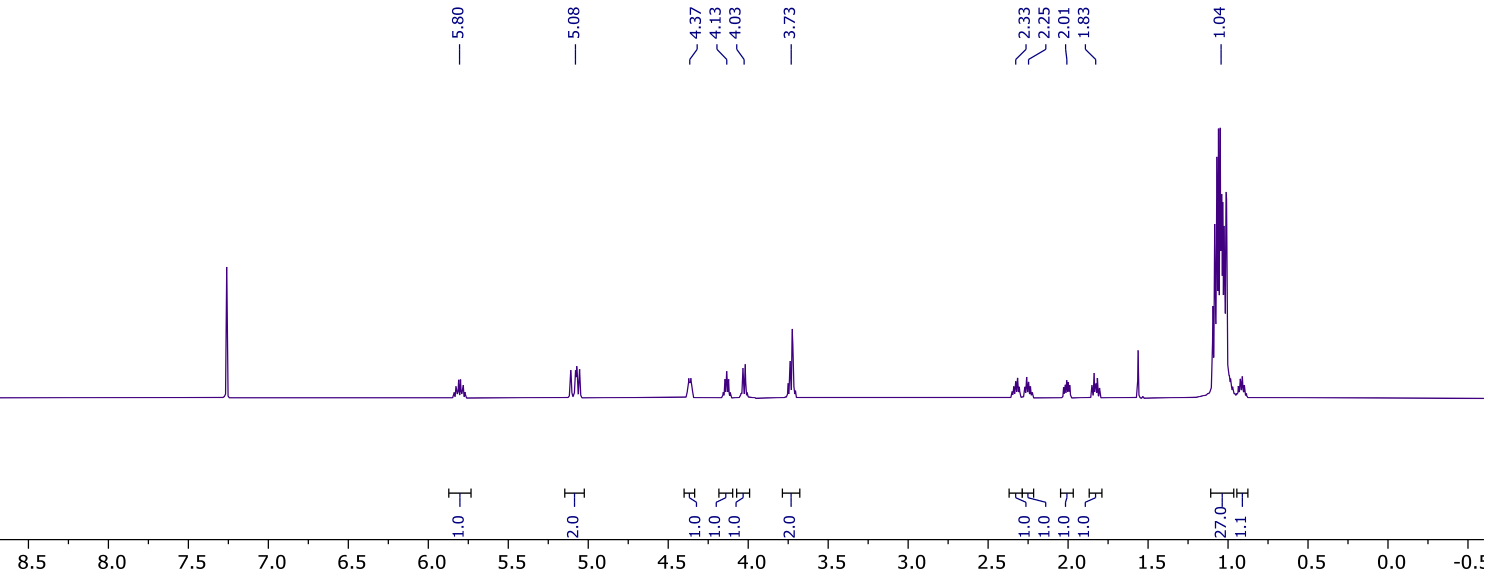
1H
CDCl3
600.13 MHz
5.80
5.08
4.37
4.13
4.03
3.73
2.33
2.25
2.01
1.83
1.04
1.0
2.0
1.0
1.0
1.0
2.0
1.0
1.0
1.0
1.0
27.0
1.1
10.0 9.5 9.0 8.5 8.0 7.5 7.0 6.5 6.0 5.5 5.0 4.5 4.0 3.5 3.0 2.5 2.0 1.5 1.0 0.5 0.0

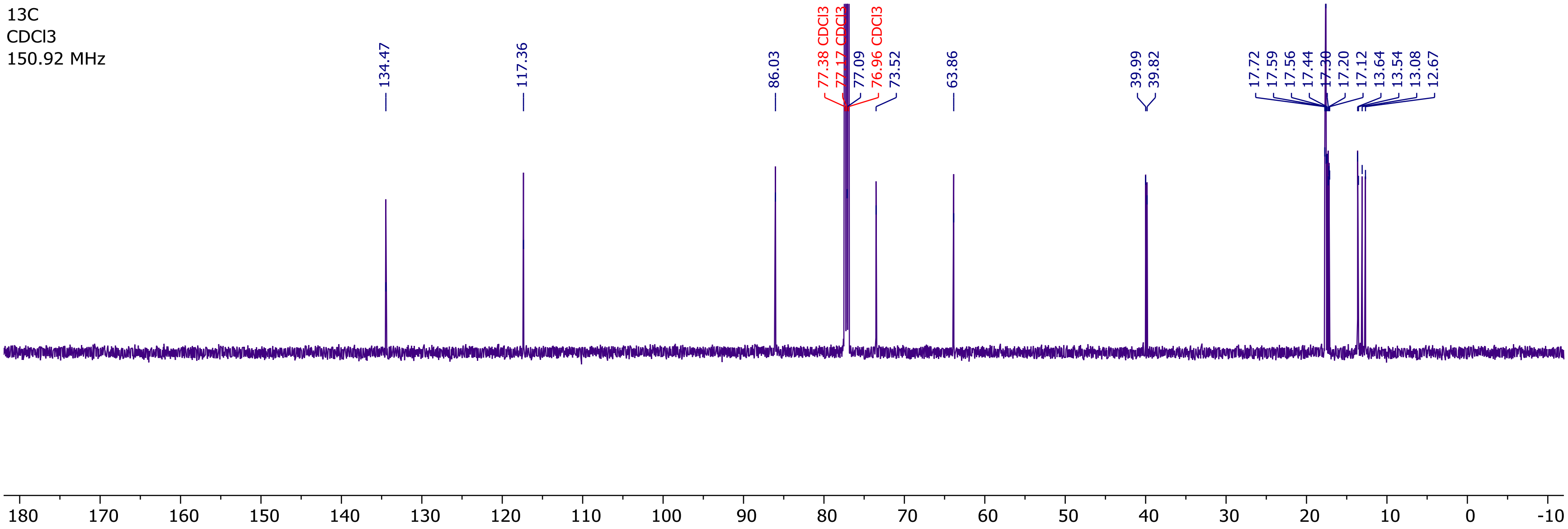
13C
CDCl3
150.92 MHz
134.47
117.36
86.03
77.38 CDCl3
77.17 CDCl3
77.09
76.96 CDCl3
73.52
63.86
39.99
39.82
17.72
17.59
17.56
17.44
17.30
17.20
17.12
13.64
13.54
13.08
12.67
180 170 160 150 140 130 120 110 100 90 80 70 60 50 40 30 20 10 0 -10

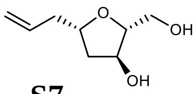

**S7**

A solution of *ca.* 1M aq. HCl in MeOH was prepared by diluting 8.2 mL of 37% (w/w) aq. HCl in reagent grade MeOH (91.8 mL). To a stirred solution of **S6** (4.23 g, 10.6 mmol, 1.0 eq.) in 1M aq. HCl in MeOH (42.4 mL, *ca.* 42.4 mmol, 4 eq.). The reaction mixture was submerged in a pre-heated oil bath (50 °C) and heating was maintained at this temperature for 1 h 50 min, after which time starting material was consumed as monitored by TLC analysis. The oil bath was replaced with an ice bath (0 °C) and $NaHCO_3$ (5 g, 60 mmol, 5.7 eq.) was added portion wise with vigorous stirring such that gentle effervescence was maintained **[caution $H_2$ (g) evolved]**. After effervescence had subsided, the reaction mixture was filtered through a cotton plug and solvent was removed in vacuo. The crude product was purified via flash column chromatography (19:1 to 23:2 $CH_2Cl_2$/MeOH). Appropriate fractions were pooled, and solvent was removed in vacuo to yield **S7** (1.54 g, 9.74 mmol, 92%) as a colorless oil.

**Analytical Data for S7:**

$R_f$ = 0.14 (19:1 $CH_2Cl_2$/MeOH)

$[\alpha]_D^{20}$ = +24° (c = 0.50, MeOH)

$^1$H NMR (500 MHz, $CD_3CN$) δ 5.86 (ddt, *J* = 17.2, 10.2, 6.9 Hz, 1H), 5.10 (ddd, *J* = 17.2, 2.2, 1.4 Hz, 1H), 5.05 (ddd, *J* = 10.3, 2.3, 1.1 Hz, 1H), 4.15 – 4.07 (m, 2H), 3.66 (td, *J* = 5.1, 2.9 Hz, 1H), 3.52 – 3.41 (m, 2H), 3.06 (d, *J* = 4.2 Hz, 1H), 2.71 (t, *J* = 6.0 Hz, 1H), 2.38 – 2.29 (m, 1H), 2.25 (dddd, *J* = 14.1, 7.3, 6.0, 1.3 Hz, 1H), 1.96 (p, *J* = 2.5 Hz, 1H), 1.83 (ddd, *J* = 13.0, 5.5, 2.2 Hz, 1H), 1.66 (ddd, *J* = 13.0, 9.8, 6.3 Hz, 1H).

$^{13}$C NMR (126 MHz, $CD_3CN$) δ 136.24, 117.06, 88.18, 78.46, 73.73, 63.85, 41.20, 40.51.

HRMS (ESI): Anal. Calcd. for $C_8H_{18}NO_3^+$ $[M+H]^+$ 176.1281, found 176.1286

IR (neat): $\nu_{max}$ $(cm^{-1})$ = 3347 (br, OH), 3078 (w, C=*CH*), 2928 (m, CH), 2873 (m, CH), 1642 (w, C=C).

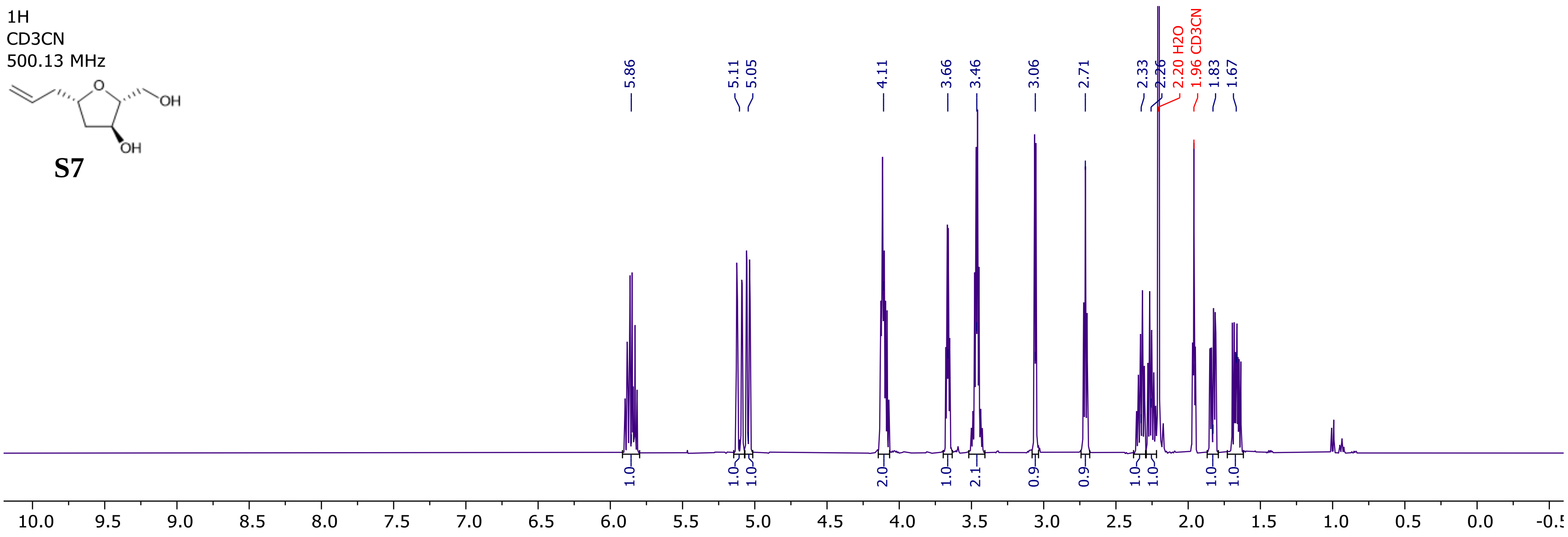
1H
CD3CN
500.13 MHz
O
OH
OH
S7
5.86
5.11
5.05
4.11
3.66
3.46
3.06
2.71
2.33
2.26
2.20 H2O
1.96 CD3CN
1.83
1.67
1.0
1.0
1.0
2.0
1.0
2.1
0.9
0.9
1.0
1.0
1.0
1.0
10.0 9.5 9.0 8.5 8.0 7.5 7.0 6.5 6.0 5.5 5.0 4.5 4.0 3.5 3.0 2.5 2.0 1.5 1.0 0.5 0.0

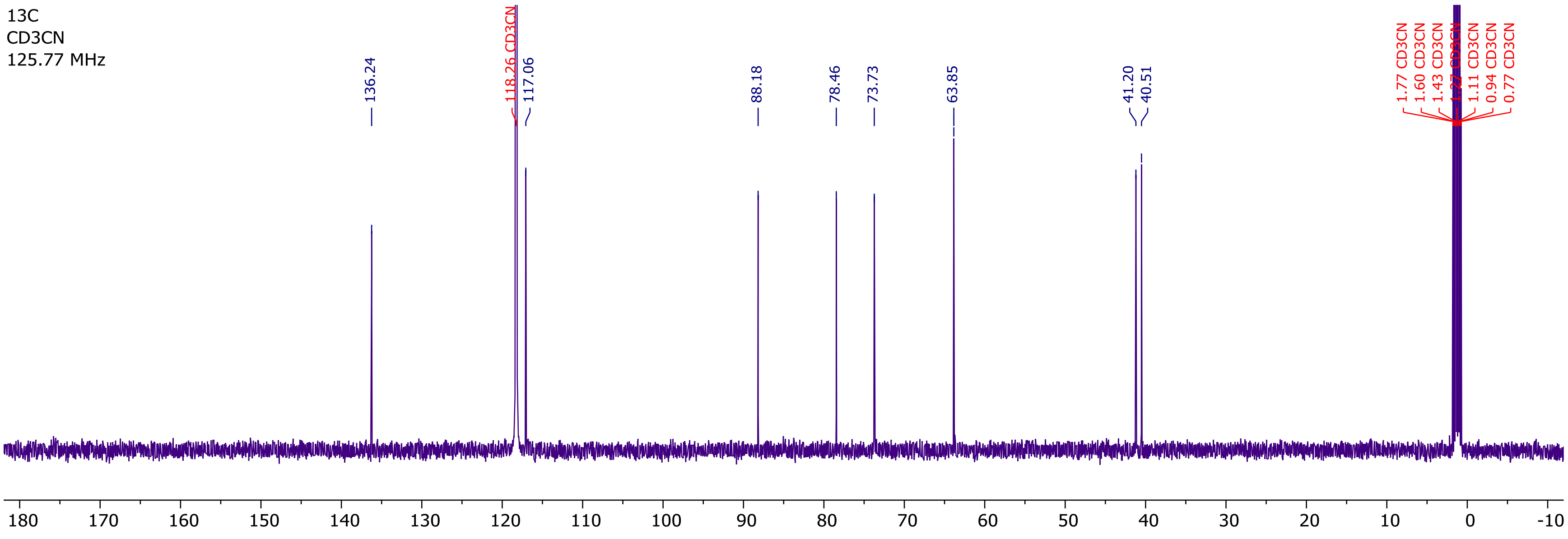
13C
CD3CN
125.77 MHz
136.24
118.26 CD3CN
117.06
88.18
78.46
73.73
63.85
41.20
40.51
1.77 CD3CN
1.60 CD3CN
1.43 CD3CN
1.11 CD3CN
0.94 CD3CN
0.77 CD3CN
180 170 160 150 140 130 120 110 100 90 80 70 60 50 40 30 20 10 0 -10

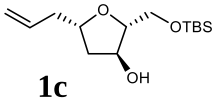

**1c**

To a cold (0 °C), stirred solution of **S7** (1.54 g, 9.74 mmol, 1.0 eq.) and imidazole (1.32 g, 19.4 mmol, 2.0 eq.) in anhydrous $CH_2Cl_2$ (40 mL) was added TBSCl (1.61 g, 10.7 mmol, 1.1 eq.) in anhydrous $CH_2Cl_2$ (10 mL) via syringe. After stirring for 2 h with the bath maintained at 0 °C, a further portion of TBSCl (80 mg, 0.53 mmol, 0.055 eq.) was added in anhydrous $CH_2Cl_2$ (1 mL). Then the bath was removed, and the reaction mixture warmed to rt. After stirring for a further 2 h 45 min at rt, the reaction mixture was poured into a separatory funnel and washed with $H_2O$ (3x 40 mL). The combined aqueous layers were back extracted with $CH_2Cl_2$ (3x 50 mL). The combined organic layers were washed with brine (50 mL), dried ($Na_2SO_4$), filtered, and solvent was removed in vacuo. The crude product was purified via flash column chromatography (3:1 Hexanes/EtOAc). Appropriate fractions were pooled, and solvent was removed in vacuo to yield **1c** (2.16 g, 7.93 mmol, 81%) as a colorless oil.

**Analytical Data for 1c:**

$R_f$ = 0.42 (7:3 Hexanes/EtOAc)

$[\alpha]_D^{20}$ = +9.1° (c = 1.90, $CH_2Cl_2$)

$^1$H NMR (600 MHz, $CDCl_3$) δ 5.80 (ddt, *J* = 17.2, 10.2, 7.0 Hz, 1H), 5.13 – 5.03 (m, 2H), 4.30 (dq, *J* = 6.2, 3.0 Hz, 1H), 4.20 (dq, *J* = 9.4, 6.1 Hz, 1H), 3.80 – 3.74 (m, 2H), 3.53 – 3.47 (m, 1H), 2.40 – 2.32 (m, 1H), 2.31 – 2.23 (m, 1H), 1.91 (ddd, *J* = 13.1, 5.8, 2.7 Hz, 1H), 1.82 – 1.74 (m, 2H), 0.89 (s, 9H), 0.06 (s, 6H).

$^{13}$C NMR (151 MHz, $CDCl_3$) δ 134.67, 117.22, 86.61, 77.87, 74.81, 64.42, 40.04, 39.96, 26.05, 18.44, -5.24, -5.29.

HRMS (ESI): Anal. Calcd. for $C_{14}H_{29}O_3Si^+$ $[M+H]^+$ 273.1881, found 273.1889

IR (neat): $\nu_{max}\ (cm^{-1})$ = 3385 (br, OH), 3077 (w, C=*CH*), 2954 (m, CH), 2929 (m, CH), 2858 (m, CH), 1643 (w, C=C).

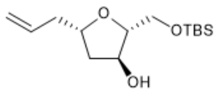
O
OTBS
OH

**1c**

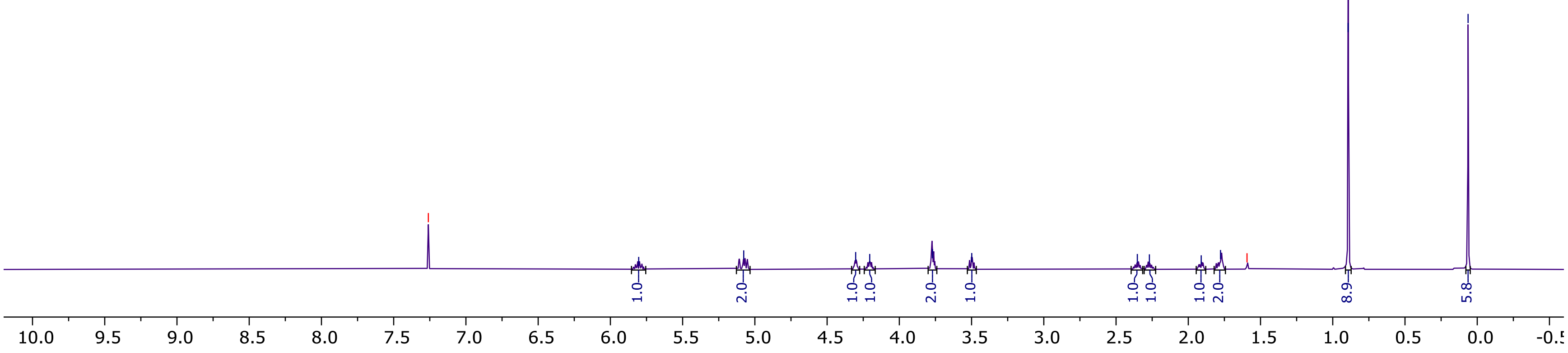
1H
CDCl3
600.13 MHz
7.26 CDCl3
5.80
5.08
4.30
4.21
3.77
3.50
2.35
2.27
1.91
1.78
1.59 H2O
0.89
0.06
1.0
2.0
1.0
1.0
2.0
1.0
1.0
1.0
1.0
2.0
8.9
5.8
10.0 9.5 9.0 8.5 8.0 7.5 7.0 6.5 6.0 5.5 5.0 4.5 4.0 3.5 3.0 2.5 2.0 1.5 1.0 0.5 0.0

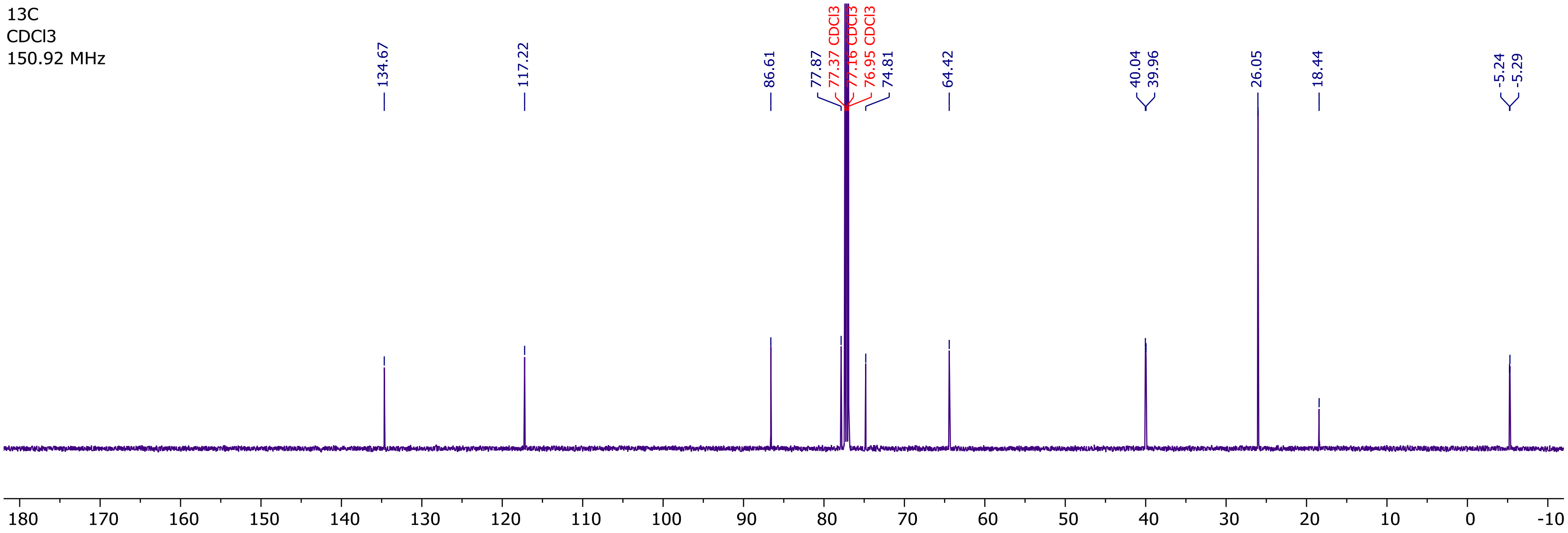
13C
CDCl3
150.92 MHz
134.67
117.22
86.61
77.87
77.37 CDCl3
77.16 CDCl3
76.95 CDCl3
74.81
64.42
40.04
39.96
26.05
18.44
-5.24
-5.29
180 170 160 150 140 130 120 110 100 90 80 70 60 50 40 30 20 10 0 -10

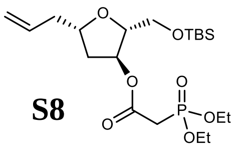

**S8**

To a stirred solution of **1c** (922 mg, 3.38 mmol, 1.0 eq.) and DIC (1.15 mL, 937 mg, 7.44 mmol, 1.6 eq.) in anhydrous $CH_2Cl_2$ (20 mL) was added diethylphosphonoacetic acid (0.92 mL, 1.13 g, 5.75 mmol, 1.7 eq.). After stirring 3 min at rt an exothermic reaction takes place and the urea byproduct rapidly crashes out of solution. After stirring a further 10 min, the reaction mixture was then poured into a separatory funnel and washed with $H_2O$ (50 mL). Then aqueous layer was back extracted with $CH_2Cl_2$ (3x 30 mL). The combined organic layers were then dried ($Na_2SO_4$), filtered, and solvent was removed in vacuo. The crude product was purified via flash column chromatography (17:3 $CH_2Cl_2$/MeCN). Appropriate fractions were pooled, and solvent was removed in vacuo to yield **S8** (1.35 g, 89%) as a viscous colorless oil.

**Analytical Data for S8:**

$R_f$ = 0.45 ($CH_2Cl_2$-MeCN 8:2)

$[\alpha]_D^{20}$ = +13.0 ° (c = 0.50, MeOH)

$^1$H NMR (600 MHz, $CDCl_3$) δ 5.81 (ddt, *J* = 17.2, 10.2, 7.0 Hz, 1H), 5.25 (d, *J* = 5.9 Hz, 1H), 5.10 (dq, *J* = 17.2, 1.7 Hz, 1H), 5.06 (dq, *J* = 10.1, 1.3 Hz, 1H), 4.21 – 4.09 (m, 5H), 3.95 (td, *J* = 3.7, 1.7 Hz, 1H), 3.76 (dd, *J* = 10.9, 3.3 Hz, 1H), 3.63 (dd, *J* = 10.9, 4.0 Hz, 1H), 2.96 (dd, *J* = 21.6, 1.2 Hz, 2H), 2.40 (dt, *J* = 13.9, 6.8 Hz, 1H), 2.30 (dt, *J* = 13.8, 6.4 Hz, 1H), 1.99 (dd, *J* = 13.6, 4.8 Hz, 1H), 1.79 (ddd, *J* = 13.5, 10.8, 5.9 Hz, 1H), 1.34 (td, *J* = 7.1, 1.4 Hz, 6H), 0.89 (s, 9H), 0.05 (s, 3H), 0.05 (s, 3H).

$^{13}$C NMR (101 MHz, $CDCl_3$) δ 165.57, 165.51, 134.55, 117.24, 85.02, 78.43, 78.42, 63.86, 62.86, 62.82, 62.80, 62.75, 39.50, 38.23, 35.35, 34.02, 26.05, 18.44, 16.51, 16.45, -5.18, -5.35.

HRMS (ESI): Anal. Calcd. for $C_{20}H_{40}O_7SiP^+$ $[M+H]^+$ 451.2275 found 451.2284.

IR (neat): $\nu_{max}$ $(cm^{-1})$ = 3076 (w, C=*CH*), 2983 (m, CH), 2959 (m, CH), 1738 (s, C=O), 1644 (w, C=C), 1259 (s).

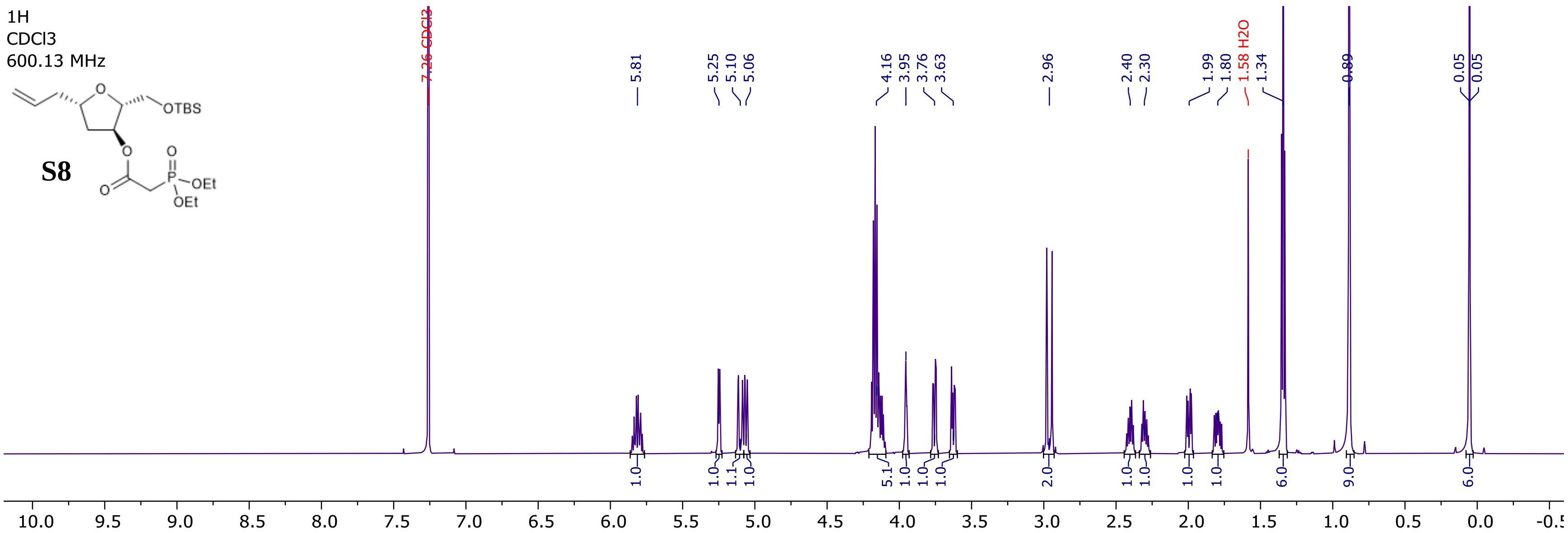
1H
CDCl3
600.13 MHz
OTBS
O
S8
OEt
OEt
P
7.26 CDCl3
5.81
5.25
5.10
5.06
4.16
3.95
3.76
3.63
2.96
2.40
2.30
1.99
1.80
1.58 H2O
1.34
0.89
0.05
0.05
1.0
1.0
1.1
1.0
5.1
1.0
1.0
1.0
2.0
1.0
1.0
1.0
1.0
6.0
9.0
6.0

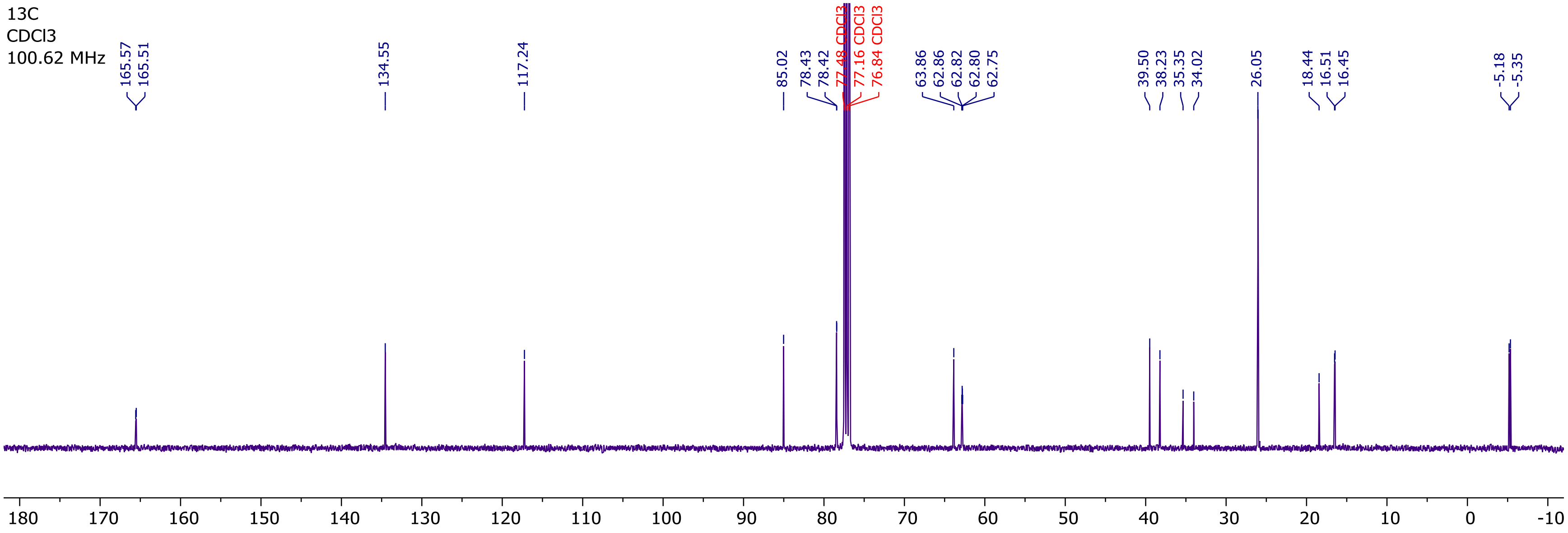
13C
CDCl3
100.62 MHz
165.57
165.51
134.55
117.24
85.02
78.43
78.42
77.48 CDCl3
77.16 CDCl3
76.84 CDCl3
63.86
62.86
62.82
62.80
62.75
39.50
38.23
35.35
34.02
26.05
18.44
16.51
16.45
-5.18
-5.35

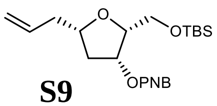


**S9**

To a cold (0 °C), stirred solution of **1c** (1.24 g, 4.50 mmol, 1.0 eq), p-$NO_2$BzOH (1.20 g, 7.2 mmol, 1.6 eq.), and $PPh_3$ (1.94 g, 7.2 mmol, 1.6 eq.) in anhydrous THF (45 mL) was added DIAD (1.46 g, 7.2 mmol, 1.6 eq.) dropwise via syringe. The reaction mixture was stirred for 2 h 45 min while slowly warming to rt, after which time starting material was consumed as monitored by TLC analysis. Then the reaction mixture was quenched with saturated $NaHCO_3$ (45 mL) and the aqueous layer extracted with $Et_2O$ (4x 40 mL). The combined organic extracts were dried ($Na_2SO_4$), filtered, 12 g celite was added, and solvent was removed in vacuo. The crude product, loaded on celite, was purified via flash column chromatography (95:5 to 93:7 to 9:1 Hexanes/$Et_2O$). Appropriate fractions were pooled, and solvent was removed in vacuo to yield **S9** (1.62 g, 85%) as a pale-yellow oil.

**Analytical Data for S9:**

$R_f$ = 0.64 (3:2 Hexanes/$Et_2O$)

$[\alpha]_D^{20}$ = -24.2° (c = 0.52, $CHCl_3$)

$^1$H NMR (400 MHz, $CDCl_3$) δ 8.34 – 8.27 (m, 2H), 8.24 – 8.16 (m, 2H), 5.82 (ddt, *J* = 17.2, 10.2, 6.9 Hz, 1H), 5.64 (ddd, *J* = 6.9, 4.3, 2.8 Hz, 1H), 5.12 – 5.00 (m, 2H), 4.07 (p, *J* = 6.6 Hz, 1H), 4.00 (ddd, *J* = 6.9, 5.6, 4.2 Hz, 1H), 3.89 (d, *J* = 6.3 Hz, 2H), 2.60 – 2.42 (m, 2H), 2.35 (dt, *J* = 13.9, 6.7 Hz, 1H), 1.82 (ddd, *J* = 14.2, 6.8, 2.8 Hz, 1H), 0.80 (s, 9H), -0.01 (s, 3H), -0.07 (s, 3H).

$^{13}$C NMR (101 MHz, $CDCl_3$) δ 164.04, 150.78, 135.82, 134.30, 130.81, 123.76, 117.59, 81.65, 75.70, 61.26, 40.42, 38.15, 25.89, 18.33, -5.27, -5.36.

HRMS (ESI): Anal. Calcd. for $C_{21}H_{32}NO_6Si^+$ $[M+H]^+$ 422.1993, found 422.2021

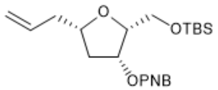
O
OTBS
OPNB

**S9**

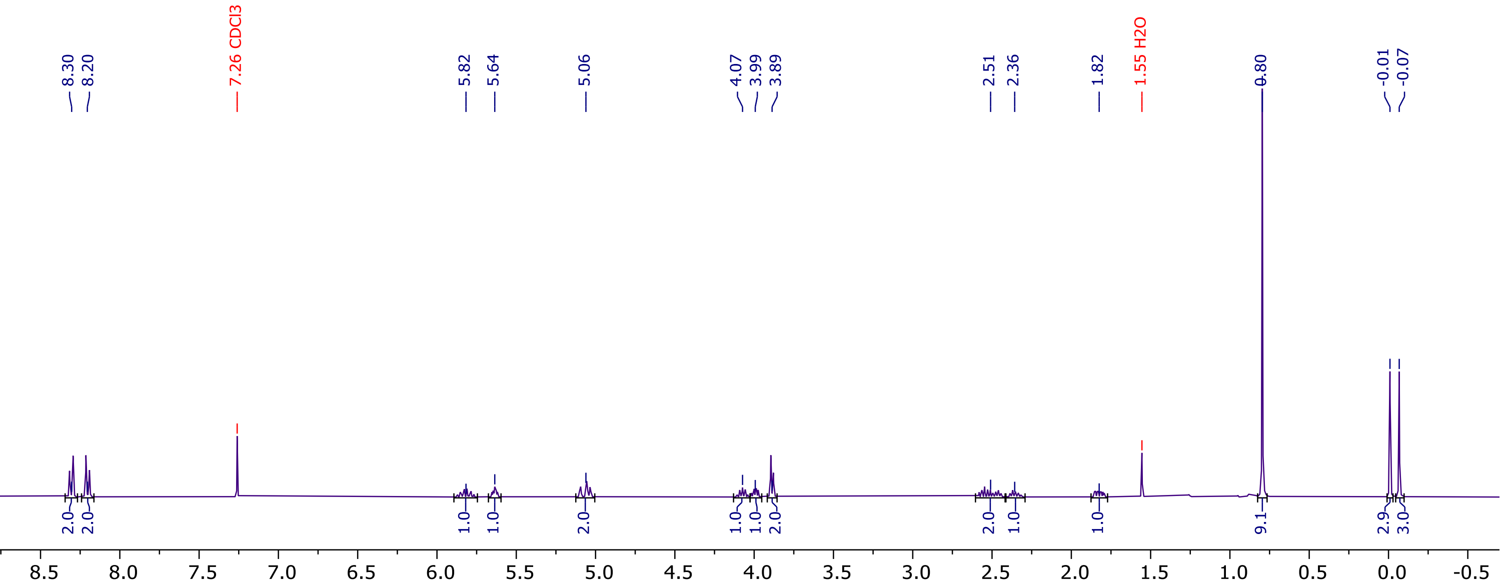
1H
CDCl3
400.13 MHz
8.30
8.20
7.26 CDCl3
5.82
5.64
5.06
4.07
3.99
3.89
2.51
2.36
1.82
1.55 H2O
0.80
-0.01
-0.07
2.0
2.0
1.0
1.0
2.0
1.0
1.0
2.0
2.0
1.0
1.0
9.1
2.9
3.0
10.0 9.5 9.0 8.5 8.0 7.5 7.0 6.5 6.0 5.5 5.0 4.5 4.0 3.5 3.0 2.5 2.0 1.5 1.0 0.5 0.0 -0.5

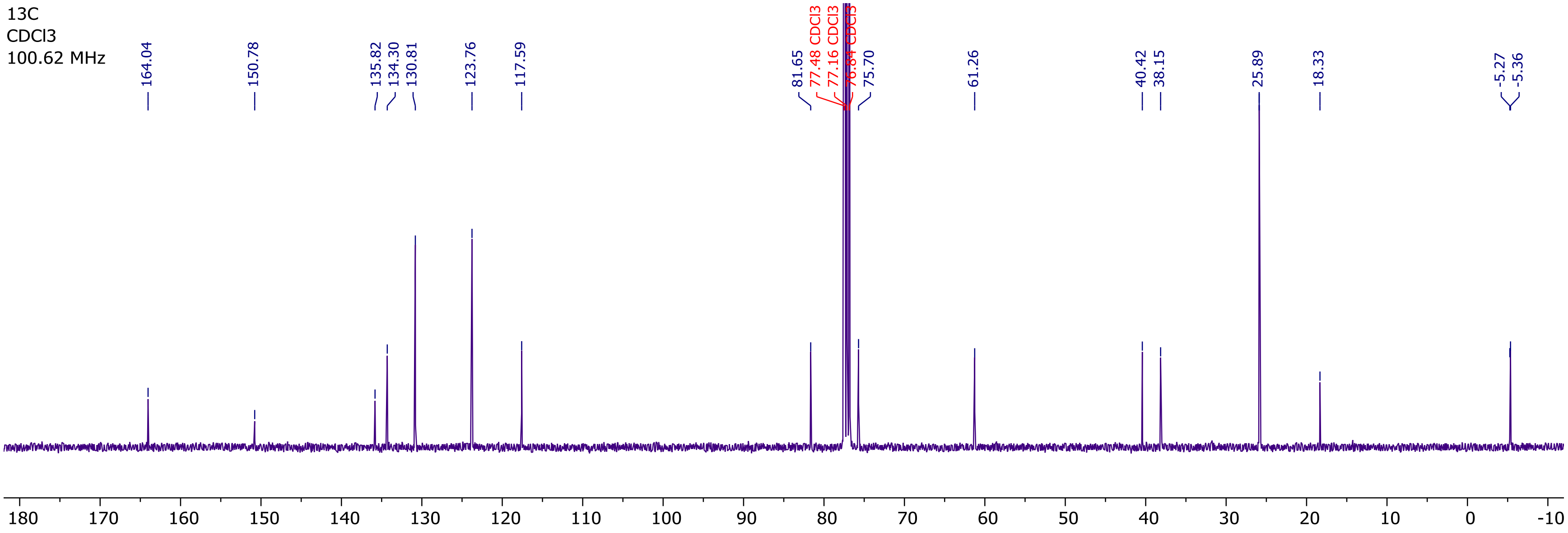
13C
CDCl3
100.62 MHz
164.04
150.78
135.82
134.30
130.81
123.76
117.59
81.65
77.48 CDCl3
77.16 CDCl3
76.84 CDCl3
75.70
61.26
40.42
38.15
25.89
18.33
-5.27
-5.36
180 170 160 150 140 130 120 110 100 90 80 70 60 50 40 30 20 10 0 -10

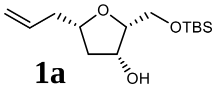


**1a**

Reaction performed open to atmosphere. To **S9** (1.62 g, 3.85 mmol, 1 eq.) in a 100 mL round bottomed flask was added 45 mL of 7N $NH_3$ in MeOH (excess). The resultant reaction mixture stirred for 1 h 30 min, after which time starting material was consumed as monitored by TLC analysis. Then the solvent was removed in vacuo. The crude product was purified via flash column chromatography (9:1 to 3:17 to 4:1 Hexanes/$Et_2O$). Appropriate fractions were pooled, and solvent was removed in vacuo to yield **1a** (937 mg, 89%) as a colorless oil.

**Analytical Data for 1a:**

$R_f$ = 0.40 (3:2 Hexanes/$Et_2O$)

$[\alpha]_D^{20}$ = -10° (c = 0.58, $CH_2Cl_2$)

$^1$H NMR (600 MHz, $CDCl_3$) δ 5.83 (ddt, *J* = 17.1, 10.1, 7.0 Hz, 1H), 5.11 (dq, *J* = 17.2, 1.6 Hz, 1H), 5.07 (d, *J* = 10.2 Hz, 1H), 4.48 (p, *J* = 5.5 Hz, 1H), 3.97 – 3.86 (m, 3H), 3.78 (q, *J* = 4.8 Hz, 1H), 3.34 (d, *J* = 5.8 Hz, 1H), 2.44 (dt, *J* = 13.8, 6.8 Hz, 1H), 2.39 – 2.27 (m, 2H), 1.63 (ddd, *J* = 12.8, 7.8, 4.6 Hz, 1H), 0.90 (s, 9H), 0.11 (s, 3H), 0.10 (s, 3H).

$^{13}$C NMR (151 MHz, $CDCl_3$) δ 134.98, 117.24, 80.58, 77.29, 73.96, 62.87, 40.98, 40.48, 25.95, 18.30, -5.24, -5.39.

HRMS (ESI): Anal. Calcd. for $C_{14}H_{32}NO_3Si^+$ $[M+NH_4]^+$ 290.2146, found 290.2144

IR (neat): $\nu_{max}$ $(cm^{-1})$ = 3457 (br, OH), 3077 (w, C=*CH*), 2931 (m, CH), 2858 (m, CH), 1642 (w, C=C), 1255 (s).

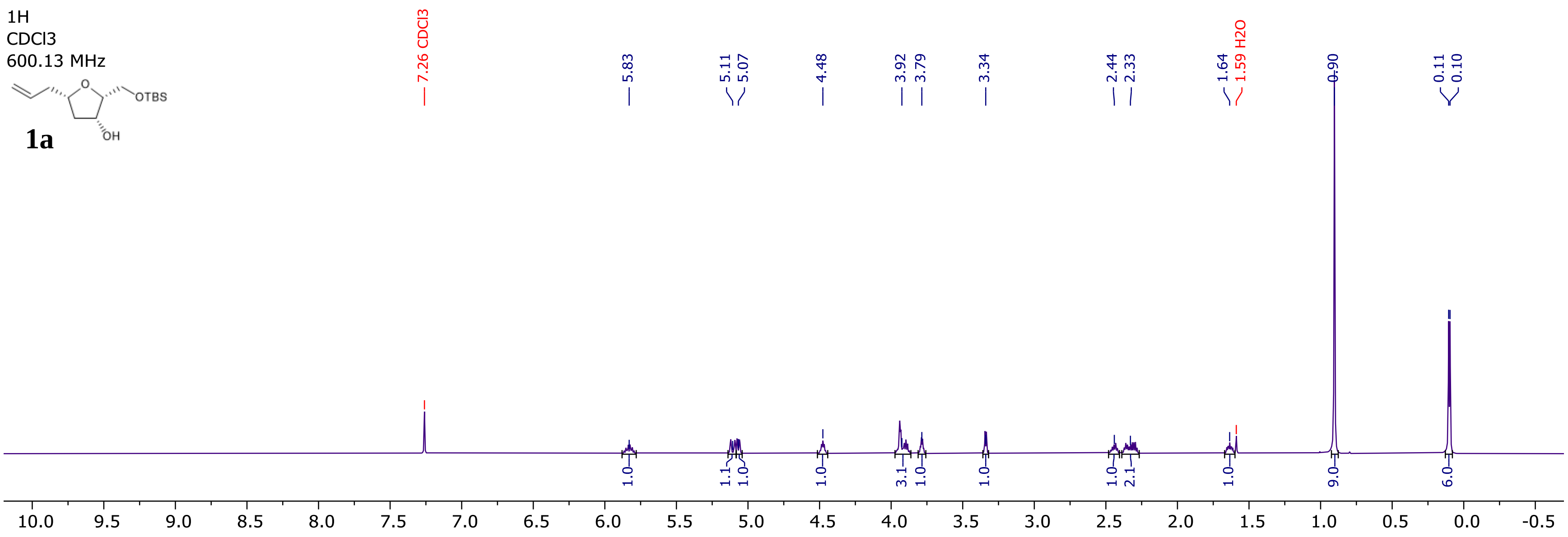
1H
CDCl3
600.13 MHz
1a
OTBS
OH
7.26 CDCl3
5.83
5.11
5.07
4.48
3.92
3.79
3.34
2.44
2.33
1.64
1.59 H2O
0.90
0.11
0.10
1.0
1.1
1.0
1.0
3.1
1.0
1.0
1.0
2.1
1.0
9.0
6.0
10.0 9.5 9.0 8.5 8.0 7.5 7.0 6.5 6.0 5.5 5.0 4.5 4.0 3.5 3.0 2.5 2.0 1.5 1.0 0.5 0.0 -0.5

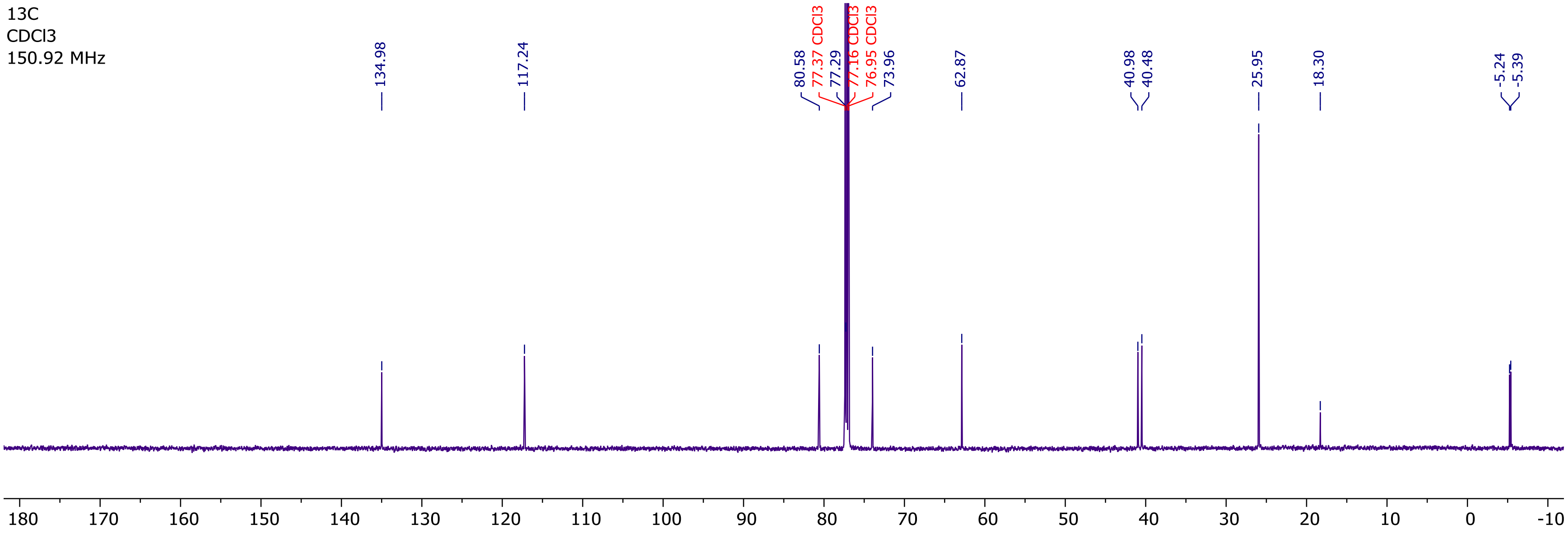
13C
CDCl3
150.92 MHz
134.98
117.24
80.58
77.37 CDCl3
77.29
77.16 CDCl3
76.95 CDCl3
73.96
62.87
40.98
40.48
25.95
18.30
-5.24
-5.39
180 170 160 150 140 130 120 110 100 90 80 70 60 50 40 30 20 10 0 -10

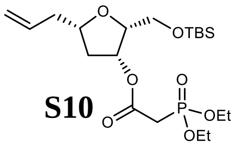


**S10**

To a stirred solution of **1a** (925 mg, 3.39 mmol, 1.0 eq.) and DIC (1.16 mL, 942 mg, 7.46 mmol, 1.6 eq.) in $CH_2Cl_2$ (20 mL) was added diethylphosphonoacetic acid (0.93 mL, 1.13 g, 5.76 mmol, 1.7 eq.). After stirring 3 min at rt an exothermic reaction takes place and the urea byproduct rapidly crashes out of solution. After stirring a further 10 min, the reaction mixture was then poured into a separatory funnel and washed with $H_2O$ (50 mL). Then aqueous layer was back extracted with $CH_2Cl_2$ (3x 30 mL). The combined organic layers were then dried ($Na_2SO_4$), filtered, and solvent was removed in vacuo. The crude product was purified via flash column chromatography (17:3 $CH_2Cl_2$/MeCN). Appropriate fractions were pooled, and solvent was removed in vacuo to yield **S10** (1.33 g, 87%) as a viscous colorless oil.

**Analytical Data for S10:**

$R_f$ = 0.45 ($CH_2Cl_2$-MeCN 8:2)

$[\alpha]_D^{20}$ = -21.2° (c = 2.76, $CH_2Cl_2$)

$^1$H NMR (600 MHz, $CDCl_3$) δ 5.79 (ddt, *J* = 17.1, 10.2, 6.9 Hz, 1H), 5.34 (ddd, *J* = 6.7, 4.1, 2.6 Hz, 1H), 5.09 (dq, *J* = 17.2, 1.6 Hz, 1H), 5.05 (ddt, *J* = 10.3, 2.2, 1.2 Hz, 1H), 4.18 (dqd, *J* = 8.3, 7.1, 1.2 Hz, 4H), 3.95 (p, *J* = 6.6 Hz, 1H), 3.85 (ddd, *J* = 6.6, 5.2, 4.0 Hz, 1H), 3.81 (d, *J* = 5.5 Hz, 2H), 3.02 – 2.88 (m, 2H), 2.50 – 2.39 (m, 2H), 2.30 (dt, *J* = 14.1, 6.9 Hz, 1H), 1.68 (ddd, *J* = 14.2, 6.8, 2.6 Hz, 1H), 1.35 (t, *J* = 7.1 Hz, 6H), 0.87 (s, 8H), 0.05 (s, 3H), 0.05 (s, 3H).

$^{13}$C NMR (151 MHz, $CDCl_3$) δ 165.35, 165.31, 134.58, 117.32, 81.73, 77.48, 75.53, 62.84, 62.80, 62.76, 61.41, 40.34, 38.31, 34.91, 34.01, 26.00, 18.43, 16.55, 16.54, 16.51, 16.50, -5.14, -5.25.

HRMS (ESI): Anal. Calcd. for $C_{20}H_{43}NO_7SiP^+$ $[M+NH_4]^+$ 468.2541, found 468.2546.

IR (neat): $\nu_{max}$ $(cm^{-1})$ = 3076 (w, C=*CH*), 2932 (m, CH), 2858 (m, CH), 1738 (s, C=O), 1647 (C=C), 1263 (s).

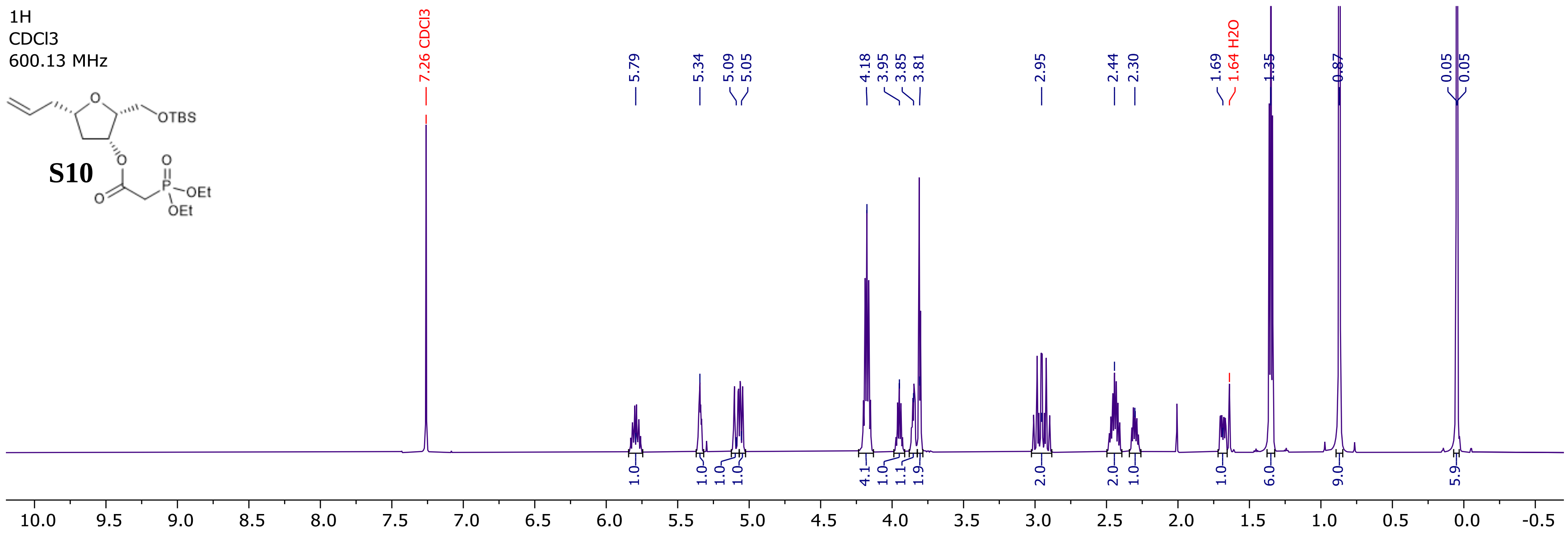
1H
CDCl3
600.13 MHz
S10
OTBS
OEt
OEt
7.26 CDCl3
5.79
5.34
5.09
5.05
4.18
3.95
3.85
3.81
2.95
2.44
2.30
1.69
1.64 H2O
1.35
0.87
0.05
0.05
1.0
1.0
1.0
1.0
4.1
1.0
1.1
1.9
2.0
2.0
1.0
1.0
6.0
9.0
5.9
10.0
9.5
9.0
8.5
8.0
7.5
7.0
6.5
6.0
5.5
5.0
4.5
4.0
3.5
3.0
2.5
2.0
1.5
1.0
0.5
0.0
-0.5

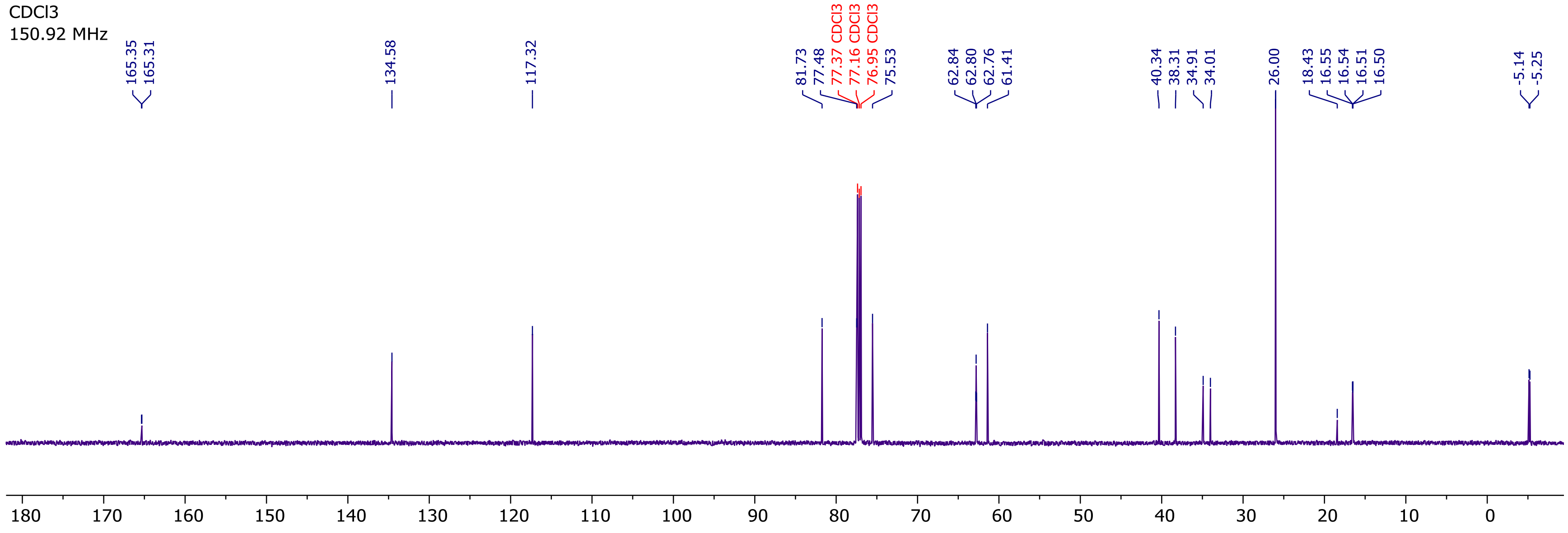
CDCl3
150.92 MHz
165.35
165.31
134.58
117.32
81.73
77.48
77.37 CDCl3
77.16 CDCl3
76.95 CDCl3
75.53
62.84
62.80
62.76
61.41
40.34
38.31
34.91
34.01
26.00
18.43
16.55
16.54
16.51
16.50
-5.14
-5.25
180
170
160
150
140
130
120
110
100
90
80
70
60
50
40
30
20
10
0

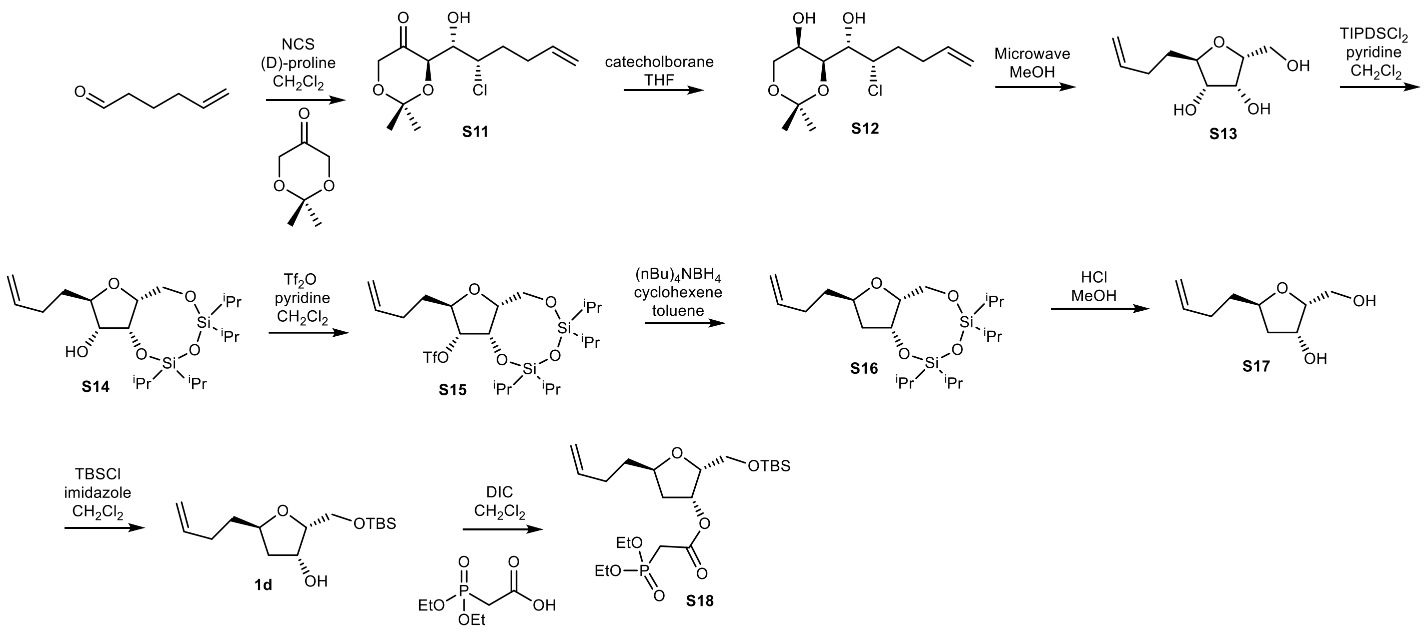


**Supplementary Fig. 28 | Synthesis of Tetrahydrofuranol 1d and Phosphonate S18.**
NCS = N-chlorosuccinimide, THF = tetrahydrofuran, TIPDSCl$_2$ = 1,3-dichloro-1,1,3,3-tetraisopropyldisiloxane, Tf$_2$O = trifluoromethylsulfonic anhydride, TfO = trifluoromethylsulfonate, TBSCl = tert-butyldimethylsilyl chloride, TBS = tert-butyldimethylsilyl, DIC = N,N'-diisopropylcarbodiimide.

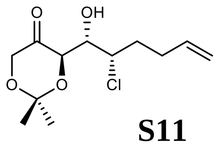


**S11**

Note: Reaction performed in capped vessel under ambient atmosphere.

To a cold (0 °C), stirred solution of hex-5-enal (7.805 g, 79.5 mmol, 1.0 eq.) in $CH_2Cl_2$ (400 mL) was added (*R*)-proline (7. 379 g, 64.1 mmol, 0.8 eq.) and NCS (10.686 g, 80.0 mmol, 1.0 eq). The mixture was stirred at 0 °C for 1 h, followed by the addition of 2,2-dimethyl-1,3-dioxan-5-one (9.396 mL, 80.0 mmol, 1.0 eq). The mixture was allowed to warm to room temperature and was stirred for 47 h, monitored by crude NMR. 1:1 water/brine (300 mL) was added and the aqueous layer was extracted with $CH_2Cl_2$ (2x 200 mL). The combined organic layers were dried ($Na_2SO_4$), filtered, and solvent was removed in vacuo. The crude product was purified via flash column chromatography (9:1 hexanes/EtOAc). Appropriate fractions were pooled, and solvent was removed in vacuo to yield **S11** (9.193 g, 44%) as a yellow oil.

**Analytical data for S11:**

$R_f$ = 0.18 (9:1 hexanes/EtOAc)

$[\alpha]_D^{20}$ = +95 ° (c = 0.99, MeOH)

$^1$H NMR (400 MHz, $CDCl_3$) δ 5.87 – 5.71 (m, 1H), 5.07 (dd, *J* = 17.1, 1.7 Hz, 1H), 5.04 – 4.97 (m, 1H), 4.41 (dd, *J* = 8.7, 1.5 Hz, 1H), 4.28 (dd, *J* = 17.6, 1.5 Hz, 1H), 4.20 (ddd, *J* = 9.3, 4.7, 1.8 Hz, 1H), 4.07 (d, *J* = 17.6 Hz, 1H), 3.92 (dd, *J* = 8.7, 1.8 Hz, 1H), 3.37 (s, 1H), 2.40 – 2.27 (m, 1H), 2.26 – 2.02 (m, 2H), 1.86 (dddd, *J* = 14.1, 9.2, 6.4, 4.6 Hz, 1H), 1.51 (s, 3H), 1.42 (s, 3H).

$^{13}$C NMR (101 MHz, $CDCl_3$) δ 212.29, 137.25, 115.77, 101.76, 73.01, 71.95, 66.61, 61.66, 33.78, 30.95, 23.94, 23.60.

HRMS (ESI): Anal. Calcd. for $C_{12}H_{20}ClO_4^+$ $[M+H]^+$ 263.1045, found 263.1056

IR (neat): $\nu_{max}$ $(cm^{-1})$ = 3511 (b, OH), 3079 (w, C=CH), 2990 (m, CH), 1739 (s, C=O), 1643 (w, C=C), 1415 (s), 1380 (s), 1223 (s)

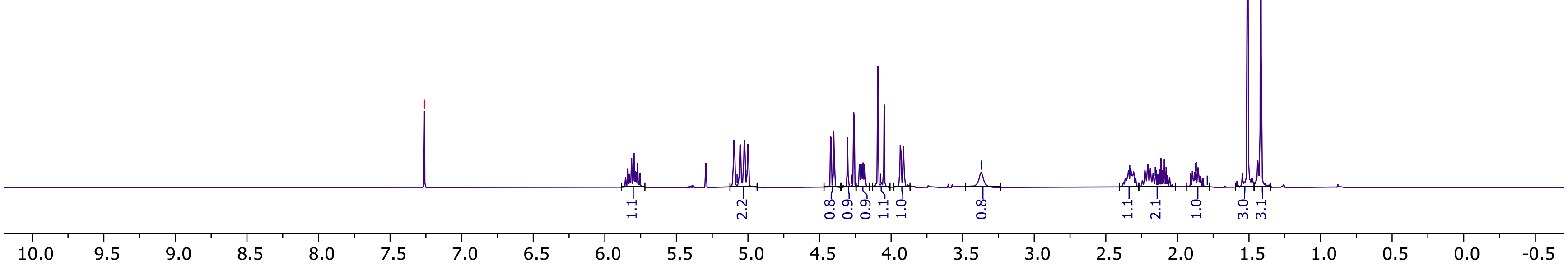
1H
CDCl3
400.13 MHz
7.26 CDCl3
5.80
5.08
5.01
4.41
4.28
4.20
4.07
3.92
3.37
2.33
2.16
1.87
1.79
1.51
1.42
1.1
2.2
0.8
0.9
0.9
1.1
1.0
0.8
1.1
2.1
1.0
3.0
3.1
10.0 9.5 9.0 8.5 8.0 7.5 7.0 6.5 6.0 5.5 5.0 4.5 4.0 3.5 3.0 2.5 2.0 1.5 1.0 0.5 0.0 -0.5

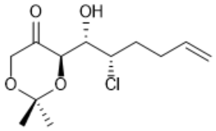
O
OH
O
O
Cl

**S11**

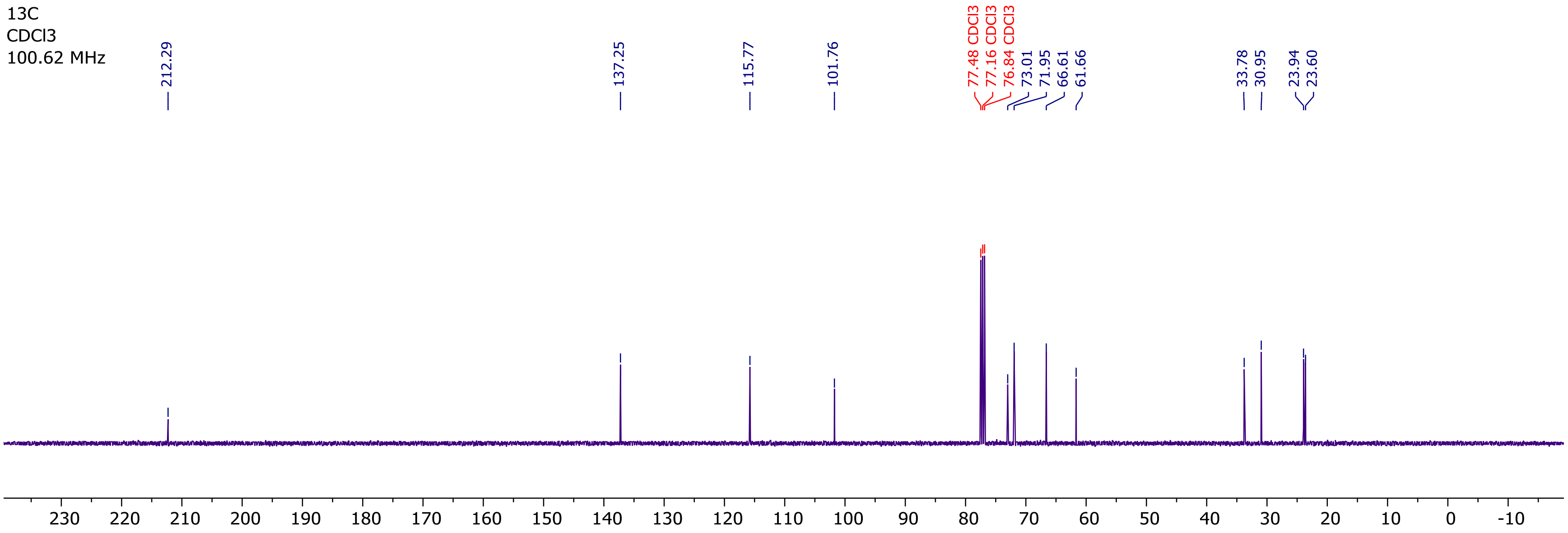
13C
CDCl3
100.62 MHz
212.29
137.25
115.77
101.76
77.48 CDCl3
77.16 CDCl3
76.84 CDCl3
73.01
71.95
66.61
61.66
33.78
30.95
23.94
23.60
230 220 210 200 190 180 170 160 150 140 130 120 110 100 90 80 70 60 50 40 30 20 10 0 -10

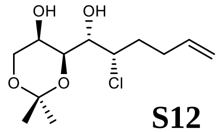


**S12**

To a round-bottom flask containing **S11** (956.4 mg, 3.64 mmol, 1.0 eq.) was added anhydrous THF (35 mL) via syringe. The solution was cooled to 0 °C and 0.1 M catecholborane in THF (8 mL, 8.01 mmol, 2.2 eq.) was added via syringe. The solution was allowed to warm to room temperature and stirred for 1 h. The mixture was diluted with MeOH (35 mL) and a saturated solution of potassium sodium tartrate (30 mL) was added and stirred vigorously for 2 h. Brine (20 mL) was added and the aqueous layer was extracted with EtOAc (4x 40 mL). The combined organic layers were washed with brine (1x 100 mL), dried ($Na_2SO_4$), filtered, and solvent was removed in vacuo. The crude product was purified via flash column chromatography (2:3 to 1:4 hexanes/EtOAc). Appropriate fractions were pooled, and solvent was removed in vacuo to yield **S12** (543.7 mg, 56%) as a brown oil.

**Analytical Data for S12:**

$R_f$ = 0.23 (2:3 hexanes/EtOAc)

$[\alpha]_D^{20}$ = -10.2 ° (c = 1.01, $CDCl_3$)

$^1$H NMR (500 MHz, $CDCl_3$) δ 5.80 (ddt, *J* = 17.0, 10.2, 6.6 Hz, 1H), 5.08 (dq, *J* = 17.1, 1.7 Hz, 1H), 5.05 – 4.98 (m, 1H), 4.27 (ddd, *J* = 9.8, 4.6, 1.3 Hz, 1H), 4.08 (dd, *J* = 12.3, 1.6 Hz, 1H), 3.93 – 3.82 (m, 3H), 3.72 (dq, *J* = 10.9, 1.8 Hz, 1H), 2.70 (d, *J* = 10.9 Hz, 1H), 2.39 – 2.28 (m, 1H), 2.25 – 2.15 (m, 2H), 2.11 – 2.00 (m, 1H), 1.86 (dddd, *J* = 14.0, 9.2, 6.7, 4.6 Hz, 1H), 1.46 (s, 3H), 1.41 (s, 3H).

$^{13}$C NMR (126 MHz, $CDCl_3$) δ 137.21, 115.80, 99.38, 72.33, 71.26, 66.03, 63.68, 62.83, 34.49, 31.02, 29.53, 18.46.

HRMS (ESI): Anal. Calcd. for $C_{12}H_{22}ClO_4^+$ $[M+H]^+$ 265.1201, found 265.1205

IR (neat): $\nu_{max}$ $(cm^{-1})$ = 3430 (br, OH), 3079 (w, C=*CH*), 2941 (m, CH), 1642 (w, C=C), 1382 (m, CH)

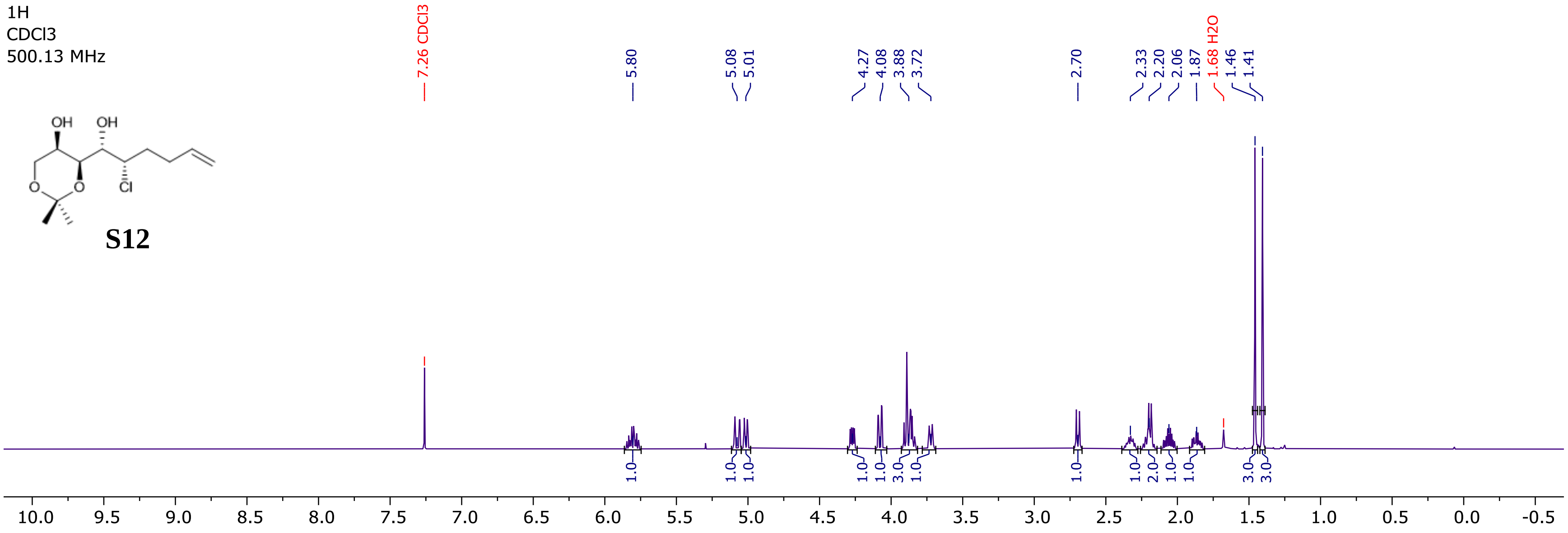
1H
CDCl3
500.13 MHz
7.26 CDCl3
5.80
5.08
5.01
4.27
4.08
3.88
3.72
2.70
2.33
2.20
2.06
1.87
1.68 H2O
1.46
1.41
1.0
1.0
1.0
1.0
1.0
3.0
1.0
1.0
1.0
2.0
1.0
1.0
3.0
3.0
10.0 9.5 9.0 8.5 8.0 7.5 7.0 6.5 6.0 5.5 5.0 4.5 4.0 3.5 3.0 2.5 2.0 1.5 1.0 0.5 0.0 -0.5
OH
OH
O
O
Cl

**S12**

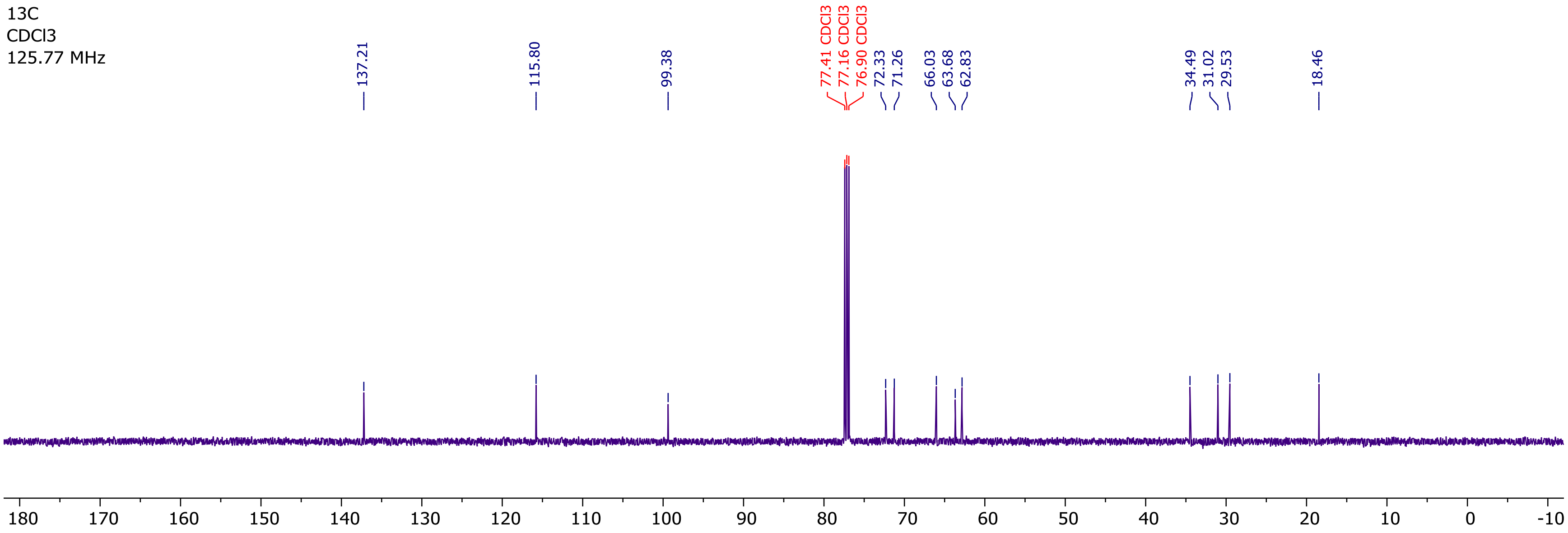
13C
CDCl3
125.77 MHz
137.21
115.80
99.38
77.41 CDCl3
77.16 CDCl3
76.90 CDCl3
72.33
71.26
66.03
63.68
62.83
34.49
31.02
29.53
18.46
180 170 160 150 140 130 120 110 100 90 80 70 60 50 40 30 20 10 0 -10

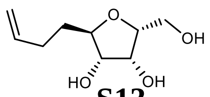

**S13**

**S12** (543.7 mg, 2.05 mmol) was dissolved in 20 mL MeOH and heated to 120 °C (with a maximum pressure of 300 psi) in a microwave reactor for 1.5 hours. The resultant dark brown solution was concentrated in vacuo and the crude product was purified via flash column chromatography (97:3 $CH_2Cl_2$/MeOH). Appropriate fractions were pooled, and solvent removed in vacuo to yield **S13** (221.7 mg, 57%) as a brown oil.

**Analytical Data for S13:**

$R_f$ = 0.17 (47:3 $CH_2Cl_2$/MeOH)

$[\alpha]_D^{20}$ = +38 ° (c = 0.72, MeOH)

$^1$H NMR (400 MHz, $CDCl_3$) δ 5.84 (ddt, *J* = 16.9, 10.2, 6.6 Hz, 1H), 5.06 (ddd, *J* = 17.0, 3.5, 1.7 Hz, 1H), 4.98 (dq, *J* = 10.2, 1.5 Hz, 1H), 4.35 (q, *J* = 4.6 Hz, 1H), 4.08 (dt, *J* = 5.3, 3.5 Hz, 1H), 3.94 – 3.83 (m, 4H), 3.77 (d, *J* = 5.7 Hz, 1H), 3.27 (s, 1H), 2.86 (s, 1H), 2.31 – 2.08 (m, 2H), 1.76 – 1.55 (m, 2H).

$^{13}$C NMR (101 MHz, $CDCl_3$) δ 138.14, 115.12, 82.58, 78.34, 76.58, 73.40, 62.05, 32.84, 30.02.

HRMS (ESI): Anal. Calcd. for $C_9H_{17}O_4^+$ $[M+H]^+$ 189.1121, found 189.1126

IR (neat): $\nu_{max}$ $(cm^{-1})$ = 3305 (br, OH), 2988 (w, CH), 2831 (m, CH)

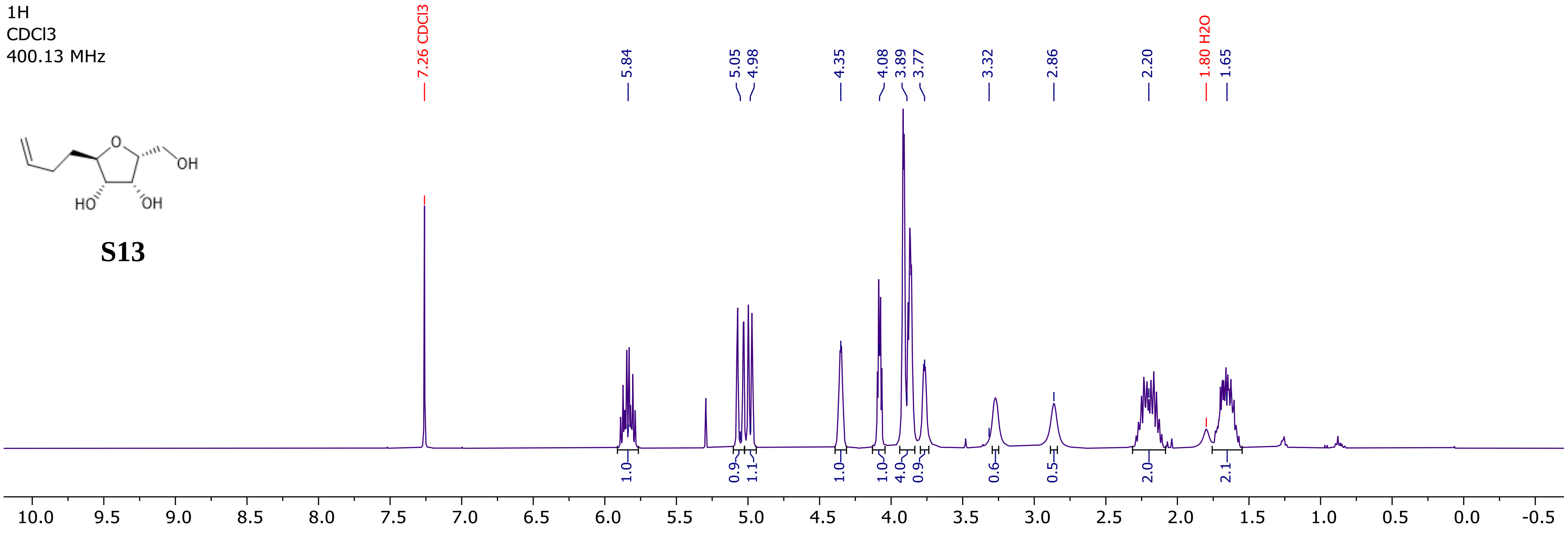
1H
CDCl3
400.13 MHz
7.26 CDCl3
5.84
5.05
4.98
4.35
4.08
3.89
3.77
3.32
2.86
2.20
1.80 H2O
1.65
1.0
0.9
1.1
1.0
1.0
4.0
0.9
0.6
0.5
2.0
2.1
S13
10.0 9.5 9.0 8.5 8.0 7.5 7.0 6.5 6.0 5.5 5.0 4.5 4.0 3.5 3.0 2.5 2.0 1.5 1.0 0.5 0.0 -0.5

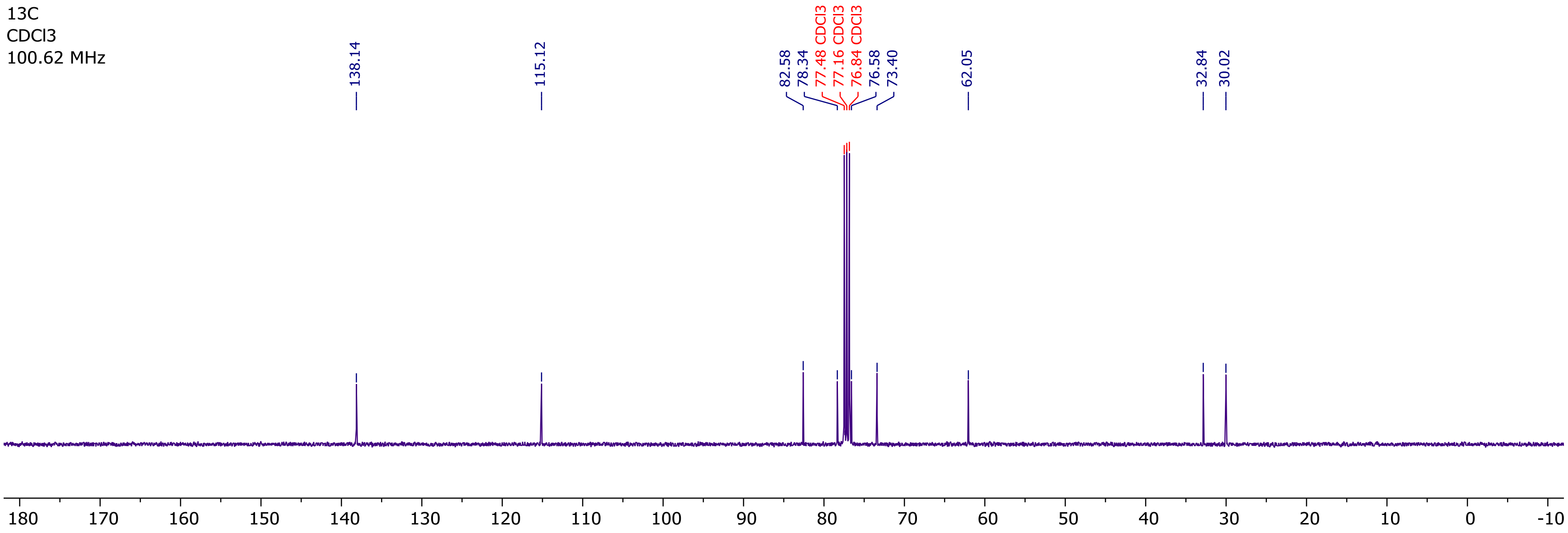
13C
CDCl3
100.62 MHz
138.14
115.12
82.58
78.34
77.48 CDCl3
77.16 CDCl3
76.84 CDCl3
76.58
73.40
62.05
32.84
30.02
180 170 160 150 140 130 120 110 100 90 80 70 60 50 40 30 20 10 0 -10

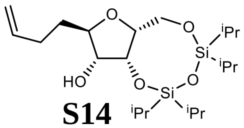

**S14**

To a cold (0 °C), stirred solution of **S13** (213 mg, 1.13 mmol, 1.0 eq) in anhydrous $CH_2Cl_2$ (3.3 mL) was added in sequence anhydrous pyridine (1.1 mL) and $TIPDSiCl_2$ (0.36 mL, 355 mg, 1.13 mmol, 1.0 eq.) via syringe. The reaction mixture was then allowed to warm to rt and stirred further for 3 h, after which time starting material was consumed as monitored by TLC analysis. The reaction mixture was diluted with $CH_2Cl_2$ (10 mL) and washed with $H_2O$ (3x 10 mL). The combined aqueous layers were back extracted with $CH_2Cl_2$ (3x 10 mL), then the combined organic layers were washed with brine (50 mL), dried ($Na_2SO_4$), filtered, and solvent was removed in vacuo. The crude product was purified via flash column chromatography (9:1 hexanes/EtOAc). Appropriate fractions were pooled, and the solvent was removed in vacuo to yield **S14** (405 mg, 0.94 mmol, 83%) as a colorless oil.

**Analytical Data for S14:**

$R_f$ = 0.47 (9:1 hexanes/EtOAc)

$[\alpha]_D^{20}$ = +8.4 ° (c = 1.13, MeOH)

$^1$H NMR (400 MHz, $CDCl_3$) δ 5.85 (ddt, *J* = 16.9, 10.2, 6.5 Hz, 1H), 5.04 (dt, *J* = 17.1, 1.8 Hz, 1H), 4.96 (dq, *J* = 10.2, 1.5 Hz, 1H), 4.36 (dd, *J* = 4.4, 3.0 Hz, 1H), 4.05 (ddd, *J* = 10.2, 4.6, 2.9 Hz, 1H), 3.93 – 3.84 (m, 2H), 3.78 (dd, *J* = 10.1, 4.7 Hz, 1H), 3.67 (td, *J* = 8.0, 4.5 Hz, 1H), 2.30 – 2.08 (m, 3H), 1.83 – 1.59 (m, 2H), 1.13 – 0.99 (m, 28H).

$^{13}$C NMR (101 MHz, $CDCl_3$) δ 138.52, 114.71, 81.54, 79.76, 77.48, 72.69, 59.77, 33.05, 29.94, 17.69, 17.64, 17.63, 17.51, 17.44, 17.38, 17.20, 17.14, 13.50, 13.07, 12.97, 12.67.

HRMS (ESI): Anal. Calcd. for $C_{21}H_{46}NO_5Si_2^+$ $[M+NH_4]^+$ 448.2909, found 448.2922

IR (neat): $\nu_{max}\ (cm^{-1})$ = 2945 (m, CH), 2867 (m, CH), 1642 (w, C=C), 1467 (m, CH), 1389 (m), 1249 (s)

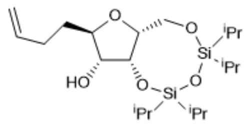

O
O
Si
iPr
iPr
O
HO
O
Si
iPr
iPr


**S14**

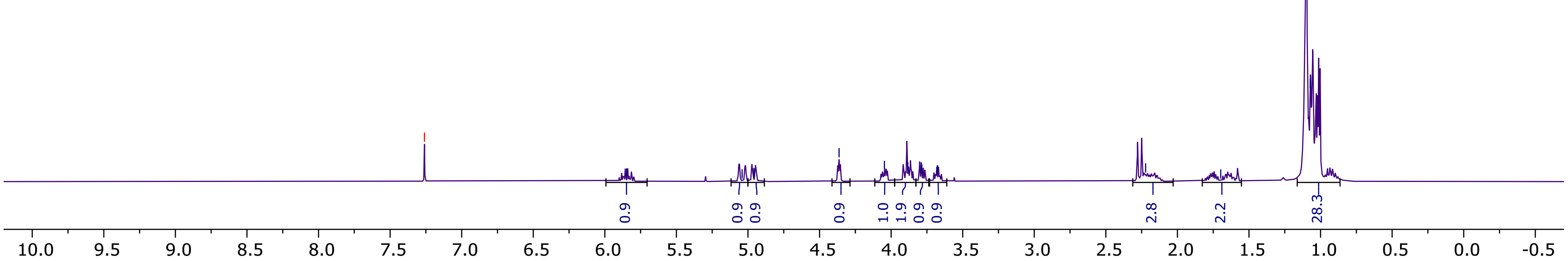

1H
CDCl3
400.13 MHz
7.26 CDCl3
5.85
5.04
4.96
4.36
4.05
3.88
3.79
3.68
2.22
1.70
1.07
0.9
0.9
0.9
0.9
1.0
1.9
0.9
0.9
2.8
2.2
28.3
10.0 9.5 9.0 8.5 8.0 7.5 7.0 6.5 6.0 5.5 5.0 4.5 4.0 3.5 3.0 2.5 2.0 1.5 1.0 0.5 0.0 -0.5


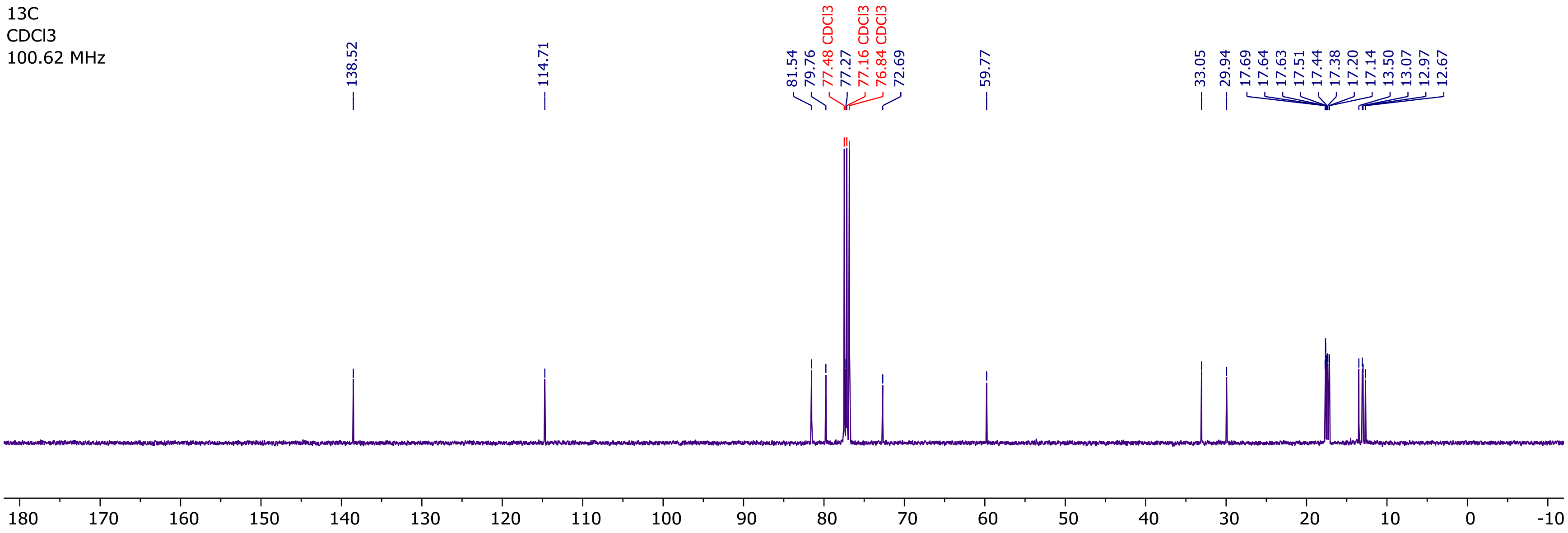

13C
CDCl3
100.62 MHz
138.52
114.71
81.54
79.76
77.48 CDCl3
77.27
77.16 CDCl3
76.84 CDCl3
72.69
59.77
33.05
29.94
17.69
17.64
17.63
17.51
17.44
17.38
17.20
17.14
13.50
13.07
12.97
12.67
180 170 160 150 140 130 120 110 100 90 80 70 60 50 40 30 20 10 0 -10

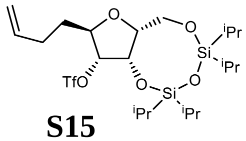


**S15**

To a cold (0°C), stirred solution of **S14** (405 mg, 0.94 mmol, 1.0 eq.) in $CH_2Cl_2$ (6 mL) was added anhydrous pyridine (0.45 mL) followed by $Tf_2O$ (0.4 mL, 66.3 mg, 2.35 mmol, 2.5 eq.) dropwise via syringe. The reaction mixture was then allowed to warm to rt and stirred for 1 h, after which time starting material was consumed as monitored by TLC analysis. The reaction mixture was then cooled (0 °C) and washed with ice-cold saturated $NaHCO_3$ (2x 15 mL). The aqueous layers were extracted with $CH_2Cl_2$ (3x 10 mL) and the combined organic extracts were dried ($Na_2SO_4$), filtered, and solvent was removed in vacuo. The crude product was purified by flash column chromatography (39:1 hexanes/EtOAc). Appropriate fractions were pooled, and the solvent was removed in vacuo to yield **S15** (443 mg, 0.79 mmol, 84%) as a colorless oil.

**Analytical Data for S15:**

$R_f$ = 0.18 (39:1 hexanes/$Et_2O$)

$[\alpha]_D^{20}$ = +35 ° (c = 0.94, MeOH)

$^1$H NMR (400 MHz, $CDCl_3$) δ 5.82 (ddt, *J* = 16.9, 10.2, 6.6 Hz, 1H), 5.05 (dd, *J* = 17.1, 1.7 Hz, 1H), 4.99 (dd, *J* = 10.2, 1.6 Hz, 1H), 4.83 (dd, *J* = 8.8, 3.9 Hz, 1H), 4.64 (t, *J* = 3.3 Hz, 1H), 4.19 (td, *J* = 8.7, 3.1 Hz, 1H), 4.07 (ddd, *J* = 10.1, 4.7, 2.7 Hz, 1H), 3.91 (t, *J* = 10.2 Hz, 1H), 3.79 (dd, *J* = 10.3, 4.7 Hz, 1H), 2.32 – 2.20 (m, 1H), 2.20 – 2.08 (m, 1H), 1.79 (dddd, *J* = 13.3, 9.7, 6.4, 3.1 Hz, 1H), 1.59 (ddt, *J* = 13.7, 9.0, 4.6 Hz, 1H), 1.16 – 0.98 (m, 28H).

$^{13}$C NMR (101 MHz, $CDCl_3$) δ 137.56, 120.32, 117.14, 115.40, 87.61, 79.47, 76.69, 71.43, 59.30, 31.58, 29.55, 17.71, 17.46, 17.43, 17.36, 17.35, 17.33, 17.17, 17.14, 13.50, 13.12, 12.97, 12.77.

HRMS (ESI): Anal. Calcd. For $C_{22}H_{45}F_3NO_7SSi_2^+$ $[M+NH_4]^+$ 580.2402, found 580.2420

IR (neat): $\nu_{max}$ $(cm^{-1})$ = 2948 (m, CH), 2895 (m, CH), 2870 (m, CH), 1643 (w, C=C), 1421 (s), 1248 (s), 1215 (s)

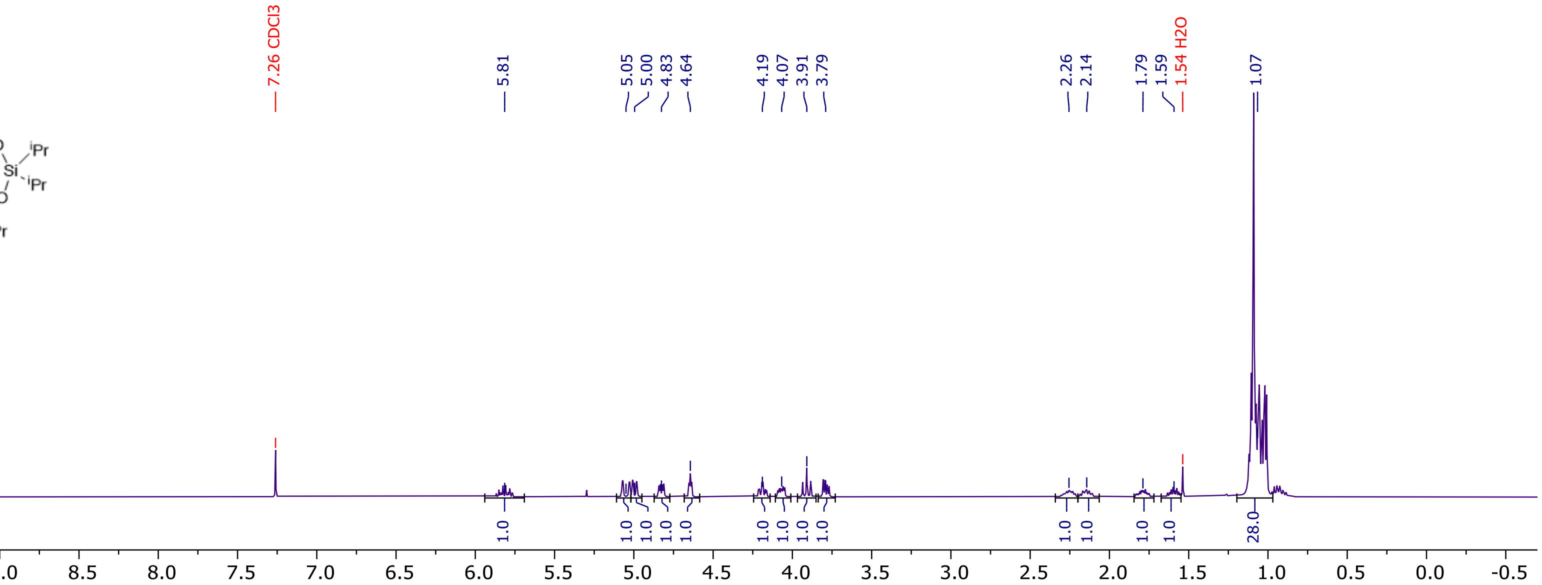
1H
CDCl3
400.13 MHz
7.26 CDCl3
5.81
5.05
5.00
4.83
4.64
4.19
4.07
3.91
3.79
2.26
2.14
1.79
1.59
1.54 H2O
1.07
1.0
1.0
1.0
1.0
1.0
1.0
1.0
1.0
1.0
1.0
1.0
1.0
1.0
28.0
10.0 9.5 9.0 8.5 8.0 7.5 7.0 6.5 6.0 5.5 5.0 4.5 4.0 3.5 3.0 2.5 2.0 1.5 1.0 0.5 0.0 -0.5

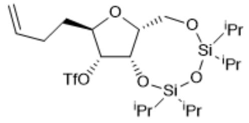
O
TfO
O
Si
O
O
Si
O
iPr
iPr
iPr
iPr

**S15**

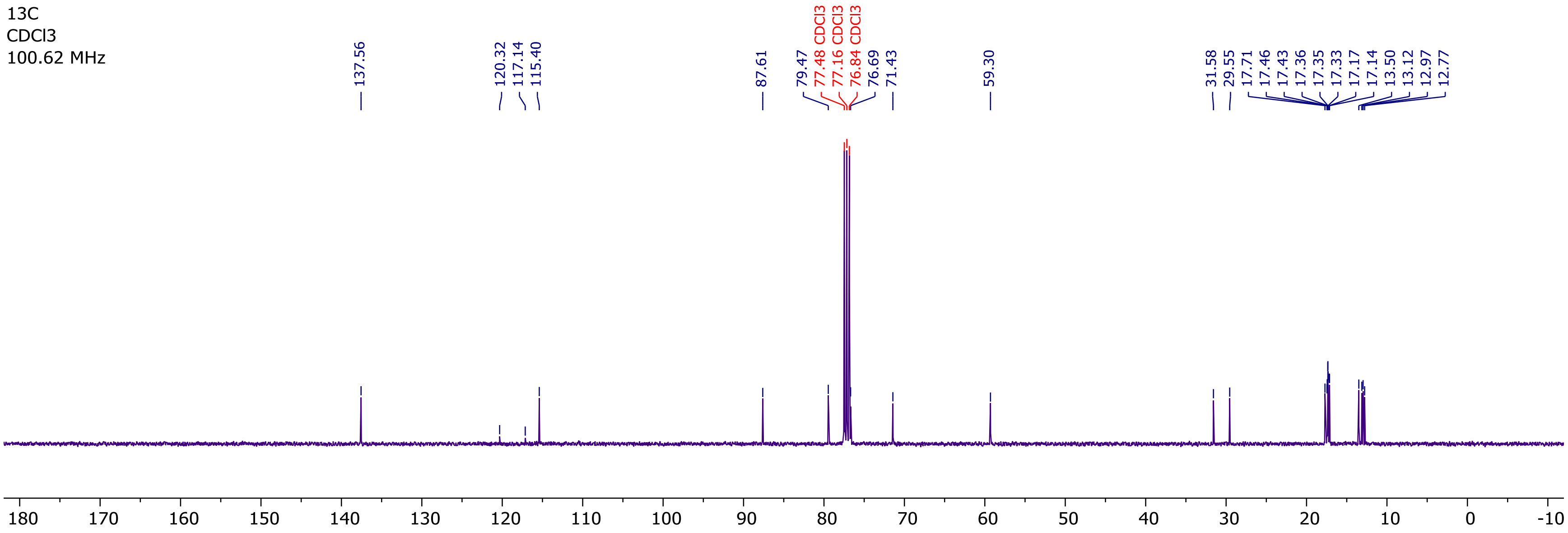
13C
CDCl3
100.62 MHz
137.56
120.32
117.14
115.40
87.61
79.47
77.48 CDCl3
77.16 CDCl3
76.84 CDCl3
76.69
71.43
59.30
31.58
29.55
17.71
17.46
17.43
17.36
17.35
17.33
17.17
17.14
13.50
13.12
12.97
12.77
180 170 160 150 140 130 120 110 100 90 80 70 60 50 40 30 20 10 0 -10

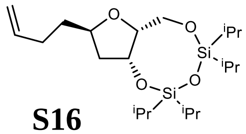

**S16**

To a round-bottom flask containing **S15** (4.763 g, 8.46 mmol, 1.0 eq.) was added anhydrous toluene (150 mL), cyclohexene (17.4 g, 21.4 mL, 211 mmol, 25.0 eq) and $(nBu)_4NBH_4$ (6.56 g, 25.5 mmol, 3.0 eq.). The mixture was heated to 60 °C and stirred for 3.5 h. The mixture was cooled to 0 °C and quenched with a saturated solution of $NaHCO_3$ (75 mL). After bubbling subsided, the aqueous layer was extracted with $Et_2O$ (3x 50 mL). The combined aqueous layers were washed with brine (50 mL) and the brine layer was back-extracted with $Et_2O$ (1x 25 mL). The combined organic layers were dried ($MgSO_4$), filtered, and the solvent was removed in vacuo. The crude product was purified via flash column chromatography (19:1 hexanes/EtOAc) to yield **S16** (1.529 g, 44% yield, 3.7 mmol) as a colorless oil.

**Analytical Data for S16:**

$R_f$ = 0.4 (7:3 toluene/hexanes)

$[\alpha]_D^{20}$ = -10 ° (c = 0.42, MeOH)

$^1$H NMR (500 MHz, $CDCl_3$) δ 5.84 (ddt, *J* = 16.8, 10.1, 6.5 Hz, 1H), 5.03 (dq, *J* = 17.1, 1.8 Hz, 1H), 4.95 (dt, *J* = 10.2, 1.6 Hz, 1H), 4.51 (t, *J* = 3.3 Hz, 1H), 4.25 (dq, *J* = 11.6, 6.0 Hz, 1H), 3.95 (ddd, *J* = 8.6, 5.8, 2.7 Hz, 1H), 3.89 – 3.79 (m, 2H), 2.16 (dddd, *J* = 17.7, 13.2, 7.4, 1.5 Hz, 1H), 2.11 – 2.02 (m, 2H), 1.76 – 1.64 (m, 2H), 1.60 – 1.50 (m, 1H), 1.11 – 0.99 (m, 28H).

$^{13}$C NMR (126 MHz, $CDCl_3$) δ 138.57, 114.60, 81.97, 78.30, 72.08, 59.91, 41.93, 35.33, 30.41, 17.76, 17.63, 17.52, 17.50, 17.44, 17.27, 17.22, 13.53, 13.09, 12.81, 12.59.

HRMS (ESI): Anal. Calcd. for $C_{21}H_{43}O_4Si_2^+$ $[M+H]^+$ 415.2694, found 415.2695

IR (neat): $\nu_{max}$ $(cm^{-1})$ = 2942 (m, CH), 2867 (m, CH), 1642 (w, C=C), 1467 (m, CH), 1389 (m), 1249 (m)

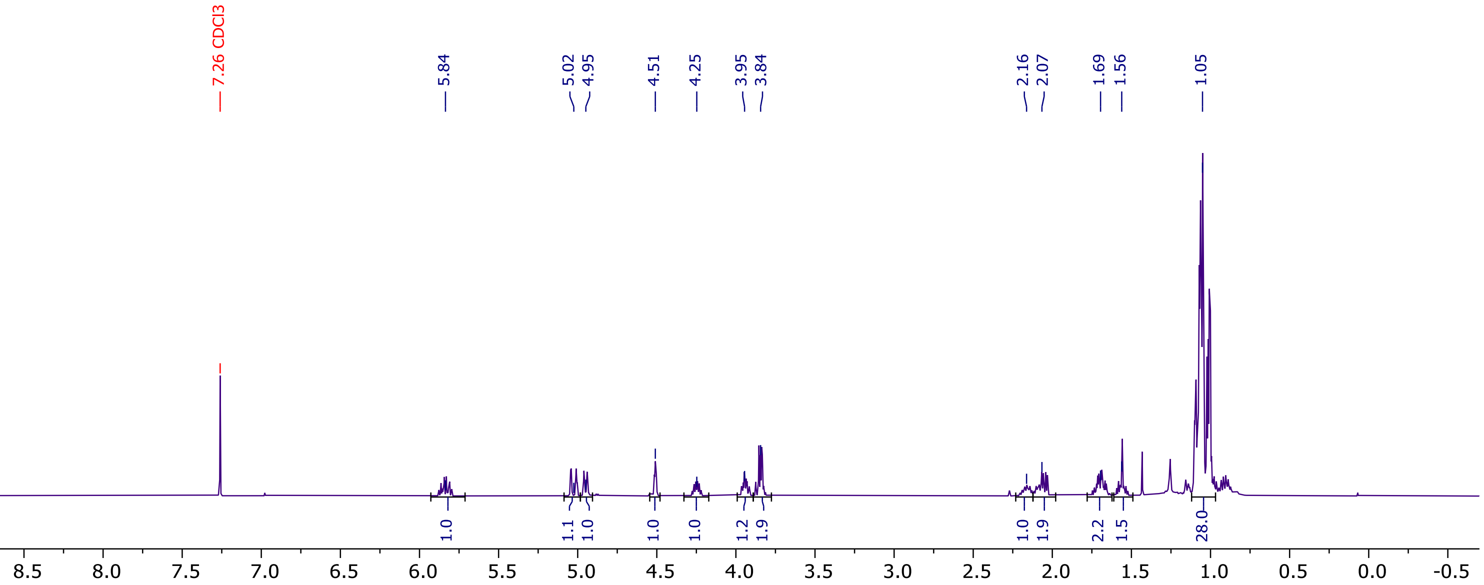
1H
CDCl3
500.13 MHz
7.26 CDCl3
5.84
5.02
4.95
4.51
4.25
3.95
3.84
2.16
2.07
1.69
1.56
1.05
1.0
1.1
1.0
1.0
1.0
1.2
1.9
1.0
1.9
2.2
1.5
28.0
10.0 9.5 9.0 8.5 8.0 7.5 7.0 6.5 6.0 5.5 5.0 4.5 4.0 3.5 3.0 2.5 2.0 1.5 1.0 0.5 0.0 -0.5

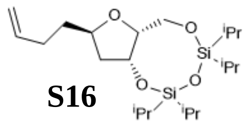
O
Si
O
O
Si
O
iPr
iPr
iPr
iPr

**S16**

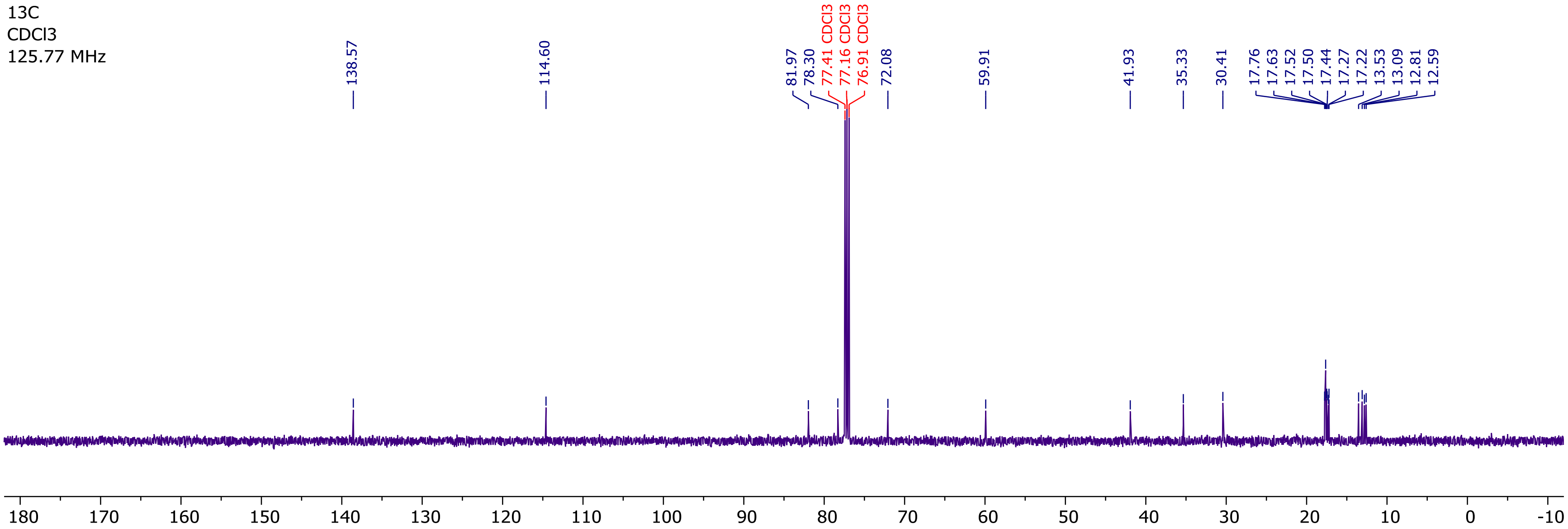
13C
CDCl3
125.77 MHz
138.57
114.60
81.97
78.30
77.41 CDCl3
77.16 CDCl3
76.91 CDCl3
72.08
59.91
41.93
35.33
30.41
17.76
17.63
17.52
17.50
17.44
17.27
17.22
13.53
13.09
12.81
12.59
180 170 160 150 140 130 120 110 100 90 80 70 60 50 40 30 20 10 0 -10

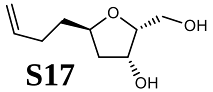


**S17**

To a cold (0 °C), stirred solution of **S16** (1.510 g, 3.64 mmol, 1.0 eq.) in anhydrous THF (13 mL), 1 M TBAF in THF (11 mL) added via syringe. Consumption of starting material was complete after 2 h as determined by TLC. The crude product was concentrated in vacuo and purified via flash column chromatography (1:1 to 3:7 hexanes/EtOAc). Appropriate fractions were pooled, and solvent was removed in vacuo to yield **S17** (325 mg, 1.89 mmol, 52% yield) as a colorless oil.

**Analytical Data for S17:**

$R_f$ = 0.12 (1:1 hexanes/EtOAc)

$[\alpha]_D^{20}$ = -23.2 ° (c = 1.98, MeOH)

$^1$H NMR (400 MHz, $CDCl_3$) δ 5.81 (ddt, *J* = 16.9, 10.1, 6.6 Hz, 1H), 5.02 (dq, *J* = 17.1, 1.7 Hz, 1H), 4.95 (dq, *J* = 10.2, 1.5 Hz, 1H), 4.50 (t, *J* = 4.0 Hz, 1H), 4.27 (dq, *J* = 9.4, 6.1 Hz, 1H), 3.97 – 3.88 (m, 3H), 3.60 (s, 1H), 3.04 (s, 1H), 2.22 – 2.05 (m, 3H), 1.71 (dtd, *J* = 13.3, 6.4, 5.7, 3.2 Hz, 2H), 1.55 (ddt, *J* = 13.3, 9.4, 6.1 Hz, 1H).

$^{13}$C NMR (101 MHz, $CDCl_3$) δ 138.23, 114.86, 80.53, 77.99, 74.40, 61.96, 42.14, 35.19, 30.31.

HRMS (ESI): Anal. Calcd. for $C_9H_{17}O_3^+$ $[M+H]^+$ 173.1172, found 173.1175

IR (neat): $\nu_{max}$ $(cm^{-1})$ = 3389 (br, OH), 2930 (m, CH), 1641 (m, C=C), 1442 (m), 1415 (m), 1330 (m), 1279 (m)

1H
CDCl3
400.13 MHz

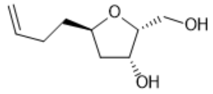


**S17**

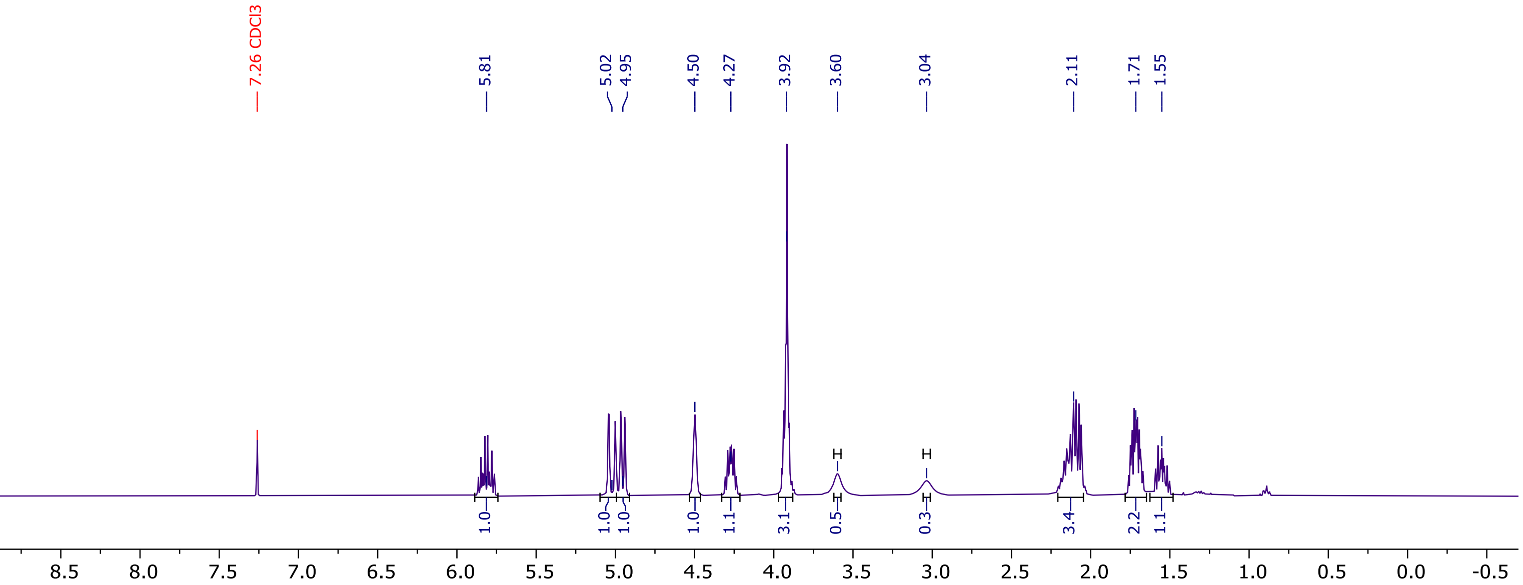


13C
CDCl3
100.62 MHz

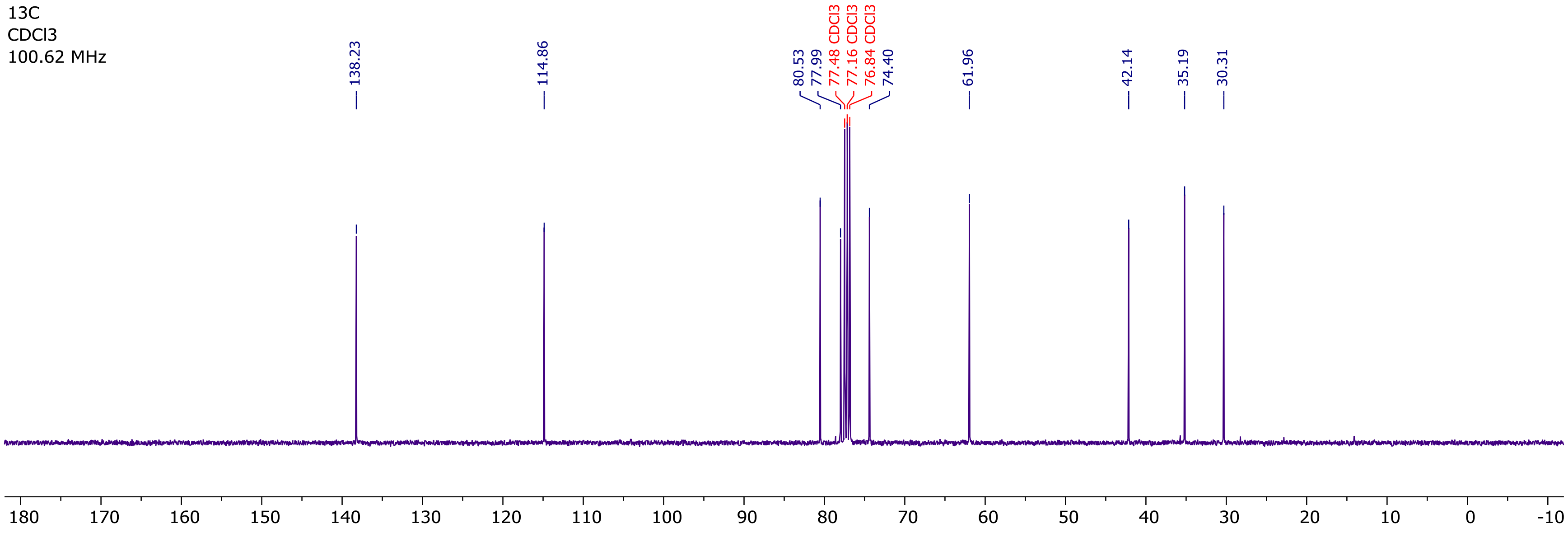

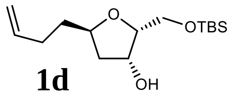

**1d**

To a cold (0 °C), stirred solution of **S17** (290.2 mg, 1.685 mmol, 1.0 eq.) and imidazole (229.4 mg, 3.37 mmol, 2.0 eq.) in anhydrous $CH_2Cl_2$ (5 mL) was added TBSCl (317.7 mg, 2.108 mmol, 1.25 eq.) in anhydrous $CH_2Cl_2$ (17.4 mL) via syringe. The reaction mixture warmed to rt and after stirring for 2 h the reaction mixture was poured into a separatory funnel with $H_2O$ (10 mL). The aqueous layer was extracted with $CH_2Cl_2$ (3x 10 mL) and the combined organic layers were washed with brine (1x 10 mL). After back extraction of the brine layer with $CH_2Cl_2$ (1x 10 mL), the combined organic layers were dried ($Na_2SO_4$), filtered, and the solvent was removed in vacuo. The crude product was purified via flash column chromatography (19:1 to 4:1 hexanes/EtOAc). Appropriate fractions were pooled, and solvent was removed in vacuo to yield **1d** (393.1 mg, 82%) as a colorless oil.

**Analytical Data for 1d:**

$R_f$ = 0.33 (4:1 hexanes/EtOAc)

$[\alpha]_D^{20}$ = -9.9 ° (c = 0.90, MeOH)

$^1$H NMR (400 MHz, $CDCl_3$) δ 5.83 (ddt, *J* = 16.9, 10.1, 6.6 Hz, 1H), 5.03 (dq, *J* = 17.1, 1.7 Hz, 1H), 4.95 (dq, *J* = 10.2, 1.4 Hz, 1H), 4.52 (q, *J* = 4.3, 3.8 Hz, 1H), 4.28 – 4.17 (m, 1H), 4.01 – 3.88 (m, 3H), 3.54 (d, *J* = 4.0 Hz, 1H), 2.24 – 2.01 (m, 3H), 1.68 (tdd, *J* = 12.1, 9.9, 5.8 Hz, 2H), 1.55 (ddt, *J* = 13.2, 9.5, 6.0 Hz, 1H), 0.90 (s, 9H), 0.10 (s, 3H), 0.09 (s, 3H).

$^{13}$C NMR (101 MHz, $CDCl_3$) δ 138.45, 114.74, 80.43, 78.08, 74.39, 63.03, 41.95, 35.19, 30.52, 25.93, 18.27, -5.31, -5.38.

HRMS (ESI): Anal. Calcd. for $C_{15}H_{31}O_3Si^+$ $[M+H]^+$ 287.2037, found 287.2039

IR (neat): $\nu_{max}$ $(cm^{-1})$ = 3455 (br, OH), 3078 (w, C=*CH*), 2930 (s, CH), 1641 (m, C=C), 1469 (m, CH), 1391 (w), 1362 (w), 1329 (w), 1254 (s)

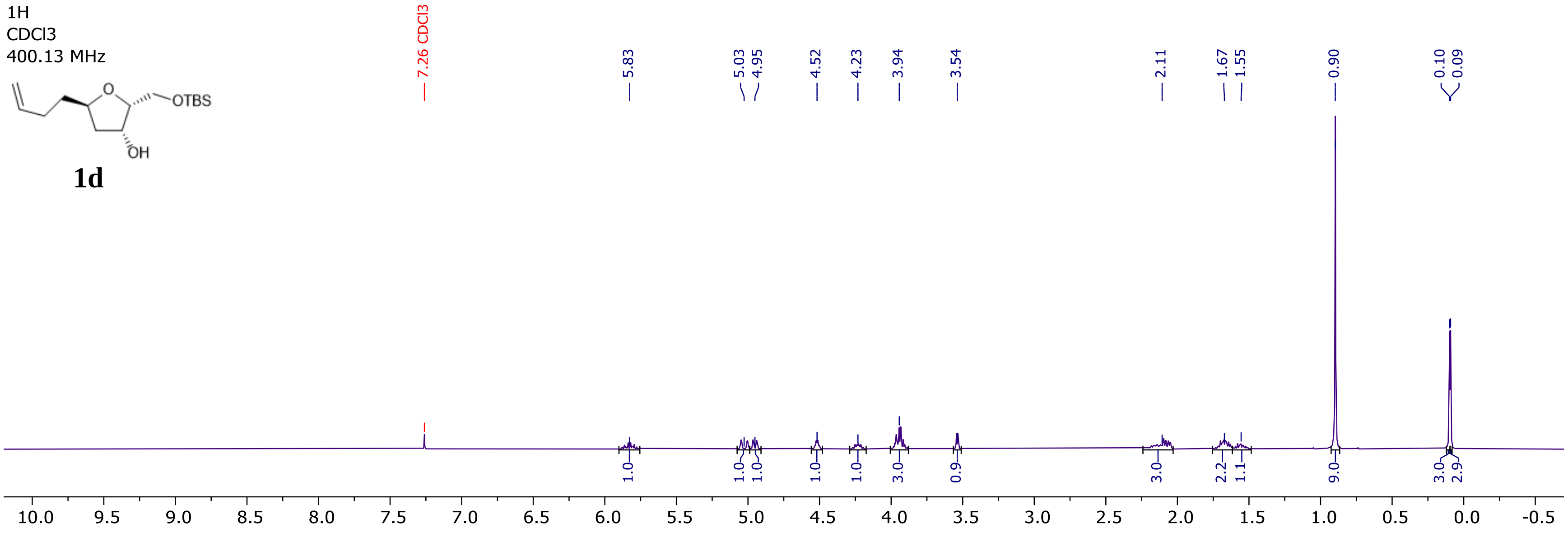
1H
CDCl3
400.13 MHz
7.26 CDCl3
5.83
5.03
4.95
4.52
4.23
3.94
3.54
2.11
1.67
1.55
0.90
0.10
0.09
1.0
1.0
1.0
1.0
1.0
3.0
0.9
3.0
2.2
1.1
9.0
3.0
2.9
10.0
9.5
9.0
8.5
8.0
7.5
7.0
6.5
6.0
5.5
5.0
4.5
4.0
3.5
3.0
2.5
2.0
1.5
1.0
0.5
0.0
-0.5
O
OTBS
OH

**1d**

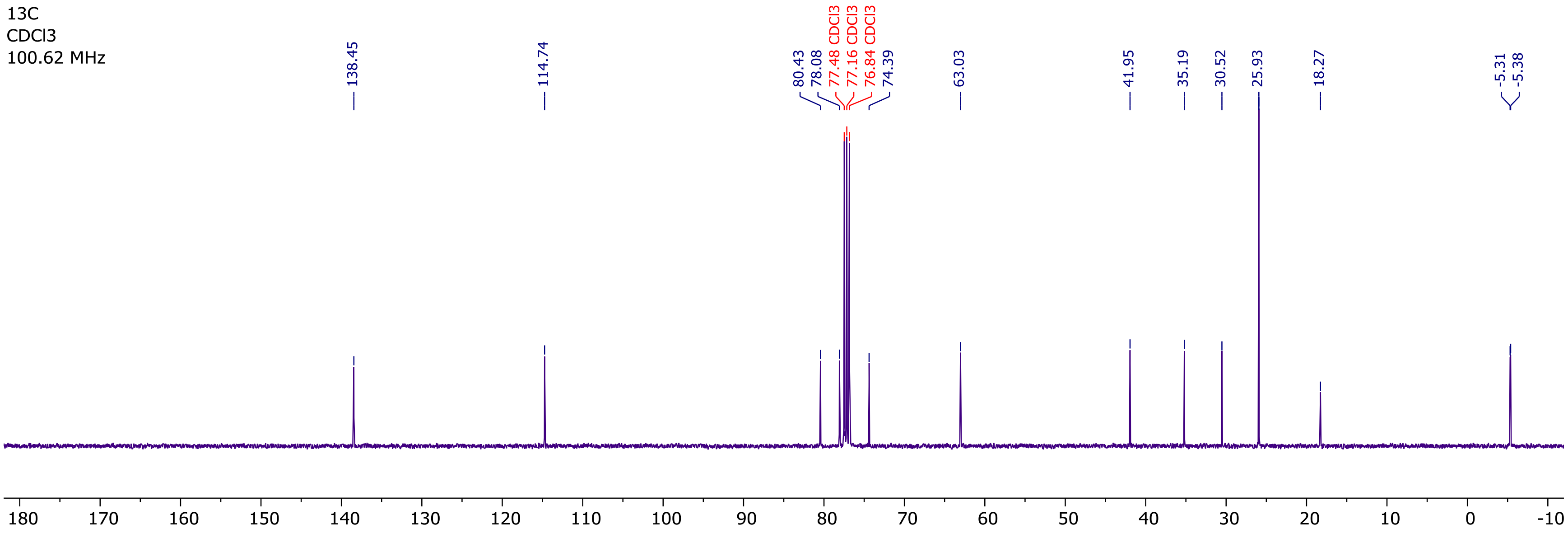
13C
CDCl3
100.62 MHz
138.45
114.74
80.43
78.08
77.48 CDCl3
77.16 CDCl3
76.84 CDCl3
74.39
63.03
41.95
35.19
30.52
25.93
18.27
-5.31
-5.38
180
170
160
150
140
130
120
110
100
90
80
70
60
50
40
30
20
10
0
-10

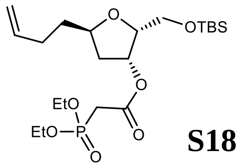

**S18**

Note: Reaction performed in capped vessel under ambient atmosphere.

To a stirred solution of **1d** (171 mg, 0.60 mmol, 1.0 eq.) and DIC (0.20 mL, 940 mg, 1.29 mmol, 2.1 eq.) in anhydrous $CH_2Cl_2$ (2 mL) was added diethylphosphonoacetic acid (199 mg, 1.02 mmol, 1.7 eq.) in anhydrous $CH_2Cl_2$ (1.5 mL). After stirring 3 min at rt an exothermic reaction takes place and the urea byproduct rapidly crashes out of solution. After stirring for 30 minutes, the reaction mixture was diluted with $CH_2Cl_2$ (15 mL) and washed with $H_2O$ (3x 10 mL) The combined aqueous layers were back extracted with $CH_2Cl_2$ (3x 10 mL). The combined organic layers were then dried ($Na_2SO_4$), filtered, and solvent was removed in vacuo. The crude product was purified via flash column chromatography (85:15 $CH_2Cl_2$/MeCN). Appropriate fractions were pooled, and solvent was removed in vacuo to yield **S18** (272 mg, 98%) as a colorless oil.

**Analytical Data for S18:**

$R_f$ = 0.66 (4:1 $CH_2Cl_2$/MeCN)

$[\alpha]_D^{20}$ = -14.8 ° (c = 1.61, MeOH)

$^1$H NMR (400 MHz, $CDCl_3$) δ 5.81 (ddt, *J* = 16.9, 10.2, 6.6 Hz, 1H), 5.43 (t, *J* = 4.2 Hz, 1H), 5.02 (dq, *J* = 17.1, 1.7 Hz, 1H), 4.95 (dq, *J* = 10.2, 1.4 Hz, 1H), 4.16 (dqd, *J* = 8.4, 7.1, 1.3 Hz, 5H), 4.06 (td, *J* = 6.3, 3.8 Hz, 1H), 3.83 – 3.68 (m, 2H), 2.95 (dq, *J* = 21.5, 14.4 Hz, 2H), 2.24 – 2.01 (m, 3H), 1.81 (ddd, *J* = 14.1, 9.5, 5.0 Hz, 1H), 1.75 – 1.61 (m, 1H), 1.55 (ddt, *J* = 13.5, 9.5, 5.9 Hz, 1H), 1.34 (td, *J* = 7.1, 1.7 Hz, 6H), 0.86 (s, 9H), 0.05 (s, 3H), 0.03 (s, 3H).

$^{13}$C NMR (101 MHz, $CDCl_3$) δ 165.23, 165.17, 138.24, 114.88, 80.80, 77.52, 76.04, 62.85, 62.78, 62.71, 61.46, 39.21, 35.24, 33.90, 30.33, 25.97, 18.37, 16.52, 16.46, -5.14, -5.27.

HRMS (ESI): Anal. Calcd. for $C_{21}H_{45}NO_7PSi^+$ $[M+NH_4]^+$ 482.2697, found 482.2717

IR (neat): $\nu_{max}$ $(cm^{-1})$ = 2931 (m, CH), 2857 (m, CH), 1740 (s, C=O), 1469 (w), 1444 (w), 1391 (w), 1258 (s)

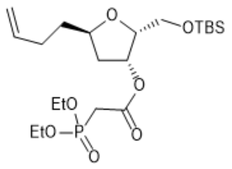
OTBS
EtO
EtO
P
O
O
O
O

**S18**

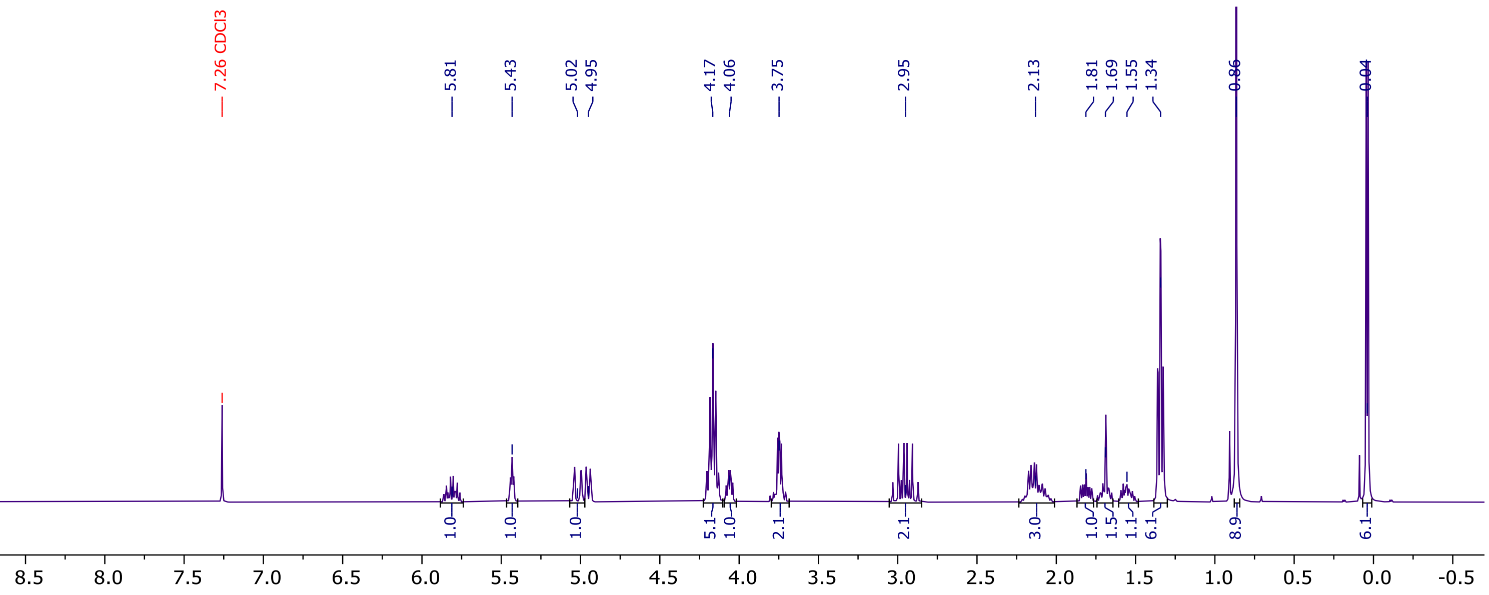
1H
CDCl3
400.13 MHz
7.26 CDCl3
5.81
5.43
5.02
4.95
4.17
4.06
3.75
2.95
2.13
1.81
1.69
1.55
1.34
0.86
0.04
1.0
1.0
1.0
5.1
1.0
2.1
2.1
3.0
1.0
1.5
1.1
6.1
8.9
6.1
10.0 9.5 9.0 8.5 8.0 7.5 7.0 6.5 6.0 5.5 5.0 4.5 4.0 3.5 3.0 2.5 2.0 1.5 1.0 0.5 0.0 -0.5

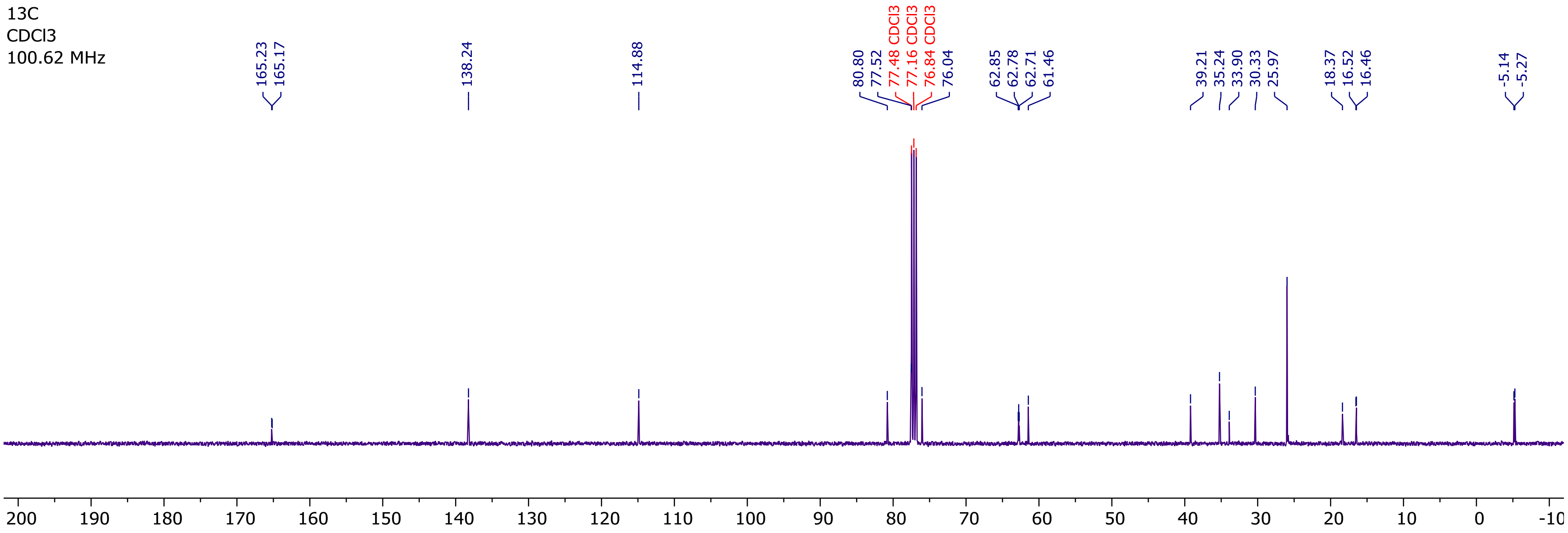
13C
CDCl3
100.62 MHz
165.23
165.17
138.24
114.88
80.80
77.52
77.48 CDCl3
77.16 CDCl3
76.84 CDCl3
76.04
62.85
62.78
62.71
61.46
39.21
35.24
33.90
30.33
25.97
18.37
16.52
16.46
-5.14
-5.27
200 190 180 170 160 150 140 130 120 110 100 90 80 70 60 50 40 30 20 10 0 -10

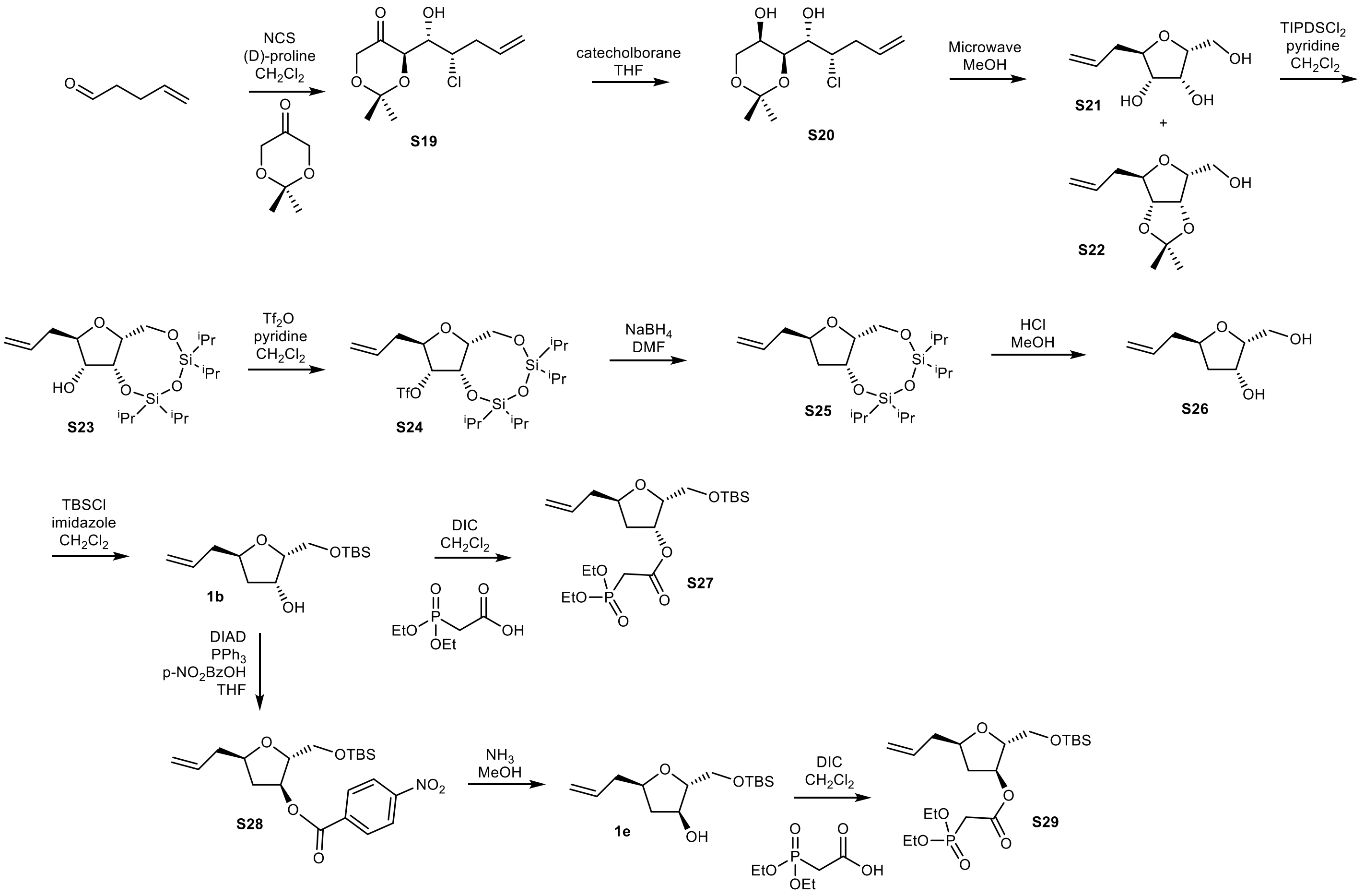


**Supplementary Fig. 29 | Synthesis of Tetrahydrofuranols 1b, 1e and Phosphonates S27, S29.**

NCS = N-chlorosuccinimide, THF = tetrahydrofuran, $TIPDSCl_2$ = 1,3-dichloro-1,1,3,3-tetraisopropyldisiloxane, $Tf_2O$ = trifluoromethanesulfonic anhydride, OTf = trifluoromethanesulfonate, DMF = dimethylformamide, TBSCl = tert-butyldimethylsilyl chloride, DIAD = diisopropyl azodicarboxylate, DIC = N,N'-diisopropylcarbodiimide.

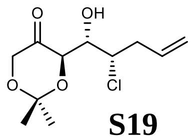

**S19**

Note: Reaction performed in capped vessel under ambient atmosphere.
To a cold (0 °C), stirred solution of pent-4-enal (10.82 g, 128.6 mmol, 1.0 eq.) in $CH_2Cl_2$ (640 mL) was added (*R*)-proline (14.78 g, 128.6 mmol, 1.0 eq.) and NCS (17.17 g, 128.6 mmol, 1.0 eq). The mixture was stirred at 0 °C for 30 minutes, followed by the addition of 2,2-dimethyl-1,3-dioxan-5-one (18 mL, 154.3 mmol, 1.2 eq). The mixture was washed with $H_2O$ (2x 300 mL) and the combined aqueous layers were extracted with $CH_2Cl_2$ (1x 250 mL). The combined organic layers were washed with brine (1x 300 mL) and the organic layers with dried ($Na_2SO_4$), filtered, and the solvent was removed in vacuo. The crude product was purified via flash column chromatography (9:1 hexanes/EtOAc). Appropriate fractions were pooled and the solvent was removed in vacuo to yield **S19** (14.1 g, 44%) as a yellow oil. Spectral data agreed with the data previously reported in the literature for **S1**.[7]

**Analytical Data for S19:**

$R_f$ = 0.18 (9:1 hexanes/EtOAc)

$[\alpha]_D^{20}$ = +124 ° (c = 1.31, MeOH)

$^1$H NMR (600 MHz, $CDCl_3$) δ 5.84 (ddt, *J* = 17.1, 10.1, 7.0 Hz, 1H), 5.19 (dq, *J* = 17.1, 1.6 Hz, 1H), 5.14 (dq, *J* = 10.2, 1.3 Hz, 1H), 4.40 (dd, *J* = 8.9, 1.5 Hz, 1H), 4.28 (dd, *J* = 17.6, 1.6 Hz, 1H), 4.22 (t, *J* = 7.3 Hz, 1H), 4.08 (d, *J* = 17.7 Hz, 1H), 3.97 (ddd, *J* = 8.8, 2.9, 1.6 Hz, 1H), 3.35 (d, *J* = 1.6 Hz, 1H), 2.75 – 2.67 (m, 1H), 2.68 – 2.60 (m, 1H), 1.51 (s, 3H), 1.42 (s, 3H).

$^{13}$C NMR (151 MHz, $CDCl_3$) δ 212.48, 134.13, 118.59, 101.77, 72.76, 70.89, 66.55, 60.99, 38.89, 23.94, 23.51.

HRMS (ESI): Anal. Calcd. for $C_{11}H_{18}ClO_4^+$ $[M+H]^+$ 249.0888, found 249.0882

IR (neat): $\nu_{max}$ $(cm^{-1})$ = 3511 (br, OH), 3079 (w, C=*CH*), 2990 (m, CH), 1740 (s, C=O), 1380 (s), 1223 (s)

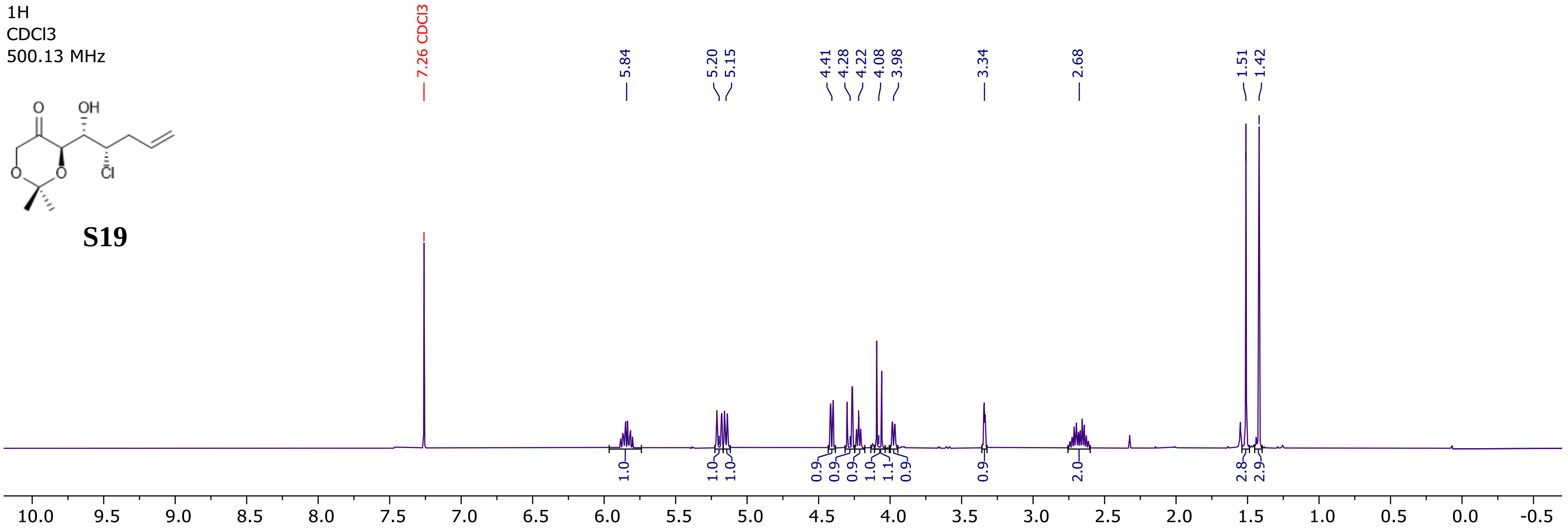
1H
CDCl3
500.13 MHz
S19
7.26 CDCl3
5.84
5.20
5.15
4.41
4.28
4.22
4.08
3.98
3.34
2.68
1.51
1.42
1.0
1.0
1.0
0.9
0.9
0.9
1.0
1.1
0.9
0.9
2.0
2.8
2.9
10.0 9.5 9.0 8.5 8.0 7.5 7.0 6.5 6.0 5.5 5.0 4.5 4.0 3.5 3.0 2.5 2.0 1.5 1.0 0.5 0.0 -0.5

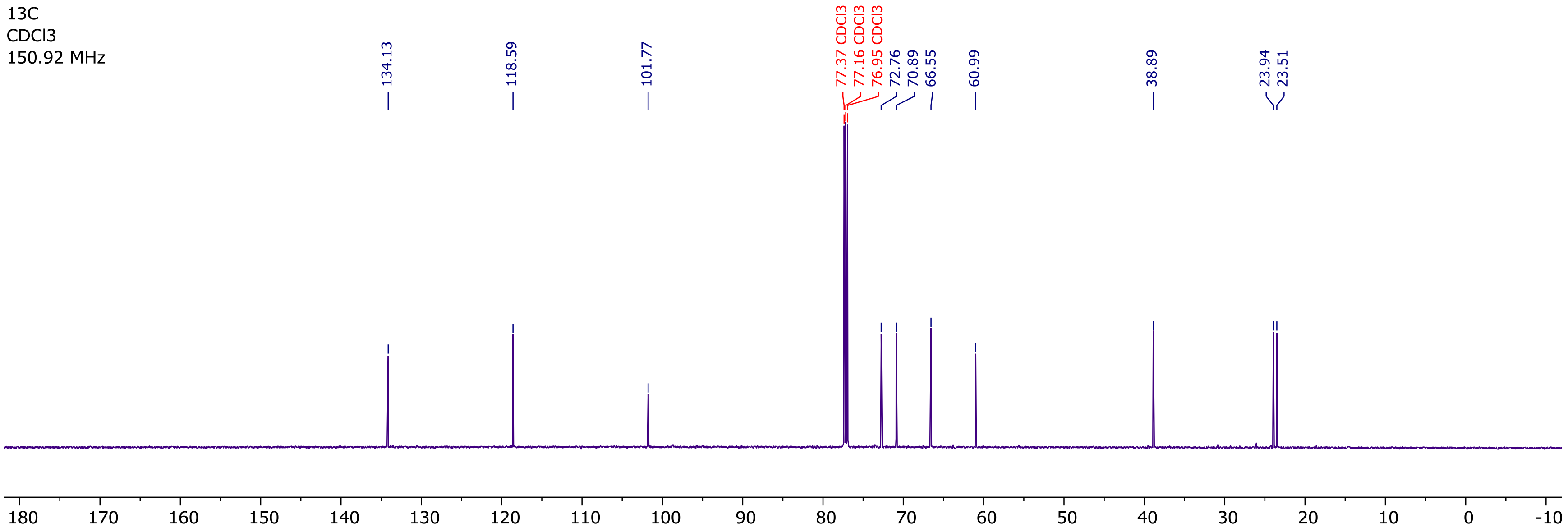
13C
CDCl3
150.92 MHz
134.13
118.59
101.77
77.37 CDCl3
77.16 CDCl3
76.95 CDCl3
72.76
70.89
66.55
60.99
38.89
23.94
23.51
180 170 160 150 140 130 120 110 100 90 80 70 60 50 40 30 20 10 0 -10

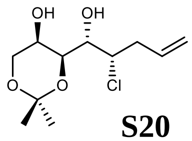


**S20**

A round-bottom flask containing **S19** (11.4 g, 46.0 mmol, 1.0 eq.) was purged with $N_2$(g). Anhydrous THF (355 mL) was added via syringe. The solution was cooled to 0 °C and 0.1 M catecholborane in THF (100 mL, 100 mmol, 2.2 eq.) was added via syringe. After 90 minutes stirring at 0 °C, the solution was allowed to warm to room temperature and stirred for an additional 1 h. The mixture was diluted with MeOH (250 mL) and a saturated solution of potassium sodium tartrate (200 mL) was added and stirred vigorously for 2 h. Brine (100 mL) and EtOAc (650 mL) were added and the aqueous layer was extracted with EtOAc (2x 500 mL). The combined organic layers were washed with brine (1x 500 mL), dried ($Na_2SO_4$), filtered, and the solvent was removed in vacuo. The crude product was purified via flash column chromatography (1:1 to 2:3 to 3:7 hexanes/EtOAc). Appropriate fractions were pooled, and solvent was removed in vacuo to yield **S20** (6.47 g, 56%) as a yellow oil.

**Analytical Data for S20:**

$R_f$ = 0.19 (1:1 hexanes/EtOAc)

$[\alpha]_D^{20}$ = -8.8 ° (c = 1.17, MeOH)

$^1$H NMR (600 MHz, $CDCl_3$) δ 5.86 (ddt, *J* = 17.1, 10.2, 7.0 Hz, 1H), 5.18 (dq, *J* = 17.1, 1.5 Hz, 1H), 5.14 (ddt, *J* = 10.2, 2.0, 1.1 Hz, 1H), 4.28 (ddd, *J* = 8.5, 6.2, 0.9 Hz, 1H), 4.08 (dd, *J* = 12.3, 1.6 Hz, 1H), 3.94 – 3.85 (m, 3H), 3.72 (d, *J* = 11.1 Hz, 1H), 2.78 (d, *J* = 11.0 Hz, 1H), 2.68 (dddt, *J* = 15.2, 8.2, 6.7, 1.3 Hz, 1H), 2.60 (dddt, *J* = 14.6, 7.3, 6.1, 1.2 Hz, 1H), 2.36 (dd, *J* = 6.6, 2.1 Hz, 1H), 1.45 (s, 3H), 1.40 (s, 3H).

$^{13}$C NMR (151 MHz, $CDCl_3$) δ 134.27, 118.42, 99.39, 72.21, 70.38, 66.00, 62.84, 62.77, 39.66, 29.53, 18.44.

HRMS (ESI): Anal. Calcd. for $C_{11}H_{20}ClO_4^+$ $[M+H]^+$ 251.1045, found 251.1047

IR (neat): $\nu_{max}$ $(cm^{-1})$ = 3404 (br, OH), 2992 (w, CH), 2942 (w, CH), 1642 (w, C=C), 1382 (m, OH), 1270 (s)

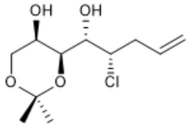
OH
OH
O
O
Cl

**S20**

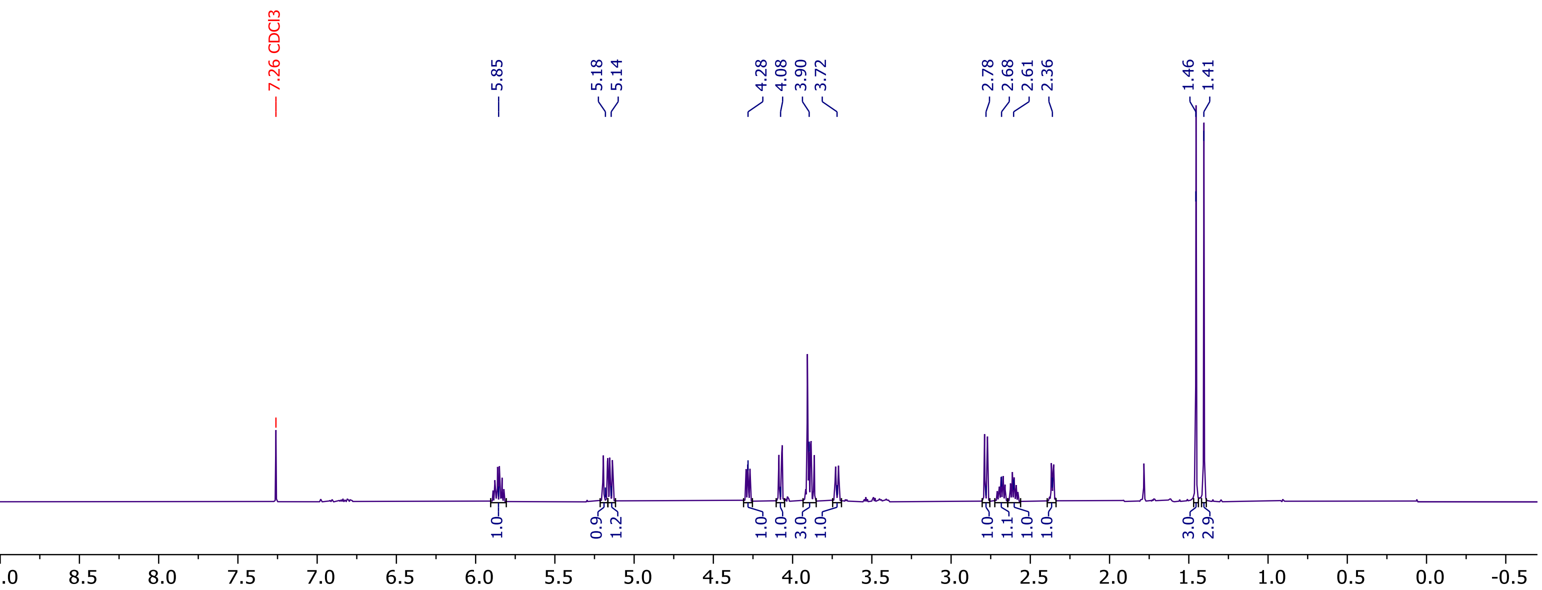
1H
CDCl3
600.13 MHz
7.26 CDCl3
5.85
5.18
5.14
4.28
4.08
3.90
3.72
2.78
2.68
2.61
2.36
1.46
1.41
1.0
0.9
1.2
1.0
1.0
3.0
1.0
1.0
1.1
1.0
1.0
3.0
2.9
10.0 9.5 9.0 8.5 8.0 7.5 7.0 6.5 6.0 5.5 5.0 4.5 4.0 3.5 3.0 2.5 2.0 1.5 1.0 0.5 0.0 -0.5

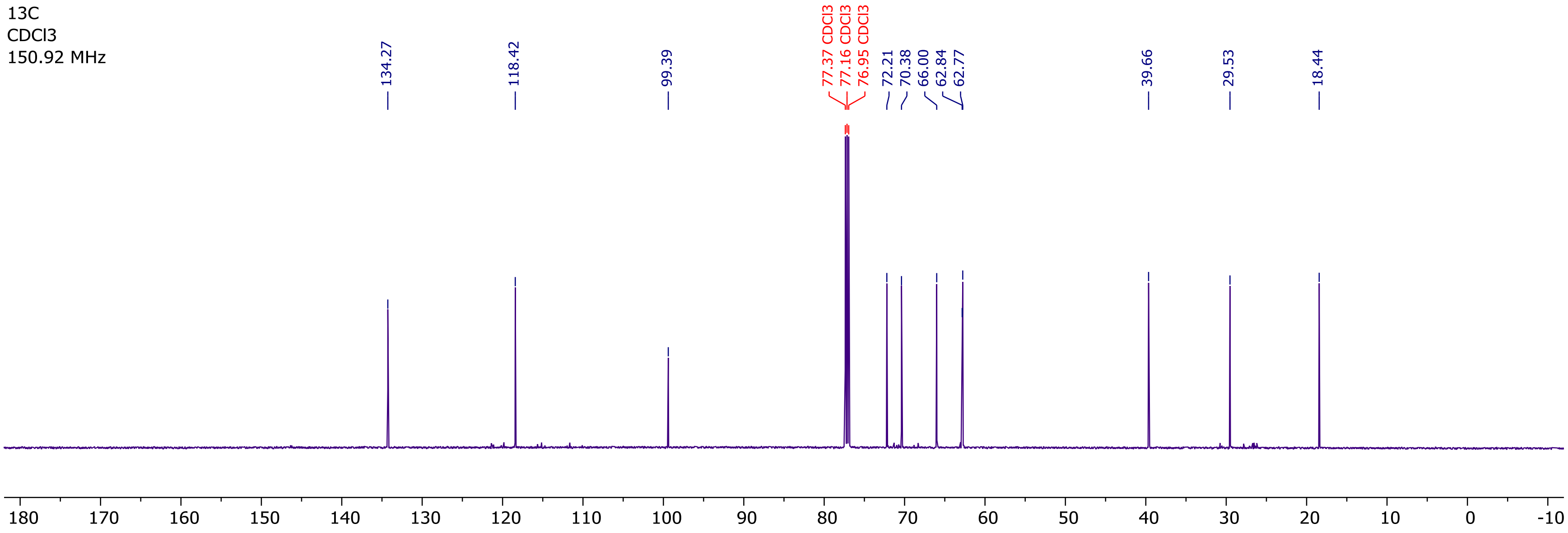
13C
CDCl3
150.92 MHz
134.27
118.42
99.39
77.37 CDCl3
77.16 CDCl3
76.95 CDCl3
72.21
70.38
66.00
62.84
62.77
39.66
29.53
18.44
180 170 160 150 140 130 120 110 100 90 80 70 60 50 40 30 20 10 0 -10

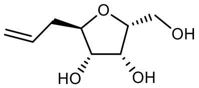


**S21**

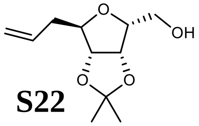


**S22**

**S20** (1.29 g, 5.14 mmol) was dissolved in MeOH (45 mL) and heated to 120 °C in a microwave reactor for 90 min (300 psi maximum pressure). The resultant dark brown solution was transferred into a 500 mL round bottomed flask. This process was repeated 4 more times (for a total of 5 batches). The combined reaction mixtures were concentrated in vacuo and the crude product was purified via flash column chromatography (25:1 to 25:2 $CH_2Cl_2$/MeOH). Appropriate fractions were pooled, and the solvent was removed in vacuo to yield **S21** (2.268 g, 51%) as a brown oil. The remaining fractions were re-purified via flash column chromatography (3:1 hexanes/EtOAc). Appropriate fractions were pooled, and the solvent was removed in vacuo to yield **S22** (1.00 g, 18%) as an orange oil. Spectral and optical rotation data agreed with the data previously reported in the literature for **S21**.[10]

**Analytical Data for S21:**

$R_f$ = 0.17 (19:1 $CH_2Cl_2$/MeOH)

$[\alpha]_D^{20}$ = +36.0 ° (c = 1.89, $CHCl_3$); lit. $[\alpha]_D^{24}$ = +32.2 ° (c = 2.5, $CHCl_3$)[10]

$^1$H NMR (600 MHz, MeOD) δ 5.92 (ddt, *J* = 17.2, 10.3, 6.9 Hz, 1H), 5.15 (dq, *J* = 17.2, 1.7 Hz, 1H), 5.09 (ddt, *J* = 10.2, 2.3, 1.1 Hz, 1H), 4.16 (t, *J* = 4.0 Hz, 1H), 4.03 (dt, *J* = 6.1, 4.5 Hz, 1H), 3.90 – 3.85 (m, 2H), 3.79 (dd, *J* = 11.5, 4.9 Hz, 1H), 3.72 (dd, *J* = 11.5, 6.2 Hz, 1H), 2.49 – 2.41 (m, 1H), 2.34 – 2.25 (m, 1H).

$^{13}$C NMR (151 MHz, MeOD) δ 135.80, 117.50, 81.92, 81.82, 76.85, 73.13, 62.15, 38.75.

$^1$H NMR (500 MHz, DMSO) δ 5.82 (ddt, *J* = 17.1, 10.2, 6.9 Hz, 1H), 5.07 (dq, *J* = 17.3, 1.7 Hz, 1H), 5.01 (ddt, *J* = 10.2, 2.4, 1.2 Hz, 1H), 4.64 (s, 1H), 3.92 (t, *J* = 3.9 Hz, 1H), 3.83 (td, *J* = 5.8, 3.8 Hz, 1H), 3.71 – 3.62 (m, 2H), 3.56 (dd, *J* = 11.2, 5.4 Hz, 1H), 3.42 (dd, *J* = 11.2, 6.3 Hz, 1H), 2.37 – 2.27 (m, 1H), 2.14 (dt, *J* = 14.2, 7.0 Hz, 1H).

$^{13}$C NMR (126 MHz, DMSO) δ 135.45, 116.67, 80.70, 79.57, 75.53, 70.97, 60.19, 37.53.

HRMS (ESI): Anal. Calcd. for $C_8H_{15}O_4^+$ $[M+H]^+$ 175.0965, found 175.0970

IR (neat): $\nu_{max}$ $(cm^{-1})$ = 3369 (br, OH), 2926 (m, CH), 2500 (br, OH), 1642 (w, C=C), 1432 (w), 1337 (w), 1209 (w)

1H
MeOD
600.13 MHz

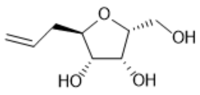


**S21**

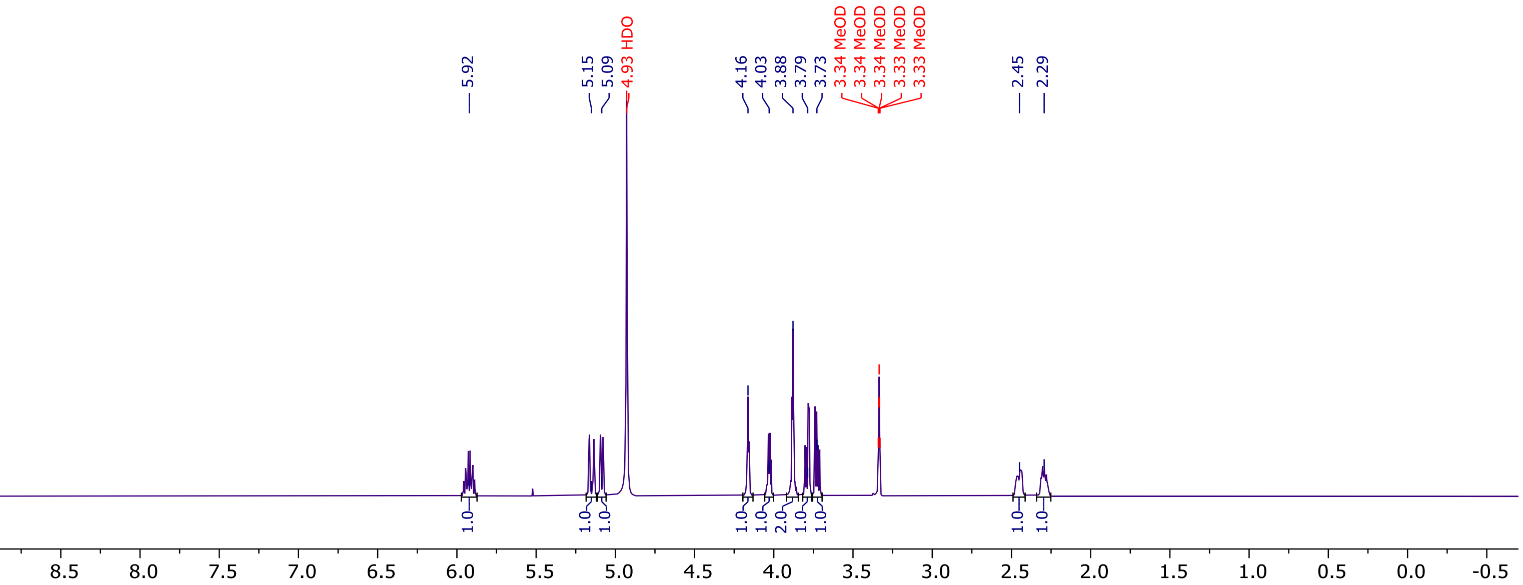


13C
MeOD
150.92 MHz

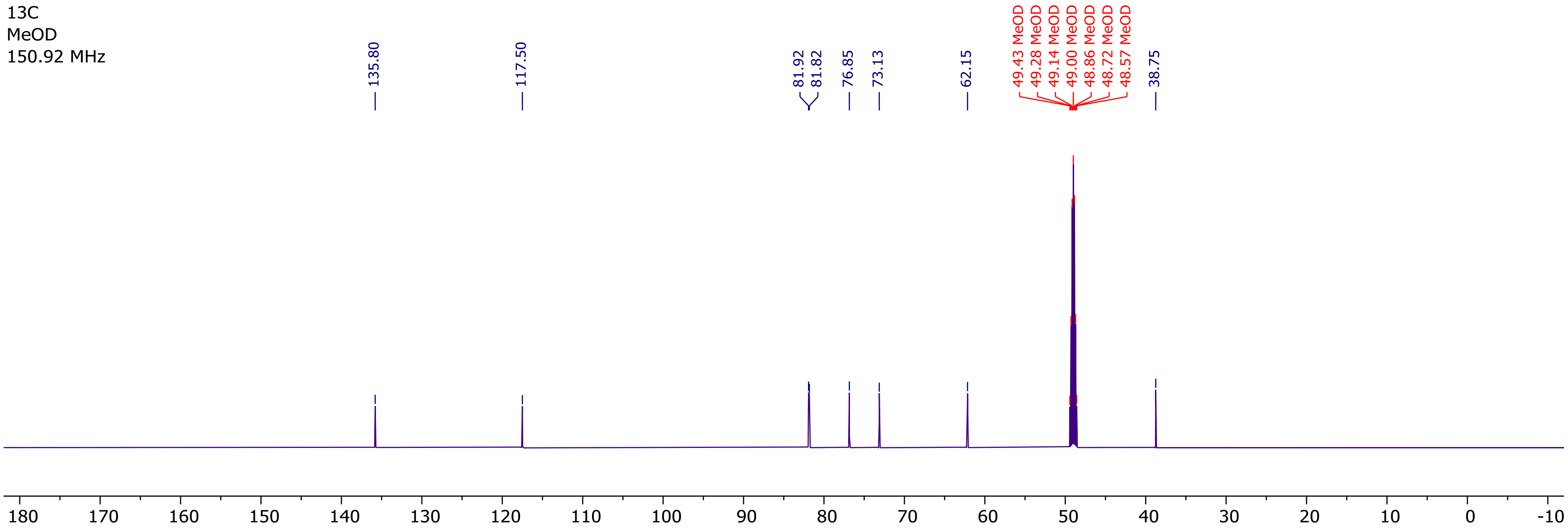

1H
DMSO
500.14 MHz

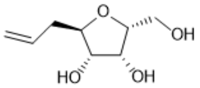


**S21**

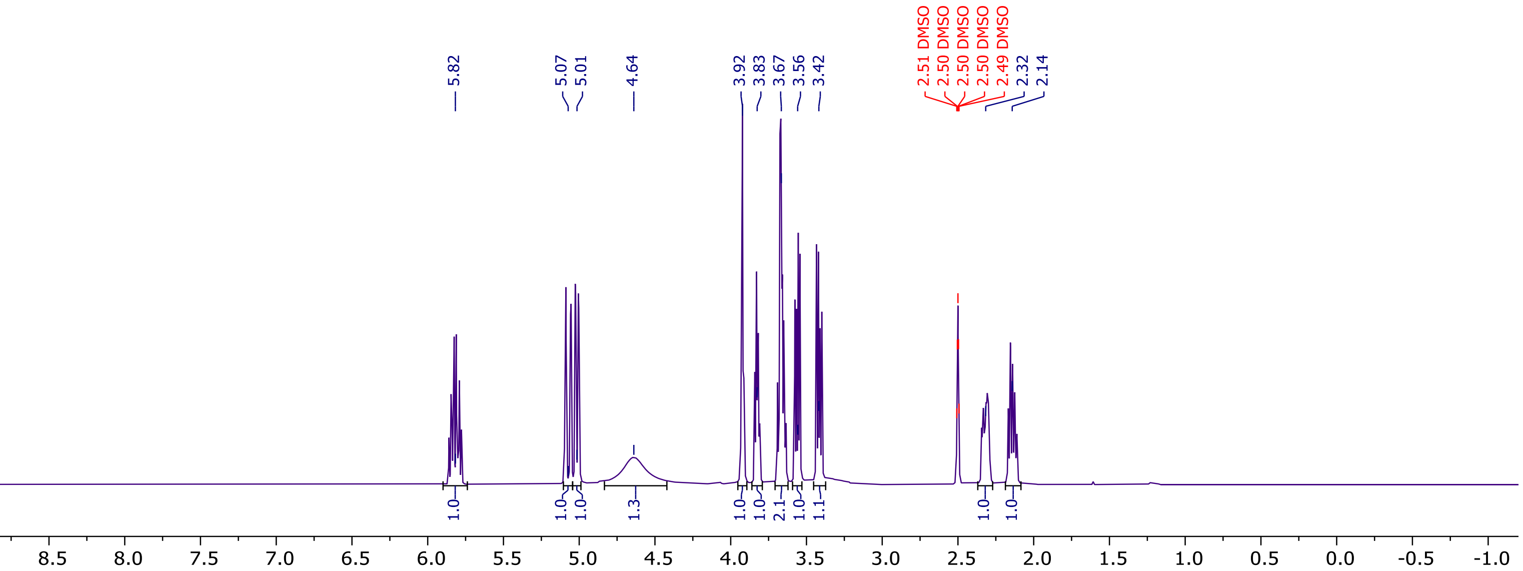


13C
DMSO
125.77 MHz

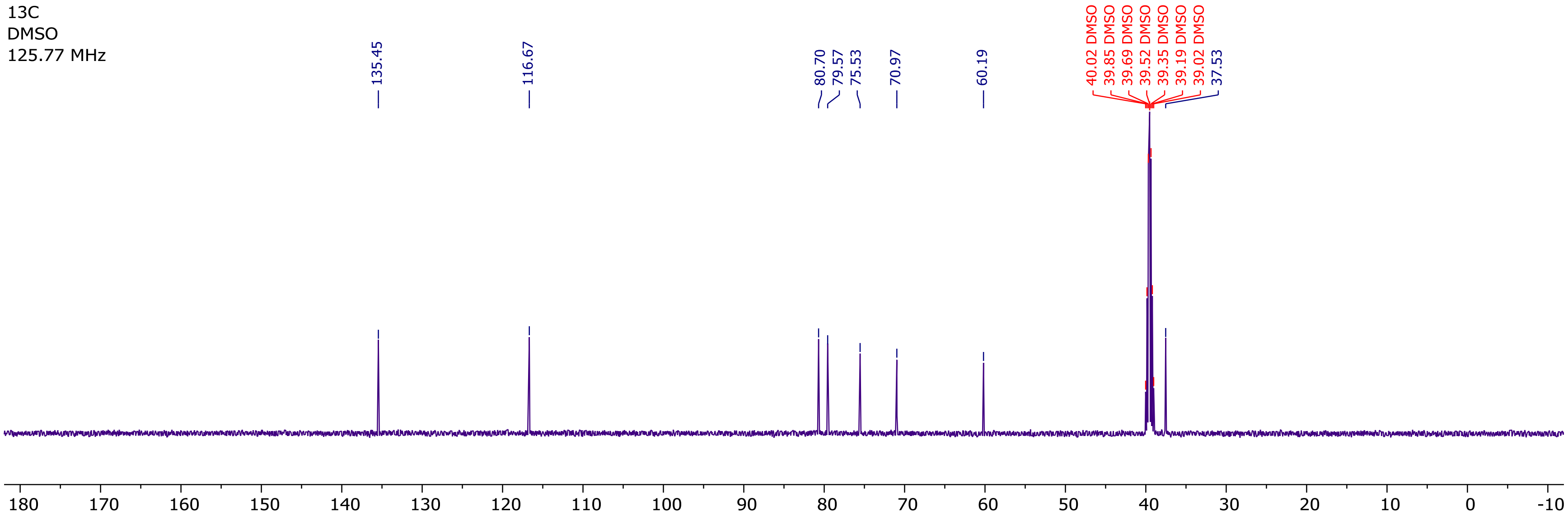

**Analytical Data for S22:**

$R_f$ = 0.16 (4:1 hexanes/$Et_2O$)

$[\alpha]_D^{20}$ = +9.1 ° (c = 1.29, MeOH)

$^1$H NMR (600 MHz, $CDCl_3$) δ 5.79 (ddt, *J* = 17.2, 10.5, 6.9 Hz, 1H), 5.13 (d, *J* = 6.1 Hz, 1H), 5.10 (s, 1H), 4.78 (dd, *J* = 6.2, 4.2 Hz, 1H), 4.56 (d, *J* = 6.1 Hz, 1H), 4.16 (t, *J* = 7.4 Hz, 1H), 3.98 (q, *J* = 5.2 Hz, 1H), 3.88 (qd, *J* = 11.7, 5.6 Hz, 2H), 2.35 – 2.24 (m, 2H), 2.21 – 2.14 (m, 1H), 1.49 (s, 3H), 1.31 (s, 3H).

$^{13}$C NMR (151 MHz, $CDCl_3$) δ 133.63, 118.09, 113.05, 84.75, 83.10, 81.50, 79.59, 61.56, 35.83, 26.24, 24.83.

HRMS (ESI): Anal. Calcd. for $C_{11}H_{19}O_4^+$ $[M+H]^+$ 215.1278, found 215.1287

IR (neat): $\nu_{max}$ ($cm^{-1}$) = 3437 (br, OH), 2940 (m, CH), 1642 (m, C=C), 1379 (s, OH)

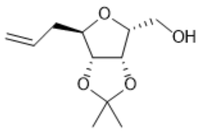
O
OH
O
O

**S22**

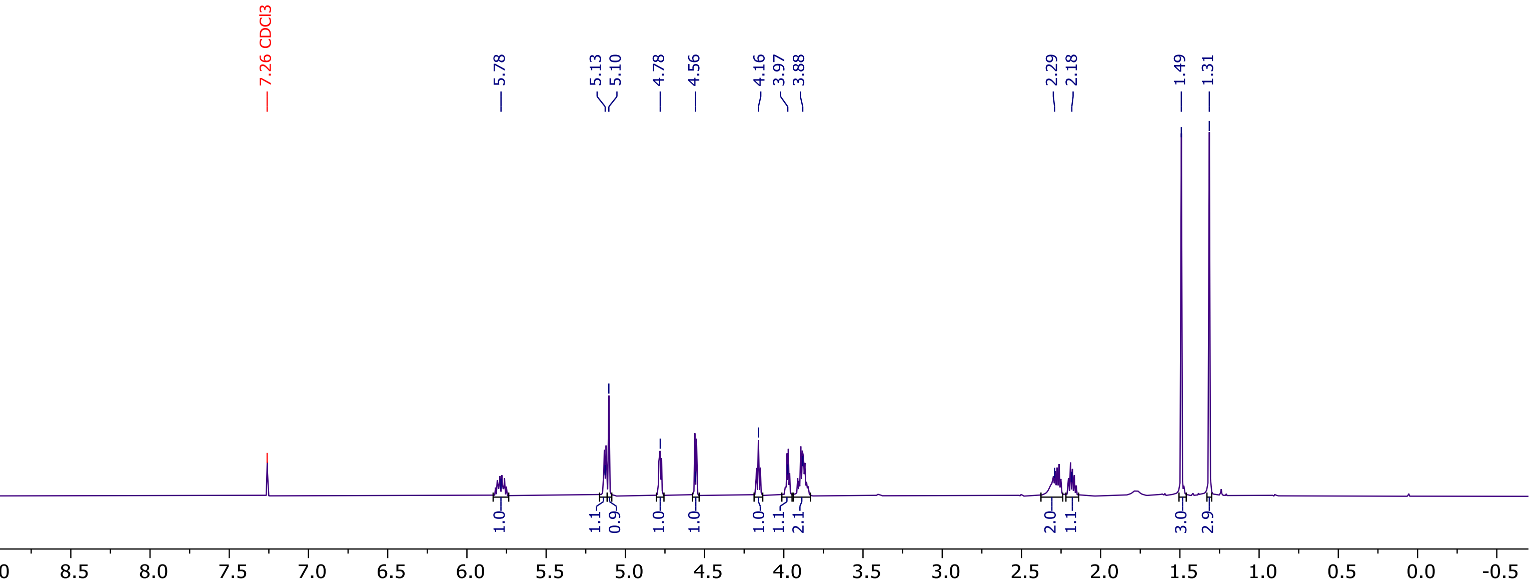
1H
CDCl3
600.13 MHz
7.26 CDCl3
5.78
5.13
5.10
4.78
4.56
4.16
3.97
3.88
2.29
2.18
1.49
1.31
1.0
1.1
0.9
1.0
1.0
1.0
1.1
2.1
2.0
1.1
3.0
2.9
10.0 9.5 9.0 8.5 8.0 7.5 7.0 6.5 6.0 5.5 5.0 4.5 4.0 3.5 3.0 2.5 2.0 1.5 1.0 0.5 0.0 -0.5

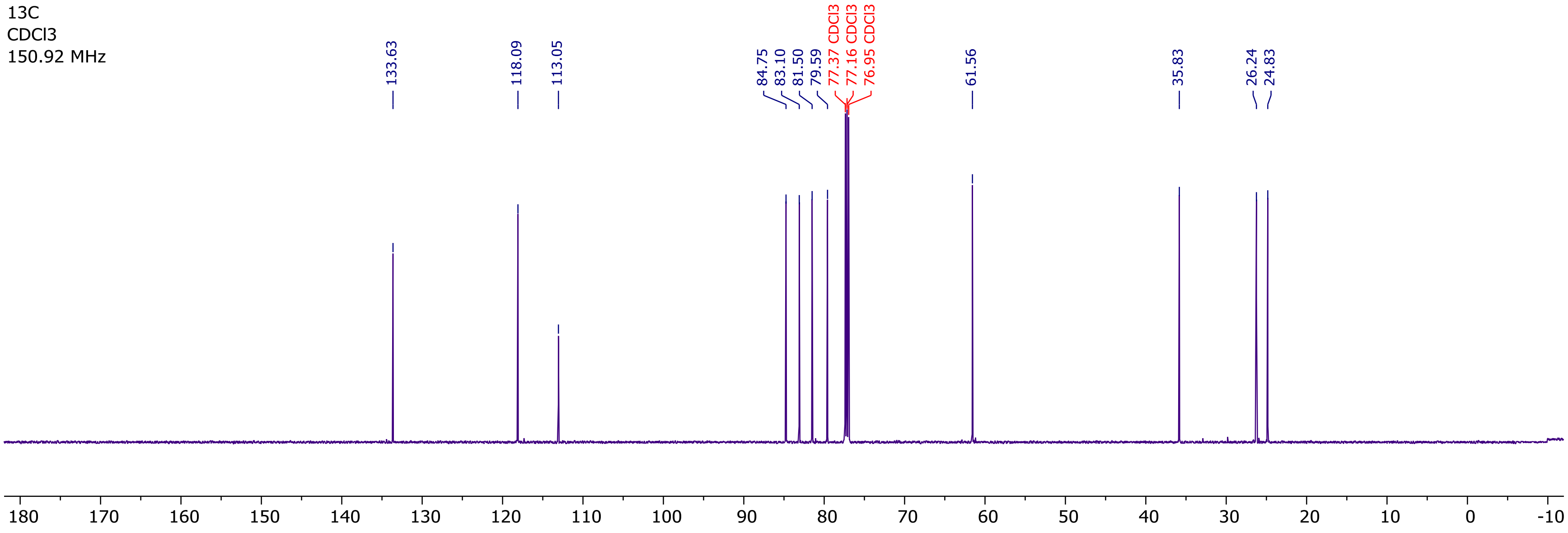
13C
CDCl3
150.92 MHz
133.63
118.09
113.05
84.75
83.10
81.50
79.59
77.37 CDCl3
77.16 CDCl3
76.95 CDCl3
61.56
35.83
26.24
24.83
180 170 160 150 140 130 120 110 100 90 80 70 60 50 40 30 20 10 0 -10

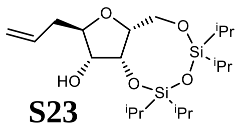

**S23**

To a cold (0 °C), stirred solution of **S21** (2.24 g, 12.9 mmol, 1.0 eq) in anhydrous $CH_2Cl_2$ (39 mL) was added in sequence anhydrous pyridine (12.8 g, 13.0 mL, 161 mmol, 12.5 eq.) and $TIPDSiCl_2$ (4.20 mL, 4.14 g, 13.1 mmol, 1.02 eq.) via syringe. The reaction mixture was then allowed to warm to rt and stirred for a further 16 h, after which time starting material was consumed as monitored by TLC analysis. The reaction mixture was diluted with $CH_2Cl_2$ (100 mL) and washed with an aqueous solution of 10% $CuSO_4$ (1x 200 mL). The aqueous layer was back extracted with $CH_2Cl_2$ (2x 100 mL). The combined organic layers were washed with brine (1x 200 mL), dried ($Na_2SO_4$), filtered, and the solvent was removed in vacuo. The crude product was purified via flash column chromatography (9:1 hexanes/$Et_2O$). Appropriate fractions were pooled, and the solvent was removed in vacuo to yield **S23** (3.91 g, 73%) as a colorless oil.

**Analytical Data for S23:**

$R_f$ = 0.26 (9:1 hexanes/$Et_2O$)

$[\alpha]_D^{20}$ = +5.3 ° (c = 1.33, MeOH)

$^1$H NMR (600 MHz, $CDCl_3$) δ 5.88 (ddt, *J* = 17.1, 10.3, 6.9 Hz, 1H), 5.16 (dt, *J* = 17.2, 1.7 Hz, 1H), 5.11 (dt, *J* = 10.4, 1.6 Hz, 1H), 4.36 (dd, *J* = 4.4, 2.9 Hz, 1H), 4.05 (ddd, *J* = 10.3, 4.7, 2.9 Hz, 1H), 3.93 (ddd, *J* = 11.9, 8.4, 4.4 Hz, 1H), 3.89 (t, *J* = 10.3 Hz, 1H), 3.79 (dd, *J* = 10.2, 4.7 Hz, 1H), 3.74 (ddd, *J* = 8.5, 6.8, 4.7 Hz, 1H), 2.48 (dddd, *J* = 12.5, 6.2, 3.9, 1.4 Hz, 1H), 2.31 (dt, *J* = 14.4, 7.2 Hz, 1H), 2.26 (d, *J* = 11.7 Hz, 1H), 1.16 – 0.98 (m, 28H).

$^{13}$C NMR (151 MHz, $CDCl_3$) δ 134.29, 117.65, 81.35, 79.95, 76.36, 72.43, 59.64, 37.74, 17.68, 17.63, 17.62, 17.49, 17.43, 17.37, 17.18, 17.13, 13.44, 13.01, 12.87, 12.57.

HRMS (ESI): Anal. Calcd. for $C_{20}H_{44}NO_5Si_2^+$ $[M+NH_4]^+$ 434.2753, found 434.2771

IR (neat): $\nu_{max}$ $(cm^{-1})$ = 2946 (m, CH), 2868 (m, CH), 1642 (w, C=C), 1466 (m), 1389 (w), 1249 (m)

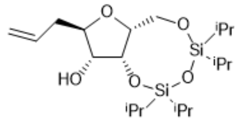
HO
O
O
O
Si
Si
O
iPr
iPr
iPr
iPr

**S23**

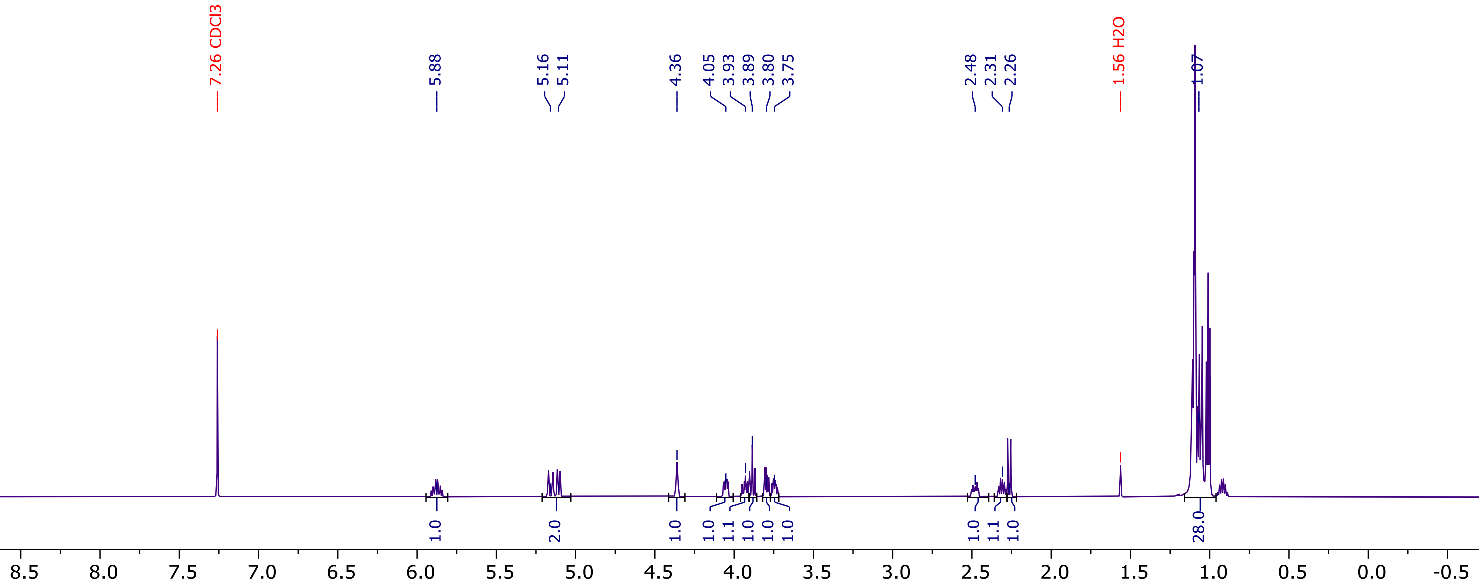
1H
CDCl3
600.13 MHz
7.26 CDCl3
5.88
5.16
5.11
4.36
4.05
3.93
3.89
3.80
3.75
2.48
2.31
2.26
1.56 H2O
1.07
1.0
2.0
1.0
1.0
1.1
1.0
1.0
1.0
1.0
1.1
1.0
28.0
10.0 9.5 9.0 8.5 8.0 7.5 7.0 6.5 6.0 5.5 5.0 4.5 4.0 3.5 3.0 2.5 2.0 1.5 1.0 0.5 0.0 -0.5

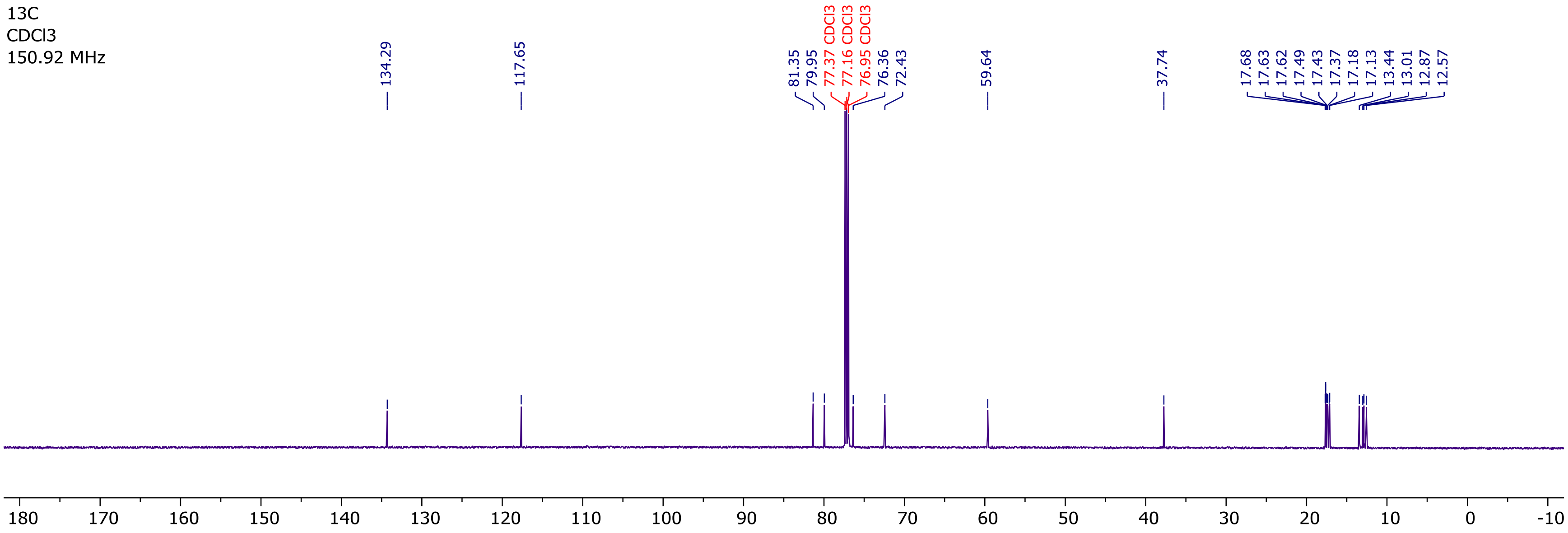
13C
CDCl3
150.92 MHz
134.29
117.65
81.35
79.95
77.37 CDCl3
77.16 CDCl3
76.95 CDCl3
76.36
72.43
59.64
37.74
17.68
17.63
17.62
17.49
17.43
17.37
17.18
17.13
13.44
13.01
12.87
12.57
180 170 160 150 140 130 120 110 100 90 80 70 60 50 40 30 20 10 0 -10

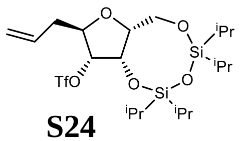

**S24**

To a cold (0°C), stirred solution of **S23** (3.89 g, 9.34 mmol, 1.0 eq.) in $CH_2Cl_2$ (60 mL) was added anhydrous pyridine (4.5 mL) followed by $Tf_2O$ (3.90 mL, 6.58 g, 23.3 mmol, 2.5 eq.) dropwise via syringe. The reaction mixture was then allowed to warm to rt and stirred for 3 h. The reaction mixture was then cooled (0 °C) and washed with ice-cold saturated $NaHCO_3$ (50 mL). The aqueous layer was extracted with $CH_2Cl_2$ (3x 50 mL) and the combined organic extracts were dried ($Na_2SO_4$), filtered and the solvent was removed in vacuo. The crude product was purified by flash column chromatography (97:3 hexanes/$Et_2O$). Appropriate fractions were pooled, and solvent was removed in vacuo to yield **S24** (4.81 g, 94%) as a colorless oil.

**Analytical Data for S24:**

$R_f$ = 0.27 (97:3 hexanes/$Et_2O$)

$[\alpha]_D^{20}$ = +35.6° (c = 3.23, MeOH)

$^1$H NMR (600 MHz, $CDCl_3$) δ 5.83 (dddd, *J* = 16.5, 10.3, 7.7, 6.1 Hz, 1H), 5.23 – 5.15 (m, 2H), 4.89 (dd, *J* = 8.8, 3.8 Hz, 1H), 4.63 (dd, *J* = 3.9, 2.6 Hz, 1H), 4.28 (ddd, *J* = 8.8, 6.4, 3.9 Hz, 1H), 4.05 (ddd, *J* = 10.2, 4.7, 2.6 Hz, 1H), 3.90 (t, *J* = 10.3 Hz, 1H), 3.80 (dd, *J* = 10.4, 4.7 Hz, 1H), 2.55 (dddd, *J* = 14.9, 5.7, 3.8, 1.8 Hz, 1H), 2.30 – 2.24 (m, 1H), 1.13 – 0.99 (m, 28H).

$^{13}$C NMR (151 MHz, $CDCl_3$) δ 132.33, 119.24, 86.55, 79.76, 76.66, 71.31, 59.11, 35.89, 17.72, 17.45, 17.41, 17.35, 17.33, 17.15, 17.12, 13.44, 13.05, 12.92, 12.68.

HRMS (ESI): Anal. Calcd. for $C_{21}H_{43}F_3NO_7SSi_2^+$ $[M+NH_4]^+$ 566.2245, found 566.2266

IR (neat): $\nu_{max}$ $(cm^{-1})$ = 2948 (m, CH), 2870 (m, CH), 1467 (m, CH), 1421 (s), 1248 (s), 1214 (s)

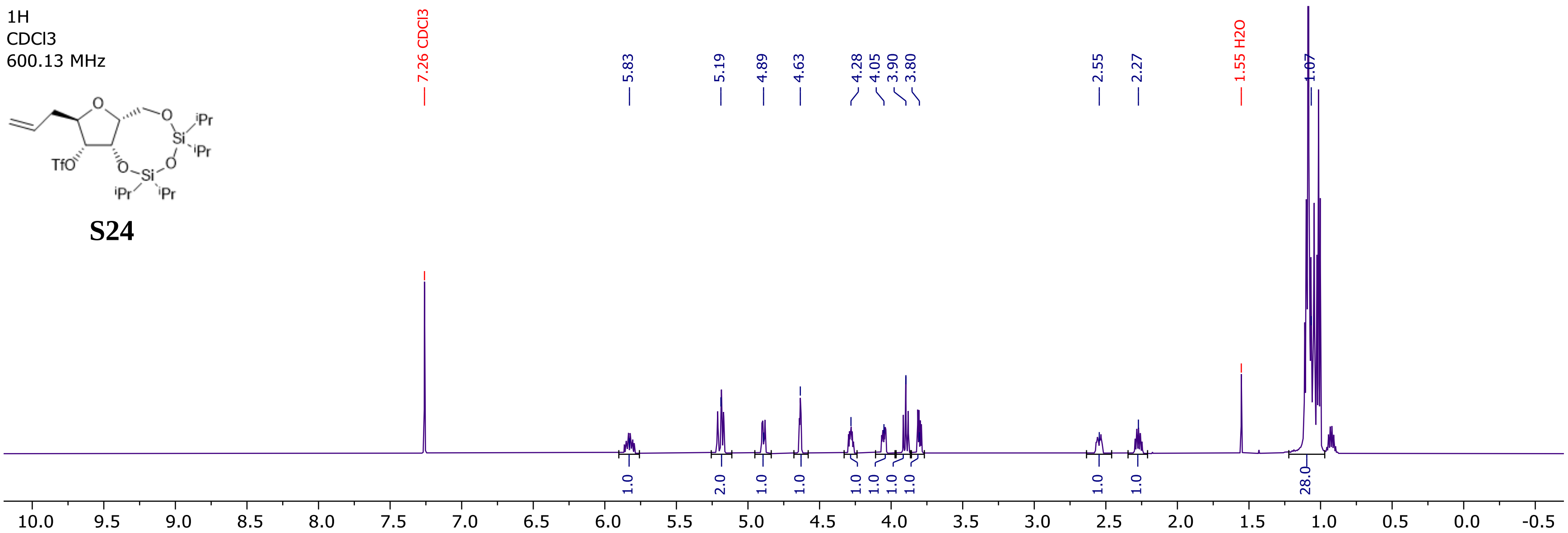
1H
CDCl3
600.13 MHz
7.26 CDCl3
5.83
5.19
4.89
4.63
4.28
4.05
3.90
3.80
2.55
2.27
1.55 H2O
1.07
1.0
2.0
1.0
1.0
1.0
1.0
1.0
1.0
1.0
1.0
28.0
10.0 9.5 9.0 8.5 8.0 7.5 7.0 6.5 6.0 5.5 5.0 4.5 4.0 3.5 3.0 2.5 2.0 1.5 1.0 0.5 0.0 -0.5
O
O
iPr
Si
iPr
TfO
O
Si
O
iPr
iPr

**S24**

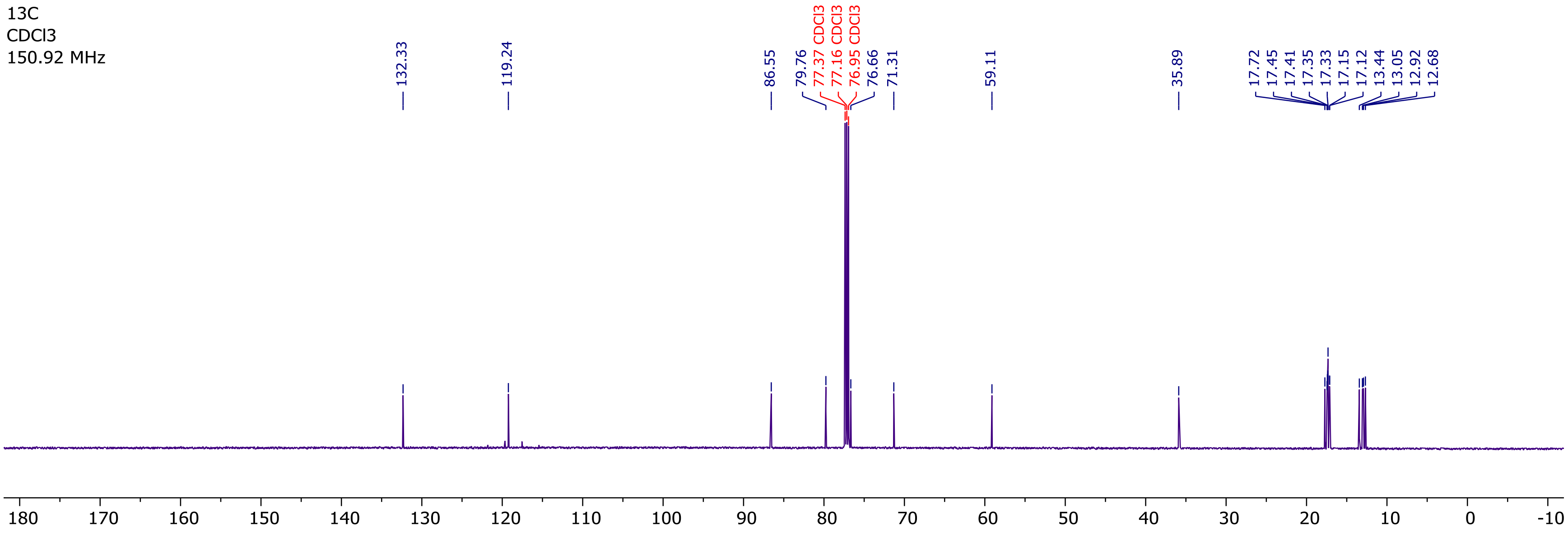
13C
CDCl3
150.92 MHz
132.33
119.24
86.55
79.76
77.37 CDCl3
77.16 CDCl3
76.95 CDCl3
76.66
71.31
59.11
35.89
17.72
17.45
17.41
17.35
17.33
17.15
17.12
13.44
13.05
12.92
12.68
180 170 160 150 140 130 120 110 100 90 80 70 60 50 40 30 20 10 0 -10

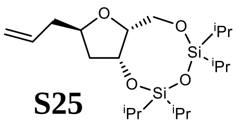


**S25**

To a stirred solution of **S24** (4.76 g, 8.68 mmol, 1.0 eq.) at rt in DMF (86 mL) was added $NaBH_4$ (825 mg, 21.8 mmol, 2.5 eq.). The reaction mixture was then submerged in a pre-heated oil bath (50 °C) and maintained at this temperature for 150 minutes, after which time starting material was consumed as monitored by TLC analysis. The oil bath was replaced with an ice bath (0 °C), and the reaction mixture was quenched by slow addition of saturated $NH_4Cl$ (90 mL) with vigorous stirring **[caution $H_2$ (g) evolved]**. After effervescence had subsided (*ca.* 20 min.), the biphasic reaction mixture was poured into a separatory funnel and extracted with $Et_2O$ (3x 100 mL). The combined organic extracts were washed with brine (1x 100 mL), dried ($MgSO_4$), filtered, and the solvent was removed in vacuo. The crude product was purified via flash column chromatography (25:1 to 20:1 hexanes/$Et_2O$). Appropriate fractions were pooled, and the solvent was removed in vacuo to yield **S25** (2.57 g, 74%) as a colorless oil.

**Analytical Data for S25:**

$R_f$ = 0.22 (24:1 hexanes/$Et_2O$)

$[\alpha]_D^{20}$ = -13.7° (c = 1.23, MeOH)

$^1$H NMR (600 MHz, $CDCl_3$) δ 5.81 (ddt, *J* = 17.2, 10.2, 7.0 Hz, 1H), 5.10 (dq, *J* = 17.2, 1.7 Hz, 1H), 5.07 (dt, *J* = 10.2, 1.6 Hz, 1H), 4.50 (t, *J* = 3.3 Hz, 1H), 4.31 (dq, *J* = 11.3, 5.9 Hz, 1H), 3.96 (ddd, *J* = 8.7, 6.4, 2.7 Hz, 1H), 3.88 – 3.79 (m, 2H), 2.41 – 2.34 (m, 1H), 2.31 – 2.25 (m, 1H), 2.02 (dd, *J* = 12.8, 5.2 Hz, 1H), 1.74 (ddd, *J* = 12.7, 10.4, 3.9 Hz, 1H), 1.19 – 0.86 (m, 28H).

$^{13}$C NMR (151 MHz, $CDCl_3$) δ 134.70, 117.31, 82.26, 77.96, 71.97, 59.82, 41.38, 40.28, 17.75, 17.62, 17.51, 17.49, 17.44, 17.26, 17.21, 13.50, 13.06, 12.75, 12.54.

HRMS (ESI): Anal. Calcd. for $C_{20}H_{41}O_4Si_2^+$ $[M+H]^+$ 401.2538, found 401.2558

IR (neat): $\nu_{max}\ (cm^{-1})$ = 2945 (m, CH), 2968 (m, CH), 1642 (w, C=C), 1467 (w), 1389 (w), 1249 (w)

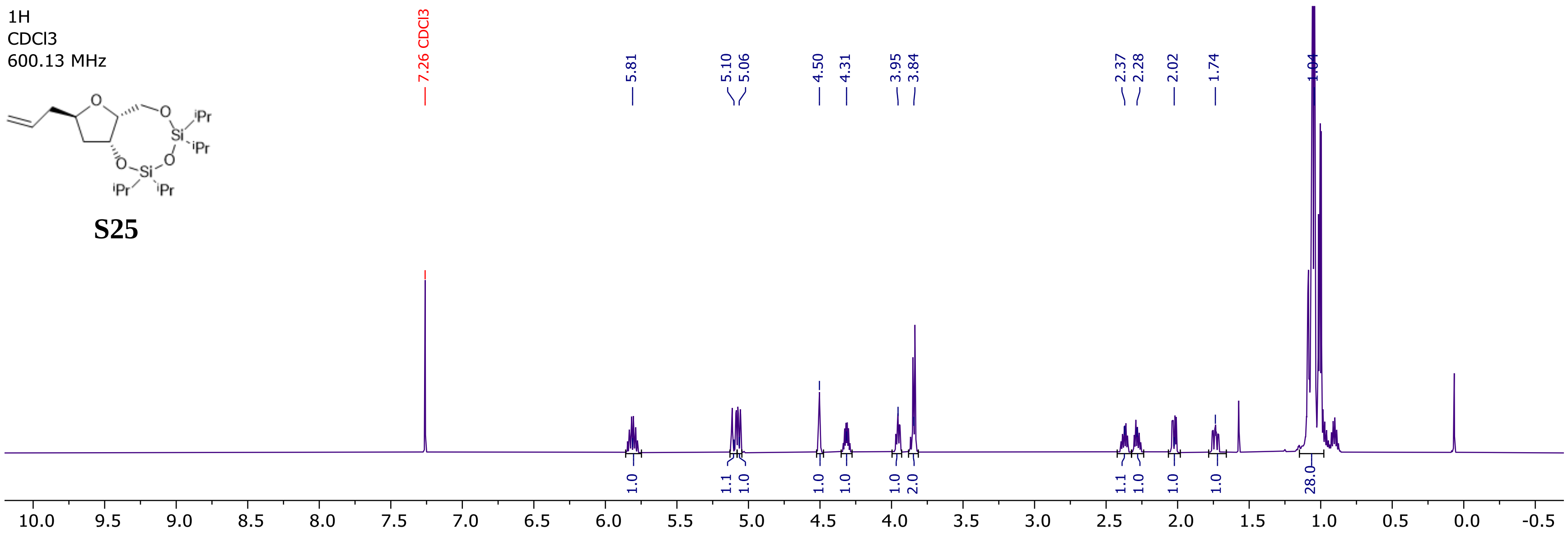
1H
CDCl3
600.13 MHz
O
iPr
Si
iPr
O
O
Si
iPr
iPr
7.26 CDCl3
5.81
5.10
5.06
4.50
4.31
3.95
3.84
2.37
2.28
2.02
1.74
1.04
1.0
1.1
1.0
1.0
1.0
1.0
2.0
1.1
1.0
1.0
1.0
28.0
10.0
9.5
9.0
8.5
8.0
7.5
7.0
6.5
6.0
5.5
5.0
4.5
4.0
3.5
3.0
2.5
2.0
1.5
1.0
0.5
0.0
-0.5

**S25**

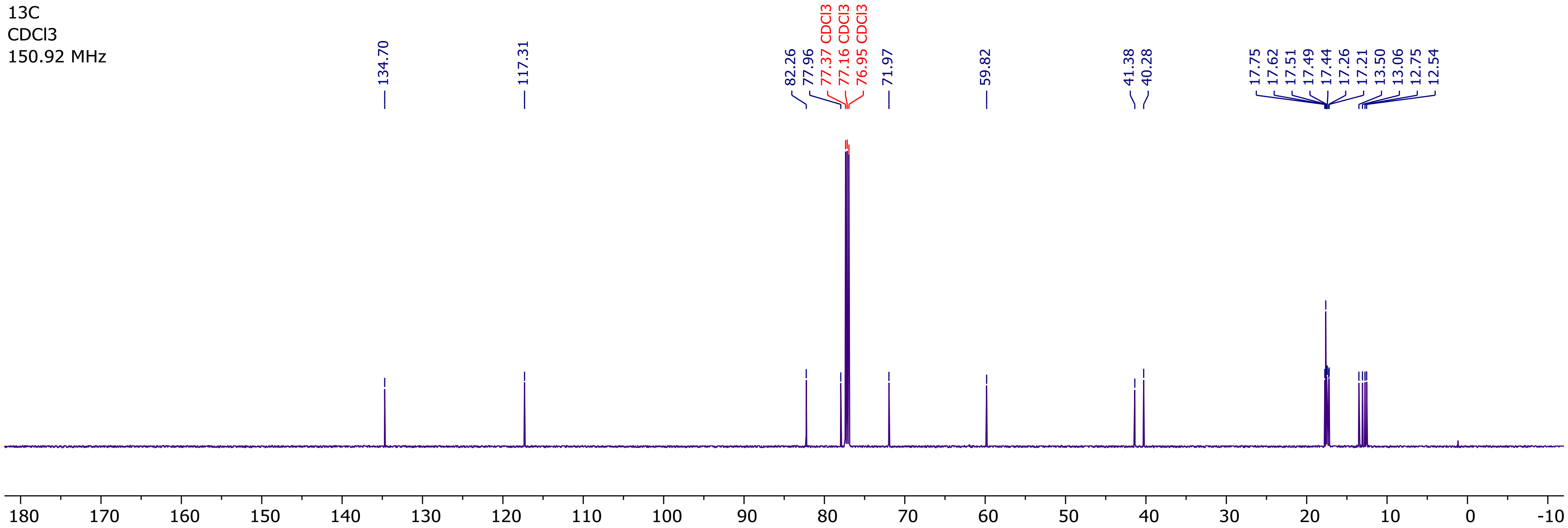
13C
CDCl3
150.92 MHz
134.70
117.31
82.26
77.96
77.37 CDCl3
77.16 CDCl3
76.95 CDCl3
71.97
59.82
41.38
40.28
17.75
17.62
17.51
17.49
17.44
17.26
17.21
13.50
13.06
12.75
12.54
180
170
160
150
140
130
120
110
100
90
80
70
60
50
40
30
20
10
0
-10

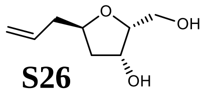

**S26**

Note: Reaction performed in capped vessel under ambient atmosphere.

A solution of *ca.* 1M aq. HCl in MeOH was prepared by diluting 8.2 mL of 37% (w/w) aq. HCl in reagent grade MeOH (91.8 mL). To a stirred solution of **S25** (2.55 g, 6.35 mmol, 1.0 eq.) in 1M aq. HCl in MeOH (24.0 mL, *ca.* 24.0 mmol, 3.78 eq.). The reaction mixture was submerged in a pre-heated oil bath (50 °C) and heating was maintained at this temperature for 2 h, after which time starting material was consumed as monitored by TLC analysis. The oil bath was replaced with an ice bath (0 °C) and $NaHCO_3$ (2.3 g, 27 mmol, 4.3 eq.) was added portion wise with vigorous stirring such that gentle effervescence was maintained **[caution $H_2$ (g) evolved]**. After effervescence had subsided, the reaction mixture was filtered through a cotton plug and solvent was removed in vacuo. The crude product was dissolved in $CH_2Cl_2$ and filtered once more, then the solvent was removed in vacuo. The crude product was purified via flash column chromatography (40:1 to 20:1 $CH_2Cl_2$/MeOH). Appropriate fractions were pooled, and solvent was removed in vacuo to yield **S26** (962 mg, 96%) as a colorless oil.

**Analytical Data for S26:**

$R_f$ = 0.11 (97:3 $CH_2Cl_2$/MeOH)

$[\alpha]_D^{20}$ = -18° (c = 0.83, MeOH)

$^1$H NMR (600 MHz, $CDCl_3$) δ 5.81 (ddt, *J* = 17.3, 10.3, 7.0 Hz, 1H), 5.15 – 5.09 (m, 1H), 5.08 (dd, *J* = 10.3, 1.9 Hz, 1H), 4.52 (q, *J* = 4.5, 4.1 Hz, 1H), 4.36 (dq, *J* = 9.6, 6.1 Hz, 1H), 4.00 – 3.90 (m, 3H), 3.39 (dd, *J* = 17.3, 9.2 Hz, 1H), 2.66 (t, *J* = 5.4 Hz, 1H), 2.38 (ddd, *J* = 13.4, 7.4, 5.9 Hz, 1H), 2.29 (dt, *J* = 13.3, 6.5 Hz, 1H), 2.07 (ddd, *J* = 13.3, 5.7, 1.5 Hz, 1H), 1.78 (ddd, *J* = 13.7, 9.5, 4.9 Hz, 1H).

$^{13}$C NMR (151 MHz, $CDCl_3$) δ 134.37, 117.58, 80.63, 77.83, 74.58, 62.06, 41.65, 40.15.

HRMS (ESI): Anal. Calcd. for $C_8H_{15}O_3^+$ $[M+H]^+$ 159.1016, found 159.1023

IR (neat): $\nu_{max}$ $(cm^{-1})$ = 3376 (br, OH), 3078 (w, C=*CH*), 2931 (m, CH), 1642 (w, C=C), 1435 (w), 1333 (w), 1281 (w)

1H
CDCl3
600.13 MHz

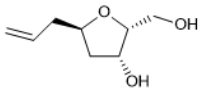


**S26**

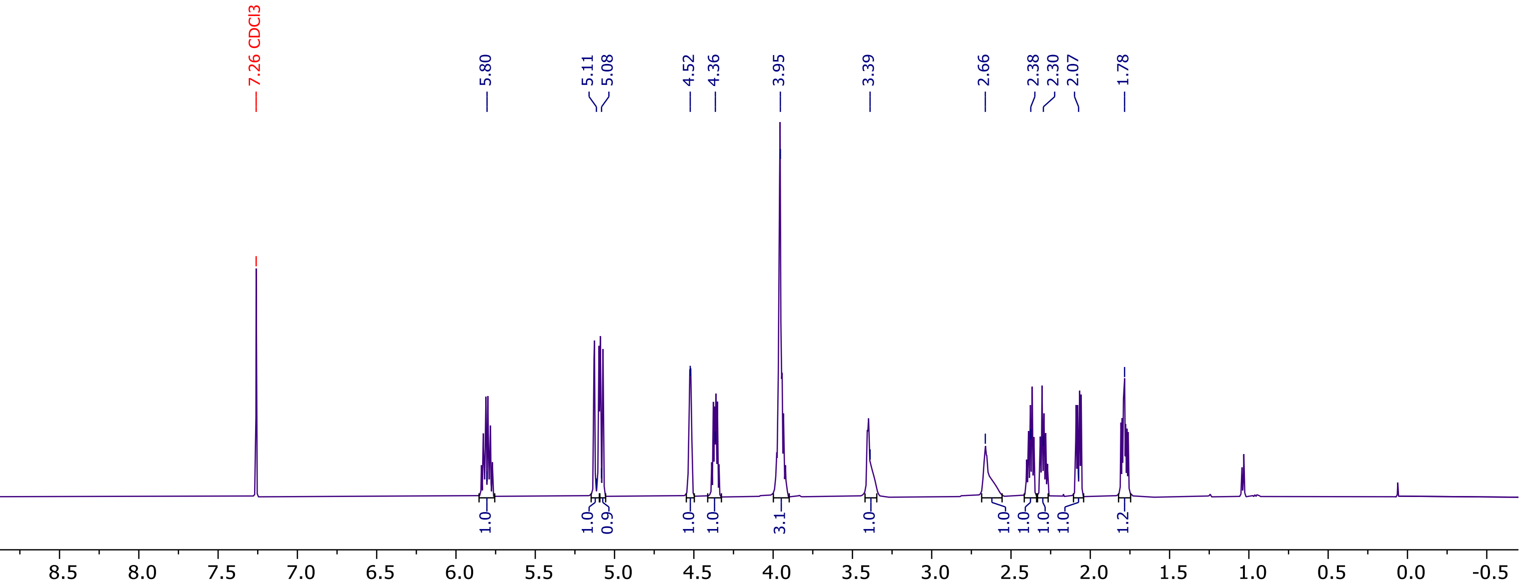


13C
CDCl3
150.92 MHz

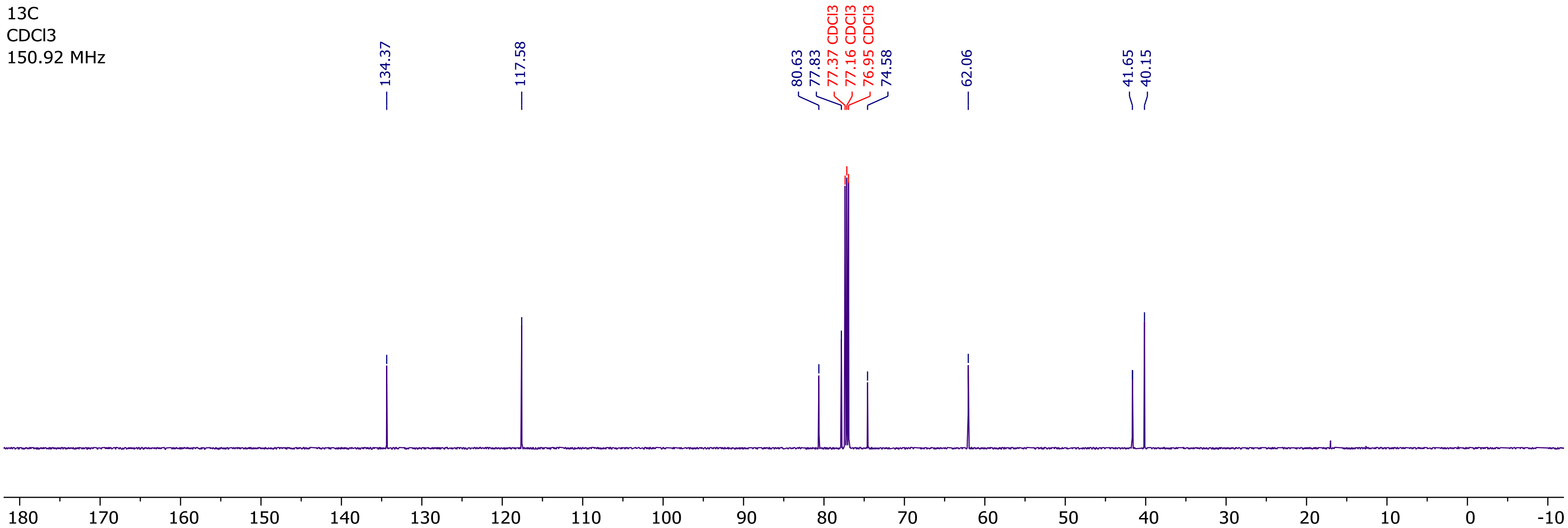

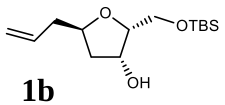

**1b**

To a cold (0 °C), stirred solution of **S26** (945 mg, 5.97 mmol, 1.0 eq.) and imidazole (814 mg, 12.0 mmol, 2.0 eq.) in anhydrous $CH_2Cl_2$ (20 mL) was added TBSCl (990 mg, 6.6 mmol, 1.1 eq.) in anhydrous $CH_2Cl_2$ (10 mL) via syringe. After stirring for 150 minutes with the bath maintained at 0 °C, a further portion of TBSCl (90 mg, 0.6 mmol, 0.1 eq.) was added in anhydrous $CH_2Cl_2$ (1 mL). Then the bath was removed, and the reaction mixture warmed to rt. After stirring for a further 30 minutes at rt, the reaction mixture was poured into a separatory funnel and washed with $H_2O$ (2x 30 mL). The combined aqueous layers were back extracted with $CH_2Cl_2$ (3x 30 mL). The combined organic layers were washed with brine (1x 50 mL), dried ($Na_2SO_4$), filtered, and the solvent was removed in vacuo. The crude product was purified via flash column chromatography (10:1 to 20:3 hexanes/EtOAc). Appropriate fractions were pooled, and the solvent was removed in vacuo to yield **1b** (1.46 g, 90%) as a colorless oil.

**Analytical Data for 1b:**

Rf = 0.2 (9:1 hexanes/EtOAc)

$[\alpha]_D^{20}$ = -14° (c = 0.74, MeOH)

$^1$H NMR (600 MHz, CDCl3) δ 5.80 (ddt, *J* = 17.2, 10.2, 6.9 Hz, 1H), 5.11 (dq, *J* = 17.2, 1.7 Hz, 1H), 5.06 (ddt, *J* = 10.2, 2.3, 1.2 Hz, 1H), 4.55 – 4.50 (m, 1H), 4.31 (dq, *J* = 9.7, 6.0 Hz, 1H), 4.01 – 3.88 (m, 3H), 3.54 (d, *J* = 3.9 Hz, 1H), 2.38 – 2.32 (m, 1H), 2.30 – 2.24 (m, 1H), 2.05 (ddd, *J* = 13.1, 5.3, 1.5 Hz, 1H), 1.77 – 1.69 (m, 1H), 1.76 – 1.69 (m, 1H), 0.89 (s, 9H), 0.10 (s, 3H), 0.09 (s, 3H).

$^{13}$C NMR (151 MHz, $CDCl_3$) δ 134.71, 117.19, 80.60, 77.81, 74.25, 62.95, 41.30, 40.05, 25.90, 18.24, -5.32, -5.39.

HRMS (ESI): Anal. Calcd. for $C_{14}H_{29}O_3Si^+$ $[M+H]^+$ 273.1880, found 273.1893

IR (neat): $\nu_{max}\ (cm^{-1})$ = 3438 (br, OH), 3078 (w, C=*CH*), 2930 (m, CH), 2858 (m, CH), 1642 (w, C=C), 1469 (w), 1362 (w), 1254 (s)

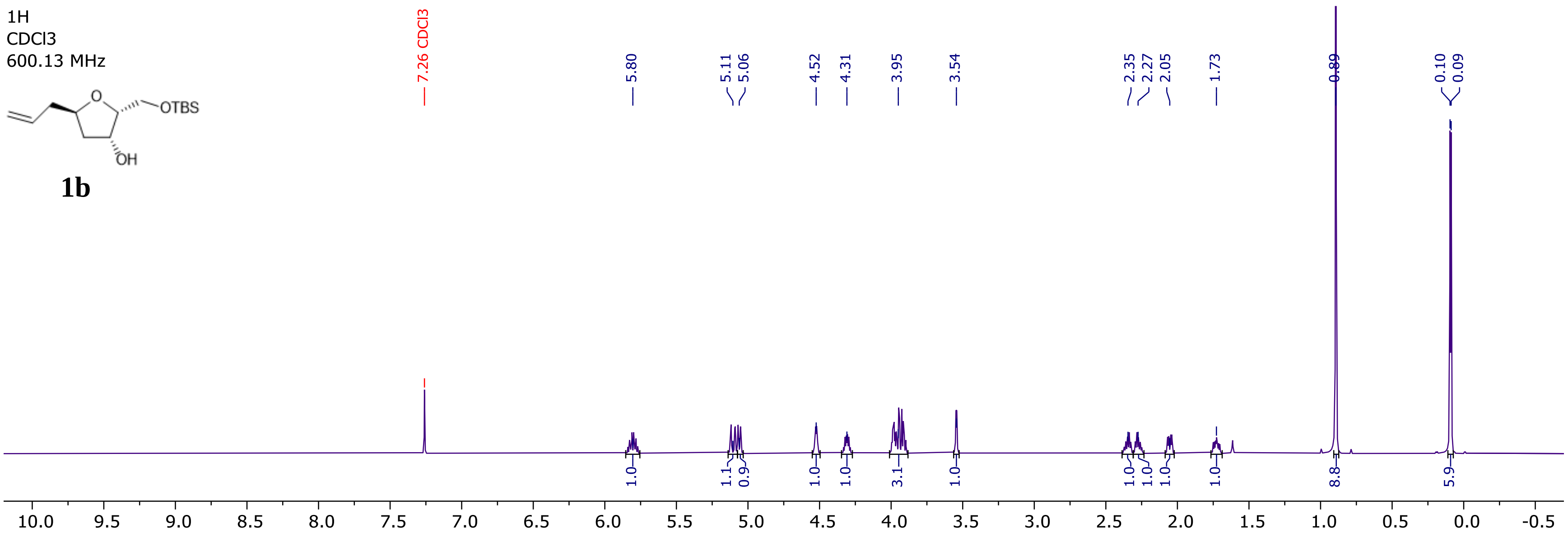
1H
CDCl3
600.13 MHz
OTBS
OH
1b
7.26 CDCl3
5.80
5.11
5.06
4.52
4.31
3.95
3.54
2.35
2.27
2.05
1.73
0.89
0.10
0.09
1.0
1.1
0.9
1.0
1.0
3.1
1.0
1.0
1.0
1.0
1.0
8.8
5.9
10.0 9.5 9.0 8.5 8.0 7.5 7.0 6.5 6.0 5.5 5.0 4.5 4.0 3.5 3.0 2.5 2.0 1.5 1.0 0.5 0.0 -0.5

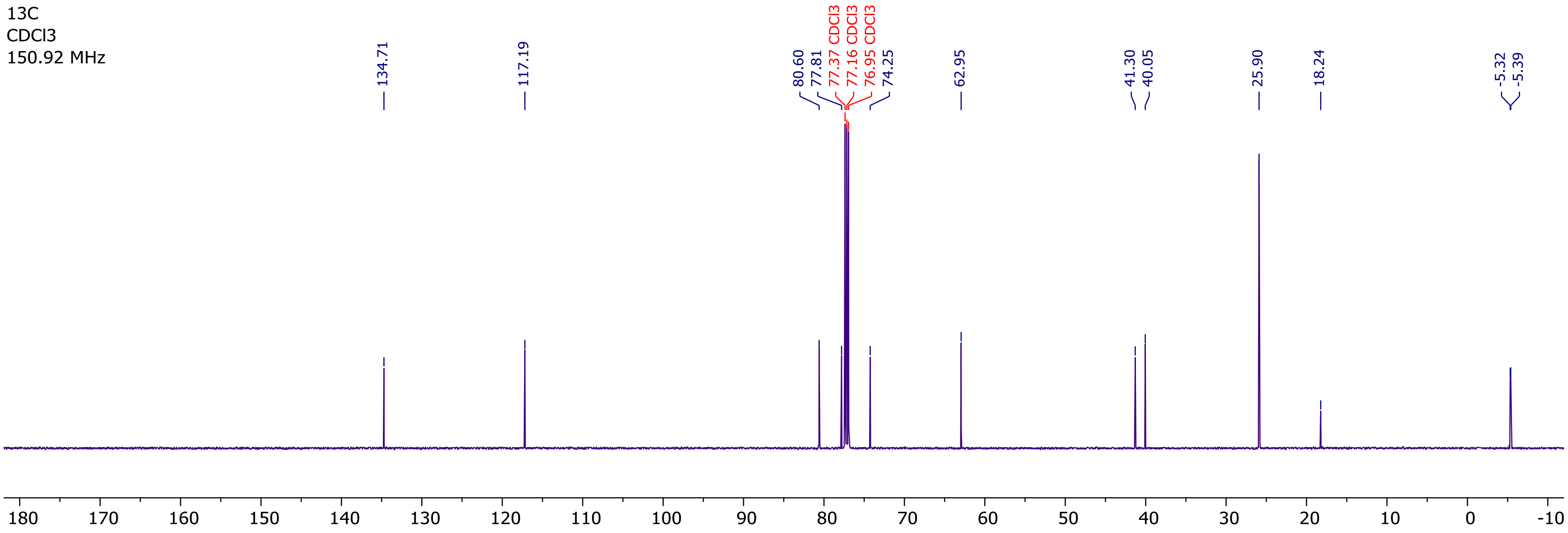
13C
CDCl3
150.92 MHz
134.71
117.19
80.60
77.81
77.37 CDCl3
77.16 CDCl3
76.95 CDCl3
74.25
62.95
41.30
40.05
25.90
18.24
-5.32
-5.39
180 170 160 150 140 130 120 110 100 90 80 70 60 50 40 30 20 10 0 -10

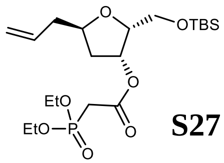

**S27**

Note: Reaction performed in capped vessel under ambient atmosphere.

To a stirred solution of **1b** (701 mg, 2.57 mmol, 1.0 eq.) and DIC (0.875 mL, 713 mg, 5.66 mmol, 2.2 eq.) in anhydrous $CH_2Cl_2$ (20 mL) was added diethylphosphonoacetic acid (0.705 mL, 863 mg, 4.4 mmol, 1.7 eq.) in anhydrous $CH_2Cl_2$ (6 mL). After stirring 3 min at rt an exothermic reaction takes place and the urea byproduct rapidly crashes out of solution. After stirring a further 30 min, the reaction mixture was diluted with $CH_2Cl_2$ (25 mL) then poured into a separatory funnel and washed with $H_2O$ (3x 50 mL). The combined aqueous layers were back extracted with $CH_2Cl_2$ (3x 40 mL). The combined organic layers were washed with brine (1x 80 mL) then dried ($Na_2SO_4$) and filtered, and the solvent was removed in vacuo. The crude product was purified via flash column chromatography (9:1 $CH_2Cl_2$/MeCN). Appropriate fractions were pooled, and solvent was removed in vacuo to yield **S27** (1.10 g, 95%) as a viscous colorless oil.

**Analytical Data for S27:**

$R_f$ = 0.26 (17:3 $CH_2Cl_2/CH_3CN$)

$[\alpha]_D^{20}$ = -18° (c = 0.88, MeOH)

$^1$H NMR (600 MHz, $CDCl_3$) δ 5.78 (ddt, *J* = 17.2, 10.2, 7.0 Hz, 1H), 5.43 (t, *J* = 3.9 Hz, 1H), 5.13 – 5.02 (m, 2H), 4.25 (dt, *J* = 9.5, 6.0 Hz, 1H), 4.20 – 4.12 (m, 4H), 4.07 (ddd, *J* = 7.0, 5.8, 3.7 Hz, 1H), 3.75 (qd, *J* = 10.2, 6.4 Hz, 2H), 2.99 (dd, *J* = 21.6, 14.3 Hz, 1H), 2.92 (dd, *J* = 21.5, 14.4 Hz, 1H), 2.36 (dt, *J* = 13.5, 6.3 Hz, 1H), 2.27 (dt, *J* = 13.8, 6.5 Hz, 1H), 2.13 (ddd, *J* = 13.9, 5.8, 1.3 Hz, 1H), 1.87 (ddd, *J* = 14.2, 9.5, 4.9 Hz, 1H), 1.34 (td, *J* = 7.1, 3.2 Hz, 6H), 0.86 (s, 9H), 0.04 (s, 3H), 0.03 (s, 3H).

$^{13}$C NMR (151 MHz, $CDCl_3$) δ 165.20, 165.16, 134.16, 117.69, 81.01, 75.88, 62.84, 62.80, 62.77, 62.73, 61.26, 40.07, 38.49, 34.93, 34.04, 25.95, 18.35, 16.52, 16.48, -5.17, -5.31.

HRMS (ESI): Anal. Calcd. for $C_{20}H_{43}NO_7PSi^+$ $[M+NH_4]^+$ 468.2541, found 468.2541

IR (neat): $\nu_{max}\ (cm^{-1})$ = 2930 (m, CH), 2857 (m, CH), 1740 (s, C=O), 1469 (w), 1443 (w), 1259 (s)

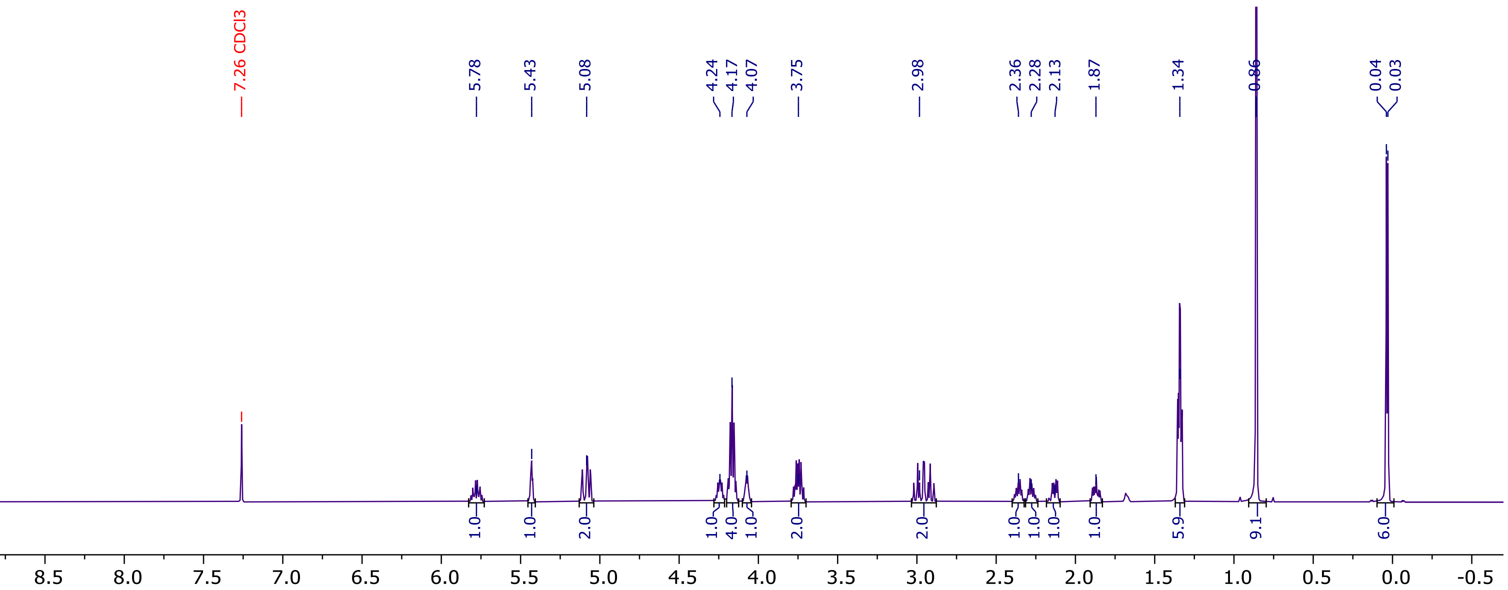
1H
CDCl3
600.13 MHz
7.26 CDCl3
5.78
5.43
5.08
4.24
4.17
4.07
3.75
2.98
2.36
2.28
2.13
1.87
1.34
0.86
0.04
0.03
1.0
1.0
2.0
1.0
4.0
1.0
2.0
2.0
1.0
1.0
1.0
1.0
5.9
9.1
6.0
10.0
9.5
9.0
8.5
8.0
7.5
7.0
6.5
6.0
5.5
5.0
4.5
4.0
3.5
3.0
2.5
2.0
1.5
1.0
0.5
0.0
-0.5

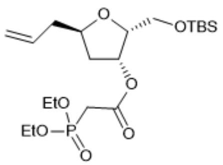
O
OTBS
O
EtO
EtO
P
O
O

**S27**

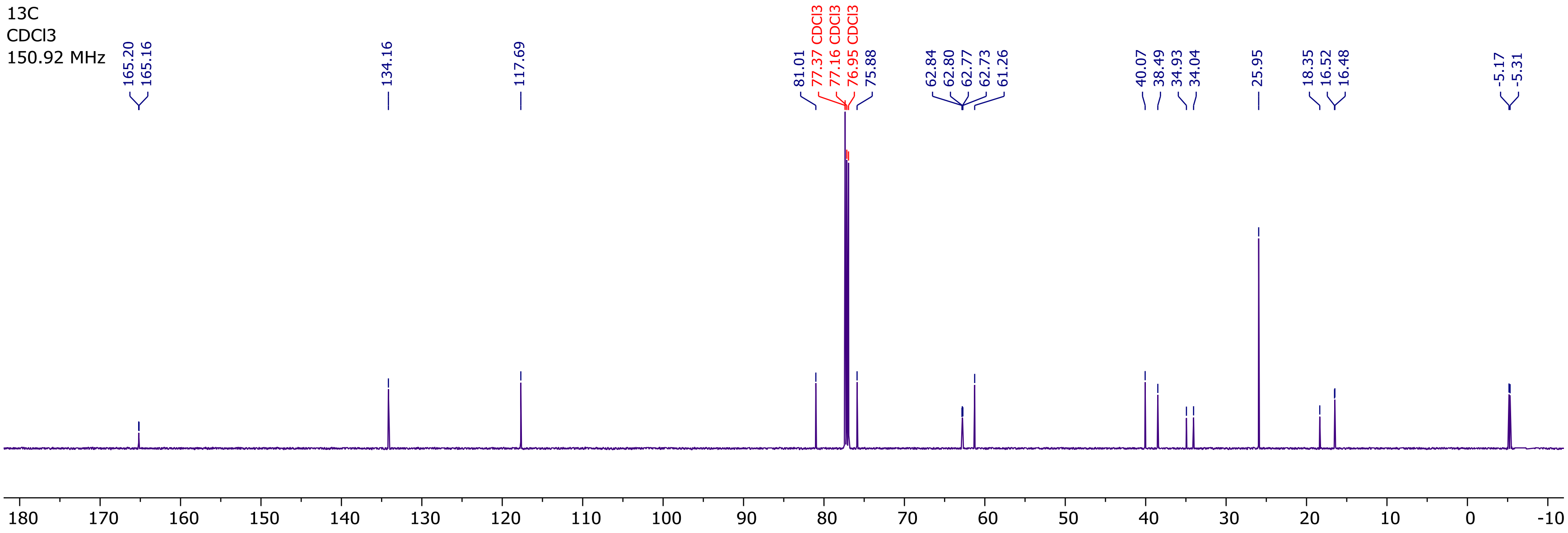
13C
CDCl3
150.92 MHz
165.20
165.16
134.16
117.69
81.01
77.37 CDCl3
77.16 CDCl3
76.95 CDCl3
75.88
62.84
62.80
62.77
62.73
61.26
40.07
38.49
34.93
34.04
25.95
18.35
16.52
16.48
-5.17
-5.31
180
170
160
150
140
130
120
110
100
90
80
70
60
50
40
30
20
10
0
-10

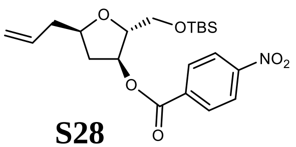


**S28**

Note: Reaction performed in capped vessel under ambient atmosphere.

To a cold (0 °C), stirred solution of **1b** (707 mg, 2.60 mmol, 1.0 eq), p-$NO_2$BzOH (694 mg, 4.15 mmol, 1.6 eq.), and $PPh_3$ (1.09 g, 4.15 mmol, 1.6 eq.) in anhydrous THF (26 mL) was added DIAD (0.820 mL, 842 mg, 4.15 mmol, 1.6 eq.) dropwise via syringe. The reaction mixture was stirred for 1 h while slowly warming to rt, after which time starting material was consumed as monitored by TLC analysis. Then the reaction mixture was quenched with saturated $NaHCO_3$ (30 mL) and the aqueous layer extracted with $Et_2O$ (3x 50 mL). The combined organic extracts were dried ($MgSO_4$), filtered, and solvent was removed in vacuo. The crude product was purified via flash column chromatography (37:3 hexanes/$Et_2O$). Appropriate fractions were pooled, and the solvent was removed in vacuo to yield **S28** (1.08 g, 95%) as a yellow oil.

**Analytical Data for S28:**

$R_f$ = 0.14 (37:3 hexanes/$Et_2O$)

$[\alpha]_D^{20}$ = +16.1° (c = 1.16, MeOH)

$^1$H NMR (600 MHz, $CDCl_3$) δ 8.33 – 8.28 (m, 2H), 8.23 – 8.18 (m, 2H), 5.84 (ddt, *J* = 17.2, 10.3, 6.9 Hz, 1H), 5.54 (ddd, *J* = 7.1, 3.4, 2.4 Hz, 1H), 5.14 – 5.06 (m, 2H), 4.33 (dt, *J* = 13.1, 6.6 Hz, 1H), 4.23 (td, *J* = 3.8, 2.3 Hz, 1H), 3.83 (dd, *J* = 10.8, 3.4 Hz, 1H), 3.73 (dd, *J* = 10.9, 4.3 Hz, 1H), 2.58 (dt, *J* = 14.1, 7.2 Hz, 1H), 2.51 – 2.45 (m, 1H), 2.35 (dtt, *J* = 13.8, 6.7, 1.3 Hz, 1H), 1.90 (ddd, *J* = 13.7, 5.9, 3.4 Hz, 1H), 0.89 (s, 9H), 0.08 (s, 3H), 0.07 (s, 3H).

$^{13}$C NMR (151 MHz, $CDCl_3$) δ 164.50, 150.74, 135.56, 134.61, 130.84, 123.76, 117.48, 84.32, 79.07, 78.58, 77.37, 77.16, 76.95, 64.39, 40.77, 37.57, 25.99, 18.37, -5.29, -5.37.

HRMS (ESI): Anal. Calcd. for $C_{21}H_{32}NO_6Si^+$ $[M+H]^+$ 422.1993, found 422.1984

IR (neat): $\nu_{max}$ $(cm^{-1})$ = 2929 (m, CH), 2857 (m, CH), 1726 (s, C=O), 1608 (w), 1520 (s), 1470 (w), 1350 (m), 1274 (s)

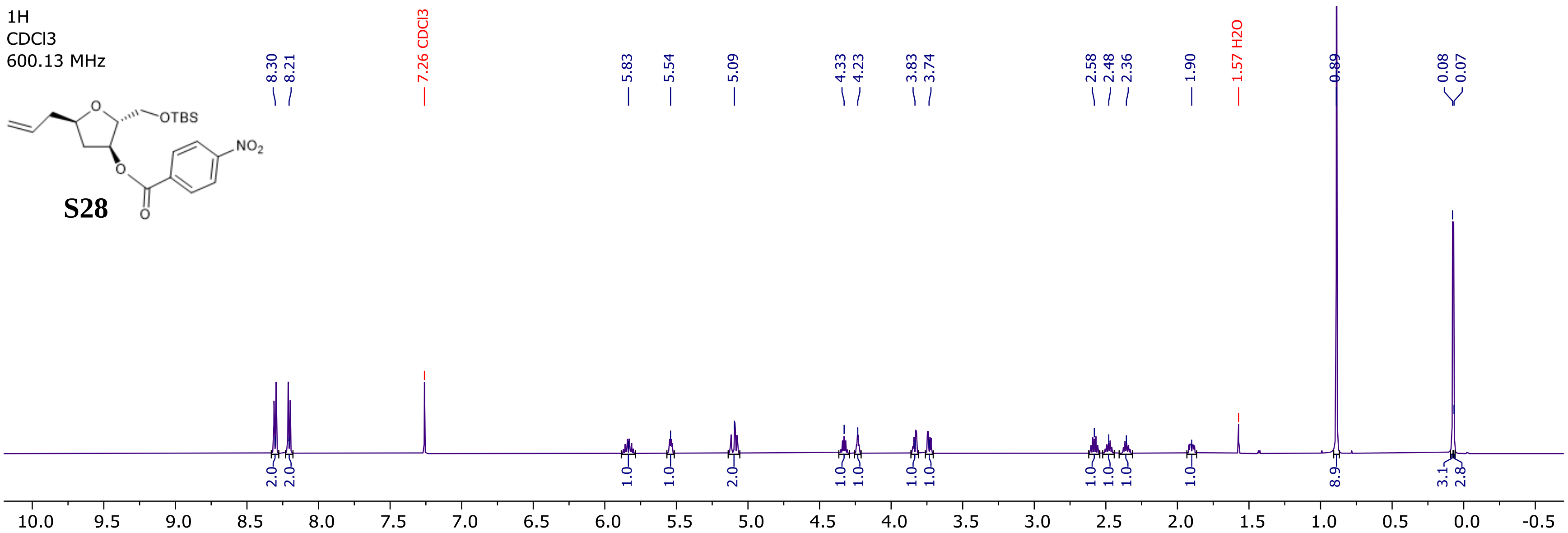
1H
CDCl3
600.13 MHz
OTBS
NO2
O
S28
8.30
8.21
7.26 CDCl3
5.83
5.54
5.09
4.33
4.23
3.83
3.74
2.58
2.48
2.36
1.90
1.57 H2O
0.89
0.08
0.07
2.0
2.0
1.0
1.0
2.0
1.0
1.0
1.0
1.0
1.0
1.0
1.0
1.0
8.9
3.1
2.8
10.0 9.5 9.0 8.5 8.0 7.5 7.0 6.5 6.0 5.5 5.0 4.5 4.0 3.5 3.0 2.5 2.0 1.5 1.0 0.5 0.0 -0.5

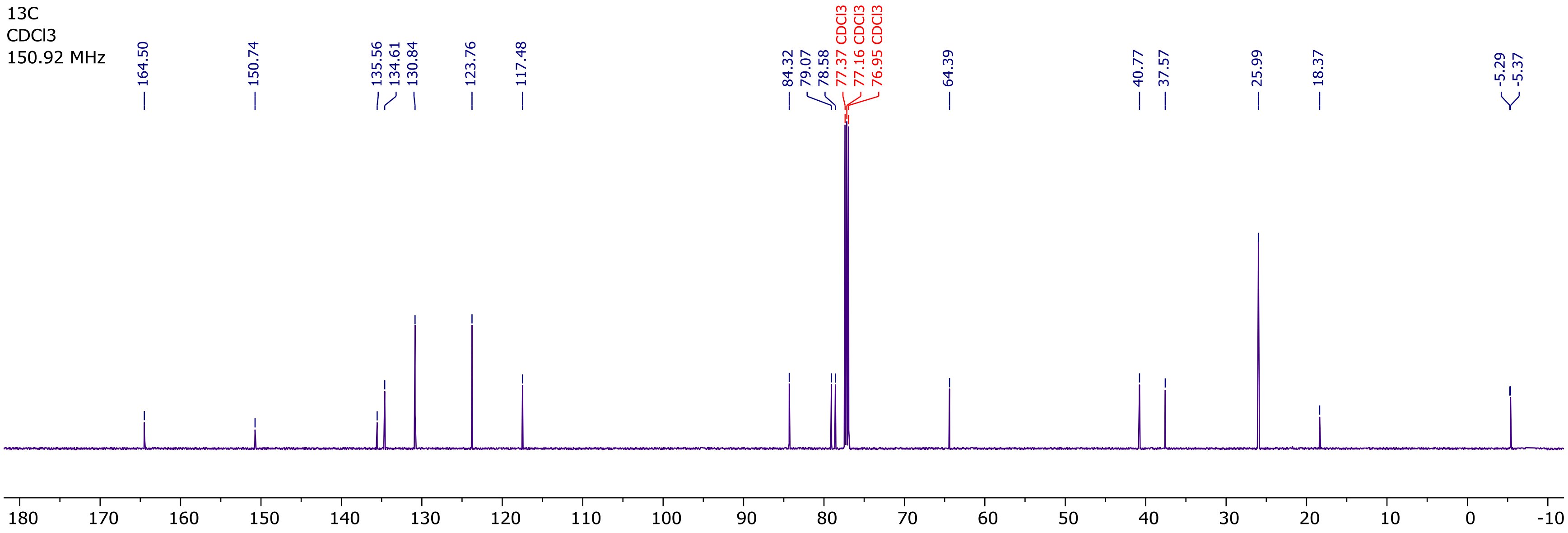
13C
CDCl3
150.92 MHz
164.50
150.74
135.56
134.61
130.84
123.76
117.48
84.32
79.07
78.58
77.37 CDCl3
77.16 CDCl3
76.95 CDCl3
64.39
40.77
37.57
25.99
18.37
-5.29
-5.37
180 170 160 150 140 130 120 110 100 90 80 70 60 50 40 30 20 10 0 -10

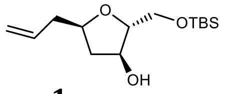


**1e**

Note: Reaction performed in capped vessel under ambient atmosphere.

To **S28** (1.06 g, 2.53 mmol, 1.0 eq.) in a 100 mL round bottomed flask was added 25 mL of 7N $NH_3$ in MeOH (excess). The resultant reaction mixture stirred for 1 h, after which time starting material was consumed as monitored by TLC analysis. The solvent was removed in vacuo and the crude product was purified via flash column chromatography (17:3 to 3:1 hexanes/$Et_2O$). Appropriate fractions were pooled, and the solvent was removed in vacuo to yield **1e** (622 mg, 90%) as a colorless oil.

**Analytical Data for S28:**

$R_f$ = 0.12 (4:1 hexanes/$Et_2O$)

$[\alpha]_D^{20}$ = +21° (c = 0.77, MeOH)

$^1$H NMR (600 MHz, $CDCl_3$) δ 5.81 (ddt, *J* = 17.2, 10.2, 7.0 Hz, 1H), 5.15 – 5.06 (m, 2H), 4.32 (tt, *J* = 6.7, 4.1 Hz, 1H), 4.12 (dt, *J* = 7.8, 6.3 Hz, 1H), 3.84 – 3.78 (m, 2H), 3.56 (dd, *J* = 11.0, 8.0 Hz, 1H), 2.42 (dt, *J* = 13.7, 6.7 Hz, 1H), 2.35 (dt, *J* = 13.0, 6.7 Hz, 2H), 2.02 (d, *J* = 3.9 Hz, 1H), 1.69 (dt, *J* = 12.7, 7.2 Hz, 1H), 0.89 (s, 9H), 0.07 (s, 6H).

$^{13}$C NMR (151 MHz, $CDCl_3$) δ 134.70, 117.49, 84.22, 78.02, 75.21, 64.76, 40.86, 39.74, 26.03, 18.40, -5.31, -5.33.

HRMS (ESI): Anal. Calcd. for $C_{14}H_{29}O_3Si^+$ $[M+H]^+$ 273.1880, found 273.1870

IR (neat): $\nu_{max}$ $(cm^{-1})$ = 3428 (br, OH), 3078 (w, C=*CH*), 2930 (m, CH), 2858 (m, CH), 1642 (w, C=C), 1468 (w), 1362 (w), 1254 (s)

1H
CDCl3
600.13 MHz

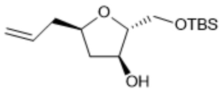

**1e**

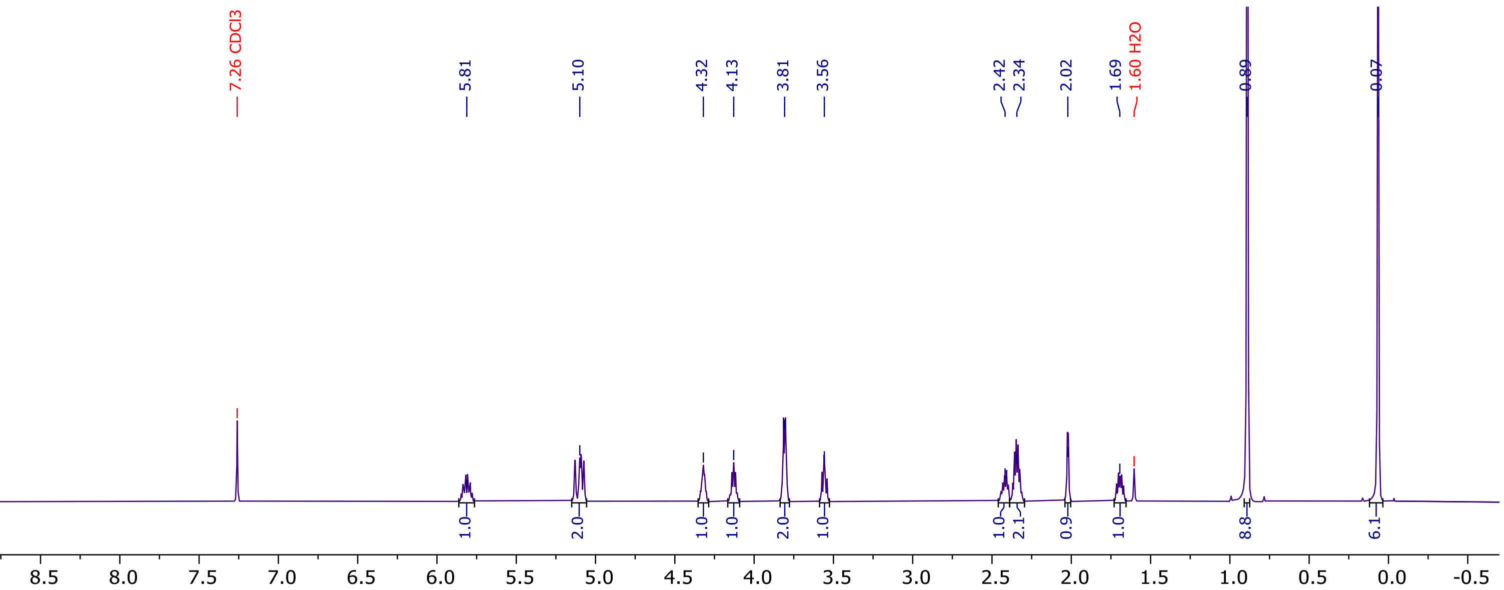


13C
CDCl3
150.92 MHz

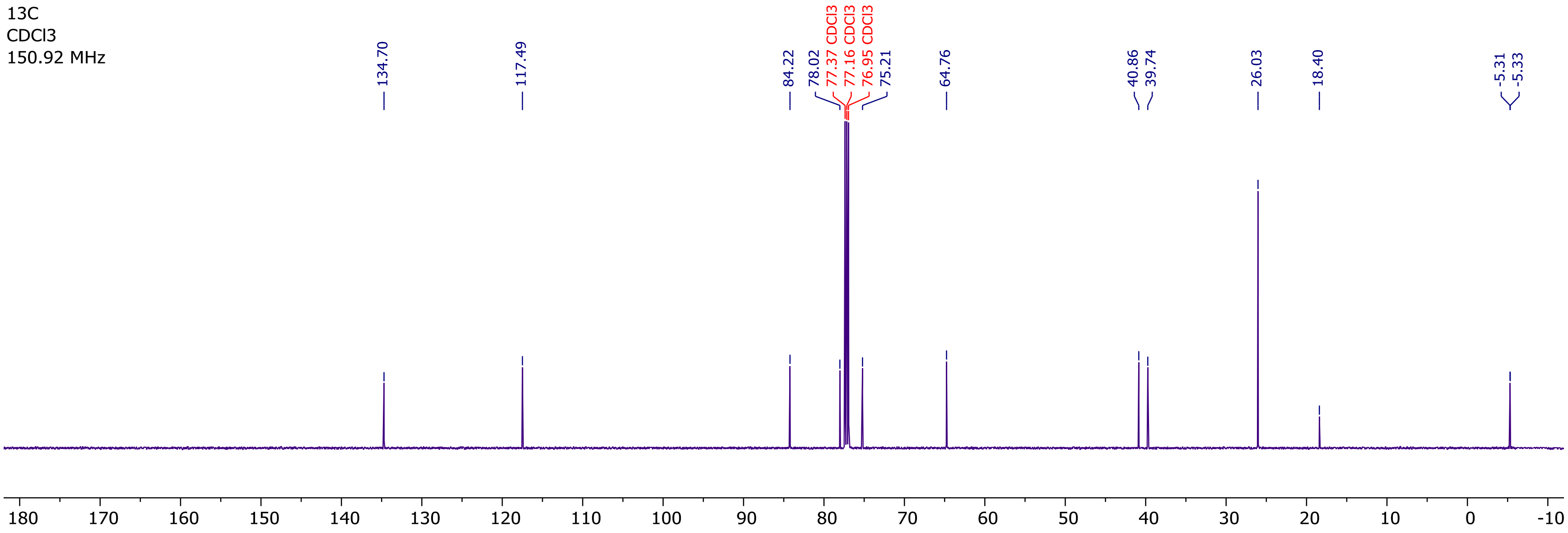

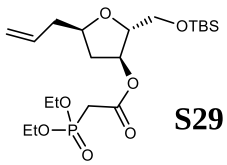

**S29**

Note: Reaction performed in capped vessel under ambient atmosphere.

To a stirred solution of **1e** (607 mg, 2.23 mmol, 1.0 eq.) and DIC (0.760 mL, 618 mg, 4.90 mmol, 2.2 eq.) in anhydrous $CH_2Cl_2$ (15 mL) was added diethylphosphonoacetic acid (0.905 mL, 743 mg, 4.4 mmol, 1.7 eq.) in anhydrous $CH_2Cl_2$ (7 mL). After stirring 3 min at rt an exothermic reaction takes place and the urea byproduct rapidly crashes out of solution. After stirring further for 12 min, the reaction mixture was diluted with $CH_2Cl_2$ (25 mL) then poured into a separatory funnel and washed with $H_2O$ (3x 50 mL). The combined aqueous layers were back extracted with $CH_2Cl_2$ (3x 40 mL). The combined organic layers were washed with brine (80 mL) then dried ($Na_2SO_4$) and filtered, and the solvent was removed in vacuo. The crude product was purified via flash column chromatography (9:1 $CH_2Cl_2$/MeCN). Appropriate fractions were pooled, and the solvent was removed in vacuo to yield **S29** (853 mg, 85%) as a viscous colorless oil.

**Analytical Data for S29:**

$R_f$ = 0.29 (17:3 $CH_2Cl_2$/$CH_3CN$)

$[\alpha]_D^{20}$ = +14° (c = 0.99, MeOH)

$^1$H NMR (600 MHz, $CDCl_3$) δ 5.80 (ddt, *J* = 17.2, 10.2, 6.9 Hz, 1H), 5.30 (dt, *J* = 7.2, 2.9 Hz, 1H), 5.10 (dq, *J* = 17.2, 1.6 Hz, 1H), 5.07 (ddt, *J* = 10.3, 2.2, 1.2 Hz, 1H), 4.23 (p, *J* = 6.6 Hz, 1H), 4.20 – 4.14 (m, 4H), 4.07 (q, *J* = 3.3 Hz, 1H), 3.74 (dd, *J* = 10.9, 3.4 Hz, 1H), 3.67 (dd, *J* = 10.9, 3.9 Hz, 1H), 2.97 (d, *J* = 21.6 Hz, 2H), 2.47 – 2.40 (m, 2H), 2.32 – 2.25 (m, 1H), 1.76 (ddd, *J* = 13.6, 5.8, 3.4 Hz, 1H), 1.34 (t, *J* = 7.1 Hz, 6H), 0.87 (s, 9H), 0.05 (s, 3H), 0.04 (s, 3H).

$^{13}$C NMR (151 MHz, $CDCl_3$) δ 165.75, 165.70, 134.83, 117.26, 84.23, 79.11, 78.03, 64.33, 62.87, 62.84, 62.83, 62.79, 40.60, 37.39, 35.03, 34.15, 25.99, 18.35, 16.52, 16.48, -5.31, -5.40.

HRMS (ESI): Anal. Calcd. for $C_{20}H_{43}NO_7PSi^+$ $[M+NH_4]^+$ 468.2541, found 468.2539

IR (neat): $\nu_{max}$ $(cm^{-1})$ = 2931 (m, CH), 2858 (m, CH), 1739 (C=O), 1469 (w), 1390 (w), 1257 (s)

1H
CDCl3
600.13 MHz

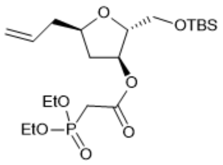


**S29**

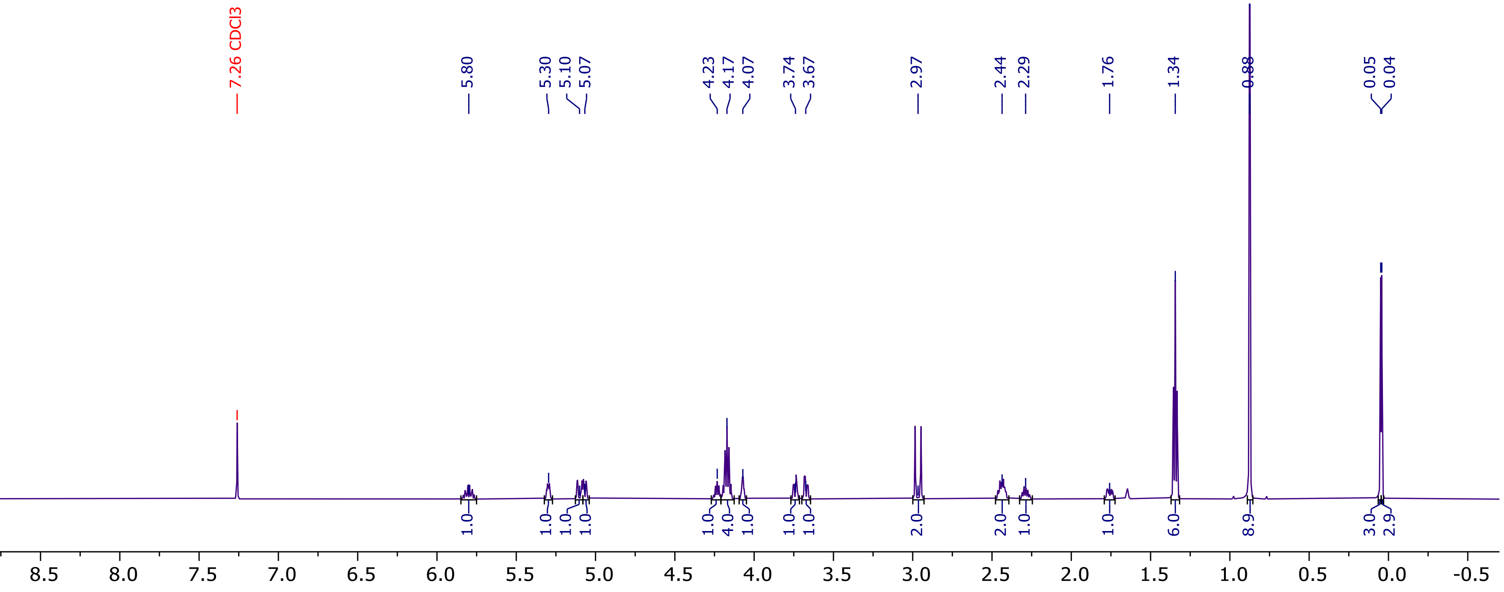


13C
CDCl3
150.92 MHz

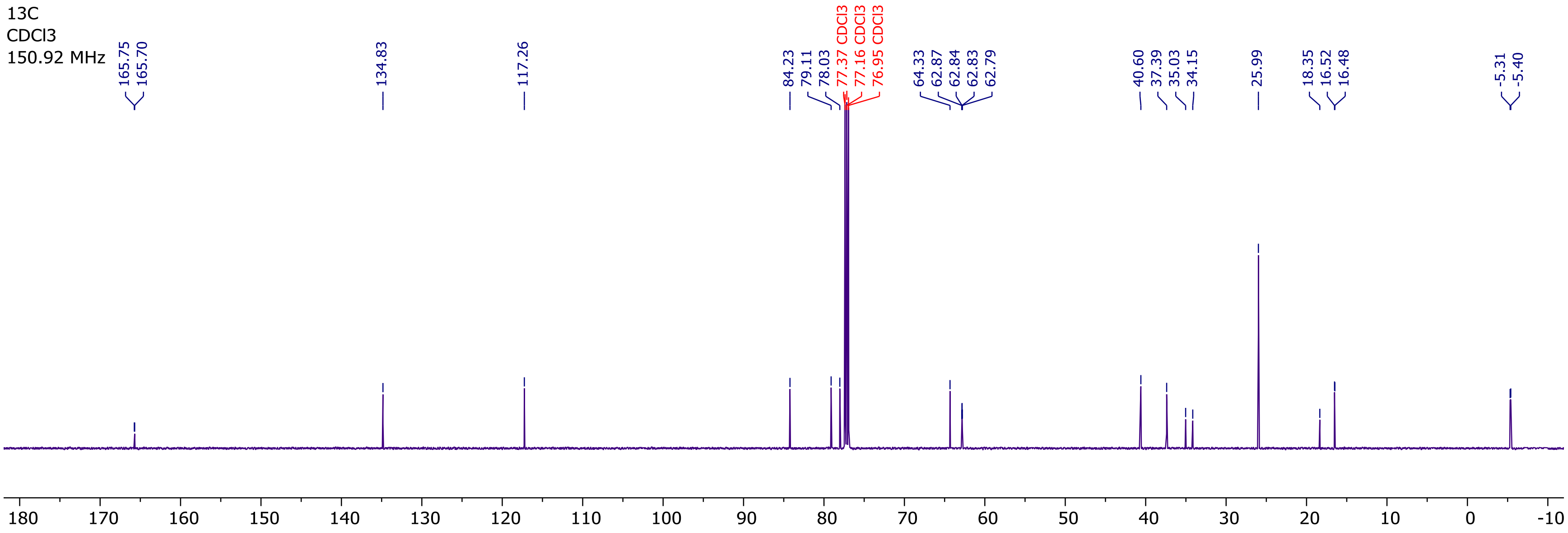

## Procedures for the Synthesis and Characterization of Acid/Aldehyde Building Blocks

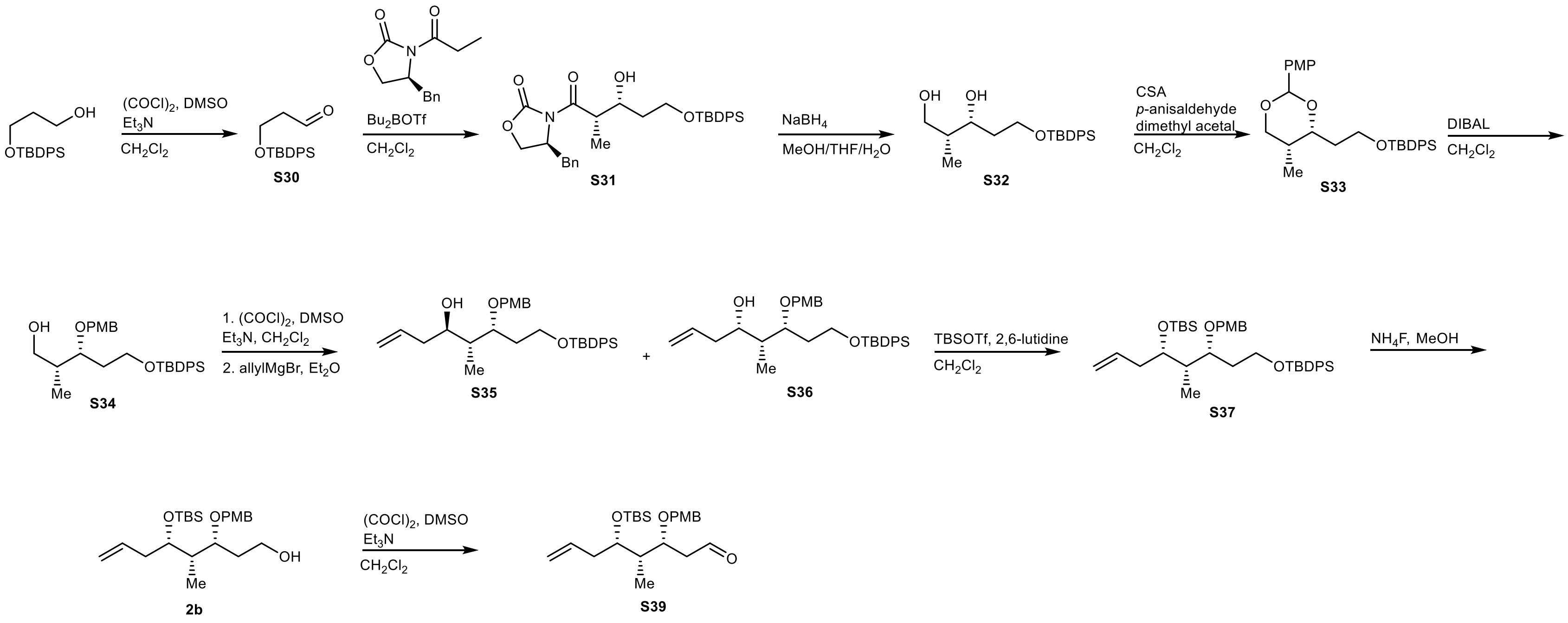


**Supplementary Fig. 30 | Synthesis of Alcohol 2b and Aldehyde S39 from OTBDPS Protected Propanediol.**
Abbreviations: DMSO = dimethylsulfoxide, Bn = Benzyl, THF = tetrahydrofuran, CSA = camphorsulfonic acid, PMP = *para*-methoxyphenyl, DIBAL = diisobutylaluminum hydride, PMB = *para*-methoxybenzyl, TBSOTf = tert-butyldimethylsilyl trifluoromethanesulfonate.

**S31**

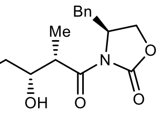


To a cold (-78 °C), stirred solution of $(COCl)_2$ (1.0 mL, 12 mmol, 1.2 eq.) in $CH_2Cl_2$ (35 mL) was added anhydrous DMSO (1.7 mL mL, 1.9 g, 24 mmol, 2.4 eq.) [Caution! $CO_2$ (g) evolved] over 5 minutes via syringe. Subsequently, 3-((tert-butyldiphenylsilyl)oxy)propan-1-ol (3.145 g, 10.0 mmol, 1.0 eq.) was added as a solution in $CH_2Cl_2$ (18 mL) over 15 min via syringe pump. After addition of the substrate, $Et_3N$ (7.0 mL, 5.1 g, 50 mmol, 5 eq.) was added via syringe pump over 5 min to give a cloudy white suspension. The reaction mixture was maintained at -78 °C for a further 2 h 15 min, at which point the $CO_2$(s)/Acetone (-78 °C) removed, and the reaction mixture warmed to room temperature. After a further

30 min, the starting material was consumed as monitored by TLC analysis. Then the reaction mixture was poured into a separatory funnel and washed with 10% aqueous $CuSO_4$ (1x 70 mL). The aqueous layer was back extracted with $Et_2O$ (3x 60 mL), dried ($MgSO_4$), filtered, and solvent was removed in vacuo to give the crude aldehyde **S30** as a yellow oil (3.02 g) which was used in the subsequent step without further purification. To a cold (0 °C), stirred solution of (*S*)-(+)-4-benzyl-3-propionyl-2-oxazolidinone (2.48 g, 10.6 mmol, 1.06 eq.) in anhydrous $CH_2Cl_2$ (27 mL) was added a 1M solution of $Bu_2BOTf$ (12 mL, 12 mmol, 1M in $CH_2Cl_2$, 1.2 eq.) in $CH_2Cl_2$ via syringe. After stirring for 15 min, $Et_3N$ (1.8 mL, 2.4 g, 12.9 mmol, 1.29 eq.) was added via syringe. After stirring for a further 50 min the ice bath was exchanged with a $CO_2$(s)/Acetone bath (-78 °C), and the crude aldehyde **S30** (*ca.* 3.02 g, *ca.* 10 mmol, 1.0 eq.) was added via syringe as a solution in anhydrous $CH_2Cl_2$ (13 mL). The reaction mixture was maintained at -78 °C for 1 h 50 min, at which point the $CO_2$(s)/Acetone bath was exchanged for an ice bath (0 °C). After 25 min of slowly warming to 0 °C, starting material was consumed as monitored by TLC analysis. After this time, the reaction was quenched by addition of a solution of 1:1 pH 7 phosphate buffer-MeOH (22 mL), followed by a solution of 3:1 30% aqueous $H_2O_2$-MeOH (20 mL). The reaction mixture was allowed to slowly warm to rt over 1 h with vigorous stirring. After this time, the reaction mixture was poured into a separatory funnel, the organic layer washed with brine (2 x 70 mL), and the aqueous layer back extracted with $Et_2O$ (3x 70 mL). The combined organic layers were washed with brine (2x 50 mL), dried ($MgSO_4$), filtered, and solvent was removed in vacuo. The crude product was purified via flash column chromatography (3:2 Hexanes/$Et_2O$). Appropriate fractions were pooled, and solvent was removed in vacuo to yield **S31** (4.07 g, 73%, 2 steps) as a viscous yellow oil. Spectral and optical rotation data agreed with the data previously reported in the literature.[6]

**Analytical Data for S31:**

$R_f$ = 0.40 (7:3 Hexanes:EtOAc)

$[\alpha]_D^{20}$ = +29.3° (c = 2.40, $CDCl_3$); lit. $[\alpha]_D^{20}$ = +30° (c = 0.34, $CHCl_3$)[6]

$^1$H NMR (500 MHz, $CDCl_3$) δ 7.71 – 7.65 (m, 4H), 7.47 – 7.37 (m, 6H), 7.37 – 7.32 (m, 2H), 7.31 – 7.26 (m, 1H), 7.24 – 7.17 (m, 2H), 4.70 (ddt, *J* = 9.5, 7.3, 3.1 Hz, 1H), 4.25 (dt, *J* = 7.1, 2.4 Hz, 1H), 4.23 – 4.16 (m, 2H), 3.92 – 3.80 (m, 3H), 3.46 (s, 1H), 3.28 (dd, *J* = 13.4, 3.3 Hz, 1H), 2.79 (dd, *J* = 13.4, 9.5 Hz, 1H), 1.82 (dddd, *J* = 14.3, 9.6, 7.2, 4.8 Hz, 1H), 1.68 (dddd, *J* = 13.9, 6.2, 4.6, 2.7 Hz, 1H), 1.30 (d, *J* = 7.0 Hz, 3H), 1.05 (s, 9H).

$^{13}$C NMR (126 MHz, $CDCl_3$) δ 176.64, 153.21, 135.70, 135.68, 135.32, 133.40, 133.32, 129.89, 129.57, 129.10, 127.88, 127.53, 70.86, 66.25, 62.65, 55.42, 42.95, 37.95, 36.09, 26.97, 19.23, 11.42.

HRMS (ESI): Anal. Calcd. for $C_{32}H_{40}NO_5Si^+$ $[M+H]^+$ 546.2670, found 546.2663

IR (neat): $\nu_{max}$ ($cm^{-1}$) = 3507 (br, OH), 3062 (w), 3029 (w), 2934 (m, CH), 2861 (m, CH), 1780 (s, C=O), 1691 (m), 1466 (m), 1385 (s). 1205 (s).

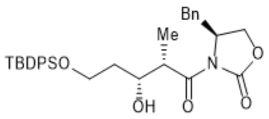
TBDPSO
Me
Bn
N
O
O
OH
O

**S31**

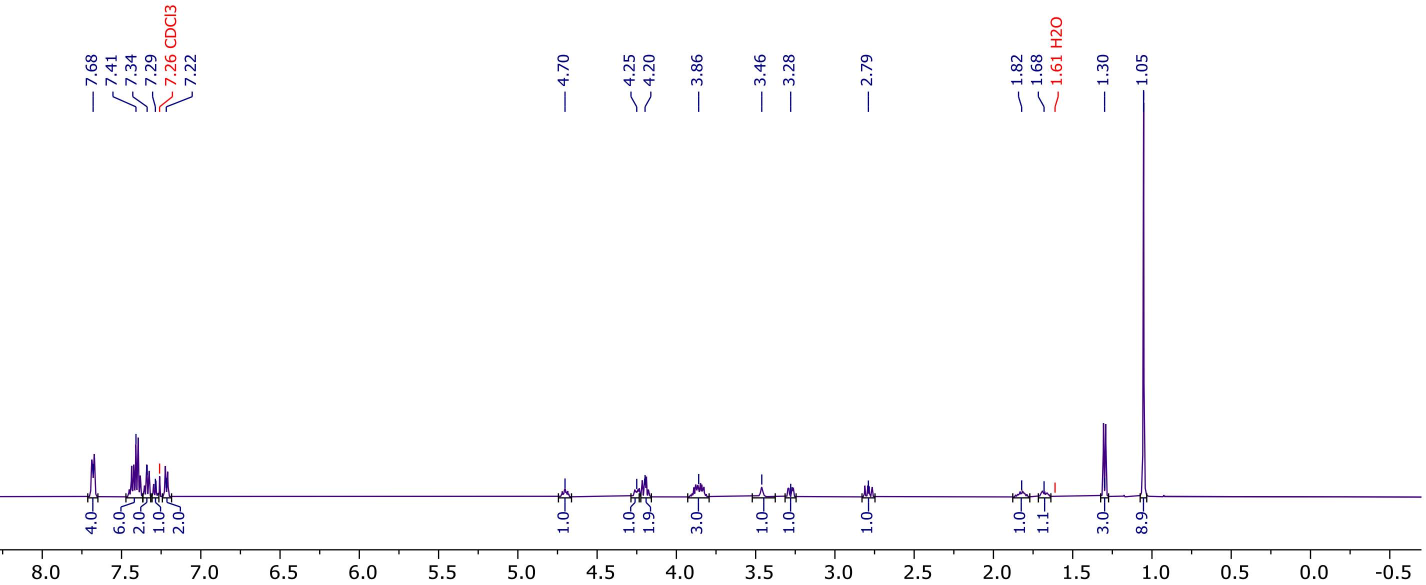
1H
CDCl3
500.14 MHz
7.68
7.41
7.34
7.29
7.26 CDCl3
7.22
4.70
4.25
4.20
3.86
3.46
3.28
2.79
1.82
1.68
1.61 H2O
1.30
1.05
4.0
6.0
2.0
1.0
2.0
1.0
1.0
1.9
3.0
1.0
1.0
1.0
1.0
1.1
3.0
8.9
10.0 9.5 9.0 8.5 8.0 7.5 7.0 6.5 6.0 5.5 5.0 4.5 4.0 3.5 3.0 2.5 2.0 1.5 1.0 0.5 0.0 -0.5

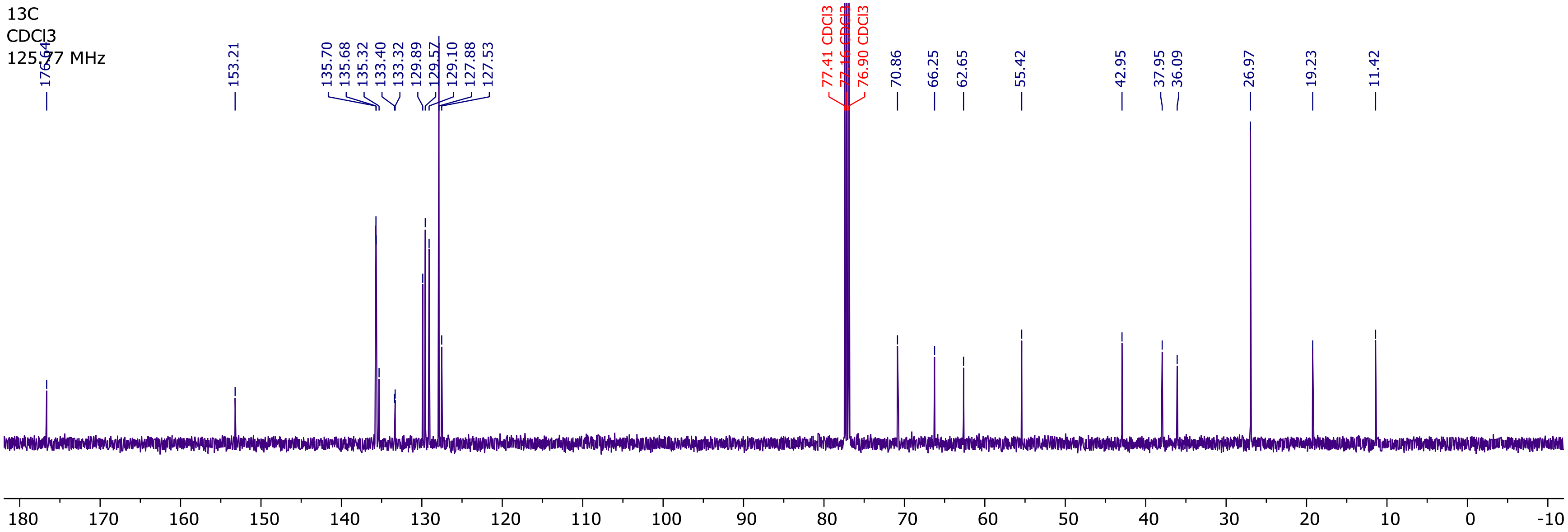
13C
CDCl3
125.77 MHz
176.64
153.21
135.70
135.68
135.32
133.40
133.32
129.89
129.57
129.10
127.88
127.53
77.41 CDCl3
77.16 CDCl3
76.90 CDCl3
70.86
66.25
62.65
55.42
42.95
37.95
36.09
26.97
19.23
11.42
180 170 160 150 140 130 120 110 100 90 80 70 60 50 40 30 20 10 0 -10

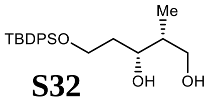


**S32**

Note: reaction was performed open to ambient atmosphere. To a rt, stirred solution of **S31 (**1.118 g**,** 2.04 mmol, 1 eq.) in MeOH:THF:$H_2O$ (1:29:10 (v/v/v), 20.5 mL) was added $NaBH_4$ (387.4, 10.2 mmol, 5 eq.) portion wise over 5 min [**Caution! $H_2$ gas evolved**]. The reaction mixture was stirred at rt for 19 h. After this time, starting material was consumed by TLC analysis, and the reaction mixture was poured onto EtOAc (50 mL). The organics were washed with $H_2O$ (2x 35 mL), and the aqueous layers were extracted with EtOAc (3x 50 mL). The combined organic layers were washed with brine (2x 50 mL), dried ($Na_2SO_4$), filtered, and solvent removed in vacuo. The crude product was purified via flash column chromatography (6.5:3.5 to 3:2 Hexanes/EtOAc). Appropriate fractions were pooled, and solvent was removed in vacuo to yield **S32** (619.4 mg, 81%) as a colorless oil. Spectral data agreed with the data previously reported in the literature.[4]

**Analytical Data for S32:**

$R_f$ = 0.30 (7:3 Hexanes:EtOAc)

$[\alpha]_D^{20}$ = -5.3° (c = 2.00, $CH_2Cl_2$); lit. $[\alpha]_D^{28}$ = -277 ° (c = 0.44, $CH_2Cl_2$)[4]

$^1$H NMR (500 MHz, $CDCl_3$) δ 7.72 – 7.64 (m, 4H), 7.49 – 7.36 (m, 6H), 4.14 (ddd, *J* = 10.1, 3.2, 2.0 Hz, 1H), 3.96 – 3.81 (m, 2H), 3.78 – 3.63 (m, 2H), 3.02 (br. s, 2H), 1.95 – 1.81 (m, 2H), 1.54 (dddd, *J* = 14.4, 4.3, 3.4, 2.0 Hz, 1H), 1.05 (s, 9H), 0.91 (d, *J* = 7.1 Hz, 3H).

$^{13}$C NMR (126 MHz, $CDCl_3$) δ 135.71, 135.70, 133.00, 132.88, 130.08, 130.06, 127.99, 75.72, 66.93, 64.35, 39.66, 34.60, 26.96, 19.17, 11.21.

HRMS (ESI): Anal. Calcd. for $C_{22}H_{33}O_3Si^+$ $[M+H]^+$ 373.2194, found 373.2187

IR (neat): $\nu_{max}$ $(cm^{-1})$ = 3662 (w, OH), 3379 (br, OH), 2963 (m, CH), 2934 (m, CH), 1469 (m), 1426 (m), 1388 (m), 1107 (s)

1H
CDCl3
500.14 MHz

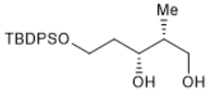


**S32**

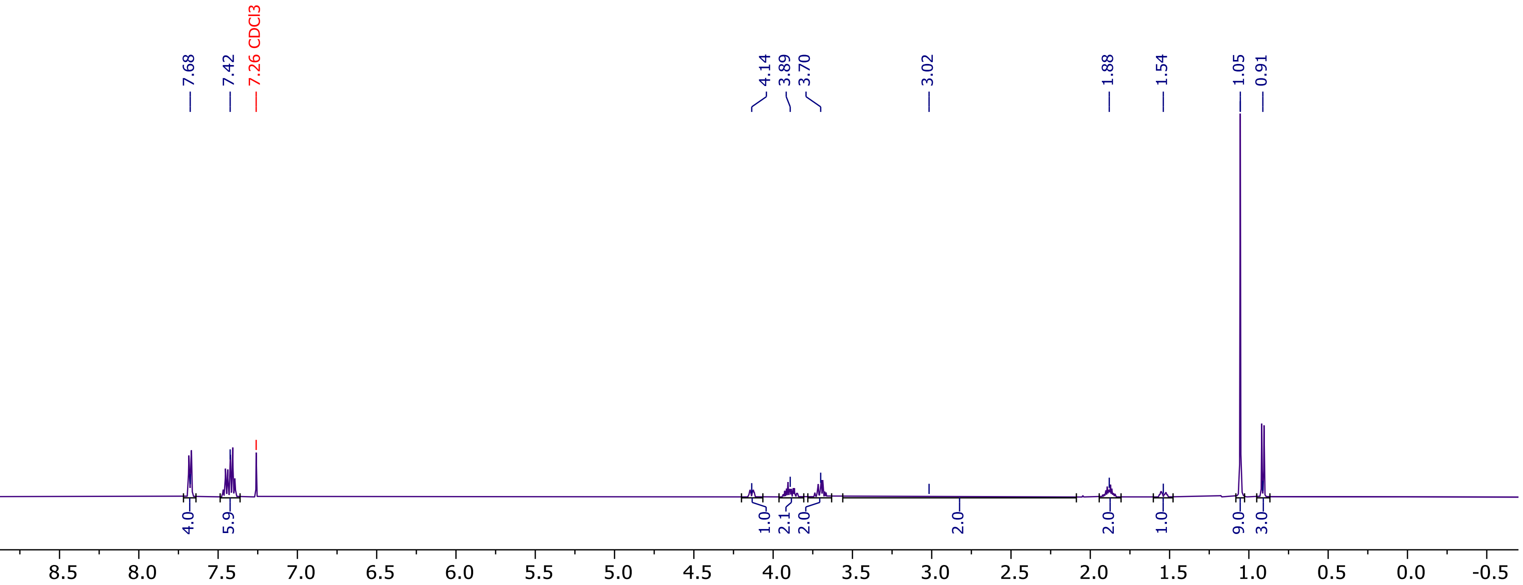


13C
CDCl3
125.77 MHz

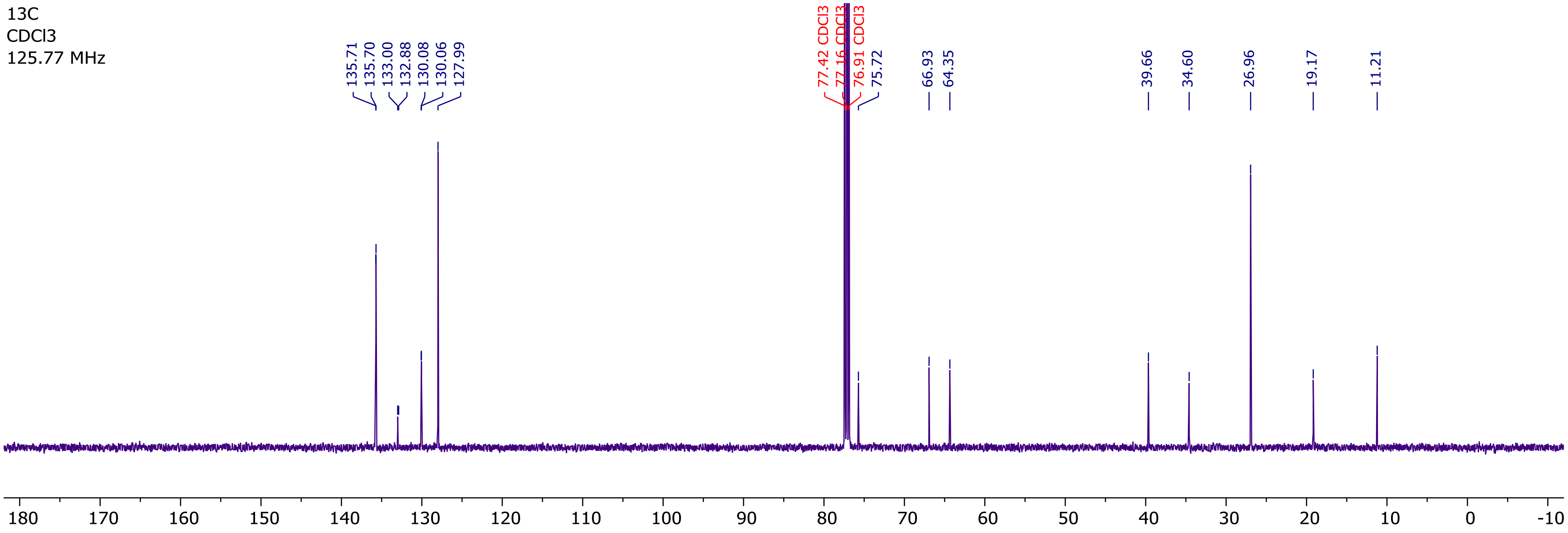

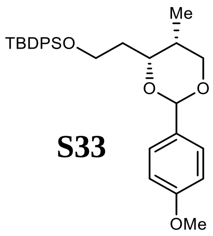


**S33**

To a rt, stirred solution of **S32** (818.7 mg, 2.20 mmol, 1 eq.) and *p*-methoxybenzaldehyde dimethylacetal (0.56 mL, 0.60 g, 3.3 mmol, 1.5 eq.) in $CH_2Cl_2$ (22 mL) was added CSA (50.9 mg, 0.22 mmol, 0.1 eq.) in one portion. After 17 h starting material was nearly consumed as monitored by TLC analysis, and the reaction was quenched with $Et_3N$ (0.2 mL, 0.15 g, 1.4 mmol, 1.4 eq.) and solvent removed in vacuo. The crude mixture was dissolved in EtOAc (10 mL), treated with a 10% aqueous solution of $NaHSO_3$ (10 mL), and stirred vigorously for 45 min. After this time, the crude mixture was poured onto $H_2O$ (50 mL) in a separatory funnel and extracted with EtOAc (4x 40 mL). The combined organic layers were washed with brine (1x 50 mL), dried ($Na_2SO_4$), and solvent was removed in vacuo. The crude product was purified via flash column chromatography (19:1 to 93:7 Hexanes/EtOAc). Appropriate fractions were pooled and solvent removed in vacuo to yield **S33** (903 mg, 84%) as a colorless oil. Note: product was highly sensitive to acid in $CHCl_3$ even if the $CHCl_3$ was shaken with $K_2CO_3$.

**Analytical Data for S33:**

$R_f$ = 0.52 (4:1 Hexanes/EtOAc)

$[\alpha]_D^{20}$ = +18.2° (c = 2.50, $CD_2Cl_2$)

$^1$H NMR (500 MHz, $CD_2Cl_2$) δ 7.67 (ddt, *J* = 7.3, 5.9, 1.5 Hz, 4H), 7.47 – 7.30 (m, 8H), 6.92 – 6.83 (m, 2H), 5.42 (s, 1H), 4.21 (ddd, *J* = 8.9, 4.3, 2.4 Hz, 1H), 4.07 (dd, *J* = 11.1, 2.6 Hz, 1H), 3.96 (dd, *J* = 11.1, 1.4 Hz, 1H), 3.85 (ddd, *J* = 10.1, 8.5, 4.9 Hz, 1H), 3.80 (s, 3H), 3.77 (ddd, *J* = 10.4, 5.9, 4.8 Hz, 1H), 1.84 (ddt, *J* = 13.9, 8.8, 4.9 Hz, 1H), 1.71 (dddd, *J* = 14.2, 8.6, 5.9, 4.3 Hz, 1H), 1.57 – 1.48 (m, 1H), 1.14 (d, *J* = 7.0 Hz, 3H), 1.06 (s, 9H).

$^{13}$C NMR (126 MHz, $CD_2Cl_2$) δ 160.27, 135.93, 134.37, 134.30, 132.24, 129.99, 129.97, 128.05, 128.03, 127.80, 113.72, 101.97, 76.39, 74.12, 60.46, 55.64, 36.28, 32.43, 27.05, 19.50, 11.45.

HRMS (ESI): Anal. Calcd. for $C_{30}H_{39}O_4Si^+$ $[M+H]^+$ 491.2612, found 491.2608

IR (neat): $\nu_{max}\ (cm^{-1})$ = 3070 (w, C=*CH*), 2958 (m, CH), 2932 (m, CH), 1616 (m, C=C), 1517 (m), 1465 (m), 1248 (s)

**S33**

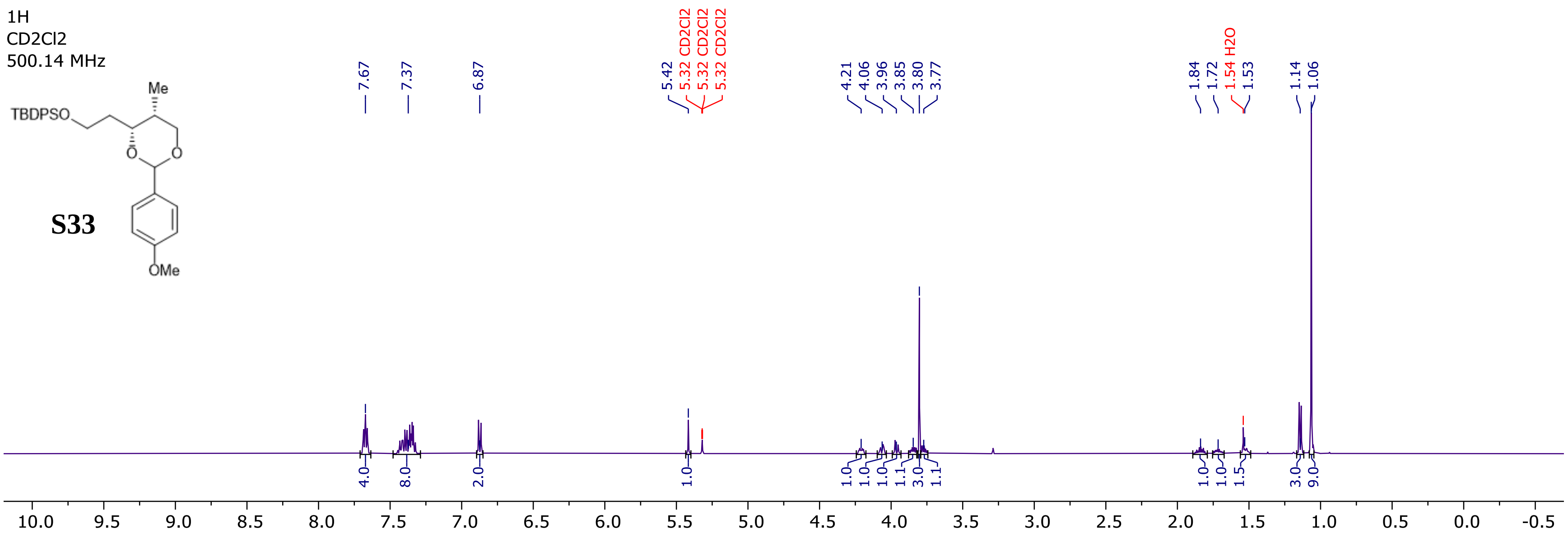
1H
CD2Cl2
500.14 MHz
Me
TBDPSO
O
O
OMe
7.67
7.37
6.87
5.42
5.32 CD2Cl2
5.32 CD2Cl2
5.32 CD2Cl2
4.21
4.06
3.96
3.85
3.80
3.77
1.84
1.72
1.54 H2O
1.53
1.14
1.06
4.0
8.0
2.0
1.0
1.0
1.0
1.0
1.1
3.0
1.1
1.0
1.0
1.5
3.0
9.0
10.0
9.5
9.0
8.5
8.0
7.5
7.0
6.5
6.0
5.5
5.0
4.5
4.0
3.5
3.0
2.5
2.0
1.5
1.0
0.5
0.0
-0.5

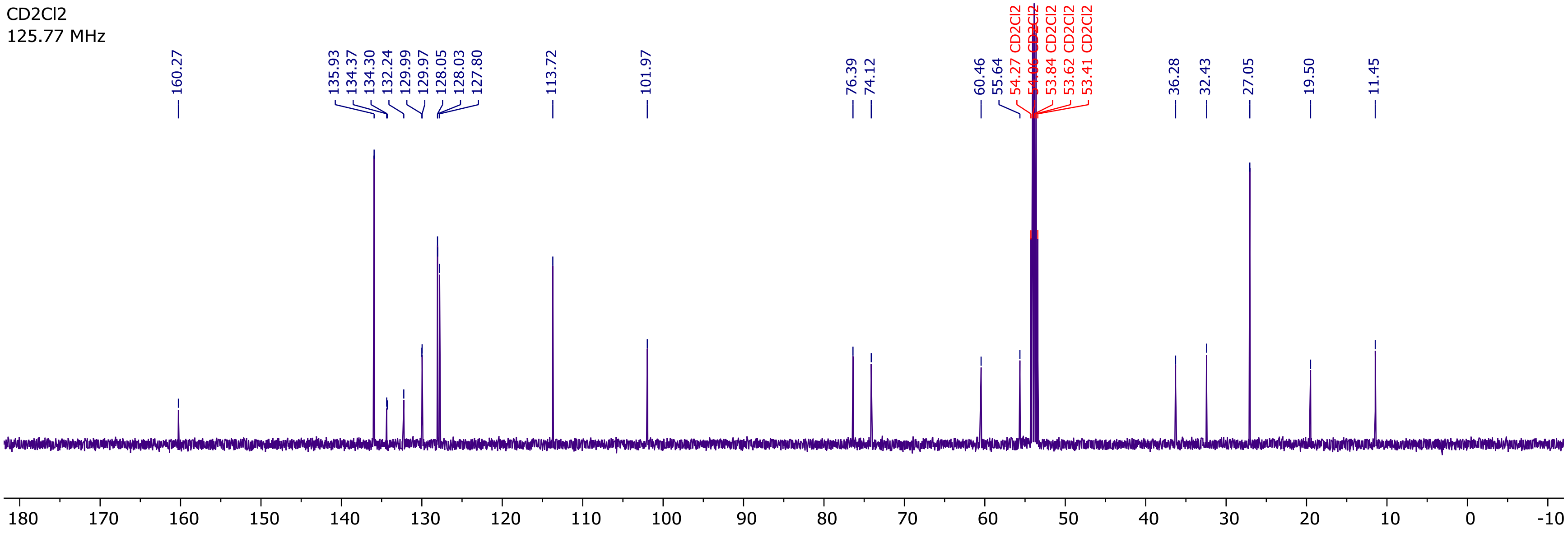
CD2Cl2
125.77 MHz
160.27
135.93
134.37
134.30
132.24
129.99
129.97
128.05
128.03
127.80
113.72
101.97
76.39
74.12
60.46
55.64
54.27 CD2Cl2
54.06 CD2Cl2
53.84 CD2Cl2
53.62 CD2Cl2
53.41 CD2Cl2
36.28
32.43
27.05
19.50
11.45
180
170
160
150
140
130
120
110
100
90
80
70
60
50
40
30
20
10
0
-10

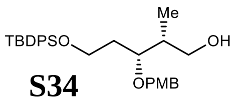


**S34**

This procedure was adapted from the literature.[4] To a cold (0 °C), stirred solution of **S33** (836.1 mg, 1.70 mmol, 1 eq.) in $CH_2Cl_2$ (13.5 mL) was added DIBAL-H (3.4 mL, 3.4 mmol, 1M in $CH_2Cl_2$, 2 eq.) via syringe and the reaction mixture was allowed to warm slowly to rt. After 2 h starting material was consumed as monitored by TLC analysis, and the reaction was quenched by addition of a saturated aqueous solution of Rochelle's salt (25 mL). The biphasic mixture was vigorously stirred for 4 hours. After this time, the mixture was transferred to a separatory funnel, the organic layer drained, and the aqueous layer extracted with $CH_2Cl_2$ (2x 60 mL). The combined organic layers were washed with brine (1x 100 mL), dried ($Na_2SO_4$), filtered, and solvent was removed in vacuo. The crude product was purified via flash column chromatography (3:2 to 1:1 Hexanes/EtOAc). Appropriate fractions were pooled, and the solvent was removed in vacuo to yield **S34** (707 mg, 84%) as a colorless oil. Spectral data agreed with the data previously reported in the literature.[4]

**Analytical Data for S34:**

$R_f$ = 0.36 (3:1 Hexanes/EtOAc)

$[\alpha]_D^{20}$ = +7.0° (c = 3.48, $CH_2Cl_2$)

$^1$H NMR (500 MHz, $CDCl_3$) δ 7.68 (ddt, *J* = 6.9, 5.5, 1.5 Hz, 4H), 7.48 – 7.42 (m, 2H), 7.42 – 7.36 (m, 4H), 7.22 – 7.16 (m, 2H), 6.88 – 6.81 (m, 2H), 4.52 (d, *J* = 11.0 Hz, 1H), 4.41 (d, *J* = 11.0 Hz, 1H), 3.80 (s, 3H), 3.83 – 3.74 (m, 3H), 3.66 (dd, *J* = 10.8, 8.2 Hz, 1H), 3.54 (dd, *J* = 10.8, 4.7 Hz, 1H), 2.53 (br. s, 1H), 2.18 – 2.07 (m, 1H), 1.85 – 1.72 (m, 2H), 1.08 (s, 9H), 0.86 (d, *J* = 7.1 Hz, 3H).

$^{13}$C NMR (126 MHz, $CDCl_3$) δ 159.34, 135.71, 133.95, 133.90, 130.55, 129.78, 129.63, 127.81, 127.79, 113.93, 79.01, 71.69, 66.12, 60.82, 55.37, 36.85, 32.90, 27.02, 19.32, 12.30.

HRMS (ESI): Anal. Calcd. for $C_{30}H_{41}O_4Si^+$ $[M+H]^+$ 493.2769, found 493.2785

IR (neat): $\nu_{max}$ $(cm^{-1})$ = 3445 (br, OH), 3070 (w, C=*CH*), 2956 (m, CH), 2932 (m, CH), 1613 (m, C=C), 1513 (m), 1467 (m), 1427, 1248 (s).

CDCl3
500.14 MHz

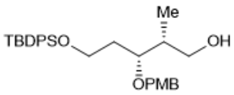


**S34**

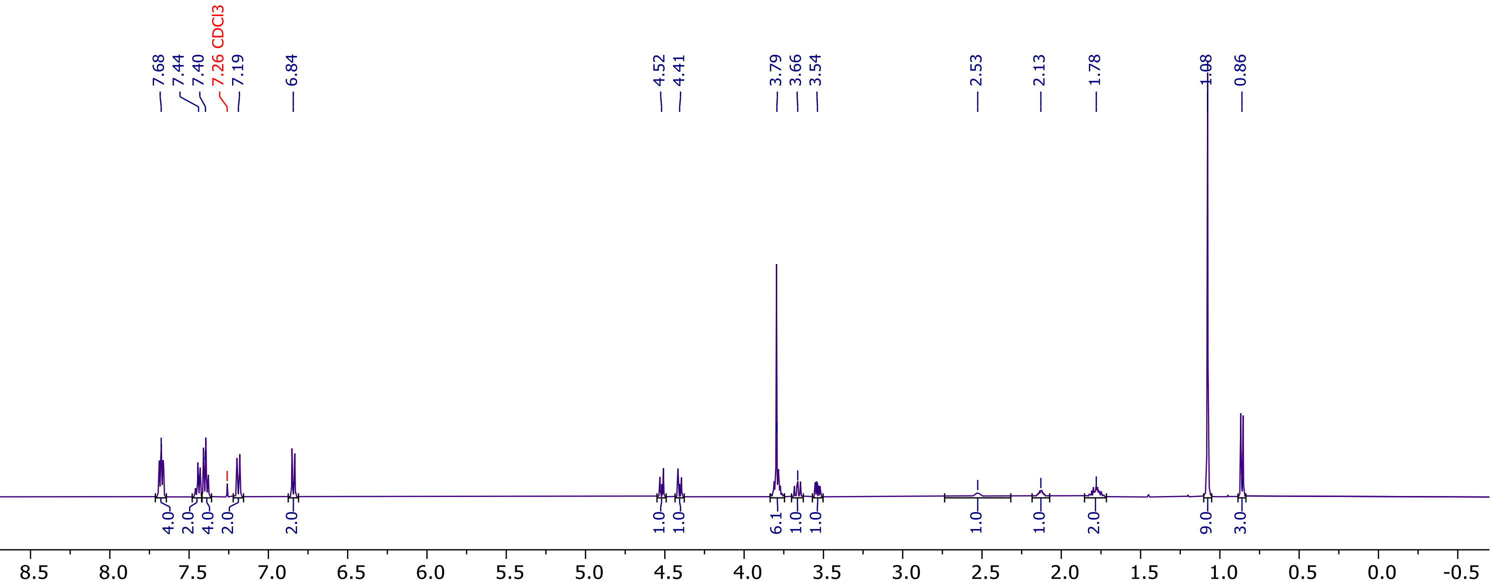


CDCl3
125.77 MHz

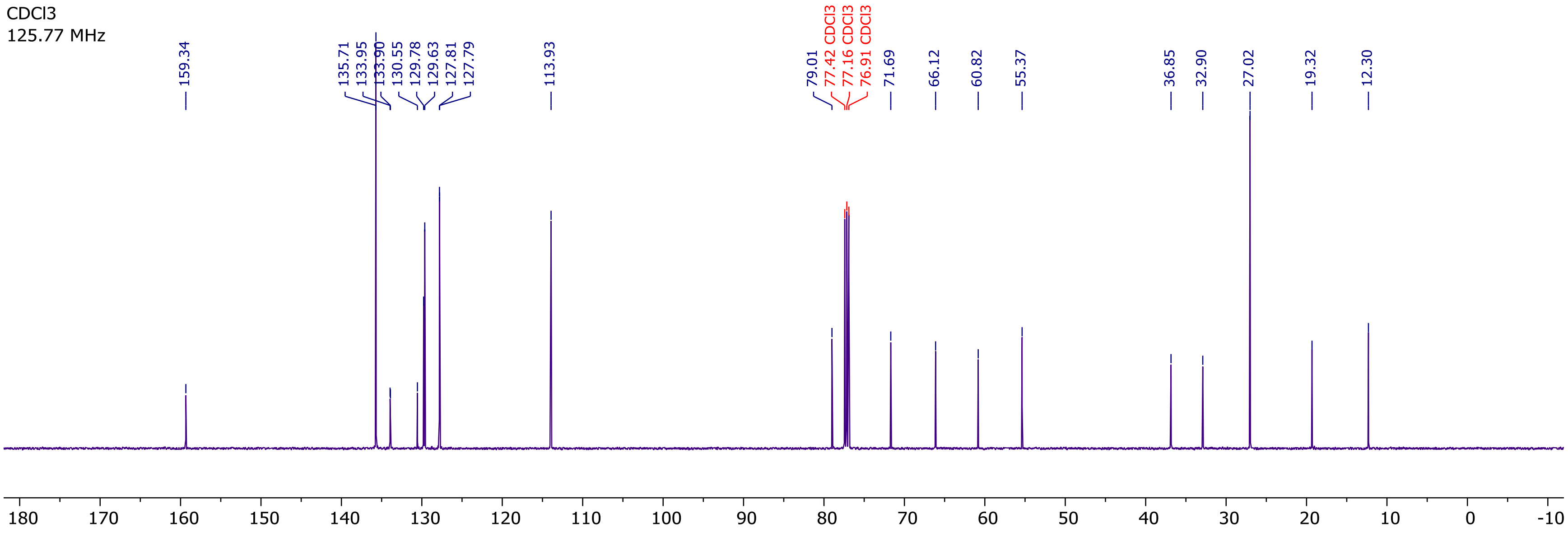

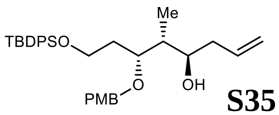


**S35**

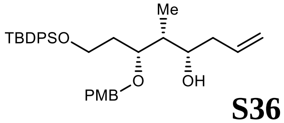


**S36**

To a cold (-78 °C), stirred solution of $(COCl)_2$ (0.13 mL, 1.5 mmol, 1.3 eq.) in $CH_2Cl_2$ (6 mL) was added anhydrous DMSO (0.21 mL, 2.96 mmol, 2.5 eq.) [**Caution! $CO_2$ (g) evolved**] over 5 minutes via syringe. Subsequently, **S34** (594.0 mg, 1.206 mmol, 1.0 eq.) was added as a solution in $CH_2Cl_2$ (1 mL + 1 mL rinse). The reaction mixture was maintained at -78 °C for 5 min, after which time $Et_3N$ (0.84 mL, 0.61 g, 6.0 mmol, 5.0 eq.) was added via syringe over 5 min to give a cloudy white suspension. The reaction mixture was allowed to slowly warm to rt over 2 h 10 min. After this time, the starting material was consumed as monitored by TLC analysis. Then the reaction mixture was poured into a separatory funnel and washed with a 10% aqueous solution of $CuSO_4$ (2x 10 mL). The organic layer was drained, and the combined aqueous layers were extracted with $Et_2O$ (3x 30 mL). The combined organic layers were washed with brine (1x 30 mL), dried ($MgSO_4$), filtered, and solvent was removed in vacuo to give the crude aldehyde (568 mg, 96%) as a colorless oil which was used immediately in the next step without further purification. To a cold (-78 °C), stirred solution of crude aldehyde (568 mg, 1.16 mmol, 1.0 eq.) in $Et_2O$ (4.4 mL) was added allyl-MgBr (1.0 M in $Et_2O$, 1.45 mL, 1.45 mmol, 1.25 eq.) via syringe. The reaction mixture was maintained at -78 °C for 1 h. After this time further allyl-MgBr was added (1.0 M in $Et_2O$, 0.1 mL, 0.1 mmol, 0.08 eq.) via syringe and the reaction mixture was warmed to -20 °C. After a further 15 min, starting material was consumed as monitored by TLC analysis, and the reaction was quenched by addition of saturated aqueous $NH_4Cl$ (6 mL) via syringe. Then the reaction mixture was warmed to rt and poured into a separatory funnel. The organic layer was separated, and the aqueous layer was extracted with $Et_2O$ (3x 30 mL). The combined organic layers were dried ($MgSO_4$), filtered, and solvent was removed in vacuo. The crude product was purified via flash column chromatography (8.5:1.5 to 4:1 Hexanes/$Et_2O$). Appropriate fractions were pooled, and solvent was removed in vacuo to yield **S35** (91.5 mg, 14%, 2 steps), **S36** (321.1 mg, 50%, 2 steps), and mixed **S35** and **S36** (63.0 mg, 10%) as colorless oils. Spectral data agreed with the data previously reported in the literature for both **S35** and **S36**.[4]

**Analytical Data for S35:**

$R_f$ = 0.42 (4:1 Hexanes/$Et_2O$)

$[\alpha]_D^{20}$ = +1.5 ° (c = 2.95, $CDCl_3$)

$^1$H NMR (500 MHz, $CDCl_3$) δ 7.66 (ddd, *J* = 8.0, 4.5, 1.5 Hz, 4H), 7.47 – 7.42 (m, 2H), 7.41 – 7.35 (m, 4H), 7.20 – 7.14 (m, 2H), 6.85 – 6.80 (m, 2H), 5.94 (dddd, *J* = 16.6, 10.2, 7.8, 6.2 Hz, 1H), 5.16 – 5.08 (m, 2H), 4.57 (d, *J* = 11.0 Hz, 1H), 4.37 (d, *J* = 11.0 Hz, 1H), 3.87 (dt, *J* = 8.7, 3.6 Hz, 1H), 3.79 (s, 3H), 3.76 (td, *J* = 4.7, 1.5 Hz, 2H), 3.68 (ddd, *J* = 8.6, 7.2, 3.7 Hz, 1H), 2.41 – 2.32 (m, 1H), 2.20 – 2.10 (m, 1H), 2.01 – 1.92 (m, 1H), 1.88 – 1.72 (m, 2H), 1.06 (s, 9H), 0.85 (d, *J* = 7.0 Hz, 3H).

$^{13}$C NMR (126 MHz, $CDCl_3$) δ 159.38, 135.70, 135.35, 133.97, 133.90, 130.24, 129.78, 129.68, 127.81, 117.05, 113.94, 79.38, 73.27, 71.57, 60.79, 55.38, 39.79, 38.42, 32.88, 27.02, 19.32, 12.88.

HRMS (ESI): Anal. Calcd. for $C_{33}H_{45}O_4Si^+$ $[M+H]^+$ 533.3082, found 533.3103

IR (neat): $\nu_{max}\ (cm^{-1})$ = 3468 (br, OH), 3071 (w, C=*CH*), 2933 (m, CH), 2892 (m, CH), 2959 (m, CH), 1613 (m, C=C), 1513 (m), 1467 (m), 1428 (m), 1249 (s).

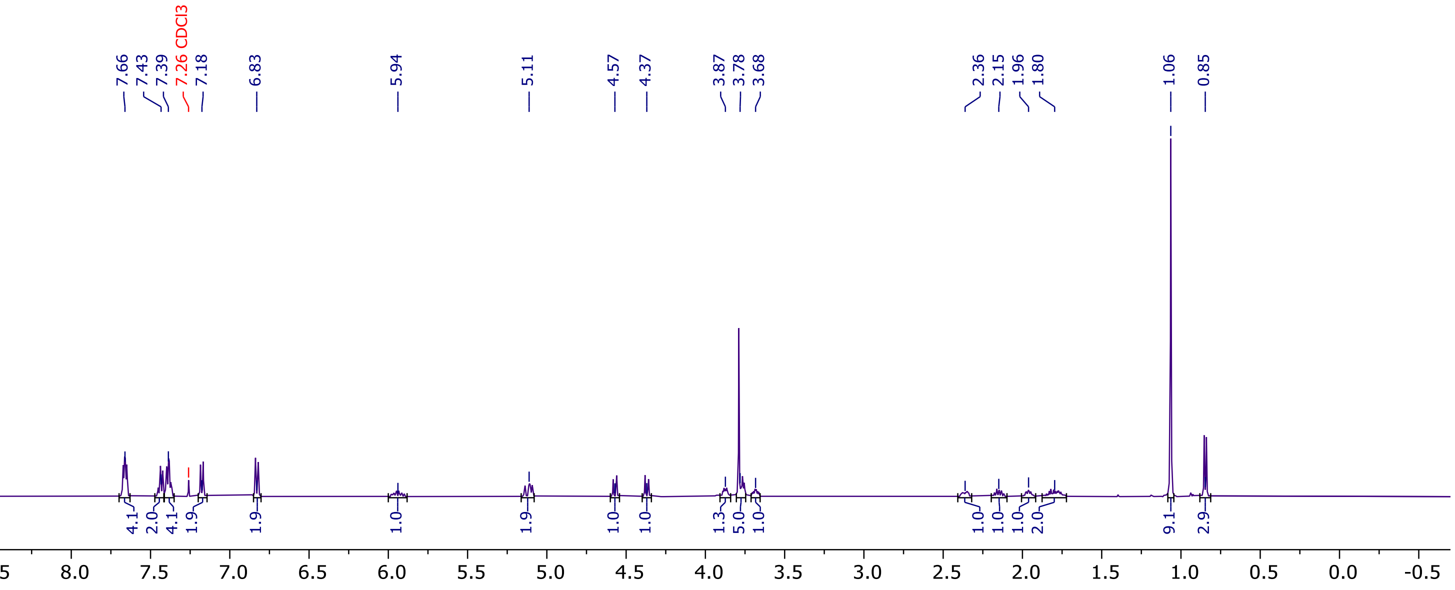
1H
CDCl3
500.14 MHz
7.66
7.43
7.39
7.26 CDCl3
7.18
6.83
5.94
5.11
4.57
4.37
3.87
3.78
3.68
2.36
2.15
1.96
1.80
1.06
0.85
4.1
2.0
4.1
1.9
1.9
1.0
1.9
1.0
1.0
1.3
5.0
1.0
1.0
1.0
1.0
2.0
9.1
2.9
10.0
9.5
9.0
8.5
8.0
7.5
7.0
6.5
6.0
5.5
5.0
4.5
4.0
3.5
3.0
2.5
2.0
1.5
1.0
0.5
0.0
-0.5

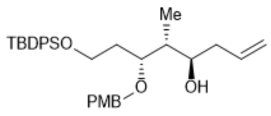
TBDPSO
Me
PMB
O
OH

**S35**

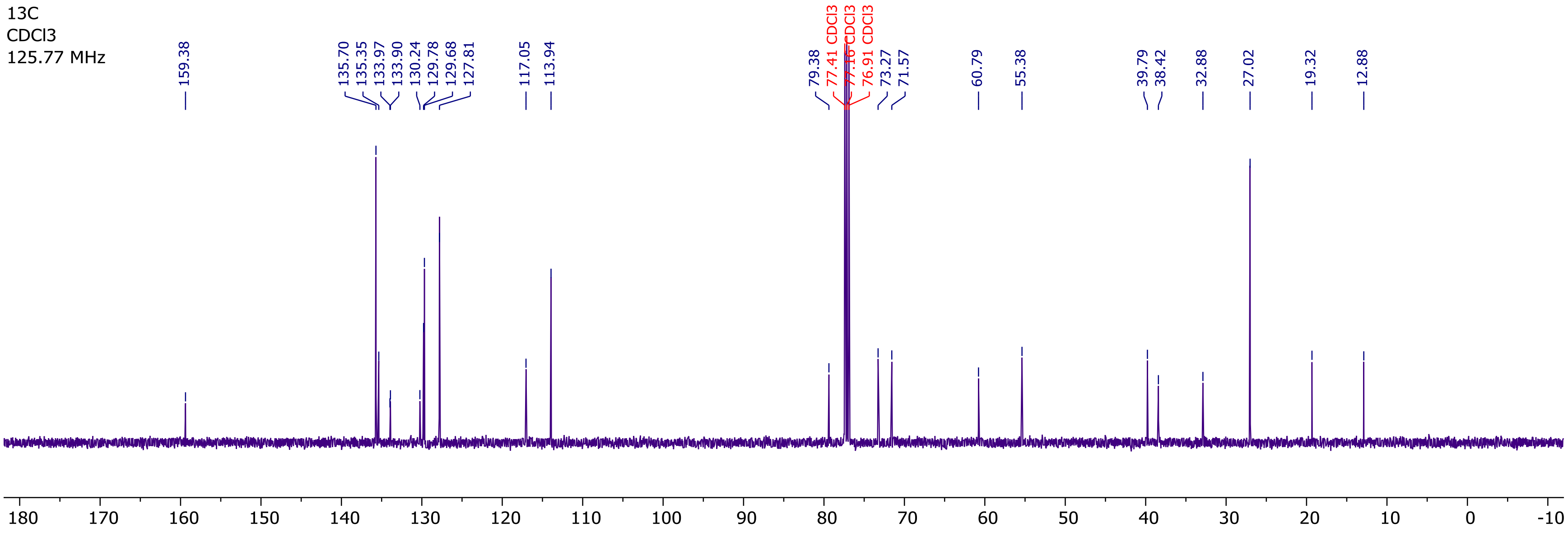
13C
CDCl3
125.77 MHz
159.38
135.70
135.35
133.97
133.90
130.24
129.78
129.68
127.81
117.05
113.94
79.38
77.41 CDCl3
77.16 CDCl3
76.91 CDCl3
73.27
71.57
60.79
55.38
39.79
38.42
32.88
27.02
19.32
12.88
180
170
160
150
140
130
120
110
100
90
80
70
60
50
40
30
20
10
0
-10

**Analytical Data for S36:**

$R_f$ = 0.34 (4:1 Hexanes/$Et_2O$)

$[\alpha]_D^{20}$ = -23.4° (c = 2.41, $CH_2Cl_2$); lit. $[\alpha]_D^{20}$ = -43° (c = 2.76, $CH_2Cl_2$)[4]

$^1$H NMR (600 MHz, $CDCl_3$) δ 7.66 (tt, *J* = 7.1, 1.4 Hz, 4H), 7.48 – 7.42 (m, 2H), 7.40 (tt, *J* = 8.1, 1.1 Hz, 4H), 7.23 – 7.18 (m, 2H), 6.88 – 6.82 (m, 2H), 5.79 (ddt, *J* = 17.2, 10.1, 7.1 Hz, 1H), 5.10 (dq, *J* = 17.1, 1.6 Hz, 1H), 5.07 (ddt, *J* = 10.2, 2.1, 1.1 Hz, 1H), 4.53 (d, *J* = 10.9 Hz, 1H), 4.36 (d, *J* = 10.9 Hz, 1H), 3.82 (ddt, *J* = 7.7, 5.4, 2.4 Hz, 2H), 3.80 (s, 3H), 3.75 (dt, *J* = 10.4, 6.0 Hz, 1H), 3.69 (ddd, *J* = 10.4, 7.0, 5.6 Hz, 1H), 3.20 (s, 1H), 2.33 – 2.24 (m, 1H), 2.18 – 2.10 (m, 1H), 1.99 (ddt, *J* = 13.9, 7.0, 5.6 Hz, 1H), 1.77 (ddt, *J* = 13.9, 7.8, 5.9 Hz, 1H), 1.71 – 1.65 (m, 1H), 1.06 (s, 9H), 0.91 (d, *J* = 7.1 Hz, 3H).

$^{13}$C NMR (151 MHz, $CDCl_3$) δ 159.34, 135.69, 135.63, 133.73, 130.23, 129.85, 129.61, 127.84, 127.83, 117.22, 113.99, 81.31, 74.83, 71.00, 60.90, 55.39, 39.83, 38.86, 33.56, 26.98, 19.30, 6.19.

HRMS (ESI): Anal. Calcd. for $C_{33}H_{45}O_4Si^+$ $[M+H]^+$ 533.3082, found 533.3065

IR (neat): $\nu_{max}$ $(cm^{-1})$ = 3477 (br, OH), 3070 (w, C=*CH*), 2934 (m, CH), 2858 (m, CH), 1614 (m, C=C), 1513 (m), 1249 (s).

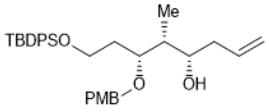
TBDPSO
Me
PMB
O
OH

**S36**

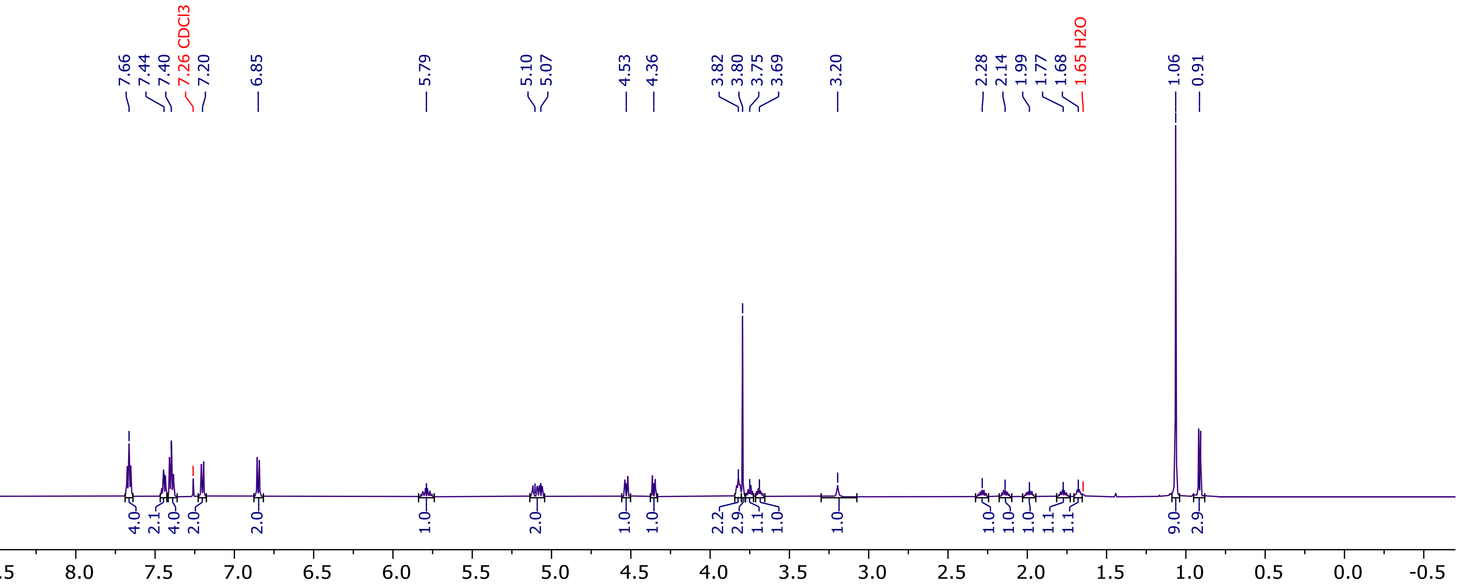
CDCl3
600.13 MHz
7.66
7.44
7.40
7.26 CDCl3
7.20
6.85
5.79
5.10
5.07
4.53
4.36
3.82
3.80
3.75
3.69
3.20
2.28
2.14
1.99
1.77
1.68
1.65 H2O
1.06
0.91
4.0
2.1
4.0
2.0
2.0
1.0
2.0
1.0
1.0
2.2
2.9
1.1
1.0
1.0
1.0
1.0
1.0
1.1
1.1
9.0
2.9
10.0
9.5
9.0
8.5
8.0
7.5
7.0
6.5
6.0
5.5
5.0
4.5
4.0
3.5
3.0
2.5
2.0
1.5
1.0
0.5
0.0
-0.5

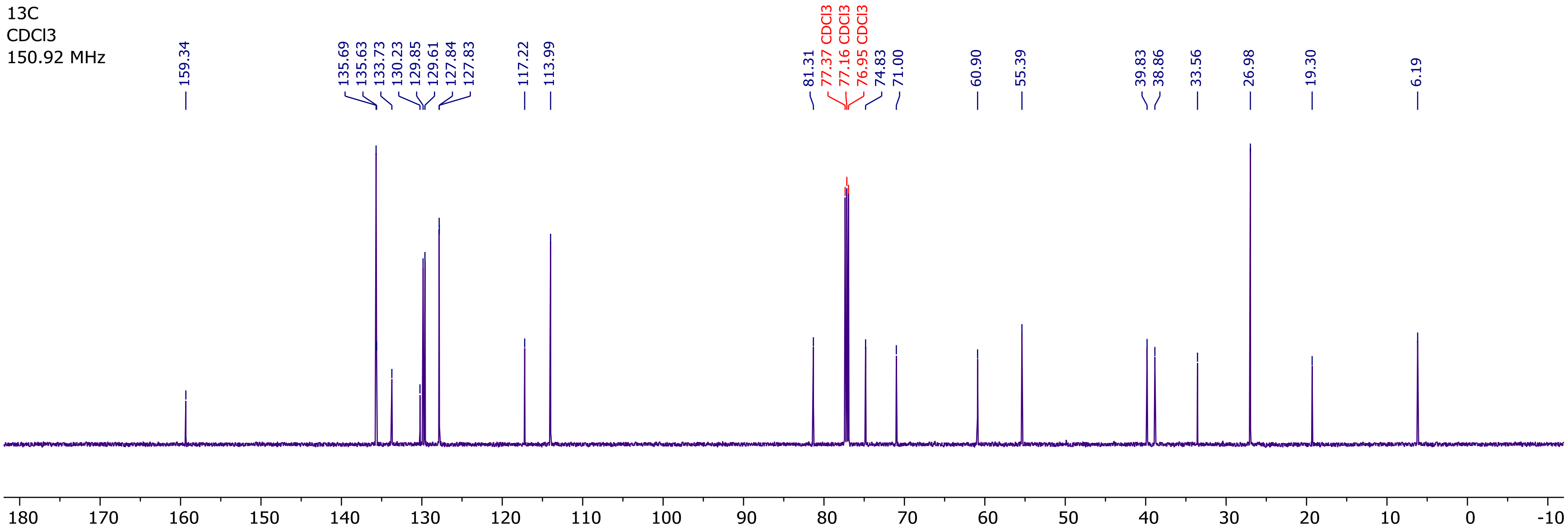
13C
CDCl3
150.92 MHz
159.34
135.69
135.63
133.73
130.23
129.85
129.61
127.84
127.83
117.22
113.99
81.31
77.37 CDCl3
77.16 CDCl3
76.95 CDCl3
74.83
71.00
60.90
55.39
39.83
38.86
33.56
26.98
19.30
6.19
180
170
160
150
140
130
120
110
100
90
80
70
60
50
40
30
20
10
0
-10

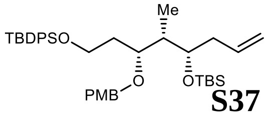

**S37**

To a cold (-20 °C), stirred solution of **S36** (1.174 g, 2.20 mmol, 1 eq.) in $CH_2Cl_2$ (15 mL) was added in sequence 2,6-lutidine (0.76 mL, 0.70 g, 6.6 mmol, 3 eq.) and TBSOTf (0.76 mL, 0.87 g, 3.3 mmol, 1.5 eq.). The reaction mixture was allowed to slowly warm to rt over 19 h. After this time starting material was consumed as monitored by TLC analysis. Then, the reaction mixture was transferred to a separatory funnel diluted with $CH_2Cl_2$ (30 mL), washed with brine (1x 30 mL), and the aqueous layer extracted with further $CH_2Cl_2$ (2x 30 mL). The combined organic layers were dried ($Na_2SO_4$), filtered, and solvent removed in vacuo. The crude product was passed through a short silica gel plug eluting with 19:1 Hexanes/$Et_2O$. The solvent was removed in vacuo to yield **S37** (1.38 g, 97%) as a colorless oil which was used immediately in the next reaction without further purification. A small quantity from a different batch of this material was purified via flash chromatography (97.5:2.5 to 97:3 Hexanes/$Et_2O$) for analytical purposes.

**Analytical Data for S37:**

$R_f$ = 0.32 (96.5:3.5 Hexanes/$Et_2O$)

$[\alpha]_D^{20}$ = +1.0 ° (c = 1.44, $CDCl_3$)

$^1H$ NMR (500 MHz, $CDCl_3$) δ 7.67 (ddt, *J* = 6.6, 5.1, 1.5 Hz, 4H), 7.46 – 7.40 (m, 2H), 7.40 – 7.34 (m, 4H), 7.19 – 7.12 (m, 2H), 6.87 – 6.79 (m, 2H), 5.72 (ddt, *J* = 17.5, 10.5, 7.2 Hz, 1H), 5.01 – 4.97 (m, 1H), 4.96 (t, *J* = 1.1 Hz, 1H), 4.37 (q, *J* = 11.0 Hz, 2H), 3.79 (s, 3H), 3.78 – 3.69 (m, 3H), 3.58 (dt, *J* = 7.4, 4.9 Hz, 1H), 2.32 – 2.16 (m, 2H), 1.89 (dtd, *J* = 12.2, 7.5, 4.7 Hz, 1H), 1.78 (qd, *J* = 7.1, 5.2 Hz, 1H), 1.74 – 1.67 (m, 1H), 1.06 (s, 9H), 0.91 (d, *J* = 6.9 Hz, 3H), 0.88 (s, 9H), 0.05 (s, 3H), 0.03 (s, 3H).

$^{13}C$ NMR (126 MHz, $CDCl_3$) δ 159.15, 135.74, 135.72, 135.44, 134.11, 134.08, 131.29, 129.71, 129.47, 127.78, 127.77, 116.93, 113.82, 76.86, 72.63, 71.57, 61.07, 55.41, 40.89, 39.73, 35.09, 27.06, 26.11, 19.32, 18.28, 10.45, -3.69, -4.25.

HRMS (ESI): Anal. Calcd. for $C_{39}H_{59}O_4Si_2^+$ $[M+H]^+$ 647.3946, found 647.3931

IR (neat): $\nu_{max}\ (cm^{-1})$ = 3072 (w, C=*CH*), 2955 (m, CH), 2932 (m, CH), 2858 (m, CH), 1614 (C=C, w), 1514 (m,) 1467 (m), 1428 (m), 1250 (s).

1H
CDCl3
500.14 MHz

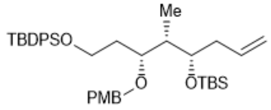


**S37**

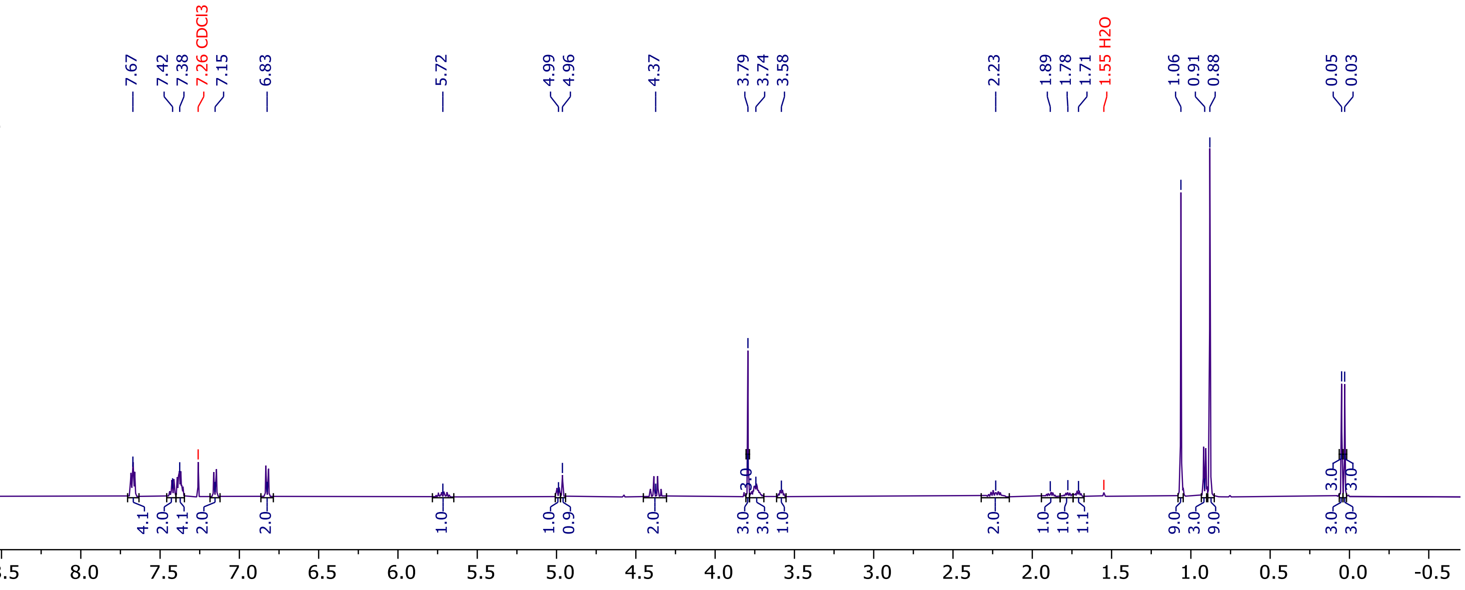


13C
CDCl3
125.77 MHz

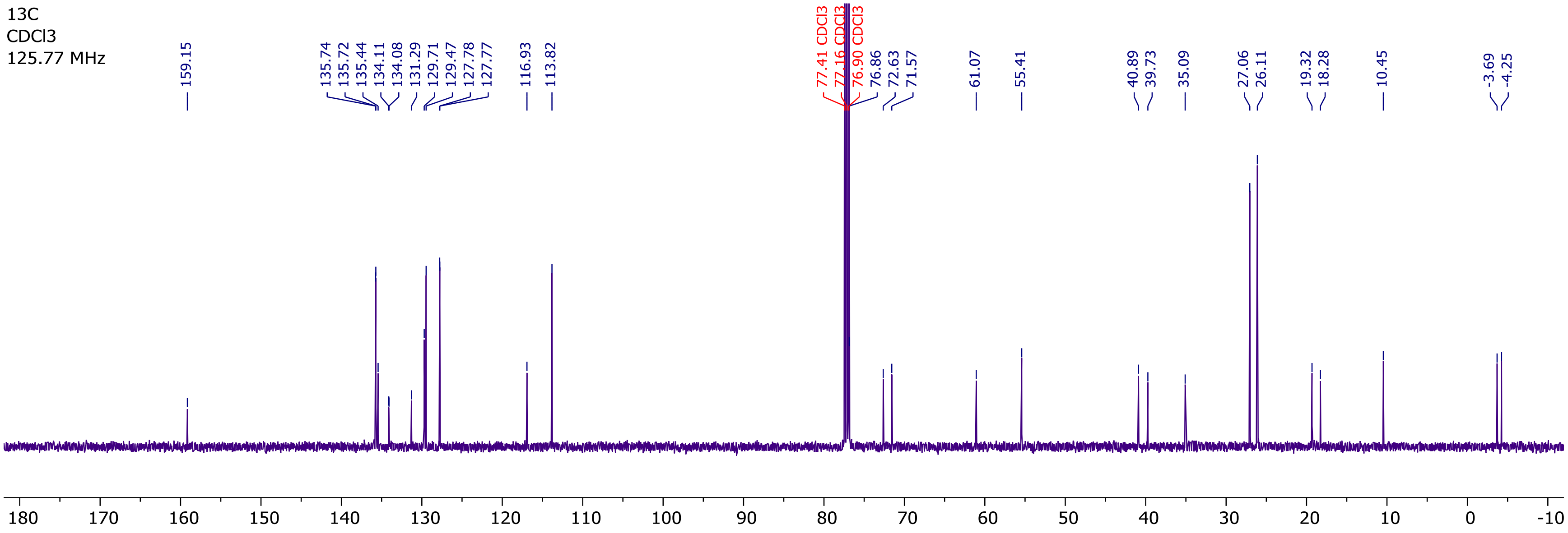

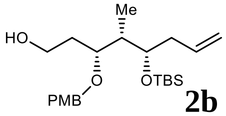

**2b**

Reaction was conducted in a sealed polypropylene tube. To a warm (60 °C), stirred solution of **S37** (1.38 g, *ca.* 2.1 mmol, 1 eq.) in MeOH (14.3 mL) was added $NH_4F$ (394.4 mg, 10.6 mmol, 5 eq.). After 8 h 15 min, starting material was consumed as monitored by TLC analysis. The reaction mixture was poured onto a saturated aqueous solution of $NaHCO_3$ (20 mL) and concentrated to a small volume in vacuo. The concentrate was transferred to a separatory funnel and extracted with $CH_2Cl_2$ (3x 20 mL). The combined organic layers were washed with brine (1x 20 mL), dried ($Na_2SO_4$), filtered, and solvent removed in vacuo. The crude product was purified via flash column chromatography (19:1 to 6.5:3.5 Hexanes:$Et_2O$). Appropriate fractions were pooled, and solvent was removed in vacuo to yield **2b** (640 mg, 75%, or 71% 2 steps) as a colorless oil.

**Analytical Data for 2b:**

$R_f$ = 0.22 (6.5:3.5 Hexanes/$Et_2O$)

$[\alpha]_D^{20}$ = +13.5 ° (c = 1.41, $CDCl_3$)

$^1$H NMR (600 MHz, $CDCl_3$) δ 7.29 – 7.24 (m, 2H), 6.92 – 6.84 (m, 2H), 5.74 (ddt, *J* = 17.5, 10.4, 7.2 Hz, 1H), 5.07 – 5.04 (m, 1H), 5.04 – 5.01 (m, 1H), 4.46 (s, 2H), 3.80 (s, 3H), 3.80 – 3.74 (m, 2H), 3.73 – 3.66 (m, 1H), 3.58 (td, *J* = 6.8, 4.2 Hz, 1H), 2.32 (t, *J* = 5.2 Hz, 1H), 2.31 – 2.21 (m, 2H), 1.95 – 1.83 (m, 2H), 1.83 – 1.74 (m, 1H), 0.96 (d, *J* = 6.9 Hz, 3H), 0.89 (s, 9H), 0.06 (s, 3H), 0.05 (s, 3H).

$^{13}$C NMR (151 MHz, $CDCl_3$) δ 159.37, 135.13, 130.53, 129.75, 117.26, 113.97, 79.89, 71.99, 71.72, 60.89, 55.41, 40.02, 39.70, 33.14, 26.05, 18.26, 10.09, -3.56, -4.36.

HRMS (ESI): Anal. Calcd. for $C_{23}H_{41}O_4Si^+$ $[M+H]^+$ 409.2769, found 409.2801

IR (neat): $\nu_{max}$ $(cm^{-1})$ = 3414 (br, OH), 3075 (w, C=*CH*), 2954 (m, CH), 2932 (m, CH), 2857 (m, CH), 1614 (m, C=C), 1514 (m), 1466 (m), 1250 (s).

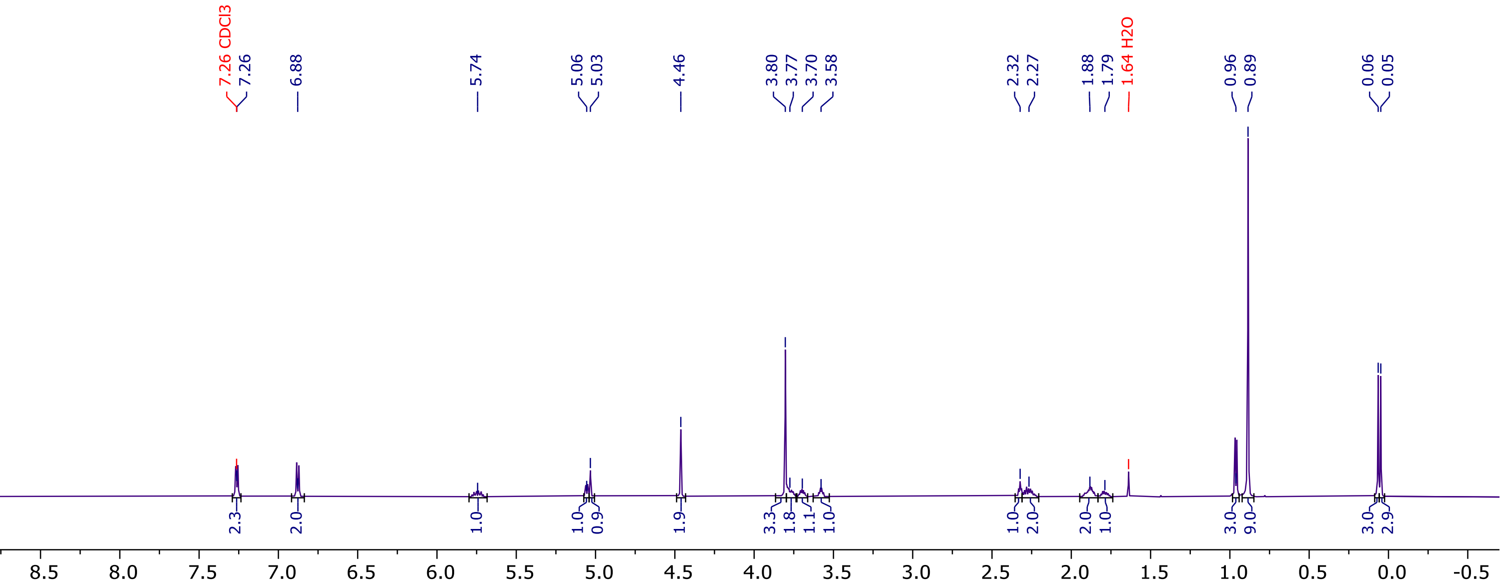
1H
CDCl3
600.13 MHz
7.26 CDCl3
7.26
6.88
5.74
5.06
5.03
4.46
3.80
3.77
3.70
3.58
2.32
2.27
1.88
1.79
1.64 H2O
0.96
0.89
0.06
0.05
2.3
2.0
1.0
1.0
0.9
1.9
3.3
1.8
1.1
1.0
1.0
2.0
2.0
1.0
3.0
9.0
3.0
2.9
10.0
9.5
9.0
8.5
8.0
7.5
7.0
6.5
6.0
5.5
5.0
4.5
4.0
3.5
3.0
2.5
2.0
1.5
1.0
0.5
0.0
-0.5

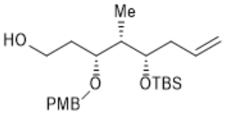
HO
Me
O
OTBS
PMB

**2b**

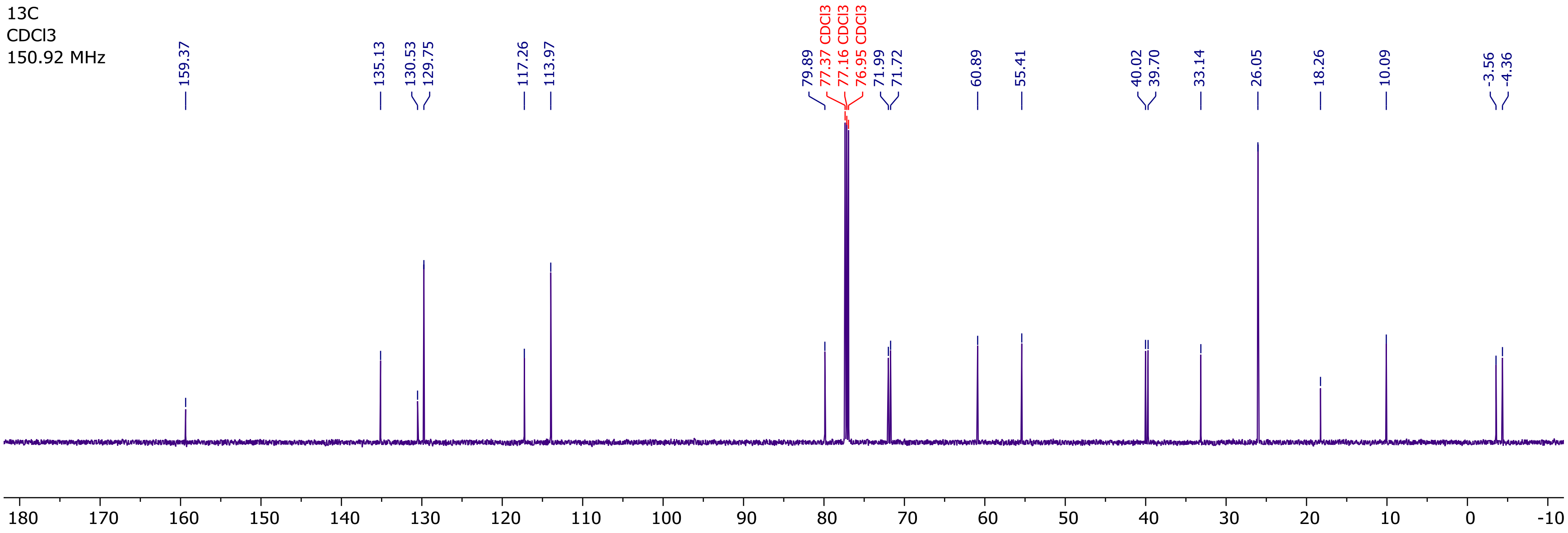
13C
CDCl3
150.92 MHz
159.37
135.13
130.53
129.75
117.26
113.97
79.89
77.37 CDCl3
77.16 CDCl3
76.95 CDCl3
71.99
71.72
60.89
55.41
40.02
39.70
33.14
26.05
18.26
10.09
-3.56
-4.36
180
170
160
150
140
130
120
110
100
90
80
70
60
50
40
30
20
10
0
-10

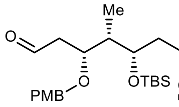

**S39** To a rt, stirred solution of **2b** (245.2 mg, 0.60 mmol, 1.0 eq.) and $NaHCO_3$ (252 mg, 3 mmol, 5.0 eq.) in $CH_2Cl_2$ (6 mL) was added Dess-Martin periodinane (305.5 mg, 0.72 mmol, 1.2 eq.) in one portion. The reaction was allowed to stir at rt for 30 min. After this time starting material was consumed as monitored by TLC analysis, and the reaction mixture was quenched with 10% aqueous $Na_2S_2O_3$ (3 mL) and saturated aqueous $NaHCO_3$ (3 mL). The cloudy mixture was vigorously stirred for 1 h. After this time, stirring was stopped and two clear layers formed. The biphasic mixture was poured into a separatory funnel and extracted with $CH_2Cl_2$ (3 x 10 mL). The combined organic layers were dried ($Na_2SO_4$), filtered, and the solvent was removed in vacuo. The crude product was passaged through a short plug of silica gel eluting with $Et_2O$. The solvent was removed in vacuo to give **S39** (244 mg, quant.) as a colorless oil.

**Analytical Data for S39:**

$R_f$ = 0.35 (8.5:1.5 Hexanes/$Et_2O$)

$[\alpha]_D^{20}$ = +3.1° (c = 2.08, $CDCl_3$)

$^1$H NMR (600 MHz, $CDCl_3$) δ 9.78 (t, *J* = 2.3 Hz, 1H), 7.25 – 7.19 (m, 2H), 6.89 – 6.84 (m, 2H), 5.73 (ddt, *J* = 16.4, 10.9, 7.2 Hz, 1H), 5.07 – 5.04 (m, 1H), 5.03 (t, *J* = 1.3 Hz, 1H), 4.46 (d, *J* = 11.0 Hz, 1H), 4.43 (d, *J* = 11.0 Hz, 1H), 3.94 (td, *J* = 6.4, 4.9 Hz, 1H), 3.83 – 3.77 (m, 4H), 2.73 – 2.64 (m, 2H), 2.32 – 2.25 (m, 1H), 2.23 (dddt, *J* = 13.9, 6.9, 5.5, 1.4 Hz, 1H), 1.89 – 1.81 (m, 1H), 0.96 (d, *J* = 6.9 Hz, 3H), 0.88 (s, 9H), 0.06 (s, 3H), 0.04 (s, 3H).

$^{13}$C NMR (151 MHz, $CDCl_3$) δ 202.17, 159.36, 135.05, 130.39, 129.60, 117.33, 113.92, 75.43, 72.19, 71.62, 55.40, 46.65, 40.97, 39.58, 26.05, 18.24, 10.27, -3.65, -4.31.

HRMS (ESI): Anal. Calcd. for $C_{23}H_{42}NO_4^+$ [M+$NH_4$]$^+$ 424.2878, found 424.2861

IR (neat): $\nu_{max}$ $(cm^{-1})$ = 3075 (w, C=*CH*), 2720 (w, O=*CH*), 2954 (m, CH), 2931 (m, CH), 2857 (m, CH), 1725 (s, C=O), 1613 (m, C=C), 1466 (m), 1389 (m), 1301 (m), 1250 (s)

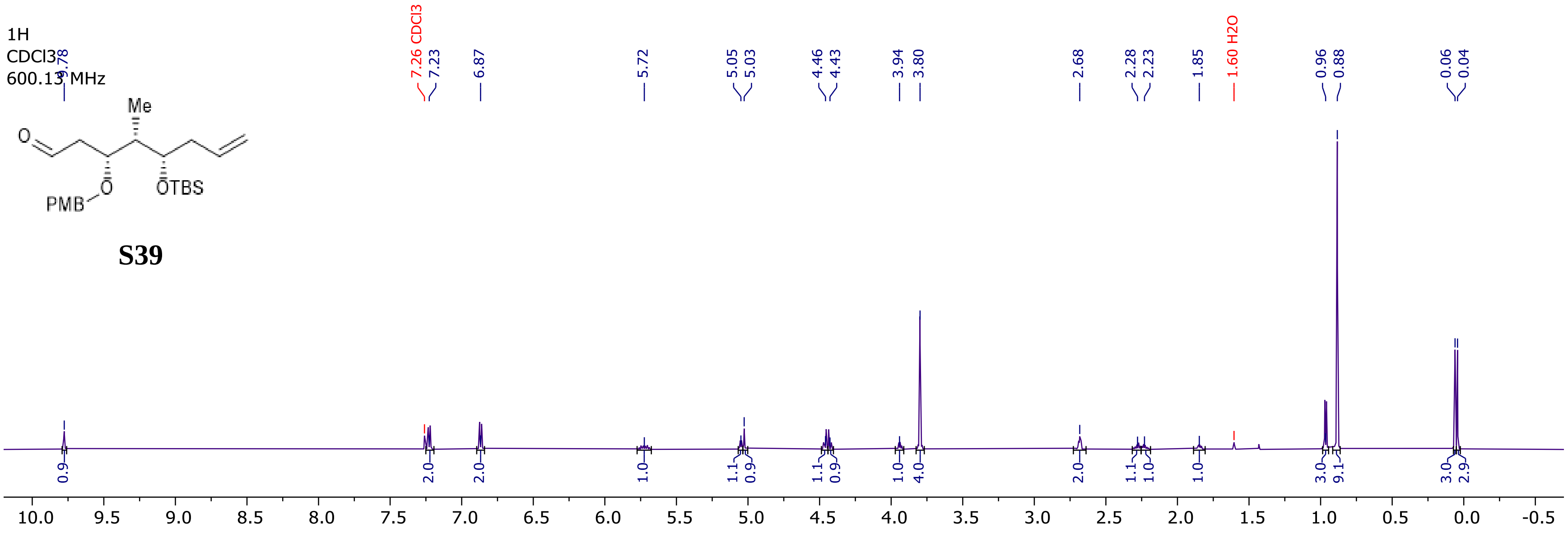
Me
O
PMB
O
OTBS
1H
CDCl3
600.13 MHz
9.78
7.26 CDCl3
7.23
6.87
5.72
5.05
5.03
4.46
4.43
3.94
3.80
2.68
2.28
2.23
1.85
1.60 H2O
0.96
0.88
0.06
0.04
0.9
2.0
2.0
1.0
1.1
0.9
1.1
0.9
1.0
4.0
2.0
1.1
1.0
1.0
3.0
9.1
3.0
2.9
10.0
9.5
9.0
8.5
8.0
7.5
7.0
6.5
6.0
5.5
5.0
4.5
4.0
3.5
3.0
2.5
2.0
1.5
1.0
0.5
0.0
-0.5

**S39**

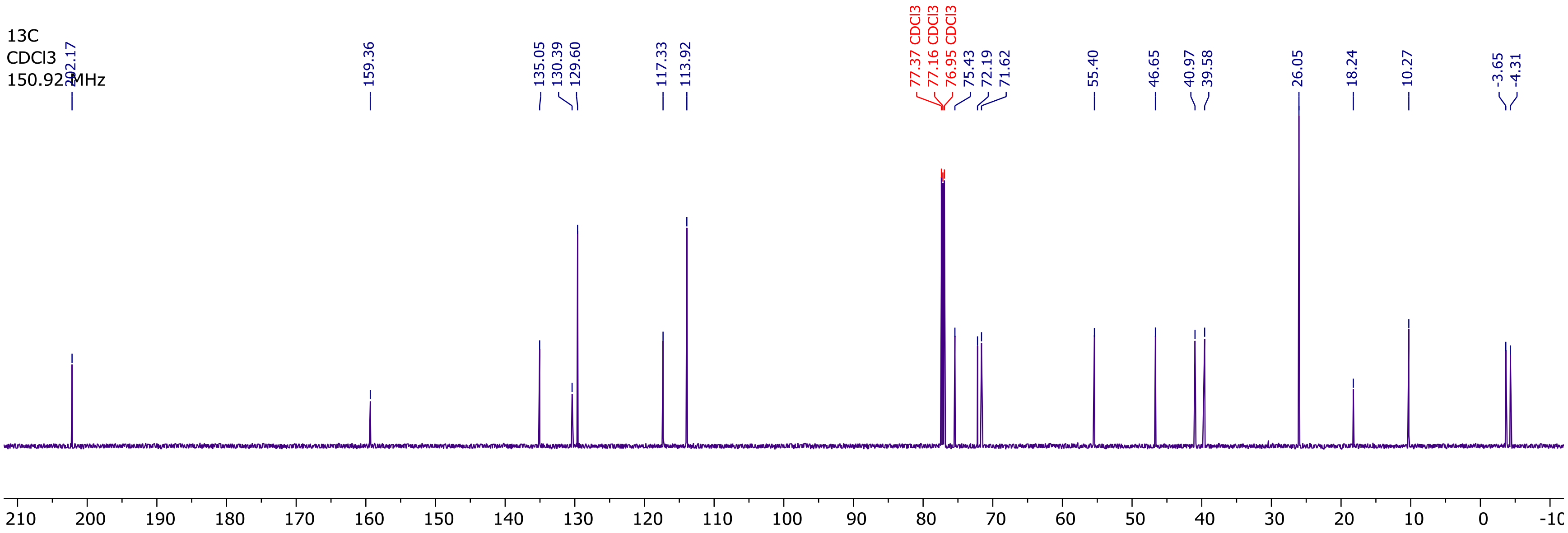
13C
CDCl3
150.92 MHz
202.17
159.36
135.05
130.39
129.60
117.33
113.92
77.37 CDCl3
77.16 CDCl3
76.95 CDCl3
75.43
72.19
71.62
55.40
46.65
40.97
39.58
26.05
18.24
10.27
-3.65
-4.31
210
200
190
180
170
160
150
140
130
120
110
100
90
80
70
60
50
40
30
20
10
0
-10

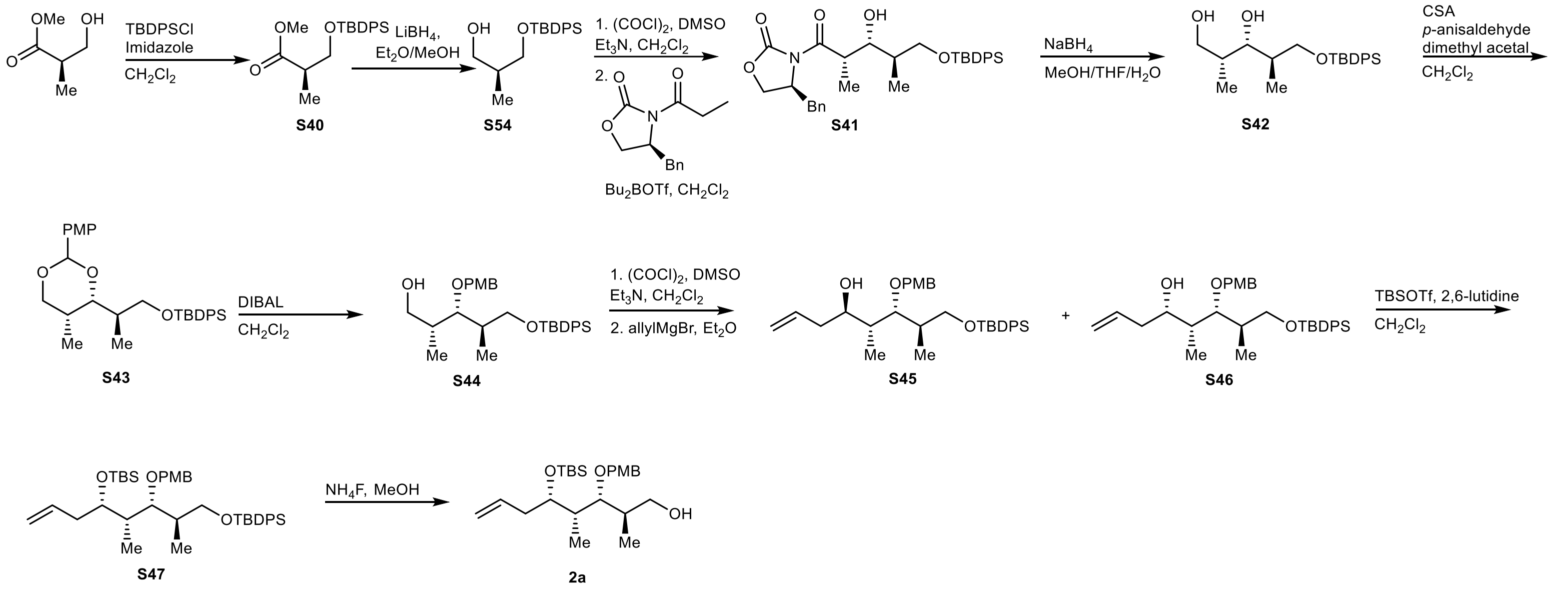


**Supplementary Fig. 31 | Synthesis of Alcohol 2a from** methyl (*R*)-(-)-3-hydroxy-2-methylpropionate.

Abbreviations: TBDPSCl = tert-butyldiphenylsilyl chloride, TBDPS = tert-butyldiphenylsilyl, DMSO = dimethylsulfoxide, THF = tetrahydrofuran, CSA = camphorsulfonic acid, PMP = *para*-methoxyphenyl, DIBAL = diisobutylaluminum hydride, PMB = *para*-methoxybenzyl, TBSOTf = tert-butyldimethylsilyl trifluoromethanesulfonate, TBS = tert-butyldimethylsilyl.

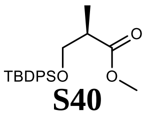

**S40**

To a cold (0 °C), stirred solution of methyl (*R*)-(-)-3-hydroxy-2-methylpropionate (1.18 g, 10.0 mmol, 1.0 eq.) and imidazole (1.02 g, 15.0 mmol, 1.5 eq.) in $CH_2Cl_2$ (20 mL) was added TBDPSCl (7.8 mL, 8.3 g, 30.0 mmol, 1.2 eq.) via syringe and the cold bath removed. The reaction mixture was stirred at rt for 3 hours. After this time, the reaction mixture was poured into a separatory funnel and washed with $H_2O$ (3x 30 mL). The aqueous layers were back extracted with $Et_2O$ (3x 70 mL). Then the combined organic layers were washed with brine (1x 50 mL), dried ($MgSO_4$), filtered, and the solvent was removed in vacuo. The crude product was purified via flash column chromatography (92.5:7.5 Hexanes/$Et_2O$). Appropriate fractions were pooled, and solvent was removed in vacuo to yield **S40** (3.18 g, 89%) as a colorless oil. Spectral and optical rotation data agreed with the data previously reported in the literature.[11]

**Analytical Data for S40:**

$R_f$ = 0.53 (17:3 Hexanes/$Et_2O$)

$[\alpha]_D^{20}$ = -14.0 ° (c = 2.25, $CDCl_3$); lit. $[\alpha]_D^{20}$ = -13.6 ° (c = 2.12, $CHCl_3$)[11]

$^1$H NMR (600 MHz, $CDCl_3$) δ 7.68 – 7.64 (m, 4H), 7.46 – 7.41 (m, 2H), 7.41 – 7.36 (m, 4H), 3.83 (dd, *J* = 9.8, 6.9 Hz, 1H), 3.73 (dd, *J* = 9.8, 5.8 Hz, 1H), 3.69 (s, 3H), 2.73 (tq, *J* = 7.1, 5.8 Hz, 1H), 1.16 (d, *J* = 7.0 Hz, 3H), 1.04 (s, 9H).

$^{13}$C NMR (151 MHz, $CDCl_3$) δ 175.55, 135.71, 133.63, 133.56, 129.79, 127.79, 66.02, 51.71, 42.51, 26.82, 19.36, 13.61.

HRMS (ESI): Anal. Calcd. for $C_{21}H_{29}O_3Si^+$ $[M+H]^+$ 357.1881, found 357.1890

IR (neat): $\nu_{max}$ $(cm^{-1})$ = 3071 (w, CH), 2935 (m, CH), 2859 (m, CH), 1741 (s, C=O), 1429 (m), 1199 (s).

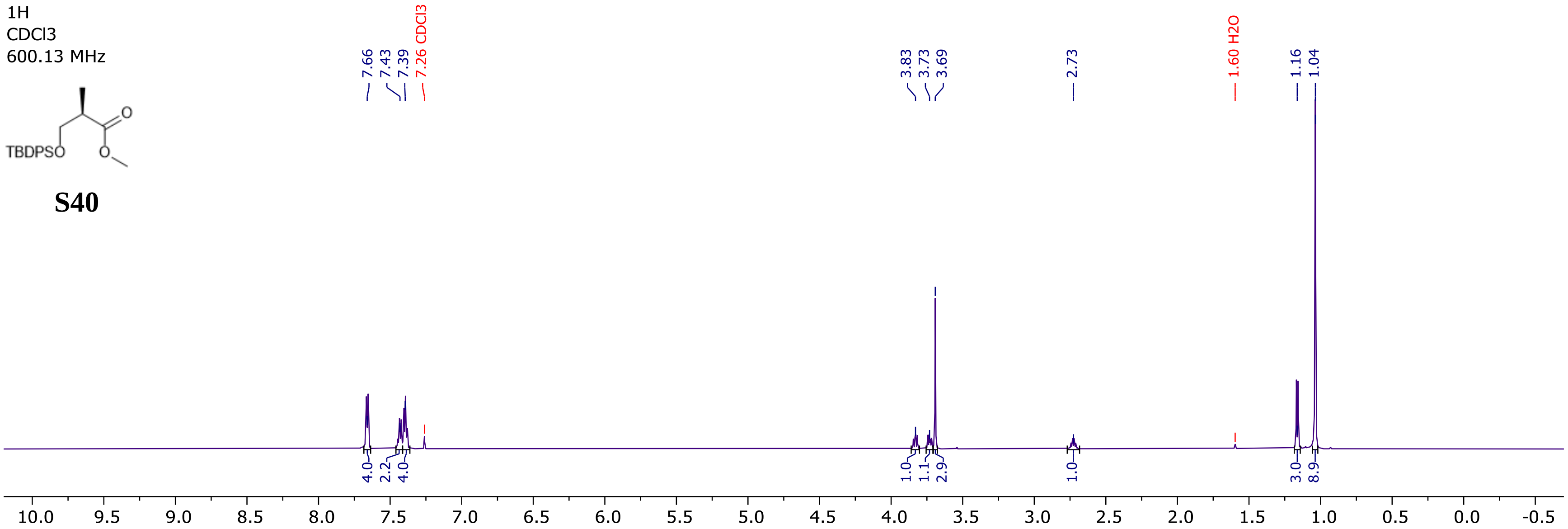
1H
CDCl3
600.13 MHz
TBDPSO
O
O
S40
7.66
7.43
7.39
7.26 CDCl3
3.83
3.73
3.69
2.73
1.60 H2O
1.16
1.04
4.0
2.2
4.0
1.0
1.1
2.9
1.0
3.0
8.9
10.0
9.5
9.0
8.5
8.0
7.5
7.0
6.5
6.0
5.5
5.0
4.5
4.0
3.5
3.0
2.5
2.0
1.5
1.0
0.5
0.0
-0.5

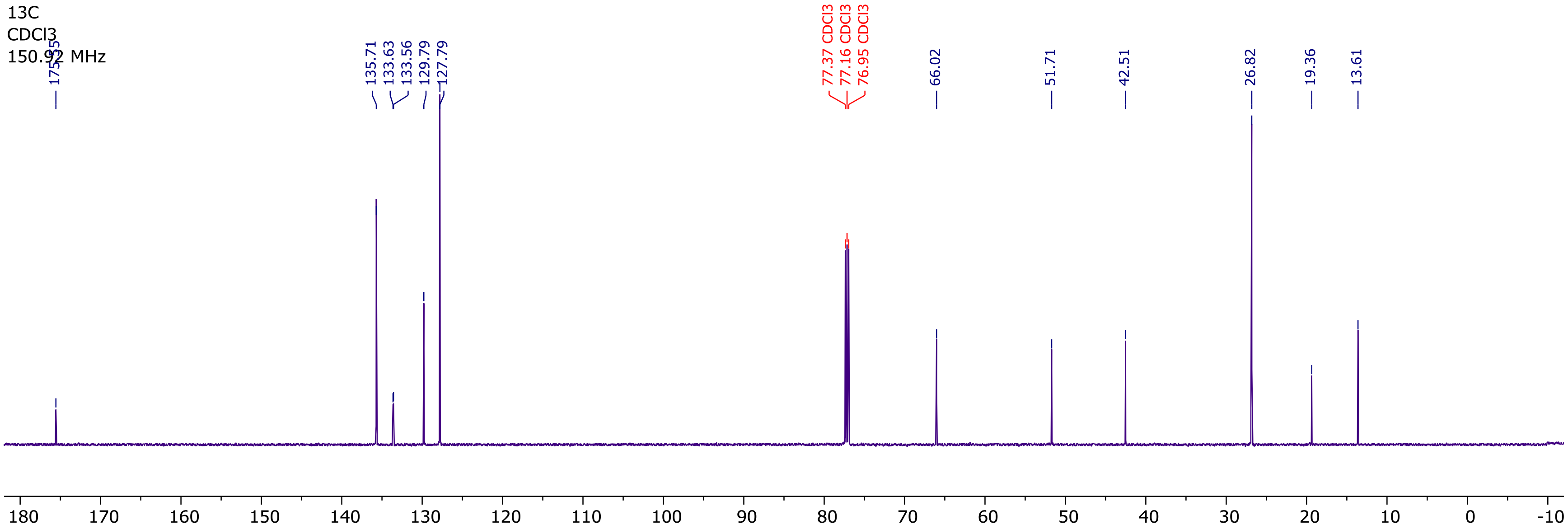
13C
CDCl3
150.92 MHz
175.55
135.71
133.63
133.56
129.79
127.79
77.37 CDCl3
77.16 CDCl3
76.95 CDCl3
66.02
51.71
42.51
26.82
19.36
13.61
180
170
160
150
140
130
120
110
100
90
80
70
60
50
40
30
20
10
0
-10

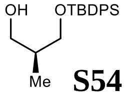

**S54**

To a cold (0 °), stirred solution of **S40** (8.91 g, 25 mmol, 1 eq.) in $Et_2O$ (45 mL) and MeOH (1 mL) was added $LiBH_4$ (1.09 g, 50 mmol, 2 eq.) portionwise over 20 min (**caution!** $H_2$ gas is liberated). Subsequently the reaction mixture was allowed to warm to room temperature, and stirred at rt for 5 h 30 min. After this time, the reaction mixture was cooled to 0 °C and quenched with a saturated aqueous solution of $NH_4Cl$ (100 mL) and stirred for 1 h while slowly warming to rt. After evolution of hydrogen gas had ceased, the reaction mixture was poured into a separatory funnel, and the aqueous layer was extracted with $Et_2O$ (3x 50 mL). The combined organic layers were washed with brine (2x 35 mL), and the brine was back extracted with $Et_2O$ (1x 30 mL). The combined organic layers were dried ($MgSO_4$), filtered, and solvent was removed in vacuo to give the crude product as a cloudy yellow oil. The crude product was purified via flash column chromatography (3:1 Hexanes/$Et_2O$) to give **S54** as a pale yellow oil. Spectroscopic data agreed with previously reported data.[12] A portion of the product was used immediately in the subsequent step.

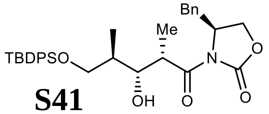

**S41**

To a cold (-78 °C), stirred solution of $(COCl)_2$ (1.9 mL, 2.8 g, 30 mmol, 1.78 eq.) in $CH_2Cl_2$ (mL) was added anhydrous DMSO (5.1 mL, 3.4 g, 72 mmol, 4.26 eq.) [**Caution! $CO_2$ (g) evolved**] over 5 minutes via syringe. Subsequently, **S54** (5.56 g, 16.9 mmol, 1.0 eq.) was added as a solution in $CH_2Cl_2$ (*ca.* 10 mL) over 5 min. The reaction mixture was maintained at -78 °C for 15 min, after which time $Et_3N$ (35.0 mL, 25.4 g, 251 mmol, 14.9 eq.) was added via syringe over 5 min to give a cloudy white suspension. The reaction mixture was maintained at -78 °C for a further 1 h, at which point the $CO_2$(s)/Acetone (-78 °C) bath was exchanged for an ice bath (0 °C) to allow slow warming to 0 °C. After a further 15 min, the starting material was consumed as monitored by TLC analysis. Then the reaction mixture was poured into a separatory funnel and washed with 10% aqueous $CuSO_4$ (2x 100 mL). The combined aqueous layers were back extracted with $Et_2O$ (2x 200 mL), dried ($MgSO_4$), filtered, and solvent was removed in vacuo to give the crude aldehyde as a yellow oil which was used in the subsequent step without further purification. To a cold (0 °C), stirred solution of (*S*)-(+)-4-benzyl-3-propionyl-2-oxazolidinone (4.34 g, 18.6 mmol, 1.1 eq.) was added a solution of $Bu_2BOTf$ (19 mL, 19 mmol, 1M in $CH_2Cl_2$, 1.1 eq.) via syringe. After stirring for 15 min, $Et_3N$ (3.2 mL, 2.4 g, 23.2 mmol, 1.37 eq.) was added via syringe. Then the reaction mixture was cooled (-78 °C), and the crude aldehyde (*ca.* 5.6 g, *ca.* 16.9 mmol, 1.0 eq.) was added via syringe as a solution in anhydrous $CH_2Cl_2$ (10 mL). The reaction mixture was maintained at -78 °C for 2 h, at which point the $CO_2$(s)/Acetone bath was exchanged for an ice bath (0 °C). After 30 min of slowly warming to 0 °C, starting material was consumed as monitored by TLC analysis. After this time, the reaction was quenched by addition of a solution of 1:1 (v/v) pH 7 phosphate buffer/MeOH, followed by a solution of 3:1 (v/v) 30% aqueous $H_2O_2$/MeOH. The reaction mixture was slowly warmed to rt over 24 h with vigorous stirring. After this time, the reaction mixture was poured into a separatory funnel, the organic layer separated, and the aqueous layer extracted with $CH_2Cl_2$ (3x 40 mL). The combined organic layers were washed with brine (1x 40 mL), dried ($Na_2SO_4$), filtered, and solvent was removed in vacuo. The crude product was purified via flash column

chromatography (4:1 to 7:3 Hexanes/$Et_2O$). Appropriate fractions were pooled, and solvent was removed in vacuo to yield **S41** (7.7 g, 81%, 2 steps) as a viscous colorless oil. Spectral and optical rotation data agreed with the data previously reported in the literature.[13]

**Analytical Data for S41:**

$R_f$ = 0.22 (3:17 Hexanes/EtOAc)

$[\alpha]_D^{20}$ = +18.1° (c = 3.10, $CDCl_3$); lit. $[\alpha]_D^{30}$ = +20.6 ° (c = 6.23, $CHCl_3$)[13]

$^1$H NMR (600 MHz, $CDCl_3$) δ 7.68 (ddt, *J* = 6.3, 4.8, 1.5 Hz, 4H), 7.47 – 7.42 (m, 2H), 7.42 – 7.37 (m, 4H), 7.34 (td, *J* = 7.2, 6.3, 1.3 Hz, 2H), 7.30 – 7.26 (m, 1H), 7.25 – 7.20 (m, 2H), 4.74 – 4.68 (m, 1H), 4.25 – 4.20 (m, 1H), 4.18 (dd, *J* = 9.0, 2.4 Hz, 1H), 3.97 (q, *J* = 3.4 Hz, 2H), 3.96 – 3.90 (m, 1H), 3.81 (dd, *J* = 10.2, 4.2 Hz, 1H), 3.70 (dd, *J* = 10.2, 6.7 Hz, 1H), 3.34 (dd, *J* = 13.3, 3.3 Hz, 1H), 2.78 (dd, *J* = 13.3, 9.7 Hz, 1H), 1.86 (dtd, *J* = 13.6, 6.7, 4.1 Hz, 1H), 1.28 (d, *J* = 6.8 Hz, 3H), 1.05 (s, 8H), 0.89 (d, *J* = 6.9 Hz, 3H).

$^{13}$C NMR (151 MHz, $CDCl_3$) δ 176.50, 153.27, 135.73, 135.72, 135.47, 133.05, 132.98, 129.95, 129.59, 129.08, 127.90, 127.88, 127.46, 75.33, 68.72, 66.30, 55.78, 40.74, 37.85, 37.63, 26.95, 19.28, 13.31, 9.39.

HRMS (ESI): Anal. Calcd. for $C_{33}H_{42}NO_5Si^+$ $[M+H]^+$ 560.2827, found 560.2833

IR (neat): $\nu_{max}$ $(cm^{-1})$ = 3376 (br, OH), 3073 (w), 2967 (m, CH), 2248 (m), 2116 (m), 1780 (s, C=O), 1697 (m), 1465 (m), 1426 (m), 1387 (m), 1212 (s).

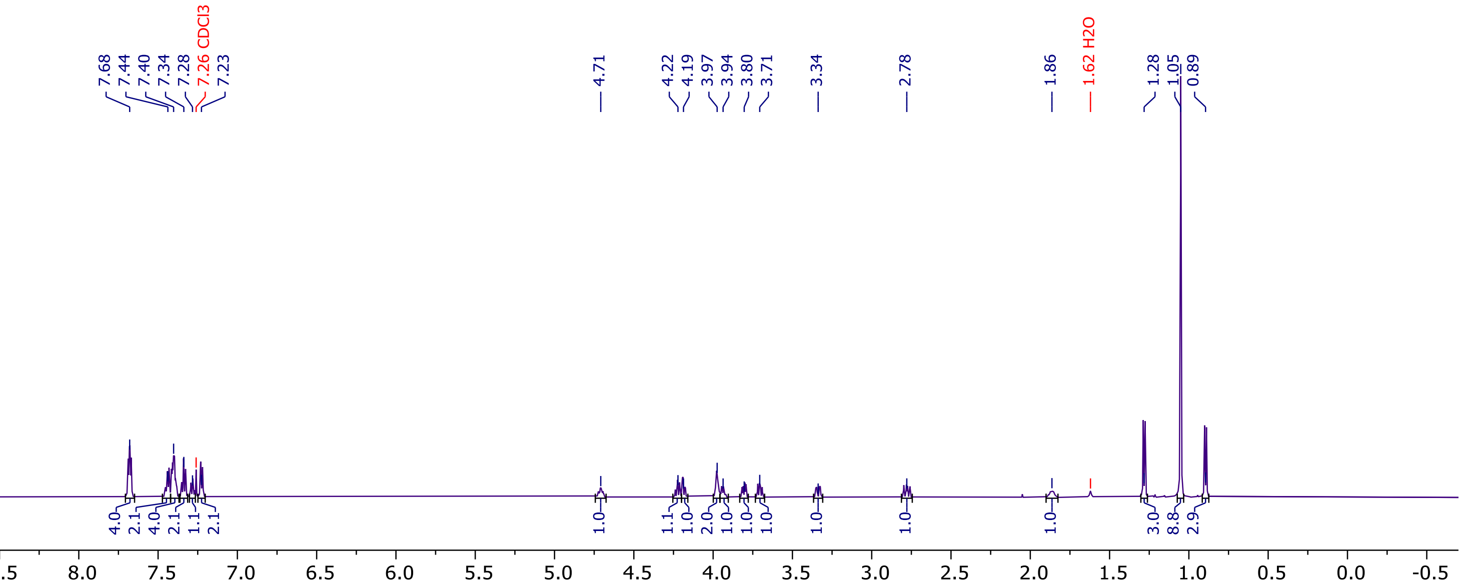
1H
CDCl3
600.13 MHz
7.68
7.44
7.40
7.34
7.28
7.26 CDCl3
7.23
4.71
4.22
4.19
3.97
3.94
3.80
3.71
3.34
2.78
1.86
1.62 H2O
1.28
1.05
0.89
4.0
2.1
4.0
2.1
1.1
2.1
1.0
1.1
1.0
2.0
1.0
1.0
1.0
1.0
1.0
1.0
3.0
8.8
2.9
10.0 9.5 9.0 8.5 8.0 7.5 7.0 6.5 6.0 5.5 5.0 4.5 4.0 3.5 3.0 2.5 2.0 1.5 1.0 0.5 0.0 -0.5

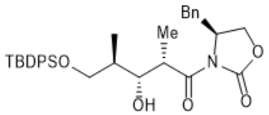
TBDPSO
Bn
Me
N
O
O
O
OH

**S41**

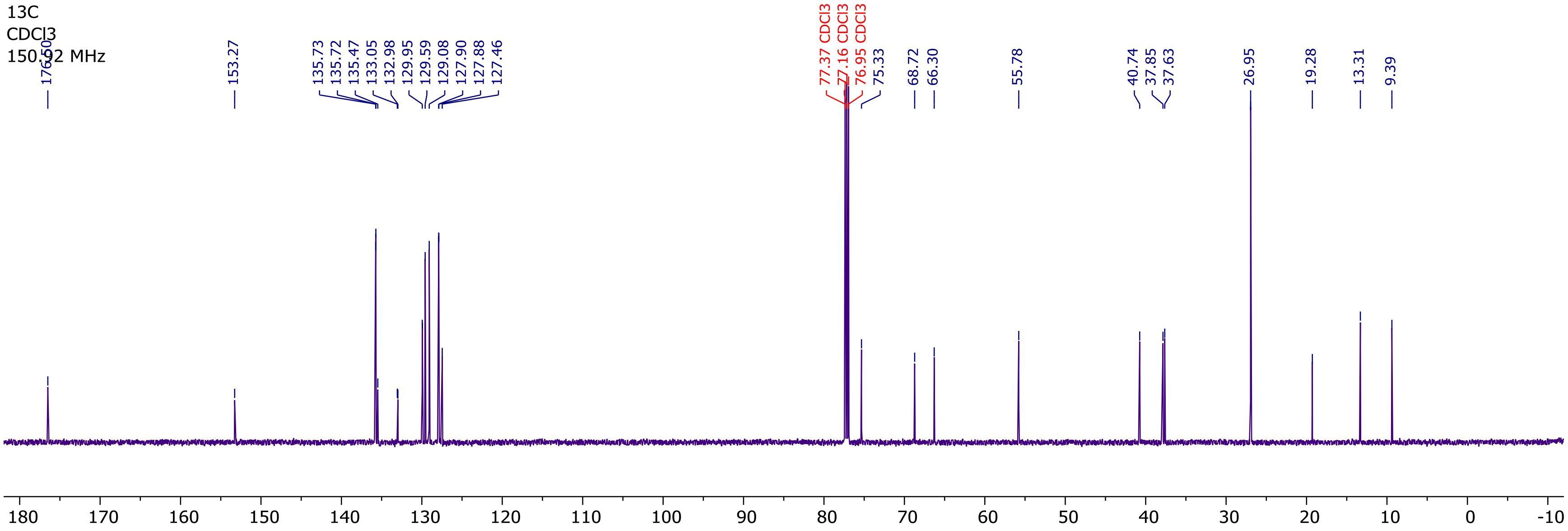
13C
CDCl3
150.92 MHz
176.90
153.27
135.73
135.72
135.47
133.05
132.98
129.95
129.59
129.08
127.90
127.88
127.46
77.37 CDCl3
77.16 CDCl3
76.95 CDCl3
75.33
68.72
66.30
55.78
40.74
37.85
37.63
26.95
19.28
13.31
9.39
180 170 160 150 140 130 120 110 100 90 80 70 60 50 40 30 20 10 0 -10

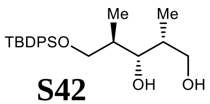


**S42**

To a cold (0 °C), stirred solution of **S41** (7.70 g, 13.8 mmol, 1.0 eq.) in MeOH/THF/$H_2O$ (1:29:10 (v/v/v), 69 mL) was added $NaBH_4$ (4.95 g, 131 mmol, 10 eq.). The mixture was stirred while slowly warming to room temperature over 18 h 30 min. After this time, the reaction mixture was cooled to 0 °C and quenched with saturated aqueous $NH_4Cl$ (100 mL). After evolution of gas had ceased, the reaction mixture was poured into a separatory funnel and extracted with $Et_2O$ (3x 70 mL) then EtOAc (2x 50 mL). The combined organic layers were washed with brine (1x 70 mL), dried ($MgSO_4$), filtered, and solvent was removed in vacuo. The crude product was purified via flash column chromatography (7:3 Hexanes/EtOAc). Appropriate fractions were pooled, and solvent was removed in vacuo to give **S42** (4.58 g, 86%) as a viscous colorless oil.

**Analytical Data for S42:**

$R_f$ = 0.37 (6.5:3.5 Hexanes/EtOAc)

$[\alpha]_D^{20}$ = -20.6° (c = 2.40, $CDCl_3$);

$^1$H NMR (500 MHz, $CDCl_3$) δ 7.68 (dt, *J* = 8.0, 1.4 Hz, 4H), 7.49 – 7.38 (m, 6H), 3.85 (dd, *J* = 9.2, 2.3 Hz, 1H), 3.82 (dd, *J* = 10.6, 3.7 Hz, 1H), 3.77 – 3.69 (m, 2H), 3.66 (dd, *J* = 10.2, 9.0 Hz, 1H), 3.42 (s, 2H), 1.92 (tqd, *J* = 9.1, 6.9, 4.0 Hz, 1H), 1.76 (pdd, *J* = 7.0, 3.7, 2.4 Hz, 1H), 1.06 (s, 9H), 1.01 (d, *J* = 7.0 Hz, 3H), 0.67 (d, *J* = 6.9 Hz, 3H).

$^{13}$C NMR (126 MHz, $CDCl_3$) δ 135.72, 132.71, 132.64, 130.12, 130.10, 128.00, 79.95, 70.66, 68.13, 37.50, 36.54, 26.92, 19.16, 12.86, 8.86.

HRMS (ESI): Anal. Calcd. for $C_{23}H_{35}O_3Si^+$ $[M+H]^+$ 387.2350, found 387.2360

IR (neat): $\nu_{max}$ $(cm^{-1})$ = 3409 (br, OH), 3071 (w), 3049 (w), 2961 (m, CH), 2932 (m, CH), 2859 (m, CH), 1589 (w), 1468 (m), 1427 (m), 1390 (m), 1247 (w).

1H
CDCl3
500.14 MHz

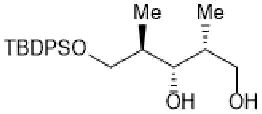


**S42**

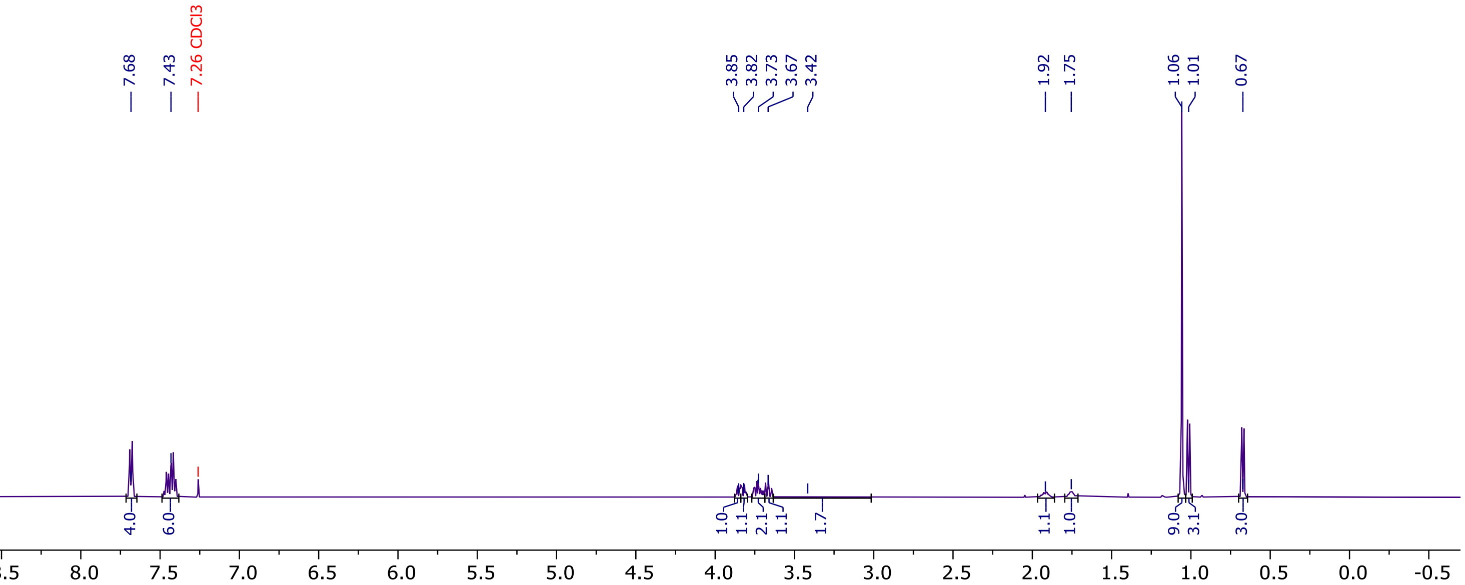


13C
CDCl3
125.77 MHz

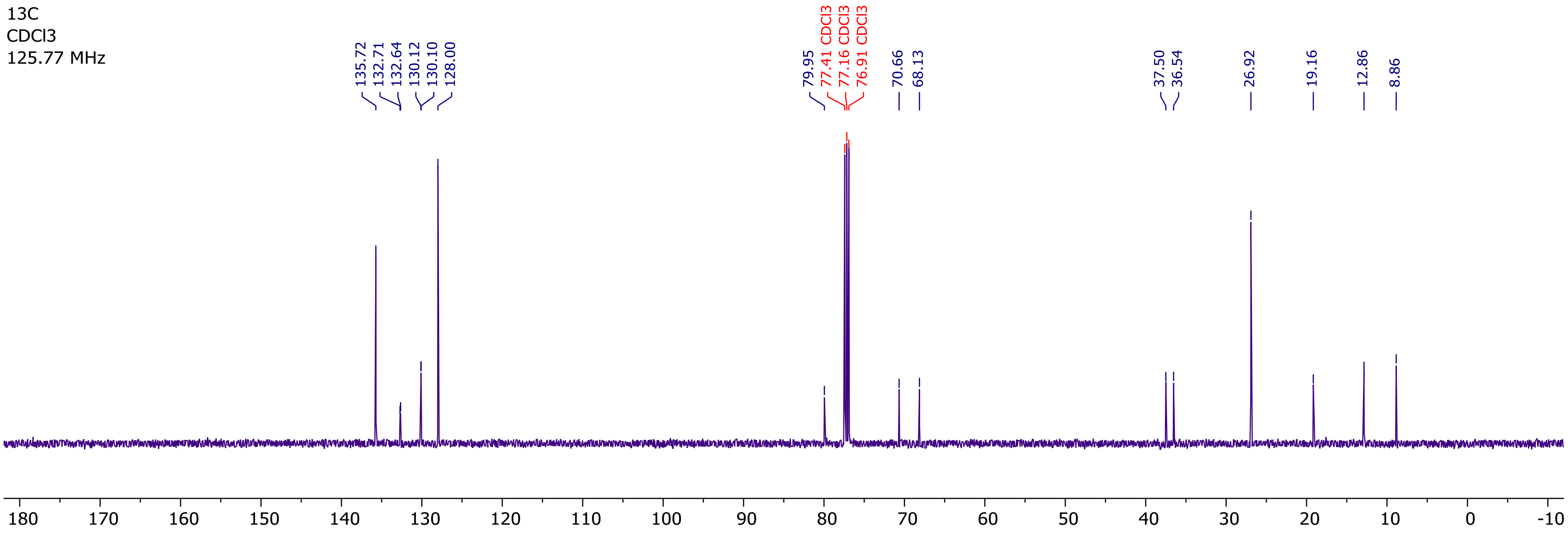

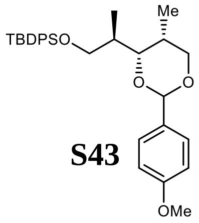


**S43**

Reaction performed in capped vessel under ambient atmosphere. To a rt stirred solution of **S42** (4.58 g, 11.8 mmol, 1.0 eq.) and p-methoxybenzaldehyde dimethyl acetal (3.6 mL, 3.9 g, 21 mmol, 1.8 eq.) dissolved in $CH_2Cl_2$ (70 mL), was added CSA (655 mg, 2.82 mmol, 0.24 eq.) in one portion. The reaction mixture was stirred for 22 h at rt, after which time starting material was consumed as monitored by TLC analysis. The reaction mixture was poured into a separatory funnel, washed with saturated aqueous $NaHCO_3$ (125 mL), and the aqueous layer back extracted with $CH_2Cl_2$ (2x 40 mL). The combined organic layers were dried ($Na_2SO_4$), filtered, and solvent was removed in vacuo. The crude product was dissolved in EtOAc (30 mL), then $H_2O$ (30 mL) and $NaHSO_3$ (3.1 g, 29.8 mmol) were added in sequence at rt. The reaction mixture was vigorously stirred at rt for 1 h. After this time, the reaction mixture was poured into a separatory funnel, the organic layer drained, and the aqueous layer extracted with EtOAc (3x 70 mL). The combined organic layers were washed with brine (1x 50 mL), dried ($Na_2SO_4$), filtered, and solvent was removed in vacuo. The crude product was purified via flash column chromatography (19:1 hexanes:EtOAc). Appropriate fractions were pooled, and solvent was removed in vacuo to yield **S43** (3.91 g, 65%) as a colorless oil.

**Analytical Data for S43:**

$R_f$ = 0.46 (9:1 Hexanes/EtOAc)

$[\alpha]_D^{20}$ = +11.6 ° (c = 1.25, $CH_2Cl_2$)

$^1$H NMR (600 MHz, $CD_2Cl_2$) δ 7.71 – 7.65 (m, 2H), 7.65 – 7.60 (m, 2H), 7.43 – 7.38 (m, 1H), 7.39 – 7.33 (m, 3H), 7.33 – 7.28 (m, 2H), 7.26 – 7.20 (m, 2H), 6.89 – 6.83 (m, 2H), 5.42 (s, 1H), 4.07 (dd, *J* = 11.1, 2.5 Hz, 1H), 4.00 (dd, *J* = 11.1, 1.5 Hz, 1H), 3.96 – 3.88 (m, 2H), 3.81 (s, 3H), 3.65 (dd, *J* = 9.6, 2.5 Hz, 1H), 1.86 – 1.77 (m, 1H), 1.71 – 1.64 (m, 1H), 1.14 (d, *J* = 6.9 Hz, 3H), 1.08 – 1.02 (m, 12H).

$^{13}$C NMR (151 MHz, $CD_2Cl_2$) δ 160.13, 135.93, 135.89, 134.28, 134.24, 132.22, 129.86, 129.79, 127.96, 127.91, 127.71, 113.64, 101.73, 79.85, 74.25, 64.94, 55.62, 37.43, 29.99, 26.96, 19.62, 12.52, 11.00

HRMS (ESI): Anal. Calcd. for $C_{31}H_{41}O_4Si^+$ $[M+H]^+$ 505.2769, found 505.2754

IR ($CDCl_3$): $\nu_{max}$ $(cm^{-1})$ = 2963 (m, CH), 2856 (m, CH), 1616 (m, C=C), 1588 (w, C=C), 1517 (m), 1465 (m), 1249 (s).

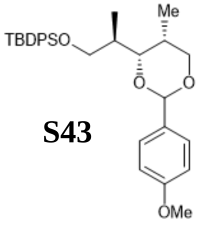
Me
TBDPSO
O
O
OMe

**S43**

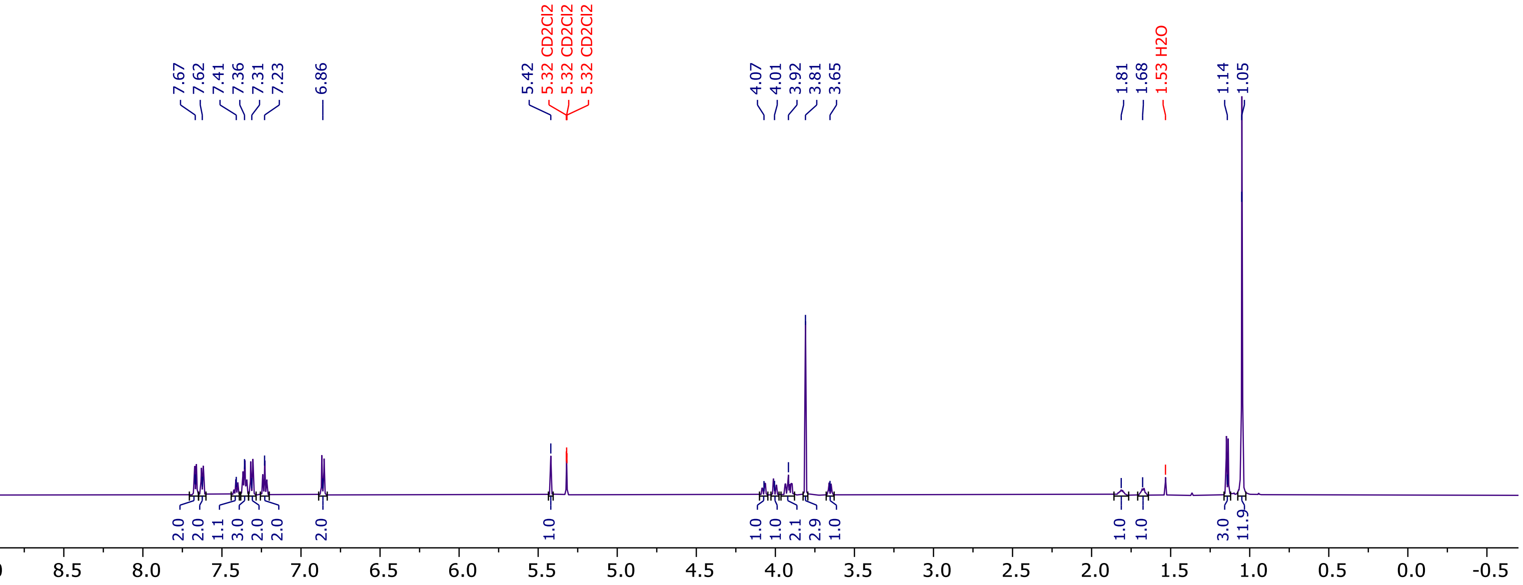
1H
CD2Cl2
600.13 MHz
7.67
7.62
7.41
7.36
7.31
7.23
6.86
5.42
5.32 CD2Cl2
5.32 CD2Cl2
5.32 CD2Cl2
4.07
4.01
3.92
3.81
3.65
1.81
1.68
1.53 H2O
1.14
1.05
2.0
2.0
1.1
3.0
2.0
2.0
2.0
1.0
1.0
1.0
2.1
2.9
1.0
1.0
1.0
3.0
11.9
10.0 9.5 9.0 8.5 8.0 7.5 7.0 6.5 6.0 5.5 5.0 4.5 4.0 3.5 3.0 2.5 2.0 1.5 1.0 0.5 0.0 -0.5

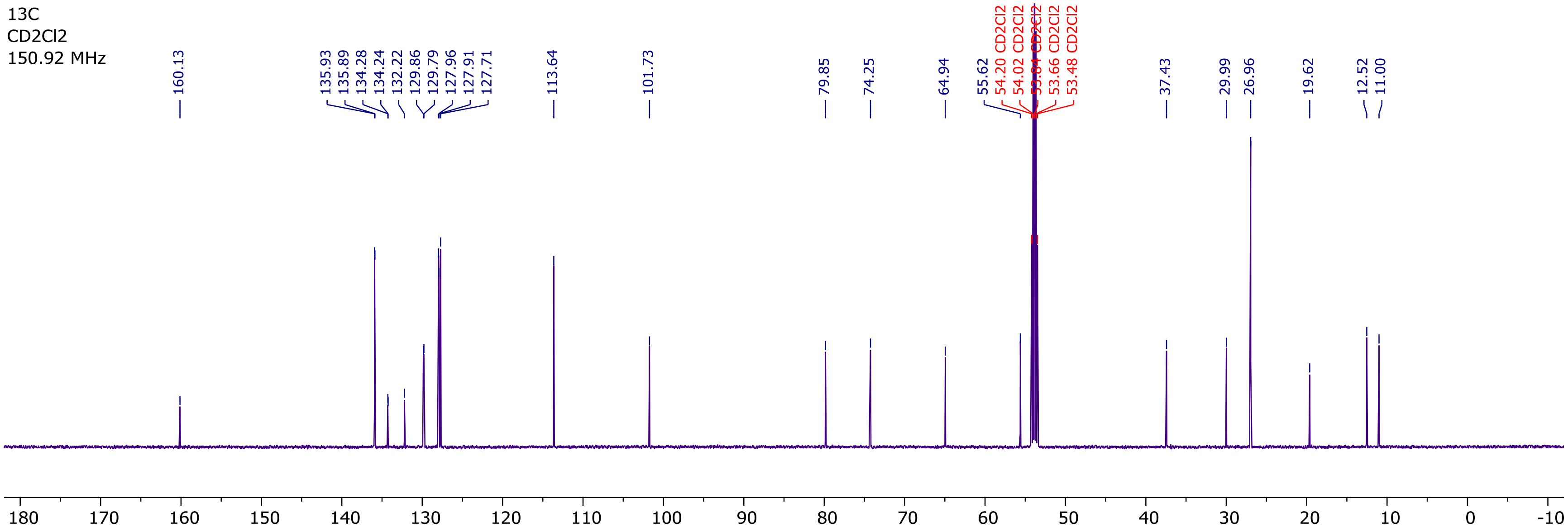
13C
CD2Cl2
150.92 MHz
160.13
135.93
135.89
134.28
134.24
132.22
129.86
129.79
127.96
127.91
127.71
113.64
101.73
79.85
74.25
64.94
55.62
54.20 CD2Cl2
54.02 CD2Cl2
53.84 CD2Cl2
53.66 CD2Cl2
53.48 CD2Cl2
37.43
29.99
26.96
19.62
12.52
11.00
180 170 160 150 140 130 120 110 100 90 80 70 60 50 40 30 20 10 0 -10

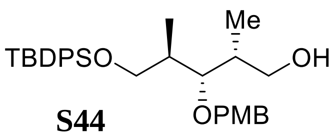


**S44**

To a cold (0 °C), stirred solution of **S43** in $CH_2Cl_2$ (65 mL) was added DIBAL-H (1.0 M in $CH_2Cl_2$, 15.5 mL, 15.5 mmol, 2.0 eq.) dropwise via syringe. The reaction mixture was slowly warmed to rt over 3 h 45 min. After this time, starting material was consumed as monitored by TLC analysis. The reaction mixture was then quenched by addition of saturated aqueous solution of Rochelle's salt (100 mL) [**Caution! $H_2$ gas evolved**] and allowed to stir overnight at rt. The reaction mixture was then poured into a separatory funnel, the organic layer drained, and the aqueous layer extracted with $CH_2Cl_2$ (3x 60 mL). The combined organic layers were then washed with brine (50 mL), dried ($Na_2SO_4$), filtered, and solvent was removed in vacuo. The crude product was purified via flash column chromatography (8:2 Hexanes/EtOAc). Appropriate fractions were pooled and solvent was removed in vacuo to yield **S44** (2.79 g, 71%) as a colorless oil.

**Analytical Data for S44:**

$R_f$ = 0.14 (9:1 Hexanes/EtOAc)

$[\alpha]_D^{20}$ = -8.7° (c = 3.12, $CDCl_3$)

$^1$H NMR (600 MHz, $CDCl_3$) δ 7.67 (ddt, *J* = 9.4, 6.7, 1.4 Hz, 4H), 7.46 – 7.40 (m, 2H), 7.40 – 7.34 (m, 4H), 7.09 – 7.03 (m, 2H), 6.82 – 6.76 (m, 2H), 4.43 (s, 2H), 3.79 (s, 3H), 3.80 – 3.75 (m, 2H), 3.64 – 3.57 (m, 3H), 1.97 – 1.87 (m, 2H), 1.72 (br. s, 1H), 1.10 (s, 9H), 0.99 (d, *J* = 6.9 Hz, 3H), 0.89 (d, *J* = 7.0 Hz, 3H).

$^{13}$C NMR (151 MHz, $CDCl_3$) δ 159.17, 135.94, 135.90, 133.91, 133.83, 130.98, 129.73, 129.52, 127.79, 127.73, 113.82, 80.68, 74.05, 66.70, 65.85, 55.39, 38.58, 37.41, 27.13, 19.46, 14.63, 10.58.

HRMS (ESI): Anal. Calcd. for $C_{31}H_{43}O_4^+$ $[M+H]^+$ 507.2925, found 507.2910

IR ($CDCl_3$): $\nu_{max}$ $(cm^{-1})$ = 3447 (br, OH), 3071 (w, C=*CH*), 2961 (m, CH), 2932 (m, CH), 1613 (m, C=C), 1514 (m), 1467 (m), 1427 (m), 1248 (s), 1110 (s)

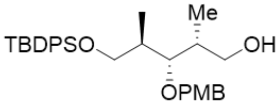
TBDPSO
Me
OH
OPMB

**S44**

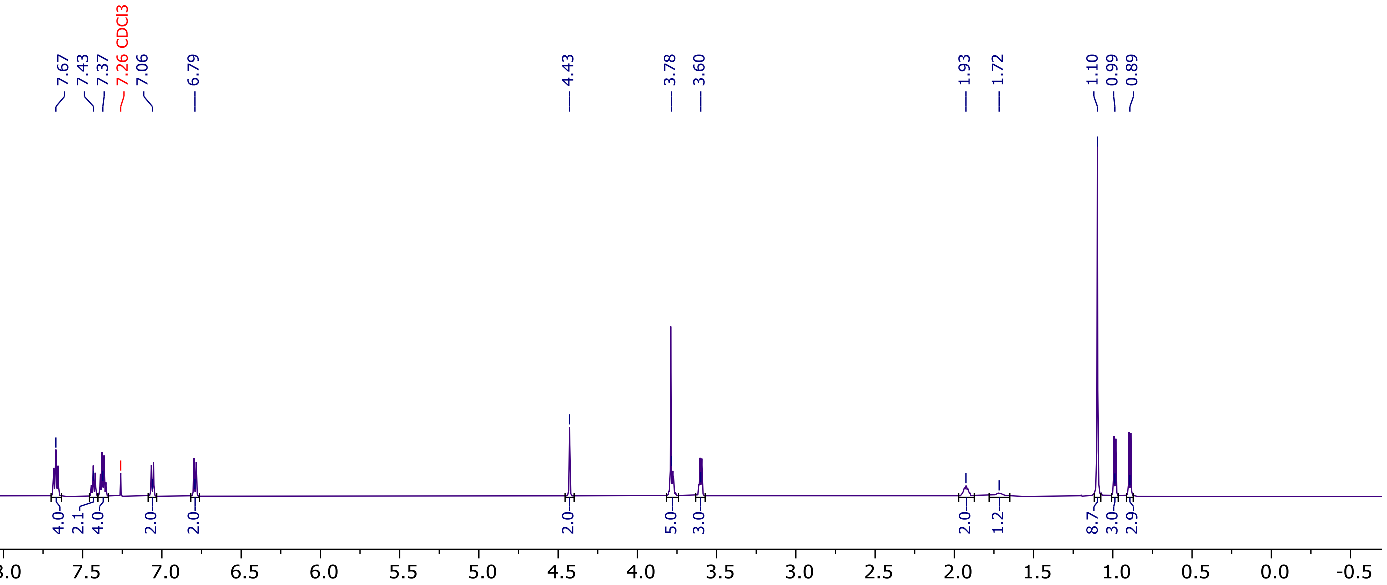
1H
CDCl3
600.13 MHz
7.67
7.43
7.37
7.26 CDCl3
7.06
6.79
4.43
3.78
3.60
1.93
1.72
1.10
0.99
0.89
4.0
2.1
4.0
2.0
2.0
2.0
5.0
3.0
2.0
1.2
8.7
3.0
2.9
10.0
9.5
9.0
8.5
8.0
7.5
7.0
6.5
6.0
5.5
5.0
4.5
4.0
3.5
3.0
2.5
2.0
1.5
1.0
0.5
0.0
-0.5

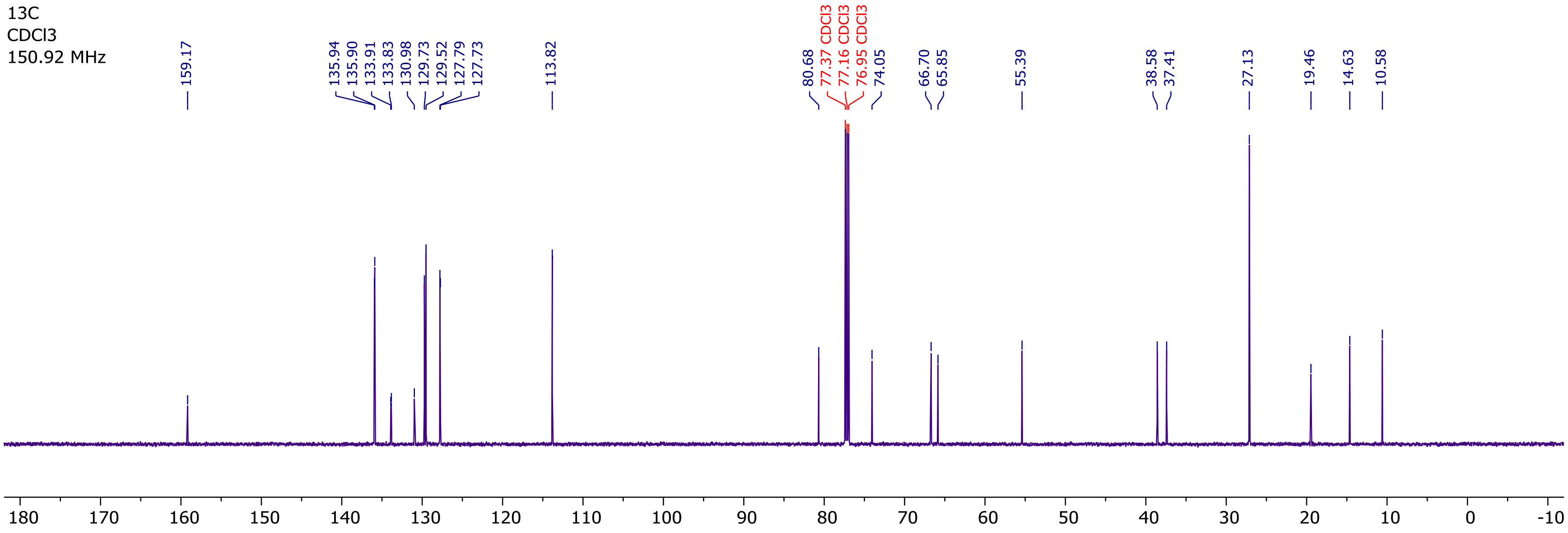
13C
CDCl3
150.92 MHz
159.17
135.94
135.90
133.91
133.83
130.98
129.73
129.52
127.79
127.73
113.82
80.68
77.37 CDCl3
77.16 CDCl3
76.95 CDCl3
74.05
66.70
65.85
55.39
38.58
37.41
27.13
19.46
14.63
10.58
180
170
160
150
140
130
120
110
100
90
80
70
60
50
40
30
20
10
0
-10

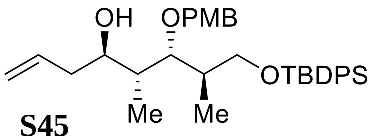


**S45**

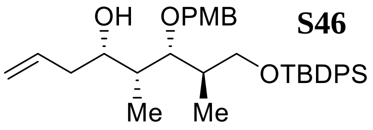


**S46**

To a cold (-78 °C), stirred solution of $(COCl)_2$ (0.61 mL, 0.90 g, 7.1 mmol, 1.3 eq.) in $CH_2Cl_2$ (20 mL) was added anhydrous DMSO (1.0 g, 0.93 mL, 13.1 mmol) [**Caution! $CO_2$ (g) evolved**] over 5 minutes via syringe. Subsequently, **S44** (2.75 g, 5.43 mmol, 1.0 eq,) was added as a solution in $CH_2Cl_2$ (7 mL) over 5 min. The reaction mixture was maintained at -78 °C for 15 min, after which time $Et_3N$ (3.8 mL, 2.8 g, 27 mmol, 5.0 eq.) was added via syringe over 5 min to give a cloudy white suspension. The reaction mixture was maintained at -78 °C for a further 15 min, at which point the $CO_2$(s)/Acetone (-78 °C) bath was exchanged for an ice bath (0 °C) to allow slow warming to 0 °C. After a further 25 min, the starting material was consumed as monitored by TLC analysis. Then the reaction mixture was poured into a separatory funnel and washed with 10% aqueous $CuSO_4$ (2x 50 mL) and the combined aqueous layers were back extracted with $CH_2Cl_2$ (3x 50 mL). The combined organic layers were washed with brine (60 mL), dried ($Na_2SO_4$), filtered, and solvent was removed in vacuo to give the crude aldehyde (2.4 g, 87%) as a colorless oil which was used immediately in the next step without further purification. To a cold (-78 °C), stirred solution of crude aldehyde (2.4 g, 4.8 mmol, 1.0 eq.) in $Et_2O$ (20 mL) was added allyl-MgBr (1.0 M in $Et_2O$, 6.2 mL, 6.2 mmol) via syringe. The reaction mixture was maintained at -78 °C for 2 h 45 min, after which time starting material was consumed as monitored by TLC analysis, and the reaction was quenched by addition of saturated aqueous $NH_4Cl$ (20 mL) via syringe. Then the reaction mixture was warmed to rt, and poured into a separatory funnel. The organic layer was drained, and the aqueous layer was extracted with EtOAc (3x 40 mL). The combined organic layers were dried ($MgSO_4$), filtered, and solvent was removed in vacuo. The crude product was purified via flash column chromatography (9:1 to 87.5:12.5 Hexanes/EtOAc). Appropriate fractions were pooled, and solvent was removed in vacuo to yield **S45** (0.62 g, 21%, 2 steps) and **S46** (1.47 g, 50%, 2 steps) as colorless oils. Spectral and optical rotation data agreed with the data previously reported in the literature for **ent-S45** and **ent-S46.**

**Analytical Data for S45:**

$R_f$ = 0.44 (9:1 Hexanes/EtOAc)

$[\alpha]_D^{20}$ = -20.2° (c = 1.10, $CDCl_3$); lit. $[\alpha]_D^{22}$ = +11.9° (c = 1.0, $CH_2Cl_2$) for **ent-S45**[14]

$^1H$ NMR (500 MHz, $CDCl_3$) δ 7.66 (tt, *J* = 6.6, 1.5 Hz, 4H), 7.45 – 7.39 (m, 2H), 7.39 – 7.33 (m, 4H), 7.06 – 7.01 (m, 2H), 6.80 – 6.74 (m, 2H), 5.91 – 5.79 (m, 1H), 5.21 – 5.16 (m, 1H), 5.15 (s, 1H), 4.53 (d, *J* = 10.8 Hz, 1H), 4.40 (d, *J* = 10.8 Hz, 1H), 3.84 (dd, *J* = 9.2, 2.0 Hz, 1H), 3.78 (s, 3H), 3.80 – 3.73 (m, 2H), 3.58 (td, *J* = 8.1, 3.6 Hz, 1H), 2.46 (dddd, *J* = 14.0, 5.0, 3.7, 2.1 Hz, 1H), 2.37 – 2.08 (m, 2H), 1.93 (dddd, *J* = 12.8, 9.3, 3.7, 1.9 Hz, 1H), 1.76 – 1.67 (m, 1H), 1.09 (s, 9H), 0.97 (d, *J* = 6.9 Hz, 3H), 0.89 (d, *J* = 7.0 Hz, 3H).

$^{13}$C NMR (126 MHz, $CDCl_3$) δ 159.12, 135.95, 135.91, 135.23, 134.04, 133.98, 131.21, 129.69, 129.38, 127.77, 127.72, 118.41, 113.80, 79.61, 73.60, 72.50, 66.12, 55.41, 40.12, 39.75, 38.84, 27.15, 19.49, 14.73, 10.68.

HRMS (ESI): Anal. Calcd. for $C_{34}H_{47}O_4Si^+$ $[M+H]^+$ 547.3238, found 547.3241

IR (neat): $\nu_{max}$ $(cm^{-1})$ = 3465 (br, OH), 3071 (w, C=*CH*), 2960 (m, CH), 2932 (m, CH), 2858 (m, CH), 1613 (m, C=C), 1514 (m), 1427 (m), 1248 (s).

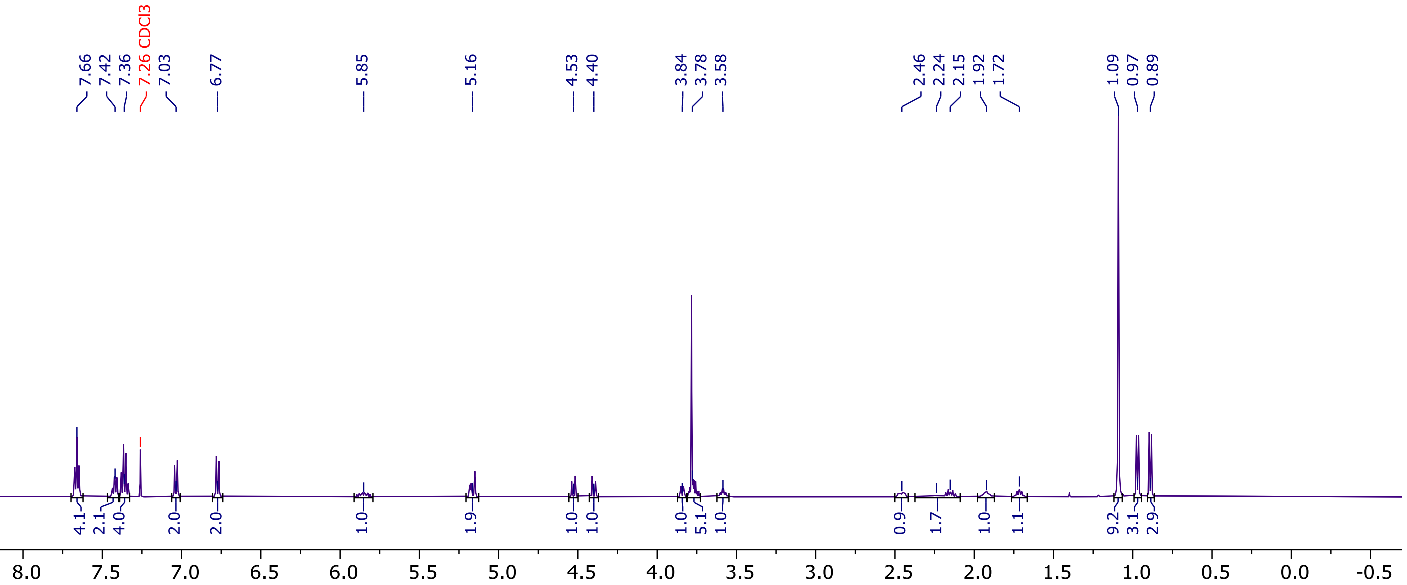

1H
CDCl3
500.14 MHz
7.66
7.42
7.36
7.26 CDCl3
7.03
6.77
5.85
5.16
4.53
4.40
3.84
3.78
3.58
2.46
2.24
2.15
1.92
1.72
1.09
0.97
0.89
4.1
2.1
4.0
2.0
2.0
1.0
1.9
1.0
1.0
1.0
5.1
1.0
0.9
1.7
1.0
1.1
9.2
3.1
2.9
10.0
9.5
9.0
8.5
8.0
7.5
7.0
6.5
6.0
5.5
5.0
4.5
4.0
3.5
3.0
2.5
2.0
1.5
1.0
0.5
0.0
-0.5


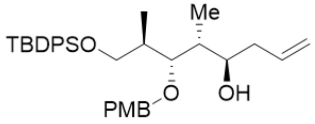

TBDPSO
Me
OH
PMB
O


**S45**

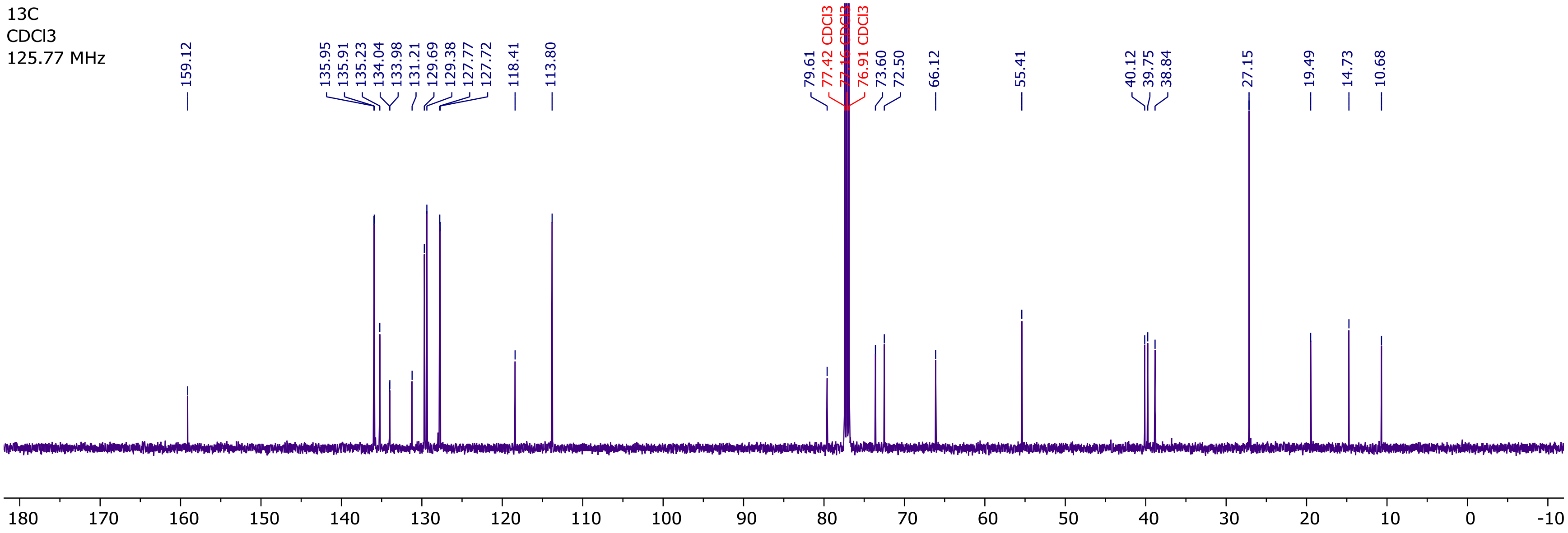

13C
CDCl3
125.77 MHz
159.12
135.95
135.91
135.23
134.04
133.98
131.21
129.69
129.38
127.77
127.72
118.41
113.80
79.61
77.42 CDCl3
77.16 CDCl3
76.91 CDCl3
73.60
72.50
66.12
55.41
40.12
39.75
38.84
27.15
19.49
14.73
10.68
180
170
160
150
140
130
120
110
100
90
80
70
60
50
40
30
20
10
0
-10

**Analytical Data for S46:**

$R_f$ = 0.37 (9:1 Hexanes/EtOAc)

$[\alpha]_D^{20}$ = -21.7° (c = 1.84, $CDCl_3$); lit. $[\alpha]_D^{22}$ = +21.7° (c = 1.0, $CH_2Cl_2$) for **ent-S46**[14]

$^1$H NMR (600 MHz, $CDCl_3$) δ 7.66 (ddt, *J* = 12.8, 6.8, 1.4 Hz, 4H), 7.47 – 7.41 (m, 2H), 7.41 – 7.34 (m, 4H), 7.06 – 6.98 (m, 2H), 6.81 – 6.75 (m, 2H), 5.83 (ddt, *J* = 17.2, 10.2, 7.1 Hz, 1H), 5.13 (dq, *J* = 17.1, 1.6 Hz, 1H), 5.09 (ddt, *J* = 10.2, 2.1, 1.1 Hz, 1H), 4.50 (d, *J* = 10.3 Hz, 1H), 4.44 (d, *J* = 10.3 Hz, 1H), 3.89 (ddd, *J* = 7.9, 5.9, 2.3 Hz, 1H), 3.81 (dd, *J* = 10.0, 5.1 Hz, 1H), 3.79 (s, 3H), 3.74 (dd, *J* = 10.0, 4.2 Hz, 1H), 3.66 (dd, *J* = 8.4, 2.9 Hz, 1H), 3.11 (s, 1H), 2.33 (dtt, *J* = 14.3, 7.2, 1.4 Hz, 1H), 2.21 (dddd, *J* = 12.8, 5.9, 3.5, 1.2 Hz, 1H), 2.04 – 1.95 (m, 1H), 1.78 (dtd, *J* = 9.6, 7.0, 3.5 Hz, 1H), 1.10 (s, 9H), 0.98 (d, *J* = 6.9 Hz, 3H), 0.91 (d, *J* = 7.1 Hz, 3H).

$^{13}$C NMR (151 MHz, $CDCl_3$) δ 159.32, 135.92, 135.89, 135.61, 133.72, 133.67, 130.18, 129.81, 129.71, 127.86, 127.77, 117.28, 113.94, 84.93, 75.13, 73.72, 65.47, 55.39, 39.84, 38.54, 37.86, 27.11, 19.43, 14.34, 6.53.

HRMS (ESI): Anal. Calcd. for $C_{34}H_{47}O_4Si^+$ $[M+H]^+$ 547.3238, found 547.3224

IR ($CDCl_3$): $\nu_{max}$ $(cm^{-1})$ = 3488 (br, OH), 3072 (w, C=*CH*), 2959 (m, CH), 2933 (m, CH), 2858 (m, CH), 1614 (m, C=C), 1541 (m), 1466 (m), 1249 (s).

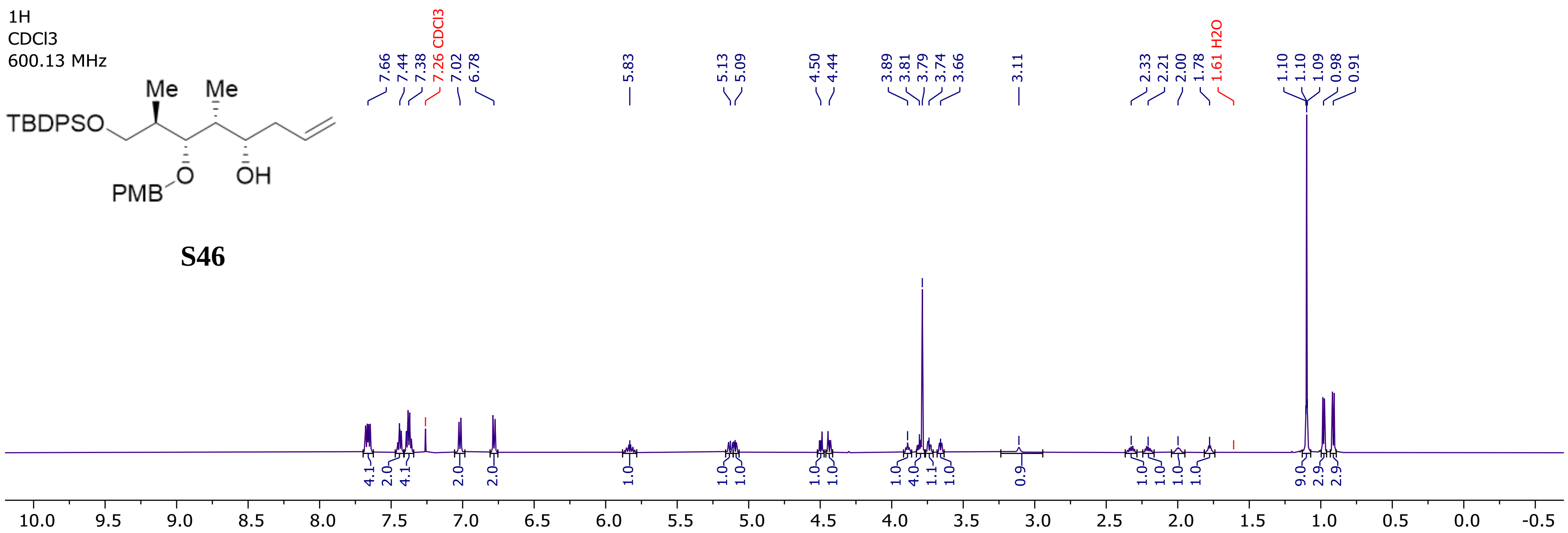
1H
CDCl3
600.13 MHz
TBDPSO
Me
Me
PMB
O
OH
7.66
7.44
7.38
7.26 CDCl3
7.02
6.78
5.83
5.13
5.09
4.50
4.44
3.89
3.81
3.79
3.74
3.66
3.11
2.33
2.21
2.00
1.78
1.61 H2O
1.10
1.10
1.09
0.98
0.91
4.1
2.0
4.1
2.0
2.0
1.0
1.0
1.0
1.0
1.0
1.0
4.0
1.1
1.0
0.9
1.0
1.0
1.0
1.0
9.0
2.9
2.9
10.0
9.5
9.0
8.5
8.0
7.5
7.0
6.5
6.0
5.5
5.0
4.5
4.0
3.5
3.0
2.5
2.0
1.5
1.0
0.5
0.0
-0.5

**S46**

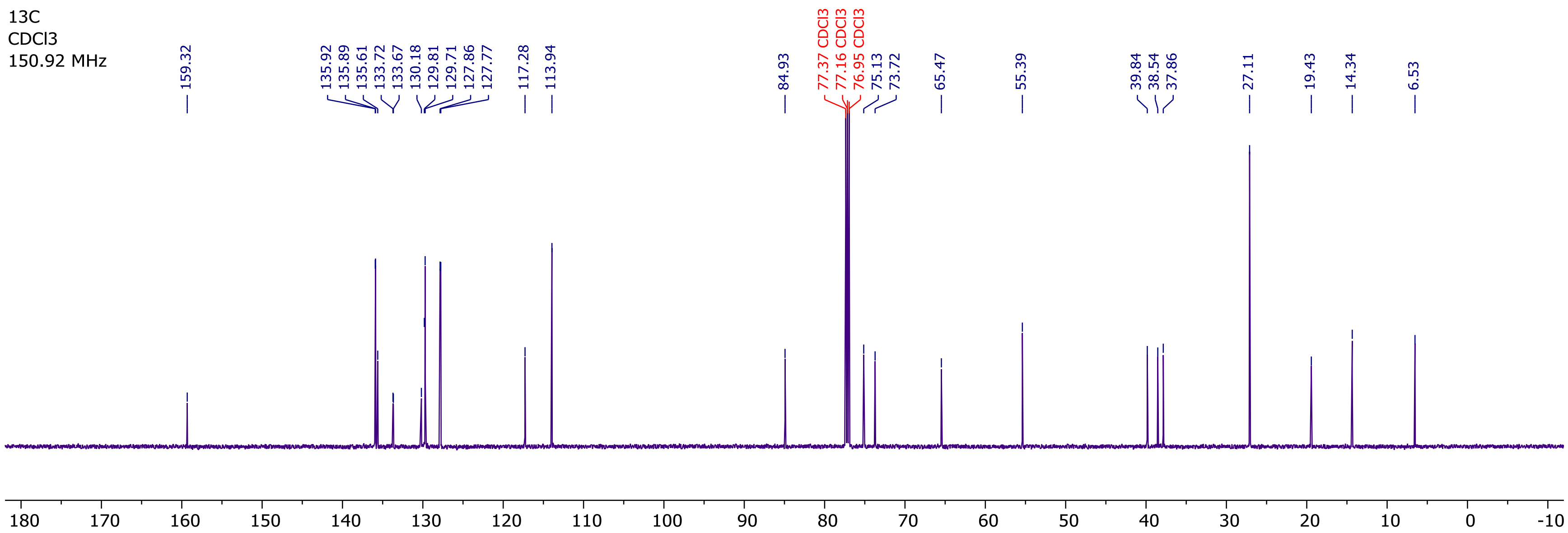
13C
CDCl3
150.92 MHz
159.32
135.92
135.89
135.61
133.72
133.67
130.18
129.81
129.71
127.86
127.77
117.28
113.94
84.93
77.37 CDCl3
77.16 CDCl3
76.95 CDCl3
75.13
73.72
65.47
55.39
39.84
38.54
37.86
27.11
19.43
14.34
6.53
180
170
160
150
140
130
120
110
100
90
80
70
60
50
40
30
20
10
0
-10

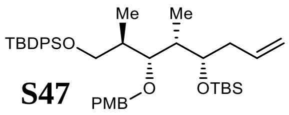

**S47**

To a cold (-20 °C), stirred solution of **S46** (1.20 g, 2.20 mmol, 1 eq.) in $CH_2Cl_2$ (15 mL) was added in sequence 2,6-lutidine (0.76 mL, 0.70 g, 6.6 mmol, 3 eq.) and TBSOTf (0.76 mL, 0.87 g, 3.3 mmol, 1.5 eq.). The reaction mixture was allowed to slowly warm to rt over 16 h. After this time starting material was consumed as monitored by TLC analysis. The reaction mixture was quenched by addition of a saturated aqueous solution of $NaHCO_3$ (50 mL). The reaction mixture was poured into a separatory funnel and the aqueous extracted with $CH_2Cl_2$ (3x 30 mL). The combined organic layers were dried ($Na_2SO_4$), filtered, and solvent removed in vacuo. The crude product was purified via flash column chromatography (24:1 Hexanes/$Et_2O$). Appropriate fractions were pooled and solvent was removed in vacuo to yield **S47** (1.41 g, 97%) as a colorless oil.

**Analytical Data for S47:**

$R_f$ = 0.80 (9:1 Hexanes/EtOAc)

$[\alpha]_D^{20}$ = -6.44° (c = 2.05, $CDCl_3$)

$^1$H NMR (500 MHz, $CDCl_3$) δ 7.67 (ddt, *J* = 10.8, 6.7, 1.4 Hz, 4H), 7.46 – 7.40 (m, 2H), 7.39 – 7.34 (m, 4H), 7.10 – 7.05 (m, 2H), 6.83 – 6.78 (m, 2H), 5.74 (ddt, *J* = 17.3, 10.2, 7.1 Hz, 1H), 5.06 – 4.97 (m, 2H), 4.44 (q, *J* = 10.8 Hz, 2H), 3.80 (s, 3H), 3.79 – 3.76 (m, 1H), 3.73 (dd, *J* = 9.8, 3.5 Hz, 1H), 3.69 (dt, *J* = 6.6, 5.2 Hz, 1H), 3.52 (dd, *J* = 7.3, 4.0 Hz, 1H), 2.37 – 2.26 (m, 2H), 1.92 (qd, *J* = 6.8, 3.4 Hz, 1H), 1.77 (pd, *J* = 6.8, 3.9 Hz, 1H), 1.10 (s, 9H), 1.02 (d, *J* = 6.9 Hz, 3H), 0.92 (s, 9H), 0.90 (d, *J* = 7.1 Hz, 3H), 0.06 (s, 3H), 0.05 (s, 3H).

$^{13}$C NMR (126 MHz, $CDCl_3$) δ 159.06, 135.92, 135.90, 135.19, 134.07, 134.03, 131.49, 129.69, 129.68, 129.16, 127.77, 127.70, 116.99, 113.80, 80.76, 74.15, 73.31, 65.54, 55.42, 39.64, 39.40, 39.07, 27.15, 26.14, 19.49, 18.31, 15.26, 9.98, -3.91, -4.29.

HRMS (ESI): Anal. Calcd. for $C_{40}H_{61}O_4Si_2^+$ $[M+H]^+$ 661.4103, found 661.4149

IR (neat): $\nu_{max}$ $(cm^{-1})$ = 3072 (w, C=*CH),* (3050, w), 2956 (m, CH), 2931 (m, CH), 2888 (m, CH), 2857 (m, CH), 1614 (m, C=C), 1514 (m), 1467 (m), 1429 (m), 1389 (m), 1301 (m), 1250 (s).

1H
CDCl3
500.14 MHz

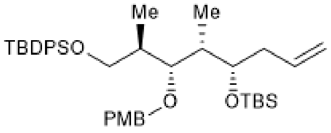


**S47**

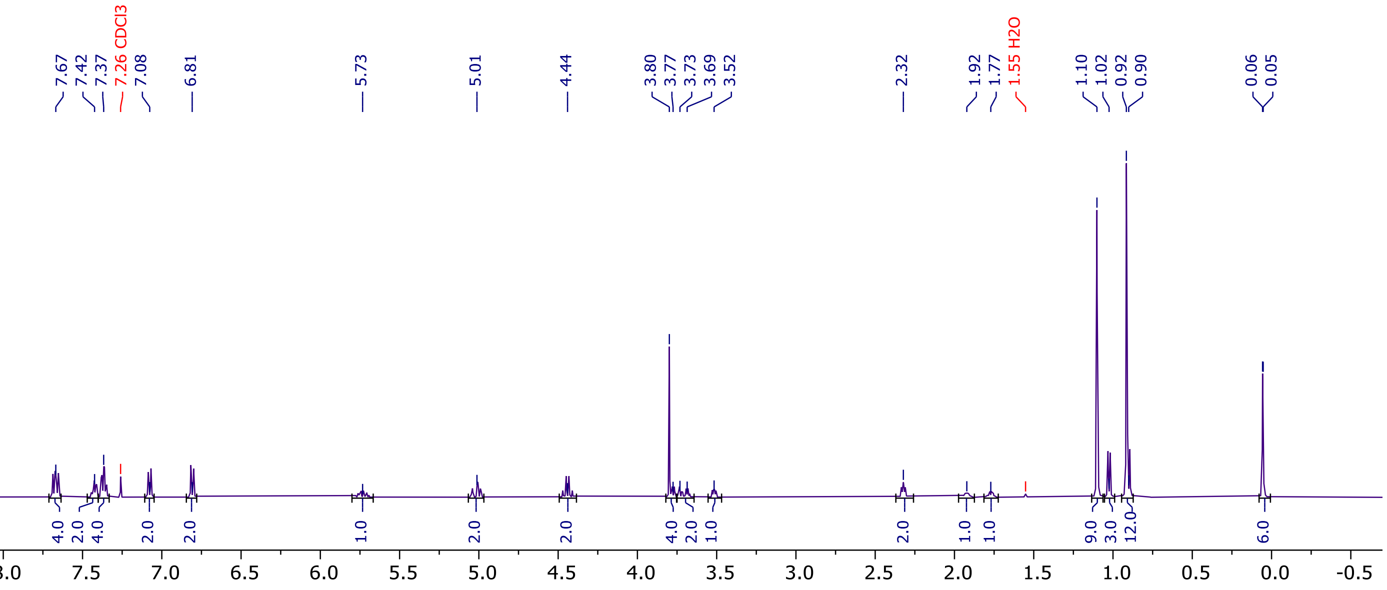


13C
CDCl3
125.77 MHz

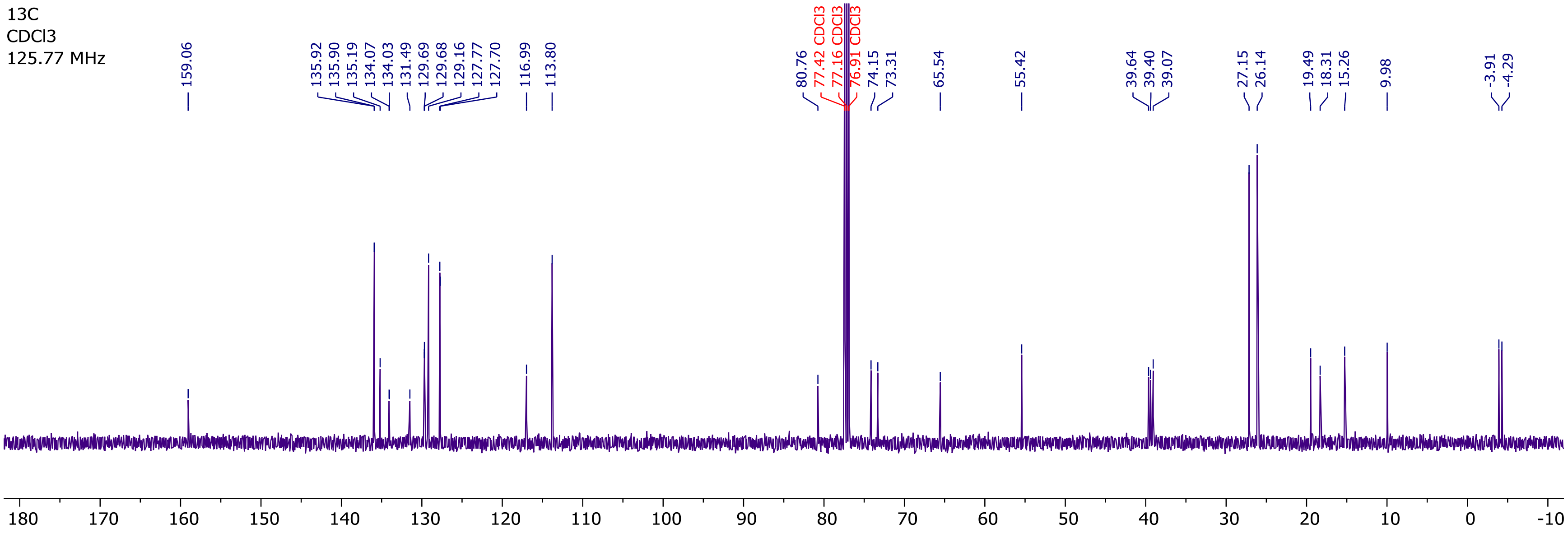

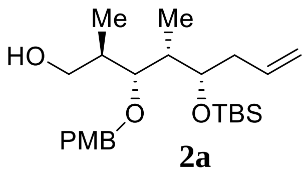

**2a**

Reaction was conducted in a sealed 60 mL polypropylene tube under ambient atmosphere. To a warm (60 °C), stirred solution of **S47** (1.41 g, 2.13 mmol, 1 eq.) in MeOH (14.25 mL) was added $NH_4F$ (396.3 mg, 10.7 mmol, 5 eq.) and the reaction mixture was maintained at 60 °C for 2 h 50 min. After this time, further $NH_4F$ (400 mg, 10.8 mmol, 5.1 eq.) was added, and the reaction mixture maintained at 60 °C for an additional 21 h 30 min. After this time further $NH_4F$ (412 mg, 11.1 mmol, 5.2 eq.) was added, and the reaction mixture maintained at 60 °C for an additional 2 d. After this time, starting material was consumed as monitored by TLC analysis and the reaction mixture was removed from the heating bath. After cooling to ambient temperature, the reaction mixture was poured onto a saturated aqueous solution of $NaHCO_3$ (100 mL) in a separatory funnel and extracted with $CH_2Cl_2$ (3x 70 mL). The combined organic layers were washed with brine (1x 50 mL), dried $Na_2SO_4$, filtered, and solvent was removed in vacuo. The crude product was purified via flash column chromatography (9:1 Hexanes/EtOAc). Appropriate fractions were pooled, and solvent removed in vacuo to yield **2a** (713 mg, 79%) as a colorless oil.

**Analytical Data for 2a:**

$R_f$ = 0.14 (9:1 Hexanes/EtOAc)

$[\alpha]_D^{20}$ = +6.8° (c = 2.25, $CDCl_3$)

$^1$H NMR (600 MHz, $CDCl_3$) δ 7.28 – 7.23 (m, 2H), 6.89 – 6.84 (m, 2H), 5.74 (ddt, *J* = 17.3, 10.2, 7.2 Hz, 1H), 5.10 – 5.01 (m, 2H), 4.52 (s, 2H), 3.79 (s, 3H), 3.76 (ddd, *J* = 11.0, 4.8, 3.4 Hz, 1H), 3.70 (dt, *J* = 6.9, 4.9 Hz, 1H), 3.57 (ddd, *J* = 11.2, 6.3, 5.1 Hz, 1H), 3.44 (dd, *J* = 6.3, 4.8 Hz, 1H), 2.81 (dd, *J* = 6.3, 4.8 Hz, 1H), 2.33 (dtt, *J* = 14.1, 7.0, 1.2 Hz, 1H), 2.28 (dddd, *J* = 14.0, 7.1, 4.0, 1.2 Hz, 1H), 1.97 – 1.86 (m, 2H), 1.07 (d, *J* = 7.0 Hz, 3H), 1.01 (d, *J* = 6.9 Hz, 3H), 0.91 (s, 9H), 0.06 (s, 3H), 0.05 (s, 3H).

$^{13}$C NMR (151 MHz, $CDCl_3$) δ 159.38, 134.83, 130.57, 129.48, 117.39, 113.98, 85.65, 75.31, 72.96, 65.43, 55.40, 40.54, 39.59, 37.12, 26.06, 18.27, 15.75, 10.04, -3.75, -4.39.

HRMS (ESI): Anal. Calcd. for $C_{24}H_{43}O_4Si^+$ $[M+H]^+$ 423.2925, found 423.2938

IR (neat): $\nu_{max}$ $(cm^{-1})$ = 3419 (br, OH), 3075 (w, C=*CH*), 2955 (m, CH), 2857 (m, CH), 1641 (m, C=C), 1465 (m), 1250 (s).

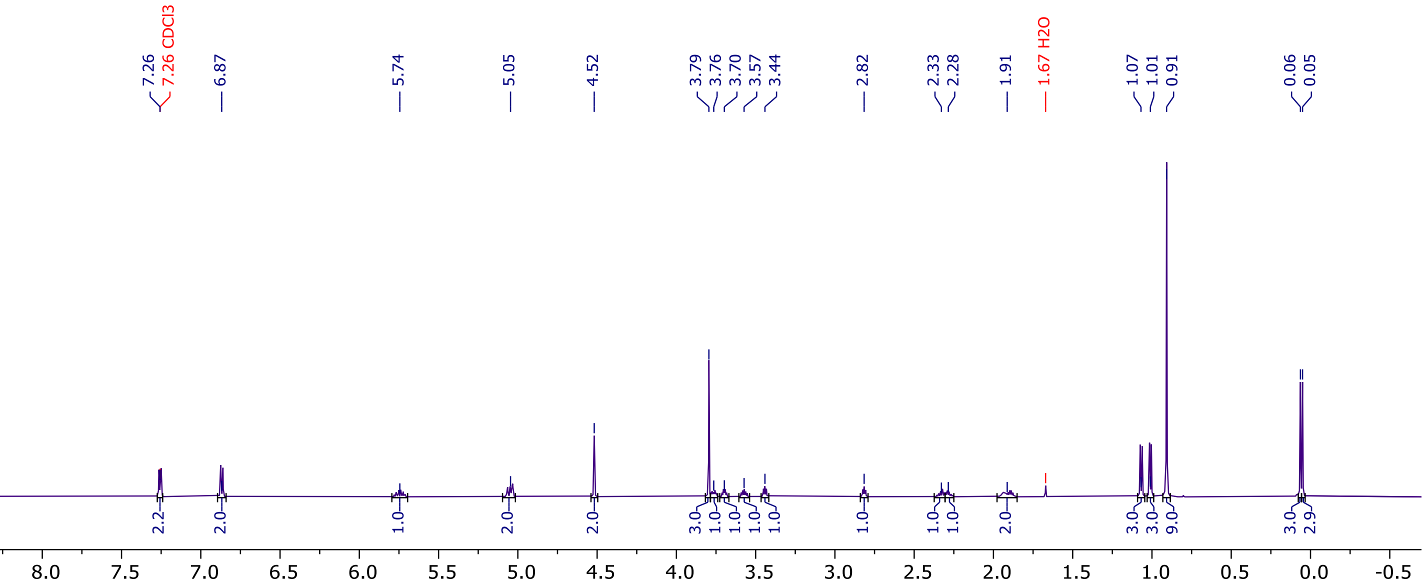
1H
CDCl3
600.13 MHz
7.26
7.26 CDCl3
6.87
5.74
5.05
4.52
3.79
3.76
3.70
3.57
3.44
2.82
2.33
2.28
1.91
1.67 H2O
1.07
1.01
0.91
0.06
0.05
2.2
2.0
1.0
2.0
2.0
3.0
1.0
1.0
1.0
1.0
1.0
1.0
1.0
2.0
3.0
3.0
9.0
3.0
2.9
10.0
9.5
9.0
8.5
8.0
7.5
7.0
6.5
6.0
5.5
5.0
4.5
4.0
3.5
3.0
2.5
2.0
1.5
1.0
0.5
0.0
-0.5

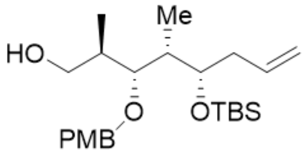
HO
Me
O
PMB
OTBS

**2a**

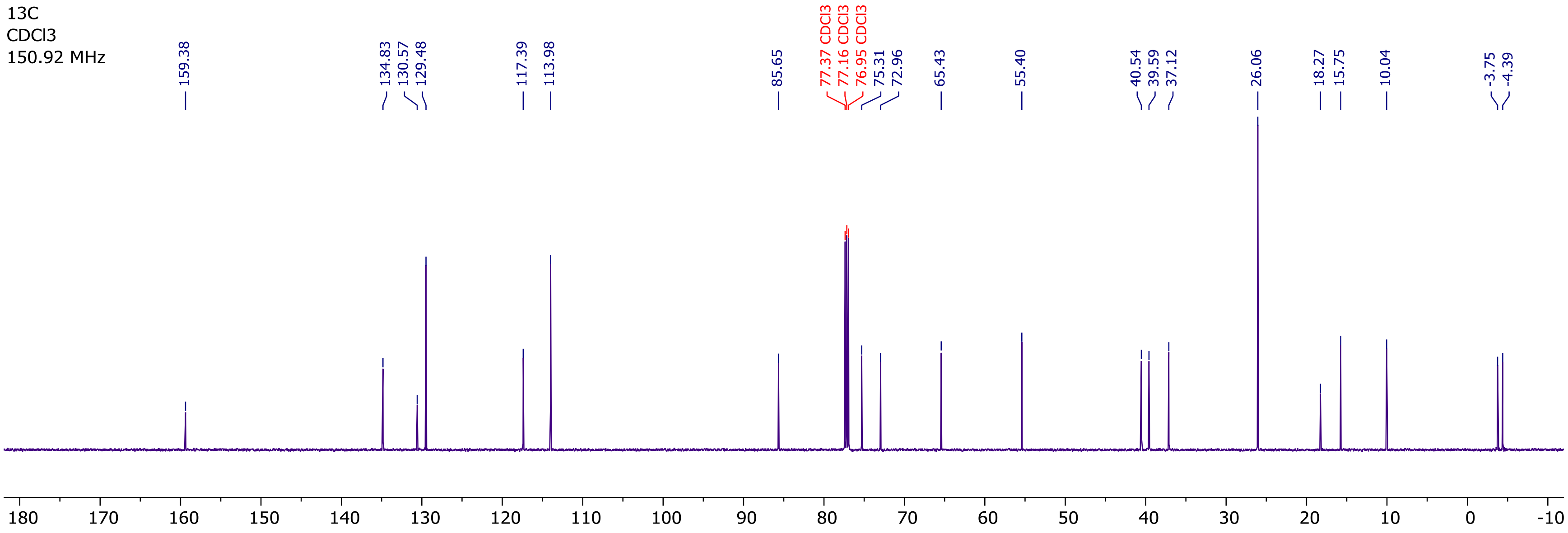
13C
CDCl3
150.92 MHz
159.38
134.83
130.57
129.48
117.39
113.98
85.65
77.37 CDCl3
77.16 CDCl3
76.95 CDCl3
75.31
72.96
65.43
55.40
40.54
39.59
37.12
26.06
18.27
15.75
10.04
-3.75
-4.39
180
170
160
150
140
130
120
110
100
90
80
70
60
50
40
30
20
10
0
-10

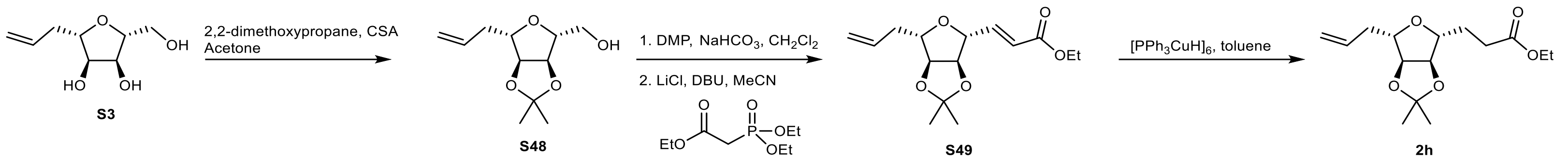


**Supplementary Fig. 32 | Synthesis of Tetrahydrofuranoate 2h.**
Abbreviations: CSA = camphorsulfonic acid, DMP = Dess-Martin periodinane, DBU = 1,8-Diazabicyclo [5.4.0]undec-7-ene.

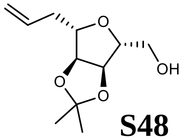
**S48**

**S48** was produced as a side product of the sequence shown in **Supplementary Fig. 26.** Alternatively, **S48** could be prepared as follows. **S3** (480 mg, 1.91 mmol, 1.0 eq.) was dissolved in MeOH (30 mL) and heated to 120 °C in a microwave reactor for 90 min. After this time, the reaction mixture was cooled to rt, transferred to a flask, and solvent was removed in vacuo. The crude residue was azeotroped from toluene (1x 25 mL). To a rt, stirred solution of the crude product (360 mg, *ca*. 1.91 mmol) in acetone (10 mL) and 2,2-dimethoxypropane (2.35 mL, 2.00 g, 19.1 mmol, 10 eq.) was added CSA (443.7 mg, 1.91 mmol, 1.0 eq.) in one portion. After stirring for 1 h at rt, starting material was consumed as monitored by TLC analysis. The reaction was quenched by addition of saturated aqueous $NaHCO_3$ (10 mL). The reaction mixture was then concentrated in vacuo to *ca.* 5 mL, and the aqueous concentrate was poured into a separatory funnel and extracted with $CH_2Cl_2$ (3x 10 mL). The combined organic layers were dried ($Na_2SO_4$), filtered, and solvent was removed in vacuo. The crude product was purified via flash column chromatography (3:1 Hexanes/EtOAc). Appropriate fractions were pooled, and solvent was removed in vacuo to yield **S48** (275 mg, 67%) as a pale-yellow oil. Spectral and optical rotation data agreed with the data previously reported in the literature for **ent-S48**.[15]

**Analytical Data for S48:**

$R_f$ = 0.29 (3:1 Hexanes/EtOAc)

$[\alpha]_D^{20}$ = -4.2° (c = 0.60, $CH_2Cl_2$); $[\alpha]_D^{24}$ = +5.8° (c = 1.00, $CH_2Cl_2$) for **ent-S48**[15]

$^1$H NMR (600 MHz, $CDCl_3$) δ 5.83 (ddt, *J* = 17.1, 10.2, 6.9 Hz, 1H), 5.17 (dq, *J* = 17.2, 1.7 Hz, 1H), 5.14 (ddt, *J* = 10.2, 2.1, 1.2 Hz, 1H), 4.61 (dd, *J* = 7.0, 4.5 Hz, 1H), 4.36 (dd, *J* = 6.9, 4.9 Hz, 1H), 4.04 – 3.95 (m, 2H), 3.84 (ddd, *J* = 11.9, 4.9, 3.2 Hz, 1H), 3.67 (ddd, *J* = 12.1, 7.9, 4.3 Hz, 1H), 2.48 – 2.34 (m, 2H), 1.81 (td, *J* = 5.5, 4.9, 1.5 Hz, 1H), 1.54 (s, 3H), 1.34 (s, 3H).

$^{13}$C NMR (151 MHz, $CDCl_3$) δ 133.51, 118.26, 114.77, 84.21, 84.19, 83.68, 81.42, 62.90, 37.82, 27.53, 25.60.

HRMS (ESI): Anal. Calcd. for $C_{11}H_{19}O_4^+$ $[M+H]^+$ 215.1278, found 215.1286

IR (neat): $\nu_{max}$ ($cm^{-1}$) = 3459 (br, OH), 3079 (w, C=*CH*), 2986 (m, CH), 2935 (m, CH), 1642 (w, C=C), 1378 (m), 1249 (s, CO), 1211 (s).

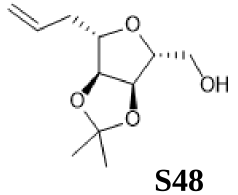

**S48**

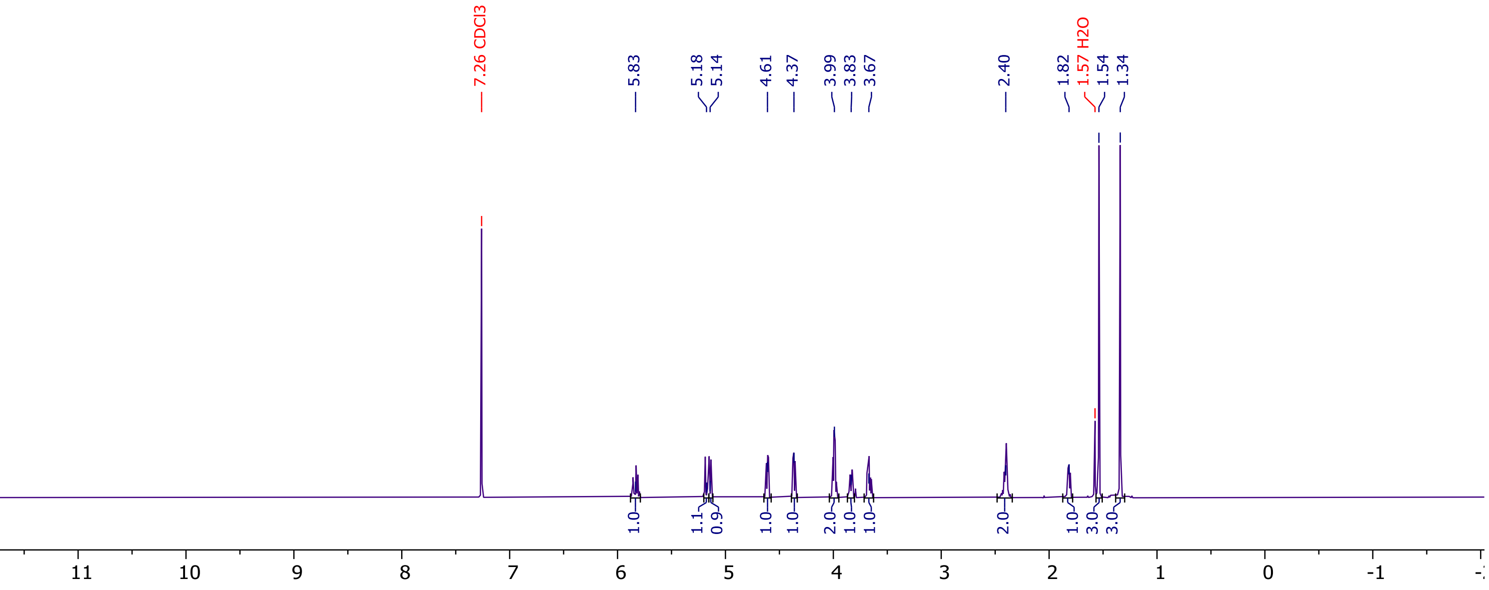
1H
CDCl3
600.13 MHz
7.26 CDCl3
5.83
5.18
5.14
4.61
4.37
3.99
3.83
3.67
2.40
1.82
1.57 H2O
1.54
1.34
1.0
1.1
0.9
1.0
1.0
2.0
1.0
1.0
2.0
1.0
3.0
3.0
13
12
11
10
9
8
7
6
5
4
3
2
1
0
-1

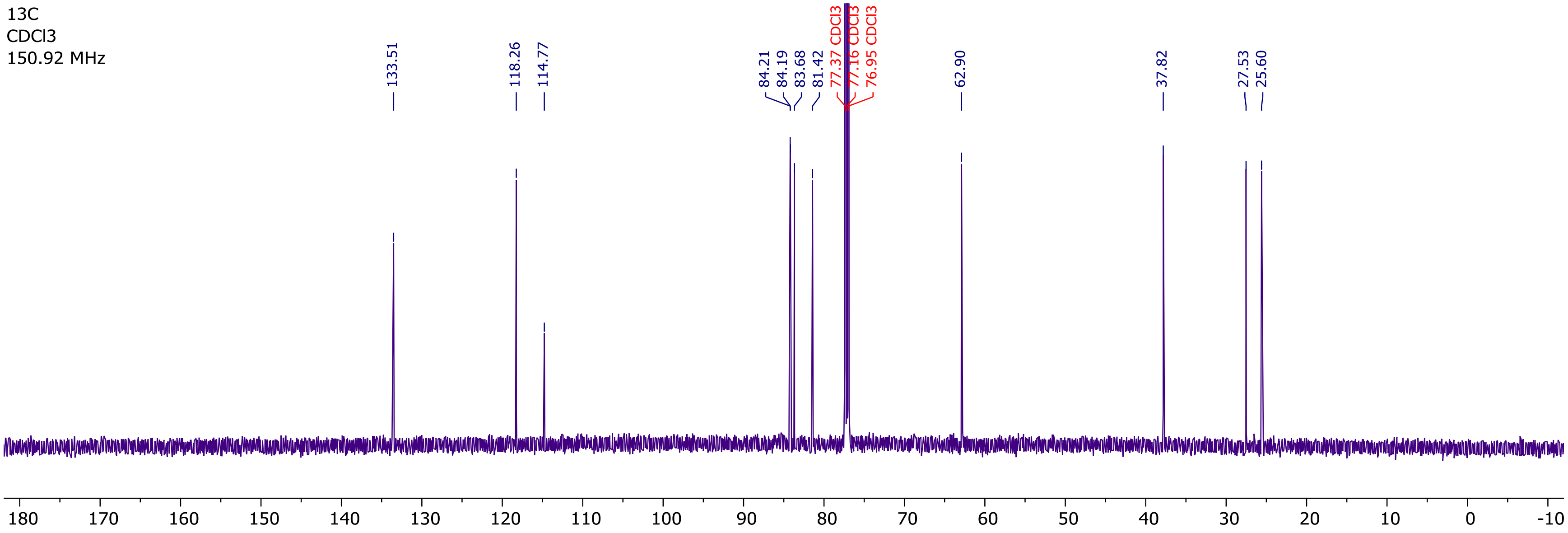
13C
CDCl3
150.92 MHz
133.51
118.26
114.77
84.21
84.19
83.68
81.42
77.37 CDCl3
77.16 CDCl3
76.95 CDCl3
62.90
37.82
27.53
25.60
180
170
160
150
140
130
120
110
100
90
80
70
60
50
40
30
20
10
0
-10

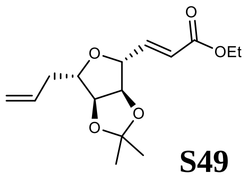


**S49**

To a cold (0 °C), stirred suspension of **S48** (506.5 mg, 2.37 mmol, 1.0 eq.) and $NaHCO_3$ (594.7 mg, 7.08 mmol, 3.0 eq.) in $CH_2Cl_2$ (25 mL) was added Dess-Martin periodinane (1.20 g, 2.83 mmol, 1.2 eq.). The cold bath was removed and the reaction was stirred for 1 h 40 min at rt. After this time, the starting material was consumed as monitored by TLC analysis. The reaction mixture was quenched by addition of a mixture of $H_2O$, saturated aqueous $NaHCO_3$, and 10% aqueous $Na_2S_2O_3$ (1:1:1 (v/v/v), 45 mL). The cloudy mixture was stirred vigorously for 1 h 25 min, after which time two clear layers formed. The biphasic mixture was poured into a separatory funnel, the organic layer separated, and the aqueous layer extracted with $CH_2Cl_2$ (3x 30 mL). The combined organic layers were dried ($Na_2SO_4$), filtered, and solvent removed in vacuo to give the crude aldehyde (452 mg, 2.13 mmol) which was used in the subsequent step without further purification.

To a cold (0 °C), stirred suspension of flame dried LiCl (200.1 mg, 4.72 mmol, 2.2 eq), triethylphosphonoacetate (0.70 mL, 790 mg, 3.5 mmol, 1.6 eq.), and DBU (0.53 mL, 530 mg, 3.48 mmol, 1.6 eq.) in MeCN (12 mL) was added the crude aldehyde (452 mg, 2.13 mmol, 1.0 eq) in additional MeCN (2 mL + 2 mL rinse) via syringe. The reaction mixture was allowed to slowly warm to room temperature over the course of 1 h. After this time, starting material was consumed as monitored by TLC analysis, and the reaction mixture was quenched by addition of a saturated aqueous solution of $NH_4Cl$ (20 mL). The biphasic mixture was poured into a separatory funnel and extracted with $CH_2Cl_2$ (2x 30 mL). The combined organic layers were dried ($Na_2SO_4$), filtered, and solvent was removed in vacuo. The crude product was purified via flash column chromatography (87.5:12.5 to 85:15 Hexanes:$Et_2O$). Appropriate fractions were pooled, and solvent was removed in vacuo to yield **S49** (398 mg, 68%, 2 steps) as a colorless oil.

**Analytical Data for S49:**

$R_f$ = 0.45 (8:2 Hexanes/$Et_2O$)

$[\alpha]_D^{20}$ = +33.5° (c = 2.00, $CDCl_3$)

$^1$H NMR (600 MHz, $CDCl_3$) δ 6.97 (ddd, *J* = 15.8, 3.6, 1.2 Hz, 1H), 6.10 (dd, *J* = 15.7, 1.5 Hz, 1H), 5.82 (ddt, *J* = 17.1, 10.3, 6.9 Hz, 1H), 5.17 (dq, *J* = 17.2, 1.6 Hz, 1H), 5.13 (ddd, *J* = 10.3, 2.0, 1.1 Hz, 1H), 4.45 – 4.42 (m, 2H), 4.40 (ddd, *J* = 6.3, 4.3, 2.3 Hz, 1H), 4.19 (qd, *J* = 7.1, 1.2 Hz, 2H), 4.04 (td, *J* = 6.4, 4.0 Hz, 1H), 2.39 (tq, *J* = 6.6, 1.5 Hz, 2H), 1.54 (s, 3H), 1.32 (s, 3H), 1.28 (t, *J* = 7.1 Hz, 3H).

$^{13}$C NMR (151 MHz, $CDCl_3$) δ 166.18, 144.65, 133.36, 122.00, 118.25, 115.13, 84.66, 84.14, 83.79, 83.03, 60.67, 37.87, 27.48, 25.59, 14.35.

HRMS (ESI): Anal. Calcd. for $C_{15}H_{26}NO_5^+$ $[M+NH_4]^+$ 300.1806, found 300.1812

IR (neat): $\nu_{max}$ $(cm^{-1})$ = 3079 (w, C=*CH*), 2985 (m, CH), 2936 (m, CH), 1722 (s, C=O), 1662 (m, C=C), 1454 (w), 1375 (m), 1301 (s), 1266 (s), 1212 (s).

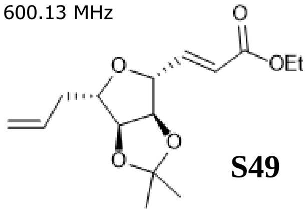
O
OEt
O
O
O

**S49**

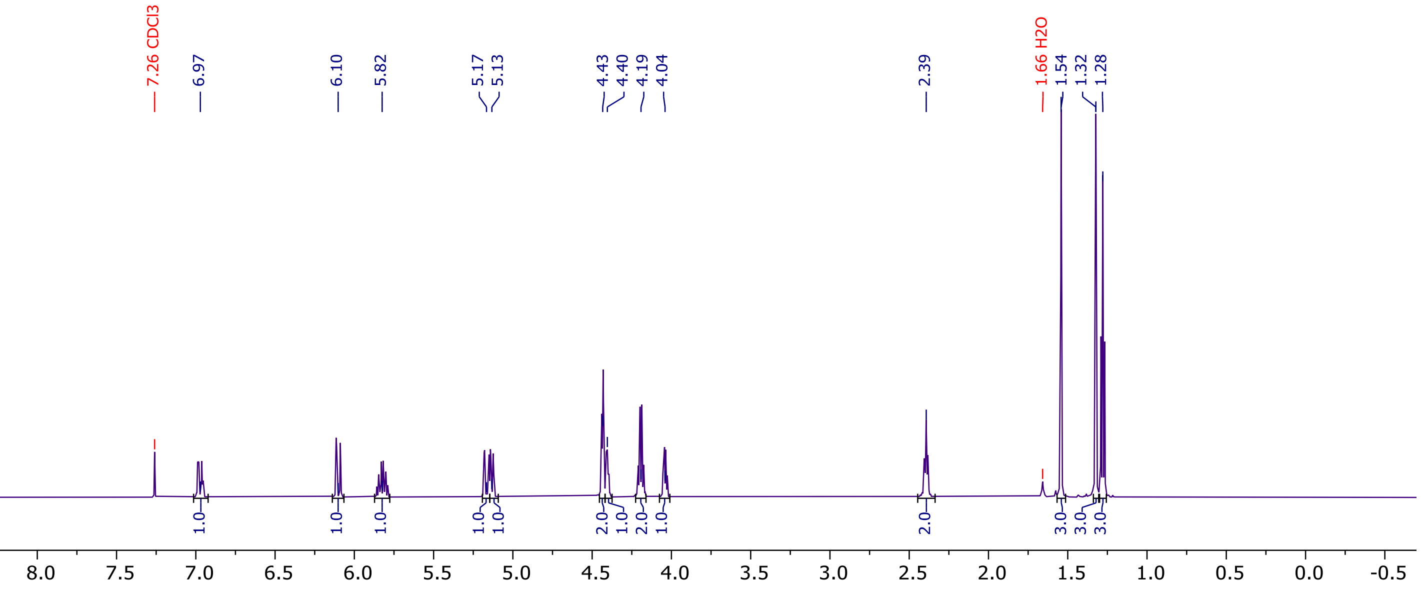
1H
CDCl3
600.13 MHz
7.26 CDCl3
6.97
6.10
5.82
5.17
5.13
4.43
4.40
4.19
4.04
2.39
1.66 H2O
1.54
1.32
1.28
1.0
1.0
1.0
1.0
1.0
2.0
1.0
2.0
1.0
2.0
3.0
3.0
3.0
10.0 9.5 9.0 8.5 8.0 7.5 7.0 6.5 6.0 5.5 5.0 4.5 4.0 3.5 3.0 2.5 2.0 1.5 1.0 0.5 0.0 -0.5

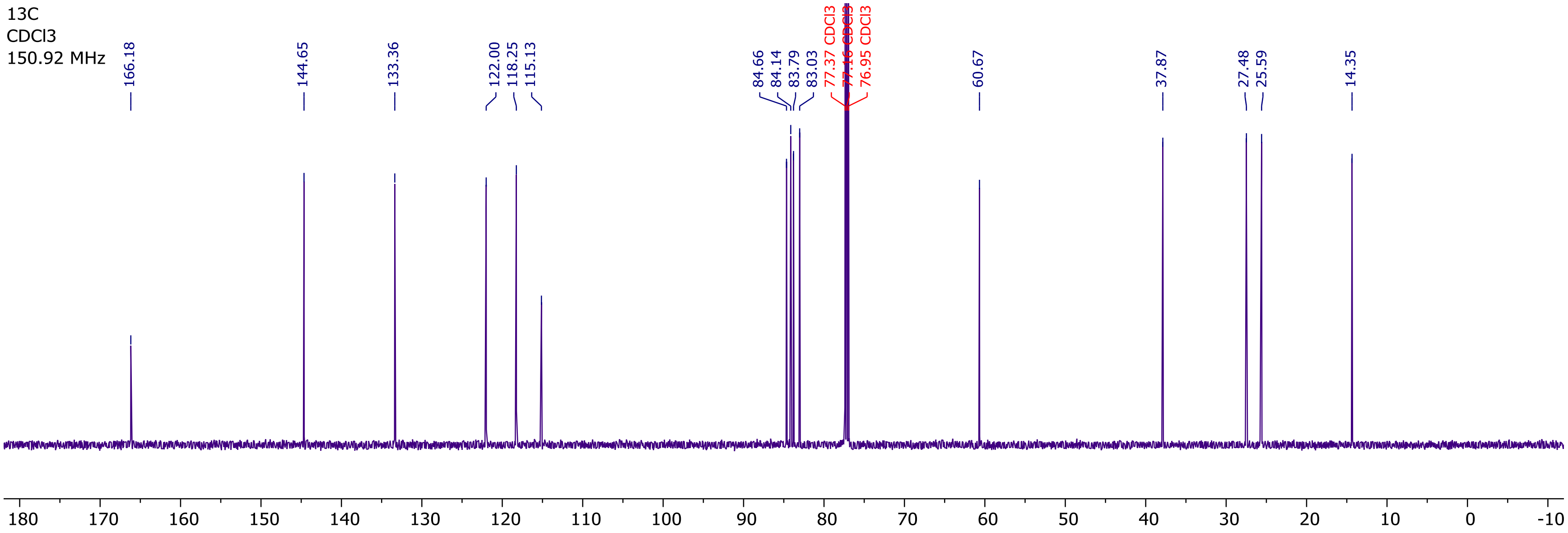
13C
CDCl3
150.92 MHz
166.18
144.65
133.36
122.00
118.25
115.13
84.66
84.14
83.79
83.03
77.37 CDCl3
77.16 CDCl3
76.95 CDCl3
60.67
37.87
27.48
25.59
14.35
180 170 160 150 140 130 120 110 100 90 80 70 60 50 40 30 20 10 0 -10

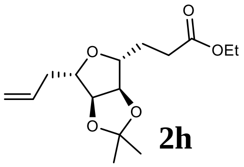
**2h**

To a rt, stirred suspension of $Cu(OAc)_2 \cdot H_2O$ (125 mg, 0.63 mmol) and $PPh_3$ (325 mg, 1.24 mmol) in deoxygenated toluene (30 min. $N_2$(g) sparge) (23.5 mL) was added 1,1,3,3-tetramethyldisiloxane (1.65 mL, 1.25 g, 9.31 mmol) via syringe. The teal-colored suspension was allowed to stir for 24 h. After this time the suspension turned into a homogenous brick-red solution, indicating the presence of $[(PPh_3)CuH]_6$. To neat **S48** (550 mg, 2.22 mmol, 1.0 eq.) was added a solution of the previously prepared reagent $[(PPh_3)CuH]_6$ (16.5 mL, *ca.* 0.4 M in organosilane, 6.6 mmol, 3 eq.). After stirring at rt for 5 h, the reaction mixture was poured onto a silica gel column and the products eluted with $Et_2O$. The solvent was removed in vacuo, and $^1$H NMR analysis of the crude indicated complete conversion. To remove silylated impurities, the crude products were dissolved in THF (8 mL) and treated with HF•Pyr (1.65 mL, 1.82 g, 70:30 (w/w) HF, 63.5 mmol, 29 eq.) and allowed to stir at rt for 21 h 30 min. After this time, the reaction mixture was pipetted onto a biphasic mixture of $Et_2O$ (50 mL) and saturated aqueous $NaHCO_3$ (70 mL) [**caution! $CO_2$ gas evolved**]. After bubbling had stopped, the aqueous layer was extracted with $Et_2O$ (3x 70 mL). The combined organic layers were dried ($MgSO_4$), filtered, and solvent removed in vacuo. The crude product was purified via flash column chromatography (82.5:17.5 Hexanes/$Et_2O$). Appropriate fractions were pooled, and solvent was removed in vacuo to yield **2h** (174 mg, 32%) as a colorless oil.

**Analytical Data for 2h:**

$R_f$ = 0.22 (92.5:7.5 Hexanes/EtOAc)

$[\alpha]_D^{20}$ = +12° (c = 0.96, $CDCl_3$)

$^1$H NMR (600 MHz, $CDCl_3$) δ 5.81 (ddt, *J* = 17.1, 10.2, 6.9 Hz, 1H), 5.14 (dq, *J* = 17.2, 1.6 Hz, 1H), 5.11 (ddt, *J* = 10.2, 2.1, 1.2 Hz, 1H), 4.35 (dd, *J* = 7.1, 4.6 Hz, 1H), 4.29 (dd, *J* = 7.1, 4.9 Hz, 1H), 4.12 (q, *J* = 7.1 Hz, 2H), 3.89 (td, *J* = 6.4, 4.5 Hz, 1H), 3.80 (dt, *J* = 7.8, 5.2 Hz, 1H), 2.49 – 2.37 (m, 2H), 2.36 (ddt, *J* = 6.7, 5.5, 1.2 Hz, 2H), 1.97 (dddd, *J* = 13.8, 9.4, 6.4, 5.5 Hz, 1H), 1.87 (dddd, *J* = 13.9, 9.3, 7.9, 6.1 Hz, 1H), 1.51 (s, 3H), 1.32 (s, 3H), 1.25 (t, *J* = 7.1 Hz, 3H).

$^{13}$C NMR (151 MHz, $CDCl_3$) δ 173.27, 133.72, 117.89, 114.92, 84.86, 84.37, 83.32, 83.09, 60.56, 38.00, 30.64, 28.84, 27.49, 25.63, 14.36.

HRMS (ESI): Anal. Calcd. for $C_{15}H_{25}O_5^+$ [M+H]$^+$ 285.1697, found 285.1700

IR (neat): $\nu_{max}$ $(cm^{-1})$ = 3079 (w, C=*CH*), 2984 (m, CH), 2935 (m, CH) 1735 (s, C=O), 1644 (w, C=C), 1445 (m), 1376 (s), 1211 (s).

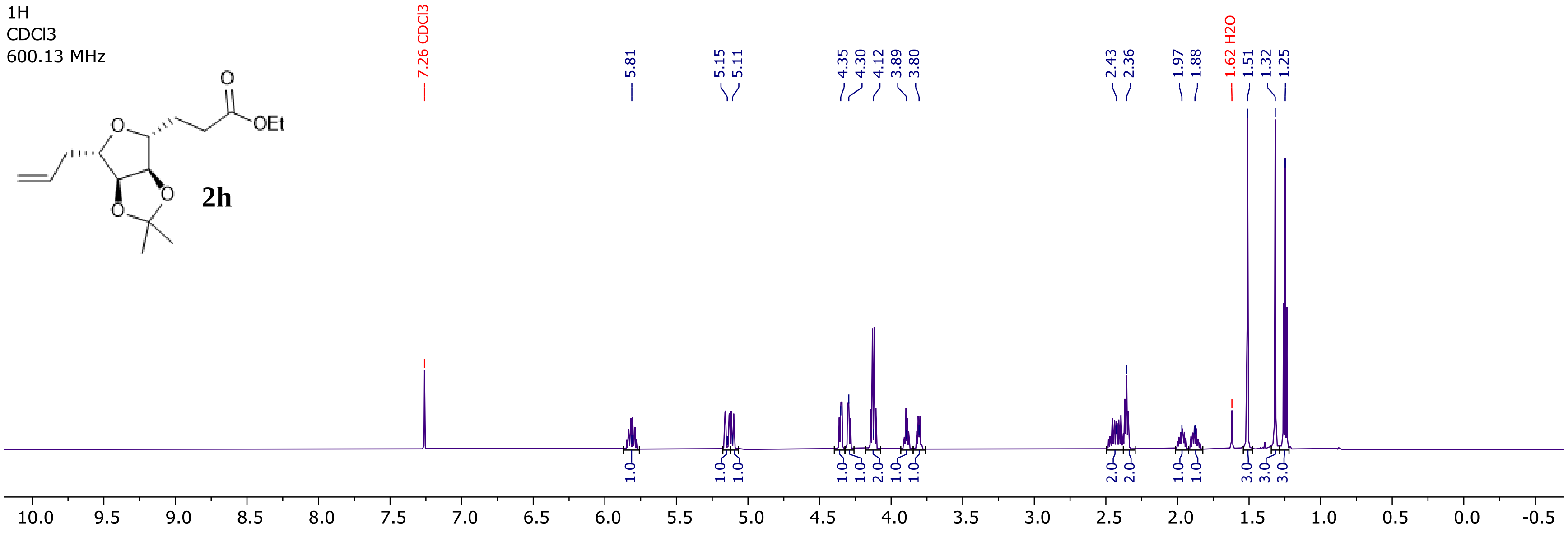
1H
CDCl3
600.13 MHz
O
OEt
O
O
O
2h
7.26 CDCl3
5.81
5.15
5.11
4.35
4.30
4.12
3.89
3.80
2.43
2.36
1.97
1.88
1.62 H2O
1.51
1.32
1.25
1.0
1.0
1.0
1.0
1.0
2.0
1.0
1.0
2.0
2.0
1.0
1.0
3.0
3.0
3.0
10.0
9.5
9.0
8.5
8.0
7.5
7.0
6.5
6.0
5.5
5.0
4.5
4.0
3.5
3.0
2.5
2.0
1.5
1.0
0.5
0.0
-0.5

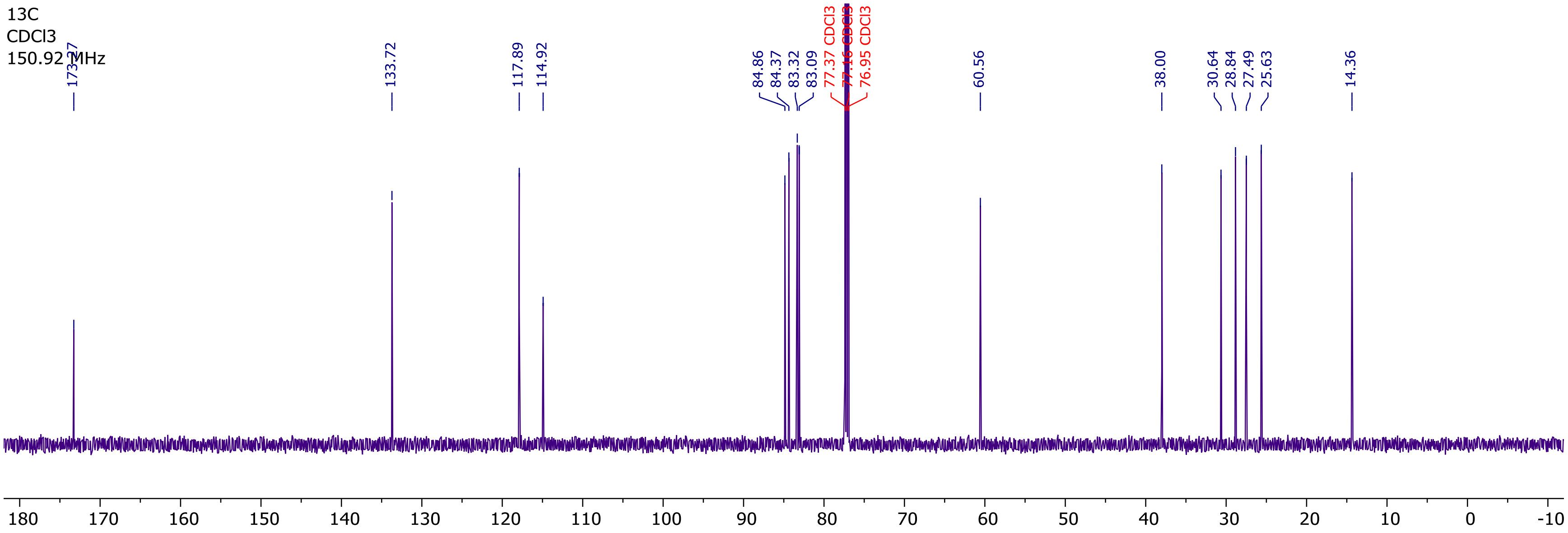
13C
CDCl3
150.92 MHz
173.37
133.72
117.89
114.92
84.86
84.37
83.32
83.09
77.37 CDCl3
77.16 CDCl3
76.95 CDCl3
60.56
38.00
30.64
28.84
27.49
25.63
14.36
180
170
160
150
140
130
120
110
100
90
80
70
60
50
40
30
20
10
0
-10

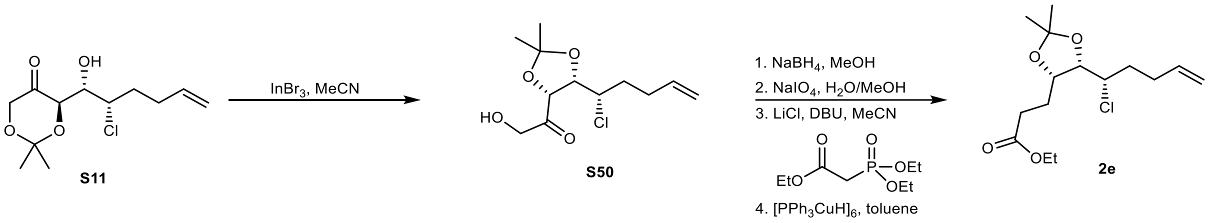


**Supplementary Fig. 33 | Synthesis of Chlorinated Ester 2e.**
Abbreviations: DBU = 1,8-Diazabicyclo [5.4.0]undec-7-ene.

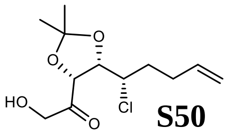
**S50**

To a r.t., stirred solution of **S11** (7.92 g, 30.1 mmol, 1.0 eq.) in strictly anhydrous MeCN (200 mL), was added $InBr_3$ (1.07 g, 3.01 mmol, 0.10 eq.) in one portion. The reaction mixture was stirred for 30 min at r.t., after which time starting material was consumed as monitored by TLC analysis. The reaction mixture was quenched by addition of a saturated aqueous solution of $NaHCO_3$ (100 mL), and the mixture was concentrated to a small volume (*ca.* 50 mL) in vacuo. The concentrated reaction mixture was poured into a separatory funnel and extracted with $CH_2Cl_2$ (3x 100 mL). The combined organic layers were dried ($Na_2SO_4$), filtered, and solvent was removed in vacuo. The crude product was purified via flash column chromatography (9:1 Hexanes/Acetone) to give **S50** (5.44 g, 69%) as a pale-yellow oil.

**Analytical Data for S50:**

$R_f$ = 0.28 (9:1 Hexanes/Acetone)

$[\alpha]_D^{20}$ = +43.2° (c = 2.75, $CH_2Cl_2$)

$^1$H NMR (500 MHz, $CD_2Cl_2$) δ 5.79 (dddd, *J* = 17.2, 10.2, 7.1, 6.2 Hz, 1H), 5.07 (dq, *J* = 17.2, 1.7 Hz, 1H), 5.02 (ddt, *J* = 10.2, 2.1, 1.2 Hz, 1H), 4.74 (dd, *J* = 20.3, 5.3 Hz, 1H), 4.66 (d, *J* = 8.5 Hz, 1H), 4.59 (dd, *J* = 8.5, 1.8 Hz, 1H), 4.40 (dd, *J* = 20.3, 4.7 Hz, 1H), 4.06 (ddd, *J* = 9.9, 4.3, 1.8 Hz, 1H), 2.84 (t, *J* = 5.1 Hz, 1H), 2.34 – 2.25 (m, 1H), 2.20 – 2.10 (m, 1H), 1.94 (dddd, *J* = 14.0, 9.9, 8.8, 5.2 Hz, 1H), 1.83 (dddd, *J* = 14.2, 8.9, 7.1, 4.3 Hz, 1H), 1.58 (s, 3H), 1.36 (s, 3H).

$^{13}$C NMR (126 MHz, $CD_2Cl_2$) δ 210.60, 137.40, 115.92, 110.97, 80.93, 80.55, 68.10, 60.28, 34.91, 31.00, 25.94, 24.49.

HRMS (ESI): Anal. Calcd. for $C_{12}H_{20}O_4Cl^+$ $[M+H]^+$ 263.1045, found 263.1066

IR (neat): $\nu_{max}$ $(cm^{-1})$ = 3468 (br, OH), 3079 (w, C=*CH*), 2965 (m, CH), 2939 (m, CH), 2911 (m, CH), 2858 (m, CH), 1722 (s, C=O), 1642 (w, C=C), 1379 (m), 1256 (m), 1213 (s).

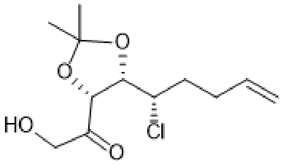
HO
O
O
O
Cl

**S50**

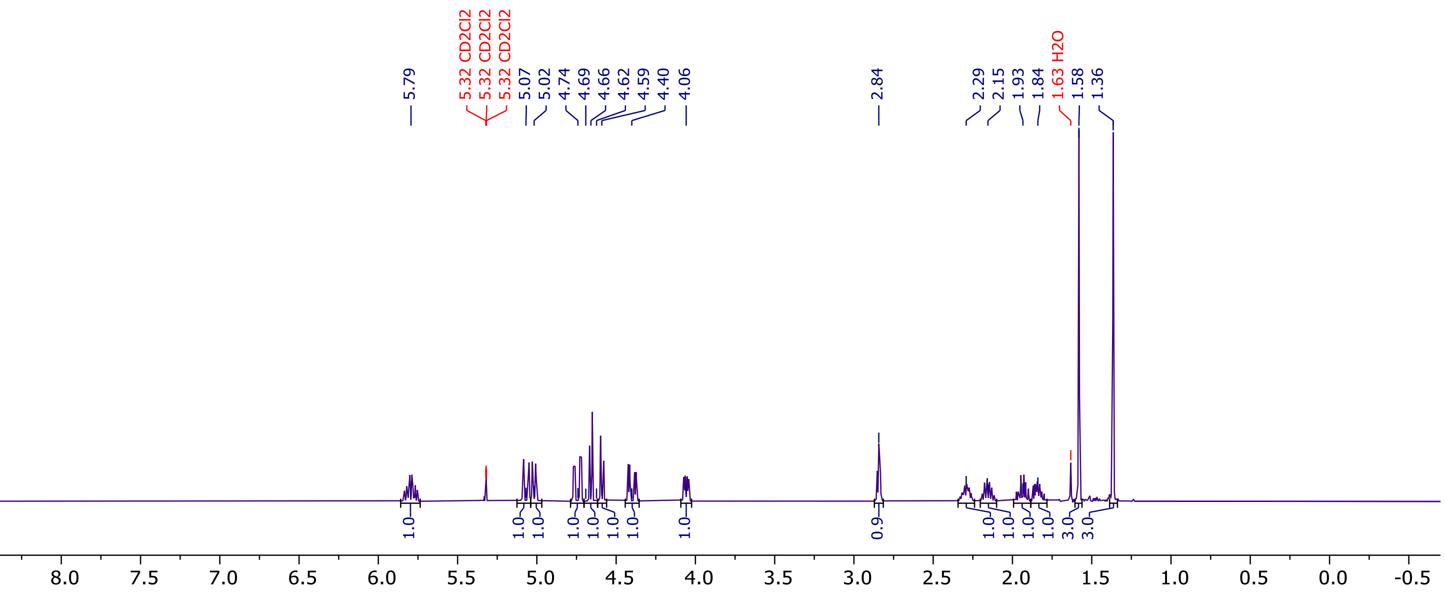
1H
CD2Cl2
500.14 MHz
5.79
5.32 CD2Cl2
5.32 CD2Cl2
5.32 CD2Cl2
5.07
5.02
4.74
4.69
4.66
4.62
4.59
4.40
4.06
2.84
2.29
2.15
1.93
1.84
1.63 H2O
1.58
1.36
1.0
1.0
1.0
1.0
1.0
1.0
1.0
1.0
0.9
1.0
1.0
1.0
1.0
3.0
3.0
10.0
9.5
9.0
8.5
8.0
7.5
7.0
6.5
6.0
5.5
5.0
4.5
4.0
3.5
3.0
2.5
2.0
1.5
1.0
0.5
0.0
-0.5

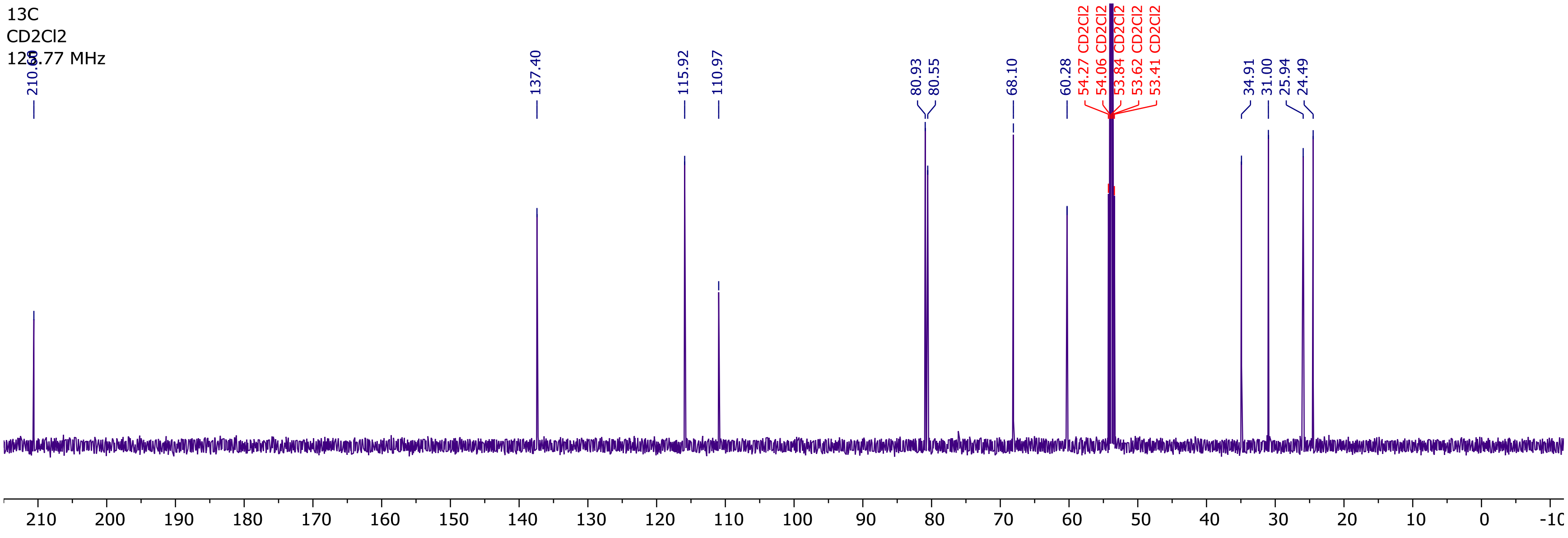
13C
CD2Cl2
125.77 MHz
210.60
137.40
115.92
110.97
80.93
80.55
68.10
60.28
54.27 CD2Cl2
54.06 CD2Cl2
53.84 CD2Cl2
53.62 CD2Cl2
53.41 CD2Cl2
34.91
31.00
25.94
24.49
210
200
190
180
170
160
150
140
130
120
110
100
90
80
70
60
50
40
30
20
10
0

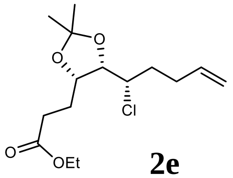

**2e**

Reaction performed open to ambient atmosphere. To a rt, stirred solution of **S50** (1.0 g, 3.8 mmol, 1.0 eq.) in MeOH (25 mL), was added $NaBH_4$ (288 mg, 7.61 mmol, 2.0 eq.) in one portion [**caution!** $H_2$(g) evolved]. The reaction mixture was allowed to stir for 25 min. After this time, starting material was consumed as monitored by TLC analysis. The reaction was quenched by addition of saturated aqueous $NH_4Cl$ (25 mL) and stirred for 30 min. until evolution of all gas had stopped. The reaction mixture was concentrated to a small volume in vacuo (*ca.* 20 mL), and the concentrate was transferred to a separatory funnel and extracted with $CH_2Cl_2$ (3x 35 mL). The combined organic extracts were dried ($Na_2SO_4$), filtered, and solvent was removed in vacuo. The crude product was passaged through a short plug of silica gel, eluting with 3:2 Hexanes/EtOAc. The solvent was removed in vacuo to give crude diol (910 mg) as a colorless oil, which was used immediately in the next step without further purification.

Reaction performed open to ambient atmosphere. To a cold (0 °C), vigorously (vigorous stirring essential due to copious precipitate) stirred solution of the crude diol from the previous step (910 mg, 3.43 mmol, 1.0 eq.) in MeOH (24 mL) and $H_2O$ (10 mL), was added $NaIO_4$ (1.10 g, 5.14 mmol, 1.5 eq.). After 5 min. the cold bath was removed, and the reaction was allowed to stir for a further 26 h 10 min. After this time, starting material was consumed as monitored by TLC analysis. The reaction mixture was poured into a separatory funnel and extracted with $Et_2O$ (3x 100 mL). The combined organic extracts were dried ($MgSO_4$), filtered, and solvent as removed in vacuo. The crude product was passaged through a short plug of silica gel eluting with $Et_2O$. The solvent was removed in vacuo to give the crude aldehyde (900 mg) as a slightly cloudy oil which was used immediately in the next step without further purification.

To a cold (-20 °C), stirred suspension of LiCl (291 mg, 6.86 mmol, 2.0 eq, flame dried in vacuo), triethylphosphonoacetate (1.16 g, 5.2 mmol, 1.5 eq.), and DBU (0.77 mL, 784 mg, 5.2 mmol, 1.5 eq.) in MeCN (20 mL) was added to the crude aldehyde from the previous step (900 mg, *ca.* 3.43 mmol, 1.0 eq.) in MeCN (3 mL + 2 mL rinse). The reaction was allowed to stir while slowly warming to rt over 45 min. After this time, starting material was consumed as monitored by TLC analysis, and the reaction was quenched by addition of saturated aqueous $NH_4Cl$ (40 mL). The biphasic mixture was poured into a separatory funnel, the organic layer was separated, and the aqueous layer was extracted with $CH_2Cl_2$ (3x 60 mL). The combined organic layers were dried ($MgSO_4$), filtered, and solvent was removed in vacuo. The crude product was passaged through a short plug of silica gel, eluting with 7:3 Hexanes/$Et_2O$. The solvent was removed in vacuo to give the crude ester (880 mg) as a colorless oil, which was used immediately in the next step without further purification.

To a rt, stirred suspension of $Cu(OAc)_2$•$H_2O$ (125 mg, 0.63 mmol) and $PPh_3$ (325 mg, 1.24 mmol) in deoxygenated toluene (23.5 mL) was added 1,1,3,3-tetramethyldisiloxane (1.65 mL, 1.25 g, 9.31 mmol) via syringe. The teal-colored suspension was allowed to stir for 28 h. After this time the suspension turned into a homogenous brick-red solution, indicating the presence of $[(PPh_3)CuH]_6$. To the crude ester from the previous step (880 mg, 2.91 mmol, 1.0 eq.) was added a solution of the previously prepared reagent $[(PPh_3)CuH]_6$

(31 mL, *ca.* 0.4 M in organosilane, 12.4 mmol, 4.3 eq.). After stirring at rt for 7 h 25 min., the reaction mixture was poured onto a silica gel column and the products eluted with $Et_2O$. The solvent was removed in vacuo, and $^1H$ NMR analysis of the crude indicated complete conversion. The crude product was purified via flash column chromatography (7:3 Hexanes/$Et_2O$). Appropriate fractions were pooled, and solvent was removed in vacuo to yield **2e** (288 mg, 25%, 4 steps) as a colorless oil.

**Analytical Data for 2e:**

$R_f$ = 0.75 (3:2 Hexanes/$Et_2O$)

$[\alpha]_D^{20}$ = -27.9° (c = 1.36, $CDCl_3$)

$^1H$ NMR (500 MHz, $CDCl_3$) δ 5.77 (dddd, *J* = 17.4, 10.2, 7.4, 6.1 Hz, 1H), 5.10 (dq, *J* = 17.1, 1.6 Hz, 1H), 5.03 (ddt, *J* = 10.2, 2.1, 1.2 Hz, 1H), 4.19 – 4.08 (m, 4H), 3.88 (ddd, *J* = 9.7, 6.1, 3.4 Hz, 1H), 2.55 (ddd, *J* = 16.7, 7.8, 5.4 Hz, 1H), 2.44 (dt, *J* = 16.6, 7.8 Hz, 1H), 2.37 (dddt, *J* = 14.5, 8.0, 5.2, 1.5 Hz, 1H), 2.24 (ddd, *J* = 14.7, 8.5, 7.2 Hz, 1H), 2.01 – 1.89 (m, 1H), 1.89 – 1.74 (m, 3H), 1.51 (s, 3H), 1.36 (s, 3H), 1.25 (t, *J* = 7.1 Hz, 3H).

$^{13}C$ NMR (126 MHz, $CDCl_3$) δ 173.35, 136.79, 116.41, 108.85, 80.60, 76.48, 60.61, 59.58, 34.41, 30.70, 30.34, 27.59, 25.85, 24.49, 14.36.

HRMS (ESI): Anal. Calcd. for $C_{15}H_{26}O_4Cl^+$ $[M+H]^+$ 305.1514, found 305.1512

IR (neat): $\nu_{max}$ $(cm^{-1})$ = 3079 (w, C=*CH*), 2984 (m, CH), 2936 (m, CH), 1732 (s, C=O), 1449 (m), 1375 (m), 1255 (s), 1217 (s).

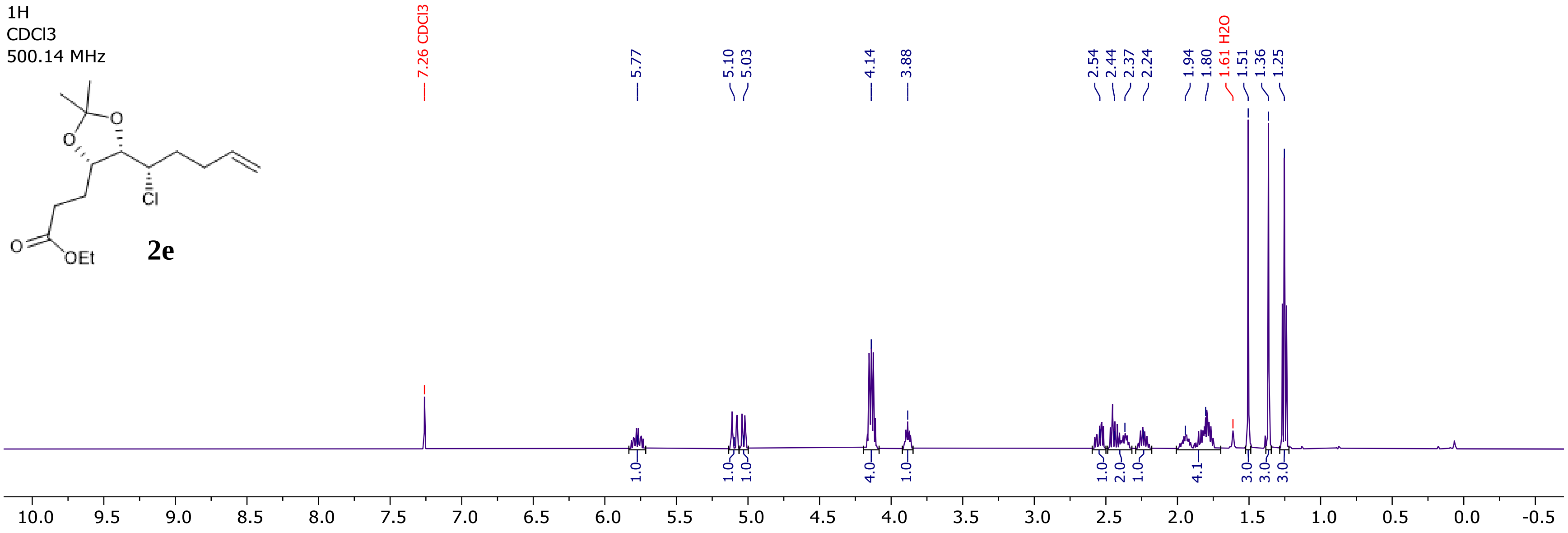
1H
CDCl3
500.14 MHz
2e
7.26 CDCl3
5.77
5.10
5.03
4.14
3.88
2.54
2.44
2.37
2.24
1.94
1.80
1.61 H2O
1.51
1.36
1.25

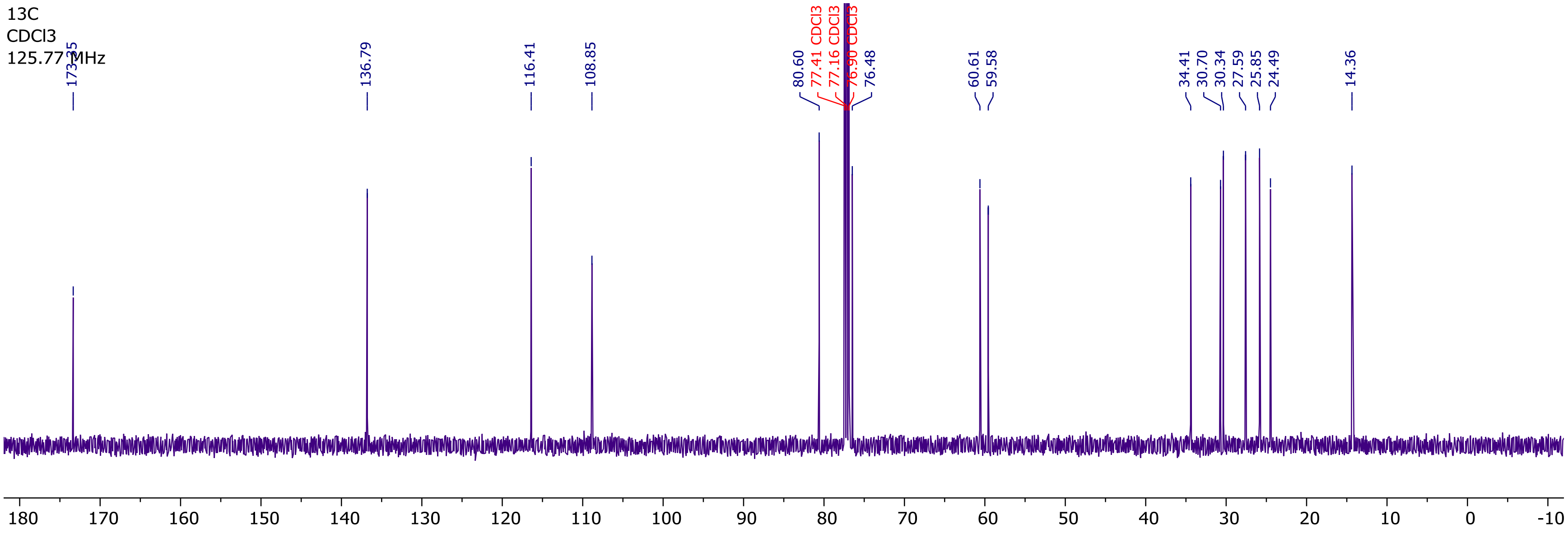
13C
CDCl3
125.77 MHz
173.35
136.79
116.41
108.85
80.60
77.41 CDCl3
77.16 CDCl3
76.90 CDCl3
76.48
60.61
59.58
34.41
30.70
30.34
27.59
25.85
24.49
14.36

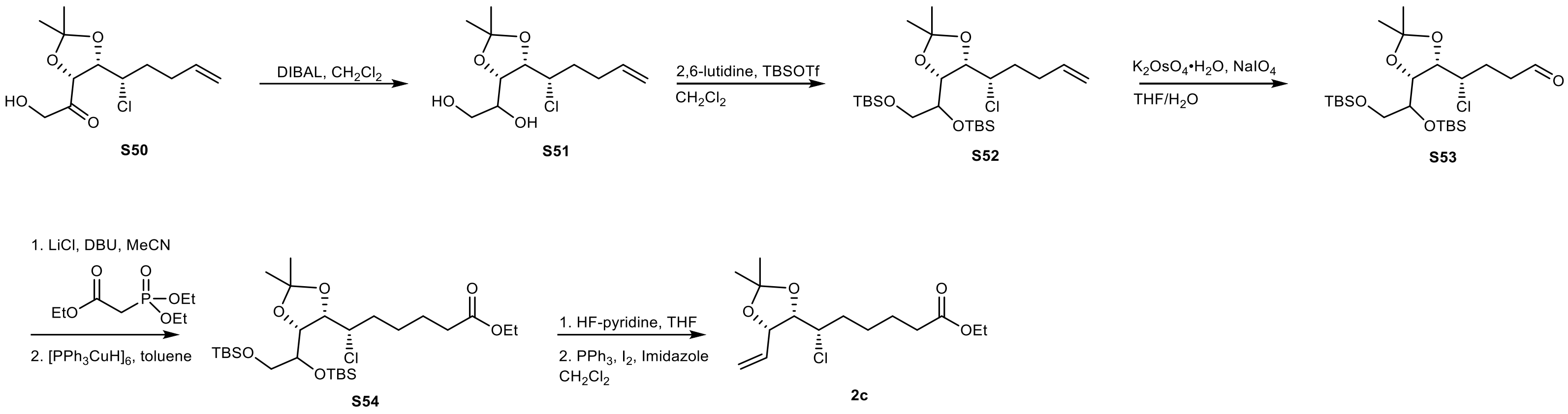


**Supplementary Fig. 34 | Synthesis of Chlorinated Ester 2c.**

Abbreviations: DIBAL = diisobutylaluminum hydride, TBSOTf = tert-butyldimethylsilyl trifluoromethanesulfonate, THF = tetrahydrofuran, DBU = 1,8-Diazabicyclo [5.4.0]undec-7-ene.

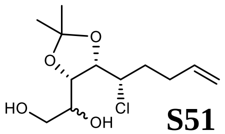

**S51**

To a cold (-78 °C), stirred solution of **S50** (5.14 g, 19.6 mmol, 1.0 eq.) in $CH_2Cl_2$ (200 mL) was added a solution of DIBAL (1 M in Hexanes, 60 mL, 60 mmol, 3.1 eq.) slowly down the sides of the reaction vessel over 10 min. The reaction mixture was maintained at -78 °C for 3 h, after which time starting material was consumed as monitored by TLC analysis. The reaction mixture was then quenched with a saturated aqueous solution of Rochelle's salt (200 mL), warmed to r.t., and stirred vigorously for 24 h. After this time, the biphasic mixture was poured into a separatory funnel, and the organic layer was separated. The aqueous layer was extracted with $CH_2Cl_2$ (4x 125 mL). The combined organic layers were dried ($Na_2SO_4$), filtered, and solvent was removed in vacuo. The crude product was purified via flash column chromatography to give **S51** (3.24 g, 63%) as a pale-yellow oil and a 10:1 mixture of diastereomers. A small quantity of this material was re-purified to remove the minor diastereomer for analytical purposes.

**Analytical Data for S51:**

$R_f$ = 0.23 (3:1 Hexanes/Acetone)

$[\alpha]_D^{20}$ = -27.5° (c = 2.35, $CD_2Cl_2$)

$^1$H NMR (500 MHz, $CD_2Cl_2$) δ 5.81 (dddd, $J$ = 17.3, 10.2, 7.2, 6.3 Hz, 1H), 5.13 – 5.06 (m, 1H), 5.03 (ddt, $J$ = 10.2, 2.1, 1.2 Hz, 1H), 4.30 – 4.17 (m, 3H), 3.85 (p, $J$ = 4.9 Hz, 1H), 3.68 – 3.58 (m, 2H), 2.57 – 2.46 (m, 1H), 2.39 (dddd, $J$ = 10.1, 8.2, 6.8, 5.4 Hz, 1H), 2.33 – 2.17 (m, 2H), 1.89 – 1.74 (m, 2H), 1.53 (s, 3H), 1.38 (s, 3H).

$^{13}$C NMR (126 MHz, $CD_2Cl_2$) δ 137.27, 116.17, 109.06, 80.14, 80.13, 77.81, 69.55, 69.52, 64.97, 64.95, 60.16, 35.25, 30.63, 26.56, 25.05.

HRMS (ESI): Anal. Calcd. for $C_{12}H_{22}O_4Cl^+$ $[M+H]^+$ 265.1201, found 265.1224

IR (neat): $\nu_{max}$ $(cm^{-1})$ = 3437 (br, OH), 3077 (w, C=*CH*), 2985 (m, CH), 2937 (m, CH), 1641 (m, C=C), 1448 (m), 1376 (m), 1255 (m), 1215 (m).

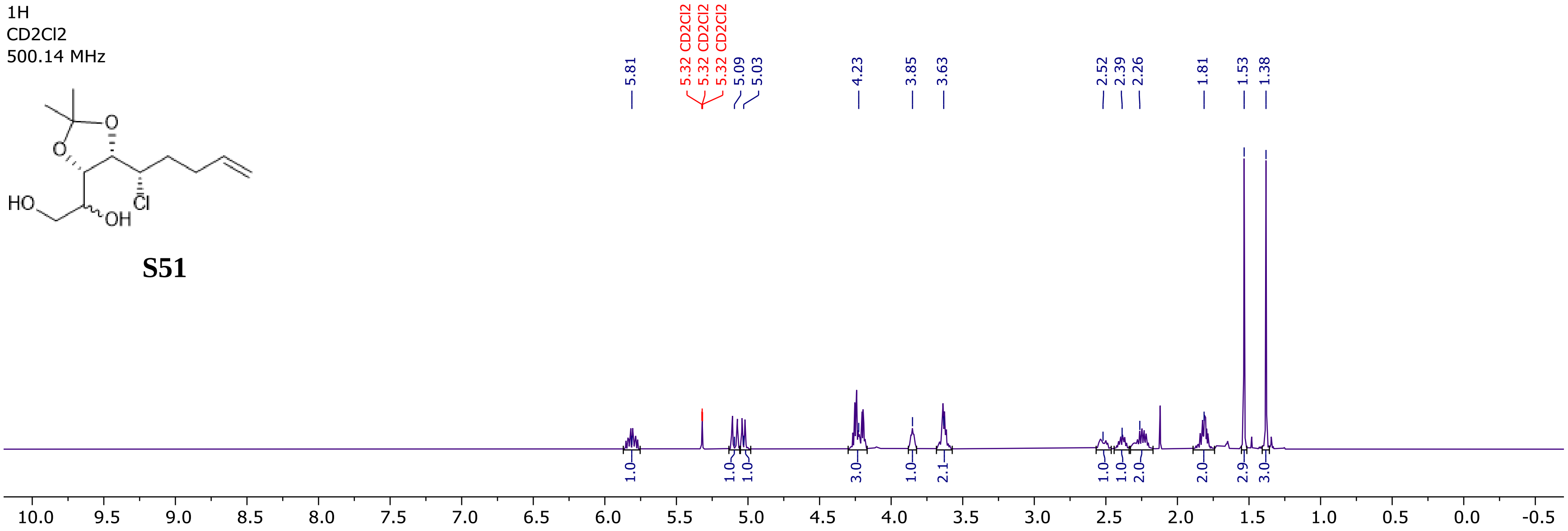
1H
CD2Cl2
500.14 MHz
O
O
HO
OH
Cl
S51
5.81
5.32 CD2Cl2
5.32 CD2Cl2
5.32 CD2Cl2
5.09
5.03
4.23
3.85
3.63
2.52
2.39
2.26
1.81
1.53
1.38
1.0
1.0
1.0
3.0
1.0
2.1
1.0
1.0
2.0
2.0
2.9
3.0
10.0 9.5 9.0 8.5 8.0 7.5 7.0 6.5 6.0 5.5 5.0 4.5 4.0 3.5 3.0 2.5 2.0 1.5 1.0 0.5 0.0 -0.5

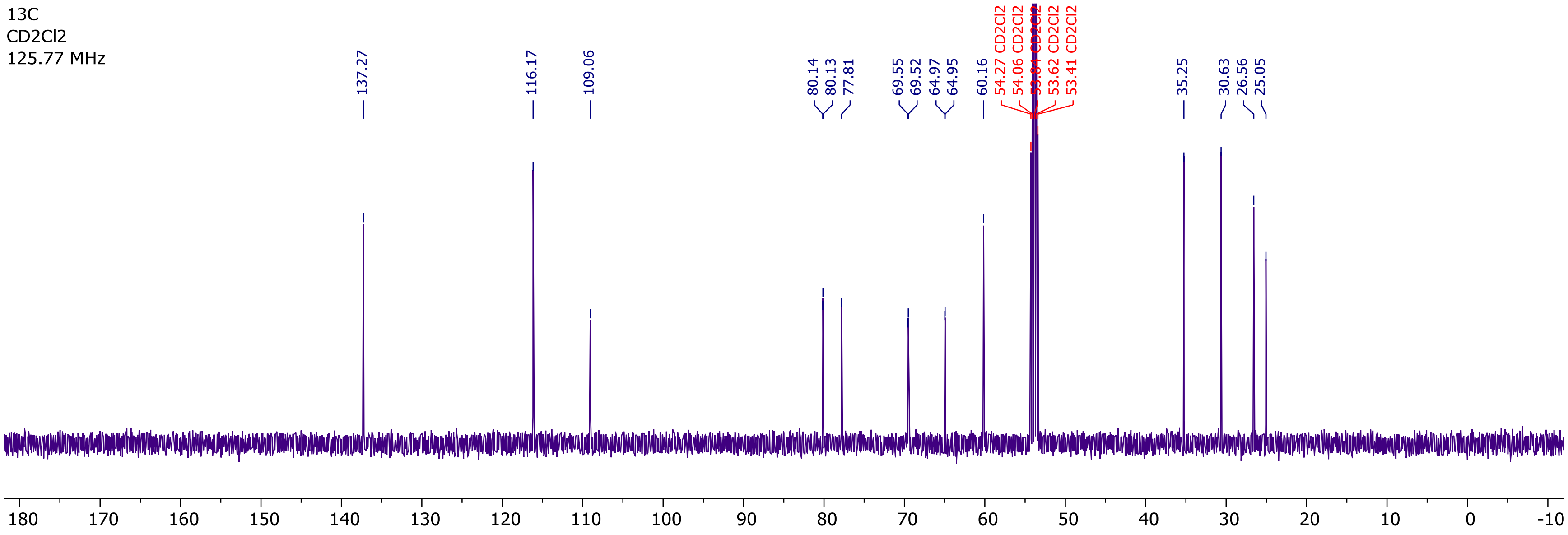
13C
CD2Cl2
125.77 MHz
137.27
116.17
109.06
80.14
80.13
77.81
69.55
69.52
64.97
64.95
60.16
54.27 CD2Cl2
54.06 CD2Cl2
53.62 CD2Cl2
53.41 CD2Cl2
35.25
30.63
26.56
25.05
180 170 160 150 140 130 120 110 100 90 80 70 60 50 40 30 20 10 0 -10

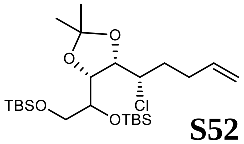


**S52**

To a cold (0 °C), stirred solution of **S51** (3.20 g, 12.2 mmol, 1 eq.) and 2,6-lutidine (7.0 mL, 6.5 g, 60 mmol, 5.0 eq.) in $CH_2Cl_2$ (120 mL) was added TBSOTf via syringe (7.0 mL, 8.1 g, 30 mmol, 2.5 eq). The reaction mixture was allowed to stir for 10 minutes, at which point the cold bath was removed and the mixture allowed to warm to rt. After stirring for an additional 20 min, starting material was consumed as monitored by TLC analysis. The reaction mixture was quenched with a saturated aqueous solution of $NaHCO_3$ (100 mL) and the mixture transferred to a separatory funnel. The organic layer was separated, and the aqueous layer was extracted with $CH_2Cl_2$ (2x 100 mL). The combined organic layers were dried ($Na_2SO_4$), filtered, and solvent was removed in vacuo. The crude product was passaged through a short plug of silica gel eluting with Hexanes/$Et_2O$ (19:1). The solvent was removed in vacuo to yield the product **S52** (5.65 g, 94%) as an inconsequential mixture (9:1) of diastereomers suitably pure for the next reaction. An analytical quantity of material was purified via flash column chromatography (49:1 Hexanes/$Et_2O$) and the major isomer was isolated for characterization purposes.

**Analytical Data for Major Isomer of S52:**

$R_f$ = 0.65 (19:1 Hexanes/$Et_2O$)

$[\alpha]_D^{20}$ = +1.8° (c = 0.57, $CDCl_3$)

$^1$H NMR (500 MHz, $CDCl_3$) δ 5.83 – 5.73 (m, 1H), 5.07 (dq, *J* = 17.1, 1.6 Hz, 1H), 4.99 (ddt, *J* = 10.2, 2.1, 1.2 Hz, 1H), 4.23 – 4.09 (m, 4H), 3.67 (dd, *J* = 10.1, 4.9 Hz, 1H), 3.53 (dd, *J* = 10.1, 7.2 Hz, 1H), 2.38 (ddddd, *J* = 12.4, 9.3, 6.2, 4.5, 1.4 Hz, 1H), 2.20 – 2.09 (m, 1H), 1.92 (dtd, *J* = 14.4, 9.9, 4.8 Hz, 1H), 1.78 – 1.68 (m, 1H), 1.56 (s, 4H), 1.36 (s, 3H), 0.89 (s, 9H), 0.89 (s, 9H), 0.11 (s, 3H), 0.11 (s, 3H), 0.06 (s, 6H).

$^{13}$C NMR (126 MHz, $CDCl_3$) δ 137.40, 115.81, 108.37, 79.82, 79.63, 70.97, 66.43, 60.60, 35.43, 30.76, 26.16, 26.13, 25.43, 18.58, 18.50, -4.11, -4.14, -5.24, -5.31.

HRMS (ESI): Anal. Calcd. for $C_{24}H_{50}O_4Si_2Cl^+$ $[M+H]^+$ 493.2931, found 493.2918

IR (neat): $\nu_{max}$ $(cm^{-1})$ = 2955 (m, CH), 2930 (m, CH), 2858 (m, CH), 1683 (w, C=C), 1469 (m), 1385 (m), 1362 (m), 1254 (m).

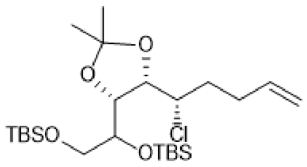
TBSO
OTBS
Cl
O
O

**S52**

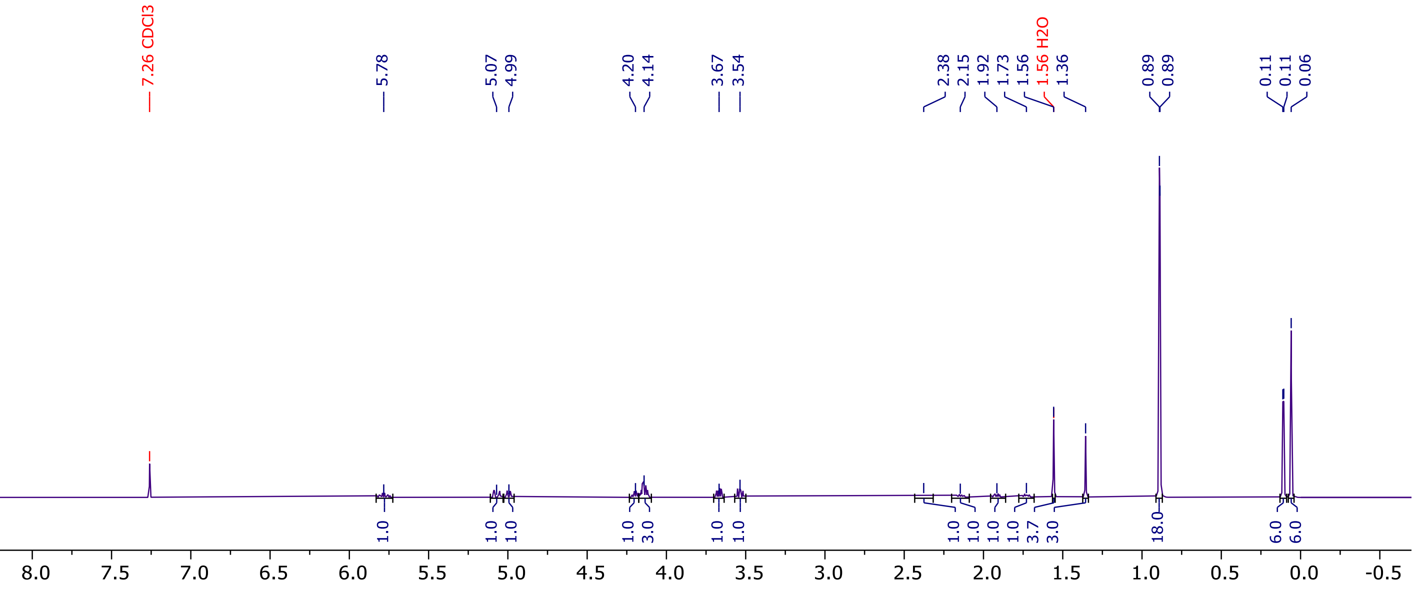
1H
CDCl3
500.14 MHz
7.26 CDCl3
5.78
5.07
4.99
4.20
4.14
3.67
3.54
2.38
2.15
1.92
1.73
1.56
1.56 H2O
1.36
0.89
0.89
0.11
0.11
0.06
1.0
1.0
1.0
1.0
3.0
1.0
1.0
1.0
1.0
1.0
1.0
3.7
3.0
18.0
6.0
6.0
10.0 9.5 9.0 8.5 8.0 7.5 7.0 6.5 6.0 5.5 5.0 4.5 4.0 3.5 3.0 2.5 2.0 1.5 1.0 0.5 0.0 -0.5

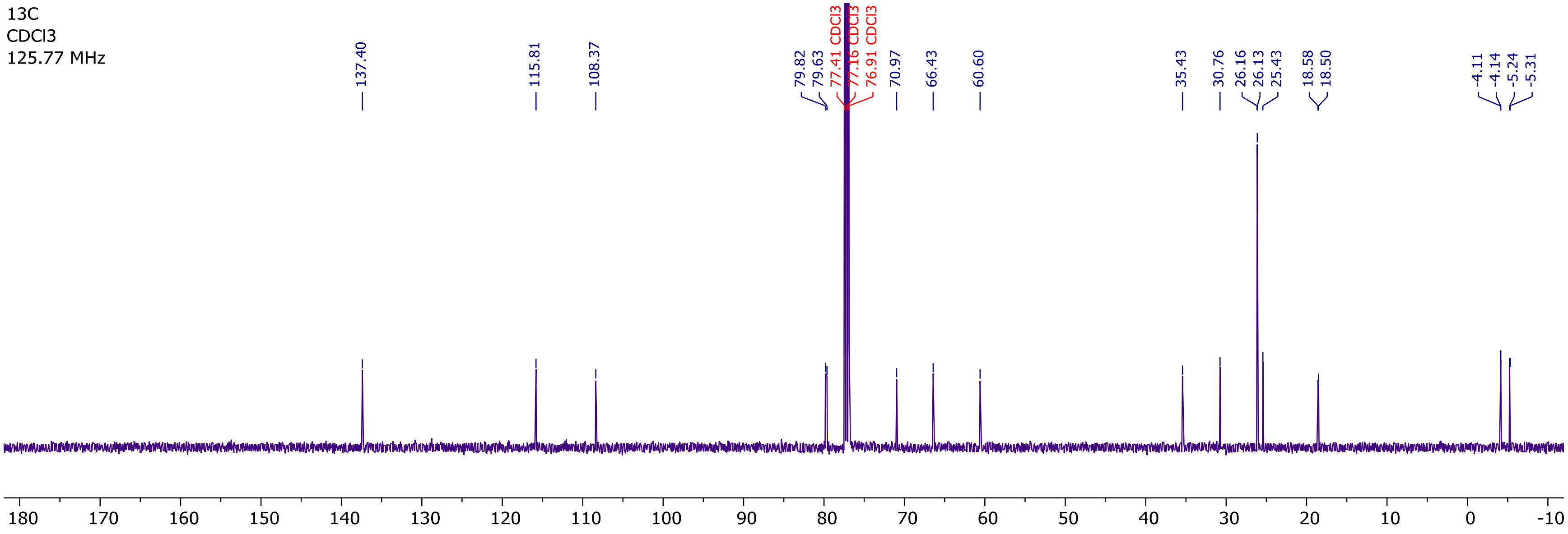
13C
CDCl3
125.77 MHz
137.40
115.81
108.37
79.82
79.63
77.41 CDCl3
77.16 CDCl3
76.91 CDCl3
70.97
66.43
60.60
35.43
30.76
26.16
26.13
25.43
18.58
18.50
-4.11
-4.14
-5.24
-5.31
180 170 160 150 140 130 120 110 100 90 80 70 60 50 40 30 20 10 0 -10

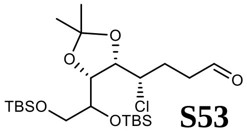


**S53**

To a rt, vigorously stirred solution of **S52** (5.55 g, 11.3 mmol, 1.0 eq.) in a mixture of THF-$H_2O$ (1:1 (v/v), 225 mL) was added $K_2OsO_4$•$H_2O$ (41.5 mg, 0.113 mmol, 0.11 eq.) and $NaIO_4$ (6.02 g, 28.1 mmol, 2.5 eq.). After stirring vigorously for 25 h 30 min, starting material was consumed as monitored by TLC analysis. The reaction mixture was poured into a separatory funnel and the aqueous layer was extracted with $Et_2O$ (3x 125 mL). The combined organic extracts were dried ($MgSO_4$), filtered, and solvent was removed in vacuo to give **S53** as a colorless oil (4.35 g, 78%) and inconsequential 10:1 mixture of diastereomers.

**Analytical Data for Major Isomer of S53:**

$R_f$ = 0.25 (9:1 Hexanes/$Et_2O$)

$[\alpha]_D^{20}$ = +1.1° (c = 2.22, $CDCl_3$)

$^1$H NMR (600 MHz, CDCl3) δ 9.79 (t, J = 1.2 Hz, 1H), 4.25 (td, J = 7.5, 4.9 Hz, 1H), 4.21 (dt, J = 10.3, 3.0 Hz, 1H), 4.16 (dd, J = 6.5, 2.9 Hz, 1H), 4.12 (dd, J = 7.5, 6.4 Hz, 1H), 3.68 (dd, J = 10.1, 4.9 Hz, 1H), 3.52 (dd, J = 10.1, 7.6 Hz, 1H), 2.79 (dddd, J = 18.3, 8.6, 5.4, 1.1 Hz, 1H), 2.59 (dddd, J = 18.3, 8.7, 6.5, 1.3 Hz, 1H), 2.16 – 2.06 (m, 1H), 2.02 (dddd, J = 14.9, 8.6, 6.5, 3.2 Hz, 1H), 1.55 (s, 3H), 1.34 (s, 3H), 0.88 (s, 9H), 0.87 (s, 9H), 0.10 (s, 3H), 0.09 (s, 3H), 0.06 (s, 6H).

$^{13}$C NMR (151 MHz, $CDCl_3$) δ 201.27, 108.43, 79.99, 79.67, 70.67, 66.55, 60.34, 41.03, 28.61, 26.14, 26.07, 26.05, 25.36, 18.58, 18.49, -4.18, -4.22, -5.30, -5.35.

HRMS (ESI): Anal. Calcd. for $C_{23}H_{48}O_5Si_2Cl^+$ $[M+H]^+$ 495.2723, found 495.2730

IR (neat): $\nu_{max}$ $(cm^{-1})$ = 2932 (m, CH), 2858 (m, CH), 1727 (s, C=O), 1468 (m), 1371 (m), 1255 (s).

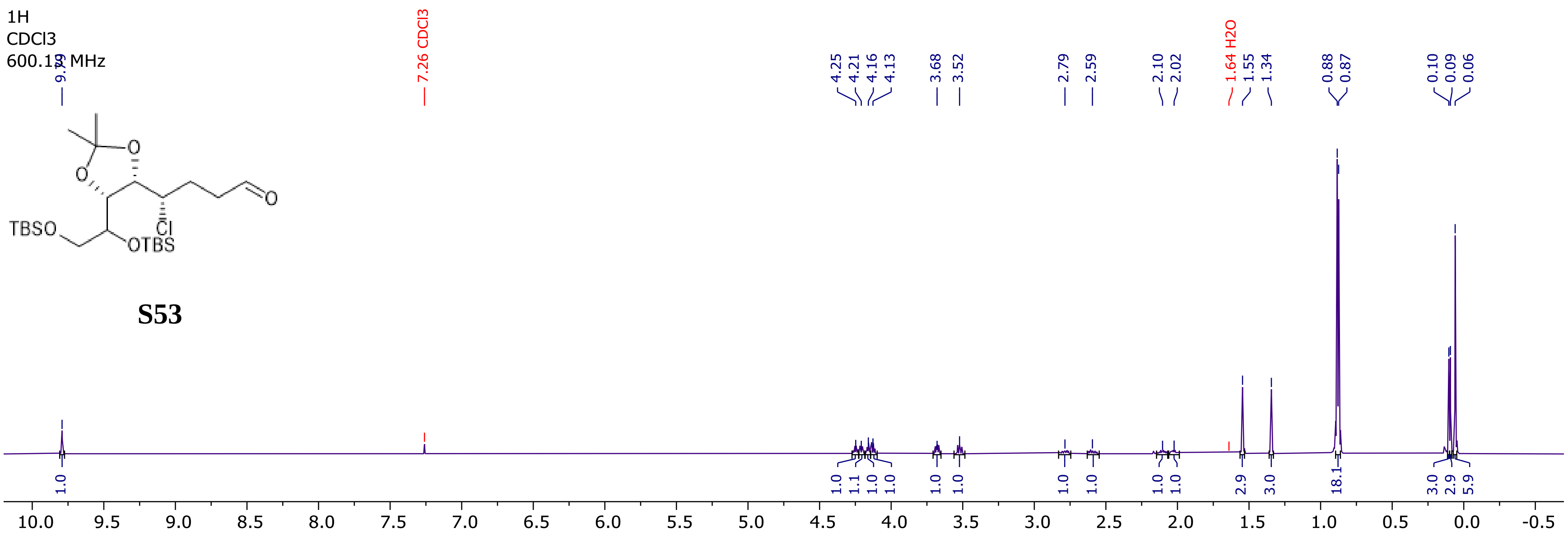
1H
CDCl3
600.13 MHz
9.79
7.26 CDCl3
4.25
4.21
4.16
4.13
3.68
3.52
2.79
2.59
2.10
2.02
1.64 H2O
1.55
1.34
0.88
0.87
0.10
0.09
0.06
TBSO
OTBS
O
O
Cl
O
1.0
1.0
1.1
1.0
1.0
1.0
1.0
1.0
1.0
1.0
1.0
2.9
3.0
18.1
3.0
2.9
5.9
10.0
9.5
9.0
8.5
8.0
7.5
7.0
6.5
6.0
5.5
5.0
4.5
4.0
3.5
3.0
2.5
2.0
1.5
1.0
0.5
0.0
-0.5

**S53**

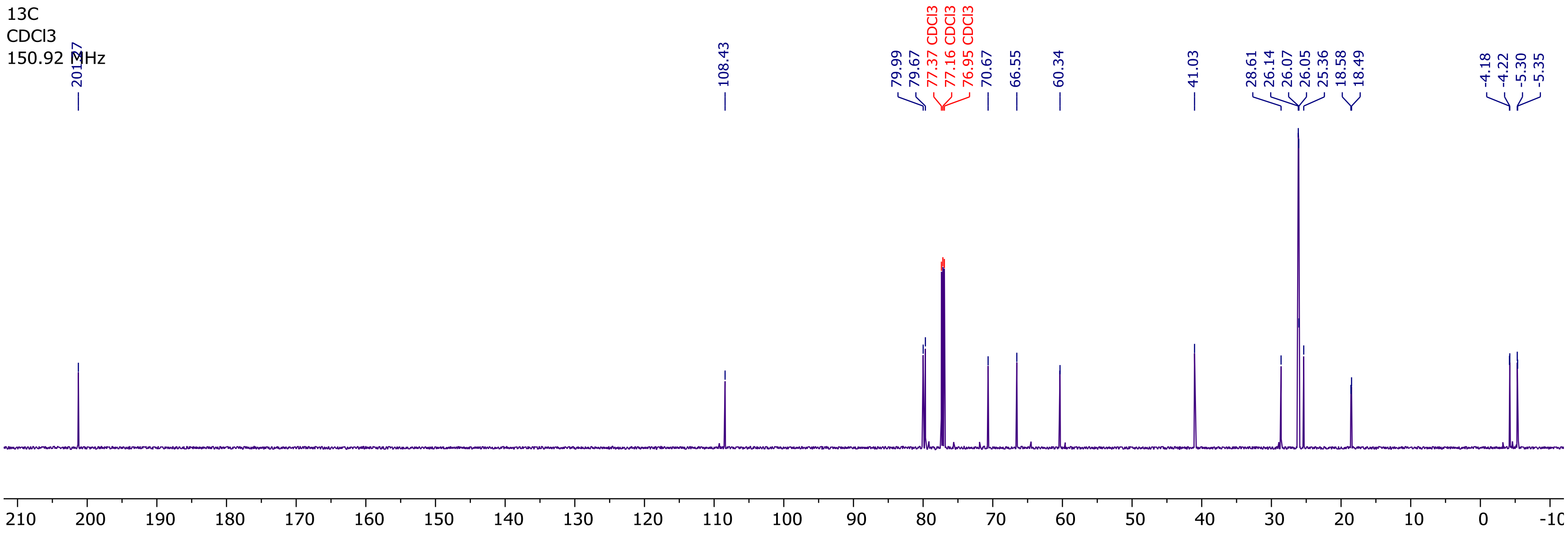
13C
CDCl3
150.92 MHz
201.37
108.43
79.99
79.67
77.37 CDCl3
77.16 CDCl3
76.95 CDCl3
70.67
66.55
60.34
41.03
28.61
26.14
26.07
26.05
25.36
18.58
18.49
-4.18
-4.22
-5.30
-5.35
210
200
190
180
170
160
150
140
130
120
110
100
90
80
70
60
50
40
30
20
10
0
-10

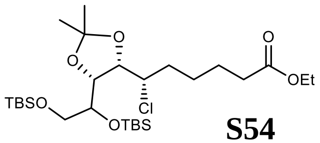


**S54**

To a cold (0 °C), stirred suspension of LiCl (745 mg, 17.7 mmol, 2.0 eq, flame dried in vacuo), triethylphosphonoacetate (2.93 g, 13.1 mmol, 1.5 eq.), and DBU (1.95 mL, 1.99 g, 13.1 mmol, 1.5 eq.) in MeCN (45 mL) was added aldehyde **S53** (4.25 g, 8.58 mmol, 1.0 eq.) in MeCN (10 mL + 5 mL rinse). The reaction was allowed to stir while slowly warming to rt over 4 h. After this time, starting material was consumed as monitored by TLC analysis, and the reaction was quenched by addition of saturated aqueous $NH_4Cl$ (50 mL). The biphasic mixture was poured into a separatory funnel, the organic layer was separated, and the aqueous layer was extracted with $CH_2Cl_2$ (4x 75 mL). The combined organic layers were dried ($Na_2SO_4$), filtered, and solvent was removed in vacuo. The crude product was purified via flash column chromatography (87.5:12.5 Hexanes/$Et_2O$). Appropriate fractions were pooled, and solvent was removed in vacuo to yield the α,β-unsaturated ester (4.50 g, 93%) as a colorless oil. A portion of this product was used immediately in the subsequent step.

At rt, the stirred suspension of ester from the previous step (1.41 g, 2.48 mmol, 1.0 eq.) and $PtO_2$ (28.4 mg, 0.125 mmol, 0.05 eq.) in $CH_2Cl_2$ (25 mL) was subjected to 3 cycles of nitrogen flushing and application of vacuum to remove oxygen. Subsequently a large balloon was filled with *ca.* 2 L of $H_2$ (g) and was fitted onto a syringe equipped with a 6-inch 18-gauge needle. The needle was inserted through the rubber septum directly into the reaction mixture and a bleed needle into the reaction flask septum. The reaction mixture was sparged with $H_2$(g) for 5 min. After this time, the bleed needle was removed, and the reaction was allowed to stir under the atmosphere of $H_2$(g) with the balloon needle pulled up into the headspace of the reaction. After stirring for a further 50 min, absence of UV activity on TLC indicated complete consumption of starting material. The $H_2$(g) balloon was removed, and the reaction mixture was sparged with $N_2$(g) for 30 minutes. After this time, the reaction mixture was filtered through a small pad of celite rinsing with $CH_2Cl_2$ (3x 100 mL). The solvent was removed in vacuo to yield **S54** (1.41 g, 93%, 2 steps) as a colorless oil and an inconsequential 10:1 mixture of diastereomers.

**Analytical Data for Major Isomer of S54:**

$R_f$ = 0.40 (19:1 Hexanes/EtOAc)

$[\alpha]_D^{20}$ = +2.5° (c = 3.61, $CDCl_3$)

$^1$H NMR (600 MHz, $CDCl_3$) δ 4.21 (td, *J* = 7.4, 4.9 Hz, 1H), 4.13 (ddt, *J* = 15.7, 9.2, 5.1 Hz, 5H), 3.67 (dd, *J* = 10.1, 4.9 Hz, 1H), 3.52 (dd, *J* = 10.0, 7.6 Hz, 1H), 2.30 (t, *J* = 7.1 Hz, 2H), 1.86 – 1.76 (m, 1H), 1.71 – 1.59 (m, 4H), 1.55 (s, 3H), 1.35 (s, 4H), 1.25 (t, *J* = 7.1 Hz, 3H), 0.88 (s, 9H), 0.88 (s, 9H), 0.11 (s, 3H), 0.10 (s, 3H), 0.05 (s, 6H).

$^{13}$C NMR (151 MHz, $CDCl_3$) δ 173.61, 108.34, 79.80, 79.78, 70.77, 66.46, 61.19, 60.40, 35.93, 34.35, 26.32, 26.14, 26.10, 25.42, 24.77, 18.57, 18.49, 14.39, -4.15, -4.18, -5.28, -5.34.

HRMS (ESI): Anal. Calcd. for $C_{27}H_{56}O_6Si_2Cl^+$ $[M+H]^+$ 567.3299, found 567.3299

IR (neat): $\nu_{max}$ $(cm^{-1})$ = 2932 (m, CH), 2858 (m, CH), 1738 (s, C=O), 1467 (m), 1372 (m), 1301 (m), 1255 (s).

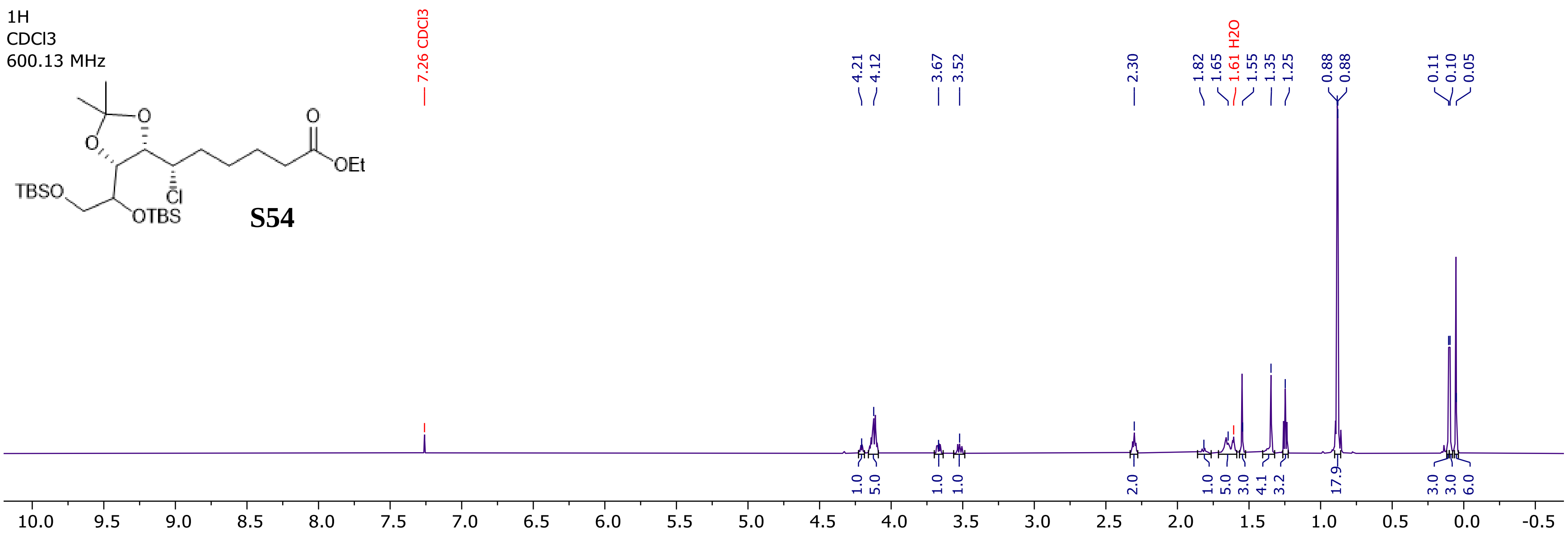
1H
CDCl3
600.13 MHz
7.26 CDCl3
4.21
4.12
3.67
3.52
2.30
1.82
1.65
1.61 H2O
1.55
1.35
1.25
0.88
0.88
0.11
0.10
0.05
TBSO
OTBS
Cl
O
OEt
S54
1.0
5.0
1.0
1.0
2.0
1.0
5.0
3.0
4.1
3.2
17.9
3.0
3.0
6.0
10.0
9.5
9.0
8.5
8.0
7.5
7.0
6.5
6.0
5.5
5.0
4.5
4.0
3.5
3.0
2.5
2.0
1.5
1.0
0.5
0.0
-0.5

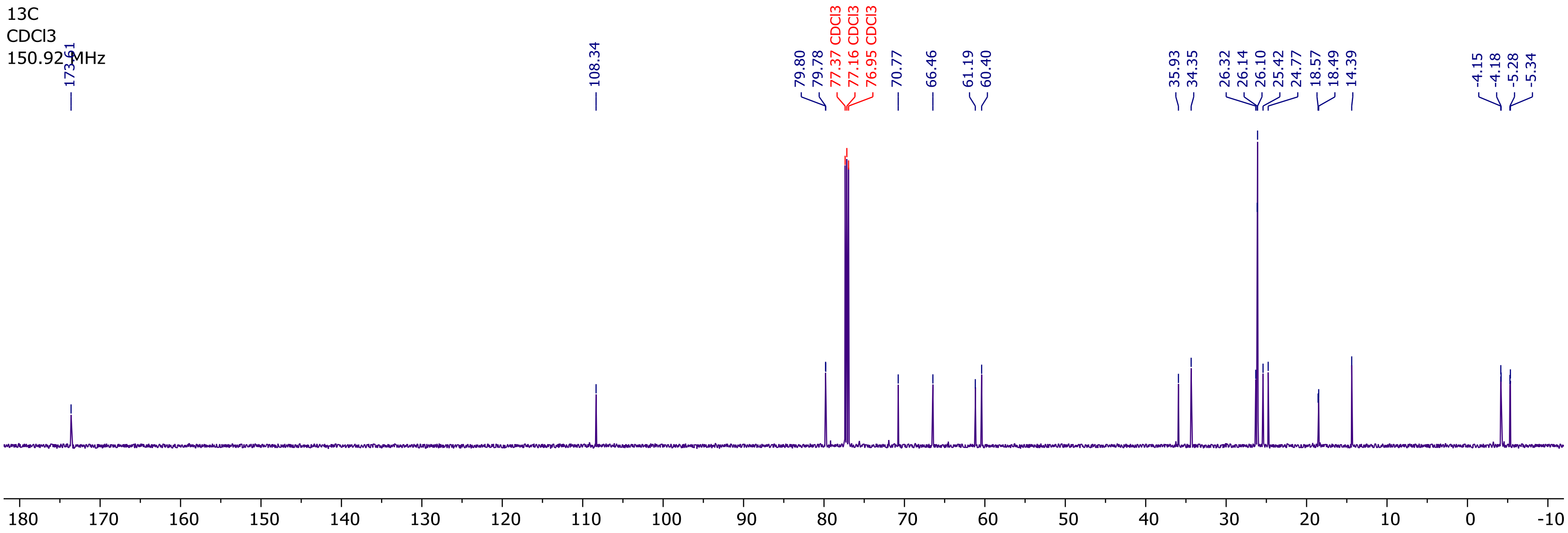
13C
CDCl3
150.92 MHz
173.61
108.34
79.80
79.78
77.37 CDCl3
77.16 CDCl3
76.95 CDCl3
70.77
66.46
61.19
60.40
35.93
34.35
26.32
26.14
26.10
25.42
24.77
18.57
18.49
14.39
-4.15
-4.18
-5.28
-5.34
180
170
160
150
140
130
120
110
100
90
80
70
60
50
40
30
20
10
0
-10

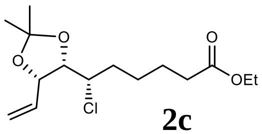

**2c**

To a rt, stirred solution of **S54** (1.41 g, 2.49 mmol, 1 eq.) in THF was added in sequence pyridine (6.4 mL, 6.3 g, 79.4 mmol) and HF-Pyridine (70:30 (w/w) HF, 3.2 mL, 2.5 g HF, 120 mmol HF, 50 eq. HF, 9.3 mmol Pyridine, 3.7 eq. Pyridine). The reaction was then cooled to 0 °C and allowed to slowly warm to rt over the course of 24 h 30 min. After this time, starting material was consumed as monitored by TLC analysis. The reaction mixture was slowly poured onto a saturated aqueous solution of $NaHCO_3$ (250 mL) [**caution!** $CO_2$ (g) evolved]. After the evolution of $CO_2$(g) had stopped, the aqueous layer was extracted with $Et_2O$ (1x 60 mL) and then $CH_2Cl_2$ (3x 150 mL). The combined organic layers were dried ($MgSO_4$), filtered, and solvent was removed in vacuo. The crude product was purified via flash column chromatography (7:3 Hexanes/Acetone) to yield the diol as a colorless oil (710 mg). This product was used in the next step immediately as follows.

To a rt, stirred solution of the diol from the previous step (710 mg, 2.10 mmol, 1.0 eq.) in $CH_2Cl_2$ (12 mL), was added imidazole (570 mg, 8.37 mmol, 4.0 eq.) and $PPh_3$ (2.2 g, 8.39 mmol, 4.0 eq.). The reaction mixture was then cooled to 0 °C and $I_2$ (1.6 g, 6.3 mmol, 3 eq.) was added in one portion. After stirring for 5 min at 0 °C, the cold bath was removed, and the reaction mixture was allowed to warm to rt. After stirring for a further 1 h, the starting material was consumed as monitored by TLC analysis. The reaction mixture was quenched by addition of a 10% aqueous solution of $Na_2S_2O_3$ (10 mL) and a saturated aqueous solution of $NaHCO_3$ (10 mL). The biphasic mixture was poured into a separatory funnel, the organic layer separated, and the aqueous layer extracted with $CH_2Cl_2$ (4x 15 mL). The combined organic layers, were dried ($Na_2SO_4$), filtered, and solvent was removed in vacuo. The crude product was triturated with ether (4x 15 mL) and filtered through a cotton plug. Celite was added to the filtrate, and the solvent was removed in vacuo to load the crude product onto celite. The crude product was purified via flash column chromatography (92.5:7.5 to 17:3 Hexanes/EtOAc) to yield **2c** (561 mg, 74%, 2 steps) as a colorless oil.

**Analytical Data for 2c:**

$R_f$ = 0.83 (7:3 Hexanes/Acetone)

$[\alpha]_D^{20}$ = -4.0° (c = 1.73, $CDCl_3$)

$^1$H NMR (500 MHz, $CDCl_3$) δ 5.99 (ddd, *J* = 17.1, 10.2, 8.6 Hz, 1H), 5.38 – 5.29 (m, 2H), 4.55 (dd, *J* = 8.6, 6.4 Hz, 1H), 4.19 (t, *J* = 6.4 Hz, 1H), 4.11 (q, *J* = 7.1 Hz, 2H), 3.79 (ddd, *J* = 8.8, 6.4, 4.3 Hz, 1H), 2.29 (t, *J* = 7.0 Hz, 2H), 1.76 – 1.56 (m, 5H), 1.55 (s, 3H), 1.48 – 1.41 (m, 1H), 1.39 (s, 3H), 1.24 (t, *J* = 7.1 Hz, 3H).

$^{13}$C NMR (126 MHz, $CDCl_3$) δ 173.56, 133.43, 120.46, 109.32, 81.11, 79.70, 60.64, 60.42, 34.39, 34.20, 27.44, 25.80, 25.59, 24.38, 14.37.

HRMS (ESI): Anal. Calcd. for $C_{15}H_{29}NO_4Cl^+$ $[M+NH_4]^+$ 322.1780, found 322.1779.

IR (neat): $\nu_{max}$ ($cm^{-1}$) = 3086 (w, C=*CH*), 2985 (m, CH), 2939 (m, CH), 2871 (m, CH), 1732 (s, C=O), 1459 (m), 1375 (m), 1254 (s), 1215 (s).

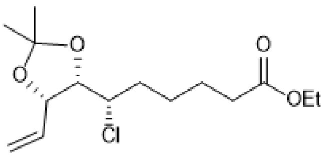
O
O
O
OEt
Cl

**2c**

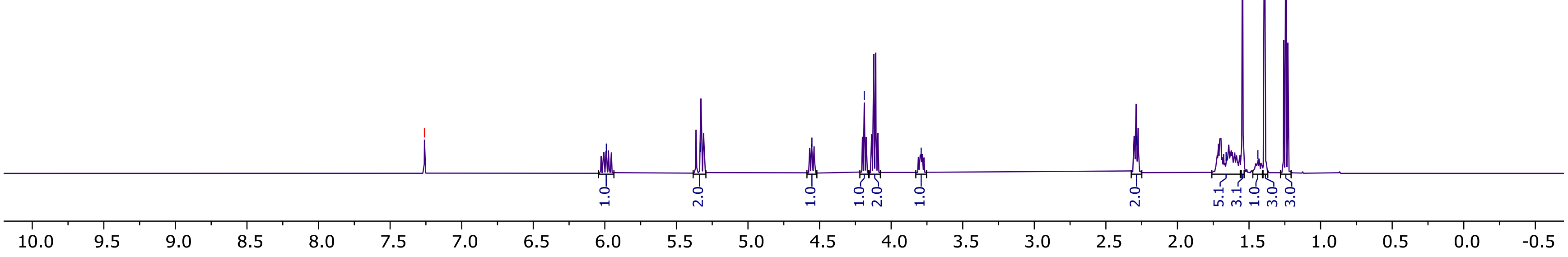
1H
CDCl3
500.14 MHz
7.26 CDCl3
5.99
5.33
4.55
4.19
4.11
3.79
2.28
1.66
1.55
1.44
1.39
1.24
1.0
2.0
1.0
1.0
2.0
1.0
2.0
5.1
3.1
1.0
3.0
3.0
10.0 9.5 9.0 8.5 8.0 7.5 7.0 6.5 6.0 5.5 5.0 4.5 4.0 3.5 3.0 2.5 2.0 1.5 1.0 0.5 0.0 -0.5

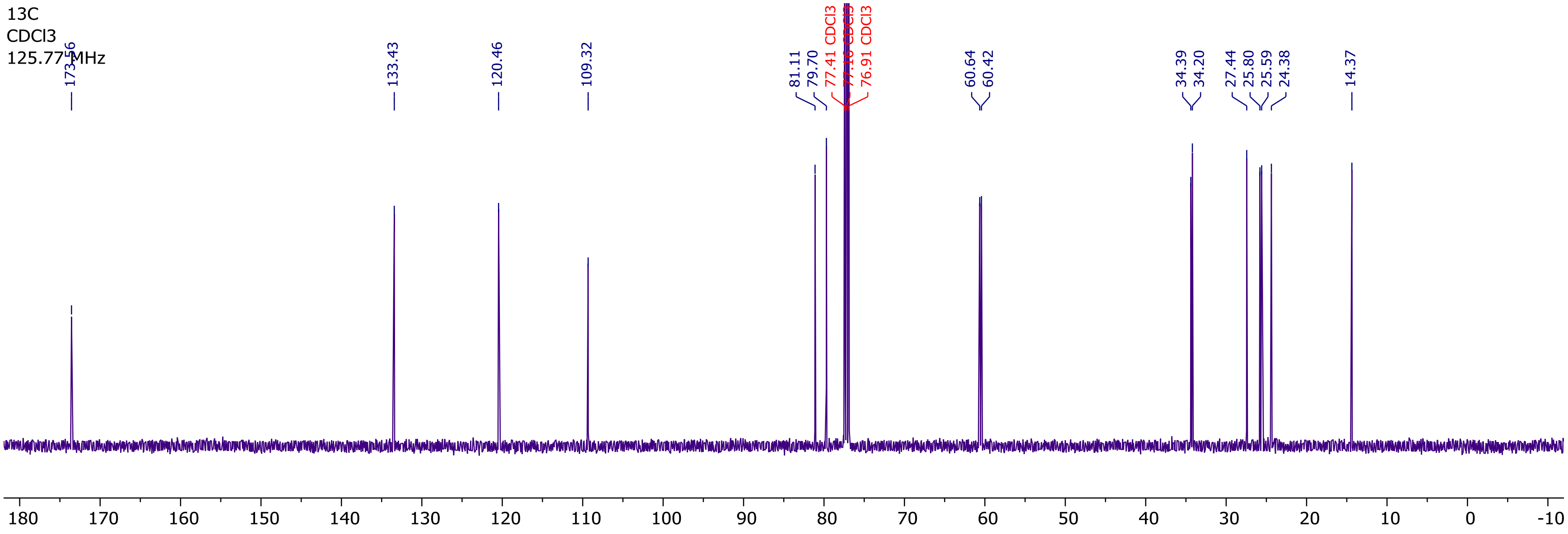
13C
CDCl3
125.77 MHz
173.56
133.43
120.46
109.32
81.11
79.70
77.41 CDCl3
77.16 CDCl3
76.91 CDCl3
60.64
60.42
34.39
34.20
27.44
25.80
25.59
24.38
14.37
180 170 160 150 140 130 120 110 100 90 80 70 60 50 40 30 20 10 0 -10

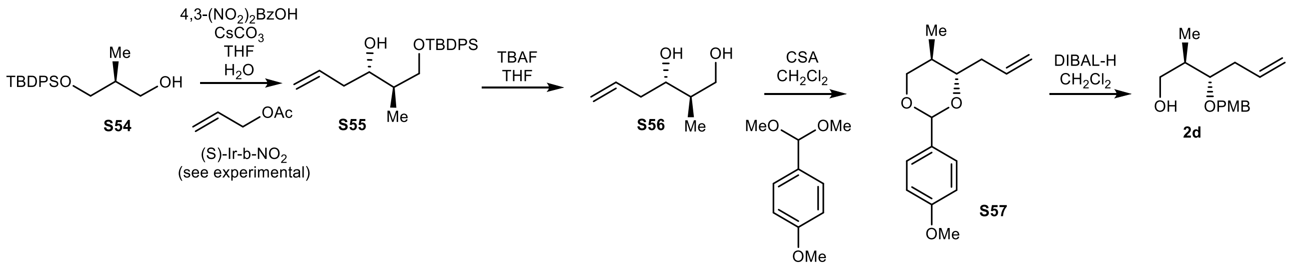


**Supplementary Fig. 35 | Synthesis of PMB-protected alcohol 2d.**

Abbreviations: TBDPS = tert-butyldiphenylsilyl, THF = tetrahydrofuran, TBAF = tetra-n-butylammonium fluoride, CSA = camphorsulfonic acid, DIBAL-H = diisobutylaluminum hydride, PMB = *para*-methoxybenzyl.

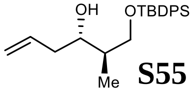


**S55**

**S55** was prepared from **S54** according to the procedure reported in the literature.[4] Spectral data agreed with the data previously reported in the literature, and optical rotation data was consistent in sign.[4,16]

**Analytical Data for S55:**

$R_f$ = 0.23 (19:1 hexanes/EtOAc)

$[\alpha]_D^{20}$ = -6.5 ° (c = 0.98, $CH_2Cl_2$); lit. $[\alpha]_D^{27}$ = -17 ° (c = 2.4, $CH_2Cl_2$)[4]; lit. $[\alpha]_D^{20}$ = -9.1 ° (c = 1.3, $CHCl_3$)[16]

$^1$H NMR (600 MHz, $CDCl_3$) δ 7.70 – 7.66 (m, 4H), 7.48 – 7.38 (m, 6H), 5.94 (ddt, *J* = 17.0, 9.4, 7.0 Hz, 1H), 5.16 – 5.09 (m, 2H), 3.77 (dd, *J* = 10.6, 4.0 Hz, 1H), 3.70 (td, *J* = 7.5, 3.5 Hz, 1H), 3.68 – 3.60 (m, 1H), 3.55 (s, 1H), 2.41 – 2.34 (m, 1H), 2.21 (dt, *J* = 14.7, 7.8 Hz, 1H), 1.83 (tt, *J* = 11.1, 5.9 Hz, 1H), 1.68 – 1.52 (m, 1H), 1.06 (s, 9H), 0.84 (d, *J* = 6.8 Hz, 3H).

$^{13}$C NMR (151 MHz, $CDCl_3$) δ 135.75, 135.72, 135.43, 132.98, 132.96, 129.99, 129.98, 127.92, 117.33, 75.35, 68.78, 39.55, 39.51, 26.98, 26.94, 19.23, 13.50.

HRMS (ESI): Anal. Calcd. for $C_{23}H_{33}O_2Si^+$ $[M+H]^+$ 369.2244, found 369.2227

IR (neat): $\nu_{max}$ $(cm^{-1})$ = 3497 (br, OH), 3072 (w, C=*CH*), 2391 (m, CH), 2859 (m, CH), 1641 (w, C=C)

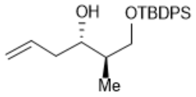
OH
OTBDPS
Me

**S55**

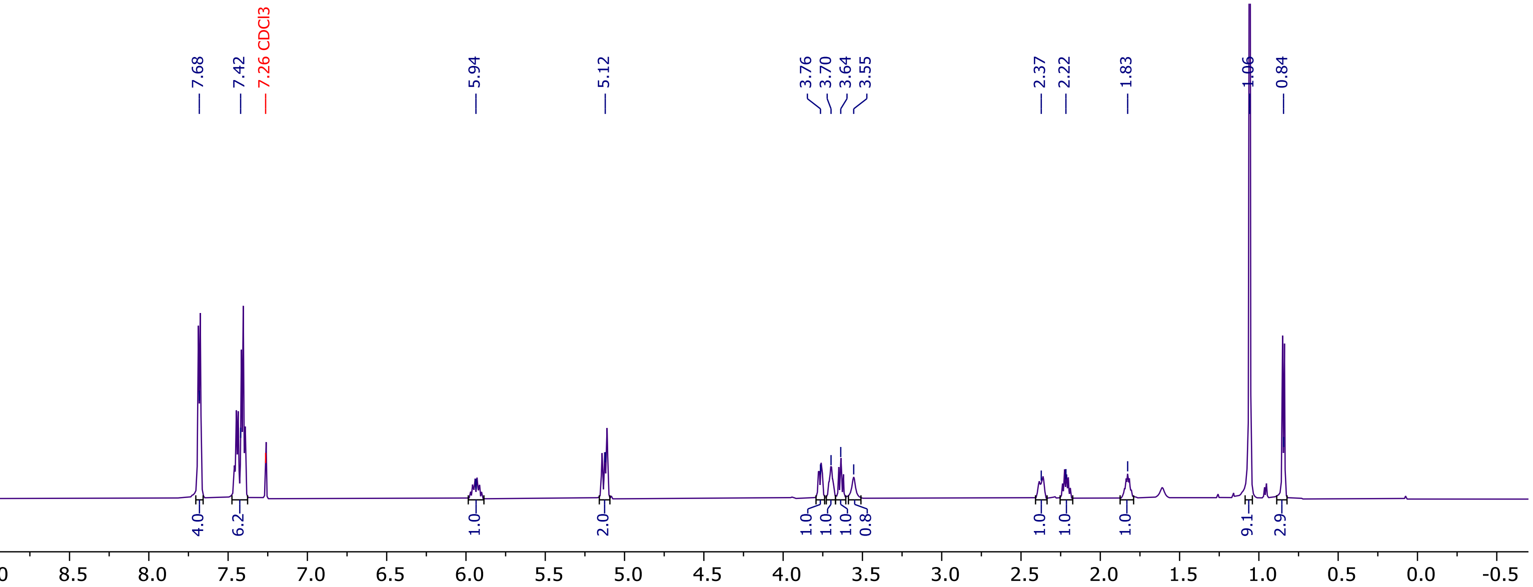
1H
CDCl3
600.13 MHz
7.68
7.42
7.26 CDCl3
5.94
5.12
3.76
3.70
3.64
3.55
2.37
2.22
1.83
1.06
0.84
4.0
6.2
1.0
2.0
1.0
1.0
1.0
0.8
1.0
1.0
1.0
9.1
2.9
10.0 9.5 9.0 8.5 8.0 7.5 7.0 6.5 6.0 5.5 5.0 4.5 4.0 3.5 3.0 2.5 2.0 1.5 1.0 0.5 0.0 -0.5

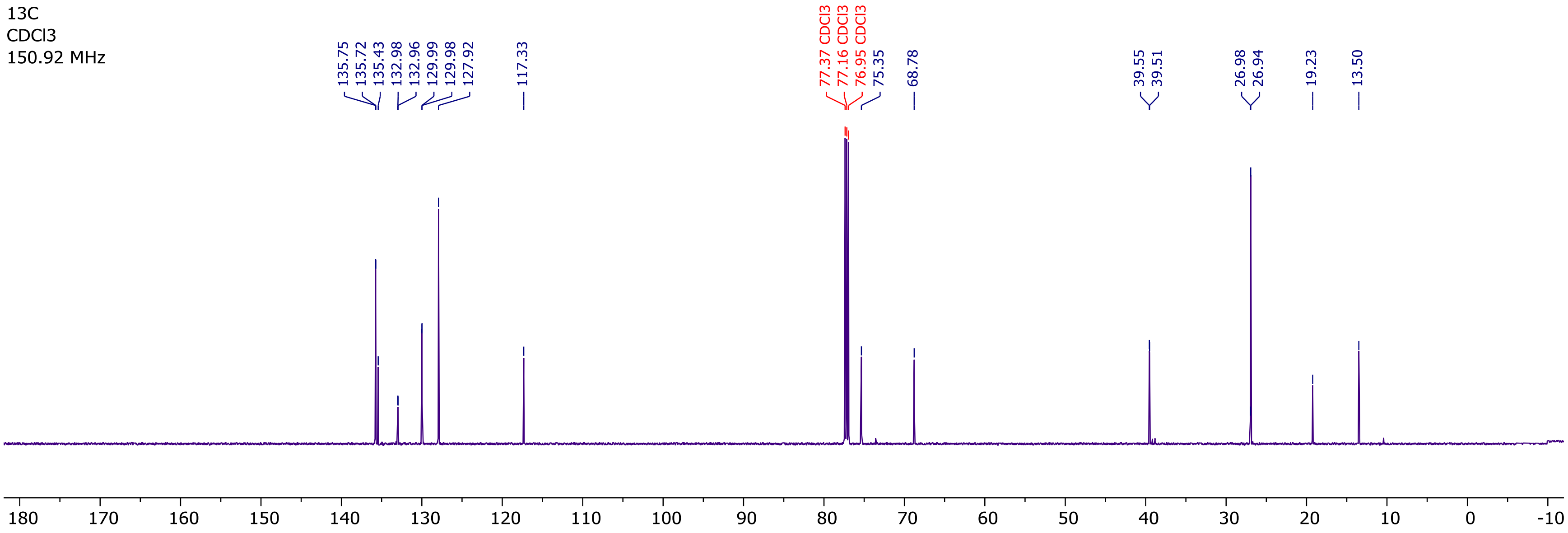
13C
CDCl3
150.92 MHz
135.75
135.72
135.43
132.98
132.96
129.99
129.98
127.92
117.33
77.37 CDCl3
77.16 CDCl3
76.95 CDCl3
75.35
68.78
39.55
39.51
26.98
26.94
19.23
13.50
180 170 160 150 140 130 120 110 100 90 80 70 60 50 40 30 20 10 0 -10

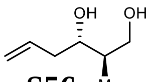

**S56**

To a stirred solution of **S55** (155 mg, 0.42 mmol, 1.0 eq.) in anhydrous THF (1.5 mL) at 0 °C was added a 1M solution of tetrabutylammonium fluoride in THF (0.63 mL, 0.63 mmol, 1.5 eq.). The reaction mixture was stirred for 20 min, after which time starting material was consumed as monitored by TLC analysis. The mixture was concentrated in vacuo, and the crude product was purified via flash column chromatography (4:1 to 1:1 hexanes/EtOAc). Appropriate fractions were pooled, and the solvent was removed in vacuo to yield **S56** (51.1 mg, 94%) as a colorless oil. Spectral data agreed with the data previously reported in the literature.[17]

**Analytical Data for S56:**

$R_f$ = 0.15 (7:3 hexanes/EtOAc)

$[\alpha]_D^{20}$ = -2.5 ° (c = 0.36, $CH_2Cl_2$)

$^1$H NMR (600 MHz, CDCl3) δ 5.84 (dtdd, J = 17.8, 12.3, 6.0, 3.1 Hz, 1H), 5.21 – 5.14 (m, 2H), 3.74 (dt, J = 11.0, 3.1 Hz, 1H), 3.65 (ddd, J = 10.5, 7.4, 2.3 Hz, 1H), 3.59 (td, J = 8.1, 4.0 Hz, 1H), 2.47 – 2.35 (m, 3H), 2.23 – 2.14 (m, 1H), 1.76 (ddq, J = 11.0, 7.4, 4.3 Hz, 1H), 0.90 (dd, J = 7.1, 2.4 Hz, 3H).

$^{13}$C NMR (151 MHz, $CDCl_3$) δ 134.52, 119.04, 76.06, 67.94, 40.15, 39.79, 13.90.

HRMS (ESI): Anal. Calcd. for $C_7H_{15}O_2^+$ $[M+H]^+$ 131.1067 found 131.1054

IR (neat): $\nu_{max}$ $(cm^{-1})$ = 3359 (br, OH), 2919 (m, CH), 1641 (w, C=C) 1466 (w), 1434 (w)

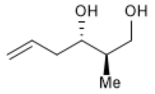

**S56**

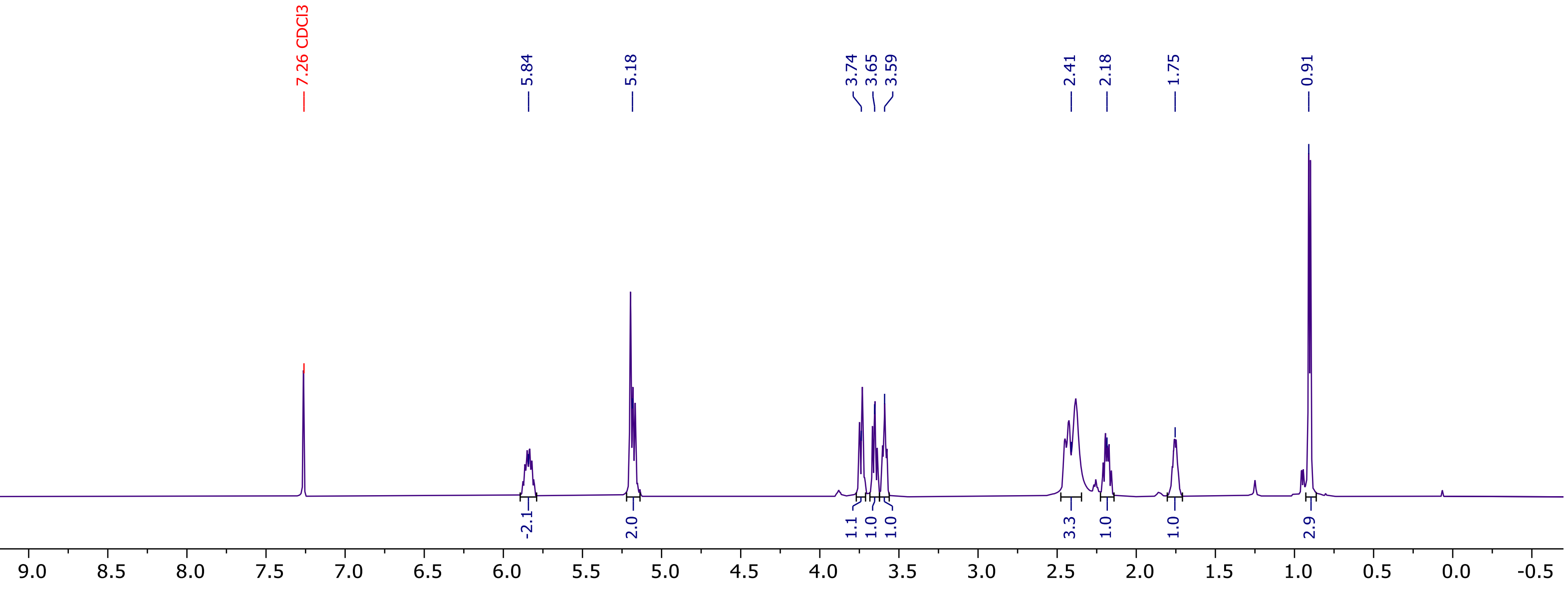
1H
CDCl3
600.13 MHz
7.26 CDCl3
5.84
5.18
3.74
3.65
3.59
2.41
2.18
1.75
0.91
-2.1
2.0
1.1
1.0
1.0
3.3
1.0
1.0
2.9
10.0 9.5 9.0 8.5 8.0 7.5 7.0 6.5 6.0 5.5 5.0 4.5 4.0 3.5 3.0 2.5 2.0 1.5 1.0 0.5 0.0 -0.5

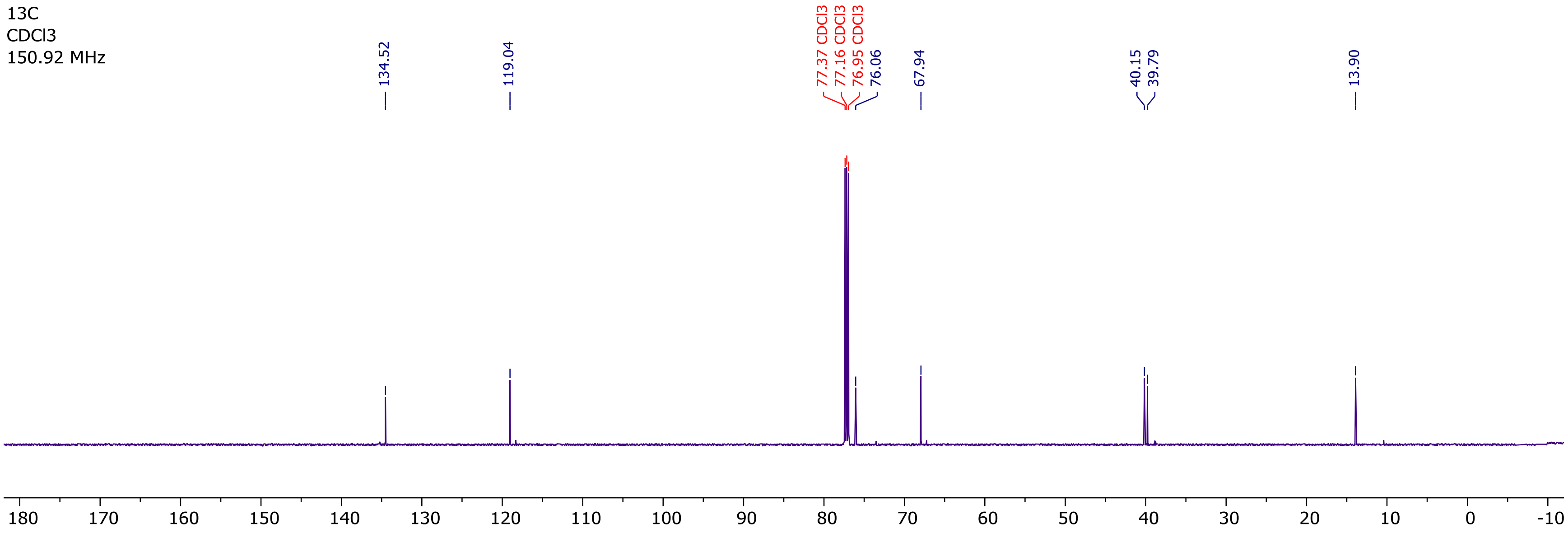
13C
CDCl3
150.92 MHz
134.52
119.04
77.37 CDCl3
77.16 CDCl3
76.95 CDCl3
76.06
67.94
40.15
39.79
13.90
180 170 160 150 140 130 120 110 100 90 80 70 60 50 40 30 20 10 0 -10

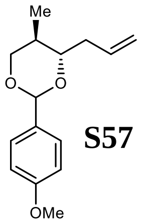

**S57**

Note: Reaction performed in capped vessel under ambient atmosphere.

To a rt stirred solution of **S56** (51.1 mg, 0.39 mmol, 1.0 eq.) and anisaldehyde dimethyl acetal (0.1 mL, 0.59 mmol, 1.5 eq.) dissolved in $CH_2Cl_2$ (3.9 mL), was added CSA (19.9 mg, 0.086 mmol, 0.22 eq.). The reaction mixture was stirred for 150 minutes at rt, after which time starting material was consumed as monitored by TLC analysis. The reaction mixture was quenched with saturated aqueous $NaHCO_3$ (5 mL) and the aqueous layer was extracted with $CH_2Cl_2$ (3x 5mL). The combined organic layers were washed with brine (1x 10 mL), then $H_2O$ (20 mL) and $NaHSO_3$ (946 mg, 9.09 mmol) were added in sequence at rt. The mixture was vigorously stirred at rt for 1 h. After this time, the reaction mixture was poured into a separatory funnel, the organic layer drained, and the aqueous layer extracted with $CH_2Cl_2$ (2x 5mL). The combined organic layers were dried ($Na_2SO_4$), filtered, and solvent was removed in vacuo. The crude product was purified via flash column chromatography (19:1 to 9:1 hexanes/$Et_2O$). Appropriate fractions were pooled, and solvent was removed in vacuo to yield **S57** (78.4 mg, 80%) as a colorless oil. Spectral and optical rotation data agreed with the data previously reported in the literature for **ent-S57**.[18]

**Analytical Data for S57:**

$R_f$ = 0.50 (4:1 hexanes/$Et_2O$)

$[\alpha]_D^{20}$ = -25 ° (c = 0.52, $CH_2Cl_2$); lit. $[\alpha]_D^{30}$ = +25.8 ° (c = 2.1, $CHCl_3$) for **ent-S57**[18]

$^1$H NMR (600 MHz, $CDCl_3$) δ 7.42 (dd, *J* = 9.1, 2.6 Hz, 2H), 6.88 (dd, *J* = 9.0, 2.6 Hz, 2H), 5.99 (ddt, *J* = 17.2, 10.2, 7.0 Hz, 1H), 5.44 (s, 1H), 5.12 (dd, *J* = 17.0, 2.0 Hz, 1H), 5.08 (dd, *J* = 10.2, 2.2 Hz, 1H), 4.08 (dd, *J* = 11.3, 4.8 Hz, 1H), 3.80 (s, 3H), 3.49 (td, *J* = 11.3, 10.8, 3.2 Hz, 2H), 2.54 – 2.47 (m, 1H), 2.33 (dt, *J* = 14.7, 7.2 Hz, 1H), 1.90 (dddd, *J* = 14.8, 10.9, 6.8, 4.6 Hz, 1H), 0.79 (d, *J* = 6.7 Hz, 3H).

$^{13}$C NMR (151 MHz, $CDCl_3$) δ 159.94, 134.78, 131.39, 127.46, 116.90, 113.68, 101.10, 82.69, 73.02, 55.43, 37.26, 33.38, 12.43.
HRMS (ESI): Anal. Calcd. for $C_{15}H_{21}O_3^+$ $[M+H]^+$ 249.1485, found 249.1478

IR (neat): $\nu_{max}$ $(cm^{-1})$ = 2956 (m, CH), 2840 (m, CH), 1615 (m, C=C), 1517 (m), 1462 (m), 1391 (m), 1304 (m), 1250 (s, C-O)

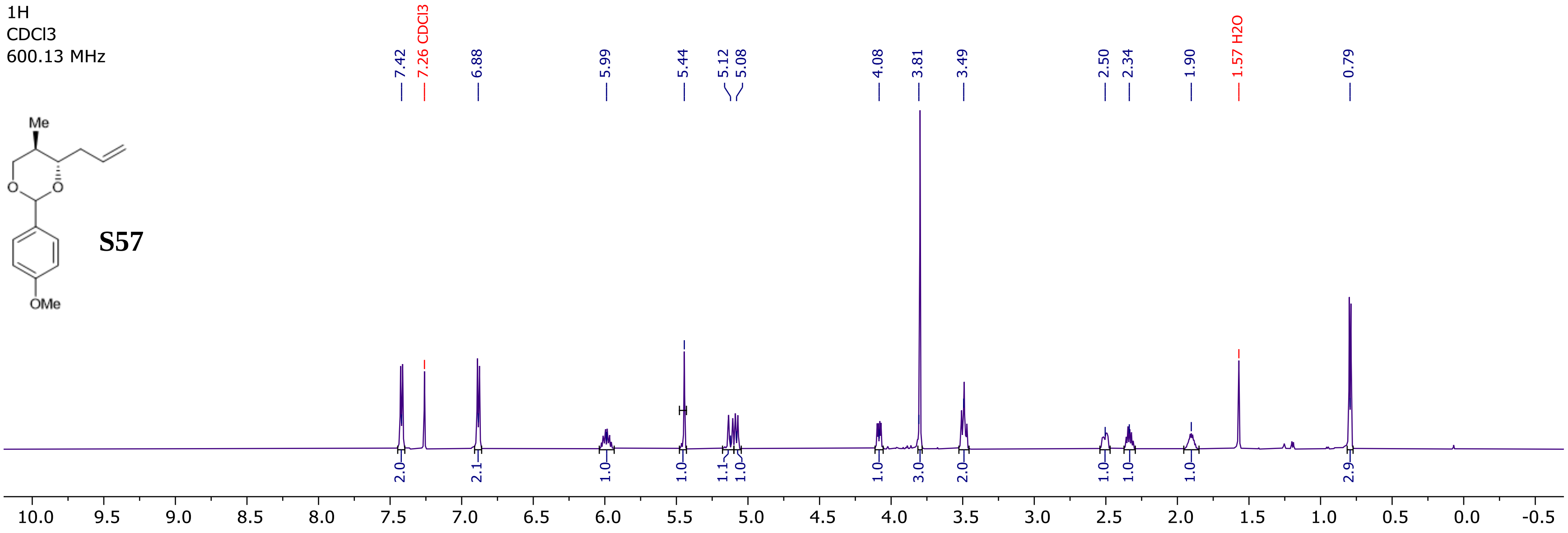
1H
CDCl3
600.13 MHz
Me
O
O
OMe
S57
7.42
7.26 CDCl3
6.88
5.99
5.44
5.12
5.08
4.08
3.81
3.49
2.50
2.34
1.90
1.57 H2O
0.79
2.0
2.1
1.0
1.0
1.1
1.0
1.0
3.0
2.0
1.0
1.0
1.0
2.9
10.0 9.5 9.0 8.5 8.0 7.5 7.0 6.5 6.0 5.5 5.0 4.5 4.0 3.5 3.0 2.5 2.0 1.5 1.0 0.5 0.0 -0.5

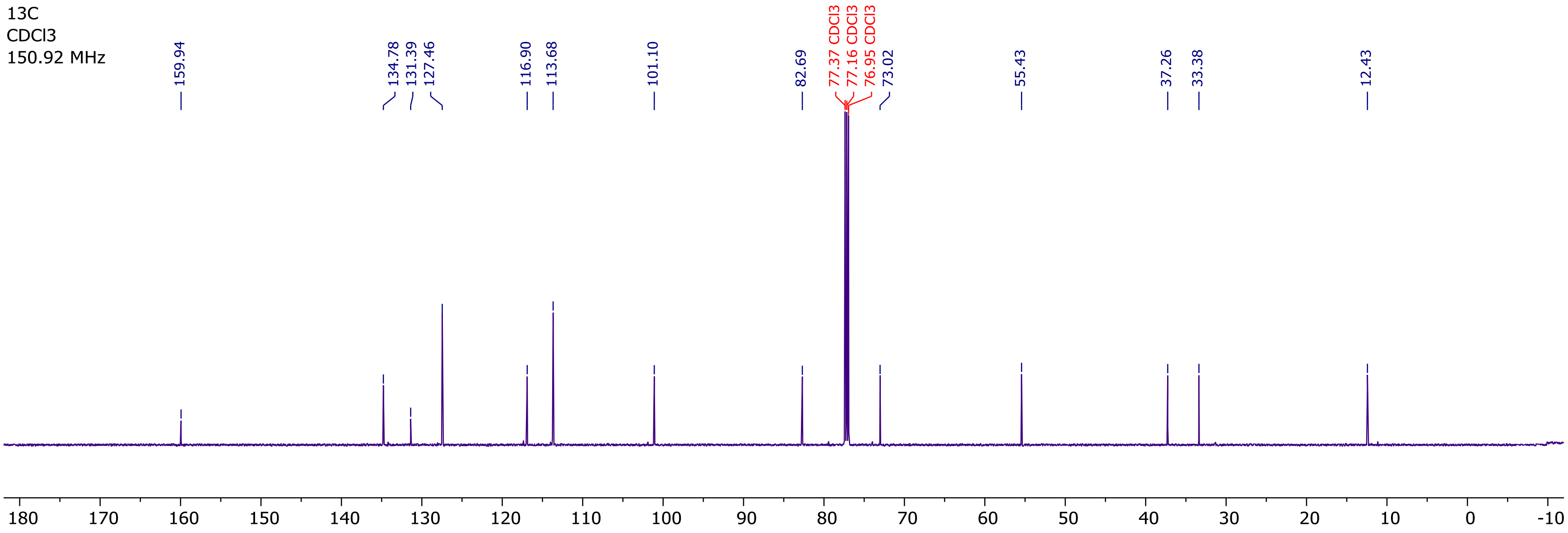
13C
CDCl3
150.92 MHz
159.94
134.78
131.39
127.46
116.90
113.68
101.10
82.69
77.37 CDCl3
77.16 CDCl3
76.95 CDCl3
73.02
55.43
37.26
33.38
12.43
180 170 160 150 140 130 120 110 100 90 80 70 60 50 40 30 20 10 0 -10

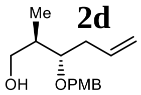
**2d**

To a 0 °C stirred solution of **S57** (7.4 mg, 0.32 mmol, 1.0 eq.) dissolved in anhydrous $CH_2Cl_2$ (2.5 mL), was added a 1M solution DIBAL-H in $CH_2Cl_2$ (0.63 mL, 0.63 mmol, 2 eq.). The reaction mixture was warmed to rt and stirred for 2 h, after which time starting material was consumed as monitored by TLC analysis. The reaction mixture quenched with a saturated solution of Rochelle's salt (2 mL) and stirred vigorously overnight. $H_2O$ was added (2 mL) and the aqueous layer was extracted with $CH_2Cl_2$ (3x 10 mL). The combined organic layers were dried ($Na_2SO_4$), filtered, and solvent was removed in vacuo. The crude product was purified via flash column chromatography (7:3 hexanes/$Et_2O$). Appropriate fractions were pooled, and solvent was removed in vacuo to yield **2d** (70.1 mg, 89%) as a colorless oil. Spectral and optical rotation data agreed with the data previously reported in the literature for **ent-2d**.[5]

**Analytical Data for 2d:**

$R_f$ = 0.14 (7:3 hexanes/$Et_2O$)

$[\alpha]_D^{20}$ = +56.7 ° (c = 1.92, $CH_2Cl_2$); lit. $[\alpha]_D^{22}$ = -60.7 ° (c = 1.0, $CHCl_3$) for **ent-2d**[5]

$^1$H NMR (600 MHz, $CDCl_3$) δ 7.26 (d, *J* = 8.2 Hz, 2H), 6.88 (d, *J* = 8.2 Hz, 2H), 5.88 (td, *J* = 17.0, 7.3 Hz, 1H), 5.18 – 5.08 (m, 2H), 4.61 (dd, *J* = 11.0, 2.2 Hz, 1H), 4.38 (dd, *J* = 10.9, 2.3 Hz, 1H), 3.80 (s, 3H), 3.66 (ddd, *J* = 10.9, 3.5, 1.9 Hz, 1H), 3.55 (dd, *J* = 10.9, 6.7 Hz, 1H), 3.46 – 3.41 (m, 1H), 2.54 – 2.47 (m, 1H), 2.38 – 2.30 (m, 1H), 1.89 (dhept, *J* = 10.0, 4.1, 3.3 Hz, 1H), 0.91 (d, *J* = 7.0 Hz, 3H).

$^{13}$C NMR (151 MHz, $CDCl_3$) δ 159.42, 134.22, 130.20, 129.68, 117.58, 114.00, 83.35, 71.47, 66.86, 55.39, 38.01, 35.49, 14.24.

HRMS (ESI): Anal. Calcd. for $C_{15}H_{23}O_3^+$ $[M+H]^+$ 251.1642, found 251.1636

IR (neat): $\nu_{max}$ $(cm^{-1})$ = 3414 (br, OH), 2918 (m, CH), 1641 (w, C=C), 1613 (m, C=C), 1513 (s), 1465 (w), 1301 (w), 1248 (s, C-O)

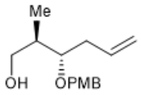
Me
OH
OPMB

**2d**

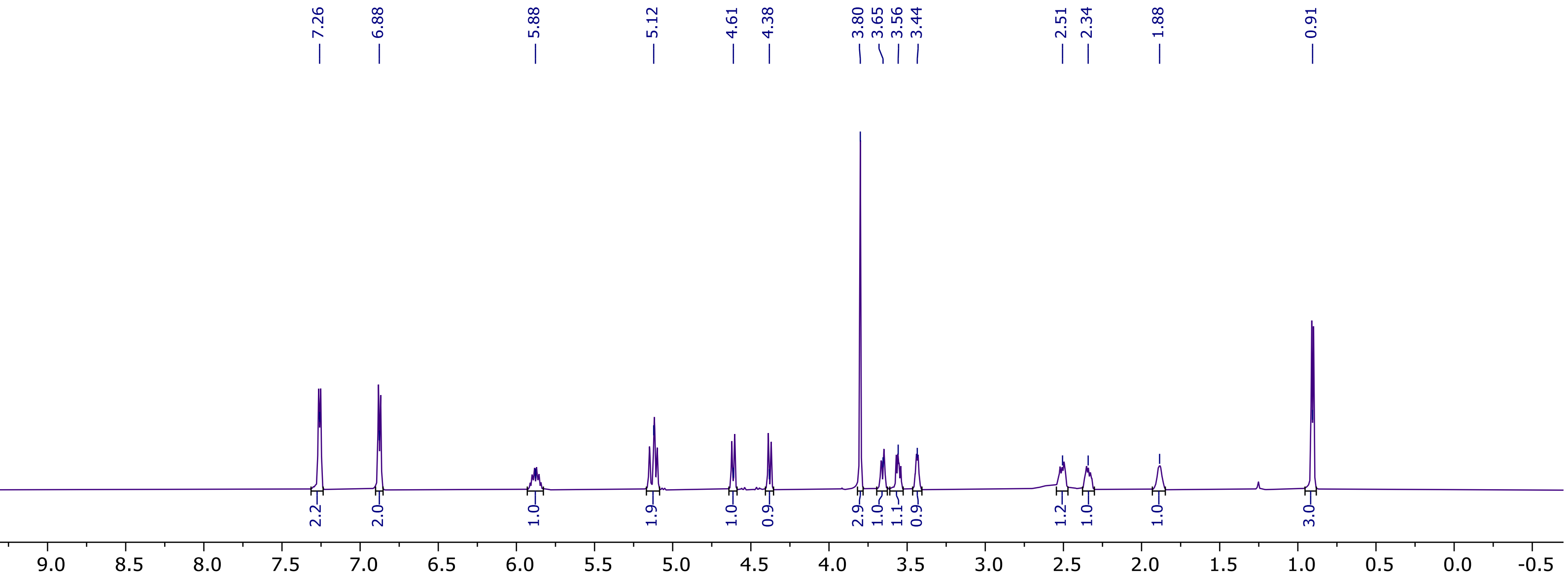
1H
CDCl3
600.13 MHz
7.26
6.88
5.88
5.12
4.61
4.38
3.80
3.65
3.56
3.44
2.51
2.34
1.88
0.91
2.2
2.0
1.0
1.9
1.0
0.9
2.9
1.0
1.1
0.9
1.2
1.0
1.0
3.0
10.0 9.5 9.0 8.5 8.0 7.5 7.0 6.5 6.0 5.5 5.0 4.5 4.0 3.5 3.0 2.5 2.0 1.5 1.0 0.5 0.0 -0.5

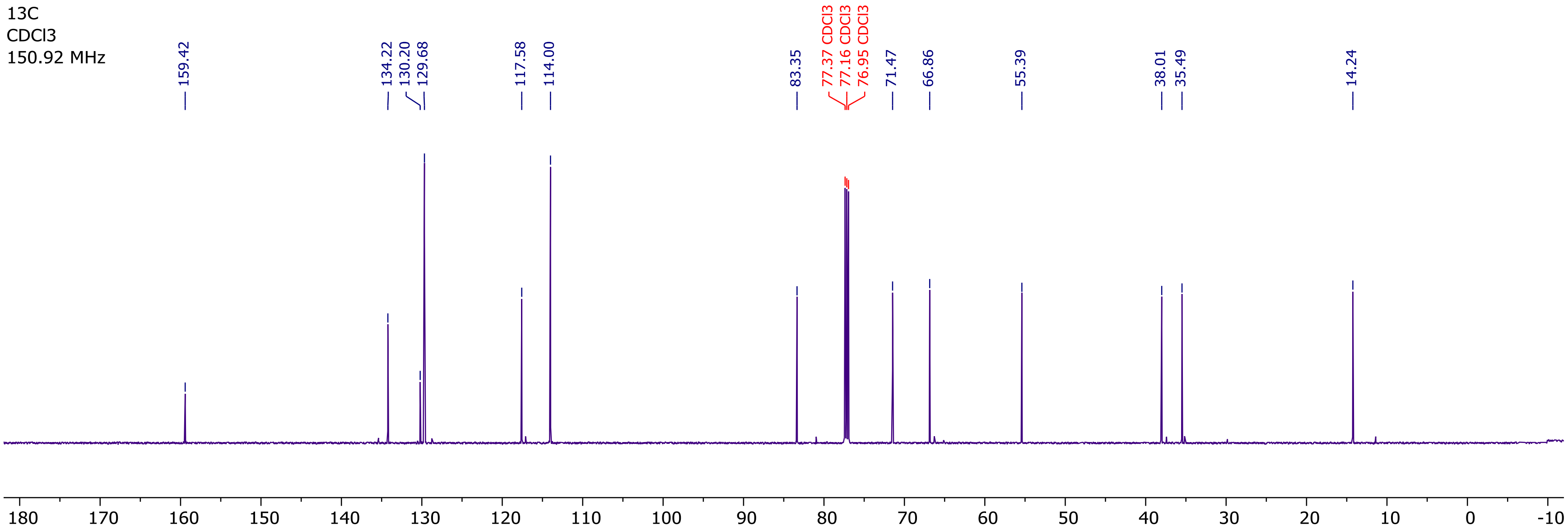
13C
CDCl3
150.92 MHz
159.42
134.22
130.20
129.68
117.58
114.00
83.35
77.37 CDCl3
77.16 CDCl3
76.95 CDCl3
71.47
66.86
55.39
38.01
35.49
14.24
180 170 160 150 140 130 120 110 100 90 80 70 60 50 40 30 20 10 0 -10

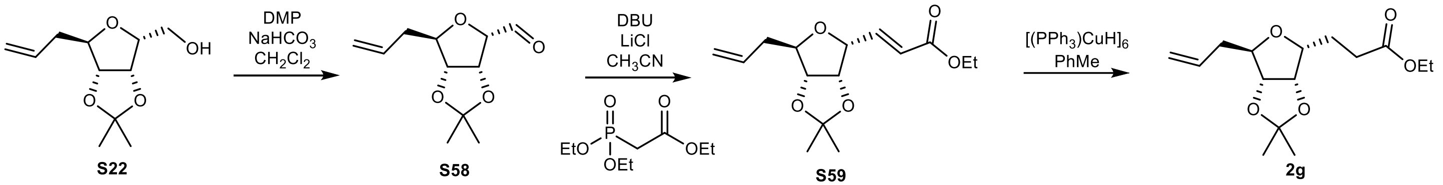


**Supplementary Fig. 36 | Synthesis of Tetrahydrofuranoate 2g.**
Abbreviations: DMP = Dess-Martin periodinane, DBU = 1,8-Diazabicyclo [5.4.0]undec-7-ene.

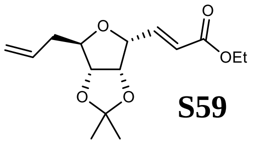


**S59**

Note: Reaction performed in capped vessel under ambient atmosphere.

To a stirred solution of **S22** (574 mg, 2.68 mmol, 1.0 eq.) in anhydrous $CH_2Cl_2$ (27.3 mL) was added $NaHCO_3$ (688 mg, 8.19 mmol, 3.1 eq.) followed by Dess-Martin Periodinane (1.737 g, 4.10 mmol, 1.5 eq.). The mixture was stirred at rt for 90 minutes and quenched with a solution of 1:1:1 $H_2O$/saturated aqueous $NaHCO_3$/saturated aqueous $Na_2S_2O_3$ (27 mL) and the biphasic mixture was stirred vigorously for 1 h. The aqueous layer was extracted with $CH_2Cl_2$ (3x 50 mL) and the combined organic layers were dried ($Na_2SO_4$), and the solvent was removed in vacuo. The crude product was run through a plug of silica gel with EtOAc and the solvent was removed in vacuo to yield the crude aldehyde **S58** (545 mg), which was used immediately in the next step.

To a stirred solution of diethyl phosphonoacetic acid ethyl ester (545 mg, 2.57 mmol, 1.0 eq.) and LiCl (231 mg, 5.46 mmol, 2.1 eq.) in anhydrous $CH_3CN$ (13.7 mL) was added DBU (613 µL, 624 mg, 4.10 mmol, 1.6 eq.) followed by crude aldehyde **S58** (545 mg, 2.57 mmol, 1.0 eq.) in anhydrous $CH_3CN$ (1.5 mL) and a few drops of $Et_2O$ to improve solubility. The reaction mixture was stirred for 30 minutes at rt, after which time starting material was consumed as monitored by TLC analysis. The reaction was quenched with a saturated solution of $NH_4Cl$ (25 mL) and the aqueous layer was extracted with $CH_2Cl_2$ (3x 30 mL). The combined organic layers were washed with brine (1x 50 mL), dried ($Na_2SO_4$), and the solvent was removed in vacuo. The crude product was purified via flash column chromatography (9:1 hexanes/EtOAc). Appropriate fractions were pooled, and the solvent was removed in vacuo to yield **S59** (557 mg, 72% over 2 steps) as a yellow oil.

**Analytical Data for S59:**

$R_f$ = 0.18 (9:1 hexanes/EtOAc)

$[\alpha]_D^{20}$ = -42 ° (c = 0.66, $CH_2Cl_2$)

$^{1}$H NMR (600 MHz, $CDCl_3$) δ 6.96 (dd, *J* = 15.8, 5.4 Hz, 1H), 6.11 (dd, *J* = 15.7, 1.5 Hz, 1H), 5.79 (ddt, *J* = 17.0, 9.9, 6.8 Hz, 1H), 5.14 (d, *J* = 4.8 Hz, 1H), 5.12 (s, 1H), 4.78 (dd, *J* = 6.0, 4.3 Hz, 1H), 4.58 (d, *J* = 6.0 Hz, 1H), 4.48 (t, *J* = 4.9 Hz, 1H), 4.20 (th, *J* = 7.4, 3.8 Hz, 3H), 2.29 (dt, *J* = 14.0, 6.9 Hz, 1H), 2.21 (dt, *J* = 14.5, 7.4 Hz, 1H), 1.46 (s, 3H), 1.31 (s, 3H), 1.28 (t, *J* = 7.1 Hz, 3H).

$^{13}$C NMR (151 MHz, $CDCl_3$) δ 166.15, 142.08, 133.52, 123.18, 118.22, 113.40, 84.84, 83.40, 82.46, 79.45, 60.57, 35.84, 26.37, 25.30, 14.37.

HRMS (ESI): Anal. Calcd. for $C_{15}H_{26}NO_5^+$ $[M+NH_4]^+$ 300.1805, found 300.1796

IR (neat): $\nu_{max}$ $(cm^{-1})$ = 2985 (m, CH), 1721 (s, C=O), 1712 (s), 1665 (w), 1382 (m), 1371 (m), 1303 (m), 1267 (s)

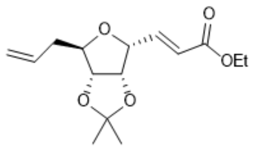

O
O
O
O
OEt


**S59**

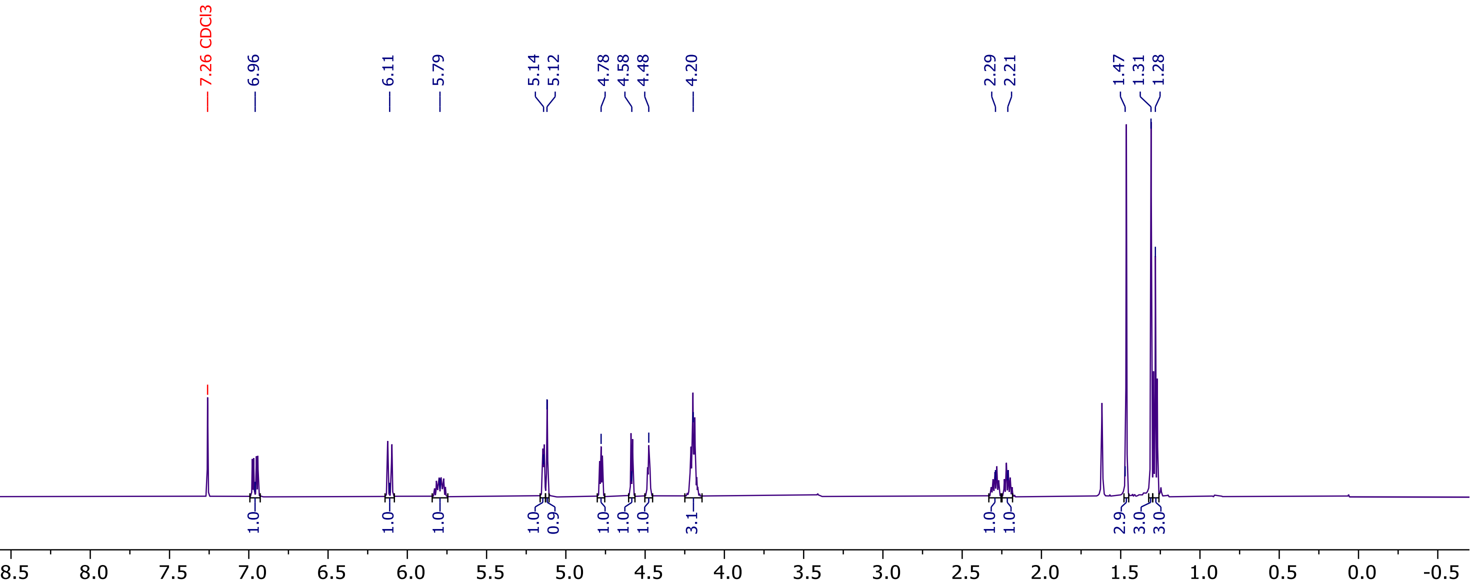

1H
CDCl3
600.13 MHz
7.26 CDCl3
6.96
6.11
5.79
5.14
5.12
4.78
4.58
4.48
4.20
2.29
2.21
1.47
1.31
1.28
1.0
1.0
1.0
1.0
0.9
1.0
1.0
1.0
3.1
1.0
1.0
2.9
3.0
3.0
10.0 9.5 9.0 8.5 8.0 7.5 7.0 6.5 6.0 5.5 5.0 4.5 4.0 3.5 3.0 2.5 2.0 1.5 1.0 0.5 0.0 -0.5


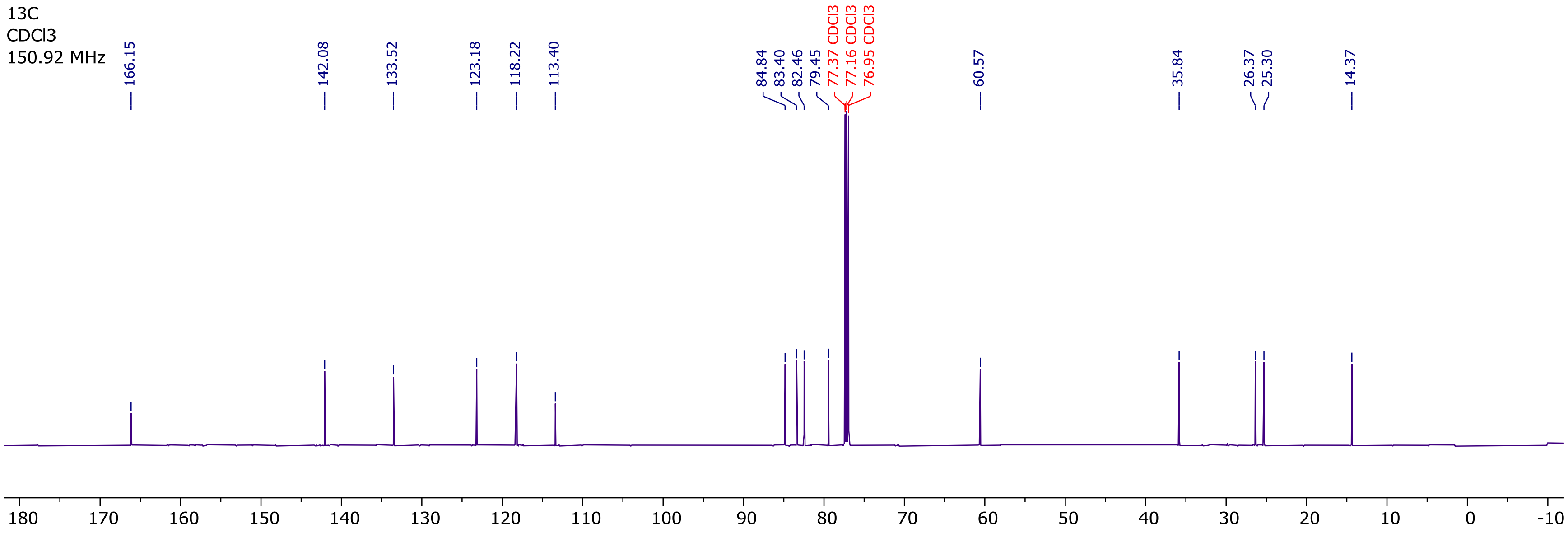

13C
CDCl3
150.92 MHz
166.15
142.08
133.52
123.18
118.22
113.40
84.84
83.40
82.46
79.45
77.37 CDCl3
77.16 CDCl3
76.95 CDCl3
60.57
35.84
26.37
25.30
14.37
180 170 160 150 140 130 120 110 100 90 80 70 60 50 40 30 20 10 0 -10

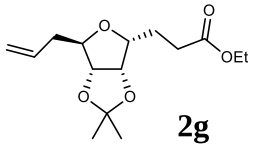


**2g**

To a stirred solution of **S59** (551 mg, 1.95 mmol, 1.0 eq) in anhydrous, deoxygenated toluene (19.5 mL) was added Stryker's reagent in anhydrous, deoxygenated toluene (0.40 M, 19.5 mL, 7.8 mmol, 4.0 eq.). The mixture was stirred for 90 minutes after which more Stryker's reagent in toluene (1.95 mL, 1.0 eq.) was added. After an additional 2 h, the reaction mixture was flushed through a plug of silica gel with $Et_2O$ and the solvent was removed in vacuo. The crude product was purified via flash column chromatography (9:1 to 3:1 hexanes/$Et_2O$). Appropriate fractions were pooled, and solvent was removed in vacuo to yield **2g** (301 mg, 55%) as a yellow oil.

**Analytical Data for 2g:**

$R_f$ = 0.32 (3:1 hexanes/$Et_2O$)

$[\alpha]_D^{20}$ = +10 ° (c = 0.58, $CH_2Cl_2$)

$^1$H NMR (600 MHz, $CDCl_3$) δ 5.77 (dt, *J* = 17.0, 9.5, 7.0 Hz, 1H), 5.10 (s, 1H), 5.08 (d, *J* = 4.2 Hz, 1H), 4.62 (dd, *J* = 6.1, 3.8 Hz, 1H), 4.52 (d, *J* = 6.1 Hz, 1H), 4.13 (q, *J* = 7.1 Hz, 2H), 4.07 (t, *J* = 7.6 Hz, 1H), 3.82 (ddd, *J* = 8.1, 5.7, 3.8 Hz, 1H), 2.45 (td, *J* = 7.6, 4.0 Hz, 2H), 2.28 – 2.19 (m, 1H), 2.19 – 2.10 (m, 1H), 2.08 – 1.95 (m, 2H), 1.48 (s, 3H), 1.32 (s, 3H), 1.25 (t, *J* = 7.1 Hz, 3H).

$^{13}$C NMR (151 MHz, $CDCl_3$) δ 173.54, 133.91, 117.80, 112.60, 84.84, 82.85, 81.54, 78.76, 60.48, 35.59, 31.05, 26.38, 25.22, 24.40, 14.37.

HRMS (ESI): Anal. Calcd. for $C_{15}H_{25}O_5^+$ $[M+H]^+$ 285.1697, found 285.1672

IR (neat): $\nu_{max}$ $(cm^{-1})$ = 2982 (m, CH), 2939 (m, CH), 1726 (s, C=O), 1642 (w, C=C), 1442 (w), 1372 (m), 1259 (s), 1209 (s)

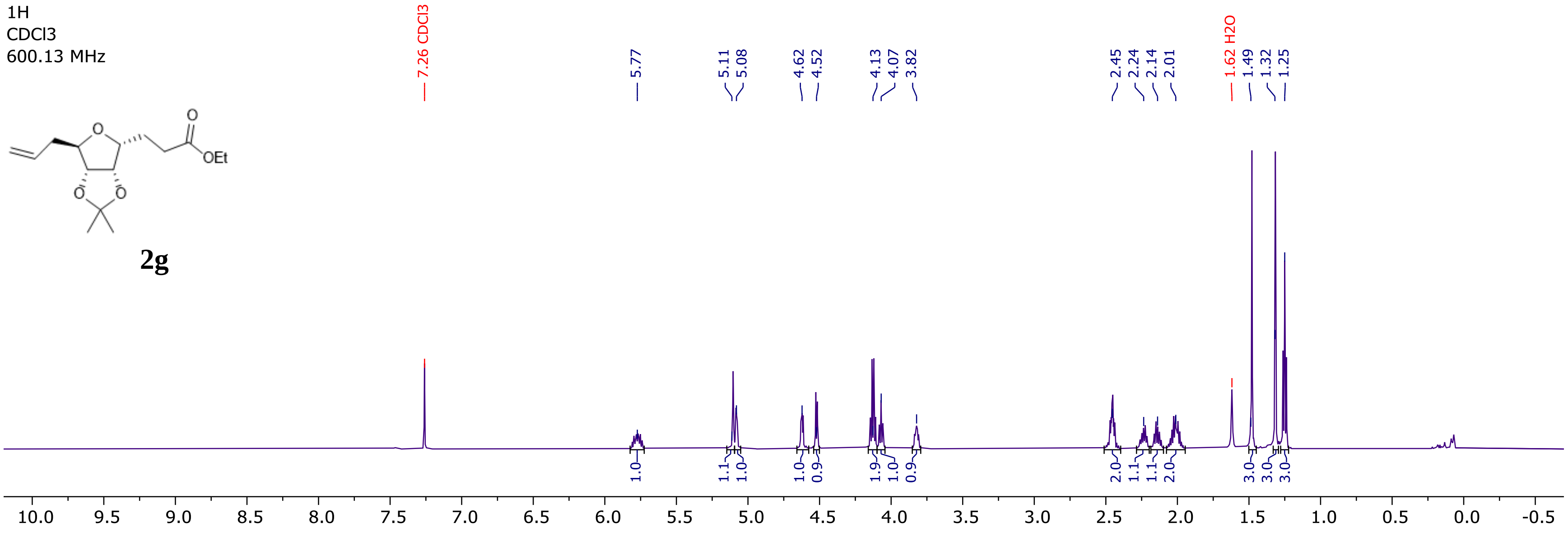
1H
CDCl3
600.13 MHz
2g
OEt
O
7.26 CDCl3
5.77
5.11
5.08
4.62
4.52
4.13
4.07
3.82
2.45
2.24
2.14
2.01
1.62 H2O
1.49
1.32
1.25
1.0
1.1
1.0
1.0
0.9
1.9
1.0
0.9
2.0
1.1
1.1
2.0
3.0
3.0
3.0
10.0
9.5
9.0
8.5
8.0
7.5
7.0
6.5
6.0
5.5
5.0
4.5
4.0
3.5
3.0
2.5
2.0
1.5
1.0
0.5
0.0
-0.5

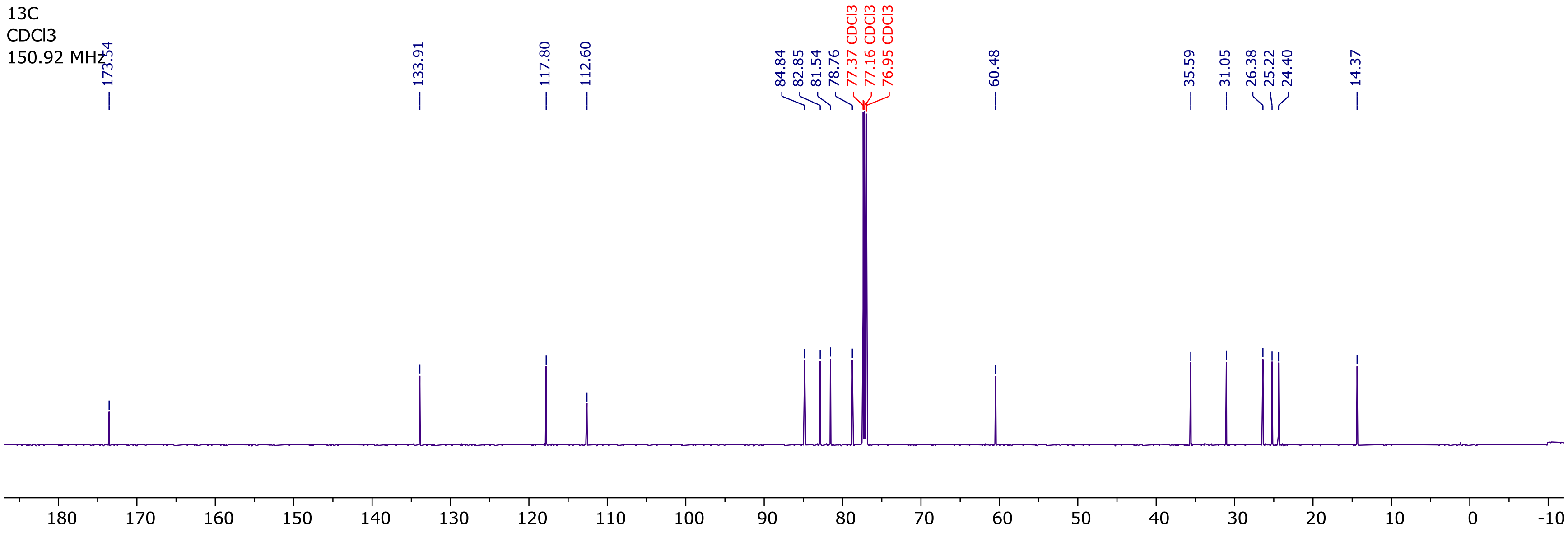
13C
CDCl3
150.92 MHz
173.54
133.91
117.80
112.60
84.84
82.85
81.54
78.76
77.37 CDCl3
77.16 CDCl3
76.95 CDCl3
60.48
35.59
31.05
26.38
25.22
24.40
14.37
180
170
160
150
140
130
120
110
100
90
80
70
60
50
40
30
20
10
0
-10

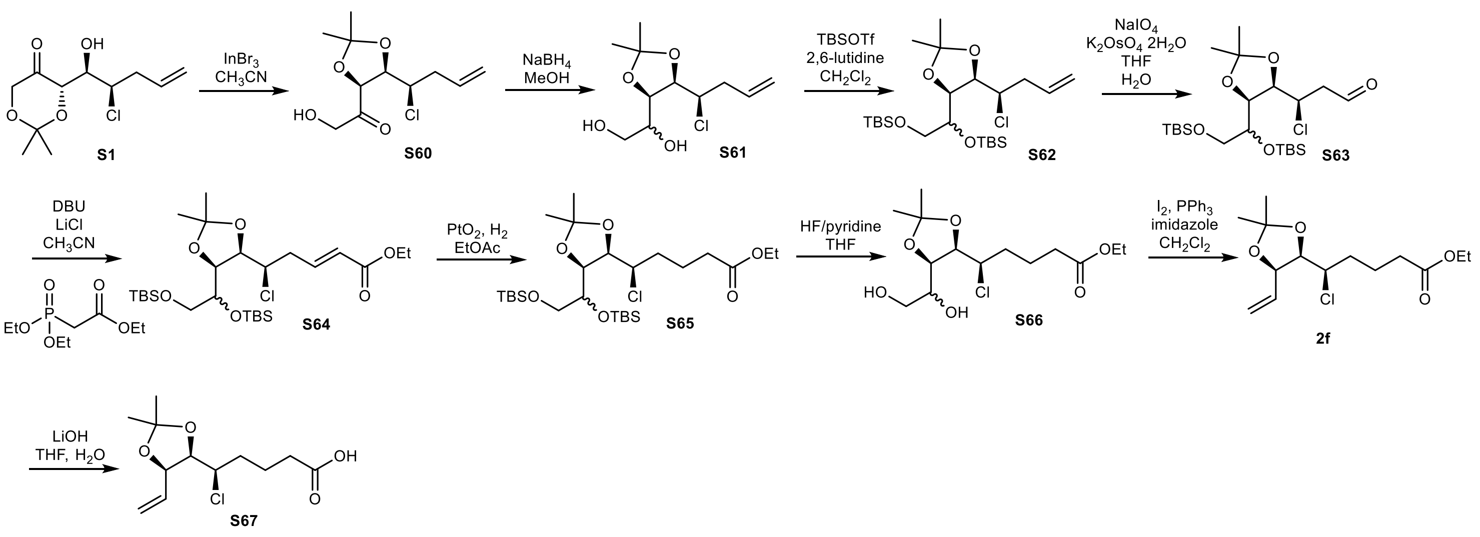


**Supplementary Fig. 37 | Synthesis of Chlorinated Ester 2f.**

Abbreviations: TBSOTf = tert-butyldimethylsilyl trifluoromethanesulfonate, TBS = tert-butyldimethylsilyl, THF = tetrahydrofuran, DBU = 1,8-Diazabicyclo [5.4.0]undec-7-ene.

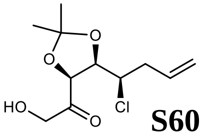

**S60** To a rt stirred solution of **S1** (2.49 g, 10.0 mmol, 1.0 eq.) dissolved in anhydrous $CH_3N$ (50 mL), was added (quickly to avoid the absorption of water) indium (III) bromide (354 mg, 1.00 mmol, 0.1 eq.). The reaction mixture was stirred for 30 minutes at rt, after which time an optimal quantity of product was formed as monitored by TLC analysis. The reaction mixture was quenched with a saturated solution of $NH_4Cl$ (50 mL) and the aqueous layer was extracted with $CH_2Cl_2$ (3x 50 mL). The combined organic layers were dried ($Na_2SO_4$), filtered, and solvent was removed in vacuo. The crude product was purified via flash column chromatography (17:3 to 3:1 hexanes/EtOAc). Appropriate fractions were pooled, and solvent was removed in vacuo to yield **S60** (1.54 g, 62%) as a colorless oil.

**Analytical Data for S60:**

$R_f$ = 0.16 (17:3 hexanes:EtOAc)

$[\alpha]_D^{20}$ = -45.1 ° (c = 1.79, $CH_2Cl_2$)

$^1H$ NMR (500 MHz, $CD_2Cl_2$) δ 5.84 (ddt, *J* = 17.1, 10.2, 7.0 Hz, 1H), 5.22 – 5.14 (m, 2H), 4.77 (dd, *J* = 20.4, 5.3 Hz, 1H), 4.70 – 4.64 (m, 2H), 4.43 (ddd, *J* = 20.4, 5.0, 0.7 Hz, 1H), 4.11 (td, *J* = 7.3, 1.6 Hz, 1H), 2.86 (t, *J* = 5.1 Hz, 1H), 2.60 (tt, *J* = 7.0, 1.3 Hz, 2H), 1.61 (s, 3H), 1.39 (s, 3H).

$^{13}C$ NMR (126 MHz, $CD_2Cl_2$) δ 210.64, 134.23, 118.64, 111.01, 80.48, 79.92, 68.11, 59.80, 40.12, 25.94, 24.51.

HRMS (ESI): Anal. Calcd. for $C_{11}H_{18}ClO_4^+$ $[M+H]^+$ 249.0888, found 249.0865

IR (neat): $\nu_{max}$ $(cm^{-1})$ = 3395 (br, OH), 2921 (w, CH), 1726 (s, C=O), 1382 (s), 1217 (s)

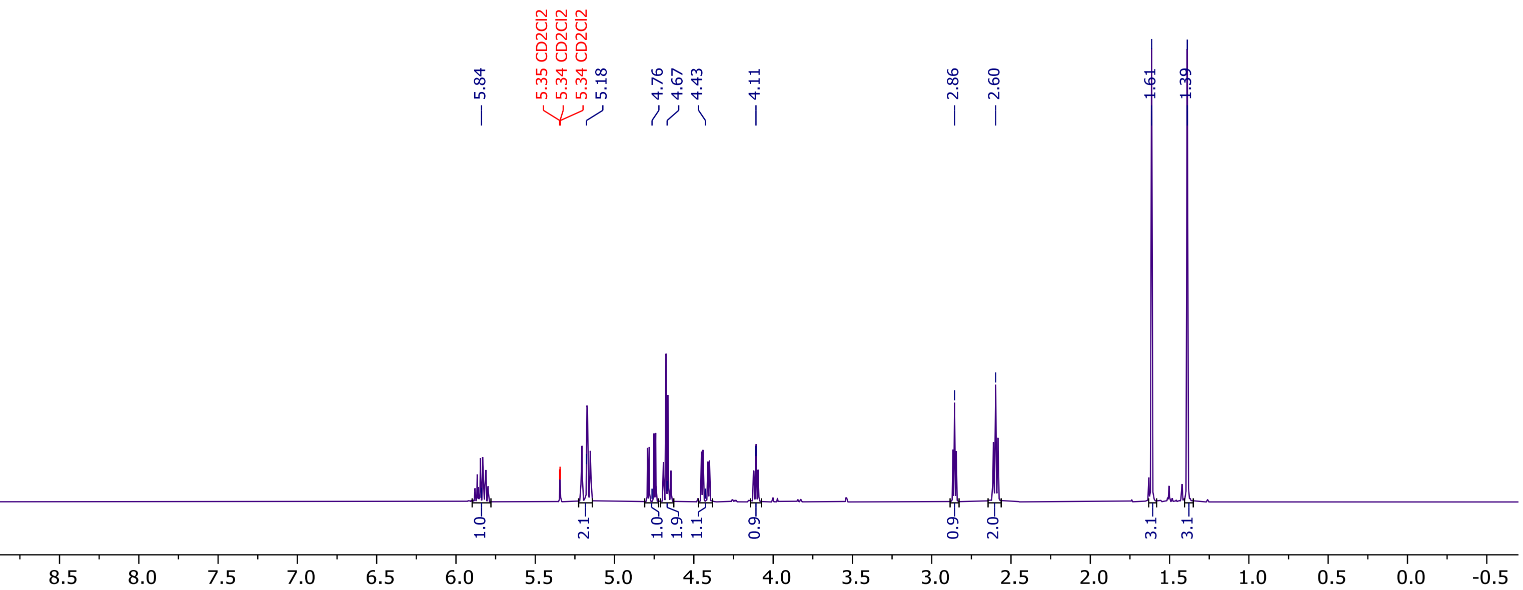
1H
CD2Cl2
500.14 MHz
5.84
5.35 CD2Cl2
5.34 CD2Cl2
5.34 CD2Cl2
5.18
4.76
4.67
4.43
4.11
2.86
2.60
1.61
1.39
1.0
2.1
1.0
1.9
1.1
0.9
0.9
2.0
3.1
3.1
10.0
9.5
9.0
8.5
8.0
7.5
7.0
6.5
6.0
5.5
5.0
4.5
4.0
3.5
3.0
2.5
2.0
1.5
1.0
0.5
0.0
-0.5

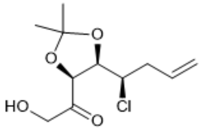
HO
O
O
O
Cl

**S60**

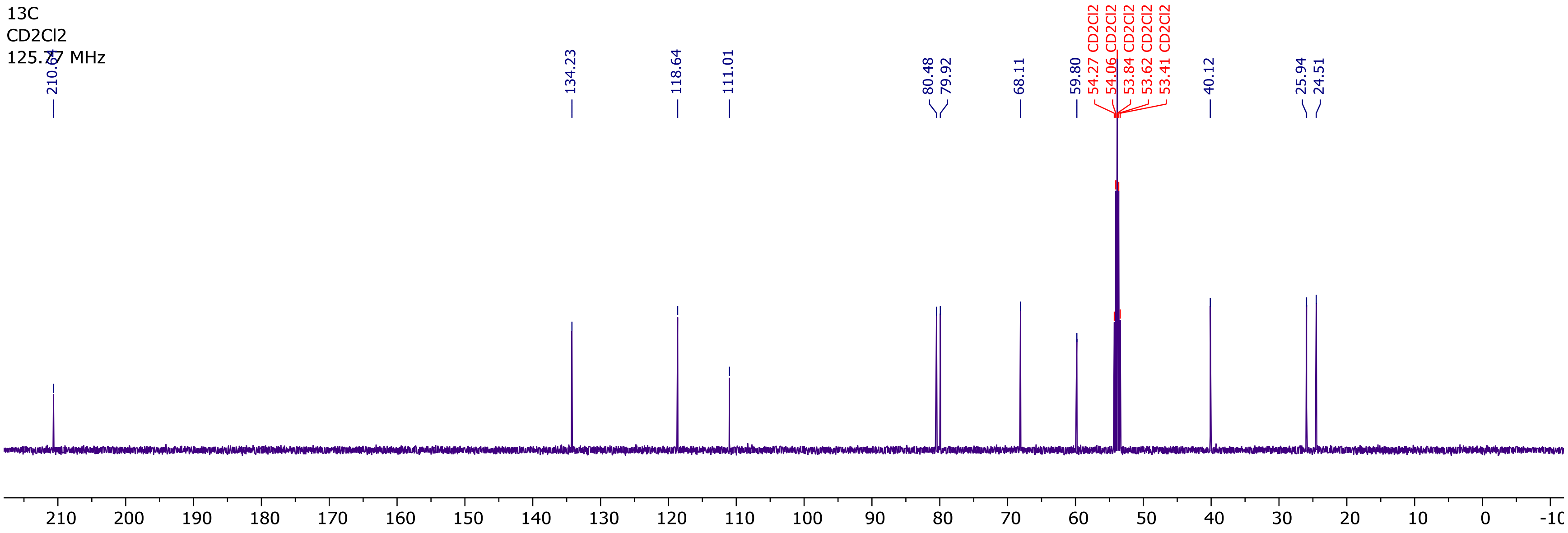
13C
CD2Cl2
125.77 MHz
210.67
134.23
118.64
111.01
80.48
79.92
68.11
59.80
54.27 CD2Cl2
54.06 CD2Cl2
53.84 CD2Cl2
53.62 CD2Cl2
53.41 CD2Cl2
40.12
25.94
24.51
210
200
190
180
170
160
150
140
130
120
110
100
90
80
70
60
50
40
30
20
10
0

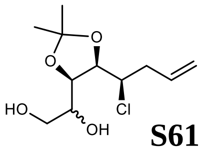


**S61**

Note: Reaction performed in capped vessel under ambient atmosphere.

To a 0 °C stirred solution of **S60** (572 mg, 2.30 mmol, 1.0 eq.) dissolved in MeOH (23 mL), was added sodium borohydride (191 mg, 5.05 mmol, 2.2 eq.) in one portion. After 15 minutes, the reaction was warmed to rt. The reaction mixture was stirred further for 10 minutes at rt, after which time starting material was consumed as monitored by TLC analysis. The reaction mixture was quenched with saturated aqueous $NH_4Cl$ (25 mL). The aqueous layer was extracted with $CH_2Cl_2$ (3x 40 mL). The combined organic layers were dried ($Na_2SO_4$), filtered, and solvent was removed in vacuo. The crude product was purified via flash column chromatography (13:7 hexanes/EtOAc). Appropriate fractions were pooled, and solvent was removed in vacuo to yield **S61** (3.91 g, 65%) as a colorless oil and a mixture of diastereomers.

**Analytical Data for S61:**

$R_f$ = 0.15 and 0.22 (3:2 hexanes/EtOAc)

$^{1}$H NMR (500 MHz, $CD_2Cl_2$) δ 6.00 – 5.86 (m, 1H), 5.25 – 5.14 (m, 2H), 4.39 – 4.26 (m, 2H), 4.25 – 4.07 (m, 1H), 4.00 – 3.90 (m, 1H), 3.74 – 3.63 (m, 2H), 2.74 – 2.66 (m, 1H), 2.66 – 2.55 (m, 1H), 2.55 – 2.46 (m, 1H), 1.58 (s, 2.4H), 1.52 (s, 0.6H), 1.41 (s, 2.4H), 1.38 (d, *J* = 2.3 Hz, 0.6H).

$^{13}$C NMR (126 MHz, $CD_2Cl_2$) δ 134.75, 133.79, 118.62, 118.12, 109.48, 109.11, 79.32, 79.23, 77.71, 77.02, 69.70, 69.54, 65.04, 64.89, 59.91, 59.80, 40.97, 40.31, 26.71, 26.55, 25.43, 25.12.

HRMS (ESI): Anal. Calcd. for $C_{11}H_{20}ClO_4^+$ $[M+H]^+$ 251.1045, found 251.1036

IR (neat): $\nu_{max}$ $(cm^{-1})$ = 3411 (br, OH), 2985 (w, CH), 2938 (w, CH), 1643 (w, C=C), 1376 (m), 1256 (m), 1215 (s)

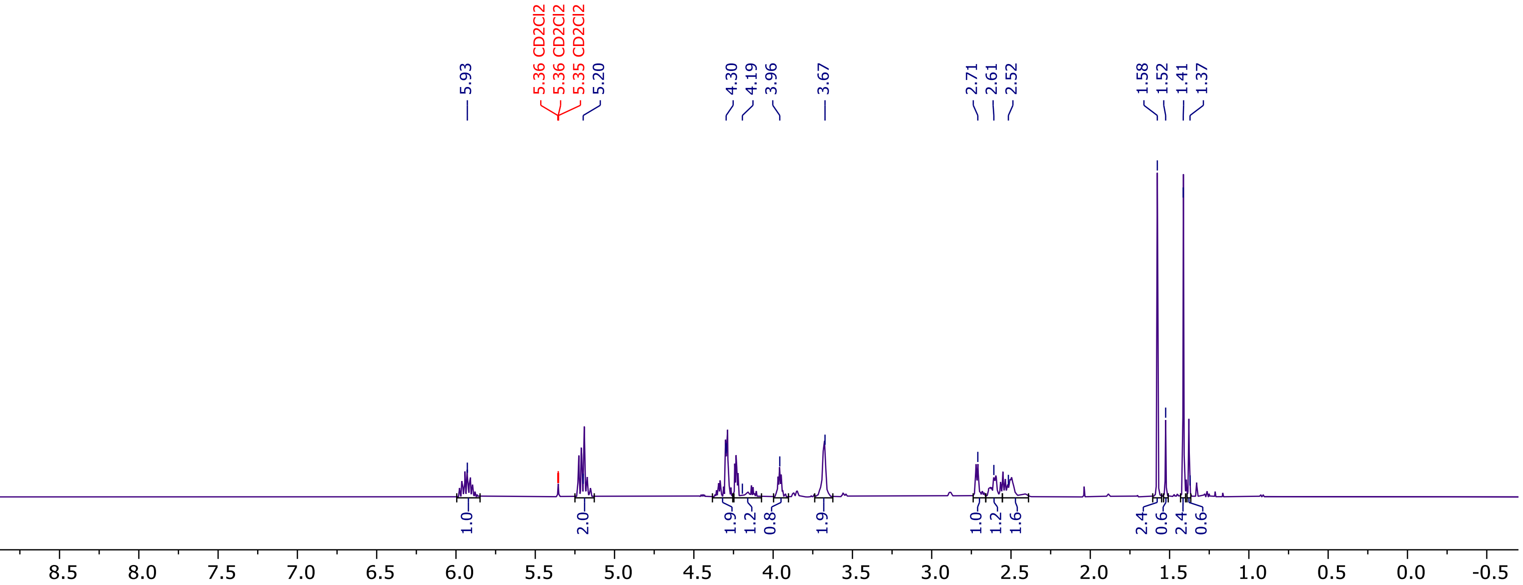

1H
CD2Cl2
500.14 MHz
5.93
5.36 CD2Cl2
5.36 CD2Cl2
5.35 CD2Cl2
5.20
4.30
4.19
3.96
3.67
2.71
2.61
2.52
1.58
1.52
1.41
1.37
1.0
2.0
1.9
1.2
0.8
1.9
1.0
1.2
1.6
2.4
0.6
2.4
0.6
10.0
9.5
9.0
8.5
8.0
7.5
7.0
6.5
6.0
5.5
5.0
4.5
4.0
3.5
3.0
2.5
2.0
1.5
1.0
0.5
0.0
-0.5


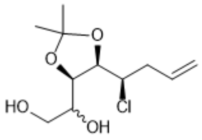

O
O
HO
OH
Cl


**S61**

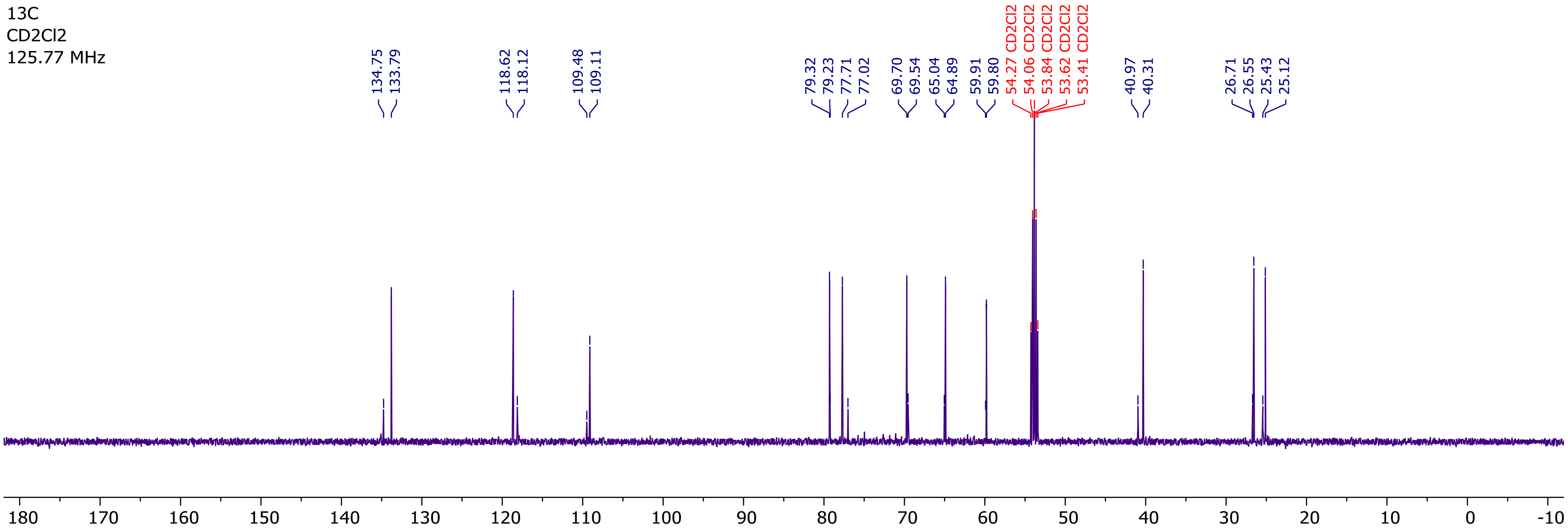

13C
CD2Cl2
125.77 MHz
134.75
133.79
118.62
118.12
109.48
109.11
79.32
79.23
77.71
77.02
69.70
69.54
65.04
64.89
59.91
59.80
54.27 CD2Cl2
54.06 CD2Cl2
53.84 CD2Cl2
53.62 CD2Cl2
53.41 CD2Cl2
40.97
40.31
26.71
26.55
25.43
25.12
180
170
160
150
140
130
120
110
100
90
80
70
60
50
40
30
20
10
0
-10

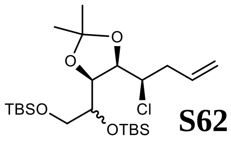


**S62**

To a -20 °C stirred solution of **S61** (532 mg, 2.12 mmol, 1.0 eq.) and 2,6-lutidine (1.23 mL, 1.14g, 10.6 mmol, 5.0 eq) dissolved in anhydrous $CH_2Cl_2$ (10.6 mL), was added Tert-butyldimethylsilyl trifluoromethanesulfonate (1.22 mL, 1.40 g, 5.30 mmol, 2.5 eq.). The reaction mixture was warmed to rt and stirred for 1 h, after which time starting material was consumed as monitored by TLC analysis. The reaction mixture was diluted with $CH_2Cl_2$ (10.6 mL) and quenched with saturated aqueous $NH_4Cl$ (21 mL). The aqueous layer was extracted with $CH_2Cl_2$ (3x 50 mL) and the combined organic layers dried ($Na_2SO_4$), filtered, and the solvent was removed in vacuo. The crude product was purified via flash column chromatography (17:3 hexanes/$Et_2O$). Appropriate fractions were pooled, and the solvent was removed in vacuo to yield **S62** (958 mg, 94%) as a colorless oil and a mixture of diastereomers.

**Analytical Data for S62:**

$R_f$ = 0.47 (19:1 hexanes/$Et_2O$)

$^1$H NMR (600 MHz, $CDCl_3$) δ 5.95 – 5.75 (m, 1H), 5.18 – 5.09 (m, 2H), 4.36 – 4.09 (m, 4H), 3.67 (dd, $J$ = 10.1, 4.8 Hz, 1H), 3.53 (dd, $J$ = 10.0, 7.7 Hz, 1H), 2.69 – 2.38 (m, 2H), 1.56 (s, 3H), 1.35 (d, $J$ = 3.3 Hz, 3H), 0.91 – 0.84 (m, 18H), 0.15 – 0.03 (m, 12H).

$^{13}$C NMR (151 MHz, $CDCl_3$) δ 134.45, 134.27, 118.26, 118.14, 109.21, 108.40, 79.75, 79.10, 78.22, 75.53, 71.93, 70.85, 66.45, 64.54, 60.21, 60.20, 59.57, 40.97, 40.56, 26.17, 26.12, 26.10, 26.07, 26.05, 25.84, 25.46, 25.41, 18.59, 18.55, 18.49, 18.33, -2.81, -3.22, -4.16, -4.19, -4.59, -5.21, -5.28, -5.31, -5.44.

HRMS (ESI): Anal. Calcd. for $C_{23}H_{48}ClO_4Si_2^+$ $[M+H]^+$ 479.2774, found 479.2756

IR (neat): $\nu_{max}$ $(cm^{-1})$ = 2954 (m, CH), 2931 (m, CH), 2858 (m, CH), 1467 (m), 1367 (m), 1255 (s), 1215 (m)

1H
CDCl3
600.13 MHz

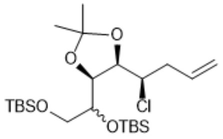


**S62**

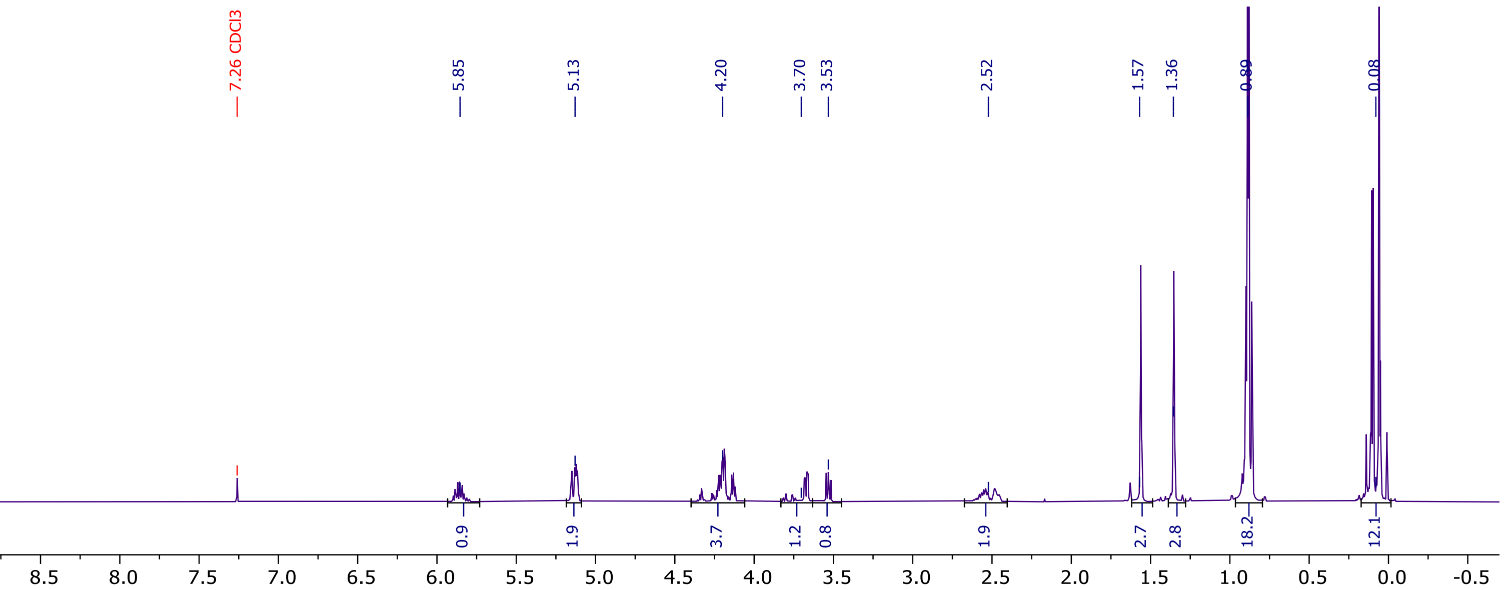


13C
CDCl3
150.92 MHz

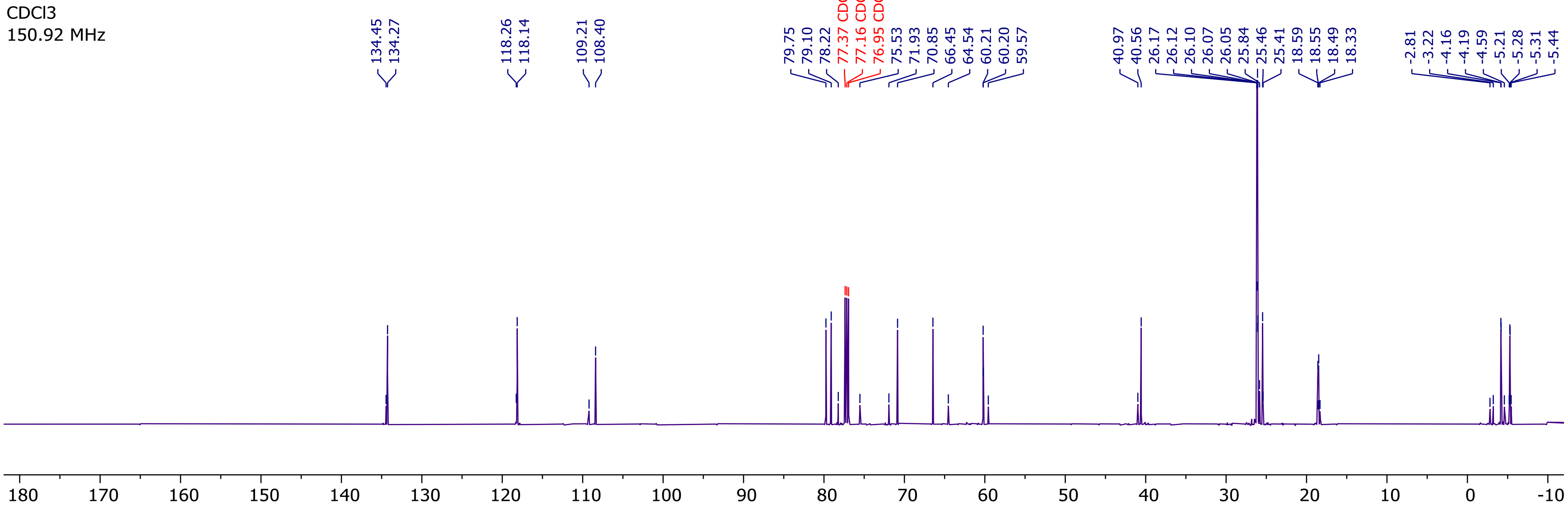

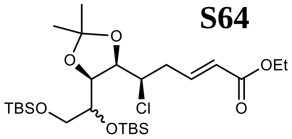

**S64**

Note: Reaction performed in capped vessel under ambient atmosphere.

To a vigorously stirred solution of **S62** (6.58 g, 13.7 mmol, 1.0 eq.) dissolved in 1:1 THF/$H_2O$ (275 mL) was added sodium periodate (7.33 g, 34.3 mmol, 2.5 eq.) followed by potassium osmate dihydrate (76 mg, 0.21 mmol, 0.015 eq.). The mixture was stirred overnight and the aqueous layer was extracted with $Et_2O$ (3x 100 mL). The combined organic layers were washed with brine (300 mL), dried ($MgSO_4$), and the solvent was removed in vacuo. The crude aldehyde **S63** was used immediately in the next step. To a stirred solution of diethyl phosphonoacetic acid ethyl ester (7.68 g, 34.3 mmol, 2.5 eq.) and) and LiCl (2.03 g, 48.0 mmol, 3.5 eq.), in anhydrous $CH_3CN$ (69 mL) was added DBU (5.12 mL, 5.23 g, 34.3 mmol, 2.5 eq.) followed by crude aldehyde **S63**. The reaction mixture was stirred for 30 minutes at rt, after which time starting material was consumed as monitored by TLC analysis. The reaction was quenched with a saturated solution of $NH_4Cl$ (70 mL) and the aqueous layer was extracted with $CH_2Cl_2$ (3x 100 mL). The combined organic layers were washed with brine (150 mL), dried ($Na_2SO_4$) and the solvent was removed in vacuo. The crude product was run through a plug of silica gel (3:1 hexanes/$Et_2O$) yielding crude ester **S64** which was used immediately in the next step.

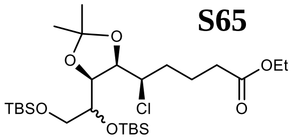

**S65**

To a rt round bottom flask containing platinum (IV) oxide (114 mg, 0.5 mmol, 0.05 eq.) under $H_2$ atmosphere was added **S64** (5.54 g, 10.0 mmol, 1.0 eq) in EtOAc (100 mL) via syringe. The reaction was purged with $H_2$ and kept under $H_2$ for the duration of the reaction using a hydrogen balloon. After 1 day, the reaction mixture was rinsed through a pad of celite using EtOAc, then the solvent was removed in vacuo. The crude ester **S65** was used immediately for the next step.

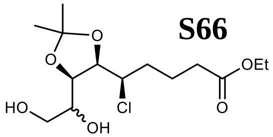

**S66**

To a plastic vessel containing crude ester **S65** (5.00 g, 9.0 mmol, 1.0 eq.) dissolved in anhydrous THF (30 mL) was added 70% HF in pyridine (10 mL, 11.0 g, 385 mmol HF). After 1 day stirring at rt, the reaction was quenched with saturated $NaHCO_3$ (450 mL) and after effervescence had subsided, the aqueous layer extracted with $CH_2Cl_2$ (4x 100 mL). The combined organic layers were dried ($Na_2SO_4$), filtered, and the solvent was removed in vacuo. The crude product was purified via flash column chromatography (3:1 hexanes/acetone). Appropriate fractions were pooled to yield diol **S66** as a clear oil. The diol **S66** was used immediately for the next step.

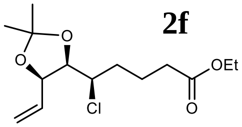

**2f**

To a cold (0 °C) stirred solution of iodine (1.01 g, 3.98 mmol, 3.0 eq.) dissolved in anhydrous $CH_2Cl_2$ (5 mL) was added triphenylphosphine (1.39 g, 5.31 mmol, 4.0 eq.) and imidazole (361 mg, 5.31 mmol, 4.0 eq.). **S66** (430.9 mg, 1.33 mmol, 1.0 eq) was added in $CH_2Cl_2$ (1.7 mL) and the mixture was stirred at rt for 1.5 h after which the starting material was consumed by TLC analysis. The reaction mixture was quenched with 1:1 saturated aqueous $NaHCO_3$/saturated aqueous $Na_2S_2O_3$ (10 mL). The aqueous layer was extracted with $CH_2Cl_2$ (3x 20mL) and the combined organic layers were dried ($Na_2SO_4$), filtered, and the solvent was removed in vacuo. The crude product was purified via flash column chromatography (39:1 hexanes/acetone). Appropriate fractions were pooled to yield **2f** as a clear oil. The material was used immediately for the next step.

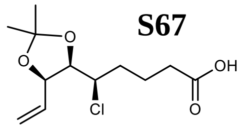


**S67**

To a rt stirred solution of **2f** (78 mg, 0.27 mmol, 1.0 eq.) dissolved in 2:1 THF/$H_2O$ (1.0 mL) was added 1 M aqueous LiOH (0.85 mL, 3.1 eq.). After 2 hours, the reaction was quenched with 1 M HCl (0.85 mL) careful to avoid acidifying the mixture under pH 4. The aqueous layer was extracted with $Et_2O$ (3x 20 mL) and the combined organic layers were dried ($MgSO_4$), filtered, and the solvent was concentrated in vacuo, to yield **S67** (69 mg, 2% yield over 6 steps) as a yellow oil.

**Analytical Data for carboxylic acid S67:**

$R_f$ = 0.18 (3:2 hexanes/$Et_2O$)

$[\alpha]_D^{20}$ = -1.4 ° (c = 0.5, $CH_2Cl_2$)

$^1$H NMR (600 MHz, $CDCl_3$) δ 6.00 (ddd, *J* = 17.1, 10.2, 8.6 Hz, 1H), 5.38 – 5.29 (m, 2H), 4.57 (dd, *J* = 8.6, 6.3 Hz, 1H), 4.21 (t, *J* = 6.4 Hz, 1H), 3.81 (ddd, *J* = 9.6, 6.4, 3.3 Hz, 1H), 2.44 – 2.32 (m, 2H), 2.00 – 1.91 (m, 1H), 1.84 – 1.68 (m, 3H), 1.56 (s, 3H), 1.40 (s, 3H).

$^{13}$C NMR (151 MHz, $CDCl_3$) δ 179.01, 133.23, 120.68, 109.44, 80.99, 79.69, 60.31, 33.88, 33.10, 27.42, 25.58, 21.40.

HRMS (ESI): Anal. Calcd. for $C_{12}H_{20}ClO_4^+$ $[M+H]^+$ 263.1045, found 263.1032

IR (neat): $\nu_{max}$ $(cm^{-1})$ = 2987 (m, CH), 2937 (m, CH), 1709 (s, C=O), 1639 (w, C=C), 1458 (m), 1429 (m), 1377 (m), 1254 (s), 1217 (s)

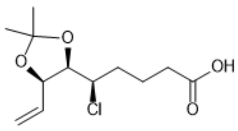
OH
O
O
O
Cl
O

**S67**

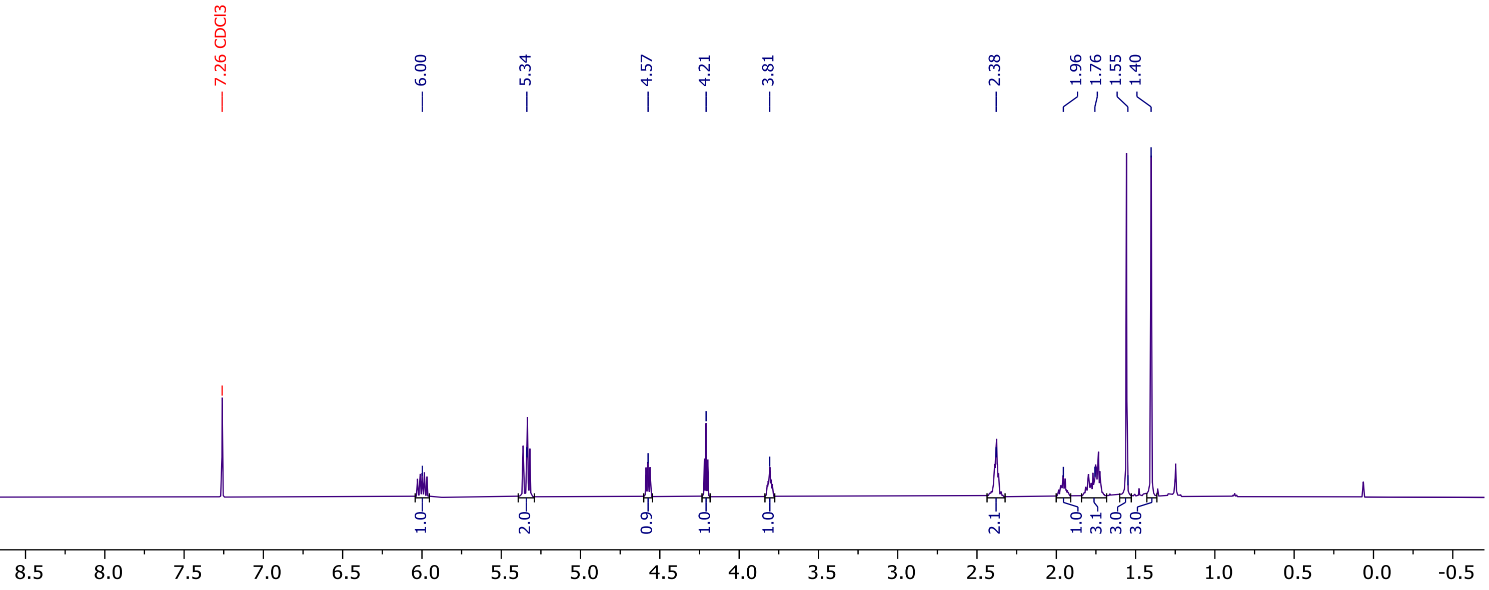
1H
CDCl3
600.13 MHz
7.26 CDCl3
6.00
5.34
4.57
4.21
3.81
2.38
1.96
1.76
1.55
1.40
1.0
2.0
0.9
1.0
1.0
2.1
1.0
3.1
3.0
3.0
10.0 9.5 9.0 8.5 8.0 7.5 7.0 6.5 6.0 5.5 5.0 4.5 4.0 3.5 3.0 2.5 2.0 1.5 1.0 0.5 0.0 -0.5

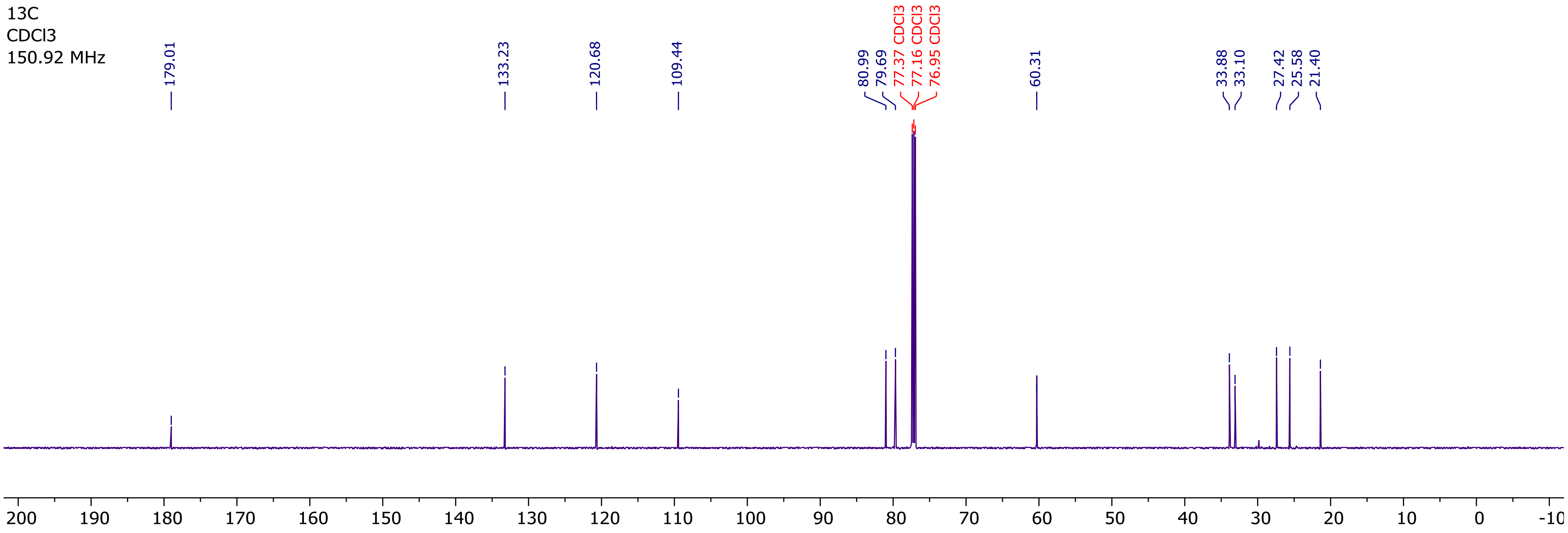
13C
CDCl3
150.92 MHz
179.01
133.23
120.68
109.44
80.99
79.69
77.37 CDCl3
77.16 CDCl3
76.95 CDCl3
60.31
33.88
33.10
27.42
25.58
21.40
200 190 180 170 160 150 140 130 120 110 100 90 80 70 60 50 40 30 20 10 0 -10

## Procedures for the Synthesis and Characterization of Vinyl Iodide Building Blocks

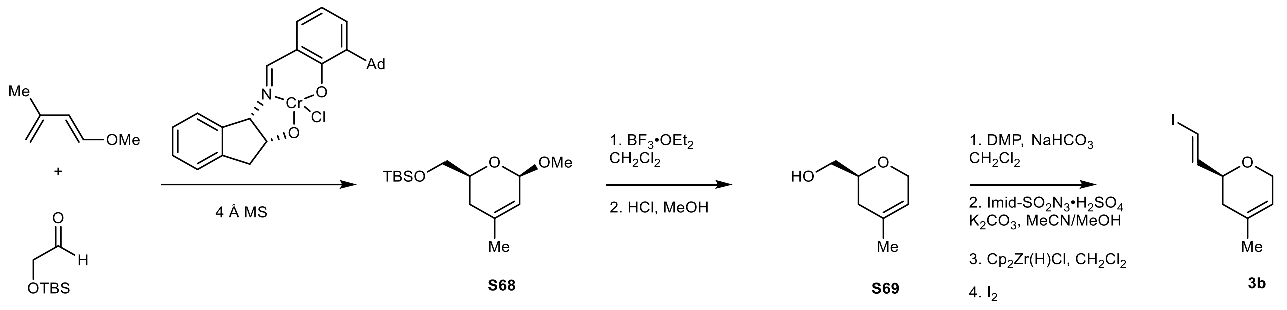


**Supplementary Fig. 38 | Synthesis of Dihydropyran Vinyl Iodide 3b.**
Abbreviations: TBS = tert-butyldimethylsilyl, Ad = adamantyl, 4 Å MS = 4 Ångstrom molecular sieves, DMP = Dess-Martin periodinane, Imid-$SO_2N_3$•$H_2SO_4$ = imidazole sulfonylazide hydrogen sulfate.

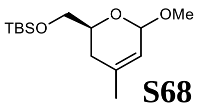


**S68**

Procedure was adapted from the literature.[19] To neat 2-((tert-butyldimethylsilyl)oxy)acetaldehyde (3.90 g, 22.4 mmol, 1.0 eq.) was added a magnetic stirring bar, freshly activated powdered 4Å molecular sieves (2.25 g), and hetero diels-alder catalyst Chromium, chloro[(1*S*,2*R*)-2,3-dihydro-1-[[[2-(hydroxy-κO)-5-methyl-3-tricyclo[3.3.1.13,7dec-1-ylphenyl]methyl-ene]amino-κN]-1H-indene-2-olato-(2-)-κO],(SP-4-4) (327.2 mg, 0.672 mmol, 3 mol%, prepared according to the literature procedure[20]). Subsequently, the dark brown reaction suspension was cooled to 0 °C and 1-methoxy-3-methylbuta-1,3-diene (prepared according to literature procedure[21]) was added via syringe (4.0 mL, 3.3 g, 34 mmol, 1.5 eq.). The cold bath was removed, and the reaction mixture was protected from light by wrapping the reaction vessel in aluminum foil. The reaction mixture was vigorously stirred for 24 h. After this time, aldehyde starting material was consumed as monitored by TLC analysis. The reaction mixture was diluted with $Et_2O$ (10 mL) and passaged through a short plug of silica gel eluting with further $Et_2O$. The solvent was removed in vacuo and the crude product was purified via flash column chromatography (19:1 Hexanes/EtOAc). Appropriate fractions were pooled, and solvent was removed in vacuo to yield **S68** (4.05 g, 66%) of a pale-yellow sweet-smelling liquid.

**Analytical Data for S68:**

$R_f$ = 0.60 (9:1 Hexanes/$Et_2O$)

$[\alpha]_D^{20}$ = -57.9° (c = 1.98, $CH_2Cl_2$)

$^1$H NMR (600 MHz, $CDCl_3$) δ 5.37 (p, *J* = 1.5 Hz, 1H), 4.97 (tt, *J* = 2.2, 1.3 Hz, 1H), 3.81 (dddd, *J* = 8.0, 6.4, 5.6, 4.5 Hz, 1H), 3.75 (dd, *J* = 10.3, 5.6 Hz, 1H), 3.64 (dd, *J* = 10.3, 6.5 Hz, 1H), 3.46 (s, 3H), 2.00 (ddtd, *J* = 17.3, 8.2, 2.3, 1.2 Hz, 1H), 1.97 – 1.91 (m, 1H), 1.73 (td, *J* = 1.6, 0.8 Hz, 3H), 0.90 (s, 9H), 0.07 (s, 6H).

$^{13}$C NMR (151 MHz, $CDCl_3$) δ 137.02, 120.94, 98.20, 72.63, 65.68, 55.37, 31.72, 26.04, 23.05, 18.49, -5.09, -5.19.

HRMS (ESI): Anal. Calcd. for $C_{13}H_{25}O_2Si^+$ $[M-CH_3O]^+$ 241.1618, found 241.1641

IR (neat): $\nu_{max}$ $(cm^{-1})$ = 2956 (m, CH), 2930 (m, CH), 2858 (m, CH), 1634 (w, C=C), 1469 (m) 1384 (m), 1362 (m), 1253 (s).

1H
CDCl3
600.13 MHz

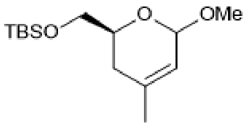


**S68**

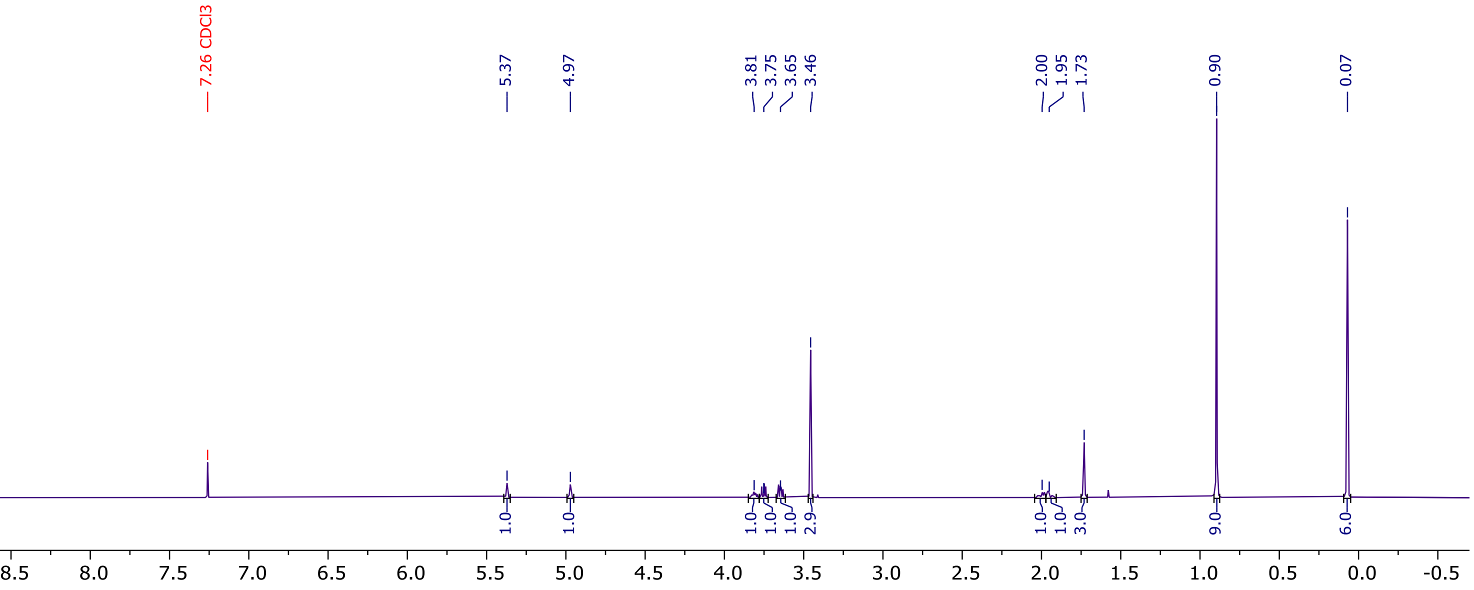


13C
CDCl3
150.92 MHz

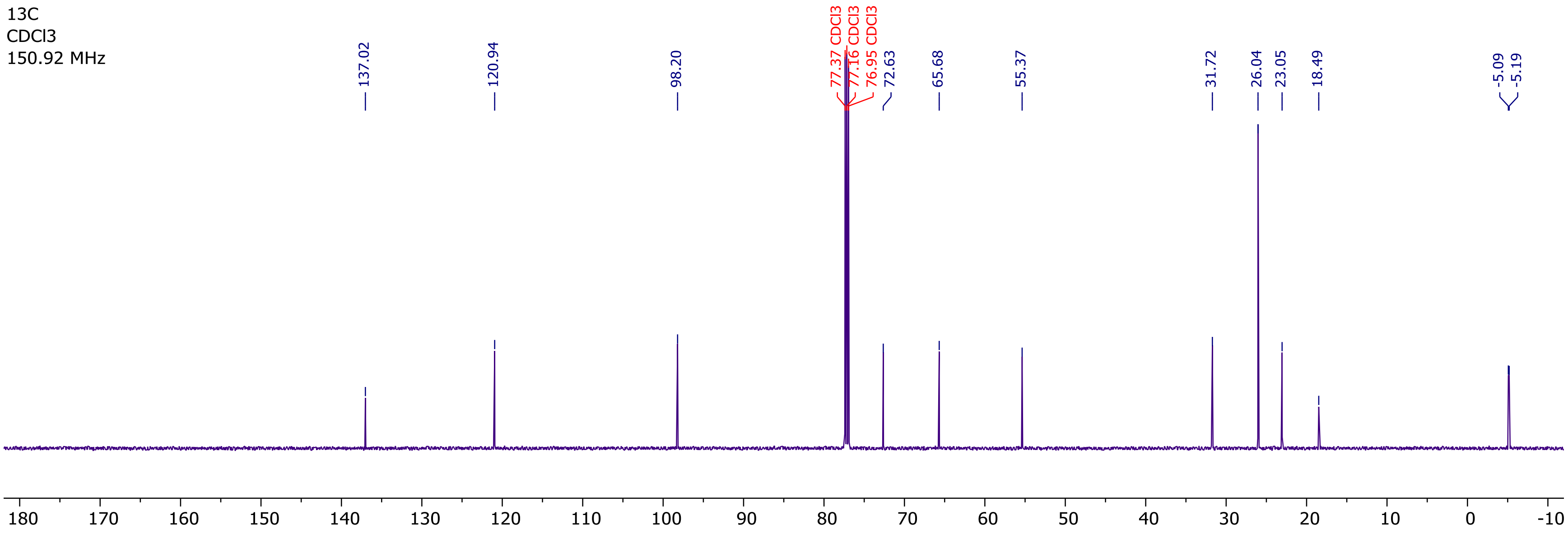

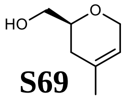

**S69**

Procedure was adapted from the literature.[19] To a cold (-78 °C), stirred solution of **S68** (4.05 g, 14.9 mmol, 1.0 eq.) and $Et_3SiH$ (3.6 mL, 2.6 g, 23 mmol, 1.5 eq.) in $CH_2Cl_2$ (50 mL) was added $BF_3$•$OEt_2$ (2.1 mL, 2.4 g, 17 mmol, 1.1 eq.) dropwise via syringe over 5 min. The reaction was maintained at -78 °C for 1 h 15 min. After this time, starting material was consumed as monitored by TLC analysis. The reaction mixture was quenched by addition of saturated aqueous $NaHCO_3$ (25 mL) and the cold bath was removed. After warming to rt, the reaction mixture was transferred to a separatory funnel, the organic layer was separated, and the aqueous layer extracted with $CH_2Cl_2$ (3x 30 mL). The organic layers were combined, and solvent was removed in vacuo. Concentrated aqueous HCl (36.5% (w/w) HCl in $H_2O$, assumed to be *ca.* 12M, 0.37 mL, *ca.* 4.44 mmol) was diluted to 10 mL with MeOH to make an approximately 0.44 M stock solution of aqueous HCl in MeOH. The crude product from the previous step was redissolved in $CH_2Cl_2$ (40 mL), a magnetic stirring bar was added, and the reaction mixture was cooled to 0 °C. The previously prepared solution of the aqueous HCl in MeOH (10 mL, 0.44 M, 4.4 mmol, 0.30 eq.) was added in via syringe. The reaction mixture was maintained at 0 °C for 1 h 25 min. After this time, starting material was consumed by TLC analysis, and the reaction mixture was quenched by addition of solid $NaHCO_3$ (815 mg, 9.7 mmol, excess). After evolution of gas had stopped, the reaction mixture was filtered through a cotton plug and solvent was removed in vacuo. The crude product was purified via flash column chromatography (1:1 Pentane/$Et_2O$). Appropriate fractions were pooled, and solvent was removed in vacuo to yield **S69** (1.50 g, 79%, 2 steps) as a pale-yellow oil. Spectral and optical rotation data agreed with the data previously reported in the literature.[22]

**Analytical Data for S69:**

$R_f$ = 0.28 (2:3 Hexanes/$Et_2O$)

$[\alpha]_D^{20}$ = -115° (c = 1.76, $CDCl_3$); lit. $[\alpha]_D^{23}$ = -121° (c = 0.24, $CHCl_3$)[22]

$^1H$ NMR (600 MHz, $CDCl_3$) δ 5.42 (dt, *J* = 3.6, 1.9 Hz, 1H), 4.22 – 4.11 (m, 2H), 3.66 (dd, *J* = 11.1, 2.8 Hz, 1H), 3.63 (ddt, *J* = 10.2, 6.4, 3.1 Hz, 1H), 3.56 (dd, *J* = 11.1, 6.9 Hz, 1H), 2.30 (s, 1H), 2.06 – 1.97 (m, 1H), 1.73 (dt, *J* = 17.0, 3.1 Hz, 1H), 1.70 (s, 3H).

$^{13}C$ NMR (151 MHz, $CDCl_3$) δ 131.42, 119.75, 74.33, 65.86, 65.77, 31.31, 23.15.

HRMS (ESI): Anal. Calcd. for $C_7H_{13}O_2^+$ $[M+H]^+$ 129.0910, found 129.0906

IR (neat): $\nu_{max}$ $(cm^{-1})$ = 3408 (br, OH), 3026 (m, C=*CH*), 2913 (m, CH), 2826 (m, CH), 1681 (w, C=C), 1446 (m), 1382 (m), 1131 (s), 1065 (s).

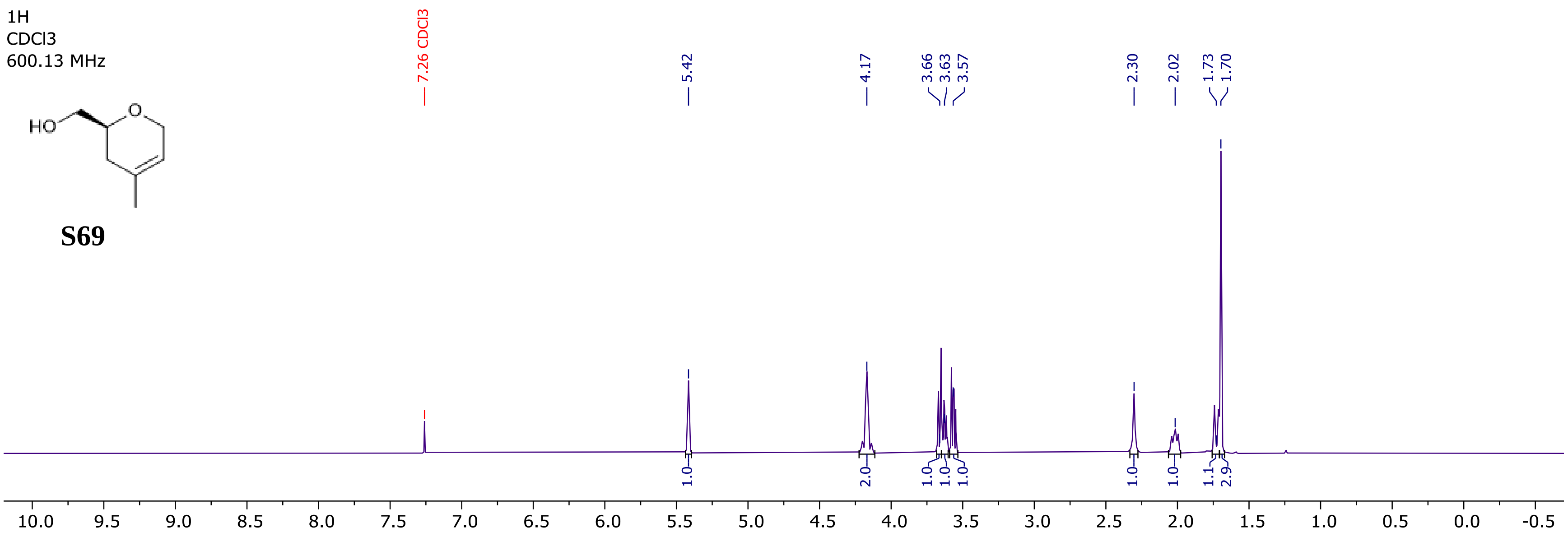
1H
CDCl3
600.13 MHz
HO
O
7.26 CDCl3
5.42
4.17
3.66
3.63
3.57
2.30
2.02
1.73
1.70
1.0
2.0
1.0
1.0
1.0
1.0
1.0
1.1
2.9
10.0 9.5 9.0 8.5 8.0 7.5 7.0 6.5 6.0 5.5 5.0 4.5 4.0 3.5 3.0 2.5 2.0 1.5 1.0 0.5 0.0 -0.5

**S69**

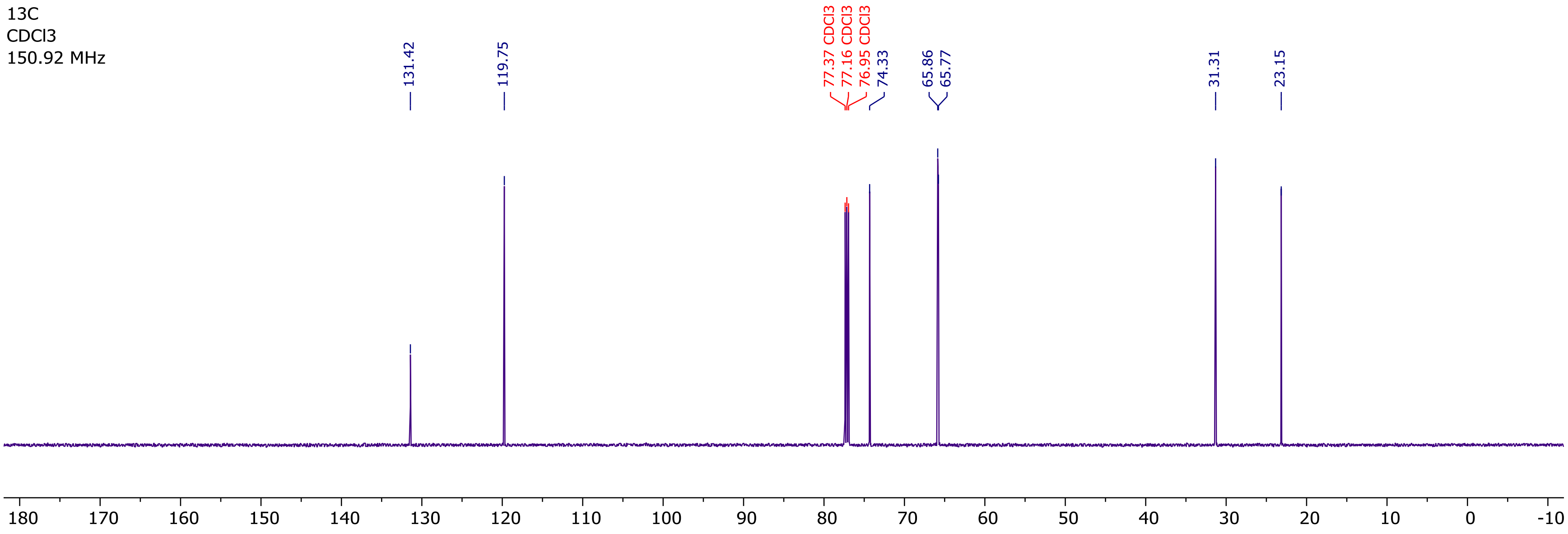
13C
CDCl3
150.92 MHz
131.42
119.75
77.37 CDCl3
77.16 CDCl3
76.95 CDCl3
74.33
65.86
65.77
31.31
23.15
180 170 160 150 140 130 120 110 100 90 80 70 60 50 40 30 20 10 0 -10

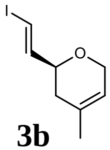

**3b**

To a cold (0 °C), stirred solution of **S69** (640 mg, 5.0 mmol, 1 eq.) was added in sequence $NaHCO_3$ (1.26 g, 15 mmol, 3.0 eq.) and Dess-Martin periodinane (2.55 g, 6 mmol, 1.2 eq.). The reaction mixture was allowed to slowly warm to rt over the course of 4 h. After this time, starting material was consumed as monitored by TLC analysis. The reaction mixture was quenched by addition of saturated aqueous $NaHCO_3$ (25 mL) and 10% aqueous $Na_2S_2O_3$ (25 mL), and vigorously stirred for a further 1 h. After this time, the reaction mixture was transferred to a separatory funnel, the organic layer separated, and the aqueous layer extracted with $CH_2Cl_2$ (3x 35 mL). The combined organic layers were dried ($Na_2SO_4$), filtered, and solvent was carefully (no lower than 400 mmHg for evaporation) concentrated in vacuo to a small volume (*ca.* 2 mL). This concentrate containing the crude aldehyde (631 mg, *ca.* 5 mmol) was used directly in the next step without further purification. The quantity of aldehyde was estimated in the solution using $^1$H NMR analysis of an aliquot and the weight of the solution.

**Danger!** The sulfonyl azide salt is known to pose an explosion hazard and is sensitive to friction and electrostatic discharge. Plastic utensils were used for all manipulations of the sulfonyl azide salt.[23]

Procedure was adapted from the literature.[24] To a rt, stirred suspension of $K_2CO_3$ (3.28, 23.8 mmol, 4.76 eq.) and Dimethyl-2-(oxopropyl)phosphonate (0.83 mL, 1.00 g, 6.0 mmol, 1.20 eq.) in MeCN (30 mL) was added imidazole sulfonyl azide hydrogen sulfate[23] (1.63 g, 6.00 mmol, 1.20 eq.) in one portion, and the suspension was stirred for 5 h. After this time, the supernatant became a deep yellow color, and the crude aldehyde (631, *ca.* 5 mmol, 1.0 eq.) concentrated from the previous step was added via syringe in MeOH (30 mL). The reaction was stirred at rt for 16 h. After this time, the reaction mixture was poured into a separatory funnel and extracted with pentane (5x 30 mL). The combined pentane extracts were dried ($MgSO_4$) and filtered. The solvent was very carefully concentrated in vacuo (no lower than 400 mmHg for evaporation) to a small volume (*ca.* 2 mL). This concentrated pentane solution containing the crude alkyne (*ca.* 280 mg, 2.29 mmol) was used directly in the next step without further purification. The quantity of alkyne was estimated in the solution using $^1$H NMR analysis of an aliquot and the weight of the solution.

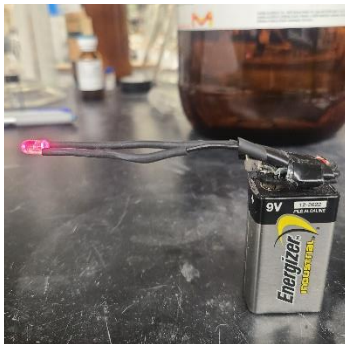


**Danger!** The vinyl iodide should be handled with extreme caution and exclusively in a fume hood. Manipulation of TLC plates outside of the hood spotted with reaction solutions or column fractions cause severe irritation of mucus membranes and labored breathing. Dilute solutions or TLC plates spotted with the vinyl iodide have a distinct overwhelming acrid smell.

Procedure was adapted from the literature.[19] All manipulations were performed in total darkness using a single red-LED light rigged to a 9V battery as the sole light source (**see left**). Attempting to manipulate the vinyl iodide in open light results in rapid photochemical degradation. To a cold (0 °C), stirred

suspension of $Cp_2Zr(H)Cl$ (681 mg, 2.64 mmol, 1.15 eq.) in $CH_2Cl_2$ (17 mL) was added the pentane solution containing the crude alkyne (*ca.* 280 mg, 2.29 mmol, 1.0 eq.). Immediately following addition of the alkyne, the suspension becomes a homogenous yellow-orange solution. After stirring for a further 20 min at 0 °C, starting material is consumed as monitored by TLC analysis. The previously prepared solution of $I_2$ (9.5 mL, 0.169 M in $CH_2Cl_2$, 1.60 mmol) in $CH_2Cl_2$ was then added to the reaction mixture via syringe just until a dark brown color persists in solution. The reaction was stirred for a further 5 min. to ensure the dark brown color persisted in solution. After this time, the reaction mixture was poured onto 10% aqueous $Na_2S_2O_3$ (30 mL). The organic layer was separated, and the aqueous layer was extracted with $CH_2Cl_2$ (2x 30 mL). The combined organic layers were dried ($Na_2SO_4$), filtered, and solvent was removed in vacuo. The crude product was purified by flash column chromatography (499:1 to 99:1 to 49:1 Hexanes/$Et_2O$). Appropriate fractions were pooled, and solvent was removed in vacuo to yield **3b** (373 mg, 30%, 3 steps) as a very pale-yellow oil that turns green/black within minutes of exposure to white light. The vinyl iodide is stored in foil wrapped and nitrogen purged vials at -20°C to avoid degradation. Under these conditions the compound is stable for approximately 6 months. Spectral and optical rotation data agreed with the data previously reported in the literature.[25]

**Analytical Data for 3b:**

$R_f$ = 0.60 (19:1 Hexanes/$Et_2O$)

$[\alpha]_D^{20}$ = -130° (c = 0.85, $CDCl_3$); lit. $[\alpha]_D$ = -106° (c = 0.76, $CHCl_3$)[25]

$^1$H NMR (600 MHz, $CDCl_3$) δ 6.63 (dd, *J* = 14.6, 5.6 Hz, 1H), 6.42 (dd, *J* = 14.5, 1.4 Hz, 1H), 5.41 (dt, *J* = 3.4, 1.9 Hz, 1H), 4.25 – 4.12 (m, 2H), 3.99 (dddd, *J* = 9.1, 5.3, 3.6, 1.4 Hz, 1H), 2.12 – 2.03 (m, 1H), 1.92 (dt, *J* = 16.9, 3.4 Hz, 1H), 1.70 (t, *J* = 1.9 Hz, 3H).

$^{13}$C NMR (151 MHz, $CDCl_3$) δ 145.99, 131.08, 119.77, 78.05, 75.57, 65.70, 35.03, 23.04.

IR (neat): $\nu_{max}\ (cm^{-1})$ = 3023 (w, C=*CH*), 2964 (m, CH), 2927 (m, CH), 2901 (m, CH), 2824 (m, CH), 1611 (m, C=C), 1446 (m), 1373 (m).

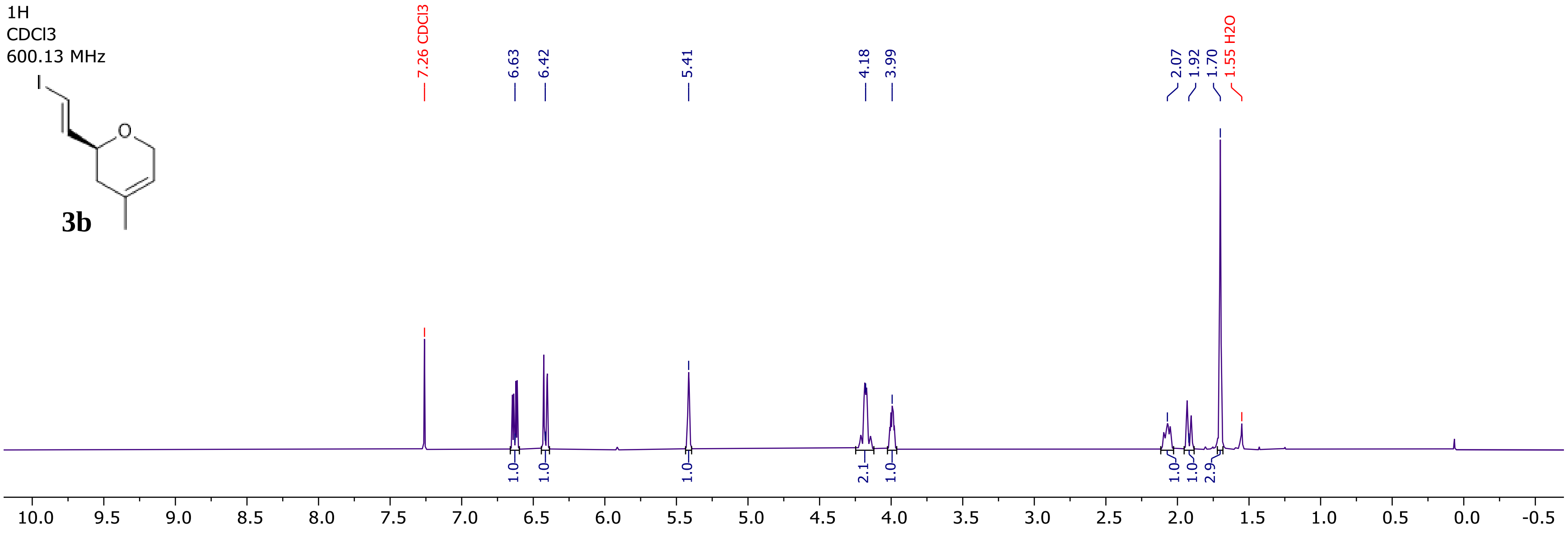

1H
CDCl3
600.13 MHz
I
O
3b
7.26 CDCl3
6.63
6.42
5.41
4.18
3.99
2.07
1.92
1.70
1.55 H2O
1.0
1.0
1.0
2.1
1.0
1.0
1.0
2.9
10.0 9.5 9.0 8.5 8.0 7.5 7.0 6.5 6.0 5.5 5.0 4.5 4.0 3.5 3.0 2.5 2.0 1.5 1.0 0.5 0.0 -0.5


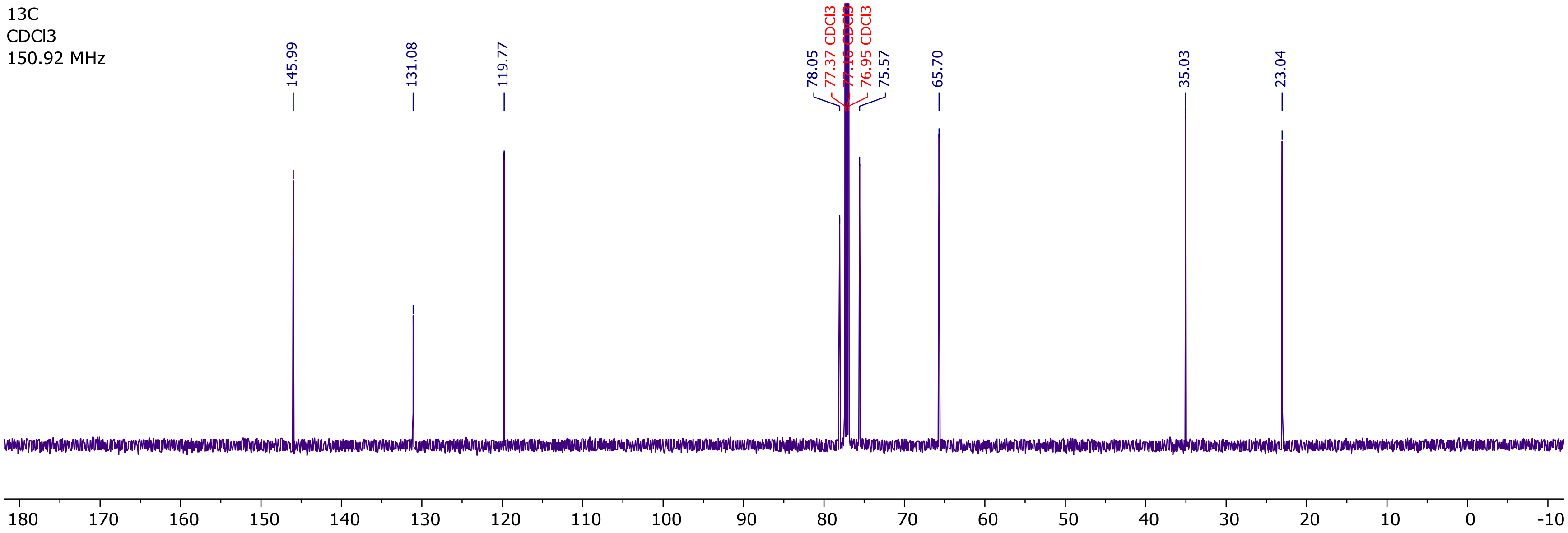

13C
CDCl3
150.92 MHz
145.99
131.08
119.77
78.05
77.37 CDCl3
77.16 CDCl3
76.95 CDCl3
75.57
65.70
35.03
23.04
180 170 160 150 140 130 120 110 100 90 80 70 60 50 40 30 20 10 0 -10

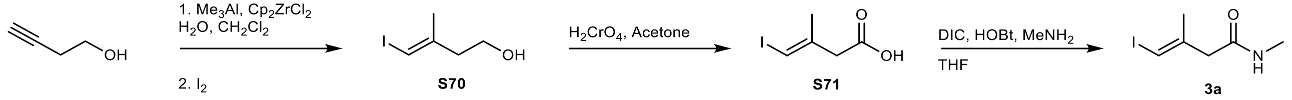


**Supplementary Fig. 39 | Synthesis of Methylamide Vinyl Iodide 3a.**
Abbreviations: DIC = N,N'-diisopropylcarbodiimide, HOBt = hydroxybenzotriazole, THF = tetrahydrofuran.

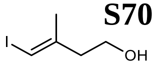

**S70** To a cold (-20 °C) stirred solution of $ZrCp_2Cl_2$ (918 mg, 3.14 mmol, 0.22 eq.) was added $AlMe_3$ (22 mL, 2M in hexanes, 44 mmol, 3.1 eq.). The reaction mixture was maintained at -20 °C and $H_2O$ (0.40 mL, 400 mg, 22.2 mmol, 1.56 eq.) [**caution!** reaction is extremely exothermic and liberates gas] was added one drop at a time waiting for gas evolution to stop between drops. The reaction mixture was maintained at -20 °C for 20 min. In a separate vessel containing a cold (0 °C), stirred solution of butyn-1-ol (1.00 g, 14.2 mmol, 1.0 eq.) in $CH_2Cl_2$ (11 mL) was added $AlMe_3$ (2.2 mL, 2M in hexanes, 4.4 mmol, 0.31 eq.). The solution of butyn-1-ol and $AlMe_3$ in $CH_2Cl_2$ (initial volume and 3x 2mL rinse) was then cannulated into the $ZrCp_2Cl_2$ and $AlMe_3$ solution over 10 min. while maintaining external cooling (-20 °C) of the receiving vessel. After completion of the addition, the cold bath was removed, and the reaction mixture was allowed to warm to rt and stirred for 17 h 25 min. After this time the reaction vessel was cooled to -20 °C, and a solution of $I_2$ (5.12 g) in $Et_2O$ (46 mL) was added via syringe to the reaction mixture. The reaction mixture was stirred for 10 min. to ensure a dark brown color persisted in solution. After this time, the reaction mixture was then quenched by addition of a saturated aqueous solution of Rochelle's salt (115 mL) and was vigorously stirred for 15 h while slowly warming to rt. After this time, the entire reaction mixture was filtered through celite, and the celite pad rinsed with $Et_2O$ (1x 100 mL). The biphasic mixture was transferred to a separatory funnel, the organic layer separated, and the aqueous layer extracted with $Et_2O$ (3x 60 mL). The combined organic layers were washed in sequence with a 10% aqueous solution of $Na_2S_2O_3$ (2x 45 mL), $H_2O$ (1x 50 mL), and brine (1x 50 mL). The organic layers were dried ($Na_2SO_4$), filtered, and solvent was removed in vacuo. The crude product was purified via flash column chromatography (9:1 to 4:1 hexanes/EtOAc) to yield **S70** (2.42, 80%) as a yellow oil. Spectral data agreed with the data previously reported in the literature.[26]

**Analytical Data for S70:**

$R_f$ = 0.28 (3:1 hexanes/EtOAc)

$^1H$ NMR (400 MHz, $CDCl_3$) δ 6.02 (q, *J* = 1.1 Hz, 1H), 3.72 (t, *J* = 6.3 Hz, 2H), 2.48 (td, *J* = 6.3, 1.1 Hz, 2H), 1.88 (d, *J* = 1.1 Hz, 3H), 1.43 (s, 1H).

$^{13}$C NMR (101 MHz, $CDCl_3$) δ 144.71, 77.00, 60.30, 42.60, 23.96.
IR (neat): $\nu_{max}$ $(cm^{-1})$ = 3322 (br, OH), 3086 (w, C=*CH*), 2940 (m, CH), 2909 (m, CH). 1617 (w, C=C), 1433 (m), 1378 (m), 1273 (s)

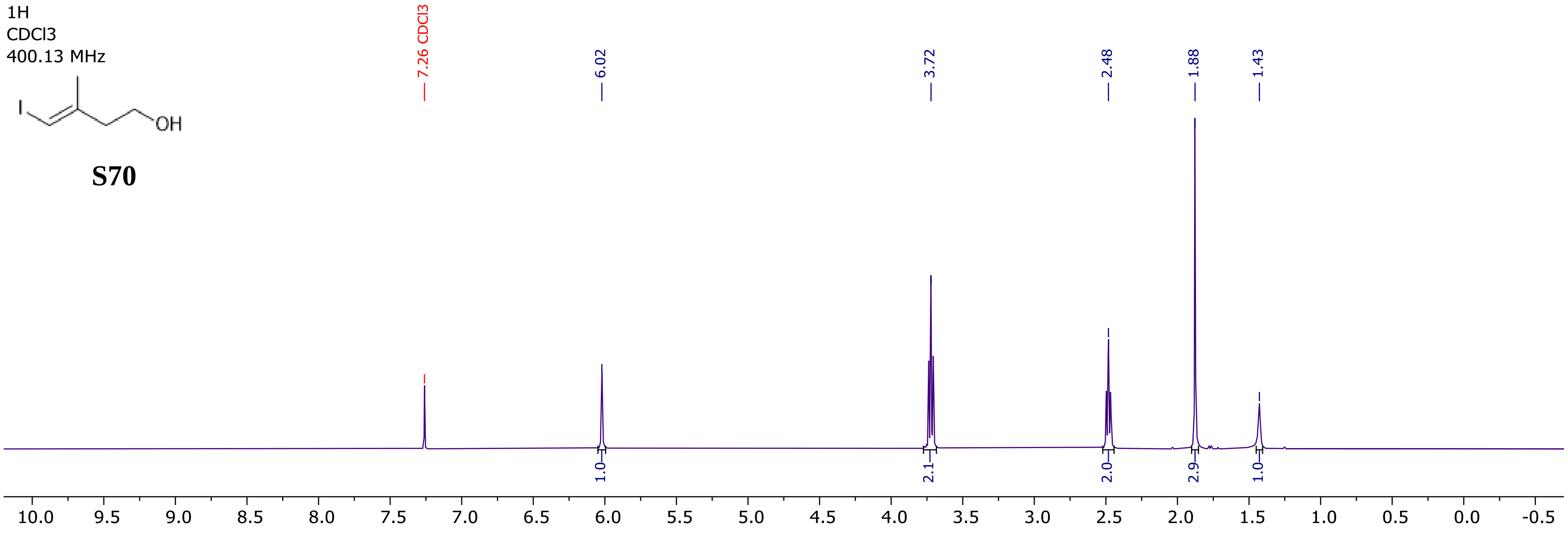
1H
CDCl3
400.13 MHz
I
OH
7.26 CDCl3
6.02
3.72
2.48
1.88
1.43
1.0
2.1
2.0
2.9
1.0
10.0
9.5
9.0
8.5
8.0
7.5
7.0
6.5
6.0
5.5
5.0
4.5
4.0
3.5
3.0
2.5
2.0
1.5
1.0
0.5
0.0
-0.5

**S70**

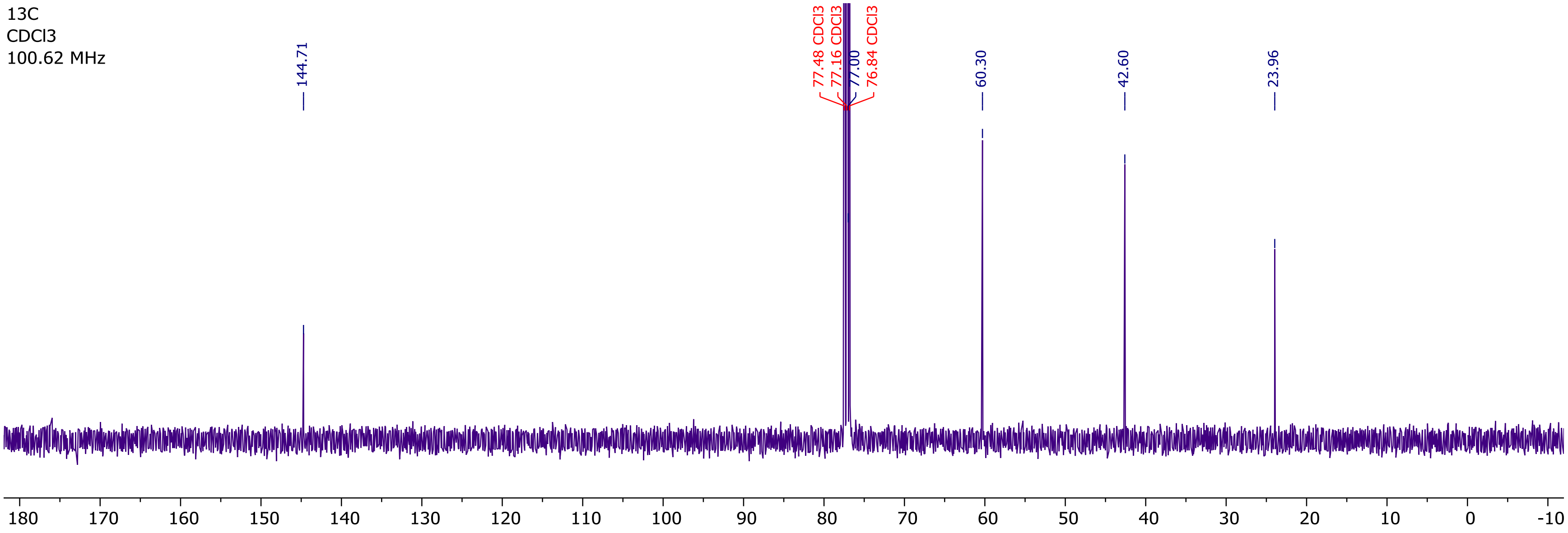
13C
CDCl3
100.62 MHz
144.71
77.48 CDCl3
77.16 CDCl3
77.00
76.84 CDCl3
60.30
42.60
23.96
180
170
160
150
140
130
120
110
100
90
80
70
60
50
40
30
20
10
0
-10

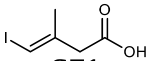
**S71**

Jones' reagent was prepared as follows: to a cold (0 °C) stirred suspension of $CrO_3$ (1.71 g, 17.1 mmol, 1 eq.) in $H_2O$ (6 mL) was added $H_2SO_4$ (1.5 mL, 28 mmol, 1.6 eq.) dropwise over 5 min. The reagent prepared in this way generates an approximately 2M solution of $H_2CrO_4$ in $H_2O$. To a cold (0 °C), stirred solution of **S70** (1.36 g, 6.42 mmol, 1.0 eq.) in acetone (58 mL) was added the previously prepared Jones' reagent solution (8 mL, 2M in $H_2O$, 16 mmol, 2.7 eq.). The reaction mixture was maintained at 0 °C for 30 min. After this time, starting material was consumed as monitored by TLC analysis and the reaction mixture was quenched cautiously with MeOH (15 mL), and allowed to stir for a further 10 minutes at 0 °C. The reaction mixture was then filtered through celite (1x 100 mL acetone wash) and the filtrate concentrated in vacuo to a small volume (*ca.* 10 mL). The concentrate was poured onto EtOAc (75 mL) and $H_2O$ (75 mL) in a separatory funnel, and the organic layer separated. The aqueous layer was extracted with EtOAc (3x 60 mL). The combined organic layers were washed with brine (1x 60 mL), dried ($Na_2SO_4$), filtered, and solvent was removed in vacuo. The crude orange solid was purified via flash column chromatography (7:3 to 1:1 Hexanes/EtOAc) to yield pure **S71** (896 mg, 62%) and slightly impure **S71** (238 mg, 16%) as white waxy solids.

**Analytical Data for S71:**

$R_f$ = 0.20 (7:3 Hexanes/EtOAc)

$^1$H NMR (600 MHz, $CDCl_3$) δ 6.21 (q, *J* = 1.2 Hz, 1H), 3.25 (s, 2H), 1.95 (d, *J* = 1.2 Hz, 3H).

$^{13}$C NMR (151 MHz, $CDCl_3$) δ 176.36, 139.77, 80.53, 43.89, 24.14.

IR (neat): $\nu_{max}$ $(cm^{-1})$ = 3060 (w, C=CH), 2954 (m, CH), 1713 (s, C=O), 1415 (m) 1281 (m).

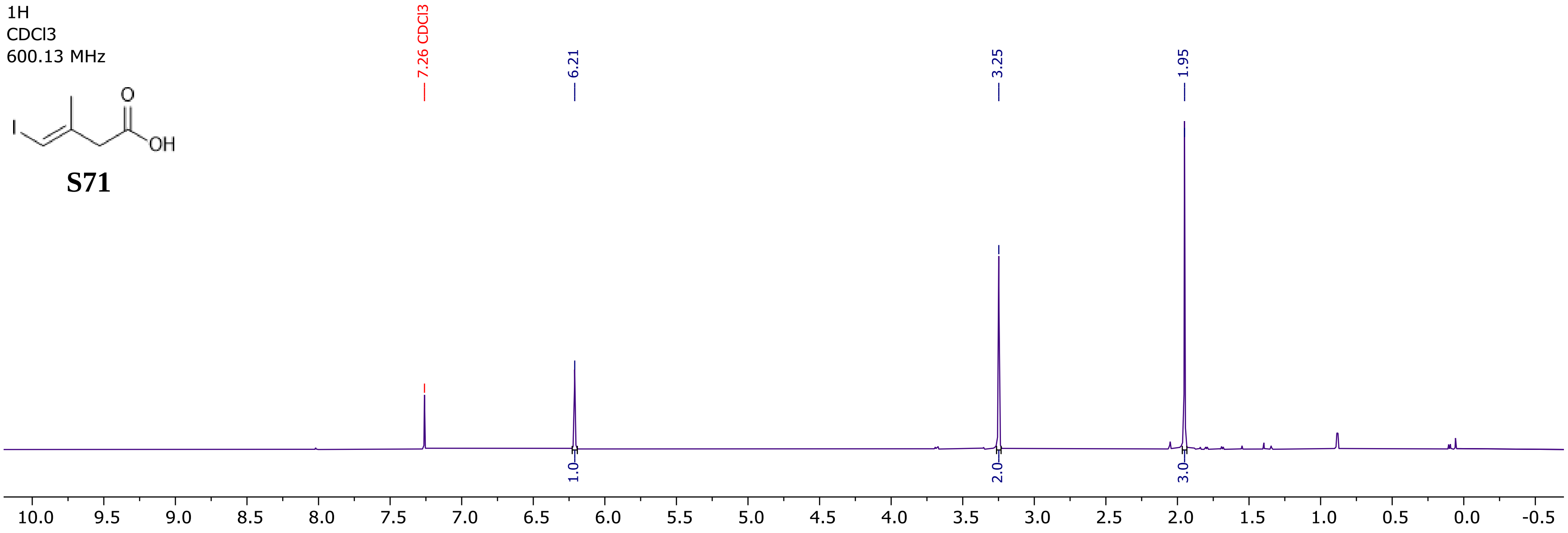
1H
CDCl3
600.13 MHz
S71
7.26 CDCl3
6.21
3.25
1.95
1.0
2.0
3.0
10.0
9.5
9.0
8.5
8.0
7.5
7.0
6.5
6.0
5.5
5.0
4.5
4.0
3.5
3.0
2.5
2.0
1.5
1.0
0.5
0.0
-0.5

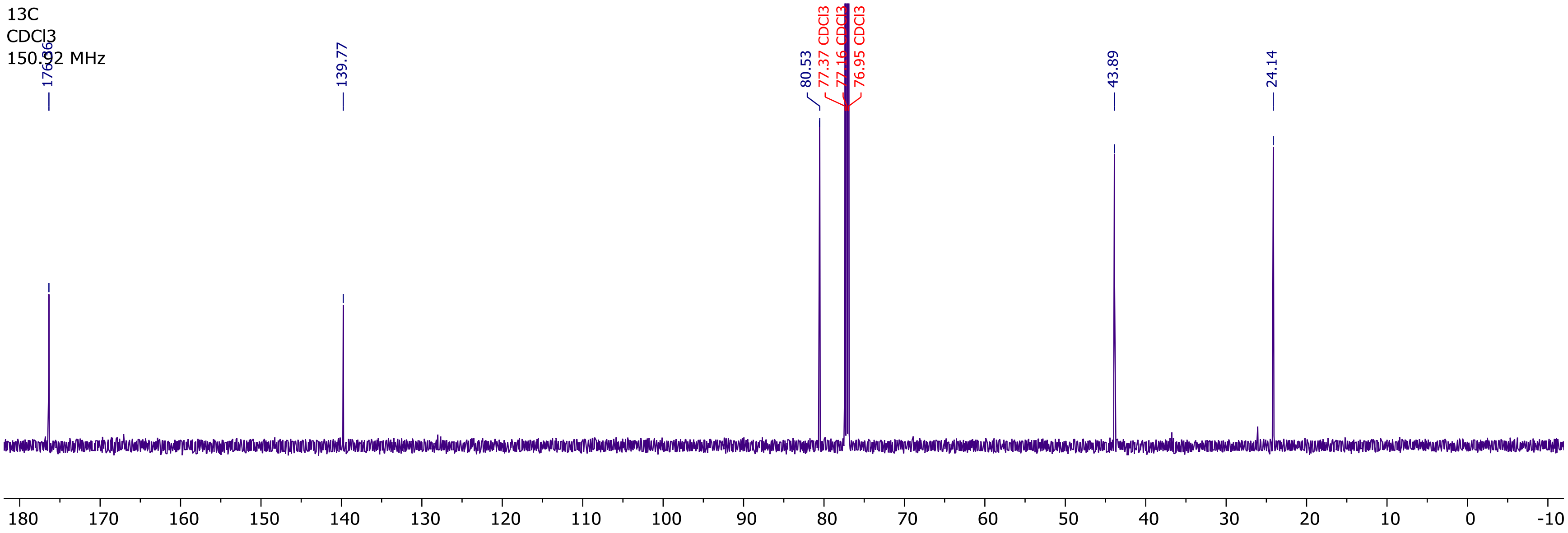
13C
CDCl3
150.92 MHz
176.96
139.77
80.53
77.37 CDCl3
77.16 CDCl3
76.95 CDCl3
43.89
24.14
180
170
160
150
140
130
120
110
100
90
80
70
60
50
40
30
20
10
0
-10

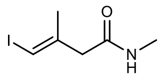

**3a**

Reaction performed in capped vial under ambient atmosphere. To a rt, stirred solution of **S71** (191.9 mg, 0.849 mmol, 1.0 eq.), HOBt (172.1 mg, 1.27 mmol, 1.5 eq.), and 4-dimethylaminopyridine (8 mg, 0.065 mmol, 0.077 eq.) in $CH_2Cl_2$ (4 mL) was added N,N'-diisopropylcarbodiimide (0.20 mL, 1.3 mmol, 1.5 eq.). The reaction mixture was allowed to stir at rt for 1 h. After this time, $MeNH_2$ (2.1 mL, 2M in THF, 4.2 mmol, 5.0 eq.) was added and the reaction mixture stirred for a further 1 h 25 min. After this time, starting material was consumed as monitored by TLC analysis and the reaction mixture was diluted with $CH_2Cl_2$ (3 mL) and poured onto $H_2O$ (10 mL). The organic layer was separated, and the aqueous layer was extracted with $CH_2Cl_2$ (3x 6 mL). The combined organic layers were dried ($Na_2SO_4$), filtered, and solvent was removed in vacuo. The crude product was purified via flash column chromatography (9:1 $CH_2Cl_2$/MeCN) to yield **3a** (110 mg, 54%) as a white solid.

**Analytical Data for 3a:**

$R_f$ = 0.20 (9:1 $CH_2Cl_2$/MeCN)

$^1$H NMR (600 MHz, $CDCl_3$) δ 6.18 (s, 1H), 5.71 (s, 1H), 3.12 (s, 2H), 2.80 (d, *J* = 4.8 Hz, 3H), 1.90 (s, 3H).

$^{13}$C NMR (151 MHz, $CDCl_3$) δ 169.67, 142.34, 80.11, 46.95, 26.67, 24.04.

HRMS (ESI): Anal. Calcd. for $C_6H_{11}NOI^+$ $[M+H]^+$ 239.9880, found 239.9876

IR (neat): $\nu_{max}$ $(cm^{-1})$ = 3286 (s, NH), 3096 (w), 3055 (w, C=*CH*), 2940 (w, CH), 2909 (w, CH), 1646 (s, C=C), 1554 (s), 1408 (m).

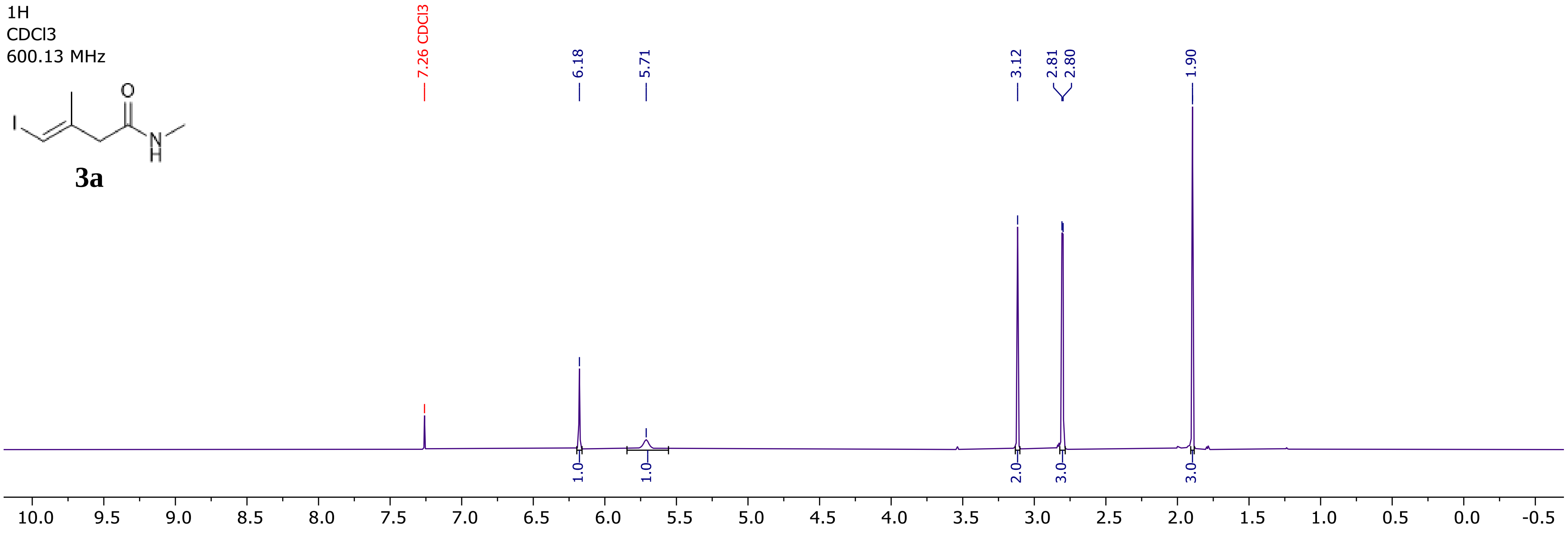

1H
CDCl3
600.13 MHz
7.26 CDCl3
6.18
5.71
3.12
2.81
2.80
1.90
1.0
1.0
2.0
3.0
3.0
10.0 9.5 9.0 8.5 8.0 7.5 7.0 6.5 6.0 5.5 5.0 4.5 4.0 3.5 3.0 2.5 2.0 1.5 1.0 0.5 0.0 -0.5


**3a**

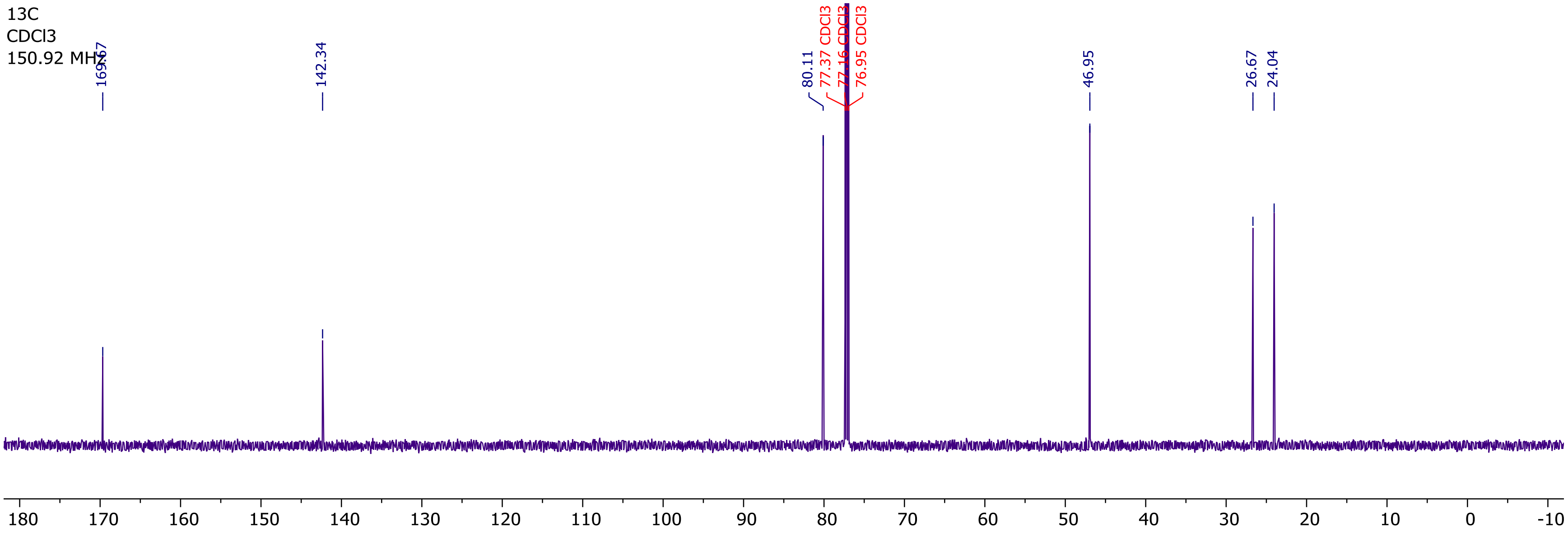

13C
CDCl3
150.92 MHz
169.67
142.34
80.11
77.37 CDCl3
77.16 CDCl3
76.95 CDCl3
46.95
26.67
24.04
180 170 160 150 140 130 120 110 100 90 80 70 60 50 40 30 20 10 0 -10

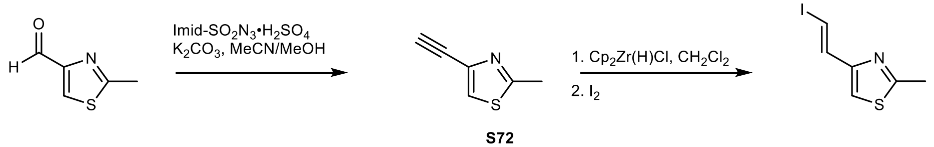


**Supplementary Fig. 40 | Synthesis of Thiazole Vinyl Iodide 3c.**
Abbreviation: Imid-$SO_2N_3$•$H_2SO_4$ = imidazolesulfonylazide hydrogensulfate.

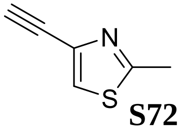
**S72**

**Danger!** The sulfonyl azide salt is known to pose an explosion hazard and is sensitive to friction and electrostatic discharge.[23] Plastic utensils were used for all manipulations of the sulfonyl azide salt.

Procedure was adapted from the literature.[24,27] To a rt, stirred suspension of $K_2CO_3$ (787.8 mg, 5.7 mmol, 5.7 eq.) and Dimethyl (2-oxopropyl)phosphonate (166 uL, 200 mg, 1.2 mmol, 1.2 eq.) in MeCN (5 mL) was added imidazole-1-sulfonyl azide sulphuric acid salt (325.5 mg, 1.2 mmol, 1.2 eq.) in one portion, and the suspension was stirred for 4 h 20 min. After this time, the supernatant became a deep yellow color and the 2-methylthiazole-4-carbaldehyde (129.2 mg, 1.0 mmol, 1.0 eq.) was added in MeOH (2.5 mL + 2.5 mL rinse). The reaction was stirred at rt for 18 h. After this time, the reaction mixture was filtered through a cotton plug, celite was added, and the solvent carefully removed in vacuo (no lower than 400 mmHg) to load the crude product onto celite. The crude product was purified via flash column chromatography (4:1 Pentane/$Et_2O$). Appropriate fractions were pooled, and solvent was removed in vacuo to yield **S72** (92.3 mg, 74%) as a colorless liquid.

**Analytical Data for S72:**

$R_f$ = 0.40 (3:1 Pentane/$Et_2O$)

$^1$H NMR (400 MHz, $CD_2Cl_2$) δ 7.39 (s, 1H), 3.12 (s, 1H), 2.68 (s, 3H).

$^{13}$C NMR (101 MHz, $CD_2Cl_2$) δ 166.21, 136.17, 124.32, 78.33, 76.99, 19.36.

HRMS (ESI): Anal. Calcd. for $C_6H_6NS^+$ $[M+H]^+$ 124.0216, found 124.0204

IR (neat): $\nu_{max}$ $(cm^{-1})$ = 3290 (s, C=*CH*), 3114 (w, Aromatic C=*CH*), 2976 (m, CH), 2923 (m, CH), 2112 (w, C≡C), 1493 (s), 1437 (s), 1377 (m), 1279 (s).

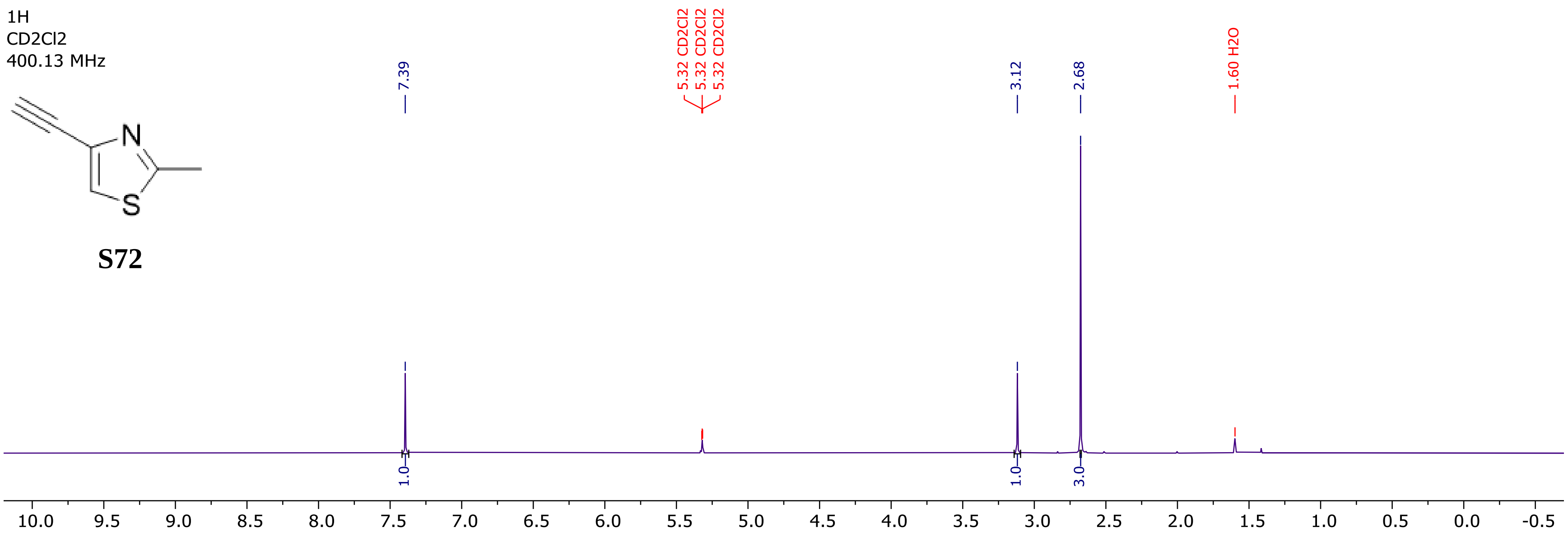

**S72**

1H
CD2Cl2
400.13 MHz
7.39
5.32 CD2Cl2
5.32 CD2Cl2
5.32 CD2Cl2
3.12
2.68
1.60 H2O
1.0
1.0
3.0
10.0 9.5 9.0 8.5 8.0 7.5 7.0 6.5 6.0 5.5 5.0 4.5 4.0 3.5 3.0 2.5 2.0 1.5 1.0 0.5 0.0 -0.5

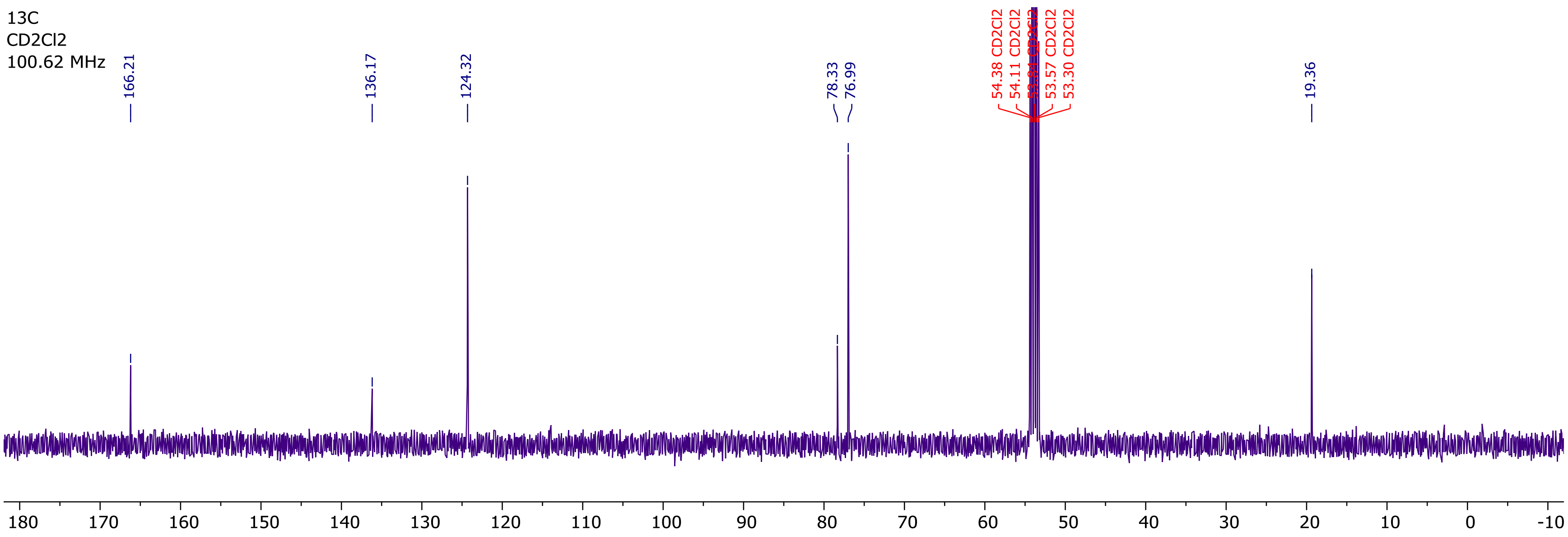
13C
CD2Cl2
100.62 MHz
166.21
136.17
124.32
78.33
76.99
54.38 CD2Cl2
54.11 CD2Cl2
53.57 CD2Cl2
53.30 CD2Cl2
19.36
180 170 160 150 140 130 120 110 100 90 80 70 60 50 40 30 20 10 0 -10

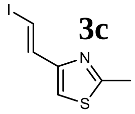
**3c**

Procedure was adapted from the literature.[27] To a cold (0 °C), stirred suspension of $Cp^2Zr(H)Cl$ (179.0 mg, 0.694 mmol, 1.3 eq.) in $CH_2Cl_2$ (2.7 mL) was added the alkyne **S72** (65.8 mg, 0.534 mmol, 1.0 eq.). Immediately following addition of the alkyne, the suspension becomes a homogenous yellow-orange solution. After stirring for a further 1 h 15 min at 0 °C, further $Cp^2Zr(H)Cl$ (77.1 mg, 0.299 mmol, 0.56 eq.) was added in one portion. After stirring for a further 30 min 0 °C, further $Cp^2Zr(H)Cl$ (35.7 mg, 0.138 mmol, 0.26 eq.). After stirring a further 20 min at 0 °C, starting material was consumed as monitored by TLC analysis and solid $I_2$ (176.2 mg, 0.694 mmol, 1.3 eq.) was added in one portion to the reaction mixture. The reaction mixture was stirred for a further 5 min. to ensure the brown color persisted in solution. After this time, the reaction mixture was quenched by addition of 10% aqueous $Na_2S_2O_3$ (3 mL) and allowed to stir for 5 min. The reaction mixture was poured onto $H_2O$ (5 mL), the organic layer separated, and the aqueous layer was extracted with $Et_2O$ (5x 10 mL). The combined organic layers were washed with $H_2O$ (2x 10 mL) and brine (1x 15 mL). The combined organic layers were dried ($MgSO_4$), filtered, and solvent was removed in vacuo. The crude product was purified by flash column chromatography (19:1 Pentane/$Et_2O$). Appropriate fractions were pooled, and solvent was removed in vacuo to yield **3c** (94 mg, 70%) as a yellow oil.

**Analytical Data for 3c:**

$R_f$ = 0.40 (9:1 Hexanes/$Et_2O$)

$^1H$ NMR (400 MHz, $CDCl_3$) δ 7.33 (d, *J* = 14.5 Hz, 1H), 7.20 (d, *J* = 14.4 Hz, 1H), 6.91 (s, 1H), 2.69 (s, 3H).

$^{13}C$ NMR (101 MHz, $CDCl_3$) δ 166.39, 153.61, 137.58, 115.27, 80.49, 19.41.

HRMS (ESI): Anal. Calcd. for $C_6H_7NSI^+$ $[M+H]^+$ 251.9338, found 251.9329

IR (neat): $\nu_{max}$ $(cm^{-1})$ = 3104 (w, C=*CH*), 3075 (w, C=*CH*), 3054 (w), 2971 (m, CH), 2920 (m, CH), 1730 (w), 1597 (m), 1502 (m), 1437 (m), 1375 (m), 1324 (m), 1281 (m).

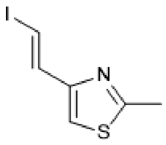
I
N
S

**3c**

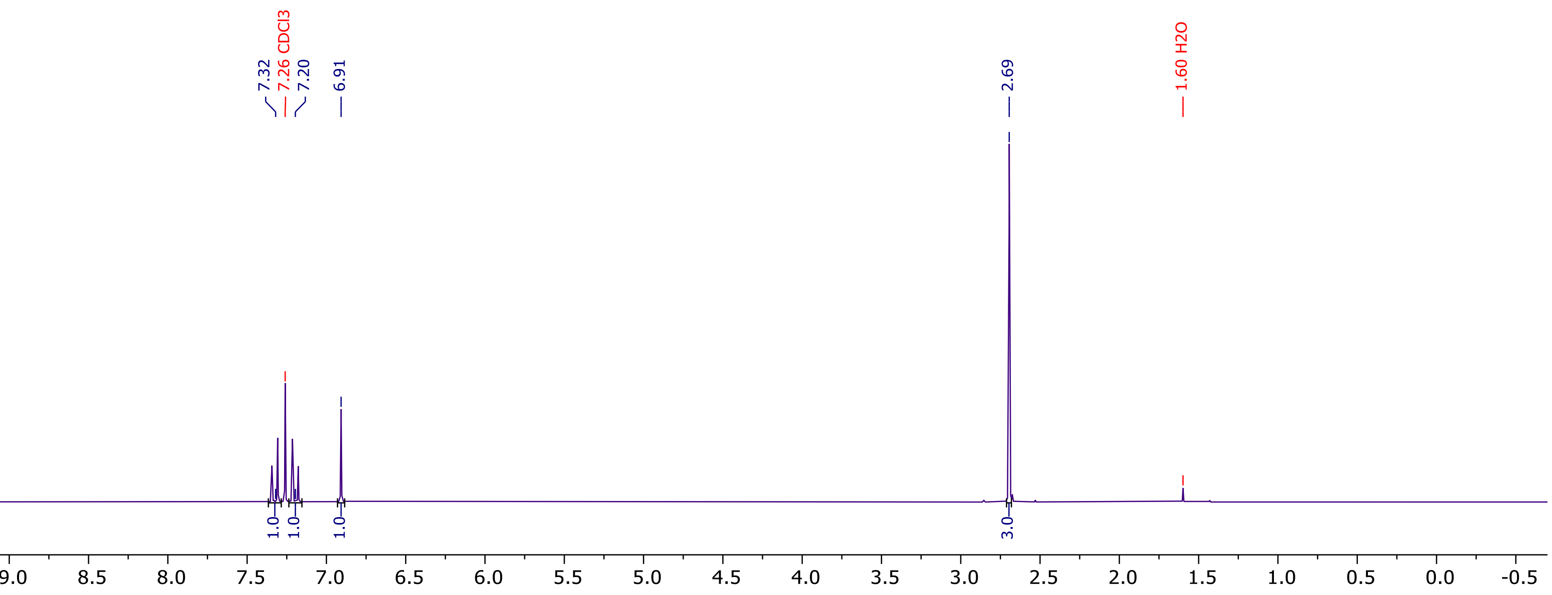
CDCl3
400.13 MHz
7.32
7.26 CDCl3
7.20
6.91
2.69
1.60 H2O
1.0
1.0
1.0
3.0
10.0 9.5 9.0 8.5 8.0 7.5 7.0 6.5 6.0 5.5 5.0 4.5 4.0 3.5 3.0 2.5 2.0 1.5 1.0 0.5 0.0 -0.5

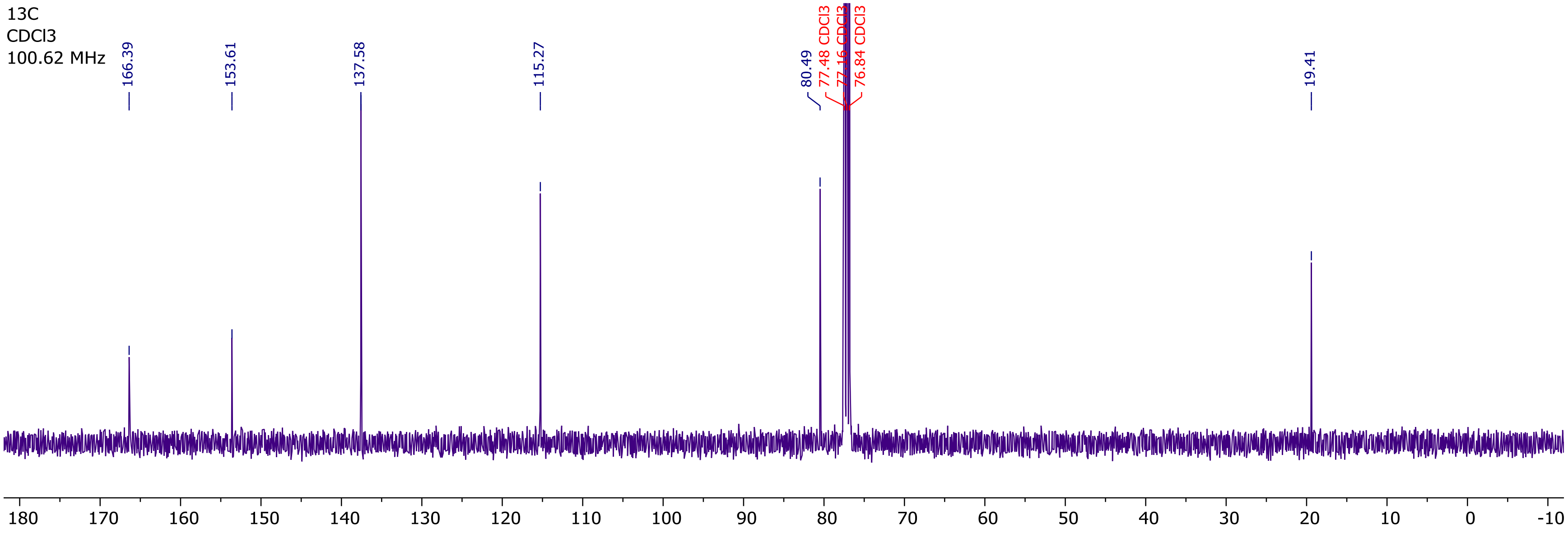
13C
CDCl3
100.62 MHz
166.39
153.61
137.58
115.27
80.49
77.48 CDCl3
77.16 CDCl3
76.84 CDCl3
19.41
180 170 160 150 140 130 120 110 100 90 80 70 60 50 40 30 20 10 0 -10

## Procedures for the Synthesis and Characterization of pMLs

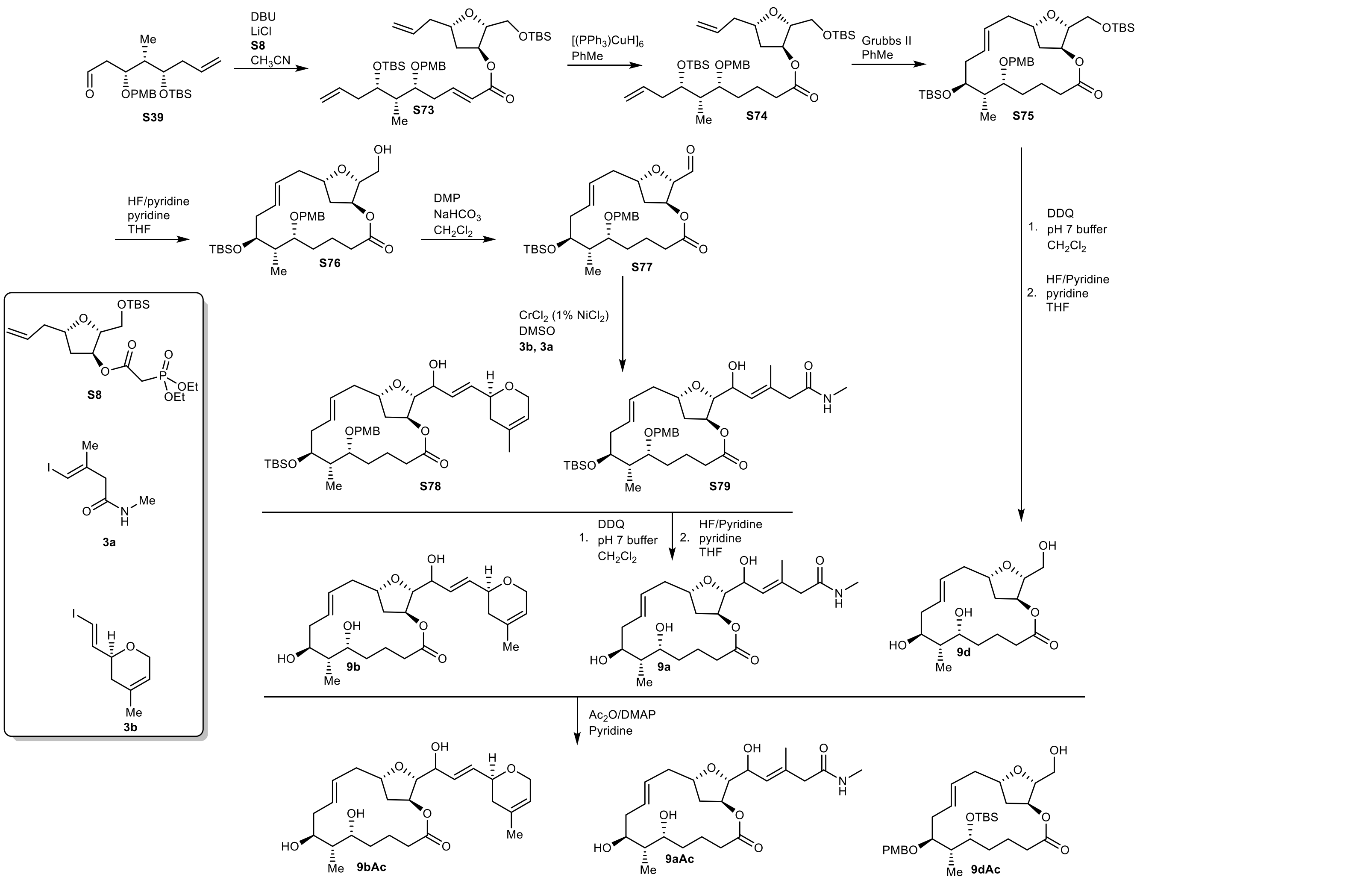


**Supplementary Fig. 41 | Synthesis of pMLs 9a, 9b, 9d, 9aAc, 9bAc, and 9dAc.**
Abbreviations: DBU = 1,8-Diazabicyclo[5.4.0]undec-7-ene, THF = tetrahydrofuran, DMSO = dimethylsulfoxide, DDQ = 2,3-dichloro-5,6-dicyano-*para*-benzoquinone, Grubbs II = Dichloro[1,3-bis(2,4,6-trimethylphenyl)-2-imidazolidinylidene](benzylidene)(tricyclohexylphosphine)ruthenium(II), DMP = Dess-Martin periodinane, DMAP = 4-dimethylaminopyridine, TBS = tert-butyldimethylsilyl, PMB = *para*-methoxybenzyl.

To a cold (0 °C), stirred suspension of LiCl (9.3 mg, 0.22 mmol, 1.3 eq., flame dried in vacuo), phosphonate **S8** (82.8 mg, 0.183 mmol, 1.1 eq.), and aldehyde **S39** (67.8 mg, 0.167 mmol, 1.0 eq.) in MeCN (2.1 mL) was added DBU (27.5 µL, 28 mg, 0.183 mmol, 1.1 eq.) via microsyringe. The reaction mixture was allowed to slowly warm to room temperature over the course of 20 min. After this time, starting material was consumed as monitored by TLC analysis, and the reaction mixture was quenched by addition of a saturated aqueous solution of $NH_4Cl$ (2 mL). The biphasic mixture was poured into a separatory funnel and extracted with $CH_2Cl_2$ (5x 5 mL). The combined organic layers were dried ($Na_2SO_4$), filtered, and solvent was removed in vacuo. The crude product was purified via flash column chromatography (9:1 Hexanes:$Et_2O$). Appropriate fractions were pooled, and solvent was removed in vacuo to yield **S73** (106.5 mg) as a colorless oil which was used immediately in the subsequent step.

To a rt, stirred suspension of $Cu(OAc)_2{\bullet}H_2O$ (125 mg, 0.63 mmol) and $PPh_3$ (325 mg, 1.24 mmol) in deoxygenated toluene (23.5 mL) was added 1,1,3,3-tetramethyldisiloxane (1.65 mL, 1.25 g, 9.31 mmol) via syringe. The teal-colored suspension was allowed to stir for 24 h. After this time the suspension turned into a homogenous brick-red solution, indicating the presence of $[(PPh_3)CuH]_6$. To **S73** (106.5 mg, 0.151 mmol, 1.0 eq.) was added a solution of the previously prepared reagent $[(PPh_3)CuH]_6$ (1.6 mL, *ca.* 0.4 M in organosilane, 0.64 mmol, 4.2 eq.). After stirring at rt for 16 h, the reaction mixture was poured onto a silica gel column and the products eluted with $Et_2O$. The solvent was removed in vacuo, and $^1H$ NMR analysis of the crude indicated complete conversion. The crude product was purified via flash column chromatography (9:1 Hexanes/$Et_2O$). Appropriate fractions were pooled, and solvent was removed in vacuo to yield **S74** (76.4 mg) as a colorless oil which was used immediately in the subsequent step.

To a warm (60 °C), stirred solution of **S74** (76.4 mg, 0.109 mmol, 1.0 eq.) in deoxygenated toluene (43 mL) that was continuously being sparged with $N_2(g)$ was added Grubbs second generation catalyst (18.4 mg, 0.0217 mmol, 0.20 eq.) in toluene (1 mL). The reaction was allowed to proceed under continuous nitrogen sparge for 3 h. After this time, the reaction did not undergo further conversion as monitored by TLC analysis. The reaction mixture was cooled to rt and quenched by addition of potassium 2-isocyanoacetate (20 mg, 0.16 mmol, 1.5 eq.) in MeOH (1 mL). The reaction mixture was stirred for a further 30 min, after which time the red solution changed to a pale-yellow color. The reaction mixture was concentrated in vacuo to give a crude solid deposited on the walls of the evaporation flask. The walls were scraped with a spatula and the solid was triturated with $Et_2O$ (3x 5 mL) and filtered through a short plug of silica gel eluting with $Et_2O$. The filtrate was concentrated in vacuo and the crude product was purified via flash column chromatography (19:1 to 17:3 Hexanes/$Et_2O$). Appropriate fractions were pooled, and solvent was removed in vacuo to yield **S75** (51 mg, 45%, 4 steps from alcohol **2b**) as a colorless oil which was subsequently combined with a previous batch of material and used in the next steps.

Reaction run under ambient atmosphere in a capped vial. To a rt, vigorously stirred solution of **S75** (4.5 mg, 0.0066 mmol, 1.0 eq.) in $CH_2Cl_2$ (1 mL) was added in sequence pH 7 phosphate buffer (0.4 mL) and 2,3-dichloro-5,6-dicyano-benzoquinone (9.0 mg, 0.040

mmol, 6 eq.). After 25 min. the starting material was consumed as monitored by TLC analysis and the reaction was quenched with saturated $NaHCO_3$ (3 mL). The aqueous layer was extracted with $CH_2Cl_2$ (3x 4 mL) and the combined organic layers were dried ($Na_2SO_4$), filtered, and solvent was removed in vacuo. The crude product was purified via flash column chromatography (3:2 Hexanes/$Et_2O$). Appropriate fractions were pooled, and solvent was removed in vacuo to yield the alcohol (2.2 mg) as a colorless oil which was used immediately in the subsequent step.

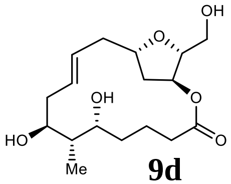

**9d**

Note: Reaction run under ambient atmosphere in a capped polypropylene vial. To a rt, stirred solution of the alcohol from the previous step (2.2 mg, 0.0038 mmol, 1.0 eq.) in THF (0.8 mL), was added in sequence pyridine (0.10 mL), and HF-Pyridine (70% (w/w) HF, 50 µL, 1.92 mmol HF, 505 eq. HF). The reaction mixture was stirred at rt for 4 d. After this time, starting material was consumed as monitored by TLC analysis, and the reaction mixture was quenched by pipetting it onto a saturated aqueous solution of $NaHCO_3$ (3 mL) [**caution!** $CO_2$(g) evolved]. After the evolution of gas had ceased, aqueous layer was extracted with $CH_2Cl_2$ (3x 4 mL). The combined organic layers were dried ($Na_2SO_4$), filtered, and solvent was removed in vacuo. The crude product was purified via flash column chromatography (97:3 to 47:3 $CH_2Cl_2$/MeOH). Appropriate fractions were pooled, and solvent was removed in vacuo to yield **9d** (0.80 mg, 16%, 6 steps from **2b**) as a colorless film.

**Analytical Data for 9d:**

$R_f$ = 0.33 (87.5:12.5 $CH_2Cl_2$/MeOH)

$[\alpha]_D^{20}$ = +140 ° (c = 0.80, MeOH)

$^1$H NMR (601 MHz, MeOD) δ 5.58 – 5.48 (m, 2H), 5.11 (d, *J* = 4.4 Hz, 1H), 4.03 (ddd, *J* = 5.6, 4.4, 1.0 Hz, 1H), 3.73 (tt, *J* = 11.2, 4.0 Hz, 1H), 3.57 (dd, *J* = 11.7, 4.5 Hz, 1H), 3.48 (dd, *J* = 11.7, 5.7 Hz, 1H), 3.36 (td, *J* = 8.7, 2.7 Hz, 1H), 3.33 – 3.32 (m, 1H), 2.54 (ddd, *J* = 11.8, 7.6, 4.4 Hz, 1H), 2.33 (ddd, *J* = 12.4, 5.7, 3.8 Hz, 1H), 2.27 (td, *J* = 12.1, 3.9 Hz, 1H), 2.22 (dd, *J* = 9.5, 5.4 Hz, 1H), 2.16 (ddd, *J* = 12.2, 7.8, 3.8 Hz, 1H), 2.06 – 1.97 (m, 2H), 1.93 – 1.85 (m, 1H), 1.74 – 1.64 (m, 1H), 1.59 (ddd, *J* = 13.5, 11.6, 4.5 Hz, 1H), 1.56 – 1.47 (m, 2H), 1.28 – 1.20 (m, 1H), 0.93 (d, *J* = 7.0 Hz, 3H).

$^{13}$C NMR (151 MHz, MeOD) δ 175.80, 131.40, 130.32, 85.02, 82.24, 78.77, 76.15, 73.44, 63.54, 41.92, 39.38, 38.15, 36.54, 36.48, 36.04, 24.16, 8.20.

HRMS (ESI): Anal. Calcd. for $C_{17}H_{29}O_6^+$ $[M+H]^+$ 329.1959, found 329.1957

IR (neat): $\nu_{max}$ ($cm^{-1}$) = 3381 (br, OH), 2920 (m, CH), 2856 (m, CH), 1717 (s, C=O), 1642 (w, C=C), 1444 (m), 1343 (m), 1256 (m).

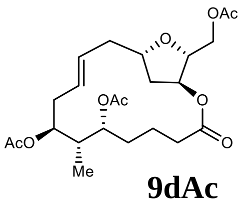


**9dAc**

**9dAc** was prepared according to general procedure A (0.73 mg, 60%)

**Analytical Data for 9dAc:**

$R_f$ = 0.80 (2:3 Hexanes/EtOAc)

HRMS (ESI): Anal. Calcd. for $[M+NH_4]^+$ 594.3273, found 594.3281

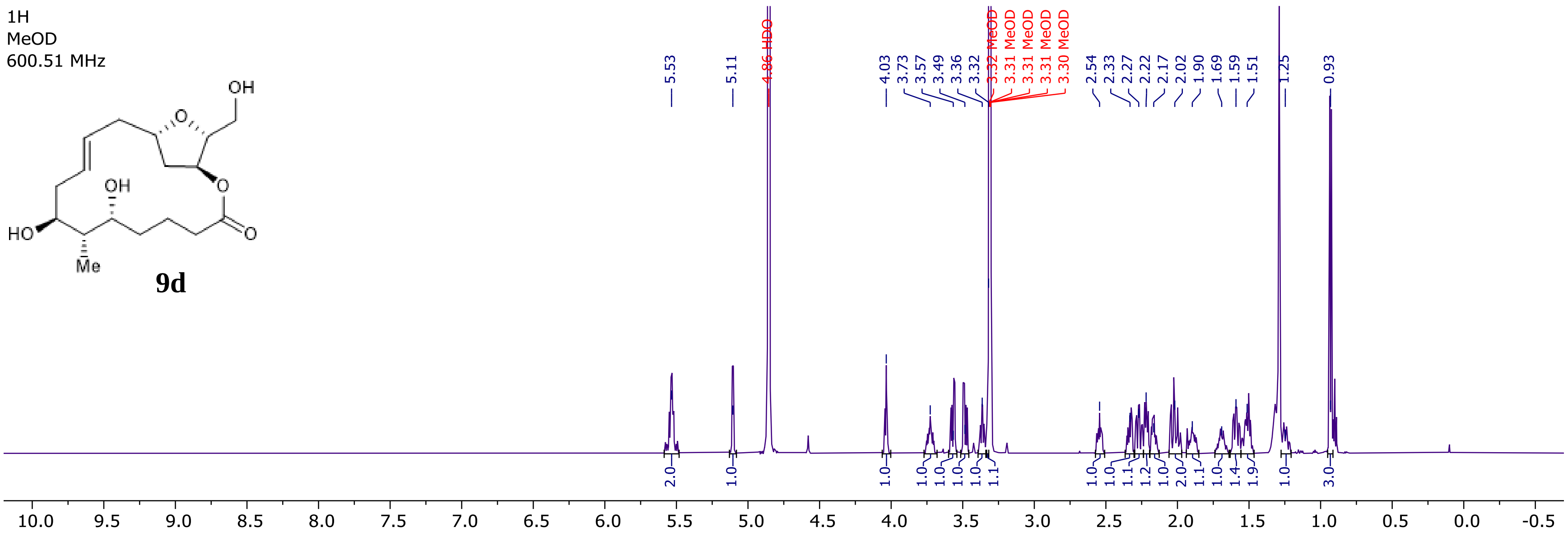
1H
MeOD
600.51 MHz
9d
5.53
5.11
4.86 HDO
4.03
3.73
3.57
3.49
3.36
3.32
3.32 MeOD
3.31 MeOD
3.31 MeOD
3.31 MeOD
3.30 MeOD
2.54
2.33
2.27
2.22
2.17
2.02
1.90
1.69
1.59
1.51
1.25
0.93

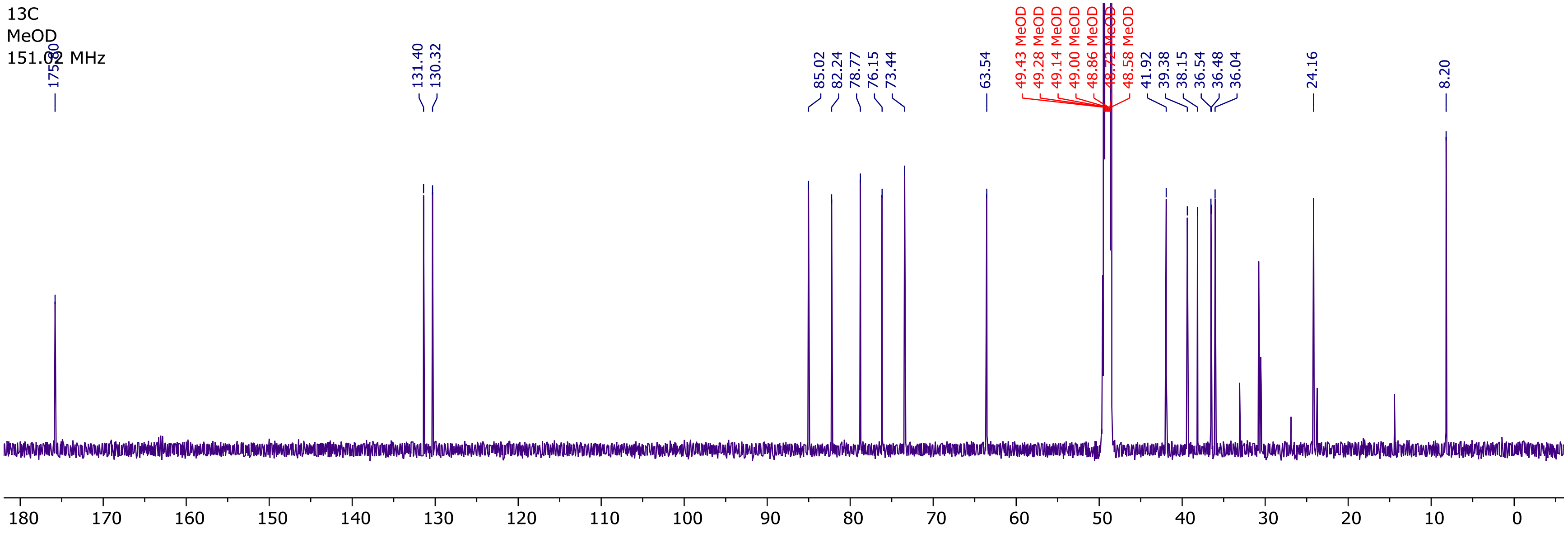
13C
MeOD
151.02 MHz
175.90
131.40
130.32
85.02
82.24
78.77
76.15
73.44
63.54
49.43 MeOD
49.28 MeOD
49.14 MeOD
49.00 MeOD
48.86 MeOD
48.72 MeOD
48.58 MeOD
41.92
39.38
38.15
36.54
36.48
36.04
24.16
8.20

An approximately 2.56 M solution of HF in THF/Pyridine was prepared as follows. HF-Pyridine (70% (w/w), 1 mL, 0.77 g HF, 38.5 mmol HF) was slowly added to a cold (0 °C) stirred solution of THF (10 mL) and pyridine (4 mL) in a polypropylene falcon tube.

Reaction run under ambient atmosphere in a capped polypropylene vial. To a rt, stirred solution of **S75** (71 mg, 0.105 mmol, 1.0 eq.) in THF (2.1 mL) was added the previously mixed HF/Pyridine/THF solution (0.84 mL, 2.56 M HF, 2.15 mmol HF, 2.15 mmol, 20 eq. HF). After stirring for 19 h at rt, further stock HF/Pyridine/THF solution was added (0.40 mL, 2.56 M HF, 1.02 mmol, 10 eq.), and the reaction mixture was stirred for a further 3 d. After this time, the reaction was slowly quenched with saturated $NaHCO_3$ (5 mL) [**caution!** $CO_2$ (g) liberated]. After evolution of gas had ceased, the aqueous layer was extracted with $CH_2Cl_2$ (3x 5 mL) and the combined organic layers were washed with brine (15 mL), dried ($Na_2SO_4$), filtered, and the solvent was removed in vacuo. The crude product was purified via flash column chromatography (2:3 Hexanes/$Et_2O$). Appropriate fractions were pooled, and solvent was removed in vacuo to yield **S76** (35 mg, 59%) as a colorless oil.

Note: Reaction run in a capped vial under ambient atmosphere. To a rt, stirred solution of **S76** (65 mg, 0.115 mmol, 1.0 eq.) in $CH_2Cl_2$ (2.3 mL) was added $NaHCO_3$ (58 mg, 0.690 mmol, 6 eq.) followed by Dess-Martin Periodinane (73.2 mg, 0.173 mmol, 1.5 eq.). The mixture was stirred at rt for 1 h. After this time, starting material was consumed as monitored by TLC analysis. The reaction mixture was quenched with a 1:1:1 (v/v/v) $H_2O$-saturated aqueous $NaHCO_3$-10% aqueous $Na_2S_2O_3$ (3 mL) and the biphasic mixture was stirred vigorously for 30 min at rt. The aqueous layer was extracted with $CH_2Cl_2$ (3x 10 mL), and the combined organic layers were dried ($Na_2SO_4$), filtered, and the solvent was removed in vacuo. The crude product was passaged through a short plug of silica gel eluting with $Et_2O$. Solvent was removed in vacuo to yield the crude aldehyde **S77** (*ca.* 24 mg) as a colorless oil which was used immediately in the next step without further purification.

To a solution of **S77** (24 mg, 0.043 mmol, 1.0 eq.) in deoxygenated (5x freeze-pump-thaw cycles) DMSO (1 mL) was added $CrCl_2$ doped with 1 % $NiCl_2$ (w/w) (52.4 mg, 0.426 mmol, 10 eq.). **3b** (25 mg, 0.10 mmol, 2.3 eq.) was added via syringe in deoxygenated DMSO (0.5 mL + 0.5 mL rinse) and the mixture was stirred for 17 h 30 min. After this time, the reaction mixture was transferred to a separatory funnel, diluted with $Et_2O$ (10 mL) and 10 mL of 1:1 (v/v) $H_2O$-brine was added. The organic layer was separated, and the aqueous layer extracted with $Et_2O$ (5x 10 mL). The combined organic layers were dried ($MgSO_4$), filtered, and solvent was removed in vacuo. The crude product was purified via flash column chromatography (9:1 to 17:3 Hexanes/Acetone). Appropriate fractions were pooled, and solvent was removed in vacuo to yield **S78** (8.3 mg) as a colorless oil and a 1:1 mixture of diastereomers. The product was used immediately in the subsequent steps.

Reaction run under ambient atmosphere in a capped vial. To a rt, vigorously stirred solution of **S78** (8.3 mg, 0.012 mmol, 1.0 eq.) in $CH_2Cl_2$ (1.2 mL) was added in sequence pH 7 phosphate buffer (0.4 mL) and 2,3-dichloro-5,6-dicyano-benzoquinone (7.5 mg, 0.033 mmol, 2.8 eq.). After 15 min, additional 2,3-dichloro-5,6-dicyano-benzoquinone (2.0 mg, 0.0088 mmol, 0.73 eq.) was added. After a

further 20 min, starting material was consumed as monitored by TLC analysis. The reaction mixture was pipetted onto saturated aqueous $NaHCO_3$ (5 mL). The aqueous layer was extracted with $CH_2Cl_2$ (3x 10 mL) and the combined organic layers were dried ($Na_2SO_4$), filtered, and solvent was removed in vacuo. The crude product was purified via flash column chromatography (4:1 to 7:3 Hexanes/Acetone). Appropriate fractions were pooled, and solvent was removed in vacuo to yield the diol (6.1 mg) as a colorless oil which was used immediately in the subsequent step.

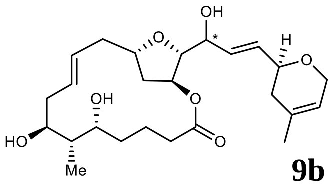


**9b**

* 1:1 mixture of diastereomers

Reaction run under ambient atmosphere in a capped polypropylene vial. To a rt, stirred solution of the diol from the previous step (6.1 mg, 0.011 mmol, 1.0 eq.) in THF (1 mL), was added in sequence pyridine (0.06 mL), and HF-pyridine (70% (w/w) HF, 0.06 mL, 2 mmol HF, 200 eq. HF). After 5 d, conversion was incomplete by TLC and additional HF-pyridine was added (70% (w/w) HF, 0.03 mL, 1 mmol HF, 100 eq. HF). The reaction mixture was stirred at rt for a further 10 d. After this time, starting material was consumed as monitored by TLC analysis, and the reaction mixture was quenched by pipetting it onto a saturated aqueous solution of $NaHCO_3$ (5 mL) [**caution!** $CO_2$(g) evolved]. After the evolution of gas had ceased, the aqueous layer was extracted with $CH_2Cl_2$ (5x 5 mL). The combined organic layers were dried ($Na_2SO_4$), filtered, and solvent was removed in vacuo. The crude product was purified via flash column chromatography (3:2 Hexanes/Acetone). Appropriate fractions were pooled, and solvent was removed in vacuo to yield **9b** (4.5 mg, 2.2%, 9 steps from **2b**) as a white solid.

**Analytical Data for 9b:**

$R_f$ = 0.23 (3:2 Hexanes/Acetone)

$^1$H NMR (601 MHz, $CD_2Cl_2$) δ 5.92 – 5.81 (m, 1H), 5.72 (dddd, *J* = 15.8, 10.3, 5.8, 1.4 Hz, 1H), 5.51 (ddt, *J* = 6.6, 4.1, 1.9 Hz, 2H), 5.42 (ddt, *J* = 3.8, 2.5, 1.3 Hz, 1H), 5.07 (dd, *J* = 4.9, 1.5 Hz, 0.6H), 5.01 (dd, *J* = 4.8, 1.4 Hz, 0.4H), 4.23 (ddt, *J* = 5.4, 4.0, 1.4 Hz, 0.6H), 4.16 – 4.12 (m, 2H), 4.07 – 4.04 (m, 0.4H), 4.04 – 3.99 (m, 1H), 3.98 (dd, *J* = 4.0, 1.5 Hz, 0.6H), 3.95 (dd, *J* = 5.5, 1.4 Hz, 0.4H), 3.73 (dtt, *J* = 14.9, 11.1, 3.8 Hz, 1H), 3.55 – 3.46 (m, 2H), 2.59 (ddt, *J* = 11.5, 6.7, 4.6 Hz, 1H), 2.35 – 2.23 (m, 2.6H), 2.21 – 2.11 (m, 2.3H), 2.05 – 1.95 (m, 2.3H), 1.95 – 1.88 (m, 1.8H), 1.81 – 1.73 (m, 2H), 1.71 – 1.68 (m, 3H), 1.64 – 1.58 (m, 1H), 1.58 – 1.50 (m, 2H), 1.50 – 1.41 (m, 2H), 0.87 (d, *J* = 7.1 Hz, 3H).

$^{13}$C NMR (151 MHz, $CD_2Cl_2$) δ 174.32, 134.13, 133.69, 131.85, 130.62, 130.57, 129.71, 129.69, 129.32, 128.27, 120.08, 120.06, 86.68, 86.37, 81.12, 80.82, 77.26, 77.14, 77.06, 76.04, 75.52, 73.46, 73.43, 72.94, 71.99, 65.94, 65.89, 40.06, 39.62, 39.09, 39.07, 37.20, 37.18, 36.06, 36.00, 35.78, 35.71, 23.04, 22.54, 22.52, 5.58.

HRMS (ESI): Anal. Calcd. for $C_{25}H_{39}O_7^+$ $[M+H]^+$ 451.2691, found 451.2682

IR (neat): $\nu_{max}$ ($cm^{-1}$) = 3376 (br, OH), 2918 (m, CH), 2853 (m, CH), 1715 (s, C=O), 1643 (s, C=C), 1447 (m) 1373 (m), 1256 (m)

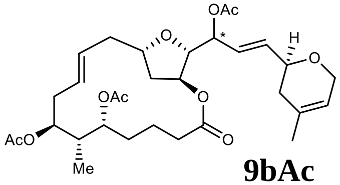


**9bAc**

* 1:1 mixture of diastereomers

**9bAc** was prepared according to general procedure A (0.99 mg, 77%)

**Analytical Data for 9bAc:**

$R_f$ = 0.86 (3:2 Hexanes/EtOAc)

HRMS (ESI): Anal. Calcd. for $C_{31}H_{48}NO_{10}^+$ $[M+NH_4]^+$ 594.3273, found 594.3281

1H
CD2Cl2
600.51 MHz

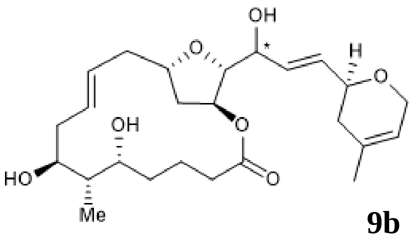


**9b**

* 1:1 mixture of diastereomers

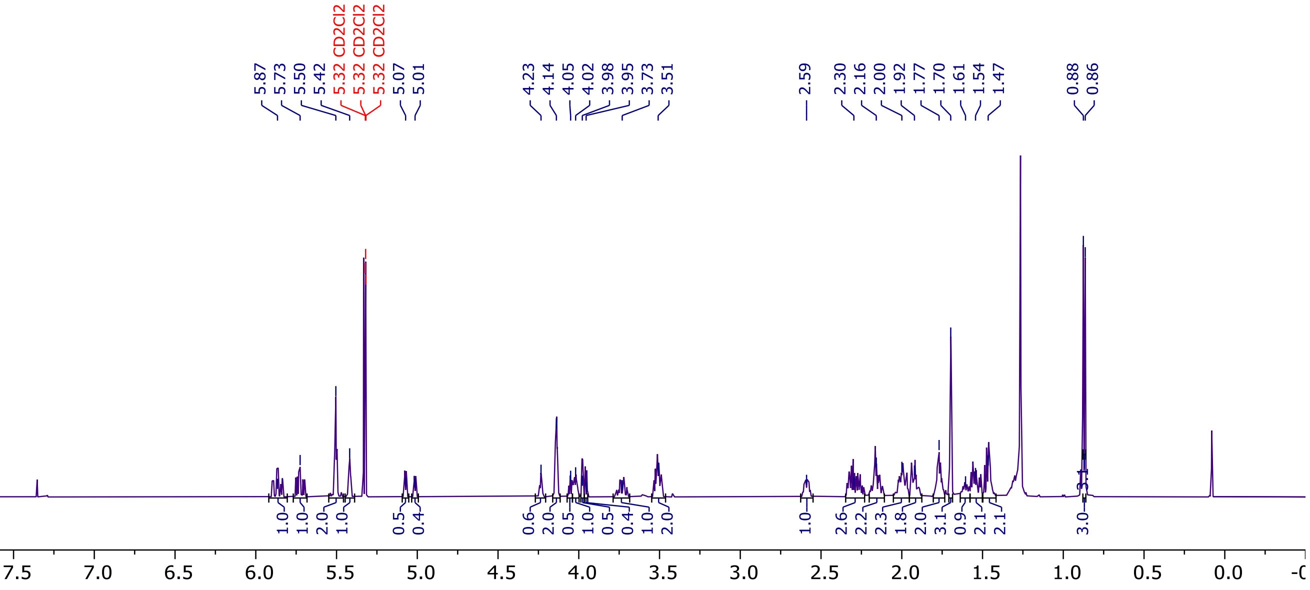


13C
CD2Cl2
151.02 MHz

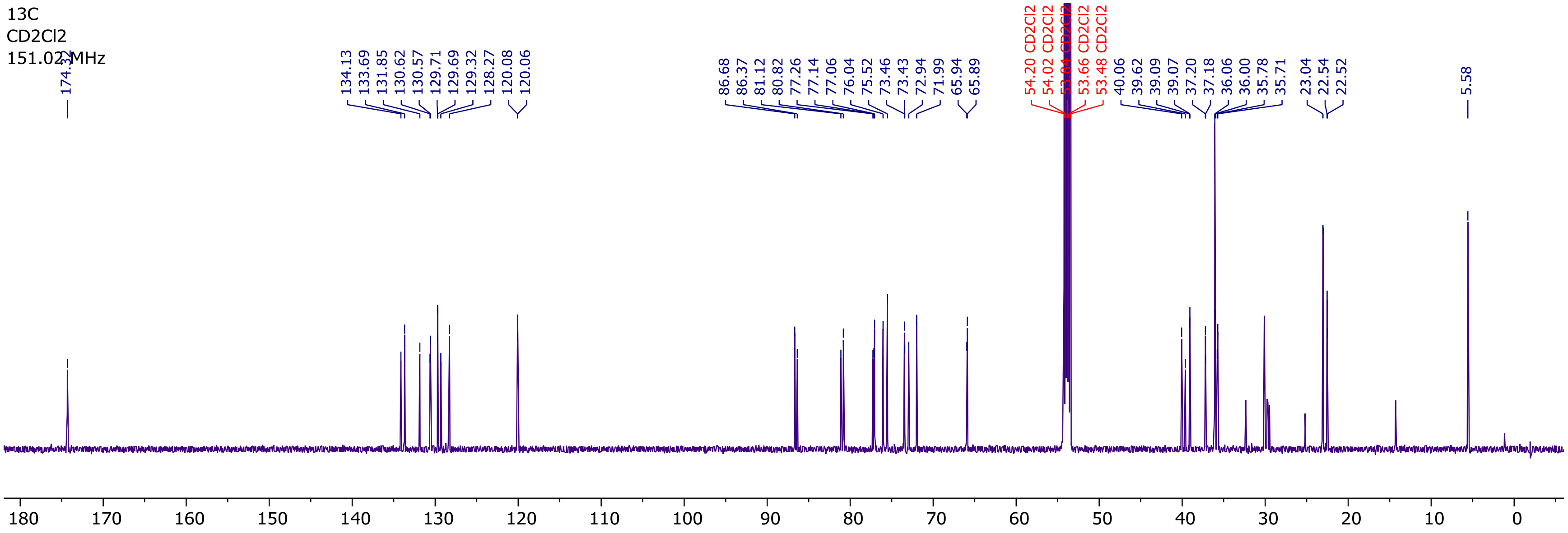

Reaction run in a capped vial under ambient atmosphere. To a rt, stirred solution of **S76** (18 mg, 0.032 mmol, 1.0 eq.) in $CH_2Cl_2$ (1 mL) was added $NaHCO_3$ (16.2 mg, 0.193 mmol, 6.0 eq.) followed by Dess-Martin Periodinane (17.7 mg, 0.0417 mmol, 1.3 eq.). The mixture was stirred at rt for 1 h 25 min. After this time, starting material was consumed as monitored by TLC analysis. The reaction mixture was quenched with a 1:1:1 (v/v/v) $H_2O$-saturated aqueous $NaHCO_3$-10% aqueous $Na_2S_2O_3$ (1 mL) and the biphasic mixture was stirred vigorously for 30 min at rt. The aqueous layer was extracted with $CH_2Cl_2$ (5x 10 mL), and the combined organic layers were dried ($Na_2SO_4$), filtered, and the solvent was removed in vacuo. The crude product was passaged through a short plug of silica gel eluting with $Et_2O$. Solvent was removed in vacuo to yield the crude aldehyde **S77** (13.1 mg) as a colorless oil which was used immediately in the next step without further purification.

To a solution of **S77** (13 mg, 0.023 mmol, 1.0 eq.) in deoxygenated (5x freeze-pump-thaw cycles) DMSO (0.6 mL) was added $CrCl_2$ doped with 1 % $NiCl_2$ (w/w) (28.3 mg, 0.23 mmol, 10 eq.). **3a** (11 mg, 0.23 mmol, 10 eq.) was added in via syringe in deoxygenated DMSO (0.6 mL) and the mixture was stirred for 22 h 45 min. After this time, the reaction mixture was transferred to a separatory funnel, diluted with $Et_2O$ (15 mL) and 10 mL of 1:1 (v/v) $H_2O$-brine was added. The organic layer was separated, and the aqueous layer extracted with $Et_2O$ (7x 15 mL). The combined organic layers were dried ($MgSO_4$), filtered, and solvent was removed in vacuo. The crude product was purified via flash column chromatography (9:1 to 4:1 Hexanes/Acetone). Appropriate fractions were pooled, and solvent was removed in vacuo to yield **S79** (3.3 mg) as a colorless oil and a 1:1 mixture of diastereomers. The product was used immediately in the subsequent steps.

Reaction run under ambient atmosphere in a capped vial. To a rt, vigorously stirred solution of **S79** (3.3 mg, 0.0049 mmol, 1.0 eq.) in $CH_2Cl_2$ (0.5 mL) was added in sequence pH 7 phosphate buffer (0.2 mL) and 2,3-dichloro-5,6-dicyano-benzoquinone (1.3 mg, 0.0057 mmol, 1.2 eq.). After 20 h additional 2,3-dichloro-5,6-dicyano-benzoquinone (1.2 mg) was added. After a further 3 h 30 min, additional 2,3-dichloro-5,6-dicyano-benzoquinone (3.0 mg) was added. After a further 20 min, additional 2,3-dichloro-5,6-dicyano-benzoquinone (1.2 mg) was added. After a further 20 min, starting material was consumed as monitored by TLC analysis. The reaction mixture was pipetted onto saturated aqueous $NaHCO_3$ (2 mL). The aqueous layer was extracted with $CH_2Cl_2$ (5x 2 mL) and the combined organic layers were dried ($Na_2SO_4$), filtered, and solvent was removed in vacuo. The crude product was purified via flash column chromatography (11:9 Hexanes/Acetone). Appropriate fractions were pooled, and solvent was removed in vacuo to yield the alcohol (2.5 mg) as a white amorphous solid which was used immediately in the subsequent step.

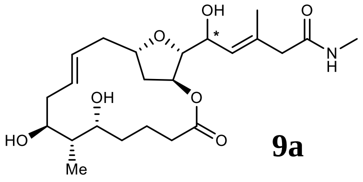

**9a**

* 1:1 mixture of diastereomers

Reaction run under ambient atmosphere in a capped polypropylene vial. To a rt, stirred solution of the alcohol from the previous step (2.5 mg, 0.0045 mmol, 1.0 eq.) in THF (0.5 mL), was added in sequence pyridine (0.03 mL), and HF-pyridine (70% (w/w) HF, 15 µL, 0.58 mmol HF, 129 eq. HF). After 2 d, conversion was less than 50% by TLC and additional HF-pyridine was added (70% (w/w) HF, 10 uL, 0.39 mmol HF, 86 eq. HF). The reaction mixture was stirred at rt for a further 2 d. After this time, starting material was consumed as monitored by TLC analysis, and the reaction mixture was quenched by pipetting it onto a saturated aqueous solution of $NaHCO_3$ (5 mL) [**caution!** $CO_2$(g) evolved]. After the evolution of gas had ceased, aqueous layer was extracted with $CH_2Cl_2$ (5x 5 mL). The combined organic layers were dried ($Na_2SO_4$), filtered, and solvent was removed in vacuo. The crude product was purified via flash column chromatography (19:1 to 4:1 $CH_2Cl_2$/MeOH). Appropriate fractions were pooled, and solvent was removed in vacuo to yield **9a** (1.8 mg, 5.5%, 9 steps from **2b**) as a white solid.

**Analytical Data for 9a:**

$R_f$ = 0.40 (87.5:12.5 $CH_2Cl_2$/MeOH)

$^1$H NMR (601 MHz, $CD_3CN$) δ 6.36 (s, 0.5H), 6.25 (s, 0.5H), 5.57 – 5.46 (m, 2H), 5.25 (ddt, *J* = 8.7, 7.4, 1.4 Hz, 1H), 5.09 (d, *J* = 4.6 Hz, 0.5H), 4.98 (d, *J* = 4.7 Hz, 0.5H), 4.31 (dt, *J* = 9.5, 5.1 Hz, 0.5H), 4.23 (ddd, *J* = 8.7, 6.0, 4.3 Hz, 0.5H), 3.89 (dd, *J* = 5.0, 1.1 Hz, 0.5H), 3.79 (dd, *J* = 6.0, 1.1 Hz, 0.5H), 3.69 (tt, *J* = 11.1, 4.0 Hz, 1H), 3.37 – 3.29 (m, 2H), 3.03 (d, *J* = 4.5 Hz, 0.5H), 2.94 (d, *J* = 5.2 Hz, 0.5H), 2.89 – 2.77 (m, 2H), 2.74 (dd, *J* = 4.8, 1.5 Hz, 1H), 2.67 (dd, *J* = 5.2, 1.4 Hz, 1H), 2.64 (t, *J* = 4.6 Hz, 3H), 2.52 – 2.46 (m, 1H), 2.31 – 2.18 (m, 2.5H), 2.12 – 2.06 (m, 2H), 1.93 – 1.87 (m, 2H), 1.78 (ddd, *J* = 15.6, 9.9, 5.3 Hz, 1H), 1.69 (t, *J* = 1.5 Hz, 3H), 1.68 – 1.60 (m, 1H), 1.59 – 1.54 (m, 1H), 1.50 – 1.40 (m, 2H), 1.27 – 1.23 (m, 1H), 0.83 (dd, *J* = 7.0, 1.4 Hz, 3H).

$^{13}$C NMR (151 MHz, $CD_3CN$) δ 174.84, 174.81, 171.45, 171.44, 135.69, 135.46, 132.44, 131.36, 131.26, 130.06, 129.88, 129.51, 128.54, 87.48, 87.27, 81.68, 81.41, 77.56, 77.44, 76.21, 76.18, 73.89, 73.68, 69.11, 47.73, 47.72, 40.60, 40.57, 39.62, 39.26, 37.85, 36.34, 36.33, 36.14, 36.12, 36.11, 36.10, 26.33, 26.28, 23.45, 17.44, 17.37, 7.34, 7.29.

HRMS (ESI): Anal. Calcd. for $C_{23}H_{38}NO_7^+$ $[M+H]^+$ 440.2643, found 440.2654

IR (neat): $\nu_{max}$ $(cm^{-1})$ = 3358 (br, OH), 2985 (w, C=*CH*), 2932 (s, CH), 2866 (s, CH), 1720 (s, C=O), 1644 (s, C=C), 1543 (m), 1430 (m), 1423 (m), 1341 (m), 1260 (m).

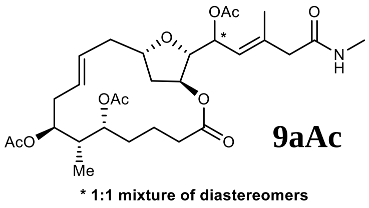


**9aAc**

**9aAc** was prepared according to general procedure A (0.16 mg, quant.)

**Analytical Data for 9aAc:**

$R_f$ = 0.35 (EtOAc)

HRMS (ESI): Anal. Calcd. for $C_{29}H_{44}NO_{10}^+$ $[M+H]^+$ 566.2960, found 566.2943

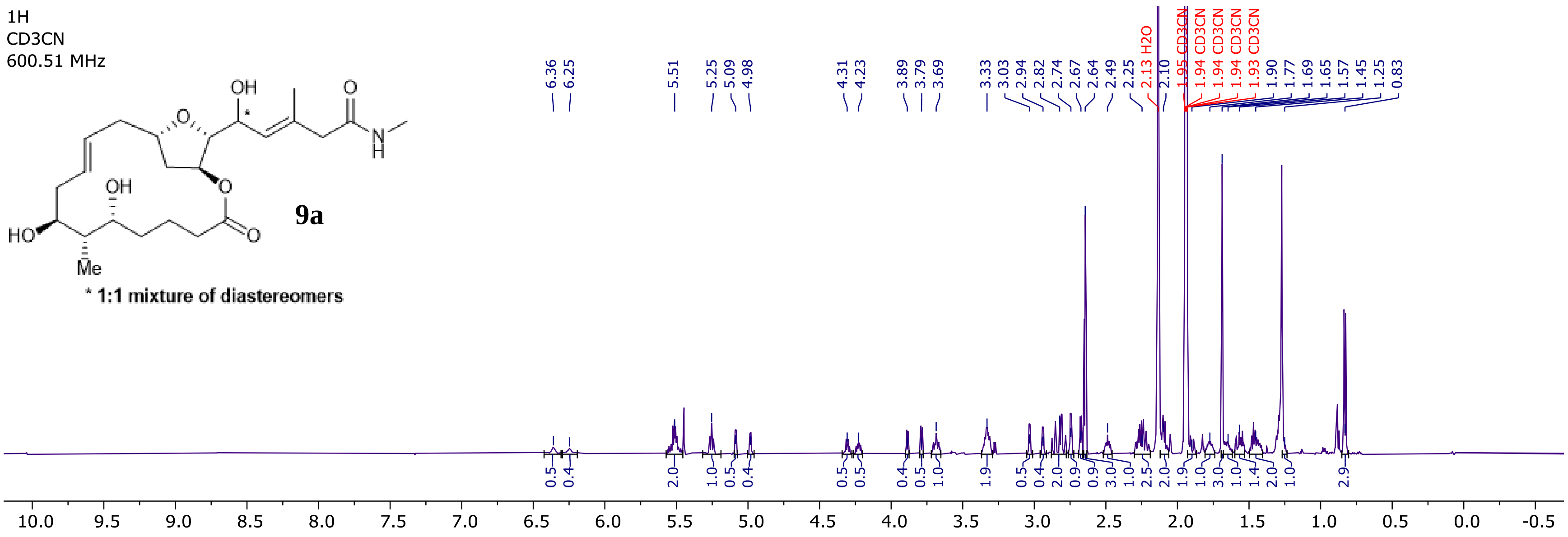


**9a**

* 1:1 mixture of diastereomers

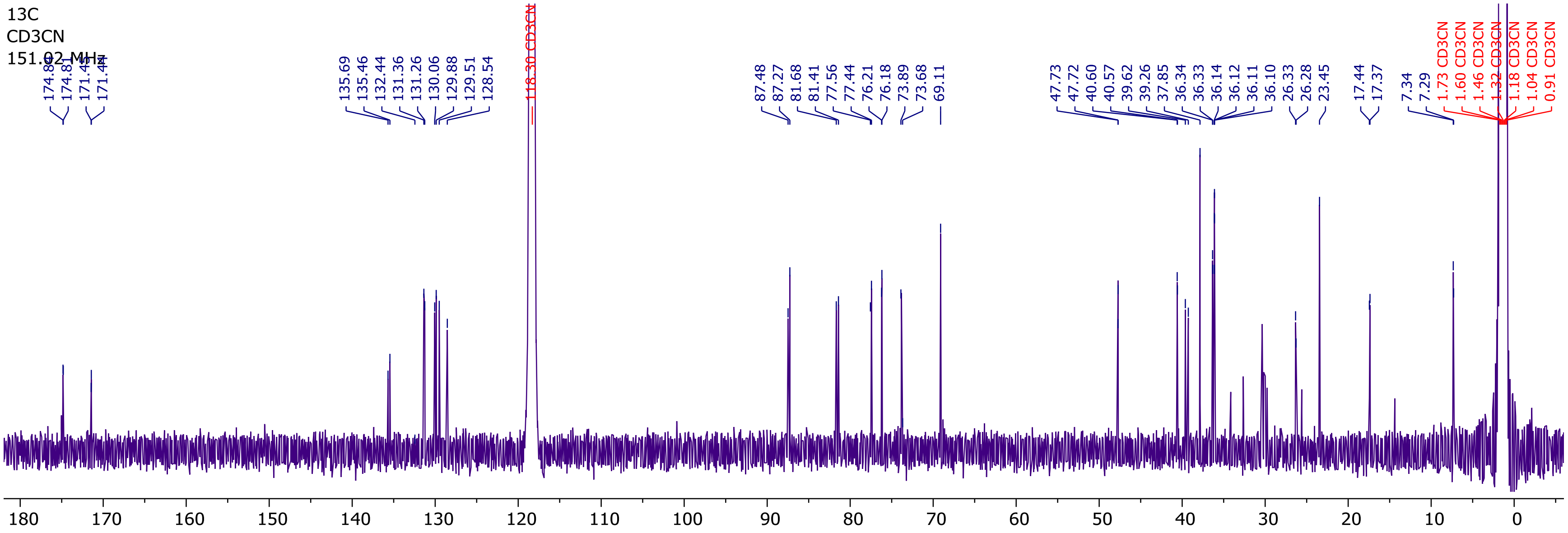

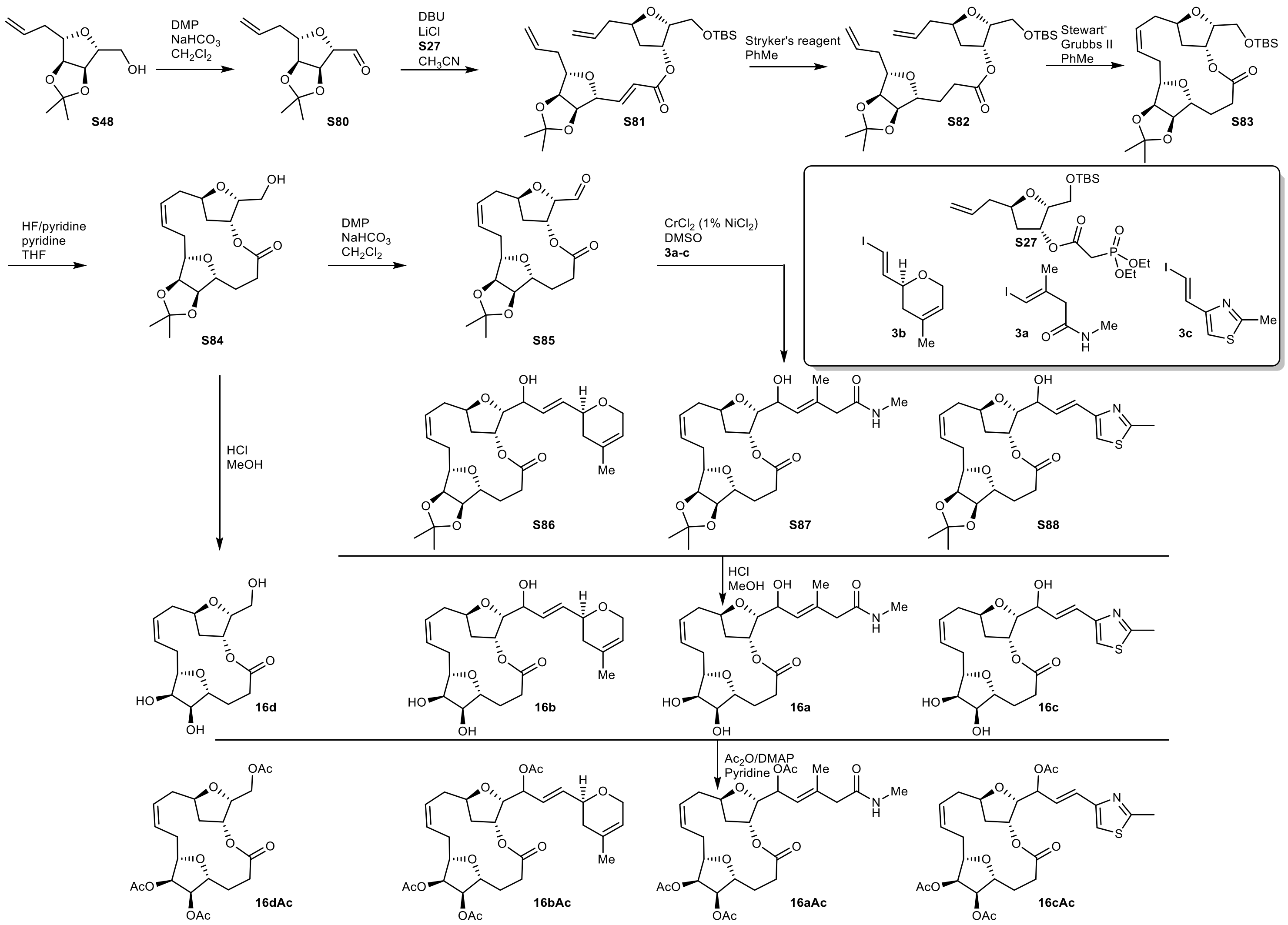


**Supplementary Fig. 42 | Synthesis of pMLs 16a-d and 16aAc-dAc.**
Abbreviations: DBU = 1,8-Diazabicyclo[5.4.0]undec-7-ene, THF = tetrahydrofuran, DMSO = dimethylsulfoxide, Stewart-Grubbs II = Dichloro[1,3-bis(2-methylphenyl)-2-imidazolidinylidene](2-isopropoxyphenylmethylene)ruthenium(II), DMP = Dess-Martin periodinane, DMAP = 4-dimethylaminopyridine, TBS = tert-butyldimethylsilyl.

Reaction run in a capped vial under ambient atmosphere. To a rt, stirred solution of **S48** (144.3 mg, 0.673 mmol, 1.0 eq.) in $CH_2Cl_2$ (4 mL) was added $NaHCO_3$ (163.8 mg, 1.95 mmol, 2.9 eq.) followed by Dess-Martin Periodinane (335.5 mg, 0.78 mmol, 1.2 eq.). The mixture was stirred at rt for 1 h 20 min. After this time, starting material was consumed as monitored by TLC analysis. The reaction mixture was quenched with a 1:1 (v/v) saturated aqueous $NaHCO_3$-10% aqueous $Na_2S_2O_3$ (4 mL) and the biphasic mixture was stirred vigorously for 40 min at rt. The reaction mixture was then poured into a separatory funnel and the aqueous layer was extracted with $CH_2Cl_2$ (3x 5 mL). The combined organic layers were washed with brine (1x 10 mL), dried ($MgSO_4$), filtered, and the solvent was removed in vacuo. The crude product was passaged through a short plug of C2 modified silica gel eluting with $Et_2O$. Solvent was removed in vacuo to yield the crude aldehyde **S80** (113 mg, 53%) as a colorless oil which was used immediately in the next step without further purification.

To a cold (-4 °C), stirred suspension of LiCl (36.7 mg, 0.866 mmol, 1.74 eq., flame dried in vacuo), phosphonate **S27** (226.7 mg, 0.50 mmol, 1.0 eq.), and aldehyde **S80** (113 mg, 0.53 mmol, 1.06 eq.) in MeCN (5 mL) was added DBU (77 µL, 78 mg, 0.51 mmol, 1.0 eq.) via microsyringe. The reaction mixture was allowed to slowly warm to room temperature over the course of 1 h 15 min. After this time, starting material was consumed as monitored by TLC analysis, and the reaction mixture was quenched by addition of a saturated aqueous solution of $NH_4Cl$ (5 mL). The biphasic mixture was poured into a separatory funnel and extracted with $CH_2Cl_2$ (3x 5 mL). The combined organic layers were dried ($Na_2SO_4$), filtered, and solvent was removed in vacuo. The crude product was purified via flash column chromatography (4:1 Hexanes:$Et_2O$). Appropriate fractions were pooled, and solvent was removed in vacuo to yield **S81** (143.5 mg, 56%) as a colorless oil which was combined with a separate batch of material and used in the subsequent step.

Note: toluene was deoxygenated before usage by sparging with $N_2$(g) for 30 min

To a rt, stirred suspension of $Cu(OAc)_2 \cdot H_2O$ (125 mg, 0.63 mmol) and $PPh_3$ (325 mg, 1.24 mmol) in deoxygenated toluene (30 min $N_2$(g) sparge) (23.5 mL) was added 1,1,3,3-tetramethyldisiloxane (1.65 mL, 1.25 g, 9.31 mmol) via syringe. The teal-colored suspension was allowed to stir for 24 h. After this time the suspension turned into a homogenous brick-red solution, indicating the presence of $[(PPh_3)CuH]_6$. To **S81** (175 mg, 0.344 mmol, 1.0 eq.) in toluene (5.2 mL) was added a solution of the previously prepared reagent $[(PPh_3)CuH]_6$ (1.75 mL, *ca.* 0.4 M in organosilane, 0.70 mmol, 2.0 eq.). After stirring at rt for 25 h, the reaction mixture was poured onto a silica gel column and the products eluted with $Et_2O$. The solvent was removed in vacuo, and $^1H$ NMR analysis of the crude indicated complete conversion. The crude product was purified via flash column chromatography (19:1 to 17:3 Hexanes/$Et_2O$). Appropriate fractions were pooled, and solvent was removed in vacuo to yield **S82** (117 mg, 67%) as a pale-yellow oil which was used in the subsequent step.

Reaction was run in two equal sized batches. The procedure shown is for a single batch, and the yield reported is for the combined batches. To a hot (110 °C), stirred solution of **S82** (47 mg, 0.092 mmol, 1.0 eq.) in deoxygenated toluene (30 min $N_2$(g) sparge) (194

mL) that was continuously being sparged with $N_2$(g) was added Stewart-Grubbs second generation catalyst (7.9 mg, 0.014 mmol, 0.15 eq.) in toluene (0.25 mL). After exactly 5 min, further Stewart-Grubbs second generation catalyst (7.9 mg, 0.014 mmol, 0.15 eq.) was added in toluene (0.25 mL). After exactly another 5 min., further Stewart-Grubbs second generation catalyst (7.9 mg, 0.014 mmol, 0.15 eq.) in toluene (0.25 mL). After exactly another 5 min., further Stewart-Grubbs second generation catalyst (7.9 mg, 0.014 mmol, 0.15 eq.) in toluene (0.25 mL). After exactly another 5 min., starting material was consumed as monitored by TLC analysis. The reaction mixture was promptly cooled to 0 °C, and the reaction mixture quenched by addition of potassium 2-isocyanoacetate (50.9 mg, 0.42 mmol, 4.6 eq.) in MeOH (5 mL). The reaction mixture was stirred for a further 35 min, after which time the green solution changed to a pale-yellow color. The reaction mixture was concentrated in vacuo to give a crude solid deposited on the walls of the evaporation flask. The walls of the flask were scraped with a spatula and the solid was triturated with 7:3 Hexanes/$Et_2O$ (5x 4 mL) and filtered through a short plug of silica gel eluting with 7:3 Hexanes/$Et_2O$. The filtrate was concentrated in vacuo and the crude product was combined with the second equal batch of material and the combined batches were purified via flash column chromatography (9:1 Hexanes/$Et_2O$). Appropriate fractions were pooled, and solvent was removed in vacuo to yield **S83** (40 mg, 44%) as a white foam. This material was then combined with a previous batch of material which arose from evaluation reactions.

An approximately 3.9 M solution of HF in THF/Pyridine was prepared as follows. HF-Pyridine (70% (w/w), 1 mL, 0.77 g HF, 38.5 mmol HF) was slowly added to a cold (0 °C) stirred solution of THF (5 mL) and pyridine (4 mL) in a polypropylene falcon tube.

Reaction run under ambient atmosphere in a capped polypropylene vial. To a rt, stirred solution of **S83** (46 mg, 0.095 mmol, 1.0 eq.) in THF (2.7 mL) was added the previously mixed HF/Pyridine/THF solution (3.8 mL, 3.9 M HF, 15 mmol HF, 160 eq. HF). The reaction mixture was stirred for 3 h at rt. After this time starting material was consumed as monitored by TLC analysis. The reaction mixture was slowly pipetted onto saturated aqueous $NaHCO_3$ (30 mL) [**caution!** $CO_2$ (g) liberated]. After evolution of gas had ceased, the aqueous layer was extracted with $CH_2Cl_2$ (3x 20 mL). The combined organic layers were dried ($Na_2SO_4$), filtered, and the solvent was removed in vacuo. The crude product was purified via flash column chromatography (3:1 Hexanes/Acetone). Appropriate fractions were pooled, and solvent was removed in vacuo to yield **S84** (29 mg, 83%) as a white amorphous solid.

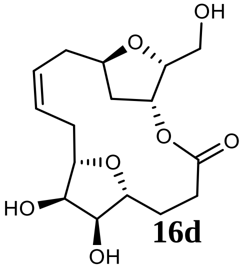

**16d**

An approximately 1.2 M solution of aqueous HCl in MeOH was prepared by slowly adding concentrated HCl (1 mL, *ca.* 12 M) to MeOH (9 mL). To a cold (0 °C), stirred solution of **S84** (1.8 mg, 0.0049 mmol, 1.0 eq.) in $CH_2Cl_2$ (0.25 mL) and MeOH (0.25 mL) was added the previously prepared solution of aqueous HCl in MeOH (20 µL, *ca.* 1.2M in MeOH, 0.024 mmol, 5.0 eq.). The reaction vessel was moved to a fridge (4 °C) and allowed to stir in the fridge for 26 h. After this time, starting material was consumed as monitored by TLC analysis, and the reaction mixture was quenched by addition of $NaHCO_3$ (30 mg, 0.36 mmol, 73 eq.) and allowed to stir vigorously for 5 min. After this time, silica gel was added and the reaction mixture was concentrated onto the

silica gel in vacuo. The crude product was then purified via flash column chromatography (19:1 to 92.5:7.5 $CH_2Cl_2$/MeOH). Appropriate fractions were pooled, and solvent was removed in vacuo to yield **16d** (1.2 mg, 10%, 5 steps from **S27**, 75% 1 step) as a white film.

**Analytical Data for 16d:**

$R_f$ = 0.21 (93:7 $CH_2Cl_2$/MeOH)

$[\alpha]_D^{20}$ = -130° (c = 0.12, MeOH)

$^1$H NMR (601 MHz, MeOD) δ 5.56 (dddd, *J* = 10.6, 8.7, 6.8, 2.0 Hz, 1H), 5.45 (tdd, *J* = 11.4, 4.6, 2.0 Hz, 1H), 5.04 (t, *J* = 3.4 Hz, 1H), 4.60 (tdd, *J* = 11.4, 6.0, 3.5 Hz, 1H), 4.19 (td, *J* = 6.6, 3.6 Hz, 1H), 3.76 – 3.68 (m, 2H), 3.62 – 3.57 (m, 2H), 3.56 – 3.51 (m, 1H), 3.48 (ddd, *J* = 9.0, 7.0, 1.8 Hz, 1H), 2.73 (ddd, *J* = 15.7, 7.2, 2.2 Hz, 1H), 2.71 – 2.67 (m, 1H), 2.42 – 2.32 (m, 2H), 2.29 (ddt, *J* = 12.5, 4.7, 1.9 Hz, 1H), 2.23 (ddd, *J* = 15.8, 12.0, 2.4 Hz, 1H), 2.05 (ddt, *J* = 14.9, 7.2, 2.2 Hz, 1H), 1.98 (dddd, *J* = 14.8, 11.8, 9.5, 2.3 Hz, 1H), 1.84 (ddd, *J* = 12.4, 11.1, 8.4 Hz, 1H), 1.50 (ddd, *J* = 13.0, 11.8, 3.3 Hz, 1H).

$^{13}$C NMR (151 MHz, MeOD) δ 174.04, 132.05, 126.24, 86.30, 85.94, 83.10, 79.19, 77.15, 76.24, 75.94, 61.61, 38.78, 35.52, 34.93, 31.00, 30.45.

HRMS (ESI): Anal. Calcd. for $C_{16}H_{25}O_7^+$ $[M+H]^+$ 329.1595, found 329.1600

IR (neat): $\nu_{max}$ $(cm^{-1})$ = 3396 (br, OH), 3016 (w, C=*CH*), 2929 (m, CH), 2875 (m, CH), 1721 (s, C=O), 1654 (m, C=C), 1432 (m), 1343 (m), 1172 (m)

**16dAc**

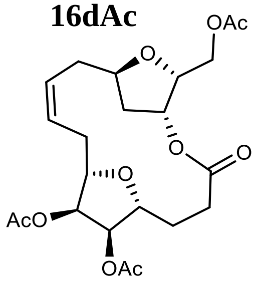


**16dAc** was prepared according to general procedure A (0.51 mg, 95%)

**Analytical Data for 16dAc:**

$R_f$ = 0.55 (2:3 Hexanes/EtOAc)

HRMS (ESI): Anal. Calcd. for $C_{22}H_{34}NO_{10}^+$ $[M+NH_4]^+$ 472.2177, found 472.2166

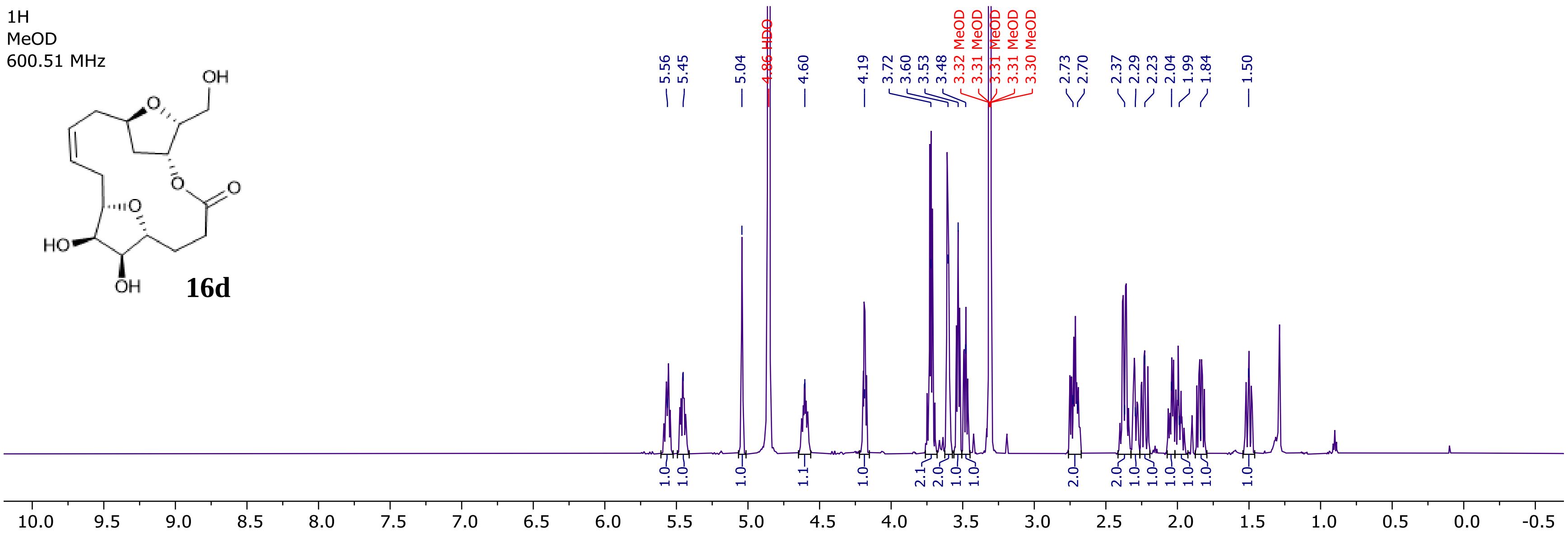
1H
MeOD
600.51 MHz
OH
O
O
O
O
HO
OH
5.56
5.45
5.04
4.86 HDO
4.60
4.19
3.72
3.60
3.53
3.48
3.32 MeOD
3.31 MeOD
3.31 MeOD
3.31 MeOD
3.30 MeOD
2.73
2.70
2.37
2.29
2.23
2.04
1.99
1.84
1.50
1.0
1.0
1.0
1.1
1.0
2.1
2.0
1.0
1.0
2.0
2.0
1.0
1.0
1.0
1.0
1.0
1.0
10.0 9.5 9.0 8.5 8.0 7.5 7.0 6.5 6.0 5.5 5.0 4.5 4.0 3.5 3.0 2.5 2.0 1.5 1.0 0.5 0.0 -0.5

**16d**

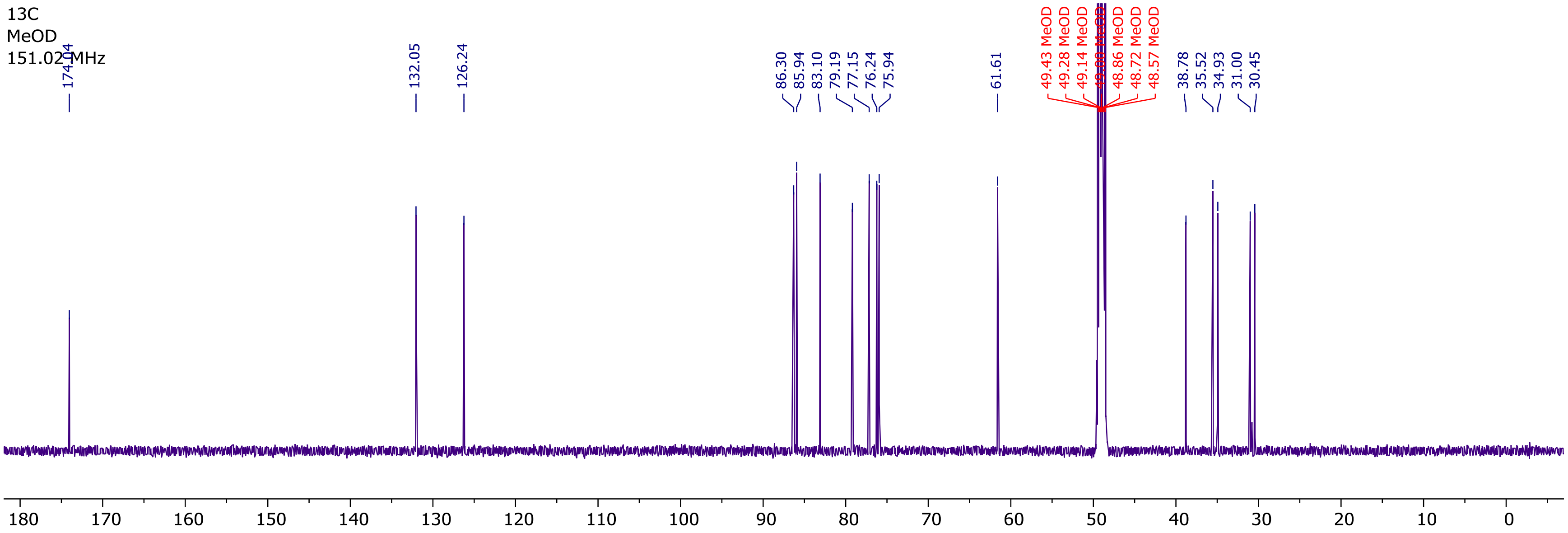
13C
MeOD
151.02 MHz
174.04
132.05
126.24
86.30
85.94
83.10
79.19
77.15
76.24
75.94
61.61
49.43 MeOD
49.28 MeOD
49.14 MeOD
49.00 MeOD
48.86 MeOD
48.72 MeOD
48.57 MeOD
38.78
35.52
34.93
31.00
30.45
180 170 160 150 140 130 120 110 100 90 80 70 60 50 40 30 20 10 0

Reaction run in a capped vial under ambient atmosphere. To a rt, stirred solution of **S84** (16 mg, 0.043 mmol, 1.0 eq.) in $CH_2Cl_2$ (1.2 mL) was added $NaHCO_3$ (10.8 mg, 0.129 mmol, 3.0 eq.) followed by Dess-Martin Periodinane (22.1 mg, 0.0521 mmol, 1.2 eq.). The mixture was stirred at rt for 1 h 20 min. After this time, further Dess-Martin Periodinane (2.5 mg, 0.0059 mmol, 0.14 eq.) was added and the reaction mixture stirred a further 10 min. After this time, starting material was consumed as monitored by TLC analysis. The reaction mixture was quenched with a 1:1 (v/v) saturated aqueous $NaHCO_3$-10% aqueous $Na_2S_2O_3$ (1 mL) and the biphasic mixture was stirred vigorously for 30 min at rt. The aqueous layer was extracted with $CH_2Cl_2$ (3x 10 mL), and the combined organic layers were dried ($Na_2SO_4$), filtered, and the solvent was removed in vacuo. The crude product was passaged through a short plug of C2 modified silica gel eluting with $Et_2O$. Solvent was removed in vacuo to yield the crude aldehyde **S85** (*ca.* 16 mg) as a colorless oil which was used immediately in the next steps without further purification.

To a solution of **S85** (8.0 mg, 0.022 mmol, 1.0 eq.) in deoxygenated (5x freeze-pump-thaw cycles) DMSO (0.5 mL) was added $CrCl_2$ doped with 1 % $NiCl_2$ (w/w) (30.2 mg, 0.309 mmol, 14 eq.). **3b** (12.5 mg, 0.050 mmol, 2.3 eq.) was added via syringe in deoxygenated DMSO (0.5 mL + 0.2 mL rinse) and the mixture was stirred for 12 h. After this time, the reaction mixture was transferred to a separatory funnel, diluted with $Et_2O$ (10 mL) and 15 mL of 1:1 (v/v) $H_2O$-brine was added. The organic layer was separated, and the aqueous layer extracted with $Et_2O$ (7x 15 mL). The combined organic layers were dried ($MgSO_4$), filtered, and solvent was removed in vacuo. The crude product was purified via flash column chromatography (4:1 to Hexanes/Acetone). Appropriate fractions were pooled, and solvent was removed in vacuo to yield **S86** (4.6 mg) as a colorless oil and a 4:1 mixture of diastereomers. The product was used immediately in the subsequent step.

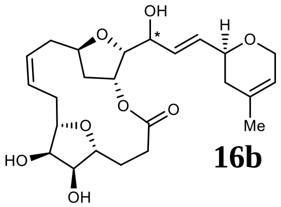


**16b**

* 4:1 mixture of diastereomers

An approximately 1.2 M solution of aqueous HCl in MeOH was prepared by slowly adding concentrated HCl (1 mL, *ca.* 12 M) to MeOH (9 mL). To a cold (0 °C), stirred solution of **S86** (4.6 mg, 0.0093 mmol, 1.0 eq.) in MeOH (2.5 mL) was added the previously prepared solution of aqueous HCl in MeOH (40 µL, *ca.* 1.2M in MeOH, 0.048 mmol, 5.2 eq.) via microsyringe. The reaction vessel was moved to a fridge (4 °C) and allowed to stir in the fridge for 28 h. After this time, starting material was consumed as monitored by TLC analysis, and the reaction mixture was quenched by addition of $NaHCO_3$ (10 mg, 0.12 mmol, 12.9 eq.) and allowed to stir vigorously for 5 min. After this time, reaction mixture was filtered and solvent was removed in vacuo. The crude product was then purified via flash column chromatography (97:3 to 19:1 $CH_2Cl_2$/MeOH). Appropriate fractions were pooled, and solvent was removed in vacuo to yield **16b** (3.6 mg, 5.0%, 7 steps from **S27**) as a white amorphous solid.

**Analytical Data for 16b:**

$R_f$ = 0.53 (9:1 $CH_2Cl_2$/MeOH)

$^1$H NMR (601 MHz, $CDCl_3$) δ 5.99 – 5.94 (m, 0.8H), 5.94 – 5.89 (m, 1.1H), 5.68 (ddd, *J* = 15.7, 5.7, 1.5 Hz, 0.2H), 5.56 (dddd, *J* = 10.6, 8.6, 7.0, 1.9 Hz, 1.1H), 5.48 – 5.38 (m, 2.1H), 5.15 (t, *J* = 3.3 Hz, 0.8H), 4.99 (t, *J* = 3.4 Hz, 0.2H), 4.60 (tdd, *J* = 11.5, 5.9, 3.4 Hz, 1H), 4.49 – 4.46 (m, 0.2H), 4.45 – 4.38 (m, 0.8H), 4.22 – 4.13 (m, 2.2H), 4.06 (dddd, *J* = 8.3, 4.6, 3.6, 1.1 Hz, 0.8H), 4.00 (dt, *J* = 9.7, 4.3 Hz, 0.2H), 3.97 (dd, *J* = 7.8, 3.5 Hz, 0.8H), 3.92 (dd, *J* = 8.5, 3.6 Hz, 0.2H), 3.71 (p, *J* = 4.7 Hz, 1.1H), 3.65 – 3.56 (m, 2H), 3.49 (ddd, *J* = 9.1, 6.8, 1.8 Hz, 1H), 2.79 – 2.63 (m, 3.6H), 2.46 – 2.37 (m, 2.1H), 2.37 – 2.27 (m, 2.1H), 2.14 – 2.05 (m, 2.5H), 2.01 (dddd, *J* = 14.7, 12.0, 9.5, 2.3 Hz, 1.4H), 1.95 – 1.90 (m, 1H), 1.86 (ddd, *J* = 12.4, 11.1, 8.4 Hz, 1.1H), 1.72 – 1.70 (m, 2.6H), 1.70 – 1.68 (m, 0.7H), 1.55 – 1.43 (m, 1H).

$^{13}$C NMR (151 MHz, $CDCl_3$) δ 171.94, 171.90, 133.59, 132.26, 131.56, 131.47, 131.02, 130.49, 130.43, 128.01, 125.77, 125.70, 119.77, 119.74, 85.11, 85.01, 84.88, 84.84, 84.79, 83.67, 78.65, 78.26, 76.63, 75.95, 74.75, 74.53, 74.51, 73.39, 73.20, 71.31, 70.42, 65.86, 65.75, 38.50, 37.96, 35.76, 35.67, 34.57, 34.54, 33.83, 33.79, 30.31, 30.09, 29.27, 29.15, 23.12, 23.08.

HRMS (ESI): Anal. Calcd. for $C_{24}H_{35}O_8^+$ $[M+H]^+$ 451.2327, found 451.2327

IR (neat): $\nu_{max}$ $(cm^{-1})$ = 3401 (br, OH), 3015 (w, C=C*H*), 2922 (m, CH), 1722 (s, CO), 1658 (m, C=C), 1435 (m), 1361 (m), 1259 (s), 1170 (m)

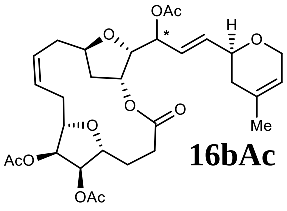


**16bAc**

* 4:1 mixture of diastereomers

**16bAc** was prepared according to general procedure A (1.4 mg, 90%)

**Analytical Data for 16bAc:**

$R_f$ = 0.58 (2:3 Hexanes/EtOAc)

HRMS (ESI): Anal. Calcd. for $C_{30}H_{44}NO_{11}^+$ $[M+NH_4]^+$ 594.2909, found 594.2861

IR (neat): $\nu_{max}$ $(cm^{-1})$ = 3017 (w, C=C*H*), 2961 (m, CH), 2927 (m, CH), 1741 (vs, CO), 1647 (m, C=C), 1435 (m), 1374 (m), 1247 (vs).

$^1$H NMR (601 MHz, $C_6D_6$) δ 6.32 (ddd, *J* = 15.8, 6.1, 1.5 Hz, 0.8H), 6.19 (ddd, *J* = 15.7, 4.8, 1.2 Hz, 0.8H), 6.12 – 6.07 (m, 0.4H), 6.04 (ddt, *J* = 8.2, 6.0, 1.1 Hz, 0.8H), 5.93 (ddd, *J* = 15.7, 6.9, 1.6 Hz, 0.2H), 5.43 – 5.36 (m, 1H), 5.21 – 5.15 (m, 1H), 5.15 – 5.09 (m, 1.8H), 5.02 – 4.97 (m, 0.2H), 4.74 – 4.63 (m, 3H), 4.20 – 4.08 (m, 2H), 4.04 – 3.97 (m, 1H), 3.96 – 3.89 (m, 1H), 3.67 – 3.56 (m, 2H), 2.86 – 2.76 (m, 1H), 2.59 – 2.51 (m, 1H), 2.49 – 2.43 (m, 0.8H), 2.34 – 2.26 (m, 0.2H), 2.13 – 1.92 (m, 5H), 1.89 – 1.81 (m, 1H), 1.79

(s, 0.6H), 1.73 – 1.68 (m, 1H), 1.67 (s, 2.4H), 1.65 (s, 3H), 1.63 (s, 0.6H), 1.63 (s, 2.4H), 1.57 (dt, *J* = 16.4, 3.3 Hz, 1H), 1.48 (br s, 1H), 1.42 (br s, 2.4H), 1.25 – 1.17 (m, 1H).

$^{13}$C NMR (151 MHz, $C_6D_6$, **major diastereomer**) δ 171.02, 169.17, 169.11, 168.78, 134.91, 131.28, 130.15, 127.26, 126.24, 120.34, 82.96, 82.62, 82.58, 78.64, 75.73, 75.05, 74.61, 73.29, 71.56, 65.78, 38.27, 36.02, 34.61, 33.47, 30.70, 29.45, 22.86, 20.62, 20.13, 20.10.

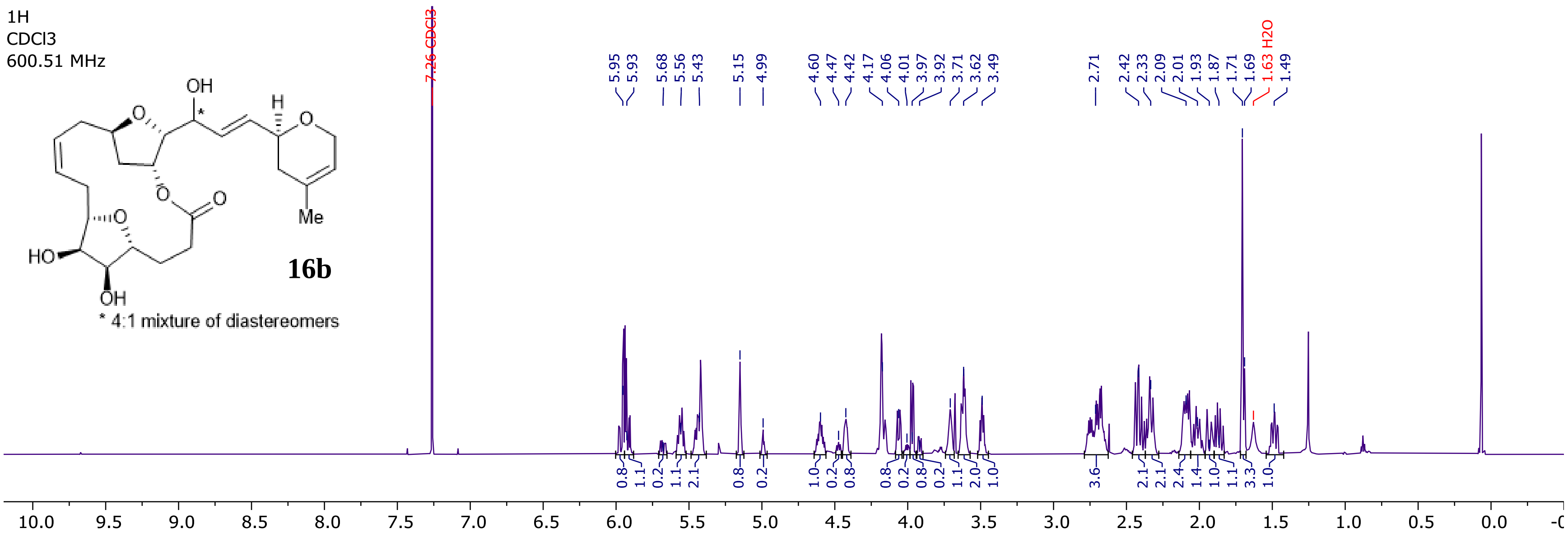
1H
CDCl3
600.51 MHz
7.26 CDCl3
5.95
5.93
5.68
5.56
5.43
5.15
4.99
4.60
4.47
4.42
4.17
4.06
4.01
3.97
3.92
3.71
3.62
3.49
2.71
2.42
2.33
2.09
2.01
1.93
1.87
1.71
1.69
1.63 H2O
1.49
0.8
1.1
0.2
1.1
2.1
0.8
0.2
1.0
0.2
0.8
0.8
0.2
0.8
0.2
1.1
2.0
1.0
3.6
2.1
2.1
2.4
1.4
1.0
1.1
3.3
1.0
OH
H
O
O
O
O
O
HO
OH
Me
16b
* 4:1 mixture of diastereomers
10.0
9.5
9.0
8.5
8.0
7.5
7.0
6.5
6.0
5.5
5.0
4.5
4.0
3.5
3.0
2.5
2.0
1.5
1.0
0.5
0.0

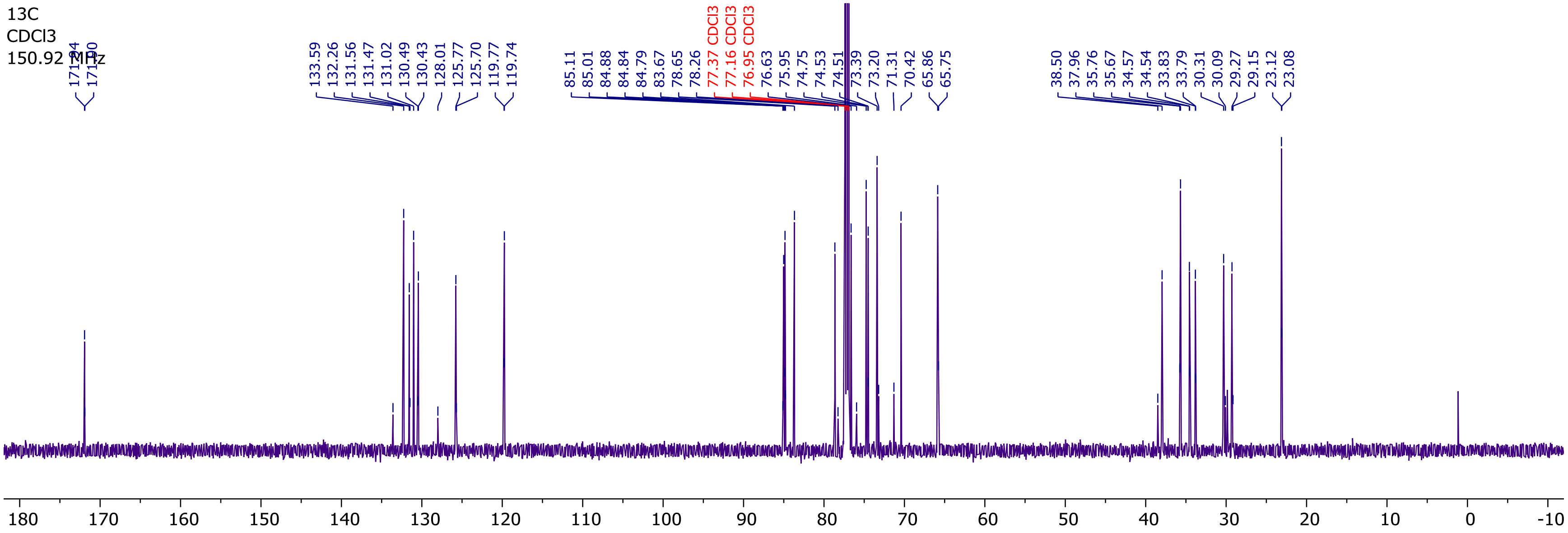
13C
CDCl3
150.92 MHz
171.94
171.90
133.59
132.26
131.56
131.47
131.02
130.49
130.43
128.01
125.77
125.70
119.77
119.74
85.11
85.01
84.88
84.84
84.79
83.67
78.65
78.26
77.37 CDCl3
77.16 CDCl3
76.95 CDCl3
76.63
75.95
74.75
74.53
74.51
73.39
73.20
71.31
70.42
65.86
65.75
38.50
37.96
35.76
35.67
34.57
34.54
33.83
33.79
30.31
30.09
29.27
29.15
23.12
23.08
180
170
160
150
140
130
120
110
100
90
80
70
60
50
40
30
20
10
0
-10

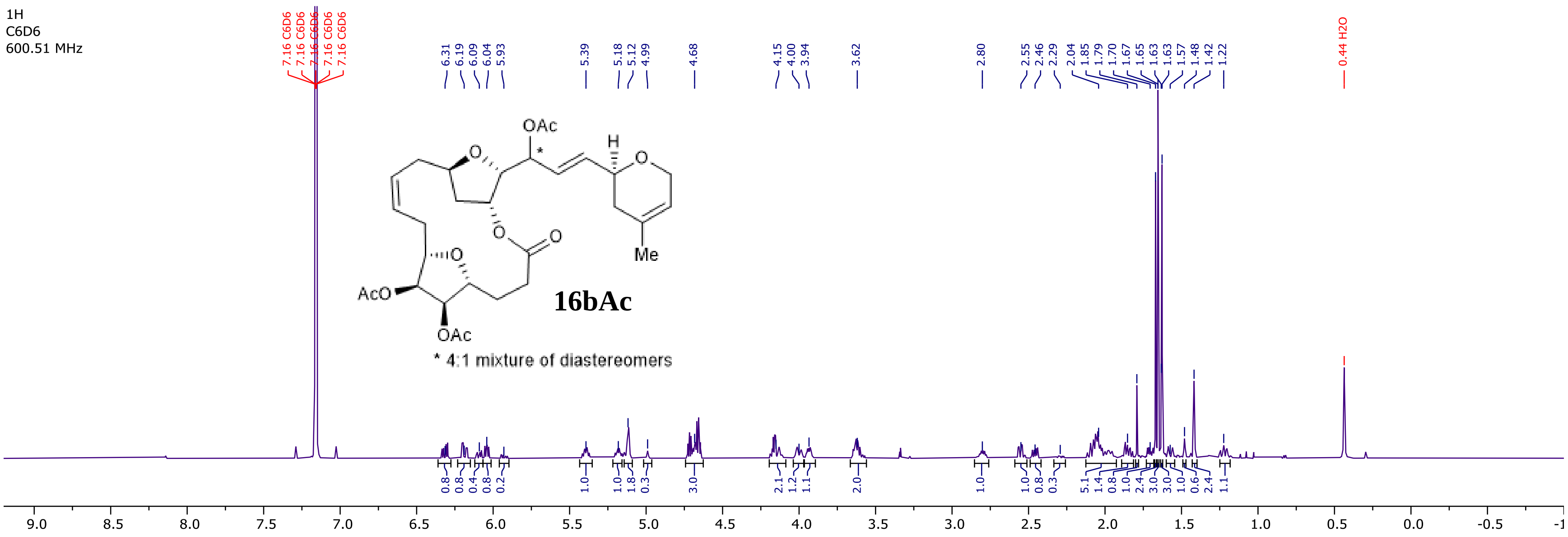
1H
C6D6
600.51 MHz
7.16 C6D6
6.31 6.19 6.09 6.04 5.93
5.39 5.18 5.12 4.99
4.68
4.15 4.00 3.94
3.62
2.80
2.55 2.46 2.29 2.04 1.85 1.79 1.70 1.67 1.65 1.63 1.63 1.57 1.48 1.42 1.22
0.44 H2O
OAc
H
O
O
O
O
Me
AcO
OAc
16bAc
* 4:1 mixture of diastereomers
0.8 0.8 0.4 0.8 0.2 1.0 1.0 1.8 0.3 3.0 2.1 1.2 1.1 2.0 1.0 1.0 0.8 0.3 5.1 1.4 0.8 1.0 2.4 3.0 3.0 1.0 0.6 2.4 1.1
9.0 8.5 8.0 7.5 7.0 6.5 6.0 5.5 5.0 4.5 4.0 3.5 3.0 2.5 2.0 1.5 1.0 0.5 0.0 -0.5

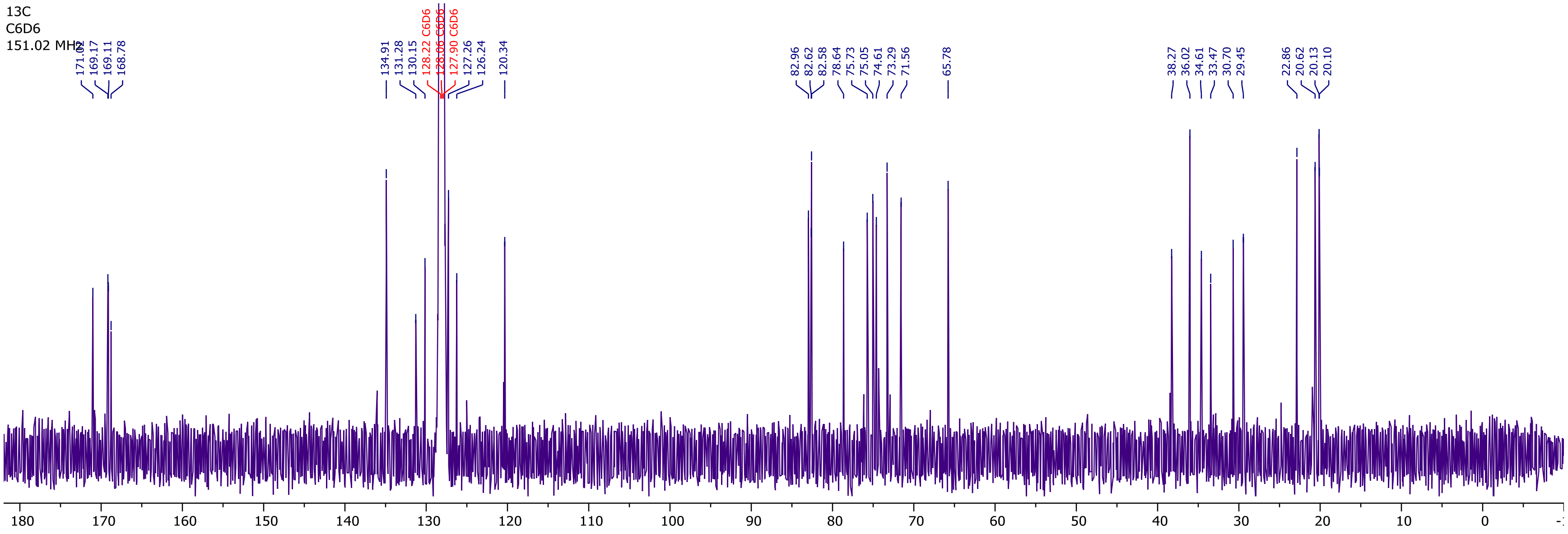
13C
C6D6
151.02 MHz
171.02 169.17 169.11 168.78
134.91 131.28 130.15 128.22 C6D6 128.06 C6D6 127.90 C6D6 127.26 126.24 120.34
82.96 82.62 82.58 78.64 75.73 75.05 74.61 73.29 71.56 65.78
38.27 36.02 34.61 33.47 30.70 29.45
22.86 20.62 20.13 20.10
180 170 160 150 140 130 120 110 100 90 80 70 60 50 40 30 20 10 0

Reaction run in a capped vial under ambient atmosphere. To a rt, stirred solution of **S84** (10 mg, 0.0271 mmol, 1.0 eq.) in $CH_2Cl_2$ (0.55 mL) was added $NaHCO_3$ (6.8 mg, 0.081 mmol, 3.0 eq.) followed by Dess-Martin Periodinane (13.8 mg, 0.0352 mmol, 1.3 eq.). The mixture was stirred at rt for 1 h 40 min. After this time, starting material was consumed as monitored by TLC analysis. The reaction mixture was quenched with a 1:1 (v/v) saturated aqueous $NaHCO_3$-10% aqueous $Na_2S_2O_3$ (1 mL) and the biphasic mixture was stirred vigorously for 30 min at rt. The mixture was transferred to a separatory funnel and saturated aqueous $NaHCO_3$ (5 mL) was added. The aqueous layer was extracted with $CH_2Cl_2$ (4x 2.5 mL), and the combined organic layers were dried ($Na_2SO_4$), filtered, and the solvent was removed in vacuo. The crude product was passaged through a short plug of C2 modified silica gel eluting with $Et_2O$. Solvent was removed in vacuo to yield the crude aldehyde **S85** (*ca.* 9.9 mg) as a colorless oil which was used immediately in the next steps without further purification.

To a solution of **S85** (9.9 mg, 0.027 mmol, 1.0 eq.) in deoxygenated DMSO (5x freeze-pump-thaw cycles) (0.7 mL) was added $CrCl_2$ doped with 1 % $NiCl_2$ (w/w) (38.0 mg, 0.309 mmol, 11.5 eq.). **3a** (12.9 mg, 0.054 mmol, 2.0 eq.) was added via syringe in deoxygenated DMSO (0.22 mL + 0.22 mL rinse) and the mixture was stirred for 17 h 30 min. After this time, the reaction mixture was transferred to a separatory funnel, diluted with $Et_2O$ (10 mL) and 15 mL of 1:1 (v/v) $H_2O$-brine was added. The organic layer was separated, and the aqueous layer extracted with $Et_2O$ (7x 15 mL). The combined organic layers were dried ($MgSO_4$), filtered, and solvent was removed in vacuo. The crude product was purified via flash column chromatography (24:1 to $CH_2Cl_2$/MeOH). Appropriate fractions were pooled, and solvent was removed in vacuo to yield **S87** (5.3 mg) as a colorless oil and a 4:1 mixture of diastereomers. The product was used immediately in the subsequent step.

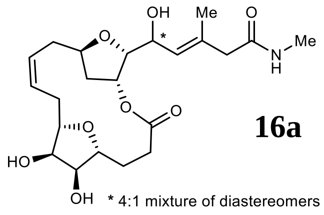


**16a**

An approximately 1.2 M solution of aqueous HCl in MeOH was prepared by slowly adding concentrated HCl (1 mL, *ca.* 12 M) to MeOH (9 mL). To a cold (0 °C), stirred solution of **S87**(5.3 mg, 0.011 mmol, 1.0 eq.) in MeOH (3 mL) was added the previously prepared solution of aqueous HCl in MeOH (46 µL, *ca.* 1.2M in MeOH, 0.055 mmol, 5.2 eq.) via microsyringe. The reaction vessel was moved to a fridge (4 °C) and allowed to stir in the fridge for 24 h. After this time, starting material was consumed as monitored by TLC analysis, and the reaction mixture was quenched by addition of $NaHCO_3$ (13 mg, 0.155 mmol, 14.1 eq.) and allowed to stir vigorously for 5 min. After this time, the reaction mixture was filtered and solvent was removed in vacuo. The crude product was then purified via flash column chromatography (97:3 to 19:1 $CH_2Cl_2$/MeOH). Appropriate fractions were pooled, and solvent was removed in vacuo to yield **16a** (4.3 mg, 5.0%, 7 steps from **S27**) as a white amorphous solid.

**Analytical Data for 16a:**

$R_f$ = 0.24 (9:1 $CH_2Cl_2$/MeOH)

$^{1}H$ NMR (601 MHz, MeOD) δ 5.56 (dddd, *J* = 10.7, 8.6, 6.9, 1.9 Hz, 1H), 5.46 (dtd, *J* = 10.8, 6.2, 5.4, 1.8 Hz, 1H), 5.42 (dq, *J* = 8.8, 1.3 Hz, 0.8H), 5.25 (dq, *J* = 9.7, 1.3 Hz, 0.2H), 5.09 (t, *J* = 3.3 Hz, 0.8H), 4.90 (t, *J* = 3.3 Hz, 0.2H), 4.69 (ddd, *J* = 11.6, 6.1, 3.6 Hz, 0.2H), 4.66 – 4.58 (m, 1.6H), 4.56 (dd, *J* = 9.6, 8.6 Hz, 0.2H), 4.02 (dd, *J* = 8.7, 3.4 Hz, 0.2H), 3.94 (dd, *J* = 9.0, 3.4 Hz, 0.8H), 3.64 – 3.57 (m, 2H), 3.56 – 3.44 (m, 2H), 3.00 (d, *J* = 14.9 Hz, 0.8H), 2.93 (m, 1H), 2.87 (dd, *J* = 3.8, 1.1 Hz, 0.2H), 2.80 (ddd, *J* = 15.8, 7.2, 2.2 Hz, 0.8H), 2.73 (s, 2.7H), 2.70 – 2.63 (m, 1.3H), 2.63 – 2.58 (m, 0.2H), 2.43 – 2.18 (m, 4H), 2.10 – 1.97 (m, 2H), 1.89 – 1.79 (m, 1H), 1.78 (d, *J* = 1.4 Hz, 2.7H), 1.75 (d, *J* = 1.4 Hz, 0.3H), 1.56 – 1.46 (m, 1H).

$^{13}C$ NMR (151 MHz, MeOD) δ 172.86, 172.65, 172.60, 172.22, 134.75, 134.01, 130.67, 130.61, 130.47, 126.51, 124.87, 124.72, 84.84, 84.79, 84.60, 84.53, 84.27, 78.40, 78.12, 76.11, 75.42, 74.97, 74.80, 74.61, 74.55, 68.15, 65.12, 48.17, 48.02, 47.88, 47.74, 47.60, 47.45, 47.31, 47.17, 46.40, 46.30, 37.56, 37.47, 34.21, 34.15, 33.58, 33.54, 29.78, 29.73, 29.13, 28.88, 25.09, 25.05, 15.85, 15.82.

HRMS (ESI): Anal. Calcd. for $C_{22}H_{34}NO_8^+$ $[M+H]^+$ 440.2279, found 440.2276

IR (neat): $\nu_{max}$ $(cm^{-1})$ = 3335 (br, OH), 3012 (w, C=*CH*), 2927 (m, CH), 2866 (m), 1722 (s, C=O), 1637 (s, C=C), 1554 (m), 1417 (m), 1351 (m), 1263 (s).

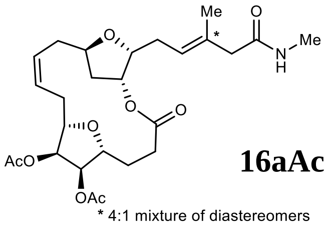


**16aAc**

**16aAc** was prepared according to general procedure A (1.4 mg, 82%)

**Analytical Data for 16aAc:**

$R_f$ = 0.19 (EtOAc)

HRMS (ESI): Anal. Calcd. for $C_{28}H_{43}N_2O_{11}^+$ $[M+NH_4]^+$ 583.2861, found 583.2842

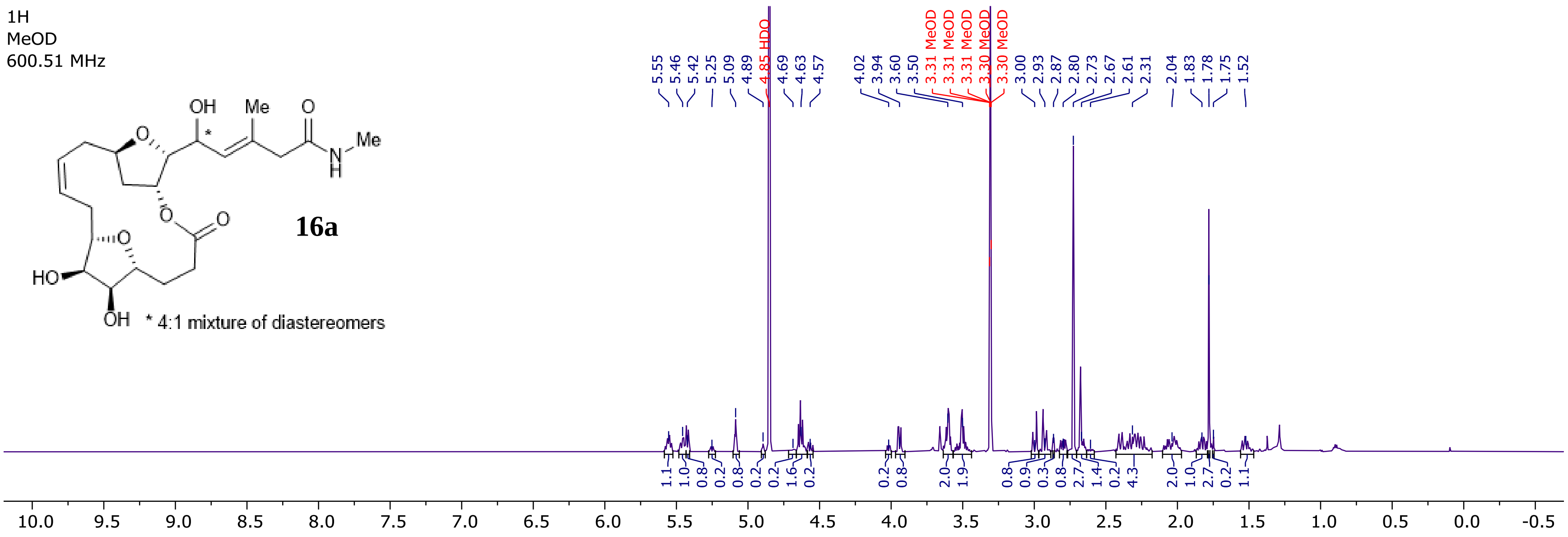
1H
MeOD
600.51 MHz
OH
Me
O
O
*
N
Me
H
O
O
O
HO
OH
16a
* 4:1 mixture of diastereomers

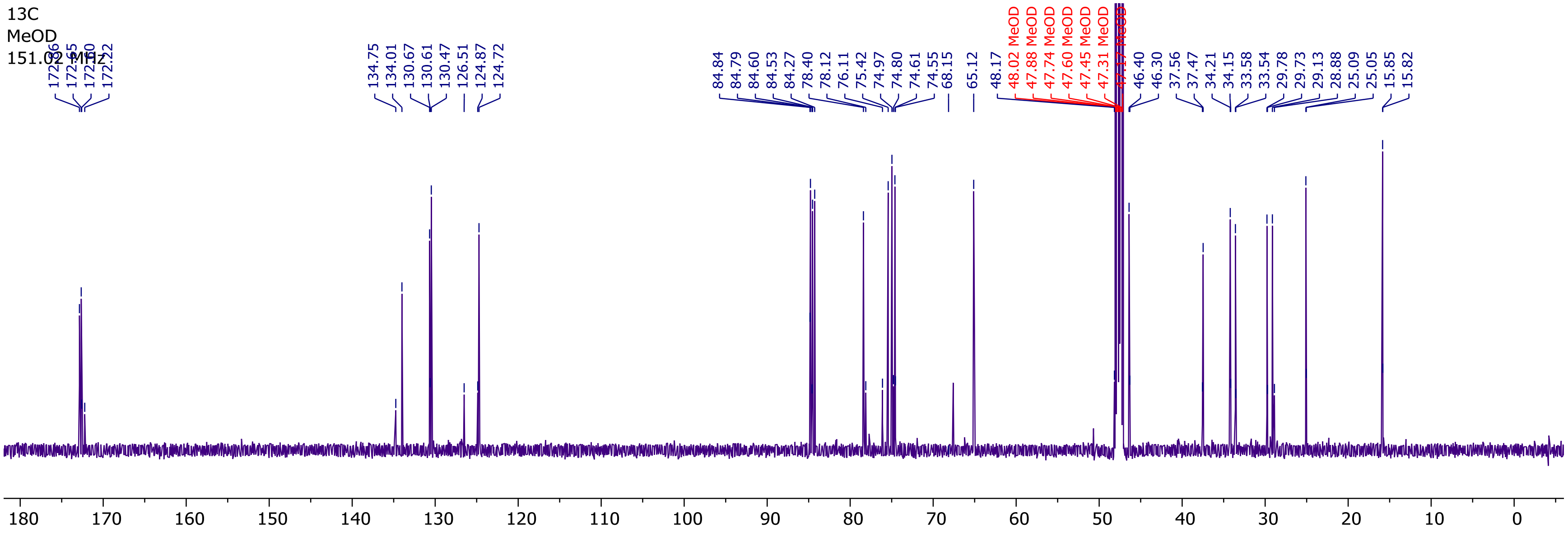
13C
MeOD
151.02 MHz

To a solution of **S85** (8.0 mg, 0.022 mmol, 1.0 eq.) in deoxygenated DMSO (5x freeze-pump-thaw cycles) (1.2 mL) was added $CrCl_2$ doped with 1 % $NiCl_2$ (w/w) (29.2 mg, 0.238 mmol, 10.8 eq.). **3c** (9.6 µL, 17 mg, 0.065 mmol, 3.0 eq.) was added via microsyringe and the mixture was stirred for 12 h. After this time, the reaction mixture was transferred to a separatory funnel, diluted with $Et_2O$ (10 mL) and 15 mL of 1:1 (v/v) $H_2O$-brine was added. The organic layer was separated, and the aqueous layer extracted with $Et_2O$ (7x 15 mL). The combined organic layers were dried ($MgSO_4$), filtered, and solvent was removed in vacuo. The crude product was purified via flash column chromatography (4:1 to Hexanes/Acetone). Appropriate fractions were pooled, and solvent was removed in vacuo to yield **S88** (1.2 mg) as a colorless oil and a 3:2 mixture of diastereomers. Note: a ground glass joint came loose overnight exposing the reaction mixture to air, resulting in a deleterious effect on the reaction yield. The product was used immediately in the subsequent step.

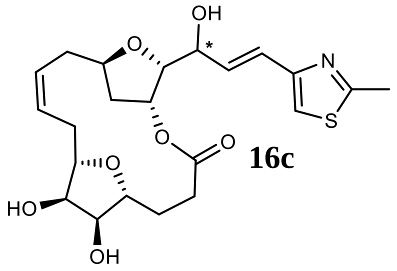


**16c**

* 3:2 mixture of diastereomers

An approximately 1.2 M solution of aqueous HCl in MeOH was prepared by slowly adding concentrated HCl (1 mL, *ca.* 12 M) to MeOH (9 mL). To a cold (0 °C), stirred solution of **S88** (1.2 mg, 0.0024 mmol, 1.0 eq.) in MeOH (0.7 mL) was added the previously prepared solution of aqueous HCl in MeOH (11 µL, *ca.* 1.2M in MeOH, 0.013 mmol, 5.5 eq.) via microsyringe. The reaction vessel was moved to a fridge (4 °C) and allowed to stir in the fridge for 28 h. After this time, starting material was consumed as monitored by TLC analysis, and the reaction mixture was quenched by addition of $NaHCO_3$ (10 mg, 0.12 mmol, 50 eq.) and allowed to stir vigorously for 5 min. After this time, reaction mixture was filtered and solvent was removed in vacuo. The crude product was then purified via flash column chromatography (97:3 to 19:1 $CH_2Cl_2$/MeOH). The resultant $^1$H spectrum showed contamination by significant amounts of aldehyde **S85**. Thus, the material was repurified via reverse phase HPLC using a kinetix™ column using an acid free isocratic mixture (1:4) of MeCN/$H_2O$ at 1.0 mL/min. Solvent was removed in vacuo to yield **16c** (0.8 mg, 1.1%, 7 steps from **S27**) as a white film.

**Analytical Data for 16c:**

$R_f$ = 0.43 (9:1 $CH_2Cl_2$/MeOH)

$^1$H NMR (601 MHz, MeOD) δ 7.20 (s, 0.4H), 7.20 (s, 0.6H), 6.73 – 6.63 (m, 1.4H), 6.47 (dd, *J* = 15.7, 5.6 Hz, 0.6H), 5.61 – 5.52 (m, 1H), 5.45 (ddddd, *J* = 12.7, 10.9, 6.2, 4.4, 1.9 Hz, 1H), 5.11 (t, *J* = 3.2 Hz, 0.4H), 4.99 (t, *J* = 3.3 Hz, 0.6H), 4.69 (dtdd, *J* = 17.6, 11.3, 6.0, 3.5 Hz, 1H), 4.52 (ddd, *J* = 8.6, 5.7, 1.5 Hz, 0.6H), 4.44 (dd, *J* = 8.8, 4.1 Hz, 0.4H), 3.99 (dd, *J* = 8.6, 3.5 Hz, 0.6H), 3.96 (dd, *J* = 8.8, 3.3 Hz, 0.4H), 3.65 – 3.58 (m, 2H), 3.56 – 3.52 (m, 1H), 3.49 (ddt, *J* = 10.5, 6.8, 3.2 Hz, 1H), 2.81 – 2.70 (m, 2H), 2.69 (s, 1.3H), 2.68 (s, 1.7H), 2.46 – 2.34 (m, 2H), 2.33 – 2.27 (m, 1H), 2.27 – 2.21 (m, 1H), 2.09 – 1.99 (m, 2H), 1.85 (dddd, *J* = 23.5, 12.3, 11.1, 8.4 Hz, 1H), 1.52 (td, *J* = 12.5, 3.2 Hz, 1H).

$^{13}$C NMR (151 MHz, MeOD) δ 174.23, 173.86, 168.62, 168.30, 154.66, 154.27, 134.34, 132.05, 132.02, 131.64, 126.28, 125.11, 124.51, 116.74, 116.05, 86.32, 86.29, 86.23, 85.95, 85.93, 85.60, 79.81, 79.40, 77.42, 77.11, 76.28, 76.25, 75.99, 72.58, 70.65, 38.95, 38.67, 35.60, 35.57, 34.96, 34.94, 31.16, 31.07, 30.52, 30.49, 18.74, 18.73.

HRMS (ESI): Anal. Calcd. for $C_{22}H_{30}NO_7S^+$ $[M+H]^+$ 452.1738, found 452.1726

IR (neat): $\nu_{max}$ $(cm^{-1})$ = 3389 (br, OH), 3017 (w, C=*CH*), 2925 (m, CH), 1723 (s, C=O), 1657 (m, C=C), 1594 (m, C=C), 1434 (m), 1352 (m), 1256 (s).

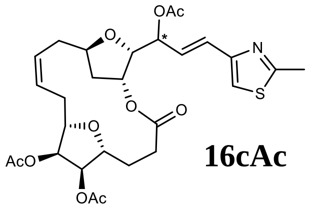


**16cAc**

* 3:2 mixture of diastereomers

**16cAc** was prepared according to general procedure A (0.25 mg, 95%)

**Analytical Data for 16cAc:**

$R_f$ = 0.43 (2:3 Hexanes/EtOAc)

HRMS (ESI): Anal. Calcd. for $C_{28}H_{36}NO_{10}S^+$ $[M+H]^+$ 578.2054, found 578.2015

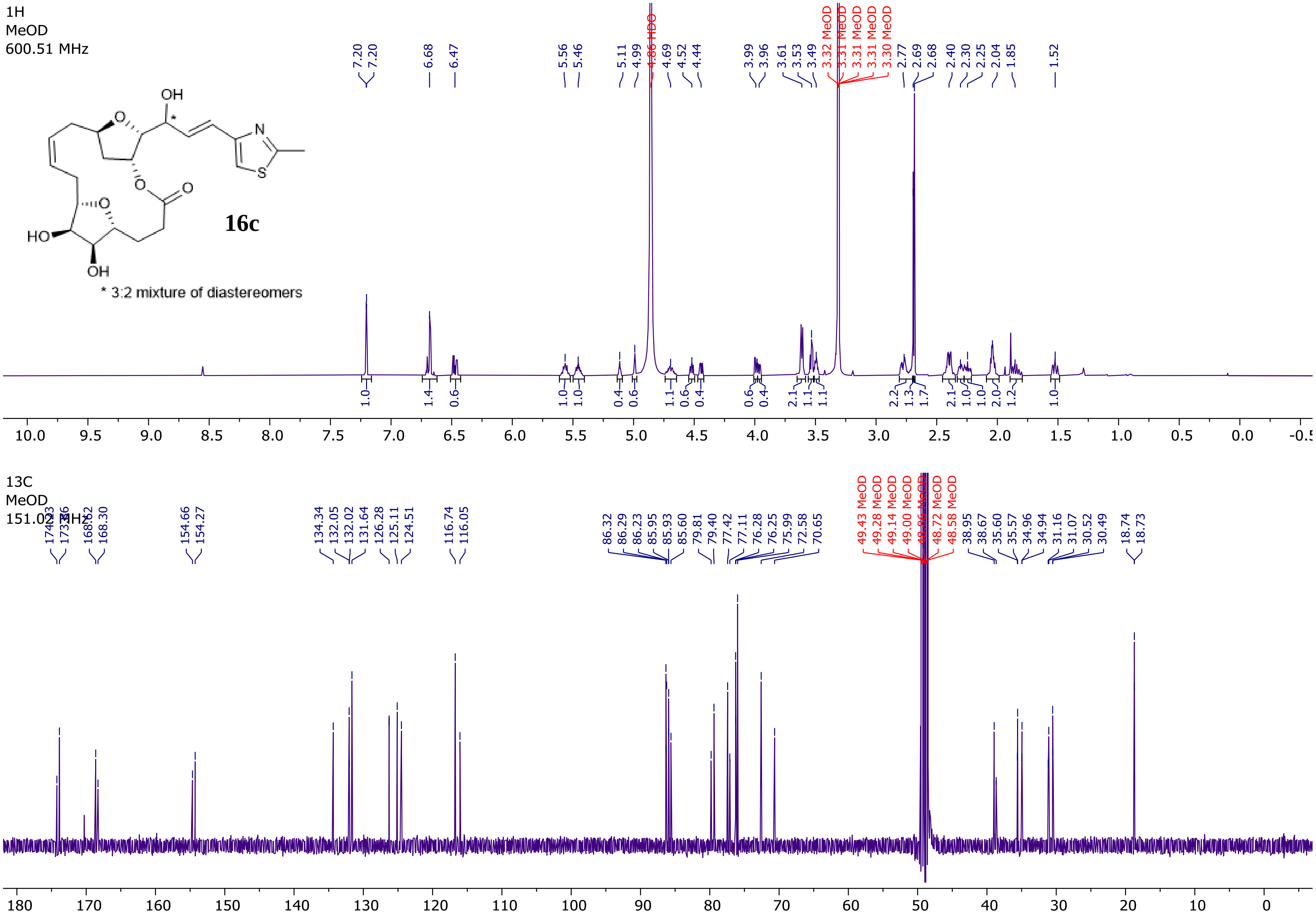
1H
MeOD
600.51 MHz
16c
* 3:2 mixture of diastereomers
13C
MeOD
151.02 MHz

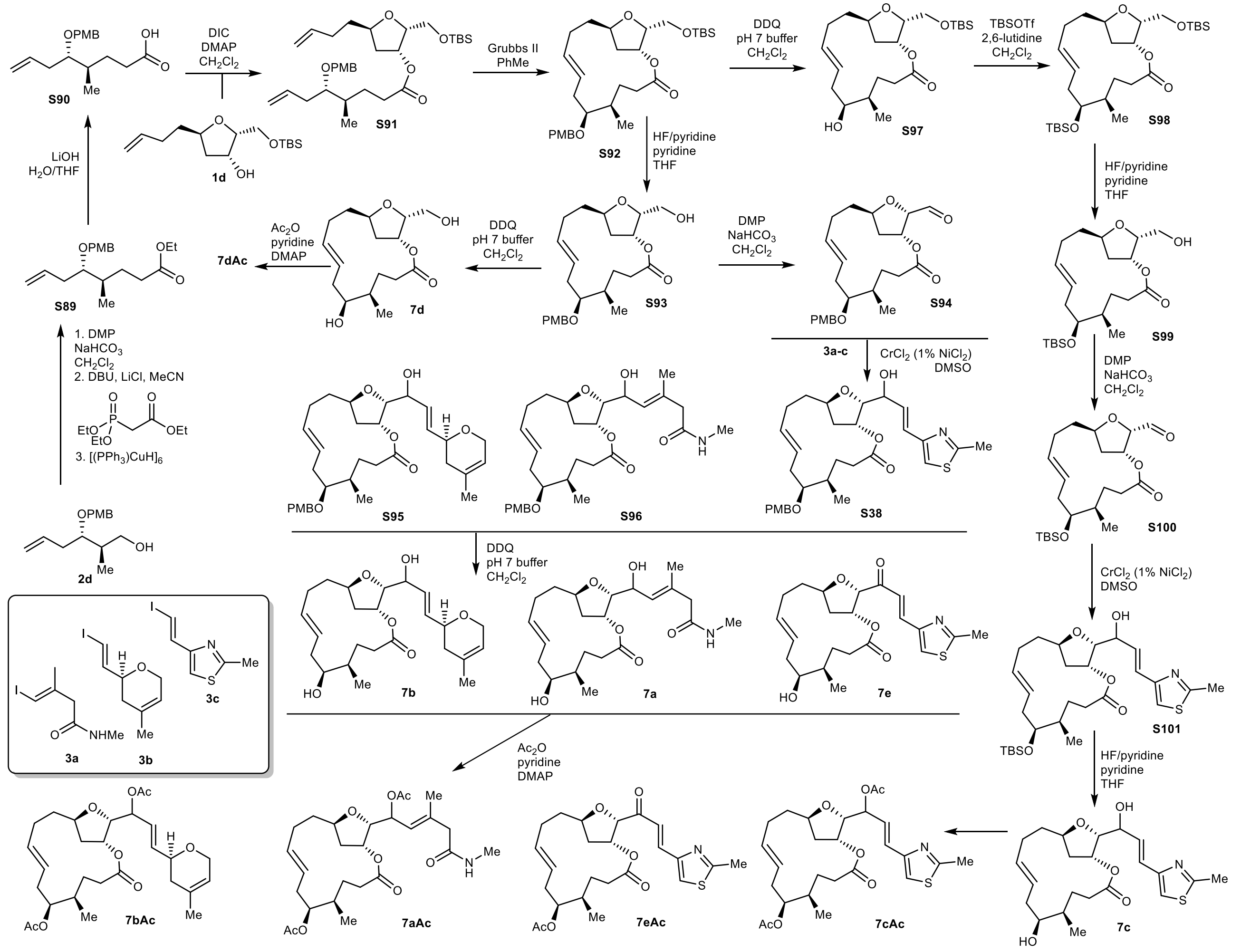


**Supplementary Fig. 43 | Synthesis of pMLs 7a-e and 7aAc-eAc.**

Abbreviations: DIC = N,N'-diisopropylcarbodiimide, THF = tetrahydrofuran, DMSO = dimethylsulfoxide, DDQ = 2,3-dichloro-5,6-dicyano-*para*-benzoquinone, TBSOTf = tert-butyldimethylsilyl trifluoromethanesulfonate, Grubbs II = Dichloro[1,3-bis(2,4,6-trimethylphenyl)-2-imidazolidinylidene](benzylidene)(tricyclohexylphosphine)ruthenium(II), DMP = Dess-Martin periodinane, DMAP = 4-dimethylaminopyridine, TBS =tert-butyldimethylsilyl, PMB = *para*-methoxybenzyl.

To a stirred solution of **2d** (177 mg, 0.70 mmol, 1.0 eq.) in anhydrous $CH_2Cl_2$ (7 mL) was added Dess-Martin Periodinane (386 mg, 0.91 mmol, 1.3 eq.). The mixture was stirred at rt for 30 minutes and quenched with a solution of 1:1:1 $H_2O$:saturated aqueous $NaHCO_3$:saturated aqueous $Na_2S_2O_3$ (20 mL). The aqueous layer was extracted with $CH_2Cl_2$ (3x 50 mL) and the combined organic layers were dried ($Na_2SO_4$), filtered, and the solvent was removed in vacuo to yield the crude aldehyde (*ca.* 174 mg) as a colorless oil, which was used immediately for the next step

To a stirred suspension of LiCl (103.9 mg, 2.45 mmol, 3.5 eq, flame dried in vacuo), triethylphosphonoacetate (314 mg, 1.4 mmol, 2.0 eq.), and DBU (0.21 mL, 1.4 mmol, 2.0 eq.) in MeCN (3.5 mL) was added to the crude aldehyde from the previous step (174 mg, *ca.* 0.70 mmol, 1.0 eq.) in MeCN (1 mL). The reaction was allowed to stir at rt for 30 min. After this time, starting material was consumed as monitored by TLC analysis, and the reaction was quenched by addition of saturated aqueous $NH_4Cl$ (20 mL). The biphasic mixture was poured into a separatory funnel, the organic layer was separated, and the aqueous layer was extracted with $CH_2Cl_2$ (3x 40 mL). The combined organic layers were dried ($Na_2$SO4), filtered, and solvent was removed in vacuo. The crude product was purified via flash column chromatography (9:1 petroleum ether/EtOAc). Appropriate fractions were pooled, and solvent was removed in vacuo to yield the ester (187 mg, 83%) as a colorless oil, which was used immediately in the next step.

To a stirred solution of the ester from the previous step (173 mg, 0.55 mmol, 1.0 eq) in anhydrous toluene (deoxygenated via sparging with nitrogen for 30 minutes) (5.5 mL) was added Stryker's reagent in anhydrous, deoxygenated toluene (0.4 M, 5.5 mL, 0.58 mmol, 4 eq.). The mixture was stirred for 4 h after which the reaction mixture was flushed through a plug of silica gel ($Et_2O$) and the solvent was removed in vacuo. The crude product was purified via flash column chromatography (97:3 petroleum ether/EtOAc). Appropriate fractions were pooled, and solvent was removed in vacuo to yield **S89** (112 mg, 63%) as a yellow oil.

To a rt stirred solution of **S89** (105 mg, 0.33 mmol, 1.0 eq.) dissolved in 2:1 THF/$H_2O$ (1.2 mL) was added 1 M aqueous LiOH (1.96 mL, 6.0 eq.). After 1 day, the reaction was quenched with 1 M HCl (0.85 mL) careful to avoid acidifying the mixture under pH 4. The aqueous layer was extracted with $Et_2O$ (3x 20 mL) and the combined organic layers were dried ($MgSO_4$), filtered, and the solvent was concentrated in vacuo, to yield the crude product **S90** (96 mg) as a yellow oil which was used immediately in the next step.

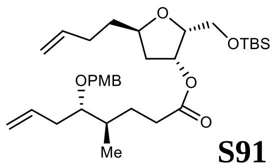

**S91**

Note: Reaction performed in capped vessel under ambient atmosphere.

To a rt stirred solution of acid **S90** (96 mg, 0.33 mmol, 1.0 eq.), alcohol **1d** (143 mg, 0.50 mmol, 1.5 eq.), and DMAP (16 mg, 0.13 mmol, 0.40 eq.) dissolved in anhydrous $CH_2Cl_2$ (3.3 mL), was added DIC (0.12 mL, 96 mg, 0.76 mmol, 2.3 eq.). The reaction mixture was stirred for 4 h at rt, after which time starting material was consumed as monitored by TLC analysis. The reaction mixture was quenched with saturated aqueous $NH_4Cl$ (10 mL) and $H_2O$ (10 mL) and the aqueous layer was extracted with $CH_2Cl_2$ (3x 40 mL). The combined organic layers were dried ($Na_2SO_4$), filtered, and solvent was removed in vacuo. The crude product was purified via flash column chromatography (47:3 Petroleum ether (bp: 36-60)/$Et_2O$). Appropriate fractions were pooled, and solvent was removed in vacuo to yield **S91** (120 mg, 66%) as a colorless oil.

**Analytical Data for S91:**

$R_f$ = 0.29 (17:3 Petroleum ether (bp: 36-60)/$Et_2O$)

$[\alpha]_D^{20}$ = +17 ° (c = 0.21, MeOH)

$^1$H NMR (500 MHz, $CDCl_3$) δ 7.29 – 7.22 (m, 2H), 6.90 – 6.83 (m, 2H), 5.84 (dddt, *J* = 20.6, 16.9, 10.2, 6.8 Hz, 2H), 5.41 (ddd, *J* = 5.1, 3.6, 1.4 Hz, 1H), 5.09 (dq, *J* = 17.1, 1.6 Hz, 1H), 5.06 – 4.98 (m, 2H), 4.96 (dq, *J* = 10.2, 1.4 Hz, 1H), 4.48 (d, *J* = 11.1 Hz, 1H), 4.41 (d, *J* = 11.1 Hz, 1H), 4.14 (ddt, *J* = 9.5, 7.2, 5.8 Hz, 1H), 4.06 (ddd, *J* = 7.0, 5.9, 3.8 Hz, 1H), 3.80 (s, 3H), 3.78 – 3.68 (m, 2H), 3.25 (dt, *J* = 6.4, 5.3 Hz, 1H), 2.38 (ddd, *J* = 15.7, 10.2, 5.5 Hz, 1H), 2.32 – 2.21 (m, 3H), 2.23 – 2.03 (m, 3H), 1.91 – 1.75 (m, 2H), 1.78 – 1.65 (m, 2H), 1.58 – 1.40 (m, 2H), 0.90 (d, *J* = 6.8 Hz, 3H), 0.86 (s, 9H), 0.04 (s, 3H), 0.03 (s, 3H).

$^{13}$C NMR (126 MHz, $CDCl_3$) δ 173.20, 159.23, 138.28, 135.57, 131.04, 129.41, 116.82, 114.87, 113.86, 82.42, 80.82, 77.60, 74.44, 71.45, 61.35, 55.41, 39.38, 35.52, 35.26, 34.95, 32.62, 30.38, 27.74, 25.98, 18.38, 15.07, -5.18, -5.30.

HRMS (ESI): Anal. Calcd. for $C_{32}H_{56}NO_6Si^+$ $[M+NH_4]^+$ 578.3871, found 578.3846

IR (neat): $\nu_{max}$ $(cm^{-1})$ = 3075 (w, C=*CH*), 2931 (m, CH), 2858 (m, CH), 1739 (s, C=O), 1614 (m, C=C), 1513 (s), 1466 (m), 1301 (w), 1249 (s)

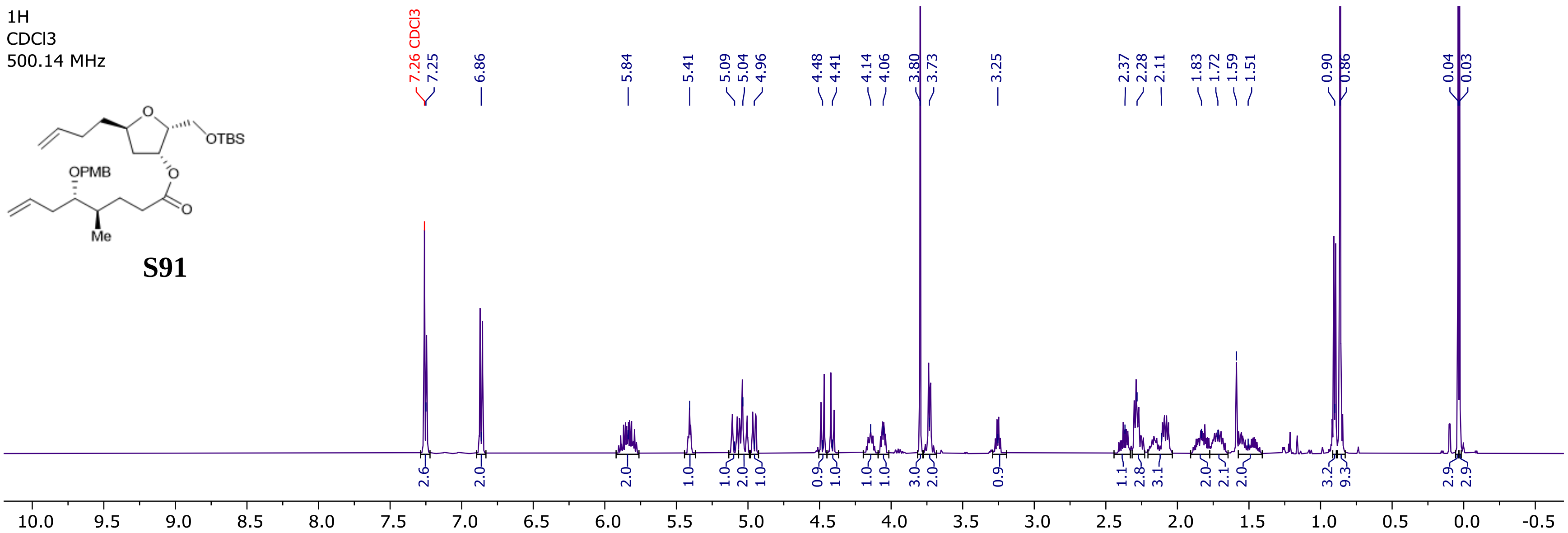
1H
CDCl3
500.14 MHz
7.26 CDCl3
7.25
6.86
5.84
5.41
5.09
5.04
4.96
4.48
4.41
4.14
4.06
3.80
3.73
3.25
2.37
2.28
2.11
1.83
1.72
1.59
1.51
0.90
0.86
0.04
0.03
2.6
2.0
2.0
1.0
1.0
2.0
1.0
0.9
1.0
1.0
1.0
3.0
2.0
0.9
1.1
2.8
3.1
2.0
2.1
2.0
3.2
9.3
2.9
2.9
10.0 9.5 9.0 8.5 8.0 7.5 7.0 6.5 6.0 5.5 5.0 4.5 4.0 3.5 3.0 2.5 2.0 1.5 1.0 0.5 0.0 -0.5

OTBS
OPMB
O
O
O
Me

**S91**

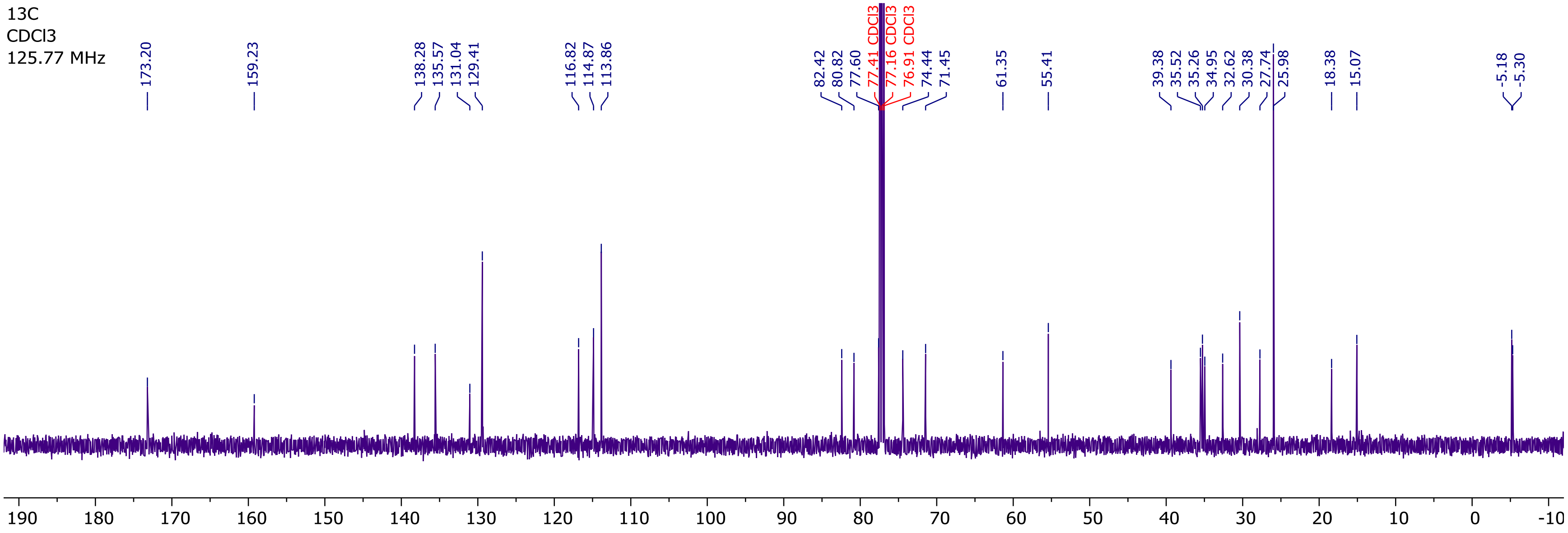
13C
CDCl3
125.77 MHz
173.20
159.23
138.28
135.57
131.04
129.41
116.82
114.87
113.86
82.42
80.82
77.60
77.41 CDCl3
77.16 CDCl3
76.91 CDCl3
74.44
71.45
61.35
55.41
39.38
35.52
35.26
34.95
32.62
30.38
27.74
25.98
18.38
15.07
-5.18
-5.30
190 180 170 160 150 140 130 120 110 100 90 80 70 60 50 40 30 20 10 0 -10

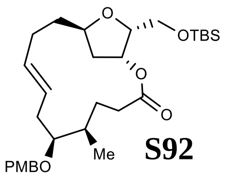


**S92**

To a stirred, heated (60 °C) solution of **S91** (124 mg, 0.22 mmol, 1.0 eq.) dissolved in anhydrous, deoxygenated toluene (deoxygenated by sparging with nitrogen for 30 minutes) (88 mL) was added dropwise Grubbs 2nd generation catalyst (38 mg, 0.044 mmol, 0.2 eq.) in anhydrous, deoxygenated toluene (2.7 mL). Throughout the course of the reaction, the reaction mixture was sparged with a stream of $N_2$(g). After 40 min stirring at 60 °C, the reaction was cooled to 0 °C and quenched with potassium 2-isocyanoacetate (38 mg, 0.30 mmol, 1.4 eq) in MeOH (25 mL). The mixture was warmed to rt and stirred for 90 min. and the solvent was removed in vacuo. The crude product was purified via flash column chromatography (9:1 to 4:1 hexanes/$Et_2O$). Appropriate fractions were pooled, and solvent was removed in vacuo to yield (*E*)-isomer **S92** (76 mg, 65%) as a colorless oil.

**Analytical Data for S92:**

$R_f$ = 0.20 (4:1 hexanes/$Et_2O$)

$[\alpha]_D^{20}$ = +25 ° (c = 0.44, $CH_2Cl_2$)

$^1$H NMR (600 MHz, $CDCl_3$) δ 7.28 – 7.24 (m, 2H), 6.90 – 6.85 (m, 2H), 5.45 (t, *J* = 12.6 Hz, 1H), 5.37 – 5.23 (m, 2H), 4.55 (d, *J* = 11.6 Hz, 1H), 4.32 (d, *J* = 11.6 Hz, 1H), 4.14 (q, *J* = 6.4 Hz, 1H), 3.94 (td, *J* = 11.3, 5.1 Hz, 1H), 3.80 (d, *J* = 1.6 Hz, 3H), 3.77 – 3.72 (m, 2H), 3.00 (d, *J* = 10.2 Hz, 1H), 2.55 – 2.46 (m, 2H), 2.42 – 2.29 (m, 3H), 2.26 – 2.20 (m, 1H), 2.14 – 2.01 (m, 2H), 1.86 (dt, *J* = 18.7, 12.2 Hz, 1H), 1.55 – 1.36 (m, 3H), 1.06 (td, *J* = 12.9, 6.4 Hz, 1H), 0.86 (s, 9H), 0.80 (d, *J* = 6.5 Hz, 3H), 0.03 (m, 6H).

$^{13}$C NMR (151 MHz, $CDCl_3$) δ 173.58, 159.09, 131.08, 130.94, 129.28, 126.60, 113.78, 80.91, 80.86, 77.64, 75.02, 70.72, 61.33, 55.41, 37.90, 33.84, 32.34, 32.29, 32.05, 29.91, 28.88, 26.00, 18.45, 14.23, -5.19, -5.38.

HRMS (ESI): Anal. Calcd. for $C_{30}H_{52}NO_6Si^+$ $[M+NH_4]^+$ 550.3558, found 550.3553

IR (neat): $\nu_{max}$ $(cm^{-1})$ = 2929 (m, CH), 2857 (m, CH), 1738 (s, C=O), 1613 (w, C=C), 1513 (m), 1466 (w), 1368 (w), 1301 (w), 1249 (s)

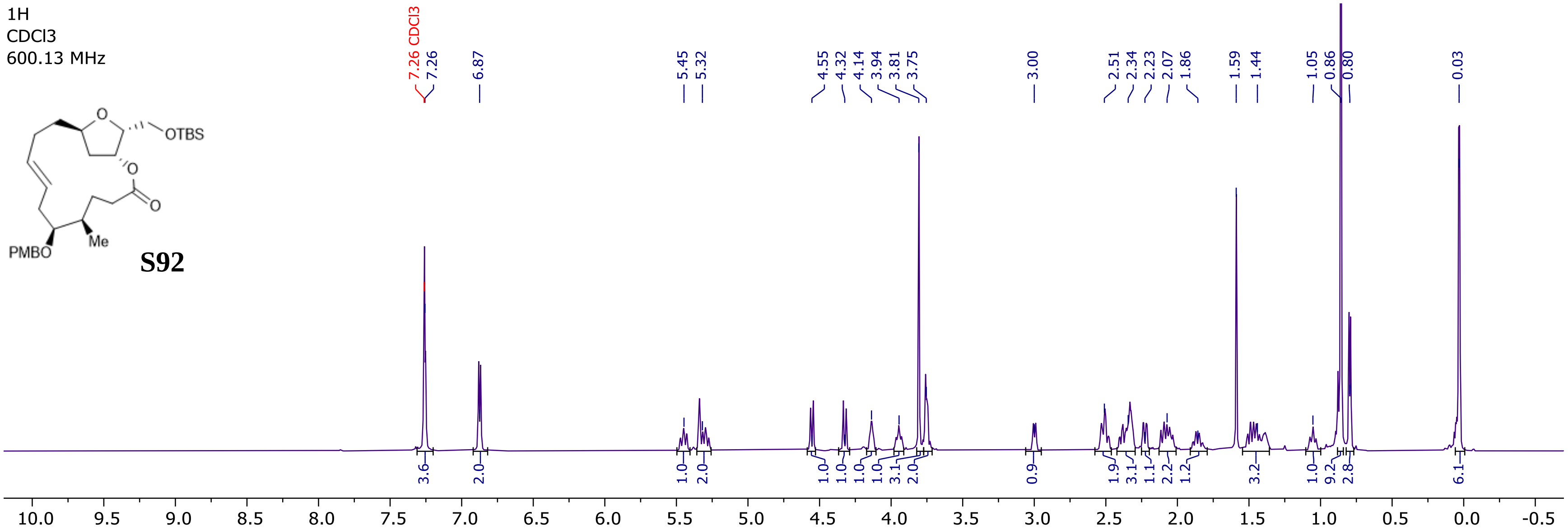
1H
CDCl3
600.13 MHz
OTBS
PMBO
Me
S92
7.26 CDCl3
7.26
6.87
5.45
5.32
4.55
4.32
4.14
3.94
3.81
3.75
3.00
2.51
2.34
2.23
2.07
1.86
1.59
1.44
1.05
0.86
0.80
0.03
3.6
2.0
1.0
2.0
1.0
1.0
1.0
1.0
3.1
2.0
0.9
1.9
3.1
1.1
2.2
1.2
3.2
1.0
9.2
2.8
6.1
10.0
9.5
9.0
8.5
8.0
7.5
7.0
6.5
6.0
5.5
5.0
4.5
4.0
3.5
3.0
2.5
2.0
1.5
1.0
0.5
0.0
-0.5

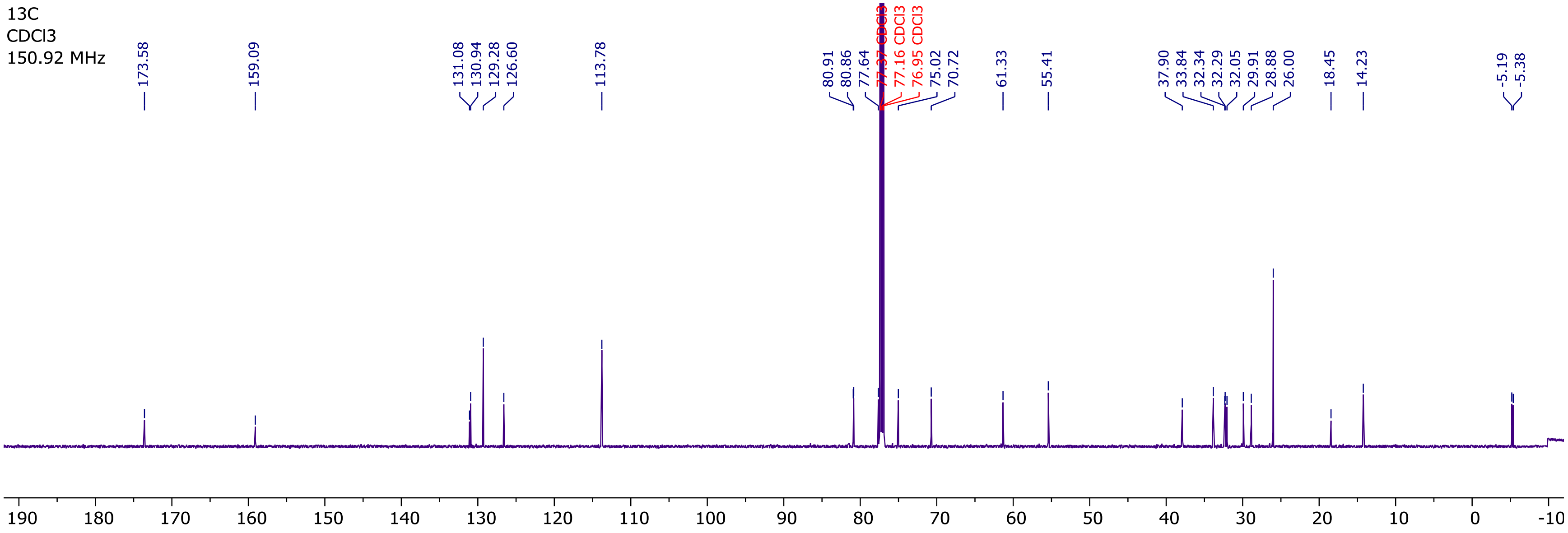
13C
CDCl3
150.92 MHz
173.58
159.09
131.08
130.94
129.28
126.60
113.78
80.91
80.86
77.64
77.37 CDCl3
77.16 CDCl3
76.95 CDCl3
75.02
70.72
61.33
55.41
37.90
33.84
32.34
32.29
32.05
29.91
28.88
26.00
18.45
14.23
-5.19
-5.38
190
180
170
160
150
140
130
120
110
100
90
80
70
60
50
40
30
20
10
0
-10

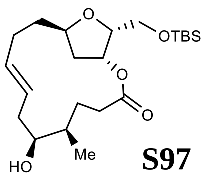


**S97**

Note: Reaction performed in capped vessel under ambient atmosphere.

To a vigorously stirred solution of **S92** (69 mg, 0.13 mmol, 1.0 eq.) in $CH_2Cl_2$ (8 mL) and pH 7 aqueous phosphate buffer (4 mL) was added 2,3-dichloro-5,6-dicyano-benzoquinone (118 mg, 0.52 mmol, 4 eq.). After 1 h the starting material was consumed as monitored by TLC analysis and the reaction was quenched with saturated $NaHCO_3$ (10 mL). The aqueous layer was extracted with $CH_2Cl_2$ (3x 30 mL) and the combined organic layers were dried ($Na_2SO_4$) and filtered, followed by removal of the solvent in vacuo. The crude product was purified via flash column chromatography (9:1 to 3:1 Petroleum ether (bp: 36-60)/$Et_2O$). Appropriate fractions were pooled, and solvent was removed in vacuo to yield **S97** (54 mg, 97%) as a colorless oil.

**Analytical Data for S97:**

$R_f$ = 0.15 (4:1 Petroleum ether (bp: 36-60)/$Et_2O$)

$[\alpha]_D^{20}$ = +0.8 ° (c = 0.26, $CH_2Cl_2$)

$^1$H NMR (600 MHz, $CDCl_3$) δ 5.47 (ddd, *J* = 14.8, 9.7, 4.6 Hz, 1H), 5.40 – 5.29 (m, 2H), 4.15 (td, *J* = 7.0, 3.6 Hz, 1H), 3.99 – 3.90 (m, 1H), 3.77 (d, *J* = 6.9 Hz, 2H), 3.37 (dt, *J* = 10.0, 3.1 Hz, 1H), 2.47 – 2.24 (m, 7H), 2.07 (tt, *J* = 13.1, 4.3 Hz, 1H), 1.88 (tdd, *J* = 14.1, 10.8, 4.3 Hz, 1H), 1.55 – 1.42 (m, 3H), 1.32 – 1.16 (m, 2H), 0.86 (s, 9H), 0.83 (d, *J* = 6.2 Hz, 3H), 0.05 – 0.01 (m, 6H).

$^{13}$C NMR (151 MHz, $CDCl_3$) δ 173.79, 131.74, 125.61, 80.86, 77.55, 77.37, 74.28, 61.24, 37.64, 37.56, 34.82, 32.21, 32.08, 29.99, 28.88, 26.00, 18.45, 14.31, -5.19, -5.38.

HRMS (ESI): Anal. Calcd. for $C_{22}H_{41}O_5Si^+$ $[M+H]^+$ 413.2718, found 413.2693

IR (neat): $\nu_{max}$ $(cm^{-1})$ = 3472 (br, OH), 2929 (m, CH), 2858 (m, CH), 1738 (s, C=O), 1710 (m), 1450 (w), 1360 (w), 1255 (m)

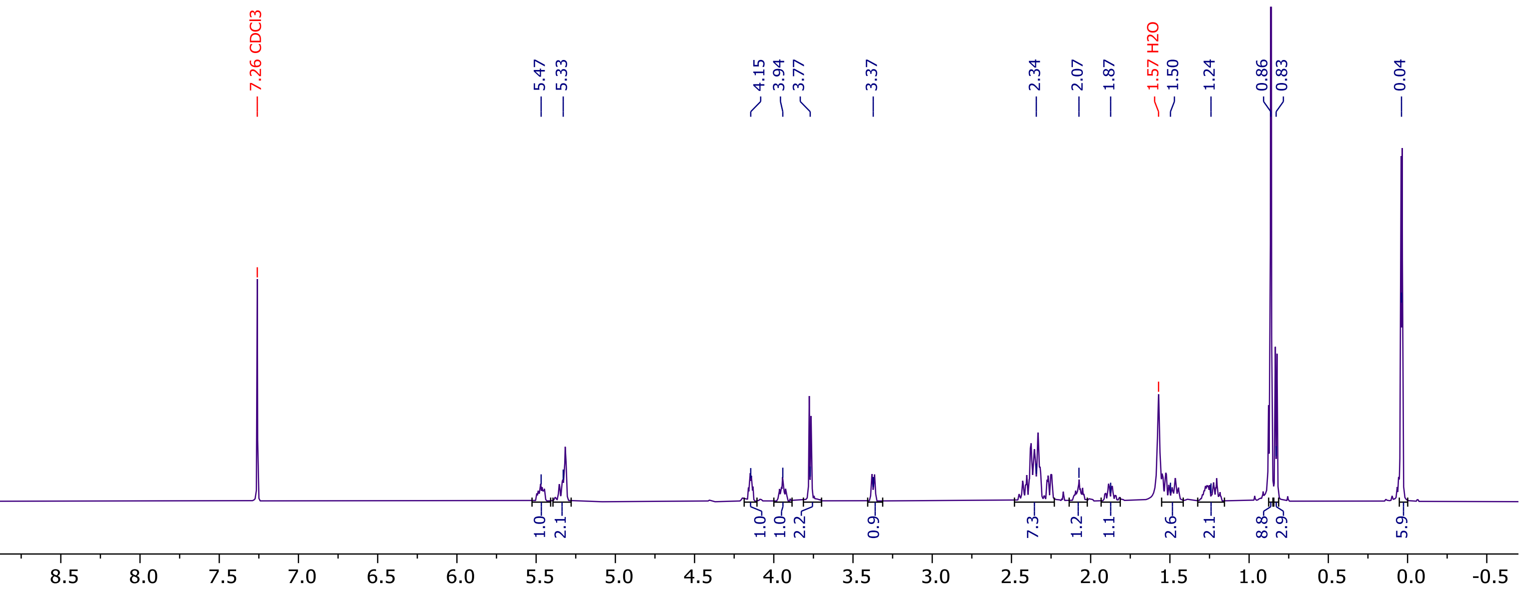
1H
CDCl3
600.13 MHz
7.26 CDCl3
5.47
5.33
4.15
3.94
3.77
3.37
2.34
2.07
1.87
1.57 H2O
1.50
1.24
0.86
0.83
0.04
1.0
2.1
1.0
1.0
2.2
0.9
7.3
1.2
1.1
2.6
2.1
8.8
2.9
5.9
10.0
9.5
9.0
8.5
8.0
7.5
7.0
6.5
6.0
5.5
5.0
4.5
4.0
3.5
3.0
2.5
2.0
1.5
1.0
0.5
0.0
-0.5

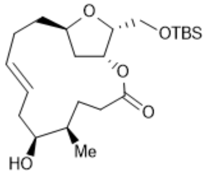
OTBS
O
O
O
HO
Me

**S97**

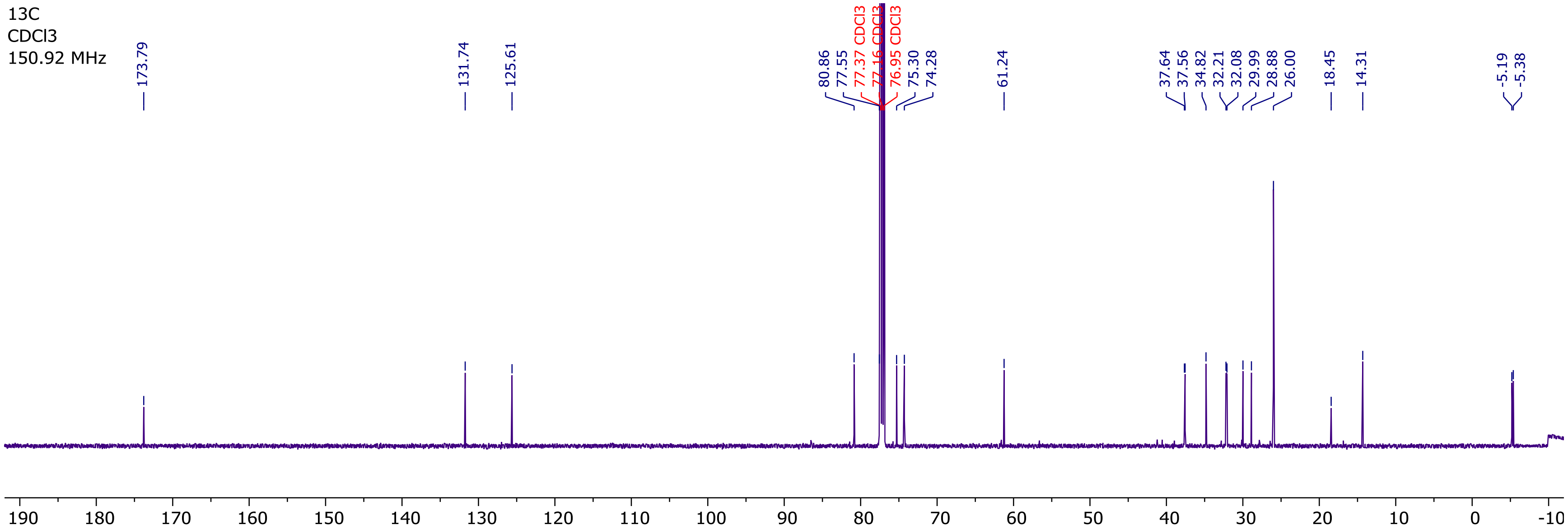
13C
CDCl3
150.92 MHz
173.79
131.74
125.61
80.86
77.55
77.37 CDCl3
77.16 CDCl3
76.95 CDCl3
75.30
74.28
61.24
37.64
37.56
34.82
32.21
32.08
29.99
28.88
26.00
18.45
14.31
-5.19
-5.38
190
180
170
160
150
140
130
120
110
100
90
80
70
60
50
40
30
20
10
0
-10

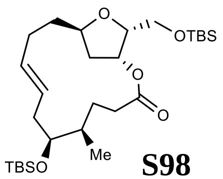

**S98**

To a cold (0 °C) stirred solution of **S97** (52 mg, 0.13 mmol, 1.0 eq.) and 2,6-lutidine (0.044 mL, 0.38 mmol, 3.0 eq) dissolved in anhydrous $CH_2Cl_2$ (1.3 mL), was added tert-butyldimethylsilyl trifluoromethanesulfonate (0.043 mL, 0.19 mmol, 1.5 eq.). The reaction mixture was warmed to rt and stirred for 30 min., after which another portion of 2,6-lutidine (0.044 mL, 0.38 mmol, 3.0 eq) and tert-butyldimethylsilyl trifluoromethanesulfonate (0.043 mL, 0.19 mmol, 1.5 eq.) was added. After a further 30 min., the starting material was consumed as monitored by TLC analysis. The reaction mixture was quenched with saturated aqueous $NH_4Cl$ (10 mL). The aqueous layer was extracted with $CH_2Cl_2$ (3x 25 mL) and the combined organic layers were dried ($Na_2SO_4$), filtered, and the solvent was removed in vacuo. The crude product was purified via flash column chromatography (19:1 Petroleum ether (bp: 36-60)/$Et_2O$). Appropriate fractions were pooled, and the solvent was removed in vacuo to yield **S98** (59 mg, 88%) as a colorless oil.

**Analytical Data for S98:**

$R_f$ = 0.28 (9:1 Petroleum ether (bp: 36-60)/$Et_2O$)

$[\alpha]_D^{20}$ = +5.5 ° (c = 0.17, $CDCl_3$)

$^1$H NMR (600 MHz, $CDCl_3$) δ 5.47 (dt, *J* = 14.7, 7.2 Hz, 1H), 5.38 – 5.22 (m, 2H), 4.14 (q, *J* = 6.7 Hz, 1H), 3.95 (ddt, *J* = 11.5, 8.4, 4.2 Hz, 1H), 3.81 – 3.72 (m, 2H), 3.30 (dd, *J* = 9.7, 3.5 Hz, 1H), 2.40 – 2.28 (m, 4H), 2.26 – 2.16 (m, 3H), 2.06 (tt, *J* = 13.0, 4.6 Hz, 1H), 1.91 – 1.80 (m, 1H), 1.54 – 1.42 (m, 2H), 1.28 (td, *J* = 11.1, 6.1 Hz, 1H), 1.02 (td, *J* = 14.9, 13.2, 5.2 Hz, 1H), 0.92 (s, 9H), 0.86 (s, 9H), 0.78 (d, *J* = 6.5 Hz, 3H), 0.08 – -0.01 (m, 12H).

$^{13}$C NMR (151 MHz, $CDCl_3$) δ 173.59, 130.75, 126.73, 80.86, 77.69, 74.99, 74.76, 61.26, 37.68, 37.65, 35.09, 32.31, 32.15, 30.02, 29.45, 26.02, 25.99, 18.43, 18.23, 14.14, -4.13, -4.81, -5.19, -5.39.

HRMS (ESI): Anal. Calcd. for $C_{28}H_{55}O_5Si_2^+$ $[M+H]^+$ 527.3583, found 527.3556

IR (neat): $\nu_{max}$ $(cm^{-1})$ = 2953 (s, CH), 2930 (s, CH), 2857 (s, CH), 1739 (s, C=O), 1469 (m), 1437 (m), 1360 (m), 1254 (s), 1235 (m)

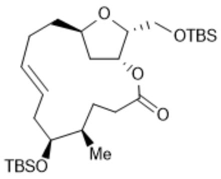
O
OTBS
O
O
TBSO
Me

**S98**

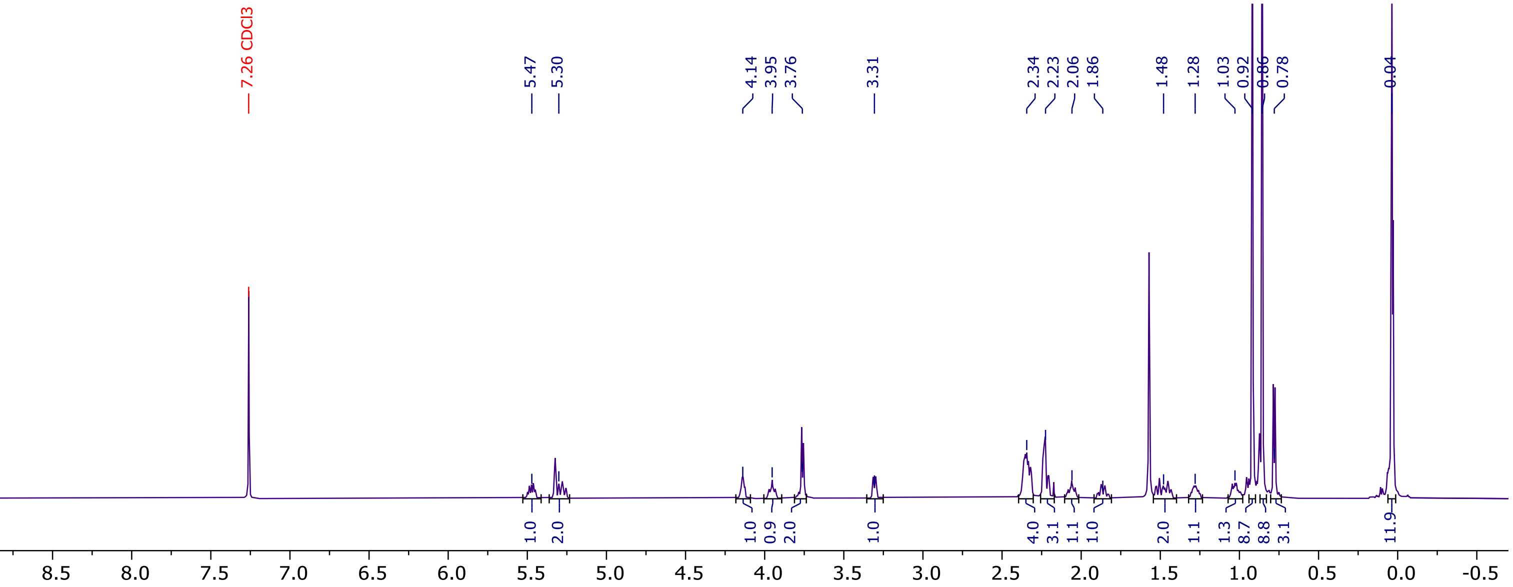
1H
CDCl3
600.13 MHz
7.26 CDCl3
5.47
5.30
4.14
3.95
3.76
3.31
2.34
2.23
2.06
1.86
1.48
1.28
1.03
0.92
0.86
0.78
0.04
1.0
2.0
1.0
0.9
2.0
1.0
4.0
3.1
1.1
1.0
2.0
1.1
1.3
8.7
8.8
3.1
11.9
10.0
9.5
9.0
8.5
8.0
7.5
7.0
6.5
6.0
5.5
5.0
4.5
4.0
3.5
3.0
2.5
2.0
1.5
1.0
0.5
0.0
-0.5

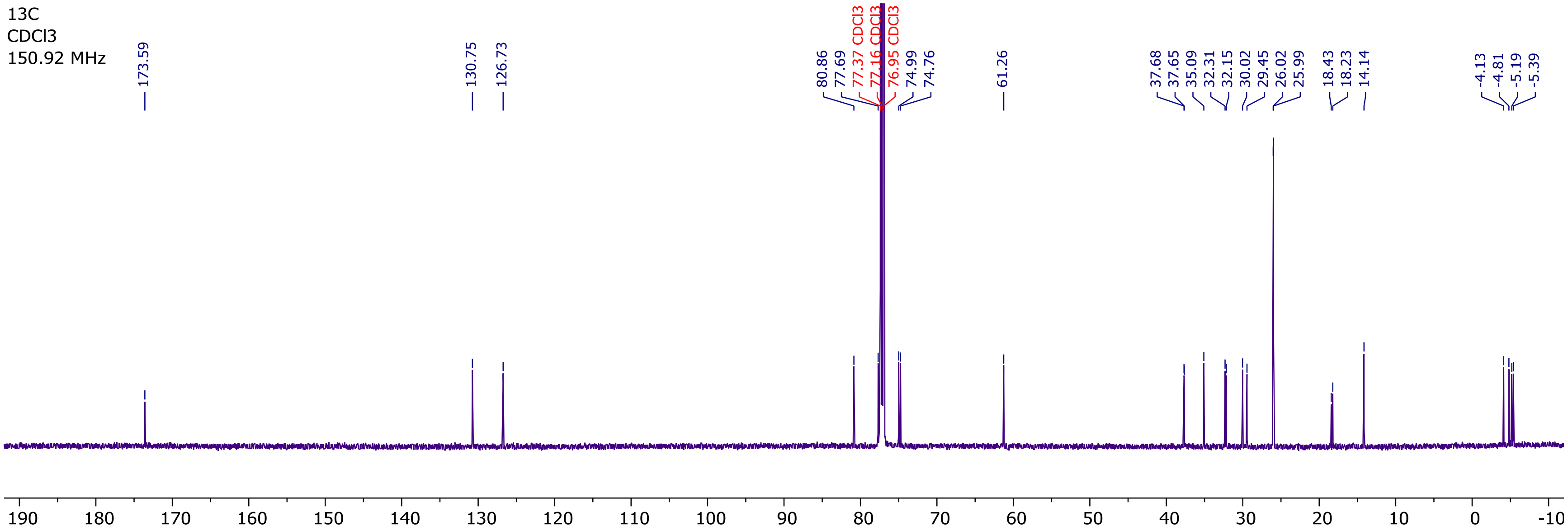
13C
CDCl3
150.92 MHz
173.59
130.75
126.73
80.86
77.69
77.37 CDCl3
77.16 CDCl3
76.95 CDCl3
74.99
74.76
61.26
37.68
37.65
35.09
32.31
32.15
30.02
29.45
26.02
25.99
18.43
18.23
14.14
-4.13
-4.81
-5.19
-5.39
190
180
170
160
150
140
130
120
110
100
90
80
70
60
50
40
30
20
10
0
-10

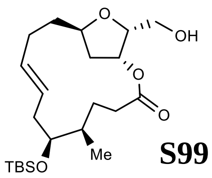


**S99**

Note: Reaction performed in capped vessel under ambient atmosphere.

To a plastic vessel containing **S98** (57 mg, 0.11 mmol, 1.0 eq.) and pyridine (1.68 mL) dissolved in anhydrous THF (0.2.3 mL) was added 70% HF/pyridine (0.42 mL, 16.2 mmol, 150 eq.). After 90 min. stirring at rt, the reaction was quenched with saturated $NaHCO_3$ (20 mL) and after effervescence had subsided, the aqueous layer extracted with $CH_2Cl_2$ (3x 20 mL). The combined organic layers were dried ($Na_2SO_4$), filtered, and the solvent was removed in vacuo. The crude product was purified via flash column chromatography (7:3 Petroleum ether (bp: 36-60)/EtOAc). Appropriate fractions were pooled to yield **S99** (41 mg, 93%) as a colorless oil.

**Analytical Data for S99:**

$R_f$ = 0.22 (3:2 Petroleum ether (bp: 36-60)/EtOAc)

$[\alpha]_D^{20}$ = +44 ° (c = 0.32, $CH_2Cl_2$)

$^1$H NMR (600 MHz, $CDCl_3$) δ 5.48 (dt, *J* = 14.7, 7.5 Hz, 1H), 5.33 (t, *J* = 4.0 Hz, 1H), 5.29 (ddd, *J* = 15.0, 11.0, 2.9 Hz, 1H), 4.22 (dt, *J* = 8.3, 4.4 Hz, 1H), 3.96 (tt, *J* = 11.5, 4.1 Hz, 1H), 3.84 (dd, *J* = 11.6, 7.4 Hz, 1H), 3.70 (dd, *J* = 11.6, 4.5 Hz, 1H), 3.31 (dt, *J* = 9.8, 3.0 Hz, 1H), 2.40 – 2.30 (m, 4H), 2.27 – 2.21 (m, 3H), 2.09 (tt, *J* = 13.2, 4.7 Hz, 1H), 1.88 (tdd, *J* = 14.0, 10.9, 4.4 Hz, 1H), 1.53 (ddd, *J* = 13.2, 11.7, 3.9 Hz, 1H), 1.47 (tt, *J* = 11.9, 3.8 Hz, 1H), 1.27 (dt, *J* = 17.2, 6.8 Hz, 1H), 1.10 – 0.99 (m, 1H), 0.91 (s, 9H), 0.90 – 0.86 (m, 1H), 0.78 (d, *J* = 6.5 Hz, 3H), 0.04 (s, 3H), 0.03 (s, 3H).

$^{13}$C NMR (151 MHz, $CDCl_3$) δ 173.51, 130.66, 126.81, 80.78, 77.43, 75.72, 74.74, 62.15, 38.09, 37.64, 35.03, 32.10, 32.05, 30.03, 29.36, 26.02, 18.22, 14.08, -4.17, -4.79.

HRMS (ESI): Anal. Calcd. for $C_{22}H_{44}NO_5Si^+$ $[M+NH_4]^+$ 430.2983, found 430.2974

IR (neat): $\nu_{max}$ $(cm^{-1})$ = 2953 (m, CH), 2930 (s, CH), 2890 (m, CH), 2857 (s, CH), 1739 (s, C=O), 1360 (m), 1254 (s), 1235 (s)

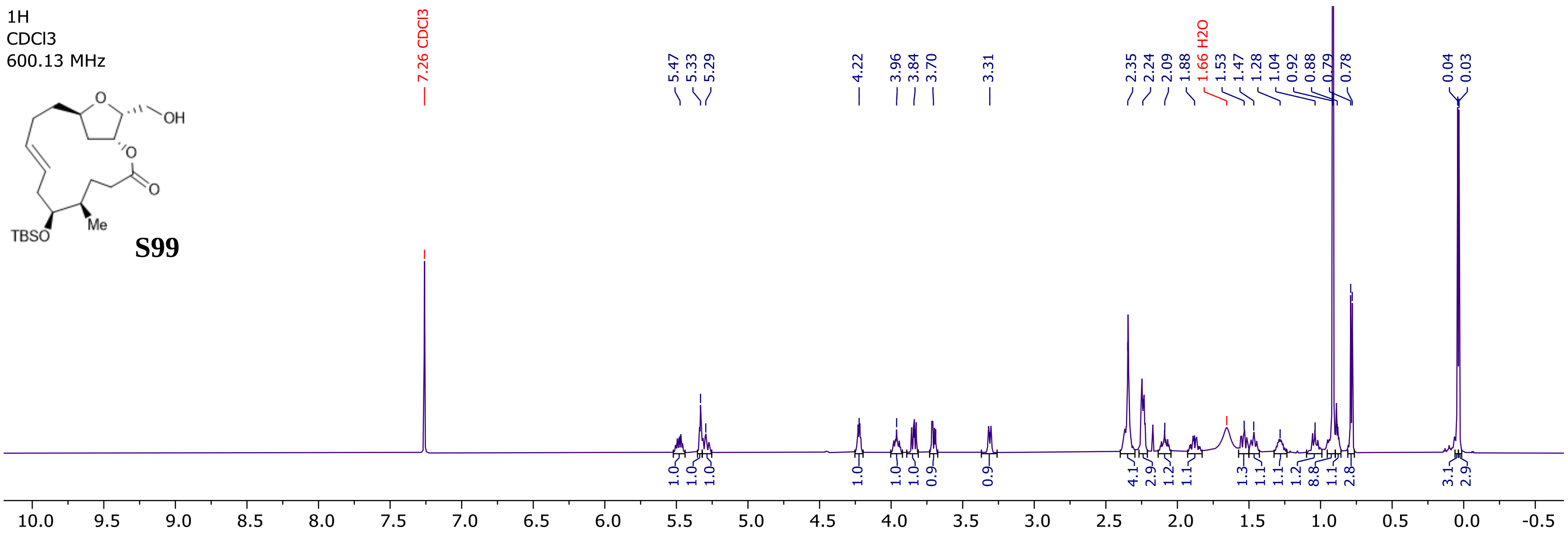
1H
CDCl3
600.13 MHz
OH
O
O
O
TBSO
Me
S99
7.26 CDCl3
5.47
5.33
5.29
4.22
3.96
3.84
3.70
3.31
2.35
2.24
2.09
1.88
1.66 H2O
1.53
1.47
1.28
1.04
0.92
0.88
0.79
0.78
0.04
0.03
1.0
1.0
1.0
1.0
1.0
1.0
0.9
0.9
4.1
2.9
1.2
1.1
1.3
1.1
1.1
1.2
8.8
1.1
2.8
3.1
2.9
10.0
9.5
9.0
8.5
8.0
7.5
7.0
6.5
6.0
5.5
5.0
4.5
4.0
3.5
3.0
2.5
2.0
1.5
1.0
0.5
0.0
-0.5

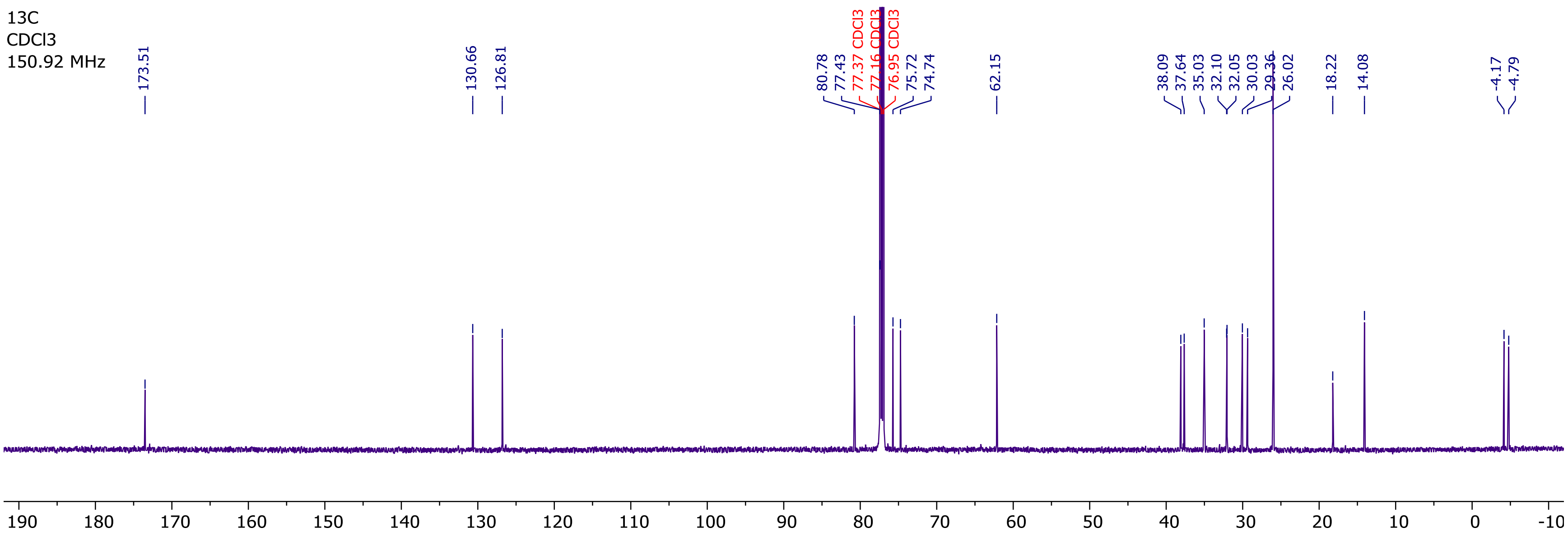
13C
CDCl3
150.92 MHz
173.51
130.66
126.81
80.78
77.43
77.37 CDCl3
77.16 CDCl3
76.95 CDCl3
75.72
74.74
62.15
38.09
37.64
35.03
32.10
32.05
30.03
29.36
26.02
18.22
14.08
-4.17
-4.79
190
180
170
160
150
140
130
120
110
100
90
80
70
60
50
40
30
20
10
0
-10

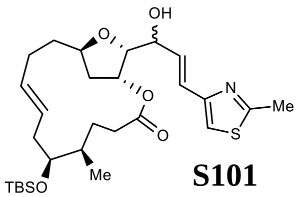


**S101**

To a stirred solution of **S99** (38 mg, 0.092 mmol, 1.0 eq.) in anhydrous $CH_2Cl_2$ (0.9 mL) was added $NaHCO_3$ (23 mg, 0.28 mmol, 3.0 eq.) followed by Dess-Martin Periodinane (59 mg, 0.14 mmol, 1.5 eq.). The mixture was stirred at rt for 1 h and quenched with a solution of 1:1:1 $H_2O$:saturated aqueous $NaHCO_3$:saturated aqueous $Na_2S_2O_3$ (3 mL). The aqueous layer was extracted with $CH_2Cl_2$ (3x 10 mL) and the combined organic layers were dried ($Na_2SO_4$), filtered, and the solvent was removed in vacuo to yield crude aldehyde **S100** (38 mg) as a colorless oil, which was used immediately for the next step.

To a solution of aldehyde **S100** (38 mg, 0.092 mmol, 1.0 eq.) in deoxygenated DMSO (deoxygenated via 5x freeze-pump-thaw cycles) (1.25 mL) was added $CrCl_2$ with doped with 1 % $NiCl_2$ (w/w) (113 mg, 0.92 mmol, 10 eq.). **3c** (61 mg, 0.24 mmol, 2.7 eq.) was added in deoxygenated DMSO (1.0 mL) and the mixture was stirred for 21 h. The reaction was cooled to 0 °C and quenched with 1:1 $H_2O$-brine (15 mL). The aqueous layer was extracted with EtOAc (8x 15 mL) and the combined organic layers were dried ($Na_2SO_4$) and filtered followed by removal of the solvent in vacuo. Residual DMSO was evaporated with air. The crude product was purified via flash column chromatography (89:11 to 87:13 Petroleum ether (bp: 36-60)/acetone). Appropriate fractions were pooled, and solvent was removed in vacuo to yield **S101** (25 mg, 51%) as a colorless oil and 5:3 mixture of diastereomers.

**Analytical Data for S101:**

$R_f$ = 0.31 (4:1 Petroleum ether (bp: 36-60)/acetone)

For the following reported NMR peaks: Several resonances in the $^1$H NMR spectrum integrated for 0.5 and 0.3 protons and are unique to both the diastereomers. In these cases an integral value of "0.5 H" and "0.3 H" is assigned. All resolved $^{13}$C NMR signals are reported.

$^1$H NMR (600 MHz, $CDCl_3$) δ 7.26 (s, 0.5H), 6.93 (d, *J* = 1.8 Hz, 0.5H), 6.89 (d, *J* = 1.8 Hz, 0.3H), 6.73 – 6.66 (m, 1.4H), 6.47 – 6.40 (m, 0.3H), 5.53 – 5.23 (m, 3.2H), 4.62 – 4.55 (m, 0.4H), 4.52 (d, *J* = 7.7 Hz, 0.6H), 4.07 – 4.00 (m, 1.5H), 3.96 (ddd, *J* = 8.6, 4.0, 1.8 Hz, 0.4H), 3.31 (ddt, *J* = 10.0, 4.9, 2.4 Hz, 1H), 2.70 (dd, *J* = 5.7, 1.8 Hz, 2.9H), 2.44 – 2.21 (m, 7.3H), 2.17 (t, *J* = 2.5 Hz, 0.3H), 2.10 (tt, *J* = 12.2, 6.1 Hz, 1.6H), 1.93 – 1.80 (m, 1.2H), 1.60 – 1.50 (m, 0.8H), 1.50 – 1.39 (m, 0.8H), 1.34 – 1.23 (m, 1.8H), 1.04 (dtd, *J* = 14.6, 7.5, 6.8, 3.6 Hz, 1.1H), 0.91 (d, *J* = 1.7 Hz, 9.2H), 0.80 (ddd, *J* = 11.0, 6.5, 1.9 Hz, 2.9H), 0.04 (q, *J* = 1.8 Hz, 6H).

$^{13}$C NMR (151 MHz, $CDCl_3$) δ 173.37, 166.18, 153.08, 132.17, 130.77, 130.71, 129.24, 126.79, 126.72, 124.76, 124.14, 115.41, 115.00, 84.03, 82.95, 78.29, 77.81, 75.90, 75.25, 74.77, 74.72, 71.15, 70.81, 38.33, 37.80, 37.67, 35.12, 35.07, 32.32, 32.26, 32.21, 32.18, 29.98, 29.96, 29.84, 29.43, 29.39, 26.03, 26.01, 19.38, 19.35, 18.22, 14.17, -4.16, -4.79.

HRMS (ESI): Anal. Calcd. for $C_{28}H_{46}NO_5SSi^+$ $[M+H]^+$ 536.2860, found 536.2838

IR (neat): $\nu_{max}$ ($cm^{-1}$) = 3351 (br, OH), 2929 (s, CH), 2857 (s, CH), 1736 (s, C=O), 1469 (w), 1437 (m), 1379 (w), 1359 (m), 1331 (w), 1254 (s), 1235 (s)

1H
CDCl3
600.13 MHz

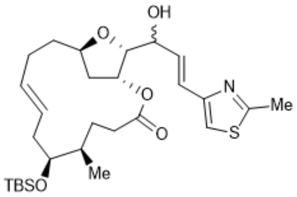


**S101**

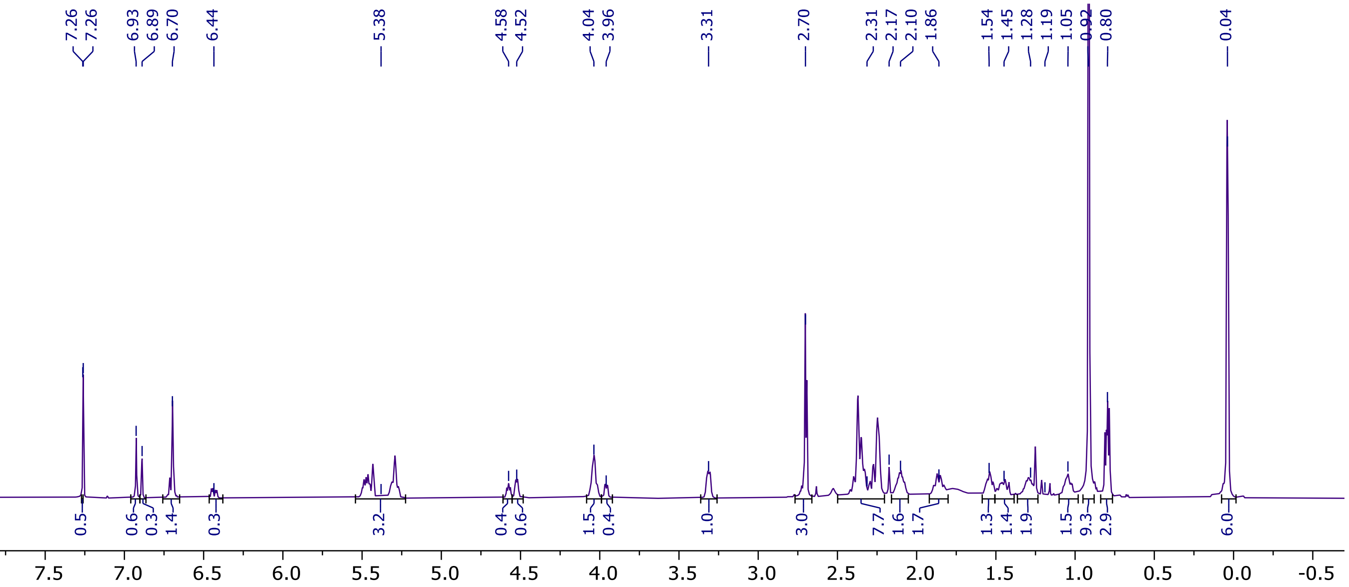


13C
CDCl3
150.92 MHz

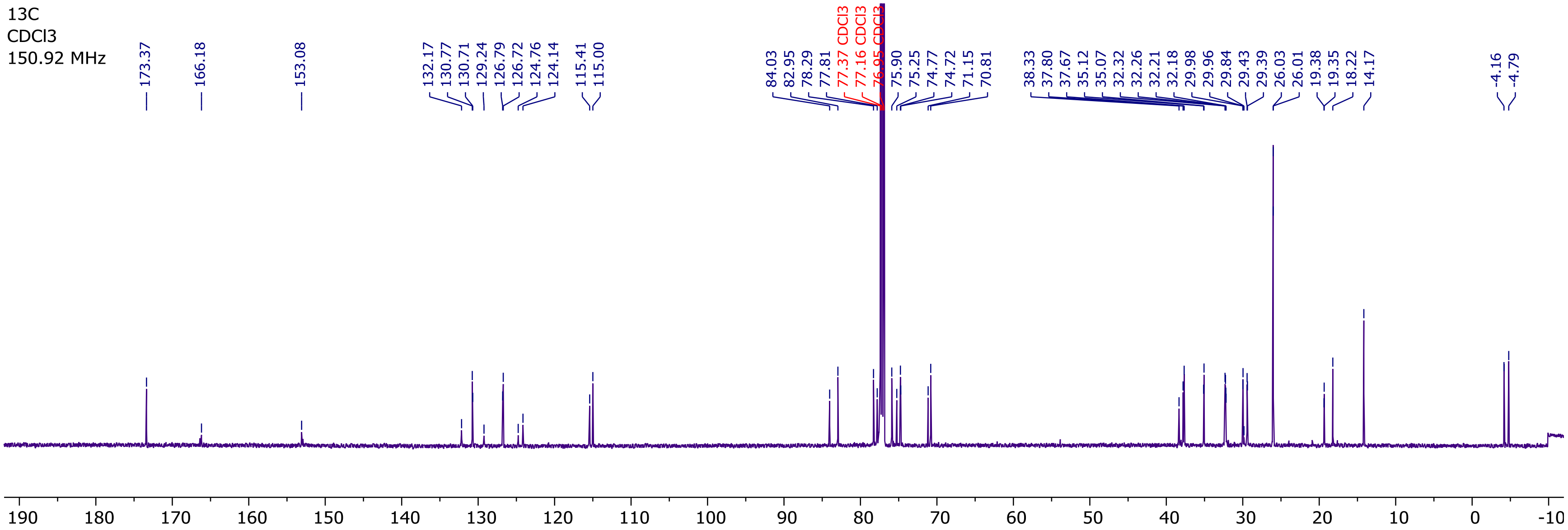

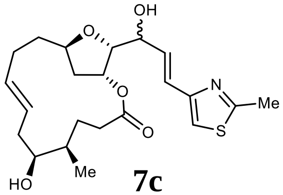


**7c**

Note: Reaction performed in capped vessel under ambient atmosphere.

To a plastic vessel containing **S101** (20 mg, 0.038 mmol, 1.0 eq.) dissolved in pyridine (0.4 mL) and anhydrous THF (0.4 mL) was added 70% HF/pyridine (0.23 mL, 8.8 mmol HF, 233 eq.). After 4 days stirring at rt, the reaction was quenched with saturated $NaHCO_3$ (15 mL) and after effervescence had subsided, the aqueous layer extracted with $CH_2Cl_2$ (3x 20 mL). The combined organic layers were dried ($Na_2SO_4$), filtered, and the solvent was removed in vacuo. The crude product was purified via flash column chromatography (4:1 to 3:2 hexanes/acetone). Appropriate fractions were pooled to yield **7c** (14.8 mg, 94%) as a colorless oil and a mixture of diastereomers (3:2).

**Analytical Data for 7c:**

$R_f$ = 0.13 (7:3 hexanes/acetone)

For the following reported NMR peaks: Several resonances in the $^1$H NMR spectrum integrated for 0.6 and 0.4 protons and are unique to both the diastereomers. In these cases an integral value of "0.6 H" and "0.4 H" is assigned.

$^1$H NMR (601 MHz, $CDCl_3$) δ 6.93 (s, 0.6H), 6.89 (s, 0.4H), 6.75 – 6.64 (m, 1.6H), 6.45 (dd, *J* = 15.6, 5.5 Hz, 0.4H), 5.52 – 5.44 (m, 1H), 5.42 (t, *J* = 3.5 Hz, 0.6H), 5.33 (ddd, *J* = 17.4, 11.3, 4.2 Hz, 1H), 5.28 (t, *J* = 3.7 Hz, 0.4H), 4.57 (ddd, *J* = 8.5, 5.5, 1.5 Hz, 0.4H), 4.51 (dd, *J* = 7.8, 4.0 Hz, 0.6H), 4.07-3.99 (m, 1.6H), 3.96 (dd, *J* = 8.4, 3.8 Hz, 0.4H), 3.36 (ddt, *J* = 10.1, 6.2, 3.0 Hz, 1H), 2.71 (s, 1.8H), 2.70 (s, 1.2H), 2.61 (d, *J* = 10.6 Hz, 0.4H), 2.45 – 2.30 (m, 7.6H), 2.10 (m, 1H), 1.92 – 1.81 (m, 1H), 1.59 – 1.49 (m, 1H), 1.49 – 1.40 (m, 1H), 1.20 (tdt, *J* = 12.2, 7.6, 3.9 Hz, 1H), 0.84 (d, *J* = 6.1 Hz, 1.2H), 0.82 (d, *J* = 6.3 Hz, 1.8H).

Major diastereomer

$^{13}$C NMR (151 MHz, $CDCl_3$) δ 173.82, 166.34, 152.94, 132.59, 131.61, 125.76, 123.90, 114.91, 83.07, 78.16, 76.10, 74.23, 70.56, 37.69, 37.54, 34.83, 32.23, 32.13, 29.93, 28.88, 19.23, 14.37.

HRMS (ESI): Anal. Calcd. for $C_{22}H_{32}NO_5S^+$ $[M+H]^+$ 422.1996, found 422.1986

IR (neat): $\nu_{max}$ $(cm^{-1})$ = 3394 (br, OH), 2980 (m, CH), 2927 (s, CH), 2863 (m, CH), 1725 (s, C=O), 1710 (s, C=O), 1502 (w), 1446 (m), 1357 (m), 1319 (m), 1238 (s)

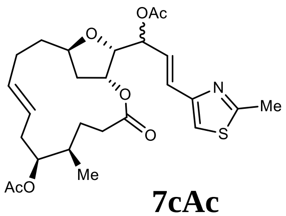


**7cAc**

**7cAc** was prepared according to general procedure A (1.3 mg, 95%).

**Analytical Data for 7cAc:**

$R_f$ = 0.29 and 0.34 (3:1 hexanes/acetone)

HRMS (ESI): Anal. Calcd. for $C_{26}H_{36}NO_7S^+$ $[M+H]^+$ 506.2207, found 506.2183

**7c**

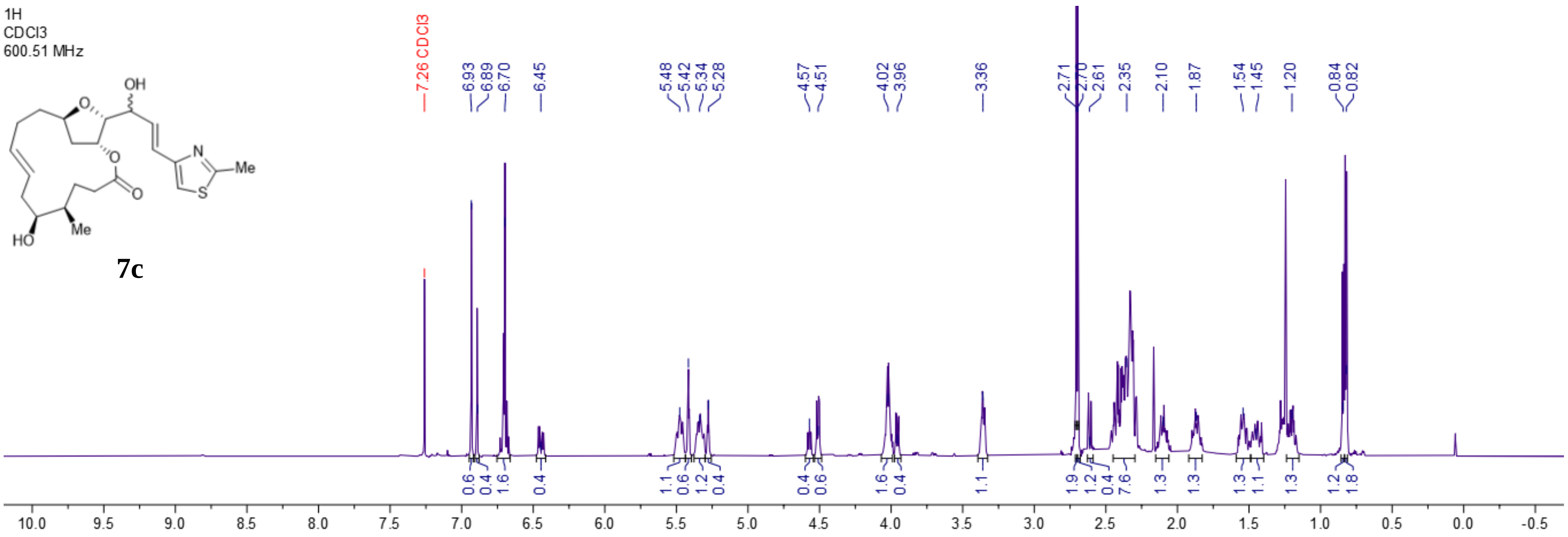
OH
O
O
O
N
S
Me
HO
Me
1H
CDCl3
600.51 MHz
7.26 CDCl3
6.93
6.89
6.70
6.45
5.48
5.42
5.34
5.28
4.57
4.51
4.02
3.96
3.36
2.71
2.70
2.61
2.35
2.10
1.87
1.54
1.45
1.20
0.84
0.82
0.6
0.4
1.6
0.4
1.1
0.6
1.2
0.4
0.4
0.6
1.6
0.4
1.1
1.9
1.2
0.4
7.6
1.3
1.3
1.3
1.1
1.3
1.2
1.8

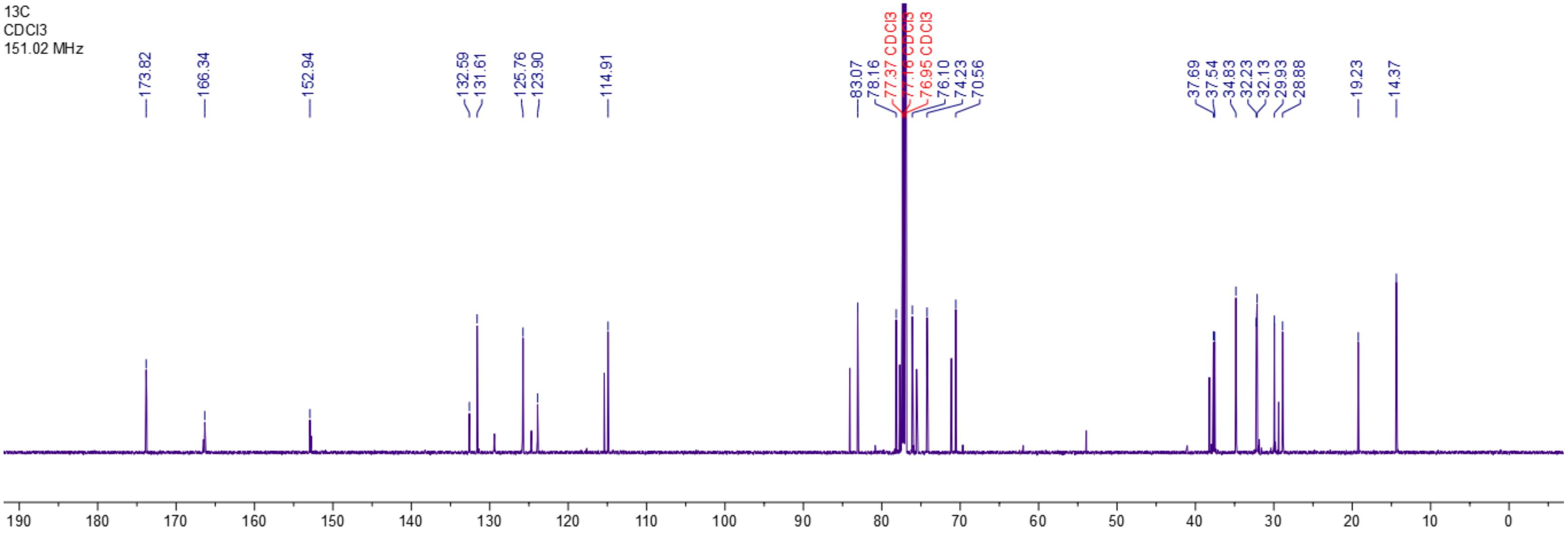
13C
CDCl3
151.02 MHz
173.82
166.34
152.94
132.59
131.61
125.76
123.90
114.91
83.07
78.16
77.37 CDCl3
77.16 CDCl3
76.95 CDCl3
76.10
74.23
70.56
37.69
37.54
34.83
32.23
32.13
29.93
28.88
19.23
14.37

Note: Reaction run under ambient atmosphere
To a plastic vessel containing a stirred solution of **S92** (84.5 mg, 0.16 mmol, 1.0 eq.) in anhydrous THF (2 mL) was added a stock solution of HF in THF-Pyridine (2.56 M HF, 6.4 mL, 16.4 mmol, 102 eq. HF, 4:1 pyridine:HF). After stirring for 16 h at rt, the reaction was quenched with saturated $NaHCO_3$ (20 mL) and the aqueous layer was extracted with $CH_2Cl_2$ (3x 25 mL) and the combined organic layers were washed with brine (20 mL), dried ($Na_2SO_4$), filtered, and the solvent was removed in vacuo. The crude product was purified via flash column chromatography (1:1 hexanes/EtOAc). Appropriate fractions were pooled, and solvent was removed in vacuo to yield **S93** (51.3 mg, 77%) as a white solid.

Note: Reaction run under ambient atmosphere
To a stirred solution of **S93** (45.8 mg, 0.11 mmol, 1.0 eq.) in anhydrous $CH_2Cl_2$ (1.3 mL) was added $NaHCO_3$ (66.7 mg, 0.79 mmol, 7.2 eq.) followed by Dess-Martin Periodinane (95.2 mg, 0.22 mmol, 2.0 eq.). The mixture was stirred at rt for 2 h and quenched with a solution of 1:1:1 $H_2O$-saturated $NaHCO_3$-saturated $Na_2S_2O_3$ (2 mL) and the biphasic mixture was stirred vigorously for 0.5 h. The aqueous layer was extracted with $CH_2Cl_2$ (3x 3 mL) and the combined organic layers were dried ($Na_2SO_4$), filtered, and the solvent was removed in vacuo. The crude product was run through a plug of silica gel with EtOAc and solvent was removed in vacuo to yield the crude aldehyde **S94** (*ca.* 45.8 mg) which was divided into 3 equal portions for the following step.

To a solution of **S94** (15.2 mg, 0.036 mmol, 1.0 eq.) in deoxygenated DMSO (deoxygenated via 5x freeze-pump-thaw cycles) (0.5 mL) was added $CrCl_2$ with doped with 1 % $NiCl_2$ (w/w) (46.7 mg, 0.38 mmol, 10.6 eq.). **3b** (15.7 mg, 0.062 mmol, 1.7 eq.) was added in deoxygenated DMSO (0.70 mL) and the mixture was stirred for 18 h. The reaction was cooled to 0 °C and quenched with 1:1 $H_2O$-brine (10 mL). The aqueous layer was extracted with $Et_2O$ (10x 10 mL) and the combined organic layers were dried ($MgSO_4$), filtered, and the solvent was removed in vacuo. The crude product was purified via flash column chromatography (17:3 hexanes/acetone). Appropriate fractions were pooled, and solvent was removed in vacuo to yield **S95** (8.8 mg, 45% over two steps) as a white solid and a mixture of diastereomers (d.r. >19:1). A portion of the material was carried forward to the next step.

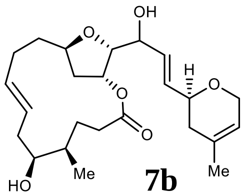

**7b**

Note: Reaction performed in capped vessel under ambient atmosphere.
To a vigorously stirred solution of **S95** (3.6 mg, 0.0067 mmol, 1.0 eq.) in $CH_2Cl_2$ (0.2 mL) and pH 7 phosphate buffer (0.2 mL) was added 2,3-dichloro-5,6-dicyano-benzoquinone (5.6 mg, 0.025 mmol, 3.7 eq.). After 3 h the starting material was consumed as monitored by TLC analysis and the reaction was quenched with saturated $NaHCO_3$ (3 mL). The aqueous layer was extracted with $CH_2Cl_2$ (3x 7 mL) and the combined organic layers were dried ($Na_2SO_4$) and filtered, followed by removal of the solvent in vacuo. The crude product was purified via

flash column chromatography (33:7 to 31:9 hexanes/acetone). Appropriate fractions were pooled, and solvent was removed in vacuo to yield **7b** (2.2 mg, 78%) as a colorless oil and a mixture of diastereomers (d.r. >19:1).

**Analytical Data for 7b:**

$R_f$ = 0.26 (13:7 hexanes/acetone)

$[\alpha]_D^{20}$ = +19 ° (c = 0.22, $CH_3CN$)

$^1$H NMR (601 MHz, $CD_3CN$) δ 5.83 (ddd, *J* = 15.7, 5.0, 1.0 Hz, 1H), 5.77 (ddd, *J* = 15.7, 5.1, 1.0 Hz, 1H), 5.51-5.41 (m, 2H), 5.35 (dddd, *J* = 15.4, 10.6, 3.0, 1.8 Hz, 1H), 5.25 (t, *J* = 3.2 Hz, 1H), 4.20 (dt, *J* = 8.8, 4.0 Hz, 1H), 4.13-4.07 (m, *J* = 4.6, 3.4, 2.8, 1.7 Hz, 2H), 3.99 (dddt, *J* = 9.7, 4.7, 3.7, 1.0 Hz, 1H), 3.92 (tt, *J* = 11.5, 4.1 Hz, 1H), 3.75 (dd, *J* = 8.9, 3.5 Hz, 1H), 3.27 – 3.22 (m, 1H), 3.00 (d, *J* = 5.4 Hz, 1H), 2.71 (d, *J* = 4.9 Hz, 1H), 2.48 – 2.41 (m, 1H), 2.33 – 2.25 (m, 4H), 2.25 – 2.20 (m, 1H), 2.19-2.16 (m, 1H), 2.02 – 1.95 (m, 2H), 1.91 – 1.82 (m, 2H), 1.68 (dt, *J* = 2.6, 1.3 Hz, 3H), 1.53 (ddd, *J* = 13.3, 11.7, 3.5 Hz, 1H), 1.42 – 1.35 (m, 1H), 1.21 (dddd, *J* = 11.9, 10.0, 6.3, 1.8 Hz, 1H), 1.15 – 1.07 (m, 1H), 0.80 (d, *J* = 6.3 Hz, 3H).

$^{13}$C NMR (151 MHz, $CD_3CN$) δ 174.44, 132.67, 132.53, 132.26, 132.00, 127.23, 120.77, 84.52, 78.63, 76.07, 74.30, 74.20, 69.76, 66.15, 38.35, 38.13, 36.46, 35.36, 33.13, 32.71, 30.38, 29.71, 23.04, 14.30.

HRMS (ESI): Anal. Calcd. for $C_{24}H_{40}NO_6^+$ $[M+NH_4]^+$ 438.2850, found 438.2874

IR (neat): $\nu_{max}$ $(cm^{-1})$ = 3448 (br, OH), 2973 (m, CH), 2928 (m, CH), 2858 (m, CH), 1725 (s, C=O), 1437 (m), 1360 (m), 1239 (s)

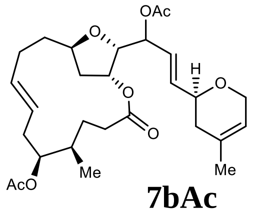


**7bAc**

7bAc was prepared according to general procedure A (0.93 mg, 65%).

**Analytical Data for 7bAc:**

$R_f$ = 0.91 (2:3 hexanes/EtOAc)

HRMS (ESI): Anal. Calcd. for $C_{28}H_{44}NO_8^+$$[M+NH_4]^+$ 522.3061, found 522.3071

1H
CD3CN
600.51 MHz

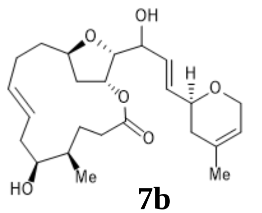

**7b**

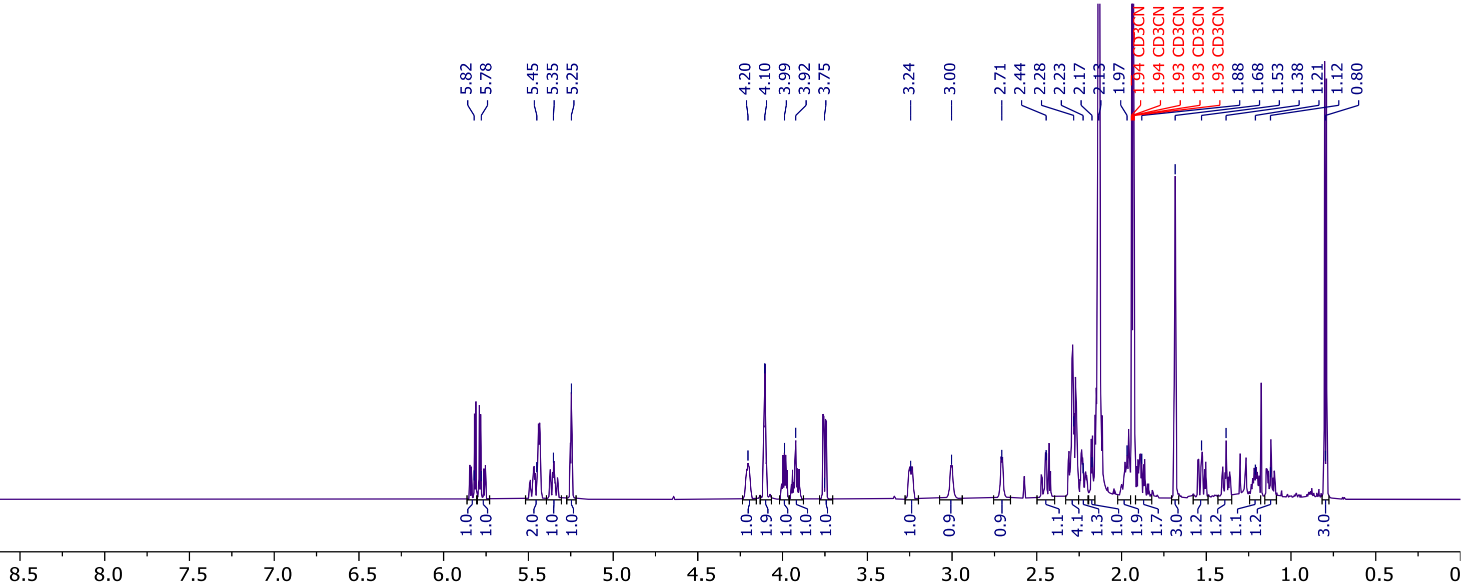

13C
CD3CN
151.02 MHz

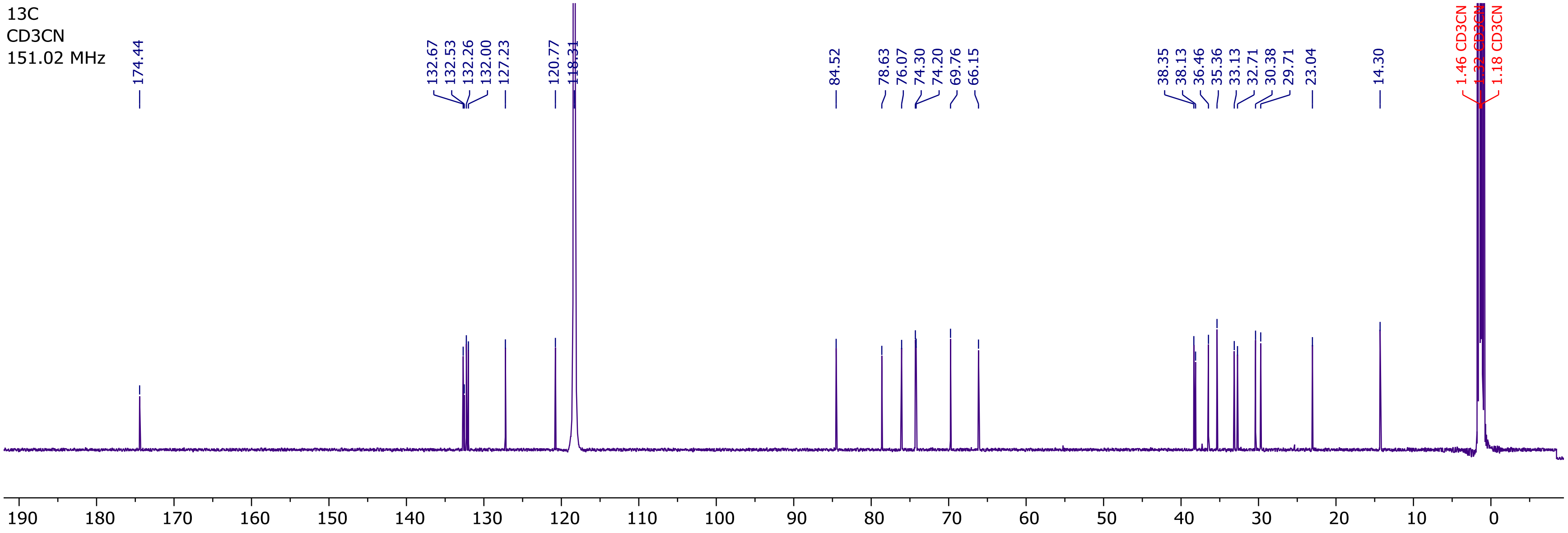

To a solution of **S94** (15.2 mg, 0.036 mmol, 1.0 eq.) in anhydrous deoxygenated DMSO (deoxygenated via 5x freeze-pump-thaw cycles) (0.5 mL) was added $CrCl_2$ with doped with 1 % $NiCl_2$ (w/w) (52.8 mg, 0.43 mmol, 11.9 eq.). **3a** (17.4 mg, 0.073 mmol, 2.0 eq.) was added in deoxygenated DMSO (0.80 mL) and the mixture was stirred for 18 h. The reaction was cooled to 0 °C and quenched with 1:1 $H_2O$-brine (10 mL). The aqueous layer was extracted with $Et_2O$ (10x 10 mL) and the combined organic layers were dried ($MgSO_4$), filtered, and the solvent was removed in vacuo. The crude product was purified via flash column chromatography (1:1 hexanes/acetone). Appropriate fractions were pooled, and solvent was removed in vacuo to yield **S96** (16.3 mg, 0.031 mmol, 84% over two steps) as a colorless oil and mixture of diastereomers (3:1).

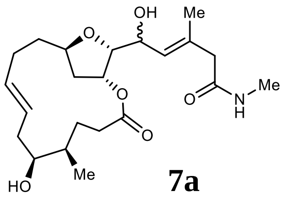


**7a**

Note: Reaction performed in capped vessel under ambient atmosphere.
To a vigorously stirred solution of **S96** (16.3 mg, 0.0308 mmol, 1.0 eq.) in $CH_2Cl_2$ (2.5 mL) and pH 7 phosphate buffer (0.5 mL) was added 2,3-dichloro-5,6-dicyano-benzoquinone (34.6 mg, 0.152 mmol, 5.0 eq.). After 90 minutes the starting material was consumed as monitored by TLC analysis and the reaction was quenched with saturated $NaHCO_3$ (4 mL). The aqueous layer was extracted with $CH_2Cl_2$ (3x 5 mL) and the combined organic layers were dried ($Na_2SO_4$) and filtered, followed by removal of the solvent in vacuo. The crude product was purified via flash column chromatography (1:1 to 1:4 hexanes/acetone). Appropriate fractions were pooled, and solvent was removed in vacuo to yield **7a** (4.8 mg, 38%) as a colorless oil and a mixture of diastereomers (3:1).

**Analytical Data for 7a:**

$R_f$ = 0.25 (2:3 hexanes/acetone)

For the following reported NMR peaks: Several resonances in the $^1$H NMR spectrum integrated for 0.3 protons and are unique to the minor diastereomer. In these cases an integral value of "0.3 H" is assigned.

$^1$H NMR (601 MHz, $CD_3CN$) δ 6.47 (s, 1H), 6.08 (s, 0.3H), 5.51 – 5.42 (m, 1.3H), 5.40 – 5.29 (m, 2.9H), 5.26 (m, 1H), 5.16 (dq, *J* = 9.6, 1.3 Hz, 0.3H), 5.08 (t, *J* = 3.5 Hz, 0.3H), 4.55 (td, *J* = 8.8, 4.5 Hz, 1H), 4.48 – 4.42 (m, 0.3H), 3.97 (tt, *J* = 11.5, 4.1 Hz, 0.3H), 3.93 (t, *J* = 3.9 Hz, 0.3H), 3.92 – 3.88 (m, 1H), 3.83 (dd, *J* = 8.9, 3.7 Hz, 1H), 3.28 – 3.21 (m, 1.3H), 2.94 – 2.91 (m, 1.3H), 2.90 – 2.87 (m, *J* = 3.8 Hz, 1H), 2.82 – 2.78 (m, 2.1H), 2.73 – 2.68 (m, *J* = 7.8, 5.0 Hz, 1.3H), 2.65 (d, *J* = 4.8 Hz, 3H), 2.62 (d, *J* = 4.8 Hz, 0.9H), 2.52 – 2.46 (m, 1.3H), 2.32 – 2.16 (m, 8.2H), 1.70 (dd, *J* = 3.4, 1.4 Hz, 3.9H), 1.62 – 1.47 (m, 1.3H), 1.45 – 1.34 (m, 1.6H), 1.18 – 1.08 (m, 3H), 0.80 (m, 3.9H).

Major diastereomer:

$^{13}$C NMR (151 MHz, $CD_3CN$) δ 174.36, 171.64, 134.65, 132.09, 132.00, 127.22, 84.52, 78.52, 75.82, 74.32, 66.37, 47.83, 38.51, 38.37, 35.33, 33.18, 32.70, 30.37, 29.65, 26.38, 17.49, 14.16.

Minor diastereomer

$^{13}$C NMR (151 MHz, $CD_3CN$) δ 174.20, 171.42, 135.90, 132.72, 131.92, 127.86, 85.08, 78.39, 76.29, 74.28, 68.55, 47.88, 38.54, 38.33, 35.31, 33.20, 33.07, 30.45, 29.71, 26.33, 17.35, 14.31.

HRMS (ESI): Anal. Calcd. for $C_{22}H_{36}NO_6^+$ $[M+H]^+$ 410.2537, found 410.2536

IR (neat): $\nu_{max}$ $(cm^{-1})$ = 3347 (br, OH), 2927 (s, CH), 2858 (m, CH), 1729 (s, C=O), 1657 (s, C=C), 1644 (s, C=C), 1548 (m), 1439 (m), 1356 (w), 1318 (w), 1239 (s)

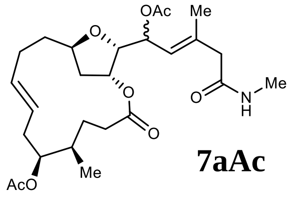


**7aAc**

**7aAc** was prepared according to general procedure A (0.85 mg, 63%).

**Analytical Data for 7aAc:**

$R_f$ = 0.26 (EtOAc)

HRMS (ESI): Anal. Calcd. for $C_{26}H_{40}NO_8^+$ $[M+H]^+$ 494.2748, found 494.2757

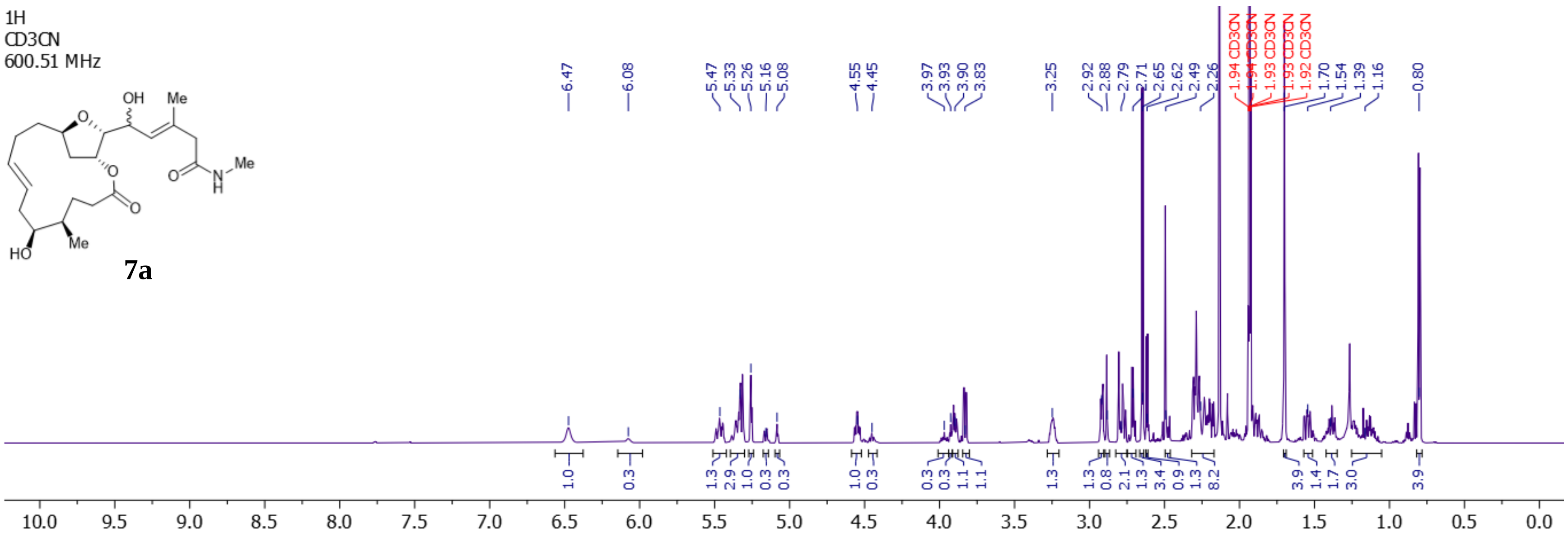
1H
CD3CN
600.51 MHz
7a

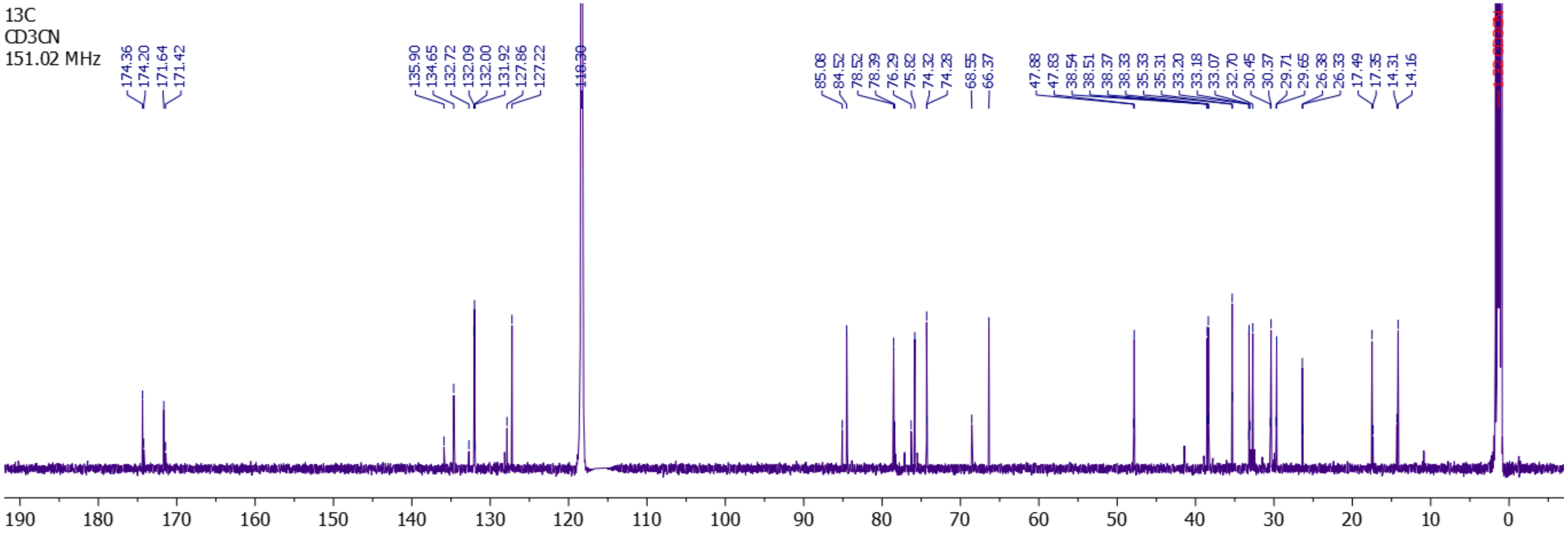
13C
CD3CN
151.02 MHz

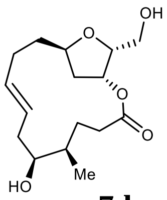


**7d**

Note: Reaction performed in capped vessel under ambient atmosphere.

To a vigorously stirred solution of **S93** (2.7 mg, 0.0065 mmol, 1.0 eq.) in $CH_2Cl_2$ (0.5 mL) and pH 7 aqueous phosphate buffer (0.2 mL) was added 2,3-dichloro-5,6-dicyano-benzoquinone (5.3 mg, 0.023 mmol, 3.5 eq.). After 1 h the starting material was consumed as monitored by TLC analysis and the reaction was quenched with saturated $NaHCO_3$ (2 mL). The aqueous layer was extracted with $CH_2Cl_2$ (3x 5 mL) and the combined organic layers were dried ($Na_2SO_4$), filtered, and the solvent was removed in vacuo. The crude product was purified via flash column chromatography (7:3 to 3:2 hexanes/acetone). Appropriate fractions were pooled, and solvent was removed in vacuo to yield **7d** (1.9 mg, 97%) as a colorless oil.

**Analytical Data for 7d:**

$R_f$ = 0.22 (3:2 hexanes/acetone)

$[\alpha]_D^{20}$ = +1.9 ° (c = 0.19, $CH_3CN$)

$^1$H NMR (601 MHz, $CD_3CN$) δ 5.47 (dddd, *J* = 14.4, 10.9, 3.4, 2.1 Hz, 1H), 5.39 – 5.32 (m, 1H), 5.24 – 5.21 (m, 1H), 4.07 (td, *J* = 6.5, 3.9 Hz, 1H), 3.88 (tt, *J* = 11.5, 4.0 Hz, 1H), 3.65 – 3.56 (m, 2H), 3.27 – 3.21 (m, 1H), 2.69 (d, *J* = 5.0 Hz, 1H), 2.65 (t, *J* = 6.1 Hz, 1H), 2.44 – 2.39 (m, 1H), 2.33 – 2.19 (m, 6H), 2.02 – 1.96 (m, 1H), 1.91 – 1.81 (m, 1H), 1.52 (ddd, *J* = 13.3, 11.7, 3.7 Hz, 1H), 1.44 – 1.35 (m, 1H), 1.26 – 1.16 (m, 1H), 1.16 – 1.07 (m, 1H), 0.79 (d, *J* = 6.4 Hz, 3H).

$^{13}$C NMR (151 MHz, $CD_3CN$) δ 174.36, 131.99, 127.13, 81.78, 77.83, 76.20, 74.26, 61.44, 38.32, 38.29, 35.25, 32.95, 30.43, 29.60, 14.18.

HRMS (ESI): Anal. Calcd. for $C_{16}H_{27}O_5^+$ $[M+H]^+$ 299.1853, found 299.1857

IR (neat): $\nu_{max}$ $(cm^{-1})$ = 3408 (br, OH), 2926 (s, CH), 2859 (m, CH), 1726 (s, C=O), 1439 (s), 1360 (m), 1318 (m), 1239 (s)

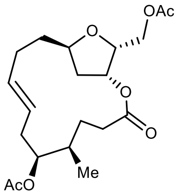


**7dAc**

**7dAc** was prepared according to general procedure A (0.86 mg, 71%).

**Analytical Data for 7dAc:**

$R_f$ = 0.75 (2:3 hexanes/EtOAc)

HRMS (ESI): Anal. Calcd. for $C_{20}H_{34}NO_7^+$ $[M+NH_4]^+$ 400.2330, found 400.2337

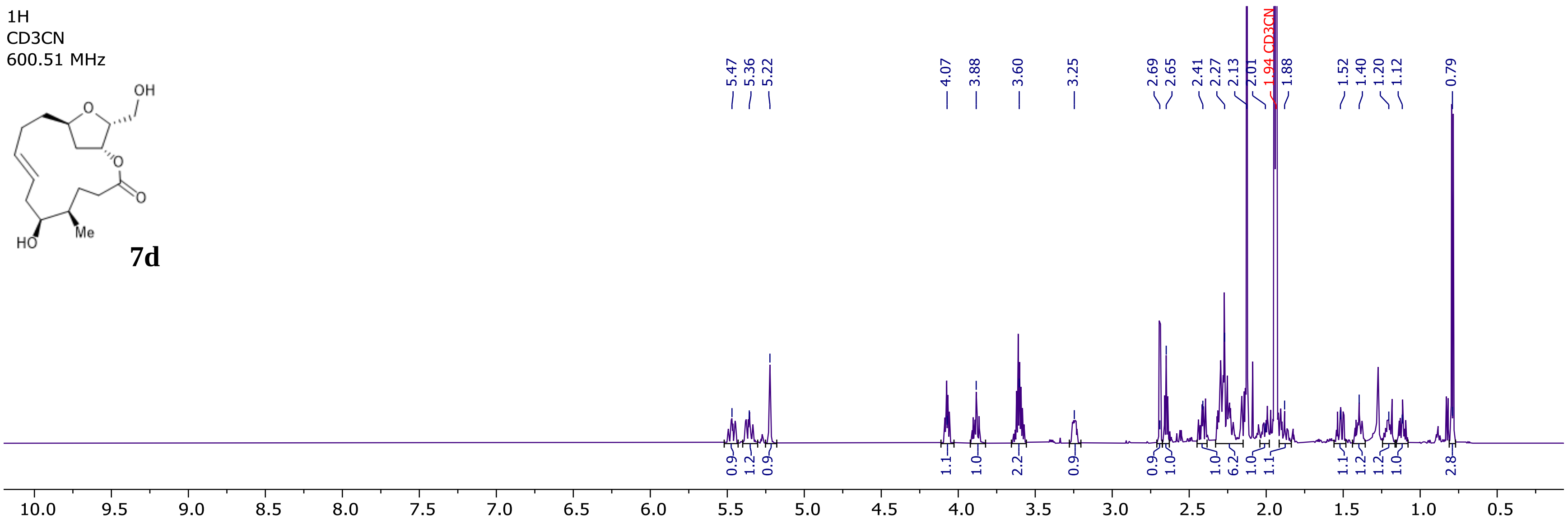
1H
CD3CN
600.51 MHz
OH
O
O
O
HO
Me
7d
5.47 5.36 5.22 4.07 3.88 3.60 3.25 2.69 2.65 2.41 2.27 2.13 2.01 1.94 CD3CN 1.88 1.52 1.40 1.20 1.12 0.79
0.9 1.2 0.9 1.1 1.0 2.2 0.9 0.9 1.0 1.0 6.2 1.0 1.1 1.1 1.2 1.2 1.0 2.8
10.0 9.5 9.0 8.5 8.0 7.5 7.0 6.5 6.0 5.5 5.0 4.5 4.0 3.5 3.0 2.5 2.0 1.5 1.0 0.5

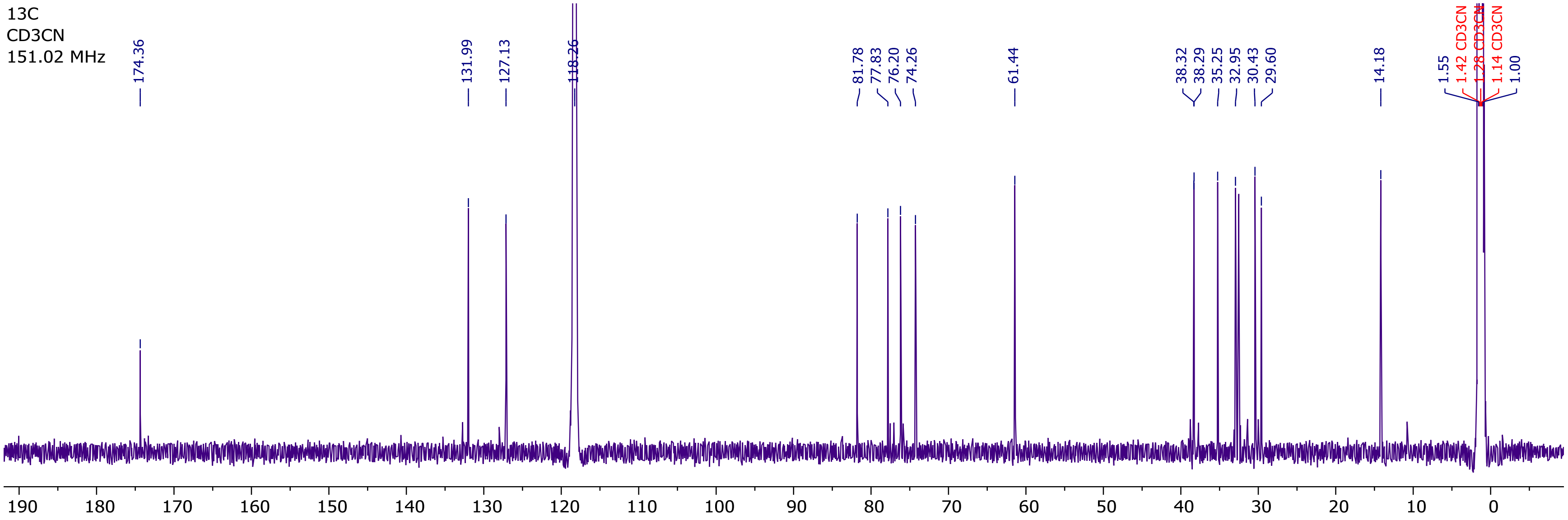
13C
CD3CN
151.02 MHz
174.36 131.99 127.13 118.26 81.78 77.83 76.20 74.26 61.44 38.32 38.29 35.25 32.95 30.43 29.60 14.18 1.55 1.42 CD3CN 1.28 CD3CN 1.14 CD3CN 1.00
190 180 170 160 150 140 130 120 110 100 90 80 70 60 50 40 30 20 10 0

To a solution of **S94** (15.2 mg, 0.036 mmol, 1.0 eq.) in anhydrous deoxygenated DMSO (deoxygenated via 5x freeze-pump-thaw cycles) (0.5 mL) was added $CrCl_2$ with doped with 1 % $NiCl_2$ (w/w) (52.8 mg, 0.43 mmol, 11.9 eq.). **3c** (18.3 mg, 0.073 mmol, 2.0 eq.) was added in deoxygenated DMSO (0.80 mL) and the mixture was stirred for 18 h. The reaction was cooled to 0 °C and quenched with 1:1 $H_2O$-brine (10 mL). The aqueous layer was extracted with $Et_2O$ (10x 10 mL) and the combined organic layers were dried ($MgSO_4$), filtered, and the solvent was removed in vacuo. The crude product was purified via flash column chromatography (1:1 hexanes/acetone). Appropriate fractions were pooled, and solvent was removed in vacuo to yield **S38** (5.6 mg, 0.010 mmol, 28% over two steps) as a colorless oil.

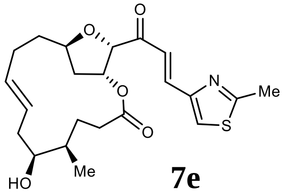


**7e**

Note: Reaction performed in capped vessel under ambient atmosphere.

To a vigorously stirred solution of **S38** (3.9 mg, 0.0093 mmol, 1.0 eq.) in $CH_2Cl_2$ (0.6 mL) and pH 7 aqueous phosphate buffer (0.3 mL) was added 2,3-dichloro-5,6-dicyano-benzoquinone (10.5 mg, 0.046 mmol, 5.0 eq.). After 1 h the starting material was consumed as monitored by TLC analysis and the reaction was quenched with saturated $NaHCO_3$ (10 mL). The aqueous layer was extracted with $CH_2Cl_2$ (3x 10 mL) and the combined organic layers were dried ($Na_2SO_4$), filtered, and the solvent was removed in vacuo. The crude product was purified via flash column chromatography (3:1 hexanes:acetone). Appropriate fractions were pooled, and solvent was removed in vacuo to yield **7e** (3.4 mg, 87%) as a clear oil.

**Analytical Data for 7e:**

$R_f$ = 0.13 (3:1 hexanes:acetone)

$[\alpha]_D^{20}$ = +74 ° (c = 0.12, $CH_3CN$)

$^1$H NMR (601 MHz, $CD_3CN$) δ 7.58 (s, 1H), 7.49 (dd, *J* = 15.5, 0.4 Hz, 1H), 7.25 (dd, *J* = 15.6, 0.5 Hz, 1H), 5.54 (t, *J* = 4.0 Hz, 1H), 5.53 – 5.40 (m, 2H), 4.72 (d, *J* = 4.6 Hz, 1H), 4.15 (tt, *J* = 11.5, 4.0 Hz, 1H), 3.25 – 3.18 (m, 1H), 2.69 (d, *J* = 5.0 Hz, 1H), 2.67 (s, 3H), 2.38 – 2.33 (m, 1H), 2.30 (dd, *J* = 10.8, 2.6 Hz, 1H), 2.26 – 2.19 (m, 3H), 2.20 – 2.13 (m, 3H), 1.58 (ddd, *J* = 13.3, 11.7, 3.8 Hz, 1H), 1.50 (dddd, *J* = 12.8, 11.2, 4.3, 3.0 Hz, 1H), 1.18 (dddd, *J* = 12.3, 10.0, 6.2, 2.2 Hz, 1H), 1.08 – 1.00 (m, 1H), 0.74 (d, *J* = 6.4 Hz, 3H).

$^{13}$C NMR (151 MHz, $CD_3CN$) δ 198.44, 173.82, 134.67, 131.91, 127.45, 124.85, 124.81, 86.04, 80.19, 77.99, 74.22, 38.35, 38.26, 35.31, 32.73, 32.40, 30.49, 29.48, 19.46, 14.20.

HRMS (ESI): Anal. Calcd. for $C_{22}H_{30}NO_5S^+$ $[M+H]^+$ 420.1839, found 420.1849

IR ($CDCl_3$): $\nu_{max}$ $(cm^{-1})$ = 3556 (br, OH), 2921 (s, CH), 2853 (m, CH), 1735 (s, C=O), 1677 (m, C=O)

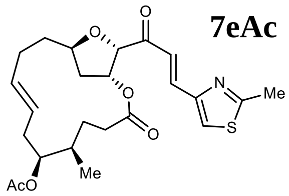

**7eAc**

**7eAc** was prepared according to general procedure A (0.58 mg, 53%).

**Analytical Data for 7eAc:**

$R_f$ = 0.29 (3:1 hexanes:acetone)

$[\alpha]_D^{20}$ = +28 ° (c = 0.26, $CH_2Cl_2$)

$^1$H NMR (600 MHz, $C_6D_6$) δ 7.93 (dd, *J* = 15.4, 1.7 Hz, 1H), 7.74 (dd, *J* = 15.3, 1.7 Hz, 1H), 6.45 (d, *J* = 1.9 Hz, 1H), 5.51 (t, *J* = 4.0 Hz, 1H), 5.41 – 5.30 (m, 1H), 4.96 (ddd, *J* = 15.6, 10.5, 2.6 Hz, 1H), 4.76 – 4.64 (m, 1H), 4.53 (dd, *J* = 4.6, 1.7 Hz, 1H), 4.18 (tt, *J* = 11.4, 3.7 Hz, 1H), 2.45 – 2.34 (m, 1H), 2.13 (d, *J* = 1.8 Hz, 3H), 2.11 – 1.91 (m, 5H), 1.88 – 1.80 (m, 1H), 1.66 (d, *J* = 1.8 Hz, 3H), 1.60 – 1.44 (m, 2H), 1.26 – 1.19 (m, 1H), 1.01 – 0.85 (m, 2H), 0.77 (ddt, *J* = 16.2, 12.4, 3.3 Hz, 1H), 0.70 (dd, *J* = 6.5, 1.8 Hz, 3H).

$^{13}$C NMR (151 MHz, $C_6D_6$) δ 197.84, 172.41, 169.74, 166.28, 152.63, 134.04, 132.41, 125.57, 124.60, 122.82, 85.78, 79.29, 77.61, 75.83, 37.86, 34.78, 32.72, 32.07, 31.61, 30.00, 28.88, 20.63, 18.82, 13.66.

IR (neat): $\nu_{max}$ $(cm^{-1})$ = 2921 (s, CH), 2851 (w, CH), 1727 (s, C=O), 1606 (s, C=O), 1437 (m), 1241 (s), 1221 (s)

HRMS (ESI): Anal. Calcd. for $C_{24}H_{32}NO_6S^+$ $[M+H]^+$ 462.1945, found 462.1939

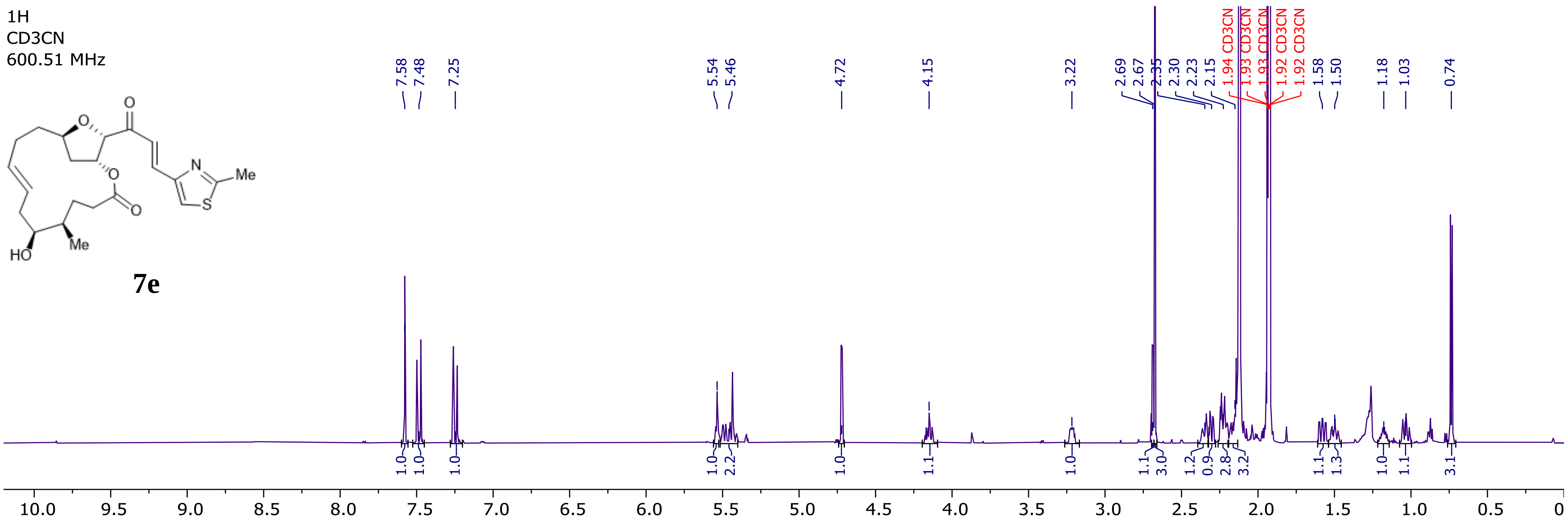
1H
CD3CN
600.51 MHz
7e

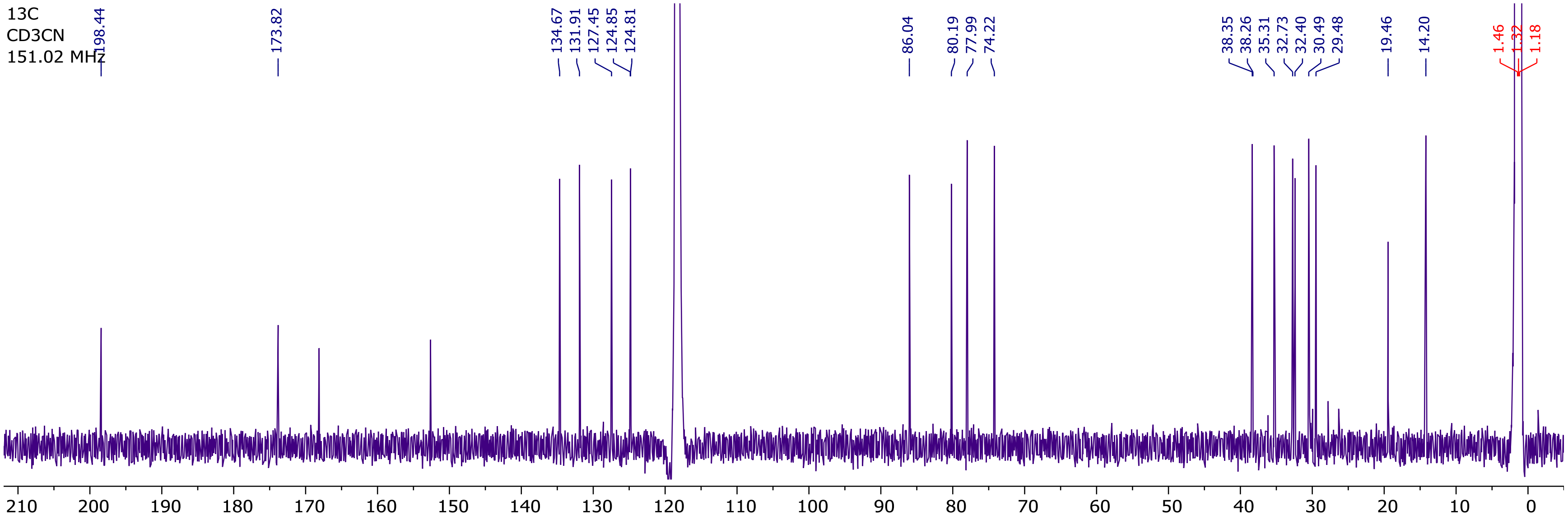
13C
CD3CN
151.02 MHz

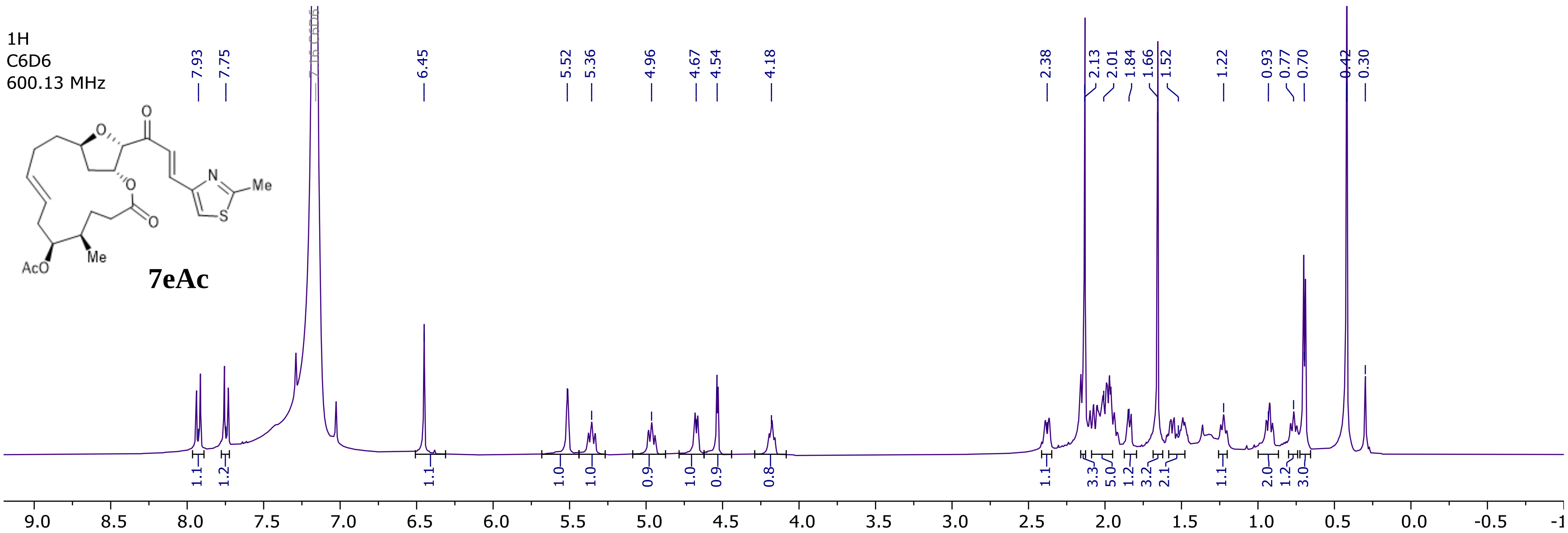
1H
C6D6
600.13 MHz
7eAc
AcO
Me
7.93
7.75
7.16 C6D6
6.45
5.52
5.36
4.96
4.67
4.54
4.18
2.38
2.13
2.01
1.84
1.66
1.52
1.22
0.93
0.77
0.70
0.42
0.30
1.1
1.2
1.1
1.0
1.0
0.9
1.0
0.9
0.8
1.1
3.3
5.0
1.2
3.2
2.1
1.1
2.0
1.2
3.0
9.0
8.5
8.0
7.5
7.0
6.5
6.0
5.5
5.0
4.5
4.0
3.5
3.0
2.5
2.0
1.5
1.0
0.5
0.0
-0.5

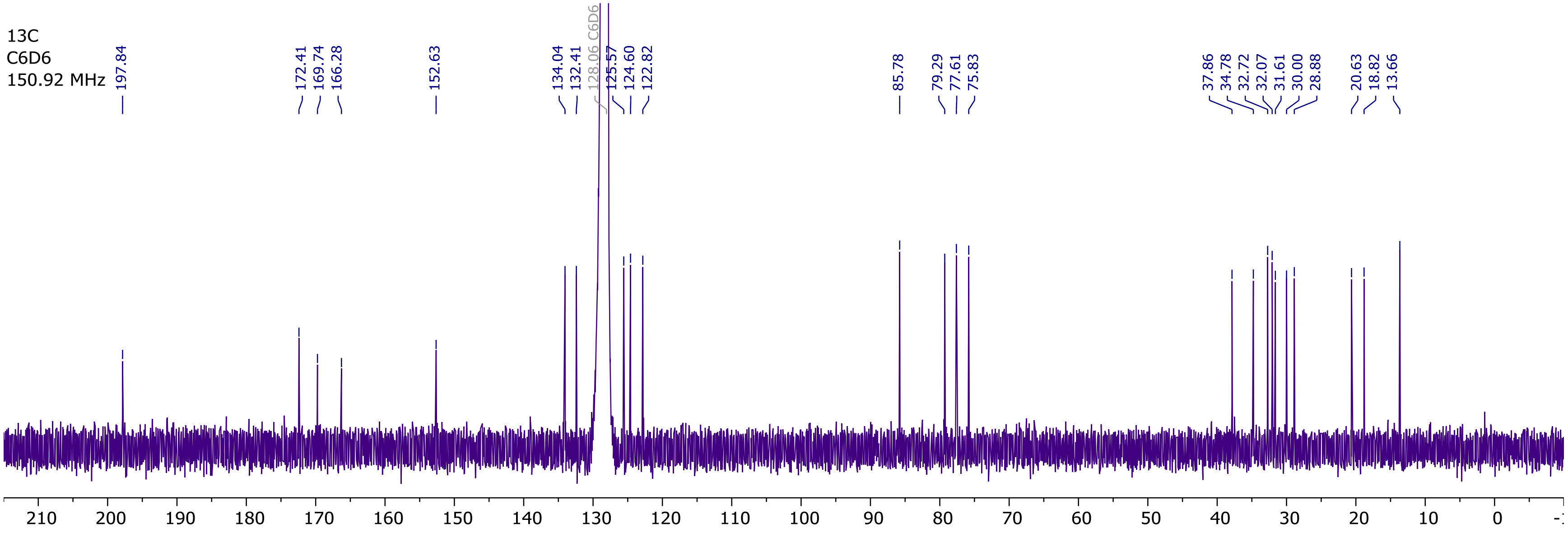
13C
C6D6
150.92 MHz
197.84
172.41
169.74
166.28
152.63
134.04
132.41
128.06 C6D6
125.57
124.60
122.82
85.78
79.29
77.61
75.83
37.86
34.78
32.72
32.07
31.61
30.00
28.88
20.63
18.82
13.66
210
200
190
180
170
160
150
140
130
120
110
100
90
80
70
60
50
40
30
20
10
0

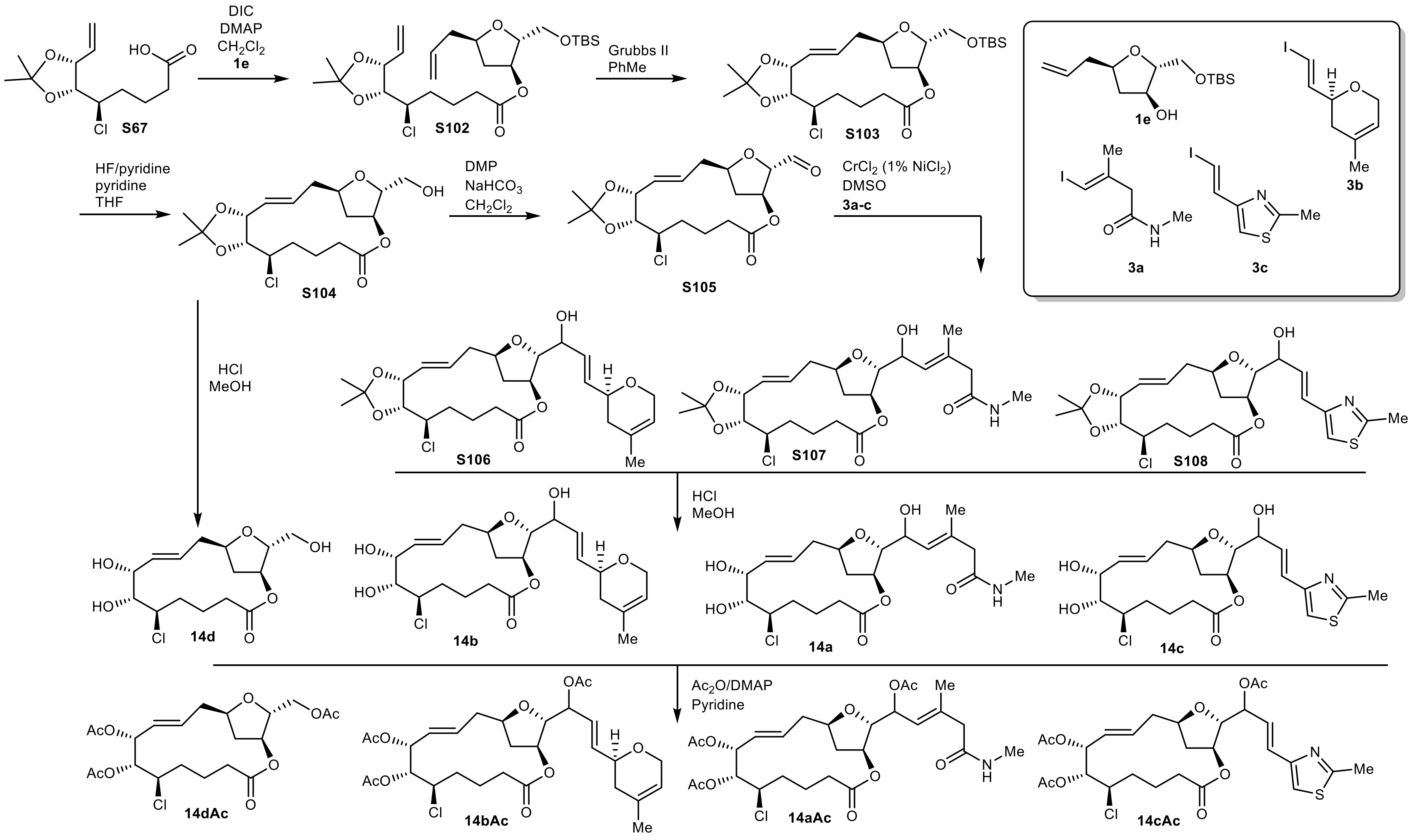


**Supplementary Fig. 44 | Synthesis of pMLs 14a-d and 14aAc-dAc.**

Abbreviations: DIC = N,N'-diisopropylcarbodiimide, TBS = tert-butyldimethylsilyl, Grubbs II = Dichloro[1,3-bis(2,4,6-trimethylphenyl)-2-imidazolidinylidene](benzylidene)(tricyclohexylphosphine)ruthenium(II), THF = tetrahydrofuran, DMP = Dess-Martin periodinane, DMSO = dimethylsulfoxide, DMAP = 4-dimethylaminopyridine.

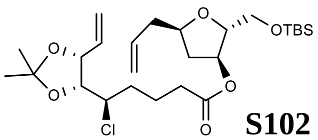


**S102**

Note: Reaction performed in capped vessel under ambient atmosphere.

To a rt stirred solution of acid **S67** (62 mg, 0.23 mmol, 1.0 eq.), alcohol **1e** (96 mg, 0.35 mmol, 1.5 eq.), and DMAP (17 mg, 0.14 mmol, 0.40 eq.) dissolved in anhydrous $CH_2Cl_2$ (2.3 mL), was added DIC (0.082 mL, 67 mg, 0.53 mmol, 2.3 eq.). The reaction mixture was stirred for 18 h at rt, after which time starting material was consumed as monitored by TLC analysis. The reaction mixture was quenched with saturated aqueous $NH_4Cl$ (10 mL) and $H_2O$ (10 mL) and the aqueous layer was extracted with $CH_2Cl_2$ (3x 25 mL). The combined organic layers were dried ($Na_2SO_4$), filtered, and solvent was removed in vacuo. The crude product was purified via flash column chromatography (7:1 hexanes/$Et_2O$). Appropriate fractions were pooled, and solvent was removed in vacuo to yield **S102** (101 mg, 83%) as a colorless oil.

**Analytical Data for S102:**

$R_f$ = 0.18 (7:1 hexanes/EtOAc)

$[\alpha]_D^{20}$ = +16 ° (c = 0.92, $CH_2Cl_2$)

$^1$H NMR (500 MHz, $CDCl_3$) δ 6.01 (ddd, *J* = 17.1, 10.2, 8.6 Hz, 1H), 5.80 (ddt, *J* = 17.2, 10.2, 6.9 Hz, 1H), 5.39 – 5.29 (m, 2H), 5.23 (ddd, *J* = 7.2, 3.6, 2.5 Hz, 1H), 5.13 – 5.05 (m, 2H), 4.57 (dd, *J* = 8.6, 6.4 Hz, 1H), 4.26 – 4.17 (m, 2H), 4.03 (q, *J* = 3.7 Hz, 1H), 3.80 (ddd, *J* = 10.0, 6.2, 3.8 Hz, 1H), 3.74 (dd, *J* = 10.8, 3.5 Hz, 1H), 3.66 (dd, *J* = 10.9, 4.1 Hz, 1H), 2.47 – 2.38 (m, 2H), 2.36 – 2.23 (m, 3H), 2.00 – 1.87 (m, 1H), 1.82 – 1.66 (m, 4H), 1.56 (s, 3H), 1.40 (s, 3H), 0.88 (s, 9H), 0.05 (s, 3H), 0.05 (s, 3H).

$^{13}$C NMR (126 MHz, $CDCl_3$) δ 172.91, 134.82, 133.32, 120.60, 117.24, 109.41, 84.33, 80.98, 79.67, 78.95, 76.84, 64.35, 60.36, 40.67, 37.58, 34.12, 33.64, 27.38, 26.00, 25.57, 21.66, 18.38, -5.29, -5.37.

HRMS (ESI): Anal. Calcd. for $C_{26}H_{49}ClNO_6Si^+$ $[M+NH_4]^+$ 534.3012, found 534.3008
IR (neat): $\nu_{max}$ $(cm^{-1})$ = 2929 (m, CH), 2856 (m, CH), 1737 (s, C=O)

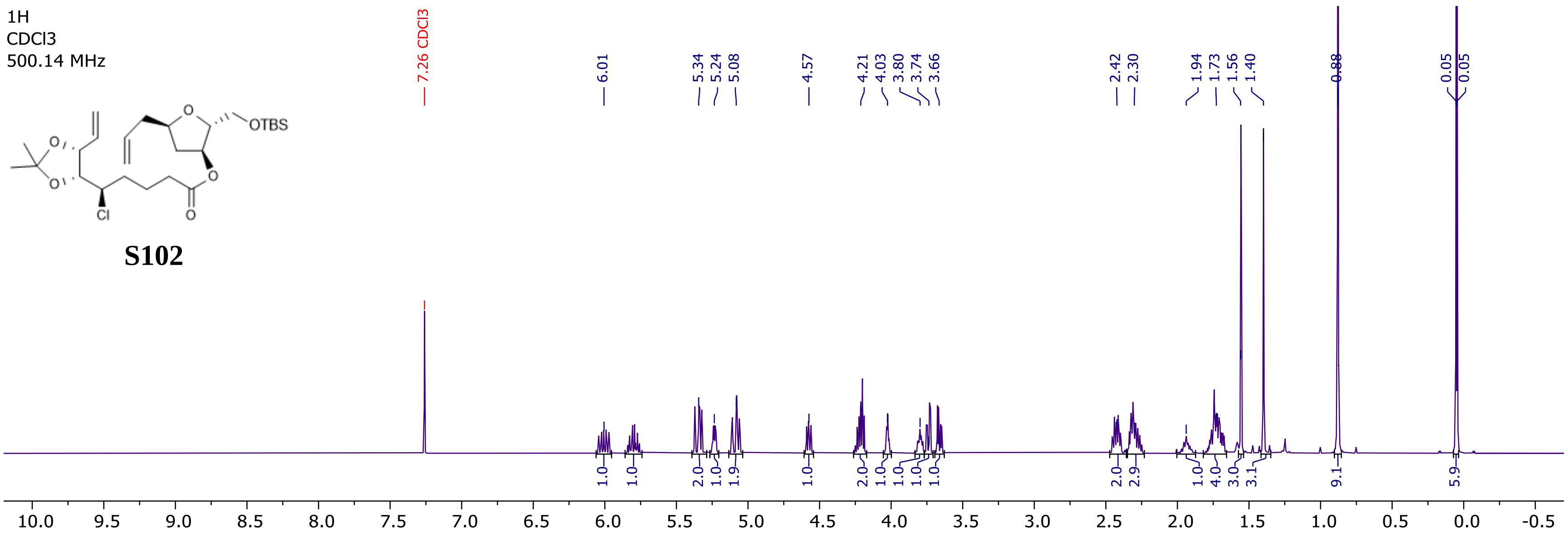
1H
CDCl3
500.14 MHz
7.26 CDCl3
6.01
5.34
5.24
5.08
4.57
4.21
4.03
3.80
3.74
3.66
2.42
2.30
1.94
1.73
1.56
1.40
0.88
0.05
0.05
1.0
1.0
2.0
1.0
1.9
1.0
2.0
1.0
1.0
1.0
1.0
2.0
2.9
1.0
4.0
3.0
3.1
9.1
5.9
10.0
9.5
9.0
8.5
8.0
7.5
7.0
6.5
6.0
5.5
5.0
4.5
4.0
3.5
3.0
2.5
2.0
1.5
1.0
0.5
0.0
-0.5
OTBS
O
O
O
O
O
Cl

**S102**

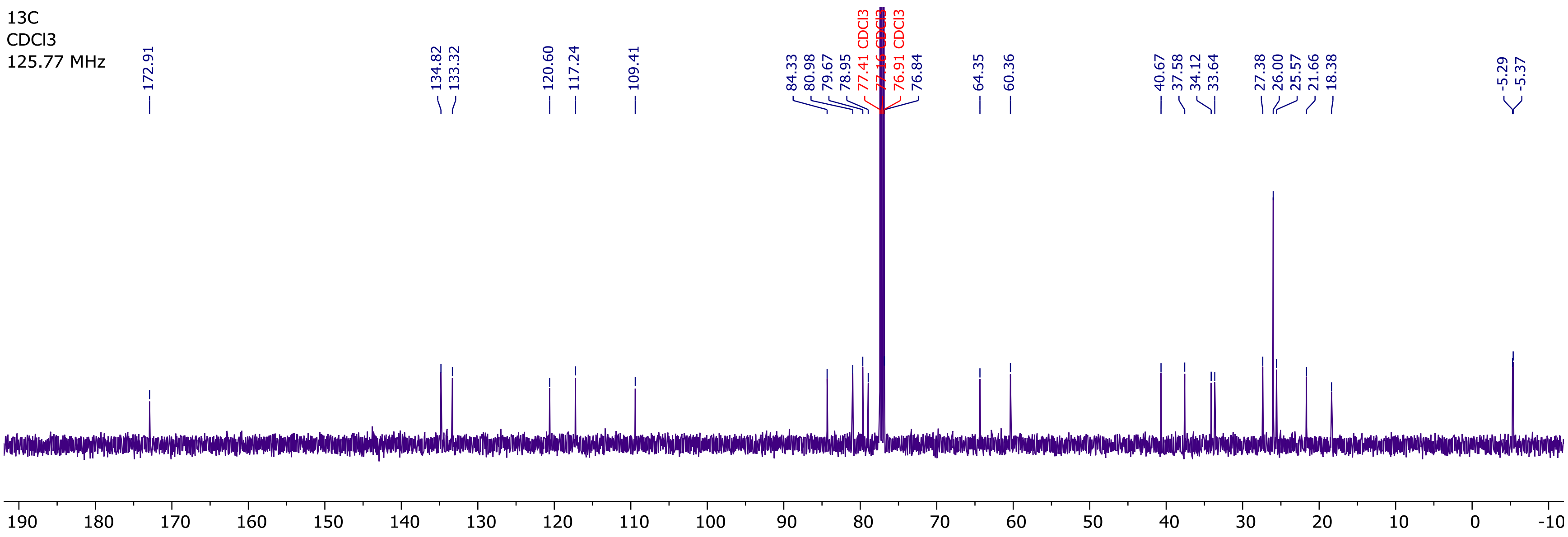
13C
CDCl3
125.77 MHz
172.91
134.82
133.32
120.60
117.24
109.41
84.33
80.98
79.67
78.95
77.41 CDCl3
77.16 CDCl3
76.91 CDCl3
76.84
64.35
60.36
40.67
37.58
34.12
33.64
27.38
26.00
25.57
21.66
18.38
-5.29
-5.37
190
180
170
160
150
140
130
120
110
100
90
80
70
60
50
40
30
20
10
0
-10

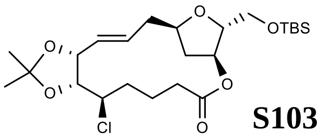

**S103**

To a stirred, heated (60 °C) solution of **S102** (101 mg, 0.20 mmol, 1.0 eq.) dissolved in anhydrous toluene (deoxygenated by sparging with nitrogen for 30 minutes) (76 mL) was added dropwise Grubbs 2nd generation catalyst (33 mg, 0.039 mmol, 0.2 eq.) in anhydrous, deoxygenated toluene (2.4 mL). Throughout the course of the reaction, the reaction mixture was sparged with a stream of $N_2$(g). After 15 min stirring at 60 °C, the reaction was cooled to 0 °C and quenched with potassium 2-isocyanoacetate (33 mg, 0.27 mmol, 1.4 eq) in MeOH (50 mL). The mixture was warmed to rt and stirred for 45 minutes and the solvent was removed in vacuo. The crude product was run through a plug of silica ($Et_2O$) and the solvent was removed in vacuo. The crude product was purified via flash column chromatography (17:3 hexanes/$Et_2O$). Appropriate fractions were pooled, and solvent was removed in vacuo to yield (*E*)-isomer **S103** (64 mg, 68%) as a white solid (d.r. = 3:1).

**Analytical Data for S103:**

$R_f$ = 0.30 (7:3 Petroleum ether (bp: 36-60)/$Et_2O$)

$[\alpha]_D^{20}$ = -36 ° (c = 0.23, $CH_2Cl_2$)

$^1$H NMR (500 MHz, $CDCl_3$) δ 5.88 (ddd, *J* = 15.2, 10.7, 4.3 Hz, 1H), 5.59 (ddd, *J* = 15.6, 9.4, 1.8 Hz, 1H), 5.17 (dt, *J* = 8.1, 1.7 Hz, 1H), 4.54 – 4.46 (m, 2H), 4.17 (dd, *J* = 8.7, 5.7 Hz, 1H), 4.13 (t, *J* = 3.6 Hz, 1H), 3.92 (ddd, *J* = 11.4, 8.6, 2.9 Hz, 1H), 3.74 (dd, *J* = 10.8, 3.4 Hz, 1H), 3.68 (dd, *J* = 10.8, 3.8 Hz, 1H), 2.79 (dddd, *J* = 14.4, 5.9, 4.3, 2.0 Hz, 1H), 2.59 – 2.47 (m, 2H), 2.20 (ddd, *J* = 15.6, 10.5, 1.8 Hz, 1H), 2.13 – 1.94 (m, 3H), 1.71 – 1.55 (m, 3H), 1.53 (s, 3H), 1.40 (s, 3H), 0.88 (s, 9H), 0.06 (s, 3H), 0.05 (s, 3H).

$^{13}$C NMR (151 MHz, $CDCl_3$) δ 173.47, 131.87, 128.91, 108.82, 85.36, 82.34, 79.94, 78.56, 78.28, 65.01, 60.92, 37.39, 37.24, 33.54, 32.76, 29.85, 28.36, 25.99, 25.82, 22.46, 18.31, -5.39.

HRMS (ESI): Anal. Calcd. for $C_{24}H_{45}ClNO_6Si^+$ $[M+NH_4]^+$ 506.2699, found 506.2678
IR (neat): $\nu_{max}$ $(cm^{-1})$ = 2954 (m, CH), 2920 (s, CH), 2852 (s, 2852), 1729 (C=O), 1379 (m), 1253 (s), 1221 (s)

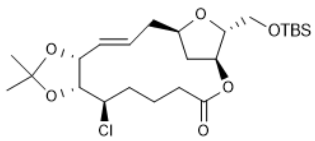
OTBS
O
O
O
O
O
Cl

**S103**

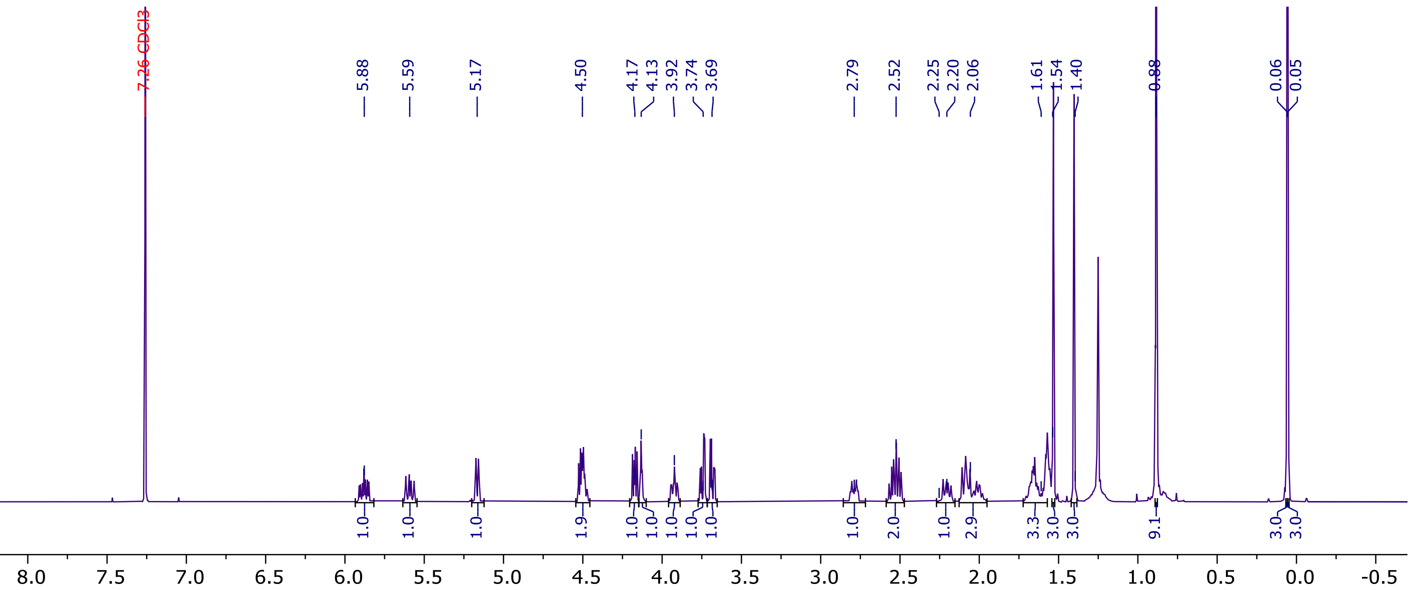
1H
CDCl3
500.14 MHz
7.26 CDCl3
5.88
5.59
5.17
4.50
4.17
4.13
3.92
3.74
3.69
2.79
2.52
2.25
2.20
2.06
1.61
1.54
1.40
0.88
0.06
0.05
1.0
1.0
1.0
1.9
1.0
1.0
1.0
1.0
1.0
1.0
2.0
1.0
2.9
3.3
3.0
3.0
9.1
3.0
3.0
10.0 9.5 9.0 8.5 8.0 7.5 7.0 6.5 6.0 5.5 5.0 4.5 4.0 3.5 3.0 2.5 2.0 1.5 1.0 0.5 0.0 -0.5

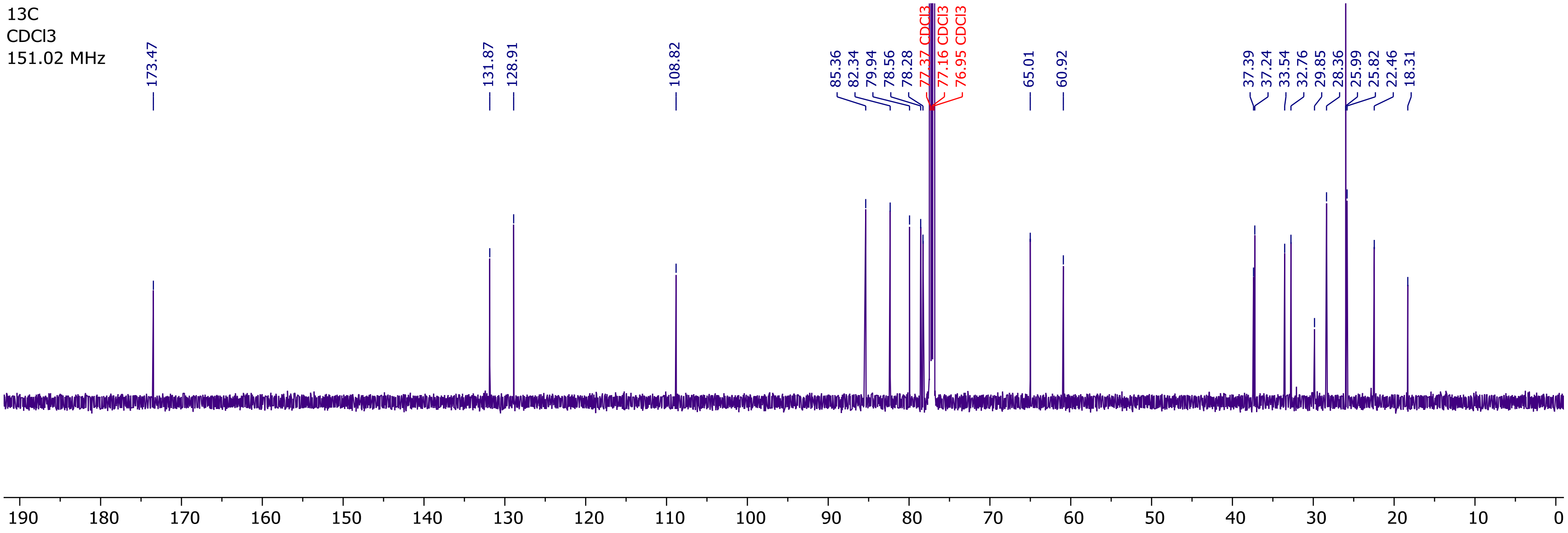
13C
CDCl3
151.02 MHz
173.47
131.87
128.91
108.82
85.36
82.34
79.94
78.56
78.28
77.37 CDCl3
77.16 CDCl3
76.95 CDCl3
65.01
60.92
37.39
37.24
33.54
32.76
29.85
28.36
25.99
25.82
22.46
18.31
190 180 170 160 150 140 130 120 110 100 90 80 70 60 50 40 30 20 10 0

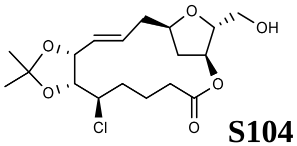

**S104**

Note: Reaction performed in capped vessel under ambient atmosphere.
To a plastic vessel containing **S103** (64 mg, 0.13 mmol, 1.0 eq.) and pyridine (0.51 mL) dissolved in anhydrous was added 70% HF/pyridine (0.51 mL, 20 mmol HF, 150 eq.). After 20 minutes stirring at rt, the reaction was quenched with saturated $NaHCO_3$ (25 mL) and after effervescence had subsided, the aqueous layer extracted with $CH_2Cl_2$ (3 x 40 mL). The combined organic layers were dried ($Na_2SO_4$), filtered, and the solvent was removed in vacuo. The crude product was purified via flash column chromatography (33:7 hexanes/acetone). Appropriate fractions were pooled to yield **S104** (48 mg, 97%) as a colorless oil.

**Analytical Data for S104:**

$R_f$ = 0.09 (4:1 hexanes/acetone)

$[\alpha]_D^{20}$ = -47.8 ° (c = 1.90, $CH_2Cl_2$)

$^1$H NMR (601 MHz, $CDCl_3$) δ 5.85 (ddd, *J* = 15.2, 10.6, 4.3 Hz, 1H), 5.62 (ddd, *J* = 15.6, 9.3, 1.8 Hz, 1H), 5.01 (dt, *J* = 8.5, 2.4 Hz, 1H), 4.52 (dd, *J* = 9.2, 5.8 Hz, 1H), 4.43 (dtd, *J* = 8.2, 6.4, 1.6 Hz, 1H), 4.22 – 4.14 (m, 2H), 3.91 (ddd, *J* = 11.2, 8.5, 2.7 Hz, 1H), 3.70 – 3.58 (m, 2H), 2.79 (dddd, *J* = 14.4, 6.0, 4.3, 1.9 Hz, 1H), 2.64 – 2.47 (m, 2H), 2.26 – 2.15 (m, 1H), 2.11 (ddd, *J* = 14.5, 10.6, 1.6 Hz, 1H), 2.08 – 1.95 (m, 3H), 1.75 – 1.60 (m, 3H), 1.52 (s, 3H), 1.39 (s, 3H).

$^{13}$C NMR (151 MHz, $CDCl_3$) δ 173.44, 131.33, 129.44, 108.85, 85.06, 82.21, 79.73, 77.29, 76.92, 62.28, 60.75, 37.36, 36.72, 33.47, 32.89, 28.27, 25.74, 22.38.

HRMS (ESI): Anal. Calcd. for $C_{18}H_{31}ClNO_6^+$ $[M+NH_4]^+$ 392.1834, found 392.1813
IR (neat): $\nu_{max}$ $(cm^{-1})$ = 3470 (br, OH), 2985 (m, CH), 2934 (m, CH), 1726 (s, C=O), 1437 (m), 1381 (s), 1341 (m), 1250 (s), 1220 (s)

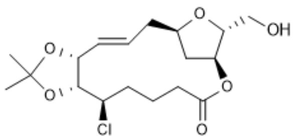
O
OH
O
O
O
Cl
O

**S104**

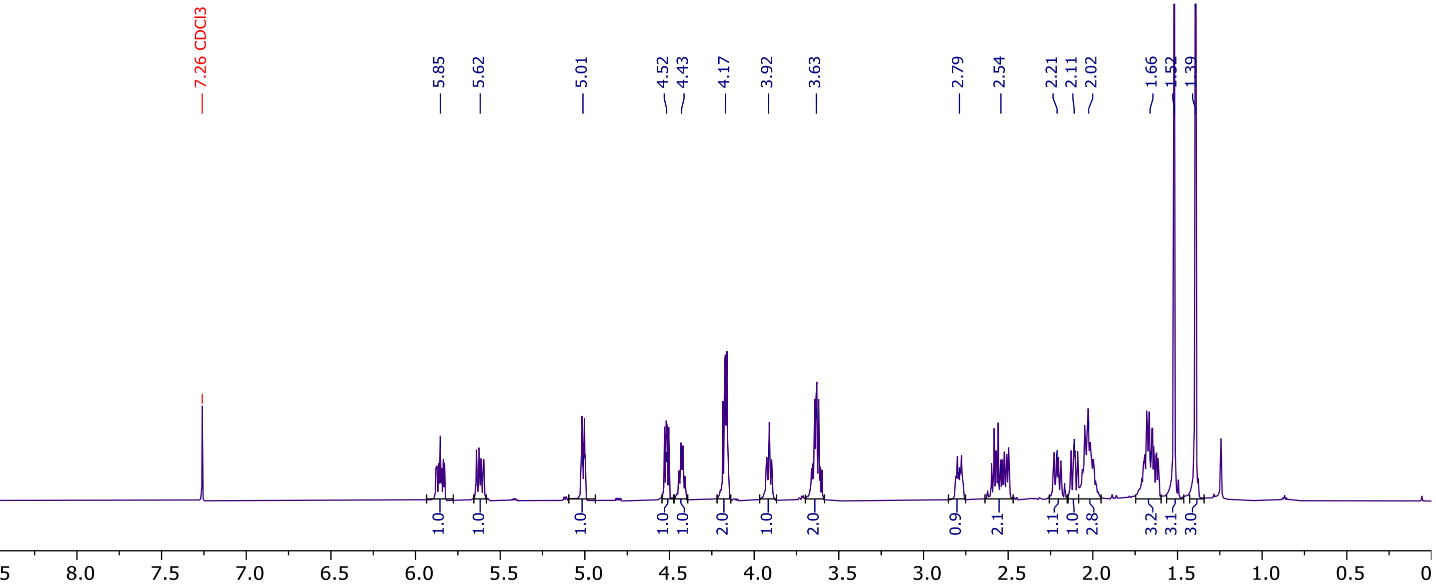
1H
CDCl3
600.51 MHz
7.26 CDCl3
5.85
5.62
5.01
4.52
4.43
4.17
3.92
3.63
2.79
2.54
2.21
2.11
2.02
1.66
1.52
1.39
1.0
1.0
1.0
1.0
1.0
2.0
1.0
2.0
0.9
2.1
1.1
1.0
2.8
3.2
3.1
3.0
10.0 9.5 9.0 8.5 8.0 7.5 7.0 6.5 6.0 5.5 5.0 4.5 4.0 3.5 3.0 2.5 2.0 1.5 1.0 0.5 0

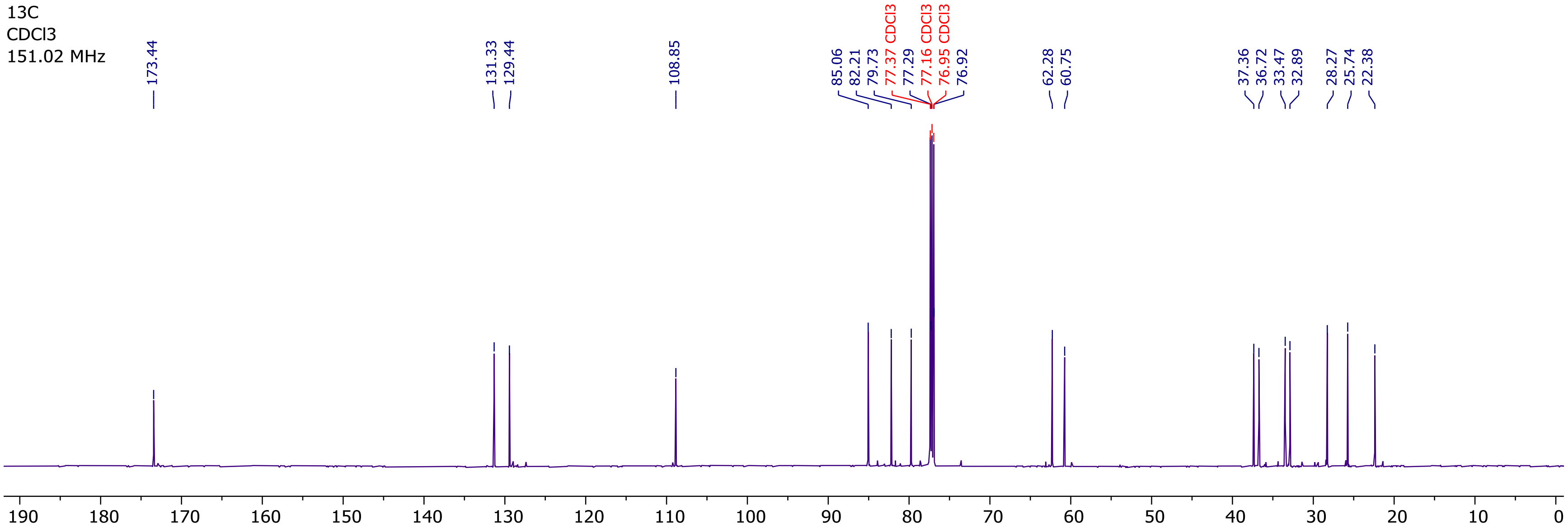
13C
CDCl3
151.02 MHz
173.44
131.33
129.44
108.85
85.06
82.21
79.73
77.37 CDCl3
77.29
77.16 CDCl3
76.95 CDCl3
76.92
62.28
60.75
37.36
36.72
33.47
32.89
28.27
25.74
22.38
190 180 170 160 150 140 130 120 110 100 90 80 70 60 50 40 30 20 10 0

Note: Reaction performed in capped vessel under ambient atmosphere.
To a stirred solution of **S104** (41.5 mg, 0.111 mmol, 1.0 eq.) in anhydrous $CH_2Cl_2$ (2.2 mL) was added $NaHCO_3$ (27.9 mg, 0.332 mmol, 3 eq.) followed by Dess-Martin Periodinane (61.1 mg, 0.144 mmol, 1.3 eq.). After stirring for 1 h at rt the mixture was quenched with a solution of 1:1:1 $H_2O$-saturated $NaHCO_3$-saturated $Na_2S_2O_3$ (2.2 mL) and the biphasic mixture was stirred vigorously for 1 h. $H_2O$ added (10 mL) and the aqueous layer was extracted with $CH_2Cl_2$ (3x 30 mL) and the combined organic layers were dried ($Na_2SO_4$), filtered, and the solvent was removed in vacuo. The crude product was run through a plug of C2-modified silica gel ($Et_2O$) and solvent was removed in vacuo to yield the crude aldehyde **S105** (36.9 mg, 89%) as a white solid which was divided into 3 equal portions for the following steps.

To a solution of **S105** (10.3 mg, 0.0276 mmol, 1.0 eq.) in anhydrous DMSO (deoxygenated via 5x freeze-pump-thaw cycles) (0.5 mL) was added $CrCl_2$ with doped with 1 % $NiCl_2$ (w/w) (34.0 mg, 0.276 mmol, 10 eq.). **3b** (38 mg, 0.152 mmol, 5.5 eq.) was added in DMSO (0.75 mL) and the mixture was stirred for 42 h. The reaction was quenched with 1:1 $H_2O$/brine (15 mL). The aqueous layer was extracted with EtOAc (8x 15 mL) and the combined organic layers were dried ($Na_2SO_4$), filtered, and the solvent was removed in vacuo. The crude product was purified via flash column chromatography (17:3 to 4:1 hexanes/acetone). Appropriate fractions were pooled, and solvent was removed in vacuo to yield **S106** (6.4 mg, 42% over two steps) as a colorless oil and a mixture of diastereomers (8:3).

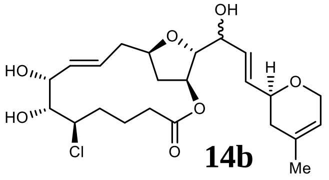


**14b**

Note: Reaction performed in capped vessel under ambient atmosphere.
To a stirred vial containing **S106** (6.4 mg, 0.013 mmol, 1.0 eq.) dissolved in 1:1 $CH_2Cl_2$/MeOH (1.2 mL) was added 1.2M HCl in MeOH (54 µL, 0.064 mmol, 5.0 eq.). After 1 day stirring at rt, the reaction was quenched with $NaHCO_{3\,(s)}$ (15 mg) and after effervescence had subsided, the solvent was removed in vacuo. The crude product was purified via flash column chromatography (97:3 to 24:1 $CH_2Cl_2$/EtOH). Appropriate fractions were pooled to yield **14b** (5.0 mg, 85%) as a colorless oil and a mixture of diastereomers (8:3).

**Analytical Data for 14b:**

$R_f$ = 0.14 (24:1 $CH_2Cl_2$/EtOH)

For the following reported NMR peaks: Several resonances in the $^1H$ NMR spectrum integrated for 0.8 and 0.3 protons and are unique to both the diastereomers. In these cases an integral value of "0.8 H" and "0.3 H" is assigned.

$^{1}$H NMR (601 MHz, $CDCl_3$) δ 5.96 – 5.80 (m, 3H), 5.74 (dtd, *J* = 15.7, 8.9, 8.2, 1.9 Hz, 1H), 5.44 – 5.39 (m, 1H), 5.19 (dt, *J* = 8.6, 1.9 Hz, 0.8H), 5.08 (dt, *J* = 8.4, 1.7 Hz, 0.3H), 4.51 – 4.44 (m, 1H), 4.24 (q, *J* = 5.3 Hz, 1H), 4.19 – 4.14 (m, 3H), 4.10 – 4.02 (m, 2H), 3.91 – 3.78 (m, 2H), 2.87 (d, *J* = 2.4 Hz, 1H), 2.75 (dddd, *J* = 14.8, 5.7, 3.9, 2.0 Hz, 1H), 2.55 – 2.47 (m, 2H), 2.44 (d, *J* = 7.5 Hz, 1H), 2.27 (dddd, *J* = 15.7, 9.6, 3.7, 2.2 Hz, 1H), 2.18 (d, *J* = 4.9 Hz, 1H), 2.16 – 2.10 (m, 1H), 2.09 – 2.00 (m, 2H), 1.96 – 1.88 (m, 2H), 1.70 (s, 3H), 1.69 – 1.64 (m, 2H).

<u>Major diastereomer</u>
$^{13}$C NMR (151 MHz, $CDCl_3$) δ 173.53, 133.34, 131.54, 131.49, 129.31, 129.13, 119.82, 87.90, 78.29, 78.19, 77.42, 73.75, 73.22, 72.87, 66.82, 65.83, 37.46, 36.07, 35.72, 32.96, 30.60, 23.08, 22.42.

<u>Minor diastereomer</u>
$^{13}$C NMR (151 MHz, $CDCl_3$) δ 173.57, 134.52, 131.50, 129.37, 128.90, 87.91, 78.33, 77.93, 77.53, 73.79, 73.21, 71.90, 37.46, 37.28, 36.02, 35.69, 32.94, 30.56.

HRMS (ESI): Anal. Calcd. for $C_{23}H_{33}ClNaO_7^+$ $[M+Na]^+$ 479.1807, found 479.1800
IR (neat): $\nu_{max}\ (cm^{-1})$ = 3412 (br, OH), 2927 (s, CH), 2909 (s, CH), 1725 (s, C=O), 1711 (s, C=O), 1440 (m), 1381 (s), 1337 (m), 1257 (s)

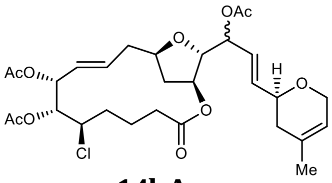


**14bAc**

**14bAc** was prepared according to general procedure A (1.46 mg, 83%).

**Analytical Data for 14bAc:**

$R_f$ = 0.63 (1:1 hexanes/EtOAc)

<u>Major diastereomer</u>

$^{1}$H NMR (600 MHz, $C_6D_6$) δ 6.12 – 5.97 (m, 3H), 5.69 (dq, *J* = 9.3, 2.0 Hz, 1H), 5.67 – 5.61 (m, 1H), 5.60 (s, 1H), 5.22 – 5.16 (m, 1H), 5.15 – 5.08 (m, 1H), 4.24 (d, *J* = 6.0 Hz, 1H), 4.14 – 4.07 (m, 1H), 4.06 – 4.01 (m, 1H), 4.00 – 3.95 (m, 1H), 3.94 – 3.88 (m, 1H), 3.78 – 3.69 (m, 1H), 2.52 (dq, *J* = 14.9, 3.1 Hz, 1H), 2.16 – 2.05 (m, 2H), 2.04 – 1.92 (m, 1H), 1.86 (d, *J* = 1.6 Hz, 3H), 1.75 (m, 7H), 1.69 (ddd, *J* = 17.9, 9.1, 1.8 Hz, 1H), 1.65 – 1.59 (m, 2H), 1.47 (d, *J* = 7.9 Hz, 4H), 1.36 – 1.21 (m, 3H).

Major diastereomer

$^{13}C$ NMR (151 MHz, $C_6D_6$) δ 172.46, 169.55, 169.52, 169.36, 135.67, 135.34, 131.15, 125.27, 124.86, 120.40, 86.67, 77.53, 77.35, 76.52, 74.02, 73.80, 72.98, 65.81, 62.85, 37.14, 35.90, 35.87, 32.67, 30.82, 22.92, 22.56, 20.80, 20.66, 20.47.

IR (neat): $\nu_{max}$ $(cm^{-1})$ = 2931 (s, CH), 1732 (s, C=O), 1437 (w), 1371 (m), 1234 (s)

HRMS (ESI): Anal. Calcd. for $C_{29}H_{43}ClNO_{10}^{+}$ $[M+NH_4]^+$ 600.2570, found 600.2587

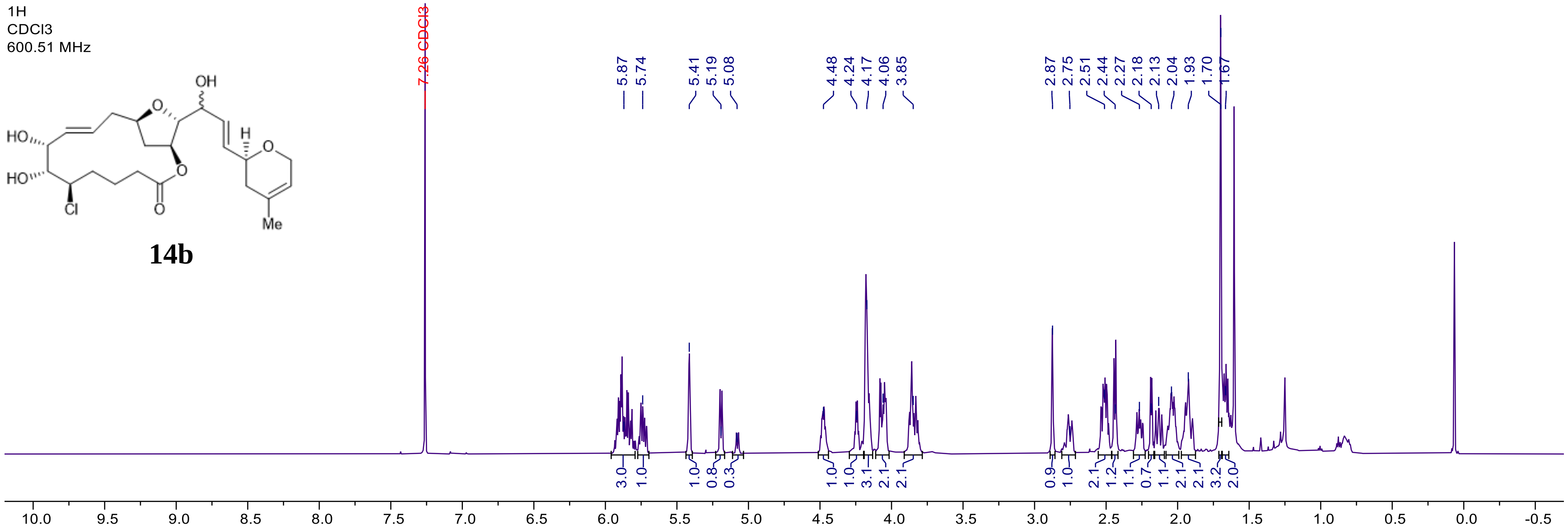
1H
CDCl3
600.51 MHz
7.26 CDCl3
5.87
5.74
5.41
5.19
5.08
4.48
4.24
4.17
4.06
3.85
2.87
2.75
2.51
2.44
2.27
2.18
2.13
2.04
1.93
1.70
1.67
3.0
1.0
1.0
0.8
0.3
1.0
1.0
3.1
2.1
2.1
0.9
1.0
2.1
1.2
1.1
0.7
1.1
1.1
2.1
2.1
3.2
2.0
10.0
9.5
9.0
8.5
8.0
7.5
7.0
6.5
6.0
5.5
5.0
4.5
4.0
3.5
3.0
2.5
2.0
1.5
1.0
0.5
0.0
-0.5
OH
HO
HO
O
O
H
O
Cl
O
Me

**14b**

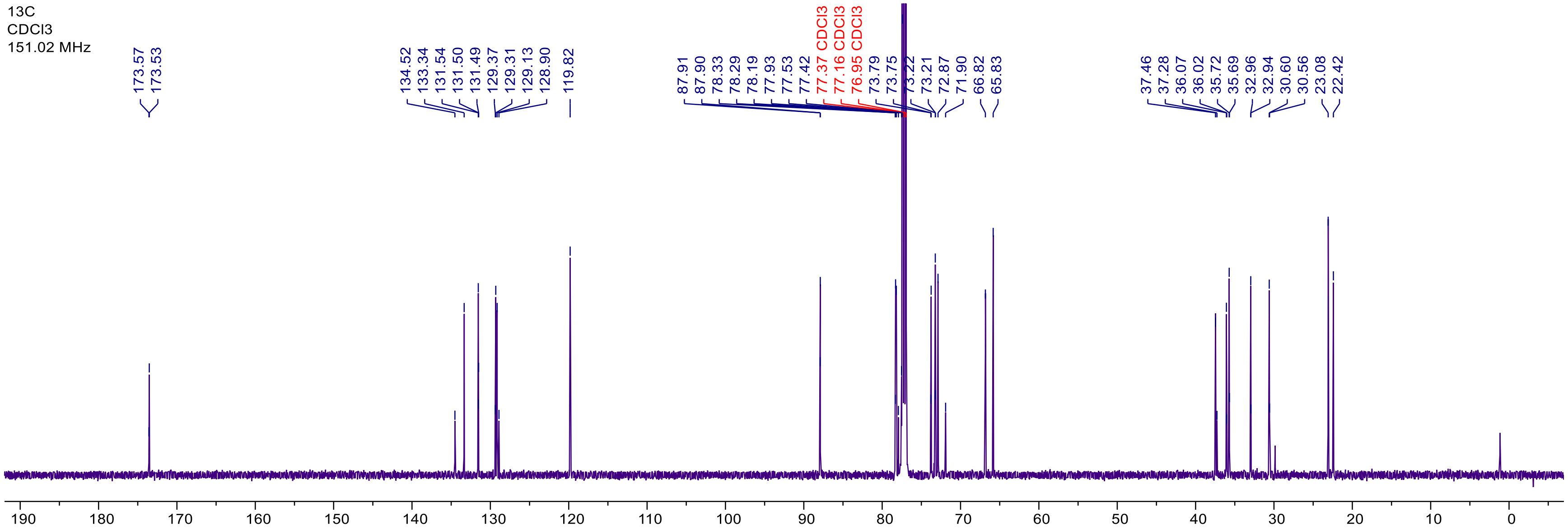
13C
CDCl3
151.02 MHz
173.57
173.53
134.52
133.34
131.54
131.50
131.49
129.37
129.31
129.13
128.90
119.82
87.91
87.90
78.33
78.29
78.19
77.93
77.53
77.42
77.37 CDCl3
77.16 CDCl3
76.95 CDCl3
73.79
73.75
73.22
73.21
72.87
71.90
66.82
65.83
37.46
37.28
36.07
36.02
35.72
35.69
32.96
32.94
30.60
30.56
23.08
22.42
190
180
170
160
150
140
130
120
110
100
90
80
70
60
50
40
30
20
10
0

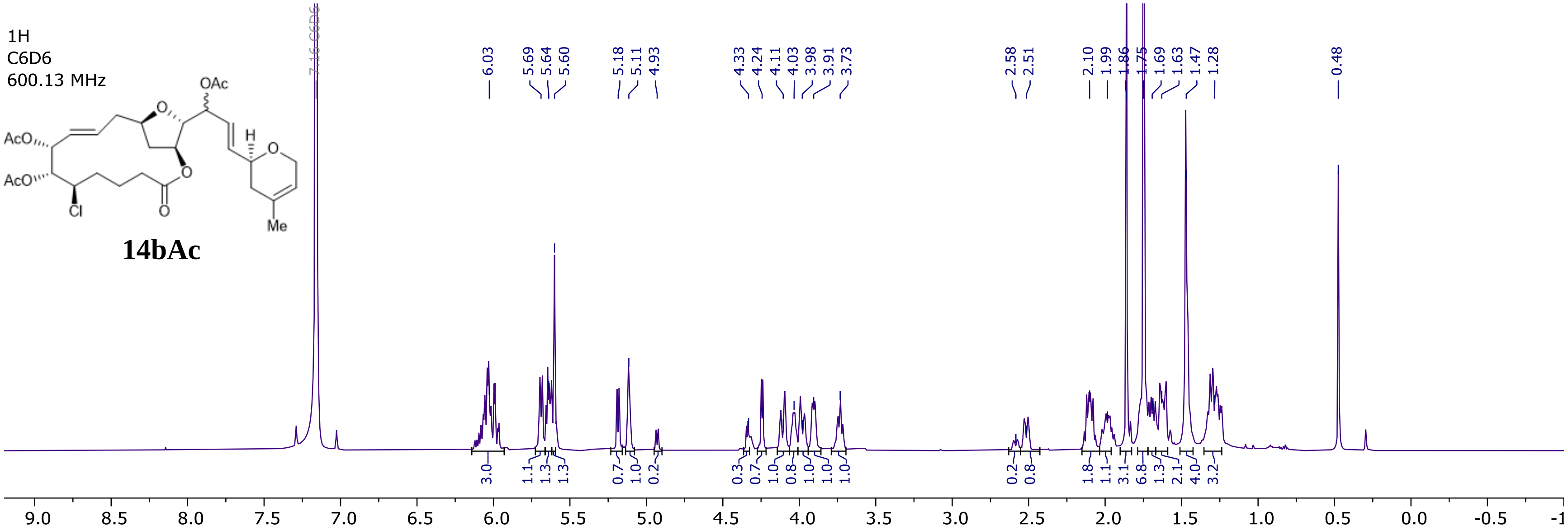
1H
C6D6
600.13 MHz
OAc
AcO
AcO
O
O
O
H
O
Cl
O
Me
14bAc
7.16 C6D6
6.03
5.69
5.64
5.60
5.18
5.11
4.93
4.33
4.24
4.11
4.03
3.98
3.91
3.73
2.58
2.51
2.10
1.99
1.86
1.75
1.69
1.63
1.47
1.28
0.48
3.0
1.1
1.3
1.3
0.7
1.0
0.2
0.3
0.7
1.0
0.8
1.0
1.0
1.0
0.2
0.8
1.8
1.1
3.1
6.8
1.3
2.1
4.0
3.2
9.0 8.5 8.0 7.5 7.0 6.5 6.0 5.5 5.0 4.5 4.0 3.5 3.0 2.5 2.0 1.5 1.0 0.5 0.0 -0.5

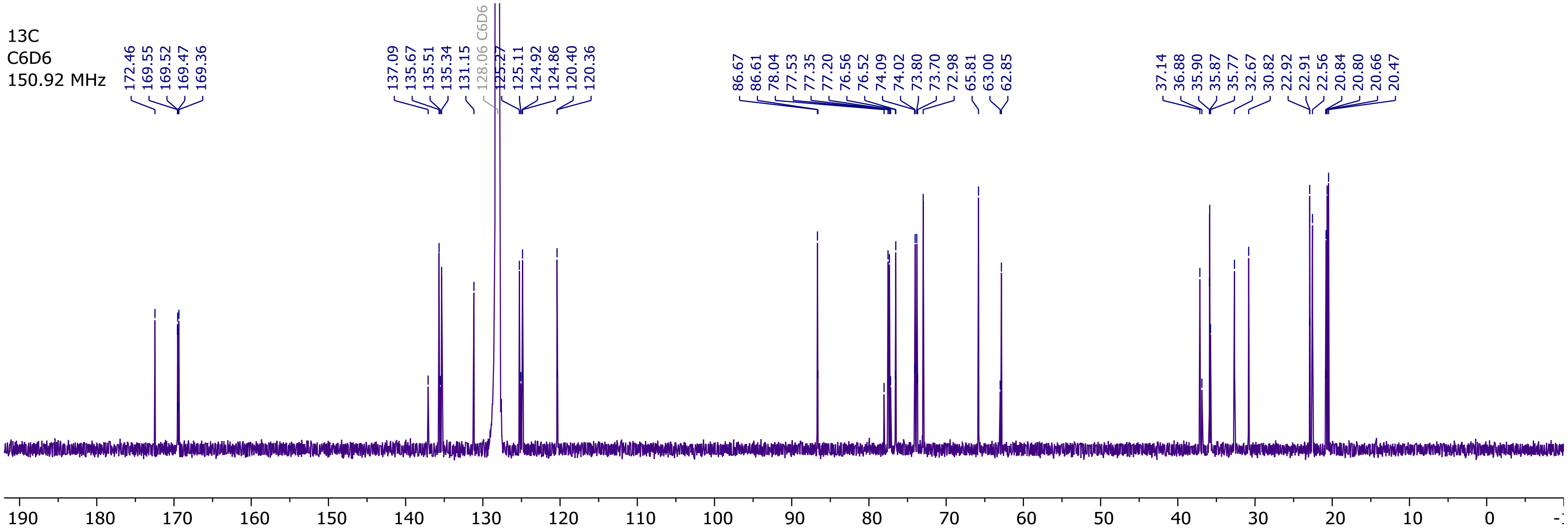
13C
C6D6
150.92 MHz
172.46
169.55
169.52
169.47
169.36
137.09
135.67
135.51
135.34
131.15
128.06 C6D6
125.27
125.11
124.92
124.86
120.40
120.36
86.67
86.61
78.04
77.53
77.35
77.20
76.56
76.52
74.09
74.02
73.80
73.70
72.98
65.81
63.00
62.85
37.14
36.88
35.90
35.87
35.77
32.67
30.82
22.92
22.91
22.56
20.84
20.80
20.66
20.47
190 180 170 160 150 140 130 120 110 100 90 80 70 60 50 40 30 20 10 0

To a solution of **S105** (10.3 mg, 0.0276 mmol, 1.0 eq.) in anhydrous DMSO (deoxygenated via 5x freeze-pump-thaw cycles) (0.5 mL) was added $CrCl_2$ with doped with 1 % $NiCl_2$ (w/w) (34.0 mg, 0.276 mmol, 10 eq.). **3a** (19.8 mg, 0.0828 mmol, 3.0 eq.) was added in deoxygenated DMSO (0.75 mL) and the mixture was stirred for 42 h. The reaction was quenched with 1:1 $H_2O$-brine (15 mL). The aqueous layer was extracted with EtOAc (8x 15 mL) and the combined organic layers were dried ($Na_2SO_4$), filtered, and the solvent was removed in vacuo. The crude product was purified via flash column chromatography (13:7 to 11:9 hexanes/acetone). Appropriate fractions were pooled, and solvent was removed in vacuo to yield **S107** (6.8 mg, 45% over two steps) as a colorless oil and a mixture of diastereomers (7:4).

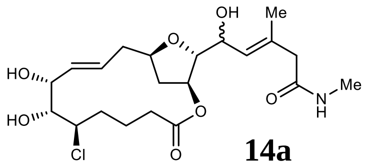


**14a**

Note: Reaction performed in capped vessel under ambient atmosphere.
To a stirred vial containing **S107** (6.8 mg, 0.014 mmol, 1.0 eq.) dissolved in MeOH (1.4 mL) was added 1.2M HCl in MeOH (58 µL, 0.070 mmol, 5.0 eq.). After 1 day stirring at rt, the reaction was quenched with $NaHCO_{3\ (s)}$ (15 mg) and after effervescence had subsided, the solvent was removed in vacuo. The crude product was purified via flash column chromatography (19:1 to 23:2 $CH_2Cl_2$:MeOH). Appropriate fractions were pooled to yield **14a** (3.7 mg, 59%) as a colorless oil and a mixture of diastereomers (7:4).

**Analytical Data for 14a:**

$R_f$ = 0.28 (23:2 $CH_2Cl_2$:MeOH)

For the following reported NMR peaks: Several resonances in the $^1$H NMR spectrum integrated for 0.6 and 0.3 protons and are unique to both the diastereomers. In these cases an integral value of "0.6 H" and "0.3 H" is assigned.

$^1$H NMR (601 MHz, $CDCl_3$) δ 6.35 – 6.24 (m, 1H), 5.96 – 5.83 (m, 1H), 5.81 – 5.72 (m, 1H), 5.44 – 5.36 (m, 1H), 5.20 (d, *J* = 8.4 Hz, 0.6H), 4.91 (d, *J* = 8.0 Hz, 0.3H), 4.56 – 4.40 (m, 2H), 4.26 (t, *J* = 8.7 Hz, 0.3H), 4.16 (d, *J* = 7.8 Hz, 1H), 4.04 (d, *J* = 5.1 Hz, 0.6H), 3.96 (d, *J* = 8.4 Hz, 0.3H), 3.86 (dtt, 2H), 3.04 (dd, *J* = 22.4, 15.0 Hz, 1H), 2.97 – 2.88 (m, 2H), 2.82 (dddd, *J* = 15.2, 5.9, 3.9, 2.2 Hz, 0.6H), 2.78 – 2.74 (m, 3H), 2.73 (ddd, *J* = 5.6, 3.7, 2.0 Hz, 0.3H), 2.63 – 2.46 (m, 3H), 2.40 – 2.36 (m, 1H), 2.33 – 2.24 (m, 1H), 2.16 (dtd, *J* = 14.9, 10.8, 1.8 Hz, 1H), 2.12 – 2.01 (m, 1H), 1.98 – 1.90 (m, 1H), 1.76 (d, *J* = 1.4 Hz, 1.0H), 1.74 (d, *J* = 1.4 Hz, 2.0H).

Major diastereomer

$^{13}$C NMR (151 MHz, $CDCl_3$) δ 173.91, 171.13, 135.03, 131.20, 129.39, 129.36, 88.41, 78.44, 78.35, 78.09, 73.82, 70.34, 67.03, 47.97, 37.44, 35.92, 33.04, 30.68, 26.60, 22.50, 17.23.

Minor diastereomer
$^{13}$C NMR (151 MHz, $CDCl_3$) δ 173.75, 170.99, 136.34, 131.17, 129.46, 128.37, 78.41, 76.51, 73.89, 67.22, 66.50, 48.37, 36.56, 35.16, 32.97, 30.66, 26.64, 22.63, 17.11.

HRMS (ESI): Anal. Calcd. for $C_{21}H_{33}ClNO_7^+$ $[M+H]^+$ 446.1940, found 446.1920
IR (neat): $\nu_{max}$ ($cm^{-1}$) = 3412 (br, OH), 2927 (s, CH), 2909 (s, CH), 1725 (s, C=O), 1711 (s, C=O), 1440 (m), 1381 (m), 1337 (m), 1257 (s)

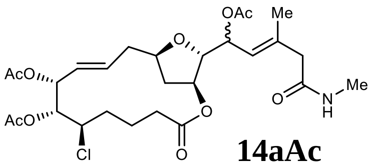


**14aAc**

**14aAc** was prepared according to general procedure A (1.93 mg, 93%).

**Analytical Data for 14aAc:**

$R_f$ = 0.32 (EtOAc)

HRMS (ESI): Anal. Calcd. for $C_{27}H_{42}ClN_2O_{10}^+$ $[M+NH_4]^+$ 589.2522, found 589.2499

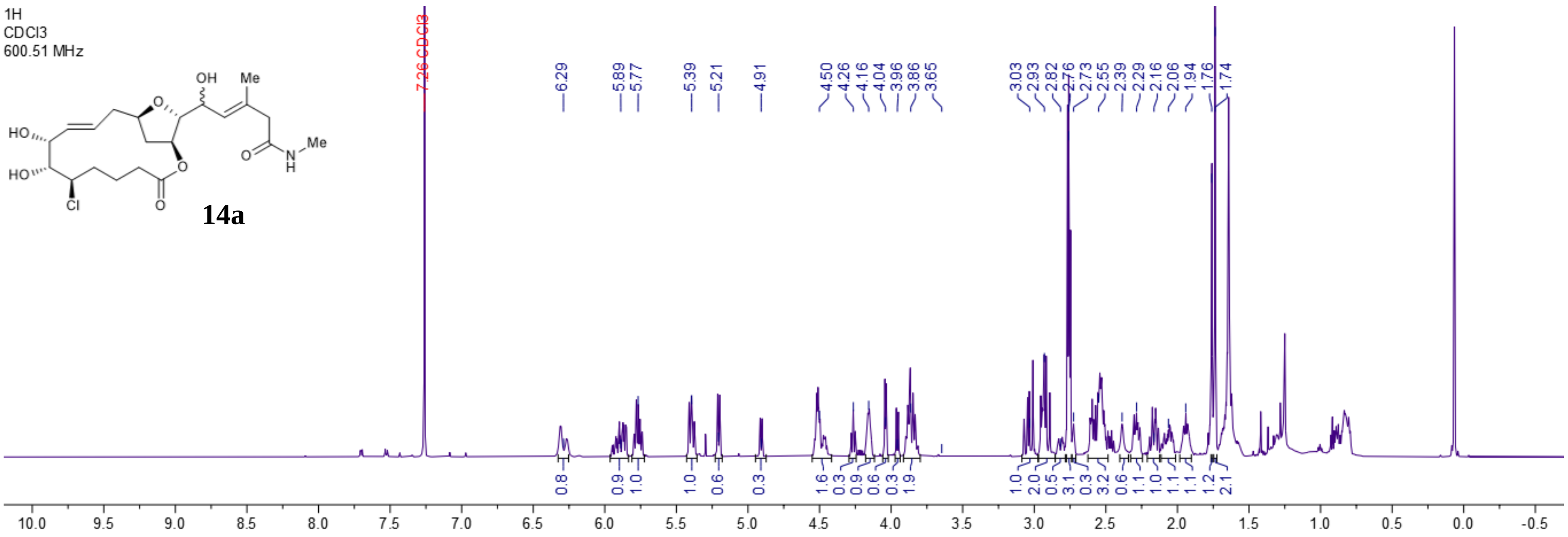
1H
CDCl3
600.51 MHz
14a
7.26 CDCl3
6.29
5.89
5.77
5.39
5.21
4.91
4.50
4.26
4.16
4.04
3.96
3.86
3.65
3.03
2.93
2.82
2.76
2.73
2.55
2.39
2.29
2.16
2.06
1.94
1.76
1.74
0.8
0.9
1.0
1.0
0.6
0.3
1.6
0.3
0.9
0.6
0.3
1.9
1.0
2.0
0.5
3.1
0.3
3.2
0.6
1.1
1.0
1.1
1.1
1.2
2.1
10.0
9.5
9.0
8.5
8.0
7.5
7.0
6.5
6.0
5.5
5.0
4.5
4.0
3.5
3.0
2.5
2.0
1.5
1.0
0.5
0.0
-0.5

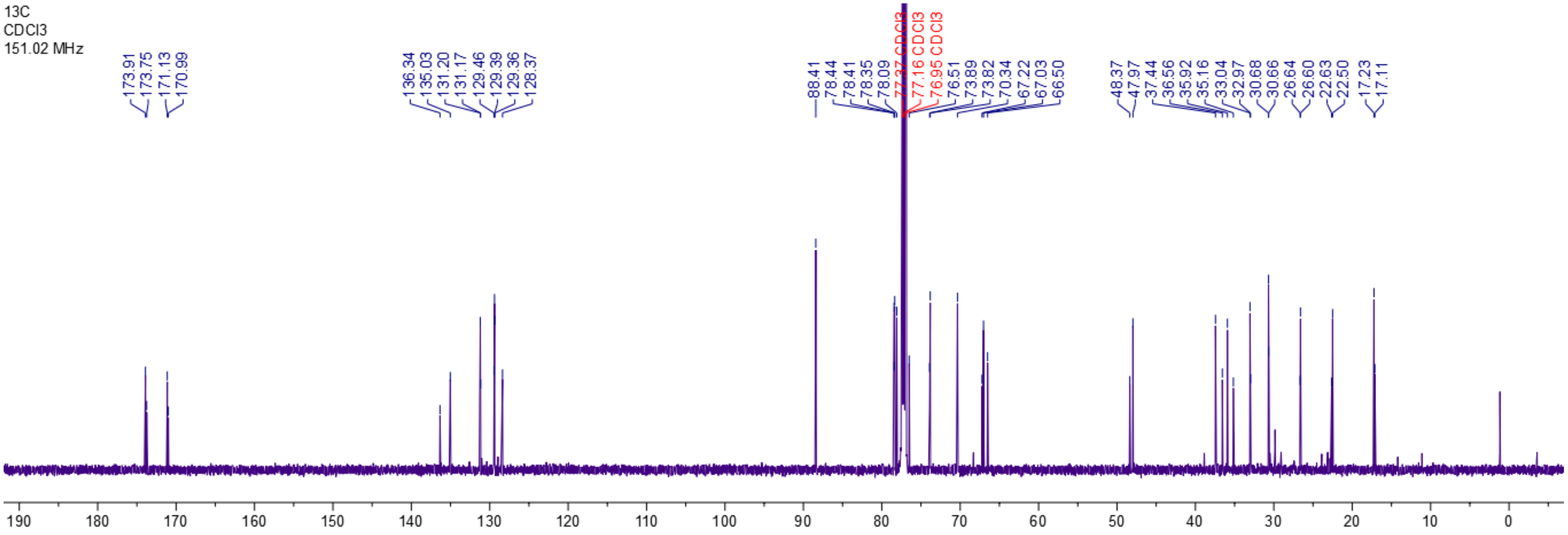
13C
CDCl3
151.02 MHz
173.91
173.75
171.13
170.99
136.34
135.03
131.20
131.17
129.46
129.39
129.36
128.37
88.41
78.44
78.41
78.35
78.09
77.37 CDCl3
77.16 CDCl3
76.95 CDCl3
76.51
73.89
73.82
70.34
67.22
67.03
66.50
48.37
47.97
37.44
36.56
35.92
35.16
33.04
32.97
30.68
30.66
26.64
26.60
22.63
22.50
17.23
17.11
190
180
170
160
150
140
130
120
110
100
90
80
70
60
50
40
30
20
10
0

To a solution of **S105** (10.3 mg, 0.0276 mmol, 1.0 eq.) in anhydrous DMSO (deoxygenated via 5x freeze-pump-thaw cycles) (0.5 mL) was added $CrCl_2$ with doped with 1 % $NiCl_2$ (w/w) (34.0 mg, 0.276 mmol, 10 eq.). **3c** (20.1 mg, 0.0800 mmol, 3.0 eq.) was added in deoxygenated DMSO (0.5 mL) and the mixture was stirred for 66 h. The reaction was quenched with 1:1 $H_2O$/brine (15 mL). The aqueous layer was extracted with EtOAc (8x 15 mL) and the combined organic layers were dried ($Na_2SO_4$), filtered, and the solvent was removed in vacuo. The crude product was purified via flash column chromatography (99:1 to 97:3 $CH_2Cl_2$/EtOH). Appropriate fractions were pooled, and solvent was removed in vacuo to yield **S108** (3.5 mg, 23% over two steps) as a colorless oil and a mixture of diastereomers (5:4).

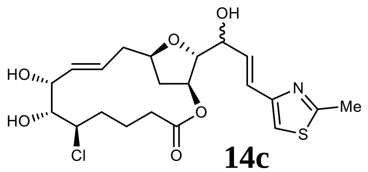


**14c**

Note: Reaction performed in capped vessel under ambient atmosphere.
To a stirred vial containing **S108** (3.5 mg, 0.0070 mmol, 1.0 eq.) dissolved in 1:1 $CH_2Cl_2$/MeOH (0.75 mL) was added 1.2M HCl in MeOH (29 µL, 0.035 mmol, 5.0 eq.). After 1 day stirring at rt, the reaction was quenched with $NaHCO_{3\ (s)}$ (15 mg) and after effervescence had subsided, the solvent was removed in vacuo. The crude product was purified via flash column chromatography (97:3 to 19:1 $CH_2Cl_2$/EtOH). Appropriate fractions were pooled to yield **14c** (2.4 mg, 75%) as a colorless oil and a mixture of diastereomers (5:4)

**Analytical Data for 14c:**

$R_f$ = 0.20 (23:2 $CH_2Cl_2$/EtOH)

For the following reported NMR peaks: Several resonances in the $^1$H NMR spectrum integrated for 0.5 and 0.4 protons and are unique to both the diastereomers. In these cases an integral value of "0.5 H" and "0.4 H" is assigned. All resolved $^{13}$C NMR signals are reported.

$^1$H NMR (601 MHz, $CDCl_3$) δ 6.95 (d, *J* = 4.4 Hz, 1H), 6.69 (dd, *J* = 15.5, 7.5 Hz, 1H), 6.58 (td, *J* = 15.5, 5.9 Hz, 1H), 5.90 (ddt, *J* = 14.9, 9.1, 4.2 Hz, 1H), 5.74 (dt, *J* = 14.2, 8.8 Hz, 1H), 5.23 (d, *J* = 8.4 Hz, 0.5H), 5.13 (d, *J* = 8.3 Hz, 0.4H), 4.55 – 4.48 (m, 1H), 4.41 (s, 0.5H), 4.34 (t, *J* = 4.4 Hz, 0.4H), 4.21 – 4.11 (m, 2H), 3.90 – 3.80 (m, 2H), 2.84 – 2.74 (m, 2H), 2.70 (d, *J* = 0.9 Hz, 3H), 2.60 – 2.49 (m, 2H), 2.39 (s, 1H), 2.31 – 2.24 (m, 1H), 2.17 – 2.09 (m, 1H), 2.06 – 1.98 (m, 1H), 1.97 – 1.89 (m, 1H), 1.72 – 1.69 (m, 3H).

$^{13}$C NMR (151 MHz, $CDCl_3$) δ 173.71, 173.64, 131.62, 131.59, 129.41, 129.31, 115.64, 115.52, 88.01, 87.96, 78.34, 78.30, 78.28, 78.01, 77.62, 77.56, 73.80, 73.77, 73.01, 71.93, 66.81, 66.74, 37.45, 37.34, 36.13, 32.96, 32.94, 30.56, 30.52, 22.84, 22.39, 22.38, 19.42.

HRMS (ESI): Anal. Calcd. for $C_{21}H_{29}ClNO_6S^+$ $[M+H]^+$ 458.1399, found 458.1398

IR (neat): $\nu_{max}$ $(cm^{-1})$ = 3412 (br, OH), 2943 (m, CH), 2905 (m, CH), 1725 (s, C=O), 1710 (s, C=O), 1504 (w), 1430 (m), 1379 (m), 1335 (m), 1257 (m)

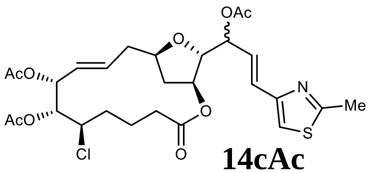


**14cAc**

**14cAc** was prepared according to general procedure A (0.12 mg, 19%).

**Analytical Data for 14cAc:**

$R_f$ = 0.57 (1:1 hexanes/EtOAc)

HRMS (ESI): Anal. Calcd. for $C_{27}H_{35}ClNO_9S^+$ $[M+H]^+$ 584.1716, found 584.1712

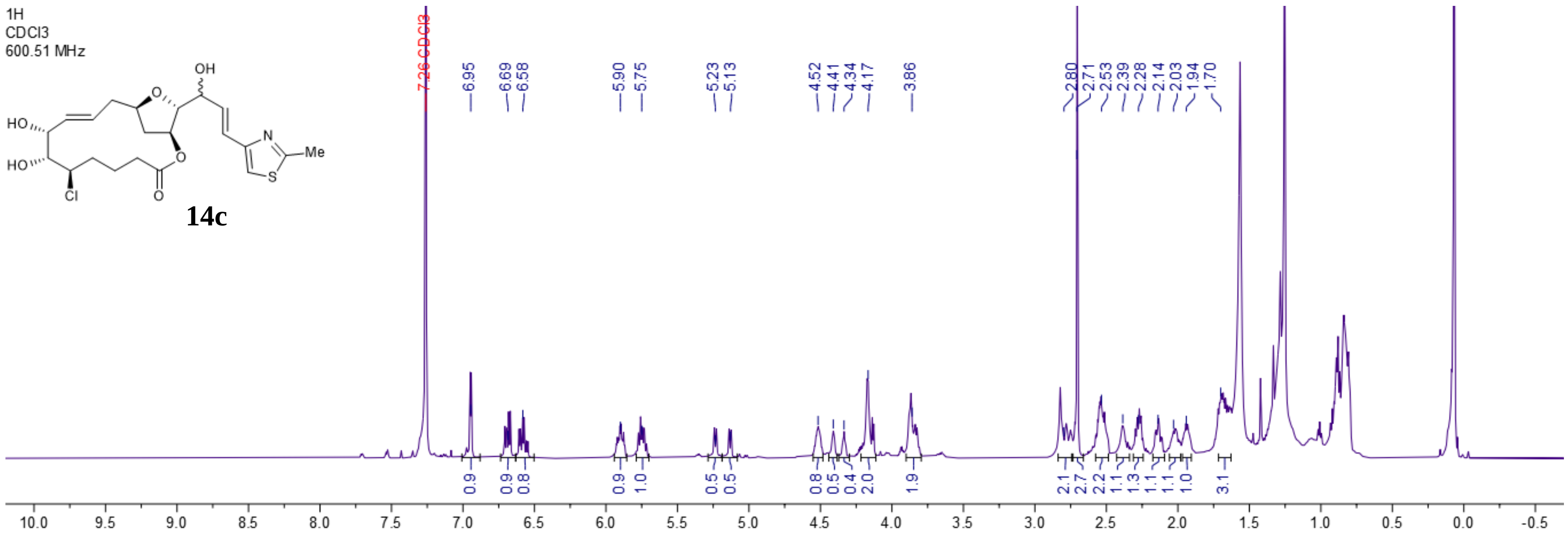
1H
CDCl3
600.51 MHz
OH
HO
HO
Cl
O
N
Me
S
14c
7.26 CDCl3
6.95
6.69
6.58
5.90
5.75
5.23
5.13
4.52
4.41
4.34
4.17
3.86
2.80
2.71
2.53
2.39
2.28
2.14
2.03
1.94
1.70
0.9
0.9
0.8
0.9
1.0
0.5
0.5
0.8
0.5
0.4
2.0
1.9
2.1
2.7
2.2
1.1
1.3
1.1
1.1
1.0
3.1
10.0 9.5 9.0 8.5 8.0 7.5 7.0 6.5 6.0 5.5 5.0 4.5 4.0 3.5 3.0 2.5 2.0 1.5 1.0 0.5 0.0 -0.5

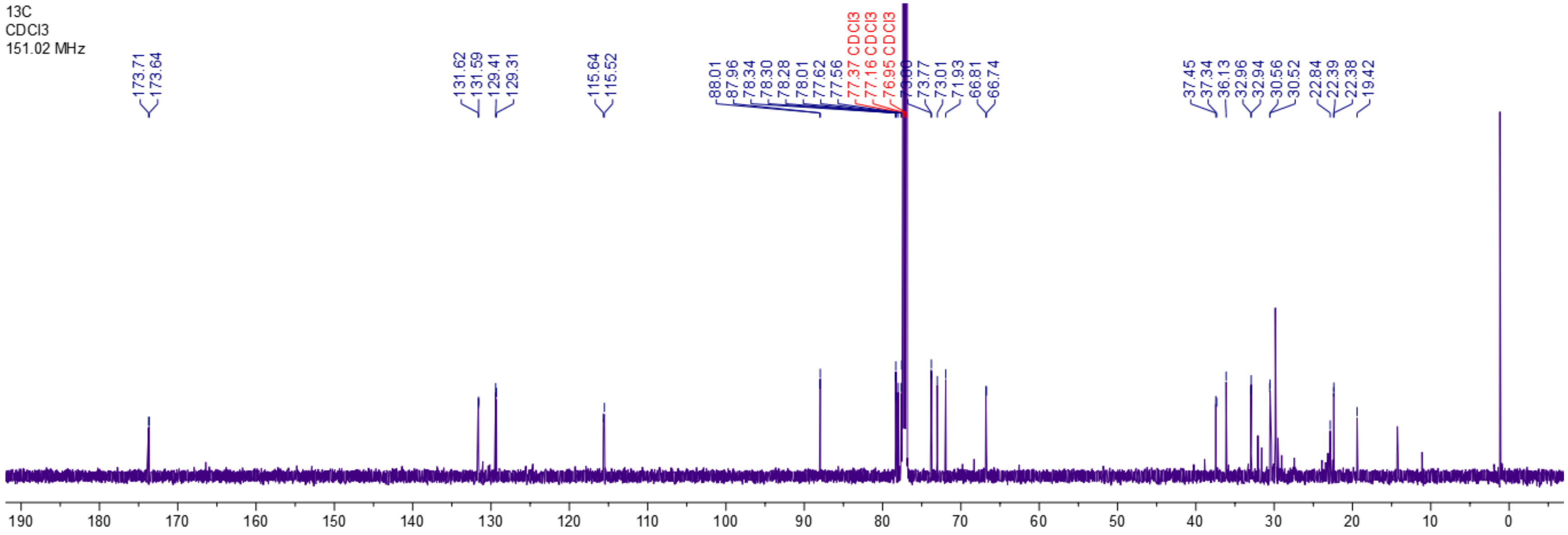
13C
CDCl3
151.02 MHz
173.71
173.64
131.62
131.59
129.41
129.31
115.64
115.52
88.01
87.96
78.34
78.30
78.28
78.01
77.62
77.56
77.37 CDCl3
77.16 CDCl3
76.95 CDCl3
73.77
73.01
71.93
66.81
66.74
37.45
37.34
36.13
32.96
32.94
30.56
30.52
22.84
22.39
22.38
19.42
190 180 170 160 150 140 130 120 110 100 90 80 70 60 50 40 30 20 10 0

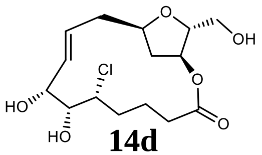


**14d**

Note: Reaction performed in capped vessel under ambient atmosphere.

To a stirred vial containing **S104** (2.4 mg, 0.0064 mmol, 1.0 eq.) dissolved in MeOH (1 mL) was added 1.2M HCl in MeOH (27 µL, 0.32 mmol, 5.0 eq.). After 1 day stirring at rt, the reaction was quenched with $NaHCO_{3\ (s)}$ (8 mg) and after effervescence had subsided, the solvent was removed in vacuo. The crude product was purified via flash column chromatography (24:1 to 19:1 $CH_2Cl_2$/EtOH). Appropriate fractions were pooled to yield **14d** (1.3 mg, 62%) as a colorless oil.

**Analytical Data for 14d:**

$R_f$ = 0.17 (47:3 $CH_2Cl_2$/EtOH)

$[\alpha]_D^{20}$ = -71 ° (c = 0.10, $CH_2Cl_2$)

$^1$H NMR (601 MHz, $CDCl_3$) δ 5.91 (ddd, *J* = 15.7, 10.5, 3.8 Hz, 1H), 5.77 (ddd, *J* = 15.6, 8.9, 1.9 Hz, 1H), 5.02 (dt, *J* = 8.6, 2.0 Hz, 1H), 4.49 – 4.44 (m, 1H), 4.20 – 4.14 (m, 2H), 3.93 – 3.82 (m, 2H), 3.71 – 3.62 (m, 2H), 2.83 (d, *J* = 1.7 Hz, 1H), 2.78 (dddd, *J* = 14.7, 5.6, 3.8, 2.1 Hz, 1H), 2.57 – 2.47 (m, 2H), 2.40 (d, *J* = 7.5 Hz, 1H), 2.29 (ddd, *J* = 15.8, 9.6, 2.1 Hz, 1H), 2.15 (ddd, *J* = 14.9, 10.4, 1.9 Hz, 1H), 2.07 – 1.99 (m, 1H), 1.99 – 1.93 (m, 1H), 1.92 – 1.87 (m, 1H), 1.76 – 1.70 (m, 2H), 1.68 – 1.62 (m, 1H).

$^{13}$C NMR (151 MHz, $CDCl_3$) δ 173.55, 131.59, 129.42, 85.66, 78.35, 77.84, 77.06, 73.79, 66.85, 62.56, 37.49, 35.81, 32.88, 30.55, 22.51.

HRMS (ESI): Anal. Calcd. for $C_{15}H_{23}ClNaO_6^+$ $[M+Na]^+$ 357.1075, found 357.1091
IR (neat): $\nu_{max}$ $(cm^{-1})$ = 3042 (br, OH), 2918 (s, CH), 1725 (s, C=O), 1709 (s, C=O), 1339 (m), 1272 (s), 1257 (s)

**14dAc**

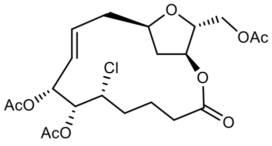


**14dAc** was prepared according to general procedure A (0.47 mg, 52%).

**Analytical Data for 14dAc:**

$R_f$ = 0.50 (1:1 hexanes/EtOAc)

HRMS (ESI): Anal. Calcd. for $C_{21}H_{33}ClNO_9^+$ $[M+NH_4]^+$ 478.1838, found 478.1845

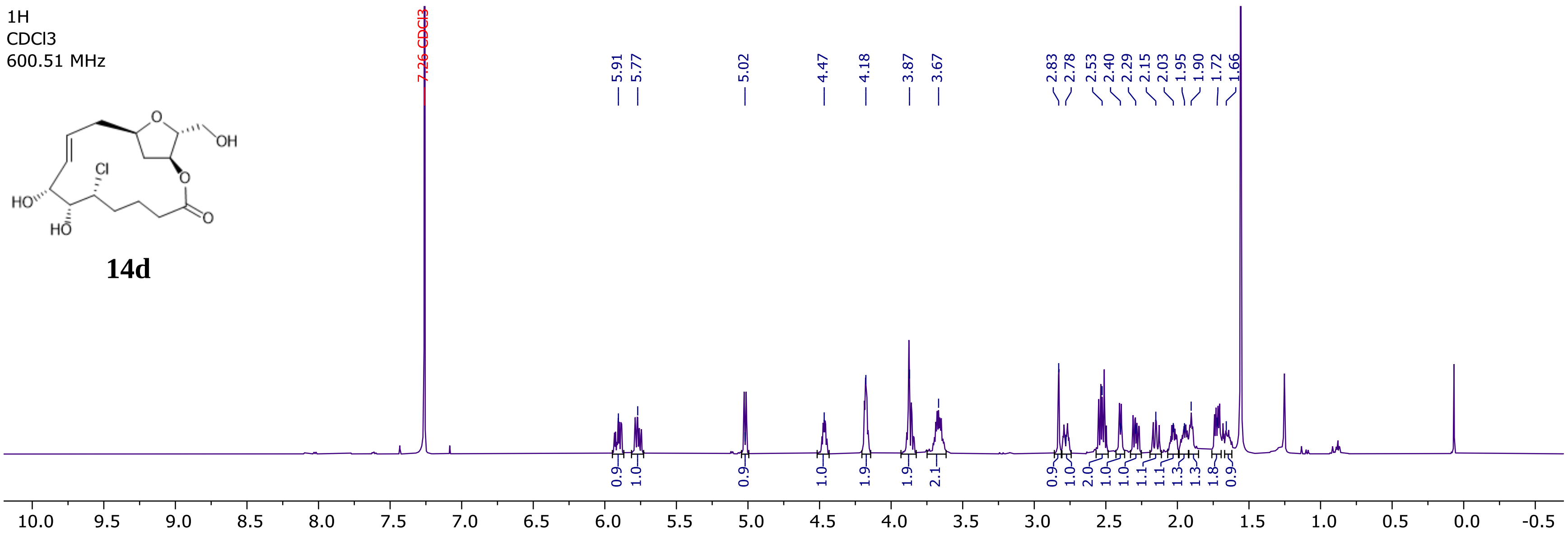
1H
CDCl3
600.51 MHz
7.26 CDCl3
5.91
5.77
5.02
4.47
4.18
3.87
3.67
2.83
2.78
2.53
2.40
2.29
2.15
2.03
1.95
1.90
1.72
1.66
0.9
1.0
0.9
1.0
1.9
1.9
2.1
0.9
1.0
2.0
1.0
1.0
1.1
1.1
1.3
1.3
1.8
0.9
10.0 9.5 9.0 8.5 8.0 7.5 7.0 6.5 6.0 5.5 5.0 4.5 4.0 3.5 3.0 2.5 2.0 1.5 1.0 0.5 0.0 -0.5
O
OH
O
O
Cl
HO
HO

**14d**

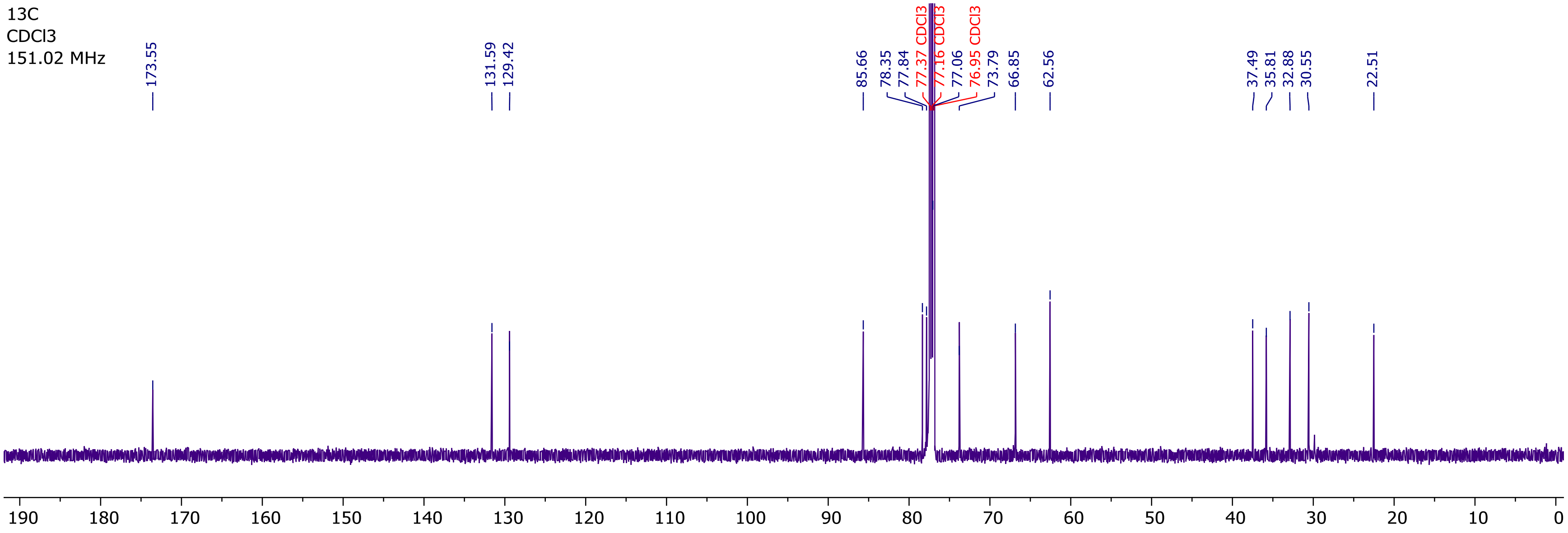
13C
CDCl3
151.02 MHz
173.55
131.59
129.42
85.66
78.35
77.84
77.37 CDCl3
77.16 CDCl3
77.06
76.95 CDCl3
73.79
66.85
62.56
37.49
35.81
32.88
30.55
22.51
190 180 170 160 150 140 130 120 110 100 90 80 70 60 50 40 30 20 10 0

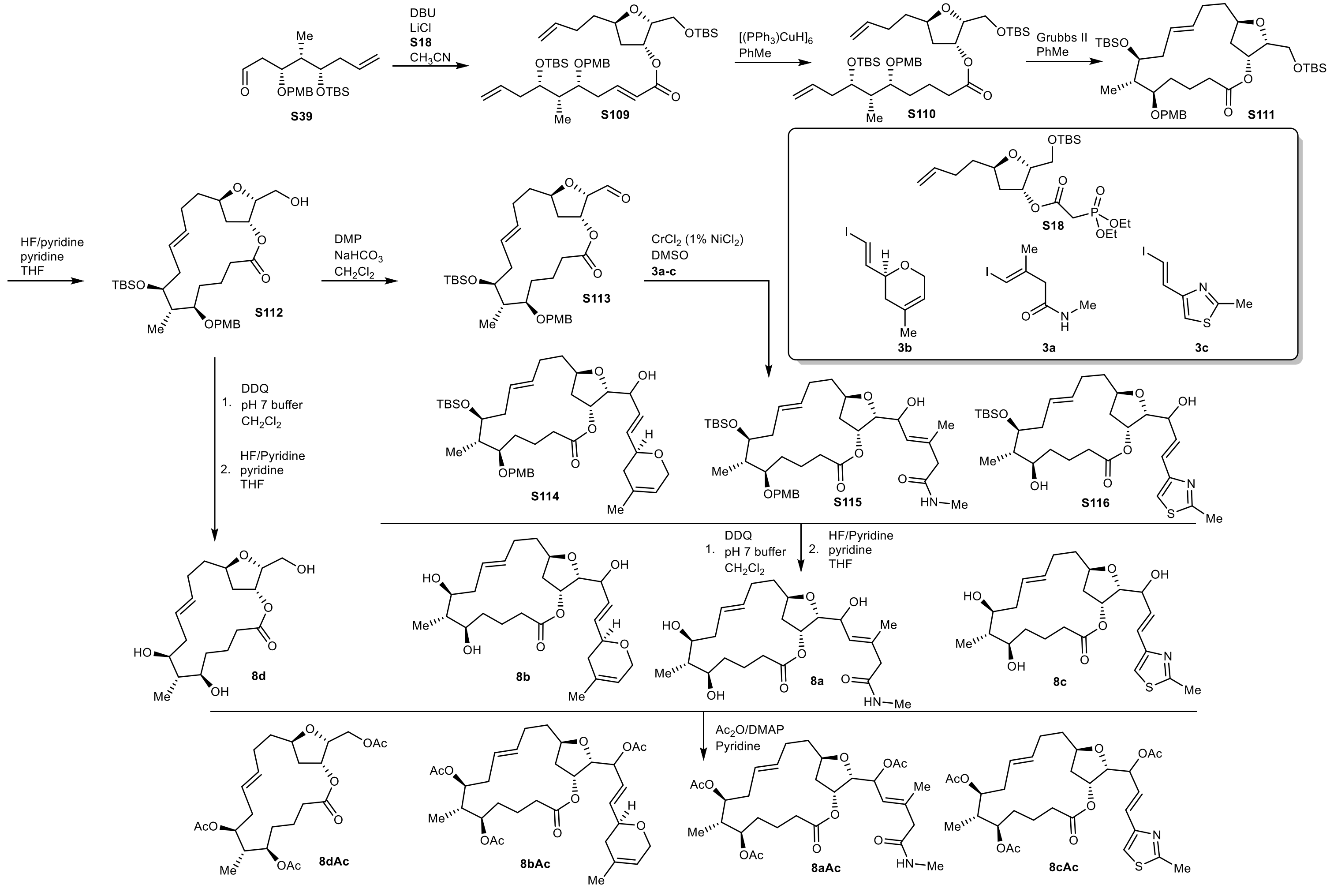


**Supplementary Fig. 45 | Synthesis of pMLs 8a-d and 8aAc-dAc.**

Abbreviations: TBS = tert-butyldimethylsilyl, PMB = para-methoxybenzyl, DBU = 1,8-Diazabicyclo[5.4.0]undec-7-ene, Grubbs II = Dichloro[1,3-bis(2,4,6-trimethylphenyl)-2-imidazolidinylidene](benzylidene)(tricyclohexylphosphine)ruthenium(II), THF = tetrahydrofuran, DMP = Dess-Martin-periodinane, DDQ = 2,3-dichloro-5,6-dicyano-*para*-benzoquinone, DMSO = dimethylsulfoxide, DMAP = 4-dimethylaminopyridine.

To a stirred solution of **S18** (246 mg, 0.53 mmol, 1.1 eq.) and LiCl (25.0 mg, 0.60 mmol, 1.2 eq.) in anhydrous $CH_3CN$ (2 mL) was added DBU (38 µL, 38 mg, 0.25 mmol, 1.1 eq.) followed by crude aldehyde **S39** (197 mg, 0.48 mmol, 1.0 eq.) After 1 h, the reaction was quenched with a saturated solution of $NH_4Cl$ (2 mL) followed by $H_2O$ (10 mL) and the aqueous layer was extracted with $CH_2Cl_2$ (3x 25 mL) and the combined organic layers were washed with brine (40 mL), dried ($Na_2SO_4$), filtered, and the solvent was removed in vacuo. The crude product was purified by flash column chromatography (37:3 to 17:3 hexanes/$Et_2O$). Appropriate fractions were pooled and the solvent was removed in vacuo to yield **S109** (320 mg, 0.45 mmol, 89% over 2 steps) as a colorless oil.

To a stirred solution of **S109** (312 mg, 0.44 mmol, 1.0 eq) in anhydrous, deoxygenated toluene (deoxygenated via sparging with nitrogen for 30 minutes) (5 mL) was added Stryker's reagent in anhydrous toluene (0.37 M, 5 mL, 2.20 mmol, 5.0 eq.). The mixture was stirred for 210 minutes then flushed through a plug of silica gel (1:1 hexanes/$Et_2O$) and the solvent was removed in vacuo. The crude product was purified via flash column chromatography (37:3 to 17:3 hexanes/$Et_2O$). Appropriate fractions were pooled, and solvent was removed in vacuo to yield **S110** (204 mg, 0.28 mmol, 65%) as a colorless oil.

To a stirred, heated (60 °C) solution of **S110** (204 mg, 0.28 mmol, 1.0 eq.) in anhydrous toluene (deoxygenated via 5x freeze-pump-thaw cycles) (112 mL) was added Grubbs 2$^{nd}$ generation catalyst (48.1 mg, 0.057 mmol, 0.20 eq.) in anhydrous, deoxygenated toluene (2.3 mL). After 1 h stirring at 60 °C, the reaction was cooled to 0 °C and quenched with potassium 2-isocyanoacetate (49.0 mg) in MeOH (2 mL). The mixture was warmed to rt and stirred for 30 minutes, then run through a plug of silica ($Et_2O$) and the solvent was removed in vacuo. The crude product was purified via flash column chromatography (9:1 hexanes/$Et_2O$). Appropriate fractions were pooled, and solvent was removed in vacuo to yield (*E*)-isomer **S111** (124 mg, 0.18 mmol, 63%) as a brown oil.

Note: Reaction run under ambient atmosphere
To a plastic vessel containing a stirred solution of **S111** (124 mg, 0.18 mmol, 1.0 eq.) in anhydrous THF (2.1 mL) was added a stock solution of HF in THF-Pyridine (2.56 M, 1.3 mL, 3.4 mmol, 19 eq. HF, 4:1 pyridine:HF). After stirring for 26 h at rt, the reaction was quenched with saturated $NaHCO_3$ (5 mL) and the aqueous layer was extracted with $CH_2Cl_2$ (3x 15 mL) and the combined organic layers were washed with brine (15 mL), dried ($Na_2SO_4$), filtered, and the solvent was removed in vacuo. The crude product was purified via flash column chromatography (7:3 hexanes/EtOAc). Appropriate fractions were pooled, and solvent was removed in vacuo to yield **S112** (103 mg, 0.18 mmol, 99%) as a colorless oil.

To a stirred solution of **S112** (48.2 mg, 0.083 mmol, 1.0 eq.) in anhydrous $CH_2Cl_2$ (1.3 mL) was added anhydrous pyridine (34 µL, 33 mg, 0.42 mmol, 5.0 eq.) followed by Dess-Martin Periodinane (70.3 mg, 0.17 mmol, 2.0 eq.). The mixture was stirred at rt for 4 h and

quenched with a solution of 1:1:1 $H_2O$-saturated $NaHCO_3$-saturated $Na_2S_2O_3$ (1 mL) and the biphasic mixture was stirred vigorously for 0.5 h. The aqueous layer was diluted with $H_2O$ (5 mL) and extracted with $CH_2Cl_2$ (3x 10 mL). Then the combined organic layers were dried ($Na_2SO_4$), filtered, and the solvent was removed in vacuo. The crude product was run through a plug of silica gel (EtOAc) and solvent was removed in vacuo to yield the crude aldehyde **S113** (48 mg) which split into equal portions for the next steps.

To a solution of **S113** (30 mg, 0.052 mmol, 1.0 eq.) in anhydrous DMSO (deoxygenated via 5x freeze-pump-thaw cycles) (0.5 mL) was added $CrCl_2$ with doped with 1 % $NiCl_2$ (w/w) (67.6 mg, 0.55 mmol, 11 eq.). **3b** (22.0 mg, 0.088 mmol, 1.7 eq.) was added in deoxygenated DMSO (1.3 mL) and the mixture was stirred for 16 h. The reaction was cooled to 0 °C and quenched with 1:1 $H_2O$-brine (10 mL). The aqueous layer was extracted with $Et_2O$ (10x 10 mL) and the combined organic layers were dried ($MgSO_4$) and filtered followed by removal of the solvent in vacuo. The crude product was purified via flash column chromatography (19:1 to 4:1 hexanes/acetone). Appropriate fractions were pooled, and solvent was removed in vacuo to yield **S114** (7.1 mg, 0.010 mmol, 20%) as a colorless oil and a mixture of diastereomers (d.r. >19:1).

Note: Reaction run under ambient atmosphere
To a vigorously stirred solution of **S114** (7.1 mg, 0.010 mmol, 1.0 eq.) in $CH_2Cl_2$ (1 mL) and pH 7 phosphate buffer (0.2 mL) was added 2,3-dichloro-5,6-dicyano-benzoquinone (8.4 mg, 0.037 mmol, 3.7 eq.). After 30 minutes the starting material was consumed as monitored by TLC analysis and the reaction was quenched with saturated $NaHCO_3$ (2 mL). The aqueous layer was extracted with $CH_2Cl_2$ (3x 10 mL) and the combined organic layers were dried ($Na_2SO_4$) and filtered, followed by removal of the solvent in vacuo. The crude product was purified via flash column chromatography (17:3 to 4:1 hexanes-acetone). Appropriate fractions were pooled, and solvent was removed in vacuo to yield the diol (3.7 mg, 0.0064 mmol, 63%) as a yellow oil.

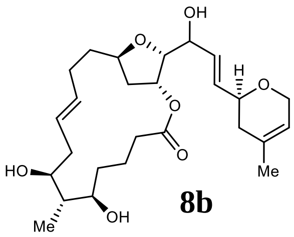

**8b**

Note: Reaction performed in capped vessel under ambient atmosphere.
To a plastic vessel containing the diol from the previous step (3.7 mg, 0.0064 mmol, 1.0 eq.) was added a 1.17 M of solution of HF in pyridine/THF (0.64 mL, 0.75 mmol HF, 117 eq., buffered 4:1 v/v pyridine:HF) and 70% HF/pyridine (0.02 mL). After 8 days stirring at rt, the reaction was quenched with saturated $NaHCO_3$ (3 mL) and after effervescence had subsided, the aqueous layer was extracted with $CH_2Cl_2$ (3x 10 mL). The combined organic layers were dried ($Na_2SO_4$), filtered, and the solvent was removed in vacuo. The crude product was purified via flash column chromatography (7:3 hexanes/acetone). Appropriate fractions were pooled to yield **8b** (2.5 mg, 84%) as a colorless oil and a mixture of diastereomers (d.r. >19:1).

**Analytical Data for 8b:**

$R_f$ = 0.22 (7:3 hexanes/acetone)

$[\alpha]_D^{20}$ = +6.4 ° (c = 0.25, MeOH)

$^1$H NMR (601 MHz, MeOD) δ 5.92 (ddd, *J* = 15.7, 5.3, 1.2 Hz, 1H), 5.84 (ddd, *J* = 15.7, 5.6, 1.3 Hz, 1H), 5.47 – 5.37 (m, 4H), 4.56 (s, 1H), 4.20 – 4.14 (m, *J* = 7.2, 4.2, 2.5, 1.5 Hz, 3H), 4.13 – 4.04 (m, 2H), 3.80 (dd, *J* = 8.8, 3.2 Hz, 1H), 3.76 – 3.68 (m, 2H), 2.56 (ddd, *J* = 14.2, 9.5, 4.8 Hz, 1H), 2.32 – 2.18 (m, 4H), 2.11 – 2.01 (m, *J* = 9.4, 8.3, 3.7, 2.6 Hz, 2H), 2.01 – 1.93 (m, 2H), 1.85 (ddt, *J* = 13.7, 8.9, 4.4 Hz, 1H), 1.81 – 1.73 (m, 3H), 1.73 – 1.70 (m, 3H), 1.61 – 1.53 (m, 2H), 1.48 (dddd, *J* = 13.3, 9.2, 5.5, 3.3 Hz, 1H), 1.36 – 1.29 (m, 3H), 0.91 (d, *J* = 7.0 Hz, 3H).

$^{13}$C NMR (151 MHz, MeOD) δ 174.44, 133.33, 132.68, 132.34, 128.97, 120.63, 84.71, 77.50, 76.62, 76.20, 75.10, 74.98, 70.39, 66.54, 40.81, 40.09, 38.70, 36.77, 35.17, 34.93, 34.42, 30.77, 29.76, 23.19, 23.05, 6.00.
HRMS (ESI): Anal. Calcd. for $C_{26}H_{41}O_7^+$ $[M+H]^+$ 465.2847, found 465.2863
IR (neat): $\nu_{max}$ $(cm^{-1})$ = 3409 (br, OH), 2924 (s, CH), 2852 (m, CH), 1725 (s, C=O), 1445 (m), 1237 (s)

HRMS (ESI): Anal. Calcd. for $C_{26}H_{41}O_7^+$ $[M+H]^+$ 465.2847, found 465.2863

IR ($CDCl_3$): $\nu_{max}$ $(cm^{-1})$ = 3409 (br, OH), 2924 (s, CH), 2852 (m, CH), 1725 (s, C=O)

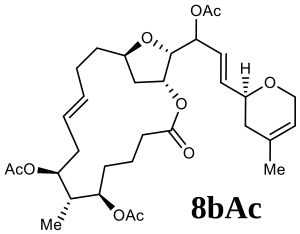


**8bAc**

**8bAc** was prepared according to general procedure A (0.68 mg, 53%).

**Analytical Data for 8bAc:**

$R_f$ = 0.86 (2:3 hexanes/EtOAc)

HRMS (ESI): Anal. Calcd. for $C_{32}H_{50}NO_{10}^+$ $[M+NH_4]^+$ 608.3429, found 608.3404

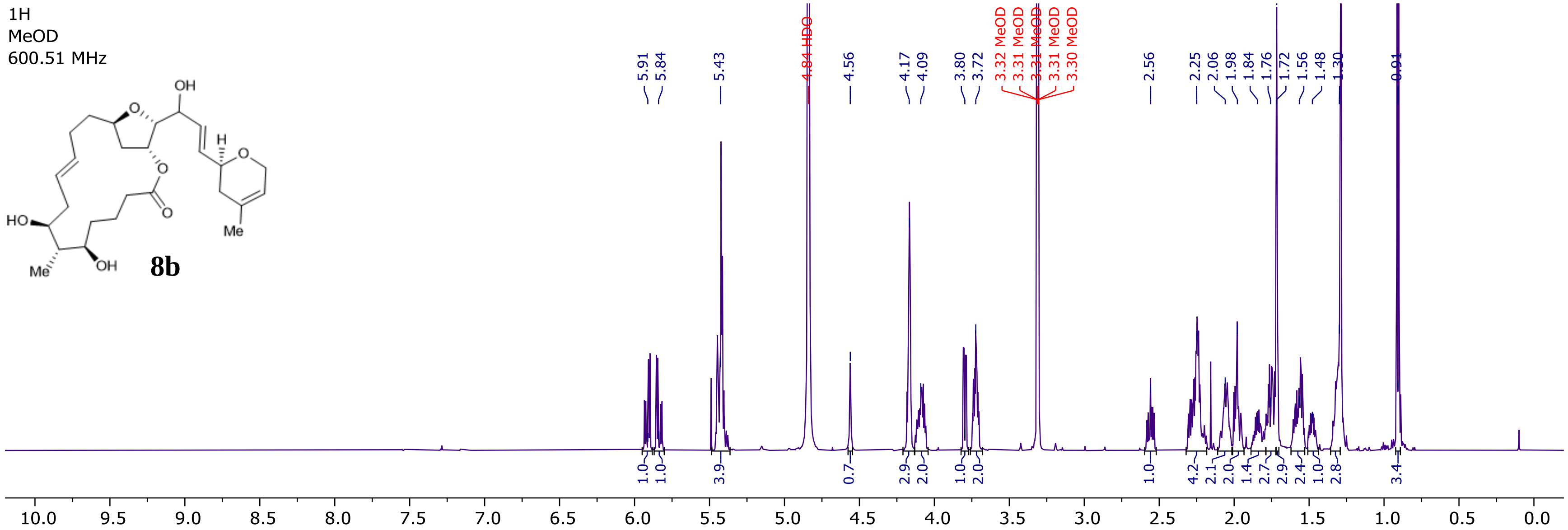
1H
MeOD
600.51 MHz
OH
O
H
O
O
O
HO
Me
Me
OH
5.91
5.84
5.43
4.84 HDO
4.56
4.17
4.09
3.80
3.72
3.32 MeOD
3.31 MeOD
3.31 MeOD
3.31 MeOD
3.30 MeOD
2.56
2.25
2.06
1.98
1.84
1.76
1.72
1.56
1.48
1.30
0.91
1.0
1.0
3.9
0.7
2.9
2.0
1.0
2.0
1.0
4.2
2.1
2.0
1.4
2.7
2.9
2.4
1.0
2.8
3.4
10.0
9.5
9.0
8.5
8.0
7.5
7.0
6.5
6.0
5.5
5.0
4.5
4.0
3.5
3.0
2.5
2.0
1.5
1.0
0.5
0.0

**8b**

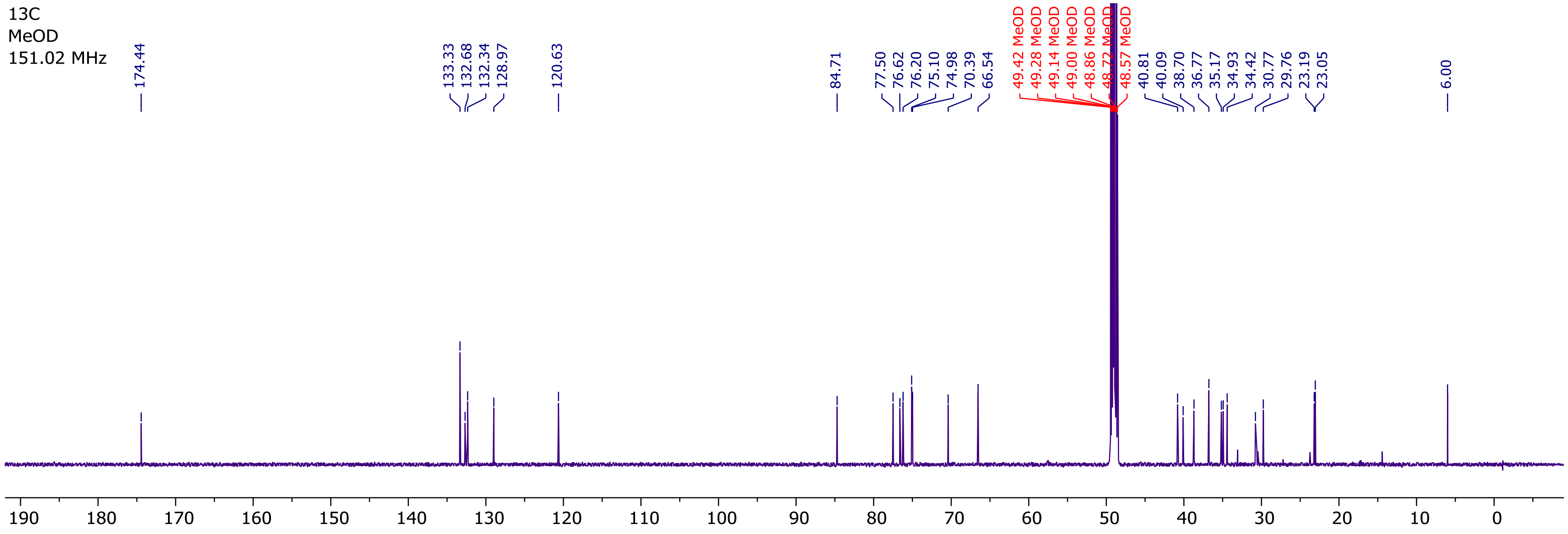
13C
MeOD
151.02 MHz
174.44
133.33
132.68
132.34
128.97
120.63
84.71
77.50
76.62
76.20
75.10
74.98
70.39
66.54
49.42 MeOD
49.28 MeOD
49.14 MeOD
49.00 MeOD
48.86 MeOD
48.72 MeOD
48.57 MeOD
40.81
40.09
38.70
36.77
35.17
34.93
34.42
30.77
29.76
23.19
23.05
6.00
190
180
170
160
150
140
130
120
110
100
90
80
70
60
50
40
30
20
10
0

To a solution of **S113** (21.6 mg, 0.038 mmol, 1.0 eq.) in DMSO (deoxygenated via 5x freeze-pump-thaw cycles) (0.5 mL) was added $CrCl_2$ with doped with 1 % $NiCl_2$ (w/w) (46.3 mg, 0.38 mmol, 10 eq.). **3a** (18 mg, 0.075 mmol, 2.0 eq.) was added in deoxygenated DMSO (0.75 mL) and the mixture was stirred for 16 h. The reaction was cooled to 0 °C and quenched with 1:1 $H_2O$-brine (10 mL). The aqueous layer was extracted with $Et_2O$ (10x 10 mL) and the combined organic layers were dried ($MgSO_4$) and filtered followed by removal of the solvent in vacuo. The crude product was purified via flash column chromatography (13:7 hexanes/acetone). Appropriate fractions were pooled, and solvent was removed in vacuo to yield **S115** (11.9 mg, 0.017 mmol, 46% over two steps) as a colorless oil and mixture of diastereomers (6:1).

Note: Reaction performed in capped vessel under ambient atmosphere.
To a vigorously stirred solution of **S115** (11.9 mg, 0.017 mmol, 1.0 eq.) in $CH_2Cl_2$ (1.5 mL) and phosphate buffer (pH 7, 0.3 mL) was added 2,3-dichloro-5,6-dicyano-benzoquinone (17 mg, 0.075 mmol, 4.4 eq.). After 15 minutes the starting material was consumed as monitored by TLC analysis and the reaction was quenched with saturated $NaHCO_3$ (2 mL). The aqueous layer was extracted with $CH_2Cl_2$ (3x 10 mL) and the combined organic layers were dried ($Na_2SO_4$) and filtered, followed by removal of the solvent in vacuo. The crude product was run through a plug of silica gel using acetone, and solvent was removed in vacuo to yield the crude diol (6.7 mg, 0.012 mmol, 68%) as a red oil and a mixture of diastereomers (6:1).

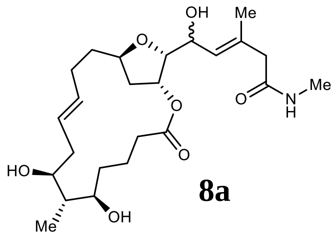


**8a**

Note: Reaction performed in capped vessel under ambient atmosphere.
To a plastic vessel containing the crude diol from the previous step (6.7 mg, 0.012 mmol, 1.0 eq.) was added a 1.17 M of solution of HF in pyridine/THF (0.90 mL, 1.05 mmol HF, 88 eq., buffered 4:1 v/v pyridine:HF) and 70% HF/pyridine (35 µL, 39 mg, 1.35 mmol HF, 113 eq.). After 2 days stirring at rt, the reaction was quenched with saturated $NaHCO_3$ (4 mL) and after effervescence had subsided, the aqueous layer was extracted with $CH_2Cl_2$ (3 x 10 mL). The combined organic layers were dried ($Na_2SO_4$), filtered, and the solvent was removed in vacuo. The crude product was purified via flash column chromatography (1:4 hexanes/acetone). Appropriate fractions were pooled to yield **8a** (4.6 mg, 86%) as a colorless oil and a mix of diastereomers (6:1).

**Analytical Data for 8a:**

$R_f$ = 0.25 (1:4 hexanes/acetone)

Major diastereomer

$^1$H NMR (601 MHz, $CDCl_3$) δ 6.25 (d, *J* = 6.0 Hz, 1H), 5.49 (t, *J* = 3.6 Hz, 1H), 5.43 – 5.29 (m, 3H), 4.42 (t, *J* = 8.3 Hz, 1H), 4.05 (ddt, *J* = 10.0, 7.8, 5.0 Hz, 1H), 3.94 – 3.86 (m, 2H), 3.84 – 3.77 (m, 1H), 3.06 (d, *J* = 15.6 Hz, 1H), 2.93 (d, *J* = 15.4 Hz, 1H), 2.78 (d, *J* = 4.7 Hz, 3H), 2.64 – 2.56 (m, 1H), 2.42 – 2.36 (m, 1H), 2.34 (t, *J* = 7.6 Hz, 1H), 2.28 – 2.23 (m, 1H), 2.22 – 2.16 (m, 2H), 2.09 (ddd, *J* = 16.8, 10.1, 4.9 Hz, 1H), 2.02 (dd, *J* = 13.4, 4.5 Hz, 1H), 1.89 – 1.79 (m, 3H), 1.76 (d, *J* = 1.4 Hz, 3H), 1.71 – 1.58 (m, 3H), 1.52 – 1.47 (m, 1H), 1.35 – 1.27 (m, 3H), 0.90 (d, *J* = 7.1 Hz, 3H).

<u>Major diastereomer</u>
$^{13}$C NMR (151 MHz, $CDCl_3$) δ 173.51, 171.50, 134.46, 132.41, 130.70, 128.08, 84.01, 76.53, 74.86, 66.48, 47.62, 39.62, 38.24, 33.91, 32.81, 32.07, 29.84, 29.47, 26.62, 21.69, 17.83, 14.26, 3.78.
HRMS (ESI): Anal. Calcd. for $C_{24}H_{40}NO_7^+$ $[M+H]^+$ 454.2799, found 454.2787
IR (neat): $\nu_{max}\ (cm^{-1})$ = 3409 (br, OH), 2927 (s, CH), 2852 (m, CH), 1727 (s, C=O), 1442 (m), 1237 (s)

HRMS (ESI): Anal. Calcd. for $C_{24}H_{40}NO_7^+$ $[M+H]^+$ 454.2799, found 454.2787

IR ($CDCl_3$): $\nu_{max}\ (cm^{-1})$ = 3409 (br, OH), 2927 (s, CH), 2852 (m, CH), 1727 (s, C=O)

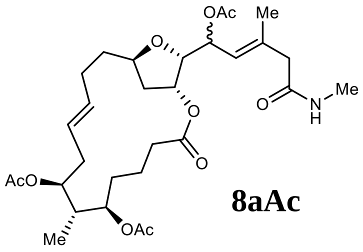


**8aAc**

**8aAc** was prepared according to general procedure A (0.71 mg, 62%).

**Analytical Data for 8aAc:**

$R_f$ = 0.42 (EtOAc)

<u>Major diastereomer</u>

$^1$H NMR (600 MHz, $C_6D_6$) δ 5.92 (t, *J* = 9.1 Hz, 1H), 5.75 – 5.66 (m, 1H), 5.51 (t, *J* = 3.5 Hz, 1H), 5.27 (dd, *J* = 22.2, 8.6 Hz, 2H), 5.22 – 5.12 (m, 2H), 5.02 – 4.95 (m, 1H), 4.16 – 4.08 (m, 1H), 3.91 (dd, *J* = 8.9, 3.4 Hz, 1H), 2.91 (d, *J* = 15.3 Hz, 1H), 2.83 (d, *J* = 15.3 Hz, 1H), 2.63 (d, *J* = 4.7 Hz, 3H), 2.57 (ddd, *J* = 15.0, 8.2, 3.3 Hz, 1H), 2.48 (ddd, *J* = 14.7, 7.5, 4.3 Hz, 1H), 2.36 – 2.30 (m, 1H), 2.06 (qt, *J* = 9.1, 4.3 Hz, 2H), 1.89 (s, 3H), 1.72 (s, 3H), 1.68 (s, 3H), 1.64 (s, 3H), 1.54 – 1.42 (m, 3H), 1.34 – 1.26 (m, 4H), 1.20 – 1.08 (m, 3H), 0.97 (d, *J* = 6.8 Hz, 3H).

Major diastereomer

$^{13}C$ NMR (151 MHz, $C_6D_6$) δ 172.50, 171.52, 170.04, 169.67, 169.28, 138.01, 132.96, 126.71, 126.18, 82.20, 77.19, 74.82, 73.52, 73.35, 68.41, 47.69, 39.24, 39.03, 34.88, 34.31, 33.89, 33.19, 28.75, 26.33, 22.49, 20.73, 20.67, 20.40, 17.90, 9.20.

IR (neat): $\nu_{max}$ $(cm^{-1})$ = 2926 (w, CH), 1738 (s, C=O), 1680 (w, 1680), 1372 (m), 1243 (s)

HRMS (ESI): Anal. Calcd. for $C_{30}H_{46}NO_{10}^+$ $[M+H]^+$ 580.3116, found 580.3133

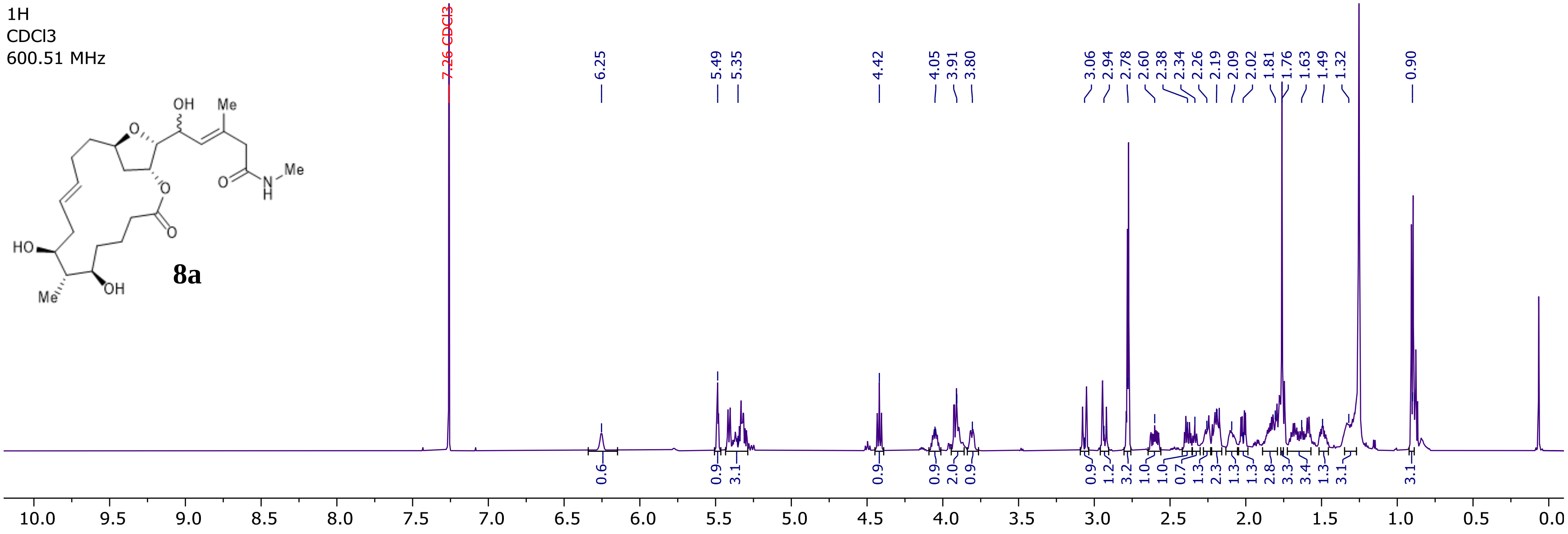
1H
CDCl3
600.51 MHz
7.26 CDCl3
6.25
5.49
5.35
4.42
4.05
3.91
3.80
3.06
2.94
2.78
2.60
2.38
2.34
2.26
2.19
2.09
2.02
1.81
1.76
1.63
1.49
1.32
0.90
0.6
0.9
3.1
0.9
0.9
2.0
0.9
0.9
1.2
3.2
1.0
1.0
0.7
1.3
2.3
1.3
1.3
2.8
3.3
3.4
1.3
3.1
3.1
10.0
9.5
9.0
8.5
8.0
7.5
7.0
6.5
6.0
5.5
5.0
4.5
4.0
3.5
3.0
2.5
2.0
1.5
1.0
0.5
0.0
OH
Me
O
O
O
N
H
Me
HO
Me
OH

**8a**

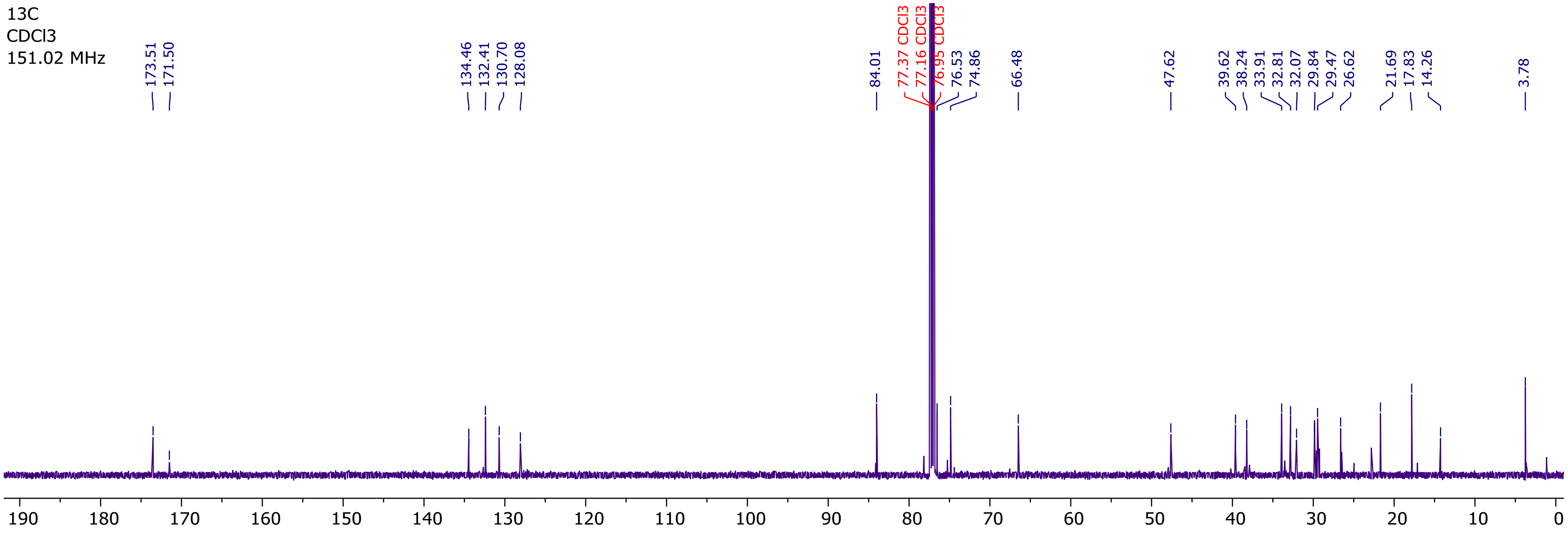
13C
CDCl3
151.02 MHz
173.51
171.50
134.46
132.41
130.70
128.08
84.01
77.37 CDCl3
77.16 CDCl3
76.95 CDCl3
76.53
74.86
66.48
47.62
39.62
38.24
33.91
32.81
32.07
29.84
29.47
26.62
21.69
17.83
14.26
3.78
190
180
170
160
150
140
130
120
110
100
90
80
70
60
50
40
30
20
10
0

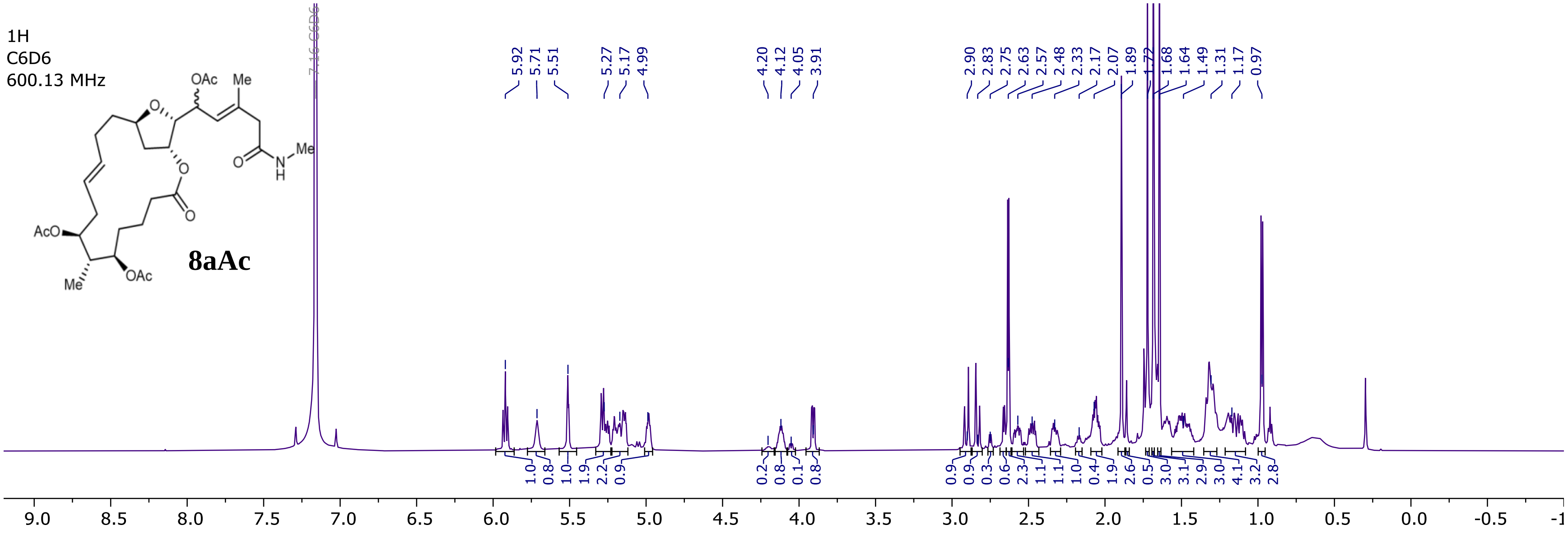
1H
C6D6
600.13 MHz
OAc
Me
O
O
O
N
H
Me
AcO
Me
OAc
8aAc
7.16 C6D6
5.92
5.71
5.51
5.27
5.17
4.99
4.20
4.12
4.05
3.91
2.90
2.83
2.75
2.63
2.57
2.48
2.33
2.17
2.07
1.89
1.72
1.68
1.64
1.49
1.31
1.17
0.97
1.0
0.8
1.0
1.9
2.2
0.9
0.2
0.8
0.1
0.8
0.9
0.9
0.3
0.6
2.3
1.1
1.1
1.0
0.4
1.9
2.6
0.5
3.0
3.1
2.9
3.0
4.1
3.2
2.8
9.0 8.5 8.0 7.5 7.0 6.5 6.0 5.5 5.0 4.5 4.0 3.5 3.0 2.5 2.0 1.5 1.0 0.5 0.0 -0.5 -1

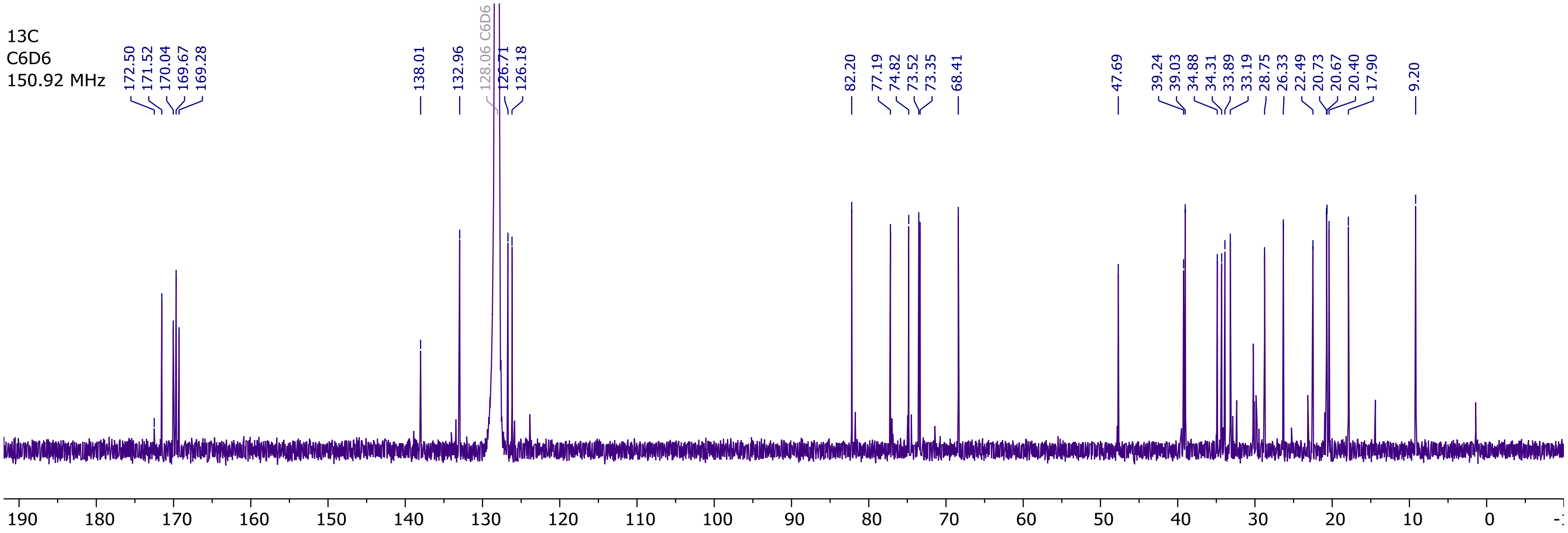
13C
C6D6
150.92 MHz
172.50
171.52
170.04
169.67
169.28
138.01
132.96
128.06 C6D6
126.71
126.18
82.20
77.19
74.82
73.52
73.35
68.41
47.69
39.24
39.03
34.88
34.31
33.89
33.19
28.75
26.33
22.49
20.73
20.67
20.40
17.90
9.20
190 180 170 160 150 140 130 120 110 100 90 80 70 60 50 40 30 20 10 0

To a solution of **S113** (21.6 mg, 0.038 mmol, 1.0 eq.) in anhydrous DMSO (deoxygenated via 5x freeze-pump-thaw cycles) (0.5 mL) was added $CrCl_2$ with doped with 1 % $NiCl_2$ (w/w) (46.3 mg, 0.38 mmol, 10 eq.). **3c** (9.0 µL, 15 mg, 0.060 mmol, 1.6 eq.) was added in deoxygenated DMSO (0.75 mL) and the mixture was stirred for 16 h. The reaction was cooled to 0 °C and quenched with 1:1 $H_2O$-brine (10 mL). The aqueous layer was extracted with $Et_2O$ (10x 10 mL) and the combined organic layers were dried ($MgSO_4$) and filtered followed by removal of the solvent in vacuo. The crude product was purified via flash column chromatography (17:3 to 4:1 hexanes/acetone). Appropriate fractions were pooled, and solvent was removed in vacuo to yield **S116** (8.8 mg, 0.013 mmol, 33% over two steps) as a colorless oil and mixture of diastereomers (5:1).

Note: Reaction performed in capped vessel under ambient atmosphere.
To a vigorously stirred solution of **S116** (8.8 mg, 0.013 mmol, 1.0 eq.) in $CH_2Cl_2$ (1 mL) and phosphate buffer (pH 7, 0.2 mL) was added 2,3-dichloro-5,6-dicyano-benzoquinone (10.8 mg, 0.048 mmol, 3.7 eq.). After 30 minutes the starting material was consumed as monitored by TLC analysis and the reaction was quenched with saturated $NaHCO_3$ (2 mL). The aqueous layer was diluted with $H_2O$ (10 mL) and extracted with $CH_2Cl_2$ (3x 20 mL). The combined organic layers were dried ($Na_2SO_4$) and filtered, followed by removal of the solvent in vacuo. The crude product was purified via flash column chromatography (3:1 hexanes/acetone). Appropriate fractions were pooled, and solvent was removed in vacuo to yield the diol (7.1 mg, 0.012 mmol, 98%) as a colorless oil and mixture of diastereomers (5:1). A portion of the material was taken to the next step.

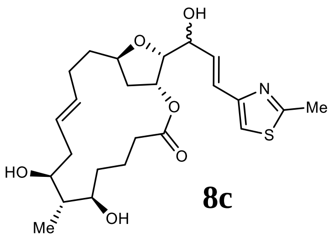


**8c**

Note: Reaction performed in capped vessel under ambient atmosphere.
To a plastic vessel containing the diol from the previous step (3.5 mg, 0.0060 mmol, 1.0 eq.) was added a 1.17 M of solution of HF in pyridine/THF (0.51 mL, 0.60 mmol HF, 100 eq., buffered 4:1 v/v pyridine:HF) and 70% HF/pyridine (31 µL, 34 mg, 1.21 mmol HF, 200 eq.). After 1 day stirring at rt, 70% HF/pyridine (50 µL, 55 mg, 1.93 mmol HF, 320 eq.) added. After 1 more day stirring at rt, the reaction was quenched with saturated $NaHCO_3$ (5 mL) and after effervescence had subsided, the aqueous layer was extracted with $CH_2Cl_2$ (3 x 15 mL). The combined organic layers were dried ($Na_2SO_4$), filtered, and the solvent was removed in vacuo. The crude product was purified via flash column chromatography (3:2 to 11:9 hexanes/acetone). Appropriate fractions were pooled to yield **8c** (2.3 mg, 82%) as a colorless oil and a mixture of diastereomers (5:1).

**Analytical Data for 8c:**

$R_f$ = 0.17 (11:9 hexanes/acetone)

Major diastereomer

$^{1}$H NMR (601 MHz, $CDCl_3$) δ 6.90 (s, 1H), 6.71 – 6.62 (m, 1H), 6.43 (dd, *J* = 15.5, 5.9 Hz, 1H), 5.41 – 5.31 (m, 3H), 4.51 (ddd, *J* = 7.6, 5.9, 1.4 Hz, 1H), 4.13 – 4.06 (m, 1H), 3.96 (dd, *J* = 8.1, 3.4 Hz, 1H), 3.88 – 3.82 (m, 2H), 2.70 (s, 3H), 2.53 (ddd, *J* = 15.0, 10.3, 4.9 Hz, 1H), 2.37 – 2.32 (m, 2H), 2.31 – 2.25 (m, *J* = 12.8, 8.5, 3.6 Hz, 2H), 2.23 – 2.16 (m, 2H), 2.11 – 2.04 (m, 1H), 2.00 (ddd, *J* = 13.6, 4.8, 0.8 Hz, 1H), 1.90 – 1.80 (m, 2H), 1.78 – 1.72 (m, 2H), 1.69 – 1.62 (m, 2H), 1.60 – 1.54 (m, 2H), 1.48 (tdd, *J* = 10.4, 4.8, 2.4 Hz, 1H), 0.89 (d, *J* = 7.0 Hz, 3H).

Major diastereomer

$^{13}$C NMR (151 MHz, $CDCl_3$) δ 172.70, 132.70, 127.94, 115.45, 83.91, 77.91, 75.37, 75.01, 71.24, 40.21, 38.55, 38.18, 33.95, 33.67, 33.53, 32.79, 32.08, 29.26, 24.90, 22.84, 21.62, 19.38, 14.27, 3.67.

HRMS (ESI): Anal. Calcd. for $C_{24}H_{36}NO_6S^+$ $[M+H]^+$ 466.2258, found 466.2267

IR (neat): $\nu_{max}\ (cm^{-1})$ = 3386 (br, OH), 2923 (s, CH), 2852 (m, CH), 1727 (s, C=O), 1441 (m), 1236 (s)

HRMS (ESI): Anal. Calcd. for $C_{24}H_{36}NO_6S^+$ $[M+H]^+$ 466.2258, found 466.2267

IR ($CHCl_3$): $\nu_{max}\ (cm^{-1})$ = 3386 (br, OH), 2923 (s, CH), 2852 (m, CH), 1727 (s, C=O)

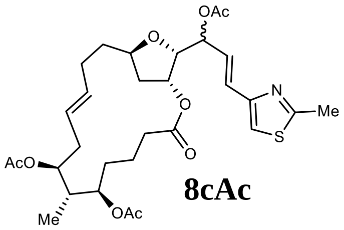


**8cAc**

**8cAc** was prepared according to general procedure A (0.73 mg, 56%).

**Analytical Data for 8cAc:**

$R_f$ = 0.52 (2:3 hexanes/EtOAc)

HRMS (ESI): Anal. Calcd. for $C_{30}H_{42}NO_9S^+$ $[M+H]^+$ 592.2575, found 592.2584

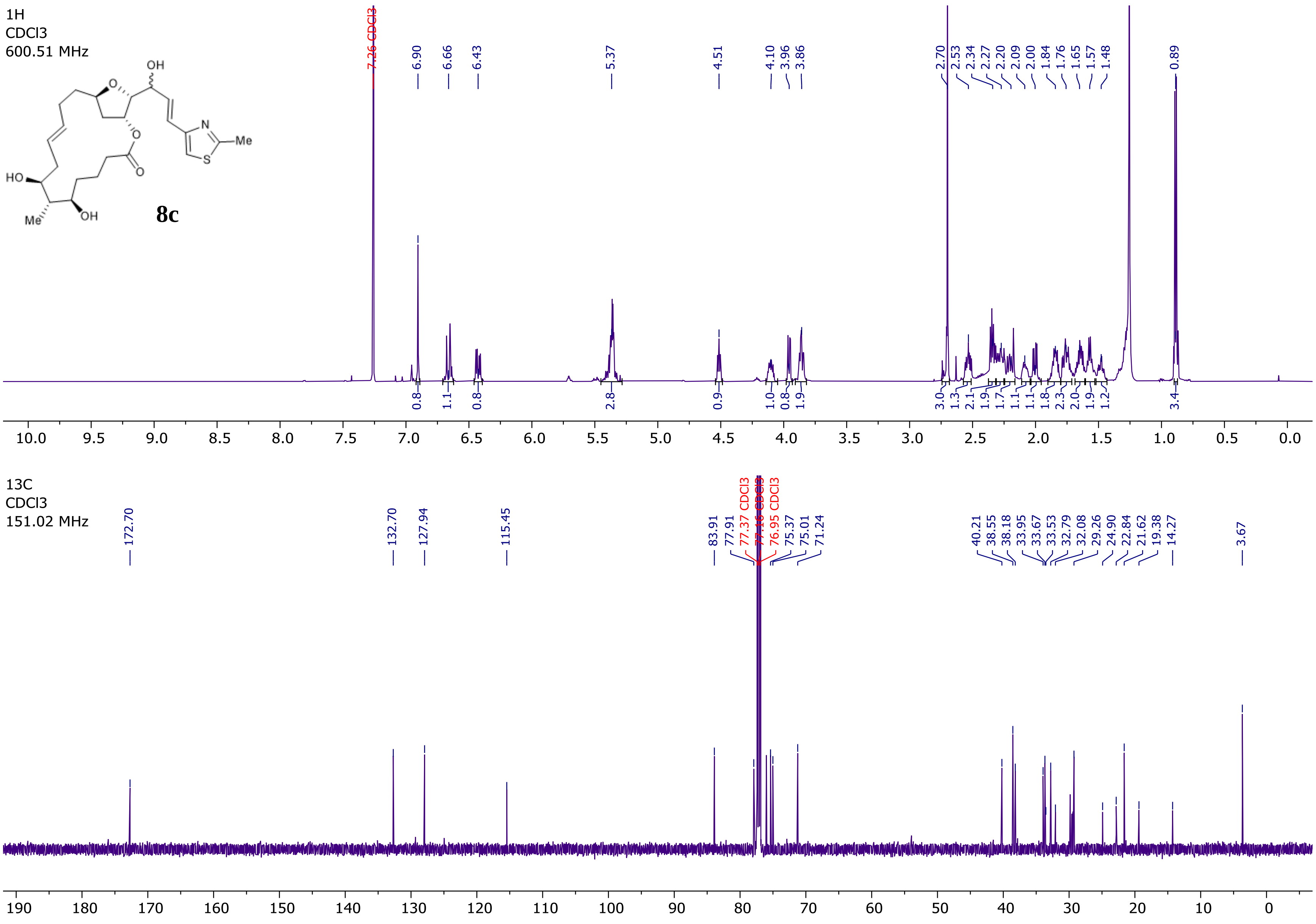
1H
CDCl3
600.51 MHz
8c
7.26 CDCl3
6.90
6.66
6.43
5.37
4.51
4.10
3.96
3.86
2.70
2.53
2.34
2.27
2.20
2.09
2.00
1.84
1.76
1.65
1.57
1.48
0.89
13C
CDCl3
151.02 MHz
172.70
132.70
127.94
115.45
83.91
77.91
77.37 CDCl3
76.95 CDCl3
75.37
75.01
71.24
40.21
38.55
38.18
33.95
33.67
33.53
32.79
32.08
29.26
24.90
22.84
21.62
19.38
14.27
3.67

Note: Reaction performed in capped vessel under ambient atmosphere.
To a vigorously stirred solution of **S112** (5.4 mg, 0.0094 mmol, 1.0 eq.) in $CH_2Cl_2$ (0.8 mL) and phosphate buffer (pH 7, 0.2 mL) was added 2,3-dichloro-5,6-dicyano-benzoquinone (8.5 mg, 0.037 mmol, 4.0 eq.). After 15 minutes the starting material was consumed as monitored by TLC analysis and the reaction was quenched with saturated $NaHCO_3$ (2 mL). The aqueous layer was extracted with $CH_2Cl_2$ (3x 15 mL) and the combined organic layers were dried ($Na_2SO_4$) and filtered, followed by removal of the solvent in vacuo. The crude product was purified via flash column chromatography (3:1 hexanes/acetone). Appropriate fractions were pooled, and solvent was removed in vacuo to yield the diol (3.1 mg, 0.0068 mmol, 73%) as a colorless oil.

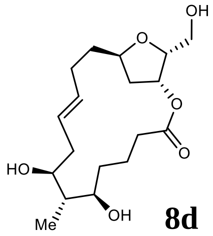

**8d**

Note: Reaction performed in capped vessel under ambient atmosphere.
To a plastic vessel containing the diol from the previous step (3.1 mg, 0.0068 mmol, 1.0 eq.) was added a 1.17 M of solution of HF in pyridine/THF (0.58 mL, 0.68 mmol HF, 100 eq., buffered 4:1 v/v pyridine:HF) and 70% HF/pyridine (27 µL, 29 mg, 1.02 mmol HF, 150 eq.). After 2 days stirring at rt, the reaction was quenched with saturated $NaHCO_3$ (10 mL) and after effervescence had subsided, the aqueous layer was extracted with $CH_2Cl_2$ (4 x 20 mL). The combined organic layers were dried ($Na_2SO_4$), filtered, and the solvent was removed in vacuo. The crude product was purified via flash column chromatography (3:2 hexanes/acetone). Appropriate fractions were pooled to yield **8d** (2.3-2.6 mg, quant.) as a colorless oil, and a mixture of product and transesterified side product (3:1).

**Analytical Data for 8d:**

$R_f$ = 0.28 (1:1 hexanes/acetone)

$[\alpha]_D^{20}$ = -3.5 ° (c = 0.26, $CH_3Cl$)

<u>Product peaks</u>
$^1$H NMR (601 MHz, $CDCl_3$) δ 5.42 (q, *J* = 4.8, 4.3 Hz, 1H), 5.36 (ddt, *J* = 5.4, 3.8, 1.9 Hz, 2H), 4.17 – 4.13 (m, 1H), 4.05 (dq, *J* = 11.6, 5.8 Hz, 1H), 3.86 (dddd, *J* = 15.2, 11.0, 3.9, 1.6 Hz, 2H), 3.74 (dd, *J* = 11.5, 6.7 Hz, 1H), 3.58 (dd, *J* = 11.6, 5.5 Hz, 1H), 2.52 (ddd, *J* = 14.8, 10.1, 5.1 Hz, 1H), 2.38 – 2.34 (m, 1H), 2.31 – 2.26 (m, 2H), 2.22 – 2.17 (m, 1H), 2.10 – 2.06 (m, 1H), 1.98 (dd, *J* = 13.5, 4.7 Hz, 1H), 1.91 – 1.81 (m, 2H), 1.76 (dddd, *J* = 13.3, 9.4, 5.9, 2.5 Hz, 2H), 1.69 – 1.62 (m, 2H), 1.62 – 1.55 (m, 2H), 1.49 (dddd, *J* = 13.7, 9.3, 5.9, 3.3 Hz, 1H), 1.37 – 1.30 (m, 2H), 0.90 (d, *J* = 7.1 Hz, 3H).

<u>Product peaks</u>
$^{13}$C NMR (151 MHz, $CDCl_3$) δ 173.17, 132.69, 127.64, 81.14, 78.22, 76.17, 75.25, 61.42, 39.60, 38.34, 38.06, 33.73, 33.69, 32.80, 29.43, 21.67, 14.27, 3.64.

HRMS (ESI): Anal. Calcd. for $C_{18}H_{31}O_6^+$ $[M+H]^+$ 343.2115, found 343.2112

IR (neat): $\nu_{max}$ $(cm^{-1})$ = 3384 (br, OH), 2918 (s, CH), 2849 (s, CH), 1725 (s, C=O), 1441 (m), 1239 (s)

**8dAc**

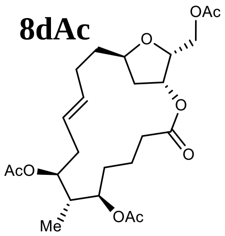


**8dAc** was prepared according to general procedure A (0.68 mg, 51%).

**Analytical Data for 8dAc:**

$R_f$ = 0.80 (2:3 hexanes/EtOAc)

HRMS (ESI): Anal. Calcd. for $C_{24}H_{40}NO_9^+$ $[M+NH_4]^+$ 486.2698, found 486.2673

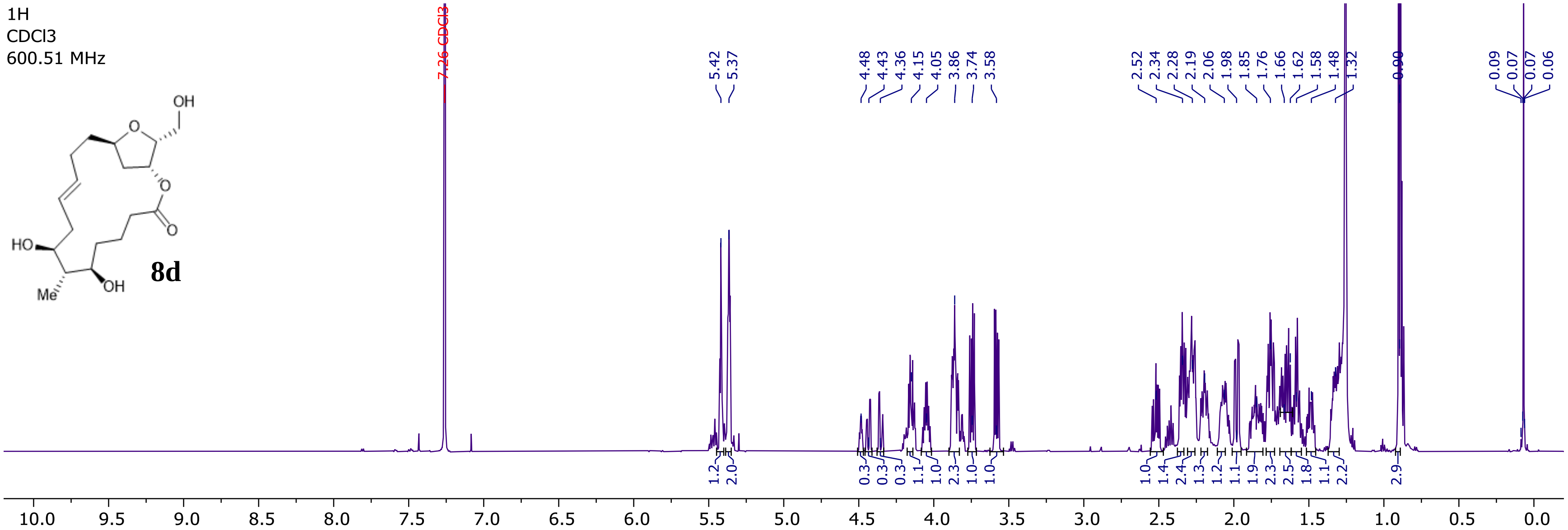
1H
CDCl3
600.51 MHz
OH
O
O
O
HO
Me
OH
8d
7.26 CDCl3
5.42
5.37
4.48
4.43
4.36
4.15
4.05
3.86
3.74
3.58
2.52
2.34
2.28
2.19
2.06
1.98
1.85
1.76
1.66
1.62
1.58
1.48
1.32
0.90
0.09
0.07
0.07
0.06
1.2
2.0
0.3
0.3
0.3
1.1
1.0
2.3
1.2
1.0
1.0
1.4
2.4
1.3
1.2
1.1
1.9
2.3
2.5
1.8
1.1
2.2
2.9
10.0
9.5
9.0
8.5
8.0
7.5
7.0
6.5
6.0
5.5
5.0
4.5
4.0
3.5
3.0
2.5
2.0
1.5
1.0
0.5
0.0

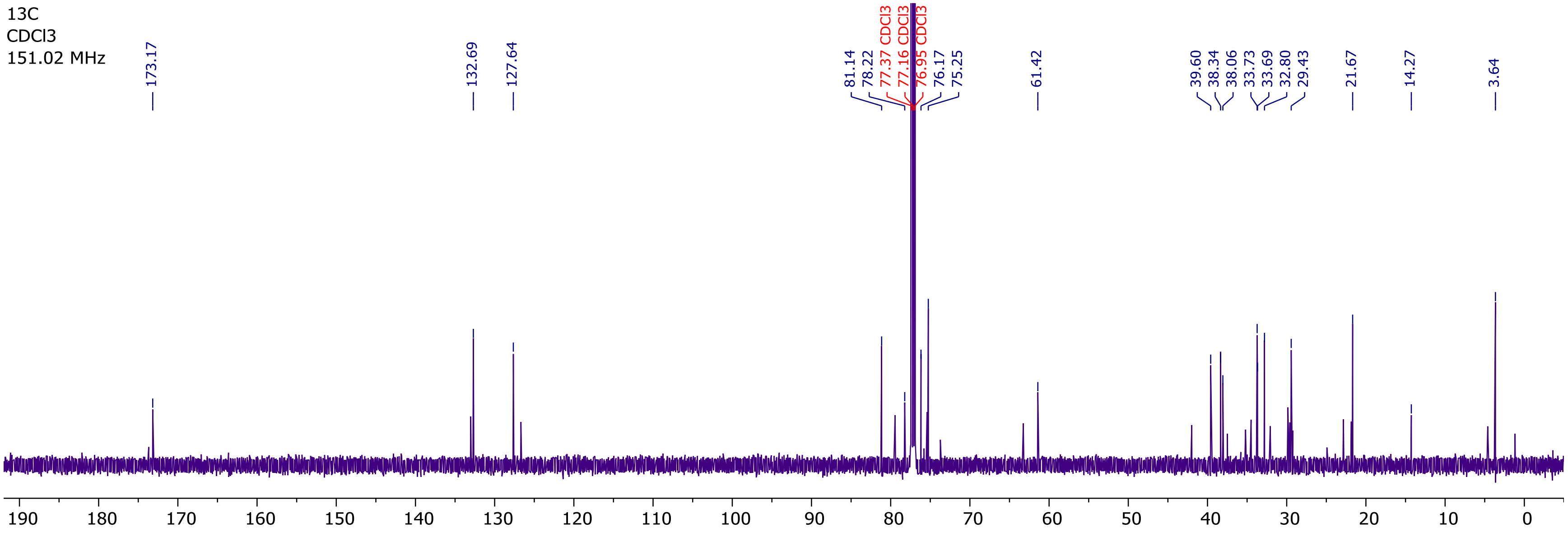
13C
CDCl3
151.02 MHz
173.17
132.69
127.64
81.14
78.22
77.37 CDCl3
77.16 CDCl3
76.95 CDCl3
76.17
75.25
61.42
39.60
38.34
38.06
33.73
33.69
32.80
29.43
21.67
14.27
3.64
190
180
170
160
150
140
130
120
110
100
90
80
70
60
50
40
30
20
10
0

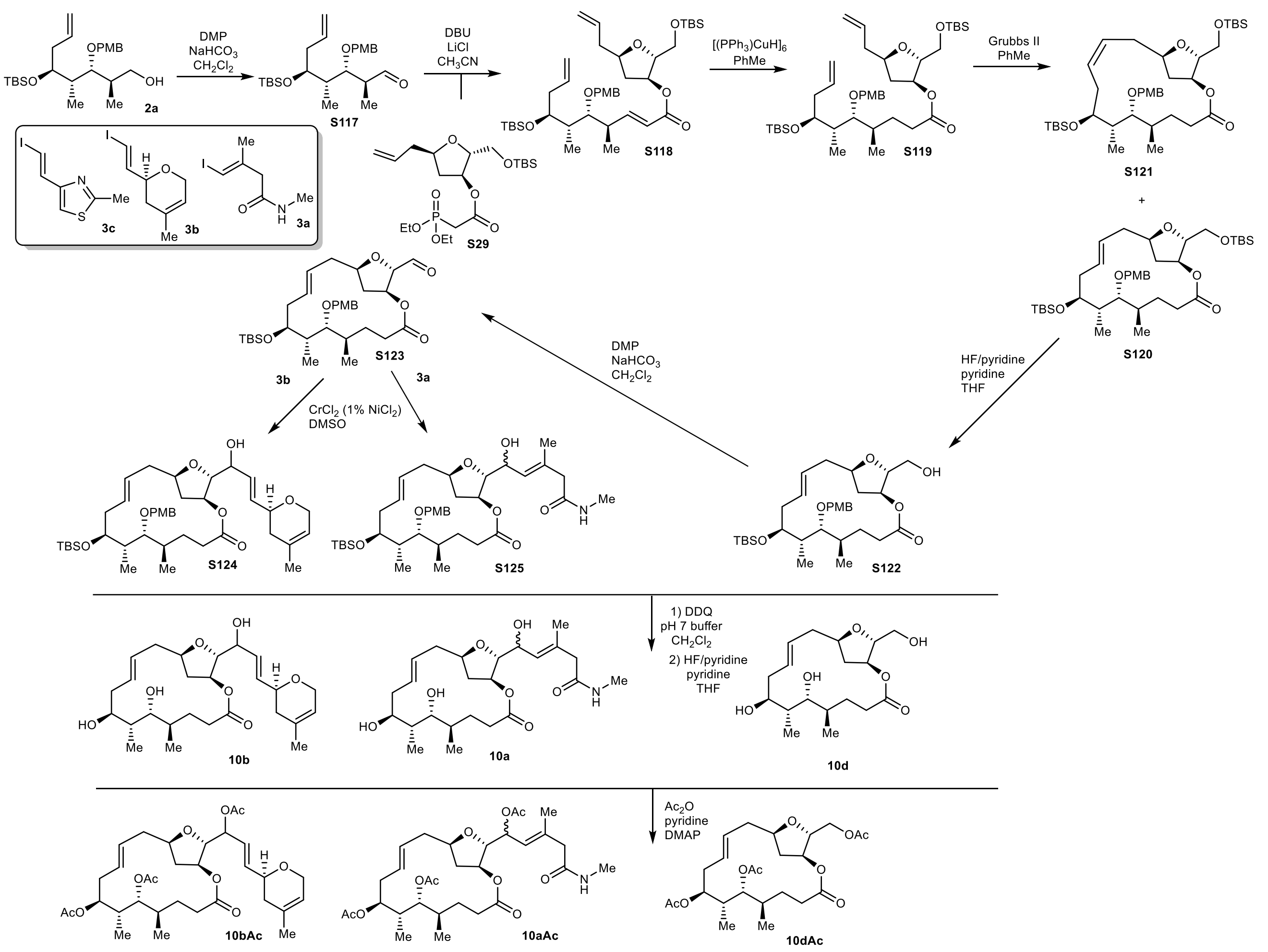


**Supplementary Fig. 46 | Synthesis of pMLs 10a-d and 10aAc-dAc.**

Abbreviations: TBS = tert-butyldimethylsilyl, PMB = *para*-methoxybenzyl, DBU = 1,8-Diazabicyclo[5.4.0]undec-7-ene, Grubbs II = Dichloro[1,3-bis(2,4,6-trimethylphenyl)-2-imidazolidinylidene](benzylidene)(tricyclohexylphosphine)ruthenium(II), THF = tetrahydrofuran, DMP = Dess-Martin periodinane, DMSO = dimethylsulfoxide, DDQ = 2,3-dichloro-5,6-dicyano-*para*-benzoquinone, DMAP = 4-dimethylaminopyridine.

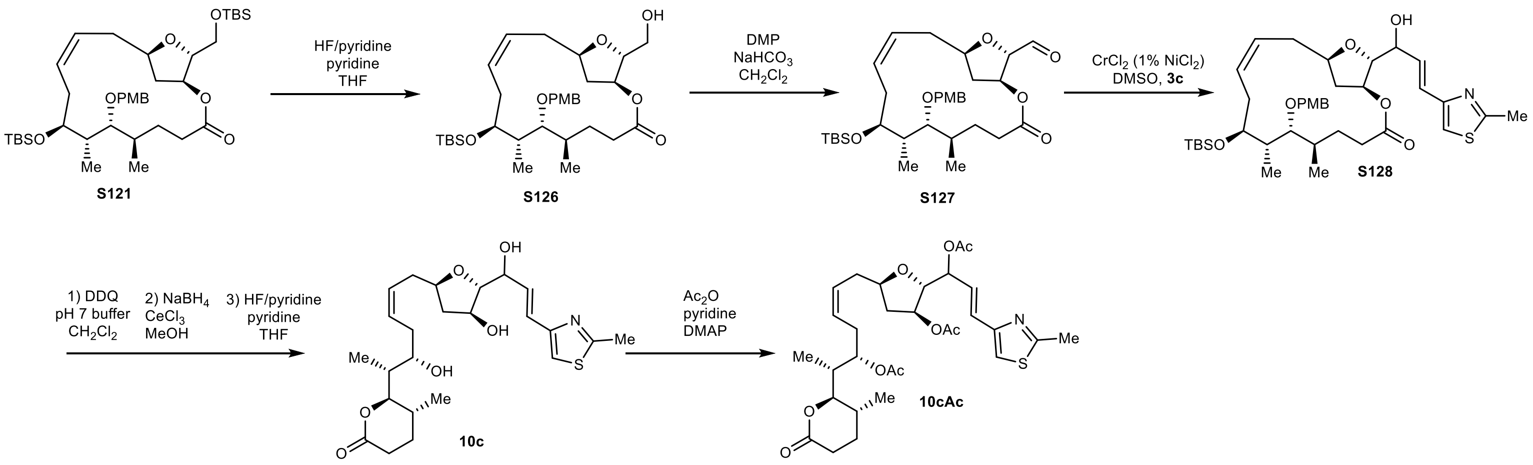


**Supplementary Fig. 46 (cont) | Synthesis of pMLs 10a-d and 10aAc-dAc.**
Abbreviations: TBS = tert-butyldimethylsilyl, PMB = *para*-methoxybenzyl, THF = tetrahydrofuran, DMP = Dess-Martin periodinane, DMSO = dimethylsulfoxide, DDQ = 2,3-dichloro-5,6-dicyano-*para*-benzoquinone, DMAP = 4-dimethylaminopyridine.

Note: Reaction run under ambient atmosphere
To a stirred solution of **2a** (248 mg, 0.59 mmol, 1.0 eq.) in anhydrous $CH_2Cl_2$ (6 mL) was added $NaHCO_3$ (252 mg, 3 mmol, 5.1 eq.) followed by Dess-Martin Periodinane (305 mg, 0.72 mmol, 1.2 eq.). The mixture was stirred at rt for 90 minutes and quenched with a solution of 1:1:1 $H_2O$-saturated $NaHCO_3$-saturated $Na_2S_2O_3$ (6 mL) and the biphasic mixture was stirred vigorously for 1 h. The aqueous layer was extracted with $CH_2Cl_2$ (4x 2 mL) and the combined organic layers were dried ($Na_2SO_4$), filtered, and the solvent was removed in vacuo. The crude product was run through a plug of silica gel ($Et_2O$) and solvent was removed in vacuo to yield the crude aldehyde **S117** as a colorless oil (198 mg, 80%).

To a cold (0 °C) stirred solution of **S29** (212 mg, 0.47 mmol, 1.0 eq.) and LiCl (24 mg, 0.56 mmol, 1.2 eq.) in anhydrous $CH_3CN$ (1 mL) was added DBU (70 µL, 72 mg, 0.47 mmol, 1.0 eq.) followed by crude aldehyde **S117** (198 mg, 0.47 mmol, 1.0 eq.) in anhydrous $CH_3CN$ (1.7mL) and a few drops of $Et_2O$ to improve solubility. After 1 h stirring at rt, the reaction was quenched with a saturated solution of $NH_4Cl$ (5 mL) followed by $H_2O$ (10 mL) and the aqueous layer was extracted with $CH_2Cl_2$ (3x 20 mL) The combined organic layers were dried ($Na_2SO_4$), filtered, and the solvent was removed in vacuo. The crude product was purified via flash column chromatography (37:3 hexanes/$Et_2O$). The solvent was removed in vacuo to yield **S118** (211 mg, 63% over two steps) as a colorless oil.

To a stirred solution of **S118** (211 mg, 0.29 mmol, 1.0 eq) in anhydrous toluene (deoxygenated via sparging with nitrogen for 30 minutes) (4.6 mL) was added Stryker's reagent in anhydrous, deoxygenated toluene (0.44 M, 1.3 mL, 0.58 mmol, 2 eq.). The mixture was stirred for 40 h after which the reaction mixture was flushed through a plug of silica gel ($Et_2O$) and the solvent was removed in vacuo. The crude product was purified via flash column chromatography (19:1 hexanes/$Et_2O$). Appropriate fractions were pooled, and solvent was removed in vacuo to yield **S119** (154 mg, 73 %) as a yellow oil.

To a stirred, heated (60 °C) solution of **S119** (151 mg, 0.21 mmol, 1.0 eq.) in anhydrous toluene (deoxygenated via 5x freeze-pump-thaw cycles) (85 mL) was added Grubbs 2$^{nd}$ generation catalyst (36 mg, 0.042 mmol, 0.2 eq.) in anhydrous, deoxygenated toluene (1.6 mL). After 1 h stirring at 60 °C, the reaction was cooled (0 °C) and quenched with potassium 2-isocyanoacetate (41 mg) in MeOH (10 mL). The mixture was warmed to rt and stirred for 1 h, then run through a plug of silica gel ($Et_2O$) and the solvent was removed in vacuo. The crude product was purified via flash column chromatography (19:1 to 17:3 hexanes/$Et_2O$). Appropriate fractions were pooled, and solvent was removed in vacuo to yield (*E*)-isomer **S120** (66 mg, 45%) as a yellow oil and (*Z*)-isomer **S121** (41 mg, 28%) as a yellow oil.

Note: Reaction run under ambient atmosphere
To a plastic vessel containing a stirred solution of **S120** (66 mg, 0.096 mmol, 1.0 eq.) was added a stock solution of HF in THF-Pyridine (3.85 M HF, 3.72 mL, 14.3 mmol, 150 eq. HF, 4:1 pyridine:HF). After stirring for 5 h at rt, the reaction was quenched with saturated $NaHCO_3$ (20 mL) and the aqueous layer was extracted with $CH_2Cl_2$ (3x 25 mL) and the combined organic layers were dried ($Na_2SO_4$), filtered, and the solvent was removed in vacuo. The crude product was purified via flash column chromatography (3:2 hexanes/EtOAc). Appropriate fractions were pooled, and solvent was removed in vacuo to yield **S122** (47 mg, 86%) as a pink oil.

Note: Reaction performed in capped vessel under ambient atmosphere.
To a stirred solution of **S122** (43 mg, 0.074 mmol, 1.0 eq.) in anhydrous $CH_2Cl_2$ (1.5 mL) was added $NaHCO_3$ (19 mg, 0.22 mmol, 3.0 eq.) followed by Dess-Martin Periodinane (38 mg, 0.089 mmol, 1.2 eq.). After 150 minutes stirring at rt, the reaction was quenched with a solution of 1:1:1 $H_2O$:saturated aqueous $NaHCO_3$:saturated aqueous $Na_2S_2O_3$ (10 mL) and the biphasic mixture was stirred vigorously for 1 h. The aqueous layer was extracted with $CH_2Cl_2$ (3x 25 mL) and the combined organic layers were dried ($Na_2SO_4$), filtered, and the solvent was removed in vacuo. The crude product was run through a plug of silica gel ($Et_2O$) and solvent was removed in vacuo to yield the crude aldehyde **S123** (43 mg) which was divided into 2 equal portions for the following step.

To a solution of **S123** (21 mg, 0.037 mmol, 1.0 eq.) in anhydrous DMSO (deoxygenated via 5x freeze-pump-thaw cycles) (0.5 mL) was added $CrCl_2$ with doped with 1 % $NiCl_2$ (w/w) (46 mg, 0.37 mmol, 10 eq.). **3b** (19 mg, 0.074 mmol, 2 eq.) was added in deoxygenated DMSO (0.75 mL) and the mixture was stirred for 18 h. The reaction was cooled to 0 °C and quenched with 1:1 $H_2O$-brine (10 mL). The aqueous layer was extracted with $Et_2O$ (11x 10 mL) and the combined organic layers were dried ($MgSO_4$), filtered, and the solvent was removed in vacuo. The crude product was purified via flash column chromatography (17:3 hexanes-acetone). Appropriate fractions were pooled, and solvent was removed in vacuo to yield **S124** (8.9 mg, 34% over two steps) as a colorless oil and a mixture of diastereomers (7:3).

Note: Reaction performed in capped vessel under ambient atmosphere.
To a vigorously stirred solution of **S124** (8.9 mg, 0.013 mmol, 1.0 eq.) in $CH_2Cl_2$ (1.3 mL) and aqueous phosphate buffer (pH 7, 0.3 mL) was added 2,3-dichloro-5,6-dicyano-benzoquinone (12 mg, 0.051 mmol, 4.0 eq.). After 1 h the starting material was consumed as monitored by TLC analysis and the reaction was quenched with saturated $NaHCO_3$ (2 mL). The aqueous layer was extracted with $CH_2Cl_2$ (3x 10 mL) and the combined organic layers were dried ($Na_2SO_4$), filtered, and the solvent was removed in vacuo. The crude product was purified via flash column chromatography (9:1 to 17:3 hexanes-acetone). Appropriate fractions were pooled, and solvent was removed in vacuo to yield the diol (4.4 mg, 60%) as a colorless oil and a mixture of diastereomers (7:3).

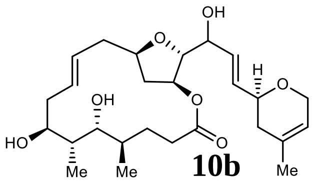

**10b**

Note: Reaction performed in capped vessel under ambient atmosphere.
To a plastic vessel containing the diol from the previous step (4.4 mg, 0.0063 mmol, 1.0 eq.) was added a 2.0 M of solution of HF in pyridine/THF (0.63 mL, 1.26 mmol HF, 200 eq., buffered 4:1 v/v pyridine:HF) and 70% HF/pyridine (50 µL, 55 mg, 1.92 mmol HF, 306 eq.). After 4 days stirring the reaction was quenched with saturated $NaHCO_3$ (5 mL) and after effervescence had subsided, the aqueous layer was extracted with $CH_2Cl_2$ (4x 10 mL). The combined organic layers were dried ($Na_2SO_4$), filtered, and the solvent was removed in vacuo. The crude product was purified via flash column chromatography (7:3 to 3:2 hexanes/acetone). Appropriate fractions were pooled to yield **10b** (1.8 mg, 49%) as a colorless oil and a mixture of diastereomers (7:3).

**Analytical Data for 10b:**

$R_f$ = 0.25 (3:2 hexanes/acetone)

For the following reported NMR peaks: Several resonances in the $^{1}$H NMR spectrum integrated for 0.7 and 0.3 protons and are unique to both the diastereomers. In these cases an integral value of "0.7 H" and "0.3 H" is assigned.

$^{1}$H NMR (600 MHz, $CDCl_3$) δ 5.93 – 5.86 (m, 1H), 5.79 – 5.72 (m, 1H), 5.58 – 5.44 (m, 2H), 5.41 (s, 1H), 5.21 (dt, *J* = 8.6, 4.5 Hz, 0.7H), 5.09 (dt, *J* = 7.8, 3.9 Hz, 0.3H), 4.42 – 4.34 (m, 1H), 4.31 (t, *J* = 5.0 Hz, 1H), 4.20 – 4.15 (m, 2H), 4.04 (ddd, *J* = 14.9, 10.5, 5.5 Hz, 1H), 3.99 (t, *J* = 4.3 Hz, 0.7H), 3.92 (dd, *J* = 6.7, 3.3 Hz, 0.3H), 3.75 – 3.69 (m, 1H), 3.56 (q, *J* = 3.4 Hz, 1H), 2.63 – 2.54 (m, 1H), 2.45 (ddd, *J* = 15.2, 6.9, 3.4 Hz, 1H), 2.41 – 2.35 (m, 1H), 2.35 – 2.21 (m, 4H), 2.14 (ddd, *J* = 14.3, 7.0, 3.9 Hz, 1H), 2.05 – 1.96 (m, 2H), 1.93 (ddt, *J* = 14.3, 11.0, 2.9 Hz, 3H), 1.87 – 1.82 (m, 2H), 1.70 (s, 3H), 1.61 (m, 1H), 1.52 (m, 1H), 1.04 (s, 3H), 1.02 (s, 3H).

Major diastereomer
$^{13}$C NMR (151 MHz, $CDCl_3$) δ 173.36, 133.07, 131.49, 131.37, 128.20, 127.71, 119.80, 85.64, 78.42, 75.93, 75.31, 73.10, 71.91, 65.77, 40.00, 39.72, 37.21, 36.61, 35.80, 35.29, 32.10, 24.57, 23.10, 17.58, 7.67.

Minor diastereomer
$^{13}$C NMR (151 MHz, $CDCl_3$) δ 173.39, 133.07, 131.53, 131.05, 128.94, 128.60, 127.52, 85.99, 78.27, 77.73, 76.47, 75.90, 73.13, 72.01, 40.16, 39.67, 36.78, 36.25, 35.70, 35.32, 32.12, 24.62, 23.13, 17.60, 7.87.

HRMS (ESI): Anal. Calcd. for $C_{26}H_{44}NO_7^+$ $[M+NH_4]^+$ 482.3112, found 482.3115
IR (neat): $\nu_{max}$ $(cm^{-1})$ = 3401 (br, OH), 2923 (s, CH), 2852 (m, CH), 1725 (s, C=O), 1709 (s, C=O), 1438 (m), 1381 (m), 1256 (s)

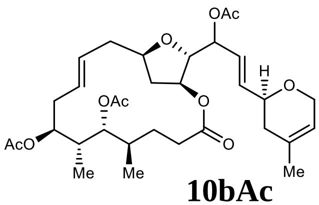


**10bAc**

**10bAc** was prepared according to general procedure A (0.95 mg, 85%).

**Analytical Data for 10bAc:**

$R_f$ = 0.69 (1:1 hexanes/EtOAc)

HRMS (ESI): Anal. Calcd. for $C_{32}H_{50}NO_{10}^+$ $[M+NH_4]^+$ 608.3429, found 608.3424

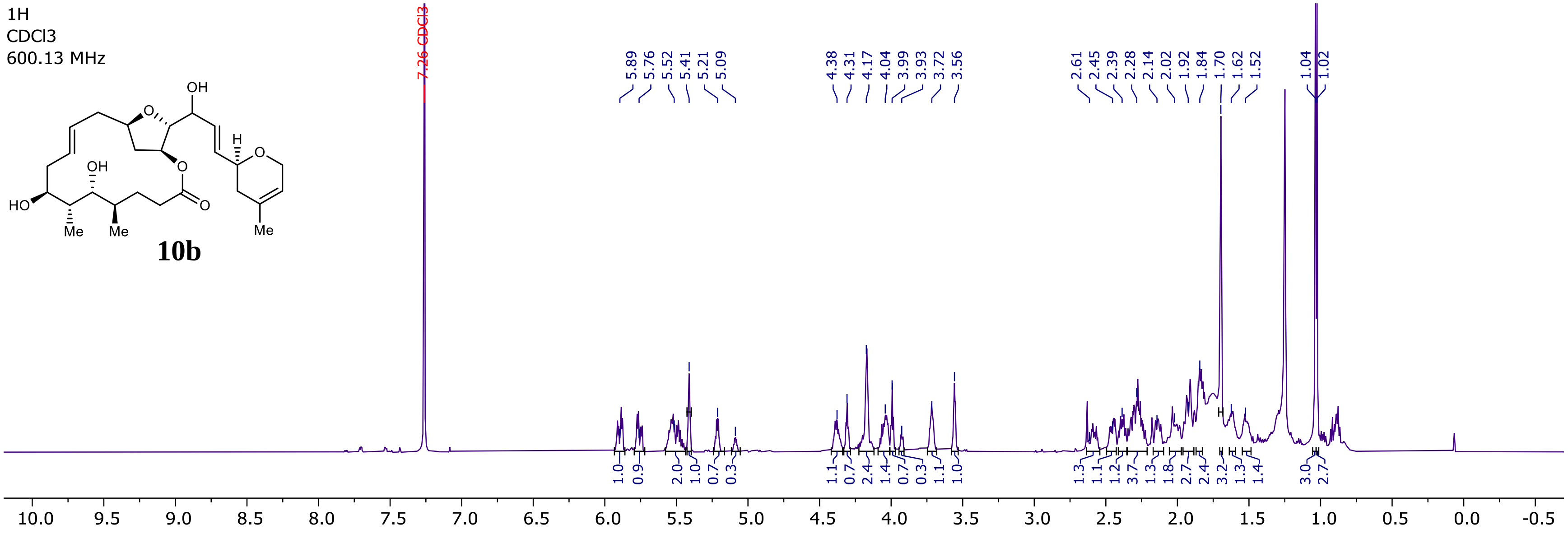
1H
CDCl3
600.13 MHz
7.26 CDCl3
5.89
5.76
5.52
5.41
5.21
5.09
4.38
4.31
4.17
4.04
3.99
3.93
3.72
3.56
2.61
2.45
2.39
2.28
2.14
2.02
1.92
1.84
1.70
1.62
1.52
1.04
1.02
1.0
0.9
2.0
1.0
0.7
0.3
1.1
0.7
2.4
1.4
0.7
0.3
1.1
1.0
1.3
1.1
1.2
3.7
1.3
1.8
2.7
2.4
3.2
1.3
1.4
3.0
2.7
10.0 9.5 9.0 8.5 8.0 7.5 7.0 6.5 6.0 5.5 5.0 4.5 4.0 3.5 3.0 2.5 2.0 1.5 1.0 0.5 0.0 -0.5

**10b**

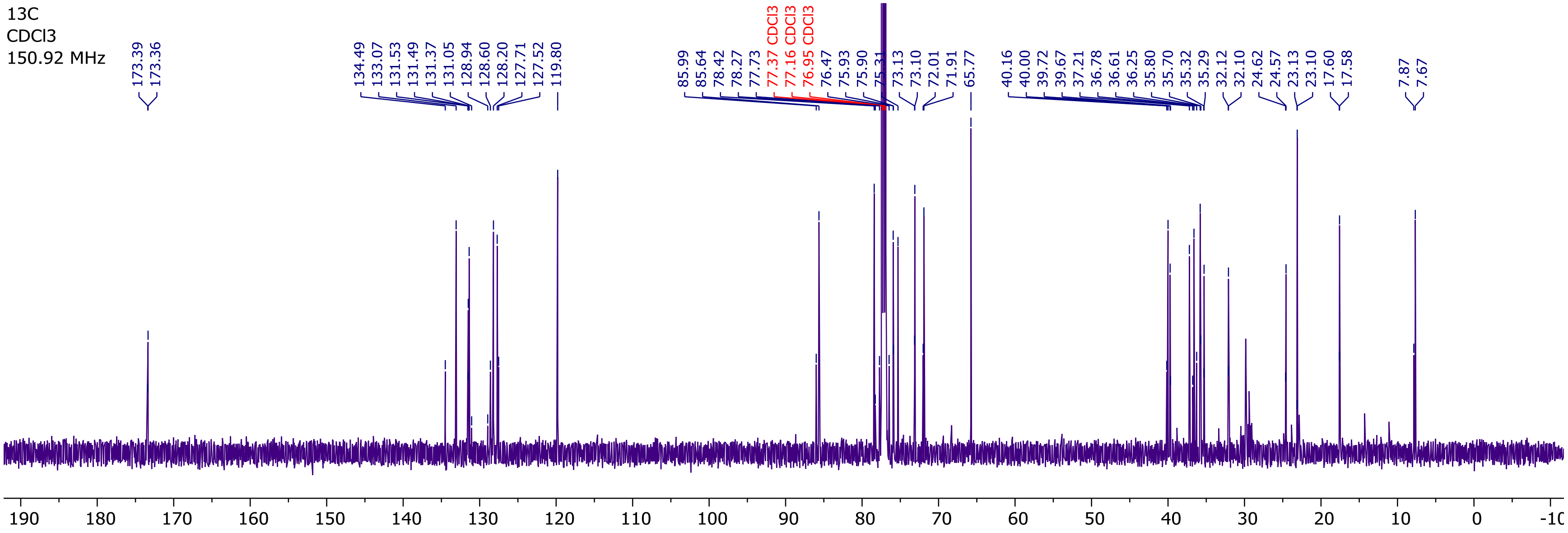
13C
CDCl3
150.92 MHz
173.39
173.36
134.49
133.07
131.53
131.49
131.37
131.05
128.94
128.60
128.20
127.71
127.52
119.80
85.99
85.64
78.42
78.27
77.73
77.37 CDCl3
77.16 CDCl3
76.95 CDCl3
76.47
75.93
75.90
75.31
73.13
73.10
72.01
71.91
65.77
40.16
40.00
39.72
39.67
37.21
36.78
36.61
36.25
35.80
35.70
35.32
35.29
32.12
32.10
24.62
24.57
23.13
23.10
17.60
17.58
7.87
7.67
190 180 170 160 150 140 130 120 110 100 90 80 70 60 50 40 30 20 10 0

To a solution of **S123** (21 mg, 0.037 mmol, 1.0 eq.) in anhydrous DMSO (deoxygenated via 5x freeze-pump-thaw cycles) (0.5 mL) was added $CrCl_2$ with doped with 1 % $NiCl_2$ (w/w) (46 mg, 0.37 mmol, 10 eq.). **3a** (27 mg, 0.11 mmol, 3 eq.) was added in deoxygenated DMSO (0.75 mL) and the mixture was stirred for 18 h. The reaction was cooled to 0 °C and quenched with 1:1 $H_2O$-brine (10 mL). The aqueous layer was extracted with $Et_2O$ (10x 10 mL then 5x 30 mL) and the combined organic layers were dried ($MgSO_4$), filtered, and the solvent was removed in vacuo. The crude product was purified via flash column chromatography (7:3 hexanes/acetone). Appropriate fractions were pooled, and solvent was removed in vacuo to yield **S125** (7.3 mg, 29% over two steps) as a colorless oil and a mixture of diastereomers (2:1).

Note: Reaction performed in capped vessel under ambient atmosphere
To a vigorously stirred solution of **S125** (7.3 mg, 0.011 mmol, 1.0 eq.) in $CH_2Cl_2$ (1.1 mL) and aqueous phosphate buffer (pH 7, 0.5 mL) was added 2,3-dichloro-5,6-dicyano-benzoquinone (9.6 mg, 0.042 mmol, 4.0 eq.). After 1 h the starting material was consumed as monitored by TLC analysis and the reaction was quenched with saturated $NaHCO_3$ (2 mL). The aqueous layer was extracted with $CH_2Cl_2$ (3x 10 mL) and the combined organic layers were dried ($Na_2SO_4$), filtered, and the solvent was removed in vacuo. The crude product was purified via flash column chromatography (13:7 to 11:9 hexanes/acetone). Appropriate fractions were pooled, and solvent was removed in vacuo to yield the diol (5.8 mg, 97%) as a colorless oil and a mixture of diastereomers (2:1).

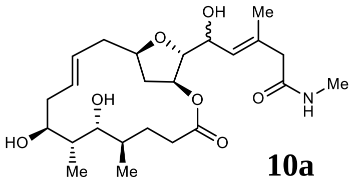


**10a**

Note: Reaction performed in capped vessel under ambient atmosphere.
To a plastic vessel containing the diol from the previous step (5.8 mg, 0.010 mmol, 1.0 eq.) was added a 2.0 M of solution of HF in pyridine/THF (1.02 mL, 2.04 mmol HF, 200 eq., buffered 4:1 v/v pyridine:HF) and 70% HF/pyridine (50 µL, 55 mg, 1.92 mmol HF, 188 eq.). After 3 days stirring at rt, the reaction was quenched with saturated $NaHCO_3$ (6 mL) and after effervescence had subsided, the aqueous layer was extracted with $CH_2Cl_2$ (4 x 10 mL). The combined organic layers were dried ($Na_2SO_4$), filtered, and the solvent was removed in vacuo. The crude product was purified via flash column chromatography (1:3 to 3:17 hexanes/acetone). Appropriate fractions were pooled to yield **10a** (4.0 mg, 87%) as a colorless oil and a mixture of diastereomers (2:1).

**Analytical Data for 10a:**

$R_f$ = 0.50 (1:4 hexanes/acetone)

For the following reported NMR peaks: Several resonances in the $^{1}$H NMR spectrum integrated for 0.7 and 0.3 protons and are unique to both the diastereomers. In these cases an integral value of "0.7 H" and "0.3 H" is assigned.

$^{1}$H NMR (601 MHz, $CDCl_3$) δ 6.18 (s, 0.3H), 6.13 (s, 0.7H), 5.63 – 5.59 (m, 0.6H), 5.56 – 5.51 (m, 1.4H), 5.39 – 5.31 (m, 1H), 5.28 (dt, *J* = 8.0, 3.5 Hz, 0.7H), 5.01 (dt, *J* = 7.5, 2.4 Hz, 0.3H), 4.47 (dd, *J* = 8.7, 5.6 Hz, 0.7H), 4.40 – 4.34 (m, 1H), 4.25 (t, *J* = 8.7 Hz, 0.3H), 3.92 (dd, *J* = 5.7, 3.0 Hz, 0.7H), 3.83 (dd, *J* = 8.4, 1.8 Hz, 0.3H), 3.80 – 3.76 (m, 0.3H), 3.73 (ddd, *J* = 8.2, 5.3, 2.9 Hz, 0.7H), 3.56 (dd, *J* = 5.4, 3.6 Hz, 1H), 3.07 – 2.90 (m, 2H), 2.79 (d, *J* = 4.8 Hz, 2.1H), 2.77 (d, *J* = 4.7 Hz, 0.9H), 2.70 – 2.64 (m, 0.6H), 2.58 – 2.52 (m, 1.4H), 2.49 – 2.39 (m, 2H), 2.39 – 2.31 (m, 2H), 2.31 – 2.25 (m, 2H), 2.21 – 2.15 (m, 1H), 1.97 (ddd, *J* = 14.3, 7.3, 2.9 Hz, 0.7H), 1.94 – 1.87 (m, *J* = 14.3, 8.6, 3.8 Hz, 2.3H), 1.85 – 1.80 (m, 1H), 1.77 (d, *J* = 1.4 Hz, 0.9H), 1.74 (d, *J* = 1.4 Hz, 2.1H), 1.71 (ddd, *J* = 9.3, 6.5, 3.3 Hz, 1H), 1.05 – 0.99 (m, 6H).

Major diastereomer
$^{13}$C NMR (151 MHz, $CDCl_3$) δ 173.73, 170.99, 135.44, 131.53, 129.07, 127.32, 86.42, 78.53, 77.98, 76.46, 75.52, 69.06, 47.81, 39.97, 39.57, 36.94, 36.06, 35.20, 32.15, 26.62, 24.72, 17.43, 17.38, 8.03.

Minor diastereomer
$^{13}$C NMR (151 MHz, $CDCl_3$) δ 170.89, 136.59, 131.47, 128.16, 127.18, 87.38, 77.68, 76.20, 75.30, 66.62, 48.32, 39.91, 39.21, 36.15, 35.55, 34.27, 32.49, 26.64, 24.92, 17.31, 17.16, 8.44.

HRMS (ESI): Anal. Calcd. for $C_{24}H_{40}NO_7^+$ $[M+H]^+$ 454.2799, found 454.2798
IR (neat): $\nu_{max}\ (cm^{-1})$ = 3365 (br, OH), 2918 (s, CH), 1726 (s, C=O), 1657 (s, C=C), 1550 (m), 1413 (w), 1261 (s)

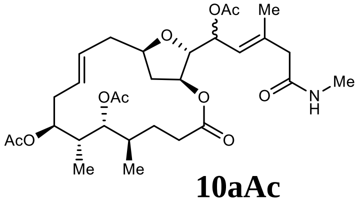

**10aAc**

**10aAc** was prepared according to general procedure A (0.95 mg, 88%).

**Analytical Data for 10aAc:**

$R_f$ = 0.37 (EtOAc)

HRMS (ESI): Anal. Calcd. for $C_{30}H_{46}NO_{10}^+$ $[M+H]^+$ 580.3116, found 580.3121

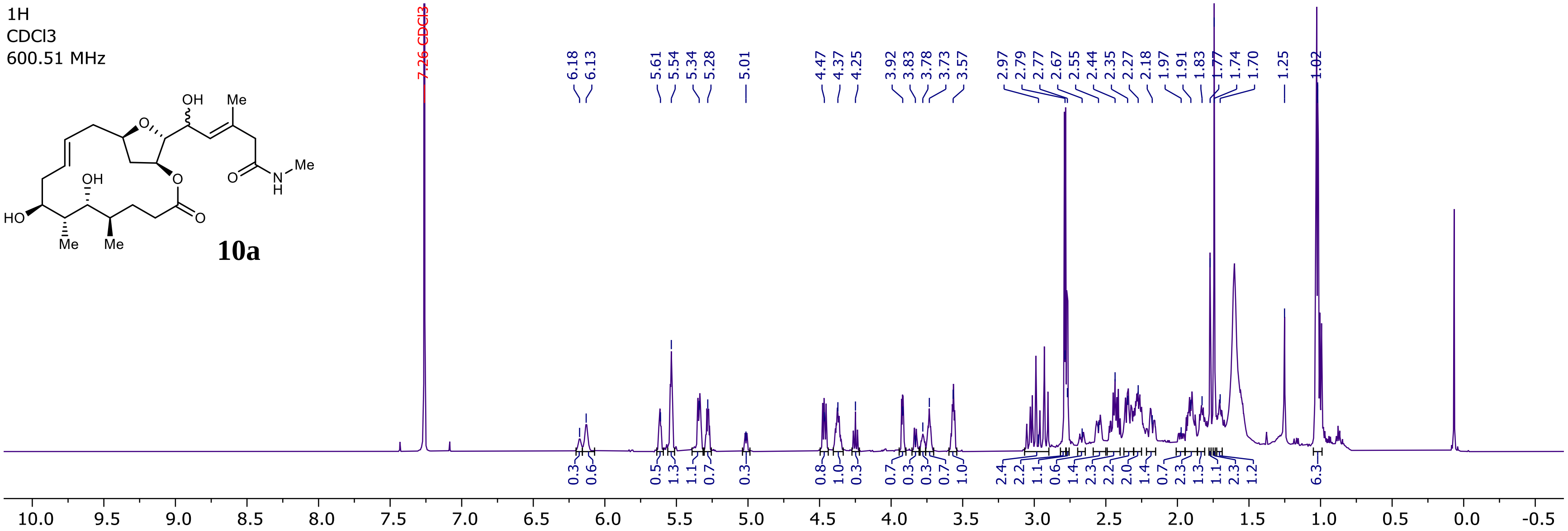
1H
CDCl3
600.51 MHz
OH
Me
O
OH
O
N
Me
H
HO
O
Me
Me
7.26 CDCl3
6.18 6.13 5.61 5.54 5.34 5.28 5.01 4.47 4.37 4.25 3.92 3.83 3.78 3.73 3.57 2.97 2.79 2.77 2.67 2.55 2.44 2.35 2.27 2.18 1.97 1.91 1.83 1.77 1.74 1.70 1.25 1.02
0.3 0.6 0.5 1.3 1.1 0.7 0.3 0.8 1.0 0.3 0.7 0.3 0.3 0.7 1.0 2.4 2.2 1.1 0.6 1.4 2.3 2.2 2.0 1.4 0.7 2.3 1.3 1.1 2.3 1.2 6.3
10.0 9.5 9.0 8.5 8.0 7.5 7.0 6.5 6.0 5.5 5.0 4.5 4.0 3.5 3.0 2.5 2.0 1.5 1.0 0.5 0.0 -0.5

**10a**

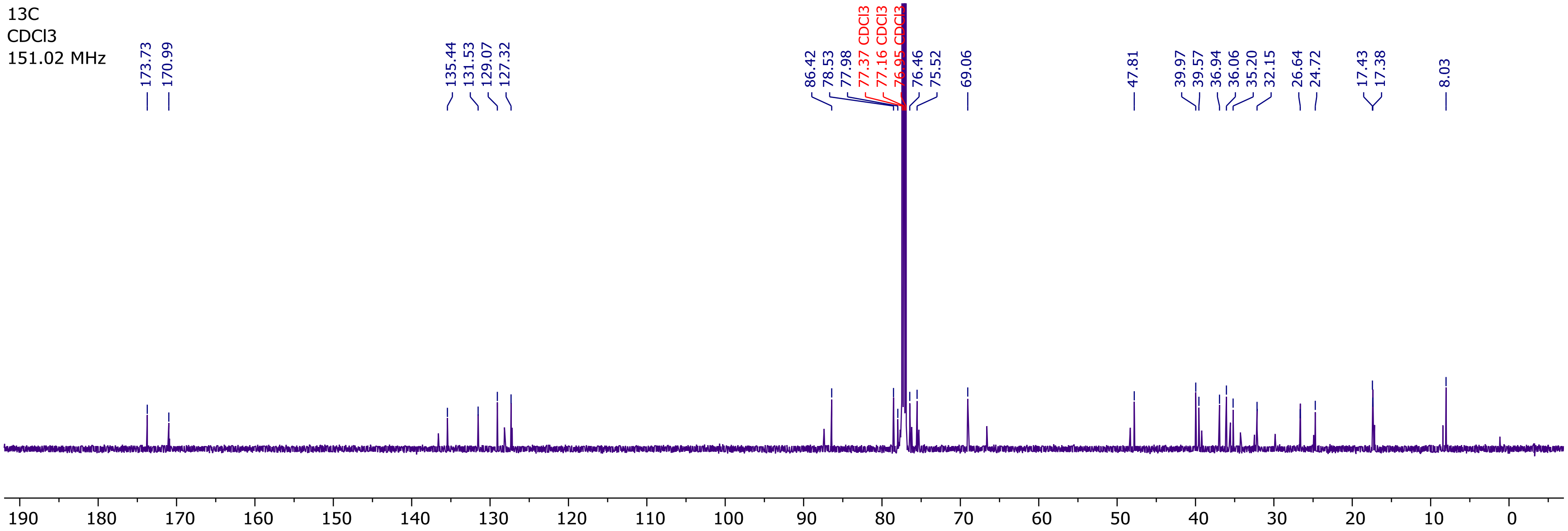
13C
CDCl3
151.02 MHz
173.73 170.99 135.44 131.53 129.07 127.32 86.42 78.53 77.98
77.37 CDCl3
77.16 CDCl3
76.95 CDCl3
76.46 75.52 69.06 47.81 39.97 39.57 36.94 36.06 35.20 32.15 26.64 24.72 17.43 17.38 8.03
190 180 170 160 150 140 130 120 110 100 90 80 70 60 50 40 30 20 10 0

Note: Reaction performed in capped vessel under ambient atmosphere
To a vigorously stirred solution of **S122** (4.6 mg, 0.0080 mmol, 1.0 eq.) in $CH_2Cl_2$ (0.8 mL) and aqueous phosphate buffer (pH 7, 0.4 mL) was added 2,3-dichloro-5,6-dicyano-benzoquinone (7.2 mg, 0.032 mmol, 4.0 eq.). After 1 h, more 2,3-dichloro-5,6-dicyano-benzoquinone was added (3 mg, 0.013 mmol, 1.6 eq.) and the reaction was stirred further for 30 minutes, then quenched with saturated $NaHCO_3$ (2 mL). The aqueous layer was extracted with $CH_2Cl_2$ (3x 10 mL) and the combined organic layers were dried ($Na_2SO_4$), filtered, and the solvent was removed in vacuo. The crude product was purified via flash column chromatography (4:1 to 3:1 hexanes/acetone). Appropriate fractions were pooled, and solvent was removed in vacuo to yield the diol (3.5 mg, 96%) as a colorless oil.

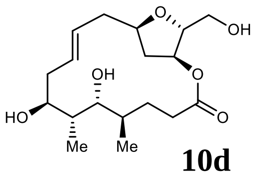

**10d**

Note: Reaction performed in capped vessel under ambient atmosphere.
To a plastic vessel containing the diol from the previous step (3.5 mg, 0.0077 mmol, 1.0 eq.) was added a 2.0 M of solution of HF in pyridine/THF (0.77 mL, 1.53 mmol HF, 200 eq., buffered 4:1 v/v pyridine:HF) and 70% HF/pyridine (31 µL, 34 mg, 1.21 mmol HF, 200 eq.). After 5 days stirring at rt, the reaction was quenched with saturated $NaHCO_3$ (5 mL) and after effervescence had subsided, the aqueous layer was extracted with $CH_2Cl_2$ (3x 10 mL). The combined organic layers were dried ($Na_2SO_4$), filtered, and the solvent was removed in vacuo. The crude product was purified via flash column chromatography (3:2 hexanes/acetone). Appropriate fractions were pooled to yield **10d** (1.7 mg, 65%) as a white solid.

**Analytical Data for 10d:**

$R_f$ = 0.12 (3:2 hexanes/acetone)

$[\alpha]_D^{20}$ = -18 ° (c = 0.16, $CH_2Cl_2$)

$^1$H NMR (601 MHz, $CDCl_3$) δ 5.62 – 5.47 (m, 2H), 5.06 (ddd, *J* = 8.3, 4.6, 3.7 Hz, 1H), 4.36 (tdd, *J* = 7.6, 5.5, 2.8 Hz, 1H), 4.09 (dt, *J* = 6.3, 3.8 Hz, 1H), 3.73 (ddd, *J* = 8.2, 5.5, 2.8 Hz, 1H), 3.69 (dd, *J* = 11.6, 3.9 Hz, 1H), 3.64 – 3.55 (m, 2H), 2.61 (dt, *J* = 14.3, 5.4 Hz, 1H), 2.47 (ddd, *J* = 15.3, 6.8, 3.7 Hz, 1H), 2.40 (dt, *J* = 13.8, 7.7 Hz, 1H), 2.36 – 2.29 (m, 2H), 2.29 – 2.22 (m, 2H), 2.14 (ddd, *J* = 14.4, 8.2, 2.8 Hz, 1H), 1.95 (ddt, *J* = 14.4, 11.1, 3.4 Hz, 1H), 1.89 – 1.83 (m, 2H), 1.64 (dtd, *J* = 9.9, 6.6, 3.0 Hz, 1H), 1.53 (ddq, *J* = 12.7, 6.2, 3.4 Hz, 1H), 1.06 – 1.01 (m, 6H).

$^{13}C$ NMR (151 MHz, $CDCl_3$) δ 173.49, 131.55, 127.56, 83.67, 78.48, 76.17, 76.03, 62.24, 40.06, 39.59, 36.83, 36.58, 35.20, 32.23, 29.85, 24.70, 17.54, 7.75.

HRMS (ESI): Anal. Calcd. for $C_{18}H_{31}O_6^+$ $[M+H]^+$ 343.2115, found 343.2123
IR (neat): $\nu_{max}$ $(cm^{-1})$ = 3398 (br, OH), 2957 (m, CH), 2918 (s, CH), 2850 (s, CH), 1725 (s, C=O), 1709 (s, C=O), 1463 (m), 1378 (m), 1261 (s)

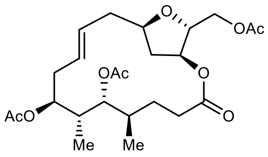

**10dAc**

**10dAc** was prepared according to general procedure A (1.34 mg, 69%).

**Analytical Data for 10dAc:**

$R_f$ = 0.63 (1:1 hexanes/EtOAc)

HRMS (ESI): Anal. Calcd. for $C_{24}H_{40}NO_9^+$ $[M+NH_4]^+$ 486.2698, found 486.2681

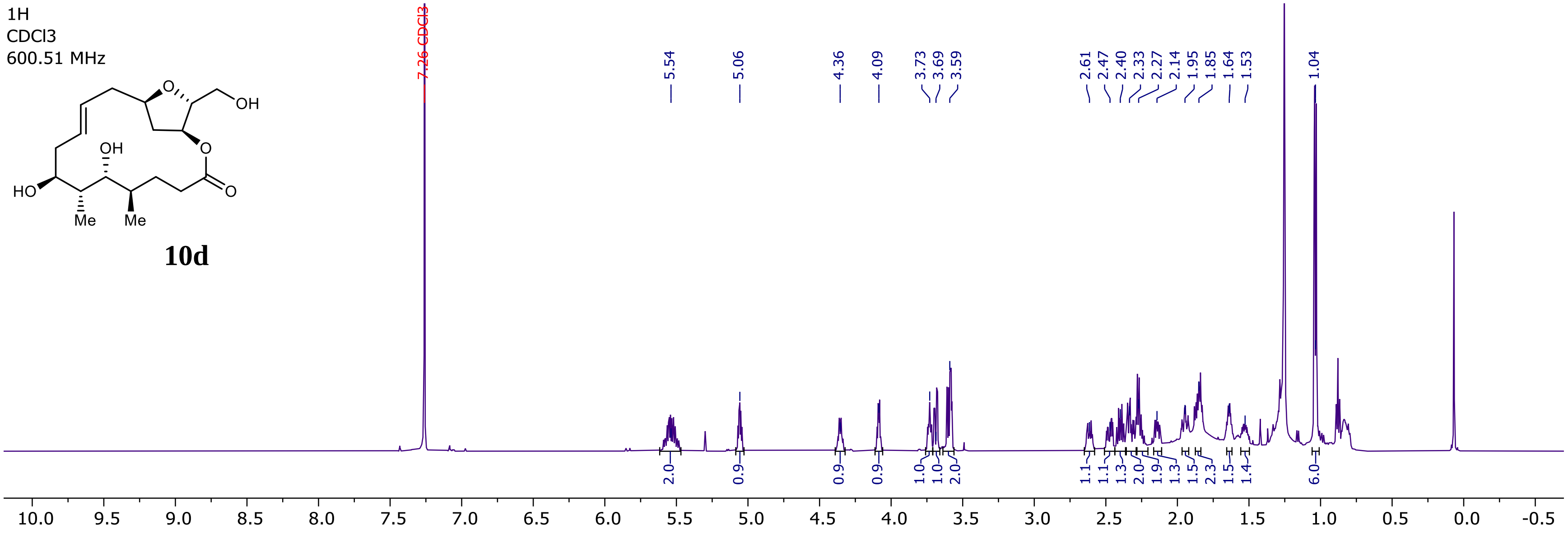
1H
CDCl3
600.51 MHz
OH
O
O
O
OH
HO
Me
Me
10d
7.26 CDCl3
5.54
5.06
4.36
4.09
3.73
3.69
3.59
2.61
2.47
2.40
2.33
2.27
2.14
1.95
1.85
1.64
1.53
1.04
2.0
0.9
0.9
0.9
1.0
1.0
2.0
1.1
1.1
1.3
2.0
1.9
1.3
1.5
2.3
1.5
1.4
6.0
10.0
9.5
9.0
8.5
8.0
7.5
7.0
6.5
6.0
5.5
5.0
4.5
4.0
3.5
3.0
2.5
2.0
1.5
1.0
0.5
0.0
-0.5

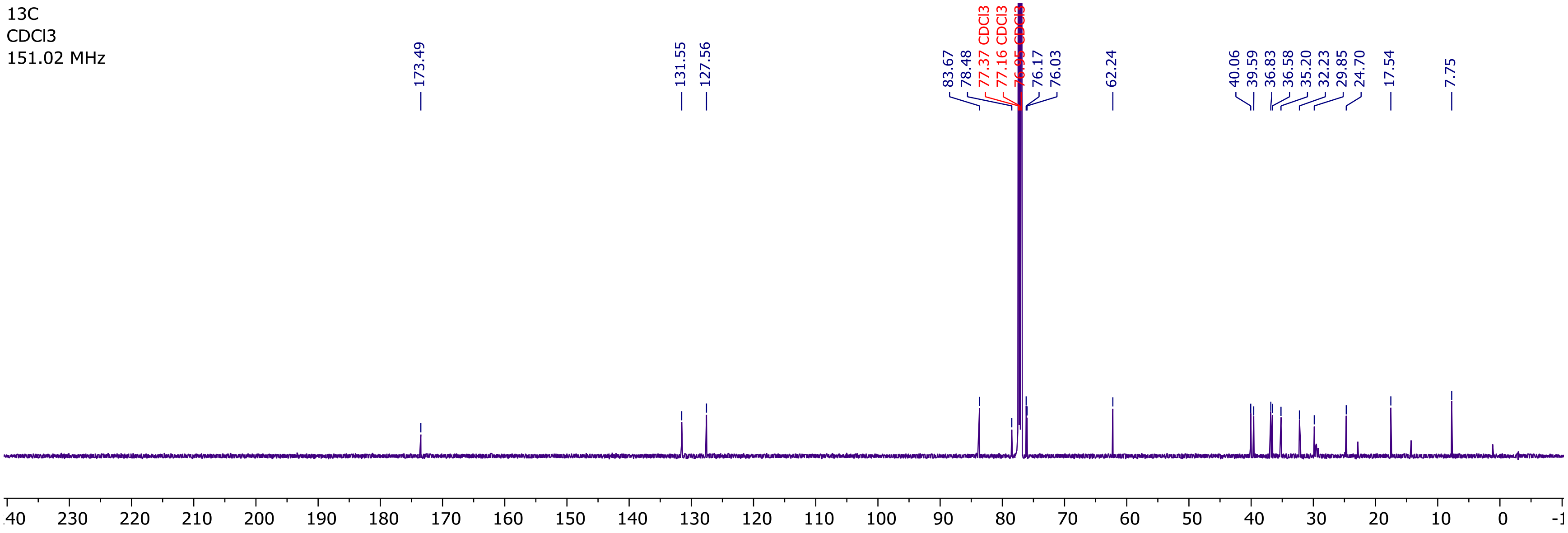
13C
CDCl3
151.02 MHz
173.49
131.55
127.56
83.67
78.48
77.37 CDCl3
77.16 CDCl3
76.95 CDCl3
76.17
76.03
62.24
40.06
39.59
36.83
36.58
35.20
32.23
29.85
24.70
17.54
7.75
230
220
210
200
190
180
170
160
150
140
130
120
110
100
90
80
70
60
50
40
30
20
10
0

Note: Reaction performed in capped vessel under ambient atmosphere.
To a plastic vessel containing a stirred solution of **S121** (41 mg, 0.059 mmol, 1.0 eq.) was added a stock solution of HF in THF-Pyridine (3.85 M HF, 2.29 mL, 8.8 mmol, 150 eq. HF, 4:1 pyridine:HF). After stirring for 5 h at rt, the reaction was quenched with saturated $NaHCO_3$ (20 mL) and the aqueous layer was extracted with $CH_2Cl_2$ (3x 25 mL) and the combined organic layers were dried ($Na_2SO_4$), filtered, and the solvent was removed in vacuo. The crude product was purified via flash column chromatography (7:3 hexanes/EtOAc). Appropriate fractions were pooled, and solvent was removed in vacuo to yield **S126** (31 mg, 90%) as a colorless oil.

Note: Reaction performed in capped vessel under ambient atmosphere.
To a stirred solution of **S126** (26 mg, 0.046 mmol, 1.0 eq.) in anhydrous $CH_2Cl_2$ (0.92 mL) was added $NaHCO_3$ (12 mg, 0.14 mmol, 3.0 eq.) followed by Dess-Martin Periodinane (23 mg, 0.055 mmol, 1.2 eq.). After 150 minutes stirring at rt, the reaction was quenched with a solution of 1:1:1 $H_2O$:saturated aqueous $NaHCO_3$:saturated aqueous $Na_2S_2O_3$ (10 mL) and the biphasic mixture was stirred vigorously for 1 h. The aqueous layer was extracted with $CH_2Cl_2$ (3x 25 mL) and the combined organic layers were dried ($Na_2SO_4$), filtered, and the solvent was removed in vacuo. The crude product was run through a plug of silica gel ($Et_2O$) and solvent was removed in vacuo to yield the crude aldehyde **S127** (26 mg) which was used immediately in the following step.

To a solution of **S127** (26 mg, 0.046 mmol, 1.0 eq.) in anhydrous DMSO (deoxygenated via 5x freeze-pump-thaw cycles) (0.5 mL) was added $CrCl_2$ with doped with 1 % $NiCl_2$ (w/w) (56 mg, 0.46 mmol, 10 eq.). **3c** (34 mg, 0.136 mmol, 3 eq.) was added in deoxygenated DMSO (0.75 mL) and the mixture was stirred for 18 h. The reaction was cooled to 0 °C and quenched with 1:1 $H_2O$-brine (10 mL). The aqueous layer was extracted with $Et_2O$ (11x 10 mL) and the combined organic layers were dried ($MgSO_4$), filtered, and the solvent was removed in vacuo. The crude product was purified via flash column chromatography (9:1 hexanes-acetone). Appropriate fractions were pooled, and solvent was removed in vacuo to yield **S128** (20.2 mg, 63% over two steps) as a colorless oil.

Note: Reaction performed in capped vessel under ambient atmosphere.
To a vigorously stirred solution of **S128** (20 mg, 0.029 mmol, 1.0 eq.) in $CH_2Cl_2$ (2.8 mL) and aqueous phosphate buffer (pH 7, 0.7 mL) was added 2,3-dichloro-5,6-dicyano-benzoquinone (26 mg, 0.12 mmol, 4.0 eq.). After 30 minutes the starting material was consumed as monitored by TLC analysis and the reaction was quenched with saturated $NaHCO_3$ (2 mL). The aqueous layer was extracted with $CH_2Cl_2$ (3x 10 mL) and the combined organic layers were dried ($Na_2SO_4$), filtered, and the solvent was removed in vacuo. The crude product was purified via flash column chromatography (9:1 to 17:3 hexanes-acetone). Appropriate fractions were pooled, and solvent was removed in vacuo to yield the ketone (9.5 mg, 57%) as a colorless oil.

To a stirred solution of the ketone prepared in the previous step (9.5 mg, 0.016 mmol, 1 eq.) in methanol (0.16 mL) was added $CeCl_3$•$H_2O$ (9.2 mg, 0.025 mmol, 1.5 eq.) and sodium borohydride (0.9 mg, 0.025 mmol, 1.5 eq.). After 1 h the reaction was quenched with a saturated solution of aqueous ammonium chloride. The aqueous layer was extracted with $CH_2Cl_2$ (3x 10 mL) and the combined organic layers were dried ($Na_2SO_4$), filtered, and the solvent was removed in vacuo. The crude product was purified via flash column chromatography (3:1 hexanes-acetone). Appropriate fractions were pooled, and solvent was removed in vacuo to yield the diol (8.2 mg, 86%) as a colorless oil. A portion of the material was taken to the next step.

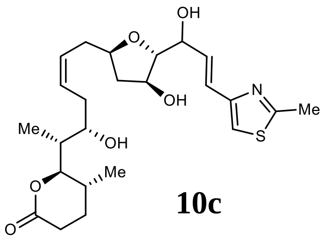


**10c**

Note: Reaction performed in capped vessel under ambient atmosphere**.**

To a plastic vessel containing the diol from the previous step (6.6 mg, 0.011 mmol, 1.0 eq.) was added a 2.0 M of solution of HF in pyridine/THF (1.14 mL, 2.28 mmol HF, 200 eq., buffered 4:1 v/v pyridine:HF) and 70% HF/pyridine (50 µL, 55 mg, 1.92 mmol HF, 168 eq.). After 4 days stirring at rt, the reaction was quenched with saturated $NaHCO_3$ (5 mL) and after effervescence had subsided, the aqueous layer was extracted with $CH_2Cl_2$ (4 x 10 mL). The combined organic layers were dried ($Na_2SO_4$), filtered, and the solvent was removed in vacuo. The crude product was purified via flash column chromatography (1:1 hexanes:acetone). Appropriate fractions were pooled to yield **10c** (5.0 mg, 94%) as a clear oil and a mixture of diastereomers (2:3).

**Analytical Data for 10c:**

$R_f$ = 0.18 (1:1 hexanes:acetone)

For the following reported NMR peaks: Several resonances in the $^1$H NMR spectrum integrated for 0.6 and 0.4 protons and are unique to both the diastereomers. In these cases an integral value of "0.6 H" and "0.4 H" is assigned.

$^1$H NMR (600 MHz, $CDCl_3$) δ 6.97 – 6.85 (m, 1H), 6.70 (s, 0.4H), 6.67 (s, 0.6H), 6.61 – 6.50 (m, 1H), 5.67 – 5.51 (m, 2H), 4.48 – 4.39 (m, 0.6H), 4.37 – 4.29 (m, 0.6H), 4.24 – 4.10 (m, 2.8H), 3.92 – 3.87 (m, 0.4H), 3.85 – 3.81 (m, 0.6H), 3.76 – 3.69 (m, 1H), 2.69 (s, 3H), 2.67 – 2.52 (m, 2H), 2.51 – 2.41 (m, 1H), 2.41 – 2.24 (m, 4H), 1.92 – 1.71 (m, 6H), 1.63 – 1.51 (m, 1H), 1.30 – 1.19 (m, 3H), 1.03 – 0.95 (m, 6H).

$^{13}$C NMR (151 MHz, $CDCl_3$) δ 171.94, 171.85, 166.35, 153.03, 152.99, 130.26, 129.97, 128.94, 128.89, 128.76, 125.02, 124.39, 115.44, 115.38, 88.15, 87.31, 87.06, 86.99, 78.57, 78.06, 73.55, 73.51, 72.99, 72.67, 72.47, 72.09, 40.11, 40.08, 39.29, 39.21, 34.49, 34.47, 32.28, 32.21, 30.10, 29.89, 28.12, 19.41, 17.28, 8.32, 8.17.

HRMS (ESI): Anal. Calcd for $C_{24}H_{36}NO_6S^+$ $[M+H]^+$ 466.2258, found 466.2258

IR ($CH_2Cl_2$): $\nu_{max}$ ($cm^{-1}$) = 3398 (br, OH), 2958 (m, CH), 2922 (s, CH), 2853 (m, CH), 1725 (s, C=O), 1709 (s, C=O), 1462 (m, CH), 1381 (m, CH), 1348 (m, OH), 1330 (m, OH)

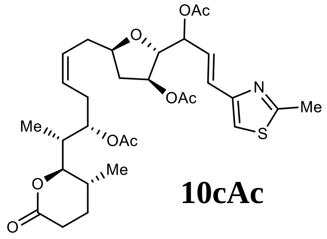


**10cAc**

**10cAc** was prepared according to general procedure A (1.27 mg, 76%).

**Analytical Data for 10cAc:**

$R_f$ = 0.24 (EtOAc)

HRMS (ESI): Anal. Calcd. for $C_{30}H_{42}NO_9^+$ $[M+H]^+$ 592.2575, found 592.2535

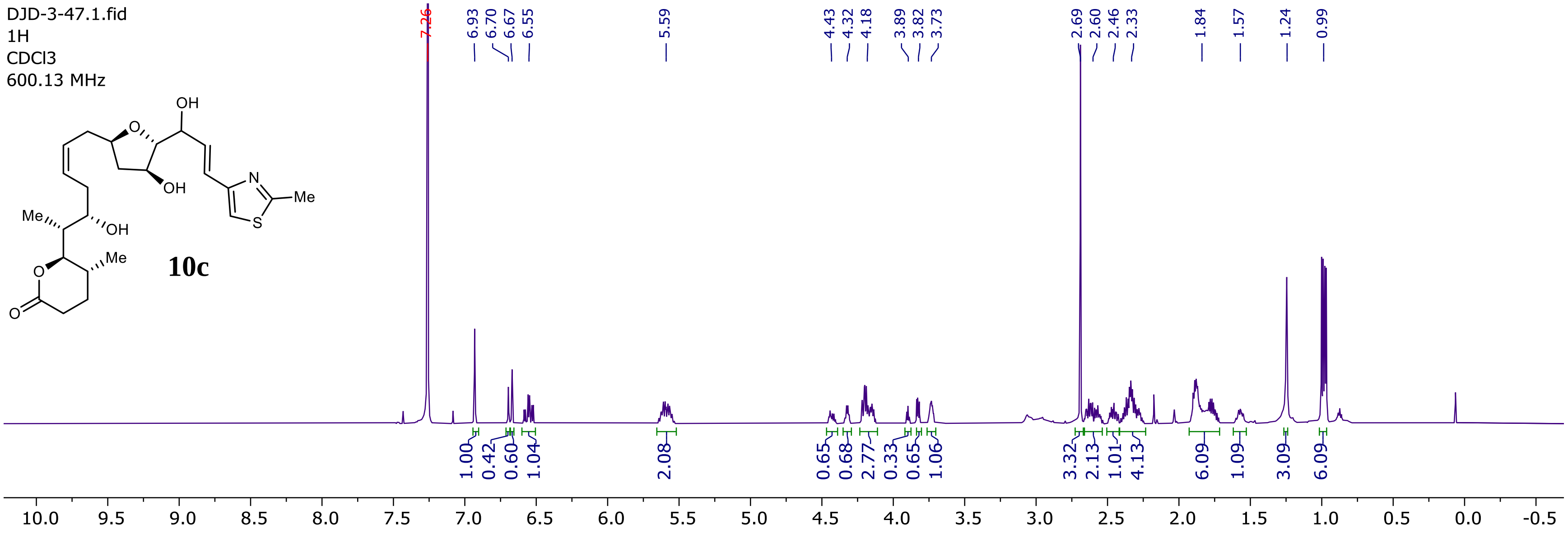
DJD-3-47.1.fid
1H
CDCl3
600.13 MHz
10c
7.26
6.93
6.70
6.67
6.55
5.59
4.43
4.32
4.18
3.89
3.82
3.73
2.69
2.60
2.46
2.33
1.84
1.57
1.24
0.99
1.00
0.42
0.60
1.04
2.08
0.65
0.68
2.77
0.33
0.65
1.06
3.32
2.13
1.01
4.13
6.09
1.09
3.09
6.09

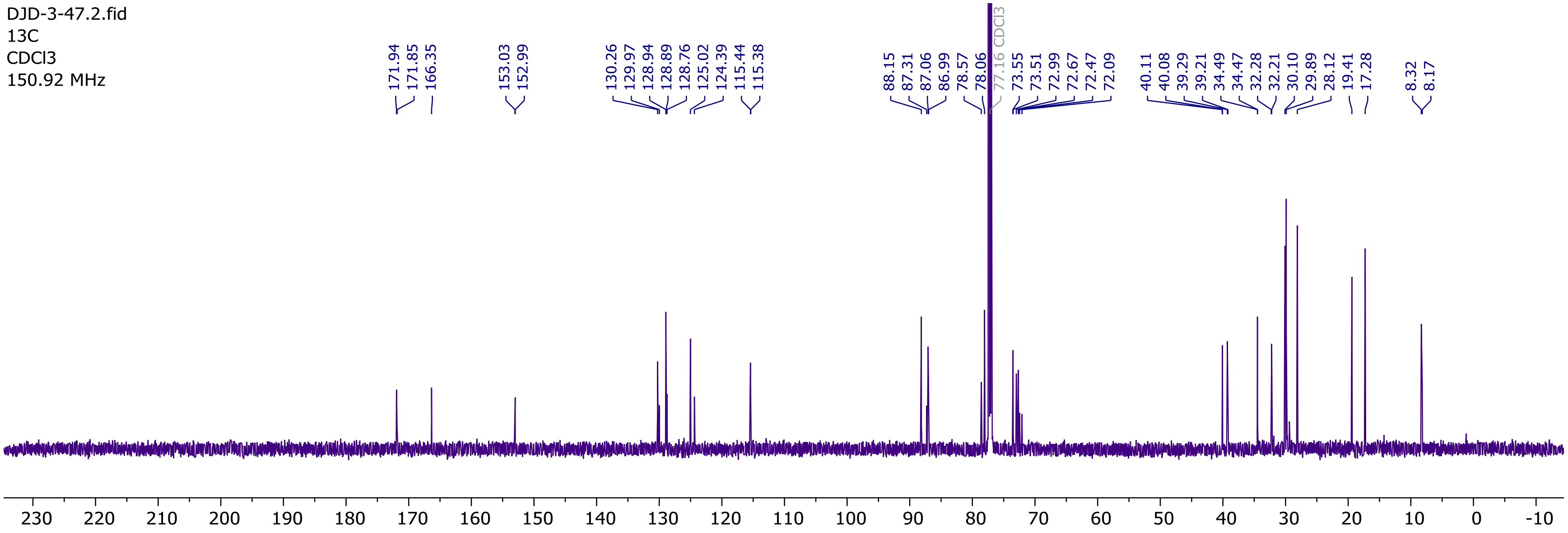
DJD-3-47.2.fid
13C
CDCl3
150.92 MHz
171.94
171.85
166.35
153.03
152.99
130.26
129.97
128.94
128.89
128.76
125.02
124.39
115.44
115.38
88.15
87.31
87.06
86.99
78.57
78.06
77.16 CDCl3
73.55
73.51
72.99
72.67
72.47
72.09
40.11
40.08
39.29
39.21
34.49
34.47
32.28
32.21
30.10
29.89
28.12
19.41
17.28
8.32
8.17

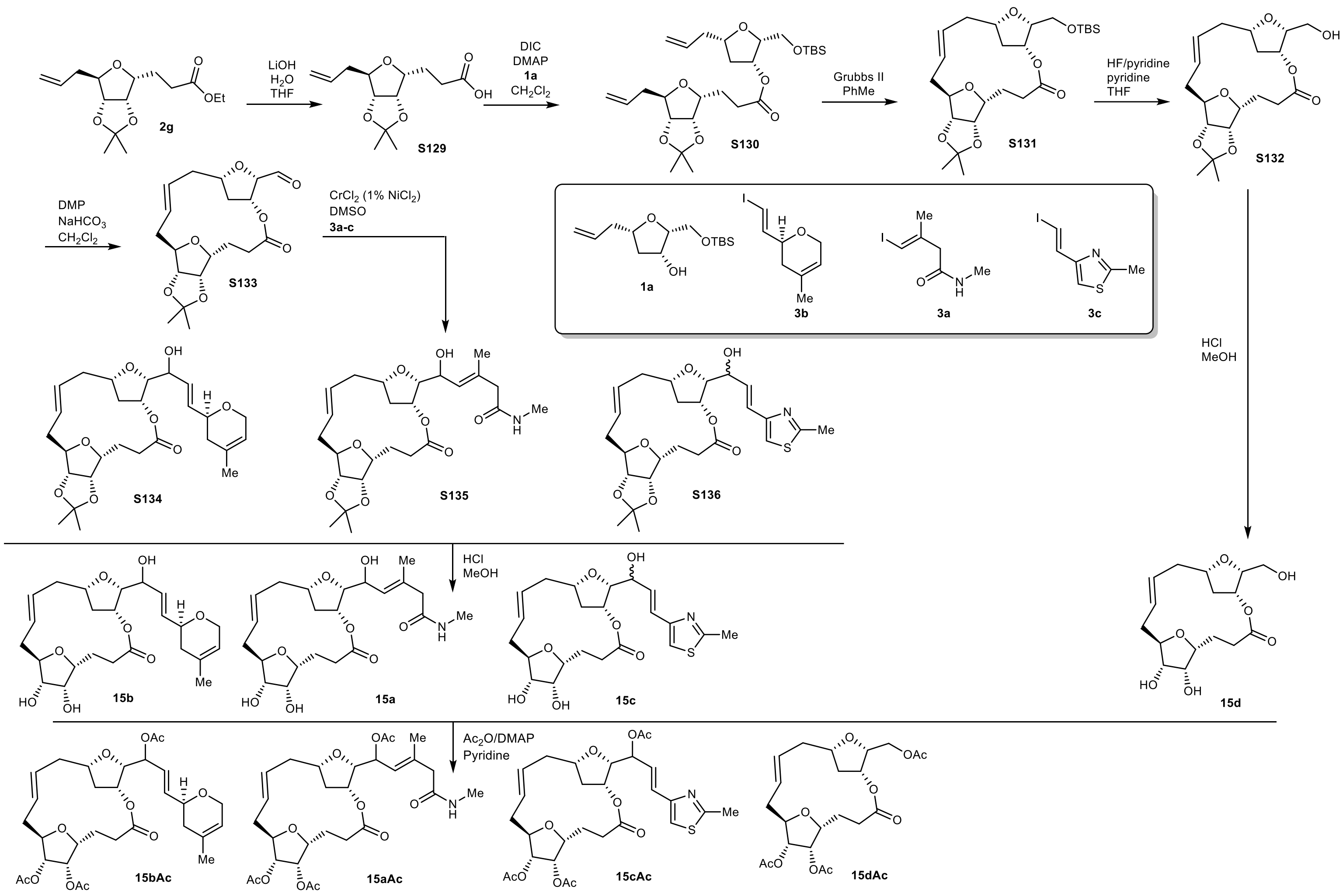


**Supplementary Fig. 47 | Synthesis of pMLs 15a-d and 15aAc-dAc.**

Abbreviations: THF = tetrahydrofuran, DIC = N,N'-diisopropylcarbodiimide, TBS = tert-butyldimethylsilyl, Grubbs II = Dichloro[1,3-bis(2,4,6-trimethylphenyl)-2-imidazolidinylidene](benzylidene)(tricyclohexylphosphine)ruthenium(II), THF = tetrahydrofuran, DMP = Dess-Martin periodinane, DMSO = dimethylsulfoxide, DMAP = 4-dimethylaminopyridine.

Note: Reaction performed in capped vessel under ambient atmosphere.
To a stirred solution of **2g** (299 mg, 1.05 mmol, 1.0 eq.) in 2:1 THF:$H_2O$ (3.9 mL) was added 1M LiOH (3.15 mL, 3.15 mmol, 3.0 eq.). The mixture was stirred vigorously at rt for 1 h and quenched with 1M HCl until slightly acidic. The mixture was extracted with $Et_2O$ (3x 50 mL) and the combined organic layers were dried ($MgSO_4$), filtered, and the solvent was removed in vacuo to yield acid **S129** (260 mg, 96%) as a yellow oil.

Note: Reaction performed in capped vessel under ambient atmosphere.
To a rt stirred solution of acid **S129** (179 mg, 0.70 mmol, 1.4 eq.), alcohol **1a** (136 mg, 0.50 mmol, 1.0 eq.), and DMAP (24 mg, 0.20 mmol, 0.40 eq.) dissolved in anhydrous $CH_2Cl_2$ (5 mL), was added DIC (0.18 mL, 145 mg, 1.15 mmol, 2.3 eq.). The reaction mixture was stirred for 2.5 h at rt, after which time starting material was consumed as monitored by TLC analysis. The reaction mixture was quenched with $H_2O$ (20 mL) and the aqueous layer was extracted with $CH_2Cl_2$ (3x 40 mL). The combined organic layers were dried ($Na_2SO_4$), filtered, and solvent was removed in vacuo. The crude product was purified via flash column chromatography (17:3 to 3:1 hexanes/$Et_2O$). Appropriate fractions were pooled, and solvent was removed in vacuo to yield **S130** (248 mg, 97%) as a pale yellow oil.

To a stirred, heated (60 °C) solution of **S130** (248 mg, 0.486 mmol, 1.0 eq.) in anhydrous toluene (deoxygenated via 5x freeze-pump-thaw cycles) (200 mL) was added dropwise Grubbs 2$^{nd}$ generation catalyst (83 mg, 0.097 mmol, 0.2 eq.) in anhydrous, deoxygenated toluene (2.2 mL). After 30 minutes stirring at 60 °C, the reaction was cooled to 0 °C and quenched with potassium 2-isocyanoacetate (100 mg) in MeOH (10 mL). The mixture was warmed to rt and stirred for 1 h, then run through a plug of silica gel ($Et_2O$) and the solvent was removed in vacuo. The crude product was purified via flash column chromatography (19:1 to 17:3 hexanes/acetone). Appropriate fractions were pooled, and solvent was removed in vacuo to yield **S131** (195 mg, 83%) as a colorless oil and a mixture of (*E*) and (*Z*) isomers (4:1).

Note: Reaction performed in capped vessel under ambient atmosphere.
To a plastic vessel containing a stirred solution of **S131** (195 mg, 0.403 mmol, 1.0 eq.) in anhydrous THF (3.7 mL) was added pyridine (1.57 mL) and 70% HF/pyridine (1.57 mL, 60.5 mmol, 150 eq.). After stirring for 15 minutes at rt, the reaction was quenched with saturated $NaHCO_3$ (70 mL) and the aqueous layer was extracted with $CH_2Cl_2$ (3x 50 mL) and the combined organic layers were dried ($Na_2SO_4$), filtered, and the solvent was removed in vacuo. The crude product was purified via flash column chromatography using $AgNO_3$ infused silica gel (3:1 to 11:9 hexanes/acetone). Appropriate fractions were pooled, and solvent was removed in vacuo to yield (*E*)-isomer **S132** (109 mg, 73%) as a white solid.

Note: Reaction performed in capped vessel under ambient atmosphere.
To a stirred solution of **S132** (102 mg, 0.278 mmol, 1.0 eq.) in anhydrous $CH_2Cl_2$ (2.8 mL) was added $NaHCO_3$ (70 mg, 0.83 mmol, 1.5 eq.) followed by Dess-Martin Periodinane (177 mg, 0. 416 mmol, 1.5 eq.). After stirring for 1 h at rt, more Dess-Martin Periodinane (59 mg, 0.139 mmol, 0.5 eq.) was added. After 2 h more, the mixture was quenched with a solution of 1:1:1 $H_2O$-saturated $NaHCO_3$-saturated $Na_2S_2O_3$ (3 mL) and the biphasic mixture was stirred vigorously for 1 h. The aqueous layer was extracted with $CH_2Cl_2$ (3x 20 mL) and the combined organic layers were dried ($Na_2SO_4$), filtered, and the solvent was removed in vacuo. The crude product was run through a plug of C2-modified silica gel ($Et_2O$) and solvent was removed in vacuo to yield the crude aldehyde **S133** (94.6 mg, 93%) which was divided into 3 equal portions for the following steps.

To a solution of **S133** (32 mg, 0.086 mmol, 1.0 eq.) in anhydrous DMSO (deoxygenated via 5x freeze-pump-thaw cycles) (0.5 mL) was added $CrCl_2$ with doped with 1 % $NiCl_2$ (w/w) (106 mg, 0.86 mmol, 10 eq.). **3b** (36 mg, 0.14 mmol, 1.7 eq.) was added in deoxygenated DMSO (1.25 mL) and the mixture was stirred for 18 h. The reaction was quenched with 1:1 $H_2O$-brine (10 mL). The aqueous layer was extracted with $Et_2O$ (10x 15 mL) and the combined organic layers were dried ($MgSO_4$), filtered, and the solvent was removed in vacuo. The crude product was purified via flash column chromatography (17:3 to 7:3 hexanes/acetone). Appropriate fractions were pooled, and solvent was removed in vacuo to yield **S134** (13.1 mg, 31%) as a colorless oil and a mixture of diastereomers (d.r. = 11:1).

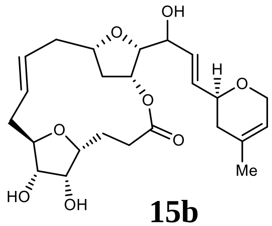


**15b**

Note: Reaction performed in capped vessel under ambient atmosphere.
To a stirred vial containing **S134** (10.3 mg, 0.0209 mmol, 1.0 eq.) dissolved in MeOH (3.4 mL) was added 1.2M HCl in MeOH (87 µL, 0.105 mmol, 5.0 eq.). After 7 days stirring at rt, the reaction was quenched with $NaHCO_{3\,(s)}$ (16 mg) and after effervescence had subsided, the solvent was removed in vacuo. The crude product was purified via flash column chromatography (39:1 to 19:1 $CH_2Cl_2$:MeOH). Appropriate fractions were pooled to yield **15b** (8.8 mg, 93%) as a white solid and a mixture of diastereomers (d.r. = 11:1).

**Analytical Data for 15b:**

$R_f$ = 0.13 (19:1 $CH_2Cl_2$/MeOH)

$[\alpha]_D^{20}$ = +66 ° (c = 0.67, $CH_2Cl_2$)

$^{1}$H NMR (600 MHz, $CDCl_3$) δ 5.99 – 5.86 (m, 2H), 5.75 – 5.67 (m, 1H), 5.53 (dtd, *J* = 15.6, 5.9, 2.8 Hz, 1H), 5.41 (s, 1H), 5.22 (ddd, *J* = 7.3, 3.9, 1.3 Hz, 1H), 4.51 (dd, *J* = 7.4, 4.3 Hz, 1H), 4.21 – 4.16 (m, 2H), 4.13 – 4.03 (m, 3H), 3.95 (dd, *J* = 4.4, 2.7 Hz, 1H), 3.92 (dt, *J* = 8.1, 4.0 Hz, 1H), 3.78 – 3.73 (m, 1H), 3.55 (dd, *J* = 7.4, 3.9 Hz, 1H), 2.86 – 2.72 (m, 2H), 2.63 (ddd, *J* = 14.3, 11.2, 4.5 Hz, 1H), 2.57 – 2.45 (m, 2H), 2.42 – 2.32 (m, 2H), 2.29 – 2.21 (m, 1H), 2.12 – 1.90 (m, 6H), 1.70 (d, *J* = 3.3 Hz, 3H), 1.65 (ddd, *J* = 14.6, 7.9, 1.4 Hz, 1H).

$^{13}$C NMR (151 MHz, $CDCl_3$) δ 174.17, 131.98, 131.61, 130.86, 129.44, 127.30, 119.70, 83.62, 80.64, 78.23, 76.89, 76.21, 74.60, 73.96, 73.47, 69.52, 65.82, 37.07, 35.74, 35.73, 34.39, 30.80, 25.91, 23.10.

HRMS (ESI): Anal. Calcd. for $C_{24}H_{35}O_8^+$ $[M+H]^+$ 451.23264, found 451.23269
IR (neat): $\nu_{max}$ $(cm^{-1})$ = 3403 (br, OH), 2925 (s, CH), 2885 (m, CH), 1725 (s, C=O), 1710 (s, C=O), 1429 (m), 1382 (m), 1339 (m), 1240 (s)

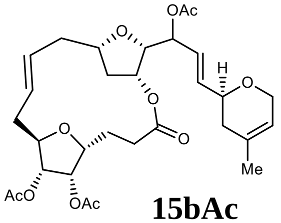


**15bAc**

**15bAc** was prepared according to general procedure A (1.01 mg, 53%).

**Analytical Data for 15bAc:**

$R_f$ = 0.50 (1:1 hexanes/EtOAc)

HRMS (ESI): Anal. Calcd. for $C_{30}H_{44}NO_{11}^+$ $[M+NH_4]^+$ 594.2909, found 594.2890

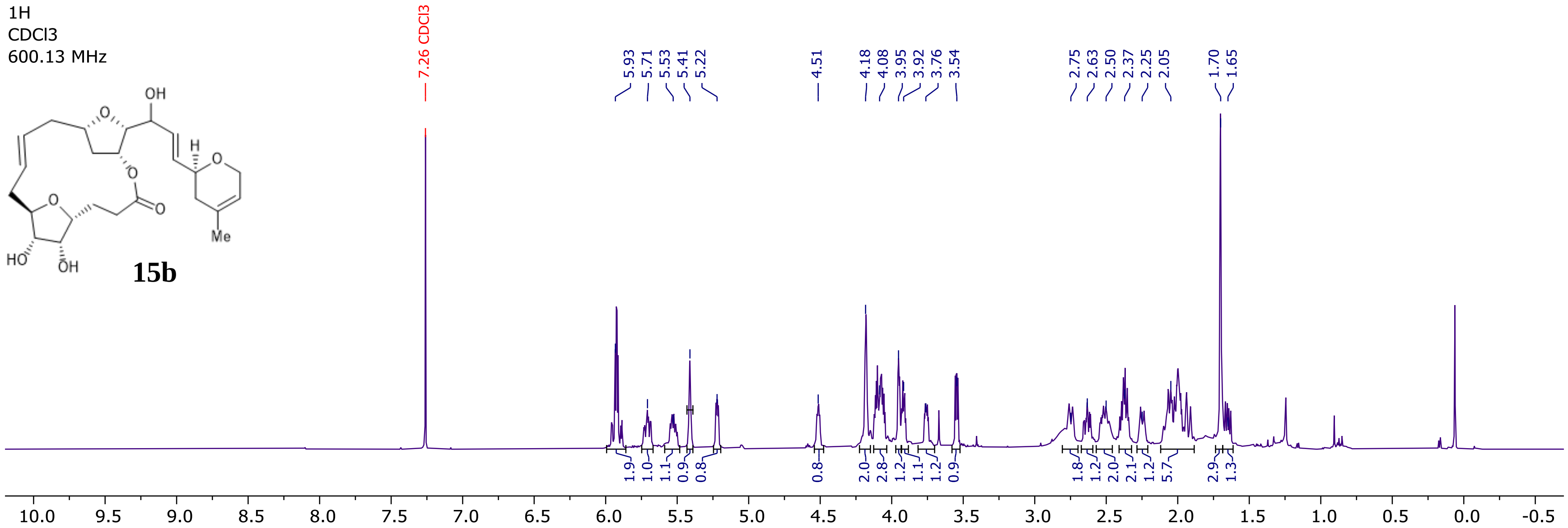
1H
CDCl3
600.13 MHz
15b
7.26 CDCl3
5.93
5.71
5.53
5.41
5.22
4.51
4.18
4.08
3.95
3.92
3.76
3.54
2.75
2.63
2.50
2.37
2.25
2.05
1.70
1.65
1.9
1.0
1.1
0.9
0.8
0.8
2.0
2.8
1.2
1.1
1.2
0.9
1.8
1.2
2.0
2.1
1.2
5.7
2.9
1.3
10.0
9.5
9.0
8.5
8.0
7.5
7.0
6.5
6.0
5.5
5.0
4.5
4.0
3.5
3.0
2.5
2.0
1.5
1.0
0.5
0.0
-0.5

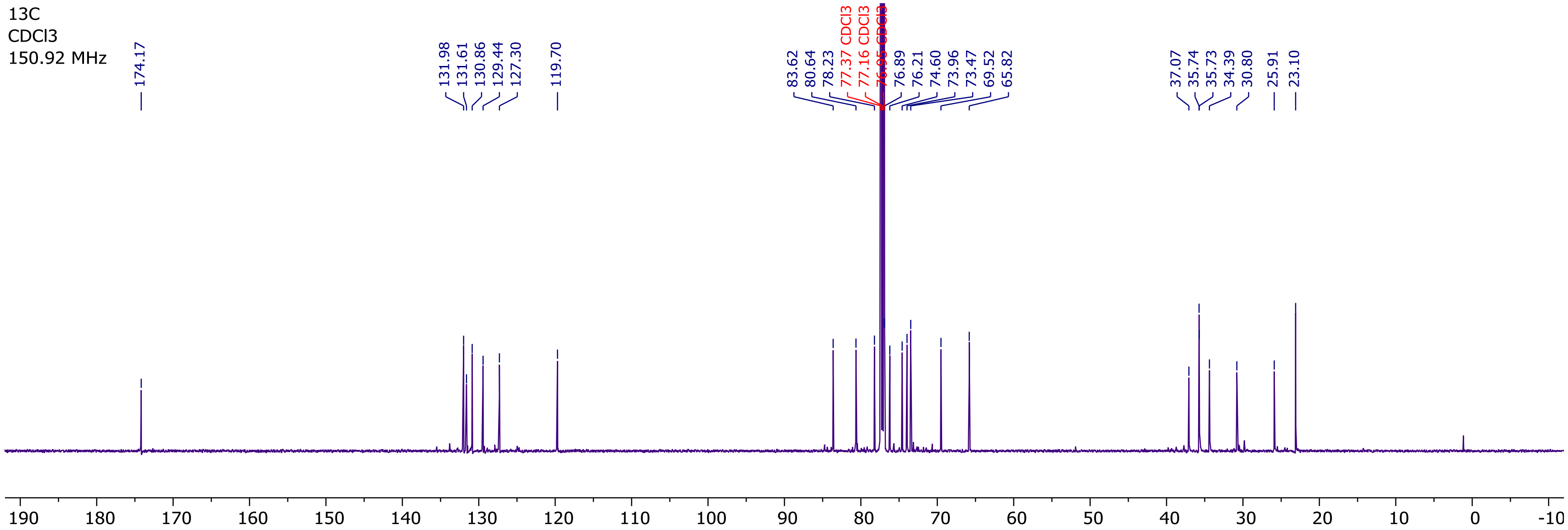
13C
CDCl3
150.92 MHz
174.17
131.98
131.61
130.86
129.44
127.30
119.70
83.62
80.64
78.23
77.37 CDCl3
77.16 CDCl3
76.95 CDCl3
76.89
76.21
74.60
73.96
73.47
69.52
65.82
37.07
35.74
35.73
34.39
30.80
25.91
23.10
190
180
170
160
150
140
130
120
110
100
90
80
70
60
50
40
30
20
10
0
-10

To a solution of **S133** (32 mg, 0.086 mmol, 1.0 eq.) in anhydrous DMSO (deoxygenated via 5x freeze-pump-thaw cycles) (0.5 mL) was added $CrCl_2$ with doped with 1 % $NiCl_2$ (w/w) (106 mg, 0.86 mmol, 10 eq.). **3a** (62 mg, 0.26 mmol, 3.0 eq.) was added in deoxygenated DMSO (1.0 mL) and the mixture was stirred for 18 h. The reaction was quenched with 1:1 $H_2O$-brine (15 mL). The aqueous layer was extracted with $Et_2O$ (10x 15 mL) and the combined organic layers were dried ($MgSO_4$), filtered, and the solvent was removed in vacuo. The crude product was purified via flash column chromatography (1:1 to 3:7 hexanes/acetone). Appropriate fractions were pooled, and solvent was removed in vacuo to yield **S135** (5.6 mg, 14%) as a colorless oil and and a mixture of diastereomers (d.r. = 13:1).

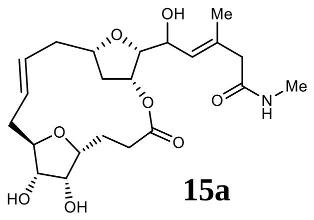


**15a**

Note: Reaction performed in capped vessel under ambient atmosphere.
To a stirred vial containing **S135** (5.6 mg, 0.012 mmol, 1.0 eq.) dissolved in MeOH (1.9 mL) was added 1.2M HCl in MeOH (49 µL, 0.58 mmol, 5.0 eq.). After 7 days stirring at rt, the reaction was quenched with $NaHCO_{3\,(s)}$ (12 mg) and after effervescence had subsided, the solvent was removed in vacuo. The crude product was purified via flash column chromatography (19:1 to 9:1 $CH_2Cl_2$/MeOH). Appropriate fractions were pooled to yield **15a** (1.0 mg, 20%) as a colorless oil and a mixture of diastereomers (d.r. = 13:1).

**Analytical Data for 15a:**

$R_f$ = 0.12 (23:2 $CH_2Cl_2$/MeOH)

$[\alpha]_D^{20}$ = +170 ° (c = 0.10, 1:1 $CH_2Cl_2$/MeOH)

$^1$H NMR (600 MHz, $CDCl_3$) δ 6.38 (d, *J* = 5.0 Hz, 1H), 5.62 (ddd, *J* = 13.9, 10.0, 3.4 Hz, 1H), 5.59 – 5.51 (m, 1H), 5.47 (dd, *J* = 9.0, 1.7 Hz, 1H), 5.27 – 5.21 (m, 1H), 4.79 (dd, *J* = 8.9, 7.7 Hz, 1H), 4.16 – 4.07 (m, 2H), 3.99 (dd, *J* = 4.4, 2.7 Hz, 1H), 3.93 (dt, *J* = 8.2, 4.0 Hz, 1H), 3.81 (dt, *J* = 9.3, 3.3 Hz, 1H), 3.62 (dd, *J* = 7.7, 3.9 Hz, 1H), 3.05 (d, *J* = 15.6 Hz, 1H), 2.94 (d, *J* = 15.6 Hz, 1H), 2.78 (d, *J* = 4.8 Hz, 3H), 2.67 (ddd, *J* = 14.1, 11.6, 4.0 Hz, 2H), 2.51 (ddd, *J* = 14.5, 8.2, 3.8 Hz, 1H), 2.48 – 2.42 (m, 1H), 2.39 (ddd, *J* = 14.3, 5.4, 3.9 Hz, 1H), 2.31 – 2.24 (m, 2H), 2.13 – 2.07 (m, 2H), 2.02 (ddt, *J* = 15.7, 11.6, 4.3 Hz, 3H), 1.80 (d, *J* = 1.4 Hz, 3H), 1.73 (ddd, *J* = 14.6, 7.9, 1.4 Hz, 1H).

$^{13}$C NMR (151 MHz, $CDCl_3$) δ 174.25, 171.50, 134.69, 130.70, 128.71, 127.87, 84.46, 80.61, 78.20, 76.79, 75.44, 74.65, 73.96, 66.01, 47.58, 37.28, 35.87, 34.40, 30.74, 26.60, 25.83, 17.73.

HRMS (ESI): Anal. Calcd. for $C_{22}H_{34}NO_8^+$ $[M+H]^+$ 440.2279, found 440.2277
IR (neat): $\nu_{max}$ ($cm^{-1}$) = 3353 (br, OH), 2926 (s, CH), 2864 (m, CH), 1726 (s, C=O), 1643 (s, C=C), 1549 (m), 1433 (m), 1337 (m), 1241 (s)

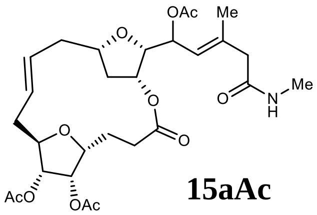


**15aAc**

**15aAc** was prepared according to general procedure A (0.98 mg, 35%).

**Analytical Data for 15aAc:**

$R_f$ = 0.15 (EtOAc)

HRMS (ESI): Anal. Calcd. for $C_{28}H_{43}N_2O_{11}^+$ $[M+NH_4]^+$ 583.2861, found 583.2860

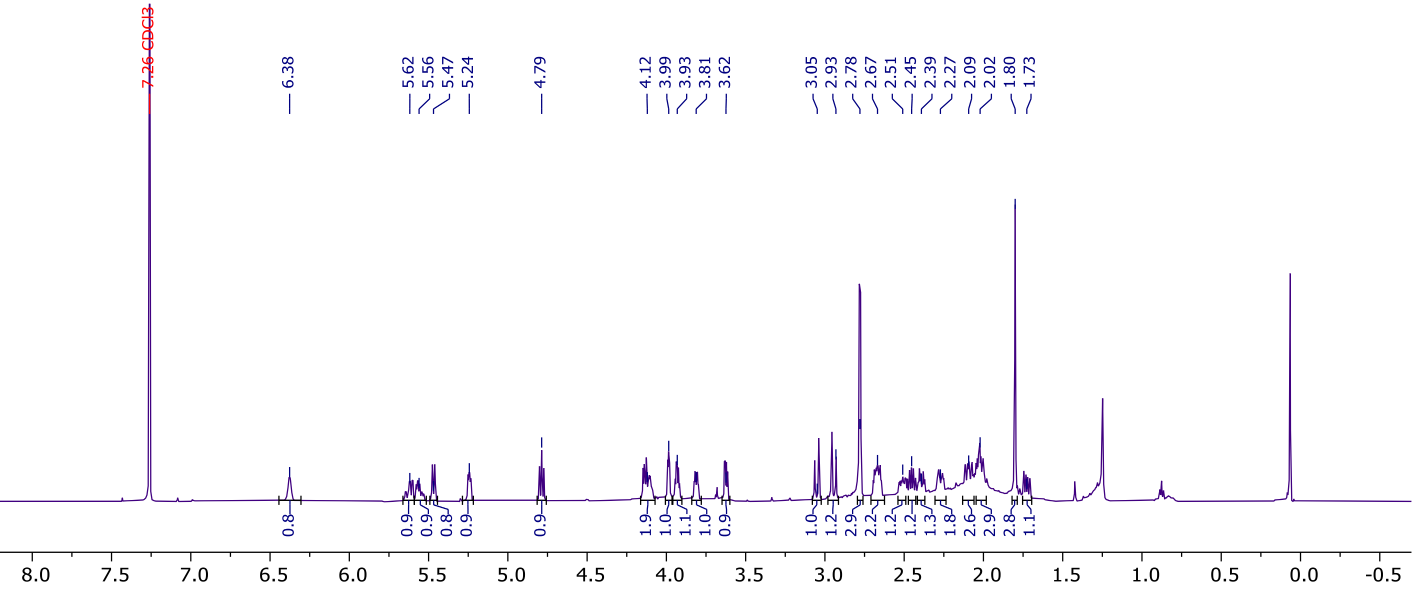
1H
CDCl3
600.13 MHz
7.26 CDCl3
6.38
5.62
5.56
5.47
5.24
4.79
4.12
3.99
3.93
3.81
3.62
3.05
2.93
2.78
2.67
2.51
2.45
2.39
2.27
2.09
2.02
1.80
1.73
0.8
0.9
0.9
0.8
0.9
0.9
1.9
1.0
1.1
1.0
0.9
1.0
1.2
2.9
2.2
1.2
1.2
1.3
1.8
2.6
2.9
2.8
1.1
10.0
9.5
9.0
8.5
8.0
7.5
7.0
6.5
6.0
5.5
5.0
4.5
4.0
3.5
3.0
2.5
2.0
1.5
1.0
0.5
0.0
-0.5

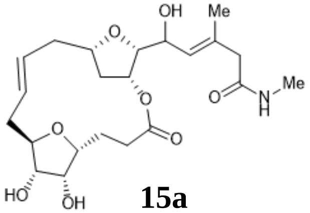
OH
Me
O
Me
O
N
H
O
O
O
HO
OH

**15a**

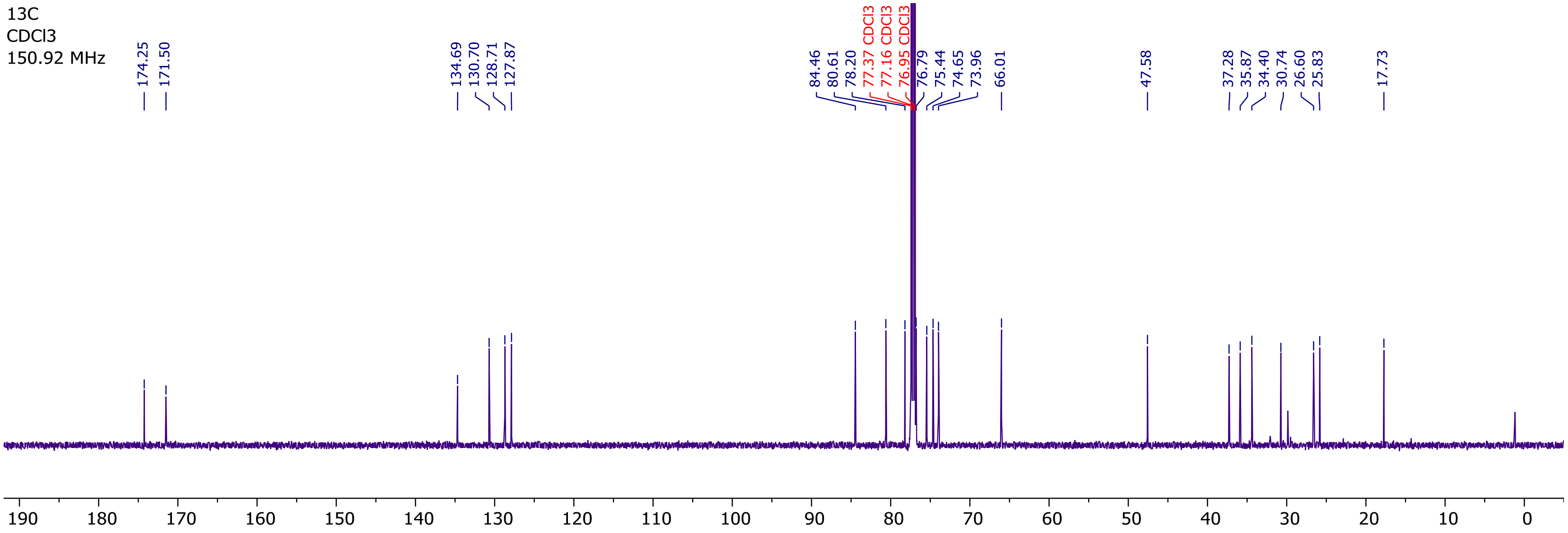
13C
CDCl3
150.92 MHz
174.25
171.50
134.69
130.70
128.71
127.87
84.46
80.61
78.20
77.37 CDCl3
77.16 CDCl3
76.95 CDCl3
76.79
75.44
74.65
73.96
66.01
47.58
37.28
35.87
34.40
30.74
26.60
25.83
17.73
190
180
170
160
150
140
130
120
110
100
90
80
70
60
50
40
30
20
10
0

To a solution of **S133** (32 mg, 0.086 mmol, 1.0 eq.) in anhydrous DMSO (deoxygenated via 5x freeze-pump-thaw cycles) (0.5 mL) was added $CrCl_2$ with doped with 1% $NiCl_2$ (w/w) (106 mg, 0.86 mmol, 10 eq.). **3c** (43 mg, 0.17, 2.0 eq.) was added in deoxygenated DMSO (0.75 mL) and the mixture was stirred for 18 h. The reaction was quenched with 1:1 $H_2O$-brine (15 mL). The aqueous layer was extracted with $Et_2O$ (10x 15 mL) and the combined organic layers were dried ($MgSO_4$), filtered, and the solvent was removed in vacuo. The crude product was purified via flash column chromatography (3:1 to 7:3 hexanes/acetone). Appropriate fractions were pooled, and solvent was removed in vacuo to yield **S136** (15.3 mg, 36%) as a colorless oil and a mixture of diastereomers (4:1).

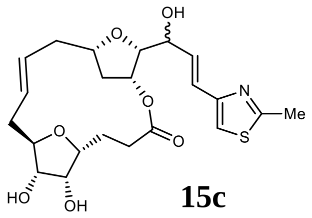


**15c**

Note: Reaction performed in capped vessel under ambient atmosphere.
To a stirred vial containing **S136** (13.3 mg, 0.027 mmol, 1.0 eq.) dissolved in MeOH (4.4 mL) was added 1.2M HCl in MeOH (110 µL, 0.132 mmol, 4.9 eq.). After 3 days stirring at rt, the reaction was quenched with $NaHCO_{3\,(s)}$ (21 mg) and after effervescence had subsided, the solvent was removed in vacuo. The crude product was purified via flash column chromatography (24:1 to 19:1 $CH_2Cl_2$/MeOH). Appropriate fractions were pooled to yield **15c** (7.5 mg, 61%) as a white solid and a mixture of diastereomers (4:1).

**Analytical Data for 15c:**

$R_f$ = 0.25 (47:3 $CH_2Cl_2$/MeOH)

For the following reported NMR peaks: Several resonances in the $^1$H NMR spectrum integrated for 0.8 and 0.2 protons and are unique to both the diastereomers. In these cases an integral value of "0.8 H" and "0.2 H" is assigned.

$^1$H NMR (601 MHz, $CDCl_3$) δ 6.94 (s, 0.8H), 6.91 (s, 0.2H), 6.74 – 6.64 (m, 2H), 5.79 – 5.68 (m, 1H), 5.57 – 5.48 (m, 1H), 5.25 (ddd, *J* = 7.4, 3.8, 1.4 Hz, 0.8H), 5.09 (dd, *J* = 6.7, 3.8 Hz, 0.2H), 4.74 (ddd, *J* = 7.8, 6.0, 1.5 Hz, 0.2H), 4.66 (t, *J* = 6.1 Hz, 0.8H), 4.15 – 4.06 (m, 2H), 3.99 – 3.92 (m, 2H), 3.78 (dt, *J* = 9.4, 3.2 Hz, 1H), 3.62 (dd, *J* = 7.4, 3.8 Hz, 1H), 3.05 (s, 1H), 2.76 (s, 1H), 2.73 (s, 1H), 2.70 (s, 2.4H), 2.68 (s, 0.6H), 2.67 – 2.62 (m, 1H), 2.52 (ddd, *J* = 12.4, 8.9, 3.7 Hz, 1H), 2.43 – 2.34 (m, 2H), 2.24 (dd, *J* = 13.8, 6.2 Hz, 1H), 2.10 – 1.94 (m, 4H), 1.85 (s, 1H), 1.68 (ddd, *J* = 14.6, 7.9, 1.4 Hz, 1H).

Major diastereomer
$^{13}$C NMR (151 MHz, $CDCl_3$) δ 174.24, 166.38, 153.22, 132.06, 129.38, 127.40, 123.92, 115.14, 83.72, 80.72, 78.32, 76.31, 74.63, 73.93, 69.76, 37.10, 35.69, 34.44, 30.85, 25.91, 19.38.

Minor diastereomer

$^{13}$C NMR (151 MHz, $CDCl_3$) δ 166.42, 152.95, 129.19, 129.05, 127.57, 125.08, 115.51, 84.78, 80.54, 76.93, 75.89, 74.69, 70.61, 37.77, 35.55, 34.41, 30.71, 25.83.

HRMS (ESI): Anal. Calcd. for $C_{22}H_{30}NO_7S^+$ $[M+H]^+$ 452.1738, found 452.1727

IR (neat): $\nu_{max}$ $(cm^{-1})$ = 3383 (br, OH), 2925 (m, CH), 2869 (m, CH), 1725 (s, C=O), 1709 (s, C=O), 1429 (m), 1338 (m), 1240 (s)

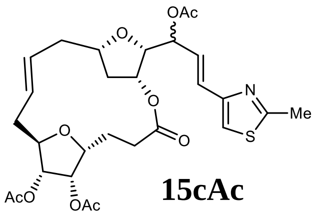


**15cAc**

**15cAc** was prepared according to general procedure A (1.20 mg, 95%).

**Analytical Data for 15cAc:**

$R_f$ = 0.47 and 0.64 ($Et_2O$)

Major diastereomer

$^{1}$H NMR (600 MHz, $C_6D_6$) δ 7.12 (dd, *J* = 15.4, 3.3 Hz, 1H), 6.70 (dt, *J* = 15.4, 1.5 Hz, 1H), 6.31 (d, *J* = 2.6 Hz, 1H), 6.18 (ddt, *J* = 9.2, 7.5, 1.6 Hz, 1H), 5.98 (ddd, *J* = 14.7, 10.6, 3.3 Hz, 1H), 5.48 (ddd, *J* = 15.2, 7.5, 4.7 Hz, 1H), 5.36 (dd, *J* = 6.9, 3.6 Hz, 1H), 5.29 (ddd, *J* = 9.1, 4.3, 1.2 Hz, 1H), 5.20 (dt, *J* = 4.8, 2.4 Hz, 1H), 4.21 (dt, *J* = 9.3, 3.8 Hz, 1H), 3.80 (ddt, *J* = 7.0, 4.7, 2.3 Hz, 1H), 3.75 – 3.66 (m, 2H), 3.64 (ddd, *J* = 9.2, 3.6, 1.2 Hz, 1H), 2.93 – 2.79 (m, 1H), 2.67 – 2.59 (m, 1H), 2.50 (ddd, *J* = 13.3, 9.0, 3.7 Hz, 1H), 2.27 – 2.22 (m, 1H), 2.21 (d, *J* = 1.2 Hz, 2H), 2.11 – 2.04 (m, 1H), 2.02 (ddd, *J* = 16.6, 10.5, 6.1 Hz, 1H), 1.95 – 1.88 (m, 1H), 1.82 – 1.77 (m, 2H), 1.74 (d, *J* = 1.1 Hz, 3H), 1.72 – 1.70 (m, 3H), 1.64 (d, *J* = 1.2 Hz, 3H), 1.57 (dd, *J* = 14.5, 7.5 Hz, 1H).

Major diastereomer

$^{13}$C NMR (151 MHz, $C_6D_6$) δ 173.01, 169.50, 169.44, 168.90, 165.35, 153.49, 130.78, 129.29, 127.15, 126.79, 115.86, 82.78, 77.64, 77.43, 76.83, 74.55, 74.09, 73.08, 71.26, 36.97, 36.01, 34.20, 30.72, 26.53, 20.69, 20.23, 20.17, 18.86.

IR (neat): $\nu_{max}$ $(cm^{-1})$ = 2928 (w, CH), 1743 (s, C=O), 1430 (w), 1372 (m), 1241 (s)

HRMS (ESI): Anal. Calcd. for $C_{28}H_{36}NO_{10}S^+$ $[M+H]^+$ 578.2054, found 578.2052

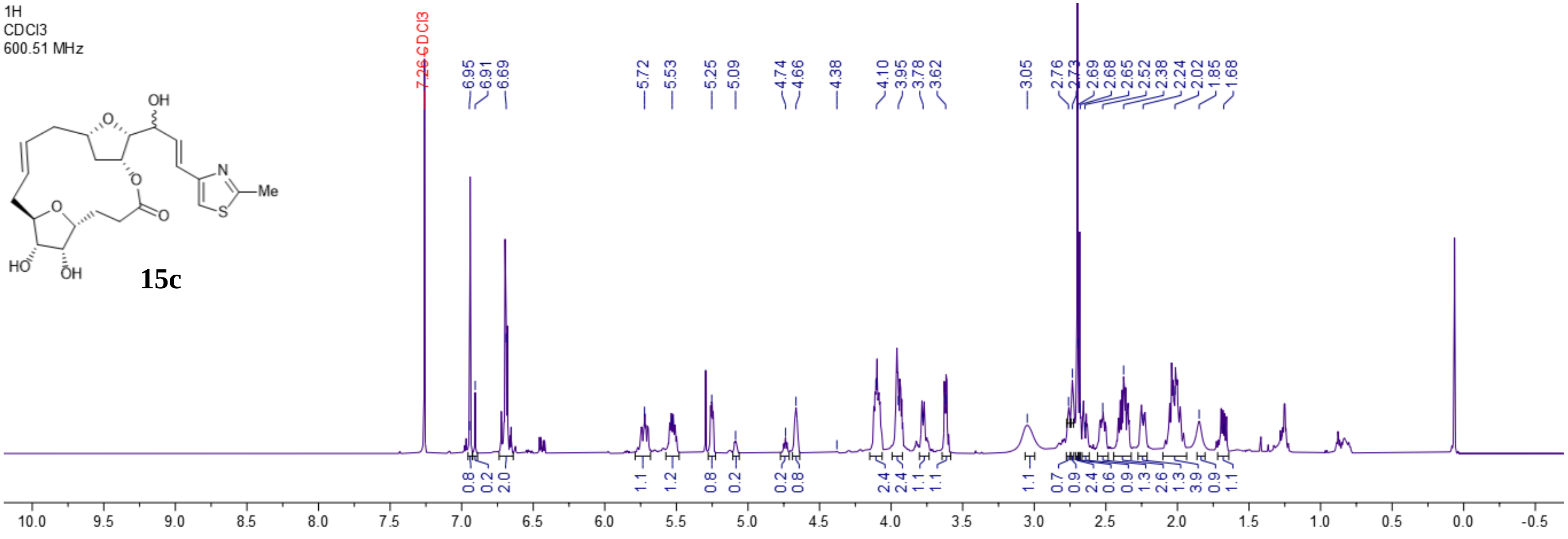
1H
CDCl3
600.51 MHz
OH
N
Me
S
O
HO
OH
15c
7.26 CDCl3
6.95
6.91
6.69
5.72
5.53
5.25
5.09
4.74
4.66
4.38
4.10
3.95
3.78
3.62
3.05
2.76
2.73
2.69
2.68
2.65
2.52
2.38
2.24
2.02
1.85
1.68

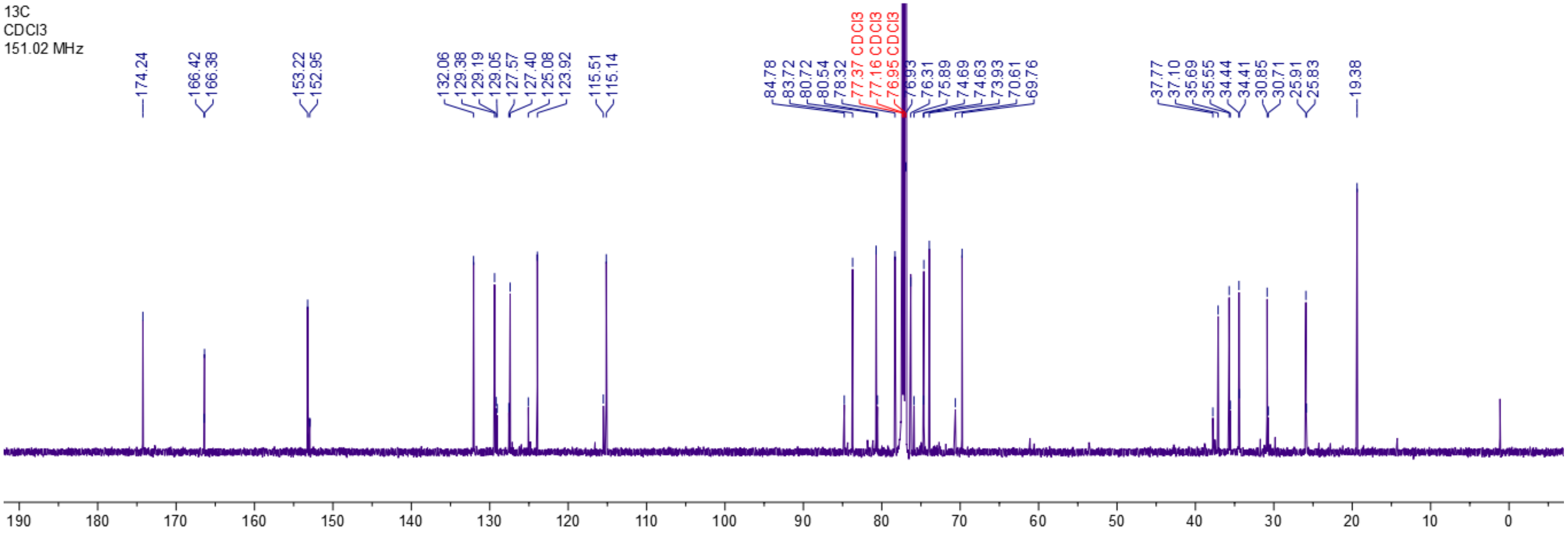
13C
CDCl3
151.02 MHz
174.24
166.42
166.38
153.22
152.95
132.06
129.38
129.19
129.05
127.57
127.40
125.08
123.92
115.51
115.14
84.78
83.72
80.72
80.54
78.32
77.37 CDCl3
77.16 CDCl3
76.95 CDCl3
76.93
76.31
75.89
74.69
74.63
73.93
70.61
69.76
37.77
37.10
35.69
35.55
34.44
34.41
30.85
30.71
25.91
25.83
19.38

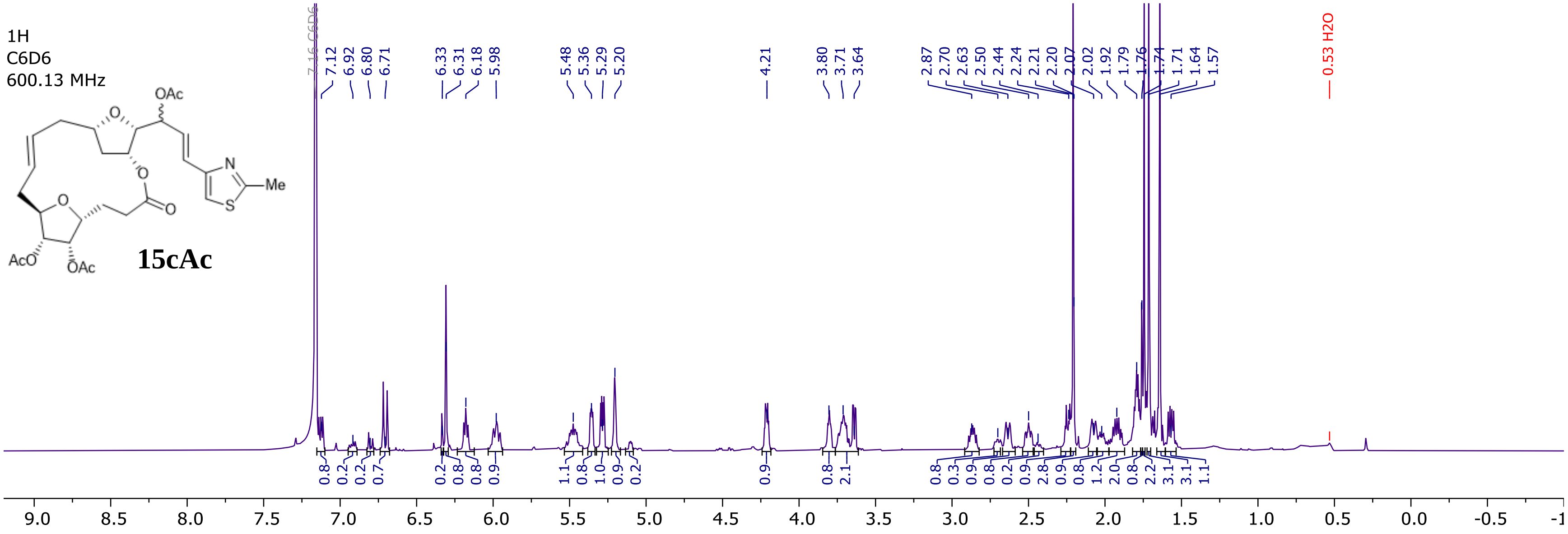
1H
C6D6
600.13 MHz
7.16 C6D6
7.12
6.92
6.80
6.71
6.33
6.31
6.18
5.98
5.48
5.36
5.29
5.20
4.21
3.80
3.71
3.64
2.87
2.70
2.63
2.50
2.44
2.24
2.21
2.20
2.07
2.02
1.92
1.79
1.76
1.74
1.71
1.64
1.57
0.53 H2O
0.8
0.2
0.2
0.7
0.2
0.8
0.8
0.9
1.1
0.8
1.0
0.9
0.2
0.9
0.8
2.1
0.8
0.3
0.9
0.8
0.2
0.9
2.8
0.9
0.8
1.2
2.0
0.8
2.2
3.1
3.1
1.1
OAc
O
O
N
Me
S
O
O
AcO
OAc
15cAc

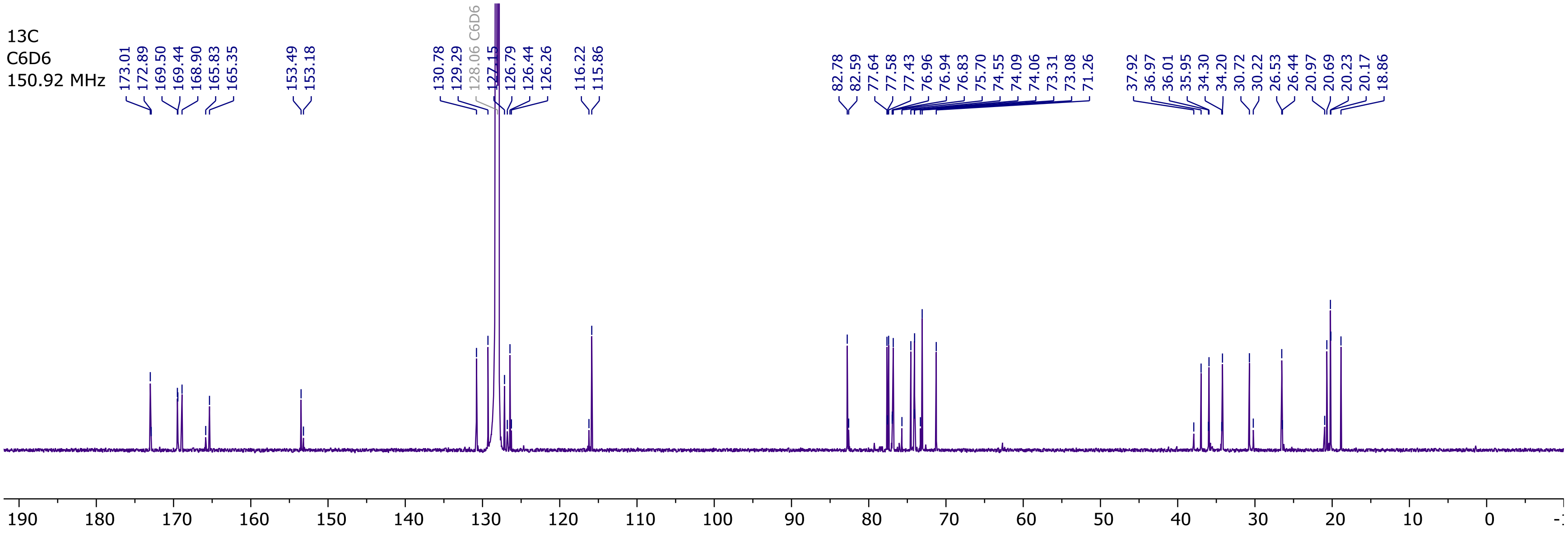
13C
C6D6
150.92 MHz
173.01
172.89
169.50
169.44
168.90
165.83
165.35
153.49
153.18
130.78
129.29
128.06 C6D6
127.15
126.79
126.44
126.26
116.22
115.86
82.78
82.59
77.64
77.58
77.43
76.96
76.94
76.83
75.70
74.55
74.09
74.06
73.31
73.08
71.26
37.92
36.97
36.01
35.95
34.30
34.20
30.72
30.22
26.53
26.44
20.97
20.69
20.23
20.17
18.86

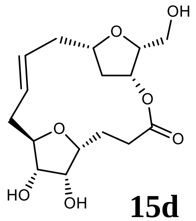

**15d**

Note: Reaction performed in capped vessel under ambient atmosphere.
To a stirred vial containing **S132** (5.4 mg, 0.015 mmol, 1.0 eq.) dissolved in MeOH (2.9 mL) was added 1.2M HCl in MeOH (61 µL, 0.073 mmol, 5.0 eq.). After 4 days stirring at 4 °C, the reaction was quenched with $NaHCO_{3\,(s)}$ (16 mg) and after effervescence had subsided, the solvent was removed in vacuo. The crude product was purified via flash column chromatography (24:1 to 93:7 $CH_2Cl_2$/MeOH). Appropriate fractions were pooled to yield **15d** (2.9 mg, 60%) as a clear oil and as a mixture with inseparable side product (4:1).

**Analytical Data for 15d:**

$R_f$ = 0.10 (19:1 $CH_2Cl_2$/MeOH)

$[\alpha]_D^{20}$ = +83 ° (c = 0.29, MeOH)

Product
$^1$H NMR (600 MHz, MeOD) δ 5.70 – 5.62 (m, 1H), 5.62 – 5.56 (m, 1H), 5.14 (ddd, *J* = 7.3, 3.5, 1.3 Hz, 1H), 4.11 – 4.04 (m, 2H), 3.99 – 3.75 (m, 7H), 3.70 (dt, *J* = 10.0, 2.9 Hz, 1H), 2.72 – 2.66 (m, 1H), 2.61 – 2.52 (m, 1H), 2.49 – 2.21 (m, 4H), 2.11 (ddd, *J* = 14.9, 10.4, 2.0 Hz, 1H), 2.03 – 1.95 (m, 1H), 1.92 – 1.85 (m, 1H), 1.64 (ddd, *J* = 14.5, 8.1, 1.4 Hz, 1H).

Product
$^{13}$C NMR (151 MHz, MeOD) δ 175.89, 130.30, 128.43, 83.35, 81.01, 79.62, 78.30, 77.13, 74.96, 74.87, 60.78, 38.30, 36.43, 35.06, 31.26, 27.03.

HRMS (ESI): Anal. Calcd. for $C_{16}H_{25}O_7^+$ $[M+H]^+$ 329.1595, found 329.1604
IR (neat): $\nu_{max}$ $(cm^{-1})$ = 3306 (br, OH), 2947 (s, CH), 2829 (m, CH), 1726 (s, C=O), 1342 (m), 1242 (s)

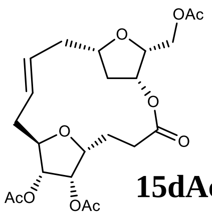


**15dAc**

**15dAc** was prepared according to general procedure A (2.07 mg, 79%).

**Analytical Data for 15dAc:**

$R_f$ = 0.43 (1:1 hexanes/EtOAc)

HRMS (ESI): Anal. Calcd. for $C_{22}H_{34}NO_{10}^+$ $[M+NH_4]^+$ 472.2177, found 472.2157

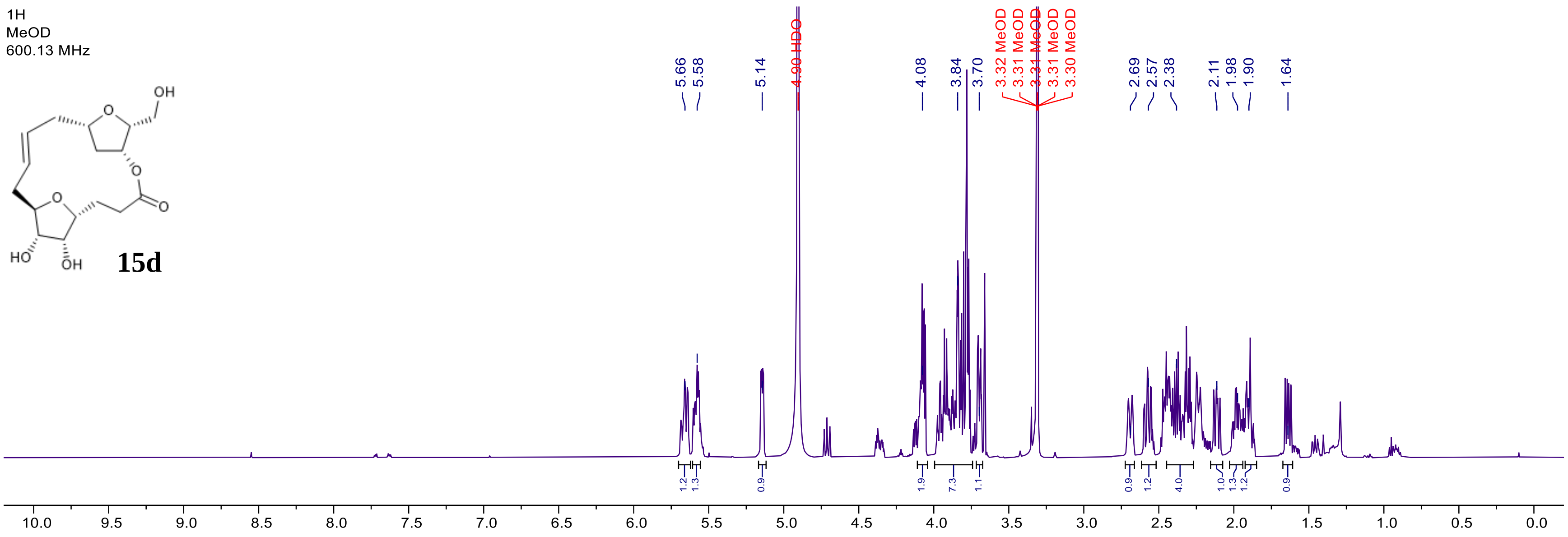
1H
MeOD
600.13 MHz
15d
5.66
5.58
5.14
4.90 HDO
4.08
3.84
3.70
3.32 MeOD
3.31 MeOD
3.31 MeOD
3.31 MeOD
3.30 MeOD
2.69
2.57
2.38
2.11
1.98
1.90
1.64
10.0 9.5 9.0 8.5 8.0 7.5 7.0 6.5 6.0 5.5 5.0 4.5 4.0 3.5 3.0 2.5 2.0 1.5 1.0 0.5 0.0

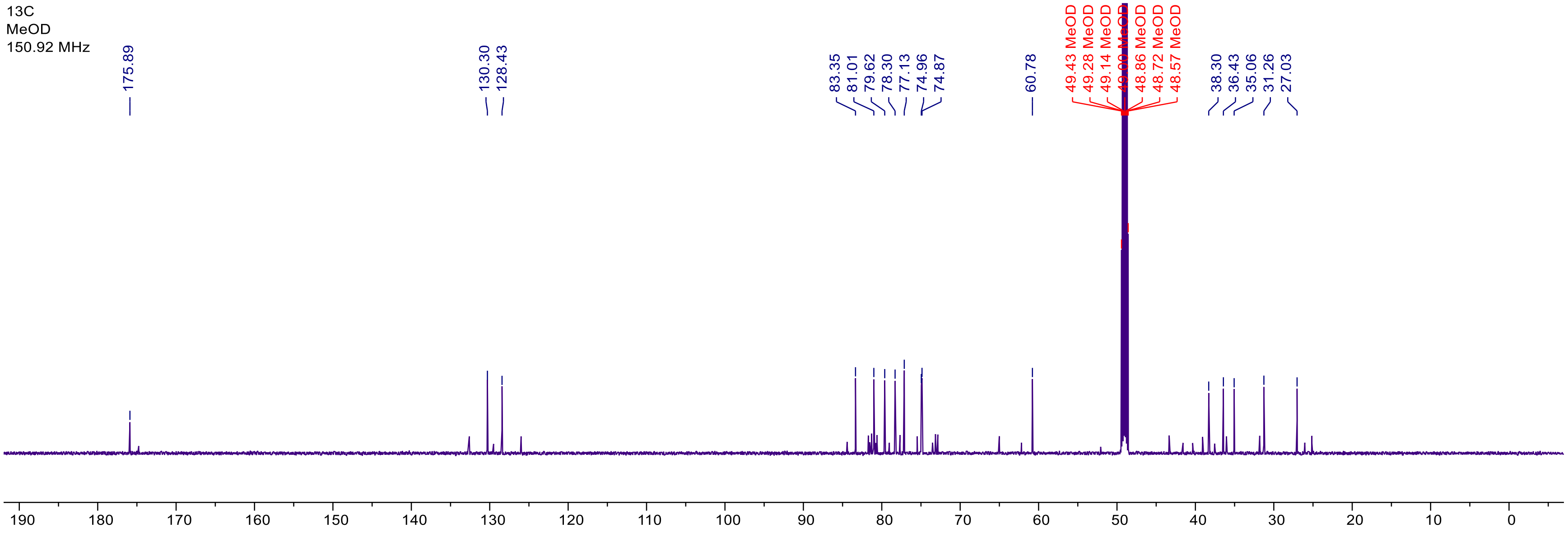
13C
MeOD
150.92 MHz
175.89
130.30
128.43
83.35
81.01
79.62
78.30
77.13
74.96
74.87
60.78
49.43 MeOD
49.28 MeOD
49.14 MeOD
49.00 MeOD
48.86 MeOD
48.72 MeOD
48.57 MeOD
38.30
36.43
35.06
31.26
27.03
190 180 170 160 150 140 130 120 110 100 90 80 70 60 50 40 30 20 10 0

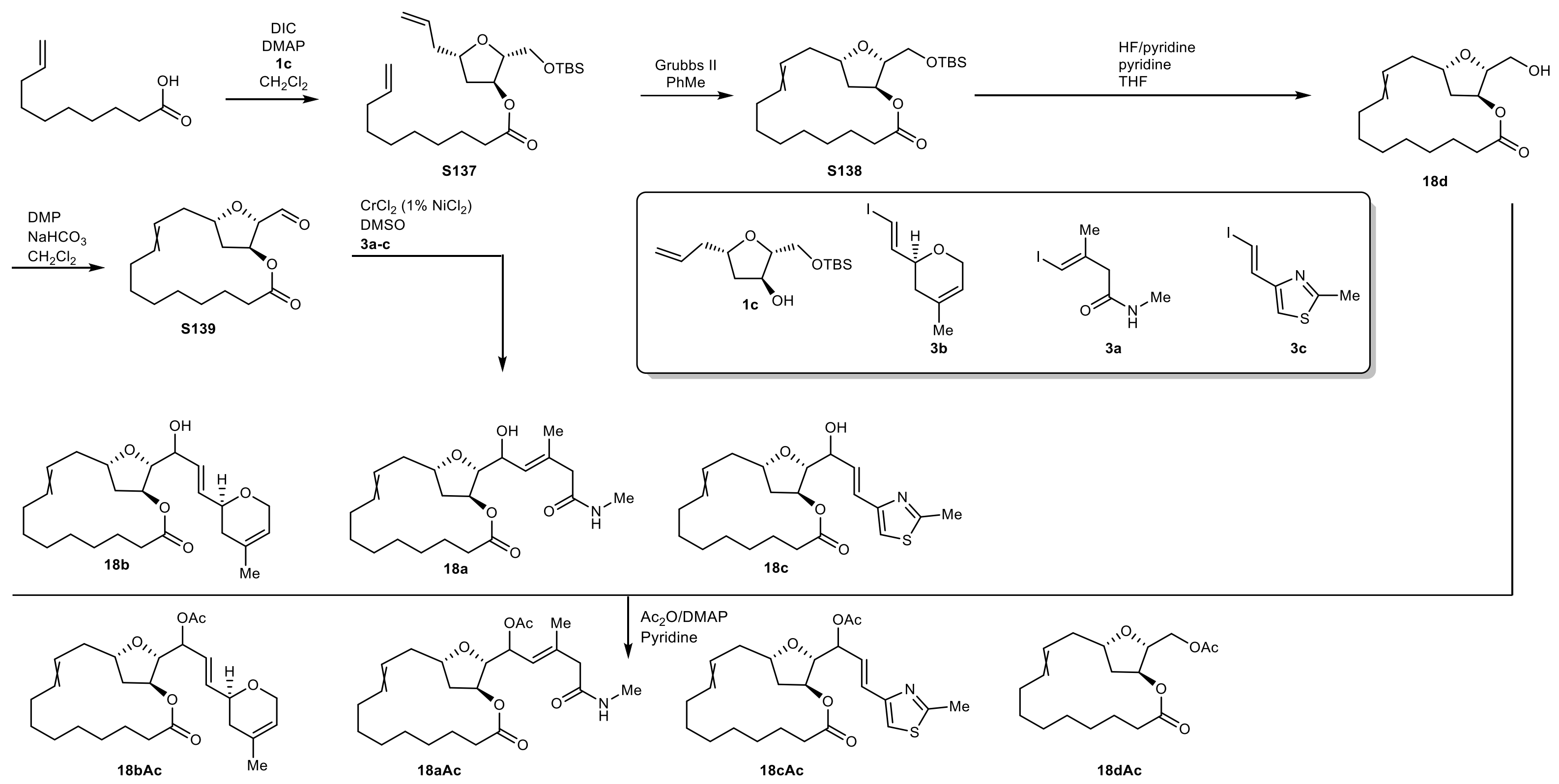


**Supplementary Fig. 48 | Synthesis of pMLs 18a-d and 18aAc-dAc.**
Abbreviations: DIC = N,N'-diisopropylcarbodiimide, DMAP = 4-dimethylaminopyridine, TBS = tert-butyldimethylsilyl, Grubbs II = Dichloro[1,3-bis(2,4,6-trimethylphenyl)-2-imidazolidinylidene](benzylidene)(tricyclohexylphosphine)ruthenium(II), THF = tetrahydrofuran, DMP = Dess-Martin periodinane, DMSO = dimethylsulfoxide.

Note: Reaction performed in capped vessel under ambient atmosphere.
To a rt stirred solution of dec-9-enoic acid (153 mg, 0.60 mmol, 1.5 eq.), alcohol **1c** (163 mg, 0.60 mmol, 1.0 eq.), and DMAP (29 mg, 0.24 mmol, 0.4 eq.) dissolved in anhydrous $CH_2Cl_2$ (6 mL), was added DIC (0.214 mL, 174 mg, 1.38 mmol, 2.3 eq.). The reaction mixture was stirred for 1 h at rt, after which time starting material was consumed as monitored by TLC analysis. The reaction mixture was quenched with $H_2O$ (20 mL) and the aqueous layer was extracted with $CH_2Cl_2$ (3x 40 mL). The combined organic layers were dried ($Na_2SO_4$), filtered, and solvent was removed in vacuo. The crude product was purified via flash column chromatography (9:1 hexanes/$Et_2O$). Appropriate fractions were pooled, and solvent was removed in vacuo to yield **S137** (246 mg, 96%) as a colorless oil.

To a stirred, heated (60 °C) solution of **S137** (246 mg, 0.58 mmol, 1.0 eq.) in anhydrous toluene (deoxygenated via 5x freeze-pump-thaw cycles) (230 mL) was added dropwise Grubbs 2nd generation catalyst (98.3 mg, 0.116 mmol, 0.20 eq.) in anhydrous, deoxygenated toluene (9.3 mL). After 30 minutes stirring at 60 °C, the reaction was cooled to 0 °C and quenched with potassium 2-isocyanoacetate (100 mg) in MeOH (50 mL). The mixture was warmed to rt and stirred for 1 h, then run through a plug of silica gel ($Et_2O$) and the solvent was removed in vacuo. The crude product was purified via flash column chromatography (19:1 to 9:1 hexanes/$Et_2O$). Appropriate fractions were pooled, and solvent was removed in vacuo to yield **S138** (86 mg, 37%) as a yellow oil and a mixture

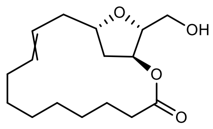


**18d**

Note: Reaction performed in capped vessel under ambient atmosphere.
To a plastic vessel containing **S138** (133 mg, 0.34 mmol, 1.0 eq.) dissolved in anhydrous THF (3.1 mL) and pyridine (1.3 mL) was added 70% HF/pyridine (1.3 mL, 51 mmol HF, 150 eq.). After 20 min stirring at rt, the reaction was quenched with saturated $NaHCO_3$ (80 mL) and after effervescence had subsided, the aqueous layer extracted with $CH_2Cl_2$ (3 x 50 mL). The combined organic layers were dried ($Na_2SO_4$), filtered, and the solvent was removed in vacuo. The crude product was purified via flash column chromatography (37:3 hexanes/acetone). Appropriate fractions were pooled to yield **18d** (94 mg, 65%) as a white solid and a mixture of (*E*) and (*Z*) isomers (3:1).

**Analytical Data for 18d:**

$R_f$ = 0.19 (17:3 hexanes/acetone)

Major diastereomer

$^{1}H$ NMR (601 MHz, $CDCl_3$) δ 5.50 (ddd, *J* = 15.2, 9.9, 5.2 Hz, 1H), 5.35 (dt, *J* = 15.5, 7.6 Hz, 1H), 5.20 (d, *J* = 4.7 Hz, 1H), 4.12 – 4.06 (m, 1H), 3.86 (tt, *J* = 11.2, 3.8 Hz, 1H), 3.72 (dd, *J* = 11.6, 4.2 Hz, 2H), 3.60 (dd, *J* = 11.8, 4.8 Hz, 1H), 2.63 (ddd, *J* = 12.6, 8.7, 4.3 Hz, 1H), 2.46 (ddd, *J* = 12.9, 6.5, 4.4 Hz, 1H), 2.43 – 2.17 (m, 3H), 2.17 – 2.10 (m, 2H), 2.04 (dd, *J* = 13.5, 3.4 Hz, 1H), 1.99 – 1.88 (m, 1H), 1.71 – 1.59 (m, 3H), 1.39 – 1.31 (m, 4H), 1.16 – 1.06 (m, 2H).

Major diastereomer

$^{13}C$ NMR (151 MHz, $CDCl_3$) δ 173.37, 134.84, 126.03, 83.51, 83.37, 81.42, 63.58, 39.91, 35.73, 35.41, 32.88, 29.08, 28.56, 28.51, 28.36, 25.91.

HRMS (ESI): Anal. Calcd. for $C_{16}H_{27}O_4^+$ $[M+H]^+$ 283.1904, found 283.1915

IR (neat): $\nu_{max}$ $(cm^{-1})$ = 3438 (br, OH), 2928 (s, CH), 2857 (s, CH), 1729 (s, C=O), 1439 (m), 1332 (w), 1244 (s)

**18dAc**

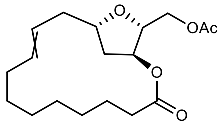


**18dAc** was prepared according to general procedure A (1.41 mg, 70%).

**Analytical Data for 18dAc:**

$R_f$ = 0.94 (1:1 hexanes/EtOAc)

HRMS (ESI): Anal. Calcd. for $C_{18}H_{32}NO_5^+$ $[M+NH_4]^+$ 342.2275, found 342.2304

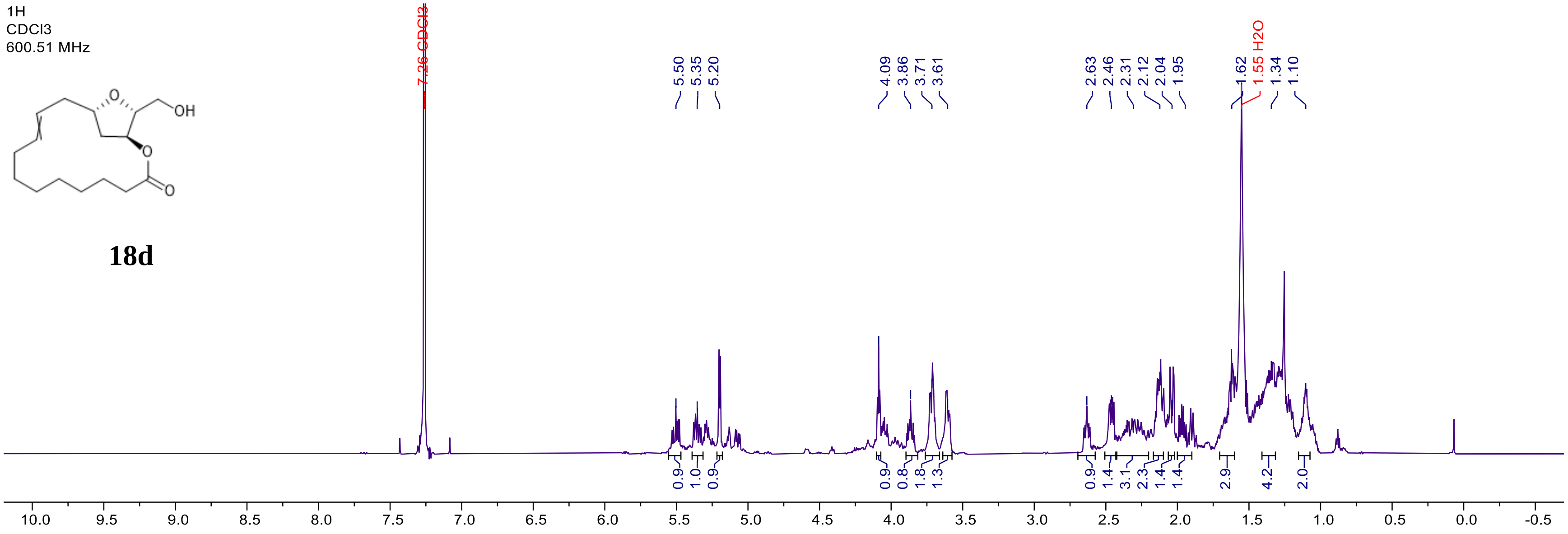
1H
CDCl3
600.51 MHz
7.26 CDCl3
5.50
5.35
5.20
4.09
3.86
3.71
3.61
2.63
2.46
2.31
2.12
2.04
1.95
1.62
1.55 H2O
1.34
1.10
0.9
1.0
0.9
0.9
0.8
1.8
1.3
0.9
1.4
3.1
2.3
1.4
1.4
2.9
4.2
2.0
10.0 9.5 9.0 8.5 8.0 7.5 7.0 6.5 6.0 5.5 5.0 4.5 4.0 3.5 3.0 2.5 2.0 1.5 1.0 0.5 0.0 -0.5

**18d**

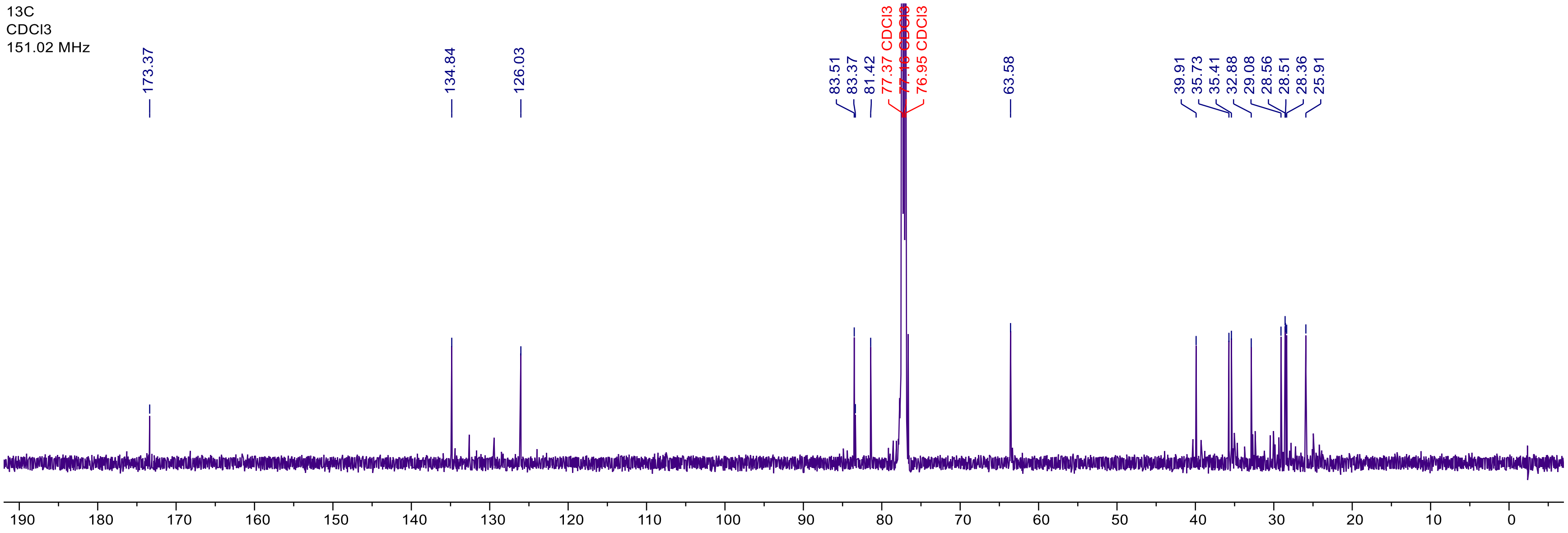
13C
CDCl3
151.02 MHz
173.37
134.84
126.03
83.51
83.37
81.42
77.37 CDCl3
77.16 CDCl3
76.95 CDCl3
63.58
39.91
35.73
35.41
32.88
29.08
28.56
28.51
28.36
25.91
190 180 170 160 150 140 130 120 110 100 90 80 70 60 50 40 30 20 10 0

To a stirred solution of **18d** (75 mg, 0.265 mmol, 1.0 eq.) in anhydrous $CH_2Cl_2$ (2.7 mL) was added $NaHCO_3$ (67 mg, 0.80 mol, 3.0 eq.) followed by Dess-Martin Periodinane (147 mg, 0.35 mmol, 1.3 eq.). The mixture was stirred at rt for 3 h and quenched with a solution of 1:1:1 $H_2O$:saturated aqueous $NaHCO_3$:saturated aqueous $Na_2S_2O_3$ (3 mL). The aqueous layer was extracted with $CH_2Cl_2$ (3x 25 mL) and the combined organic layers were dried ($Na_2SO_4$), filtered, and the solvent was removed in vacuo to yield crude aldehyde **S139** (76 mg) as a colorless oil, which was split into 4 equal portions and used immediately for the next step.

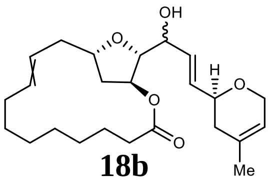

**18b**

To a solution of aldehyde **S139** (19 mg, 0.066 mmol, 1.0 eq.) in anhydrous DMSO (deoxygenated via 5x freeze-pump-thaw cycles) (1.0 mL) was added $CrCl_2$ doped with 1% $NiCl_2$ (w/w) (82 mg, 0.66 mmol, 10 eq.). **3b** (38 mg, 0.15 mmol, 2.2 eq.) was added in deoxygenated DMSO (0.5 mL) and the mixture was stirred for 21 h after which the reaction was quenched with 1:1 $H_2O$-brine (15 mL). The aqueous layer was extracted with $Et_2O$ (10x 15 mL) and the combined organic layers were dried ($MgSO_4$), filtered, and the solvent was removed in vacuo. Residual DMSO was evaporated with air. The crude product was purified via flash column chromatography (9:1 hexanes/acetone). Appropriate fractions were pooled, and solvent was removed in vacuo to yield **18b** (14 mg, 51%) as a colorless oil and a mixture of diastereomers (3:1 *E*/*Z* at the macrocyclic olefin, epimers at the alcohol carbon).

**Analytical Data for 18b:**

$R_f$ = 0.31 (4:1 hexanes/acetone)

Major (*E*)-diastereomer
$^1$H NMR (601 MHz, $CDCl_3$) δ 5.95 – 5.85 (m, 1H), 5.85 – 5.73 (m, 1H), 5.57 – 5.46 (m, 1H), 5.41 (d, *J* = 3.4 Hz, 1H), 5.39 – 5.31 (m, 1H), 5.26 – 5.16 (m, 1H), 4.32 – 4.24 (m, 1H), 4.20 – 4.13 (m, 2H), 4.08 – 4.03 (m, 1H), 4.01 – 3.98 (m, 1H), 3.84 (dtt, *J* = 15.1, 11.2, 3.8 Hz, 1H), 2.64 (ddt, *J* = 12.6, 8.9, 4.7 Hz, 1H), 2.45 (dtd, *J* = 12.9, 6.4, 3.3 Hz, 1H), 2.14 – 1.91 (m, 8H), 1.70 (s, 3H), 1.58 – 1.53 (m, 3H), 1.43 – 1.37 (m, 2H), 1.36 – 1.27 (m, 4H), 1.11 – 1.04 (m, 2H).

Major (*E*)-diastereomer
$^{13}$C NMR (151 MHz, $CDCl_3$) δ 173.28, 134.79, 133.52, 131.56, 127.81, 126.06, 119.80, 86.13, 81.24, 73.11, 71.89, 65.77, 40.09, 35.75, 35.64, 35.48, 32.86, 29.06, 28.54, 28.50, 28.35, 25.96, 23.08.

HRMS (ESI): Anal. Calcd. for $C_{24}H_{37}O_5^+$ $[M+H]^+$ 405.2636, found 405.2631
IR (neat): $\nu_{max}$ $(cm^{-1})$ = 3432 (br, OH), 2928 (s, CH), 2856 (s, CH), 1730 (s, C=O), 1440 (m), 1380 (m), 1242 (s)

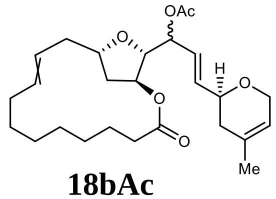


**18bAc**

**18bAc** was prepared according to general procedure A (1.15 mg, 80%).

**Analytical Data for 18bAc:**

$R_f$ = 0.42 and 0.83 (1:1 hexanes/EtOAc)

HRMS (ESI): Anal. Calcd. for $C_{26}H_{42}NO_6^+$ $[M+NH_4]^+$ 464.3007, found 464.2991

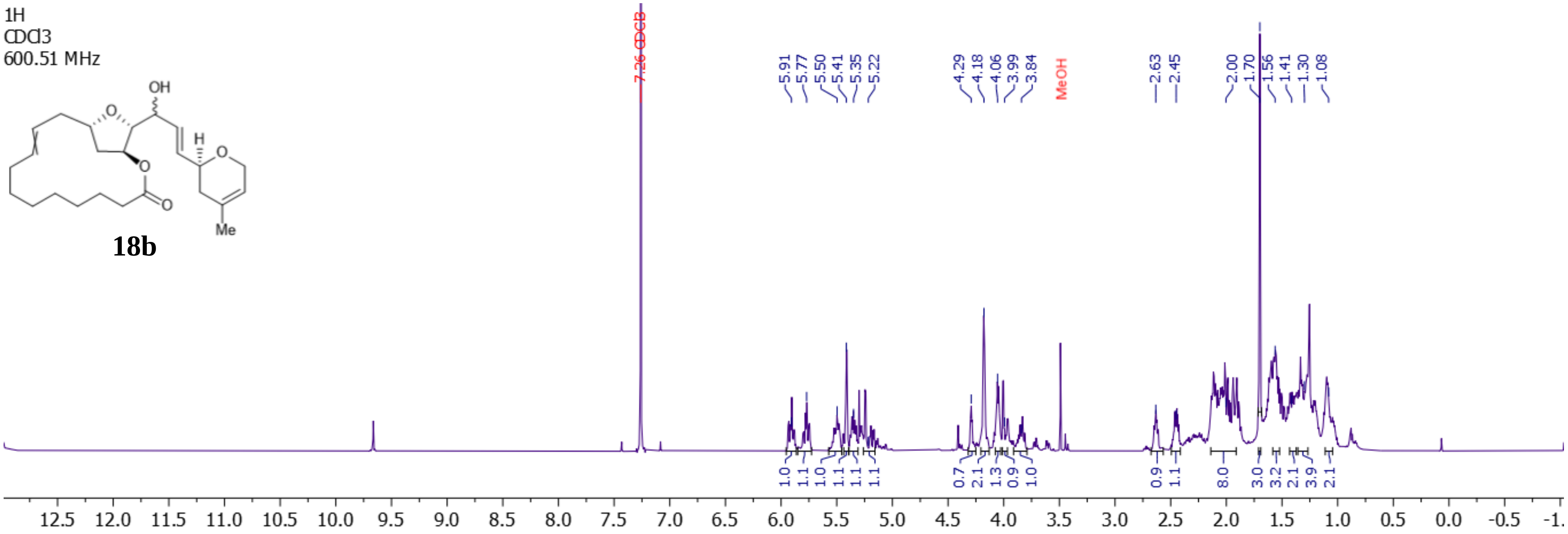
1H
CDCl3
600.51 MHz
OH
O
H
O
O
O
Me
18b
7.26 CDCl3
5.91
5.77
5.50
5.41
5.35
5.22
4.29
4.18
4.06
3.99
3.84
MeOH
2.63
2.45
2.00
1.70
1.56
1.41
1.30
1.08
1.0
1.1
1.0
1.1
1.1
1.1
0.7
2.1
1.3
0.9
1.0
0.9
1.1
8.0
3.0
3.2
2.1
3.9
2.1
12.5 12.0 11.5 11.0 10.5 10.0 9.5 9.0 8.5 8.0 7.5 7.0 6.5 6.0 5.5 5.0 4.5 4.0 3.5 3.0 2.5 2.0 1.5 1.0 0.5 0.0 -0.5 -1.

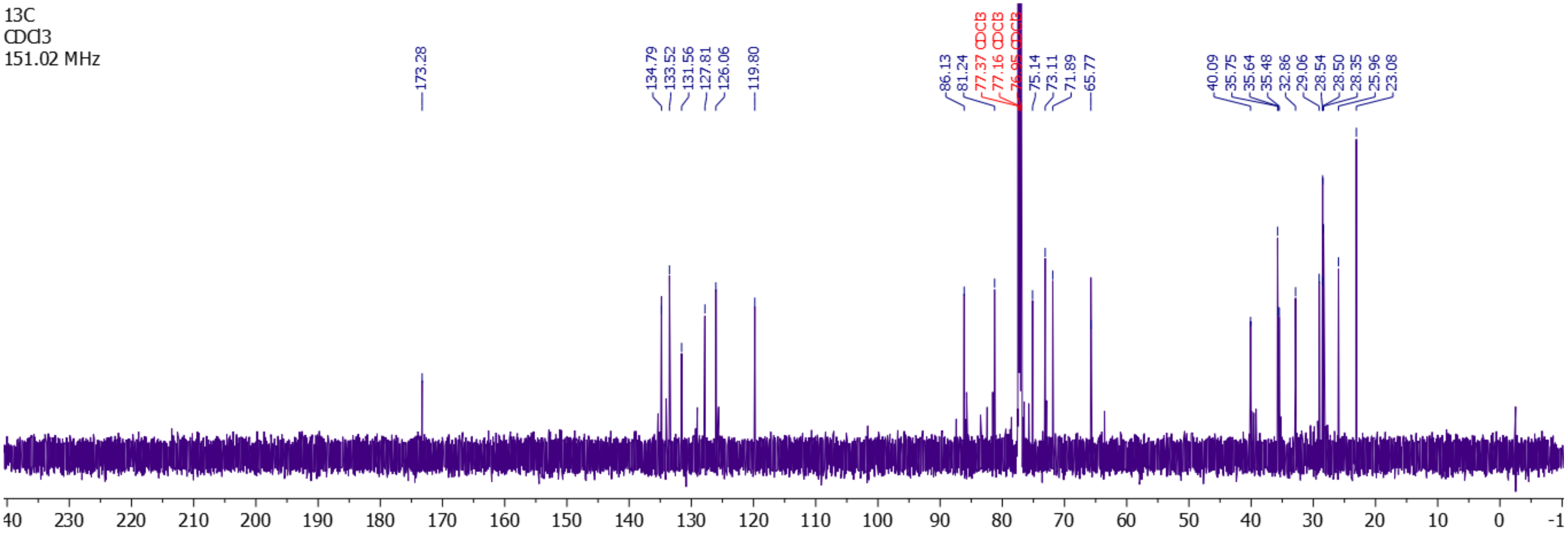
13C
CDCl3
151.02 MHz
173.28
134.79
133.52
131.56
127.81
126.06
119.80
86.13
81.24
77.37 CDCl3
77.16 CDCl3
76.95 CDCl3
75.14
73.11
71.89
65.77
40.09
35.75
35.64
35.48
32.86
29.06
28.54
28.50
28.35
25.96
23.08
40 230 220 210 200 190 180 170 160 150 140 130 120 110 100 90 80 70 60 50 40 30 20 10 0 -1

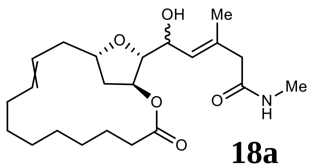

**18a**

To a solution of aldehyde **S139** (19 mg, 0.066 mmol, 1.0 eq.) in anhydrous DMSO (deoxygenated via 5x freeze-pump-thaw cycles) (1.0 mL) was added $CrCl_2$ doped with 1% $NiCl_2$ (w/w) (82 mg, 0.66 mmol, 10 eq.). **3a** (48 mg, 0.20 mmol, 3.0 eq.) was added in deoxygenated DMSO (0.75 mL) and the mixture was stirred for 21 h after which the reaction was quenched with 1:1 $H_2O$-brine (15 mL). The aqueous layer was extracted with $Et_2O$ (10x 15 mL) and the combined organic layers were dried ($MgSO_4$), filtered, and the solvent was removed in vacuo. Residual DMSO was evaporated with air. The crude product was purified via flash column chromatography (4:1 to 13:7 hexanes/acetone). Appropriate fractions were pooled, and solvent was removed in vacuo to yield **18a** (7.3 mg, 28%) as a colorless oil and a mixture of diastereomers (3:1 *E*/*Z* at the macrocyclic olefin, 1:1 epimers at the alcohol carbon).

**Analytical Data for 18a:**

$R_f$ = 0.30 (1:1 hexanes/acetone)

For the following reported NMR peaks: Several resonances in the $^1$H NMR spectrum integrated for 0.5 protons and are unique to a diastereomer. In these cases an integral value of "0.5 H" is assigned.

<u>(*E*)-diastereomers</u>
$^1$H NMR (601 MHz, $CDCl_3$) δ 6.08 (s, 0.5H), 5.75 (s, 0.5H), 5.54 – 5.46 (m, 1H), 5.41 – 5.30 (m, 2.5H), 5.11 (d, *J* = 4.6 Hz, 0.5H), 4.43 – 4.36 (m, 0.5H), 4.28 – 4.19 (m, 0.5H), 4.05 – 3.81 (m, 2H), 3.03 – 2.91 (m, 2H), 2.80 – 2.76 (m, 3H), 2.65 (ddd, *J* = 12.6, 8.5, 4.4 Hz, 0.5H), 2.58 (ddd, *J* = 12.6, 8.8, 4.5 Hz, 0.5H), 2.51 – 2.43 (m, 1H), 2.39 – 2.27 (m, 1H), 2.22 – 2.10 (m, 2H), 2.05 (td, *J* = 13.1, 3.6 Hz, 2H), 2.00 – 1.85 (m, 3H), 1.79 (dd, *J* = 8.3, 1.4 Hz, 1.5H), 1.74 (dd, *J* = 5.0, 1.5 Hz, 1.5H), 1.65 – 1.57 (m, 2H), 1.55 – 1.47 (m, 1H), 1.42 – 1.23 (m, 5H), 1.12 – 1.05 (m, 1H).

<u>(*E*)-diastereomers</u>
$^{13}$C NMR (151 MHz, $CDCl_3$) δ 173.27, 173.20, 170.93, 170.71, 136.44, 135.79, 135.06, 134.96, 128.68, 128.44, 125.96, 125.75, 86.41, 86.20, 81.84, 81.19, 75.96, 75.61, 69.45, 68.86, 47.97, 47.79, 39.56, 39.54, 35.68, 35.67, 35.37, 35.31, 32.91, 32.87, 29.09, 28.66, 28.60, 28.56, 28.53, 28.35, 28.33, 26.64, 26.61, 25.82, 25.74, 17.57, 17.32.

HRMS (ESI): Anal. Calcd. for $C_{22}H_{36}NO_5^+$ $[M+H]^+$ 394.2588, found 394.2592

IR (neat): $\nu_{max}$ ($cm^{-1}$) = 3328 (br, OH), 2928 (s, CH), 2857 (s, CH), 1729 (s, C=O), 1661 (s, C=C), 1644 (s, C=C), 1550 (m), 1440 (m), 1412 (w), 1333 (w), 1244 (s)

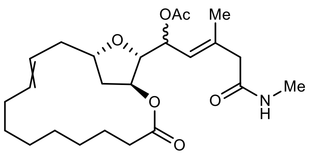


**18aAc**

**18aAc** was prepared according to general procedure A (1.26 mg, 71%).

**Analytical Data for 18aAc:**

$R_f$ = 0.44 (EtOAc)

HRMS (ESI): Anal. Calcd. for $C_{24}H_{38}NO_6^+$ $[M+H]^+$ 436.2694, found 436.2687

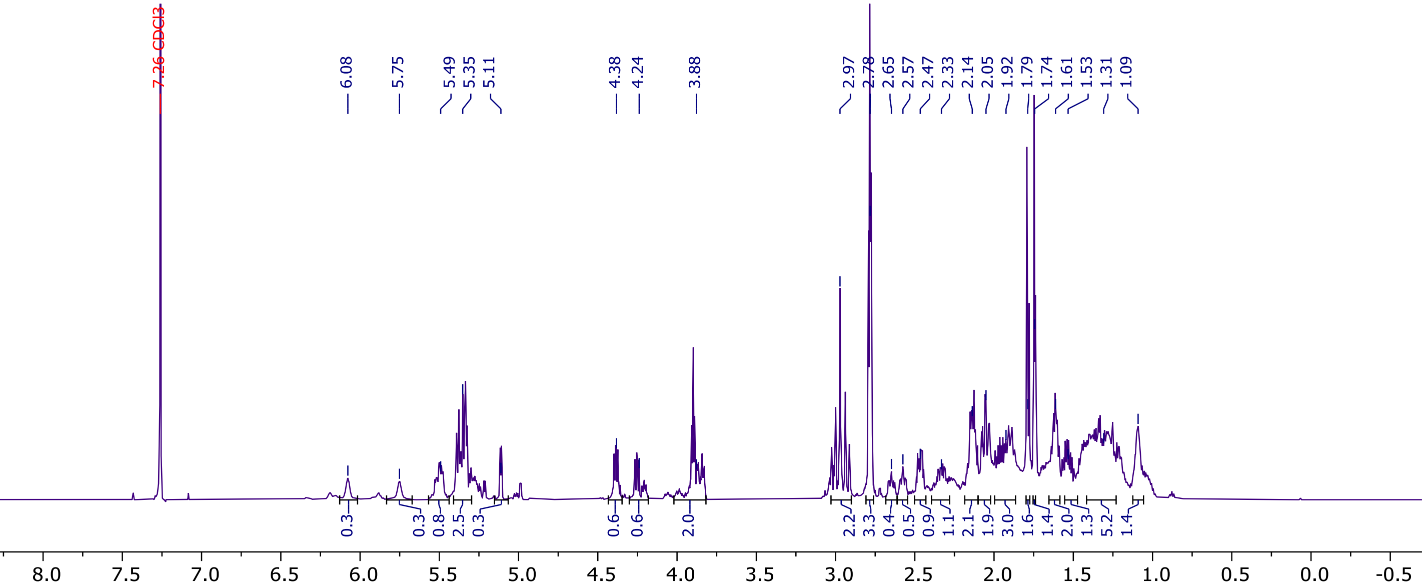
1H
CDCl3
600.51 MHz
7.26 CDCl3
6.08
5.75
5.49
5.35
5.11
4.38
4.24
3.88
2.97
2.78
2.65
2.57
2.47
2.33
2.14
2.05
1.92
1.79
1.74
1.61
1.53
1.31
1.09
0.3
0.3
0.8
2.5
0.3
0.6
0.6
2.0
2.2
3.3
0.4
0.5
0.5
0.9
1.1
2.1
1.9
3.0
1.6
1.4
2.0
1.3
5.2
1.4
10.0
9.5
9.0
8.5
8.0
7.5
7.0
6.5
6.0
5.5
5.0
4.5
4.0
3.5
3.0
2.5
2.0
1.5
1.0
0.5
0.0
-0.5

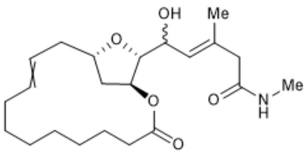
OH
Me
O
O
O
O
N
H
Me

**18a**

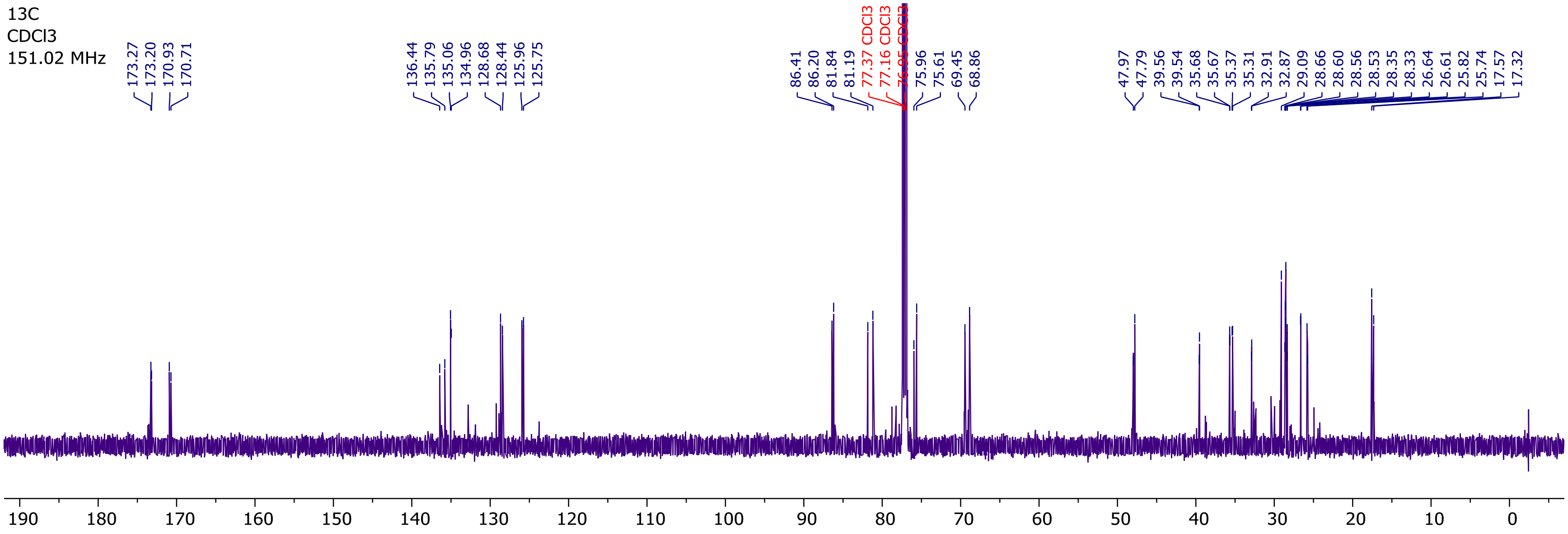
13C
CDCl3
151.02 MHz
173.27
173.20
170.93
170.71
136.44
135.79
135.06
134.96
128.68
128.44
125.96
125.75
86.41
86.20
81.84
81.19
77.37 CDCl3
77.16 CDCl3
76.95 CDCl3
75.96
75.61
69.45
68.86
47.97
47.79
39.56
39.54
35.68
35.67
35.37
35.31
32.91
32.87
29.09
28.66
28.60
28.56
28.53
28.35
28.33
26.64
26.61
25.82
25.74
17.57
17.32
190
180
170
160
150
140
130
120
110
100
90
80
70
60
50
40
30
20
10
0

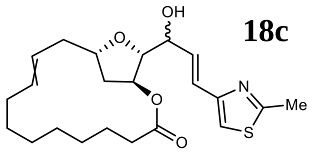

**18c** To a solution of aldehyde **S139** (19 mg, 0.066 mmol, 1.0 eq.) in anhydrous DMSO (deoxygenated via 5x freeze-pump-thaw cycles) (1.0 mL) was added $CrCl_2$ doped with 1% $NiCl_2$ (w/w) (82 mg, 0.66 mmol, 10 eq.). **3c** (50 mg, 0.20 mmol, 3.0 eq.) was added in deoxygenated DMSO (1.0 mL) and the mixture was stirred for 21 h after which the reaction was quenched with 1:1 $H_2O$-brine (15 mL). The aqueous layer was extracted with $Et_2O$ (10x 15 mL) and the combined organic layers were dried ($MgSO_4$), filtered, and the solvent was removed in vacuo. Residual DMSO was evaporated with air. The crude product was purified via flash column chromatography (4:1 to 13:7 hexanes/acetone). Appropriate fractions were pooled, and solvent was removed in vacuo to yield **18c** (13 mg, 48%) as a colorless oil and a mixture of diastereomers (3:1 *E*/*Z* at the macrocyclic olefin, 7:3 epimers at the alcohol carbon).

**Analytical Data for 18c:**

$R_f$ = 0.17 (49:1 $CH_2Cl_2$/MeOH)

For the following reported NMR peaks: Several resonances in the $^1$H NMR spectrum integrated for 0.7 and 0.3 protons and are unique to both the diastereomers. In these cases an integral value of "0.7 H" and "0.3 H" is assigned.

$^1$H NMR (600 MHz, $CDCl_3$) δ 6.93 (s, 0.7H), 6.93 (s, 0.3H), 6.74 – 6.63 (m, 1H), 6.58 – 6.48 (m, 1H), 5.50 (ddt, *J* = 15.1, 10.0, 5.2 Hz, 0.5H), 5.37 – 5.31 (m, 0.5H), 5.31 – 5.08 (m, 2H), 4.51 – 4.45 (m, 0.3H), 4.19 (qd, *J* = 6.1, 2.9 Hz, 0.7H), 4.13 – 3.91 (m, 1.4H), 3.90 – 3.81 (m, 0.6H), 2.71 – 2.69 (m, 3H), 2.65 (ddd, *J* = 12.5, 8.7, 4.3 Hz, 1H), 2.48 – 2.42 (m, 1H), 2.37 – 2.16 (m, 2H), 2.13 – 1.95 (m, 4H), 1.94 – 1.84 (m, 1H), 1.63 – 1.54 (m, 3H), 1.40 – 1.31 (m, 2H), 1.29 – 1.26 (m, 1H), 1.22 – 1.16 (m, 1H), 1.14 – 1.01 (m, 2H).

Major diastereomer
$^{13}$C NMR (151 MHz, $CDCl_3$) δ 173.37, 166.24, 152.86, 134.88, 130.06, 125.97, 125.47, 115.69, 85.71, 81.53, 76.48, 73.06, 39.51, 35.83, 35.42, 32.89, 29.08, 28.50, 28.49, 28.33, 25.93, 19.46.

HRMS (ESI): Anal. Calcd. for $C_{22}H_{32}NO_4S^+$ $[M+H]^+$ 406.2047, found 406.2041
IR (neat): $\nu_{max}$ $(cm^{-1})$ = 3388 (br, OH), 2928 (s, CH), 2856 (m, CH), 1726 (s, C=O), 1438 (m), 1373 (w), 1330 (w), 1243 (s)

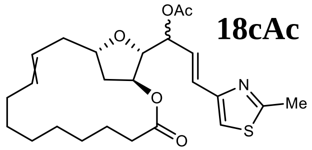


**18cAc**

**18cAc** was prepared according to general procedure A (1.25 mg, 82%).

**Analytical Data for 18cAc:**

$R_f$ = 0.84 (1:1 hexanes/EtOAc)

Major diastereomer

$^1$H NMR (600 MHz, $C_6D_6$) δ 7.08 – 7.00 (m, 1H), 6.67 (d, *J* = 15.5 Hz, 1H), 6.34 – 6.29 (m, 1H), 5.82 (q, *J* = 6.8, 6.3 Hz, 1H), 5.54 (d, *J* = 4.5 Hz, 1H), 5.16 (td, *J* = 9.8, 4.9 Hz, 1H), 5.11 – 5.00 (m, 1H), 4.44 (d, *J* = 5.9 Hz, 1H), 3.85 (tq, *J* = 10.9, 3.3, 2.8 Hz, 1H), 2.68 (ddt, *J* = 12.7, 8.5, 4.3 Hz, 1H), 2.19 (s, 3H), 2.14 – 2.09 (m, 1H), 2.01 (ddd, *J* = 16.5, 12.4, 4.8 Hz, 2H), 1.97 – 1.92 (m, 1H), 1.90 – 1.84 (m, 1H), 1.65 (s, 3H), 1.54 (ddt, *J* = 14.5, 7.9, 3.8 Hz, 1H), 1.47 – 1.33 (m, 2H), 1.30 – 1.21 (m, 2H), 1.20 – 1.12 (m, 2H), 1.07 – 0.98 (m, 3H), 0.95 – 0.87 (m, 1H), 0.87 – 0.80 (m, 1H).

Major diastereomer

$^{13}$C NMR (151 MHz, $C_6D_6$) δ 172.06, 169.13, 165.38, 152.82, 133.91, 127.46, 126.54, 126.36, 115.93, 84.06, 81.19, 76.05, 73.86, 39.45, 35.79, 35.00, 32.79, 28.98, 28.28, 28.26, 28.15, 25.71, 20.34, 18.57.

$^{13}$C NMR (151 MHz, $C_6D_6$) δ 172.40, 169.43, 165.68, 153.12, 134.21, 127.76, 126.84, 126.66, 116.23, 84.36, 81.49, 76.35, 74.16, 39.75, 36.09, 35.30, 33.09, 30.23, 28.58, 28.56, 28.45, 26.01, 20.64, 18.87.

IR (neat): $\nu_{max}$ $(cm^{-1})$ = 2929 (s, CH), 2856 (w, CH), 1739 (s, C=O), 1438 (w), 1371 (m), 1232 (s)

HRMS (ESI): Anal. Calcd. for $C_{24}H_{34}NO_5S^+$ $[M+H]^+$ 448.2152, found 448.2136

1H
CDCl3
600.13 MHz

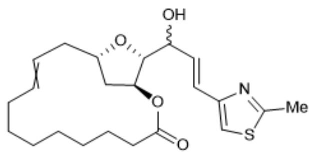


**18c**

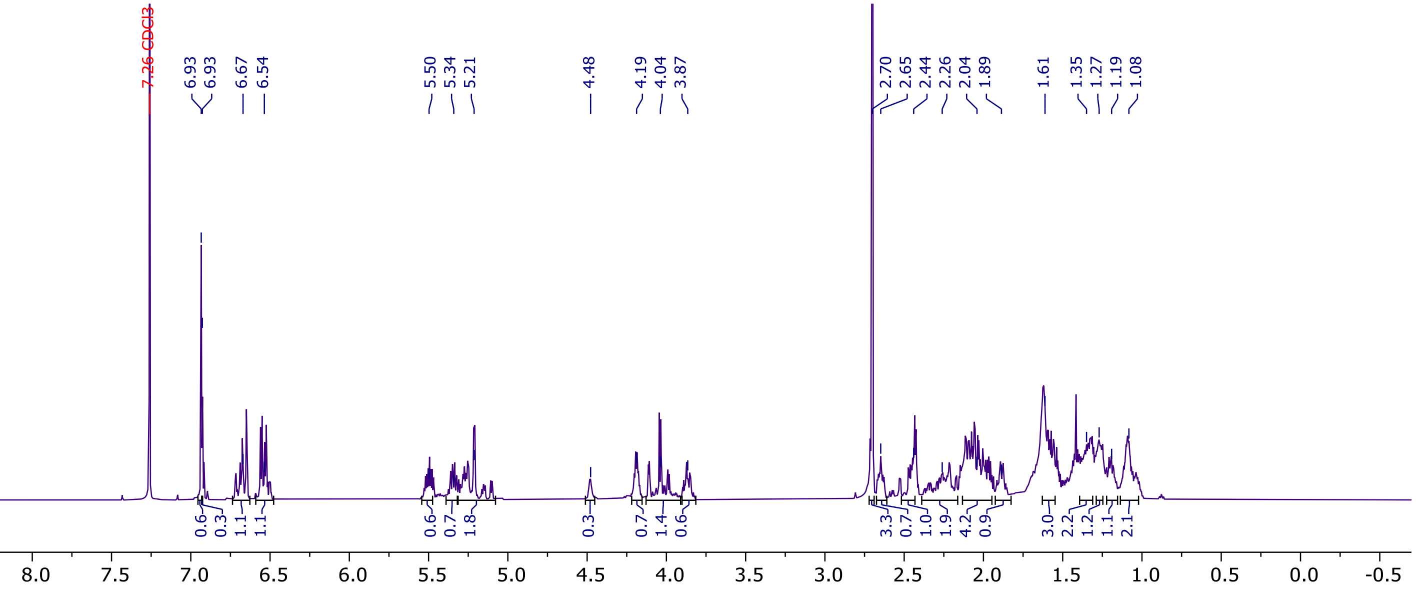


13C
CDCl3
150.92 MHz

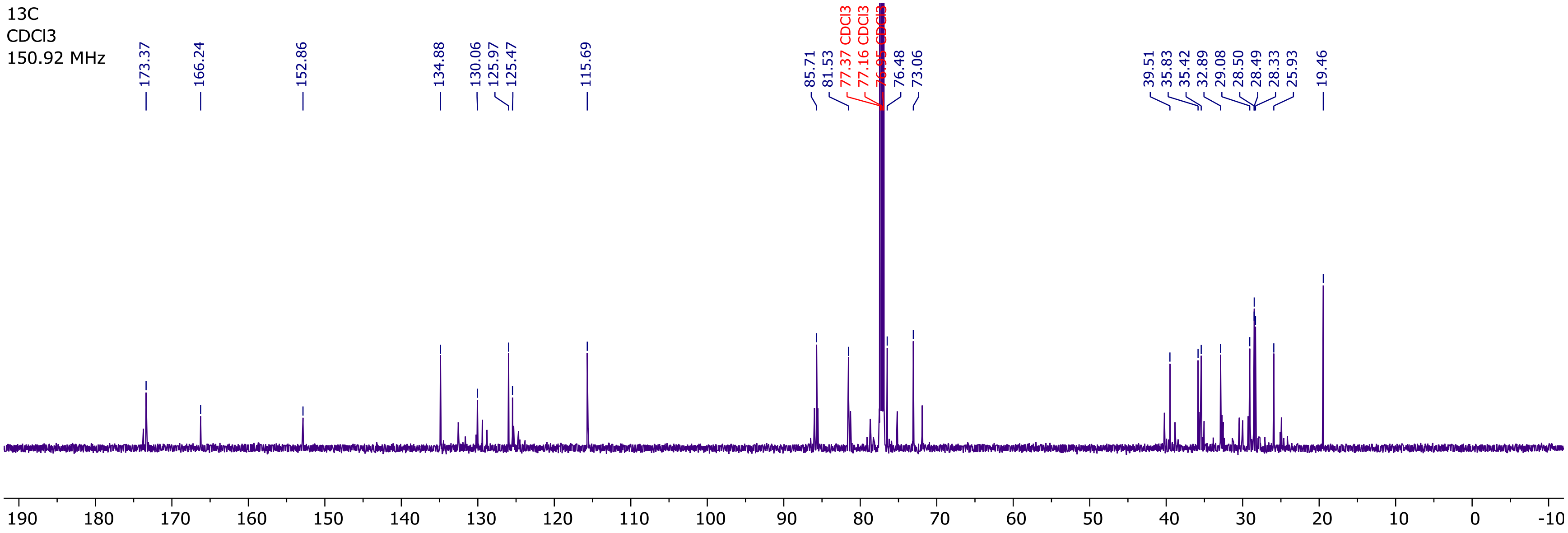

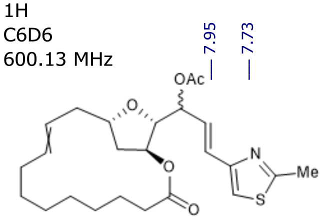
OAc
O
O
O
N
S
Me

**18cAc**

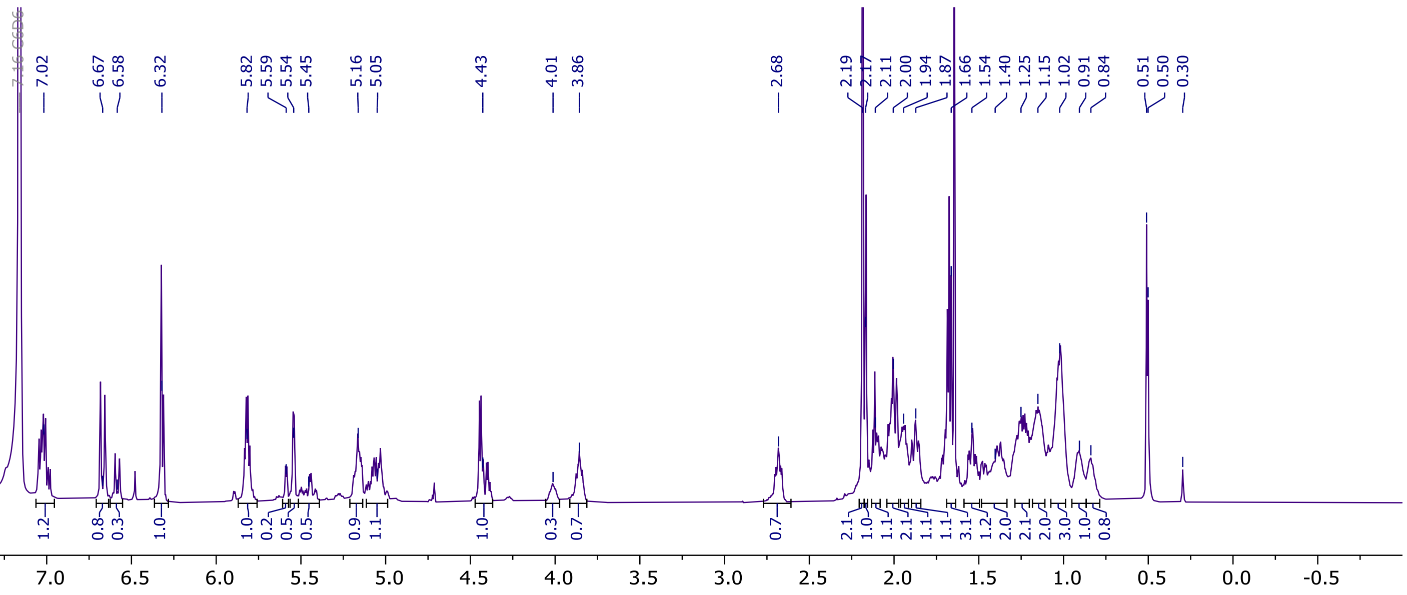
1H
C6D6
600.13 MHz
7.95
7.73
7.16 C6D6
7.02
6.67
6.58
6.32
5.82
5.59
5.54
5.45
5.16
5.05
4.43
4.01
3.86
2.68
2.19
2.17
2.11
2.00
1.94
1.87
1.66
1.54
1.40
1.25
1.15
1.02
0.91
0.84
0.51
0.50
0.30
1.2
0.8
0.3
1.0
1.0
0.2
0.5
0.5
0.9
1.1
1.0
0.3
0.7
0.7
2.1
1.0
1.1
2.1
1.1
1.1
3.1
1.2
2.0
2.1
2.0
3.0
1.0
0.8
9.0
8.5
8.0
7.5
7.0
6.5
6.0
5.5
5.0
4.5
4.0
3.5
3.0
2.5
2.0
1.5
1.0
0.5
0.0
-0.5

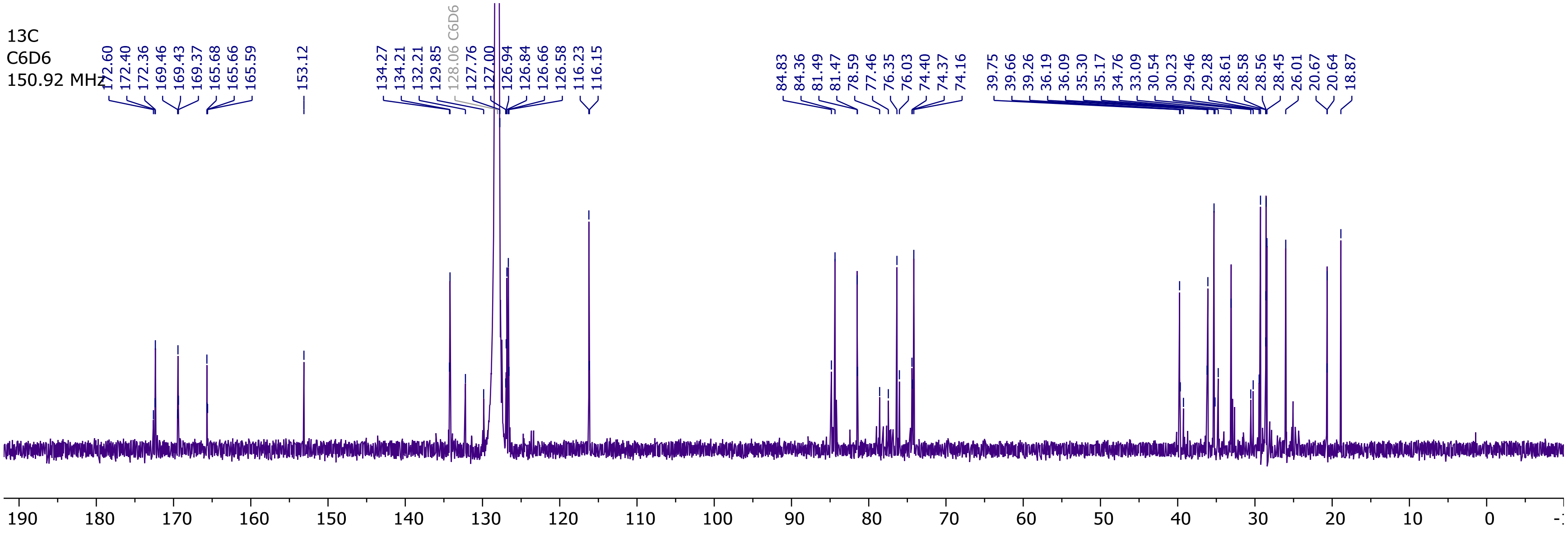
13C
C6D6
150.92 MHz
172.60
172.40
172.36
169.46
169.43
169.37
165.68
165.66
165.59
153.12
134.27
134.21
132.21
129.85
128.06 C6D6
127.76
127.00
126.94
126.84
126.66
126.58
116.23
116.15
84.83
84.36
81.49
81.47
78.59
77.46
76.35
76.03
74.40
74.37
74.16
39.75
39.66
39.26
36.19
36.09
35.30
35.17
34.76
33.09
30.54
30.23
29.46
29.28
28.61
28.58
28.56
28.45
26.01
20.67
20.64
18.87
190
180
170
160
150
140
130
120
110
100
90
80
70
60
50
40
30
20
10
0

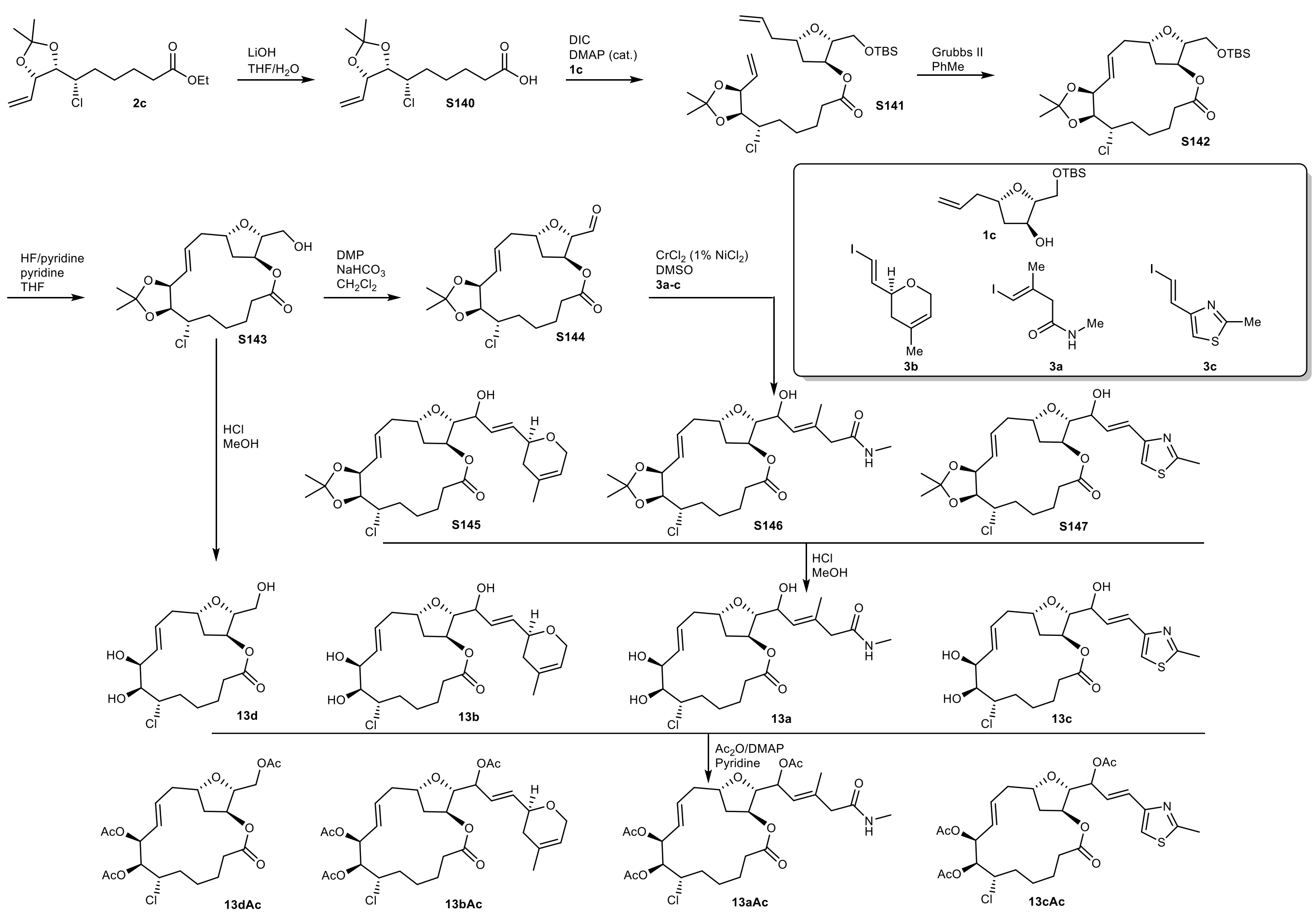


**Supplementary Fig. 49 | Synthesis of pMLs 13a-d and 13aAc-dAc.**

Abbreviations: THF = tetrahydrofuran, DIC = N,N'-diisopropylcarbodiimide, DMAP = 4-dimethylaminopyridine, TBS = tert-butyldimethylsilyl, Grubbs II = Dichloro[1,3-bis(2,4,6-trimethylphenyl)-2-imidazolidinylidene](benzylidene)(tricyclohexylphosphine)ruthenium(II), DMP = Dess-Martin periodinane, DMSO = dimethylsulfoxide.

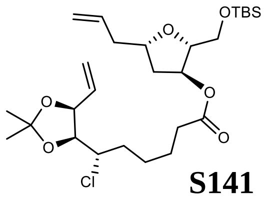
**S141**

To a rt, stirred solution of **2c** (274.3 mg, 0.90 mmol, 1.0 eq.) in THF (4.5 mL) and $H_2O$ (2.7 mL) was added LiOH•$H_2O$ (113.3 mg, 2.70 mmol, 3.0 eq.). To aid the solubility of LiOH, further $H_2O$ was added (2.7 mL). The reaction mixture was vigorously stirred for 3 h 20 min. After this time, starting material was consumed as monitored by TLC analysis. The reaction mixture was diluted with $H_2O$ (15 mL) and $Et_2O$ (15 mL), transferred to a separatory funnel, and the mixture adjusted to *ca.* pH 4-5 with HCl (0.4M in $H_2O$). The organic layer was then separated, and the aqueous layer was extracted with $Et_2O$ (2x 15 mL), EtOAc (3x 10 mL) and $CH_2Cl_2$ (3x 20 mL). The combined organic layers were dried ($MgSO_4$), filtered, and solvent was removed in vacuo to yield crude acid **S140** (223 mg) as a colorless oil which was used immediately in the next step without further purification.

To a rt, stirred solution of acid **S140** (223 mg, 0.806 mmol, 1.34 eq.), alcohol **1c** (163.5 mg, 0.60 mmol, 1.0 eq.), and 4-dimethylaminopyridine (29.3 mg, 0.24 mmol, 0.40 eq.) in $CH_2Cl_2$ (6 mL) was added N,N'-diisopropylcarbodiimide (0.18 mL, 150 mg, 1.1 mmol, 1.8 eq.). The reaction mixture was allowed to stir for 16 h 15 min. at rt. After this time, starting material was consumed as monitored by TLC analysis. The cloudy reaction mixture was poured onto $H_2O$ (15 mL) in a separatory funnel, and the aqueous layer extracted with $CH_2Cl_2$ (2x 15 mL). The combined organic layers were dried ($Na_2SO_4$), filtered, and solvent was removed in vacuo to yield an oily residue. The residue was triturated vigorously with $Et_2O$ (3x 2 mL) and filtered. The filtrate was evaporated in vacuo to yield a colorless oil with small amounts of precipitate. The crude product was purified via flash column chromatography (9:1 to 4:1 Hexanes/$Et_2O$) to give **S141** (318.7 mg, 98%) as a colorless oil.

**Analytical Data for S141:**

$R_f$ = 0.72 (3:1 Hexanes/EtOAc)

$[\alpha]_D^{20}$ = +12° (c = 0.50, $CDCl_3$)

$^1H$ NMR (600 MHz, $CDCl_3$) δ 6.00 (ddd, *J* = 17.1, 10.2, 8.7 Hz, 1H), 5.82 (ddt, *J* = 17.2, 10.2, 7.0 Hz, 1H), 5.39 – 5.31 (m, 2H), 5.18 (dt, *J* = 6.2, 1.3 Hz, 1H), 5.10 (dq, *J* = 17.1, 1.6 Hz, 1H), 5.06 (ddt, *J* = 10.2, 2.2, 1.1 Hz, 1H), 4.56 (dd, *J* = 8.6, 6.3 Hz, 1H), 4.19 (t, *J* = 6.4 Hz, 1H), 4.14 – 4.07 (m, 1H), 3.90 (td, *J* = 3.7, 1.7 Hz, 1H), 3.79 (ddd, *J* = 8.8, 6.3, 4.4 Hz, 1H), 3.76 (dd, *J* = 10.9, 3.4 Hz, 1H), 3.63 (dd, *J* = 10.9, 4.1 Hz, 1H), 2.44 – 2.36 (m, 1H), 2.36 – 2.25 (m, 3H), 1.94 (ddd, *J* = 13.5, 4.8, 1.0 Hz, 1H), 1.79 (ddd, *J* = 13.5, 10.8, 6.0 Hz, 1H), 1.75 – 1.67 (m, 2H), 1.67 – 1.57 (m, 3H), 1.56 (s, 3H), 1.48 – 1.41 (m, 1H), 1.40 (s, 3H), 0.89 (s, 9H), 0.05 (d, *J* = 1.8 Hz, 6H).

$^{13}C$ NMR (151 MHz, $CDCl_3$) δ 173.17, 134.61, 133.37, 120.59, 117.28, 109.38, 85.28, 81.12, 79.72, 78.43, 63.85, 60.62, 39.55, 38.36, 34.40, 34.27, 27.44, 26.03, 25.80, 25.60, 24.33, 18.43, -5.18, -5.36.

HRMS (ESI): Anal. Calcd. for $C_{27}H_{51}NO_6SiCl$ $[M+NH_4]^+$ 548.3169, found 548.3169

IR (neat): $\nu_{max}$ $(cm^{-1})$ = 3078 (w, C=*CH*), 2931 (m, CH), 2858 (m, CH), 1734 (s, C=O), 1465 (m), 1433 (m), 1254 (s)

1H
CDCl3
600.13 MHz

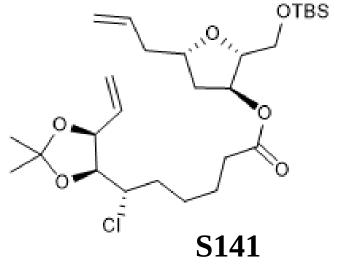


**S141**

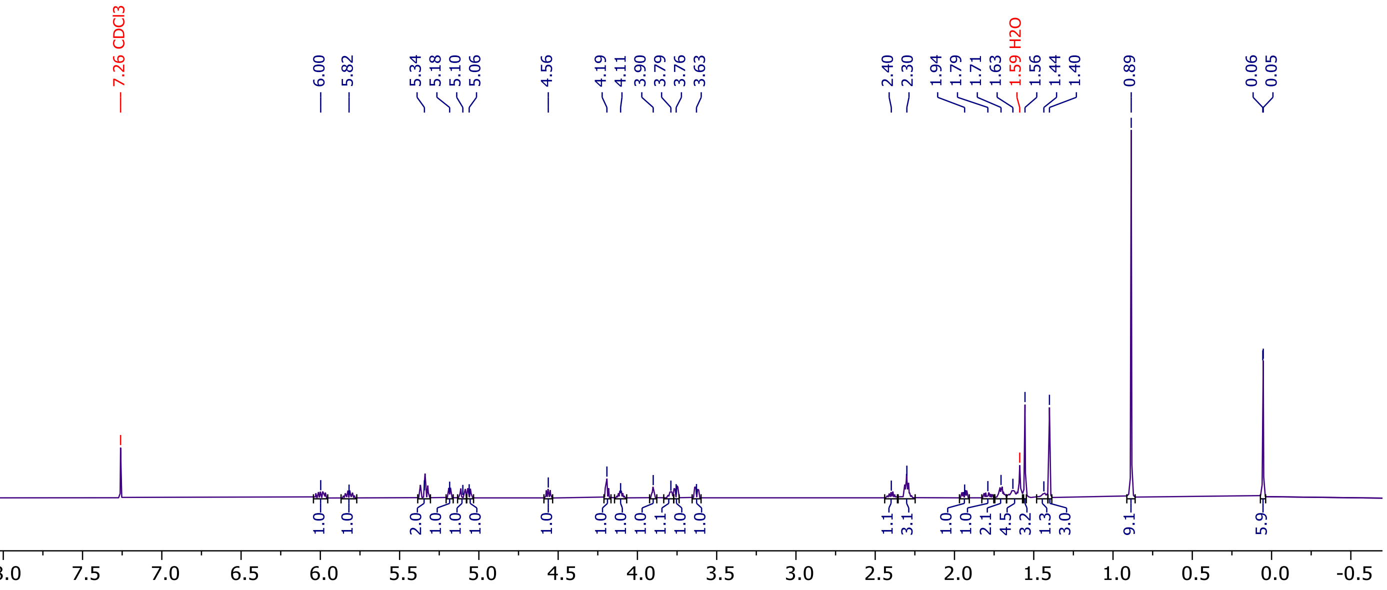


13C
CDCl3
150.92 MHz

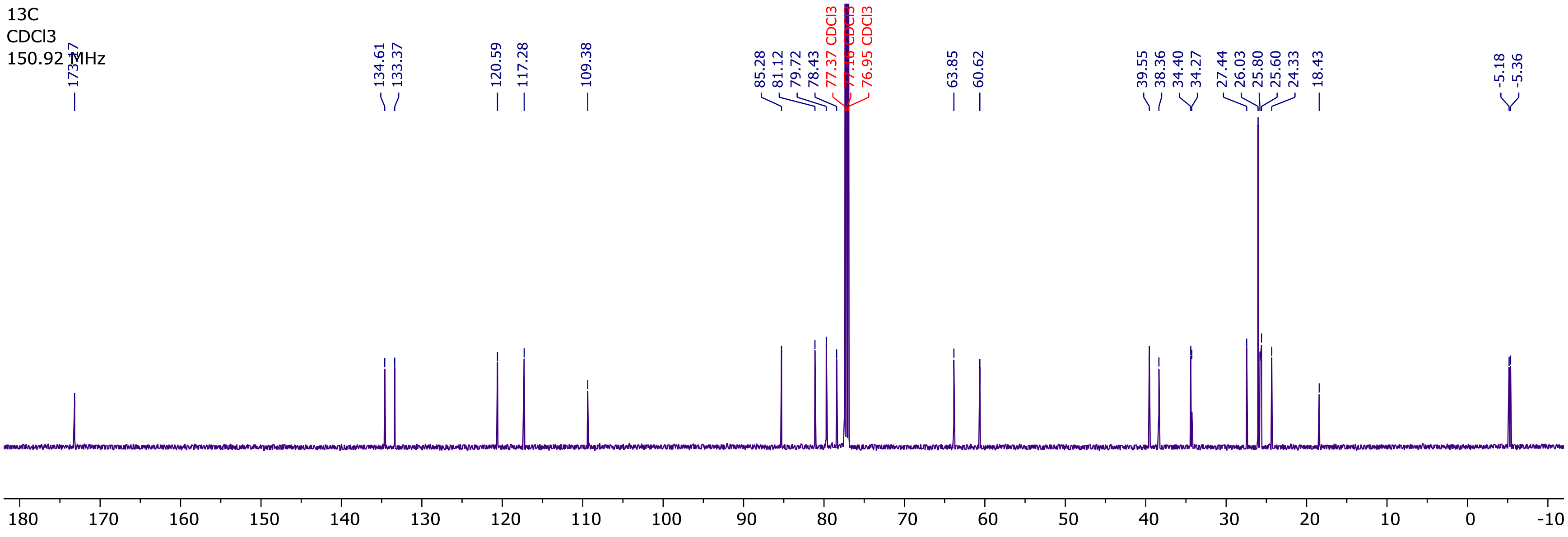

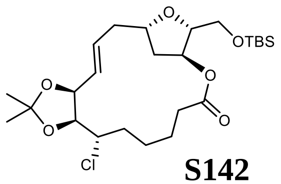
**S142**

To a warm (60 °C), stirred solution of **S141** (286 mg, 0.54 mmol, 1.0 eq.) in toluene (225 mL) that was continuously being sparged with $N_2$(g) was added Grubbs second generation catalyst (91.7 mg, 0.0108 mmol, 0.20 eq.) in toluene (3 mL). The reaction was allowed to proceed under continuous nitrogen sparge for 2 h. After this time, starting material was consumed as monitored by TLC analysis, the reaction mixture was cooled to rt and quenched by addition of potassium 2-isocyanoacetate (120 mg, 0.98 mmol, 1.82 eq.) in MeOH (5 mL). The reaction mixture was stirred for a further 4 h 20 min., after which time the red solution changed to a pale-yellow color. The reaction mixture was concentrated in vacuo to give a crude solid deposited on the walls of the evaporation flask. The walls were scraped with a spatula and the solid was triturated with $Et_2O$ (5x 5 mL) and filtered through a short plug of silica gel eluting with $Et_2O$. The filtrate was concentrated in vacuo and the crude product was purified via flash column chromatography (3:1 Hexanes/$Et_2O$). Appropriate fractions were pooled, and solvent was removed in vacuo to yield the products **S142** (190 mg, 70%) as a white amorphous solid.

**Analytical Data for S142:**

$R_f$ = 0.38 (7:3 Hexanes/$Et_2O$)

$[\alpha]_D^{20}$ = +104° (c = 0.44, $CDCl_3$)

$^1$H NMR (600 MHz, $CDCl_3$) δ 5.67 – 5.59 (m, 2H), 5.27 (d, *J* = 4.3 Hz, 1H), 4.45 (q, *J* = 5.3 Hz, 1H), 4.14 (dd, *J* = 9.3, 5.6 Hz, 1H), 4.03 (t, *J* = 4.4 Hz, 1H), 3.76 – 3.66 (m, 3H), 3.56 (dd, *J* = 10.9, 5.1 Hz, 1H), 2.66 (ddt, *J* = 9.1, 6.6, 4.0 Hz, 1H), 2.32 – 2.21 (m, 2H), 2.09 (td, *J* = 11.7, 3.1 Hz, 1H), 1.85 – 1.78 (m, 1H), 1.78 (dd, *J* = 13.1, 3.3 Hz, 1H), 1.75 – 1.67 (m, 1H), 1.65 – 1.51 (m, 6H), 1.51 – 1.41 (m, 2H), 1.39 (s, 3H), 0.88 (s, 9H), 0.05 (s, 6H).

$^{13}$C NMR (151 MHz, $CDCl_3$) δ 174.19, 131.71, 129.90, 109.32, 83.28, 81.22, 80.77, 79.20, 63.60, 60.29, 39.42, 36.09, 34.80, 34.53, 28.38, 26.00, 25.88, 25.81, 25.32, 18.42, -5.17, -5.33.

HRMS (ESI): Anal. Calcd. for $C_{25}H_{47}NO_6Si^+$ $[M+NH_4]^+$ 520.2856, found 520.2858

IR (neat): $\nu_{max}$ ($cm^{-1}$) = 2952 (m, CH), 2931 (m, CH), 2859 (m, CH), 1730 (s, C=O), 1465 (m), 1372 (m), 1335 (m), 1254 (s)

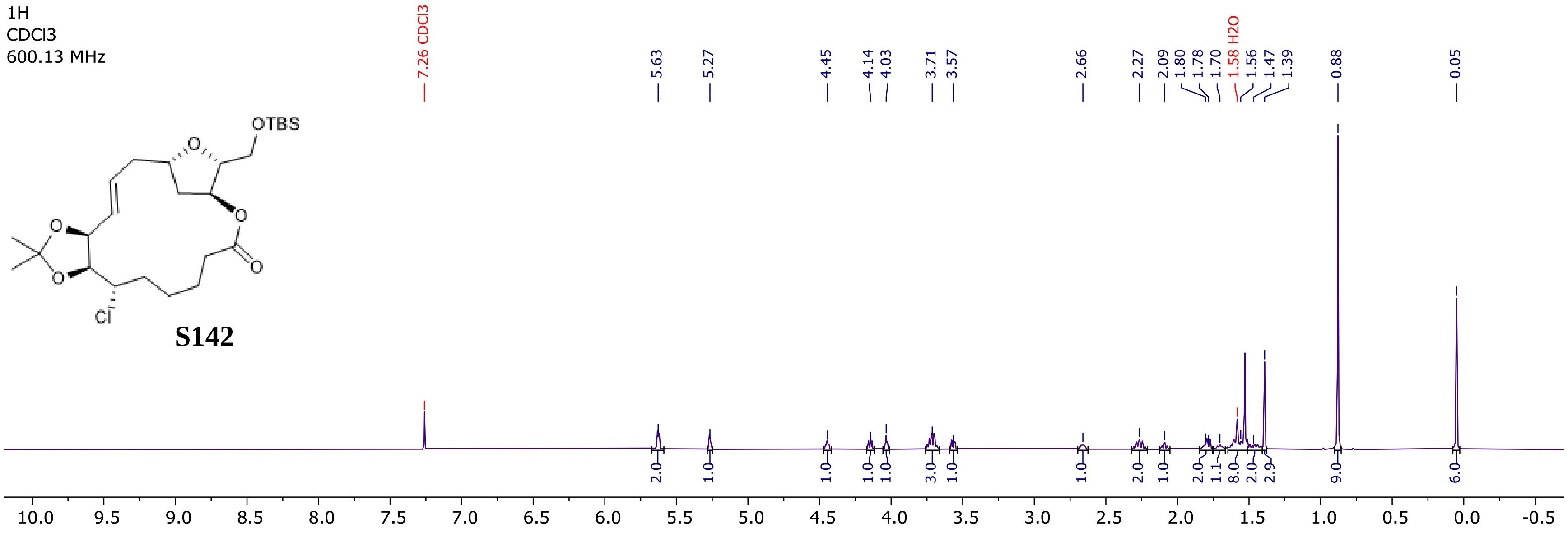
OTBS
O
O
O
O
O
Cl
S142
1H
CDCl3
600.13 MHz
7.26 CDCl3
5.63
5.27
4.45
4.14
4.03
3.71
3.57
2.66
2.27
2.09
1.80
1.78
1.70
1.58 H2O
1.56
1.47
1.39
0.88
0.05
2.0
1.0
1.0
1.0
1.0
3.0
1.0
1.0
2.0
1.0
2.0
1.1
8.0
2.0
2.9
9.0
6.0
10.0
9.5
9.0
8.5
8.0
7.5
7.0
6.5
6.0
5.5
5.0
4.5
4.0
3.5
3.0
2.5
2.0
1.5
1.0
0.5
0.0
-0.5

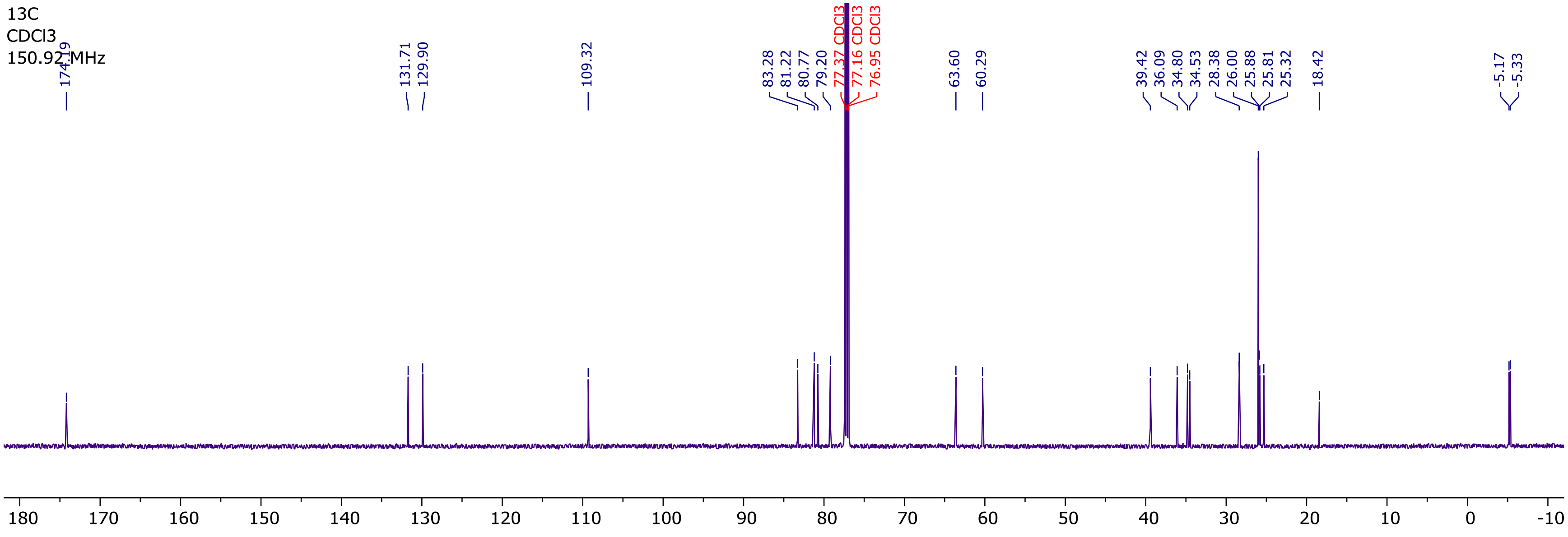
13C
CDCl3
150.92 MHz
174.19
131.71
129.90
109.32
83.28
81.22
80.77
79.20
77.37 CDCl3
77.16 CDCl3
76.95 CDCl3
63.60
60.29
39.42
36.09
34.80
34.53
28.38
26.00
25.88
25.81
25.32
18.42
-5.17
-5.33
180
170
160
150
140
130
120
110
100
90
80
70
60
50
40
30
20
10
0
-10

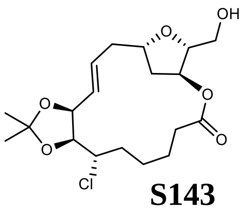

**S143**

To a rt, stirred solution of **S142** (185 mg, 0.368 mmol, 1.0 eq.) in a mixture of THF (4 mL) and pyridine (1.9 mL), was added HF-Pyridine (70% (w/w) HF, 0.48 mL, 320 mg HF, 26 mmol HF, 72 eq. HF) dropwise over 5 min. The reaction mixture was stirred at rt for 23 h. After this time, starting material was consumed as monitored by TLC analysis. The reaction mixture was quenched by slowly pipetting it onto saturated aqueous $NaHCO_3$ (30 mL) [**caution!** $CO_2$(g) evolved] over 5 min. After evolution of $CO_2$(g) had stopped, the aqueous layer was extracted with $CH_2Cl_2$ (3x 25 mL). The combined organic layers were dried ($MgSO_4$), filtered, and solvent was removed in vacuo. The crude product was purified via flash column chromatography (7:3 Hexanes/Acetone). Appropriate fractions were pooled, and solvent was removed in vacuo to yield **S143** (134 mg, 94%) as a white amorphous solid.

**Analytical Data for S143:**

$R_f$ = 0.64 (3:2 Hexanes/Acetone)

$[\alpha]_D^{20}$ = +117° (c = 0.80, $CDCl_3$)

$^1$H NMR (600 MHz, $CDCl_3$) δ 5.69 – 5.58 (m, 2H), 5.22 (dd, *J* = 4.5, 1.3 Hz, 1H), 4.45 (dd, *J* = 8.7, 5.6 Hz, 1H), 4.15 (dd, *J* = 9.3, 5.6 Hz, 1H), 4.10 (td, *J* = 4.1, 1.3 Hz, 1H), 3.77 – 3.69 (m, 3H), 3.63 (dd, *J* = 11.8, 4.4 Hz, 1H), 2.69 (ddd, *J* = 12.3, 8.5, 4.0 Hz, 1H), 2.30 (ddd, *J* = 12.6, 8.5, 2.1 Hz, 1H), 2.24 (ddd, *J* = 12.6, 10.5, 2.1 Hz, 1H), 2.16 – 2.09 (m, 1H), 1.83 (dd, *J* = 13.3, 3.3 Hz, 1H), 1.86 – 1.78 (m, 1H), 1.78 (s, 1H), 1.77 – 1.69 (m, 1H), 1.63 – 1.48 (m, 4H), 1.53 (s, 3H), 1.48 – 1.39 (m, 1H), 1.39 (s, 3H).

$^{13}$C NMR (151 MHz, $CDCl_3$) δ 174.15, 131.30, 130.18, 109.36, 83.17, 81.16, 81.00, 79.10, 77.13, 63.23, 60.26, 40.20, 35.91, 34.69, 34.57, 28.37, 25.86, 25.80, 25.44.

HRMS (ESI): Anal. Calcd. for $C_{19}H_{33}NO_6Cl^+$ $[M+NH_4]^+$ 406.1991, found 406.1993

IR (neat): $\nu_{max}$ $(cm^{-1})$ = 3463 (br, OH), 3043 (w, C=*CH*), 2991 (m, CH), 2937 (m, CH), 2871 (m, CH), 1726 (s, C=O), 1673 (m, C=C), 1447 (m), 1375 (m), 1258 (s), 1221 (s).

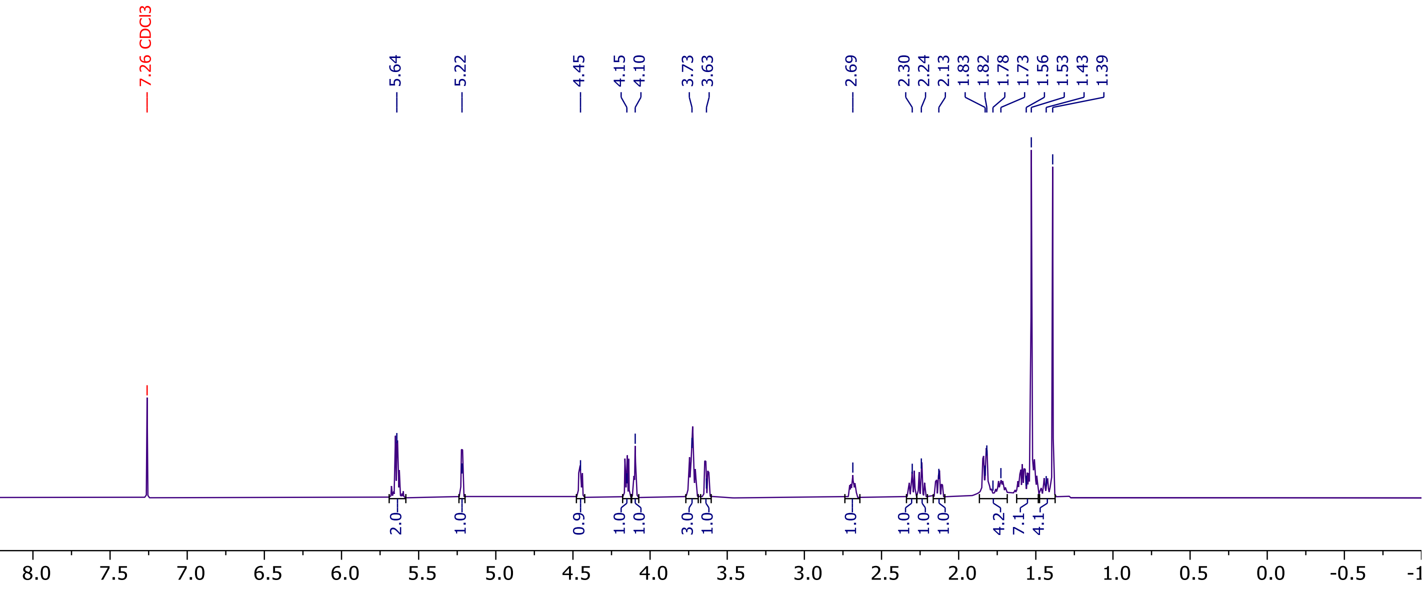
1H
CDCl3
600.13 MHz
7.26 CDCl3
5.64
5.22
4.45
4.15
4.10
3.73
3.63
2.69
2.30
2.24
2.13
1.83
1.82
1.78
1.73
1.56
1.53
1.43
1.39
2.0
1.0
0.9
1.0
1.0
3.0
1.0
1.0
1.0
1.0
1.0
4.2
7.1
4.1
10.0 9.5 9.0 8.5 8.0 7.5 7.0 6.5 6.0 5.5 5.0 4.5 4.0 3.5 3.0 2.5 2.0 1.5 1.0 0.5 0.0 -0.5

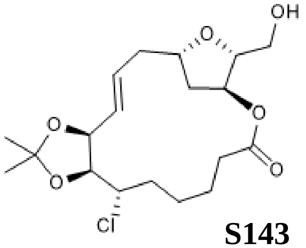
OH
O
O
O
O
O
Cl

**S143**

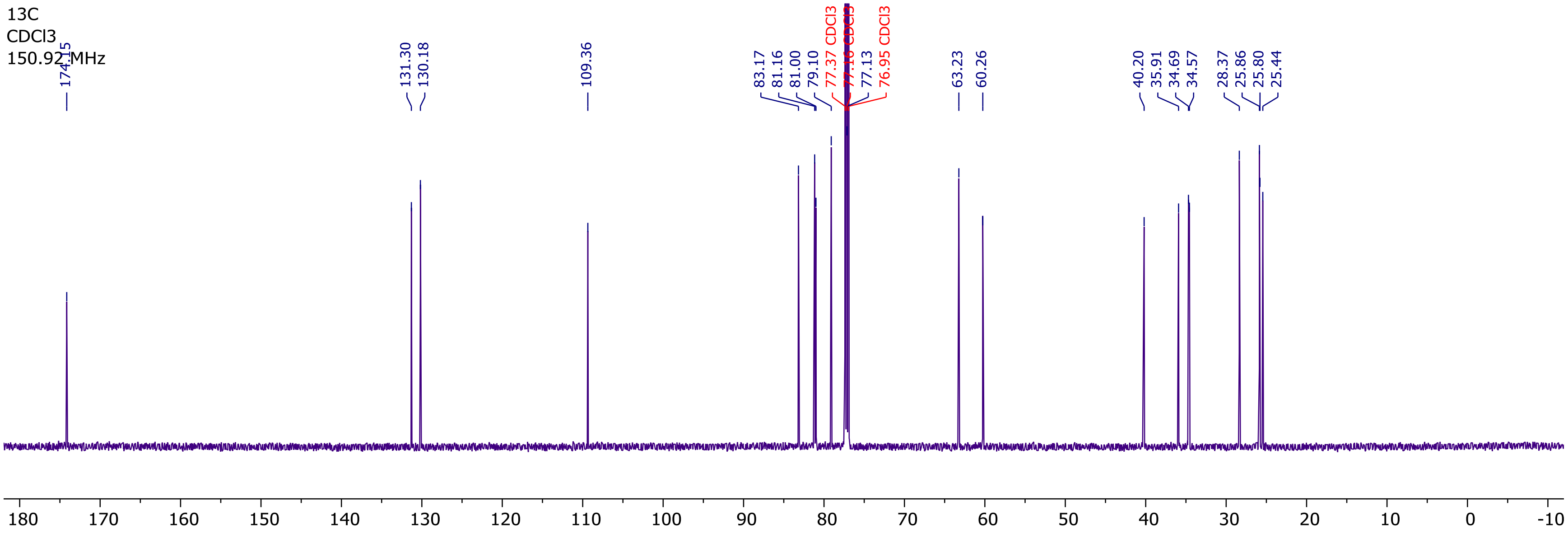
13C
CDCl3
150.92 MHz
174.15
131.30
130.18
109.36
83.17
81.16
81.00
79.10
77.37 CDCl3
77.13
76.95 CDCl3
63.23
60.26
40.20
35.91
34.69
34.57
28.37
25.86
25.80
25.44
180 170 160 150 140 130 120 110 100 90 80 70 60 50 40 30 20 10 0 -10

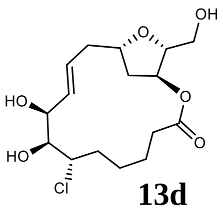

**13d**

An approximately 1.2 M solution of aqueous HCl in MeOH was prepared by slowly adding concentrated HCl (1 mL, *ca.* 12 M) to MeOH (9 mL). To a cold (0 °C), stirred solution of **S143** (5.0 mg, 0.012 mmol, 1.0 eq.) in MeOH (1.4 mL) was added the previously prepared solution of aqueous HCl in MeOH (50 µL, *ca.* 1.2M in MeOH, 0.060 mmol, 5.0 eq.). The reaction vessel was moved to a fridge (4 °C) and allowed to stir in the fridge for 23 h. After this time, starting material was consumed as monitored by TLC analysis, and the reaction mixture was quenched by addition of $NaHCO_3$ (25 mg, 0.30 mmol, 25 eq.) and allowed to stir vigorously for 5 min. After this time, the reaction mixture was filtered through a cotton plug. Then silica gel was added and the reaction mixture was concentrated onto the silica gel in vacuo. The crude product was then purified via flash column chromatography (97:3 to 92.5:7.5 $CH_2Cl_2$/MeOH). Appropriate fractions were pooled, and solvent was removed in vacuo to yield **13d** (3.3 mg, 73%) as a white film.

**Analytical Data for 13d:**

$R_f$ = 0.50 (9:1 $CH_2Cl_2$/MeOH)

$[\alpha]_D^{20}$ = +150° (c = 0.13, MeOH)

$^1$H NMR (601 MHz, MeOD) δ 5.74 – 5.64 (m, 2H), 5.17 (d, *J* = 4.3 Hz, 1H), 4.28 (dt, *J* = 5.7, 2.8 Hz, 1H), 4.03 (ddd, *J* = 5.5, 4.3, 1.0 Hz, 1H), 3.87 (ddd, *J* = 8.0, 6.6, 4.8 Hz, 1H), 3.78 (tt, *J* = 11.4, 3.8 Hz, 1H), 3.65 (dd, *J* = 6.6, 2.9 Hz, 1H), 3.58 (dd, *J* = 11.7, 4.4 Hz, 1H), 3.50 (dd, *J* = 11.7, 5.6 Hz, 1H), 2.61 (ddd, *J* = 12.6, 8.3, 4.4 Hz, 1H), 2.41 (ddd, *J* = 12.7, 8.0, 3.6 Hz, 1H), 2.19 (ddd, *J* = 12.7, 9.0, 3.7 Hz, 1H), 2.13 – 2.05 (m, 1H), 1.98 (dd, *J* = 13.5, 3.2 Hz, 1H), 1.91 – 1.81 (m, 1H), 1.74 – 1.54 (m, 4H), 1.53 – 1.39 (m, 2H).

$^{13}$C NMR (151 MHz, MeOD) δ 175.21, 133.30, 131.21, 84.82, 81.48, 78.55, 77.05, 75.61, 64.71, 63.47, 39.76, 36.48, 36.16, 34.99, 27.09, 26.62.

HRMS (ESI): Anal. Calcd. for $C_{16}H_{24}ClO_5^+$ $[M+H-H_2O]^+$ 331.1307, found 331.1315

IR (neat): $\nu_{max}$ $(cm^{-1})$ = 3396 (br, OH), 2929 (m, CH), 2870 (m, CH), 1723 (s, C=O), 1656 (m, C=C), 1443 (m), 1375 (m), 1337 (m), 1259 (m)

**13dAc** was prepared according to general procedure A (1.33 mg, quant.)

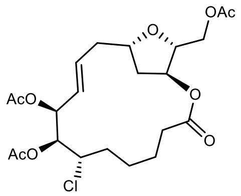


**13dAc**

**Analytical Data for 13dAc:**

$R_f$ = 0.82 (1:3 Hexanes/EtOAc)

HRMS (ESI): Anal. Calcd. for $C_{22}H_{35}NO_9Cl^+$ $[M+NH_4]^+$ 492.1995, found 492.1970

1H
MeOD
600.51 MHz

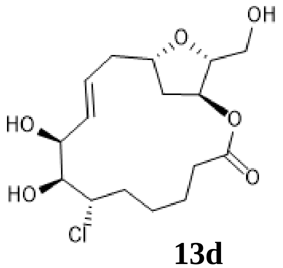


**13d**

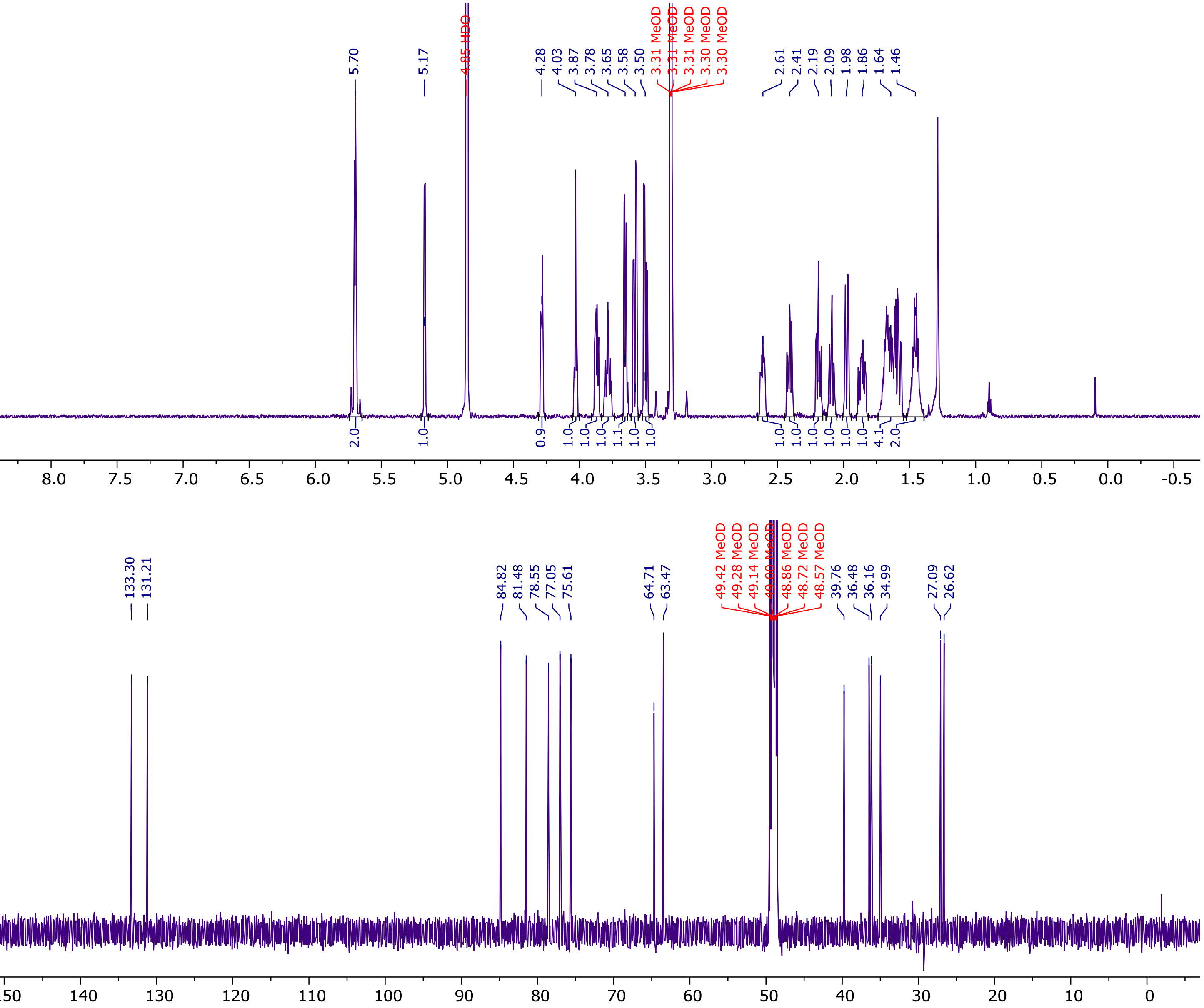

To a rt, stirred solution of **S143** (116 mg, 0.298 mmol, 1.0 eq.) in $CH_2Cl_2$ (6 mL) was added $NaHCO_3$ (75.1 mg, 0.894 mmol, 3.0 eq.) followed by Dess-Martin Periodinane (164.4 mg, 0.387 mmol, 1.3 eq.). The mixture was stirred at rt for 1 h 10 min. After this time, starting material was consumed as monitored by TLC analysis. The reaction mixture was quenched with a 1:1 (v/v) saturated aqueous $NaHCO_3$-10% aqueous $Na_2S_2O_3$ (10 mL) and the biphasic mixture was stirred vigorously for 30 min at rt. The aqueous layer was extracted with $CH_2Cl_2$ (3x 5 mL), and the combined organic layers were dried ($Na_2SO_4$), filtered, and the solvent was removed in vacuo. The crude product was passaged through a short plug of C2 modified silica gel eluting with $Et_2O$. Solvent was removed in vacuo to yield the crude aldehyde **S144** (*ca.* 116 mg) as a colorless oil which was portioned and used immediately in the next steps without further purification.

To a solution of **S144** (29.0 mg, 0.075 mmol, 1.0 eq.) in deoxygenated DMSO (5x freeze-pump-thaw cycles) (0.7 mL) was added $CrCl_2$ doped with 1 % $NiCl_2$ (w/w) (100.7 mg, 0.819 mmol, 10.9 eq.). **3b** (38.0 mg, 0.15 mmol, 2.0 eq.) was added via syringe in deoxygenated DMSO (1 mL + 0.3 mL rinse) and the mixture was stirred for 36 h at rt. After this time, the reaction mixture was transferred to a separatory funnel, diluted with $Et_2O$ (10 mL) and 10 mL of 1:1 (v/v) $H_2O$-brine was added. The organic layer was separated, and the aqueous layer extracted with $Et_2O$ (21x 10 mL). The combined organic layers were dried ($MgSO_4$), filtered, and solvent was removed in vacuo. The crude product was purified via flash column chromatography (9:1 to 8.5:1.5 Hexanes/Acetone). Appropriate fractions were pooled, and solvent was removed in vacuo to yield **S145** (10.8 mg) as a colorless oil and a 1:1 mixture of diastereomers. The product was used immediately in the subsequent step.

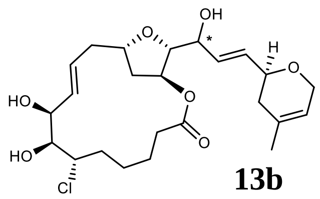


**13b**

* 1:1 mixture of diastereomers

Note: an approximately 1.2 M solution of aqueous HCl in MeOH by slowly adding concentrated HCl (1 mL, *ca.* 12 M) to MeOH (9 mL)**.** To a cold (0 °C), stirred solution of **S145** (10.8 mg, 0.0211 mmol, 1.0 eq.) in $CH_2Cl_2$ (1 mL) and MeOH (1 mL) was added the previously prepared solution of aqueous HCl in MeOH (88 µL, *ca.* 1.2M in MeOH, 0.106 mmol, 5.0 eq.) via microsyringe. The reaction vessel was moved to a fridge (4 °C) and allowed to stir in the fridge for 18 h. After this time, the reaction mixture was warmed to rt, and allowed to stir for a further 7 h. After this time, the reaction mixture was quenched by addition of $NaHCO_3$ (53.5 mg, 0.637 mmol, 30.2 eq.) and allowed to stir vigorously for 5 min. After this time, the reaction mixture was filtered and solvent was removed in vacuo. The crude product was then purified via flash column chromatography (99:1 to 24:1 $CH_2Cl_2$/EtOH). Appropriate fractions were pooled, and solvent was removed in vacuo to yield **13b** (7.5 mg, 13.7%, 6 steps from **1c**) as a white foam and a mixture of diastereomers (1:1).

**Analytical Data for 13b:**

$R_f$ = 0.75 (93:7 $CH_2Cl_2$/MeOH)

$^{1}$H NMR (601 MHz, $CDCl_3$) δ 5.96 – 5.87 (m, 1H), 5.81 – 5.73 (m, 1H), 5.71 – 5.62 (m, 2H), 5.41 (ddt, *J* = 3.8, 2.8, 1.4 Hz, 1H), 5.20 (dd, *J* = 4.5, 1.3 Hz, 0.5H), 5.12 (d, *J* = 4.4 Hz, 0.5H), 4.33 – 4.27 (m, 1.5H), 4.22 – 4.13 (m, 2H), 4.11 – 3.97 (m, 2.5H), 3.92 (dq, *J* = 7.7, 6.0 Hz, 1H), 3.73 (dtt, *J* = 15.2, 11.4, 3.7 Hz, 1H), 3.65 (dq, *J* = 6.1, 4.1 Hz, 1H), 2.87 (s, 1H), 2.70 (ddt, *J* = 12.3, 8.4, 4.7 Hz, 1H), 2.49 – 2.30 (m, 2H), 2.27 – 2.19 (m, 1H), 2.14 – 2.07 (m, 1H), 2.07 – 1.99 (m, 1H), 1.95 – 1.84 (m, 3H), 1.72 (m, 1H), 1.70 (s, 3H), 1.67 – 1.57 (m, 3H), 1.57 – 1.50 (m, 1H), 1.48 – 1.35 (m, 2H).

$^{13}$C NMR (151 MHz, $CDCl_3$) δ 173.53, 173.49, 134.30, 133.64, 131.93, 131.81, 131.52, 131.47, 130.55, 130.45, 128.77, 127.62, 119.84, 119.80, 85.90, 85.54, 80.78, 80.53, 75.71, 75.08, 74.77, 74.74, 73.11, 73.06, 72.73, 71.73, 65.79, 64.15, 64.02, 39.93, 39.44, 35.77, 35.73, 35.46, 35.42, 35.26, 35.22, 34.36, 34.32, 25.92, 25.87, 23.07.

HRMS (ESI): Anal. Calcd. for $C_{24}H_{36}ClO_7^+$ $[M+H]^+$ 471.2144, found 471.2144

IR (neat): $\nu_{max}$ $(cm^{-1})$ = 3420 (br, OH), 2926 (m, CH), 2873 (m, CH), 1727 (s, C=O), 1672 (m, C=C), 1441 (m), 1378 (m), 1336 (m), 1257 (m).

**13bAc** was prepared according to general procedure A (1.34 mg, quant.)

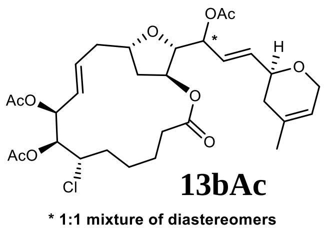


**13bAc**

* 1:1 mixture of diastereomers

**Analytical Data for 13bAc:**

$R_f$ = 0.90 (2:3 Hexanes/EtOAc)

HRMS (ESI): Anal. Calcd. for $C_{30}H_{41}O_{10}NaCl^+$ $[M+Na]^+$ 619.2281, found 619.2300

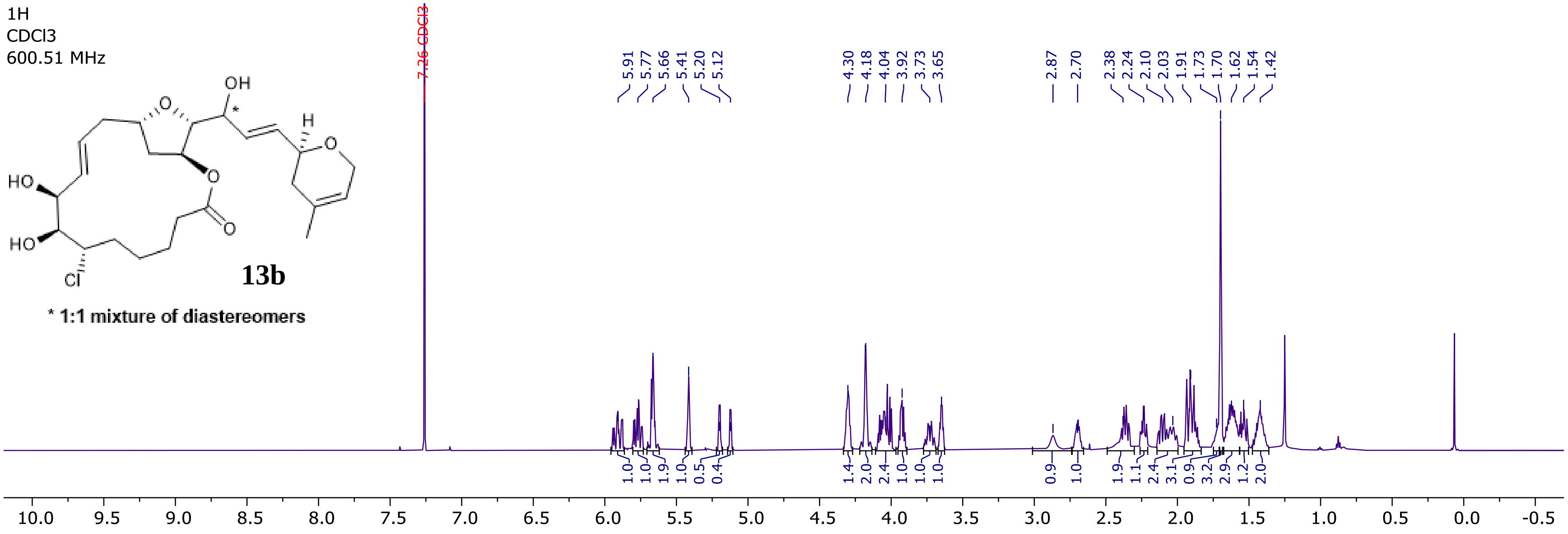

**13b**

* 1:1 mixture of diastereomers



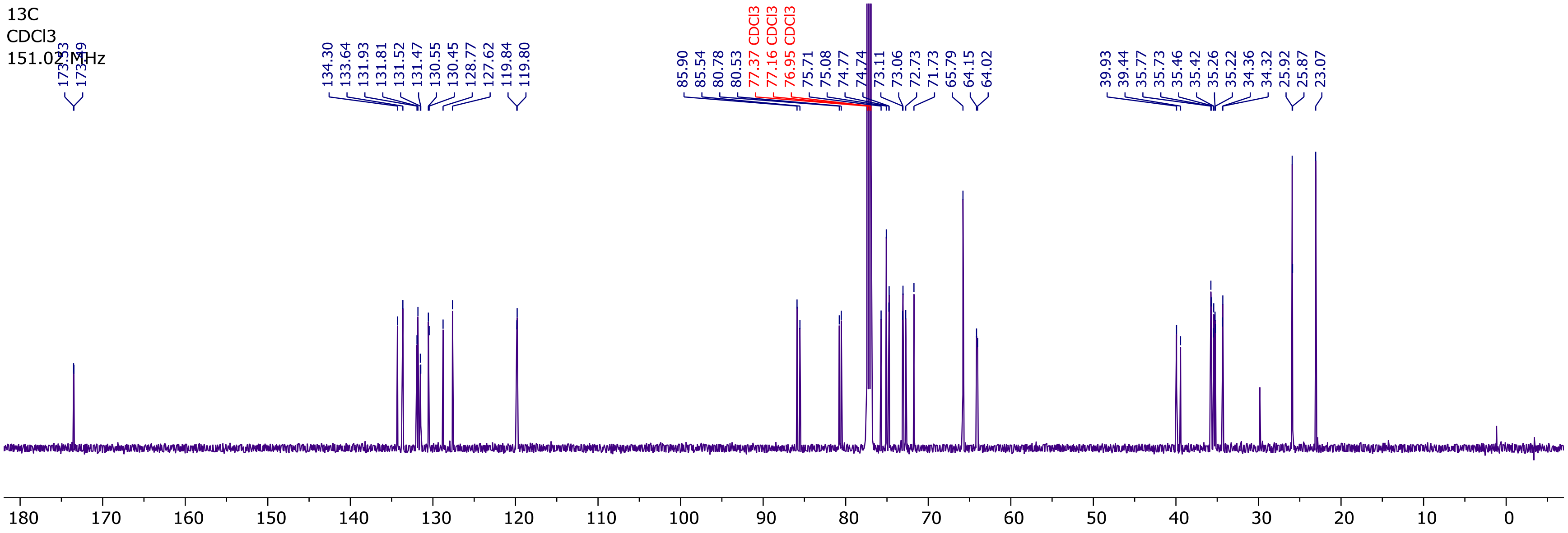

To a solution of **S144** (29.0 mg, 0.075 mmol, 1.0 eq.) in deoxygenated DMSO (5x freeze-pump-thaw cycles) (0.7 mL) was added $CrCl_2$ doped with 1 % $NiCl_2$ (w/w) (93.3 mg, 0.759 mmol, 10.1 eq.). **3a** (53.8 mg, 0.225 mmol, 3.0 eq.) was added via syringe in deoxygenated DMSO (1 mL + 0.3 mL rinse) and the mixture was stirred for 36 h at rt. After this time, the reaction mixture was transferred to a separatory funnel, diluted with $Et_2O$ (10 mL) and 10 mL of 1:1 (v/v) $H_2O$-brine was added. The organic layer was separated, and the aqueous layer extracted with $Et_2O$ (30x 10 mL). The combined organic layers were dried ($MgSO_4$), filtered, and solvent was removed in vacuo. The crude product was purified via flash column chromatography (7:3 to 3:2 Hexanes/Acetone). Appropriate fractions were pooled, and solvent was removed in vacuo to yield **S146** (13.7 mg) as a colorless oil and a 1:1 mixture of diastereomers. The product was used immediately in the subsequent step.

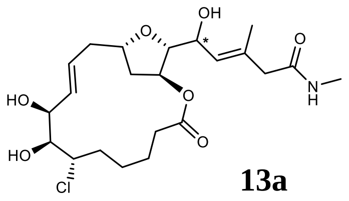


**13a**

* 1:1 mixture of diastereomers

An approximately 1.2 M solution of aqueous HCl in MeOH was prepared by slowly adding concentrated HCl (1 mL, *ca.* 12 M) to MeOH (9 mL). To a cold (0 °C), stirred solution of **S146** (13.7, 0.0274 mmol, 1.0 eq.) in $CH_2Cl_2$ (1.3 mL) MeOH (1.3 mL) was added the previously prepared solution of aqueous HCl in MeOH (114 µL, *ca.* 1.2M in MeOH, 0.137 mmol, 5.0 eq.) via microsyringe. The reaction vessel was moved to a fridge (4 °C) and allowed to stir in the fridge for 18 h. After this time, the reaction mixture was warmed to rt, and allowed to stir for a further 9 h. After this time, starting material was consumed as monitored by TLC analysis, and the reaction mixture was quenched by addition of $NaHCO_3$ (54.2 mg, 0.645 mmol, 23.7 eq.) and allowed to stir vigorously for 5 min. After this time, the reaction mixture was filtered and solvent was removed in vacuo. The crude product was then purified via flash column chromatography (97:3 to 47:3 $CH_2Cl_2$/MeOH). Appropriate fractions were pooled, and solvent was removed in vacuo to yield **13a** (10.0 mg, 18.7%, 6 steps from **1c**) as a white amorphous solid and a mixture of diastereomers (1:1).

**Analytical Data for 13a:**

$R_f$ = 0.23 (93:7 $CH_2Cl_2$/MeOH)

$^1$H NMR (601 MHz, MeOD) δ 5.70 (dtt, *J* = 5.1, 2.8, 1.3 Hz, 2H), 5.34 (ddq, *J* = 11.3, 8.7, 1.3 Hz, 1H), 5.24 (d, *J* = 4.3 Hz, 0.5H), 5.16 – 5.12 (m, 0.5H), 4.38 (dd, *J* = 8.9, 5.0 Hz, 0.5H), 4.31 (dd, *J* = 8.7, 5.5 Hz, 0.5H), 4.28 (dq, *J* = 5.7, 2.9 Hz, 1H), 3.99 (dd, *J* = 5.0, 1.1 Hz, 0.5H), 3.89 (dd, *J* = 5.4, 1.0 Hz, 0.5H), 3.88 – 3.84 (m, 1H), 3.80 – 3.73 (m, 1H), 3.65 (dt, *J* = 6.6, 2.7 Hz, 1H), 2.96 – 2.87 (m, 2H), 2.72 (s, 1.5H), 2.71 (s, 1.5H), 2.63 – 2.57 (m, 1H), 2.40 (dddd, *J* = 13.2, 8.4, 3.5, 1.7 Hz, 1H), 2.19 (ddt, *J* = 12.7, 8.9, 3.3 Hz, 1H), 2.13 – 2.04 (m, 1H), 1.95 (ddd, *J* = 22.2, 13.4, 3.3 Hz, 1H), 1.89 – 1.81 (m, 1H), 1.74 (t, *J* = 1.3 Hz, 3H), 1.72 – 1.57 (m, 4H), 1.45 (dddd, *J* = 19.5, 13.4, 5.8, 2.5 Hz, 2H).

$^{13}$C NMR (151 MHz, MeOD) δ 175.20, 175.18, 174.06, 173.97, 135.91, 135.73, 133.34, 133.22, 131.33, 131.15, 129.32, 128.72, 87.60, 87.43, 81.65, 81.41, 78.03, 77.82, 77.04, 75.61, 75.59, 69.29, 64.72, 47.69, 47.56, 40.10, 39.83, 36.42, 36.41, 36.17, 34.98, 27.11, 26.63, 26.46, 26.45, 17.24.

HRMS (ESI): Anal. Calcd. for $C_{22}H_{35}ClNO_7^+$ $[M+H]^+$ 460.2097, found 460.2103

IR (neat): $\nu_{max}$ $(cm^{-1})$ = 3349 (br, OH), 2922 (m, CH), 2859 (m, CH), 1728 (s, C=O), 1683 (s, C=C), 1550 (m), 1443 (m), 1414 (m), 1264 (m)

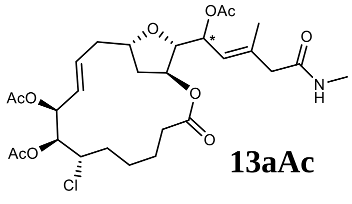


**13aAc**

* 1:1 mixture of diastereomers

**13aAc** was prepared according to general procedure A (1.03 mg, 72%)

**Analytical Data for 13aAc:**

$R_f$ = 0.50 (EtOAc)

$^1$H NMR (601 MHz, $C_6D_6$) δ 5.72 (dd, *J* = 9.2, 5.3 Hz, 0.5H), 5.67 – 5.64 (m, 0.5H), 5.62 (dd, *J* = 8.9, 3.0 Hz, 0.5H), 5.56 (dd, *J* = 9.2, 5.6 Hz, 0.5H), 5.50 (ddd, *J* = 14.9, 9.7, 5.0 Hz, 0.5H), 5.46 – 5.41 (m, 2.5H), 5.39 (d, *J* = 4.4 Hz, 0.5H), 5.35 (d, *J* = 4.3 Hz, 0.5H), 5.21 – 5.16 (m, 1.5H), 4.71 (q, *J* = 4.5 Hz, 0.5H), 4.30 (dd, *J* = 5.3, 1.1 Hz, 0.5H), 4.17 (dd, *J* = 5.6, 1.1 Hz, 0.5H), 3.68 – 3.58 (m, 1H), 3.57 – 3.44 (m, 1H), 2.69 (dd, *J* = 14.9, 1.0 Hz, 0.5H), 2.64 – 2.59 (m, 1H), 2.54 – 2.48 (m, 2.5H), 2.45 (d, *J* = 4.8 Hz, 1.5H), 2.44 – 2.40 (m, 0.5H), 2.08 (td, *J* = 11.3, 5.1 Hz, 0.5H), 1.93 – 1.83 (m, 5H), 1.79 (d, *J* = 1.4 Hz, 1.5H), 1.75 – 1.73 (m, 3H), 1.72 – 1.66 (m, 3H), 1.66 – 1.63 (m, 3H), 1.55 (ddd, *J* = 13.5, 11.6, 4.6 Hz, 0.5H), 1.35 – 1.27 (m, 3.5H), 1.24 – 1.10 (m, 3H).

$^{13}$C NMR (151 MHz, $C_6D_6$) δ 172.96, 172.84, 169.70, 169.66, 169.61, 169.28, 169.25, 169.20, 138.23, 138.15, 135.43, 134.86, 126.74, 126.38, 124.53, 123.94, 84.41, 84.34, 80.44, 80.15, 76.41, 74.93, 74.91, 73.44, 73.38, 71.27, 71.08, 60.69, 60.56, 47.50, 47.40, 39.60, 39.29, 36.01, 35.86, 34.90, 34.88, 33.44, 33.37, 26.23, 26.19, 25.78, 25.75, 25.30, 25.26, 20.65, 20.63, 20.60, 20.41, 17.66, 17.56.

HRMS (ESI): Anal. Calcd. for $C_{28}H_{41}NO_{10}Cl^+$ $[M+H]^+$ 586.2414, found 586.2442

IR (neat): $\nu_{max}$ $(cm^{-1})$ = 3384 (br, NH), 3319 (br, NH), 3056 (w, C=*CH*), 2943 (m, CH), 2871 (m, CH), 1737 (vs, CO), 1659 (m, C=C), 1545 (m), 1437 (m), 1372 (m), 1235 (vs, CO).

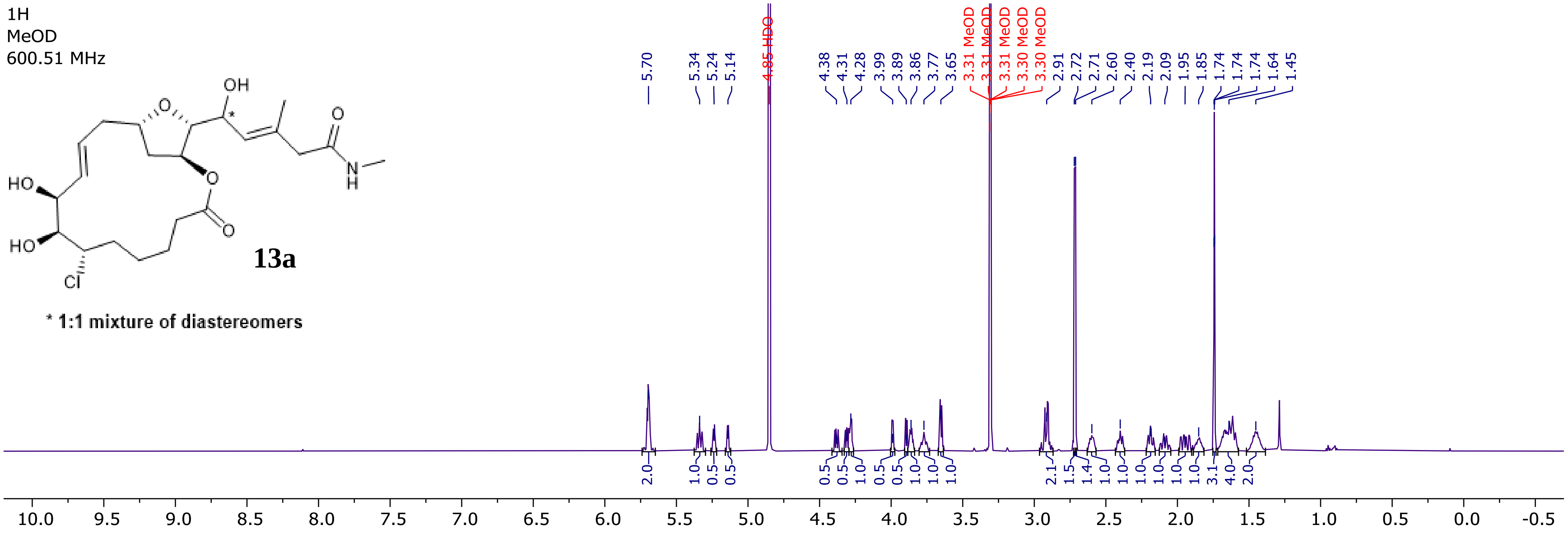
1H
MeOD
600.51 MHz
OH
O
HO
HO
Cl
N
H
13a
* 1:1 mixture of diastereomers
5.70
5.34
5.24
5.14
4.85 HDO
4.38
4.31
4.28
3.99
3.89
3.86
3.77
3.65
3.31 MeOD
3.31 MeOD
3.31 MeOD
3.30 MeOD
3.30 MeOD
2.91
2.72
2.71
2.60
2.40
2.19
2.09
1.95
1.85
1.74
1.74
1.74
1.64
1.45
10.0
9.5
9.0
8.5
8.0
7.5
7.0
6.5
6.0
5.5
5.0
4.5
4.0
3.5
3.0
2.5
2.0
1.5
1.0
0.5
0.0
-0.5

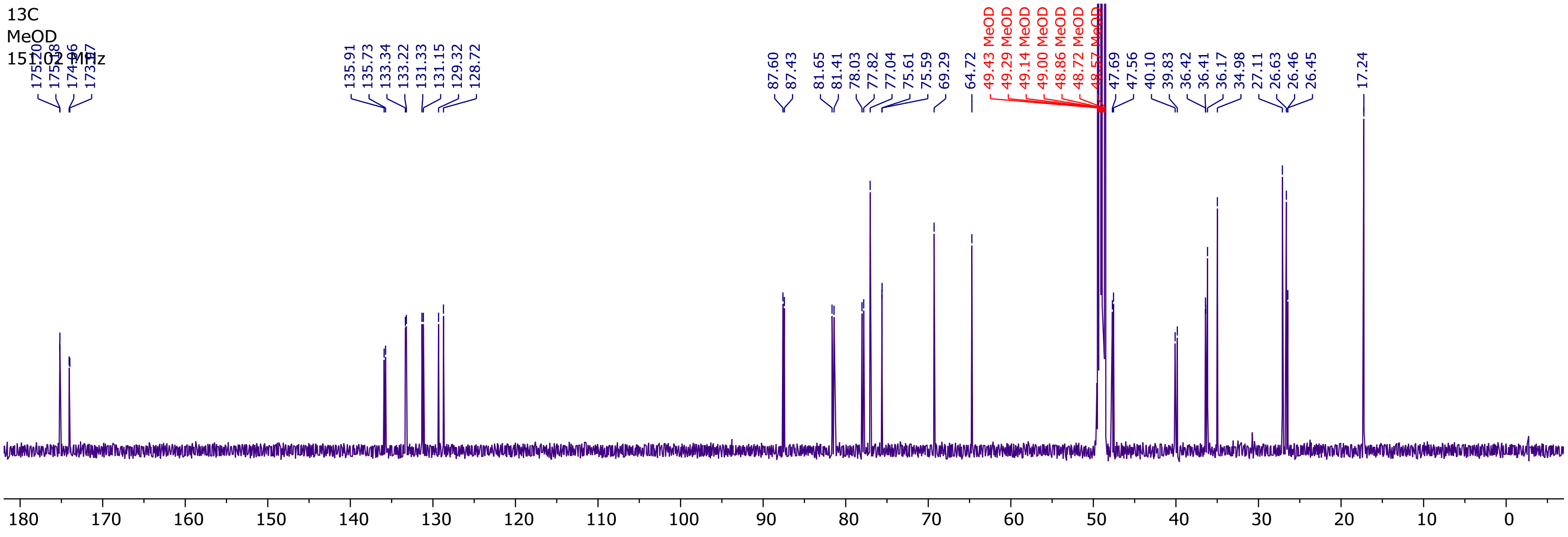
13C
MeOD
151.02 MHz
135.91
135.73
133.34
133.22
131.33
131.15
129.32
128.72
87.60
87.43
81.65
81.41
78.03
77.82
77.04
75.61
75.59
69.29
64.72
49.43 MeOD
49.29 MeOD
49.14 MeOD
49.00 MeOD
48.86 MeOD
48.72 MeOD
47.69
47.56
40.10
39.83
36.42
36.41
36.17
34.98
27.11
26.63
26.46
26.45
17.24
180
170
160
150
140
130
120
110
100
90
80
70
60
50
40
30
20
10
0

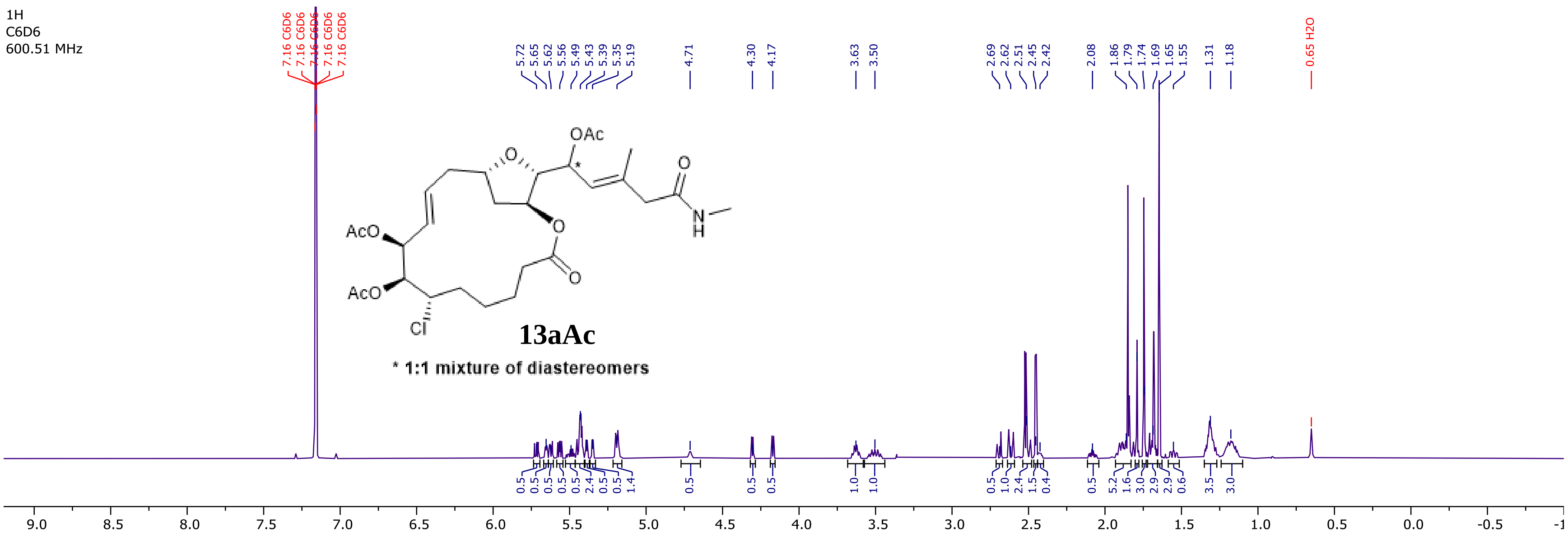
1H
C6D6
600.51 MHz
AcO
AcO
Cl
OAc
O
N
H
13aAc
* 1:1 mixture of diastereomers

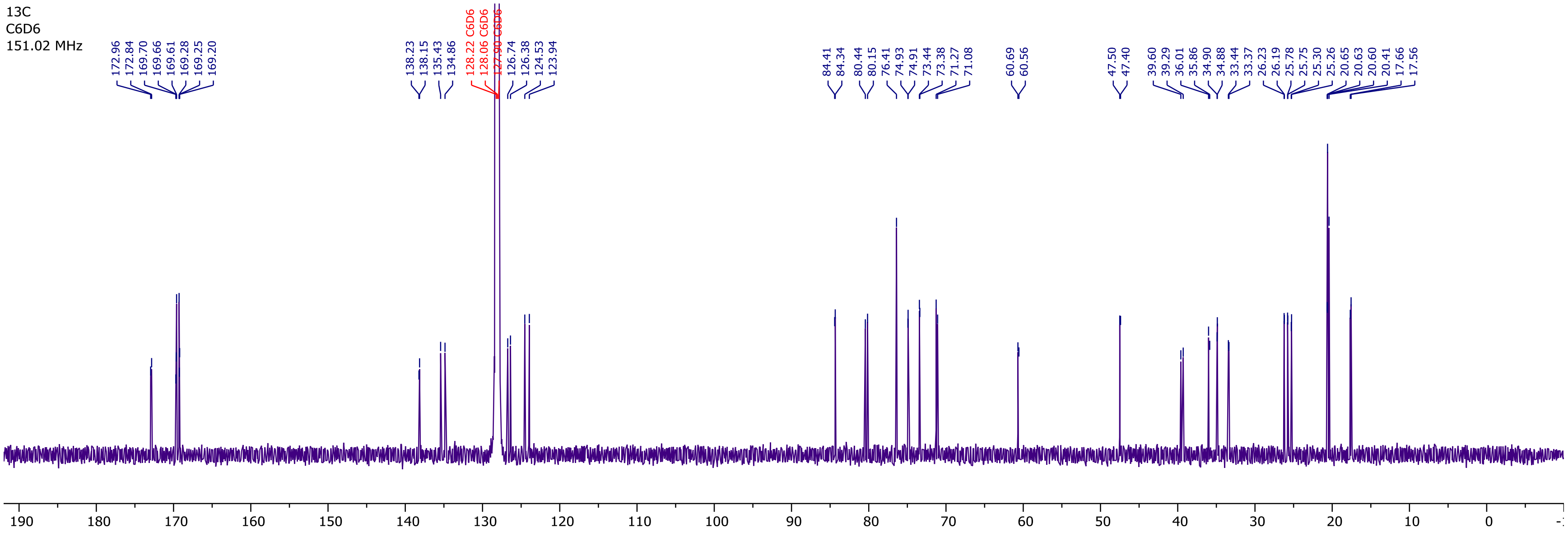
13C
C6D6
151.02 MHz

To a solution of **S144** (29.0 mg, 0.075 mmol, 1.0 eq.) in deoxygenated DMSO (5x freeze-pump-thaw cycles) (2 mL) was added $CrCl_2$ doped with 1 % $NiCl_2$ (w/w) (96.2 mg, 0.783 mmol, 10.4 eq.). **3c** (33.5 µL, 56.7 mg, 0.225 mmol, 3.0 eq.) was added via microsyringe and the mixture was stirred for 36 h at rt. After this time, the reaction mixture was transferred to a separatory funnel, diluted with $Et_2O$ (10 mL) and 10 mL of 1:1 (v/v) $H_2O$-brine was added. The organic layer was separated, and the aqueous layer extracted with $Et_2O$ (21x 10 mL). The combined organic layers were dried ($MgSO_4$), filtered, and solvent was removed in vacuo. The crude product was purified via flash column chromatography (9:1 to 8.5:1.5 Hexanes/Acetone). Appropriate fractions were pooled, and solvent was removed in vacuo to yield **S147** (13.6 mg, 35%) as a colorless oil and a 7:3 mixture of diastereomers. A portion of the product was used in the subsequent step.

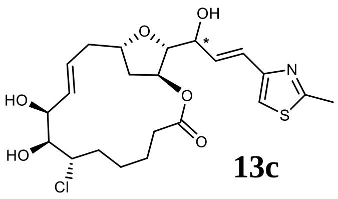


**13c**

* 7:3 mixture of diastereomers

An approximately 1.2 M solution of aqueous HCl in MeOH was prepared by slowly adding concentrated HCl (1 mL, *ca.* 12 M) to MeOH (9 mL). To a cold (0 °C), stirred solution of **S147** (10.7 mg, 0.0209 mmol, 1.0 eq.) in $CH_2Cl_2$ (1 mL) MeOH (1 mL) was added the previously prepared solution of aqueous HCl in MeOH (87 µL, *ca.* 1.2M in MeOH, 0.104 mmol, 5.0 eq.) via microsyringe. The reaction vessel was moved to a fridge (4 °C) and allowed to stir in the fridge for 18 h. After this time, the reaction mixture was warmed to rt, and allowed to stir for a further 11 h. After this time, starting material was consumed as monitored by TLC analysis, and the reaction mixture was quenched by addition of $NaHCO_3$ (51.5 mg, 0.613 mmol, 29.3 eq.) and allowed to stir vigorously for 5 min. After this time, the reaction mixture was filtered and solvent was removed in vacuo. The crude product was then purified via flash column chromatography (99:1 to 24:1 $CH_2Cl_2$/MeOH). Appropriate fractions were pooled, and solvent was removed in vacuo to yield **13c** (9.1 mg, 92%, or 20.8% 6 steps from **1c**) as a white amorphous solid.

**Analytical Data for 13c:**

$R_f$ = 0.42 (93:7 $CH_2Cl_2$/MeOH)

$^1$H NMR (601 MHz, $CDCl_3$) δ 6.94 (s, 7H), 6.93 (s, 0.3H), 6.70 (dd, *J* = 15.6, 1.7 Hz, 0.3H), 6.66 (dd, *J* = 15.6, 1.3 Hz, 0.7H), 6.58 – 6.48 (m, 1H), 5.72 – 5.60 (m, 2H), 5.22 (d, *J* = 4.6 Hz, 0.3H), 5.17 (d, *J* = 4.4 Hz, 0.7H), 4.48 (s, 0.3H), 4.31 (s, 1H), 4.25 – 4.20 (m, 0.7H), 4.13 (dd, *J* = 3.5, 1.2 Hz, 0.3H), 4.08 (dd, *J* = 5.7, 1.1 Hz, 0.7H), 3.96 – 3.90 (m, 1H), 3.79 – 3.71 (m, 1H), 3.67 – 3.62 (m, 1H), 2.89 (d, *J* = 5.5 Hz, 1H), 2.74 – 2.71 (m, 0.3H), 2.70 (s, 3H), 2.41 (d, *J* = 7.0 Hz, 1H), 2.40 – 2.32 (m, 1.7H), 2.22 (dddd, *J* = 15.7, 12.5, 9.1, 3.1 Hz, 1H), 2.17 – 2.07 (m, 1H), 1.95 (dd, *J* = 13.4, 3.2 Hz, 1H), 1.91 – 1.85 (m, 1H), 1.75 – 1.68 (m, 1H), 1.67 – 1.54 (m, 4H), 1.48 – 1.36 (m, 2H).

$^{13}C$ NMR (151 MHz, $CDCl_3$) δ 173.58, 173.52, 166.37, 152.87, 152.77, 131.96, 131.87, 130.51, 130.44, 129.84, 128.74, 125.66, 124.85, 115.84, 115.79, 85.84, 85.59, 80.79, 80.59, 75.74, 75.07, 74.80, 74.77, 72.90, 71.81, 64.10, 63.97, 40.08, 39.43, 35.47, 35.30, 35.23, 34.38, 34.36, 25.92, 25.88, 25.85, 19.45.

HRMS (ESI): Anal. Calcd. for $C_{22}H_{31}ClNO_6S^+$ $[M+H]^+$ 472.1555, found 472.1547

IR (neat): $\nu_{max}$ ($cm^{-1}$) = 3380 (br, OH), 2922 (m, CH), 2861 (m, CH), 1724 (s, C=O), 1658 (m, C=C), 1438 (m), 1375 (m), 1335 (m), 1262 (m)

**13cAc** was prepared according to general procedure A (0.65 mg, 56%)

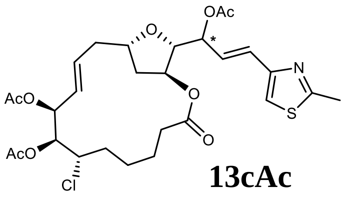


**13cAc**

* 7:3 mixture of diastereomers

**Analytical Data for 13cAc:**

$R_f$ = 0.64 (2:3 Hexanes/EtOAc)

HRMS (ESI): Anal. Calcd. for $C_{28}H_{36}NO_9NaSCl^+$ $[M+Na]^+$ 620.1692, found 620.1668

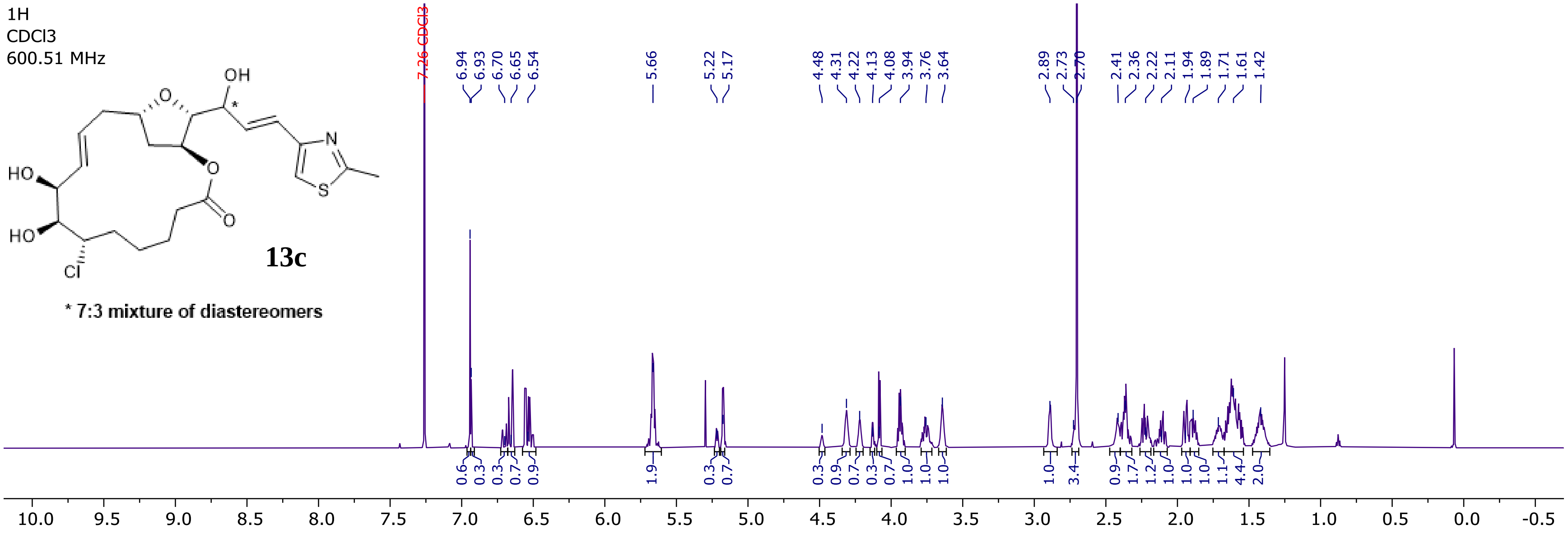
1H
CDCl3
600.51 MHz
13c
* 7:3 mixture of diastereomers
7.26 CDCl3
6.94 6.93 6.70 6.65 6.54
5.66
5.22 5.17
4.48 4.31 4.22 4.13 4.08 3.94 3.76 3.64
2.89 2.73 2.70
2.41 2.36 2.22 2.11 1.94 1.89 1.71 1.61 1.42
0.6 0.3 0.3 0.7 0.9 1.9 0.3 0.7 0.3 0.9 0.7 0.3 0.7 1.0 1.0 1.0 1.0 3.4 0.9 1.7 1.2 1.0 1.0 1.0 1.1 4.4 2.0
10.0 9.5 9.0 8.5 8.0 7.5 7.0 6.5 6.0 5.5 5.0 4.5 4.0 3.5 3.0 2.5 2.0 1.5 1.0 0.5 0.0 -0.5

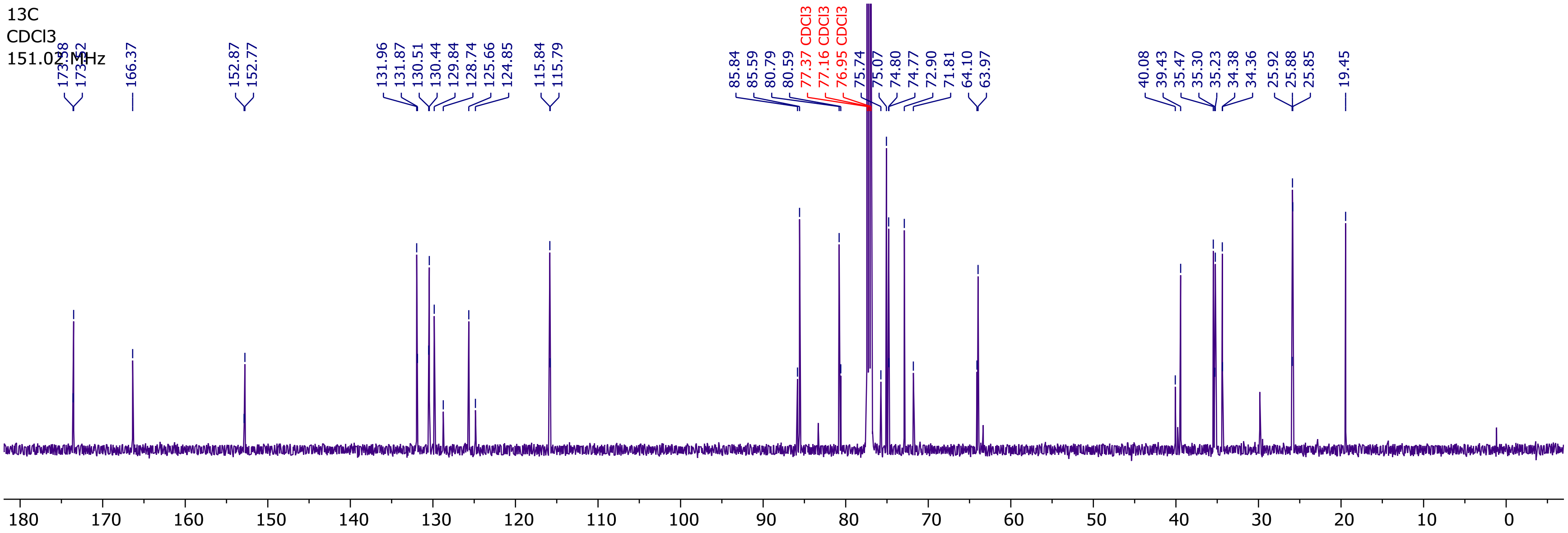
13C
CDCl3
151.02 MHz
173.88 173.52
166.37
152.87 152.77
131.96 131.87 130.51 130.44 129.84 128.74 125.66 124.85
115.84 115.79
85.84 85.59 80.79 80.59
77.37 CDCl3 77.16 CDCl3 76.95 CDCl3
75.74 75.07 74.80 74.77 72.90 71.81 64.10 63.97
40.08 39.43 35.47 35.30 35.23 34.38 34.36 25.92 25.88 25.85
19.45
180 170 160 150 140 130 120 110 100 90 80 70 60 50 40 30 20 10 0

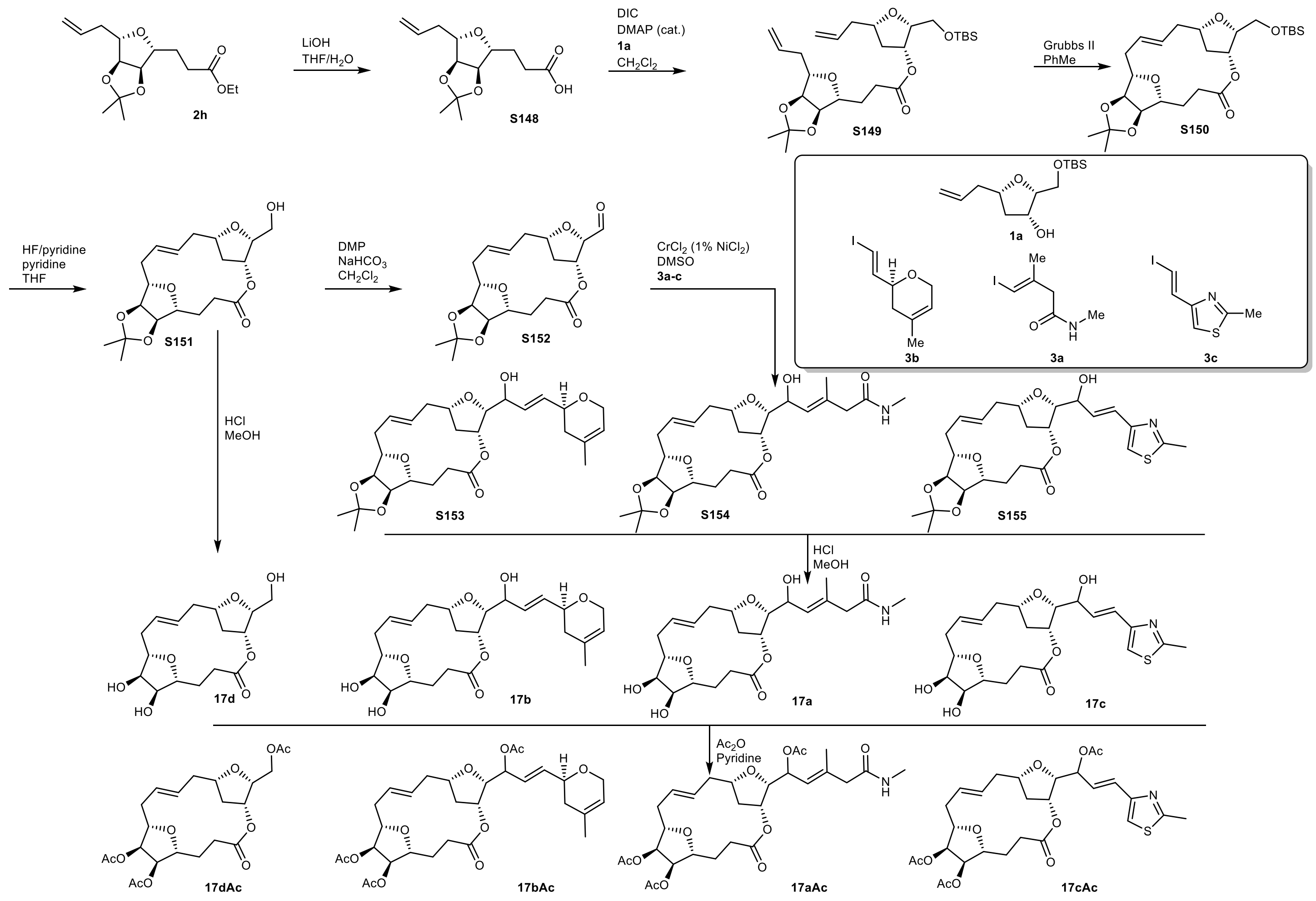


**Supplementary Fig. 50 | Synthesis of pMLs 17a-d and 17aAc-dAc.**

Abbreviations: THF = tetrahydrofuran, DIC = N,N'-diisopropylcarbodiimide, DMAP = 4-dimethylaminopyridine, TBS = tert-butyldimethylsilyl, Grubbs II = Dichloro[1,3-bis(2,4,6-trimethylphenyl)-2-imidazolidinylidene](benzylidene)(tricyclohexylphosphine)ruthenium(II), DMP = Dess-Martin periodinane, DMSO = dimethylsulfoxide.

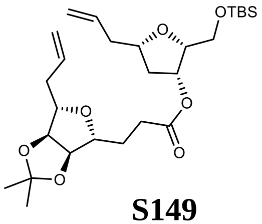

**S149**

To a rt, stirred solution of **2h** (255.9 mg, 0.90 mmol, 1.0 eq.) in THF (4.3 mL) and $H_2O$ (2.3 mL) was added a solution of LiOH (2.7 mL, 1M in $H_2O$, 2.7 mmol, 3.0 eq.). The reaction mixture was vigorously stirred for 3 h 20 min. After this time, starting material was consumed as monitored by TLC analysis. The reaction mixture was diluted with $H_2O$ (15 mL) and $Et_2O$ (15 mL), transferred to a separatory funnel, and the mixture adjusted to *ca.* pH 4-5 with HCl (1M in $H_2O$). The organic layer was then separated, and the aqueous layer was extracted with EtOAc (4x 20 mL). The combined organic layers were dried ($MgSO_4$), filtered, and solvent was removed in vacuo to yield crude acid **S148** (230.5 mg) as a colorless oil which was used immediately in the next step without further purification.

To a rt, stirred solution of acid **S148** (230.5 mg, 0.90 mmol, 1.5 eq.), alcohol **1a** (161.5 mg, 0.60 mmol, 1.0 eq.), and 4-dimethylaminopyridine (29.3 mg, 0.24 mmol, 0.40 eq.) in $CH_2Cl_2$ (6 mL) was added N,N'-diisopropylcarbodiimide (0.18 mL, 146 mg, 1.15 mmol, 1.9 eq.). The reaction mixture was allowed to stir for 19 h at rt. After this time, starting material was consumed as monitored by TLC analysis. The cloudy reaction mixture was poured onto $H_2O$ (10 mL) in a separatory funnel, and the aqueous layer extracted with $CH_2Cl_2$ (3x 10 mL). The combined organic layers were dried ($Na_2SO_4$), filtered, and solvent was removed in vacuo to yield an oily residue. The residue was triturated vigorously with $Et_2O$ (3x 2 mL) and filtered. The filtrate was evaporated in vacuo to yield a red oil. The crude product was purified via flash column chromatography (4:1 Hexanes/EtOAc) to give **S149** (253 mg, 86%) as a colorless oil.

**Analytical Data for S149:**

$R_f$ = 0.68 (4:1 Hexanes/EtOAc)

$[\alpha]_D^{20}$ = -16° (c = 0.45, $CDCl_3$)

$^1$H NMR (600 MHz, $CDCl_3$) δ 5.86 – 5.74 (m, 2H), 5.32 (ddd, *J* = 6.9, 4.3, 2.8 Hz, 1H), 5.15 (dq, *J* = 17.2, 1.6 Hz, 1H), 5.11 (ddt, *J* = 10.2, 2.1, 1.2 Hz, 1H), 5.10 – 5.03 (m, 2H), 4.36 (dd, *J* = 7.1, 4.6 Hz, 1H), 4.29 (dd, *J* = 7.1, 4.9 Hz, 1H), 4.00 – 3.93 (m, 1H), 3.89 (td, *J* = 6.4, 4.6 Hz, 1H), 3.86 (td, *J* = 6.0, 4.3 Hz, 1H), 3.83 – 3.75 (m, 3H), 2.51 – 2.38 (m, 4H), 2.38 – 2.34 (m, 2H), 2.29 (dtt, *J* = 13.9, 6.8, 1.3 Hz, 1H), 1.98 (ddt, *J* = 13.8, 9.9, 5.8 Hz, 1H), 1.85 (dddd, *J* = 13.8, 9.8, 8.0, 5.8 Hz, 1H), 1.64 (ddd, *J* = 14.1, 6.8, 2.8 Hz, 1H), 1.52 (s, 3H), 1.32 (s, 3H), 0.87 (s, 9H), 0.05 (s, 3H), 0.04 (s, 3H).

$^{13}$C NMR (151 MHz, $CDCl_3$) δ 172.51, 134.61, 133.66, 117.95, 117.31, 114.98, 84.88, 84.36, 83.33, 83.05, 81.70, 77.42, 74.18, 61.41, 40.45, 38.29, 38.00, 30.71, 28.77, 27.49, 26.01, 25.62, 18.44, -5.16, -5.26.

HRMS (ESI): Anal. Calcd. for $C_{27}H_{50}NO_7Si^+$ $[M+NH_4]^+$ 528.3351, found 528.3347

IR (neat): $\nu_{max}$ ($cm^{-1}$) = 3078 (w, C=*CH*), 2931 (m, CH), 2858 (m, CH), 1738 (s, C=O), 1695 (w, C=C), 1468 (w), 1438 (w), 1378 (m), 1254 (s).

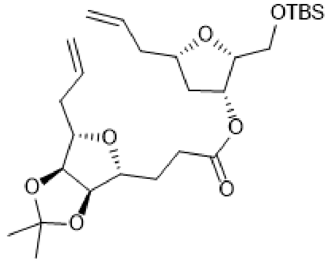
OTBS
O
O
O
O
O
O

**S149**

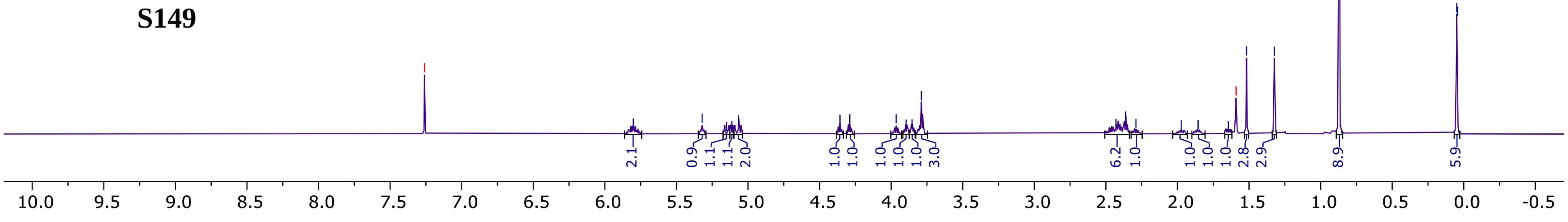
1H
CDCl3
600.13 MHz
7.26 CDCl3
5.80
5.32
5.15
5.11
5.07
4.36
4.29
3.97
3.90
3.85
3.79
2.43
2.36
2.29
1.97
1.86
1.64
1.59 H2O
1.52
1.32
0.87
0.05
0.04
2.1
0.9
1.1
1.1
2.0
1.0
1.0
1.0
1.0
1.0
3.0
6.2
1.0
1.0
1.0
1.0
2.8
2.9
8.9
5.9
10.0
9.5
9.0
8.5
8.0
7.5
7.0
6.5
6.0
5.5
5.0
4.5
4.0
3.5
3.0
2.5
2.0
1.5
1.0
0.5
0.0
-0.5

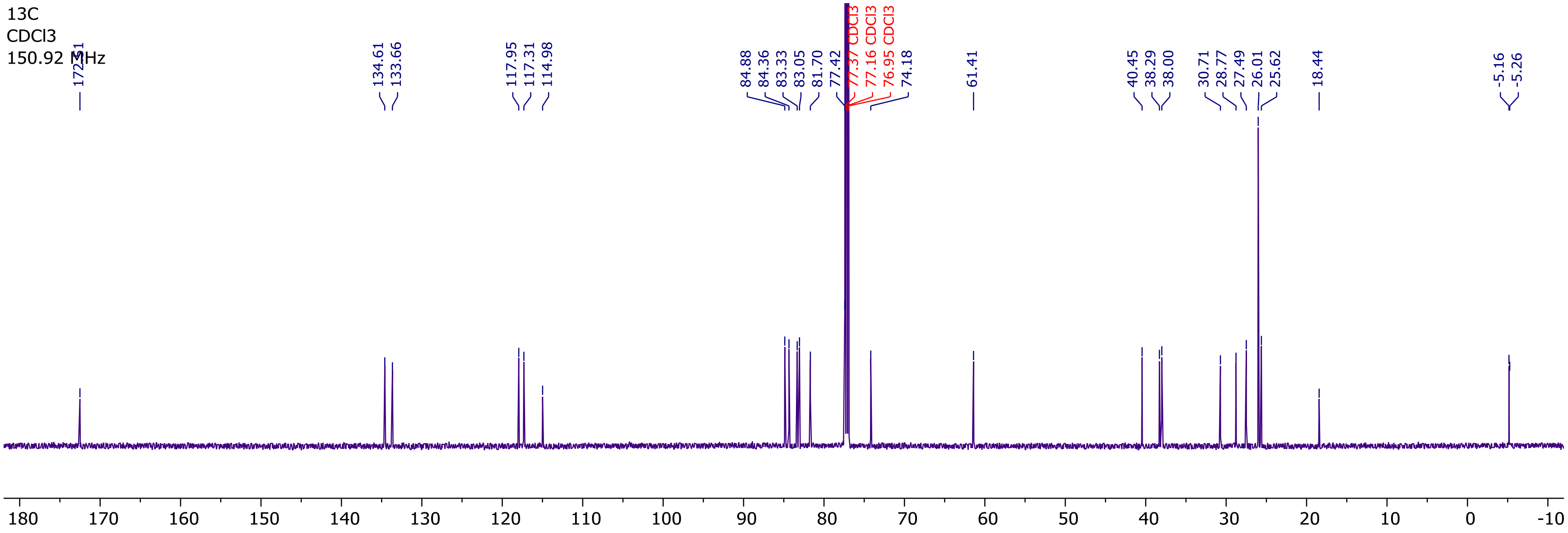
13C
CDCl3
150.92 MHz
172.51
134.61
133.66
117.95
117.31
114.98
84.88
84.36
83.33
83.05
81.70
77.42
77.37 CDCl3
77.16 CDCl3
76.95 CDCl3
74.18
61.41
40.45
38.29
38.00
30.71
28.77
27.49
26.01
25.62
18.44
-5.16
-5.26
180
170
160
150
140
130
120
110
100
90
80
70
60
50
40
30
20
10
0
-10

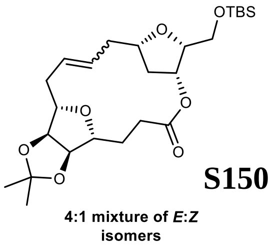


**S150**

To a warm (60 °C), stirred solution of **S149** (246 mg, 0.493 mmol, 1.0 eq.) in deoxygenated toluene (30 min. $N_2$(g) sparge) (200 mL) that was continuously being sparged with $N_2$(g) was added Grubbs second generation catalyst (83.7 mg, 0.0986 mmol, 0.20 eq.) in toluene (5 mL). The reaction was allowed to proceed under continuous nitrogen sparge for 2 h 35 min. After this time, starting material was consumed as monitored by TLC analysis, the reaction mixture was cooled to rt and quenched by addition of potassium 2-isocyanoacetate (85 mg, 0.69 mmol, 1.4 eq.) in MeOH (5 mL). The reaction mixture was stirred for a further 1 h, after which time the red solution changed to a pale-yellow color. The reaction mixture was concentrated in vacuo to give a crude solid deposited on the walls of the evaporation flask. The walls were scraped with a spatula and the solid was triturated with $Et_2O$ (3x 10 mL) and filtered through a short plug of silica gel eluting with $Et_2O$. The filtrate was concentrated in vacuo and the crude product was purified via flash column chromatography (4:1 to 3:2 Hexanes/$Et_2O$) on $AgNO_3$ impregnated silica gel. Appropriate fractions were pooled, and solvent was removed in vacuo to yield the products **S150** (181 mg, 78%) as a white amorphous solid and an inseparable mixture of *E* and *Z* isomers (4:1). Isomers were completely inseparable despite multiple attempts to separate them.

**Analytical Data for S150 as a 4:1 mixture of *E*:Z isomers:**

$R_f$ = 0.41 (7:3 Hexanes/$Et_2O$)

$[\alpha]_D^{20}$ = +26° (c = 0.38, $CDCl_3$)

$^1$H NMR (600 MHz, $CDCl_3$) δ 5.49 (dddd, *J* = 14.9, 10.9, 3.8, 1.7 Hz, 1H), 5.33 (dddd, *J* = 14.8, 10.4, 3.9, 1.6 Hz, 1H), 5.02 (dd, *J* = 5.7, 3.5 Hz, 0.2H), 4.96 (ddd, *J* = 7.2, 3.9, 1.4 Hz, 0.8H), 4.35 (dd, *J* = 7.1, 3.7 Hz, 0.2H), 4.27 – 4.21 (m, 2H), 4.08 (tdd, *J* = 8.0, 3.9, 2.4 Hz, 0.8H), 4.02 – 3.97 (m, 0.2H), 3.94 (dt, *J* = 11.8, 2.7 Hz, 1H), 3.91 – 3.81 (m, 3H), 3.74 (ddd, *J* = 6.8, 5.8, 3.9 Hz, 0.8H), 2.66 (ddt, *J* = 13.5, 4.0, 2.0 Hz, 0.8H), 2.47 (ddt, *J* = 14.0, 4.5, 2.4 Hz, 1H), 2.43 (ddd, *J* = 15.5, 9.6, 2.6 Hz, 1H), 2.33 (dddd, *J* = 11.5, 9.5, 4.8, 2.4 Hz, 1H), 2.30 – 2.19 (m, 2H), 2.08 (ddd, *J* = 13.8, 11.7, 10.2 Hz, 0.9H), 1.99 (ddd, *J* = 14.1, 11.0, 2.4 Hz, 1H), 1.91 (ddd, *J* = 14.7, 7.7, 1.5 Hz, 1H), 1.82 (dddd, *J* = 14.7, 8.7, 5.8, 2.6 Hz, 0.8H), 1.52 (s, 0.7H), 1.52 (s, 2.2H), 1.33 (s, 0.6H), 1.32 (s, 2.3H), 0.87 (s, 6.9H), 0.86 (s, 2.0H), 0.06 (s, 0.4H), 0.06 (s, 2.4H), 0.05 (s, 2.5H), 0.04 (s, 0.6H).

$^{13}$C NMR (151 MHz, $CDCl_3$) δ 173.44, 130.03, 127.91, 127.49, 126.96, 115.07, 114.64, 85.12, 85.07, 84.75, 83.68, 83.39, 82.92, 82.16, 82.13, 81.95, 81.76, 77.50, 76.22, 75.48, 61.10, 60.76, 38.92, 36.27, 35.83, 35.79, 33.86, 32.85, 30.67, 29.92, 27.76, 27.62, 26.65, 26.12, 26.02, 25.99, 25.80, 25.75, 18.47, 18.44, -5.14, -5.17, -5.29, -5.33.

HRMS (ESI): Anal. Calcd. for $C_{25}H_{46}NO_7Si^+$ $[M+NH_4]^+$ 500.3038, found 500.3035

IR (neat): $\nu_{max}$ $(cm^{-1})$ = 3040 (w, C=*CH*), 2930 (m, CH), 2857 (m, CH), 1731 (s, C=O), 1467 (w), 1428 (w), 1379 (m), 1255 (s)

1H
CDCl3
600.13 MHz

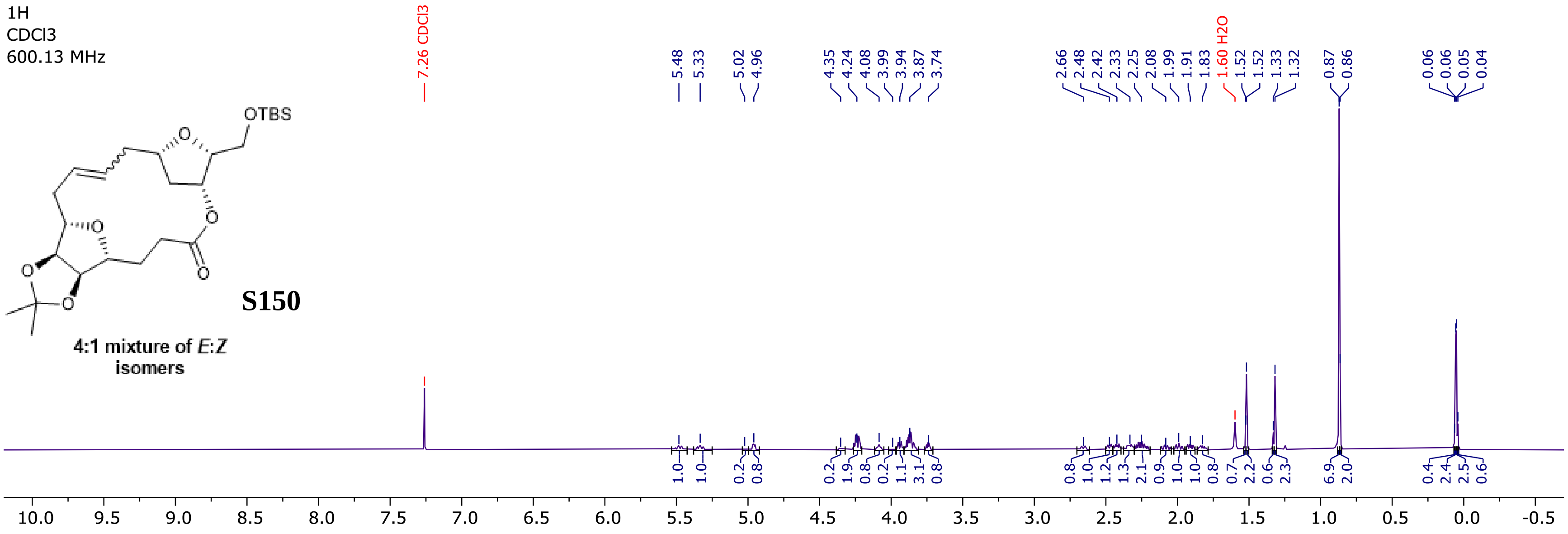


**S150**

13C
CDCl3
150.92 MHz

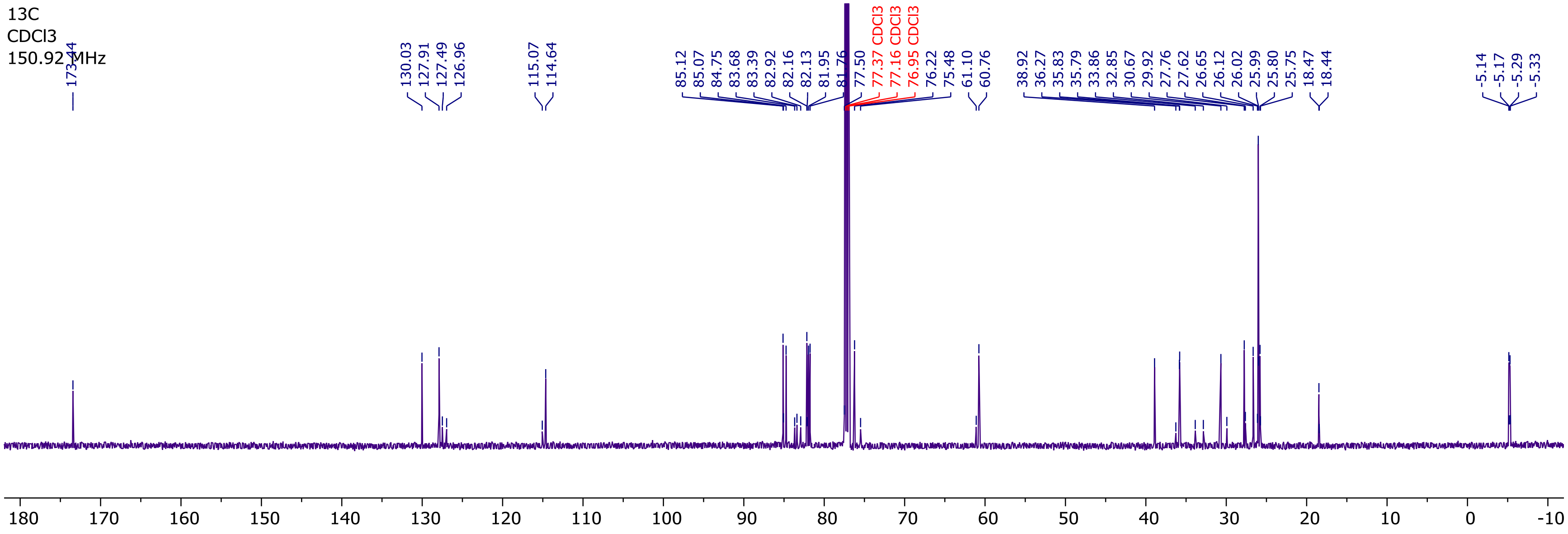

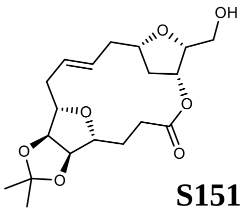

**S151**

To a rt, stirred solution of **S150** (178.6 mg, 0.38 mmol, 1.0 eq.) in a mixture of THF (4 mL) and pyridine (2 mL), was added HF-Pyridine (70% (w/w) HF, 0.50 mL, 550 mg HF, 28 mmol HF, 74 eq. HF) dropwise over 5 min. The reaction mixture was stirred at rt for 22 h. After this time, starting material was consumed as monitored by TLC analysis. The reaction mixture was quenched by slowly pipetting it onto saturated aqueous $NaHCO_3$ (35 mL) [**caution!** $CO_2$(g) evolved] over 5 min. After evolution of $CO_2$(g) had stopped, the aqueous layer was extracted with $Et_2O$ (1x 15 mL) and $CH_2Cl_2$ (2x 35 mL). The combined organic layers were dried ($MgSO_4$), filtered, and solvent was removed in vacuo. The crude product was purified via flash column chromatography (7:1.5:1.5 Hexanes/$Et_2O$/Acetone) on $AgNO_3$ impregnated silica gel. Appropriate fractions were pooled, and solvent was removed in vacuo to yield pure E isomer **S151** (73.5 mg, 54%) and a mixture of E and Z isomers (26 mg, 19%) as colorless oils.

**Analytical Data for S151:**

$R_f$ = 0.21 (3:1 Hexanes/Acetone)

$[\alpha]_D^{20}$ = +48° (c = 0.28, $CH_2Cl_2$)

$^1$H NMR (601 MHz, $CDCl_3$) δ 5.53 (dddd, *J* = 15.0, 10.9, 3.9, 1.8 Hz, 1H), 5.38 – 5.30 (m, 1H), 4.98 (ddd, *J* = 7.1, 4.4, 2.3 Hz, 1H), 4.25 (dd, *J* = 6.7, 3.2 Hz, 1H), 4.21 (t, *J* = 6.6 Hz, 1H), 4.10 (tdd, *J* = 8.1, 4.1, 2.3 Hz, 1H), 3.98 – 3.89 (m, 2H), 3.87 – 3.78 (m, 3H), 2.69 (ddt, *J* = 14.2, 4.1, 2.0 Hz, 1H), 2.51 (ddt, *J* = 14.1, 4.2, 2.4 Hz, 1H), 2.49 – 2.43 (m, 1H), 2.34 (dt, *J* = 15.1, 7.7 Hz, 1H), 2.30 – 2.19 (m, 2H), 2.10 (ddd, *J* = 13.8, 11.8, 10.3 Hz, 1H), 2.04 – 1.96 (m, 2H), 1.94 – 1.73 (m, 2H), 1.52 (s, 3H), 1.32 (s, 3H).

$^{13}$C NMR (151 MHz, $CDCl_3$) δ 172.98, 130.46, 127.71, 114.84, 85.09, 84.77, 82.94, 81.98, 81.50, 77.29, 77.26, 61.58, 38.97, 36.29, 35.53, 31.77, 27.74, 27.23, 25.79.

HRMS (ESI): Anal. Calcd. for $C_{19}H_{29}O_7^+$ $[M+H]^+$ 369.19078, found 369.19063

IR (neat): $\nu_{max}$ $(cm^{-1})$ = 3433 (br, OH), 2984 (m, CH), 2929 (m, CH), 1729 (s, C=O), 1428 (m), 1379 (m), 1268 (m), 1214 (s)

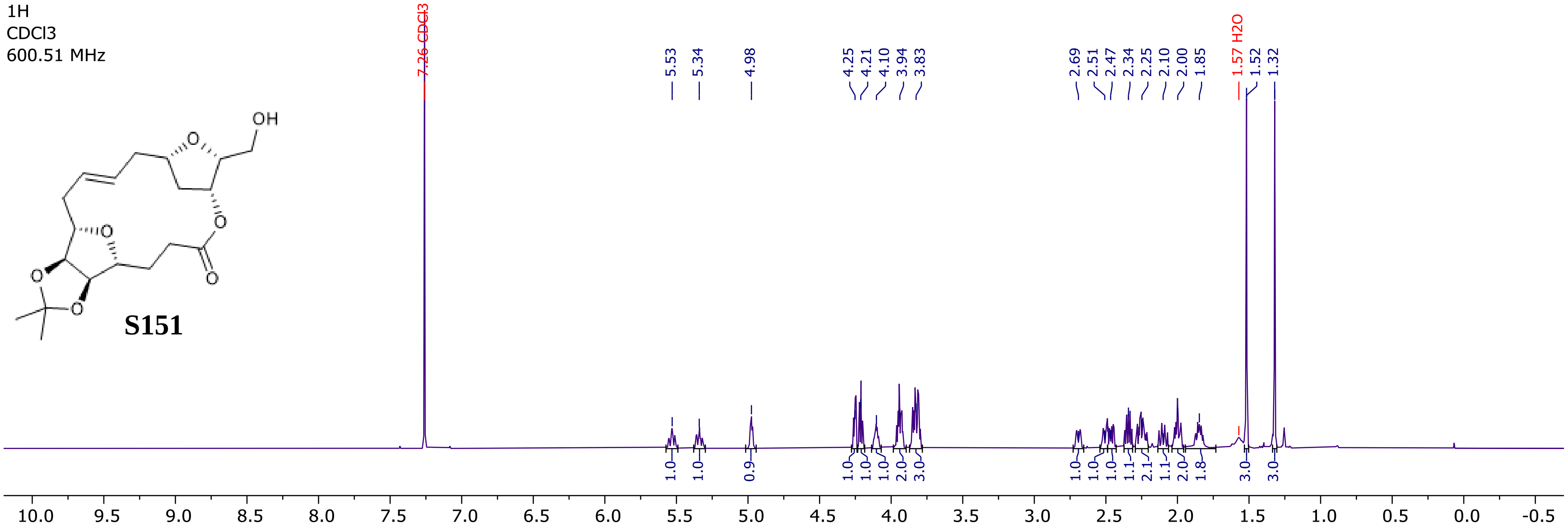
1H
CDCl3
600.51 MHz
OH
O
O
O
O
O
O
7.26 CDCl3
5.53
5.34
4.98
4.25
4.21
4.10
3.94
3.83
2.69
2.51
2.47
2.34
2.25
2.10
2.00
1.85
1.57 H2O
1.52
1.32
1.0
1.0
0.9
1.0
1.0
1.0
2.0
3.0
1.0
1.0
1.0
1.1
2.1
1.1
2.0
1.8
3.0
3.0
10.0 9.5 9.0 8.5 8.0 7.5 7.0 6.5 6.0 5.5 5.0 4.5 4.0 3.5 3.0 2.5 2.0 1.5 1.0 0.5 0.0 -0.5

**S151**

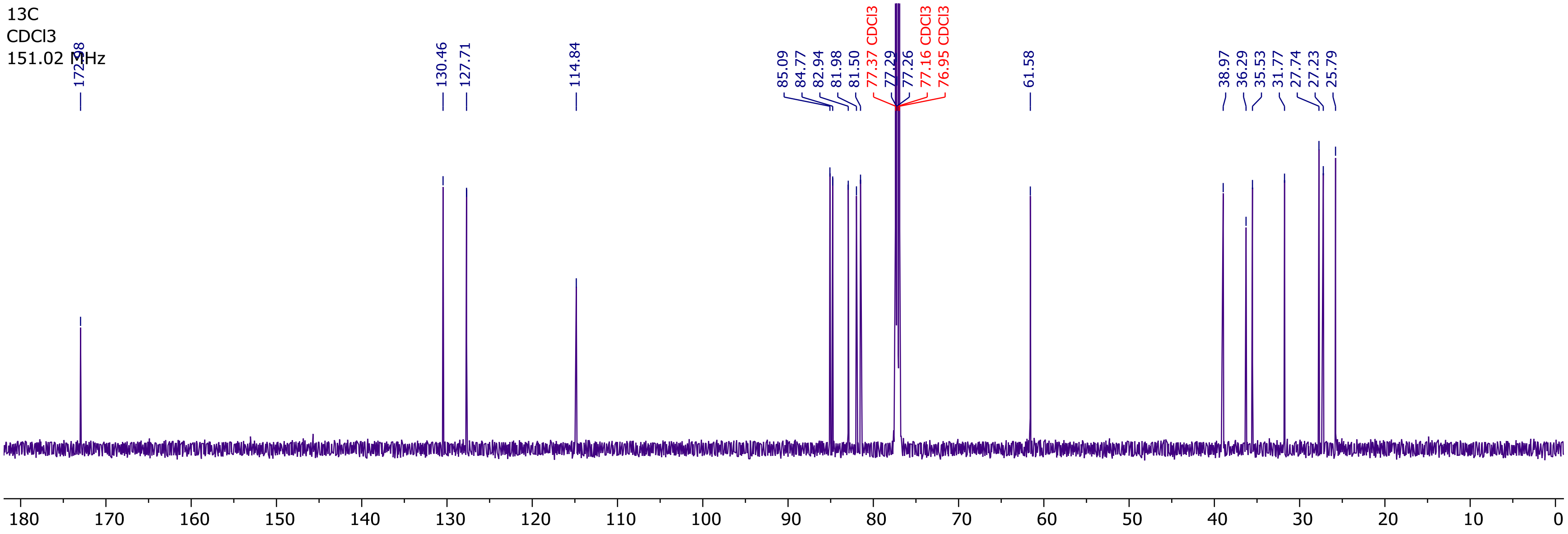
13C
CDCl3
151.02 MHz
172.98
130.46
127.71
114.84
85.09
84.77
82.94
81.98
81.50
77.37 CDCl3
77.29
77.26
77.16 CDCl3
76.95 CDCl3
61.58
38.97
36.29
35.53
31.77
27.74
27.23
25.79
180 170 160 150 140 130 120 110 100 90 80 70 60 50 40 30 20 10 0

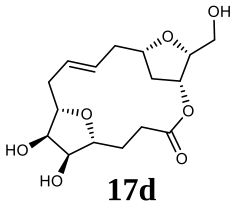


**17d**

An approximately 1.2 M solution of aqueous HCl in MeOH was prepared by slowly adding concentrated HCl (1 mL, *ca.* 12 M) to MeOH (9 mL). To a cold (0 °C), stirred solution of **S151** (3.0 mg, 0.0081 mmol, 1.0 eq.) in $CH_2Cl_2$ (0.4 mL) MeOH (0.4 mL) was added the previously prepared solution of aqueous HCl in MeOH (35 µL, *ca.* 1.2M in MeOH, 0.0042 mmol, 5.2 eq.). The reaction vessel was moved to a fridge (4 °C) and allowed to stir in the fridge for 25 h. After this time, starting material was consumed as monitored by TLC analysis, and the reaction mixture was quenched by addition of $NaHCO_3$ (20 mg, 0.238 mmol, 29.4 eq.) and allowed to stir vigorously for 5 min. After this time, the reaction mixture was filtered through a cotton plug. Then silica gel was added and the reaction mixture was concentrated onto the silica gel in vacuo. The crude product was then purified via flash column chromatography (97:3 to 92.5:7.5 $CH_2Cl_2$/MeOH). Appropriate fractions were pooled, and solvent was removed in vacuo to yield **17d** (0.95 mg, 36%) as a white film.

**Analytical Data for 17d:**

$R_f$ = 0.40 (9:1 $CH_2Cl_2$/MeOH)

$[\alpha]_D^{20}$ = +57° (c = 0.095, MeOH)

$^1$H NMR (601 MHz, MeOD) δ 5.48 (dddd, *J* = 14.9, 11.0, 3.6, 2.0 Hz, 1H), 5.31 (dddd, *J* = 15.3, 10.4, 3.6, 1.7 Hz, 1H), 4.98 – 4.93 (m, 1H), 4.12 (dddd, *J* = 11.0, 7.0, 3.8, 2.4 Hz, 1H), 3.83 – 3.75 (m, 5H), 3.68 (dd, *J* = 5.0, 1.9 Hz, 1H), 3.62 (dd, *J* = 8.5, 5.1 Hz, 1H), 2.60 (dq, *J* = 14.5, 3.3, 2.9 Hz, 1H), 2.49 (ddd, *J* = 15.8, 9.5, 2.2 Hz, 1H), 2.36 (dp, *J* = 13.8, 2.5 Hz, 1H), 2.29 – 2.17 (m, 3H), 2.06 – 1.93 (m, 3H), 1.77 (dddd, *J* = 15.4, 10.0, 5.8, 2.2 Hz, 1H).

$^{13}$C NMR (151 MHz, MeOD) δ 175.24, 131.84, 128.43, 84.55, 83.35, 81.27, 78.53, 77.58, 76.38, 75.40, 60.89, 40.53, 36.86, 36.75, 31.55, 26.93.

HRMS (ESI): Anal. Calcd. for $C_{16}H_{25}O_7^+$ $[M+H]^+$ 325.1595, found 325.1595

IR (neat): $\nu_{max}$ $(cm^{-1})$ = 3378 (br, OH), 2922 (m, CH), 2853 (m, CH), 1724 (m, C=O), 1659 (m, C=C), 1638 (m), 1433 (m), 1384 (w), 1265 (s)

**17dAc**

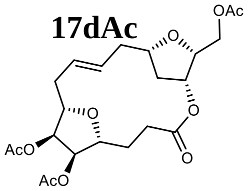


**17dAc** was prepared according to general procedure A (1.10 mg, 85%)

**Analytical Data for 17dAc:**

$R_f$ = 0.50 (2:3 Hexanes/EtOAc)

HRMS (ESI): Anal. Calcd. for $C_{22}H_{30}O_{10}Na^+$ $[M+Na]^+$ 477.1731, found 477.1701

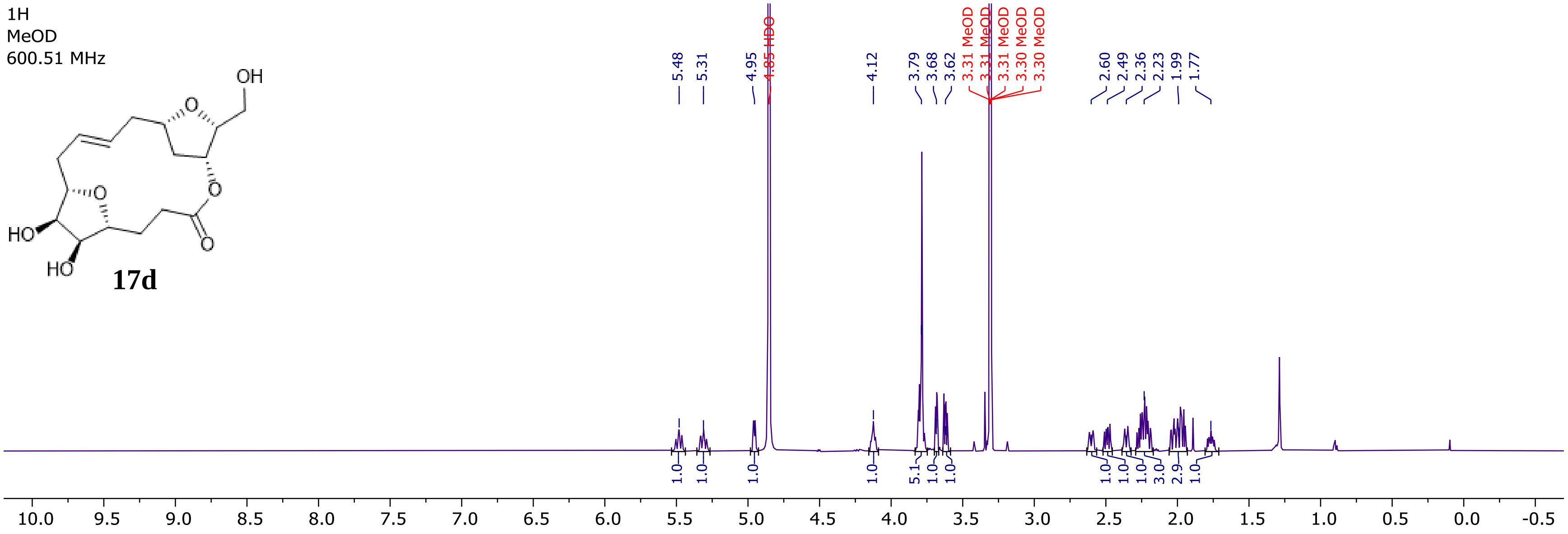
1H
MeOD
600.51 MHz
5.48
5.31
4.95
4.85 HDO
4.12
3.79
3.68
3.62
3.31 MeOD
3.31 MeOD
3.31 MeOD
3.30 MeOD
3.30 MeOD
2.60
2.49
2.36
2.23
1.99
1.77
1.0
1.0
1.0
1.0
5.1
1.0
1.0
1.0
1.0
1.0
3.0
2.9
1.0
10.0
9.5
9.0
8.5
8.0
7.5
7.0
6.5
6.0
5.5
5.0
4.5
4.0
3.5
3.0
2.5
2.0
1.5
1.0
0.5
0.0
-0.5
OH
O
O
O
HO
HO
O

**17d**

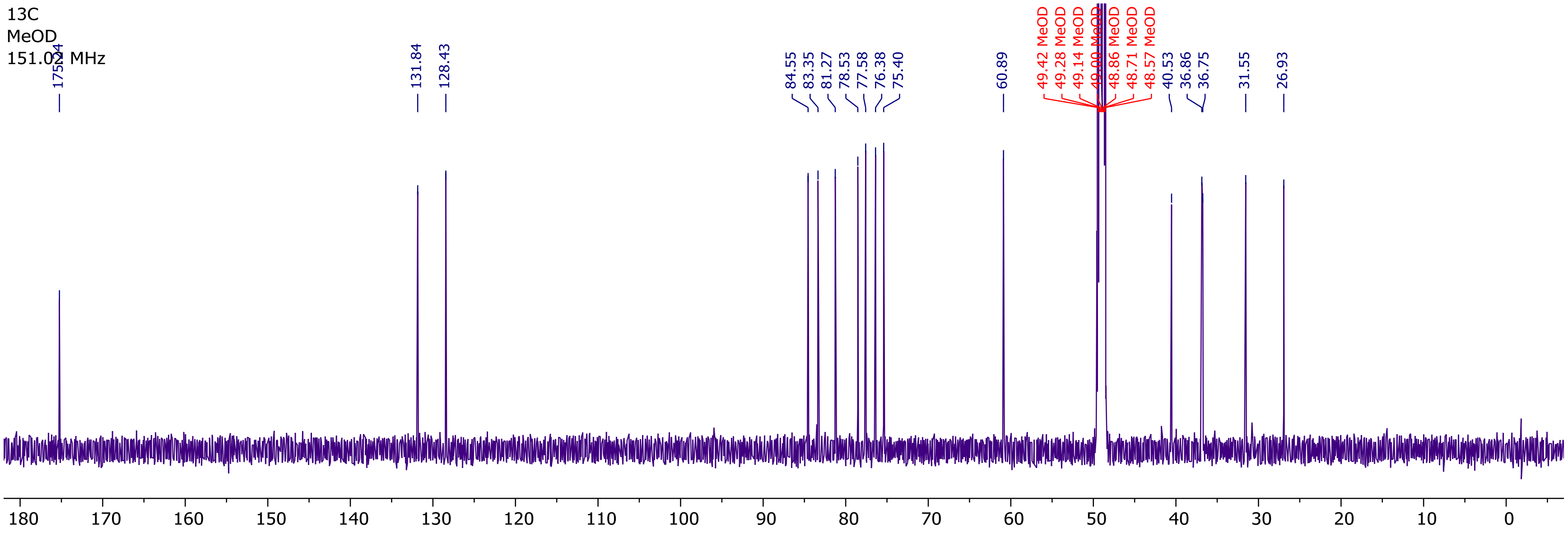
13C
MeOD
151.02 MHz
175.24
131.84
128.43
84.55
83.35
81.27
78.53
77.58
76.38
75.40
60.89
49.42 MeOD
49.28 MeOD
49.14 MeOD
49.00 MeOD
48.86 MeOD
48.71 MeOD
48.57 MeOD
40.53
36.86
36.75
31.55
26.93
180
170
160
150
140
130
120
110
100
90
80
70
60
50
40
30
20
10
0

To a rt, stirred solution of **S151** (66 mg, 0.185 mmol, 1.0 eq.) in $CH_2Cl_2$ (4.2 mL) was added $NaHCO_3$ (46.6 mg, 0.555 mmol, 3.0 eq.) followed by Dess-Martin Periodinane (103.9 mg, 0.245 mmol, 1.3 eq.). The mixture was stirred at rt for 3 h. After this time, starting material was consumed as monitored by TLC analysis. The reaction mixture was quenched with a 1:1 (v/v) saturated aqueous $NaHCO_3$-10% aqueous $Na_2S_2O_3$ (6 mL) and the biphasic mixture was stirred vigorously for 50 min at rt. The reaction mixture was diluted with $CH_2Cl_2$ (10 mL) and $H_2O$. The aqueous layer was extracted with $CH_2Cl_2$ (2x 15 mL), and the combined organic layers were dried ($Na_2SO_4$), filtered, and the solvent was removed in vacuo. The crude product was passaged through a short plug of C2 modified silica gel eluting with $Et_2O$. Solvent was removed in vacuo to yield the crude aldehyde **S152** (*ca.* 66 mg) as a colorless oil which was portioned and used immediately in the next steps without further purification.

To a solution of **S152** (21.8 mg, 0.0595 mmol, 1.0 eq.) in deoxygenated DMSO (5x freeze-pump-thaw cycles) (1 mL) was added $CrCl_2$ doped with 1 % $NiCl_2$ (w/w) (84.7 mg, 0.689 mmol, 11.6 eq.). **3b** (37.5 mg, 0.15 mmol, 2.5 eq.) was added via syringe in deoxygenated DMSO (0.5 mL + 2x 0.25 mL rinses) and the mixture was stirred for 42 h at rt. After this time, the reaction mixture was transferred to a separatory funnel, diluted with $Et_2O$ (10 mL) and 15 mL of 1:1 (v/v) $H_2O$-brine was added. The organic layer was separated, and the aqueous layer extracted with $Et_2O$ (15x 20 mL). The combined organic layers were dried ($MgSO_4$), filtered, and solvent was removed in vacuo. The crude product was purified via flash column chromatography (7.5:1.88:0.62 Hexanes/$Et_2O$/MeOH). Appropriate fractions were pooled, and solvent was removed in vacuo to yield **S153** (16.6 mg) as a white foam and a 5.8:1 mixture of diastereomers. The product was used immediately in the subsequent step.

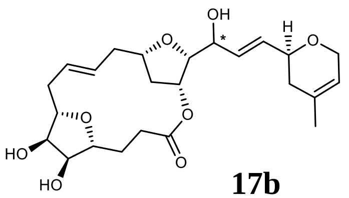


**17b**

An approximately 1.2 M solution of aqueous HCl in MeOH was prepared by slowly adding concentrated HCl (1 mL, *ca.* 12 M) to MeOH (9 mL). To a cold (0 °C), stirred solution of **S153** (16.6 mg, 0.0338 mmol, 1.0 eq.) in $CH_2Cl_2$ (1.7 mL) and MeOH (1.7 mL) was added the previously prepared solution of aqueous HCl in MeOH (141 µL, *ca.* 1.2M in MeOH, 0.169 mmol, 5.0 eq.) via microsyringe. The reaction vessel was moved to a fridge (4 °C) and allowed to stir in the fridge for 18 h 30 min. After this time, the reaction mixture was quenched by addition of $NaHCO_3$ (100 mg, 1.19 mmol, 35.2 eq.) and allowed to stir vigorously for 5 min. After this time, the reaction mixture was filtered and solvent was removed in vacuo. The crude product was then purified via flash column chromatography (49:1 to 47:3 $CH_2Cl_2$/MeOH). Approphriate fractions were pooled, and solvent was removed in vacuo to yield minor diastereomer **17b1** (1.8 mg, 2.4%, 6 steps from **1a**) major diastereomer **17b2 (**10.6 mg, 14.5%, 6 steps from **1a)** and a mixture of both isomers of **17b** (2.0 mg, 2.7%, 6 steps from **1a**) all as white foams.

**Analytical Data for 17b1 (Minor Diastereomer of 17b):**

$R_f$ = 0.55 (92.5:7.5 $CH_2Cl_2$/MeOH)

$[\alpha]_D^{20}$ = -25° (c = 0.18, MeOH)

$^1$H NMR (601 MHz, MeOD) δ 5.83 (ddd, *J* = 15.6, 5.4, 1.1 Hz, 1H), 5.68 (ddd, *J* = 15.6, 7.0, 1.4 Hz, 1H), 5.55 (dddd, *J* = 14.8, 11.1, 3.6, 1.9 Hz, 1H), 5.42 (dq, *J* = 2.7, 1.3 Hz, 1H), 5.32 (dddd, *J* = 15.4, 10.6, 3.5, 1.5 Hz, 1H), 4.84 (d, *J* = 4.0 Hz, 1H), 4.44 – 4.39 (m, 1H), 4.17 – 4.13 (m, 1H), 4.12 (p, *J* = 2.4 Hz, 2H), 4.01 (dtd, *J* = 8.7, 5.1, 1.4 Hz, 1H), 3.84 – 3.78 (m, 2H), 3.69 (dd, *J* = 5.0, 1.9 Hz, 1H), 3.64 (dd, *J* = 8.4, 5.0 Hz, 1H), 3.54 (dd, *J* = 8.6, 3.5 Hz, 1H), 2.68 – 2.61 (m, 1H), 2.49 (ddd, *J* = 15.7, 9.8, 2.3 Hz, 1H), 2.36 (dt, *J* = 13.8, 3.1 Hz, 1H), 2.28 – 2.18 (m, 3H), 2.06 – 1.91 (m, 5H), 1.78 (dddd, *J* = 15.1, 9.8, 5.4, 2.1 Hz, 1H), 1.69 (tt, *J* = 2.0, 1.1 Hz, 3H).

$^{13}$C NMR (151 MHz, MeOD) δ 173.66, 133.21, 131.11, 130.31, 128.63, 127.18, 119.30, 84.66, 83.13, 79.70, 77.07, 76.02, 75.03, 73.96, 73.13, 70.67, 65.05, 39.13, 35.58, 35.39, 35.27, 30.00, 25.31, 21.61.

HRMS (ESI): Anal. Calcd. for $C_{24}H_{25}O_8^+$ $[M+H]^+$ 451.2327, found 451.2313

IR (neat): $\nu_{max}$ $(cm^{-1})$ = 3406 (br, OH), 2911 (m, CH), 1728 (s, C=O), 1647 (m, C=C), 1429 (m), 1382 (m), 1337 (m). 1217 (m)

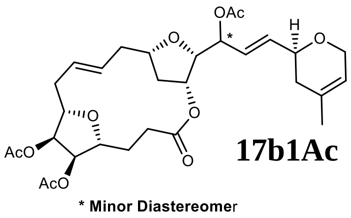


**17b1Ac**

**17b1Ac** was prepared according to general procedure A (0.55 mg, 61%)

**Analytical Data for Minor Diastereomer of 17b1Ac:**

$R_f$ = 0.50 (2:3 Hexanes/EtOAc)

HRMS (ESI): Anal. Calcd. for $C_{30}H_{44}NO_{11}^+$ $[M+NH_4]^+$ 594.2909, found 594.2883

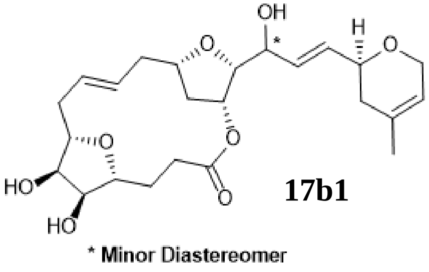
OH
*
H
O
O
O
O
HO
HO
O
* Minor Diastereomer

**17b1**

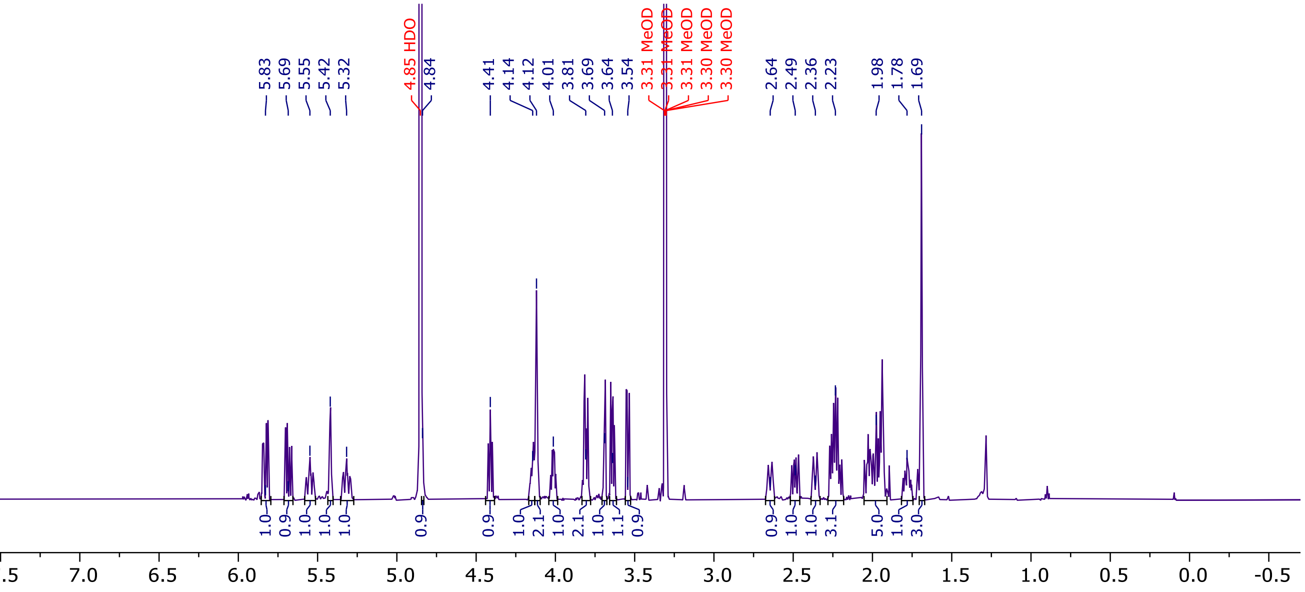
1H
MeOD
600.51 MHz
5.83
5.69
5.55
5.42
5.32
4.85 HDO
4.84
4.41
4.14
4.12
4.01
3.81
3.69
3.64
3.54
3.31 MeOD
3.31 MeOD
3.31 MeOD
3.30 MeOD
3.30 MeOD
2.64
2.49
2.36
2.23
1.98
1.78
1.69
1.0
0.9
1.0
1.0
1.0
0.9
0.9
1.0
2.1
1.0
2.1
1.0
1.1
0.9
0.9
1.0
1.0
3.1
5.0
1.0
3.0
10.0
9.5
9.0
8.5
8.0
7.5
7.0
6.5
6.0
5.5
5.0
4.5
4.0
3.5
3.0
2.5
2.0
1.5
1.0
0.5
0.0
-0.5

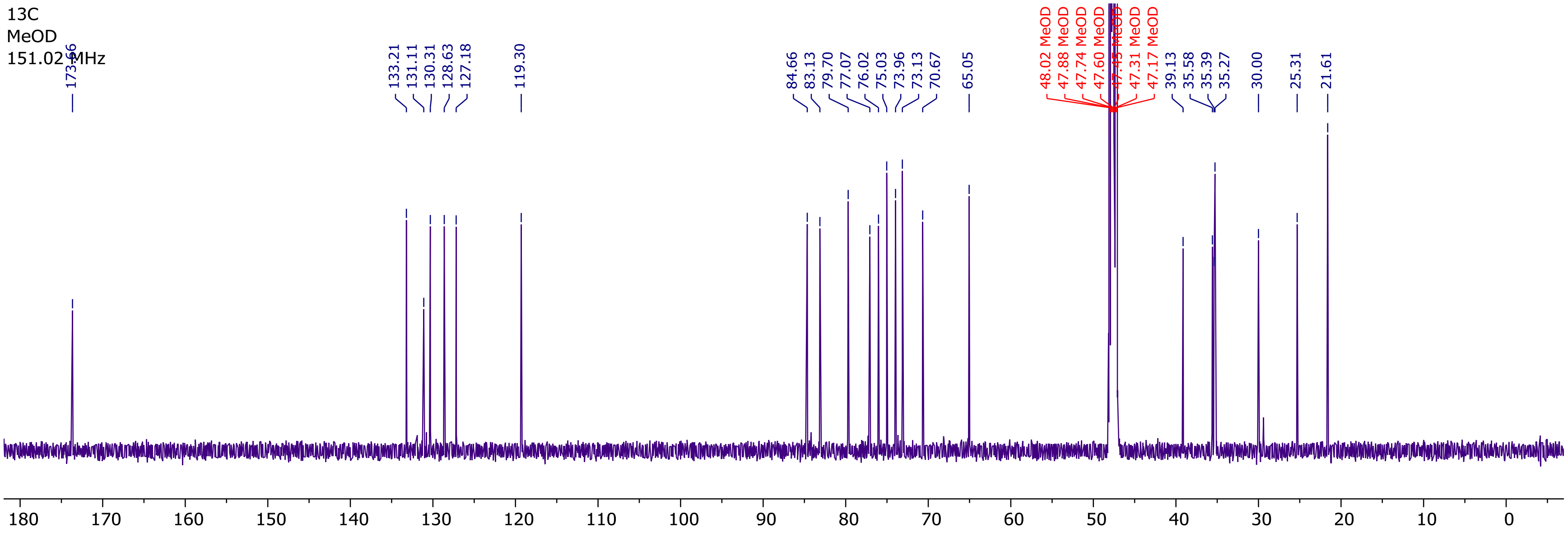
13C
MeOD
151.02 MHz
173.66
133.21
131.11
130.31
128.63
127.18
119.30
84.66
83.13
79.70
77.07
76.02
75.03
73.96
73.13
70.67
65.05
48.02 MeOD
47.88 MeOD
47.74 MeOD
47.60 MeOD
47.45 MeOD
47.31 MeOD
47.17 MeOD
39.13
35.58
35.39
35.27
30.00
25.31
21.61
180
170
160
150
140
130
120
110
100
90
80
70
60
50
40
30
20
10
0

**Analytical Data for 17b2 (Major Diastereomer of 17b):**

$R_f$ = 0.46 (92.5:7.5 $CH_2Cl_2$/MeOH)

$[\alpha]_D^{20}$ = +57° (c = 0.54, MeOH)

$^1$H NMR (601 MHz, MeOD) δ 5.95 (ddd, *J* = 15.8, 5.3, 1.3 Hz, 1H), 5.86 (ddd, *J* = 15.7, 5.5, 1.3 Hz, 1H), 5.49 (dddd, *J* = 14.9, 11.1, 3.5, 1.9 Hz, 1H), 5.44 (qq, *J* = 2.5, 1.3 Hz, 1H), 5.35 – 5.27 (m, 1H), 5.02 (dd, *J* = 6.3, 3.1 Hz, 1H), 4.37 (ddt, *J* = 8.6, 5.4, 1.1 Hz, 1H), 4.16 (dq, *J* = 4.9, 2.2 Hz, 2H), 4.11 (dddd, *J* = 9.0, 6.7, 3.7, 2.3 Hz, 1H), 4.07 (dddt, *J* = 10.1, 5.6, 3.6, 1.1 Hz, 1H), 3.85 – 3.78 (m, 2H), 3.69 (dd, *J* = 5.0, 1.9 Hz, 1H), 3.64 (dd, *J* = 8.4, 5.0 Hz, 1H), 3.47 (dd, *J* = 8.6, 3.5 Hz, 1H), 2.59 (dq, *J* = 14.5, 3.0 Hz, 1H), 2.52 (ddd, *J* = 15.7, 9.7, 2.3 Hz, 1H), 2.36 (dp, *J* = 13.8, 2.4 Hz, 1H), 2.29 – 2.20 (m, 3H), 2.11 – 2.04 (m, 1H), 2.04 – 1.91 (m, 4H), 1.79 (dddd, *J* = 15.6, 10.3, 5.8, 2.5 Hz, 1H), 1.71 (p, *J* = 1.8 Hz, 3H).

$^{13}$C NMR (151 MHz, MeOD) δ 175.36, 133.30, 132.73, 132.19, 131.66, 128.56, 120.62, 85.59, 84.50, 81.12, 78.68, 77.14, 76.43, 75.37, 75.06, 69.54, 66.61, 40.52, 37.03, 36.72, 36.37, 31.31, 26.71, 23.06.

HRMS (ESI): Anal. Calcd. for $C_{24}H_{35}O_8^+$ $[M+H]^+$ 451.2327, found 451.2319

IR (neat): $\nu_{max}$ $(cm^{-1})$ = 3407 (br, OH), 2915 (m, CH), 1722 (s, C=O), 1647 (m, C=C), 1429 (m), 1384 (m), 1337 (m) 1270 (m), 1226 (m)

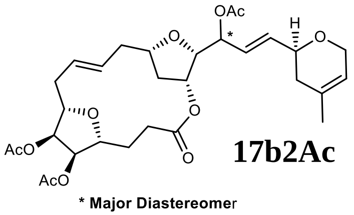


**17b2Ac**

**17b2Ac** was prepared according to general procedure A (1.14 mg, 80%)

**Analytical Data for Major Diastereomer of 17b2Ac:**

$R_f$ = 0.79 (1:3 Hexanes/EtOAc)

HRMS (ESI): Anal. Calcd. for $C_{30}H_{44}NO_{11}^+$ $[M+NH_4]^+$ 594.2909, found 594.2903

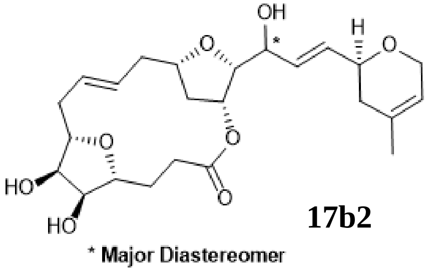
OH
H
O
O
O
O
HO
HO
O
*
* Major Diastereomer

**17b2**

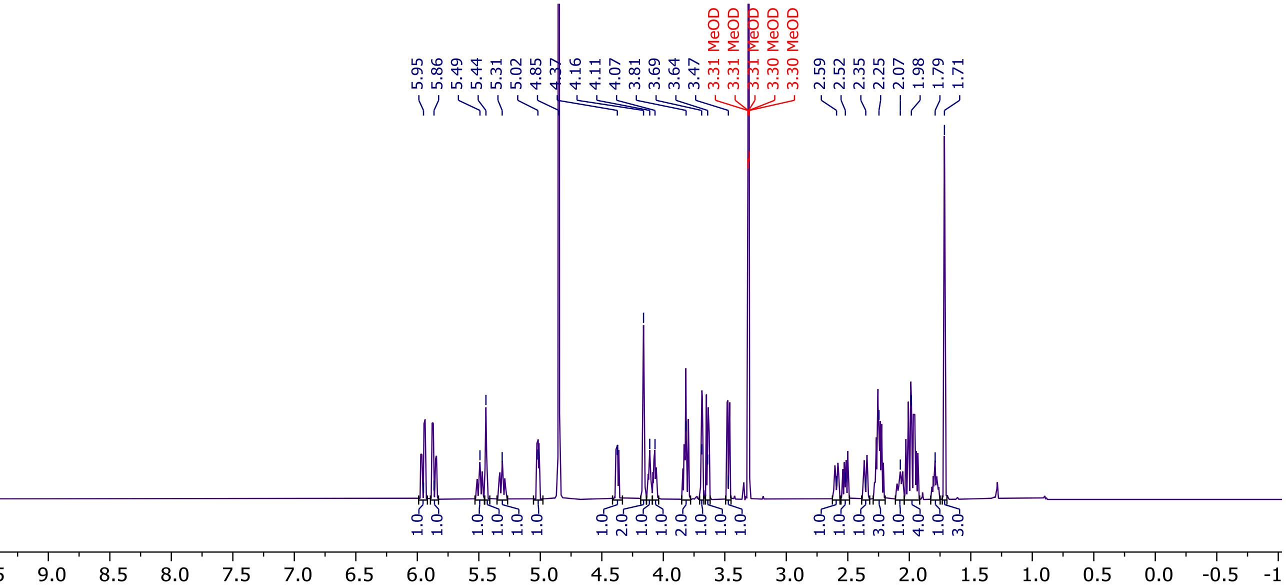
1H
MeOD
600.51 MHz
5.95
5.86
5.49
5.44
5.31
5.02
4.85
4.37
4.16
4.11
4.07
3.81
3.69
3.64
3.47
3.31 MeOD
3.31 MeOD
3.31 MeOD
3.30 MeOD
3.30 MeOD
2.59
2.52
2.35
2.25
2.07
1.98
1.79
1.71
12.5 12.0 11.5 11.0 10.5 10.0 9.5 9.0 8.5 8.0 7.5 7.0 6.5 6.0 5.5 5.0 4.5 4.0 3.5 3.0 2.5 2.0 1.5 1.0 0.5 0.0 -0.5 -1

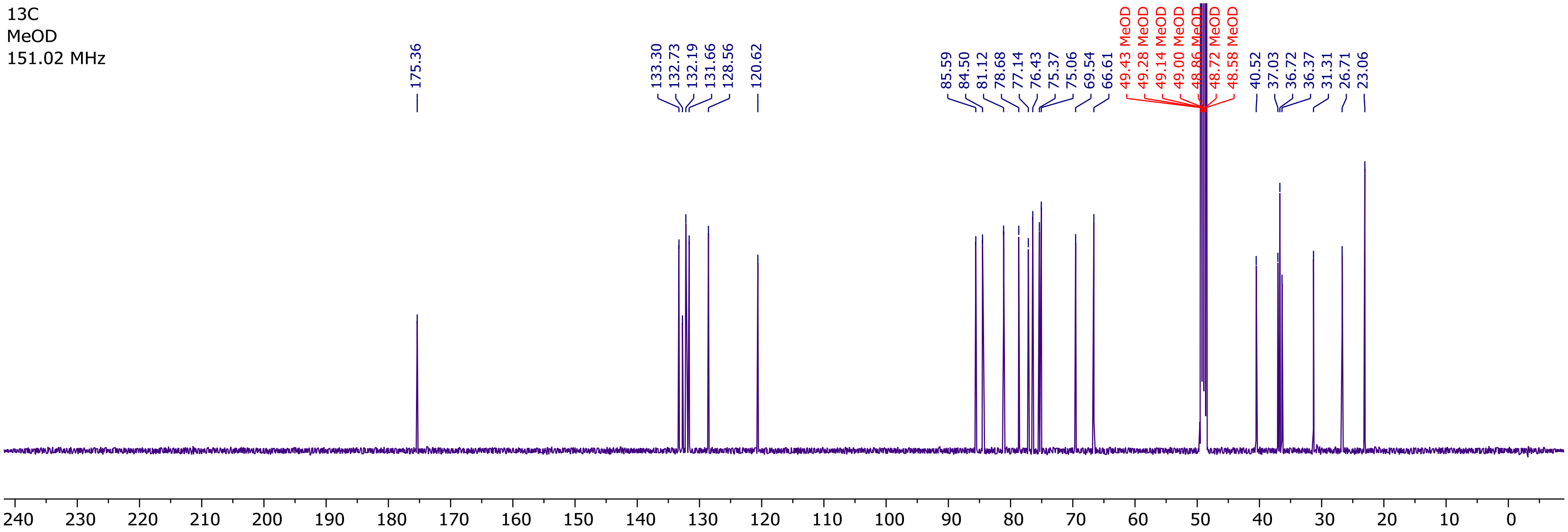
13C
MeOD
151.02 MHz
175.36
133.30
132.73
132.19
131.66
128.56
120.62
85.59
84.50
81.12
78.68
77.14
76.43
75.37
75.06
69.54
66.61
49.43 MeOD
49.28 MeOD
49.14 MeOD
49.00 MeOD
48.86 MeOD
48.72 MeOD
48.58 MeOD
40.52
37.03
36.72
36.37
31.31
26.71
23.06
240 230 220 210 200 190 180 170 160 150 140 130 120 110 100 90 80 70 60 50 40 30 20 10 0

To a solution of **S152** (21.8 mg, 0.0595 mmol, 1.0 eq.) in deoxygenated DMSO (5x freeze-pump-thaw cycles) (1 mL) was added $CrCl_2$ doped with 1 % $NiCl_2$ (w/w) (87.4 mg, 0.711 mmol, 12.0 eq.). **3a** (44.2 mg, 0.185 mmol, 3.1 eq.) was added via syringe in deoxygenated DMSO (0.5 mL + 2x 0.25 mL rinses) and the mixture was stirred for 42 h at rt. After this time, the reaction mixture was transferred to a separatory funnel, diluted with $Et_2O$ (10 mL) and 15 mL of 1:1 (v/v) $H_2O$-brine was added. The organic layer was separated, and the aqueous layer extracted with $Et_2O$ (15x 20 mL). The combined organic layers were dried ($MgSO_4$), filtered, and solvent was removed in vacuo. The crude product was purified via flash column chromatography (6.5:2.8:0.7 toluene/THF/MeOH). Appropriate fractions were pooled, and solvent was removed in vacuo to yield **S154**(14.0 mg) as a white foam and a 4.5:1 mixture of diastereomers. The product was used immediately in the subsequent step.

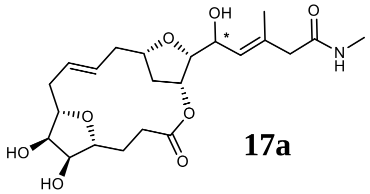


**17a**

* 4.5:1 mixture of diastereomers

An approximately 1.2 M solution of aqueous HCl in MeOH was prepared by slowly adding concentrated HCl (1 mL, *ca.* 12 M) to MeOH (9 mL). To a cold (0 °C), stirred solution of **S154** (14.0 mg, 0.0292 mmol, 1.0 eq.) in $CH_2Cl_2$ (1.5 mL) and MeOH (1.5 mL) was added the previously prepared solution of aqueous HCl in MeOH (122 µL, *ca.* 1.2M in MeOH, 0.146 mmol, 5.0 eq.) via microsyringe. The reaction vessel was moved to a fridge (4 °C) and allowed to stir in the fridge for 18 h 30 min. After this time, the reaction mixture was quenched by addition of $NaHCO_3$ (100 mg, 1.19 mmol, 40.8 eq.) and allowed to stir vigorously for 5 min. After this time, the reaction mixture was filtered and solvent was removed in vacuo. The crude product was then purified via flash column chromatography (9:1 $CH_2Cl_2$/MeOH). Appropriate fractions were pooled, and solvent was removed in vacuo to yield **17a (**12.0 mg, 16.6%, 6 steps from **1a)** a white foam.

**Analytical Data for 17a:**

$R_f$ = 0.26 (9:1 $CH_2Cl_2$/MeOH)

$^1$H NMR (601 MHz, MeOD) δ 5.55 (dddd, *J* = 14.7, 9.1, 3.6, 1.8 Hz, 0.2H), 5.50 – 5.40 (m, 1.8H), 5.36 – 5.27 (m, 1H), 5.05 – 5.00 (m, 0.8H), 4.82 (dd, *J* = 6.6, 3.6 Hz, 0.2H), 4.71 (t, *J* = 8.7 Hz, 0.8H), 4.64 (dd, *J* = 9.8, 8.6 Hz, 0.2H), 4.13 (dtdd, *J* = 9.1, 6.7, 4.0, 1.8 Hz, 1H), 3.87 – 3.77 (m, 2H), 3.72 – 3.67 (m, 1H), 3.67 – 3.60 (m, 1.2H), 3.56 (dd, *J* = 8.7, 3.6 Hz, 0.8H), 2.99 (dd, *J* = 14.9, 1.2 Hz, 0.8H), 2.93 (d, *J* = 14.9 Hz, 0.8H), 2.90 – 2.83 (m, 0.4H), 2.73 (s, 2.6H), 2.68 (s, 0.4H), 2.58 – 2.50 (m, 1.8H), 2.42 – 2.33 (m, 1.2H), 2.32 – 2.19 (m, 3H), 2.06 – 1.88 (m, 3H), 1.80 (d, *J* = 1.4 Hz, 0.4H), 1.80 (m, 1H), 1.79 (d, *J* = 1.4 Hz, 2.6H).

$^{13}$C NMR (151 MHz, MeOD) δ 175.33, 174.92, 174.27, 173.96, 136.38, 134.94, 131.87, 131.83, 131.72, 128.55, 128.21, 127.85, 86.29, 85.77, 84.49, 81.08, 80.94, 78.52, 78.48, 77.58, 76.87, 76.40, 76.37, 75.32, 75.23, 67.95, 66.02, 47.72, 40.53, 40.47, 37.13, 36.95, 36.59, 31.22, 31.20, 26.62, 26.50, 26.45, 26.43, 17.29, 17.21.

HRMS (ESI): Anal. Calcd. for $C_{22}H_{34}NO_8^+$ $[M+H]^+$ 440.2279, found 440.2269

IR (neat): $\nu_{max}$ $(cm^{-1})$ = 3355 (br, OH), 2985 (m, CH), 2905 (m, CH), 1722 (s, C=O), 1636 (s, C=C), 1554 (m), 1420 (m), 1384 (m), 1336 (m), 1271 (m), 1220 (m)

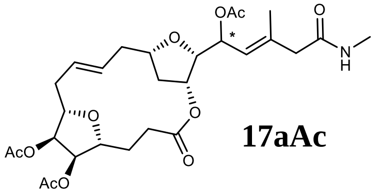


**17aAc**

* 4.5:1 mixture of diastereomers

**17aAc** was prepared according to general procedure A (1.15 mg, 84%)

**Analytical Data for 17aAc:**

$R_f$ = 0.33 (EtOAc)

HRMS (ESI): Anal. Calcd. for $C_{28}H_{43}N_2O_{11}^+$ $[M+NH_4]^+$ 583.2861, found 583.2837

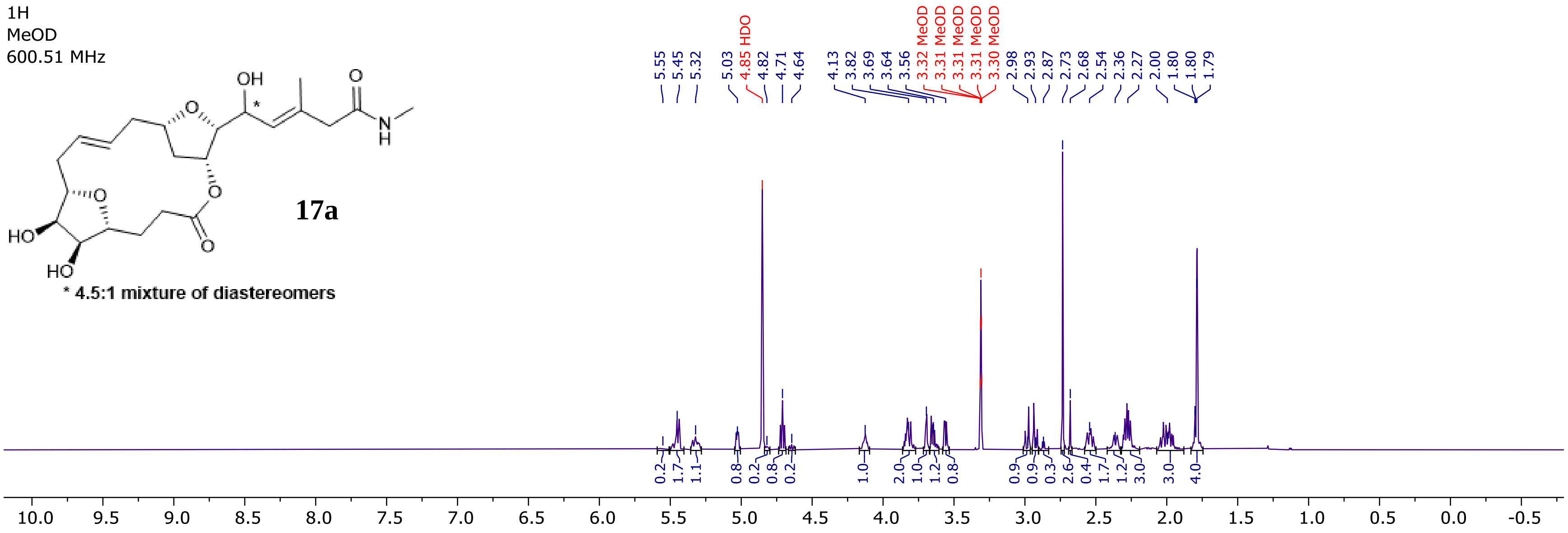
1H
MeOD
600.51 MHz
17a
* 4.5:1 mixture of diastereomers
5.55 5.45 5.32 5.03 4.85 HDO 4.82 4.71 4.64 4.13 3.82 3.69 3.64 3.56 3.32 MeOD 3.31 MeOD 3.31 MeOD 3.31 MeOD 3.30 MeOD 2.98 2.93 2.87 2.73 2.68 2.54 2.36 2.27 2.00 1.80 1.80 1.79
0.2 1.7 1.1 0.8 0.2 0.8 0.2 1.0 2.0 1.0 1.2 0.8 0.9 0.9 0.3 2.6 0.4 1.7 1.2 3.0 3.0 4.0
10.0 9.5 9.0 8.5 8.0 7.5 7.0 6.5 6.0 5.5 5.0 4.5 4.0 3.5 3.0 2.5 2.0 1.5 1.0 0.5 0.0 -0.5

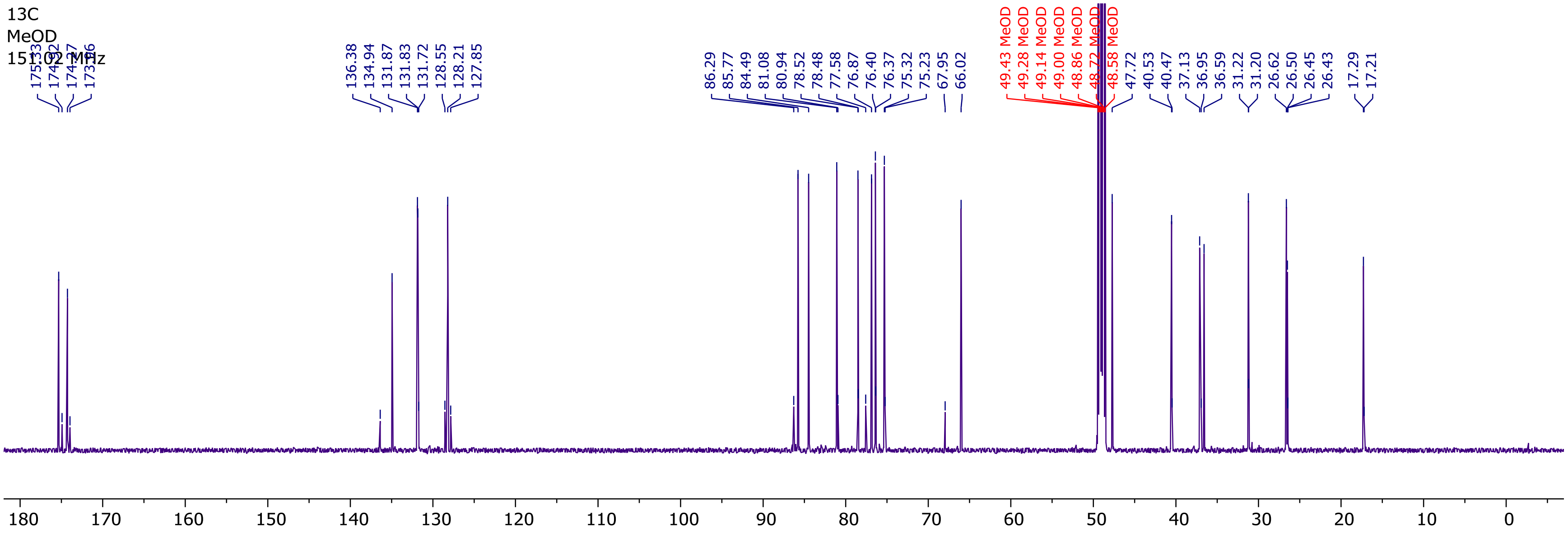
13C
MeOD
151.02 MHz
175.33 174.32 174.27 173.96
136.38 134.94 131.87 131.83 131.72 128.55 128.21 127.85
86.29 85.77 84.49 81.08 80.94 78.52 78.48 77.58 76.87 76.40 76.37 75.32 75.23 67.95 66.02
49.43 MeOD 49.28 MeOD 49.14 MeOD 49.00 MeOD 48.86 MeOD 48.72 MeOD 48.58 MeOD 47.72 40.53 40.47 37.13 36.95 36.59 31.22 31.20 26.62 26.50 26.45 26.43 17.29 17.21
180 170 160 150 140 130 120 110 100 90 80 70 60 50 40 30 20 10 0

To a solution of **S152** (21.8 mg, 0.0595 mmol, 1.0 eq.) in deoxygenated DMSO (5x freeze-pump-thaw cycles) (2 mL) was added $CrCl_2$ doped with 1 % $NiCl_2$ (w/w) (84.4 mg, 0.687 mmol, 11.5 eq.). **3c** (27.3 µL, 44.2 mg, 0.185 mmol, 3.1 eq.) was added via microsyringe and the mixture was stirred for 42 h at rt. After this time, the reaction mixture was transferred to a separatory funnel, diluted with $Et_2O$ (10 mL) and 15 mL of 1:1 (v/v) $H_2O$-brine was added. The organic layer was separated, and the aqueous layer extracted with $Et_2O$ (15x 20 mL). The combined organic layers were dried ($MgSO_4$), filtered, and solvent was removed in vacuo. The crude product was purified via flash column chromatography (49:1 $CH_2Cl_2$/EtOH). Appropriate fractions were pooled, and solvent was removed in vacuo to yield **S155** (17.1 mg) as a white foam and a near 1:1 mixture of diastereomers. The product was used immediately in the subsequent step.

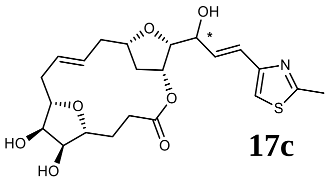


**17c**

An approximately 1.2 M solution of aqueous HCl in MeOH was prepared by slowly adding concentrated HCl (1 mL, *ca.* 12 M) to MeOH (9 mL). To a cold (0 °C), stirred solution of **S155** (17.1 mg, 0.0348 mmol, 1.0 eq.) in $CH_2Cl_2$ (1.8 mL) and MeOH (1.8 mL) was added the previously prepared solution of aqueous HCl in MeOH (145 µL, *ca.* 1.2M in MeOH, 0.174 mmol, 5.0 eq.) via microsyringe. The reaction vessel was moved to a fridge (4 °C) and allowed to stir in the fridge for 18 h 30 min. After this time, the reaction mixture was quenched by addition of $NaHCO_3$ (100 mg, 1.19 mmol, 34.2 eq.) and allowed to stir vigorously for 5 min. After this time, the reaction mixture was filtered and solvent was removed in vacuo. The crude product was then purified via flash column chromatography (97:3 to 95.5:4.5 $CH_2Cl_2$/MeOH). Appropriate fractions were pooled, and solvent was removed in vacuo to yield the faster eluting diastereomer **17c1 (**5.0 mg, 6.7%, 6 steps from **1a)** the slower eluting diastereomer **17c2** (5.2 mg, 7.0%, 6 steps from **1a**) and a mixture of both diastereomers of **17c** (5.0 mg, 6.7%, 6 steps from **1a**) all as white foams.

**Analytical Data for 17c1 (Faster Eluting Diastereomer of 17c):**

$R_f$ = 0.36 (92.5:7.5 $CH_2Cl_2$/MeOH)

$[\alpha]_D^{20}$ = -39° (c = 0.36, MeOH)

$^1$H NMR (601 MHz, MeOD) δ 7.21 (s, 1H), 6.68 (dd, *J* = 15.7, 1.3 Hz, 1H), 6.45 (dd, *J* = 15.7, 6.2 Hz, 1H), 5.57 (dddd, *J* = 14.9, 11.1, 3.6, 1.9 Hz, 1H), 5.38 – 5.26 (m, 1H), 4.89 (dd, *J* = 6.7, 3.6 Hz, 1H), 4.58 (ddd, *J* = 8.6, 6.2, 1.4 Hz, 1H), 4.16 (dddd, *J* = 9.1, 6.8, 3.9, 2.4 Hz, 1H), 3.86 – 3.77 (m, 2H), 3.69 (dd, *J* = 5.0, 1.9 Hz, 1H), 3.62 (ddd, *J* = 16.3, 8.5, 4.3 Hz, 2H), 2.68 (s, 3H), 2.67 – 2.64 (m, 1H), 2.52 (ddd, *J* = 16.2, 9.2, 1.8 Hz, 1H), 2.37 (dt, *J* = 13.8, 3.0 Hz, 1H), 2.30 – 2.21 (m, 3H), 2.09 – 1.95 (m, 3H), 1.79 (dddd, *J* = 14.0, 8.7, 4.9, 1.9 Hz, 1H).

$^{13}$C NMR (151 MHz, MeOD) δ 175.08, 168.61, 154.16, 131.74, 131.35, 128.57, 125.44, 116.84, 86.18, 84.55, 81.10, 78.45, 77.54, 76.43, 75.34, 71.92, 40.56, 36.97, 36.90, 31.38, 26.71, 18.74.

HRMS (ESI): Anal. Calcd. for $C_{22}H_{30}NO_7S^+$ $[M+H]^+$ 452.1738, found 452.1730

IR (neat): $\nu_{max}$ $(cm^{-1})$ = 3398 (br, OH), 3058 (w, C=*CH*), 1726 (s. C=O), 1646 (m, C=C), 1505 (m), 1429 (m), 1329 (m), 1335 (m), 1266 (m).

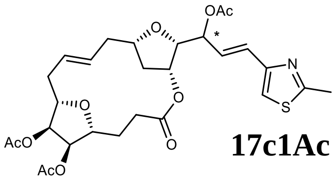


**17c1Ac**

* Faster Eluting Diastereomer

**17c1Ac** was prepared according to general procedure A (1.34 mg, quant.)

**Analytical Data for 17c1Ac:**

$R_f$ = 0.29 (1:1 Hexanes/EtOAc)

$[\alpha]_D^{20}$ = +35° (c = 0.26, $C_6D_6$)

$^1$H NMR (601 MHz, $C_6D_6$) δ 6.91 (dd, *J* = 15.5, 6.8 Hz, 1H), 6.86 (d, *J* = 15.5 Hz, 1H), 6.40 (s, 1H), 6.31 (dd, *J* = 8.8, 6.8 Hz, 1H), 5.74 – 5.66 (m, 1H), 5.09 – 5.02 (m, 1H), 5.01 (dd, *J* = 5.1, 2.6 Hz, 1H), 4.91 (dd, *J* = 6.5, 3.4 Hz, 1H), 4.83 (dd, *J* = 8.4, 5.1 Hz, 1H), 4.11 (dt, *J* = 8.3, 4.8 Hz, 1H), 3.85 – 3.79 (m, 2H), 3.67 (dd, *J* = 8.8, 3.4 Hz, 1H), 2.69 (dt, *J* = 14.6, 2.8 Hz, 1H), 2.22 (s, 3H), 2.22 – 2.17 (m, 2H), 2.13 (dq, *J* = 12.4, 3.6 Hz, 1H), 1.99 (ddd, *J* = 14.7, 6.8, 1.0 Hz, 1H), 1.93 – 1.83 (m, 2H), 1.77 (s, 4H), 1.73 – 1.67 (m, 1H), 1.66 (s, 3H), 1.65 (s, 3H), 1.47 – 1.39 (m, 1H).

$^{13}$C NMR (151 MHz, $C_6D_6$) δ 172.17, 169.60, 169.45, 169.40, 165.90, 153.30, 129.74, 128.47, 127.52, 127.18, 116.01, 82.61, 81.17, 77.70, 77.27, 76.14, 75.33, 73.73, 73.48, 38.70, 36.64, 36.07, 29.50, 25.40, 20.96, 20.31, 20.16, 18.89.

HRMS (ESI): Anal. Calcd. for $C_{28}H_{36}NO_{10}S^+$ $[M+H]^+$ 578.2054, found 578.2032

IR (neat): $\nu_{max}$ $(cm^{-1})$ = 2922 (m, CH), 2857 (m, CH), 1740 (vs, CO), 1646 (w, C=C), 1432 (m), 1373 (m), 1242 (vs, CO).

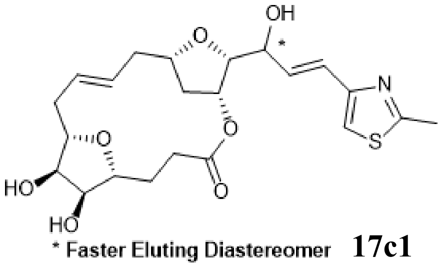


* Faster Eluting Diastereomer **17c1**

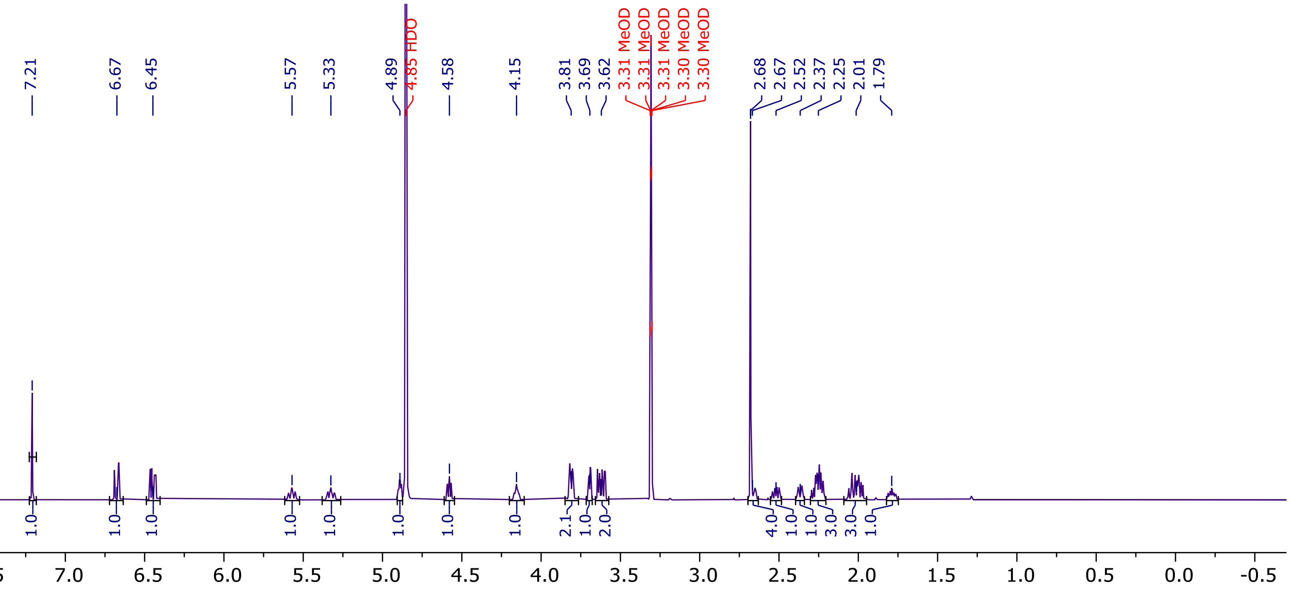


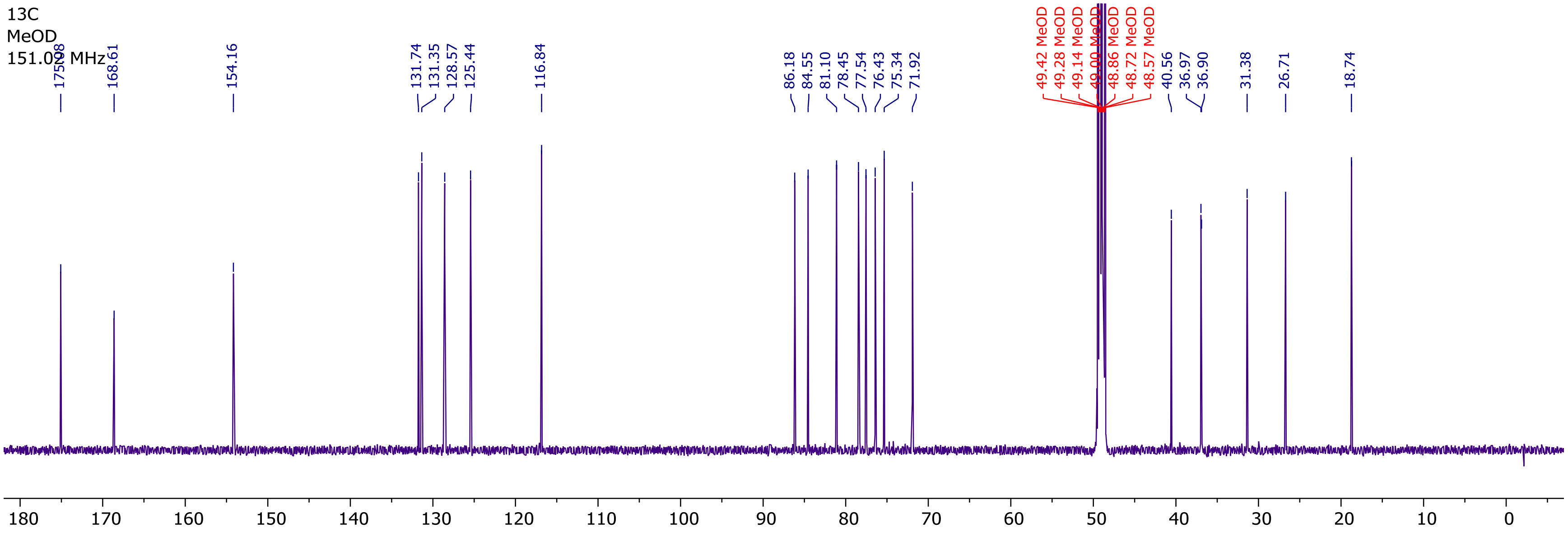

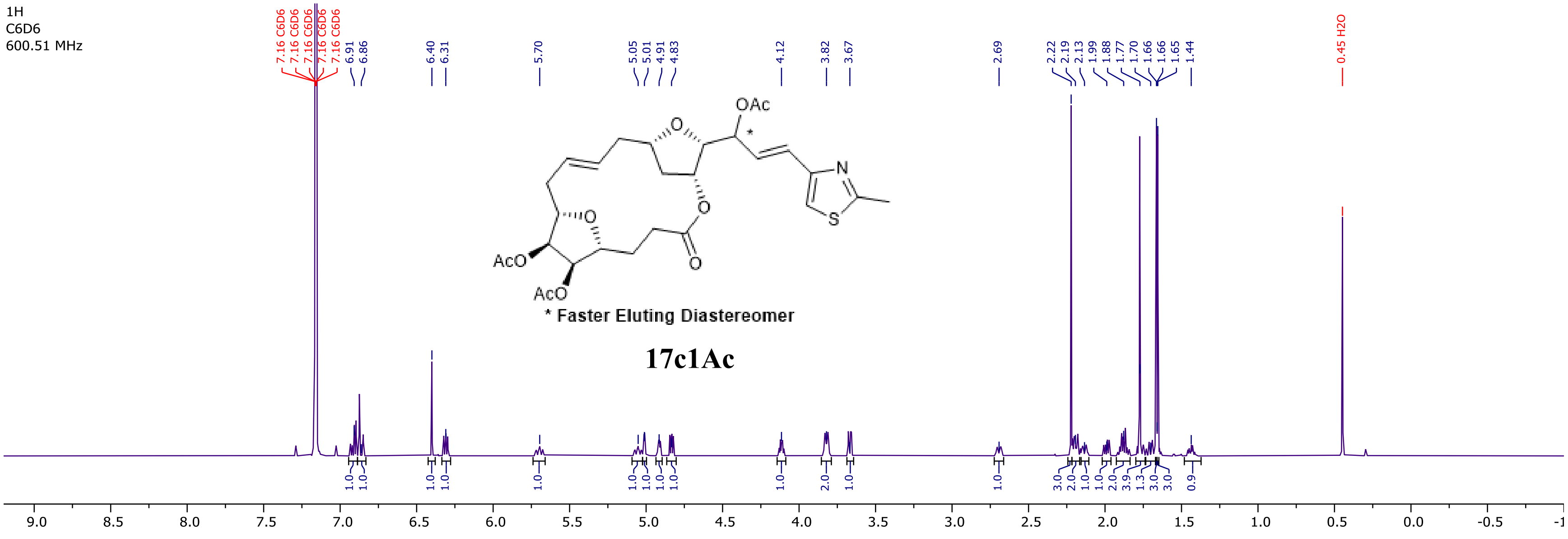
1H
C6D6
600.51 MHz
7.16 C6D6
6.91
6.86
6.40
6.31
5.70
5.05
5.01
4.91
4.83
4.12
3.82
3.67
2.69
2.22
2.19
2.13
1.99
1.88
1.77
1.70
1.66
1.66
1.65
1.44
0.45 H2O
OAc
N
S
O
AcO
AcO
* Faster Eluting Diastereomer
17c1Ac

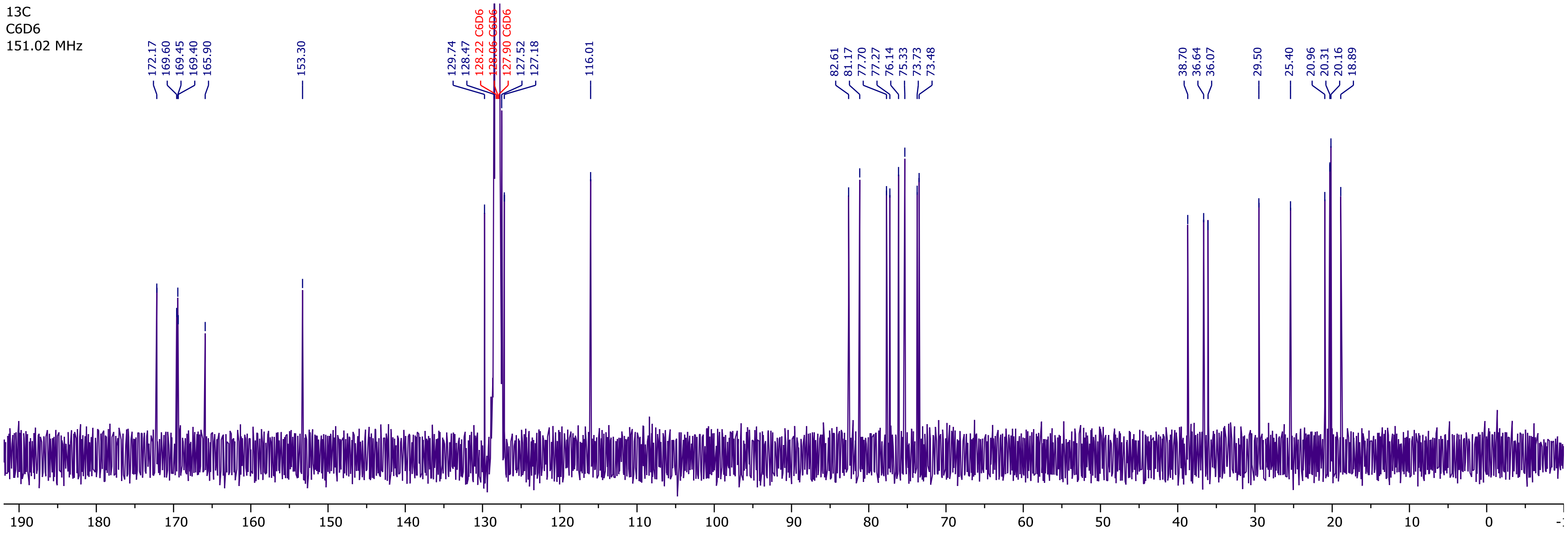
13C
C6D6
151.02 MHz
172.17
169.60
169.45
169.40
165.90
153.30
129.74
128.47
128.22 C6D6
128.06 C6D6
127.90 C6D6
127.52
127.18
116.01
82.61
81.17
77.70
77.27
76.14
75.33
73.73
73.48
38.70
36.64
36.07
29.50
25.40
20.96
20.31
20.16
18.89

**Analytical Data for 17c2 (Slower Eluting Diastereomer of 17c):**

$R_f$ = 0.35 (92.5:7.5 $CH_2Cl_2$/MeOH)

$[\alpha]_D^{20}$ = +160° (c = 0.50, MeOH)

$^1$H NMR (601 MHz, MeOD) δ 7.20 (s, 1H), 6.72 – 6.65 (m, 2H), 5.52 (dddd, *J* = 14.9, 11.1, 3.6, 1.9 Hz, 1H), 5.31 (dddd, *J* = 15.4, 10.4, 3.7, 1.7 Hz, 1H), 5.06 (ddd, *J* = 7.0, 3.6, 1.1 Hz, 1H), 4.54 (ddd, *J* = 8.5, 2.7, 1.4 Hz, 1H), 4.13 (dddd, *J* = 9.1, 6.9, 3.8, 2.3 Hz, 1H), 3.86 – 3.79 (m, 2H), 3.69 (dd, *J* = 5.0, 1.9 Hz, 1H), 3.63 (dd, *J* = 8.5, 5.0 Hz, 1H), 3.56 (dd, *J* = 8.6, 3.6 Hz, 1H), 2.69 (s, 3H), 2.61 (ddt, *J* = 14.4, 5.8, 3.3 Hz, 1H), 2.54 (ddd, *J* = 16.3, 9.7, 2.2 Hz, 1H), 2.36 (dp, *J* = 13.7, 2.4 Hz, 1H), 2.26 (dddq, *J* = 14.0, 7.3, 5.2, 2.4 Hz, 3H), 2.04 – 1.94 (m, 3H), 1.80 (dddd, *J* = 14.7, 11.5, 6.5, 2.5 Hz, 1H).

$^{13}$C NMR (151 MHz, MeOD) δ 175.38, 168.31, 154.65, 134.52, 131.66, 128.58, 124.11, 115.99, 85.60, 84.52, 81.15, 78.71, 77.16, 76.43, 75.38, 69.83, 40.52, 37.03, 36.41, 31.38, 26.76, 18.73.

HRMS (ESI): Anal. Calcd. for $C_{22}H_{30}NO_7S^+$ $[M+H]^+$ 452.1738, found 452.1732

IR (neat): $\nu_{max}$ $(cm^{-1})$ = 3395 (br, OH), 2905 (m, CH), 1723 (s, C=O), 1668 (m, C=C), 1508 (w), 1430 (m), 1386 (m), 1335 (m), 1272 (m), 1223 (m)

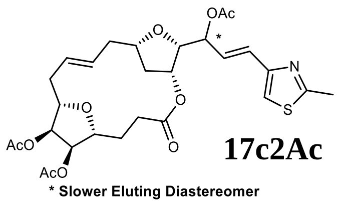


**17c2Ac**

**17c2Ac** was prepared according to general procedure A (1.18 mg, 94%)

**Analytical Data for 17c2Ac:**

$R_f$ = 0.67 (1:3 Hexanes/EtOAc)

$[\alpha]_D^{20}$ = +105° (c = 0.35, $C_6D_6$)

$^1$H NMR (601 MHz, $C_6D_6$) δ 7.23 (dd, *J* = 15.5, 6.9 Hz, 1H), 6.86 (dd, *J* = 15.5, 1.2 Hz, 1H), 6.33 (s, 1H), 6.22 (ddd, *J* = 8.2, 7.0, 1.2 Hz, 1H), 5.68 (dddd, *J* = 14.9, 11.1, 3.7, 1.8 Hz, 1H), 5.10 – 5.00 (m, 3H), 4.77 (dd, *J* = 8.5, 5.0 Hz, 1H), 4.10 (ddd, *J* = 8.4, 6.5, 4.1 Hz, 1H), 3.82 (dt, *J* = 12.2, 2.3 Hz, 1H), 3.77 (tt, *J* = 7.3, 2.3 Hz, 1H), 3.66 (dd, *J* = 8.2, 3.7 Hz, 1H), 2.68 – 2.59 (m, 1H), 2.38 (ddd, *J* = 15.9, 9.4, 2.5 Hz, 1H), 2.27 (s, 3H), 2.20 – 2.14 (m, 1H), 2.14 – 2.05 (m, 2H), 2.02 (ddd, *J* = 14.6, 7.3, 1.4 Hz, 1H), 1.93 (ddd, *J* = 14.5, 8.5, 6.9 Hz, 1H), 1.81 (ddd, *J* = 13.7, 12.1, 10.4 Hz, 1H), 1.71 – 1.59 (m, 11H).

$^{13}$C NMR (151 MHz, $C_6D_6$) δ 172.24, 169.45, 169.31, 168.90, 165.52, 153.58, 129.86, 129.51, 128.48, 126.79, 115.76, 82.53, 81.24, 78.44, 77.72, 75.65, 75.36, 74.04, 71.57, 38.87, 36.31, 35.67, 31.17, 26.39, 20.62, 20.32, 20.16, 18.92.

HRMS (ESI): Anal. Calcd. for $C_{28}H_{36}NO_{10}S^{+}$ $[M+H]^{+}$ 578.2054, found 578.2030

IR (neat): $\nu_{max}$ $(cm^{-1})$ = 2922 (m, CH), 2866 (m, CH), 1739 (vs, CO), 1647 (w, C=C), 1432 (m), 1373 (m), 1239 (vs, CO).

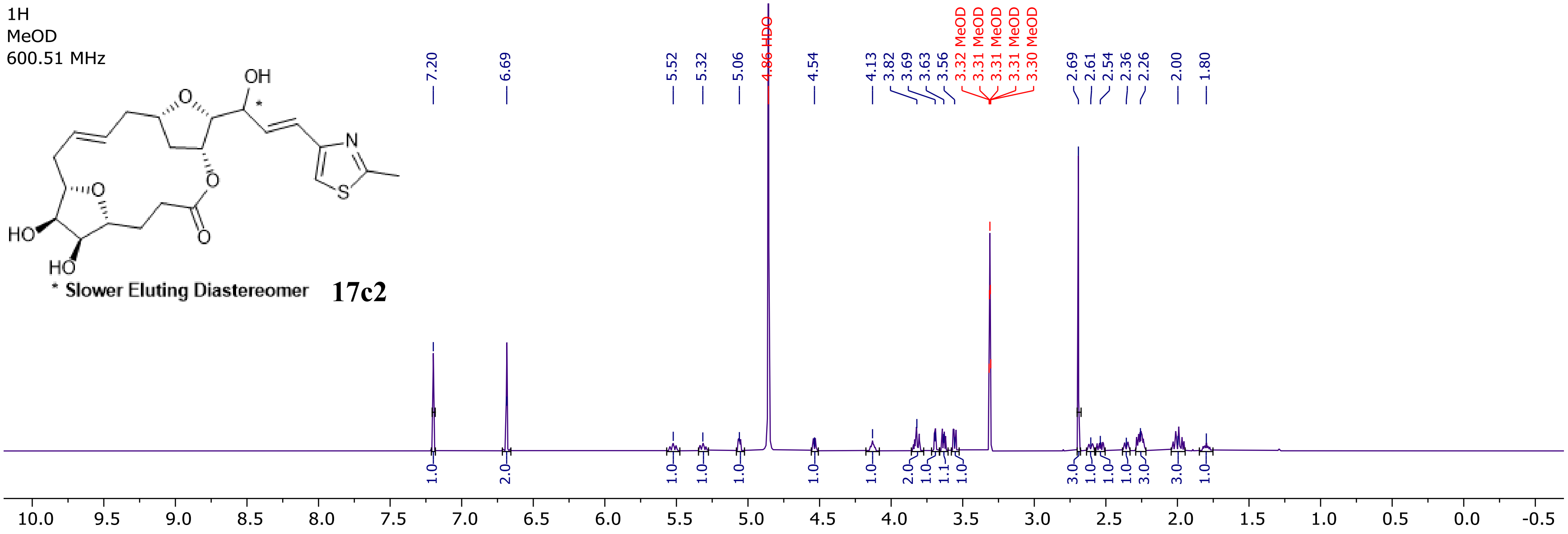


* Slower Eluting Diastereomer **17c2**

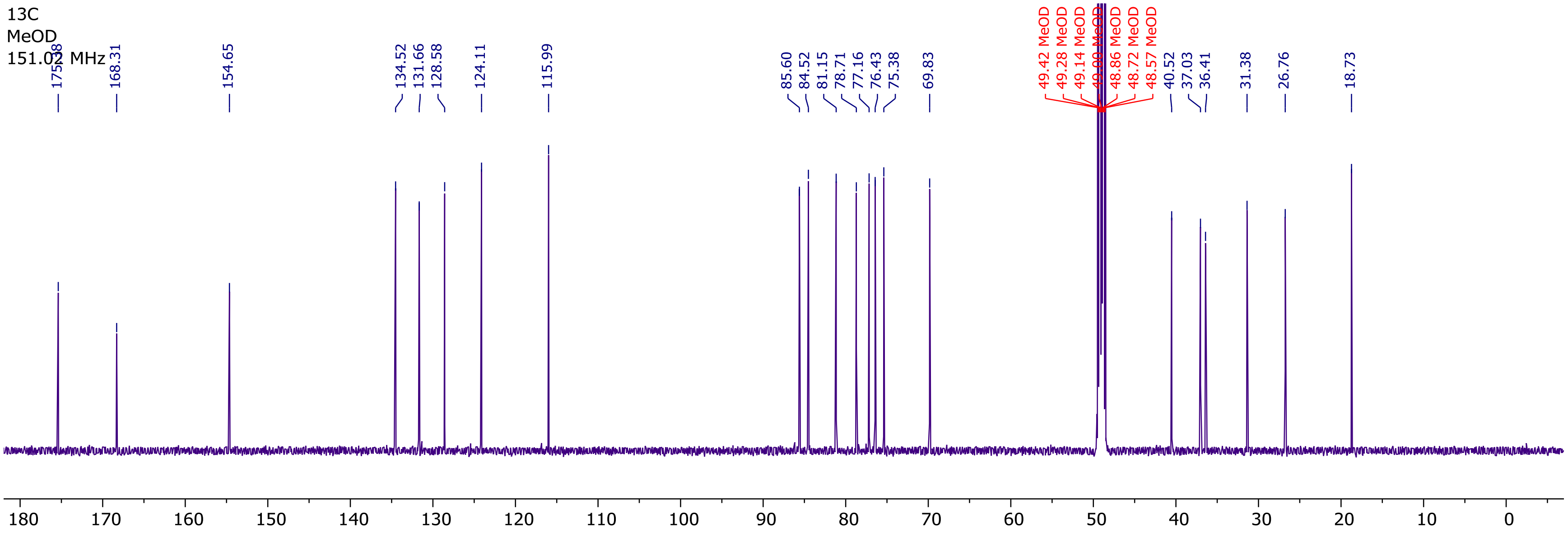

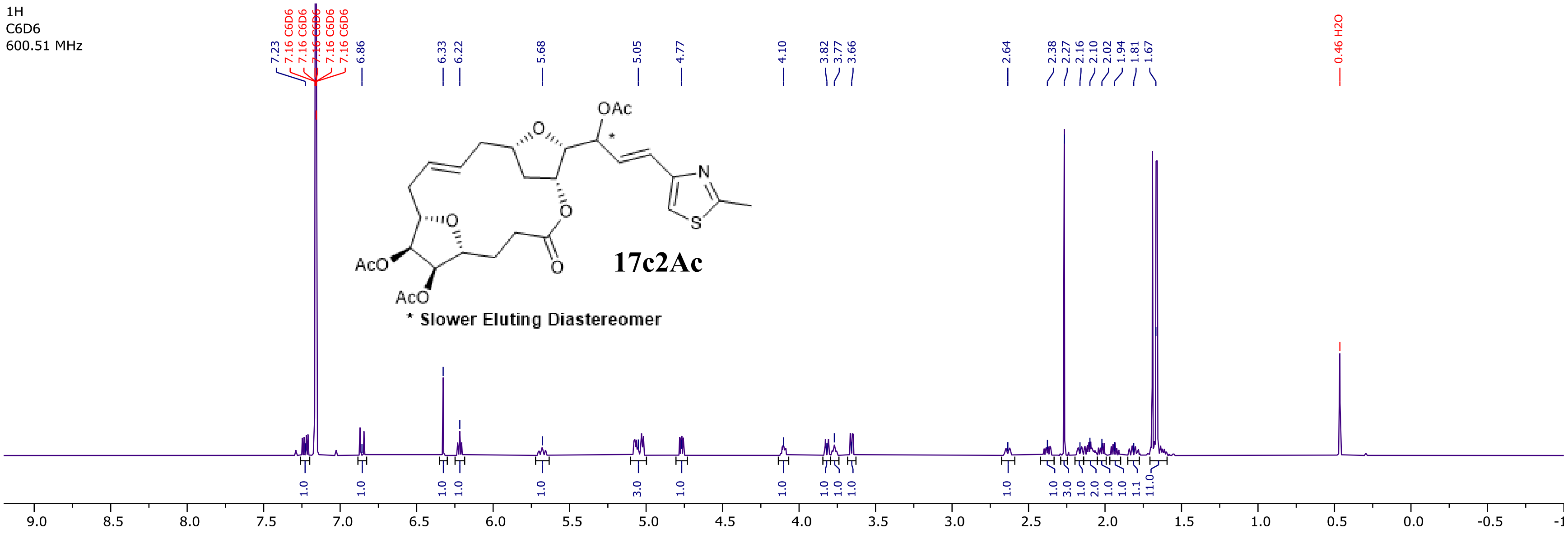
1H
C6D6
600.51 MHz
7.23
7.16 C6D6
6.86
6.33
6.22
5.68
5.05
4.77
4.10
3.82
3.77
3.66
2.64
2.38
2.27
2.16
2.10
2.02
1.94
1.81
1.67
0.46 H2O
OAc
AcO
AcO
17c2Ac
* Slower Eluting Diastereomer

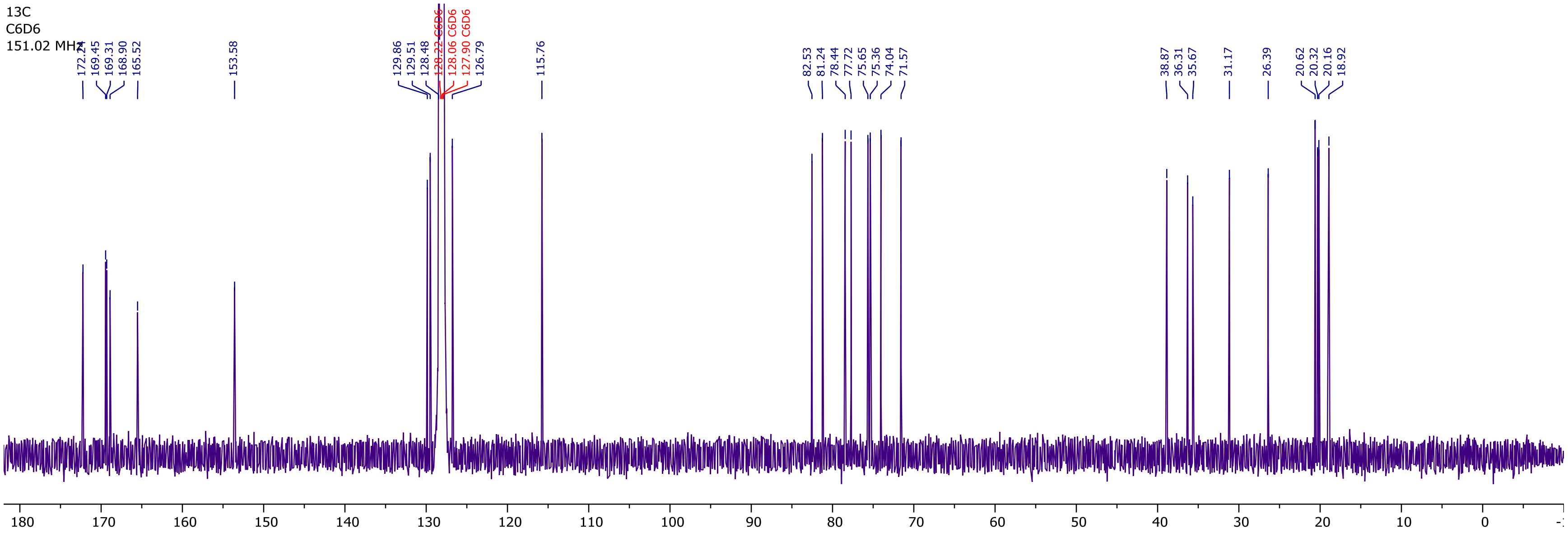
13C
C6D6
151.02 MHz
172.24
169.45
169.31
168.90
165.52
153.58
129.86
129.51
128.48
128.22 C6D6
128.06 C6D6
127.90 C6D6
126.79
115.76
82.53
81.24
78.44
77.72
75.65
75.36
74.04
71.57
38.87
36.31
35.67
31.17
26.39
20.62
20.32
20.16
18.92

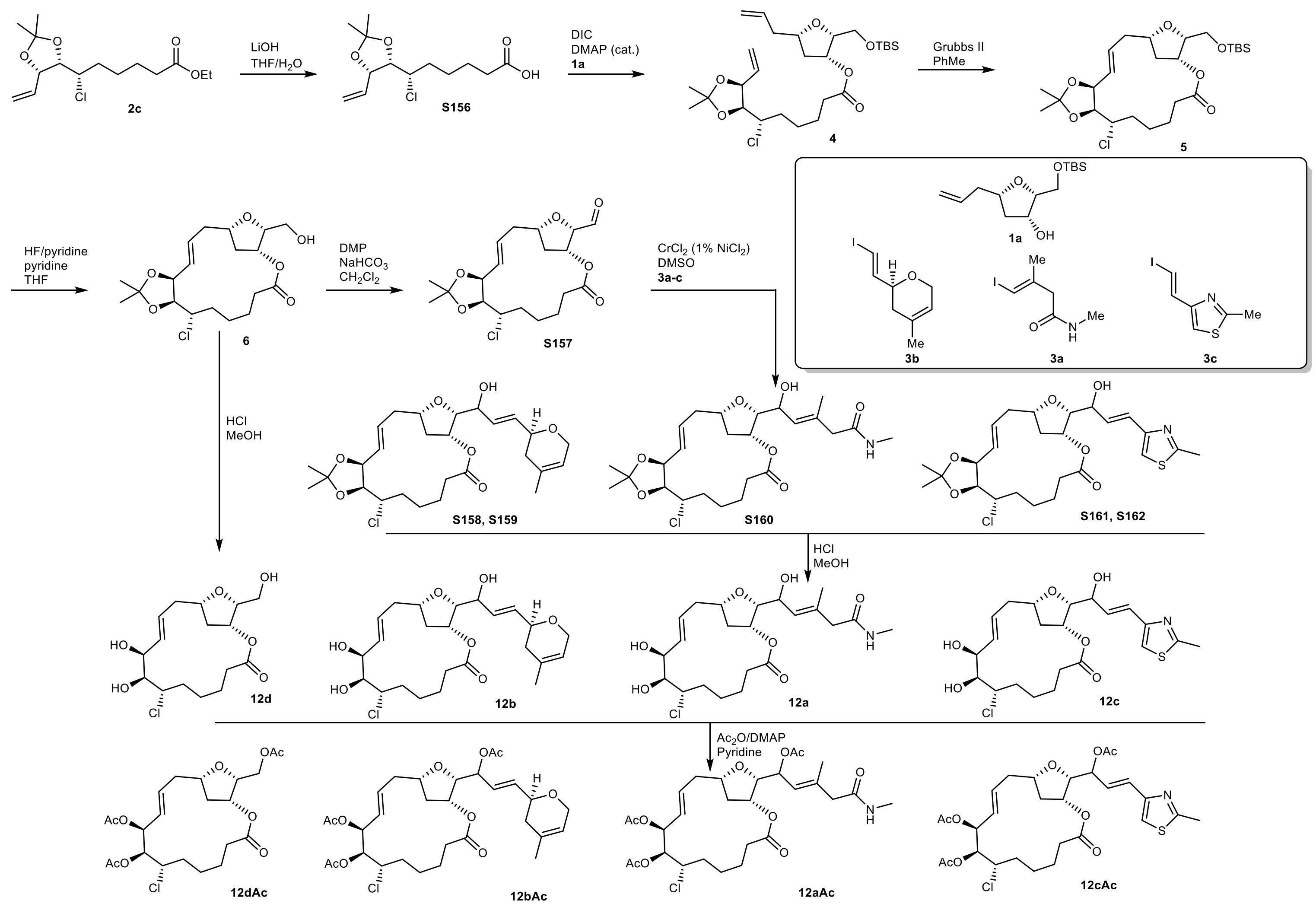


**Supplementary Fig. 51 | Synthesis of pMLs 12a-d and 12aAc-dAc.**

Abbreviations: THF = tetrahydrofuran, DIC = N,N’-diisopropylcarbodiimide, DMAP = 4-dimethylaminopyridine, TBS = tert-butyldimethylsilyl, Grubbs II = Dichloro[1,3-bis(2,4,6-trimethylphenyl)-2-imidazolidinylidene](benzylidene)(tricyclohexylphosphine)ruthenium(II), DMP = Dess-Martin periodinane, DMSO = dimethylsulfoxide.

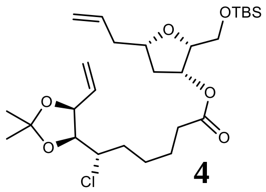

**4**

To a rt, stirred solution of **2c** (228.6 mg, 0.75 mmol, 1.0 eq.) in THF (2.5) and $H_2O$ (1.3 mL) was added a solution of LiOH (2.3 mL, 1M in $H_2O$, 2.7 mmol, 3.0 eq.). The reaction mixture was vigorously stirred for 4 h 10 min. After this time, starting material was consumed as monitored by TLC analysis. The reaction mixture was diluted with saturated aqueous $NH_4Cl$ (15 mL) and $Et_2O$ (15 mL), transferred to a separatory funnel. The organic layer was then separated, and the aqueous layer was extracted with $Et_2O$ (2x 15 mL) and $CH_2Cl_2$ (5x 30 mL). The combined organic layers were dried ($MgSO_4$), filtered, and solvent was removed in vacuo to yield crude acid **S156** (176 mg) as a colorless oil which was used immediately in the next step without further purification.

To a rt, stirred solution of acid **S156** (176 mg, 0.64 mmol, 1.3 eq.), alcohol **1a** (137.6 mg, 0.51 mmol, 1.0 eq.), and 4-dimethylaminopyridine (24.4 mg, 0.20 mmol, 0.39 eq.) in $CH_2Cl_2$ (3.3 mL) was added N,N'-diisopropylcarbodiimide (0.15, 120 mg, 0.96 mmol, 1.9 eq.). The reaction mixture was allowed to stir for 15 h at rt. After this time, starting material was consumed as monitored by TLC analysis. The cloudy reaction mixture was poured onto $H_2O$ (10 mL) in a separatory funnel, and the aqueous layer extracted with $CH_2Cl_2$ (3x 10 mL). The combined organic layers were dried ($Na_2SO_4$), filtered, and solvent was removed in vacuo to yield an oily residue. The residue was triturated vigorously with $Et_2O$ (3x 2 mL) and filtered. The filtrate was evaporated in vacuo to yield a pale-yellow oil. The crude product was purified via flash column chromatography (1:1:18 Hexanes/$Et_2O$/EtOAc) to give **4** (245 mg, 90%) as a colorless oil.

**Analytical Data for 4:**

$R_f$ = 0.13 (8.5:1.5 Hexanes/$Et_2O$)

$[\alpha]_D^{20}$ = -25.2° (c = 1.00, $CDCl_3$)

$^1H$ NMR (600 MHz, $CDCl_3$) δ 6.00 (ddd, *J* = 17.1, 10.2, 8.6 Hz, 1H), 5.79 (ddt, *J* = 17.2, 10.3, 7.0 Hz, 1H), 5.38 – 5.32 (m, 2H), 5.31 (dt, *J* = 4.2, 2.6 Hz, 1H), 5.11 – 5.02 (m, 2H), 4.56 (dd, *J* = 8.7, 6.3 Hz, 1H), 4.19 (t, *J* = 6.4 Hz, 1H), 3.97 (dq, *J* = 7.7, 6.5 Hz, 1H), 3.85 (td, *J* = 6.0, 4.2 Hz, 1H), 3.82 – 3.76 (m, 3H), 2.47 – 2.36 (m, 2H), 2.33 – 2.24 (m, 3H), 1.76 – 1.61 (m, 5H), 1.60 – 1.57 (m, 1H), 1.56 (s, 3H), 1.47 – 1.41 (m, 1H), 1.40 (s, 3H), 0.87 (s, 9H), 0.05 (s, 3H), 0.04 (s, 3H).

$^{13}C$ NMR (151 MHz, $CDCl_3$) δ 172.80, 134.57, 133.38, 120.58, 117.32, 109.37, 81.67, 81.12, 79.71, 77.41, 74.06, 61.33, 60.58, 40.47, 38.33, 34.41, 34.32, 27.44, 26.00, 25.90, 25.60, 24.33, 18.43, -5.17, -5.27.

HRMS (ESI): Anal. Calcd. for $C_{27}H_{51}NO_6SiCl^+$ $[M+NH_4]^+$ 548.3169, found 548.3164

IR (neat): $\nu_{max}$ ($cm^{-1}$) = 3078 (w, C=*CH*), 2938 (m, CH), 2862 (m, CH), 1736 (s, C=O), 1646 (w, C=C), 1464 (m), 1375 (m), 1253 (s).

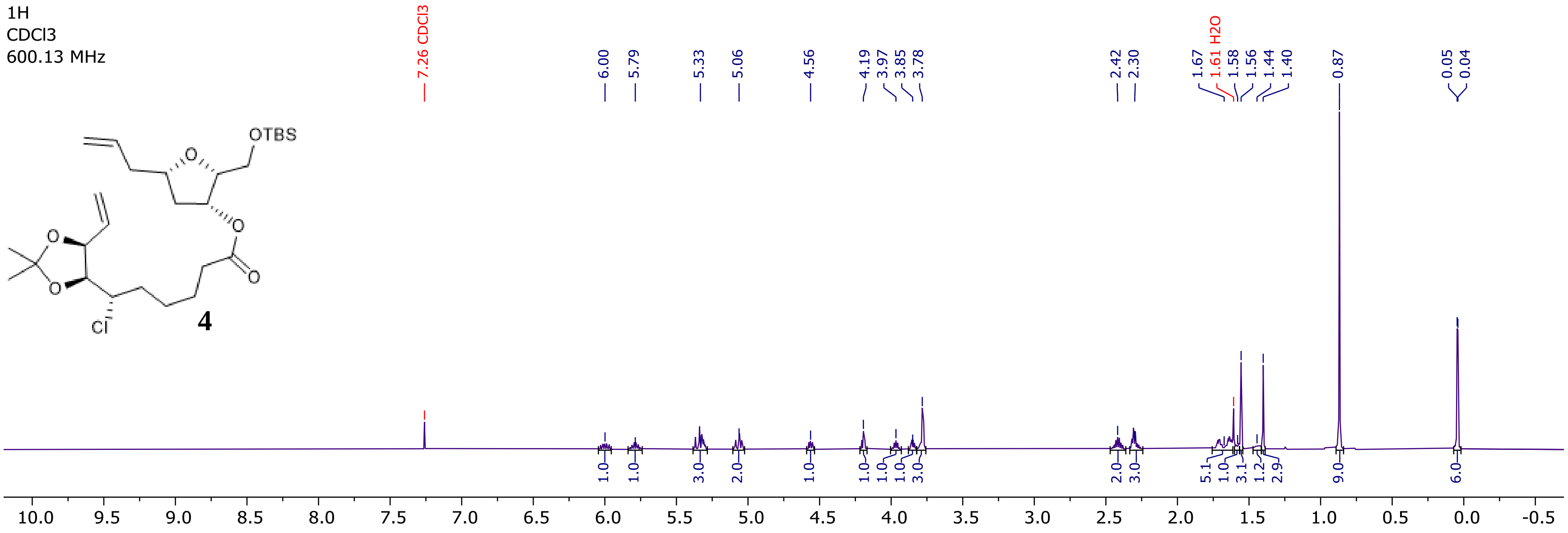
1H
CDCl3
600.13 MHz
7.26 CDCl3
6.00
5.79
5.33
5.06
4.56
4.19
3.97
3.85
3.78
2.42
2.30
1.67
1.61 H2O
1.58
1.56
1.44
1.40
0.87
0.05
0.04
1.0
1.0
3.0
2.0
1.0
1.0
1.0
1.0
3.0
2.0
3.0
5.1
1.0
3.1
1.2
2.9
9.0
6.0
10.0
9.5
9.0
8.5
8.0
7.5
7.0
6.5
6.0
5.5
5.0
4.5
4.0
3.5
3.0
2.5
2.0
1.5
1.0
0.5
0.0
-0.5
OTBS
O
O
O
O
O
Cl
**4**

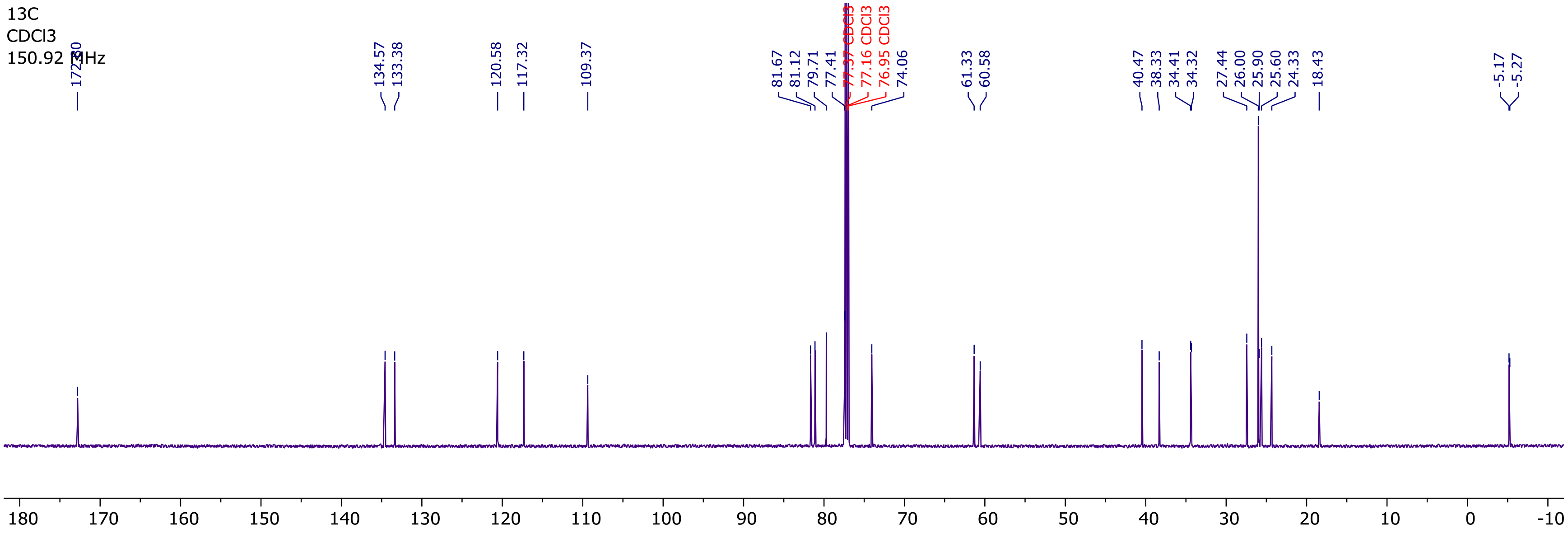
13C
CDCl3
150.92 MHz
134.57
133.38
120.58
117.32
109.37
81.67
81.12
79.71
77.41
77.16 CDCl3
76.95 CDCl3
74.06
61.33
60.58
40.47
38.33
34.41
34.32
27.44
26.00
25.90
25.60
24.33
18.43
-5.17
-5.27
180
170
160
150
140
130
120
110
100
90
80
70
60
50
40
30
20
10
0
-10

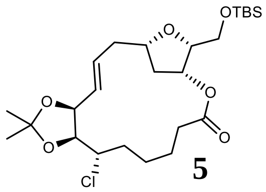


**5**

To a warm (60 °C), stirred solution of **4** (234 mg, 0.44 mmol, 1.0 eq.) in deoxygenated toluene (30 min. $N_2$(g) sparge) (180 mL) that was continuously being sparged with $N_2$(g) was added Grubbs second generation catalyst (74.8 mg, 0.088 mmol, 0.20 eq.) in toluene (3 mL). The reaction was allowed to proceed under continuous nitrogen sparge for 50 min. After this time, starting material was consumed as monitored by TLC analysis, the reaction mixture was cooled to rt and quenched by addition of potassium 2-isocyanoacetate (90 mg, 0.74 mmol, 1.7 eq.) in MeOH (15 mL). The reaction mixture was stirred for a further 7 h, after which time the red solution changed to a pale-yellow color. The reaction mixture was concentrated in vacuo to give a crude solid deposited on the walls of the evaporation flask. The walls were scraped with a spatula and the solid was triturated with $Et_2O$ (3x 10 mL) and filtered through a short plug of silica gel eluting with $Et_2O$. The filtrate was concentrated in vacuo and the crude product was purified via flash column chromatography (9:1 Hexanes/EtOAc). Appropriate fractions were pooled, and solvent was removed in vacuo to yield **5** (166 mg, 75%) as a white amorphous solid.

**Analytical Data for 5:**

$R_f$ = 0.36 (8.5:1.5 Hexanes/EtOAc)

$[\alpha]_D^{20}$ = +34° (c = 0.52, $CH_2Cl_2$)

$^1$H NMR (601 MHz, $CDCl_3$) δ 5.84 (ddd, *J* = 15.5, 10.9, 3.1 Hz, 1H), 5.56 (ddd, *J* = 15.6, 10.0, 2.2 Hz, 1H), 5.36 (ddd, *J* = 7.5, 4.0, 1.5 Hz, 1H), 4.46 (dd, *J* = 10.0, 5.1 Hz, 1H), 4.15 (dd, *J* = 10.0, 5.1 Hz, 1H), 4.07 (tdd, *J* = 8.2, 4.7, 1.9 Hz, 1H), 3.87 – 3.78 (m, 3H), 3.76 (td, *J* = 6.2, 3.9 Hz, 1H), 2.85 (ddt, *J* = 15.3, 5.2, 2.7 Hz, 1H), 2.45 – 2.38 (m, 1H), 2.37 – 2.33 (m, 1H), 2.28 (ddd, *J* = 14.8, 11.1, 3.3 Hz, 1H), 2.16 (ddd, *J* = 15.4, 10.9, 2.0 Hz, 1H), 2.02 – 1.92 (m, 1H), 1.86 – 1.72 (m, 2H), 1.62 – 1.55 (m, 1H), 1.52 (s, 3H), 1.48 – 1.41 (m, 1H), 1.40 (s, 3H), 1.34 (ddd, *J* = 14.6, 8.3, 1.7 Hz, 1H), 1.29 (ddd, *J* = 14.1, 9.2, 5.1 Hz, 1H), 0.87 (s, 9H), 0.05 (s, 3H), 0.04 (s, 3H).

$^{13}$C NMR (151 MHz, $CDCl_3$) δ 172.74, 131.44, 128.99, 108.93, 82.12, 81.35, 79.67, 76.05, 74.45, 60.72, 60.50, 37.49, 35.94, 32.91, 32.80, 28.79, 26.11, 26.00, 24.70, 24.02, 18.46, -5.18, -5.31, -5.37.

HRMS (ESI): Anal. Calcd. for $C_{27}H_{47}NO_6SiCl^+$ $[M+NH_4]^+$ 520.2856, found 520.2874

IR (neat): $\nu_{max}$ $(cm^{-1})$ = 2977 (s, CH), 2900 (s, CH), 1735 (s, C=O), 1688 (w), 1249 (s).

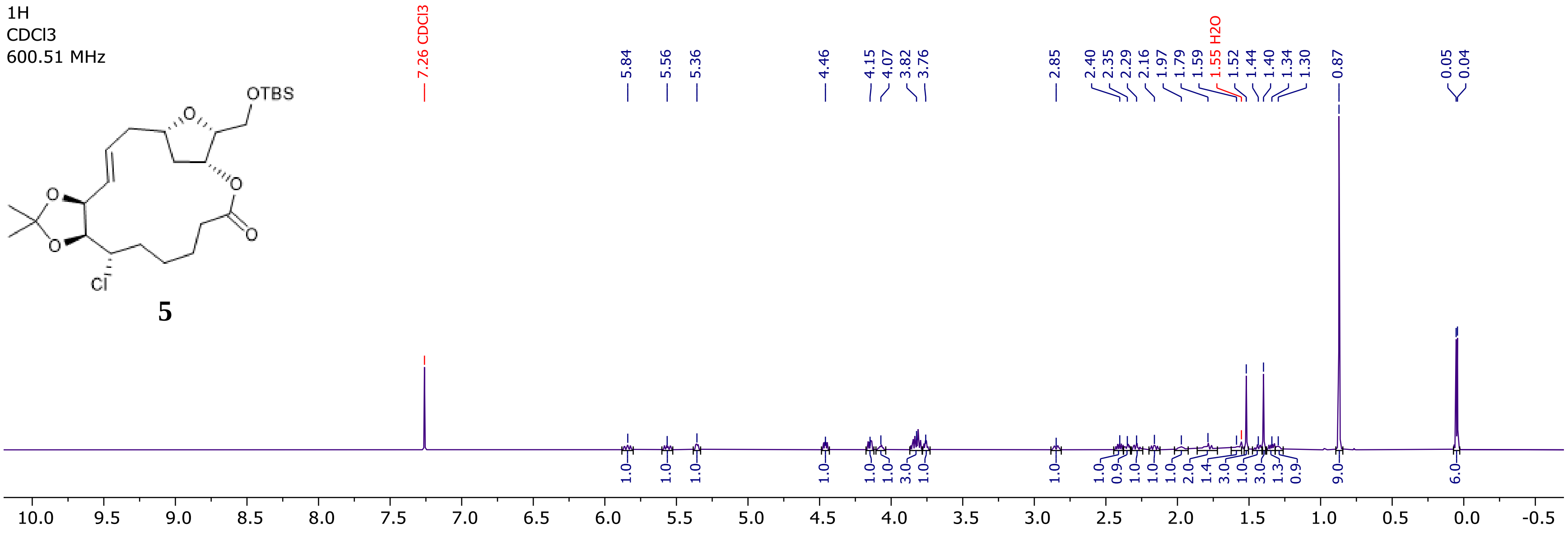
1H
CDCl3
600.51 MHz
OTBS
O
O
O
O
O
Cl
5
7.26 CDCl3
5.84
5.56
5.36
4.46
4.15
4.07
3.82
3.76
2.85
2.40
2.35
2.29
2.16
1.97
1.79
1.59
1.55 H2O
1.52
1.44
1.40
1.34
1.30
0.87
0.05
0.04
1.0
1.0
1.0
1.0
1.0
1.0
3.0
1.0
1.0
1.0
0.9
1.0
1.0
2.0
1.4
3.0
1.0
3.0
1.3
0.9
9.0
6.0
10.0 9.5 9.0 8.5 8.0 7.5 7.0 6.5 6.0 5.5 5.0 4.5 4.0 3.5 3.0 2.5 2.0 1.5 1.0 0.5 0.0 -0.5

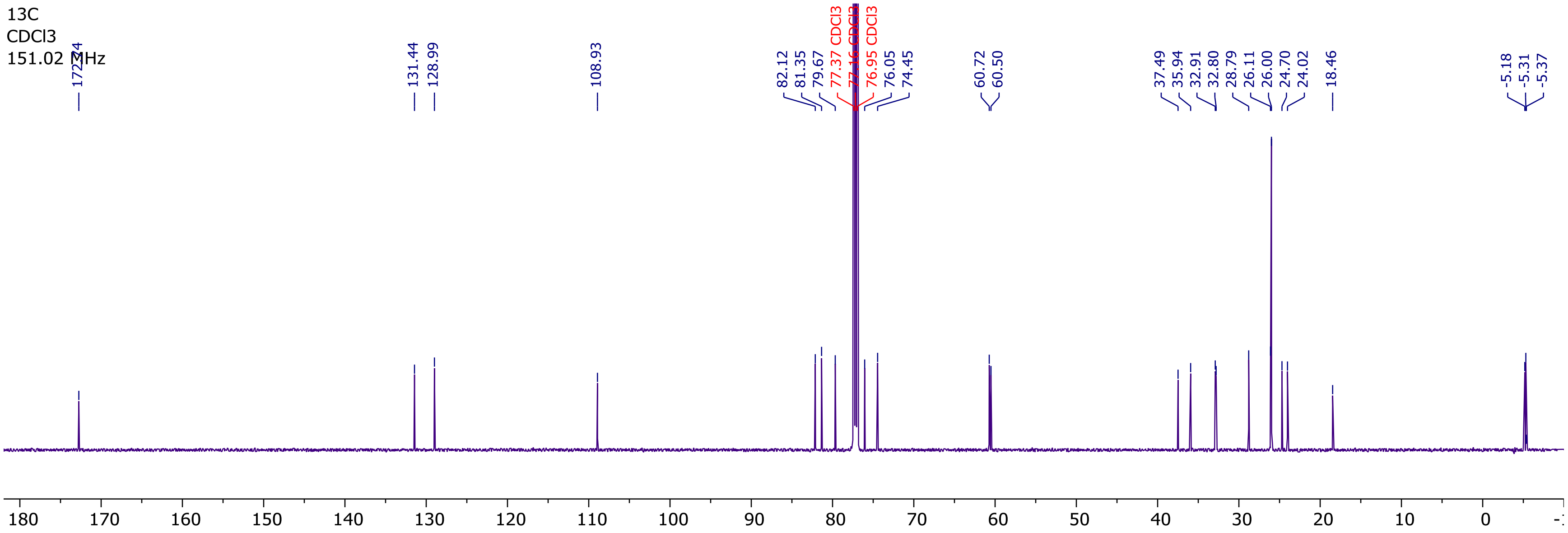
13C
CDCl3
151.02 MHz
172.24
131.44
128.99
108.93
82.12
81.35
79.67
77.37 CDCl3
77.16 CDCl3
76.95 CDCl3
76.05
74.45
60.72
60.50
37.49
35.94
32.91
32.80
28.79
26.11
26.00
24.70
24.02
18.46
-5.18
-5.31
-5.37
180 170 160 150 140 130 120 110 100 90 80 70 60 50 40 30 20 10 0

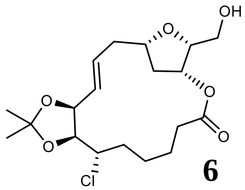

**6**

To a rt, stirred solution of **5** (164 mg, 0.33 mmol, 1.0 eq.) in a mixture of THF (4 mL) and pyridine (1.7 mL), was added HF-Pyridine (70% (w/w) HF, 0.42 mL, 320 mg HF, 16 mmol HF, 48 eq. HF) dropwise over 5 min. The reaction mixture was stirred at rt for 22 h. After this time, starting material was consumed as monitored by TLC analysis. The reaction mixture was quenched by slowly pipetting it onto saturated aqueous $NaHCO_3$ (30 mL) [**caution!** $CO_2$(g) evolved] over 5 min. After evolution of $CO_2$(g) had stopped, the aqueous layer was extracted with $Et_2O$ (2x 20 mL) and $CH_2Cl_2$ (3x 20 mL). The combined organic layers were dried ($MgSO_4$), filtered, and solvent was removed in vacuo. The crude product was purified via flash column chromatography (7:3 Hexanes/Acetone). Appropriate fractions were pooled, and solvent was removed in vacuo to yield **6** (121.6 mg, 96%) as a white amorphous solid.

**Analytical Data for 6:**

$R_f$ = 0.28 (7:3 Hexanes/Acetone)

$[\alpha]_D^{20}$ = +67° (c = 0.76, $CH_2Cl_2$)

$^1$H NMR (601 MHz, $CDCl_3$) δ 5.83 (ddd, *J* = 15.5, 10.9, 3.3 Hz, 1H), 5.58 (ddd, *J* = 15.6, 9.9, 2.1 Hz, 1H), 5.35 (ddd, *J* = 7.5, 4.0, 1.8 Hz, 1H), 4.47 (dd, *J* = 9.9, 5.1 Hz, 1H), 4.14 (dd, *J* = 9.9, 5.1 Hz, 1H), 4.09 (tdd, *J* = 8.2, 4.6, 2.0 Hz, 1H), 3.87 (dd, *J* = 11.4, 6.9 Hz, 1H), 3.84 – 3.76 (m, 2H), 3.72 (dd, *J* = 11.4, 4.4 Hz, 1H), 2.87 (ddt, *J* = 15.1, 5.1, 2.5 Hz, 1H), 2.43 (dt, *J* = 14.5, 7.8 Hz, 1H), 2.38 – 2.28 (m, 2H), 2.18 (ddd, *J* = 15.3, 11.0, 2.1 Hz, 1H), 1.98 – 1.90 (m, 1H), 1.90 – 1.82 (m, 2H), 1.81 (tdd, *J* = 12.5, 4.1, 2.2 Hz, 1H), 1.58 (ddq, *J* = 12.9, 6.5, 4.4 Hz, 1H), 1.52 (s, 3H), 1.47 – 1.37 (m, 2H), 1.40 (s, 3H), 1.30 – 1.21 (m, 1H).

$^{13}$C NMR (151 MHz, $CDCl_3$) δ 173.19, 130.94, 129.47, 108.99, 82.20, 81.47, 79.62, 76.08, 74.96, 60.85, 60.47, 37.27, 35.90, 33.06, 32.64, 28.75, 26.10, 24.89, 24.01.

HRMS (ESI): Anal. Calcd. for $C_{19}H_{33}NO_6Cl^+$ $[M+NH_4]^+$ 406.1991, found 406.2011

IR (neat): $\nu_{max}$ $(cm^{-1})$ = 3450 (br, OH), 3054 (w, C=*CH*), 2987 (m, CH), 2940 (m, CH), 2871 (m, CH), 1728 (s, C=O), 1439 (m), 1376 (s), 1229 (s).

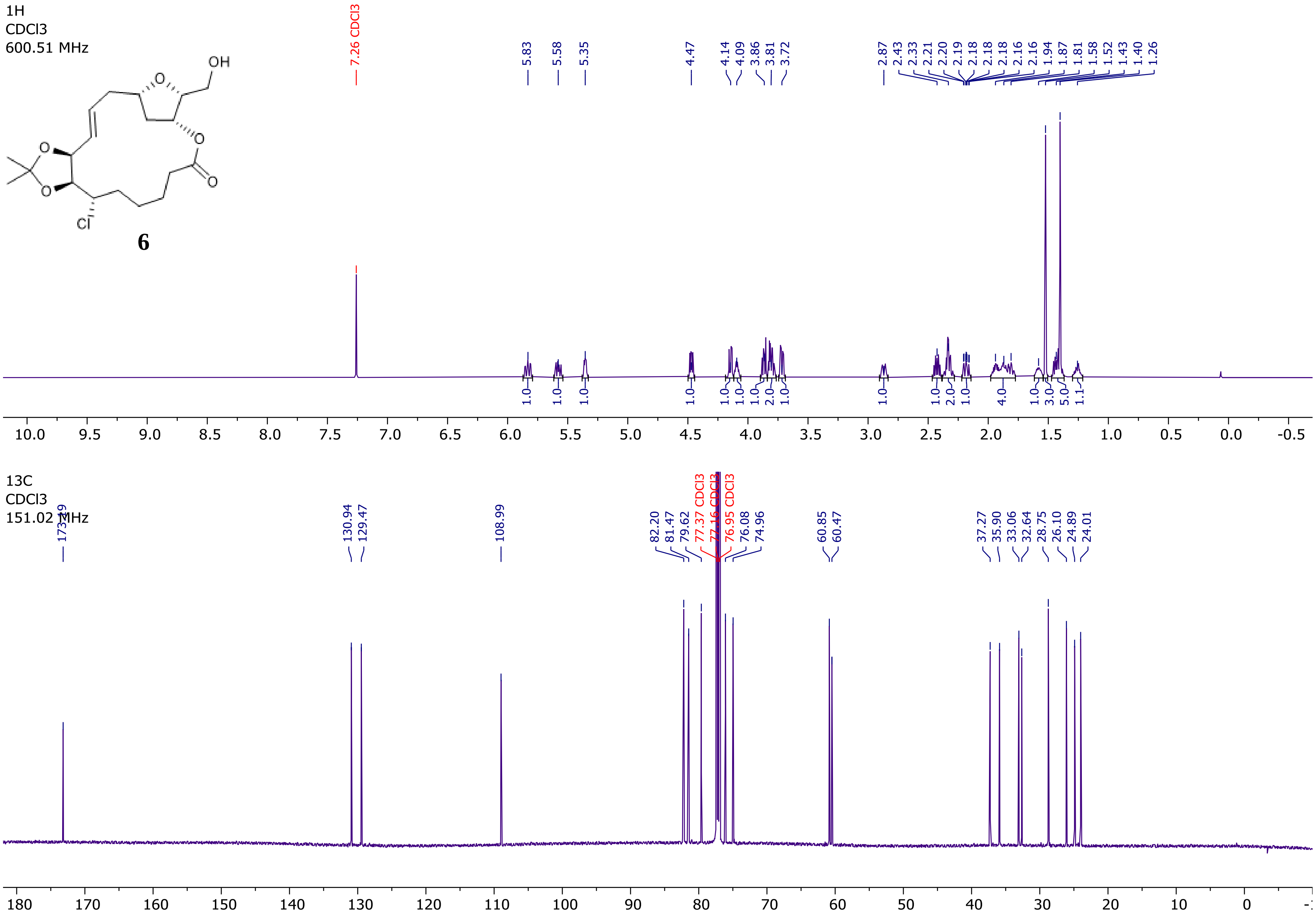
1H
CDCl3
600.51 MHz
OH
O
O
O
O
O
Cl
6
7.26 CDCl3
5.83
5.58
5.35
4.47
4.14
4.09
3.86
3.81
3.72
2.87
2.43
2.33
2.21
2.20
2.19
2.18
2.18
2.16
2.16
1.94
1.87
1.81
1.58
1.52
1.43
1.40
1.26
13C
CDCl3
151.02 MHz
173.19
130.94
129.47
108.99
82.20
81.47
79.62
77.37 CDCl3
77.16 CDCl3
76.95 CDCl3
76.08
74.96
60.85
60.47
37.27
35.90
33.06
32.64
28.75
26.10
24.89
24.01

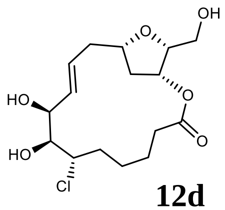


**12d**

Note: an approximately 1.2 M solution of aqueous HCl in MeOH was prepared by slowly adding concentrated HCl (1 mL, *ca.* 12 M) to MeOH (9 mL). To a cold (0 °C), stirred solution of **6** (5.0 mg, 0.0129 mmol, 1.0 eq.) in $CH_2Cl_2$ (0.6 mL) MeOH (0.6 mL) was added the previously prepared solution of aqueous HCl in MeOH (53.5 µL, *ca.* 1.2M in MeOH, 0.0642 mmol, 5.0 eq.) via microsyringe. The reaction vessel was moved to a fridge (4 °C) and allowed to stir in the fridge for 46 h. After this time, starting material was consumed as monitored by TLC analysis, and the reaction mixture was quenched by addition of $NaHCO_3$ (30 mg, 0.357 mmol, 27.7 eq.) and allowed to stir vigorously for 10 min. After this time, the reaction mixture was filtered through a cotton plug. Then silica gel was added and the reaction mixture was concentrated onto the silica gel in vacuo. The crude product was then purified via flash column chromatography (97:3 $CH_2Cl_2$/MeOH). Appropriate fractions were pooled, and solvent was removed in vacuo to yield **12d** (1.6 mg, 23%, 4 steps from **1a**) as a white film.

**Analytical Data for 12d:**

$R_f$ = 0.45 (9:1 $CH_2Cl_2$/MeOH)

$[\alpha]_D^{20}$ = +67 ° (c = 0.16, MeOH)

$^1$H NMR (601 MHz, MeOD) δ 5.88 (ddd, *J* = 15.8, 10.4, 2.6 Hz, 1H), 5.75 (ddd, *J* = 15.7, 9.4, 2.2 Hz, 1H), 5.40 (ddd, *J* = 7.2, 3.6, 1.2 Hz, 1H), 4.16 (dd, *J* = 9.4, 2.8 Hz, 1H), 4.16 – 4.10 (m, 1H), 3.80 – 3.67 (m, 4H), 3.52 (ddd, *J* = 11.6, 9.5, 1.9 Hz, 1H), 2.77 (ddt, *J* = 15.8, 4.3, 2.5 Hz, 1H), 2.49 – 2.40 (m, 2H), 2.37 (ddd, *J* = 14.9, 5.7, 4.1 Hz, 1H), 2.33 – 2.26 (m, 1H), 1.98 – 1.85 (m, 3H), 1.57 (dddd, *J* = 13.7, 10.1, 7.1, 4.0 Hz, 1H), 1.45 (ddd, *J* = 14.6, 7.5, 1.3 Hz, 1H), 1.38 – 1.27 (m, 1H), 1.18 – 1.08 (m, 1H).

$^{13}$C NMR (151 MHz, MeOD) δ 174.41, 132.08, 130.70, 83.51, 78.85, 77.45, 76.00, 74.42, 68.38, 60.82, 37.84, 36.32, 32.97, 32.32, 26.03, 25.22.

HRMS (ESI): Anal. Calcd. for $C_{16}H_{26}ClO_6^+$ $[M+H]^+$ 349.1413, found 349.1408

IR (neat): $\nu_{max}$ $(cm^{-1})$ = 3405 (br, OH), 2945 (m, CH), 2869 (m, CH), 1727 (s, C=O), 1660 (m, C=C), 1448 (m), 1431 (m), 1374 (m), 1237 (m)

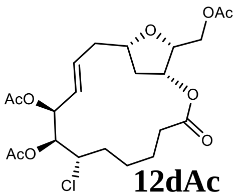


**12dAc**

**12dAc** was prepared according to general procedure A (1.02 mg, 63%)

**Analytical Data for 12dAc:**

$R_f$ = 0.58 (2:3 Hexanes/EtOAc)

HRMS (ESI): Anal. Calcd. for $C_{22}H_{35}NO_9Cl^+$ $[M+H]^+$ 492.1995, found 492.1982

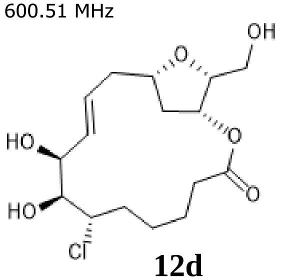

OH
O
HO
HO
O
O
Cl


**12d**

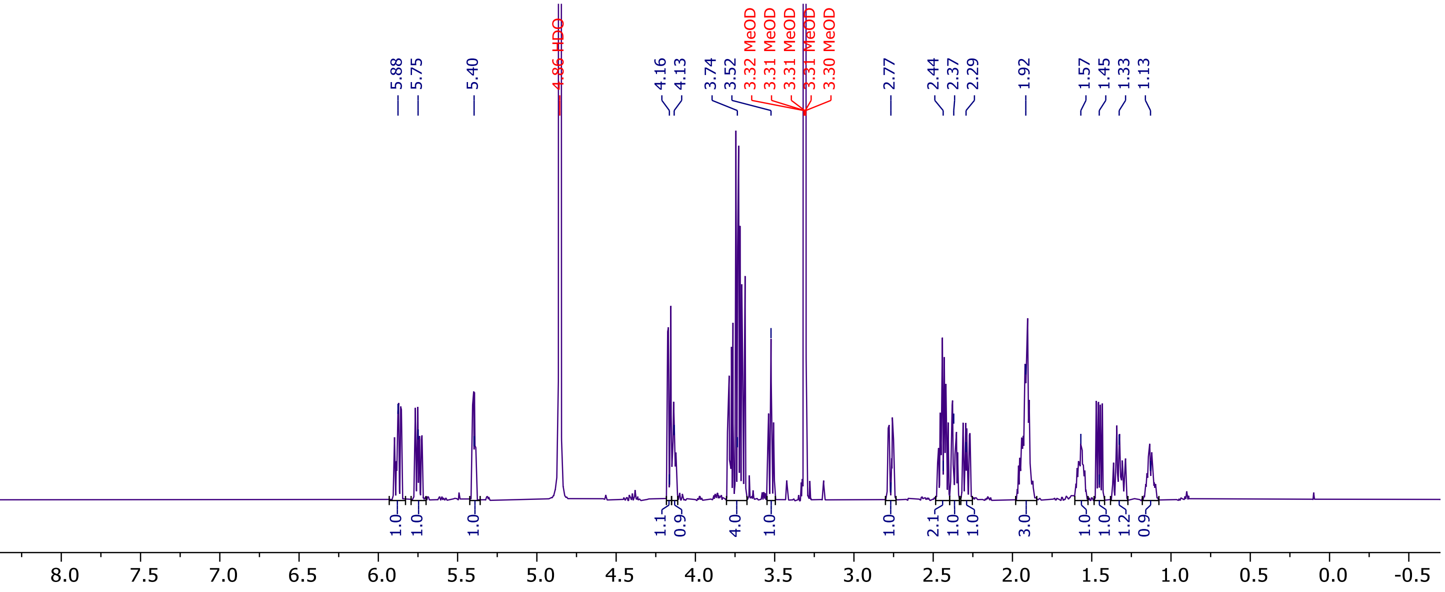

1H
MeOD
600.51 MHz
5.88
5.75
5.40
4.86 HDO
4.16
4.13
3.74
3.52
3.32 MeOD
3.31 MeOD
3.31 MeOD
3.31 MeOD
3.30 MeOD
2.77
2.44
2.37
2.29
1.92
1.57
1.45
1.33
1.13
1.0
1.0
1.0
1.1
0.9
4.0
1.0
1.0
2.1
1.0
1.0
3.0
1.0
1.0
1.2
0.9
10.0
9.5
9.0
8.5
8.0
7.5
7.0
6.5
6.0
5.5
5.0
4.5
4.0
3.5
3.0
2.5
2.0
1.5
1.0
0.5
0.0
-0.5


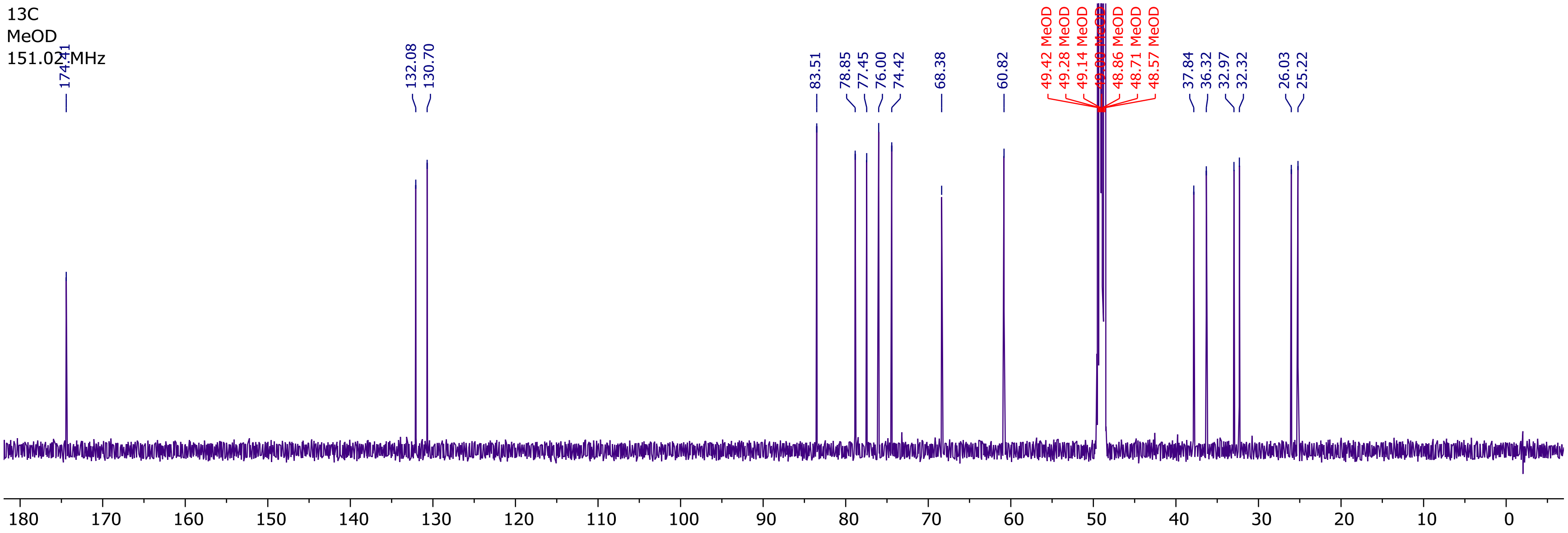

13C
MeOD
151.02 MHz
174.41
132.08
130.70
83.51
78.85
77.45
76.00
74.42
68.38
60.82
49.42 MeOD
49.28 MeOD
49.14 MeOD
49.00 MeOD
48.86 MeOD
48.71 MeOD
48.57 MeOD
37.84
36.32
32.97
32.32
26.03
25.22
180
170
160
150
140
130
120
110
100
90
80
70
60
50
40
30
20
10
0

To a rt, stirred solution of **6** (111 mg, 0.285 mmol, 1.0 eq.) in $CH_2Cl_2$ (6.3 mL) was added $NaHCO_3$ (71.8 mg, 0.854 mmol, 3.0 eq.) followed by Dess-Martin Periodinane (157.2 mg, 0.371 mmol, 1.3 eq.). The mixture was stirred at rt for 3 h. After this time, starting material was consumed as monitored by TLC analysis. The reaction mixture was quenched with a 1:1 (v/v) saturated aqueous $NaHCO_3$-10% aqueous $Na_2S_2O_3$ (15 mL) and the biphasic mixture was stirred vigorously for 30 min at rt. The aqueous layer was extracted with $CH_2Cl_2$ (2x 15 mL), and the combined organic layers were dried ($Na_2SO_4$), filtered, and the solvent was removed in vacuo. The crude product was passaged through a short plug of C2 modified silica gel eluting with $Et_2O$. Solvent was removed in vacuo to yield the crude aldehyde **S157** (*ca.* 111 mg) as a colorless oil which was portioned and used immediately in the next steps without further purification.

To a solution of **S157** (36.8 mg, 0.095 mmol, 1.0 eq.) in deoxygenated DMSO (5x freeze-pump-thaw-cycles) (1 mL) was added $CrCl_2$ doped with 1 % $NiCl_2$ (w/w) (120 mg, 0.976 mmol, 10.3 eq.). **3b** (76 mg, 0.30 mmol, 3.2 eq.) was added via syringe in deoxygenated DMSO (0.5 mL + 2x 0.25 mL rinses) and the mixture was stirred for 67 h at rt. After this time, the reaction mixture was transferred to a separatory funnel, diluted with EtOAc (10 mL) and 15 mL of 1:1 (v/v) $H_2O$-brine was added. The organic layer was separated, and the aqueous layer extracted with EtOAc (10x 20 mL). The combined organic layers were dried ($MgSO_4$), filtered, and solvent was removed in vacuo. The crude product was purified via flash column chromatography (3:2 Hexanes/Acetone). Appropriate fractions were pooled, and solvent was removed in vacuo to yield major diastereomer **S158** (22 mg, 45%), minor diastereomer **S159** (2.7 mg, 5.6%) and a mixture of the major and minor diastereomers (5.7 mg, 12%) all as white foams. The products were used immediately in the subsequent steps.

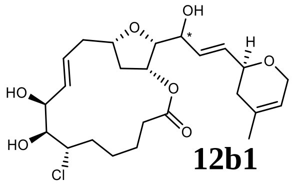


**12b1**

An approximately 1.2 M solution of aqueous HCl in MeOH was prepared by slowly adding concentrated HCl (1 mL, *ca.* 12 M) to MeOH (9 mL). To a cold (0 °C), stirred solution of the major diastereomer of **S158** (22 mg, 0.043 mmol, 1.0 eq.) in $CH_2Cl_2$ (1.0 mL) and MeOH (1.0 mL) was added the previously prepared solution of aqueous HCl in MeOH (179 µL, *ca.* 1.2M in MeOH, 0.215 mmol, 5.0 eq.) via microsyringe. The reaction vessel was moved to a fridge (4 °C) and allowed to stir in the fridge for 22 h 30 min. After this time, the reaction mixture was warmed to rt, and stirred for 3 h 30 min at rt. After this time, the reaction vessel was transferred back to the fridge (4 °C) and allowed to stir in the fridge for a further 17 h. After this time, the reaction mixture was quenched by addition of $NaHCO_3$ (75 mg, 0.89 mmol, 21 eq.) and allowed to stir vigorously for 10 min. After this time, the reaction mixture was filtered and solvent was removed in vacuo. The crude product was then purified via flash column chromatography (7:2.25:0.75 Hexanes/EtOAc/EtOH to 6:3:1 Hexanes/EtOAc/EtOH). Appropriate fractions were pooled, and solvent was removed in vacuo to yield the major diastereomer of **12b** (17.1 mg, 25%, 6 steps from **1a**).

**Analytical Data for 12b1 (major diastereomer of 12b):**

$R_f$ = 0.54 (92.5:7.5 $CH_2Cl_2$/MeOH)

$[\alpha]_D^{20}$ = +59.6 ° (c = 0.855, MeOH)

$^1$H NMR (601 MHz, $CDCl_3$) δ 5.95 (dd, *J* = 15.8, 4.6 Hz, 1H), 5.93 – 5.83 (m, 2H), 5.74 (ddd, *J* = 15.7, 9.5, 2.2 Hz, 1H), 5.49 (dd, *J* = 6.9, 3.4 Hz, 1H), 5.42 (ddt, *J* = 4.3, 3.0, 1.7 Hz, 1H), 4.28 – 4.23 (m, 1H), 4.23 – 4.14 (m, 3H), 4.13 – 4.08 (m, 1H), 4.06 (dt, *J* = 10.0, 4.0 Hz, 1H), 3.84 (dd, *J* = 9.7, 3.0 Hz, 1H), 3.63 (ddd, *J* = 11.2, 9.6, 1.8 Hz, 1H), 3.49 (dd, *J* = 8.0, 3.4 Hz, 1H), 2.84 (dq, *J* = 15.7, 2.8 Hz, 1H), 2.50 – 2.16 (m, 7H), 2.12 – 2.05 (m, 1H), 2.00 (ddd, *J* = 15.0, 9.8, 5.4 Hz, 1H), 1.96 – 1.89 (m, 3H), 1.70 (t, *J* = 1.8 Hz, 3H), 1.62 – 1.53 (m, 1H), 1.47 (ddd, *J* = 14.6, 7.4, 1.2 Hz, 1H), 1.34 (tdd, *J* = 13.5, 10.6, 2.5 Hz, 1H), 1.21 – 1.12 (m, 1H).

$^{13}$C NMR (151 MHz, $CDCl_3$) δ 173.56, 132.12, 131.65, 131.58, 130.32, 129.19, 119.78, 84.56, 77.29, 75.99, 74.83, 73.57, 73.46, 68.84, 67.74, 65.84, 36.32, 35.83, 35.68, 32.35, 30.74, 24.71, 24.59, 23.09.

HRMS (ESI): Anal. Calcd. for $C_{24}H_{35}ClO_7Na^+$ $[M+Na]^+$ 493.1963, found 493.1951

IR (neat): $\nu_{max}$ $(cm^{-1})$ = 3410 (br, OH), 2913 (m, CH), 2863 (m, CH), 1726 (s, C=O), 1653 (w, C=C), 1439 (m), 1378 (m), 1343 (m), 1233 (m)

**12b1Ac** was prepared according to general procedure A (1.17 mg, 75%)

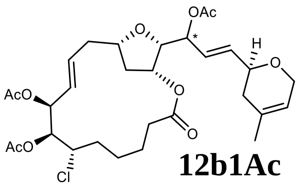


**12b1Ac**

* Major Diastereomer

**Analytical Data for 12b1Ac:**

$R_f$ = 0.41 (5.5:4.5 Hexanes/EtOAc)

$[\alpha]_D^{20}$ = +34° (c = 0.40, $C_6D_6$)

$^1$H NMR (601 MHz, $C_6D_6$) δ 6.18 (ddd, *J* = 15.7, 6.1, 1.6 Hz, 1H), 6.11 – 6.02 (m, 2H), 5.82 (ddt, *J* = 9.1, 6.1, 1.0 Hz, 1H), 5.71 (dd, *J* = 10.2, 2.6 Hz, 1H), 5.64 – 5.54 (m, 2H), 5.47 (dd, *J* = 6.9, 3.4 Hz, 1H), 5.11 – 5.06 (m, 1H), 4.10 (ddt, *J* = 16.0, 4.2, 2.3 Hz, 1H), 3.95 (dddd, *J* = 15.9, 5.6, 3.9, 2.1 Hz, 1H), 3.88 – 3.83 (m, 1H), 3.58 (ddd, *J* = 11.2, 10.1, 2.1 Hz, 1H), 3.55 – 3.50 (m, 1H), 3.46 (dd, *J* = 9.0, 3.4 Hz, 1H), 2.55 (dq, *J* = 15.7, 2.9 Hz, 1H), 2.09 (ddd, *J* = 14.4, 5.4, 4.2 Hz, 1H), 1.99 (ddd, *J* = 14.4, 11.9, 3.8 Hz, 1H), 1.96 – 1.89 (m, 1H), 1.84 (s, 3H), 1.81 – 1.68 (m, 9H), 1.67 – 1.59 (m, 2H), 1.55 – 1.49 (m, 1H), 1.40 (s, 3H), 1.13 – 1.04 (m, 1H), 1.04 – 0.98 (m, 2H), 0.98 – 0.89 (m, 1H).

$^{13}$C NMR (151 MHz, $C_6D_6$) δ 172.21, 169.49, 169.37, 169.03, 135.07, 134.90, 131.30, 126.62, 125.52, 120.30, 83.09, 76.15, 75.62, 73.95, 73.13, 72.81, 70.22, 65.83, 63.58, 36.28, 35.99, 35.65, 32.54, 30.76, 25.26, 24.38, 22.86, 20.78, 20.63, 20.44.

HRMS (ESI): Anal. Calcd. for $C_{30}H_{45}NO_{10}Cl$ $[M+NH_4]^+$ 614.2727, found 614.2690

IR (neat): $\nu_{max}$ $(cm^{-1})$ = 2925 (m, CH), 2857 (m, CH), 1741 (vs, CO), 1646 (w, C=C), 1441 (m), 1371 (m), 1235 (vs, CO).

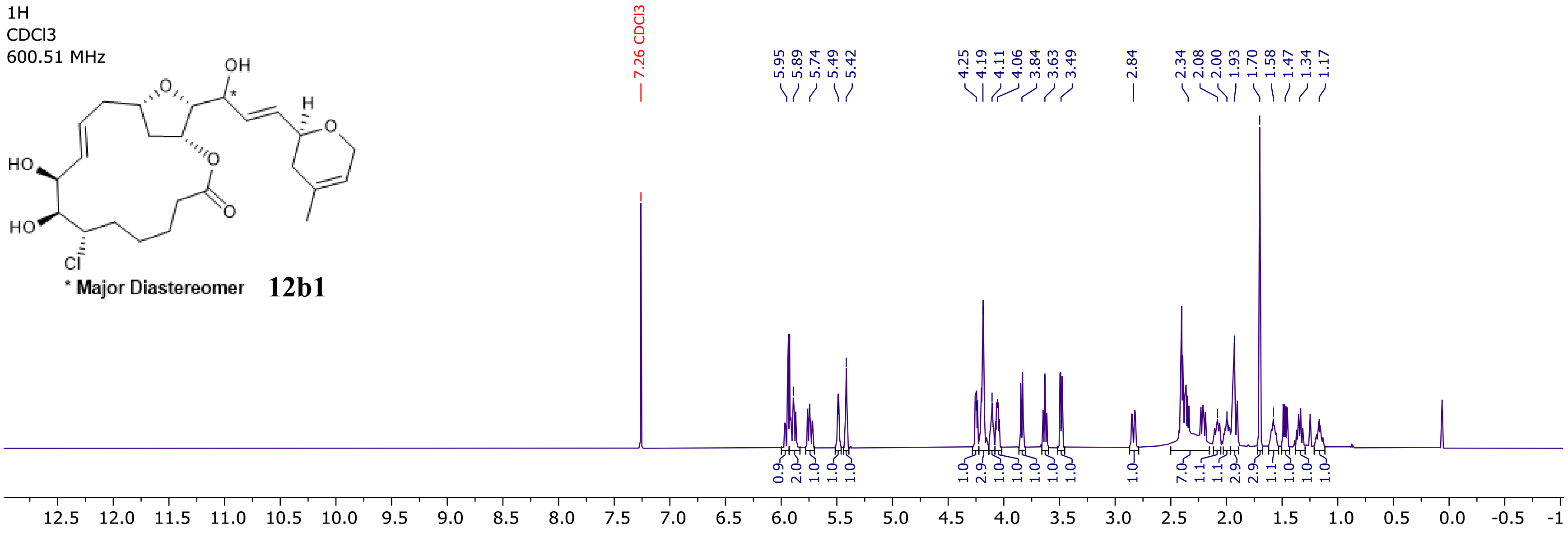
1H
CDCl3
600.51 MHz
OH
H
O
HO
HO
Cl
* Major Diastereomer
12b1
7.26 CDCl3
5.95
5.89
5.74
5.49
5.42
4.25
4.19
4.11
4.06
3.84
3.63
3.49
2.84
2.34
2.08
2.00
1.93
1.70
1.58
1.47
1.34
1.17
0.9
2.0
1.0
1.0
1.0
1.0
2.9
1.0
1.0
1.0
1.0
1.0
1.0
7.0
1.1
1.1
2.9
2.9
1.1
1.0
1.0
1.0
12.5 12.0 11.5 11.0 10.5 10.0 9.5 9.0 8.5 8.0 7.5 7.0 6.5 6.0 5.5 5.0 4.5 4.0 3.5 3.0 2.5 2.0 1.5 1.0 0.5 0.0 -0.5 -1

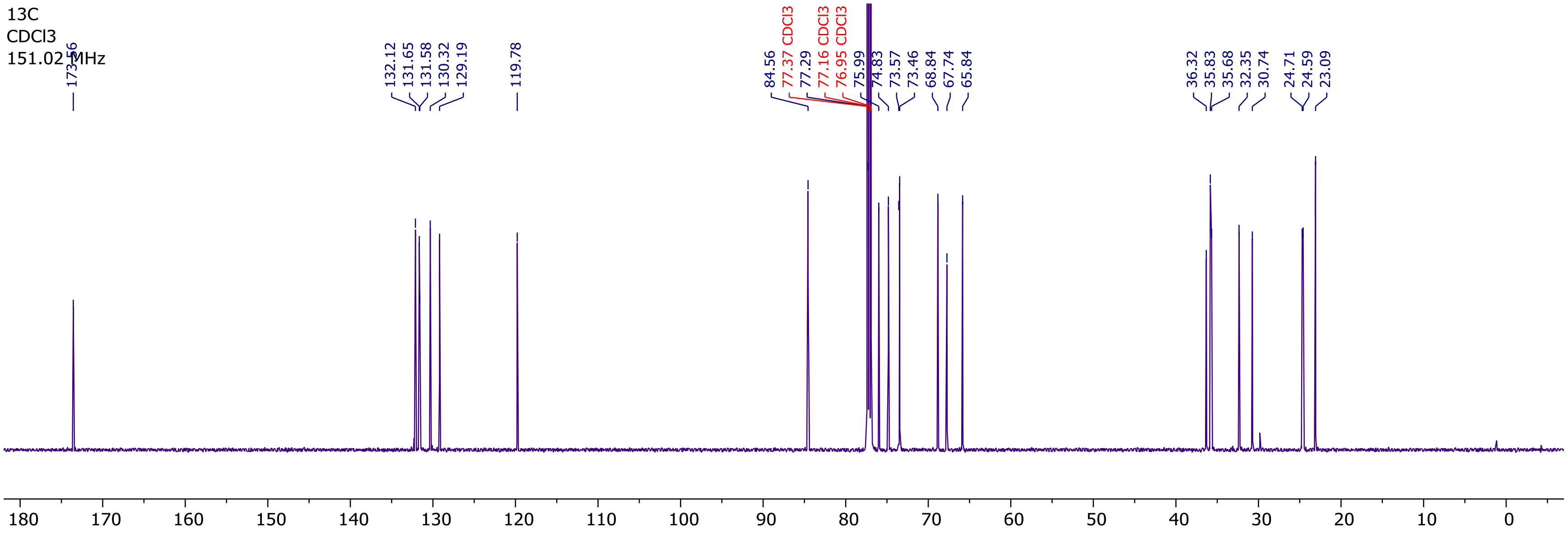
13C
CDCl3
151.02 MHz
173.56
132.12
131.65
131.58
130.32
129.19
119.78
84.56
77.37 CDCl3
77.29
77.16 CDCl3
76.95 CDCl3
75.99
74.83
73.57
73.46
68.84
67.74
65.84
36.32
35.83
35.68
32.35
30.74
24.71
24.59
23.09
180 170 160 150 140 130 120 110 100 90 80 70 60 50 40 30 20 10 0

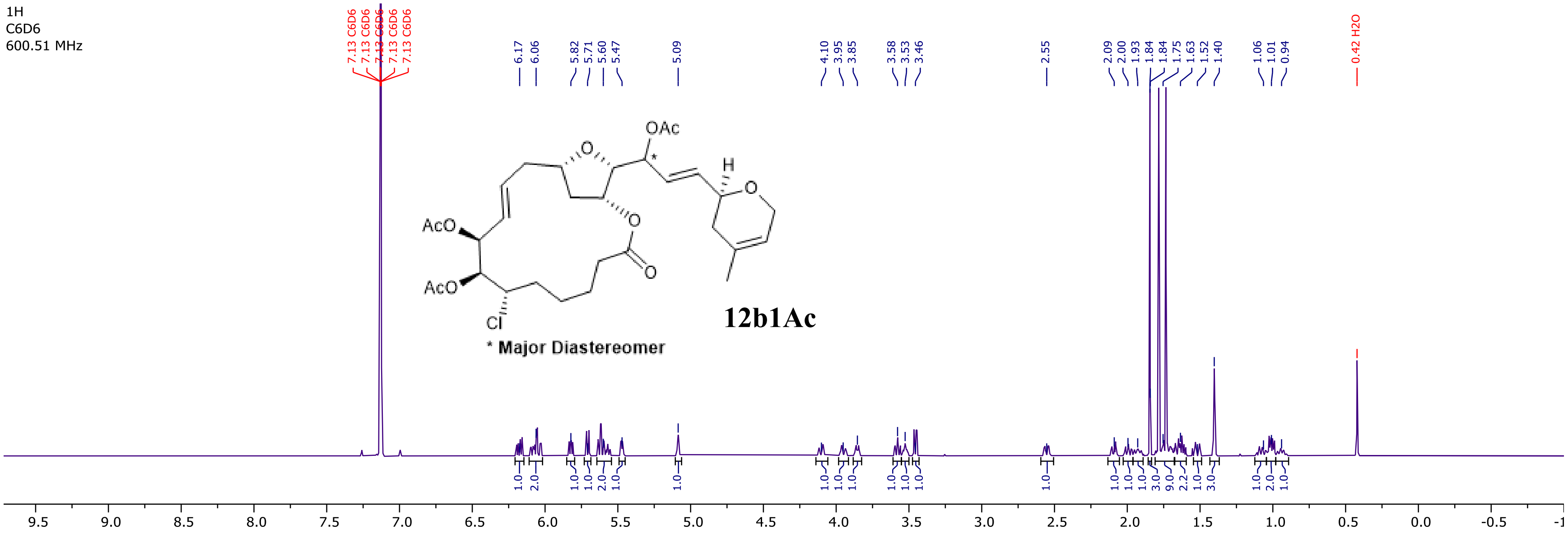
1H
C6D6
600.51 MHz
7.13 C6D6
6.17
6.06
5.82
5.71
5.60
5.47
5.09
4.10
3.95
3.85
3.58
3.53
3.46
2.55
2.09
2.00
1.93
1.84
1.84
1.75
1.63
1.52
1.40
1.06
1.01
0.94
0.42 H2O
OAc
H
O
AcO
AcO
Cl
* Major Diastereomer
12b1Ac

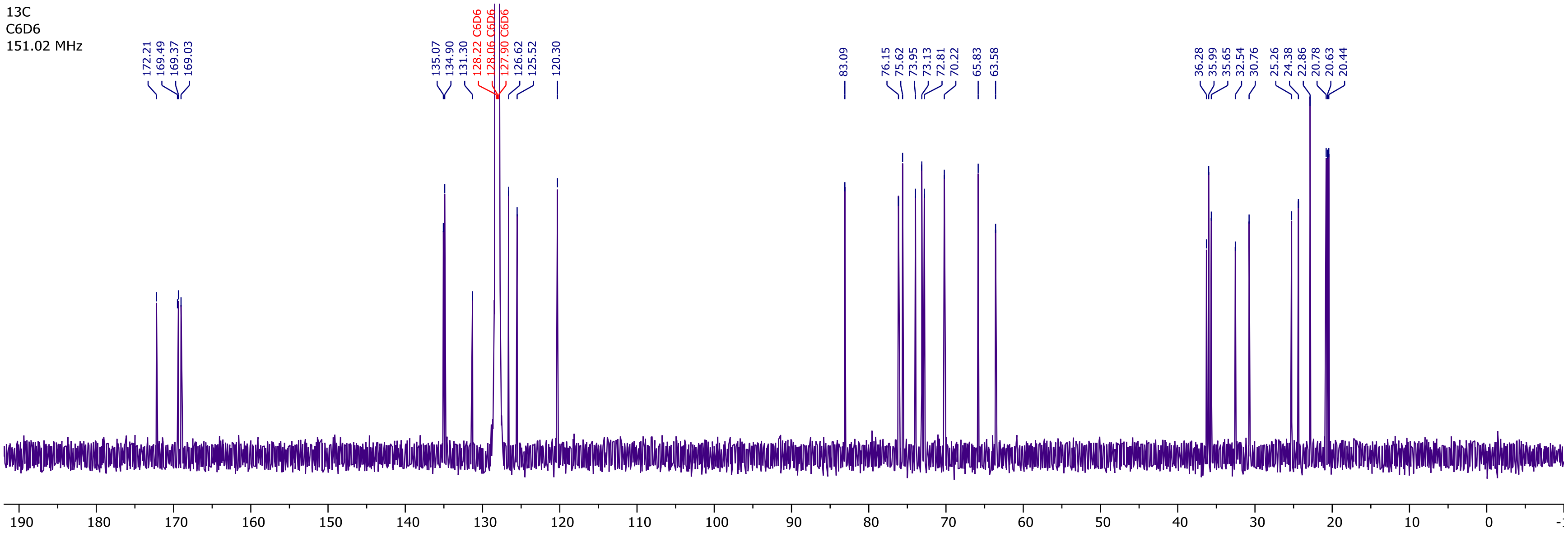
13C
C6D6
151.02 MHz
172.21
169.49
169.37
169.03
135.07
134.90
131.30
128.22 C6D6
128.06 C6D6
127.90 C6D6
126.62
125.52
120.30
83.09
76.15
75.62
73.95
73.13
72.81
70.22
65.83
63.58
36.28
35.99
35.65
32.54
30.76
25.26
24.38
22.86
20.78
20.63
20.44

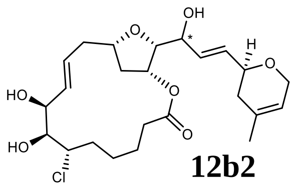

**12b2**

An approximately 1.2 M solution of aqueous HCl in MeOH was prepared by slowly adding concentrated HCl (1 mL, *ca.* 12 M) to MeOH (9 mL). To a cold (0 °C), stirred solution of a mixture of the major and minor diastereomers of **S158** and **S159** (5.7 mg, 0.011 mmol, 1.0 eq.) in $CH_2Cl_2$ (0.55 mL) and MeOH (0.55 mL) was added the previously prepared solution of aqueous HCl in MeOH (46.5 µL, *ca.* 1.2M in MeOH, 0.0558 mmol, 5.1 eq.) via microsyringe. The reaction vessel was moved to a fridge (4 °C) and allowed to stir in the fridge for 17 h. After this time, the reaction mixture was warmed to rt, and stirred for 4 h 30 min at rt. After this time, the reaction mixture was quenched by addition of $NaHCO_3$ (30 mg, 0.357 mmol, 32.5 eq.) and allowed to stir vigorously for 10 min. After this time, the reaction mixture was filtered and solvent was removed in vacuo. The crude product was then purified via flash column chromatography (7:2.25:0.75 Hexanes/EtOAc/EtOH to 6:3:1 Hexanes/EtOAc/EtOH). Appropriate fractions were pooled, and solvent was removed in vacuo to yield the minor diastereomer of **12b** (1.8 mg, 2.6%, 6 steps from **1a**).

**Analytical Data for 12b2 (minor diastereomer of 12b):**

$R_f$ = 0.42 (92.5:7.5 $CH_2Cl_2$/MeOH)

$[\alpha]_D^{20}$ = -32° (c = 0.14, $CH_2Cl_2$)

$^1$H NMR (601 MHz, MeOD) δ 5.94 (ddd, *J* = 15.8, 10.3, 2.6 Hz, 1H), 5.81 (ddd, *J* = 15.6, 5.1, 1.0 Hz, 1H), 5.75 (ddd, *J* = 15.7, 9.5, 2.3 Hz, 1H), 5.66 (ddd, *J* = 15.6, 7.1, 1.5 Hz, 1H), 5.42 (ddt, *J* = 3.9, 2.6, 1.3 Hz, 1H), 5.28 (dd, *J* = 6.8, 3.5 Hz, 1H), 4.36 (dd, *J* = 8.5, 7.2 Hz, 1H), 4.16 (dd, *J* = 9.5, 2.9 Hz, 1H), 4.15 – 4.10 (m, 3H), 4.02 – 3.96 (m, 1H), 3.70 (dd, *J* = 9.6, 2.8 Hz, 1H), 3.57 (dd, *J* = 8.4, 3.6 Hz, 1H), 3.51 (td, *J* = 10.3, 9.5, 1.8 Hz, 1H), 2.85 – 2.77 (m, 1H), 2.49 – 2.34 (m, 3H), 2.30 (ddd, *J* = 16.2, 10.3, 2.4 Hz, 1H), 2.01 – 1.83 (m, 5H), 1.71 – 1.67 (m, 3H), 1.57 (ddt, *J* = 13.3, 9.9, 4.2 Hz, 1H), 1.42 (ddd, *J* = 14.5, 7.5, 1.1 Hz, 1H), 1.38 – 1.31 (m, 1H), 1.16 – 1.06 (m, 1H).

$^{13}$C NMR (151 MHz, MeOD) δ 174.16, 134.95, 132.56, 132.25, 130.55, 129.62, 120.69, 86.00, 78.78, 77.40, 75.77, 74.46, 74.43, 72.04, 68.34, 66.55, 38.33, 36.51, 36.24, 33.14, 32.25, 26.03, 24.95, 23.02.

HRMS (ESI): Anal. Calcd. for $C_{24}H_{36}ClO_7^+$ $[M+H]^+$ 471.2144, found 471.2137

IR (neat): $\nu_{max}$ $(cm^{-1})$ = 3410 (br, OH), 2935 (m, CH), 2902 (m, CH), 2866 (m, CH), 2835 (m, CH), 1726 (s, C=O), 1441 (m), 1376 (m), 1339 (w), 1245 (w)

**12b2Ac** was prepared according to general procedure A (0.57 mg, 45%)

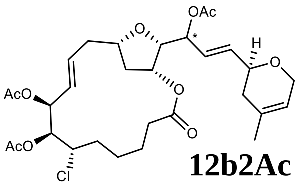


**12b2Ac**

* Minor Diastereomer

**Analytical Data for 12b2Ac:**

$R_f$ = 0.64 (2:3 Hexanes/EtOAc)

HRMS (ESI): Anal. Calcd. for $C_{30}H_{45}NO_{10}Cl^+$ $[M+NH_4]^+$ 614.2727, found 614.2706

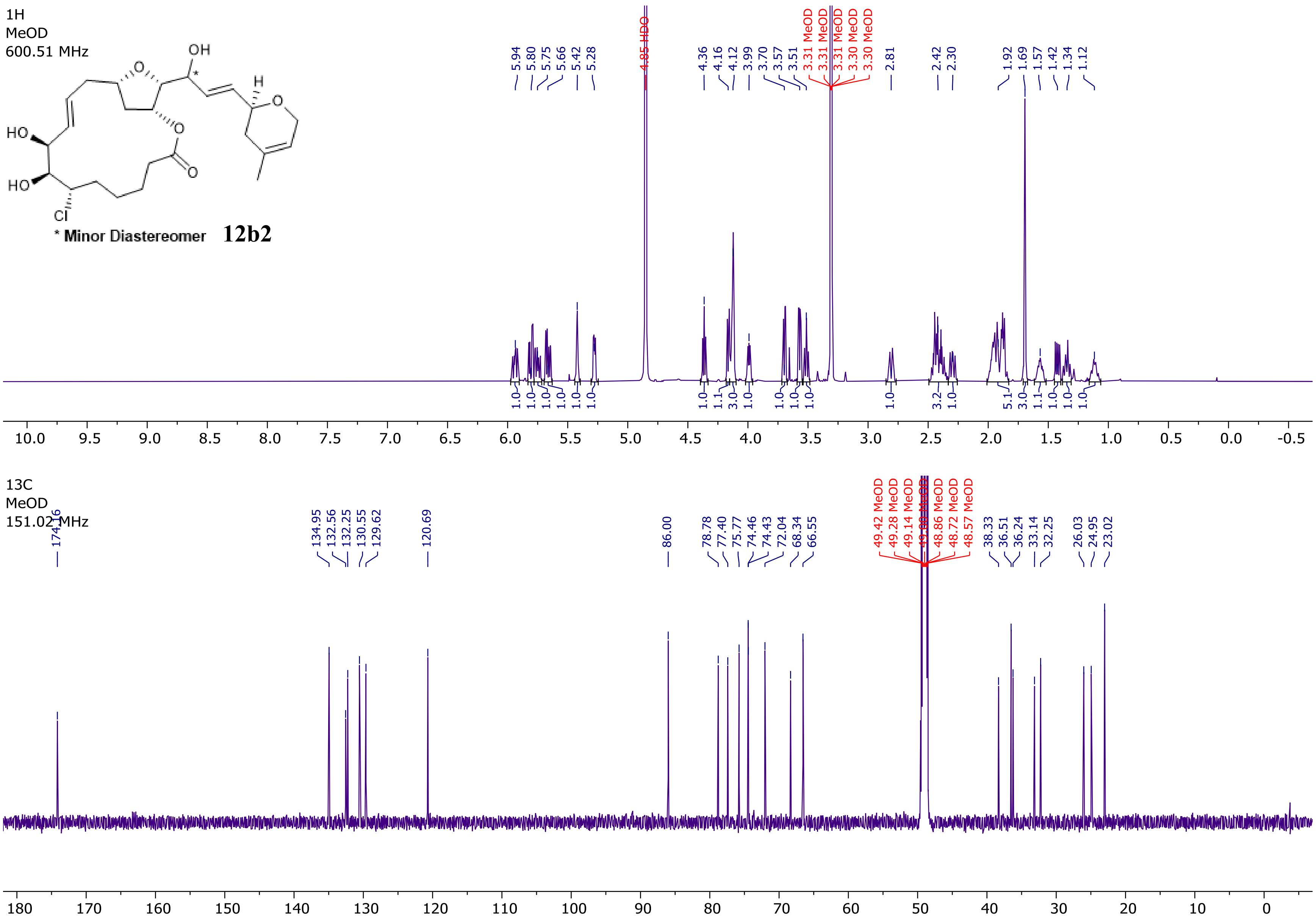
1H
MeOD
600.51 MHz
OH
HO
HO
Cl
O
O
O
H
O
* Minor Diastereomer
13C
MeOD
151.02 MHz

**12b2**

To a solution of **S157** (36.8 mg, 0.095 mmol, 1.0 eq.) in deoxygenated DMSO (5x freeze-pump-thaw cycles) (1 mL) was added $CrCl_2$ doped with 1 % $NiCl_2$ (w/w) (122.2 mg, 0.994 mmol, 10.5 eq.). **3a** (68.1 mg, 0.285 mmol, 3.0 eq.) was added via syringe in deoxygenated DMSO (0.5 mL + 2x 0.25 mL rinses) and the mixture was stirred for 68 h at rt. After this time, the reaction mixture was transferred to a separatory funnel, diluted with EtOAc (10 mL) and 15 mL of 1:1 (v/v) $H_2O$-brine was added. The organic layer was separated, and the aqueous layer extracted with EtOAc (10x 20 mL). The combined organic layers were dried ($MgSO_4$), filtered, and solvent was removed in vacuo. The crude product was purified via flash column chromatography (97:3 $CH_2Cl_2$/EtOH). Appropriate fractions were pooled, and solvent was removed in vacuo to yield **S160** (20 mg, 42%) as a white foam and a 4:1 mixture of diastereomers. A portion of the product was used immediately in the subsequent step.

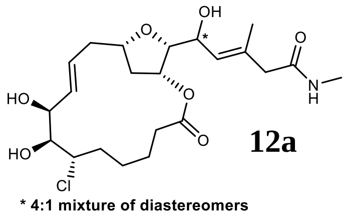

**12a**

An approximately 1.2 M solution of aqueous HCl in MeOH was prepared as follows. Concentrated HCl (1 mL, *ca.* 12 M) was slowly added to MeOH (9 mL). To a cold (0 °C), stirred solution of **S160** (4.8 mg, 0.0096 mmol, 1.0 eq.) in $CH_2Cl_2$ (0.5 mL) and MeOH (0.5 mL) was added the previously prepared solution of aqueous HCl in MeOH (41 µL, *ca.* 1.2M in MeOH, 0.049 mmol, 5.1 eq.) via microsyringe. The reaction vessel was moved to a fridge (4 °C) and allowed to stir in the fridge for 38 h. After this time, the reaction mixture was warmed to rt, and stirred for 5 h 30 min at rt. After this time, the reaction mixture was quenched by addition of $NaHCO_3$ (16.4 mg, 0.195 mmol, 20.3 eq.) and allowed to stir vigorously for 10 min. After this time, the reaction mixture was filtered and solvent was removed in vacuo. The crude product was then purified via flash column chromatography (3:5.25:1.75 Hexanes/EtOAc/EtOH). Appropriate fractions were pooled, and solvent was removed in vacuo to yield **12a** (2.7 mg, 17%, 6 steps from **1a**) as a mixture of diastereomers (4:1).

**Analytical Data for 12a:**

$R_f$ = 0.32 (9:1 $CH_2Cl_2$/MeOH)

$^1$H NMR (601 MHz, $CDCl_3$) δ 6.30 (d, *J* = 6.6 Hz, 0.8H), 5.95 (ddd, *J* = 15.7, 10.5, 2.7 Hz, 0.2H), 5.86 – 5.73 (m, 2H), 5.51 (dd, *J* = 7.1, 3.3 Hz, 0.8H), 5.46 – 5.41 (m, 1H), 5.33 – 5.28 (m, 0.2H), 4.62 (dd, *J* = 9.4, 8.4 Hz, 0.2H), 4.49 (t, *J* = 8.5 Hz, 0.8H), 4.23 – 4.19 (m, 1H), 4.19 – 4.12 (m, 1H), 3.86 (dd, *J* = 9.7, 2.9 Hz, 1H), 3.68 – 3.59 (m, 1.2H), 3.57 (dd, *J* = 8.2, 3.2 Hz, 0.8H), 3.05 (d, *J* = 15.6 Hz, 0.8H), 2.98 (d, *J* = 15.3 Hz, 0.2H), 2.94 (d, *J* = 15.6 Hz, 1H), 2.91 – 2.72 (m, 4H), 2.55 – 2.13 (m, 7H), 2.06 – 1.84 (m, 3H), 1.78 – 1.73 (m, 3H), 1.67 – 1.52 (m, 1.8H), 1.49 – 1.43 (m, 0.2H), 1.41 – 1.32 (m, 1H), 1.24 – 1.16 (m, 0.8H), 1.07 (q, *J* = 12.7 Hz, 0.2H).

$^{13}$C NMR (151 MHz, $CDCl_3$) δ 173.94, 172.96, 171.26, 170.69, 137.56, 134.93, 131.38, 130.65, 130.27, 129.92, 129.47, 126.97, 85.19, 77.24, 76.13, 75.92, 74.39, 73.74, 73.51, 73.47, 67.68, 66.72, 65.56, 47.95, 47.60, 37.51, 36.25, 36.01, 35.49, 32.54, 32.27, 30.71, 30.66, 29.84, 26.62, 26.60, 24.81, 24.73, 24.64, 17.79, 17.03.

HRMS (ESI): Anal. Calcd. for $C_{22}H_{35}ClNO_7^+$ [M+H]$^+$ 460.2097, found 460.2086

IR (neat): $\nu_{max}$ $(cm^{-1})$ = 3358 (br, OH), 2942 (m, CH), 2869 (m, CH), 1725 (s, C=O), 1636 (s, C=C), 1552 (m), 1418 (m), 1378 (m), 1342 (m), 1231 (m)

**12aAc** was prepared according to general procedure A (1.12 mg, 71%)

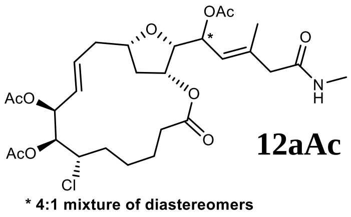


**12aAc**

**Analytical Data for 12aAc:**

$R_f$ = 0.17 (EtOAc)

HRMS (ESI): Anal. Calcd. for $C_{28}H_{41}NO_{10}Cl^+$ [M+H]$^+$ 586.2414, found 586.2395

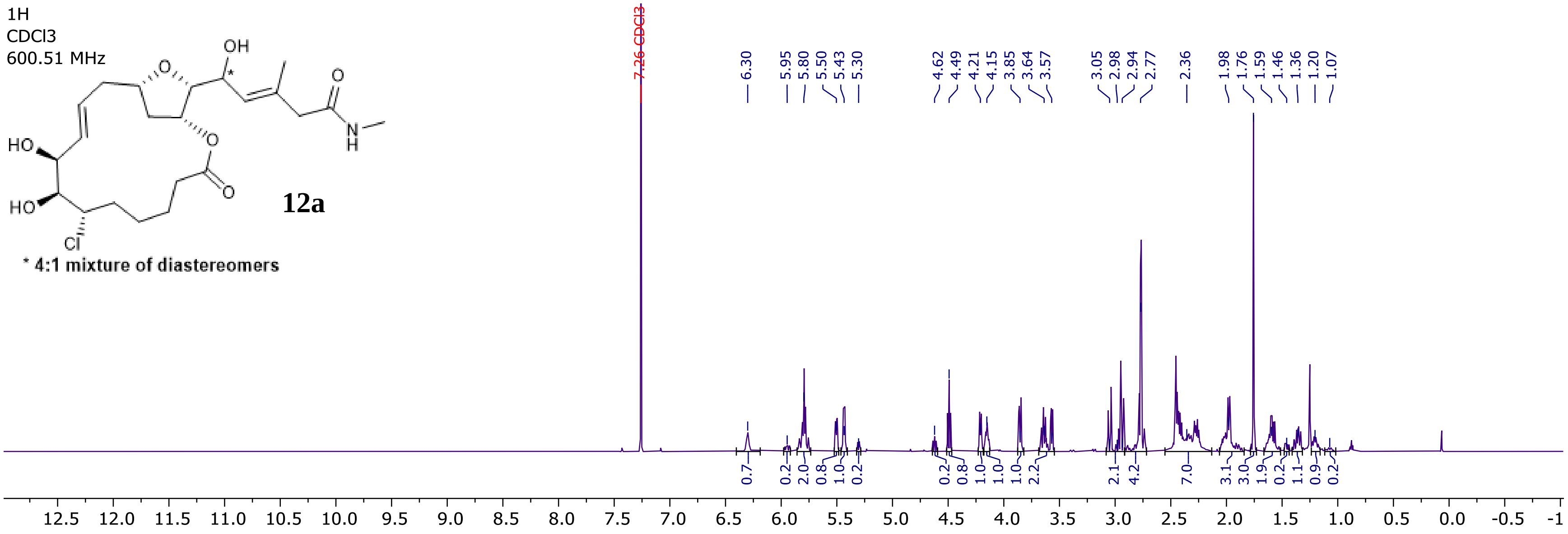
1H
CDCl3
600.51 MHz
OH
HO
HO
Cl
O
N
H
12a
* 4:1 mixture of diastereomers
7.26 CDCl3
6.30
5.95
5.80
5.50
5.43
5.30
4.62
4.49
4.21
4.15
3.85
3.64
3.57
3.05
2.98
2.94
2.77
2.36
1.98
1.76
1.59
1.46
1.36
1.20
1.07

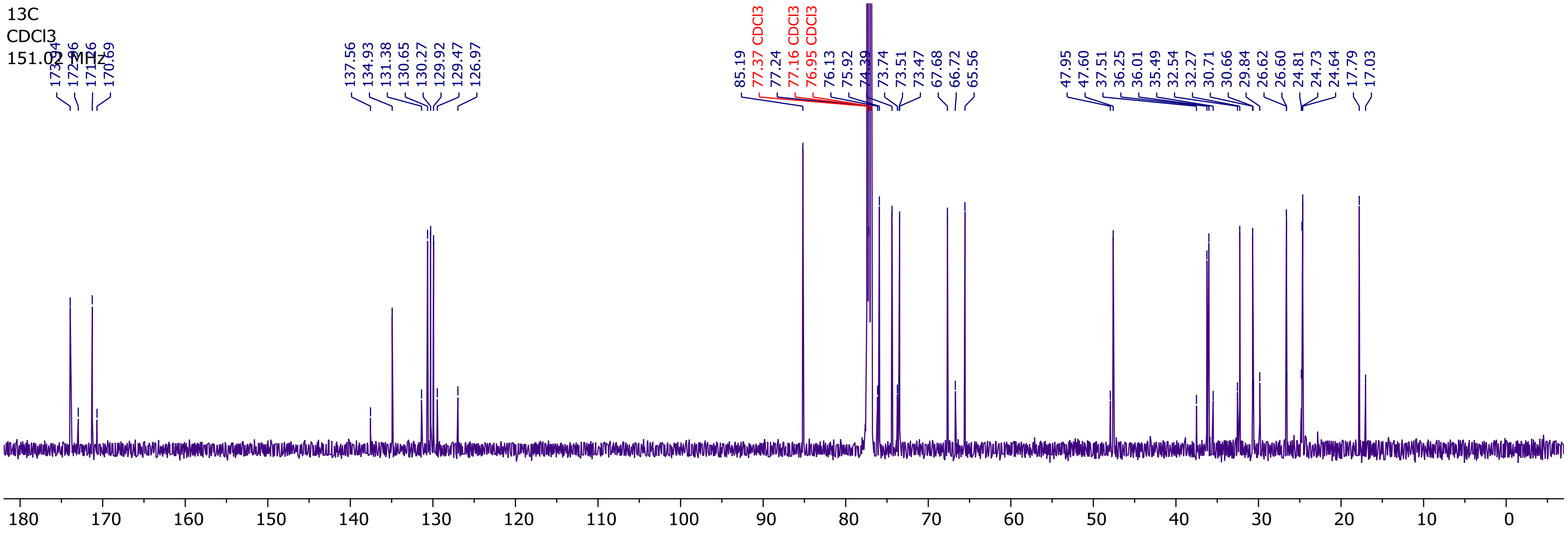
13C
CDCl3
151.02 MHz
137.56
134.93
131.38
130.65
130.27
129.92
129.47
126.97
85.19
77.37 CDCl3
77.24
77.16 CDCl3
76.95 CDCl3
76.13
75.92
73.74
73.51
73.47
67.68
66.72
65.56
47.95
47.60
37.51
36.25
36.01
35.49
32.54
32.27
30.71
30.66
29.84
26.62
26.60
24.81
24.73
24.64
17.79
17.03

To a solution of **S157** (36.8 mg, 0.095 mmol, 1.0 eq.) in deoxygenated DMSO (5x freeze-pump-thaw cycles) (2 mL) was added $CrCl_2$ doped with 1 % $NiCl_2$ (w/w) (111.2 mg, 0.905 mmol, 9.5 eq.). **3c** (42 µL, 71.8 mg, 0.284 mmol, 3.0 eq.) was added via microsyringe and the mixture was stirred for 65 h at rt. After this time, the reaction mixture was transferred to a separatory funnel, diluted with EtOAc (10 mL) and 15 mL of 1:1 (v/v) $H_2O$-brine was added. The organic layer was separated, and the aqueous layer extracted with EtOAc (10x 20 mL). The combined organic layers were dried ($MgSO_4$), filtered, and solvent was removed in vacuo. The crude product was purified via flash column chromatography (3:2 Hexanes/Acetone). Appropriate fractions were pooled, and solvent was removed in vacuo to yield the faster eluting diastereomer **S161** (10.5 mg, 22%), slower eluting diastereomer **S162** (10.8 mg, 22%) and a mixture of the major and minor diastereomers (4 mg, 8.2%) all as white foams. The products were used immediately in the subsequent steps.

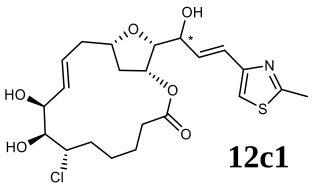


**12c1**

* Faster Eluting Diastereomer

An approximately 1.2 M solution of aqueous HCl in MeOH was prepared by slowly adding concentrated HCl (1 mL, *ca.* 12 M) to MeOH (9 mL). To a cold (0 °C), stirred solution of the faster eluting diastereomer of **S161** (10.5 mg, 0.0204 mmol, 1.0 eq.) in $CH_2Cl_2$ (1.0 mL) and MeOH (1.0 mL) was added the previously prepared solution of aqueous HCl in MeOH (85 µL, *ca.* 1.2M in MeOH, 0.102 mmol, 5.0 eq.) via microsyringe. The reaction vessel was moved to a fridge (4 °C) and allowed to stir in the fridge for 22 h 30 min. After this time, the reaction mixture was warmed to rt, and stirred for 3 h 30 min at rt. After this time, the reaction vessel was transferred back to the fridge (4 °C) and allowed to stir in the fridge for a further 17 h. After this time, the reaction mixture was quenched by addition of $NaHCO_3$ (55 mg, 0.655 mmol, 32.1 eq.) and allowed to stir vigorously for 10 min. After this time, the reaction mixture was filtered and solvent was removed in vacuo. The crude product was then purified via flash column chromatography (49:1 to 24:1 $CH_2Cl_2$/MeOH). Appropriate fractions were pooled, and solvent was removed in vacuo to yield the faster eluting diastereomer of **12c** (7.9 mg, 12%, 6 steps from **1a**) as a white foam.

**Analytical Data for 12c1 (faster eluting diastereomer of 12c):**

$R_f$ = 0.47 (92.5:7.5 $CH_2Cl_2$/MeOH)

$[\alpha]_D^{20}$ = +160° (c = 0.71, MeOH)

$^1$H NMR (601 MHz, MeOD) δ 7.19 (s, 1H), 6.71 – 6.63 (m, 2H), 5.92 (ddd, *J* = 15.8, 10.3, 2.6 Hz, 1H), 5.75 (ddd, *J* = 15.7, 9.4, 2.2 Hz, 1H), 5.51 (ddd, *J* = 7.3, 3.6, 1.1 Hz, 1H), 4.45 (dd, *J* = 8.3, 4.5 Hz, 1H), 4.17 (dd, *J* = 9.5, 2.8 Hz, 1H), 4.13 (tt, *J* = 7.9, 2.0 Hz, 1H), 3.70 (dd, *J* = 9.6, 2.7 Hz, 1H), 3.58 (dd, *J* = 8.4, 3.6 Hz, 1H), 3.53 (ddd, *J* = 11.5, 9.5, 1.8 Hz, 1H), 2.77 (ddt, *J* = 15.9, 4.5, 2.6 Hz, 1H), 2.69 (s, 3H), 2.51 – 2.37 (m, 3H), 2.28 (ddd, *J* = 16.2, 10.3, 2.4 Hz, 1H), 2.00 – 1.85 (m, 3H), 1.58 (ddt, *J* = 15.3, 12.5, 4.8 Hz, 1H), 1.45 (ddd, *J* = 14.5, 7.5, 1.2 Hz, 1H), 1.38 – 1.30 (m, 1H), 1.14 (tdd, *J* = 16.1, 8.8, 4.0 Hz, 1H).

$^{13}$C NMR (151 MHz, MeOD) δ 174.43, 168.35, 154.57, 134.29, 132.21, 130.56, 124.22, 116.12, 85.64, 78.81, 77.67, 75.61, 74.44, 69.91, 68.39, 37.73, 36.33, 33.16, 32.33, 26.06, 25.13, 18.75.

HRMS (ESI): Anal. Calcd. for $C_{22}H_{31}ClNO_6S^+$ $[M+H]^+$ 472.1555, found 472.1551

IR (neat): $\nu_{max}$ $(cm^{-1})$ = 3399 (br, OH), 3112 (w, C=*CH*), 3058 (w, C=*CH*), 2942 (m, CH), 2870 (m, CH), 1724 (s, C=O), 1669 (w, C=C), 1507 (w), 1433 (m), 1378 (m), 1378 (m), 1231 (m)

**12c1Ac** was prepared according to general procedure A (1.17 mg, 75%)

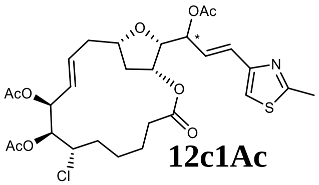


**12c1Ac**

* Faster Eluting Diastereomer

**Analytical Data for 12c1Ac:**

$R_f$ = 0.69 (2:3 Hexanes/EtOAc)

$[\alpha]_D^{20}$ = +130° (c = 0.67, $C_6D_6$)

$^1$H NMR (601 MHz, $C_6D_6$) δ 7.15 – 7.11 (m, 1H), 6.78 (dd, *J* = 15.5, 1.1 Hz, 1H), 6.30 (s, 1H), 6.10 (ddd, *J* = 15.4, 10.7, 2.6 Hz, 1H), 5.98 (ddd, *J* = 8.9, 7.0, 1.1 Hz, 1H), 5.74 (dd, *J* = 10.2, 2.6 Hz, 1H), 5.65 (dd, *J* = 10.0, 2.7 Hz, 1H), 5.59 (ddd, *J* = 15.4, 9.9, 2.2 Hz, 1H), 5.50 (dd, *J* = 6.9, 3.4 Hz, 1H), 3.61 (ddd, *J* = 11.2, 10.2, 2.1 Hz, 1H), 3.56 – 3.50 (m, 2H), 2.54 (dq, *J* = 15.5, 2.8 Hz, 1H), 2.23 (s, 3H), 2.13 (ddd, *J* = 14.5, 5.6, 4.3 Hz, 1H), 2.05 (ddd, *J* = 14.4, 11.8, 3.8 Hz, 1H), 1.87 (s, 3H), 1.84 – 1.69 (m, 9H), 1.68 – 1.62 (m, 2H), 1.16 – 1.02 (m, 3H), 0.98 (ddt, *J* = 16.0, 9.7, 5.0 Hz, 1H).

$^{13}$C NMR (151 MHz, $C_6D_6$) δ 172.22, 169.50, 169.37, 169.16, 165.57, 153.32, 135.17, 128.99, 127.00, 125.41, 116.07, 82.92, 76.12, 75.61, 73.97, 72.88, 70.82, 63.58, 36.31, 35.59, 32.59, 30.82, 25.28, 24.40, 20.81, 20.64, 20.44, 18.88.

HRMS (ESI): Anal. Calcd. for $C_{28}H_{37}ClNO_9S^+$ $[M+H]^+$ 598.1872, found 598.1885

IR (neat): $\nu_{max}$ $(cm^{-1})$ = 2950 (m, CH), 2869 (m, CH), 1739 (vs, CO), 1659 (w, C=C), 1509 (w), 1435 (w), 1371 (m), 1235 (vs, CO).

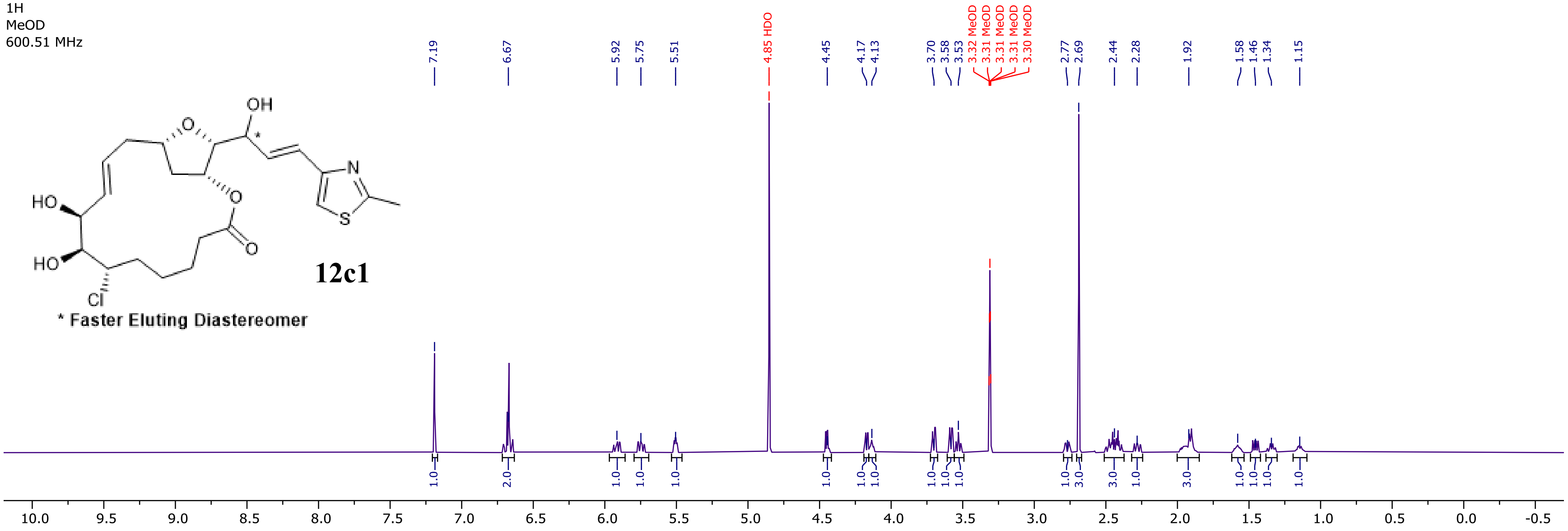
1H
MeOD
600.51 MHz
OH
O
HO
HO
O
O
N
S
Cl
* Faster Eluting Diastereomer
12c1
7.19
6.67
5.92
5.75
5.51
4.85 HDO
4.45
4.17
4.13
3.70
3.58
3.53
3.32 MeOD
3.31 MeOD
3.31 MeOD
3.31 MeOD
3.30 MeOD
2.77
2.69
2.44
2.28
1.92
1.58
1.46
1.34
1.15
10.0 9.5 9.0 8.5 8.0 7.5 7.0 6.5 6.0 5.5 5.0 4.5 4.0 3.5 3.0 2.5 2.0 1.5 1.0 0.5 0.0 -0.5

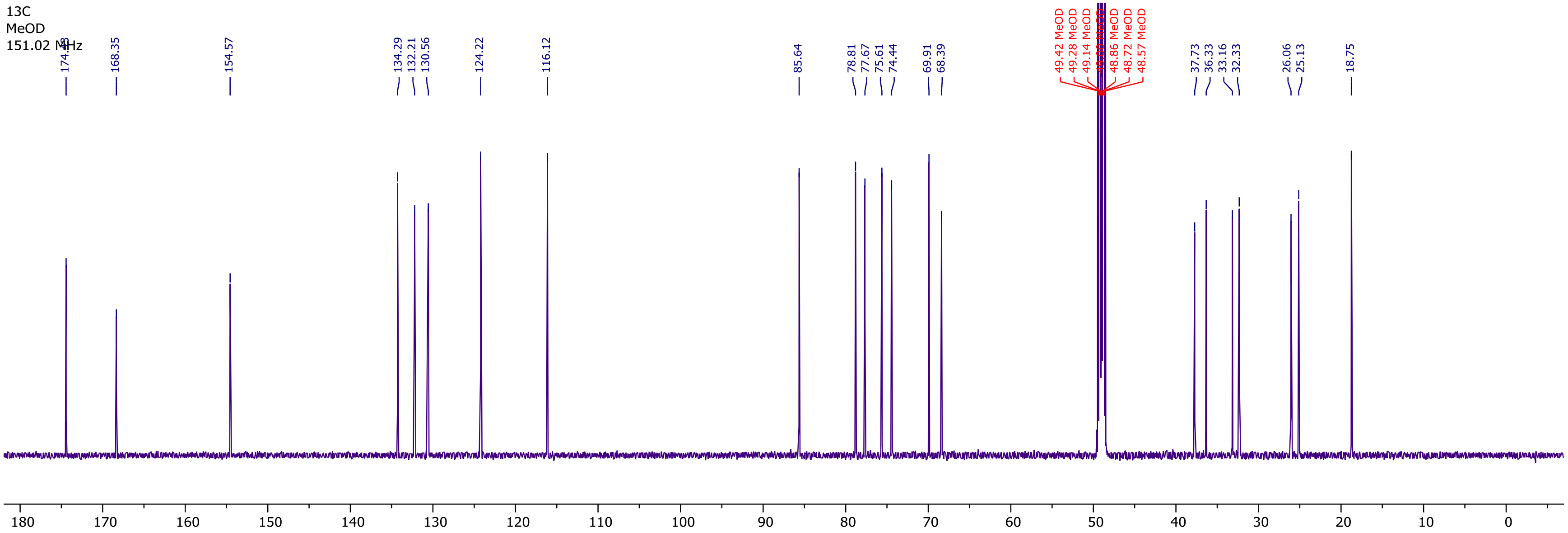
13C
MeOD
151.02 MHz
174.43
168.35
154.57
134.29
132.21
130.56
124.22
116.12
85.64
78.81
77.67
75.61
74.44
69.91
68.39
49.42 MeOD
49.28 MeOD
49.14 MeOD
48.86 MeOD
48.72 MeOD
48.57 MeOD
37.73
36.33
33.16
32.33
26.06
25.13
18.75
180 170 160 150 140 130 120 110 100 90 80 70 60 50 40 30 20 10 0

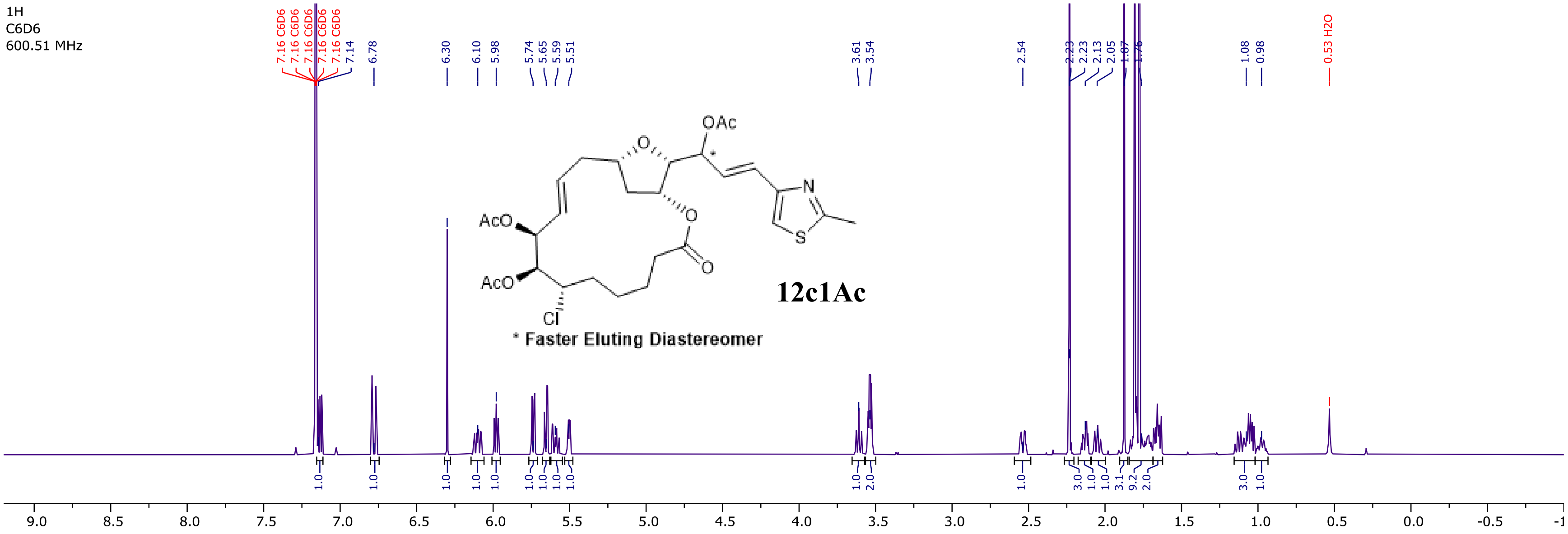
1H
C6D6
600.51 MHz
7.16 C6D6
7.16 C6D6
7.16 C6D6
7.16 C6D6
7.16 C6D6
7.14
6.78
6.30
6.10
5.98
5.74
5.65
5.59
5.51
3.61
3.54
2.54
2.23
2.23
2.13
2.05
1.87
1.76
1.08
0.98
0.53 H2O
OAc
O
N
S
AcO
AcO
Cl
O
O
12c1Ac
* Faster Eluting Diastereomer
1.0
1.0
1.0
1.0
1.0
1.0
1.0
1.0
1.0
1.0
2.0
1.0
3.0
1.0
1.0
3.1
9.2
2.0
3.0
1.0
9.0
8.5
8.0
7.5
7.0
6.5
6.0
5.5
5.0
4.5
4.0
3.5
3.0
2.5
2.0
1.5
1.0
0.5
0.0
-0.5

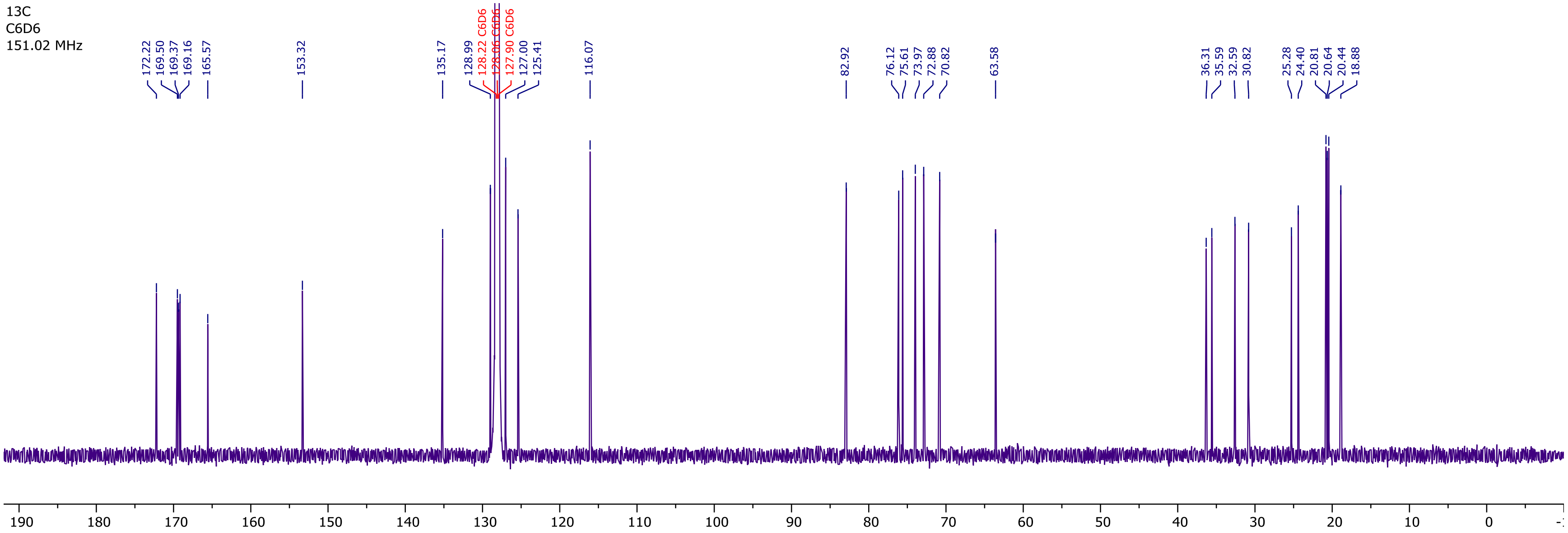
13C
C6D6
151.02 MHz
172.22
169.50
169.37
169.16
165.57
153.32
135.17
128.99
128.22 C6D6
128.06 C6D6
127.90 C6D6
127.00
125.41
116.07
82.92
76.12
75.61
73.97
72.88
70.82
63.58
36.31
35.59
32.59
30.82
25.28
24.40
20.81
20.64
20.44
18.88
190
180
170
160
150
140
130
120
110
100
90
80
70
60
50
40
30
20
10
0

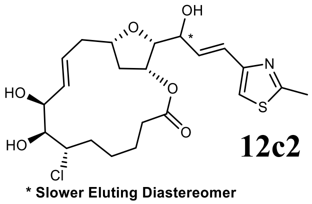


**12c2**

An approximately 1.2 M solution of aqueous HCl in MeOH was prepared by slowly adding concentrated HCl (1 mL, *ca.* 12 M) to MeOH (9 mL). To a cold (0 °C), stirred solution of the faster eluting diastereomer of **S162** (10.8 mg, 0.0211 mmol, 1.0 eq.) in $CH_2Cl_2$ (1.0 mL) and MeOH (1.0 mL) was added the previously prepared solution of aqueous HCl in MeOH (87.5 µL, *ca.* 1.2M in MeOH, 0.105 mmol, 5.0 eq.) via microsyringe. The reaction vessel was moved to a fridge (4 °C) and allowed to stir in the fridge for 22 h 30 min. After this time, the reaction mixture was warmed to rt, and stirred for 3 h 30 min at rt. After this time, the reaction vessel was transferred back to the fridge (4 °C) and allowed to stir in the fridge for a further 17 h. After this time, the reaction mixture was quenched by addition of $NaHCO_3$ (50 mg, 0.595 mmol, 28.2 eq.) and allowed to stir vigorously for 10 min. After this time, the reaction mixture was filtered and solvent was removed in vacuo. The crude product was then purified via flash column chromatography (49:1 to 19:1 $CH_2Cl_2$/MeOH). Appropriate fractions were pooled, and solvent was removed in vacuo to yield the slower eluting diastereomer of **12c** (6.8 mg, 10%, 6 steps from **1a**) as a white foam.

**Analytical Data for 12c2 (slower eluting diastereomer of 12c):**

$R_f$ = 0.35 (92.5:7.5 $CH_2Cl_2$/MeOH)

$[\alpha]_D^{20}$ = -31° (c = 0.53, MeOH)

$^1$H NMR (601 MHz, MeOD) δ 7.21 (s, 1H), 6.63 (dd, *J* = 15.7, 1.2 Hz, 1H), 6.41 (dd, *J* = 15.7, 6.8 Hz, 1H), 5.97 (ddd, *J* = 15.8, 10.3, 2.6 Hz, 1H), 5.76 (ddd, *J* = 15.7, 9.5, 2.3 Hz, 1H), 5.31 (dd, *J* = 6.9, 3.6 Hz, 1H), 4.53 (ddd, *J* = 8.2, 6.8, 1.3 Hz, 1H), 4.22 – 4.10 (m, 2H), 3.70 (dd, *J* = 9.6, 2.8 Hz, 1H), 3.66 (dd, *J* = 8.3, 3.6 Hz, 1H), 3.52 (ddd, *J* = 11.3, 9.5, 1.8 Hz, 1H), 2.86 – 2.79 (m, 1H), 2.68 (s, 3H), 2.51 – 2.42 (m, 2H), 2.39 (dt, *J* = 15.3, 4.7 Hz, 1H), 2.31 (ddd, *J* = 16.1, 10.3, 2.4 Hz, 1H), 1.97 – 1.81 (m, 3H), 1.61 – 1.51 (m, 1H), 1.45 (ddd, *J* = 14.6, 7.5, 1.1 Hz, 1H), 1.39 – 1.31 (m, 1H), 1.12 (dtd, *J* = 14.1, 10.5, 4.7 Hz, 1H).

$^{13}$C NMR (151 MHz, MeOD) δ 174.15, 168.53, 154.09, 132.25, 131.05, 130.57, 125.93, 116.89, 85.98, 78.77, 77.40, 75.92, 74.44, 72.05, 68.35, 38.42, 36.22, 33.07, 32.26, 26.01, 24.90, 18.76.

HRMS (ESI): Anal. Calcd. for $C_{22}H_{31}ClNO_6S^+$ $[M+H]^+$ 472.1555, found 472.1548

IR (neat): $\nu_{max}$ $(cm^{-1})$ = 3410 (br, OH), 3113 (w, C=*CH*), 2933 (m, CH), 2867 (m, CH), 1722 (s, C=O), 1659 (w, C=C), 1505 (w), 1433 (m), 1375 (m), 1338 (m), 1248 (m), 1220 (m)

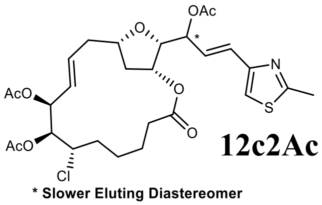


**12c2Ac**

**12c2Ac** was prepared according to general procedure A (1.26 mg, 77%)

**Analytical Data for 12c2Ac:**

$R_f$ = 0.38 (2:3 Hexanes/EtOAc)

$[\alpha]_D^{20}$ = +10° (c = 0.36, $C_6D_6$)

$^1$H NMR (601 MHz, $C_6D_6$) δ 6.82 (dd, *J* = 15.4, 7.5 Hz, 1H), 6.77 (d, *J* = 15.5 Hz, 1H), 6.44 (s, 1H), 6.17 (ddd, *J* = 15.5, 10.4, 2.5 Hz, 1H), 6.11 (dd, *J* = 8.5, 7.5 Hz, 1H), 5.78 (dd, *J* = 10.2, 2.6 Hz, 1H), 5.67 (dd, *J* = 9.9, 2.7 Hz, 1H), 5.60 (ddd, *J* = 15.6, 9.9, 2.2 Hz, 1H), 5.19 (dd, *J* = 6.7, 3.6 Hz, 1H), 3.63 – 3.56 (m, 3H), 2.63 (ddt, *J* = 15.7, 4.3, 2.5 Hz, 1H), 2.23 (s, 3H), 1.87 (s, 3H), 1.84 – 1.63 (m, 13H), 1.23 (tdd, *J* = 13.3, 10.9, 4.1 Hz, 1H), 1.14 – 1.08 (m, 1H), 1.05 (ddd, *J* = 14.6, 7.3, 1.1 Hz, 1H), 1.03 – 0.96 (m, 1H).

$^{13}$C NMR (151 MHz, $C_6D_6$) δ 171.74, 169.54, 169.51, 169.44, 166.05, 153.06, 135.41, 128.31, 126.36, 125.28, 116.30, 82.38, 75.72, 75.52, 74.04, 73.92, 73.39, 63.48, 37.45, 35.49, 31.60, 30.78, 24.98, 23.69, 20.87, 20.61, 20.43, 18.91.

HRMS (ESI): Anal. Calcd. for $C_{28}H_{37}ClNO_9S^+$ $[M+H]^+$ 598.1872, found 598.1837

IR (neat): $\nu_{max}$ $(cm^{-1})$ = 2979 (m, CH), 2925 (m, CH), 1739 (vs, CO), 1659 (w, C=C), 1435 (m), 1372 (m), 1238 (vs, CO).

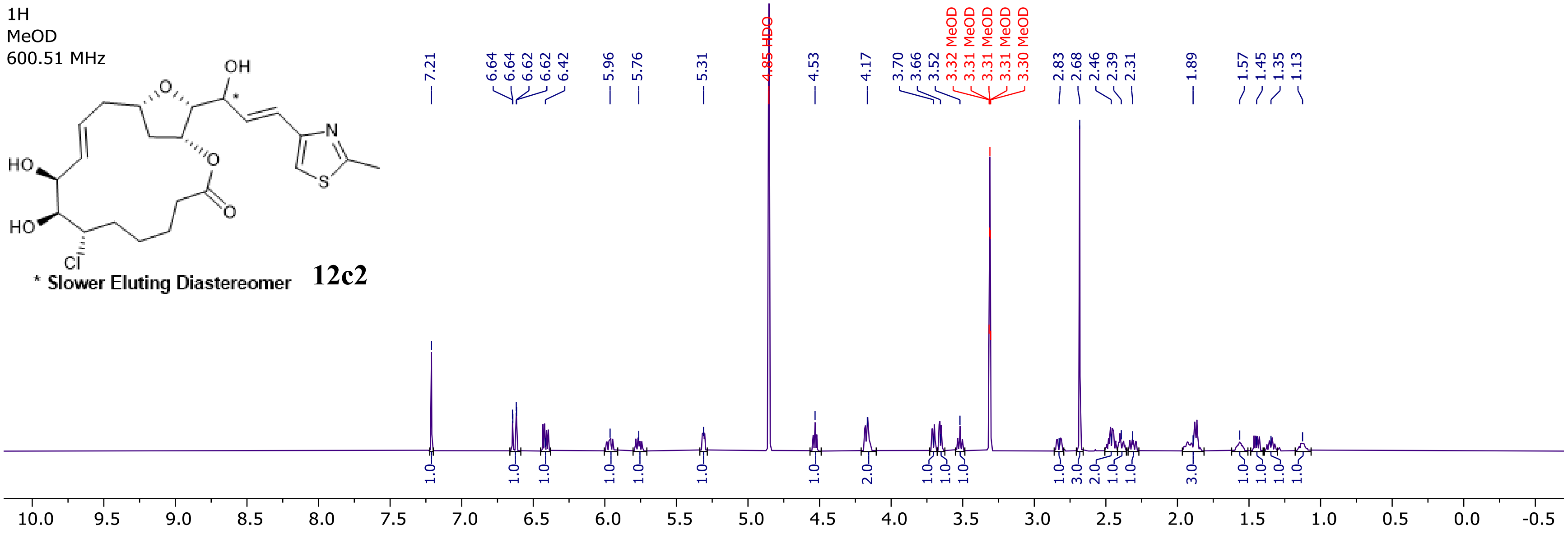
1H
MeOD
600.51 MHz
OH
HO
HO
Cl
O
O
O
N
S
* Slower Eluting Diastereomer
12c2
7.21
6.64
6.64
6.62
6.62
6.42
5.96
5.76
5.31
4.85 HDO
4.53
4.17
3.70
3.66
3.52
3.32 MeOD
3.31 MeOD
3.31 MeOD
3.31 MeOD
3.30 MeOD
2.83
2.68
2.46
2.39
2.31
1.89
1.57
1.45
1.35
1.13
1.0
1.0
1.0
1.0
1.0
1.0
1.0
2.0
1.0
1.0
1.0
1.0
3.0
2.0
1.0
1.0
3.0
1.0
1.0
1.0
1.0
10.0
9.5
9.0
8.5
8.0
7.5
7.0
6.5
6.0
5.5
5.0
4.5
4.0
3.5
3.0
2.5
2.0
1.5
1.0
0.5
0.0
-0.5

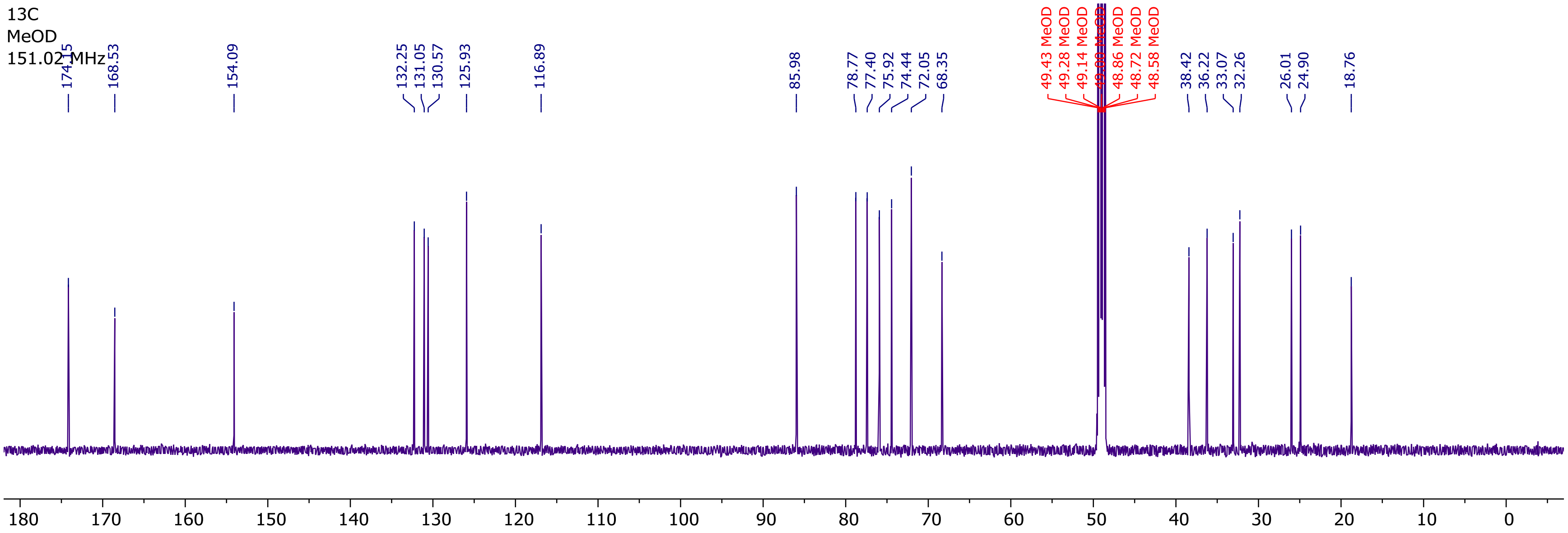
13C
MeOD
151.02 MHz
174.15
168.53
154.09
132.25
131.05
130.57
125.93
116.89
85.98
78.77
77.40
75.92
74.44
72.05
68.35
49.43 MeOD
49.28 MeOD
49.14 MeOD
48.86 MeOD
48.72 MeOD
48.58 MeOD
38.42
36.22
33.07
32.26
26.01
24.90
18.76
180
170
160
150
140
130
120
110
100
90
80
70
60
50
40
30
20
10
0

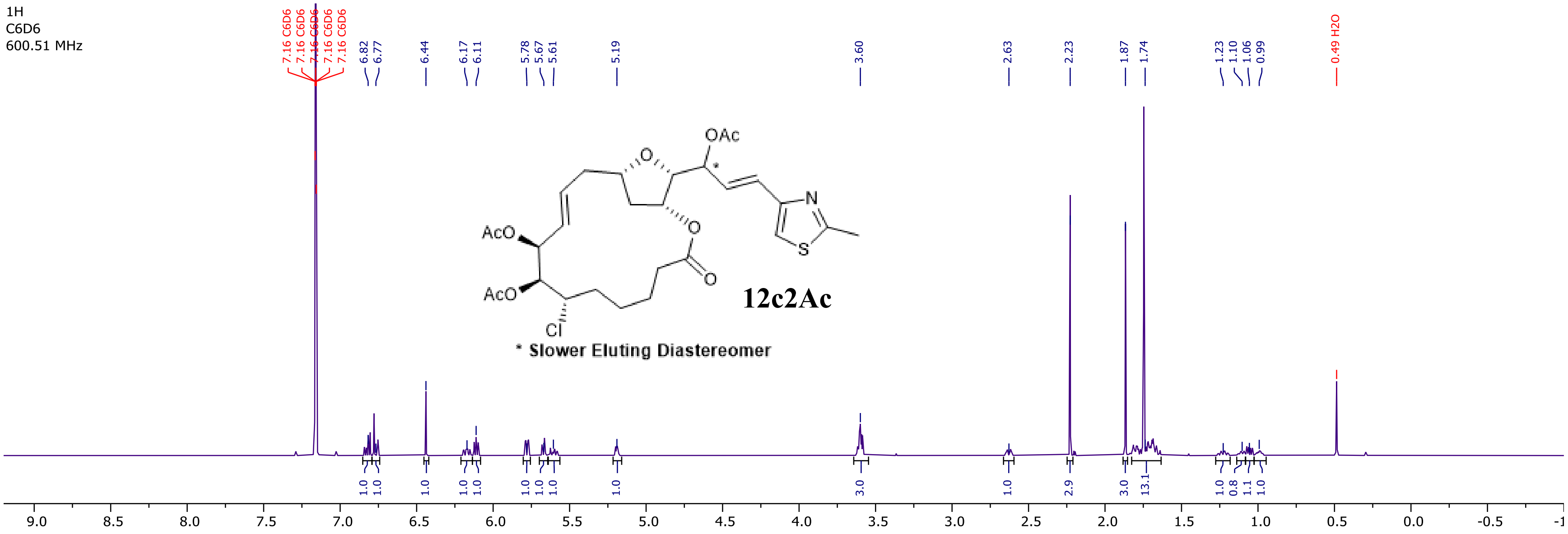
1H
C6D6
600.51 MHz
7.16 C6D6 7.16 C6D6 7.16 C6D6 7.16 C6D6 7.16 C6D6
6.82 6.77 6.44 6.17 6.11 5.78 5.67 5.61 5.19 3.60 2.63 2.23 1.87 1.74 1.23 1.10 1.06 0.99
0.49 H2O
OAc
O
N
S
AcO
AcO
Cl
12c2Ac
* Slower Eluting Diastereomer
1.0 1.0 1.0 1.0 1.0 1.0 1.0 1.0 1.0 3.0 1.0 2.9 3.0 13.1 1.0 0.8 1.1 1.0
9.0 8.5 8.0 7.5 7.0 6.5 6.0 5.5 5.0 4.5 4.0 3.5 3.0 2.5 2.0 1.5 1.0 0.5 0.0 -0.5

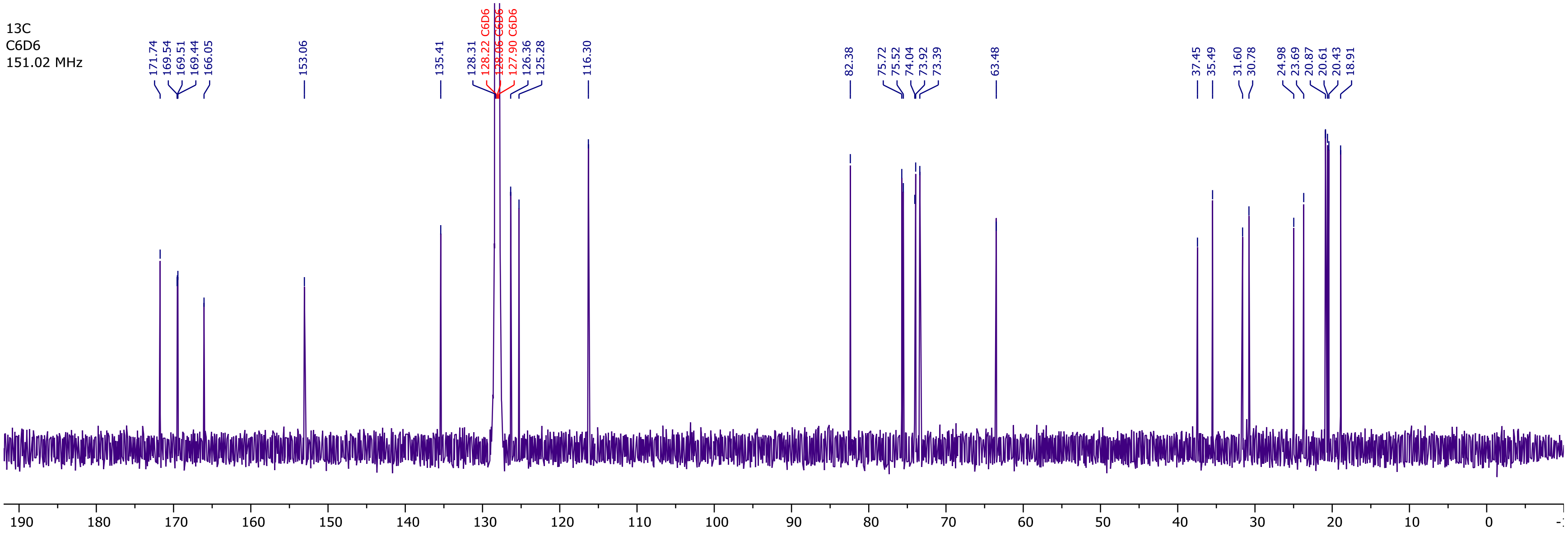
13C
C6D6
151.02 MHz
171.74 169.54 169.51 169.44 166.05 153.06 135.41 128.31
128.22 C6D6 128.06 C6D6 127.90 C6D6
126.36 125.28 116.30 82.38 75.72 75.52 74.04 73.92 73.39 63.48 37.45 35.49 31.60 30.78 24.98 23.69 20.87 20.61 20.43 18.91
190 180 170 160 150 140 130 120 110 100 90 80 70 60 50 40 30 20 10 0

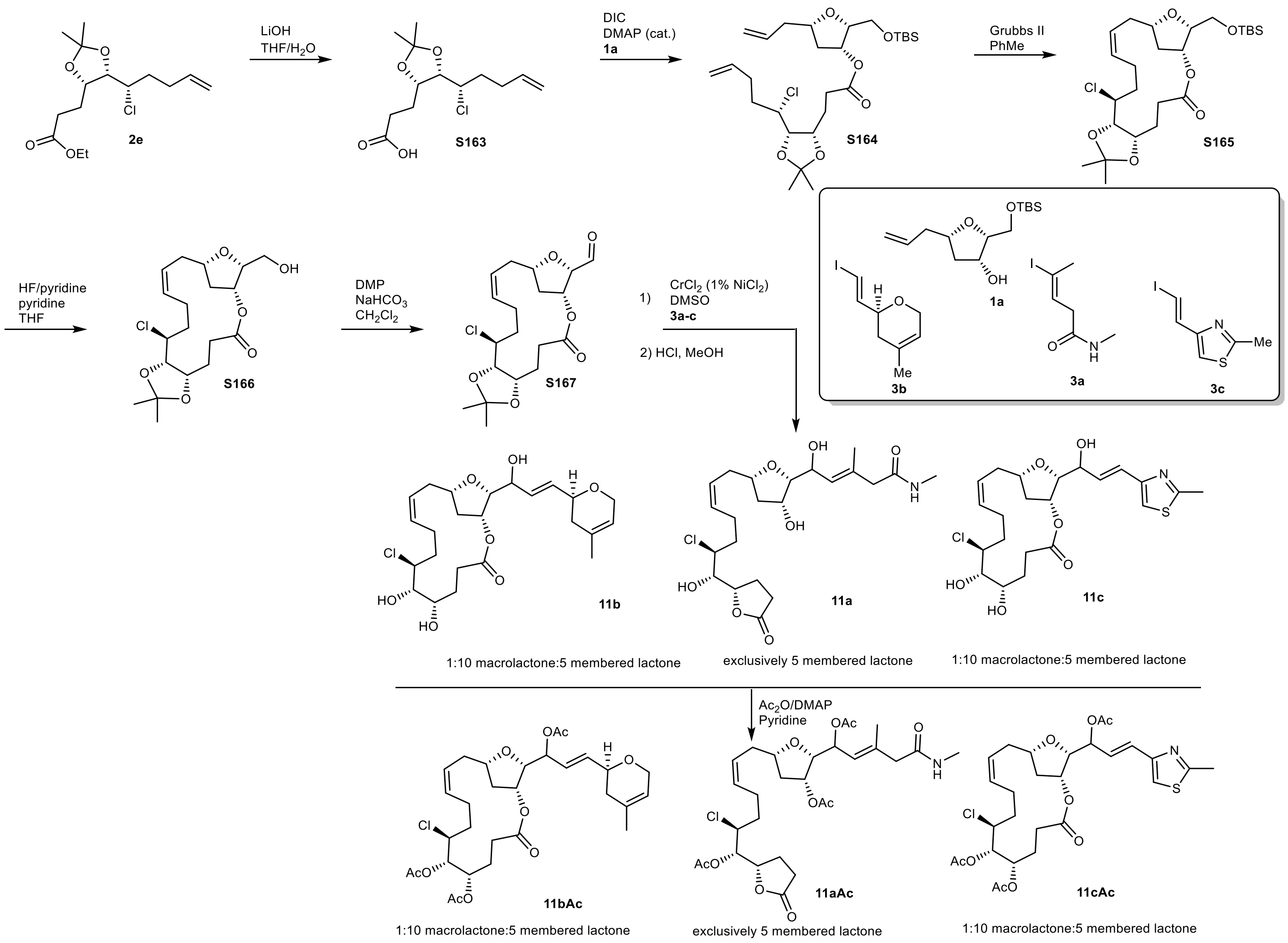


**Supplementary Fig. 52 | Synthesis of pMLs 11b-c and 11bAc-cAc.**

THF = tetrahydrofuran, DIC = N,N'-diisopropylcarbodiimide, DMAP = 4-dimethylaminopyridine, TBS = tert-butyldimethylsilyl, Grubbs II = Dichloro[1,3-bis(2,4,6-trimethylphenyl)-2-imidazolidinylidene](benzylidene)(tricyclohexylphosphine)ruthenium(II), DMP = Dess-Martin periodinane, DMSO = dimethylsulfoxide.

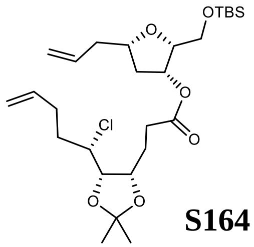


**S164**

To a rt, stirred solution of **2e** (252 mg, 0.82 mmol, 1.0 eq.) in THF (3.1 mL) and $H_2O$ (1.5 mL) was added a solution of LiOH (2.7 mL, 1M in $H_2O$, 2.7 mmol, 3.3 eq.). The reaction mixture was vigorously stirred for 3 h 30 min. After this time, starting material was consumed as monitored by TLC analysis. The reaction mixture was diluted with $H_2O$ (15 mL) and $Et_2O$ (15 mL), transferred to a separatory funnel, and the mixture adjusted to *ca.* pH 4-5 with HCl (0.6M in $H_2O$). The organic layer was then separated, and the aqueous layer was extracted with EtOAc (4x 15 mL). The combined organic layers were dried ($MgSO_4$), filtered, and solvent was removed in vacuo to yield crude acid **S163** (224.5 mg) as a colorless oil of which a portion was used immediately in the next step without further purification.

To a rt, stirred solution of acid **S163** (162 mg, 0.58 mmol, 1.2 eq.), alcohol **1a** (137.5 mg, 0.50 mmol, 1.0 eq.), and 4-dimethylaminopyridine (24.4 mg, 0.20 mmol, 0.40 eq.) in $CH_2Cl_2$ (4.5 mL) was added N,N'-diisopropylcarbodiimide (0.10 mL, 81 mg, 0.64 mmol, 1.3 eq.). The reaction mixture was allowed to stir for 16 h 30 min. at rt. After this time, starting material was consumed as monitored by TLC analysis. The cloudy reaction mixture was poured onto $H_2O$ (10 mL) in a separatory funnel, and the aqueous layer extracted with $CH_2Cl_2$ (3x 10 mL). The combined organic layers were dried ($Na_2SO_4$), filtered, and solvent was removed in vacuo to yield an oily white residue. The crude product was purified via flash column chromatography (4:1 Hexanes/EtOAc) to give **S164** (251 mg, 94%) as a yellow oil.

**Analytical Data for S164:**

$R_f$ = 0.67 (3:2 Hexanes/$Et_2O$)

$[\alpha]_D^{20}$ = -43° (c = 0.90, $CDCl_3$)

$^1$H NMR (600 MHz, $CDCl_3$) δ 5.84 – 5.72 (m, 2H), 5.33 (p, *J* = 3.5 Hz, 1H), 5.13 – 5.01 (m, 4H), 4.18 – 4.14 (m, 1H), 4.14 – 4.10 (m, 1H), 3.97 (p, *J* = 6.7 Hz, 1H), 3.90 – 3.84 (m, 2H), 3.78 (d, *J* = 5.6 Hz, 2H), 2.57 (ddd, *J* = 16.7, 8.3, 5.2 Hz, 1H), 2.47 – 2.39 (m, 3H), 2.39 – 2.33 (m, 1H), 2.29 (dt, *J* = 13.7, 6.6 Hz, 1H), 2.23 (dt, *J* = 15.2, 7.7 Hz, 1H), 2.02 – 1.93 (m, 1H), 1.84 (dtt, *J* = 16.1, 11.7, 5.8 Hz, 1H), 1.80 – 1.72 (m, 2H), 1.64 (ddd, *J* = 14.2, 6.7, 2.6 Hz, 1H), 1.51 (s, 3H), 1.36 (s, 3H), 0.87 (s, 9H), 0.05 (s, 3H), 0.04 (s, 3H).

$^{13}$C NMR (151 MHz, $CDCl_3$) δ 172.64, 136.75, 134.54, 117.35, 116.46, 108.89, 81.68, 80.54, 77.41, 76.42, 74.23, 61.32, 59.54, 40.45, 38.26, 34.42, 30.84, 30.36, 27.56, 26.00, 25.85, 24.35, 18.42, -5.17, -5.27.

HRMS (ESI): Anal. Calcd. for $C_{27}H_{51}NO_6SiCl^+$ $[M+NH_4]^+$ 548.3169, found 548.3156

IR (neat): $\nu_{max}$ $(cm^{-1})$ = 3078 (w, C=*CH*), 2982 (m, CH), 2932 (m, CH), 2857 (m, CH), 1736 (s, C=O), 1466 (m), 1442 (m), 1254

1H
CDCl3
600.13 MHz

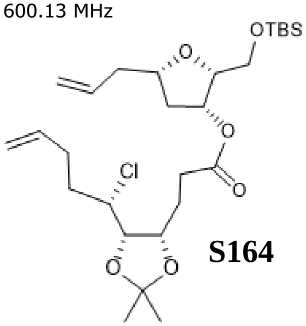


**S164**

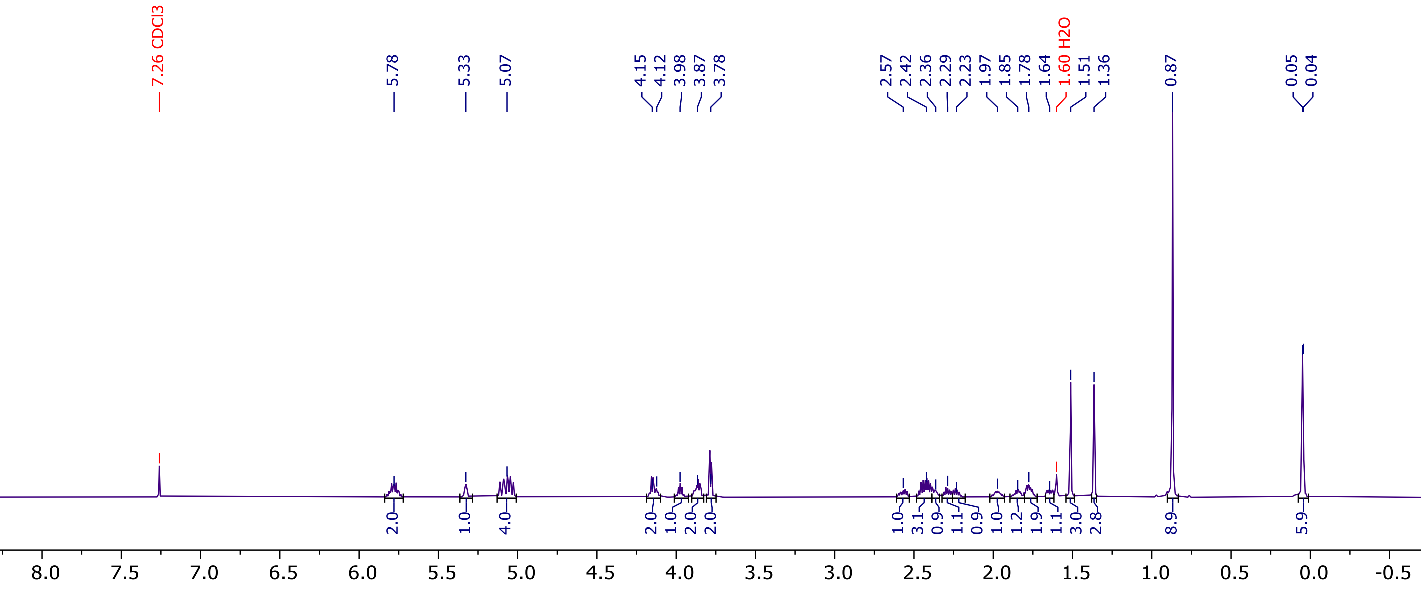


13C
CDCl3
150.92 MHz

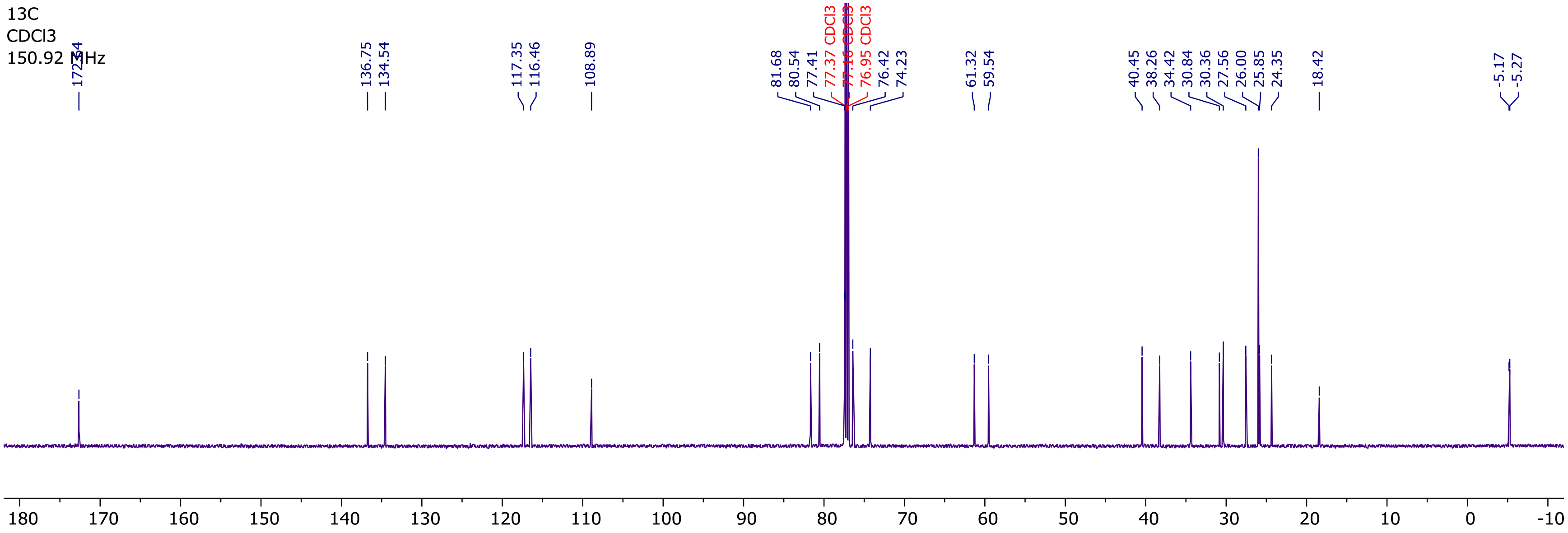

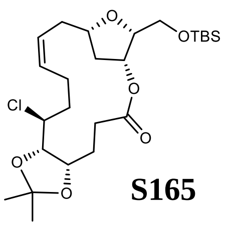


**S165**

To a warm (60 °C), stirred solution of **S164** (326.5 mg, 0.61 mmol, 1.0 eq.) in toluene (235 mL) that was continuously being sparged with $N_2$(g) was added Grubbs second generation catalyst (100.5 mg, 0.118 mmol, 0.19 eq.) in toluene (5 mL). The reaction was allowed to proceed under continuous nitrogen sparge for 50 min. After this time, starting material was consumed as monitored by TLC analysis, the reaction mixture was cooled to rt and quenched by addition of potassium 2-isocyanoacetate (156 mg, 1.27 mmol, 2.1 eq.) in MeOH (10 mL). The reaction mixture was stirred for a further 1 h, after which time the red solution changed to a pale-yellow color. The reaction mixture was concentrated in vacuo to give a crude solid deposited on the walls of the evaporation flask. The walls were scraped with a spatula and the solid was triturated with $Et_2O$ (3x 10 mL) and filtered through a short plug of silica gel eluting with $Et_2O$. The filtrate was concentrated in vacuo and the crude product was purified via flash column chromatography (93:7 to 4:1 Hexanes/$Et_2O$). Appropriate fractions were pooled, and solvent was removed in vacuo to yield the major isomer **S165** (131.5 mg, 43%) as a waxy white solid.

**Analytical Data for S165:**

$R_f$ = 0.58 (3:2 Hexanes/$Et_2O$)

$[\alpha]_D^{20}$ = -29° (c = 0.69, $CDCl_3$)

$^1$H NMR (600 MHz, $CDCl_3$) δ 5.66 – 5.58 (m, 1H), 5.43 (dd, *J* = 5.8, 2.9 Hz, 1H), 5.33 (td, *J* = 10.5, 4.5 Hz, 1H), 4.22 – 4.13 (m, 2H), 4.04 (ddd, *J* = 11.5, 5.2, 1.7 Hz, 1H), 3.90 – 3.83 (m, 2H), 3.80 – 3.73 (m, 2H), 2.57 (ddd, *J* = 14.6, 12.0, 2.9 Hz, 1H), 2.50 (ddd, *J* = 14.0, 6.0, 3.1 Hz, 1H), 2.44 (dtd, *J* = 13.8, 4.7, 2.3 Hz, 1H), 2.38 – 2.26 (m, 3H), 2.22 (tdt, *J* = 13.0, 9.2, 5.0 Hz, 1H), 1.79 – 1.69 (m, 2H), 1.66 (tdd, *J* = 13.9, 3.2, 1.8 Hz, 1H), 1.48 (s, 5H), 1.39 (s, 3H), 0.86 (s, 9H), 0.04 (s, 3H), 0.03 (s, 3H).

$^{13}$C NMR (151 MHz, $CDCl_3$) δ 171.28, 129.36, 127.29, 109.51, 82.53, 80.82, 77.24, 75.39, 73.87, 61.08, 59.45, 37.89, 35.74, 35.19, 30.50, 30.44, 28.81, 26.32, 25.97, 25.92, 24.32, 18.42, -5.23, -5.33.

HRMS (ESI): Anal. Calcd. for $C_{25}H_{47}NO_6SiCl^+$ $[M+NH_4]^+$ 520.2856, found 520.2851

IR (neat): $\nu_{max}\ (cm^{-1})$ = 2923 (m, CH), 2858 (m, CH), 1736 (s, C=O), 1651 (w, C=C), 1466 (m), 1249 (s)

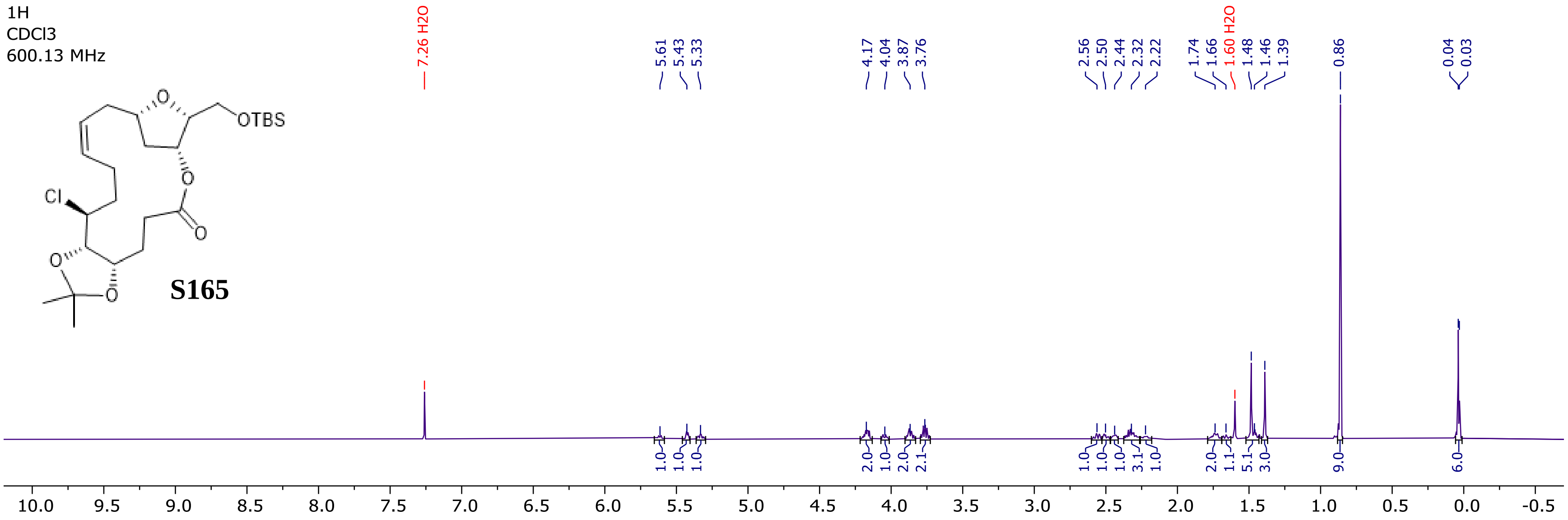
1H
CDCl3
600.13 MHz
OTBS
Cl
S165
7.26 H2O
5.61
5.43
5.33
4.17
4.04
3.87
3.76
2.56
2.50
2.44
2.32
2.22
1.74
1.66
1.60 H2O
1.48
1.46
1.39
0.86
0.04
0.03
1.0
1.0
1.0
2.0
1.0
2.0
2.1
1.0
1.0
1.0
3.1
1.0
2.0
1.1
5.1
3.0
9.0
6.0
10.0 9.5 9.0 8.5 8.0 7.5 7.0 6.5 6.0 5.5 5.0 4.5 4.0 3.5 3.0 2.5 2.0 1.5 1.0 0.5 0.0 -0.5

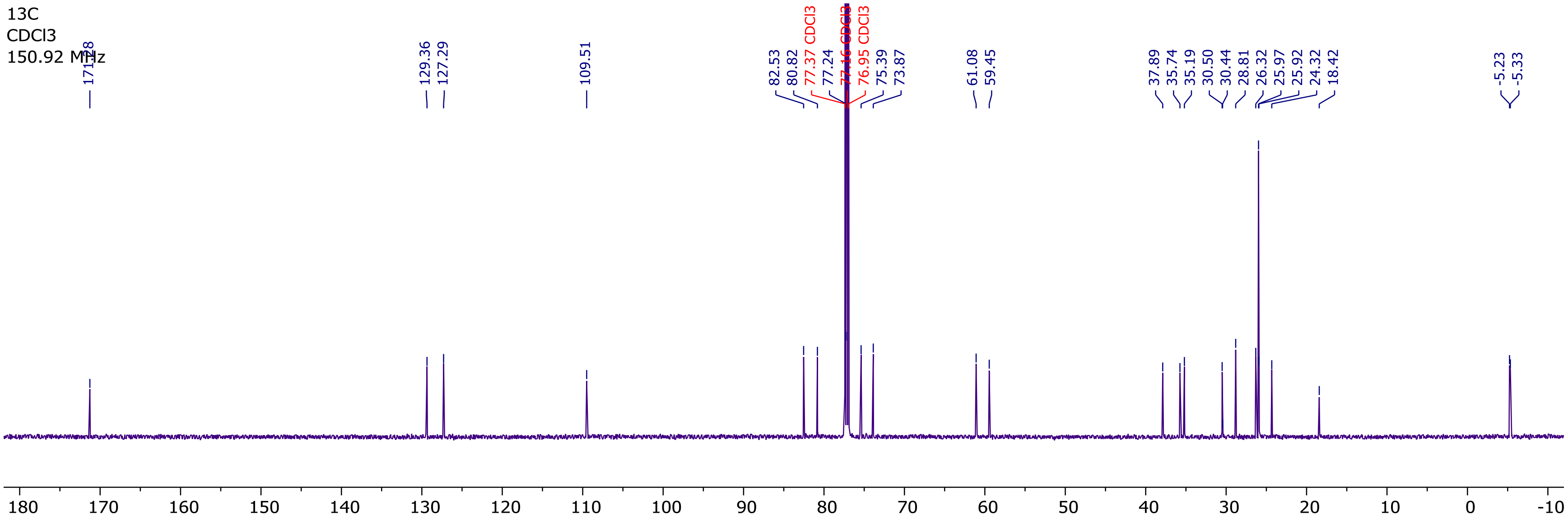
13C
CDCl3
150.92 MHz
171.28
129.36
127.29
109.51
82.53
80.82
77.37 CDCl3
77.24
77.16 CDCl3
76.95 CDCl3
75.39
73.87
61.08
59.45
37.89
35.74
35.19
30.50
30.44
28.81
26.32
25.97
25.92
24.32
18.42
-5.23
-5.33
180 170 160 150 140 130 120 110 100 90 80 70 60 50 40 30 20 10 0 -10

To a rt, stirred solution of **S165** (126 mg, 0.25 mmol, 1.0 eq.) in THF (4.4 mL) was added in sequence Pyridine (2 mL, 24.93 mmol) followed by HF-Pyr (0.49 mL, 70% w/w HF, 30% w/w Pyridine, 18.9 mmol HF, 76 eq. HF). The reaction mixture was stirred at rt for 22 h. After this time, starting material was consumed as monitored by TLC analysis. The reaction was quenched by slowly pipetting the mixture onto 35 mL saturated sodium bicarbonate. **Caution!** copious $CO_2$ (g) evolved. The aqueous layer was extracted with $CH_2Cl_2$ (3x 20 mL), and the combined organic layers were dried ($Na_2SO_4$), filtered, and solvent was removed in vacuo. The crude product was purified via flash column chromatography (3:1 Hexanes/Acetone). Appropriate fractions were pooled, and solvent as removed in vacuo to yield **S166** (86.6 mg, 87%) as a white foam, which was used immediately in the subsequent step.

To a rt, stirred solution of the above macrocyclic alcohol **S166** (77 mg, 0.20 mmol, 1.0 eq.) in $CH_2Cl_2$ (3 mL) was added $NaHCO_3$ (50 mg, 0.59 mmol, 3 eq.) followed by Dess-Martin Periodinane (109 mg, 0.257 mmol, 1.3 eq.). The mixture was stirred at rt for 1 h 10 min. After this time, starting material was consumed as monitored by TLC analysis. The reaction mixture was quenched with a 1:1:1 (v/v) saturated aqueous $NaHCO_3$-10% aqueous $Na_2S_2O_3$-$H_2O$ (3 mL) and the biphasic mixture was stirred vigorously for 1 h 20 min at rt. After this time, the aqueous layer was extracted with $CH_2Cl_2$ (5x 2 mL), and the combined organic layers were dried ($Na_2SO_4$), filtered, and the solvent was removed in vacuo. The crude product was passaged through a short plug of C2 modified silica gel eluting with $Et_2O$. Solvent was removed in vacuo to yield the crude aldehyde **S167** (*ca.* 75 mg) as a colorless oil which was portioned and used immediately in the next steps without further purification.

To a solution of **S167** (25 mg, 0.065 mmol, 1.0 eq.) in deoxygenated DMSO (5x freeze-pump-thaw cycles) (1.8 mL) was added $CrCl_2$ doped with 1 % $NiCl_2$ (w/w) (80 mg, 0.65 mmol, 10 eq.). **3b** (49 mg, 0.195 mmol, 3.0 eq.) was added via syringe in deoxygenated DMSO (2x 0.4 mL) and the mixture was stirred for 18 h at rt. After this time, the reaction mixture was transferred to a separatory funnel, diluted with $Et_2O$ (10 mL) and 10 mL of 1:1 (v/v) $H_2O$-brine was added. The organic layer was separated, and the aqueous layer extracted with $Et_2O$ (15x 15 mL). The combined organic layers were dried ($MgSO_4$), filtered, and solvent was removed in vacuo. The crude product was purified via flash column chromatography (9:1 to 8.5:1.5 Hexanes/Acetone). Appropriate fractions were pooled, and solvent was removed in vacuo to yield the alcohol (11.3 mg) as a colorless oil and a 3:1 mixture of diastereomers. The product was used immediately in the subsequent step.

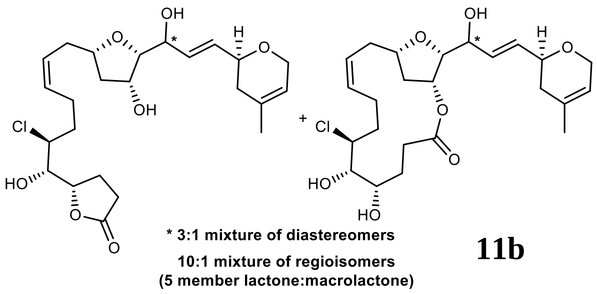


**11b**

Note: an approximately 1.2 M solution of aqueous HCl in MeOH by slowly adding concentrated HCl (1 mL, *ca.* 12 M) to MeOH (9 mL)**.** To a cold (0 °C), stirred solution of the alcohol from the previous step (11.3 mg, 0.0211 mmol, 1.0 eq.) in $CH_2Cl_2$ (2 mL) and MeOH (6 mL) was added the previously prepared solution of aqueous HCl in MeOH (93 µL, *ca.* 1.2M in MeOH, 0.11 mmol, 5.0 eq.) via microsyringe. The reaction vessel was moved to a fridge (4 °C) and allowed to stir in the fridge for 42 h. After this time, the reaction mixture was warmed to rt, and allowed to stir for a further 4 h 45 min. After this time, the reaction mixture was quenched by addition of $NaHCO_3$ (20 mg, 0.24 mmol, 11 eq.) and allowed to stir vigorously for 5 min. After this time, the reaction mixture was filtered and solvent was removed in vacuo. The crude product was then purified via flash column chromatography (49:1 to 19:1 $CH_2Cl_2$/MeOH). Appropriate fractions were pooled, and solvent was removed in vacuo to yield **11b** (9.0 mg, 25 %, 4 steps from **S165**) as 10:1 mixture of regioisomers (5 membered lactone:macrolactone) and a 3:1 mixture of epimers at the NHK centre, as a white foam.

**Analytical Data for 11b:**

$R_f$ = 0.40 (93:7 $CH_2Cl_2$/MeOH)

$^1$H NMR (601 MHz, $CDCl_3$) δ 5.97 (d, *J* = 4.3 Hz, 0.1H), 5.94 (dd, *J* = 5.0, 4.2 Hz, 1.2H), 5.92 – 5.88 (m, 0.7H), 5.63 (ddd, *J* = 10.6, 8.5, 6.7 Hz, 0.1H), 5.58 – 5.45 (m, 1.9H), 5.44 – 5.40 (m, 1.1H), 5.35 (td, *J* = 10.1, 5.0 Hz, 0.1H), 4.60 – 4.53 (m, 1.5H), 4.51 – 4.45 (m, 0.9H), 4.45 – 4.37 (m, 0.3H), 4.26 (ddd, *J* = 8.3, 5.8, 1.9 Hz, 0.9H), 4.22 – 4.10 (m, 2.3H), 4.10 – 3.89 (m, 1.8H), 3.82 (dd, *J* = 8.5, 4.0 Hz, 0.1H), 3.79 (dt, *J* = 10.8, 3.4 Hz, 0.1H), 3.71 – 3.62 (m, 1.2H), 3.61 – 3.55 (m, 0.8H), 3.42 (ddt, *J* = 7.0, 4.7, 1.9 Hz, 0.1H), 2.75 (ddd, *J* = 15.3, 12.0, 3.0 Hz, 0.2H), 2.64 – 2.37 (m, 5.3H), 2.35 – 2.19 (m, 4.2H), 2.14 – 1.97 (m, 2.1H), 1.97 – 1.83 (m, 2.2H), 1.75 – 1.67 (m, 4.1H).

$^{13}$C NMR (151 MHz, $CDCl_3$) δ 176.95, 176.89, 175.05, 173.45, 133.00, 132.88, 132.12, 131.57, 130.84, 130.50, 130.48, 130.16, 130.12, 129.53, 129.47, 129.40, 128.50, 127.39, 127.24, 127.11, 127.04, 119.78, 119.71, 85.24, 83.95, 83.87, 79.56, 79.45, 78.03, 77.90, 76.24, 76.04, 75.03, 74.60, 74.46, 73.61, 73.50, 73.46, 73.39, 73.35, 73.23, 73.17, 72.00, 71.93, 71.76, 71.73, 71.32, 71.27, 68.93, 68.79, 65.90, 65.86, 65.84, 65.79, 64.53, 64.13, 64.07, 60.95, 40.95, 40.16, 37.35, 35.80, 35.77, 35.75, 35.12, 34.99, 34.96, 34.83, 34.51, 34.40, 33.88, 33.70, 32.06, 30.88, 30.58, 29.83, 29.80, 29.49, 28.29, 28.26, 28.21, 24.73, 24.67, 24.62, 24.57, 24.54, 24.50, 24.43, 23.09, 23.07, 22.83, 14.33, 14.26.

HRMS (ESI): Anal. Calcd. for $C_{24}H_{34}ClO_6^+$ $[M-H_2O+H]^+$ 453.2038, found 453.2030

IR (neat): $\nu_{max}\ (cm^{-1})$ = 3403 (br, OH), 2932 (m, CH), 1770 (s, CO), 1646 (w, C=*CH*), 1441 (m), 1184 (m), 1106 (m), 1058 (m)

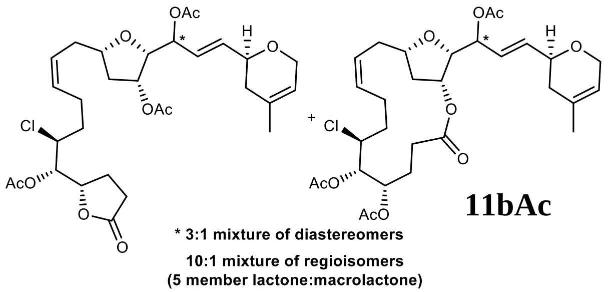


**11bAc**

**11bAc** was prepared according to general procedure A (1.27 mg, quant.)

**Analytical Data for 11bAc:**

$R_f$ = 0.72 (2:3 Hexanes/EtOAc)

HRMS (ESI): Anal. Calcd. for $C_{30}H_{45}ClNO_{10}^+$ $[M+NH_4]^+$ 614.2727, found 614.2711

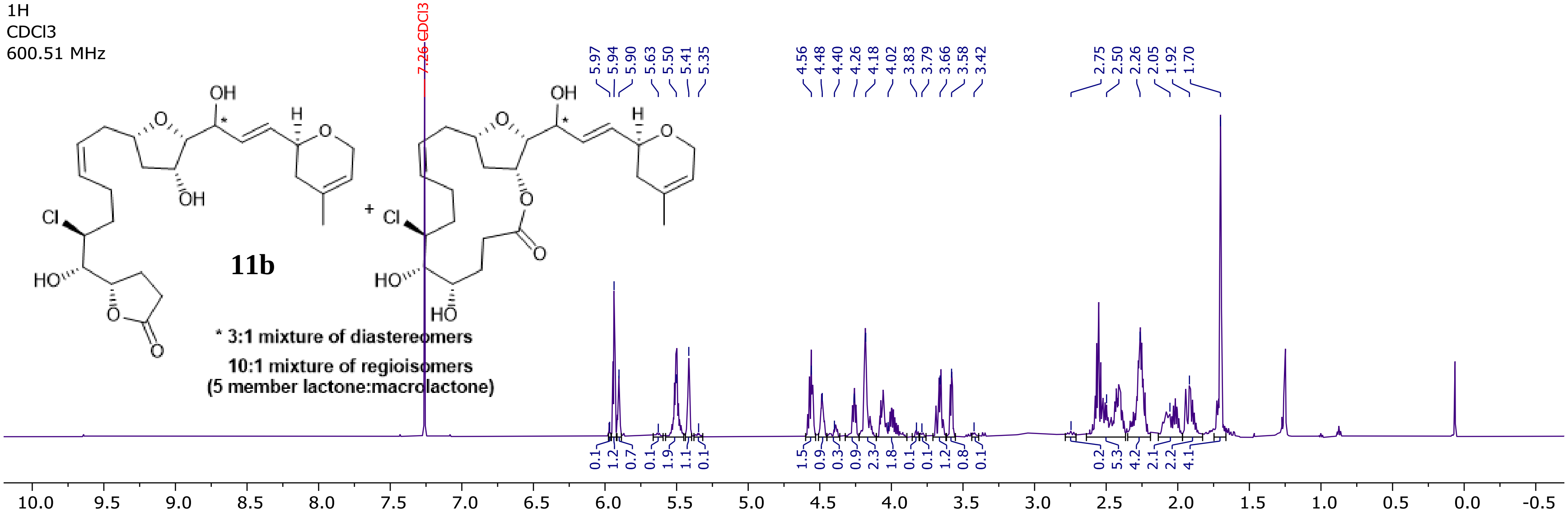
1H
CDCl3
600.51 MHz
11b
* 3:1 mixture of diastereomers
10:1 mixture of regioisomers
(5 member lactone:macrolactone)

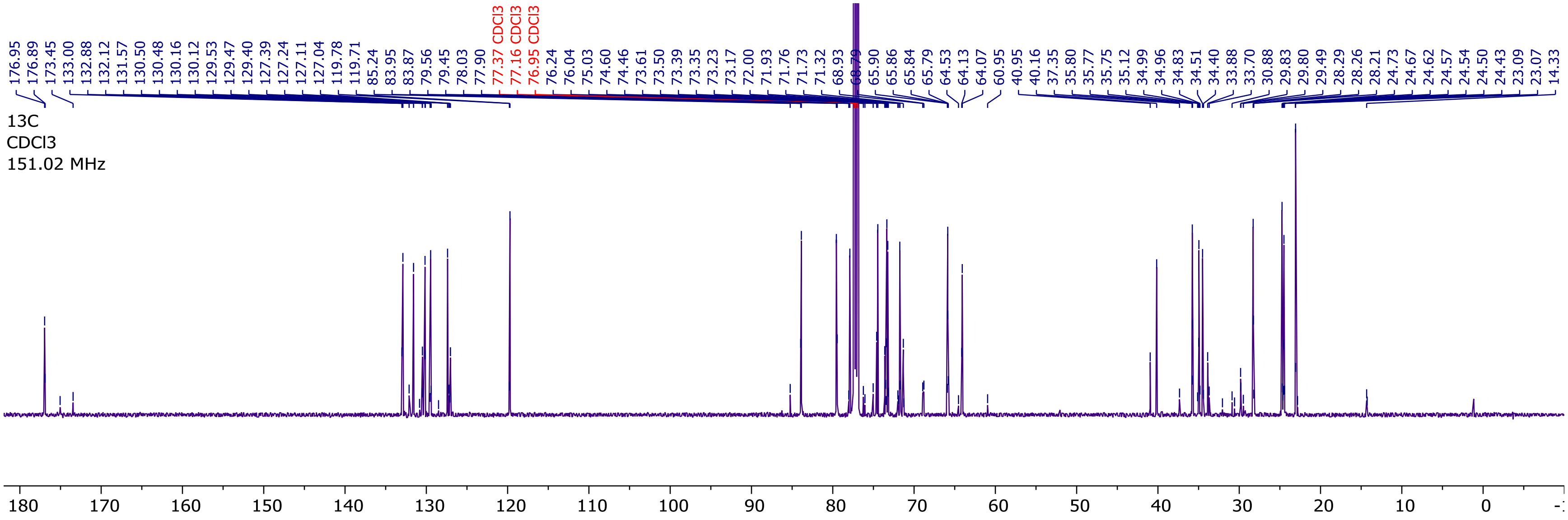
13C
CDCl3
151.02 MHz

To solution of **S167** (25 mg, 0.065 mmol, 1.0 eq.) in deoxygenated DMSO (5x freeze-pump-thaw cycles) (1.6 mL) was added $CrCl_2$ doped with 1 % $NiCl_2$ (w/w) (80 mg, 0.65 mmol, 10 eq.). **3a** (47 mg, 0.195 mmol, 3.0 eq.) was added via syringe in deoxygenated DMSO (2x 0.4 mL) and the mixture was stirred for 18 h at rt. After this time, the reaction mixture was transferred to a separatory funnel, diluted with $Et_2O$ (10 mL) and 10 mL of 1:1 (v/v) $H_2O$-brine was added. The organic layer was separated, and the aqueous layer extracted with $Et_2O$ (15x 20 mL). The combined organic layers were dried ($MgSO_4$), filtered, and solvent was removed in vacuo. The crude product was purified via flash column chromatography (24:1 $CH_2Cl_2$/MeOH). Appropriate fractions were pooled, and solvent was removed in vacuo to yield the diol (11 mg) as an oily solid and as a 3:1 mixture of diastereomers. The product was used immediately in the subsequent step.

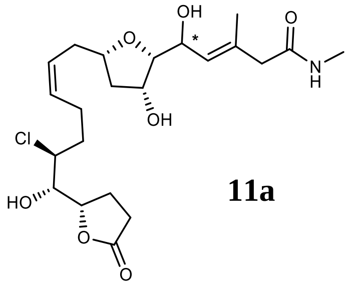


**11a**

* 3:1 mixture of diastereomers

An approximately 1.2 M solution of aqueous HCl in MeOH was prepared by slowly adding concentrated HCl (1 mL, *ca.* 12 M) to MeOH (9 mL). To a cold (0 °C), stirred solution of the diol from the previous step (11 mg, 0.022 mmol, 1.0 eq.) in $CH_2Cl_2$ (2 mL) MeOH (6 mL) was added the previously prepared solution of aqueous HCl in MeOH (92, *ca.* 1.2M in MeOH, 0.11 mmol, 5.0 eq.) via microsyringe. The reaction vessel was moved to a fridge (4 °C) and allowed to stir in the fridge for 42 h. After this time, the reaction mixture was warmed to rt, and allowed to stir for a further 7 h 30 min. Afte this time, the reaction vessel was again transferred back to the fridge (4 °C), and allowed to stir for a further 18 h 30 min. After this time, starting material was consumed as monitored by TLC analysis, and the reaction mixture was quenched by addition of $NaHCO_3$ (30 mg, 0.36 mmol, 16 eq.) and allowed to stir vigorously for 5 min. After this time, the reaction mixture was filtered and solvent was removed in vacuo. The crude product was then purified via flash column chromatography (193:7 to 19:1 to 23:2 $CH_2Cl_2$/MeOH). Appropriate fractions were pooled, and solvent was removed in vacuo to yield **11a** (5.5 mg, 16 %, 4 steps from **S165**) as a 3:1 mixture of the 5 membered translactonization product which was a colorless oil.

**Analytical Data for 11a:**

$R_f$ = 0.22 (93:7 $CH_2Cl_2$/MeOH)

$^1$H NMR (600 MHz, MeOD) δ 5.60 – 5.42 (m, 2.9H), 5.39 (dq, *J* = 9.3, 1.4 Hz, 0.2H), 4.70 – 4.57 (m, 1.6H), 4.44 (dq, *J* = 6.6, 3.2, 2.8 Hz, 0.8H), 4.40 – 4.33 (m, 0.4H), 4.24 (dt, *J* = 6.8, 3.1 Hz, 0.2H), 4.17 – 4.09 (m, 0.6H), 3.94 – 3.82 (m, 1H), 3.73 – 3.67 (m, 0.7H), 3.65 – 3.60 (m, 0.5H), 3.56 – 3.50 (m, 1H), 3.00 – 2.88 (m, 2H), 2.78 – 2.67 (m, 3H), 2.63 – 2.11 (m, 8.7H), 2.02 – 1.73 (m, 5.1H), 1.71 – 1.58 (m, 1.4H).

$^{13}C$ NMR (151 MHz, MeOD) δ 178.19, 172.98, 134.24, 133.18, 130.33, 130.31, 129.92, 129.65, 129.60, 127.90, 126.90, 126.83, 126.57, 85.88, 85.07, 80.04, 80.01, 77.98, 77.96, 77.78, 76.13, 76.09, 74.20, 74.13, 71.75, 71.37, 70.25, 67.42, 66.16, 63.33, 63.28, 46.39, 46.22, 40.75, 39.90, 35.03, 35.00, 34.41, 34.38, 33.85, 33.83, 33.64, 29.72, 28.78, 27.57, 25.19, 25.08, 24.28, 24.09, 23.96, 23.95, 15.90, 15.75.

HRMS (ESI): Anal. Calcd. for $C_{22}H_{35}ClNO_7^+$ $[M+H]^+$ 460.2097, found 460.2083

IR (neat): $\nu_{max}$ $(cm^{-1})$ = 3335 (br, OH), 2922 (m, CH), 2856 (m, CH), 1769 (s, CO), 1635 (s), 1549 (m), 1440 (m), 1415 (m), 1341 (m), 1267 (m)

**11aAc** was prepared according to general procedure A (0.73 mg, 57%)

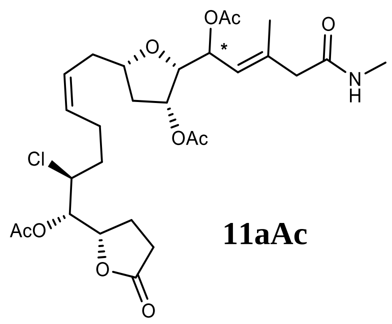


**11aAc**

* 3:1 mixture of diastereomers

**Analytical Data for 11aAc:**

$R_f$ = 0.27 (100% EtOAc)

HRMS (ESI): Anal. Calcd. for $C_{28}H_{41}ClNO_{10}^+$ $[M+H]^+$ 586.2414, found 586.2386

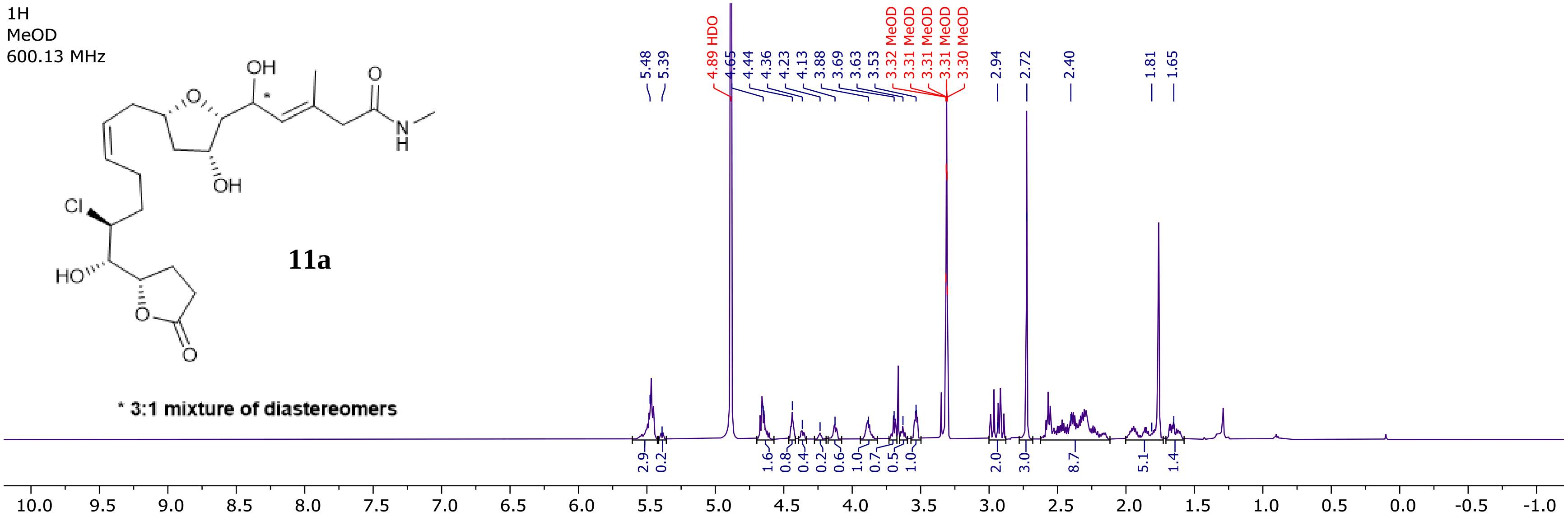


**11a**

* 3:1 mixture of diastereomers

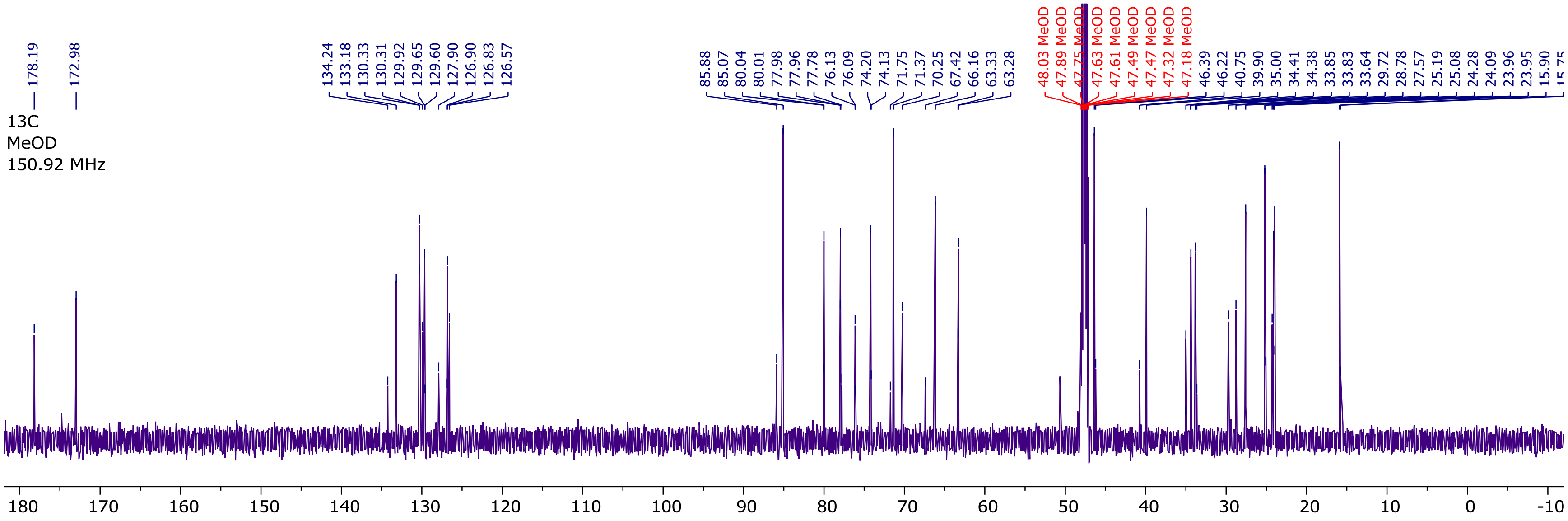

To a solution of **S167** (25.0 mg, 0.065 mmol, 1.0 eq.) in deoxygenated DMSO (5x freeze-pump-thaw cycles) (2.6 mL) was added $CrCl_2$ doped with 1 % $NiCl_2$ (w/w) (80 mg, 0.65 mmol, 10 eq.). **3c** (38 µL, 65 mg, 0.26 mmol, 4.0 eq.) was added via microsyringe and the mixture was stirred for 18 h at rt. After this time, the reaction mixture was transferred to a separatory funnel, diluted with $Et_2O$ (10 mL) and 10 mL of 1:1 (v/v) $H_2O$-brine was added. The organic layer was separated, and the aqueous layer extracted with $Et_2O$ (15x 20 mL). The combined organic layers were dried ($MgSO_4$), filtered, and solvent was removed in vacuo. The crude product was purified via flash column chromatography (9:1 to 3:1 Hexanes/Acetone). Appropriate fractions were pooled, and solvent was removed in vacuo to yield the alcohol (12.7 mg) as a colorless oil and a 1:1 mixture of diastereomers. The product was used immediately in the subsequent step.

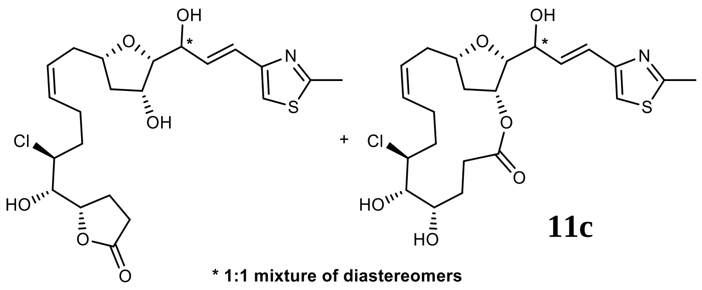

**11c**

* 1:1 mixture of diastereomers
10:1 mixture of regioisomers (5 membered lactone:macrolactone)

An approximately 1.2 M solution of aqueous HCl in MeOH was prepared by slowly adding concentrated HCl (1 mL, *ca.* 12 M) to MeOH (9 mL). To a cold (0 °C), stirred solution of the alcohol from the previous step (12.7 mg, 0.0248 mmol, 1.0 eq.) in $CH_2Cl_2$ (2 mL) MeOH (6.7 mL) was added the previously prepared solution of aqueous HCl in MeOH (103 µL, *ca.* 1.2M in MeOH, 0.124 mmol, 5.0 eq.) via microsyringe. The reaction vessel was moved to a fridge (4 °C) and allowed to stir in the fridge for 42 h. After this time, the reaction mixture was warmed to rt, and allowed to stir for a further 7 h 30 min. After this time, the reaction vessel was again moved to a fridge (4 °C), and allowed to stir in the fridge for a further 18 h 30 min. After this time, starting material was consumed as monitored by TLC analysis, and the reaction mixture was quenched by addition of $NaHCO_3$ (30 mg, 0.35 mmol, 14 eq.) and allowed to stir vigorously for 5 min. After this time, the reaction mixture was filtered and solvent was removed in vacuo. The crude product was then purified via flash column chromatography (97:3 to 24:1 to 19:1 to 95.5:4.5 $CH_2Cl_2$/EtOH). Appropriate fractions were pooled, and solvent was removed in vacuo to yield **11c** (4.3 mg, 12 %, 4 steps from **S165**) as a 1:1 mixture of diastereomers, a 10:1 mixture of regioisomers (5 membered lactone:macrolactone), and as a white amorphous solid.

**Analytical Data for 11c:**

$R_f$ = 0.37 (93:7 $CH_2Cl_2$/MeOH)

$^1$H NMR (600 MHz, MeOD) δ 7.23 – 7.18 (m, 1H), 6.77 – 6.71 (m, 0.5H), 6.70 – 6.63 (m, 1.5H), 5.64 (q, *J* = 8.9 Hz, 0.2H), 5.60 – 5.45 (m, 2H), 5.43 (dd, *J* = 6.0, 2.9 Hz, 0.2H), 5.41 – 5.34 (m, 0.3H), 4.76 – 4.71 (m, 0.1H), 4.70 – 4.63 (m, 0.6H), 4.59 (ddd, *J* = 7.0, 5.3, 1.4 Hz, 0.5H), 4.53 (dd, *J* = 7.8, 4.3 Hz, 0.4H), 4.48 (ddd, *J* = 6.7, 4.3, 2.4 Hz, 0.4H), 4.44 – 4.36 (m, 0.7H), 4.33 (ddt, *J* = 6.1,

4.0, 2.0 Hz, 0.5H), 4.15 (tdd, *J* = 9.7, 4.6, 2.2 Hz, 0.8H), 3.97 (td, *J* = 8.0, 3.9 Hz, 0.2H), 3.94 – 3.86 (m, 0.9H), 3.81 (dt, *J* = 10.3, 3.3 Hz, 0.2H), 3.79 – 3.70 (m, 0.8H), 3.70 – 3.62 (m, 1H), 3.54 (dtd, *J* = 7.2, 3.8, 2.5 Hz, 0.8H), 2.76 – 2.67 (m, 3.3H), 2.63 – 2.13 (m, 10.2H), 2.05 – 1.57 (m, 4.6H).

$^{13}$C NMR (151 MHz, MeOD) δ 174.83, 166.38, 153.24, 153.17, 133.00, 132.99, 132.75, 131.24, 129.60, 129.57, 128.96, 128.19, 126.91, 126.85, 126.69, 126.64, 123.26, 122.86, 122.69, 114.84, 114.80, 114.57, 85.97, 85.94, 84.94, 84.93, 84.65, 80.03, 78.05, 78.01, 77.83, 77.80, 76.98, 76.44, 76.07, 74.37, 74.11, 74.07, 71.66, 71.62, 70.80, 70.78, 70.26, 69.87, 68.87, 68.28, 63.30, 63.24, 50.65, 40.79, 39.83, 37.66, 35.02, 34.51, 34.40, 34.38, 33.91, 33.77, 33.75, 29.72, 28.76, 27.57, 24.26, 24.24, 24.06, 23.93, 17.34, 17.32.

HRMS (ESI): Anal. Calcd. for $C_{22}H_{31}ClNO_6S^+$ $[M+H]^+$ 472.1555, found 472.1551

IR (neat): $\nu_{max}$ $(cm^{-1})$ = 3402 (br, OH), 3011 (w, C=*CH*), 2929 (m, CH), 2858 (m, CH), 1768 (s, C=O), 1725 (s, C=O), 1560 (w, C=C), 1508 (w), 1440 (s), 1260 (s)

**11cAc** was prepared according to general procedure A (1.52 mg, quant.)

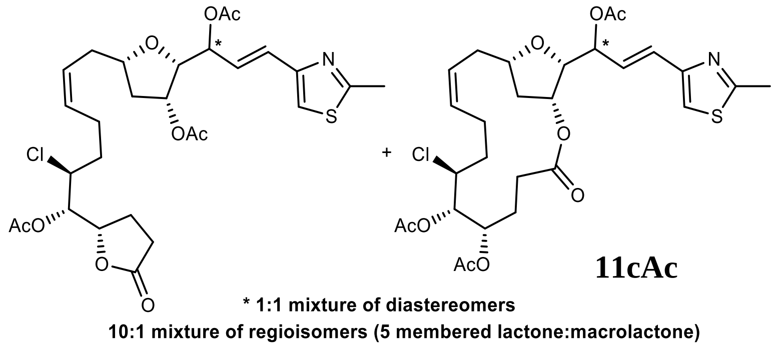


**11cAc**

**Analytical Data for 11cAc:**

$R_f$ = 0.75 (2:3 Hexanes/EtOAc)

HRMS (ESI): Anal. Calcd. for $C_{28}H_{37}ClNO_9S^+$ 598.1872, found 598.1840

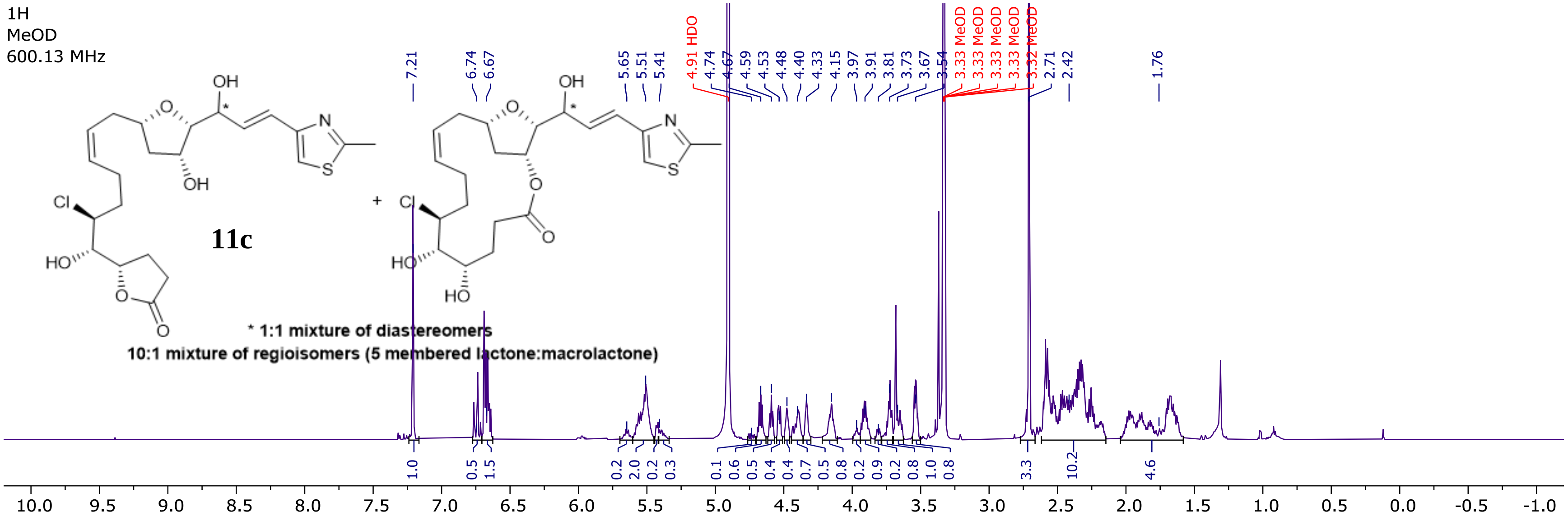
1H
MeOD
600.13 MHz
11c
* 1:1 mixture of diastereomers
10:1 mixture of regioisomers (5 membered lactone:macrolactone)

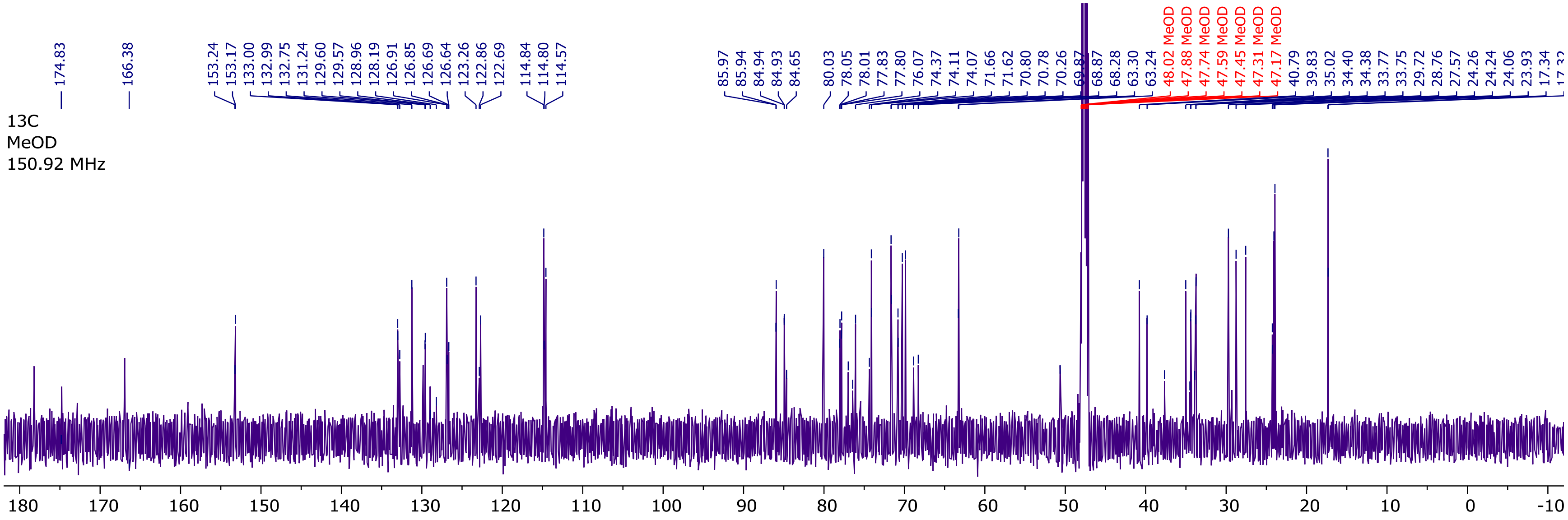
13C
MeOD
150.92 MHz

# Supplementary References


1. Chen, I.-J. & Foloppe, N. Tackling the conformational sampling of larger flexible compounds and macrocycles in pharmacology and drug discovery. *Bioorg. Med. Chem.* **21**, 7898–7920 (2013).
2. Grant, J. A., Gallardo, M. A. & Pickup, B. T. A fast method of molecular shape comparison: a simple application of a gaussian description of molecular shape. *J. Comput. Chem.* **17**, 1653–1666 (1996).
3. Hawkins, P. C. D., Skillman, A. G. & Nicholls, A. Comparison of shape-matching and docking as virtual screening tools. *J. Med. Chem.* **50**, 74–82 (2007).
4. Schmitt, D. C., Dechert-Schmitt, A.-M. R. & Krische, M. J. Iridium-catalyzed allylation of chiral β-stereogenic alcohols: bypassing discrete formation of epimerizable aldehydes. *Org. Lett.* **14**, 6302–6305 (2012).
5. Sit, M. K. *et al.* Synthesis of the macrolactone cores of maltepolides via a diene–ene ring-closing metathesis strategy. *Org. Lett.* **25**, 1633–1637 (2023).
6. Smith, A. B., Minbiole, K. P., Verhoest, P. R. & Schelhaas, M. Total synthesis of (+)-phorboxazole A exploiting the Petasis−Ferrier rearrangement. *J. Am. Chem. Soc.* **123**, 10942–10953 (2001).
7. Bergeron-Brlek, M., Teoh, T. & Britton, R. A tandem organocatalytic α-chlorination–aldol reaction that proceeds with dynamic kinetic resolution: a powerful tool for carbohydrate synthesis. *Org. Lett.* **15**, 3554–3557 (2013).

8. Chavan, L. N., Chegondi, R. & Chandrasekhar, S. Tandem organocatalytic approach to C28–C35 fragment of eribulin mesylate. *Tetrahedron Lett.* **56**, 4286–4288 (2015).
9. Otero Martinez, H., Reinke, H., Michalik, D. & Vogel, C. Peracetylated β-allyl C-glycosides of D-ribofuranose and 2-deoxy-D-ribofuranose in the chemical literature: until now, mirages in the literature. *Synthesis* **2009**, 1834–1840 (2009).
10. Doboszewski, B. Easy synthesis of 1-allyl-1-deoxy-β- and α- D-lyxofuranoses. *J. Carbohydr. Chem.* **21**, 79–88 (2002).
11. Canova, S. *et al.* Total synthesis of herbimycin A. *Org. Lett.* **9**, 145–148 (2007).
12. Kishimoto, S. *et al.* Tumescenamide c, an antimicrobial cyclic lipodepsipeptide from Streptomyces sp. *Tetrahedron* **68**, 5572–5578 (2012).
13. Chakraborty, T. K. & Chattopadhyay, A. K. Total synthesis of cruentaren b. *J. Org. Chem.* **73**, 3578–3581 (2008).
14. Herrmann, A. T., Saito, T., Stivala, C. E., Tom, J. & Zakarian, A. Regio- and stereocontrol in rhenium-catalyzed transposition of allylic alcohols. *J. Am. Chem. Soc.* **132**, 5962–5963 (2010).
15. Rankin, S. S., Caldwell, J. J., Cronin, N. B., van Montfort, R. L. M. & Collins, I. Synthesis of a ribose-incorporating medium ring scaffold via a challenging ring-closing metathesis reaction. *Eur. J. Org. Chem.* **2016**, 4496–4507 (2016).
16. Paños, J. *et al.* Synthesis and biological evaluation of truncated α-tubulin-binding pironetin analogues lacking alkyl pendants in the side chain or the dihydropyrone ring. *Org. Biomol. Chem.* **11**, 5809–5826 (2013).

17. Gersbach, P. *et al.* A ring-closing metathesis (RCM)-based approach to Mycolactones A/B. *Chem. – Eur. J.* **17**, 13017–13031 (2011).

18. Gade, N. R. & Iqbal, J. Stereoselective formal synthesis of macrolide core of migrastatin using late stage C–H oxidation. *Tetrahedron Lett.* **54**, 4225–4227 (2013).

19. Paterson, I., De Savi, C. & Tudge, M. Total synthesis of the microtubule-stabilizing agent (−)-laulimalide. *Org. Lett.* **3**, 3149–3152 (2001).

20. Chavez, D. E. & Jacobsen, E. N. An efficient, highly diastereo- and enantioselective hetero-Diels-Alder catalyst. preparation of (2S,6R)-6-(tert-butyldimethyl-silyloxymethyl)-2-methoxy-2,5-dihydropyran. *Org. Synth.* **82**, 34 (2005).

21. Maddaluno, J., Gaonac'h, O., Le Gallic, Y. & Duhamel, L. Conjugate-elimination on unsaturated acetals: a one-step route to functionalized 1,3-dienes. *Tetrahedron Lett.* **36**, 8591–8594 (1995).

22. Ghosh, A. K., Wang, Y. & Kim, J. T. Total synthesis of microtubule-stabilizing agent (−)-laulimalide. *J. Org. Chem.* **66**, 8973–8982 (2001).

23. Fischer, N. *et al.* Sensitivities of some imidazole-1-sulfonyl azide salts. *J. Org. Chem.* **77**, 1760–1764 (2012).

24. Jepsen, T. H. & Kristensen, J. L. In situ generation of the Ohira–Bestmann reagent from stable sulfonyl azide: scalable synthesis of alkynes from aldehydes. *J. Org. Chem.* **79**, 9423–9426 (2014).

25. Nelson, S. G., Cheung, W. S., Kassick, A. J. & Hilfiker, M. A. A de novo enantioselective total synthesis of (−)-laulimalide. *J. Am. Chem. Soc.* **124**, 13654–13655 (2002).

26. Rand, C. L., Van Horn, D. E., Moore, M. W. & Negishi, E. A versatile and selective route to difunctional trisubstituted (E)-alkene synthons via zirconium-catalyzed carboalumination of alkynes. *J. Org. Chem.* **46**, 4093–4096 (1981).

27. Paterson, I. *et al.* Design, synthesis and biological evaluation of novel, simplified analogues of laulimalide: modification of the side chain. *Bioorg. Med. Chem. Lett.* **15**, 2243–2247 (2005).
