# Supplementary material for: Targeted sampling of natural product space to identify bioactive natural product-like polyketide macrolides
Source: Nat Commun. 2024 Mar 21;15:2534. doi: 10.1038/s41467-024-46721-x (PMC10958047; doi:10.1038/s41467-024-46721-x)
Supplement: Supplementary file 2 — Reporting summary [file 41467_2024_46721_MOESM2_ESM.pdf]

nature portfolio

Corresponding author(s): Robert Britton
Last updated by author(s): Jan 27, 2024

Reporting Summary

Nature Portfolio wishes to improve the reproducibility of the work that we publish. This form provides structure for consistency and transparency in reporting. For further information on Nature Portfolio policies, see our Editorial Policies and the Editorial Policy Checklist.

Statistics

For all statistical analyses, confirm that the following items are present in the figure legend, table legend, main text, or Methods section.

n/a	Confirmed	
☐	☒	The exact sample size (*n*) for each experimental group/condition, given as a discrete number and unit of measurement
☒	☐	A statement on whether measurements were taken from distinct samples or whether the same sample was measured repeatedly
☒	☐	The statistical test(s) used AND whether they are one- or two-sided<br>*Only common tests should be described solely by name; describe more complex techniques in the Methods section.*
☒	☐	A description of all covariates tested
☒	☐	A description of any assumptions or corrections, such as tests of normality and adjustment for multiple comparisons
☒	☐	A full description of the statistical parameters including central tendency (e.g. means) or other basic estimates (e.g. regression coefficient) AND variation (e.g. standard deviation) or associated estimates of uncertainty (e.g. confidence intervals)
☒	☐	For null hypothesis testing, the test statistic (e.g. *F*, *t*, *r*) with confidence intervals, effect sizes, degrees of freedom and *P* value noted<br>*Give P values as exact values whenever suitable.*
☒	☐	For Bayesian analysis, information on the choice of priors and Markov chain Monte Carlo settings
☒	☐	For hierarchical and complex designs, identification of the appropriate level for tests and full reporting of outcomes
☒	☐	Estimates of effect sizes (e.g. Cohen's *d*, Pearson's *r*), indicating how they were calculated

Our web collection on statistics for biologists contains articles on many of the points above.

Software and code

Policy information about availability of computer code

Data collection

Conformational sampling was performed by parametrization with SDF2TINKERXYZ and molecular geometries/conformations for scoring scoring were collected using Tinker (v8.2).

Scoring of individual conformers of NP reference compounds vs. library pMLs was done with FastROCS Tk (OpenEye toolkits v2017.10.1) on NVIDIA Tesla P100 or V100 GPUs.

Cheminformatics parameters (e.g. cLogP, TPSA, etc) of NP reference compounds and were calculated using RDKit (v2017.09.01).

NMR data were recorded in Bruker topspin (v3.5 PL6) on a Bruker Avance III 600 equipped with a 5 mm QCI cryoprobe (600 MHz), Bruker Avance II 600 equipped with a 5 mm QNP cryoprobe (600 MHz), Bruker Avance III 500 equipped with a 5 mm TXI ambient temperature probe (500 MHz), or Bruker Avance III 400 equipped with 5 mm BBOF ambient temperature probe (400 MHz).

HRMS data were recorded on either an Agilent 6210 TOF LC/MS using ESI-MS, Waters SYNAPT UPLC-ESI-qTOF, or Waters RDa ESI-TOF.

Optical rotation data were recorded on a Perkin Elmer 341 polarimeter at 589 nm and 20°C.

Infrared Spectroscopy data were recorded on a Nexus 670 Fourier transform spectrometer.

Data analysis

Scoring data from FastROCSTk (OpenEye toolkits v2017.10.1) was averaged in either Excel or python (v3.6).

Scoring data (Cs), cheminformatics parameters (e.g. cLogP, TPSA, etc), and macrocycle specific parameters (e.g. % HA in substituents, ring

size) and their associated plots or tables were processed/plotted in Excel and/or OriginPro v2021-v2024.

Raw NMR data was processed in MestreNova (v14.1). 1H NMR data was Fourier transformed with no apodization function, and the resultant NMR spectrum was phase corrected and baseline corrected with either a Whittaker smoother or ablative algorithm. 13C NMR data was Fourier transformed with a 0.5-1.0 Hz exponential apodization function, and the resultant NMR spectrum was phase corrected and baseline corrected with either a Whittaker smoother or polynomial function.

Infrared spectroscopy data was processed in ACD/spectrus processor (v2018.1.1) and was subjected to multipoint baseline correction and a 30-50 pt smoothing algorithm was applied prior to selecting the maximum (vmax) absorptions for each frequency.

HRMS data were processed in Mestrenova (v14.1).

For manuscripts utilizing custom algorithms or software that are central to the research but not yet described in published literature, software must be made available to editors and reviewers. We strongly encourage code deposition in a community repository (e.g. GitHub). See the Nature Portfolio guidelines for submitting code & software for further information.

Data

Policy information about availability of data

All manuscripts must include a data availability statement. This statement should provide the following information, where applicable:

- Accession codes, unique identifiers, or web links for publicly available datasets
- A description of any restrictions on data availability
- For clinical datasets or third party data, please ensure that the statement adheres to our policy

Raw NMR data files for all final pML library compounds have been deposited in the Zenodo database under accession code 10.5281/zenodo.10576116.

Raw data for the cell painting fingerprint generation and activity cutoff plots have been deposited in the Zenodo database under accession code 10.5281/zenodo.10576116.

All conformers of the natural products reference set compounds and the associated maximum TanimotoCombo scores used to calculate the Cs among the natural products have been deposited in the Zenodo database under accession code 10.5281/zenodo.10576116.

All conformers of the in silico pMLs and natural products reference set compounds and the associated maximum TanimotoCombo scores used to calculate the Cs between natural products and pMLs have been deposited in the Zenodo database under accession code 10.5281/zenodo.10576116.

Experimental procedures, chemical characterization data and processed 1H and 13C NMR spectra are provided in the Supplementary Information.

Source data are provided with this paper.

Research involving human participants, their data, or biological material

Policy information about studies with human participants or human data. See also policy information about sex, gender (identity/presentation), and sexual orientation and race, ethnicity and racism.

Reporting on sex and gender	N/A
Reporting on race, ethnicity, or other socially relevant groupings	N/A
Population characteristics	N/A
Recruitment	N/A
Ethics oversight	N/A

Note that full information on the approval of the study protocol must also be provided in the manuscript.

Field-specific reporting

Please select the one below that is the best fit for your research. If you are not sure, read the appropriate sections before making your selection.

☒ Life sciences ☐ Behavioural & social sciences ☐ Ecological, evolutionary & environmental sciences

For a reference copy of the document with all sections, see nature.com/documents/nr-reporting-summary-flat.pdf

Life sciences study design

All studies must disclose on these points even when the disclosure is negative.

Sample size	The macrolide screening library size was selected based on computational assessment of chemical space coverage and synthetic tractability. No synthesized final compounds were excluded from the study.
Data exclusions	No data were excluded from screening in bacterial or mammalian cell assays. All BioMAP screening data passed QC (Z' factor > 0.5)
Replication	Biological screening was performed on dilution series of all test compounds (16 x 2-fold dilutions) in triplicate for all bacterial assays, and as a single replicate for Cell Painting and MTT assays.
Randomization	Test compounds were arrayed as dilution series vertically down each column of 384 well compound plates step dilutions were not randomized to plate location.
Blinding	Plate maps for test compounds were created with unique alphanumeric codes for each molecule. Molecular structures were not revealed until activity values had been defined for each compound in each assay by the screening group.

Reporting for specific materials, systems and methods

We require information from authors about some types of materials, experimental systems and methods used in many studies. Here, indicate whether each material, system or method listed is relevant to your study. If you are not sure if a list item applies to your research, read the appropriate section before selecting a response.

Materials & experimental systems

n/a	Involved in the study
☒	☐ Antibodies
☐	☒ Eukaryotic cell lines
☒	☐ Palaeontology and archaeology
☒	☐ Animals and other organisms
☒	☐ Clinical data
☒	☐ Dual use research of concern
☒	☐ Plants

Methods

n/a	Involved in the study
☒	☐ ChIP-seq
☒	☐ Flow cytometry
☒	☐ MRI-based neuroimaging

Eukaryotic cell lines

Policy information about cell lines and Sex and Gender in Research

Cell line source(s)	U2-OS HTB-96 was sourced directly from the ATCC
Authentication	cell lines received from ATCC and used directly without further authentication.
Mycoplasma contamination	Cell lines were tested upon receipt for Mycoplasma contamination and tested negative. Additionally they were intermittently tested for mycoplasma contamination. We used a microscopy based kit for testing (MycoFluor Mycoplasama Detection Kit, Fisher, Catalog No. M7006).
Commonly misidentified lines (See ICLAC register)	No commonly misidentified cell lines were used in this study

Plants

Seed stocks	*Report on the source of all seed stocks or other plant material used. If applicable, state the seed stock centre and catalogue number. If plant specimens were collected from the field, describe the collection location, date and sampling procedures.*
Novel plant genotypes	*Describe the methods by which all novel plant genotypes were produced. This includes those generated by transgenic approaches, gene editing, chemical/radiation-based mutagenesis and hybridization. For transgenic lines, describe the transformation method, the number of independent lines analyzed and the generation upon which experiments were performed. For gene-edited lines, describe the editor used, the endogenous sequence targeted for editing, the targeting guide RNA sequence (if applicable) and how the editor was applied.*
Authentication	*Describe any authentication procedures for each seed stock used or novel genotype generated. Describe any experiments used to assess the effect of a mutation and, where applicable, how potential secondary effects (e.g. second site T-DNA insertions, mosiacism, off-target gene editing) were examined.*
